# Detection of single nucleotide polymorphisms associated with litter size in goats using genotyping-by-sequencing and association analysis

**DOI:** 10.5713/ab.24.0533

**Published:** 2025-01-24

**Authors:** Satoshi Kubota, Thara Wongdee, Pramote Paengkoum

**Affiliations:** 1 School of Animal Technology and Innovation, Institute of Agricultural Technology, Suranaree University of Technology, Nakhon Ratchasima, Thailand; 2 Section of Goat, Group of Animal Science, Suranaree University of Technology Farm, Nakhon Ratchasima, Thailand

**Keywords:** Association Analysis, Genotyping By Sequencing, Goat Doe Reproduction Traits, Litter Size, Polymerase Chain Reaction Allele Competitive Extension Genotyping, Single Nucleotide Polymorphism

## Abstract

**Objective:**

Improving fertility is a key goal in goat production. This study aimed to detect single nucleotide polymorphisms (SNPs) associated with female goat reproductive performance for use in selection processes.

**Methods:**

Nine reproductive traits were evaluated, including litter size and age at the first, second, and third parities, as well as intervals between parities, in 31 female goats (2 purebred and 29 crossbred goats in various combinations of seven breeds). DNA was extracted from blood, and SNP data were obtained using the genotyping by sequencing method. After filtering for allele depth and missing genotype data, the retained SNPs were subjected to population structure analysis and association analysis with the nine traits. For the association analysis, SNPs with false discovery rates ≤ 0.05 were considered significant. Polymerase chain reaction allele competitive extension (PACE) genotyping assay was applied to develop genetic markers.

**Results:**

An average of 304,852 SNPs were initially detected in the 31 female goats. After filtering, 21,665 SNPs were retained. The first two principal components obtained from individual genotypes classified the 31 goats into three clusters. In the association analysis, six SNPs on four chromosomes were significantly associated with the litter size at first parity. The most significant SNP was detected on chromosome 4, and three genes—IKAROS family zinc finger 1 (*IKZF1*), fidgetin-like 1 (*FIGNL1*), and dopa decarboxylase (*DDC*)—were found within 100 kb downstream and upstream of the SNP. The PACE genotyping assay confirmed genotypes at this SNP with a 96% concordance rate.

**Conclusion:**

SNPs significantly associated with litter size at first parity, candidate genes, and the PACE genotyping methods applied in this study can be used for selecting female goats in future genetic improvement programs. However, further study on the frequency of genetic mutation with a larger sample size and functional studies of the candidate genes are required.

## INTRODUCTION

Growing demand for lean, nutritious meat has increased the popularity of goat meat worldwide [1]. In Thailand, the number of goats raised for meat production continues to increase, although most goats are owned by smallholder farmers [2]. The traditional goat production system in Thailand is extensive grazing. To meet the increasing demand, an intensive production system is needed; however, labor and financial constraints make it difficult for smallholder farmers to invest in equipment. Ahlawat et al [3] reported that increasing the reproductive rate of low-producing goat breeds could help bridge the gap between meat demand and production. Moreover, improved reproductive performance of livestock breeds increases the number of available offspring, which in turn increases farmers’ profitability. Thus, high fertility in goats is an important factor for farmers.

Litter size, defined as the total number of kids born per kidding per goat, is a key component trait of doe reproduction. It is positively correlated with parity in goats, and multiple birth data are used in selection [4]. Most traits related to reproduction, including litter size, have low heritability, and genetic improvement is slow when selection is based on one or a few phenotypic records [5]. Genetic marker-assisted selection based on molecular indicators may accelerate the genetic improvement of goat fecundity. Zhang et al [6] reported that 69 molecular markers have been identified to improve the litter size of goats; however, the presence and value of these markers vary among breeds. Moreover, de Lima et al [5] suggest that litter size traits involve multiple genes and loci incorporating environmental factors and maternal influences such as maternal age and the intrauterine environment. Therefore, genomic analysis with a large number of molecular markers across the entire genome, rather than single genes, is appropriate to find markers associated with doe reproduction traits.

The genotyping by sequencing (GBS) technique is a simple, highly multiplexed, reproducible, and cost-effective method for the simultaneous discovery and genotyping of many markers [7]. The application of the GBS method to livestock provides an adequate solution for population genetic and genomic studies. Regarding fertility traits, this method has been successfully used to identify selection signatures of litter size in sheep [8] and candidate genes in Indian buffalo [9]. In this study, we performed single nucleotide polymorphism (SNP) discovery using the GBS method and conducted an association analysis to identify SNPs and putative genes related to doe reproduction traits, including litter size and age at the first, second, and third parities, as well as the intervals between parities, in goats. Our results may help to further accelerate the genetic improvement of goats, and the obtained SNP markers can be used to select genetically superior individuals in goat production.

## MATERIALS AND METHODS

### Ethics statement

The study was approved by the Animal Care and Use Committee of the Suranaree University of Technology (SUT, Nakhon Ratchasima, Thailand) (SUT 4/2558). The research was conducted following regulations on animal experiments and the Guidelines for the Use of Animals in Research, as recommended by the National Research Council of Thailand (U1-08780-2563).

### Animals

A total of 31 female goats with multiple breeding records (age and litter size of individuals in the first, second, and third parities) from the SUT Farm were used in this study. The 31 goats were born between 2012 and 2019 and reached their third parity at ages ranging from 2 years and 5 months to 6 years and 7 months. Of the 31 goats, there were one Anglo-Nubian purebred, one Boer purebred, and 29 crossbred goats. The crossbred goats were produced using a total of 7 breeds: 5 goats were crossed using 2 breeds, 22 goats were crossed using 3 breeds, and 2 goats were crossed using 4 breeds. The breeding information for each goat analyzed in this study is shown in Supplement 1. The breed ratios of most crossbred goats were different, with only two of 29 crossbred goats having the same breed ratio. The breed proportions of the 31 goats used in this study were: Anglo-Nubian 32%, Saanen 26%, Chami 18%, Boer 11%, Thai native breed 9%, Alpine 2%, and Kalahari Red 2%. All goats were born on the SUT farm and reared according to the farm’s animal husbandry standards for meat production. The farm uses natural or artificial insemination to breed only healthy females that are at least 8 months old and weigh at least 30 kg when in heat. The farm also uses breeding records up to the third parity for selection when culling female goats.

### Sample collection and DNA extraction

Approximately 3.0 mL of blood was collected aseptically from the jugular vein of each doe into a blood collection tube containing K3 EDTA (Hebei Xinle Sci&Tech Co. Ltd., Hebei, China) in October 2022. Genomic DNA was extracted from blood using the DNeasy Blood & Tissue Kit (Qiagen, Hilden, Germany) according to the manufacturer’s instructions. The quality of DNA was measured by the NanoDrop 2000 spectrophotometer (Thermo Fisher Scientific, Massachusetts, MA, USA). The integrity of DNA was checked by 1% agarose (w/v) gel electrophoresis.

### Genotyping by sequencing

Novogene Biotechnology Company (Novogene, Beijing, China) conducted the DNA library preparation and subsequent sequencing. The 250 ng of genomic DNA from each sample was digested with 5 units of MseI in a 20 μL reaction. The reaction was incubated at 37°C for 2 hours, followed by heat inactivation of the enzyme at 65°C for 20 min. The resulting fragments were ligated with two barcode adapters that either with a compatible sticky end with the digestion enzyme and the Illumina P5 or P7 universal sequences. The ligation reaction was incubated at 22°C for 2 hours, followed by heat inactivation at 65°C for 20 min. After several rounds of polymerase chain reaction (PCR) amplification, all the samples were pooled, purified using magnetic beads, and size-selected. Library quality was assessed and quantified, and genome DNA libraries with an average insert size of ~350 bp were constructed. Libraries were sequenced on an Illumina Novaseq 6000 instrument (Illumina, San Diego, CA, USA) to generate 150 bp paired-end reads. The raw reads obtained from Illumina sequencing were quality-controlled to obtain clean reads. Specifically, adapter-containing read and paired reads with more than 10% uncertain nucleotides or more than 50% low-quality nucleotides (base quality less than 5) were discarded using fastp version 0.20.0 [10]. The clean reads were aligned to the reference genome of the goat, ARS1 (INSDC Assembly GCA_001704415.1), using Burrows-Wheeler Aligner version 0.7.8-r455 [11] with parameters: mem -t 4 -k 32 -M. SNPs were called using the command ‘mpileup’ in SAMtools version 0.1.19-44428cd [12] with the parameters set as ‘-m 2 -F 0.002 -d 1000’. SNPs with a depth of the variant position > 4 and mapping quality >20 were retained for future analysis.

### Genotype data quality control

The genotype data quality was controlled using VCFtools [13] before the association analysis. An SNP was removed if it (i) had more than two alternative alleles, (ii) had a sequencing depth <10, (iii) had an assigned genotype quality <20, (iv) had more than 5% missing genotypes, or (v) had a minor allele frequency <0.05. After filtering by SNP characteristics, 21,665 SNPs were retained. The obtained dataset of 21,665 SNPs was imputed using the LD-kNNi method in Tassel version 5.2.94 [14] and used for subsequent analyses.

### Population structure and linkage disequilibrium

With 21,665 SNPs, principal components (PCs), kinship, and linkage disequilibrium (LD) among the genome-wide markers were calculated using Tassel version 5.2.94 [14]. The same 21,665 SNPs were used in STRUCTURE [15], testing population subgroups with K = 1 to 5 to determine the optimal number of population subgroups. Using the admixture model in STRUCTURE, K = 1 to 5 were tested with 150,000 reps, declaring the first 50,000 as burn-ins and performing 10 iterations for each K value. To determine the optimal number of population subgroups, cross-validation analysis was performed using ADMIXTURE version 1.3.0 [16]. LD decay plot was generated based on the r^2^ values and the distance between each SNP pair.

### Identification of single nucleotide polymorphisms associated with reproduction traits

All doe reproduction traits were tested for deviation from normality using the Shapiro–Wilk test. Before the association analysis, the square root transformation was used to analyze the non-normally distributed trait, the interval between the first and second parities in days. Litter size data, consisting of two levels (single and twin births), were treated as a qualitative trait. The litter size of individuals at the first, second, and third parities was converted with single birth coded as 0 and twin birth coded as 1. Association analysis was performed using the general linear model analysis in Tassel version 5.2.94 [14], and the first four PC vectors were used as covariates to account for population structure. The results were visualized in a Manhattan plot. The calculated p-values were subjected to false discovery rate (FDR) correction, and SNPs with FDR ≤ 0.05 were determined to be significant. For the significant SNPs, the goat reference genome (GCF_001704415.2) on the NCBI database was used to search for candidate genes within a 100 kb downstream and upstream window from the SNPs. The local LD was viewed for the significant SNPs, and LD plots were created using Haploview 4.2 [17]. The LD blocks were defined using the confidence interval method and default parameters. For litter size traits that showed correlation, genome-wide association analysis was performed using the R package rrBLUP [18], with parity as a fixed effect. The significance of SNPs was determined in the same manner as described above.

### Genotyping method for candidate single nucleotide polymorphism

This work has targeted only SNP marker development. Genotypes of the significant SNP containing candidate genes were confirmed using the PCR allele competitive extension (PACE) genotyping assay. The PACE genotyping assay involves PCR with endpoint fluorescence measurement using two allele-specific forward primers and one common reverse primer [19]. Three primers were designed by 3CR Bioscience (Essex, UK): allele-specific primer 1 (HEX-GCACAGCTA GTATTTTGGTTTTCCC), allele-specific primer 2 (FAM-AGCACAGCTAGTATTTTGGTTTTCCA), and the common primer (GAGTGGGTTTGCCTGACGAGAAAA). The fluorescent PACE genotyping assay was performed on a CFX Opus Real-Time PCR System (Bio-Rad, Hercules, CA, USA) in a final volume of 10 μL, containing 1× PACE genotyping master mix (3CR Bioscience), 12 μM of a mix of extended allele-specific primers, 30 μM of common primer, and 1 to 10 ng of template DNA. The touch-down PCR amplification conditions for PACE genotyping included hot-start activation at 94°C for 15 min, followed by 10 cycles of 94°C for 20 s, 65°C for 60 s (dropping 0.8°C per cycle), then 30 cycles of 94°C for 20 s and 57°C for 60 s, with a final step at 37°C for 60 s. CFX Maestro Software 2.3 (Bio-Rad) was used to analyze SNP genotypes, distinguishing between two homozygous genotypes emitting either FAM or HEX and a heterozygous genotype emitting both fluorescences. The assay was conducted with 31 DNA samples and two negative controls.

## RESULTS

### Litter size and age for the three parities

**Figure 1:**
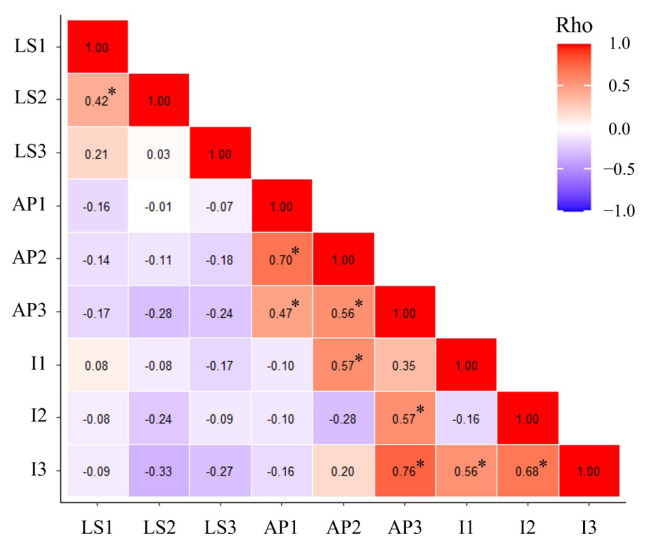
Heatmap of correlation coefficients between doe reproduction traits. Significant correlation is indicated by an asterisk (p<0.05). LS1, litter size at first parity; LS2, litter size at second parity; LS3, litter size at third parity; AP1, age at first parity (days); AP2, age at second parity (days); AP3, age at third parity (days); I1, interval between the first and second parities (days); I2, interval between the second and third parities (days); I3, interval between the first and third parities (days).

The ages and numbers of kids born at the three parities of the 31 female goats are summarized in Table 1. Nine traits were considered as doe reproduction traits: litter size at the first, second, and third parities (LS1, LS2, and LS3), age in days at the first, second, and third parities (AP1, AP2, and AP3), and the intervals in days between the first and second, second and third, and first and third parities (I1, I2, and I3). The averages of LS1, LS2, and LS3 were 1.12, 1.45, and 1.77, with standard deviations of 0.34, 0.50, and 0.42, respectively. All goats gave birth to one or two kids during each of the three parities, and litter size tended to increase with parity. The averages of AP1, AP2, and AP3 were 762, 1156, and 1655 days, with standard deviations of 242, 313, and 356 days, respectively. The averages of I1, I2, and I3 were 393, 499, and 893 days, with standard deviations of 219, 276, and 309 days, respectively. The nine reproduction traits of the individual goats analyzed in this study are presented in Supplement 2. Correlation between doe reproduction traits was examined using Spearman’s rank correlation coefficient test (Figure 1). A moderately significant correlation was found between LS1 and LS2.

### Sequencing, mapping, and single nucleotide polymorphism calling

Sequence quality assessment and SNP calling results are summarized in Table 2. After filtering out low-quality reads, an average of 9,263,800 reads were mapped to the goat reference genome, with a mapping rate of 99.72%. An average of 304,852 SNPs were initially detected in the 31 female goats. The sequencing, mapping, and SNP calling results for individual goats are presented in Supplement 3. The 21,665 SNPs were retained after filtering by SNP characteristics. The information about each SNP, including physical distance between SNPs, heterozygosity, and SNP distribution across each chromosome, is presented in Supplements 4, 5.

### Genomic relatedness and population structure in studied goats

**Figure 2:**
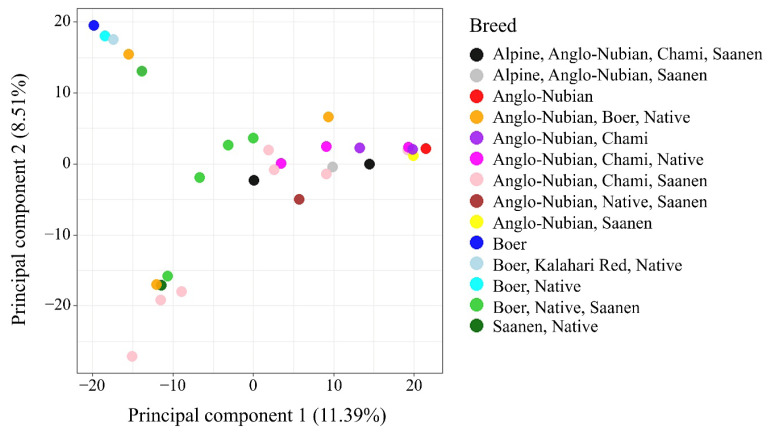
Two first principal components of 31 female goats obtained from individual genotypes. Each goat is classified and colored based on the breeds used in its production.

**Figure 3:**
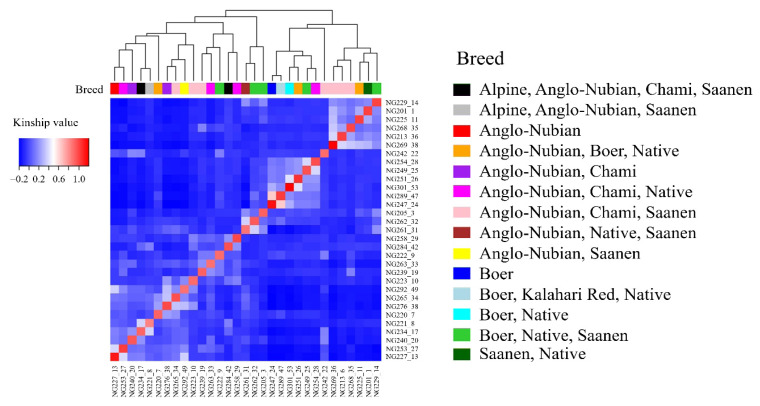
Heatmap of kinship between 31 female goats. Each goat is classified and colored based on the breeds used in its production.

**Figure 4:**
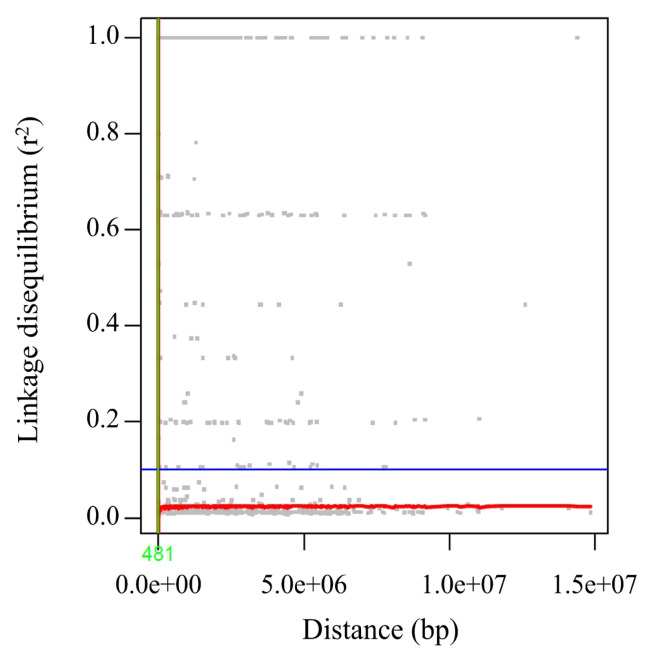
Decay of linkage disequilibrium in the 31 female goats. Pairwise linkage disequilibrium (r^2^) values are plotted against the physical distance (bp). The red line shows non-linear regression of r^2^ on physical distance. The blue horizontal line and the green vertical line represent the critical value of r^2^ (0.1) and linkage disequilibrium decay value, respectively. bp, base pair.

The 21,665 SNPs were used to examine the genetic structure of the 31 goats. The relatedness between 31 goat individuals was illustrated by PC analysis plots and kinship heatmap (Figures 2, 3). In the figure, each goat is classified and colored based on the breeds used in its production. The PC analysis revealed genetic differentiation, with the first two components explaining 19.90% of the genetic variation (11.39% for PC1 and 8.51% for PC2) (Figure 2). The purebred goats, Anglo-Nubian and Boer, were plotted at opposite positions on the PC1 axis (red and blue color, respectively). The crossbred goat that was bred with 87.5% Saanen and 12.5% Thai native breed was plotted in the lower left of the figure (dark green). Figure 3 facilitates understanding the genetic relationships between individuals. The 31 goats were divided into two large clusters based on kinship value. The first cluster contained 18 goats and the second cluster contained 13 goats, with kinship values of 0.02 and 0.08, respectively. The heatmap displays the self-relatedness along the diagonal and relatedness between individuals outside the diagonal, with each breed group as an axis. A high relatedness, kinship value higher than 0.25, was observed in 15 pairs, indicating the 31 female goats include individuals related by pedigree. The information about the PC of each goat and kinship is shown in Supplements 6, 7. The model parameters for determining the optimal number of populations obtained from STRUCTURE and ADMIXTURE are shown in Table 3. Results from STRUCTURE suggested K = 2 as the most likely scenario, however, the ADMIXTURE cross-validation analysis indicated that K = 1 was the optimal modeling choice, revealing a weak genetic structure of the 31 goats. The LD half-decay distance of 481 bp was found in the 31 goat population (Figure 4).

### Detection of single nucleotide polymorphisms associated with doe reproduction traits

**Figure 5:**
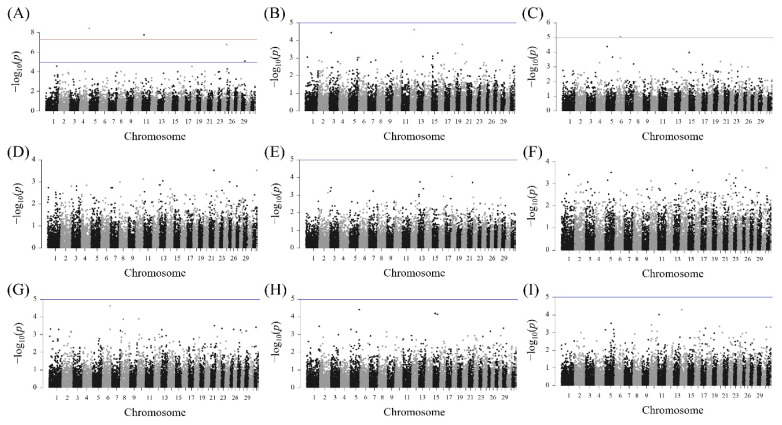
Manhattan plots of the association analysis result for the nine doe reproduction traits. The blue and red horizontal lines show the suggestive and genome-wide significance thresholds, respectively. (A) Litter size at first parity (B) litter size at second parity (C) litter size at third parity (D) age at first parity (days) (E) age at second parity (days) (F) age at third parity (days) (G) interval between the first and second parities (days) (H) interval between the second and third parities (days) (I) interval between the first and third parities (days).

The results of the association analysis of the doe reproduction traits are shown in Figure 5. Among the nine traits, significant SNPs were detected only for the LS1 trait (Figure 5A). No SNPs were found to be significantly associated with LS2, LS3, AP1, AP2, AP3, I1, I2, and I3 (Figures 5B–5I). A total of six SNPs on chromosomes 4, 11, 24, and 29 exhibited a significant association with the LS1 trait (Table 4). Five SNPs identified genes within 100 kb downstream and upstream of the significant SNPs. The most significant SNP was detected on chromosome 4, with three genes identified around it: IKAROS family zinc finger 1 (*IKZF1*), fidgetin-like 1 (*FIGNL1*), and dopa decarboxylase (*DDC*). Three SNPs detected on chromosome 29 were located on the asparaginase and isoaspartyl peptidase 1 (*ASRGL1*) gene.

**Figure 6:**
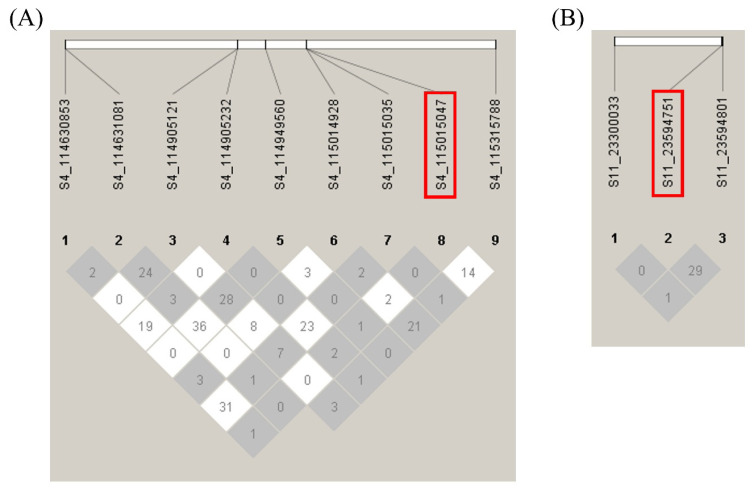
Linkage disequilibrium plot of the genomic regions harboring the significant SNP associated with litter size at first parity. (A) chromosome 4 (B) chromosome 11. Numbers in squares indicate 100-fold r^2^ values of each pair of SNPs. The intensity of gray represents the level of r^2^. The bars above the linkage disequilibrium plot represent the physical position of SNPs. The significant SNP associated with litter size at first parity is boxed in red. SNP, single nucleotide polymorphism.

**Figure 7:**
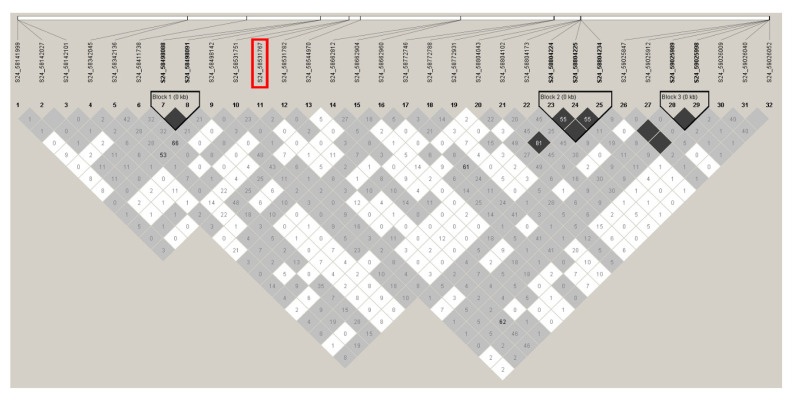
Linkage disequilibrium plot of the genomic regions on chromosome 24 harboring the significant SNP associated with litter size at first parity. Numbers in squares indicate 100-fold r^2^ values of each pair of SNPs. The intensity of gray represents the level of r^2^. Black triangles that outline parts of linkage disequilibrium plots indicate the defined linkage disequilibrium blocks. The bars above the linkage disequilibrium plot represent the physical position of SNPs. The significant SNP associated with litter size at first parity is boxed in red. SNP, single nucleotide polymorphism.

**Figure 8:**
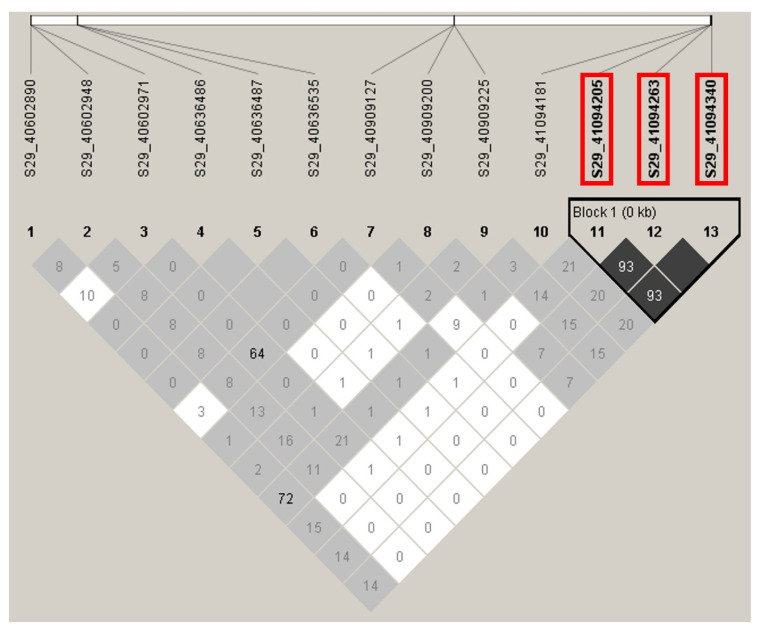
Linkage disequilibrium plot of the genomic regions on chromosome 29 harboring the significant SNP associated with litter size at first parity. Numbers in squares indicate 100-fold r^2^ values of each pair of SNPs. The intensity of gray represents the level of r^2^. Black triangles that outline parts of linkage disequilibrium plots indicate the defined linkage disequilibrium blocks. The bars above the linkage disequilibrium plot represent the physical position of SNPs. The significant SNP associated with litter size at first parity is boxed in red. SNP, single nucleotide polymorphism.

**Figure 9:**
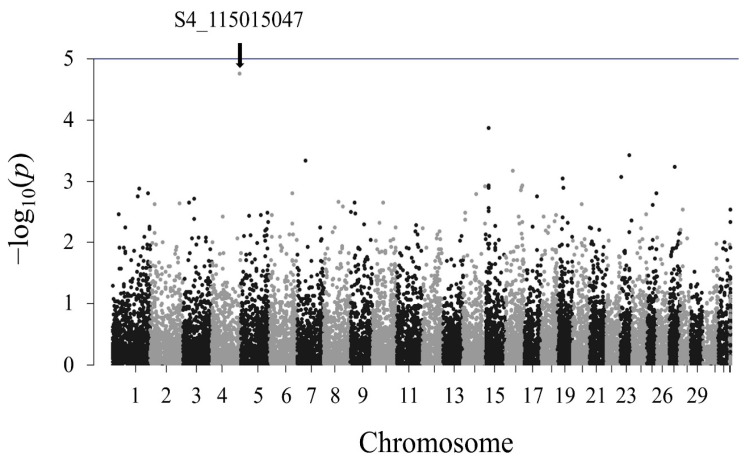
Manhattan plots of the association analysis result for the litter size at the first and second parities, with parity as a fixed effect. The blue horizontal lines show the suggestive significance threshold.

LD plots were generated for the significant SNPs on chromosomes 4, 11, 24, and 29 to examine the local LD blocks. No LD block containing significant SNP was identified on chromosomes 4, 11, and 24 (Figures 6, 7). Three significant SNPs on chromosome 29 were located within an LD block (Figure 8). However, no other SNPs were detected within the same LD block. Since a moderately significant correlation was observed between LS1 and LS2, further association analysis was performed by combining the LS1 and LS2 data as the phenotype and adjusting for parity as a fixed effect. Although no significant SNPs were identified, the most significant SNP (S4_115015047) in the LS1 trait analysis also had the lowest p-value in this analysis, slightly falling below the suggestive significance threshold (Figure 9).

### Development of a genotyping method for candidate single nucleotide polymorphism

**Figure 10:**
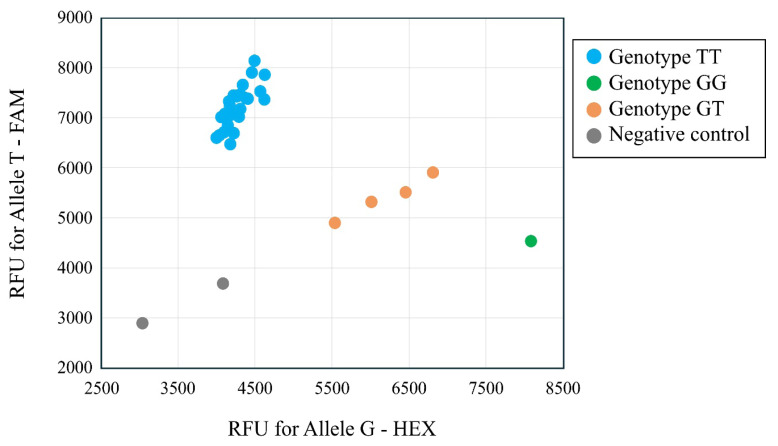
Allelic discrimination plots of the SNP marker based on a PACE genotyping assay. Blue, green, and orange dots indicate homozygous genotypes TT, GG, and heterozygous genotype GT, respectively. Gray dots indicate negative control. SNP, single nucleotide polymorphism; PACE, polymerase chain reaction allele competitive extension.

The genotypes at the most significant SNP (S4_115015047) for the LS1 trait were confirmed using the PACE genotyping assay (Figure 10). The PACE genotyping assay identified three genotypes: 26 individuals with genotype TT, 4 with genotype GT, and 1 with genotype GG. In contrast, the genotyping data obtained from the GBS method showed 25 individuals with genotype TT, 5 with genotype GT, and missing data for 1 individual. The genotypes determined by the GBS method and the PACE genotyping assay showed nearly identical results, with a 96% concordance rate (29/30) when excluding the missing genotype data (Supplement 8).

## DISCUSSION

Improving goat reproductive performance enhances farmers’ productivity and profitability. Identifying DNA variants associated with goat reproductive traits will enable selection based on these variants and accelerate genetic improvement. In this study, the GBS library from 31 female goats successfully generated 304,852 SNPs. However, this method is limited by the high amount of missing data and insufficient coverage depth, which often results in incorrect genotyping. Minimizing missing data can be achieved by sequencing fewer sites with higher target coverage [20]. Consequently, we selected 21,665 SNPs by primarily filtering based on sequencing depth and then examined the association between these SNPs and nine doe reproduction traits.

The PC plot obtained using the 21,665 SNPs confirmed three clusters, explaining 19.90% of the variation when the first two components were combined. The purebred Boer goat is plotted where PC2 values are higher than 10, the purebred Anglo-Nubian goat is placed where PC2 values are between −10 and 10, and the crossbred goat with a high proportion of Saanen breed is located where PC2 values are lower than −10, suggesting that PC2 contains components related to breed characteristics. The two main clusters generated based on kinship values included purebred Anglo-Nubian and Boer breeds in their respective clusters. All goats in the cluster belonging to the purebred Anglo-Nubian breed had PC1 values greater than −6. The other goats in the cluster belonging to the purebred Boer breed had PC1 values less than −8, except for one individual, NG242_22. This suggests that the components of PC1 include kinship values. In a study of Tunisian sheep using 115,121 SNPs obtained through the GBS method, Bedhiaf-Romdhani et al [21] reported that their PCA results showed the first two components together explained 20.8% of the variation, revealing clear differentiation between two European-origin breeds and four other breeds. These findings suggest that even a relatively small number of SNPs can reveal genetic characteristics by breed in the female goats studied. Our population structure analysis determined K = 1, suggesting that the 31 goats form a single genetic group with no clear subpopulation differentiation. This indicated extensive admixture due to interbreeding of the seven breeds in various combinations. The rapid LD decay of 481 bp reflects high genetic diversity within the population, which is consistent with the unique breed ratios observed in most crossbred individuals. These results represent the genetic characteristics of the 31 female goats used in this study, specifically the genetic homogeneity at the population level and genetic diversity at the individual level.

Genome-wide association studies (GWAS) are a powerful tool for detecting the genetic basis of complex traits. Adequate statistical power for GWAS requires a larger sample size and appropriate case-control designs. In this study, 31 female goats with multiple birth records were used due to the importance of these traits for on-farm female goat selection. Previous studies have successfully identified candidate genes associated with litter size in goats using relatively small sample sizes (31 and 40) from single farms [22,23], suggesting that key genes may also be detectable in this study. Among the 21,665 SNPs analyzed, only six SNPs were associated with litter size at the first parity (LS1). Of these, three SNPs formed one LD block and were located on gene *ASRGL1*. ASRGL1 is an enzyme involved in L-aspartic acid production, and Fitzgerald et al [24] reported that ASRGL1 protein was specifically expressed in the proliferating uterine fluid of infertile women. However, little is known about its role in fertility.

Although no LD block was identified, the most significant SNP associated with LS1 was detected on chromosome 4 (S4_115015047), and three genes—*IKZF1*, *FIGNL1*, and *DDC*—were identified within 100 kbp of it. IKAROS (IKZF1) is a transcription factor and a member of the IKAROS gene family. Members of this family play an important role in lymphocyte development and proliferative responses [25] and are significant regulators in the hematopoietic system [26]. They regulate the expression of target genes by binding to promoter regions and play a role in the gene regulatory network in the human body [27]. In mouse pituitary tissue, IKAROS is expressed and regulates the expression of adrenocorticotropic hormone and the secretion of adrenal cortical hormones [28]. However, the role of IKAROS in other tissues is largely unknown. In goats, Zhang et al [29] suggested that *IKZF1* may share similar functions with *RUNX3* by analyzing single-nucleus transcriptomics data from the ovaries of polytocous and monotocous goats (Nubian and Du’an goats, respectively) during the follicular phase. In female mice, the RUNX3 transcription factor regulates ovarian function and ovulation [30]. Moreover, Ojima et al [30] demonstrated that *RUNX3* regulates folliculogenesis and steroidogenesis in granulosa cells of immature mice. Follicle maturation and ovulation rate are reported to be critical factors affecting litter size, increasing fertilization rates and the likelihood of more offspring [31]. Fidgetin (FIGN) is an ATP-dependent microtubule-associated factor involved in chromosome segregation and cell division that promotes rapid reassembly and nucleation of microtubules from the centrosome [32]. *FIGNL1* is a gene involved in homologous recombination repair, which in mice causes male infertility due to meiotic defects [33]. In female mice, analysis of in vitro mature oocytes by Li et al [34] showed that knockdown of FIGN results in multisperm fertilization. Moreover, they found that FIGNL1 complemented the role of FIGN in female FIGN knockout mice, resulting in healthy and normal fertility. In female rats, the expression of FIGNL1 protein in primordial follicles obtained from the ovarian tissue of aged rats was downregulated compared to those of immature rats [35]. In humans, variations in the *FIGNL1* gene have been associated with premature ovarian insufficiency, a disorder characterized by the cessation of menstrual cycles before the age of 40 due to the depletion or dysfunction of ovarian follicles [36]. The *DDC* gene is responsible for the synthesis of dopamine, which is essential for a healthy pregnancy [37]. Gratz et al [37] found that patients with recurrent miscarriages had lower levels of DDC and the D_2_-dopamine receptor in the trophoblast and decidua compared to healthy controls. In an analysis comparing the transcriptomes of 12 brain regions in 1-month pregnant and non-pregnant Murciano-Granadina goats, Luigi-Sierra et al [38] found that *DDC* was downregulated in the brainstem and olfactory bulb of 1-month pregnant goats. Dopamine is also a key modulator of maternal behavior in rodents [39]. In Small Tail Han sheep, Wang et al [40] reported that the levels of neurotransmitters, including dopamine, in the serum of ewes with twin lambs were significantly higher than those of ewes with single lambs (p<0.05), and ewes that had twin lambs showed better maternal behavior quality. These findings support the notion that the three genes (*IKZF1*, *FIGN1*, and *DDC*) identified near the most significant SNP in this study may be associated with fertility in female goats. Unlike the other five SNPs, the SNP S4_115015047 also showed the lowest p-value in the association analysis considering the number of parities, suggesting this SNP plays a role in determining litter size between the first and second parities. However, the mechanism by which genotype differences at the detected SNP relate to goat litter size remains unclear.

Genotypes of the SNP S4_115015047 associated with LS1, as obtained by the GBS method, were confirmed for the same individuals by the PACE genotyping assay, yielding a 96% concordance rate. One discrepancy was observed where an individual was heterozygous for genotype GT by the GBS method but homozygous for genotype GG by the PACE genotyping assay. This discrepancy may be attributed to differences in methodology; the PACE genotyping is a targeted method using allele-specific primers and does not require the complex bioinformatics involved in GBS variant calling. Interestingly, all individuals determined to be heterozygous for genotype GT by the PACE genotyping assay had twins at their first parity in this study. To test the usefulness of this marker, it is necessary to check the genotypes of other individuals; however, this was not possible due to the availability of samples in this study. Increasing goat litter size through selection improves production efficiency and provides economic benefits to farmers. The identified SNP and the applied PACE genotyping methods in this study could potentially be used to select female goats for future genetic improvement programs. However, because the litter size data analyzed in this study consisted of two levels, association analysis applying logistic regression would be necessary for a rigorous analysis. Moreover, future studies are required to further elucidate the significance of the mutation discovery by examining the genotypes of other female goats for the SNPs significantly associated with the litter size in this study.

## CONCLUSION

Litter size variability is a significant economic trait with low heritability in domestic animals. In this study, we performed an association analysis in 31 goats using 21,665 SNPs obtained through the GBS method, focusing on nine doe reproduction traits. We identified six SNPs significantly associated with litter size at first parity. Three candidate genes, *IKZF1*, *FIGNL1*, and *DDC*, were located within 100 kb of the most significant SNP, suggesting their potential biological relevance. Furthermore, a straightforward genotyping method with allele-specific primers was established for this SNP, and all individuals identified as heterozygous for genotype GT had twins at their first parity. These findings may advance marker-assisted selection in goat breeding programs. However, the study’s limitation includes the relatively small sample size, attributed to the challenges in obtaining female goats with extensive breeding records and the limited functional understanding of the detected genes in goats. Therefore, further study on the frequency of genetic mutation with larger sample sizes and functional studies of candidate genes are necessary to further elucidate the significance of the mutation discovery and understand the mechanisms by which genotype differences affect goat fertility.

## Figures and Tables

**Table 1:** Summary of the doe reproduction traits^1)^

Traits	Abbreviation of traits	Mean	Minimum	Maximum	Standard deviation	p-value for Shapiro–Wilk test^2)^
Litter size at first parity	LS1	1.12	1	2	0.34	0.001*
Litter size at second parity	LS2	1.45	1	2	0.50	0.001*
Litter size at third parity	LS3	1.77	1	2	0.42	0.001*
Age at first parity (days)	AP1	762	380	1313	242	0.612
Age at second parity (days)	AP2	1156	662	1815	313	0.299
Age at third parity (days)	AP3	1655	922	2451	356	0.939
Interval between the first and second parities (days)	I1	393	52	860	219	0.031*
Interval between the second and third parities (days)	I2	499	114	1142	276	0.058
Interval between the first and third parities (days)	I3	893	455	1498	309	0.108

1) Values are averages from 31 goats.

2) Deviation from normality is indicated by an asterisk (p<0.05).

**Table 2:** Summary of sequencing quality, mapping, and SNP calling information^1)^

Items	Mean
Raw base (bp)	1,337,801,472
Clean base (bp)	1,337,336,167
Q20 (%)^2)^	95.27
GC content (%)	40.61
Mapped reads	9,263,800
Mapping rate (%)	99.72
Depth (×)	7.14
Coverage at least 4× (%)	3.38
Total number of SNPs	304,852

1) Values are averages of individual DNA data from 31 goats.

2) Q20 indicates the percentage of bases with a Phred value ≥20.

SNP, single nucleotide polymorphism; bp, base pair; GC, guanine-cytosine.

**Table 3:** Summary of model parameters for determining the optimal number of population^1)^

K^2)^	Mean LnP(K)^3)^	Standard deviation of LnP(K)	Delta K	Cross-validation error
1	−605,510.00	33.32	Not applicable	0.55
2	−585,023.30	145.09	52.19	0.57
3	−572,110.48	176.21	45.41	0.64
4	−567,200.35	265.42	4.20	0.75
5	−563,405.98	685.19	Not applicable	0.83

1) LnP(K) and delta K values are obtained using the STRUCTURE software, and the cross-validation error values are obtained using the ADMIXTURE software.

2) K indicates the number of genetic populations.

3) LnP(K) indicates the log probability of the data given K.

**Table 4:** Association analysis results and annotated genes associated with litter size at first parity in goats

SNP name	Chromosome	SNP position	Reference allele/alternate allele	p-value	Gene at SNP	Genes near SNP^1)^
S4_115015047	4	115015047	G/T	3.73E–09	-	*IKZF1, FIGNL1, DDC*
S11_23594751	11	23594751	A/G	1.65E–08	-	*-*
S24_58531767	24	58531767	G/C	1.61E–07	-	*RAX, CPLX4, LMAN1, CCBE1*
S29_41094205	29	41094205	G/C	8.29E–06	*ASRGL1*	*LOC102180194, LOC102180465, LOC106503745, AHNAK*
S29_41094263	29	41094263	C/T	8.25E–06	*ASRGL1*	*LOC102180194, LOC102180465, LOC106503745, AHNAK*
S29_41094340	29	41094340	C/T	8.25E–06	*ASRGL1*	*LOC102180194, LOC102180465, LOC106503745, AHNAK*

1) Genes within a 100 kb downstream and upstream window from significant SNPs are shown.

SNP, single nucleotide polymorphism; *IKZF1*, IKAROS family zinc finger 1; *FIGNL1*, fidgetin-like 1; *DDC*, dopa decarboxylase; *RAX*, retina and anterior neural fold homeobox; *CPLX4*, complexin 4; *LMAN1*, lectin, mannose binding 1; *CCBE1*, collagen and calcium binding EGF domains 1; *ASRGL1*, asparaginase and isoaspartyl peptidase 1; *LOC102180194*, secretoglobin family 1D member; *LOC102180465*, mammaglobin-A; *LOC106503745*, uncharacterized LOC106503745; *AHNAK*, *AHNAK* nucleoprotein.

## SUPPLEMENTARY MATERIAL

**Supplement 1.** Individual breed information of the 31 female goats analyzed in this study

**Supplement 2.** Individual reproduction traits of the 31 female goats analyzed in this study

**Supplement 3.** Individual sequencing data, mapping rates, depth and coverage, and SNP calling results for the 31 female goats analyzed in this study

**Supplement 4.** Information about 21,665 SNPs used for structure and association analyses in this study

**Supplement 5.** Distribution of 21,665 SNPs on each chromosome

**Supplement 6.** Principal components of the 31 female goats analyzed in this study

**Supplement 7.** Kinship of the 31 female goats analyzed in this study

**Supplement 8.** Genotypes of 31 female goats determined by the GBS method and PACE genotyping assay

## References

[1] Pophiwa P, Webb EC, Frylinck L (2020). A review of factors affecting goat meat quality and mitigating strategies. Small Rumin Res.

[2] Chaosap C, Chauychuwong N, Chauychuwong R, Sriprem C, Sivapirunthep P, Sazili AQ (2021). Carcass composition, meat quality, calpain activity, fatty acid composition and ribonucleotide content in southern Thai native goats and three-way crossbred goats. Foods.

[3] Ahlawat S, Sharma R, Maitra A, Tantia MS (2015). Current status of molecular genetics research of goat fecundity. Small Rumin Res.

[4] Akpa GN, Alphonsus C, Dalha SY, Yakubu H, Garba Y (2011). Relationship between litter size and parity of doe in smallholder goat herds in Kano and its environs, Nigeria. Afr J Agric Res.

[5] de Lima LG, de Souza NOB, Rios RR (2020). Advances in molecular genetic techniques applied to selection for litter size in goats (Capra hircus): a review. J Appl Anim Res.

[6] Zhang S, Gao X, Jiang Y (2021). Population validation of reproductive gene mutation loci and association with the litter size in Nubian goat. Arch Anim Breed.

[7] Elshire RJ, Glaubitz JC, Sun Q (2011). A robust, simple genotyping-by-sequencing (GBS) approach for high diversity species. PLOS ONE.

[8] Zhang Z, Sui Z, Zhang J (2022). Identification of signatures of selection for litter size and pubertal initiation in two sheep populations. Animals.

[9] Vohra V, Chhotaray S, Gowane G (2021). Genome-wide association studies in Indian buffalo revealed genomic regions for lactation and fertility. Front Genet.

[10] Chen S, Zhou Y, Chen Y, Gu J (2018). fastp: an ultra-fast all-in-one FASTQ preprocessor. Bioinformatics.

[11] Li H, Durbin R (2009). Fast and accurate short read alignment with Burrows–Wheeler transform. Bioinformatics.

[12] Li H, Handsaker B, Wysoker A (2009). The sequence alignment/map format and SAMtools. Bioinformatics.

[13] Danecek P, Auton A, Abecasis G (2011). The variant call format and VCFtools. Bioinformatics.

[14] Bradbury PJ, Zhang Z, Kroon DE, Casstevens TM, Ramdoss Y, Buckler ES (2007). TASSEL: software for association mapping of complex traits in diverse samples. Bioinformatics.

[15] Pritchard JK, Stephens M, Donnelly P (2000). Inference of population structure using multilocus genotype data. Genetics.

[16] Alexander DH, Novembre J, Lange K (2009). Fast model-based estimation of ancestry in unrelated individuals. Genome Res.

[17] Barrett JC, Fry B, Maller J, Daly MJ (2005). Haploview: analysis and visualization of LD and haplotype maps. Bioinformatics.

[18] Endelman JB (2011). Ridge regression and other kernels for genomic selection with R package rrBLUP. Plant Genome.

[19] von Maydell D, Shavrukov Y (2023). PCR allele competitive extension (PACE). Plant genotyping Methods in molecular biology.

[20] Beissinger TM, Hirsch CN, Sekhon RS (2013). Marker density and read depth for genotyping populations using genotyping-by-sequencing. Genetics.

[21] Bedhiaf-Romdhani S, Baazaoui I, Dodds KG (2023). Efficiency of genotyping by sequencing in inferring genomic relatedness and molecular insights into fat tail selection in Tunisian sheep. Anim Genet.

[22] EGX, Duan XH, Zhang JH (2019). Genome-wide selection signatures analysis of litter size in Dazu black goats using single-nucleotide polymorphism. 3 Biotech.

[23] Wang JJ, Zhang T, Chen QM (2020). Genomic signatures of selection associated with litter size trait in Jining Gray goat. Front Genet.

[24] Fitzgerald HC, Evans J, Johnson N (2018). Idiopathic infertility in women is associated with distinct changes in proliferative phase uterine fluid proteins. Biol Reprod.

[25] Merkenschlager M (2010). Ikaros in immune receptor signaling, lymphocyte differentiation, and function. FEBS Lett.

[26] Chen Q, Shi Y, Chen Y, Ji T, Li Y, Yu L (2019). Multiple functions of Ikaros in hematological malignancies, solid tumor and autoimmune diseases. Gene.

[27] Oh KS, Gottschalk RA, Lounsbury NW (2018). Dual roles for Ikaros in regulation of macrophage chromatin state and inflammatory gene expression. J Immunol.

[28] Ezzat S, Mader R, Yu S, Ning T, Poussier P, Asa SL (2005). Ikaros integrates endocrine and immune system development. J Clin Invest.

[29] Zhang S, Wei Y, Gao X, Song Y, Huang Y, Jiang Q (2024). Unveiling the ovarian cell characteristics and molecular mechanism of prolificacy in goats via single-nucleus transcriptomics data analysis. Curr Issues Mol Biol.

[30] Ojima F, Saito Y, Tsuchiya Y (2019). Runx3 regulates folliculogenesis and steroidogenesis in granulosa cells of immature mice. Cell Tissue Res.

[31] Fogarty NM (2009). A review of the effects of the Booroola gene (FecB) on sheep production. Small Rumin Res.

[32] Mukherjee S, Diaz Valencia JD, Stewman S (2012). Human Fidgetin is a microtubule severing the enzyme and minus-end depolymerase that regulates mitosis. Cell Cycle.

[33] L’Hôte D, Vatin M, Auer J (2011). Fidgetin-like1 is a strong candidate for a dynamic impairment of male meiosis leading to reduced testis weight in mice. PLOS ONE.

[34] Li CR, Wang RL, Xie SY (2022). Fidgetin knockdown and knockout influences female reproduction distinctly in mice. J Biomed Res.

[35] Govindaraj V, Rao AJ (2015). Comparative proteomic analysis of primordial follicles from ovaries of immature and aged rats. Syst Biol Reprod Med.

[36] Turkyilmaz A, Alavanda C, Ates EA (2022). Whole-exome sequencing reveals new potential genes and variants in patients with premature ovarian insufficiency. J Assist Reprod Genet.

[37] Gratz MJ, Stavrou S, Kuhn C (2018). Dopamine synthesis and dopamine receptor expression are disturbed in recurrent miscarriages. Endocr Connect.

[38] Luigi-Sierra MG, Guan D, López-Béjar M (2023). A protein-coding gene expression atlas from the brain of pregnant and non-pregnant goats. Front Genet.

[39] Nevard RP, Pant SD, Broster JC, Norman ST, Stephen CP (2023). Maternal behavior in beef cattle: the physiology, assessment and future directions—a review. Vet Sci.

[40] Wang H, Han C, Li M (2021). Effects of parity, litter size and lamb sex on maternal behavior of small Tail Han sheep and their neuroendocrine mechanisms. Small Rumin Res.

