# Supplementary material for: Detection of single nucleotide polymorphisms associated with litter size in goats using genotyping-by-sequencing and association analysis
Source: Anim Biosci. 2025 Jan 24;38(8):1580–93. doi: 10.5713/ab.24.0533 (PMC12229939; doi:10.5713/ab.24.0533)
Supplement: Supplementary file 1 [file ab-24-0533-Supplementary-1.pdf]

Supplement 1. Individual breed information of the 31 female goats analyzed in this study

Sample ID	Breeds	Date of birth	Father ID	Mother ID
NG201_1	87.5%Saanen 12.5%Native	17/12/2014	1/56	20/56
NG205_3	75%Saanen 12.5%Native 12.5%Boer	15/5/2014	1/51	2/55
NG213_6	50%Saanen 25%Chami 25%Anglo-Nubian	22/4/2019	5/57	6/54
NG220_7	62.5%Anglo-Nubian 25%Boer 12.5%Native	19/8/2013	14/54	5/54
NG221_8	50%Anglo-Nubian 25%Saanen 25%Alpine	7/8/2014	10/53	7/54
NG222_9	71.875%Saanen 21.875%Boer 6.25%Native	5/8/2016	1/56	3/54
NG223_10	62.5%Anglo-Nubian 25%Chami 12.5%Saanen	29/10/2015	1/55	10/55
NG225_11	75%Anglo-Nubian 12.5%Native 12.5%Boer	30/3/2015	14/54	12/53
NG227_13	100%Anglo-Nubian	12/2/2015	AI (unknown ID)	11/55
NG229_14	71.875%Saanen 21.875%Native 6.25%Boer	25/12/2013	23/54	26/55
NG234_17	50%Chami 25%Anglo-Nubian 12.5%Saanen 12.5%Alpine	20/4/2018	1/60	32/57
NG239_19	37.5%Saanen 37.5%Anglo-Nubian 25%Chami	11/7/2017	5/57	033
NG240_20	56.25%Anglo-Nubian 43.75%Chami	25/12/2017	1/55	4/55
NG242_22	50%Saanen 42.5%Chami 7.5%Anglo-Nubian	14/7/2017	23/54	1/54
NG247_24	100%Boer	9/12/2017	59/1	59/3
NG249_25	71.875%Saanen 21.875%Boer 6.25%Native	23/6/2015	22/54	4/54
NG251_26	62.5%Native 25%Boer 12.5%Anglo-Nubian	18/7/2015	3/56	6/53
NG253_27	50%Anglo-Nubian 25%Native 25%Chami	20/5/2017	14/54	14/55
NG254_28	50%Chami 37.5%Anglo-Nubian 12.5%Native	29/6/2015	1/55	4/58
NG258_29	62.5%Chami 25%Anglo-Nubian 12.5%Native	10/5/2017	5/57	9/57
NG261_31	50%Anglo-Nubian 21.875%Saanen 28.125%Native	16/11/2014	14/54	17/54
NG262_32	43.75%Saanen 43.75%Native 12.5%Boer	2/3/2014	1/53	17/55
NG263_33	62.5%Anglo-Nubian 25%Native 12.5%Chami	2/6/2015	5/53	6/56
NG265_34	75.25%Saanen 12.5%Chami 12.25%Anglo-Nubian	7/2/2017	AI (unknown ID)	2/54
NG268_35	62.5%Chami 18.75%Saanen 18.75%Anglo-Nubian	19/7/2018	AI (unknown ID)	22/60
NG269_36	46.875%Saanen 28.125%Anglo-Nubian 25%Chami	12/11/2018	5/57	10/58
NG276_38	50%Chami 50%Anglo-Nubian	13/3/2014	1/55	24/55
NG284_42	42.5%Chami 25%Saanen 25%Alpine 7.5%Anglo-Nubian	19/3/2015	2/53	7/54
NG289_47	50%Kalahari Red 37.5%Boer 12.5%Native	30/6/2012	AI (unknown ID)	7/50
NG292_49	87.5%Anglo-Nubian 12.5%Saanen	17/1/2015	AI (unknown ID)	10/55
NG301_53	75%Boer 25%Native	19/2/2015	AI (unknown ID)	30/52
