# Supplementary material for: Detection of single nucleotide polymorphisms associated with litter size in goats using genotyping-by-sequencing and association analysis
Source: Anim Biosci. 2025 Jan 24;38(8):1580–93. doi: 10.5713/ab.24.0533 (PMC12229939; doi:10.5713/ab.24.0533)
Supplement: Supplementary file 2 [file ab-24-0533-Supplementary-2.pdf]

Supplement 2. Individual reproduction traits of the 31 female goats analyzed in this study

Sample ID	LS1	LS2	LS3	AP1	AP2	AP3	I1	I2	I3
NG201_1	1	1	2	964	1240	1522	276	282	558
NG205_3	2	2	2	690	1091	1732	401	641	1042
NG213_6	1	2	2	415	664	1095	249	431	680
NG220_7	1	1	2	1031	1815	2451	784	636	1420
NG221_8	1	1	1	684	1495	2182	811	687	1498
NG222_9	1	1	2	861	1098	1318	237	220	457
NG223_10	1	1	1	924	1206	1798	282	592	874
NG225_11	1	2	2	519	759	1086	240	327	567
NG227_13	1	1	2	971	1130	1995	159	865	1024
NG229_14	1	1	2	391	725	1821	334	1096	1430
NG234_17	2	2	2	772	1395	1745	623	350	973
NG239_19	1	1	2	683	1543	1846	860	303	1163
NG240_20	1	1	2	443	739	1881	296	1142	1438
NG242_22	1	2	2	1102	1391	1683	289	292	581
NG247_24	1	1	1	902	1198	2204	296	1006	1302
NG249_25	2	2	2	740	1056	1423	316	367	683
NG251_26	2	2	2	380	662	922	282	260	542
NG253_27	1	1	2	843	1173	1723	330	550	880
NG254_28	1	2	1	587	1259	1419	672	160	832
NG258_29	1	1	1	748	1006	1814	258	808	1066
NG261_31	1	2	1	1054	1758	1975	704	217	921
NG262_32	1	2	2	1313	1731	2218	418	487	905
NG263_33	1	2	2	930	982	1385	52	403	455
NG265_34	1	2	2	1138	1210	1826	72	616	688
NG268_35	1	2	1	641	941	1359	300	418	718
NG269_36	1	1	2	528	1172	1306	644	134	778
NG276_38	1	1	2	566	1071	1682	505	611	1116
NG284_42	1	1	2	700	1301	1525	601	224	825
NG289_47	1	2	2	482	962	1598	480	636	1116
NG292_49	1	1	2	995	1359	1473	364	114	478
NG301_53	1	1	2	651	728	1326	77	598	675
Average	1.12	1.45	1.77	762	1156	1655	393	499	893
