# Supplementary material for: Detection of single nucleotide polymorphisms associated with litter size in goats using genotyping-by-sequencing and association analysis
Source: Anim Biosci. 2025 Jan 24;38(8):1580–93. doi: 10.5713/ab.24.0533 (PMC12229939; doi:10.5713/ab.24.0533)
Supplement: Supplementary file 3 [file ab-24-0533-Supplementary-3.pdf]

Supplement 3. Individual sequencing data, mapping rates, depth and coverage, and SNP calling results for the 31 female goats analyzed in this study

Sample ID	Raw base (bp)	Clean base (bp)	Q20 (%)	GC content (%)	Mapped reads	Total reads	Tag4 number	Mapping rate (%)	Average depth (×)	Coverage at least 1× (%)	Coverage at least 4× (%)	Total number of SNPs
NG201_1	908,915,616	908,692,858	94.53	39.66	6293347	6311242	565114	99.72	5.49	5.57	2.69	240,377
NG205_3	1,031,154,912	1,030,814,962	94.12	40.70	7137101	7160040	653157	99.68	6.39	5.47	3.08	281,175
NG213_6	953,792,352	953,470,912	94.15	40.64	6605030	6622888	620602	99.73	5.81	5.56	2.95	263,665
NG220_7	1,251,103,104	1,250,717,030	95.32	40.47	8664672	8687196	713523	99.74	7.18	5.90	3.33	304,060
NG221_8	926,082,432	925,722,660	96.34	40.45	6414152	6430290	617897	99.75	5.77	5.44	2.93	264,506
NG222_9	973,482,048	973,106,486	95.76	40.41	6741932	6759434	622930	99.74	5.85	5.64	2.96	268,331
NG223_10	1,013,339,520	1,013,074,208	95.69	40.24	7012274	7036266	646094	99.66	6.62	5.19	3.02	276,413
NG225_11	1,101,197,088	1,100,825,464	96.62	40.42	7626545	7646188	674160	99.74	6.50	5.75	3.16	289,830
NG227_13	980,928,576	980,393,860	95.95	40.86	6793874	6811200	621809	99.75	5.56	5.99	3.01	245,180
NG229_14	1,003,168,224	1,002,868,172	93.98	40.41	6943088	6965744	635266	99.67	6.22	5.46	3.00	272,906
NG234_17	996,938,208	996,545,374	94.74	41.01	6902111	6922494	630718	99.71	5.86	5.76	3.01	268,923
NG239_19	1,252,833,408	1,252,304,540	95.25	40.92	8675103	8699066	703882	99.72	6.76	6.28	3.34	304,716
NG240_20	1,215,370,368	1,214,944,154	93.80	40.90	8413526	8439208	692867	99.70	6.79	6.06	3.27	297,088
NG242_22	1,206,955,872	1,206,273,740	96.70	40.52	8360446	8380500	706807	99.76	6.16	6.66	3.40	309,168
NG247_24	1,334,861,568	1,334,330,858	95.12	40.90	9241226	9268820	727769	99.70	7.11	6.37	3.44	302,555
NG249_25	1,119,945,888	1,119,495,600	95.53	40.77	7756247	7776502	672775	99.74	6.36	5.97	3.19	289,875
NG251_26	1,376,191,008	1,375,793,156	94.30	40.72	9527009	9555926	738945	99.70	7.77	6.01	3.44	315,180
NG253_27	1,195,726,464	1,195,378,652	93.70	40.51	8277434	8302754	686184	99.70	7.09	5.71	3.21	277,985
NG254_28	1,670,829,696	1,670,462,002	95.51	39.77	11572015	11601642	768467	99.74	8.67	6.51	3.54	330,271
NG258_29	1,422,271,872	1,421,789,018	94.52	40.95	9849878	9875858	740134	99.74	7.67	6.28	3.48	318,247
NG261_31	1,516,360,608	1,515,852,168	95.69	40.31	10501850	10529040	762394	99.74	7.65	6.71	3.57	329,530
NG262_32	1,565,492,544	1,565,067,236	95.78	40.60	10842030	10870114	767141	99.74	8.23	6.45	3.56	331,051
NG263_33	1,667,846,304	1,667,463,562	95.53	39.86	11549073	11580928	752264	99.72	8.17	6.88	3.52	320,926
NG265_34	1,606,320,288	1,605,710,088	95.40	40.95	11123540	11153662	793734	99.73	7.83	6.95	3.77	328,559
NG268_35	1,621,898,208	1,621,210,716	95.16	41.25	11217882	11261818	797826	99.61	7.69	7.13	3.80	345,549
NG269_36	1,778,659,200	1,778,156,166	95.81	40.28	12319780	12350292	802195	99.75	8.41	7.15	3.76	331,126
NG276_38	1,783,438,272	1,782,699,620	95.41	41.03	12352953	12383460	831772	99.75	8.15	7.42	3.96	346,851
NG284_42	1,630,553,184	1,630,048,716	94.29	41.03	11294236	11322006	796330	99.75	8.16	6.77	3.73	346,884
NG289_47	1,824,102,432	1,823,495,638	96.67	40.90	12638442	12665674	834077	99.78	9.07	6.83	3.90	356,261
NG292_49	1,853,879,328	1,853,207,446	95.79	40.72	12836902	12872504	838069	99.72	8.45	7.43	3.97	356,405
NG301_53	1,688,207,040	1,687,506,118	96.28	40.84	11694131	11722052	815914	99.76	7.88	7.27	3.88	336,807
Average	1,337,801,472	1,337,336,167	95.27	40.61	9263800.94	9289187.35	717123.10	99.72	7.14	6.28	3.38	304,852
