# Supplementary material for: Detection of single nucleotide polymorphisms associated with litter size in goats using genotyping-by-sequencing and association analysis
Source: Anim Biosci. 2025 Jan 24;38(8):1580–93. doi: 10.5713/ab.24.0533 (PMC12229939; doi:10.5713/ab.24.0533)
Supplement: Supplementary file 4 [file ab-24-0533-Supplementary-4.pdf]

Supplement 4. Information about 21,665 SNPs used for structure and association analyses in this study

| Site number | SNP name | Chromosome | Physical position | Physical distance from the previous SNP | Reference allele | Alternative allele | Major allele | Major allele frequency | Minor allele | Minor allele frequency | Number heterozygous | Heterozygosity |
|---|---|---|---|---|---|---|---|---|---|---|---|---|
| 1 | S1_64056 | 1 | 64056 | 0 | G | A | A | 0.54839 | G | 0.45161 | 18 | 0.58065 |
| 2 | S1_232277 | 1 | 232277 | 168221 | T | C | T | 0.74194 | C | 0.25806 | 10 | 0.32258 |
| 3 | S1_232313 | 1 | 232313 | 36 | C | A | C | 0.93548 | A | 0.06452 | 2 | 0.06452 |
| 4 | S1_232314 | 1 | 232314 | 1 | G | A | G | 0.75806 | A | 0.24194 | 11 | 0.35484 |
| 5 | S1_342394 | 1 | 342394 | 110080 | G | A | G | 0.91935 | A | 0.08065 | 5 | 0.16129 |
| 6 | S1_342418 | 1 | 342418 | 24 | C | T | C | 0.91935 | T | 0.08065 | 5 | 0.16129 |
| 7 | S1_628007 | 1 | 628007 | 285589 | C | T | C | 0.93548 | T | 0.06452 | 4 | 0.12903 |
| 8 | S1_797399 | 1 | 797399 | 169392 | A | G | G | 0.54839 | A | 0.45161 | 18 | 0.58065 |
| 9 | S1_797464 | 1 | 797464 | 65 | T | C | T | 0.83871 | C | 0.16129 | 8 | 0.25806 |
| 10 | S1_797498 | 1 | 797498 | 34 | G | C | G | 0.79032 | C | 0.20968 | 9 | 0.29032 |
| 11 | S1_797514 | 1 | 797514 | 16 | C | T | C | 0.79032 | T | 0.20968 | 9 | 0.29032 |
| 12 | S1_797554 | 1 | 797554 | 40 | C | G | C | 0.79032 | G | 0.20968 | 9 | 0.29032 |
| 13 | S1_797600 | 1 | 797600 | 46 | G | A | G | 0.79032 | A | 0.20968 | 9 | 0.29032 |
| 14 | S1_797629 | 1 | 797629 | 29 | G | A | G | 0.79032 | A | 0.20968 | 9 | 0.29032 |
| 15 | S1_891679 | 1 | 891679 | 94050 | A | G | A | 0.87097 | G | 0.12903 | 8 | 0.25806 |
| 16 | S1_1202947 | 1 | 1202947 | 311268 | T | C | T | 0.67742 | C | 0.32258 | 14 | 0.45161 |
| 17 | S1_1202969 | 1 | 1202969 | 22 | T | G | T | 0.91935 | G | 0.08065 | 5 | 0.16129 |
| 18 | S1_1202987 | 1 | 1202987 | 18 | T | C | T | 0.67742 | C | 0.32258 | 14 | 0.45161 |
| 19 | S1_1203039 | 1 | 1203039 | 52 | T | C | T | 0.67742 | C | 0.32258 | 14 | 0.45161 |
| 20 | S1_1203066 | 1 | 1203066 | 27 | G | C | C | 0.80645 | G | 0.19355 | 12 | 0.3871 |
| 21 | S1_1203181 | 1 | 1203181 | 115 | G | A | G | 0.75806 | A | 0.24194 | 9 | 0.29032 |
| 22 | S1_1445685 | 1 | 1445685 | 242504 | A | G | A | 0.51613 | G | 0.48387 | 14 | 0.45161 |
| 23 | S1_1445800 | 1 | 1445800 | 115 | C | T | C | 0.95161 | T | 0.04839 | 3 | 0.09677 |
| 24 | S1_1499551 | 1 | 1499551 | 53751 | G | A | G | 0.77419 | A | 0.22581 | 12 | 0.3871 |
| 25 | S1_1499640 | 1 | 1499640 | 89 | C | T | C | 0.77419 | T | 0.22581 | 12 | 0.3871 |
| 26 | S1_1499711 | 1 | 1499711 | 71 | C | T | C | 0.77419 | T | 0.22581 | 12 | 0.3871 |
| 27 | S1_1920651 | 1 | 1920651 | 420940 | T | C | T | 0.85484 | C | 0.14516 | 7 | 0.22581 |
| 28 | S1_2002242 | 1 | 2002242 | 81591 | A | G | A | 0.85484 | G | 0.14516 | 9 | 0.29032 |
| 29 | S1_2003545 | 1 | 2003545 | 1303 | A | G | A | 0.6129 | G | 0.3871 | 14 | 0.45161 |
| 30 | S1_2068681 | 1 | 2068681 | 65136 | A | C | A | 0.82258 | C | 0.17742 | 11 | 0.35484 |
| 31 | S1_2068816 | 1 | 2068816 | 135 | G | A | G | 0.82258 | A | 0.17742 | 11 | 0.35484 |
| 32 | S1_2068926 | 1 | 2068926 | 110 | C | T | T | 0.66129 | C | 0.33871 | 13 | 0.41935 |
| 33 | S1_2293210 | 1 | 2293210 | 224284 | C | T | T | 0.82258 | C | 0.17742 | 9 | 0.29032 |
| 34 | S1_2293232 | 1 | 2293232 | 22 | A | T | A | 0.56452 | T | 0.43548 | 17 | 0.54839 |
| 35 | S1_2513975 | 1 | 2513975 | 220743 | G | A | G | 0.8871 | A | 0.1129 | 7 | 0.22581 |
| 36 | S1_2514080 | 1 | 2514080 | 105 | C | T | C | 0.90323 | T | 0.09677 | 6 | 0.19355 |
| 37 | S1_2514095 | 1 | 2514095 | 15 | C | T | T | 0.64516 | C | 0.35484 | 14 | 0.45161 |
| 38 | S1_2514119 | 1 | 2514119 | 24 | G | A | A | 0.51613 | G | 0.48387 | 12 | 0.3871 |
| 39 | S1_2514710 | 1 | 2514710 | 591 | G | A | G | 0.91935 | A | 0.08065 | 5 | 0.16129 |
| 40 | S1_2514770 | 1 | 2514770 | 60 | T | C | T | 0.90323 | C | 0.09677 | 6 | 0.19355 |
| 41 | S1_2514834 | 1 | 2514834 | 64 | T | C | T | 0.90323 | C | 0.09677 | 6 | 0.19355 |
| 42 | S1_2724978 | 1 | 2724978 | 210144 | A | G | A | 0.74194 | G | 0.25806 | 10 | 0.32258 |
| 43 | S1_3676900 | 1 | 3676900 | 951922 | A | G | G | 0.6129 | A | 0.3871 | 12 | 0.3871 |
| 44 | S1_3676916 | 1 | 3676916 | 16 | A | G | G | 0.6129 | A | 0.3871 | 12 | 0.3871 |
| 45 | S1_3676943 | 1 | 3676943 | 27 | G | A | G | 0.6129 | A | 0.3871 | 12 | 0.3871 |
| 46 | S1_3676969 | 1 | 3676969 | 26 | G | A | G | 0.6129 | A | 0.3871 | 12 | 0.3871 |
| 47 | S1_3676980 | 1 | 3676980 | 11 | G | A | G | 0.8871 | A | 0.1129 | 5 | 0.16129 |
| 48 | S1_3677013 | 1 | 3677013 | 33 | C | T | C | 0.6129 | T | 0.3871 | 12 | 0.3871 |
| 49 | S1_3980930 | 1 | 3980930 | 303917 | C | T | C | 0.85484 | T | 0.14516 | 5 | 0.16129 |
| 50 | S1_3980954 | 1 | 3980954 | 24 | T | C | T | 0.90323 | C | 0.09677 | 6 | 0.19355 |
| 51 | S1_4096833 | 1 | 4096833 | 115879 | G | T | G | 0.62903 | T | 0.37097 | 15 | 0.48387 |
| 52 | S1_4096997 | 1 | 4096997 | 164 | A | G | G | 0.70968 | A | 0.29032 | 14 | 0.45161 |

| Site number | SNP name | Chromosome | Physical position | Physical distance from the previous SNP | Reference allele | Alternative allele | Major allele | Major allele frequency | Minor allele | Minor allele frequency | Number heterozygous | Heterozygosity |
|---|---|---|---|---|---|---|---|---|---|---|---|---|
| 53 | S1_4434016 | 1 | 4434016 | 337019 | C | A | C | 0.72581 | A | 0.27419 | 11 | 0.35484 |
| 54 | S1_4473268 | 1 | 4473268 | 39252 | C | T | C | 0.72581 | T | 0.27419 | 15 | 0.48387 |
| 55 | S1_5026267 | 1 | 5026267 | 552999 | G | T | G | 0.90323 | T | 0.09677 | 4 | 0.12903 |
| 56 | S1_5026321 | 1 | 5026321 | 54 | G | A | G | 0.77419 | A | 0.22581 | 12 | 0.3871 |
| 57 | S1_5060737 | 1 | 5060737 | 34416 | G | A | G | 0.87097 | A | 0.12903 | 8 | 0.25806 |
| 58 | S1_5061183 | 1 | 5061183 | 446 | C | T | C | 0.90323 | T | 0.09677 | 6 | 0.19355 |
| 59 | S1_5138496 | 1 | 5138496 | 77313 | T | G | T | 0.77419 | G | 0.22581 | 10 | 0.32258 |
| 60 | S1_5249189 | 1 | 5249189 | 110693 | C | A | C | 0.8871 | A | 0.1129 | 7 | 0.22581 |
| 61 | S1_5249392 | 1 | 5249392 | 203 | C | A | C | 0.82258 | A | 0.17742 | 9 | 0.29032 |
| 62 | S1_5378035 | 1 | 5378035 | 128643 | T | C | T | 0.69355 | C | 0.30645 | 17 | 0.54839 |
| 63 | S1_5378071 | 1 | 5378071 | 36 | G | C | G | 0.67742 | C | 0.32258 | 16 | 0.51613 |
| 64 | S1_5378152 | 1 | 5378152 | 81 | C | T | C | 0.87097 | T | 0.12903 | 8 | 0.25806 |
| 65 | S1_5568370 | 1 | 5568370 | 190218 | A | G | G | 0.79032 | A | 0.20968 | 9 | 0.29032 |
| 66 | S1_5568417 | 1 | 5568417 | 47 | T | C | C | 0.79032 | T | 0.20968 | 9 | 0.29032 |
| 67 | S1_5568453 | 1 | 5568453 | 36 | G | C | G | 0.82258 | C | 0.17742 | 9 | 0.29032 |
| 68 | S1_5568577 | 1 | 5568577 | 124 | G | A | G | 0.82258 | A | 0.17742 | 9 | 0.29032 |
| 69 | S1_5650838 | 1 | 5650838 | 82261 | A | G | A | 0.90323 | G | 0.09677 | 6 | 0.19355 |
| 70 | S1_5650926 | 1 | 5650926 | 88 | C | T | C | 0.90323 | T | 0.09677 | 4 | 0.12903 |
| 71 | S1_5650962 | 1 | 5650962 | 36 | A | C | A | 0.90323 | C | 0.09677 | 4 | 0.12903 |
| 72 | S1_6187996 | 1 | 6187996 | 537034 | G | C | G | 0.72581 | C | 0.27419 | 13 | 0.41935 |
| 73 | S1_6648086 | 1 | 6648086 | 460090 | T | C | T | 0.82258 | C | 0.17742 | 9 | 0.29032 |
| 74 | S1_6648190 | 1 | 6648190 | 104 | G | T | G | 0.95161 | T | 0.04839 | 3 | 0.09677 |
| 75 | S1_8263570 | 1 | 8263570 | 1615380 | G | A | G | 0.64516 | A | 0.35484 | 10 | 0.32258 |
| 76 | S1_8263578 | 1 | 8263578 | 8 | C | T | C | 0.87097 | T | 0.12903 | 6 | 0.19355 |
| 77 | S1_8468550 | 1 | 8468550 | 204972 | A | G | A | 0.82258 | G | 0.17742 | 7 | 0.22581 |
| 78 | S1_8468721 | 1 | 8468721 | 171 | A | T | A | 0.93548 | T | 0.06452 | 2 | 0.06452 |
| 79 | S1_8536993 | 1 | 8536993 | 68272 | C | T | C | 0.83871 | T | 0.16129 | 6 | 0.19355 |
| 80 | S1_8537084 | 1 | 8537084 | 91 | G | A | G | 0.83871 | A | 0.16129 | 6 | 0.19355 |
| 81 | S1_8616913 | 1 | 8616913 | 79829 | G | A | G | 0.83871 | A | 0.16129 | 6 | 0.19355 |
| 82 | S1_8616982 | 1 | 8616982 | 69 | C | T | C | 0.95161 | T | 0.04839 | 3 | 0.09677 |
| 83 | S1_8617025 | 1 | 8617025 | 43 | C | A | C | 0.91935 | A | 0.08065 | 5 | 0.16129 |
| 84 | S1_8675602 | 1 | 8675602 | 58577 | G | A | G | 0.83871 | A | 0.16129 | 8 | 0.25806 |
| 85 | S1_8675609 | 1 | 8675609 | 7 | C | T | C | 0.91935 | T | 0.08065 | 3 | 0.09677 |
| 86 | S1_8731432 | 1 | 8731432 | 55823 | C | T | C | 0.75806 | T | 0.24194 | 13 | 0.41935 |
| 87 | S1_8915579 | 1 | 8915579 | 184147 | G | A | G | 0.77419 | A | 0.22581 | 12 | 0.3871 |
| 88 | S1_8915657 | 1 | 8915657 | 78 | G | A | G | 0.80645 | A | 0.19355 | 10 | 0.32258 |
| 89 | S1_8915780 | 1 | 8915780 | 123 | A | G | A | 0.79032 | G | 0.20968 | 9 | 0.29032 |
| 90 | S1_8958501 | 1 | 8958501 | 42721 | T | C | T | 0.53226 | C | 0.46774 | 13 | 0.41935 |
| 91 | S1_8958525 | 1 | 8958525 | 24 | A | G | A | 0.87097 | G | 0.12903 | 4 | 0.12903 |
| 92 | S1_8958538 | 1 | 8958538 | 13 | A | G | A | 0.53226 | G | 0.46774 | 13 | 0.41935 |
| 93 | S1_8958541 | 1 | 8958541 | 3 | A | G | A | 0.93548 | G | 0.06452 | 4 | 0.12903 |
| 94 | S1_8958576 | 1 | 8958576 | 35 | C | T | C | 0.53226 | T | 0.46774 | 13 | 0.41935 |
| 95 | S1_8958615 | 1 | 8958615 | 39 | T | C | T | 0.85484 | C | 0.14516 | 9 | 0.29032 |
| 96 | S1_8958625 | 1 | 8958625 | 10 | T | C | T | 0.80645 | C | 0.19355 | 12 | 0.3871 |
| 97 | S1_8958709 | 1 | 8958709 | 84 | C | T | C | 0.77419 | T | 0.22581 | 12 | 0.3871 |
| 98 | S1_10116578 | 1 | 10116578 | 1157869 | T | C | T | 0.77419 | C | 0.22581 | 12 | 0.3871 |
| 99 | S1_10116611 | 1 | 10116611 | 33 | A | G | A | 0.83871 | G | 0.16129 | 8 | 0.25806 |
| 100 | S1_10116797 | 1 | 10116797 | 186 | C | T | C | 0.6129 | T | 0.3871 | 14 | 0.45161 |
| 101 | S1_10116801 | 1 | 10116801 | 4 | G | T | G | 0.82258 | T | 0.17742 | 11 | 0.35484 |
| 102 | S1_10612180 | 1 | 10612180 | 495379 | T | C | T | 0.79032 | C | 0.20968 | 13 | 0.41935 |
| 103 | S1_10612301 | 1 | 10612301 | 121 | A | G | A | 0.58065 | G | 0.41935 | 18 | 0.58065 |
| 104 | S1_10612307 | 1 | 10612307 | 6 | A | G | A | 0.90323 | G | 0.09677 | 4 | 0.12903 |
| 105 | S1_10612401 | 1 | 10612401 | 94 | A | C | A | 0.90323 | C | 0.09677 | 4 | 0.12903 |
| 106 | S1_11268205 | 1 | 11268205 | 655804 | G | A | G | 0.79032 | A | 0.20968 | 9 | 0.29032 |

| Site number | SNP name | Chromosome | Physical position | Physical distance from the previous SNP | Reference allele | Alternative allele | Major allele | Major allele frequency | Minor allele | Minor allele frequency | Number heterozygous | Heterozygosity |
|---|---|---|---|---|---|---|---|---|---|---|---|---|
| 107 | S1_11268256 | 1 | 11268256 | 51 | C | T | C | 0.79032 | T | 0.20968 | 9 | 0.29032 |
| 108 | S1_11268345 | 1 | 11268345 | 89 | C | T | C | 0.95161 | T | 0.04839 | 3 | 0.09677 |
| 109 | S1_11268362 | 1 | 11268362 | 17 | T | G | T | 0.85484 | G | 0.14516 | 5 | 0.16129 |
| 110 | S1_11268396 | 1 | 11268396 | 34 | G | A | G | 0.91935 | A | 0.08065 | 5 | 0.16129 |
| 111 | S1_11331707 | 1 | 11331707 | 63311 | C | T | C | 0.93548 | T | 0.06452 | 4 | 0.12903 |
| 112 | S1_11331708 | 1 | 11331708 | 1 | C | G | C | 0.93548 | G | 0.06452 | 4 | 0.12903 |
| 113 | S1_11331746 | 1 | 11331746 | 38 | T | G | G | 0.58065 | T | 0.41935 | 10 | 0.32258 |
| 114 | S1_11331866 | 1 | 11331866 | 120 | G | T | T | 0.58065 | G | 0.41935 | 10 | 0.32258 |
| 115 | S1_11331868 | 1 | 11331868 | 2 | G | T | G | 0.85484 | T | 0.14516 | 9 | 0.29032 |
| 116 | S1_11331871 | 1 | 11331871 | 3 | C | T | T | 0.58065 | C | 0.41935 | 10 | 0.32258 |
| 117 | S1_11421946 | 1 | 11421946 | 90075 | G | A | G | 0.90323 | A | 0.09677 | 6 | 0.19355 |
| 118 | S1_11421965 | 1 | 11421965 | 19 | G | A | G | 0.56452 | A | 0.43548 | 15 | 0.48387 |
| 119 | S1_12338622 | 1 | 12338622 | 916657 | C | G | G | 0.82258 | C | 0.17742 | 7 | 0.22581 |
| 120 | S1_12338638 | 1 | 12338638 | 16 | A | C | C | 0.82258 | A | 0.17742 | 7 | 0.22581 |
| 121 | S1_12338678 | 1 | 12338678 | 40 | A | G | G | 0.82258 | A | 0.17742 | 7 | 0.22581 |
| 122 | S1_12338691 | 1 | 12338691 | 13 | G | C | G | 0.87097 | C | 0.12903 | 6 | 0.19355 |
| 123 | S1_12338728 | 1 | 12338728 | 37 | G | A | A | 0.82258 | G | 0.17742 | 7 | 0.22581 |
| 124 | S1_12338730 | 1 | 12338730 | 2 | T | C | C | 0.82258 | T | 0.17742 | 7 | 0.22581 |
| 125 | S1_12338777 | 1 | 12338777 | 47 | C | T | T | 0.82258 | C | 0.17742 | 7 | 0.22581 |
| 126 | S1_12338804 | 1 | 12338804 | 27 | G | A | G | 0.87097 | A | 0.12903 | 6 | 0.19355 |
| 127 | S1_12338822 | 1 | 12338822 | 18 | A | G | A | 0.85484 | G | 0.14516 | 9 | 0.29032 |
| 128 | S1_13366253 | 1 | 13366253 | 1027431 | A | G | G | 0.77419 | A | 0.22581 | 12 | 0.3871 |
| 129 | S1_13366376 | 1 | 13366376 | 123 | C | A | C | 0.91935 | A | 0.08065 | 5 | 0.16129 |
| 130 | S1_13450397 | 1 | 13450397 | 84021 | A | G | A | 0.72581 | G | 0.27419 | 13 | 0.41935 |
| 131 | S1_13450492 | 1 | 13450492 | 95 | T | A | T | 0.72581 | A | 0.27419 | 13 | 0.41935 |
| 132 | S1_13586198 | 1 | 13586198 | 135706 | T | A | A | 0.56452 | T | 0.43548 | 17 | 0.54839 |
| 133 | S1_13586287 | 1 | 13586287 | 89 | A | T | A | 0.67742 | T | 0.32258 | 14 | 0.45161 |
| 134 | S1_13690743 | 1 | 13690743 | 104456 | G | A | G | 0.56452 | A | 0.43548 | 17 | 0.54839 |
| 135 | S1_13915308 | 1 | 13915308 | 224565 | C | G | C | 0.91935 | G | 0.08065 | 5 | 0.16129 |
| 136 | S1_13915415 | 1 | 13915415 | 107 | C | T | C | 0.91935 | T | 0.08065 | 5 | 0.16129 |
| 137 | S1_14018111 | 1 | 14018111 | 102696 | T | C | T | 0.87097 | C | 0.12903 | 8 | 0.25806 |
| 138 | S1_14018183 | 1 | 14018183 | 72 | T | C | T | 0.83871 | C | 0.16129 | 8 | 0.25806 |
| 139 | S1_15507600 | 1 | 15507600 | 1489417 | A | G | G | 0.59677 | A | 0.40323 | 13 | 0.41935 |
| 140 | S1_15507664 | 1 | 15507664 | 64 | C | T | C | 0.70968 | T | 0.29032 | 8 | 0.25806 |
| 141 | S1_15507806 | 1 | 15507806 | 142 | C | G | C | 0.87097 | G | 0.12903 | 4 | 0.12903 |
| 142 | S1_15782213 | 1 | 15782213 | 274407 | T | C | T | 0.67742 | C | 0.32258 | 14 | 0.45161 |
| 143 | S1_15782318 | 1 | 15782318 | 105 | C | A | C | 0.54839 | A | 0.45161 | 12 | 0.3871 |
| 144 | S1_16798783 | 1 | 16798783 | 1016465 | T | G | G | 0.56452 | T | 0.43548 | 13 | 0.41935 |
| 145 | S1_16798804 | 1 | 16798804 | 21 | G | A | G | 0.51613 | A | 0.48387 | 16 | 0.51613 |
| 146 | S1_16975946 | 1 | 16975946 | 177142 | G | A | G | 0.77419 | A | 0.22581 | 10 | 0.32258 |
| 147 | S1_16976111 | 1 | 16976111 | 165 | C | T | T | 0.64516 | C | 0.35484 | 12 | 0.3871 |
| 148 | S1_17035007 | 1 | 17035007 | 58896 | G | T | G | 0.6129 | T | 0.3871 | 12 | 0.3871 |
| 149 | S1_17076648 | 1 | 17076648 | 41641 | T | C | T | 0.72581 | C | 0.27419 | 11 | 0.35484 |
| 150 | S1_17552025 | 1 | 17552025 | 475377 | A | C | A | 0.67742 | C | 0.32258 | 12 | 0.3871 |
| 151 | S1_17552059 | 1 | 17552059 | 34 | A | G | A | 0.67742 | G | 0.32258 | 12 | 0.3871 |
| 152 | S1_17552127 | 1 | 17552127 | 68 | A | G | A | 0.67742 | G | 0.32258 | 12 | 0.3871 |
| 153 | S1_17932518 | 1 | 17932518 | 380391 | C | T | C | 0.83871 | T | 0.16129 | 8 | 0.25806 |
| 154 | S1_17932555 | 1 | 17932555 | 37 | G | A | G | 0.93548 | A | 0.06452 | 2 | 0.06452 |
| 155 | S1_18081242 | 1 | 18081242 | 148687 | G | T | T | 0.79032 | G | 0.20968 | 9 | 0.29032 |
| 156 | S1_18108847 | 1 | 18108847 | 27605 | C | T | C | 0.64516 | T | 0.35484 | 12 | 0.3871 |
| 157 | S1_18109013 | 1 | 18109013 | 166 | T | C | T | 0.75806 | C | 0.24194 | 11 | 0.35484 |
| 158 | S1_18154599 | 1 | 18154599 | 45586 | C | T | C | 0.77419 | T | 0.22581 | 8 | 0.25806 |
| 159 | S1_18208340 | 1 | 18208340 | 53741 | A | G | G | 0.67742 | A | 0.32258 | 16 | 0.51613 |
| 160 | S1_18411978 | 1 | 18411978 | 203638 | T | G | T | 0.67742 | G | 0.32258 | 12 | 0.3871 |

| Site number | SNP name | Chromosome | Physical position | Physical distance from the previous SNP | Reference allele | Alternative allele | Major allele | Major allele frequency | Minor allele | Minor allele frequency | Number heterozygous | Heterozygosity |
|---|---|---|---|---|---|---|---|---|---|---|---|---|
| 161 | S1_18412037 | 1 | 18412037 | 59 | C | T | C | 0.67742 | T | 0.32258 | 12 | 0.3871 |
| 162 | S1_18412117 | 1 | 18412117 | 80 | C | T | C | 0.67742 | T | 0.32258 | 12 | 0.3871 |
| 163 | S1_18520859 | 1 | 18520859 | 108742 | C | G | G | 0.8871 | C | 0.1129 | 7 | 0.22581 |
| 164 | S1_18520937 | 1 | 18520937 | 78 | T | A | A | 0.83871 | T | 0.16129 | 10 | 0.32258 |
| 165 | S1_18520947 | 1 | 18520947 | 10 | A | G | G | 0.90323 | A | 0.09677 | 6 | 0.19355 |
| 166 | S1_18520992 | 1 | 18520992 | 45 | G | A | A | 0.90323 | G | 0.09677 | 6 | 0.19355 |
| 167 | S1_18521003 | 1 | 18521003 | 11 | A | G | G | 0.66129 | A | 0.33871 | 13 | 0.41935 |
| 168 | S1_18521009 | 1 | 18521009 | 6 | T | C | T | 0.95161 | C | 0.04839 | 3 | 0.09677 |
| 169 | S1_19112670 | 1 | 19112670 | 591661 | C | A | C | 0.62903 | A | 0.37097 | 17 | 0.54839 |
| 170 | S1_19690992 | 1 | 19690992 | 578322 | C | A | C | 0.93548 | A | 0.06452 | 4 | 0.12903 |
| 171 | S1_19691023 | 1 | 19691023 | 31 | T | G | T | 0.75806 | G | 0.24194 | 11 | 0.35484 |
| 172 | S1_19691049 | 1 | 19691049 | 26 | A | G | A | 0.80645 | G | 0.19355 | 12 | 0.3871 |
| 173 | S1_20613081 | 1 | 20613081 | 922032 | A | C | C | 0.8871 | A | 0.1129 | 7 | 0.22581 |
| 174 | S1_20935000 | 1 | 20935000 | 321919 | A | G | A | 0.62903 | G | 0.37097 | 15 | 0.48387 |
| 175 | S1_20935011 | 1 | 20935011 | 11 | G | A | G | 0.59677 | A | 0.40323 | 15 | 0.48387 |
| 176 | S1_21710516 | 1 | 21710516 | 775505 | A | G | A | 0.85484 | G | 0.14516 | 9 | 0.29032 |
| 177 | S1_21710526 | 1 | 21710526 | 10 | C | T | T | 0.62903 | C | 0.37097 | 17 | 0.54839 |
| 178 | S1_21845839 | 1 | 21845839 | 135313 | C | T | C | 0.91935 | T | 0.08065 | 3 | 0.09677 |
| 179 | S1_21845881 | 1 | 21845881 | 42 | A | G | A | 0.85484 | G | 0.14516 | 7 | 0.22581 |
| 180 | S1_21845886 | 1 | 21845886 | 5 | C | T | C | 0.91935 | T | 0.08065 | 3 | 0.09677 |
| 181 | S1_21894543 | 1 | 21894543 | 48657 | A | G | A | 0.77419 | G | 0.22581 | 8 | 0.25806 |
| 182 | S1_21894655 | 1 | 21894655 | 112 | C | T | C | 0.8871 | T | 0.1129 | 7 | 0.22581 |
| 183 | S1_22994686 | 1 | 22994686 | 1100031 | G | C | G | 0.69355 | C | 0.30645 | 15 | 0.48387 |
| 184 | S1_22994729 | 1 | 22994729 | 43 | G | C | G | 0.83871 | C | 0.16129 | 8 | 0.25806 |
| 185 | S1_22994796 | 1 | 22994796 | 67 | G | T | G | 0.93548 | T | 0.06452 | 4 | 0.12903 |
| 186 | S1_23134007 | 1 | 23134007 | 139211 | G | A | G | 0.93548 | A | 0.06452 | 4 | 0.12903 |
| 187 | S1_23136804 | 1 | 23136804 | 2797 | G | A | G | 0.91935 | A | 0.08065 | 5 | 0.16129 |
| 188 | S1_23136832 | 1 | 23136832 | 28 | C | A | C | 0.95161 | A | 0.04839 | 3 | 0.09677 |
| 189 | S1_23136933 | 1 | 23136933 | 101 | G | A | G | 0.91935 | A | 0.08065 | 5 | 0.16129 |
| 190 | S1_23136978 | 1 | 23136978 | 45 | T | G | G | 0.69355 | T | 0.30645 | 15 | 0.48387 |
| 191 | S1_23785670 | 1 | 23785670 | 648692 | C | T | C | 0.93548 | T | 0.06452 | 4 | 0.12903 |
| 192 | S1_23785682 | 1 | 23785682 | 12 | G | A | G | 0.93548 | A | 0.06452 | 4 | 0.12903 |
| 193 | S1_23785812 | 1 | 23785812 | 130 | G | A | G | 0.93548 | A | 0.06452 | 4 | 0.12903 |
| 194 | S1_24514593 | 1 | 24514593 | 728781 | G | A | G | 0.69355 | A | 0.30645 | 15 | 0.48387 |
| 195 | S1_24514718 | 1 | 24514718 | 125 | T | C | C | 0.93548 | T | 0.06452 | 4 | 0.12903 |
| 196 | S1_24514725 | 1 | 24514725 | 7 | G | A | G | 0.69355 | A | 0.30645 | 15 | 0.48387 |
| 197 | S1_24514809 | 1 | 24514809 | 84 | T | C | T | 0.69355 | C | 0.30645 | 15 | 0.48387 |
| 198 | S1_24514830 | 1 | 24514830 | 21 | A | T | A | 0.69355 | T | 0.30645 | 15 | 0.48387 |
| 199 | S1_24848074 | 1 | 24848074 | 333244 | G | A | A | 0.90323 | G | 0.09677 | 4 | 0.12903 |
| 200 | S1_25066368 | 1 | 25066368 | 218294 | G | T | G | 0.95161 | T | 0.04839 | 3 | 0.09677 |
| 201 | S1_25066452 | 1 | 25066452 | 84 | A | G | A | 0.53226 | G | 0.46774 | 15 | 0.48387 |
| 202 | S1_25194153 | 1 | 25194153 | 127701 | A | G | G | 0.85484 | A | 0.14516 | 7 | 0.22581 |
| 203 | S1_25194157 | 1 | 25194157 | 4 | G | A | G | 0.95161 | A | 0.04839 | 3 | 0.09677 |
| 204 | S1_25934667 | 1 | 25934667 | 740510 | T | C | C | 0.6129 | T | 0.3871 | 16 | 0.51613 |
| 205 | S1_25934668 | 1 | 25934668 | 1 | G | A | A | 0.6129 | G | 0.3871 | 16 | 0.51613 |
| 206 | S1_25934755 | 1 | 25934755 | 87 | C | T | C | 0.85484 | T | 0.14516 | 5 | 0.16129 |
| 207 | S1_25934862 | 1 | 25934862 | 107 | G | A | A | 0.6129 | G | 0.3871 | 16 | 0.51613 |
| 208 | S1_25952812 | 1 | 25952812 | 17950 | C | T | C | 0.95161 | T | 0.04839 | 1 | 0.03226 |
| 209 | S1_25952861 | 1 | 25952861 | 49 | T | A | T | 0.95161 | A | 0.04839 | 1 | 0.03226 |
| 210 | S1_25962222 | 1 | 25962222 | 9361 | A | G | A | 0.93548 | G | 0.06452 | 4 | 0.12903 |
| 211 | S1_25962272 | 1 | 25962272 | 50 | C | T | T | 0.54839 | C | 0.45161 | 18 | 0.58065 |
| 212 | S1_25962287 | 1 | 25962287 | 15 | A | G | A | 0.90323 | G | 0.09677 | 6 | 0.19355 |
| 213 | S1_25962319 | 1 | 25962319 | 32 | T | A | T | 0.75806 | A | 0.24194 | 13 | 0.41935 |
| 214 | S1_26537461 | 1 | 26537461 | 575142 | C | T | C | 0.90323 | T | 0.09677 | 6 | 0.19355 |

| Site number | SNP name | Chromosome | Physical position | Physical distance from the previous SNP | Reference allele | Alternative allele | Major allele | Major allele frequency | Minor allele | Minor allele frequency | Number heterozygous | Heterozygosity |
|---|---|---|---|---|---|---|---|---|---|---|---|---|
| 215 | S1_27421861 | 1 | 27421861 | 884400 | C | T | C | 0.93548 | T | 0.06452 | 4 | 0.12903 |
| 216 | S1_28039014 | 1 | 28039014 | 617153 | T | G | T | 0.53226 | G | 0.46774 | 11 | 0.35484 |
| 217 | S1_28039039 | 1 | 28039039 | 25 | G | A | G | 0.53226 | A | 0.46774 | 11 | 0.35484 |
| 218 | S1_28039052 | 1 | 28039052 | 13 | G | A | G | 0.62903 | A | 0.37097 | 11 | 0.35484 |
| 219 | S1_28039115 | 1 | 28039115 | 63 | G | A | G | 0.90323 | A | 0.09677 | 6 | 0.19355 |
| 220 | S1_28039141 | 1 | 28039141 | 26 | C | T | C | 0.53226 | T | 0.46774 | 11 | 0.35484 |
| 221 | S1_28039143 | 1 | 28039143 | 2 | C | T | C | 0.62903 | T | 0.37097 | 11 | 0.35484 |
| 222 | S1_28039157 | 1 | 28039157 | 14 | C | T | C | 0.53226 | T | 0.46774 | 11 | 0.35484 |
| 223 | S1_28072640 | 1 | 28072640 | 33483 | A | G | A | 0.66129 | G | 0.33871 | 13 | 0.41935 |
| 224 | S1_28072709 | 1 | 28072709 | 69 | C | T | C | 0.80645 | T | 0.19355 | 10 | 0.32258 |
| 225 | S1_28072721 | 1 | 28072721 | 12 | C | T | C | 0.8871 | T | 0.1129 | 7 | 0.22581 |
| 226 | S1_28072901 | 1 | 28072901 | 180 | T | C | T | 0.64516 | C | 0.35484 | 12 | 0.3871 |
| 227 | S1_28073129 | 1 | 28073129 | 228 | C | T | C | 0.64516 | T | 0.35484 | 12 | 0.3871 |
| 228 | S1_28115980 | 1 | 28115980 | 42851 | T | C | C | 0.59677 | T | 0.40323 | 17 | 0.54839 |
| 229 | S1_28986594 | 1 | 28986594 | 870614 | C | T | C | 0.91935 | T | 0.08065 | 5 | 0.16129 |
| 230 | S1_28986648 | 1 | 28986648 | 54 | G | A | G | 0.74194 | A | 0.25806 | 10 | 0.32258 |
| 231 | S1_29011106 | 1 | 29011106 | 24458 | C | G | C | 0.90323 | G | 0.09677 | 6 | 0.19355 |
| 232 | S1_29964951 | 1 | 29964951 | 953845 | T | C | T | 0.90323 | C | 0.09677 | 6 | 0.19355 |
| 233 | S1_29965002 | 1 | 29965002 | 51 | C | T | C | 0.83871 | T | 0.16129 | 10 | 0.32258 |
| 234 | S1_29965146 | 1 | 29965146 | 144 | C | T | C | 0.93548 | T | 0.06452 | 2 | 0.06452 |
| 235 | S1_30268225 | 1 | 30268225 | 303079 | C | T | C | 0.83871 | T | 0.16129 | 10 | 0.32258 |
| 236 | S1_30268354 | 1 | 30268354 | 129 | A | G | G | 0.83871 | A | 0.16129 | 8 | 0.25806 |
| 237 | S1_30268385 | 1 | 30268385 | 31 | C | T | C | 0.90323 | T | 0.09677 | 6 | 0.19355 |
| 238 | S1_32656178 | 1 | 32656178 | 2387793 | G | T | G | 0.85484 | T | 0.14516 | 7 | 0.22581 |
| 239 | S1_32656254 | 1 | 32656254 | 76 | G | C | G | 0.93548 | C | 0.06452 | 4 | 0.12903 |
| 240 | S1_32656328 | 1 | 32656328 | 74 | G | A | G | 0.95161 | A | 0.04839 | 3 | 0.09677 |
| 241 | S1_32656349 | 1 | 32656349 | 21 | T | C | C | 0.80645 | T | 0.19355 | 10 | 0.32258 |
| 242 | S1_32656369 | 1 | 32656369 | 20 | G | A | G | 0.62903 | A | 0.37097 | 11 | 0.35484 |
| 243 | S1_32656414 | 1 | 32656414 | 45 | T | C | T | 0.85484 | C | 0.14516 | 7 | 0.22581 |
| 244 | S1_33969069 | 1 | 33969069 | 1312655 | T | C | T | 0.77419 | C | 0.22581 | 12 | 0.3871 |
| 245 | S1_34698211 | 1 | 34698211 | 729142 | C | T | C | 0.72581 | T | 0.27419 | 9 | 0.29032 |
| 246 | S1_34698230 | 1 | 34698230 | 19 | C | G | C | 0.85484 | G | 0.14516 | 7 | 0.22581 |
| 247 | S1_34924936 | 1 | 34924936 | 226706 | G | A | G | 0.90323 | A | 0.09677 | 6 | 0.19355 |
| 248 | S1_34925128 | 1 | 34925128 | 192 | C | T | T | 0.62903 | C | 0.37097 | 19 | 0.6129 |
| 249 | S1_35003833 | 1 | 35003833 | 78705 | G | A | G | 0.95161 | A | 0.04839 | 3 | 0.09677 |
| 250 | S1_35003840 | 1 | 35003840 | 7 | C | T | C | 0.95161 | T | 0.04839 | 3 | 0.09677 |
| 251 | S1_35003876 | 1 | 35003876 | 36 | C | A | C | 0.95161 | A | 0.04839 | 3 | 0.09677 |
| 252 | S1_35003891 | 1 | 35003891 | 15 | C | T | C | 0.95161 | T | 0.04839 | 3 | 0.09677 |
| 253 | S1_35003910 | 1 | 35003910 | 19 | G | C | C | 0.77419 | G | 0.22581 | 8 | 0.25806 |
| 254 | S1_35003922 | 1 | 35003922 | 12 | G | A | G | 0.95161 | A | 0.04839 | 3 | 0.09677 |
| 255 | S1_35003926 | 1 | 35003926 | 4 | G | A | G | 0.95161 | A | 0.04839 | 3 | 0.09677 |
| 256 | S1_35003935 | 1 | 35003935 | 9 | G | A | G | 0.85484 | A | 0.14516 | 5 | 0.16129 |
| 257 | S1_35003960 | 1 | 35003960 | 25 | G | T | G | 0.95161 | T | 0.04839 | 3 | 0.09677 |
| 258 | S1_35003968 | 1 | 35003968 | 8 | C | T | C | 0.95161 | T | 0.04839 | 3 | 0.09677 |
| 259 | S1_35003981 | 1 | 35003981 | 13 | C | T | C | 0.95161 | T | 0.04839 | 3 | 0.09677 |
| 260 | S1_35021872 | 1 | 35021872 | 17891 | T | C | C | 0.82258 | T | 0.17742 | 11 | 0.35484 |
| 261 | S1_35021912 | 1 | 35021912 | 40 | T | G | G | 0.70968 | T | 0.29032 | 12 | 0.3871 |
| 262 | S1_35022047 | 1 | 35022047 | 135 | A | G | A | 0.64516 | G | 0.35484 | 12 | 0.3871 |
| 263 | S1_35022118 | 1 | 35022118 | 71 | T | A | T | 0.69355 | A | 0.30645 | 11 | 0.35484 |
| 264 | S1_35405378 | 1 | 35405378 | 383260 | T | C | T | 0.72581 | C | 0.27419 | 13 | 0.41935 |
| 265 | S1_35405457 | 1 | 35405457 | 79 | A | G | G | 0.56452 | A | 0.43548 | 19 | 0.6129 |
| 266 | S1_36154778 | 1 | 36154778 | 749321 | A | T | A | 0.83871 | T | 0.16129 | 10 | 0.32258 |
| 267 | S1_36154838 | 1 | 36154838 | 60 | T | C | T | 0.8871 | C | 0.1129 | 7 | 0.22581 |
| 268 | S1_36154951 | 1 | 36154951 | 113 | G | A | G | 0.95161 | A | 0.04839 | 3 | 0.09677 |

| Site number | SNP name | Chromosome | Physical position | Physical distance from the previous SNP | Reference allele | Alternative allele | Major allele | Major allele frequency | Minor allele | Minor allele frequency | Number heterozygous | Heterozygosity |
|---|---|---|---|---|---|---|---|---|---|---|---|---|
| 269 | S1_36330849 | 1 | 36330849 | 175898 | C | T | T | 0.75806 | C | 0.24194 | 9 | 0.29032 |
| 270 | S1_37601155 | 1 | 37601155 | 1270306 | C | T | C | 0.6129 | T | 0.3871 | 16 | 0.51613 |
| 271 | S1_37601340 | 1 | 37601340 | 185 | T | C | C | 0.56452 | T | 0.43548 | 17 | 0.54839 |
| 272 | S1_37959385 | 1 | 37959385 | 358045 | C | T | C | 0.95161 | T | 0.04839 | 3 | 0.09677 |
| 273 | S1_38284856 | 1 | 38284856 | 325471 | G | A | G | 0.87097 | A | 0.12903 | 8 | 0.25806 |
| 274 | S1_38284872 | 1 | 38284872 | 16 | T | C | T | 0.79032 | C | 0.20968 | 9 | 0.29032 |
| 275 | S1_38285044 | 1 | 38285044 | 172 | G | A | G | 0.93548 | A | 0.06452 | 4 | 0.12903 |
| 276 | S1_38285075 | 1 | 38285075 | 31 | T | C | T | 0.91935 | C | 0.08065 | 5 | 0.16129 |
| 277 | S1_38285078 | 1 | 38285078 | 3 | T | C | T | 0.8871 | C | 0.1129 | 7 | 0.22581 |
| 278 | S1_38360559 | 1 | 38360559 | 75481 | T | C | T | 0.93548 | C | 0.06452 | 4 | 0.12903 |
| 279 | S1_38360582 | 1 | 38360582 | 23 | T | C | C | 0.58065 | T | 0.41935 | 18 | 0.58065 |
| 280 | S1_38360725 | 1 | 38360725 | 143 | T | C | C | 0.58065 | T | 0.41935 | 18 | 0.58065 |
| 281 | S1_38360741 | 1 | 38360741 | 16 | G | A | G | 0.91935 | A | 0.08065 | 5 | 0.16129 |
| 282 | S1_38793408 | 1 | 38793408 | 432667 | A | G | G | 0.93548 | A | 0.06452 | 4 | 0.12903 |
| 283 | S1_39377269 | 1 | 39377269 | 583861 | C | T | C | 0.87097 | T | 0.12903 | 8 | 0.25806 |
| 284 | S1_39379802 | 1 | 39379802 | 2533 | T | C | T | 0.87097 | C | 0.12903 | 8 | 0.25806 |
| 285 | S1_39379854 | 1 | 39379854 | 52 | G | A | G | 0.87097 | A | 0.12903 | 8 | 0.25806 |
| 286 | S1_39379889 | 1 | 39379889 | 35 | G | A | G | 0.77419 | A | 0.22581 | 12 | 0.3871 |
| 287 | S1_39379940 | 1 | 39379940 | 51 | G | A | G | 0.93548 | A | 0.06452 | 4 | 0.12903 |
| 288 | S1_39590409 | 1 | 39590409 | 210469 | C | T | C | 0.59677 | T | 0.40323 | 11 | 0.35484 |
| 289 | S1_39590410 | 1 | 39590410 | 1 | A | G | G | 0.70968 | A | 0.29032 | 12 | 0.3871 |
| 290 | S1_39590411 | 1 | 39590411 | 1 | C | T | C | 0.95161 | T | 0.04839 | 3 | 0.09677 |
| 291 | S1_39590508 | 1 | 39590508 | 97 | C | T | C | 0.87097 | T | 0.12903 | 8 | 0.25806 |
| 292 | S1_39762134 | 1 | 39762134 | 171626 | G | A | G | 0.91935 | A | 0.08065 | 5 | 0.16129 |
| 293 | S1_39994894 | 1 | 39994894 | 232760 | C | T | C | 0.54839 | T | 0.45161 | 10 | 0.32258 |
| 294 | S1_40052182 | 1 | 40052182 | 57288 | A | C | A | 0.87097 | C | 0.12903 | 6 | 0.19355 |
| 295 | S1_40052278 | 1 | 40052278 | 96 | G | A | A | 0.95161 | G | 0.04839 | 3 | 0.09677 |
| 296 | S1_40052307 | 1 | 40052307 | 29 | C | G | C | 0.95161 | G | 0.04839 | 3 | 0.09677 |
| 297 | S1_40052308 | 1 | 40052308 | 1 | C | A | C | 0.95161 | A | 0.04839 | 3 | 0.09677 |
| 298 | S1_40115579 | 1 | 40115579 | 63271 | A | G | A | 0.95161 | G | 0.04839 | 3 | 0.09677 |
| 299 | S1_40115592 | 1 | 40115592 | 13 | C | G | C | 0.87097 | G | 0.12903 | 8 | 0.25806 |
| 300 | S1_40549952 | 1 | 40549952 | 434360 | T | A | T | 0.69355 | A | 0.30645 | 15 | 0.48387 |
| 301 | S1_40549995 | 1 | 40549995 | 43 | G | A | G | 0.95161 | A | 0.04839 | 3 | 0.09677 |
| 302 | S1_40934903 | 1 | 40934903 | 384908 | T | C | T | 0.83871 | C | 0.16129 | 10 | 0.32258 |
| 303 | S1_40934960 | 1 | 40934960 | 57 | G | A | G | 0.85484 | A | 0.14516 | 9 | 0.29032 |
| 304 | S1_40934982 | 1 | 40934982 | 22 | A | G | A | 0.95161 | G | 0.04839 | 3 | 0.09677 |
| 305 | S1_40937168 | 1 | 40937168 | 2186 | G | A | G | 0.87097 | A | 0.12903 | 8 | 0.25806 |
| 306 | S1_41083138 | 1 | 41083138 | 145970 | C | T | C | 0.83871 | T | 0.16129 | 8 | 0.25806 |
| 307 | S1_41083158 | 1 | 41083158 | 20 | G | A | G | 0.83871 | A | 0.16129 | 8 | 0.25806 |
| 308 | S1_41193851 | 1 | 41193851 | 110693 | G | A | A | 0.90323 | G | 0.09677 | 6 | 0.19355 |
| 309 | S1_41193866 | 1 | 41193866 | 15 | T | C | C | 0.90323 | T | 0.09677 | 6 | 0.19355 |
| 310 | S1_41193916 | 1 | 41193916 | 50 | G | C | C | 0.90323 | G | 0.09677 | 6 | 0.19355 |
| 311 | S1_41193928 | 1 | 41193928 | 12 | G | A | A | 0.8871 | G | 0.1129 | 5 | 0.16129 |
| 312 | S1_41193978 | 1 | 41193978 | 50 | T | C | C | 0.90323 | T | 0.09677 | 6 | 0.19355 |
| 313 | S1_41248307 | 1 | 41248307 | 54329 | A | G | A | 0.95161 | G | 0.04839 | 3 | 0.09677 |
| 314 | S1_41280418 | 1 | 41280418 | 32111 | C | T | C | 0.90323 | T | 0.09677 | 6 | 0.19355 |
| 315 | S1_41280556 | 1 | 41280556 | 138 | A | G | A | 0.53226 | G | 0.46774 | 11 | 0.35484 |
| 316 | S1_41280667 | 1 | 41280667 | 111 | C | T | C | 0.95161 | T | 0.04839 | 3 | 0.09677 |
| 317 | S1_41280668 | 1 | 41280668 | 1 | A | G | A | 0.95161 | G | 0.04839 | 3 | 0.09677 |
| 318 | S1_41447129 | 1 | 41447129 | 166461 | C | T | C | 0.8871 | T | 0.1129 | 5 | 0.16129 |
| 319 | S1_41553560 | 1 | 41553560 | 106431 | A | G | A | 0.90323 | G | 0.09677 | 6 | 0.19355 |
| 320 | S1_41616389 | 1 | 41616389 | 62829 | T | G | T | 0.93548 | G | 0.06452 | 4 | 0.12903 |
| 321 | S1_41875303 | 1 | 41875303 | 258914 | A | G | A | 0.93548 | G | 0.06452 | 4 | 0.12903 |
| 322 | S1_41875457 | 1 | 41875457 | 154 | G | A | G | 0.75806 | A | 0.24194 | 11 | 0.35484 |

| Site number | SNP name | Chromosome | Physical position | Physical distance from the previous SNP | Reference allele | Alternative allele | Major allele | Major allele frequency | Minor allele | Minor allele frequency | Number heterozygous | Heterozygosity |
|---|---|---|---|---|---|---|---|---|---|---|---|---|
| 323 | S1_41875478 | 1 | 41875478 | 21 | A | T | T | 0.51613 | A | 0.48387 | 10 | 0.32258 |
| 324 | S1_42662002 | 1 | 42662002 | 786524 | G | A | G | 0.91935 | A | 0.08065 | 3 | 0.09677 |
| 325 | S1_42662232 | 1 | 42662232 | 230 | G | A | G | 0.8871 | A | 0.1129 | 5 | 0.16129 |
| 326 | S1_42704726 | 1 | 42704726 | 42494 | G | A | G | 0.87097 | A | 0.12903 | 8 | 0.25806 |
| 327 | S1_42704908 | 1 | 42704908 | 182 | G | A | G | 0.87097 | A | 0.12903 | 8 | 0.25806 |
| 328 | S1_42816505 | 1 | 42816505 | 111597 | T | C | T | 0.58065 | C | 0.41935 | 16 | 0.51613 |
| 329 | S1_43045100 | 1 | 43045100 | 228595 | G | T | T | 0.95161 | G | 0.04839 | 3 | 0.09677 |
| 330 | S1_43330496 | 1 | 43330496 | 285396 | T | C | T | 0.80645 | C | 0.19355 | 10 | 0.32258 |
| 331 | S1_43330550 | 1 | 43330550 | 54 | A | G | A | 0.80645 | G | 0.19355 | 10 | 0.32258 |
| 332 | S1_43681511 | 1 | 43681511 | 350961 | T | C | T | 0.95161 | C | 0.04839 | 3 | 0.09677 |
| 333 | S1_43681524 | 1 | 43681524 | 13 | T | G | T | 0.77419 | G | 0.22581 | 14 | 0.45161 |
| 334 | S1_44447389 | 1 | 44447389 | 765865 | G | A | G | 0.77419 | A | 0.22581 | 12 | 0.3871 |
| 335 | S1_44447493 | 1 | 44447493 | 104 | T | C | C | 0.62903 | T | 0.37097 | 15 | 0.48387 |
| 336 | S1_44447494 | 1 | 44447494 | 1 | G | A | G | 0.93548 | A | 0.06452 | 4 | 0.12903 |
| 337 | S1_44496572 | 1 | 44496572 | 49078 | C | G | C | 0.87097 | G | 0.12903 | 8 | 0.25806 |
| 338 | S1_44553999 | 1 | 44553999 | 57427 | C | T | C | 0.87097 | T | 0.12903 | 8 | 0.25806 |
| 339 | S1_45503234 | 1 | 45503234 | 949235 | T | C | T | 0.72581 | C | 0.27419 | 15 | 0.48387 |
| 340 | S1_45510505 | 1 | 45510505 | 7271 | C | T | T | 0.93548 | C | 0.06452 | 4 | 0.12903 |
| 341 | S1_45510506 | 1 | 45510506 | 1 | A | G | G | 0.95161 | A | 0.04839 | 3 | 0.09677 |
| 342 | S1_45510553 | 1 | 45510553 | 47 | C | T | T | 0.93548 | C | 0.06452 | 4 | 0.12903 |
| 343 | S1_45510642 | 1 | 45510642 | 89 | C | T | C | 0.93548 | T | 0.06452 | 4 | 0.12903 |
| 344 | S1_45575014 | 1 | 45575014 | 64372 | C | T | C | 0.77419 | T | 0.22581 | 10 | 0.32258 |
| 345 | S1_45817519 | 1 | 45817519 | 242505 | A | G | G | 0.77419 | A | 0.22581 | 10 | 0.32258 |
| 346 | S1_46222493 | 1 | 46222493 | 404974 | A | G | G | 0.80645 | A | 0.19355 | 8 | 0.25806 |
| 347 | S1_46222578 | 1 | 46222578 | 85 | C | T | C | 0.82258 | T | 0.17742 | 7 | 0.22581 |
| 348 | S1_46988683 | 1 | 46988683 | 766105 | T | C | T | 0.8871 | C | 0.1129 | 5 | 0.16129 |
| 349 | S1_46988684 | 1 | 46988684 | 1 | C | T | C | 0.87097 | T | 0.12903 | 4 | 0.12903 |
| 350 | S1_46988787 | 1 | 46988787 | 103 | A | G | A | 0.80645 | G | 0.19355 | 10 | 0.32258 |
| 351 | S1_47379489 | 1 | 47379489 | 390702 | C | G | C | 0.87097 | G | 0.12903 | 6 | 0.19355 |
| 352 | S1_47599544 | 1 | 47599544 | 220055 | G | C | G | 0.69355 | C | 0.30645 | 17 | 0.54839 |
| 353 | S1_47599551 | 1 | 47599551 | 7 | C | G | C | 0.69355 | G | 0.30645 | 17 | 0.54839 |
| 354 | S1_47599650 | 1 | 47599650 | 99 | A | G | A | 0.91935 | G | 0.08065 | 5 | 0.16129 |
| 355 | S1_47599657 | 1 | 47599657 | 7 | C | G | C | 0.91935 | G | 0.08065 | 5 | 0.16129 |
| 356 | S1_47599739 | 1 | 47599739 | 82 | A | G | G | 0.8871 | A | 0.1129 | 7 | 0.22581 |
| 357 | S1_47599773 | 1 | 47599773 | 34 | G | A | G | 0.69355 | A | 0.30645 | 17 | 0.54839 |
| 358 | S1_49264280 | 1 | 49264280 | 1664507 | G | A | G | 0.85484 | A | 0.14516 | 9 | 0.29032 |
| 359 | S1_50825774 | 1 | 50825774 | 1561494 | C | G | C | 0.74194 | G | 0.25806 | 8 | 0.25806 |
| 360 | S1_50825958 | 1 | 50825958 | 184 | A | C | C | 0.53226 | A | 0.46774 | 13 | 0.41935 |
| 361 | S1_50825962 | 1 | 50825962 | 4 | C | T | C | 0.64516 | T | 0.35484 | 14 | 0.45161 |
| 362 | S1_51305811 | 1 | 51305811 | 479849 | T | C | T | 0.93548 | C | 0.06452 | 4 | 0.12903 |
| 363 | S1_51374743 | 1 | 51374743 | 68932 | C | A | C | 0.95161 | A | 0.04839 | 3 | 0.09677 |
| 364 | S1_51374760 | 1 | 51374760 | 17 | A | G | A | 0.93548 | G | 0.06452 | 4 | 0.12903 |
| 365 | S1_51410774 | 1 | 51410774 | 36014 | C | T | C | 0.79032 | T | 0.20968 | 7 | 0.22581 |
| 366 | S1_51410799 | 1 | 51410799 | 25 | A | G | A | 0.93548 | G | 0.06452 | 2 | 0.06452 |
| 367 | S1_51627919 | 1 | 51627919 | 217120 | T | G | T | 0.74194 | G | 0.25806 | 10 | 0.32258 |
| 368 | S1_51627964 | 1 | 51627964 | 45 | A | G | A | 0.74194 | G | 0.25806 | 10 | 0.32258 |
| 369 | S1_51646097 | 1 | 51646097 | 18133 | C | T | C | 0.69355 | T | 0.30645 | 11 | 0.35484 |
| 370 | S1_51682439 | 1 | 51682439 | 36342 | C | T | C | 0.8871 | T | 0.1129 | 5 | 0.16129 |
| 371 | S1_51682468 | 1 | 51682468 | 29 | T | C | T | 0.85484 | C | 0.14516 | 7 | 0.22581 |
| 372 | S1_51682495 | 1 | 51682495 | 27 | T | C | C | 0.64516 | T | 0.35484 | 12 | 0.3871 |
| 373 | S1_52615641 | 1 | 52615641 | 933146 | G | T | G | 0.90323 | T | 0.09677 | 6 | 0.19355 |
| 374 | S1_52720730 | 1 | 52720730 | 105089 | T | C | C | 0.69355 | T | 0.30645 | 13 | 0.41935 |
| 375 | S1_53347659 | 1 | 53347659 | 626929 | A | T | A | 0.93548 | T | 0.06452 | 4 | 0.12903 |
| 376 | S1_53347769 | 1 | 53347769 | 110 | T | C | T | 0.90323 | C | 0.09677 | 4 | 0.12903 |

| Site number | SNP name | Chromosome | Physical position | Physical distance from the previous SNP | Reference allele | Alternative allele | Major allele | Major allele frequency | Minor allele | Minor allele frequency | Number heterozygous | Heterozygosity |
|---|---|---|---|---|---|---|---|---|---|---|---|---|
| 377 | S1_53347773 | 1 | 53347773 | 4 | C | T | T | 0.58065 | C | 0.41935 | 20 | 0.64516 |
| 378 | S1_53347866 | 1 | 53347866 | 93 | C | G | C | 0.93548 | G | 0.06452 | 4 | 0.12903 |
| 379 | S1_53724369 | 1 | 53724369 | 376503 | C | T | C | 0.79032 | T | 0.20968 | 9 | 0.29032 |
| 380 | S1_53724501 | 1 | 53724501 | 132 | T | C | C | 0.67742 | T | 0.32258 | 10 | 0.32258 |
| 381 | S1_53784838 | 1 | 53784838 | 60337 | A | G | A | 0.95161 | G | 0.04839 | 3 | 0.09677 |
| 382 | S1_53784900 | 1 | 53784900 | 62 | T | C | C | 0.74194 | T | 0.25806 | 12 | 0.3871 |
| 383 | S1_53950902 | 1 | 53950902 | 166002 | G | A | G | 0.67742 | A | 0.32258 | 12 | 0.3871 |
| 384 | S1_53950947 | 1 | 53950947 | 45 | C | T | T | 0.90323 | C | 0.09677 | 6 | 0.19355 |
| 385 | S1_54022114 | 1 | 54022114 | 71167 | C | T | C | 0.91935 | T | 0.08065 | 5 | 0.16129 |
| 386 | S1_54347809 | 1 | 54347809 | 325695 | A | G | A | 0.79032 | G | 0.20968 | 9 | 0.29032 |
| 387 | S1_54976911 | 1 | 54976911 | 629102 | C | T | C | 0.79032 | T | 0.20968 | 11 | 0.35484 |
| 388 | S1_54976918 | 1 | 54976918 | 7 | C | A | C | 0.67742 | A | 0.32258 | 16 | 0.51613 |
| 389 | S1_54977015 | 1 | 54977015 | 97 | G | A | G | 0.67742 | A | 0.32258 | 16 | 0.51613 |
| 390 | S1_55553706 | 1 | 55553706 | 576691 | A | G | A | 0.70968 | G | 0.29032 | 14 | 0.45161 |
| 391 | S1_55706686 | 1 | 55706686 | 152980 | C | T | C | 0.93548 | T | 0.06452 | 4 | 0.12903 |
| 392 | S1_56086844 | 1 | 56086844 | 380158 | G | A | G | 0.91935 | A | 0.08065 | 5 | 0.16129 |
| 393 | S1_56086851 | 1 | 56086851 | 7 | G | C | G | 0.95161 | C | 0.04839 | 3 | 0.09677 |
| 394 | S1_56086968 | 1 | 56086968 | 117 | G | A | G | 0.85484 | A | 0.14516 | 7 | 0.22581 |
| 395 | S1_56377905 | 1 | 56377905 | 290937 | C | T | C | 0.69355 | T | 0.30645 | 11 | 0.35484 |
| 396 | S1_56377997 | 1 | 56377997 | 92 | C | A | C | 0.69355 | A | 0.30645 | 13 | 0.41935 |
| 397 | S1_56378022 | 1 | 56378022 | 25 | C | A | C | 0.93548 | A | 0.06452 | 4 | 0.12903 |
| 398 | S1_56452890 | 1 | 56452890 | 74868 | T | C | T | 0.87097 | C | 0.12903 | 8 | 0.25806 |
| 399 | S1_56614474 | 1 | 56614474 | 161584 | C | T | C | 0.83871 | T | 0.16129 | 8 | 0.25806 |
| 400 | S1_56614523 | 1 | 56614523 | 49 | C | T | C | 0.64516 | T | 0.35484 | 14 | 0.45161 |
| 401 | S1_56740163 | 1 | 56740163 | 125640 | A | G | A | 0.82258 | G | 0.17742 | 7 | 0.22581 |
| 402 | S1_57134316 | 1 | 57134316 | 394153 | T | C | T | 0.70968 | C | 0.29032 | 12 | 0.3871 |
| 403 | S1_57169421 | 1 | 57169421 | 35105 | T | G | T | 0.93548 | G | 0.06452 | 4 | 0.12903 |
| 404 | S1_57509872 | 1 | 57509872 | 340451 | G | A | A | 0.80645 | G | 0.19355 | 8 | 0.25806 |
| 405 | S1_58207947 | 1 | 58207947 | 698075 | C | G | C | 0.59677 | G | 0.40323 | 13 | 0.41935 |
| 406 | S1_58365552 | 1 | 58365552 | 157605 | C | T | C | 0.91935 | T | 0.08065 | 5 | 0.16129 |
| 407 | S1_58365650 | 1 | 58365650 | 98 | T | A | T | 0.91935 | A | 0.08065 | 5 | 0.16129 |
| 408 | S1_58490209 | 1 | 58490209 | 124559 | C | T | T | 0.66129 | C | 0.33871 | 17 | 0.54839 |
| 409 | S1_59879226 | 1 | 59879226 | 1389017 | C | T | T | 0.85484 | C | 0.14516 | 9 | 0.29032 |
| 410 | S1_59879249 | 1 | 59879249 | 23 | C | G | C | 0.93548 | G | 0.06452 | 4 | 0.12903 |
| 411 | S1_59879292 | 1 | 59879292 | 43 | G | A | G | 0.91935 | A | 0.08065 | 5 | 0.16129 |
| 412 | S1_60578605 | 1 | 60578605 | 699313 | G | T | G | 0.93548 | T | 0.06452 | 4 | 0.12903 |
| 413 | S1_60578625 | 1 | 60578625 | 20 | G | T | G | 0.91935 | T | 0.08065 | 5 | 0.16129 |
| 414 | S1_60578653 | 1 | 60578653 | 28 | G | C | C | 0.5 | G | 0.5 | 19 | 0.6129 |
| 415 | S1_60622901 | 1 | 60622901 | 44248 | T | A | T | 0.85484 | A | 0.14516 | 7 | 0.22581 |
| 416 | S1_60622932 | 1 | 60622932 | 31 | A | G | A | 0.93548 | G | 0.06452 | 4 | 0.12903 |
| 417 | S1_60622961 | 1 | 60622961 | 29 | C | A | C | 0.87097 | A | 0.12903 | 6 | 0.19355 |
| 418 | S1_60623029 | 1 | 60623029 | 68 | G | T | G | 0.70968 | T | 0.29032 | 12 | 0.3871 |
| 419 | S1_60702598 | 1 | 60702598 | 79569 | A | G | A | 0.77419 | G | 0.22581 | 14 | 0.45161 |
| 420 | S1_60702621 | 1 | 60702621 | 23 | G | C | G | 0.90323 | C | 0.09677 | 4 | 0.12903 |
| 421 | S1_60702671 | 1 | 60702671 | 50 | G | A | G | 0.77419 | A | 0.22581 | 12 | 0.3871 |
| 422 | S1_60702796 | 1 | 60702796 | 125 | C | T | C | 0.74194 | T | 0.25806 | 14 | 0.45161 |
| 423 | S1_60772701 | 1 | 60772701 | 69905 | C | A | C | 0.74194 | A | 0.25806 | 12 | 0.3871 |
| 424 | S1_60772814 | 1 | 60772814 | 113 | G | A | G | 0.70968 | A | 0.29032 | 12 | 0.3871 |
| 425 | S1_60839353 | 1 | 60839353 | 66539 | G | A | G | 0.93548 | A | 0.06452 | 4 | 0.12903 |
| 426 | S1_60839470 | 1 | 60839470 | 117 | C | T | T | 0.91935 | C | 0.08065 | 5 | 0.16129 |
| 427 | S1_61755681 | 1 | 61755681 | 916211 | C | T | C | 0.80645 | T | 0.19355 | 10 | 0.32258 |
| 428 | S1_61903828 | 1 | 61903828 | 148147 | T | C | T | 0.90323 | C | 0.09677 | 6 | 0.19355 |
| 429 | S1_61904016 | 1 | 61904016 | 188 | C | T | T | 0.64516 | C | 0.35484 | 10 | 0.32258 |
| 430 | S1_62317323 | 1 | 62317323 | 413307 | T | A | T | 0.72581 | A | 0.27419 | 11 | 0.35484 |

| Site number | SNP name | Chromosome | Physical position | Physical distance from the previous SNP | Reference allele | Alternative allele | Major allele | Major allele frequency | Minor allele | Minor allele frequency | Number heterozygous | Heterozygosity |
|---|---|---|---|---|---|---|---|---|---|---|---|---|
| 431 | S1_62317485 | 1 | 62317485 | 162 | G | A | G | 0.95161 | A | 0.04839 | 3 | 0.09677 |
| 432 | S1_62807289 | 1 | 62807289 | 489804 | A | G | G | 0.64516 | A | 0.35484 | 16 | 0.51613 |
| 433 | S1_62807323 | 1 | 62807323 | 34 | T | C | C | 0.93548 | T | 0.06452 | 4 | 0.12903 |
| 434 | S1_62807477 | 1 | 62807477 | 154 | T | C | T | 0.93548 | C | 0.06452 | 4 | 0.12903 |
| 435 | S1_62852598 | 1 | 62852598 | 45121 | C | T | C | 0.8871 | T | 0.1129 | 7 | 0.22581 |
| 436 | S1_63197528 | 1 | 63197528 | 344930 | A | T | A | 0.70968 | T | 0.29032 | 8 | 0.25806 |
| 437 | S1_63227940 | 1 | 63227940 | 30412 | T | A | T | 0.87097 | A | 0.12903 | 6 | 0.19355 |
| 438 | S1_63227993 | 1 | 63227993 | 53 | G | A | G | 0.83871 | A | 0.16129 | 10 | 0.32258 |
| 439 | S1_63228005 | 1 | 63228005 | 12 | C | A | C | 0.90323 | A | 0.09677 | 6 | 0.19355 |
| 440 | S1_63228086 | 1 | 63228086 | 81 | C | T | C | 0.82258 | T | 0.17742 | 11 | 0.35484 |
| 441 | S1_63228107 | 1 | 63228107 | 21 | G | C | G | 0.85484 | C | 0.14516 | 9 | 0.29032 |
| 442 | S1_63228135 | 1 | 63228135 | 28 | G | A | G | 0.85484 | A | 0.14516 | 9 | 0.29032 |
| 443 | S1_63228161 | 1 | 63228161 | 26 | G | T | G | 0.85484 | T | 0.14516 | 9 | 0.29032 |
| 444 | S1_63372485 | 1 | 63372485 | 144324 | G | A | G | 0.90323 | A | 0.09677 | 6 | 0.19355 |
| 445 | S1_63372618 | 1 | 63372618 | 133 | A | T | A | 0.90323 | T | 0.09677 | 6 | 0.19355 |
| 446 | S1_63372645 | 1 | 63372645 | 27 | G | A | G | 0.91935 | A | 0.08065 | 5 | 0.16129 |
| 447 | S1_63602007 | 1 | 63602007 | 229362 | G | A | G | 0.87097 | A | 0.12903 | 8 | 0.25806 |
| 448 | S1_63602049 | 1 | 63602049 | 42 | A | C | C | 0.93548 | A | 0.06452 | 4 | 0.12903 |
| 449 | S1_63998789 | 1 | 63998789 | 396740 | G | A | G | 0.87097 | A | 0.12903 | 8 | 0.25806 |
| 450 | S1_64227212 | 1 | 64227212 | 228423 | G | A | G | 0.59677 | A | 0.40323 | 19 | 0.6129 |
| 451 | S1_64227266 | 1 | 64227266 | 54 | A | G | A | 0.58065 | G | 0.41935 | 20 | 0.64516 |
| 452 | S1_64227386 | 1 | 64227386 | 120 | G | A | G | 0.58065 | A | 0.41935 | 20 | 0.64516 |
| 453 | S1_64460725 | 1 | 64460725 | 233339 | C | T | C | 0.79032 | T | 0.20968 | 13 | 0.41935 |
| 454 | S1_64460800 | 1 | 64460800 | 75 | G | A | G | 0.91935 | A | 0.08065 | 3 | 0.09677 |
| 455 | S1_64460804 | 1 | 64460804 | 4 | C | T | C | 0.90323 | T | 0.09677 | 4 | 0.12903 |
| 456 | S1_65176215 | 1 | 65176215 | 715411 | C | A | A | 0.75806 | C | 0.24194 | 11 | 0.35484 |
| 457 | S1_65176340 | 1 | 65176340 | 125 | G | A | G | 0.77419 | A | 0.22581 | 12 | 0.3871 |
| 458 | S1_65228864 | 1 | 65228864 | 52524 | C | T | C | 0.91935 | T | 0.08065 | 5 | 0.16129 |
| 459 | S1_65229005 | 1 | 65229005 | 141 | C | T | C | 0.66129 | T | 0.33871 | 13 | 0.41935 |
| 460 | S1_65653209 | 1 | 65653209 | 424204 | T | G | T | 0.64516 | G | 0.35484 | 18 | 0.58065 |
| 461 | S1_65829981 | 1 | 65829981 | 176772 | G | A | G | 0.93548 | A | 0.06452 | 2 | 0.06452 |
| 462 | S1_66168439 | 1 | 66168439 | 338458 | C | T | C | 0.62903 | T | 0.37097 | 17 | 0.54839 |
| 463 | S1_66168473 | 1 | 66168473 | 34 | G | A | G | 0.62903 | A | 0.37097 | 17 | 0.54839 |
| 464 | S1_66168502 | 1 | 66168502 | 29 | C | T | C | 0.93548 | T | 0.06452 | 4 | 0.12903 |
| 465 | S1_66759221 | 1 | 66759221 | 590719 | G | A | G | 0.85484 | A | 0.14516 | 5 | 0.16129 |
| 466 | S1_66759234 | 1 | 66759234 | 13 | A | C | A | 0.85484 | C | 0.14516 | 5 | 0.16129 |
| 467 | S1_66759284 | 1 | 66759284 | 50 | C | G | C | 0.85484 | G | 0.14516 | 5 | 0.16129 |
| 468 | S1_66759285 | 1 | 66759285 | 1 | C | T | C | 0.93548 | T | 0.06452 | 4 | 0.12903 |
| 469 | S1_66759292 | 1 | 66759292 | 7 | C | T | C | 0.91935 | T | 0.08065 | 3 | 0.09677 |
| 470 | S1_66759437 | 1 | 66759437 | 145 | C | T | C | 0.85484 | T | 0.14516 | 5 | 0.16129 |
| 471 | S1_66810261 | 1 | 66810261 | 50824 | C | T | C | 0.8871 | T | 0.1129 | 7 | 0.22581 |
| 472 | S1_66810381 | 1 | 66810381 | 120 | T | G | T | 0.8871 | G | 0.1129 | 7 | 0.22581 |
| 473 | S1_66810384 | 1 | 66810384 | 3 | G | A | G | 0.93548 | A | 0.06452 | 4 | 0.12903 |
| 474 | S1_66810397 | 1 | 66810397 | 13 | C | T | C | 0.8871 | T | 0.1129 | 7 | 0.22581 |
| 475 | S1_66810421 | 1 | 66810421 | 24 | C | T | C | 0.91935 | T | 0.08065 | 3 | 0.09677 |
| 476 | S1_66882487 | 1 | 66882487 | 72066 | C | A | C | 0.90323 | A | 0.09677 | 6 | 0.19355 |
| 477 | S1_66894322 | 1 | 66894322 | 11835 | T | C | T | 0.93548 | C | 0.06452 | 4 | 0.12903 |
| 478 | S1_66894340 | 1 | 66894340 | 18 | G | A | G | 0.93548 | A | 0.06452 | 4 | 0.12903 |
| 479 | S1_66894515 | 1 | 66894515 | 175 | G | A | G | 0.90323 | A | 0.09677 | 6 | 0.19355 |
| 480 | S1_67179613 | 1 | 67179613 | 285098 | G | A | G | 0.87097 | A | 0.12903 | 8 | 0.25806 |
| 481 | S1_67179637 | 1 | 67179637 | 24 | G | A | G | 0.95161 | A | 0.04839 | 3 | 0.09677 |
| 482 | S1_67752165 | 1 | 67752165 | 572528 | G | A | A | 0.5 | G | 0.5 | 15 | 0.48387 |
| 483 | S1_67752217 | 1 | 67752217 | 52 | C | T | C | 0.8871 | T | 0.1129 | 7 | 0.22581 |
| 484 | S1_67855942 | 1 | 67855942 | 103725 | G | A | G | 0.90323 | A | 0.09677 | 6 | 0.19355 |

| Site number | SNP name | Chromosome | Physical position | Physical distance from the previous SNP | Reference allele | Alternative allele | Major allele | Major allele frequency | Minor allele | Minor allele frequency | Number heterozygous | Heterozygosity |
|---|---|---|---|---|---|---|---|---|---|---|---|---|
| 485 | S1_67855980 | 1 | 67855980 | 38 | C | T | C | 0.8871 | T | 0.1129 | 5 | 0.16129 |
| 486 | S1_67856011 | 1 | 67856011 | 31 | A | G | A | 0.8871 | G | 0.1129 | 5 | 0.16129 |
| 487 | S1_67856020 | 1 | 67856020 | 9 | G | A | G | 0.8871 | A | 0.1129 | 5 | 0.16129 |
| 488 | S1_67856052 | 1 | 67856052 | 32 | A | G | A | 0.8871 | G | 0.1129 | 5 | 0.16129 |
| 489 | S1_67856156 | 1 | 67856156 | 104 | T | C | T | 0.75806 | C | 0.24194 | 15 | 0.48387 |
| 490 | S1_67874474 | 1 | 67874474 | 18318 | A | C | A | 0.93548 | C | 0.06452 | 4 | 0.12903 |
| 491 | S1_68155897 | 1 | 68155897 | 281423 | C | T | C | 0.93548 | T | 0.06452 | 4 | 0.12903 |
| 492 | S1_68297267 | 1 | 68297267 | 141370 | C | T | C | 0.93548 | T | 0.06452 | 4 | 0.12903 |
| 493 | S1_68389596 | 1 | 68389596 | 92329 | G | A | G | 0.90323 | A | 0.09677 | 6 | 0.19355 |
| 494 | S1_68389643 | 1 | 68389643 | 47 | T | C | T | 0.67742 | C | 0.32258 | 18 | 0.58065 |
| 495 | S1_68512678 | 1 | 68512678 | 123035 | A | G | A | 0.69355 | G | 0.30645 | 17 | 0.54839 |
| 496 | S1_68512782 | 1 | 68512782 | 104 | A | G | A | 0.69355 | G | 0.30645 | 17 | 0.54839 |
| 497 | S1_68524491 | 1 | 68524491 | 11709 | A | G | A | 0.90323 | G | 0.09677 | 6 | 0.19355 |
| 498 | S1_68816029 | 1 | 68816029 | 291538 | C | T | C | 0.87097 | T | 0.12903 | 8 | 0.25806 |
| 499 | S1_68873505 | 1 | 68873505 | 57476 | T | C | T | 0.69355 | C | 0.30645 | 15 | 0.48387 |
| 500 | S1_68990344 | 1 | 68990344 | 116839 | A | T | T | 0.83871 | A | 0.16129 | 10 | 0.32258 |
| 501 | S1_69086867 | 1 | 69086867 | 96523 | G | A | G | 0.93548 | A | 0.06452 | 4 | 0.12903 |
| 502 | S1_69307389 | 1 | 69307389 | 220522 | T | C | T | 0.93548 | C | 0.06452 | 4 | 0.12903 |
| 503 | S1_69307489 | 1 | 69307489 | 100 | C | A | C | 0.93548 | A | 0.06452 | 4 | 0.12903 |
| 504 | S1_69307539 | 1 | 69307539 | 50 | C | A | C | 0.93548 | A | 0.06452 | 4 | 0.12903 |
| 505 | S1_69395007 | 1 | 69395007 | 87468 | T | C | T | 0.91935 | C | 0.08065 | 5 | 0.16129 |
| 506 | S1_69395119 | 1 | 69395119 | 112 | C | G | C | 0.80645 | G | 0.19355 | 8 | 0.25806 |
| 507 | S1_70142515 | 1 | 70142515 | 747396 | T | C | T | 0.85484 | C | 0.14516 | 9 | 0.29032 |
| 508 | S1_70142521 | 1 | 70142521 | 6 | G | A | G | 0.95161 | A | 0.04839 | 3 | 0.09677 |
| 509 | S1_70350790 | 1 | 70350790 | 208269 | T | C | T | 0.91935 | C | 0.08065 | 5 | 0.16129 |
| 510 | S1_70350887 | 1 | 70350887 | 97 | C | T | C | 0.8871 | T | 0.1129 | 7 | 0.22581 |
| 511 | S1_70350931 | 1 | 70350931 | 44 | G | A | A | 0.72581 | G | 0.27419 | 9 | 0.29032 |
| 512 | S1_70920762 | 1 | 70920762 | 569831 | A | T | A | 0.93548 | T | 0.06452 | 4 | 0.12903 |
| 513 | S1_70977118 | 1 | 70977118 | 56356 | C | T | C | 0.90323 | T | 0.09677 | 6 | 0.19355 |
| 514 | S1_71575085 | 1 | 71575085 | 597967 | C | A | C | 0.85484 | A | 0.14516 | 9 | 0.29032 |
| 515 | S1_71678477 | 1 | 71678477 | 103392 | T | A | T | 0.66129 | A | 0.33871 | 13 | 0.41935 |
| 516 | S1_71678480 | 1 | 71678480 | 3 | C | A | C | 0.66129 | A | 0.33871 | 13 | 0.41935 |
| 517 | S1_71678497 | 1 | 71678497 | 17 | A | G | A | 0.79032 | G | 0.20968 | 13 | 0.41935 |
| 518 | S1_71678503 | 1 | 71678503 | 6 | C | A | A | 0.93548 | C | 0.06452 | 2 | 0.06452 |
| 519 | S1_71678626 | 1 | 71678626 | 123 | G | A | G | 0.93548 | A | 0.06452 | 4 | 0.12903 |
| 520 | S1_72460603 | 1 | 72460603 | 781977 | C | A | C | 0.59677 | A | 0.40323 | 19 | 0.6129 |
| 521 | S1_72738249 | 1 | 72738249 | 277646 | T | C | T | 0.58065 | C | 0.41935 | 12 | 0.3871 |
| 522 | S1_72856002 | 1 | 72856002 | 117753 | C | T | C | 0.83871 | T | 0.16129 | 8 | 0.25806 |
| 523 | S1_73852721 | 1 | 73852721 | 996719 | T | C | T | 0.95161 | C | 0.04839 | 3 | 0.09677 |
| 524 | S1_73938815 | 1 | 73938815 | 86094 | C | T | C | 0.85484 | T | 0.14516 | 9 | 0.29032 |
| 525 | S1_74331087 | 1 | 74331087 | 392272 | G | A | G | 0.87097 | A | 0.12903 | 6 | 0.19355 |
| 526 | S1_74331114 | 1 | 74331114 | 27 | C | T | C | 0.95161 | T | 0.04839 | 3 | 0.09677 |
| 527 | S1_74665219 | 1 | 74665219 | 334105 | C | T | T | 0.82258 | C | 0.17742 | 11 | 0.35484 |
| 528 | S1_74665259 | 1 | 74665259 | 40 | A | C | C | 0.8871 | A | 0.1129 | 7 | 0.22581 |
| 529 | S1_75887905 | 1 | 75887905 | 1222646 | C | T | C | 0.90323 | T | 0.09677 | 4 | 0.12903 |
| 530 | S1_75887935 | 1 | 75887935 | 30 | T | C | T | 0.77419 | C | 0.22581 | 10 | 0.32258 |
| 531 | S1_75887952 | 1 | 75887952 | 17 | A | G | G | 0.87097 | A | 0.12903 | 8 | 0.25806 |
| 532 | S1_75887959 | 1 | 75887959 | 7 | G | T | G | 0.54839 | T | 0.45161 | 16 | 0.51613 |
| 533 | S1_75887981 | 1 | 75887981 | 22 | T | C | T | 0.87097 | C | 0.12903 | 8 | 0.25806 |
| 534 | S1_75887982 | 1 | 75887982 | 1 | G | A | G | 0.87097 | A | 0.12903 | 8 | 0.25806 |
| 535 | S1_75887984 | 1 | 75887984 | 2 | A | C | A | 0.87097 | C | 0.12903 | 8 | 0.25806 |
| 536 | S1_75888022 | 1 | 75888022 | 38 | T | C | T | 0.87097 | C | 0.12903 | 8 | 0.25806 |
| 537 | S1_75888053 | 1 | 75888053 | 31 | A | G | A | 0.77419 | G | 0.22581 | 10 | 0.32258 |
| 538 | S1_75963642 | 1 | 75963642 | 75589 | T | G | T | 0.91935 | G | 0.08065 | 5 | 0.16129 |

| Site number | SNP name | Chromosome | Physical position | Physical distance from the previous SNP | Reference allele | Alternative allele | Major allele | Major allele frequency | Minor allele | Minor allele frequency | Number heterozygous | Heterozygosity |
|---|---|---|---|---|---|---|---|---|---|---|---|---|
| 539 | S1_75963713 | 1 | 75963713 | 71 | G | A | G | 0.91935 | A | 0.08065 | 5 | 0.16129 |
| 540 | S1_75963749 | 1 | 75963749 | 36 | T | C | T | 0.91935 | C | 0.08065 | 5 | 0.16129 |
| 541 | S1_75963761 | 1 | 75963761 | 12 | C | T | C | 0.95161 | T | 0.04839 | 3 | 0.09677 |
| 542 | S1_75963782 | 1 | 75963782 | 21 | G | A | G | 0.91935 | A | 0.08065 | 5 | 0.16129 |
| 543 | S1_76022249 | 1 | 76022249 | 58467 | T | A | T | 0.90323 | A | 0.09677 | 4 | 0.12903 |
| 544 | S1_76022251 | 1 | 76022251 | 2 | A | C | A | 0.90323 | C | 0.09677 | 4 | 0.12903 |
| 545 | S1_76343563 | 1 | 76343563 | 321312 | T | A | T | 0.74194 | A | 0.25806 | 10 | 0.32258 |
| 546 | S1_76343576 | 1 | 76343576 | 13 | C | T | C | 0.87097 | T | 0.12903 | 6 | 0.19355 |
| 547 | S1_76378004 | 1 | 76378004 | 34428 | A | G | A | 0.95161 | G | 0.04839 | 1 | 0.03226 |
| 548 | S1_77112244 | 1 | 77112244 | 734240 | C | T | T | 0.64516 | C | 0.35484 | 16 | 0.51613 |
| 549 | S1_77145420 | 1 | 77145420 | 33176 | G | A | A | 0.75806 | G | 0.24194 | 13 | 0.41935 |
| 550 | S1_77145424 | 1 | 77145424 | 4 | T | C | T | 0.90323 | C | 0.09677 | 6 | 0.19355 |
| 551 | S1_77585671 | 1 | 77585671 | 440247 | A | C | A | 0.83871 | C | 0.16129 | 8 | 0.25806 |
| 552 | S1_77585757 | 1 | 77585757 | 86 | T | C | T | 0.79032 | C | 0.20968 | 11 | 0.35484 |
| 553 | S1_77585787 | 1 | 77585787 | 30 | C | T | C | 0.79032 | T | 0.20968 | 11 | 0.35484 |
| 554 | S1_77699355 | 1 | 77699355 | 113568 | T | C | T | 0.93548 | C | 0.06452 | 4 | 0.12903 |
| 555 | S1_77716701 | 1 | 77716701 | 17346 | C | T | C | 0.54839 | T | 0.45161 | 20 | 0.64516 |
| 556 | S1_77716731 | 1 | 77716731 | 30 | G | A | G | 0.95161 | A | 0.04839 | 3 | 0.09677 |
| 557 | S1_78537659 | 1 | 78537659 | 820928 | G | A | G | 0.59677 | A | 0.40323 | 19 | 0.6129 |
| 558 | S1_78744714 | 1 | 78744714 | 207055 | T | C | T | 0.8871 | C | 0.1129 | 7 | 0.22581 |
| 559 | S1_78744892 | 1 | 78744892 | 178 | G | A | G | 0.69355 | A | 0.30645 | 11 | 0.35484 |
| 560 | S1_78793122 | 1 | 78793122 | 48230 | T | C | T | 0.72581 | C | 0.27419 | 13 | 0.41935 |
| 561 | S1_78793253 | 1 | 78793253 | 131 | G | A | G | 0.90323 | A | 0.09677 | 6 | 0.19355 |
| 562 | S1_79363427 | 1 | 79363427 | 570174 | C | T | T | 0.54839 | C | 0.45161 | 12 | 0.3871 |
| 563 | S1_79363477 | 1 | 79363477 | 50 | A | G | G | 0.56452 | A | 0.43548 | 13 | 0.41935 |
| 564 | S1_80104315 | 1 | 80104315 | 740838 | C | T | C | 0.85484 | T | 0.14516 | 5 | 0.16129 |
| 565 | S1_80239624 | 1 | 80239624 | 135309 | G | A | A | 0.90323 | G | 0.09677 | 6 | 0.19355 |
| 566 | S1_80239641 | 1 | 80239641 | 17 | T | C | T | 0.90323 | C | 0.09677 | 4 | 0.12903 |
| 567 | S1_80239716 | 1 | 80239716 | 75 | C | T | C | 0.95161 | T | 0.04839 | 3 | 0.09677 |
| 568 | S1_80714321 | 1 | 80714321 | 474605 | G | A | G | 0.79032 | A | 0.20968 | 9 | 0.29032 |
| 569 | S1_81082596 | 1 | 81082596 | 368275 | T | A | T | 0.90323 | A | 0.09677 | 6 | 0.19355 |
| 570 | S1_81163103 | 1 | 81163103 | 80507 | G | A | A | 0.87097 | G | 0.12903 | 8 | 0.25806 |
| 571 | S1_81163153 | 1 | 81163153 | 50 | C | A | A | 0.87097 | C | 0.12903 | 8 | 0.25806 |
| 572 | S1_81606624 | 1 | 81606624 | 443471 | T | A | T | 0.93548 | A | 0.06452 | 4 | 0.12903 |
| 573 | S1_81606708 | 1 | 81606708 | 84 | T | C | C | 0.64516 | T | 0.35484 | 14 | 0.45161 |
| 574 | S1_81606854 | 1 | 81606854 | 146 | A | G | G | 0.74194 | A | 0.25806 | 12 | 0.3871 |
| 575 | S1_81805215 | 1 | 81805215 | 198361 | A | C | A | 0.83871 | C | 0.16129 | 6 | 0.19355 |
| 576 | S1_81829876 | 1 | 81829876 | 24661 | C | T | C | 0.91935 | T | 0.08065 | 5 | 0.16129 |
| 577 | S1_82031741 | 1 | 82031741 | 201865 | C | T | C | 0.93548 | T | 0.06452 | 4 | 0.12903 |
| 578 | S1_82684312 | 1 | 82684312 | 652571 | C | T | C | 0.95161 | T | 0.04839 | 3 | 0.09677 |
| 579 | S1_82684326 | 1 | 82684326 | 14 | G | A | G | 0.95161 | A | 0.04839 | 3 | 0.09677 |
| 580 | S1_83176820 | 1 | 83176820 | 492494 | C | T | T | 0.77419 | C | 0.22581 | 12 | 0.3871 |
| 581 | S1_83440631 | 1 | 83440631 | 263811 | G | A | G | 0.93548 | A | 0.06452 | 4 | 0.12903 |
| 582 | S1_83440743 | 1 | 83440743 | 112 | C | T | C | 0.74194 | T | 0.25806 | 14 | 0.45161 |
| 583 | S1_83440789 | 1 | 83440789 | 46 | G | A | G | 0.87097 | A | 0.12903 | 6 | 0.19355 |
| 584 | S1_83440806 | 1 | 83440806 | 17 | C | T | C | 0.87097 | T | 0.12903 | 6 | 0.19355 |
| 585 | S1_84067949 | 1 | 84067949 | 627143 | C | T | C | 0.72581 | T | 0.27419 | 13 | 0.41935 |
| 586 | S1_84068008 | 1 | 84068008 | 59 | T | A | T | 0.82258 | A | 0.17742 | 9 | 0.29032 |
| 587 | S1_84068114 | 1 | 84068114 | 106 | G | T | G | 0.82258 | T | 0.17742 | 9 | 0.29032 |
| 588 | S1_84068148 | 1 | 84068148 | 34 | G | A | G | 0.87097 | A | 0.12903 | 8 | 0.25806 |
| 589 | S1_84068156 | 1 | 84068156 | 8 | A | G | A | 0.80645 | G | 0.19355 | 8 | 0.25806 |
| 590 | S1_87040099 | 1 | 87040099 | 2971943 | G | A | G | 0.82258 | A | 0.17742 | 11 | 0.35484 |
| 591 | S1_87155589 | 1 | 87155589 | 115490 | T | G | T | 0.54839 | G | 0.45161 | 16 | 0.51613 |
| 592 | S1_87155642 | 1 | 87155642 | 53 | C | T | C | 0.91935 | T | 0.08065 | 3 | 0.09677 |

| Site number | SNP name | Chromosome | Physical position | Physical distance from the previous SNP | Reference allele | Alternative allele | Major allele | Major allele frequency | Minor allele | Minor allele frequency | Number heterozygous | Heterozygosity |
|---|---|---|---|---|---|---|---|---|---|---|---|---|
| 593 | S1_87566088 | 1 | 87566088 | 410446 | T | C | T | 0.87097 | C | 0.12903 | 6 | 0.19355 |
| 594 | S1_87566223 | 1 | 87566223 | 135 | C | T | C | 0.93548 | T | 0.06452 | 4 | 0.12903 |
| 595 | S1_88569378 | 1 | 88569378 | 1003155 | G | A | G | 0.69355 | A | 0.30645 | 13 | 0.41935 |
| 596 | S1_88569521 | 1 | 88569521 | 143 | C | T | C | 0.93548 | T | 0.06452 | 4 | 0.12903 |
| 597 | S1_88569580 | 1 | 88569580 | 59 | C | A | A | 0.87097 | C | 0.12903 | 6 | 0.19355 |
| 598 | S1_89148448 | 1 | 89148448 | 578868 | A | C | A | 0.70968 | C | 0.29032 | 16 | 0.51613 |
| 599 | S1_89148496 | 1 | 89148496 | 48 | A | G | A | 0.69355 | G | 0.30645 | 15 | 0.48387 |
| 600 | S1_89421788 | 1 | 89421788 | 273292 | G | A | G | 0.66129 | A | 0.33871 | 13 | 0.41935 |
| 601 | S1_89461450 | 1 | 89461450 | 39662 | G | A | G | 0.80645 | A | 0.19355 | 8 | 0.25806 |
| 602 | S1_89551204 | 1 | 89551204 | 89754 | T | C | T | 0.91935 | C | 0.08065 | 5 | 0.16129 |
| 603 | S1_89551408 | 1 | 89551408 | 204 | C | T | C | 0.90323 | T | 0.09677 | 4 | 0.12903 |
| 604 | S1_89852263 | 1 | 89852263 | 300855 | A | G | G | 0.74194 | A | 0.25806 | 14 | 0.45161 |
| 605 | S1_89852361 | 1 | 89852361 | 98 | C | T | T | 0.74194 | C | 0.25806 | 14 | 0.45161 |
| 606 | S1_90194665 | 1 | 90194665 | 342304 | G | C | G | 0.91935 | C | 0.08065 | 5 | 0.16129 |
| 607 | S1_90194683 | 1 | 90194683 | 18 | A | G | A | 0.82258 | G | 0.17742 | 9 | 0.29032 |
| 608 | S1_91160399 | 1 | 91160399 | 965716 | C | T | C | 0.8871 | T | 0.1129 | 5 | 0.16129 |
| 609 | S1_91160531 | 1 | 91160531 | 132 | G | A | G | 0.95161 | A | 0.04839 | 3 | 0.09677 |
| 610 | S1_91630154 | 1 | 91630154 | 469623 | C | T | T | 0.91935 | C | 0.08065 | 3 | 0.09677 |
| 611 | S1_91782742 | 1 | 91782742 | 152588 | C | A | C | 0.91935 | A | 0.08065 | 5 | 0.16129 |
| 612 | S1_91799474 | 1 | 91799474 | 16732 | T | C | T | 0.56452 | C | 0.43548 | 15 | 0.48387 |
| 613 | S1_92507375 | 1 | 92507375 | 707901 | C | G | C | 0.64516 | G | 0.35484 | 10 | 0.32258 |
| 614 | S1_92635829 | 1 | 92635829 | 128454 | C | A | C | 0.90323 | A | 0.09677 | 6 | 0.19355 |
| 615 | S1_92635895 | 1 | 92635895 | 66 | G | C | G | 0.75806 | C | 0.24194 | 11 | 0.35484 |
| 616 | S1_92635916 | 1 | 92635916 | 21 | G | A | G | 0.75806 | A | 0.24194 | 11 | 0.35484 |
| 617 | S1_92827617 | 1 | 92827617 | 191701 | C | T | C | 0.66129 | T | 0.33871 | 15 | 0.48387 |
| 618 | S1_92827632 | 1 | 92827632 | 15 | C | A | C | 0.90323 | A | 0.09677 | 6 | 0.19355 |
| 619 | S1_92827705 | 1 | 92827705 | 73 | C | T | C | 0.93548 | T | 0.06452 | 4 | 0.12903 |
| 620 | S1_93004944 | 1 | 93004944 | 177239 | T | C | T | 0.82258 | C | 0.17742 | 9 | 0.29032 |
| 621 | S1_93005014 | 1 | 93005014 | 70 | T | G | G | 0.53226 | T | 0.46774 | 13 | 0.41935 |
| 622 | S1_93005097 | 1 | 93005097 | 83 | C | T | C | 0.95161 | T | 0.04839 | 3 | 0.09677 |
| 623 | S1_93738402 | 1 | 93738402 | 733305 | C | T | C | 0.93548 | T | 0.06452 | 4 | 0.12903 |
| 624 | S1_93738519 | 1 | 93738519 | 117 | G | A | G | 0.56452 | A | 0.43548 | 17 | 0.54839 |
| 625 | S1_95126437 | 1 | 95126437 | 1387918 | C | T | C | 0.64516 | T | 0.35484 | 14 | 0.45161 |
| 626 | S1_95228554 | 1 | 95228554 | 102117 | T | C | T | 0.58065 | C | 0.41935 | 16 | 0.51613 |
| 627 | S1_95228715 | 1 | 95228715 | 161 | A | G | A | 0.80645 | G | 0.19355 | 8 | 0.25806 |
| 628 | S1_95228747 | 1 | 95228747 | 32 | G | A | G | 0.77419 | A | 0.22581 | 14 | 0.45161 |
| 629 | S1_95912712 | 1 | 95912712 | 683965 | C | G | C | 0.90323 | G | 0.09677 | 4 | 0.12903 |
| 630 | S1_95912813 | 1 | 95912813 | 101 | C | T | C | 0.87097 | T | 0.12903 | 8 | 0.25806 |
| 631 | S1_95912931 | 1 | 95912931 | 118 | A | T | A | 0.79032 | T | 0.20968 | 13 | 0.41935 |
| 632 | S1_96409464 | 1 | 96409464 | 496533 | C | G | C | 0.87097 | G | 0.12903 | 6 | 0.19355 |
| 633 | S1_96409639 | 1 | 96409639 | 175 | C | T | C | 0.95161 | T | 0.04839 | 3 | 0.09677 |
| 634 | S1_96485116 | 1 | 96485116 | 75477 | G | A | G | 0.74194 | A | 0.25806 | 12 | 0.3871 |
| 635 | S1_96831310 | 1 | 96831310 | 346194 | A | G | A | 0.79032 | G | 0.20968 | 11 | 0.35484 |
| 636 | S1_97030957 | 1 | 97030957 | 199647 | G | C | G | 0.75806 | C | 0.24194 | 13 | 0.41935 |
| 637 | S1_97065859 | 1 | 97065859 | 34902 | C | T | C | 0.83871 | T | 0.16129 | 8 | 0.25806 |
| 638 | S1_97065960 | 1 | 97065960 | 101 | T | C | T | 0.6129 | C | 0.3871 | 16 | 0.51613 |
| 639 | S1_97066086 | 1 | 97066086 | 126 | C | T | C | 0.6129 | T | 0.3871 | 16 | 0.51613 |
| 640 | S1_97161189 | 1 | 97161189 | 95103 | C | T | C | 0.79032 | T | 0.20968 | 11 | 0.35484 |
| 641 | S1_97161190 | 1 | 97161190 | 1 | G | A | G | 0.62903 | A | 0.37097 | 11 | 0.35484 |
| 642 | S1_97161346 | 1 | 97161346 | 156 | G | A | G | 0.90323 | A | 0.09677 | 6 | 0.19355 |
| 643 | S1_97185268 | 1 | 97185268 | 23922 | A | G | G | 0.90323 | A | 0.09677 | 4 | 0.12903 |
| 644 | S1_97185343 | 1 | 97185343 | 75 | C | T | C | 0.93548 | T | 0.06452 | 4 | 0.12903 |
| 645 | S1_97185364 | 1 | 97185364 | 21 | T | C | T | 0.91935 | C | 0.08065 | 5 | 0.16129 |
| 646 | S1_97599645 | 1 | 97599645 | 414281 | C | A | C | 0.83871 | A | 0.16129 | 10 | 0.32258 |

| Site number | SNP name | Chromosome | Physical position | Physical distance from the previous SNP | Reference allele | Alternative allele | Major allele | Major allele frequency | Minor allele | Minor allele frequency | Number heterozygous | Heterozygosity |
|---|---|---|---|---|---|---|---|---|---|---|---|---|
| 647 | S1_97599734 | 1 | 97599734 | 89 | A | G | G | 0.93548 | A | 0.06452 | 4 | 0.12903 |
| 648 | S1_97792783 | 1 | 97792783 | 193049 | G | A | G | 0.93548 | A | 0.06452 | 4 | 0.12903 |
| 649 | S1_97792841 | 1 | 97792841 | 58 | T | C | T | 0.70968 | C | 0.29032 | 14 | 0.45161 |
| 650 | S1_97792871 | 1 | 97792871 | 30 | C | T | C | 0.77419 | T | 0.22581 | 12 | 0.3871 |
| 651 | S1_99160638 | 1 | 99160638 | 1367767 | T | G | G | 0.51613 | T | 0.48387 | 18 | 0.58065 |
| 652 | S1_99160661 | 1 | 99160661 | 23 | T | G | T | 0.79032 | G | 0.20968 | 13 | 0.41935 |
| 653 | S1_99436025 | 1 | 99436025 | 275364 | C | G | C | 0.83871 | G | 0.16129 | 10 | 0.32258 |
| 654 | S1_99724394 | 1 | 99724394 | 288369 | A | T | A | 0.8871 | T | 0.1129 | 7 | 0.22581 |
| 655 | S1_99724496 | 1 | 99724496 | 102 | T | C | C | 0.87097 | T | 0.12903 | 8 | 0.25806 |
| 656 | S1_100083656 | 1 | 100083656 | 359160 | T | C | T | 0.85484 | C | 0.14516 | 9 | 0.29032 |
| 657 | S1_100083668 | 1 | 100083668 | 12 | C | A | C | 0.77419 | A | 0.22581 | 12 | 0.3871 |
| 658 | S1_100084988 | 1 | 100084988 | 1320 | C | T | C | 0.77419 | T | 0.22581 | 12 | 0.3871 |
| 659 | S1_100085115 | 1 | 100085115 | 127 | A | C | A | 0.77419 | C | 0.22581 | 12 | 0.3871 |
| 660 | S1_100085211 | 1 | 100085211 | 96 | C | T | C | 0.95161 | T | 0.04839 | 3 | 0.09677 |
| 661 | S1_100277163 | 1 | 100277163 | 191952 | C | T | C | 0.95161 | T | 0.04839 | 3 | 0.09677 |
| 662 | S1_100277327 | 1 | 100277327 | 164 | C | A | C | 0.91935 | A | 0.08065 | 5 | 0.16129 |
| 663 | S1_100277330 | 1 | 100277330 | 3 | T | C | T | 0.93548 | C | 0.06452 | 4 | 0.12903 |
| 664 | S1_100277331 | 1 | 100277331 | 1 | G | A | G | 0.93548 | A | 0.06452 | 4 | 0.12903 |
| 665 | S1_100277343 | 1 | 100277343 | 12 | C | G | C | 0.53226 | G | 0.46774 | 13 | 0.41935 |
| 666 | S1_100277353 | 1 | 100277353 | 10 | C | T | C | 0.91935 | T | 0.08065 | 5 | 0.16129 |
| 667 | S1_100753426 | 1 | 100753426 | 476073 | C | T | C | 0.83871 | T | 0.16129 | 10 | 0.32258 |
| 668 | S1_101227082 | 1 | 101227082 | 473656 | G | A | A | 0.66129 | G | 0.33871 | 13 | 0.41935 |
| 669 | S1_101227139 | 1 | 101227139 | 57 | C | T | C | 0.79032 | T | 0.20968 | 11 | 0.35484 |
| 670 | S1_101227283 | 1 | 101227283 | 144 | A | G | A | 0.54839 | G | 0.45161 | 16 | 0.51613 |
| 671 | S1_101470086 | 1 | 101470086 | 242803 | A | C | C | 0.8871 | A | 0.1129 | 7 | 0.22581 |
| 672 | S1_101470145 | 1 | 101470145 | 59 | G | A | G | 0.90323 | A | 0.09677 | 6 | 0.19355 |
| 673 | S1_101470261 | 1 | 101470261 | 116 | C | T | T | 0.82258 | C | 0.17742 | 9 | 0.29032 |
| 674 | S1_101470321 | 1 | 101470321 | 60 | G | C | C | 0.82258 | G | 0.17742 | 9 | 0.29032 |
| 675 | S1_101684731 | 1 | 101684731 | 214410 | C | T | C | 0.83871 | T | 0.16129 | 10 | 0.32258 |
| 676 | S1_101684746 | 1 | 101684746 | 15 | T | C | C | 0.6129 | T | 0.3871 | 16 | 0.51613 |
| 677 | S1_103019724 | 1 | 103019724 | 1334978 | A | C | A | 0.95161 | C | 0.04839 | 3 | 0.09677 |
| 678 | S1_103019744 | 1 | 103019744 | 20 | A | T | A | 0.91935 | T | 0.08065 | 5 | 0.16129 |
| 679 | S1_103019784 | 1 | 103019784 | 40 | G | A | G | 0.95161 | A | 0.04839 | 3 | 0.09677 |
| 680 | S1_103019893 | 1 | 103019893 | 109 | C | G | C | 0.95161 | G | 0.04839 | 3 | 0.09677 |
| 681 | S1_103549245 | 1 | 103549245 | 529352 | C | T | C | 0.90323 | T | 0.09677 | 6 | 0.19355 |
| 682 | S1_103549297 | 1 | 103549297 | 52 | T | G | T | 0.69355 | G | 0.30645 | 15 | 0.48387 |
| 683 | S1_103549298 | 1 | 103549298 | 1 | G | T | G | 0.69355 | T | 0.30645 | 15 | 0.48387 |
| 684 | S1_104207475 | 1 | 104207475 | 658177 | G | T | T | 0.69355 | G | 0.30645 | 17 | 0.54839 |
| 685 | S1_104207558 | 1 | 104207558 | 83 | C | T | T | 0.69355 | C | 0.30645 | 17 | 0.54839 |
| 686 | S1_104292832 | 1 | 104292832 | 85274 | G | A | G | 0.70968 | A | 0.29032 | 16 | 0.51613 |
| 687 | S1_105244520 | 1 | 105244520 | 951688 | C | G | C | 0.90323 | G | 0.09677 | 4 | 0.12903 |
| 688 | S1_105244522 | 1 | 105244522 | 2 | G | A | G | 0.90323 | A | 0.09677 | 4 | 0.12903 |
| 689 | S1_105316300 | 1 | 105316300 | 71778 | G | A | G | 0.67742 | A | 0.32258 | 16 | 0.51613 |
| 690 | S1_105316309 | 1 | 105316309 | 9 | T | C | T | 0.67742 | C | 0.32258 | 16 | 0.51613 |
| 691 | S1_105316396 | 1 | 105316396 | 87 | G | C | G | 0.82258 | C | 0.17742 | 9 | 0.29032 |
| 692 | S1_105316399 | 1 | 105316399 | 3 | C | T | T | 0.95161 | C | 0.04839 | 3 | 0.09677 |
| 693 | S1_105316479 | 1 | 105316479 | 80 | A | G | A | 0.70968 | G | 0.29032 | 12 | 0.3871 |
| 694 | S1_105448549 | 1 | 105448549 | 132070 | C | G | C | 0.80645 | G | 0.19355 | 8 | 0.25806 |
| 695 | S1_105448553 | 1 | 105448553 | 4 | G | A | G | 0.77419 | A | 0.22581 | 10 | 0.32258 |
| 696 | S1_105448583 | 1 | 105448583 | 30 | A | G | G | 0.91935 | A | 0.08065 | 5 | 0.16129 |
| 697 | S1_105448621 | 1 | 105448621 | 38 | T | C | C | 0.77419 | T | 0.22581 | 10 | 0.32258 |
| 698 | S1_105448634 | 1 | 105448634 | 13 | T | C | T | 0.93548 | C | 0.06452 | 4 | 0.12903 |
| 699 | S1_105456156 | 1 | 105456156 | 7522 | A | G | G | 0.70968 | A | 0.29032 | 14 | 0.45161 |
| 700 | S1_105456233 | 1 | 105456233 | 77 | G | T | G | 0.5 | T | 0.5 | 15 | 0.48387 |

| Site number | SNP name | Chromosome | Physical position | Physical distance from the previous SNP | Reference allele | Alternative allele | Major allele | Major allele frequency | Minor allele | Minor allele frequency | Number heterozygous | Heterozygosity |
|---|---|---|---|---|---|---|---|---|---|---|---|---|
| 701 | S1_105502684 | 1 | 105502684 | 46451 | C | T | T | 0.62903 | C | 0.37097 | 17 | 0.54839 |
| 702 | S1_106112344 | 1 | 106112344 | 609660 | C | A | C | 0.95161 | A | 0.04839 | 3 | 0.09677 |
| 703 | S1_106171476 | 1 | 106171476 | 59132 | C | T | C | 0.8871 | T | 0.1129 | 7 | 0.22581 |
| 704 | S1_106171478 | 1 | 106171478 | 2 | C | T | C | 0.8871 | T | 0.1129 | 7 | 0.22581 |
| 705 | S1_106171521 | 1 | 106171521 | 43 | T | G | G | 0.64516 | T | 0.35484 | 16 | 0.51613 |
| 706 | S1_106858466 | 1 | 106858466 | 686945 | G | A | A | 0.58065 | G | 0.41935 | 18 | 0.58065 |
| 707 | S1_106858516 | 1 | 106858516 | 50 | C | T | T | 0.54839 | C | 0.45161 | 20 | 0.64516 |
| 708 | S1_106858541 | 1 | 106858541 | 25 | A | G | G | 0.6129 | A | 0.3871 | 18 | 0.58065 |
| 709 | S1_107363166 | 1 | 107363166 | 504625 | C | T | C | 0.95161 | T | 0.04839 | 3 | 0.09677 |
| 710 | S1_107363221 | 1 | 107363221 | 55 | T | C | T | 0.79032 | C | 0.20968 | 11 | 0.35484 |
| 711 | S1_107363225 | 1 | 107363225 | 4 | A | G | A | 0.79032 | G | 0.20968 | 11 | 0.35484 |
| 712 | S1_107363226 | 1 | 107363226 | 1 | A | C | A | 0.74194 | C | 0.25806 | 16 | 0.51613 |
| 713 | S1_107363230 | 1 | 107363230 | 4 | G | T | G | 0.79032 | T | 0.20968 | 11 | 0.35484 |
| 714 | S1_107363274 | 1 | 107363274 | 44 | T | C | T | 0.95161 | C | 0.04839 | 3 | 0.09677 |
| 715 | S1_107363381 | 1 | 107363381 | 107 | T | C | T | 0.82258 | C | 0.17742 | 9 | 0.29032 |
| 716 | S1_107363390 | 1 | 107363390 | 9 | A | G | G | 0.90323 | A | 0.09677 | 4 | 0.12903 |
| 717 | S1_107886148 | 1 | 107886148 | 522758 | A | G | G | 0.51613 | A | 0.48387 | 14 | 0.45161 |
| 718 | S1_107886184 | 1 | 107886184 | 36 | A | G | A | 0.95161 | G | 0.04839 | 3 | 0.09677 |
| 719 | S1_107886207 | 1 | 107886207 | 23 | A | C | A | 0.66129 | C | 0.33871 | 15 | 0.48387 |
| 720 | S1_107886262 | 1 | 107886262 | 55 | G | A | G | 0.83871 | A | 0.16129 | 8 | 0.25806 |
| 721 | S1_108042648 | 1 | 108042648 | 156386 | T | A | A | 0.90323 | T | 0.09677 | 6 | 0.19355 |
| 722 | S1_108252724 | 1 | 108252724 | 210076 | A | G | A | 0.74194 | G | 0.25806 | 12 | 0.3871 |
| 723 | S1_108252728 | 1 | 108252728 | 4 | G | A | G | 0.91935 | A | 0.08065 | 5 | 0.16129 |
| 724 | S1_108252837 | 1 | 108252837 | 109 | G | C | G | 0.91935 | C | 0.08065 | 5 | 0.16129 |
| 725 | S1_108807757 | 1 | 108807757 | 554920 | T | C | T | 0.95161 | C | 0.04839 | 3 | 0.09677 |
| 726 | S1_109101409 | 1 | 109101409 | 293652 | C | T | C | 0.8871 | T | 0.1129 | 7 | 0.22581 |
| 727 | S1_109494348 | 1 | 109494348 | 392939 | T | C | C | 0.8871 | T | 0.1129 | 7 | 0.22581 |
| 728 | S1_109872182 | 1 | 109872182 | 377834 | T | C | T | 0.66129 | C | 0.33871 | 13 | 0.41935 |
| 729 | S1_109872188 | 1 | 109872188 | 6 | C | T | C | 0.82258 | T | 0.17742 | 11 | 0.35484 |
| 730 | S1_109872244 | 1 | 109872244 | 56 | G | A | G | 0.77419 | A | 0.22581 | 12 | 0.3871 |
| 731 | S1_109872308 | 1 | 109872308 | 64 | G | A | G | 0.64516 | A | 0.35484 | 14 | 0.45161 |
| 732 | S1_110007980 | 1 | 110007980 | 135672 | C | T | C | 0.8871 | T | 0.1129 | 7 | 0.22581 |
| 733 | S1_110138223 | 1 | 110138223 | 130243 | A | G | A | 0.80645 | G | 0.19355 | 8 | 0.25806 |
| 734 | S1_111091570 | 1 | 111091570 | 953347 | C | G | G | 0.77419 | C | 0.22581 | 14 | 0.45161 |
| 735 | S1_111091642 | 1 | 111091642 | 72 | C | A | C | 0.93548 | A | 0.06452 | 4 | 0.12903 |
| 736 | S1_111091650 | 1 | 111091650 | 8 | C | A | C | 0.93548 | A | 0.06452 | 4 | 0.12903 |
| 737 | S1_111091679 | 1 | 111091679 | 29 | A | G | A | 0.90323 | G | 0.09677 | 6 | 0.19355 |
| 738 | S1_111091682 | 1 | 111091682 | 3 | C | T | C | 0.95161 | T | 0.04839 | 3 | 0.09677 |
| 739 | S1_111091758 | 1 | 111091758 | 76 | C | T | C | 0.93548 | T | 0.06452 | 4 | 0.12903 |
| 740 | S1_111889938 | 1 | 111889938 | 798180 | C | T | C | 0.93548 | T | 0.06452 | 4 | 0.12903 |
| 741 | S1_112135011 | 1 | 112135011 | 245073 | A | G | A | 0.95161 | G | 0.04839 | 3 | 0.09677 |
| 742 | S1_112135042 | 1 | 112135042 | 31 | C | T | C | 0.77419 | T | 0.22581 | 10 | 0.32258 |
| 743 | S1_112135049 | 1 | 112135049 | 7 | C | T | T | 0.6129 | C | 0.3871 | 12 | 0.3871 |
| 744 | S1_112632301 | 1 | 112632301 | 497252 | G | A | G | 0.72581 | A | 0.27419 | 11 | 0.35484 |
| 745 | S1_112632345 | 1 | 112632345 | 44 | C | T | C | 0.66129 | T | 0.33871 | 13 | 0.41935 |
| 746 | S1_112632433 | 1 | 112632433 | 88 | T | A | T | 0.72581 | A | 0.27419 | 11 | 0.35484 |
| 747 | S1_112632462 | 1 | 112632462 | 29 | G | A | G | 0.91935 | A | 0.08065 | 5 | 0.16129 |
| 748 | S1_112708821 | 1 | 112708821 | 76359 | G | T | G | 0.80645 | T | 0.19355 | 10 | 0.32258 |
| 749 | S1_112708840 | 1 | 112708840 | 19 | A | C | A | 0.85484 | C | 0.14516 | 7 | 0.22581 |
| 750 | S1_112708888 | 1 | 112708888 | 48 | C | T | C | 0.80645 | T | 0.19355 | 10 | 0.32258 |
| 751 | S1_112708903 | 1 | 112708903 | 15 | C | T | C | 0.80645 | T | 0.19355 | 10 | 0.32258 |
| 752 | S1_112708913 | 1 | 112708913 | 10 | C | T | C | 0.80645 | T | 0.19355 | 10 | 0.32258 |
| 753 | S1_112708999 | 1 | 112708999 | 86 | G | C | G | 0.80645 | C | 0.19355 | 10 | 0.32258 |
| 754 | S1_112709026 | 1 | 112709026 | 27 | G | A | G | 0.90323 | A | 0.09677 | 6 | 0.19355 |

| Site number | SNP name | Chromosome | Physical position | Physical distance from the previous SNP | Reference allele | Alternative allele | Major allele | Major allele frequency | Minor allele | Minor allele frequency | Number heterozygous | Heterozygosity |
|---|---|---|---|---|---|---|---|---|---|---|---|---|
| 755 | S1_113042801 | 1 | 113042801 | 333775 | C | T | C | 0.90323 | T | 0.09677 | 4 | 0.12903 |
| 756 | S1_113042811 | 1 | 113042811 | 10 | G | A | G | 0.91935 | A | 0.08065 | 5 | 0.16129 |
| 757 | S1_113042840 | 1 | 113042840 | 29 | C | T | C | 0.95161 | T | 0.04839 | 3 | 0.09677 |
| 758 | S1_113042906 | 1 | 113042906 | 66 | C | G | C | 0.90323 | G | 0.09677 | 6 | 0.19355 |
| 759 | S1_113082659 | 1 | 113082659 | 39753 | A | G | G | 0.53226 | A | 0.46774 | 15 | 0.48387 |
| 760 | S1_113169294 | 1 | 113169294 | 86635 | C | T | C | 0.8871 | T | 0.1129 | 7 | 0.22581 |
| 761 | S1_113169312 | 1 | 113169312 | 18 | C | G | C | 0.80645 | G | 0.19355 | 12 | 0.3871 |
| 762 | S1_113169357 | 1 | 113169357 | 45 | C | T | C | 0.80645 | T | 0.19355 | 12 | 0.3871 |
| 763 | S1_113169374 | 1 | 113169374 | 17 | C | T | C | 0.80645 | T | 0.19355 | 12 | 0.3871 |
| 764 | S1_113169421 | 1 | 113169421 | 47 | C | T | C | 0.95161 | T | 0.04839 | 3 | 0.09677 |
| 765 | S1_113169445 | 1 | 113169445 | 24 | C | T | T | 0.64516 | C | 0.35484 | 18 | 0.58065 |
| 766 | S1_113169481 | 1 | 113169481 | 36 | G | A | G | 0.80645 | A | 0.19355 | 12 | 0.3871 |
| 767 | S1_113169537 | 1 | 113169537 | 56 | C | T | C | 0.80645 | T | 0.19355 | 12 | 0.3871 |
| 768 | S1_114506088 | 1 | 114506088 | 1336551 | C | G | C | 0.90323 | G | 0.09677 | 4 | 0.12903 |
| 769 | S1_114506146 | 1 | 114506146 | 58 | T | C | T | 0.70968 | C | 0.29032 | 16 | 0.51613 |
| 770 | S1_114506184 | 1 | 114506184 | 38 | A | G | A | 0.90323 | G | 0.09677 | 4 | 0.12903 |
| 771 | S1_114704723 | 1 | 114704723 | 198539 | G | A | G | 0.79032 | A | 0.20968 | 13 | 0.41935 |
| 772 | S1_114704818 | 1 | 114704818 | 95 | G | A | G | 0.79032 | A | 0.20968 | 13 | 0.41935 |
| 773 | S1_114704846 | 1 | 114704846 | 28 | T | C | C | 0.51613 | T | 0.48387 | 20 | 0.64516 |
| 774 | S1_114704870 | 1 | 114704870 | 24 | T | C | T | 0.79032 | C | 0.20968 | 13 | 0.41935 |
| 775 | S1_114704952 | 1 | 114704952 | 82 | A | G | A | 0.79032 | G | 0.20968 | 13 | 0.41935 |
| 776 | S1_115350728 | 1 | 115350728 | 645776 | T | C | T | 0.93548 | C | 0.06452 | 2 | 0.06452 |
| 777 | S1_115350815 | 1 | 115350815 | 87 | G | A | G | 0.93548 | A | 0.06452 | 2 | 0.06452 |
| 778 | S1_115997259 | 1 | 115997259 | 646444 | C | T | C | 0.90323 | T | 0.09677 | 6 | 0.19355 |
| 779 | S1_115997409 | 1 | 115997409 | 150 | G | A | A | 0.56452 | G | 0.43548 | 13 | 0.41935 |
| 780 | S1_115997415 | 1 | 115997415 | 6 | T | G | G | 0.91935 | T | 0.08065 | 5 | 0.16129 |
| 781 | S1_116124108 | 1 | 116124108 | 126693 | C | T | C | 0.80645 | T | 0.19355 | 10 | 0.32258 |
| 782 | S1_116124166 | 1 | 116124166 | 58 | G | A | G | 0.95161 | A | 0.04839 | 3 | 0.09677 |
| 783 | S1_116157198 | 1 | 116157198 | 33032 | C | T | T | 0.90323 | C | 0.09677 | 6 | 0.19355 |
| 784 | S1_116157204 | 1 | 116157204 | 6 | T | C | T | 0.80645 | C | 0.19355 | 12 | 0.3871 |
| 785 | S1_116157323 | 1 | 116157323 | 119 | A | G | A | 0.95161 | G | 0.04839 | 3 | 0.09677 |
| 786 | S1_116606814 | 1 | 116606814 | 449491 | C | T | C | 0.93548 | T | 0.06452 | 4 | 0.12903 |
| 787 | S1_116606904 | 1 | 116606904 | 90 | A | G | A | 0.91935 | G | 0.08065 | 5 | 0.16129 |
| 788 | S1_116622788 | 1 | 116622788 | 15884 | G | A | G | 0.93548 | A | 0.06452 | 4 | 0.12903 |
| 789 | S1_116791076 | 1 | 116791076 | 168288 | C | T | C | 0.90323 | T | 0.09677 | 4 | 0.12903 |
| 790 | S1_116791103 | 1 | 116791103 | 27 | C | T | C | 0.6129 | T | 0.3871 | 16 | 0.51613 |
| 791 | S1_116791118 | 1 | 116791118 | 15 | C | T | T | 0.6129 | C | 0.3871 | 16 | 0.51613 |
| 792 | S1_116962623 | 1 | 116962623 | 171505 | G | A | G | 0.56452 | A | 0.43548 | 21 | 0.67742 |
| 793 | S1_117211036 | 1 | 117211036 | 248413 | G | T | T | 0.90323 | G | 0.09677 | 6 | 0.19355 |
| 794 | S1_117303865 | 1 | 117303865 | 92829 | G | A | G | 0.90323 | A | 0.09677 | 6 | 0.19355 |
| 795 | S1_117869595 | 1 | 117869595 | 565730 | T | C | T | 0.58065 | C | 0.41935 | 18 | 0.58065 |
| 796 | S1_117869613 | 1 | 117869613 | 18 | C | T | C | 0.95161 | T | 0.04839 | 1 | 0.03226 |
| 797 | S1_117869624 | 1 | 117869624 | 11 | G | A | G | 0.95161 | A | 0.04839 | 3 | 0.09677 |
| 798 | S1_117869731 | 1 | 117869731 | 107 | C | T | C | 0.8871 | T | 0.1129 | 5 | 0.16129 |
| 799 | S1_117869732 | 1 | 117869732 | 1 | A | G | A | 0.87097 | G | 0.12903 | 6 | 0.19355 |
| 800 | S1_117958309 | 1 | 117958309 | 88577 | A | G | A | 0.82258 | G | 0.17742 | 9 | 0.29032 |
| 801 | S1_118058302 | 1 | 118058302 | 99993 | G | A | G | 0.90323 | A | 0.09677 | 4 | 0.12903 |
| 802 | S1_118209563 | 1 | 118209563 | 151261 | C | T | C | 0.91935 | T | 0.08065 | 3 | 0.09677 |
| 803 | S1_118246195 | 1 | 118246195 | 36632 | T | C | T | 0.67742 | C | 0.32258 | 14 | 0.45161 |
| 804 | S1_118246209 | 1 | 118246209 | 14 | G | A | G | 0.67742 | A | 0.32258 | 14 | 0.45161 |
| 805 | S1_118246285 | 1 | 118246285 | 76 | G | C | G | 0.66129 | C | 0.33871 | 15 | 0.48387 |
| 806 | S1_118246312 | 1 | 118246312 | 27 | G | A | G | 0.67742 | A | 0.32258 | 14 | 0.45161 |
| 807 | S1_118246322 | 1 | 118246322 | 10 | C | G | C | 0.66129 | G | 0.33871 | 15 | 0.48387 |
| 808 | S1_118351697 | 1 | 118351697 | 105375 | A | G | A | 0.93548 | G | 0.06452 | 4 | 0.12903 |

| Site number | SNP name | Chromosome | Physical position | Physical distance from the previous SNP | Reference allele | Alternative allele | Major allele | Major allele frequency | Minor allele | Minor allele frequency | Number heterozygous | Heterozygosity |
|---|---|---|---|---|---|---|---|---|---|---|---|---|
| 809 | S1_118351716 | 1 | 118351716 | 19 | C | T | C | 0.93548 | T | 0.06452 | 4 | 0.12903 |
| 810 | S1_118351763 | 1 | 118351763 | 47 | C | T | C | 0.91935 | T | 0.08065 | 3 | 0.09677 |
| 811 | S1_118351778 | 1 | 118351778 | 15 | A | G | A | 0.93548 | G | 0.06452 | 4 | 0.12903 |
| 812 | S1_118351782 | 1 | 118351782 | 4 | G | A | G | 0.93548 | A | 0.06452 | 4 | 0.12903 |
| 813 | S1_118351785 | 1 | 118351785 | 3 | A | G | A | 0.95161 | G | 0.04839 | 3 | 0.09677 |
| 814 | S1_118351787 | 1 | 118351787 | 2 | A | G | A | 0.93548 | G | 0.06452 | 4 | 0.12903 |
| 815 | S1_118351875 | 1 | 118351875 | 88 | T | C | T | 0.93548 | C | 0.06452 | 4 | 0.12903 |
| 816 | S1_118351881 | 1 | 118351881 | 6 | C | T | C | 0.93548 | T | 0.06452 | 4 | 0.12903 |
| 817 | S1_118452983 | 1 | 118452983 | 101102 | C | T | C | 0.93548 | T | 0.06452 | 4 | 0.12903 |
| 818 | S1_118452989 | 1 | 118452989 | 6 | T | C | C | 0.58065 | T | 0.41935 | 14 | 0.45161 |
| 819 | S1_118453121 | 1 | 118453121 | 132 | A | G | A | 0.59677 | G | 0.40323 | 15 | 0.48387 |
| 820 | S1_118453137 | 1 | 118453137 | 16 | A | G | A | 0.59677 | G | 0.40323 | 15 | 0.48387 |
| 821 | S1_118453153 | 1 | 118453153 | 16 | A | G | A | 0.59677 | G | 0.40323 | 15 | 0.48387 |
| 822 | S1_118453182 | 1 | 118453182 | 29 | A | C | A | 0.59677 | C | 0.40323 | 15 | 0.48387 |
| 823 | S1_118503828 | 1 | 118503828 | 50646 | A | G | A | 0.62903 | G | 0.37097 | 13 | 0.41935 |
| 824 | S1_118503844 | 1 | 118503844 | 16 | C | T | C | 0.59677 | T | 0.40323 | 13 | 0.41935 |
| 825 | S1_118856971 | 1 | 118856971 | 353127 | G | A | G | 0.93548 | A | 0.06452 | 4 | 0.12903 |
| 826 | S1_118962783 | 1 | 118962783 | 105812 | T | C | T | 0.8871 | C | 0.1129 | 7 | 0.22581 |
| 827 | S1_119594655 | 1 | 119594655 | 631872 | C | T | C | 0.83871 | T | 0.16129 | 10 | 0.32258 |
| 828 | S1_119594756 | 1 | 119594756 | 101 | C | A | C | 0.90323 | A | 0.09677 | 4 | 0.12903 |
| 829 | S1_119594781 | 1 | 119594781 | 25 | T | C | T | 0.66129 | C | 0.33871 | 17 | 0.54839 |
| 830 | S1_120555746 | 1 | 120555746 | 960965 | C | T | C | 0.69355 | T | 0.30645 | 15 | 0.48387 |
| 831 | S1_120555852 | 1 | 120555852 | 106 | G | T | G | 0.95161 | T | 0.04839 | 3 | 0.09677 |
| 832 | S1_120555854 | 1 | 120555854 | 2 | G | T | G | 0.8871 | T | 0.1129 | 5 | 0.16129 |
| 833 | S1_120787509 | 1 | 120787509 | 231655 | A | T | A | 0.79032 | T | 0.20968 | 9 | 0.29032 |
| 834 | S1_121958357 | 1 | 121958357 | 1170848 | T | G | G | 0.69355 | T | 0.30645 | 15 | 0.48387 |
| 835 | S1_121958374 | 1 | 121958374 | 17 | A | G | A | 0.95161 | G | 0.04839 | 3 | 0.09677 |
| 836 | S1_121958486 | 1 | 121958486 | 112 | G | A | G | 0.93548 | A | 0.06452 | 4 | 0.12903 |
| 837 | S1_121958561 | 1 | 121958561 | 75 | G | A | G | 0.8871 | A | 0.1129 | 5 | 0.16129 |
| 838 | S1_121958582 | 1 | 121958582 | 21 | C | T | C | 0.90323 | T | 0.09677 | 4 | 0.12903 |
| 839 | S1_121958583 | 1 | 121958583 | 1 | G | A | G | 0.8871 | A | 0.1129 | 5 | 0.16129 |
| 840 | S1_122219412 | 1 | 122219412 | 260829 | C | A | C | 0.77419 | A | 0.22581 | 14 | 0.45161 |
| 841 | S1_122219445 | 1 | 122219445 | 33 | T | G | G | 0.90323 | T | 0.09677 | 6 | 0.19355 |
| 842 | S1_122219504 | 1 | 122219504 | 59 | G | A | G | 0.90323 | A | 0.09677 | 6 | 0.19355 |
| 843 | S1_122219626 | 1 | 122219626 | 122 | A | G | A | 0.6129 | G | 0.3871 | 20 | 0.64516 |
| 844 | S1_122904046 | 1 | 122904046 | 684420 | G | A | G | 0.91935 | A | 0.08065 | 3 | 0.09677 |
| 845 | S1_122904090 | 1 | 122904090 | 44 | G | A | G | 0.91935 | A | 0.08065 | 3 | 0.09677 |
| 846 | S1_122904094 | 1 | 122904094 | 4 | A | C | A | 0.91935 | C | 0.08065 | 3 | 0.09677 |
| 847 | S1_123269539 | 1 | 123269539 | 365445 | T | C | T | 0.95161 | C | 0.04839 | 3 | 0.09677 |
| 848 | S1_123269670 | 1 | 123269670 | 131 | C | T | T | 0.66129 | C | 0.33871 | 13 | 0.41935 |
| 849 | S1_123269743 | 1 | 123269743 | 73 | C | T | C | 0.91935 | T | 0.08065 | 3 | 0.09677 |
| 850 | S1_123269760 | 1 | 123269760 | 17 | A | G | G | 0.83871 | A | 0.16129 | 10 | 0.32258 |
| 851 | S1_123376486 | 1 | 123376486 | 106726 | T | C | T | 0.91935 | C | 0.08065 | 3 | 0.09677 |
| 852 | S1_123376493 | 1 | 123376493 | 7 | C | T | C | 0.91935 | T | 0.08065 | 3 | 0.09677 |
| 853 | S1_123376535 | 1 | 123376535 | 42 | G | A | G | 0.80645 | A | 0.19355 | 6 | 0.19355 |
| 854 | S1_124562799 | 1 | 124562799 | 1186264 | A | G | G | 0.67742 | A | 0.32258 | 12 | 0.3871 |
| 855 | S1_124562806 | 1 | 124562806 | 7 | T | C | C | 0.79032 | T | 0.20968 | 11 | 0.35484 |
| 856 | S1_124562813 | 1 | 124562813 | 7 | T | C | C | 0.79032 | T | 0.20968 | 11 | 0.35484 |
| 857 | S1_125287092 | 1 | 125287092 | 724279 | A | T | A | 0.93548 | T | 0.06452 | 4 | 0.12903 |
| 858 | S1_125287103 | 1 | 125287103 | 11 | G | C | G | 0.87097 | C | 0.12903 | 8 | 0.25806 |
| 859 | S1_125582207 | 1 | 125582207 | 295104 | G | A | G | 0.93548 | A | 0.06452 | 4 | 0.12903 |
| 860 | S1_125582299 | 1 | 125582299 | 92 | T | A | T | 0.93548 | A | 0.06452 | 4 | 0.12903 |
| 861 | S1_125582303 | 1 | 125582303 | 4 | G | A | G | 0.91935 | A | 0.08065 | 5 | 0.16129 |
| 862 | S1_125582324 | 1 | 125582324 | 21 | G | A | G | 0.91935 | A | 0.08065 | 5 | 0.16129 |

| Site number | SNP name | Chromosome | Physical position | Physical distance from the previous SNP | Reference allele | Alternative allele | Major allele | Major allele frequency | Minor allele | Minor allele frequency | Number heterozygous | Heterozygosity |
|---|---|---|---|---|---|---|---|---|---|---|---|---|
| 863 | S1_125774183 | 1 | 125774183 | 191859 | A | G | A | 0.83871 | G | 0.16129 | 10 | 0.32258 |
| 864 | S1_125774196 | 1 | 125774196 | 13 | C | T | C | 0.8871 | T | 0.1129 | 7 | 0.22581 |
| 865 | S1_125774236 | 1 | 125774236 | 40 | G | A | G | 0.80645 | A | 0.19355 | 10 | 0.32258 |
| 866 | S1_125774286 | 1 | 125774286 | 50 | G | A | G | 0.83871 | A | 0.16129 | 10 | 0.32258 |
| 867 | S1_125774307 | 1 | 125774307 | 21 | C | T | C | 0.56452 | T | 0.43548 | 17 | 0.54839 |
| 868 | S1_125774309 | 1 | 125774309 | 2 | T | C | T | 0.75806 | C | 0.24194 | 13 | 0.41935 |
| 869 | S1_126339743 | 1 | 126339743 | 565434 | G | T | G | 0.70968 | T | 0.29032 | 16 | 0.51613 |
| 870 | S1_126559289 | 1 | 126559289 | 219546 | T | G | G | 0.59677 | T | 0.40323 | 15 | 0.48387 |
| 871 | S1_126559461 | 1 | 126559461 | 172 | G | A | G | 0.91935 | A | 0.08065 | 5 | 0.16129 |
| 872 | S1_126559503 | 1 | 126559503 | 42 | A | C | A | 0.91935 | C | 0.08065 | 5 | 0.16129 |
| 873 | S1_126567115 | 1 | 126567115 | 7612 | C | T | T | 0.54839 | C | 0.45161 | 18 | 0.58065 |
| 874 | S1_126970852 | 1 | 126970852 | 403737 | T | A | A | 0.83871 | T | 0.16129 | 10 | 0.32258 |
| 875 | S1_127037117 | 1 | 127037117 | 66265 | G | C | G | 0.83871 | C | 0.16129 | 8 | 0.25806 |
| 876 | S1_127037132 | 1 | 127037132 | 15 | G | A | G | 0.93548 | A | 0.06452 | 4 | 0.12903 |
| 877 | S1_127037287 | 1 | 127037287 | 155 | A | G | G | 0.53226 | A | 0.46774 | 13 | 0.41935 |
| 878 | S1_127037301 | 1 | 127037301 | 14 | T | C | T | 0.59677 | C | 0.40323 | 17 | 0.54839 |
| 879 | S1_127079216 | 1 | 127079216 | 41915 | G | T | G | 0.90323 | T | 0.09677 | 6 | 0.19355 |
| 880 | S1_127079350 | 1 | 127079350 | 134 | T | C | T | 0.90323 | C | 0.09677 | 6 | 0.19355 |
| 881 | S1_127583240 | 1 | 127583240 | 503890 | C | A | C | 0.95161 | A | 0.04839 | 3 | 0.09677 |
| 882 | S1_127586071 | 1 | 127586071 | 2831 | A | G | A | 0.83871 | G | 0.16129 | 10 | 0.32258 |
| 883 | S1_127591937 | 1 | 127591937 | 5866 | G | T | G | 0.91935 | T | 0.08065 | 5 | 0.16129 |
| 884 | S1_127591995 | 1 | 127591995 | 58 | G | A | G | 0.91935 | A | 0.08065 | 5 | 0.16129 |
| 885 | S1_127643388 | 1 | 127643388 | 51393 | C | T | T | 0.87097 | C | 0.12903 | 8 | 0.25806 |
| 886 | S1_127736586 | 1 | 127736586 | 93198 | C | T | C | 0.91935 | T | 0.08065 | 5 | 0.16129 |
| 887 | S1_127736657 | 1 | 127736657 | 71 | C | T | C | 0.66129 | T | 0.33871 | 13 | 0.41935 |
| 888 | S1_127973276 | 1 | 127973276 | 236619 | G | A | G | 0.8871 | A | 0.1129 | 7 | 0.22581 |
| 889 | S1_127973338 | 1 | 127973338 | 62 | T | C | T | 0.54839 | C | 0.45161 | 16 | 0.51613 |
| 890 | S1_128018720 | 1 | 128018720 | 45382 | T | C | T | 0.69355 | C | 0.30645 | 11 | 0.35484 |
| 891 | S1_128018722 | 1 | 128018722 | 2 | C | T | C | 0.93548 | T | 0.06452 | 4 | 0.12903 |
| 892 | S1_128018788 | 1 | 128018788 | 66 | G | A | G | 0.90323 | A | 0.09677 | 6 | 0.19355 |
| 893 | S1_128018844 | 1 | 128018844 | 56 | G | A | G | 0.83871 | A | 0.16129 | 8 | 0.25806 |
| 894 | S1_128018870 | 1 | 128018870 | 26 | G | A | G | 0.93548 | A | 0.06452 | 4 | 0.12903 |
| 895 | S1_128018878 | 1 | 128018878 | 8 | C | A | C | 0.93548 | A | 0.06452 | 4 | 0.12903 |
| 896 | S1_128064159 | 1 | 128064159 | 45281 | T | C | T | 0.72581 | C | 0.27419 | 15 | 0.48387 |
| 897 | S1_128064166 | 1 | 128064166 | 7 | C | T | T | 0.72581 | C | 0.27419 | 15 | 0.48387 |
| 898 | S1_128064167 | 1 | 128064167 | 1 | A | G | G | 0.72581 | A | 0.27419 | 15 | 0.48387 |
| 899 | S1_128064250 | 1 | 128064250 | 83 | G | A | G | 0.91935 | A | 0.08065 | 5 | 0.16129 |
| 900 | S1_128395581 | 1 | 128395581 | 331331 | A | T | A | 0.56452 | T | 0.43548 | 15 | 0.48387 |
| 901 | S1_128395612 | 1 | 128395612 | 31 | C | A | A | 0.82258 | C | 0.17742 | 9 | 0.29032 |
| 902 | S1_128395630 | 1 | 128395630 | 18 | C | T | C | 0.87097 | T | 0.12903 | 6 | 0.19355 |
| 903 | S1_128708702 | 1 | 128708702 | 313072 | A | G | A | 0.93548 | G | 0.06452 | 4 | 0.12903 |
| 904 | S1_129159070 | 1 | 129159070 | 450368 | G | A | A | 0.53226 | G | 0.46774 | 17 | 0.54839 |
| 905 | S1_129580450 | 1 | 129580450 | 421380 | T | C | T | 0.85484 | C | 0.14516 | 7 | 0.22581 |
| 906 | S1_129580476 | 1 | 129580476 | 26 | A | T | A | 0.85484 | T | 0.14516 | 7 | 0.22581 |
| 907 | S1_129580501 | 1 | 129580501 | 25 | A | G | G | 0.91935 | A | 0.08065 | 5 | 0.16129 |
| 908 | S1_129616715 | 1 | 129616715 | 36214 | T | C | T | 0.95161 | C | 0.04839 | 3 | 0.09677 |
| 909 | S1_129814160 | 1 | 129814160 | 197445 | A | G | G | 0.85484 | A | 0.14516 | 9 | 0.29032 |
| 910 | S1_129814204 | 1 | 129814204 | 44 | A | C | A | 0.91935 | C | 0.08065 | 3 | 0.09677 |
| 911 | S1_129814359 | 1 | 129814359 | 155 | A | G | G | 0.85484 | A | 0.14516 | 9 | 0.29032 |
| 912 | S1_129814373 | 1 | 129814373 | 14 | C | G | G | 0.85484 | C | 0.14516 | 9 | 0.29032 |
| 913 | S1_130261386 | 1 | 130261386 | 447013 | G | A | G | 0.8871 | A | 0.1129 | 5 | 0.16129 |
| 914 | S1_130278671 | 1 | 130278671 | 17285 | G | A | G | 0.82258 | A | 0.17742 | 9 | 0.29032 |
| 915 | S1_130780620 | 1 | 130780620 | 501949 | C | G | C | 0.77419 | G | 0.22581 | 12 | 0.3871 |
| 916 | S1_130780652 | 1 | 130780652 | 32 | T | C | T | 0.77419 | C | 0.22581 | 12 | 0.3871 |

| Site number | SNP name | Chromosome | Physical position | Physical distance from the previous SNP | Reference allele | Alternative allele | Major allele | Major allele frequency | Minor allele | Minor allele frequency | Number heterozygous | Heterozygosity |
|---|---|---|---|---|---|---|---|---|---|---|---|---|
| 917 | S1_130780753 | 1 | 130780753 | 101 | G | T | G | 0.82258 | T | 0.17742 | 9 | 0.29032 |
| 918 | S1_131056909 | 1 | 131056909 | 276156 | C | T | C | 0.93548 | T | 0.06452 | 4 | 0.12903 |
| 919 | S1_131056924 | 1 | 131056924 | 15 | C | T | C | 0.79032 | T | 0.20968 | 11 | 0.35484 |
| 920 | S1_131056925 | 1 | 131056925 | 1 | A | G | G | 0.83871 | A | 0.16129 | 10 | 0.32258 |
| 921 | S1_131056962 | 1 | 131056962 | 37 | C | T | C | 0.93548 | T | 0.06452 | 4 | 0.12903 |
| 922 | S1_131057048 | 1 | 131057048 | 86 | G | A | G | 0.54839 | A | 0.45161 | 20 | 0.64516 |
| 923 | S1_131223953 | 1 | 131223953 | 166905 | T | C | T | 0.90323 | C | 0.09677 | 6 | 0.19355 |
| 924 | S1_131223974 | 1 | 131223974 | 21 | C | T | C | 0.90323 | T | 0.09677 | 6 | 0.19355 |
| 925 | S1_131224020 | 1 | 131224020 | 46 | T | C | T | 0.6129 | C | 0.3871 | 20 | 0.64516 |
| 926 | S1_131327405 | 1 | 131327405 | 103385 | A | T | A | 0.80645 | T | 0.19355 | 10 | 0.32258 |
| 927 | S1_131327434 | 1 | 131327434 | 29 | G | T | G | 0.51613 | T | 0.48387 | 18 | 0.58065 |
| 928 | S1_132494832 | 1 | 132494832 | 1167398 | G | A | G | 0.80645 | A | 0.19355 | 10 | 0.32258 |
| 929 | S1_133663903 | 1 | 133663903 | 1169071 | T | G | G | 0.8871 | T | 0.1129 | 5 | 0.16129 |
| 930 | S1_133663931 | 1 | 133663931 | 28 | C | A | C | 0.90323 | A | 0.09677 | 6 | 0.19355 |
| 931 | S1_133664022 | 1 | 133664022 | 91 | G | C | G | 0.95161 | C | 0.04839 | 3 | 0.09677 |
| 932 | S1_133936275 | 1 | 133936275 | 272253 | G | C | G | 0.72581 | C | 0.27419 | 11 | 0.35484 |
| 933 | S1_133977213 | 1 | 133977213 | 40938 | G | C | G | 0.90323 | C | 0.09677 | 6 | 0.19355 |
| 934 | S1_133977255 | 1 | 133977255 | 42 | C | G | C | 0.74194 | G | 0.25806 | 14 | 0.45161 |
| 935 | S1_134070728 | 1 | 134070728 | 93473 | T | C | T | 0.93548 | C | 0.06452 | 4 | 0.12903 |
| 936 | S1_134070760 | 1 | 134070760 | 32 | G | A | G | 0.93548 | A | 0.06452 | 4 | 0.12903 |
| 937 | S1_134070767 | 1 | 134070767 | 7 | C | T | C | 0.80645 | T | 0.19355 | 12 | 0.3871 |
| 938 | S1_134070915 | 1 | 134070915 | 148 | C | T | C | 0.80645 | T | 0.19355 | 12 | 0.3871 |
| 939 | S1_134248967 | 1 | 134248967 | 178052 | G | A | G | 0.80645 | A | 0.19355 | 10 | 0.32258 |
| 940 | S1_134362601 | 1 | 134362601 | 113634 | A | G | A | 0.93548 | G | 0.06452 | 4 | 0.12903 |
| 941 | S1_134362631 | 1 | 134362631 | 30 | C | T | C | 0.93548 | T | 0.06452 | 4 | 0.12903 |
| 942 | S1_134362693 | 1 | 134362693 | 62 | T | A | T | 0.93548 | A | 0.06452 | 4 | 0.12903 |
| 943 | S1_134362715 | 1 | 134362715 | 22 | C | T | C | 0.93548 | T | 0.06452 | 4 | 0.12903 |
| 944 | S1_134362720 | 1 | 134362720 | 5 | C | T | C | 0.87097 | T | 0.12903 | 6 | 0.19355 |
| 945 | S1_134362721 | 1 | 134362721 | 1 | C | T | C | 0.95161 | T | 0.04839 | 3 | 0.09677 |
| 946 | S1_134991391 | 1 | 134991391 | 628670 | C | T | T | 0.75806 | C | 0.24194 | 13 | 0.41935 |
| 947 | S1_135062778 | 1 | 135062778 | 71387 | A | G | A | 0.93548 | G | 0.06452 | 4 | 0.12903 |
| 948 | S1_135062818 | 1 | 135062818 | 40 | T | A | T | 0.93548 | A | 0.06452 | 4 | 0.12903 |
| 949 | S1_135062993 | 1 | 135062993 | 175 | C | T | T | 0.74194 | C | 0.25806 | 16 | 0.51613 |
| 950 | S1_135277236 | 1 | 135277236 | 214243 | C | T | C | 0.93548 | T | 0.06452 | 4 | 0.12903 |
| 951 | S1_135538328 | 1 | 135538328 | 261092 | C | A | C | 0.95161 | A | 0.04839 | 3 | 0.09677 |
| 952 | S1_135538348 | 1 | 135538348 | 20 | C | T | C | 0.95161 | T | 0.04839 | 3 | 0.09677 |
| 953 | S1_136525448 | 1 | 136525448 | 987100 | C | T | C | 0.80645 | T | 0.19355 | 10 | 0.32258 |
| 954 | S1_136525510 | 1 | 136525510 | 62 | G | A | G | 0.80645 | A | 0.19355 | 10 | 0.32258 |
| 955 | S1_136553313 | 1 | 136553313 | 27803 | A | G | A | 0.75806 | G | 0.24194 | 13 | 0.41935 |
| 956 | S1_136553319 | 1 | 136553319 | 6 | C | T | C | 0.80645 | T | 0.19355 | 10 | 0.32258 |
| 957 | S1_136553334 | 1 | 136553334 | 15 | T | C | T | 0.75806 | C | 0.24194 | 13 | 0.41935 |
| 958 | S1_136553342 | 1 | 136553342 | 8 | G | A | G | 0.75806 | A | 0.24194 | 13 | 0.41935 |
| 959 | S1_136553348 | 1 | 136553348 | 6 | G | A | G | 0.75806 | A | 0.24194 | 13 | 0.41935 |
| 960 | S1_137137326 | 1 | 137137326 | 583978 | G | A | G | 0.8871 | A | 0.1129 | 7 | 0.22581 |
| 961 | S1_137794947 | 1 | 137794947 | 657621 | A | G | G | 0.51613 | A | 0.48387 | 14 | 0.45161 |
| 962 | S1_137795000 | 1 | 137795000 | 53 | G | T | T | 0.54839 | G | 0.45161 | 12 | 0.3871 |
| 963 | S1_137795071 | 1 | 137795071 | 71 | T | C | C | 0.54839 | T | 0.45161 | 12 | 0.3871 |
| 964 | S1_137795129 | 1 | 137795129 | 58 | T | C | C | 0.54839 | T | 0.45161 | 12 | 0.3871 |
| 965 | S1_137795177 | 1 | 137795177 | 48 | C | T | C | 0.64516 | T | 0.35484 | 16 | 0.51613 |
| 966 | S1_138052088 | 1 | 138052088 | 256911 | A | T | A | 0.70968 | T | 0.29032 | 14 | 0.45161 |
| 967 | S1_138793868 | 1 | 138793868 | 741780 | A | G | A | 0.83871 | G | 0.16129 | 8 | 0.25806 |
| 968 | S1_138794004 | 1 | 138794004 | 136 | G | A | G | 0.83871 | A | 0.16129 | 8 | 0.25806 |
| 969 | S1_139413857 | 1 | 139413857 | 619853 | T | C | T | 0.95161 | C | 0.04839 | 3 | 0.09677 |
| 970 | S1_139413936 | 1 | 139413936 | 79 | A | G | G | 0.75806 | A | 0.24194 | 15 | 0.48387 |

| Site number | SNP name | Chromosome | Physical position | Physical distance from the previous SNP | Reference allele | Alternative allele | Major allele | Major allele frequency | Minor allele | Minor allele frequency | Number heterozygous | Heterozygosity |
|---|---|---|---|---|---|---|---|---|---|---|---|---|
| 971 | S1_139441887 | 1 | 139441887 | 27951 | A | G | A | 0.8871 | G | 0.1129 | 5 | 0.16129 |
| 972 | S1_139775643 | 1 | 139775643 | 333756 | G | C | G | 0.91935 | C | 0.08065 | 5 | 0.16129 |
| 973 | S1_139775700 | 1 | 139775700 | 57 | G | A | G | 0.62903 | A | 0.37097 | 17 | 0.54839 |
| 974 | S1_139882535 | 1 | 139882535 | 106835 | G | A | G | 0.93548 | A | 0.06452 | 4 | 0.12903 |
| 975 | S1_140471214 | 1 | 140471214 | 588679 | C | T | C | 0.80645 | T | 0.19355 | 10 | 0.32258 |
| 976 | S1_140471349 | 1 | 140471349 | 135 | G | C | G | 0.80645 | C | 0.19355 | 10 | 0.32258 |
| 977 | S1_140496554 | 1 | 140496554 | 25205 | C | T | C | 0.87097 | T | 0.12903 | 8 | 0.25806 |
| 978 | S1_140496612 | 1 | 140496612 | 58 | T | C | C | 0.70968 | T | 0.29032 | 16 | 0.51613 |
| 979 | S1_140496632 | 1 | 140496632 | 20 | G | A | G | 0.90323 | A | 0.09677 | 6 | 0.19355 |
| 980 | S1_140496655 | 1 | 140496655 | 23 | G | A | A | 0.70968 | G | 0.29032 | 16 | 0.51613 |
| 981 | S1_140496674 | 1 | 140496674 | 19 | A | G | G | 0.82258 | A | 0.17742 | 9 | 0.29032 |
| 982 | S1_140688261 | 1 | 140688261 | 191587 | T | C | T | 0.54839 | C | 0.45161 | 18 | 0.58065 |
| 983 | S1_140688377 | 1 | 140688377 | 116 | A | G | A | 0.66129 | G | 0.33871 | 13 | 0.41935 |
| 984 | S1_141358240 | 1 | 141358240 | 669863 | G | A | G | 0.74194 | A | 0.25806 | 12 | 0.3871 |
| 985 | S1_141358300 | 1 | 141358300 | 60 | T | C | C | 0.58065 | T | 0.41935 | 12 | 0.3871 |
| 986 | S1_141437102 | 1 | 141437102 | 78802 | G | A | G | 0.62903 | A | 0.37097 | 11 | 0.35484 |
| 987 | S1_141437122 | 1 | 141437122 | 20 | T | C | T | 0.85484 | C | 0.14516 | 7 | 0.22581 |
| 988 | S1_141437245 | 1 | 141437245 | 123 | C | T | C | 0.87097 | T | 0.12903 | 6 | 0.19355 |
| 989 | S1_141487835 | 1 | 141487835 | 50590 | T | C | C | 0.53226 | T | 0.46774 | 15 | 0.48387 |
| 990 | S1_141567555 | 1 | 141567555 | 79720 | T | C | T | 0.87097 | C | 0.12903 | 8 | 0.25806 |
| 991 | S1_141567714 | 1 | 141567714 | 159 | T | A | T | 0.8871 | A | 0.1129 | 5 | 0.16129 |
| 992 | S1_141647117 | 1 | 141647117 | 79403 | G | A | G | 0.91935 | A | 0.08065 | 5 | 0.16129 |
| 993 | S1_141647185 | 1 | 141647185 | 68 | G | A | G | 0.56452 | A | 0.43548 | 19 | 0.6129 |
| 994 | S1_141647222 | 1 | 141647222 | 37 | C | T | C | 0.91935 | T | 0.08065 | 5 | 0.16129 |
| 995 | S1_141866196 | 1 | 141866196 | 218974 | C | T | C | 0.85484 | T | 0.14516 | 9 | 0.29032 |
| 996 | S1_141996159 | 1 | 141996159 | 129963 | G | A | G | 0.93548 | A | 0.06452 | 4 | 0.12903 |
| 997 | S1_142187542 | 1 | 142187542 | 191383 | T | C | C | 0.80645 | T | 0.19355 | 10 | 0.32258 |
| 998 | S1_142187636 | 1 | 142187636 | 94 | T | C | T | 0.6129 | C | 0.3871 | 18 | 0.58065 |
| 999 | S1_142187710 | 1 | 142187710 | 74 | T | C | C | 0.80645 | T | 0.19355 | 10 | 0.32258 |
| 1000 | S1_142236692 | 1 | 142236692 | 48982 | C | T | C | 0.79032 | T | 0.20968 | 11 | 0.35484 |
| 1001 | S1_142236761 | 1 | 142236761 | 69 | G | A | G | 0.56452 | A | 0.43548 | 17 | 0.54839 |
| 1002 | S1_142236773 | 1 | 142236773 | 12 | G | A | G | 0.56452 | A | 0.43548 | 17 | 0.54839 |
| 1003 | S1_142236785 | 1 | 142236785 | 12 | C | A | C | 0.91935 | A | 0.08065 | 5 | 0.16129 |
| 1004 | S1_142236796 | 1 | 142236796 | 11 | T | C | T | 0.54839 | C | 0.45161 | 16 | 0.51613 |
| 1005 | S1_142236797 | 1 | 142236797 | 1 | G | A | G | 0.54839 | A | 0.45161 | 16 | 0.51613 |
| 1006 | S1_142272228 | 1 | 142272228 | 35431 | G | A | G | 0.79032 | A | 0.20968 | 9 | 0.29032 |
| 1007 | S1_142272246 | 1 | 142272246 | 18 | A | G | A | 0.79032 | G | 0.20968 | 9 | 0.29032 |
| 1008 | S1_142272267 | 1 | 142272267 | 21 | C | T | C | 0.79032 | T | 0.20968 | 9 | 0.29032 |
| 1009 | S1_142272299 | 1 | 142272299 | 32 | A | C | A | 0.79032 | C | 0.20968 | 9 | 0.29032 |
| 1010 | S1_142272306 | 1 | 142272306 | 7 | A | G | A | 0.79032 | G | 0.20968 | 9 | 0.29032 |
| 1011 | S1_142272317 | 1 | 142272317 | 11 | T | C | T | 0.79032 | C | 0.20968 | 9 | 0.29032 |
| 1012 | S1_142272337 | 1 | 142272337 | 20 | C | T | C | 0.80645 | T | 0.19355 | 12 | 0.3871 |
| 1013 | S1_142272339 | 1 | 142272339 | 2 | C | T | C | 0.79032 | T | 0.20968 | 9 | 0.29032 |
| 1014 | S1_142272348 | 1 | 142272348 | 9 | G | T | G | 0.79032 | T | 0.20968 | 9 | 0.29032 |
| 1015 | S1_142272409 | 1 | 142272409 | 61 | T | G | T | 0.79032 | G | 0.20968 | 9 | 0.29032 |
| 1016 | S1_142272450 | 1 | 142272450 | 41 | G | C | G | 0.79032 | C | 0.20968 | 9 | 0.29032 |
| 1017 | S1_142405434 | 1 | 142405434 | 132984 | G | A | G | 0.93548 | A | 0.06452 | 4 | 0.12903 |
| 1018 | S1_142405461 | 1 | 142405461 | 27 | A | G | A | 0.85484 | G | 0.14516 | 9 | 0.29032 |
| 1019 | S1_142405468 | 1 | 142405468 | 7 | T | A | T | 0.85484 | A | 0.14516 | 9 | 0.29032 |
| 1020 | S1_142405497 | 1 | 142405497 | 29 | T | C | T | 0.85484 | C | 0.14516 | 9 | 0.29032 |
| 1021 | S1_142405499 | 1 | 142405499 | 2 | C | A | C | 0.85484 | A | 0.14516 | 9 | 0.29032 |
| 1022 | S1_142405535 | 1 | 142405535 | 36 | C | T | C | 0.85484 | T | 0.14516 | 9 | 0.29032 |
| 1023 | S1_142405568 | 1 | 142405568 | 33 | T | A | T | 0.87097 | A | 0.12903 | 8 | 0.25806 |
| 1024 | S1_142405578 | 1 | 142405578 | 10 | C | A | C | 0.87097 | A | 0.12903 | 8 | 0.25806 |

| Site number | SNP name | Chromosome | Physical position | Physical distance from the previous SNP | Reference allele | Alternative allele | Major allele | Major allele frequency | Minor allele | Minor allele frequency | Number heterozygous | Heterozygosity |
|---|---|---|---|---|---|---|---|---|---|---|---|---|
| 1025 | S1_142405593 | 1 | 142405593 | 15 | A | G | A | 0.93548 | G | 0.06452 | 4 | 0.12903 |
| 1026 | S1_142405609 | 1 | 142405609 | 16 | A | G | A | 0.93548 | G | 0.06452 | 4 | 0.12903 |
| 1027 | S1_142405655 | 1 | 142405655 | 46 | T | A | A | 0.72581 | T | 0.27419 | 11 | 0.35484 |
| 1028 | S1_142405664 | 1 | 142405664 | 9 | T | C | T | 0.87097 | C | 0.12903 | 8 | 0.25806 |
| 1029 | S1_142683284 | 1 | 142683284 | 277620 | G | A | G | 0.90323 | A | 0.09677 | 6 | 0.19355 |
| 1030 | S1_142683416 | 1 | 142683416 | 132 | T | C | T | 0.90323 | C | 0.09677 | 6 | 0.19355 |
| 1031 | S1_142683451 | 1 | 142683451 | 35 | T | G | T | 0.90323 | G | 0.09677 | 6 | 0.19355 |
| 1032 | S1_142699538 | 1 | 142699538 | 16087 | A | C | A | 0.91935 | C | 0.08065 | 5 | 0.16129 |
| 1033 | S1_142699556 | 1 | 142699556 | 18 | C | T | C | 0.91935 | T | 0.08065 | 5 | 0.16129 |
| 1034 | S1_142699585 | 1 | 142699585 | 29 | T | C | T | 0.93548 | C | 0.06452 | 4 | 0.12903 |
| 1035 | S1_142699688 | 1 | 142699688 | 103 | A | T | A | 0.82258 | T | 0.17742 | 9 | 0.29032 |
| 1036 | S1_142849022 | 1 | 142849022 | 149334 | A | G | A | 0.83871 | G | 0.16129 | 10 | 0.32258 |
| 1037 | S1_142849048 | 1 | 142849048 | 26 | C | T | C | 0.79032 | T | 0.20968 | 9 | 0.29032 |
| 1038 | S1_142940210 | 1 | 142940210 | 91162 | C | T | T | 0.54839 | C | 0.45161 | 18 | 0.58065 |
| 1039 | S1_143383548 | 1 | 143383548 | 443338 | A | G | A | 0.82258 | G | 0.17742 | 9 | 0.29032 |
| 1040 | S1_143383584 | 1 | 143383584 | 36 | T | C | T | 0.82258 | C | 0.17742 | 9 | 0.29032 |
| 1041 | S1_143383594 | 1 | 143383594 | 10 | C | T | C | 0.82258 | T | 0.17742 | 9 | 0.29032 |
| 1042 | S1_143383599 | 1 | 143383599 | 5 | G | A | G | 0.82258 | A | 0.17742 | 9 | 0.29032 |
| 1043 | S1_143383601 | 1 | 143383601 | 2 | T | C | T | 0.82258 | C | 0.17742 | 9 | 0.29032 |
| 1044 | S1_143383628 | 1 | 143383628 | 27 | T | C | T | 0.82258 | C | 0.17742 | 9 | 0.29032 |
| 1045 | S1_143383646 | 1 | 143383646 | 18 | A | G | A | 0.82258 | G | 0.17742 | 9 | 0.29032 |
| 1046 | S1_143383654 | 1 | 143383654 | 8 | C | T | C | 0.90323 | T | 0.09677 | 6 | 0.19355 |
| 1047 | S1_143383692 | 1 | 143383692 | 38 | T | A | T | 0.82258 | A | 0.17742 | 9 | 0.29032 |
| 1048 | S1_143383715 | 1 | 143383715 | 23 | T | C | T | 0.82258 | C | 0.17742 | 9 | 0.29032 |
| 1049 | S1_143383719 | 1 | 143383719 | 4 | A | G | A | 0.82258 | G | 0.17742 | 9 | 0.29032 |
| 1050 | S1_143383770 | 1 | 143383770 | 51 | C | T | C | 0.67742 | T | 0.32258 | 14 | 0.45161 |
| 1051 | S1_143383773 | 1 | 143383773 | 3 | T | C | T | 0.67742 | C | 0.32258 | 14 | 0.45161 |
| 1052 | S1_143383778 | 1 | 143383778 | 5 | C | T | C | 0.67742 | T | 0.32258 | 14 | 0.45161 |
| 1053 | S1_143383781 | 1 | 143383781 | 3 | C | A | C | 0.67742 | A | 0.32258 | 14 | 0.45161 |
| 1054 | S1_143383784 | 1 | 143383784 | 3 | A | G | A | 0.67742 | G | 0.32258 | 14 | 0.45161 |
| 1055 | S1_143612694 | 1 | 143612694 | 228910 | G | C | G | 0.8871 | C | 0.1129 | 7 | 0.22581 |
| 1056 | S1_144382487 | 1 | 144382487 | 769793 | T | G | T | 0.93548 | G | 0.06452 | 4 | 0.12903 |
| 1057 | S1_144382493 | 1 | 144382493 | 6 | G | A | G | 0.85484 | A | 0.14516 | 7 | 0.22581 |
| 1058 | S1_144382530 | 1 | 144382530 | 37 | G | A | G | 0.69355 | A | 0.30645 | 11 | 0.35484 |
| 1059 | S1_144382586 | 1 | 144382586 | 56 | T | A | A | 0.69355 | T | 0.30645 | 13 | 0.41935 |
| 1060 | S1_144420780 | 1 | 144420780 | 38194 | A | G | A | 0.77419 | G | 0.22581 | 10 | 0.32258 |
| 1061 | S1_144420787 | 1 | 144420787 | 7 | A | G | A | 0.77419 | G | 0.22581 | 10 | 0.32258 |
| 1062 | S1_144420836 | 1 | 144420836 | 49 | G | A | G | 0.77419 | A | 0.22581 | 10 | 0.32258 |
| 1063 | S1_144420955 | 1 | 144420955 | 119 | T | C | T | 0.8871 | C | 0.1129 | 7 | 0.22581 |
| 1064 | S1_144420983 | 1 | 144420983 | 28 | A | C | A | 0.87097 | C | 0.12903 | 6 | 0.19355 |
| 1065 | S1_144420994 | 1 | 144420994 | 11 | C | T | C | 0.77419 | T | 0.22581 | 10 | 0.32258 |
| 1066 | S1_145519914 | 1 | 145519914 | 1098920 | G | A | A | 0.91935 | G | 0.08065 | 5 | 0.16129 |
| 1067 | S1_145520081 | 1 | 145520081 | 167 | C | T | T | 0.67742 | C | 0.32258 | 18 | 0.58065 |
| 1068 | S1_145527995 | 1 | 145527995 | 7914 | G | A | G | 0.62903 | A | 0.37097 | 11 | 0.35484 |
| 1069 | S1_145599370 | 1 | 145599370 | 71375 | G | A | A | 0.53226 | G | 0.46774 | 19 | 0.6129 |
| 1070 | S1_145599512 | 1 | 145599512 | 142 | T | C | C | 0.53226 | T | 0.46774 | 19 | 0.6129 |
| 1071 | S1_145599599 | 1 | 145599599 | 87 | G | A | A | 0.53226 | G | 0.46774 | 19 | 0.6129 |
| 1072 | S1_145743812 | 1 | 145743812 | 144213 | A | G | A | 0.59677 | G | 0.40323 | 13 | 0.41935 |
| 1073 | S1_145743887 | 1 | 145743887 | 75 | A | G | A | 0.72581 | G | 0.27419 | 11 | 0.35484 |
| 1074 | S1_145838693 | 1 | 145838693 | 94806 | G | A | G | 0.87097 | A | 0.12903 | 8 | 0.25806 |
| 1075 | S1_145838727 | 1 | 145838727 | 34 | C | A | C | 0.90323 | A | 0.09677 | 6 | 0.19355 |
| 1076 | S1_145838748 | 1 | 145838748 | 21 | A | G | A | 0.90323 | G | 0.09677 | 6 | 0.19355 |
| 1077 | S1_145838759 | 1 | 145838759 | 11 | C | T | C | 0.82258 | T | 0.17742 | 11 | 0.35484 |
| 1078 | S1_145838768 | 1 | 145838768 | 9 | C | G | C | 0.90323 | G | 0.09677 | 6 | 0.19355 |

| Site number | SNP name | Chromosome | Physical position | Physical distance from the previous SNP | Reference allele | Alternative allele | Major allele | Major allele frequency | Minor allele | Minor allele frequency | Number heterozygous | Heterozygosity |
|---|---|---|---|---|---|---|---|---|---|---|---|---|
| 1079 | S1_145838792 | 1 | 145838792 | 24 | C | T | C | 0.95161 | T | 0.04839 | 3 | 0.09677 |
| 1080 | S1_145838816 | 1 | 145838816 | 24 | T | C | T | 0.70968 | C | 0.29032 | 16 | 0.51613 |
| 1081 | S1_145847446 | 1 | 145847446 | 8630 | A | G | A | 0.8871 | G | 0.1129 | 7 | 0.22581 |
| 1082 | S1_145847522 | 1 | 145847522 | 76 | T | C | T | 0.75806 | C | 0.24194 | 15 | 0.48387 |
| 1083 | S1_145911861 | 1 | 145911861 | 64339 | A | T | A | 0.79032 | T | 0.20968 | 7 | 0.22581 |
| 1084 | S1_145911866 | 1 | 145911866 | 5 | G | A | G | 0.79032 | A | 0.20968 | 7 | 0.22581 |
| 1085 | S1_145911910 | 1 | 145911910 | 44 | G | A | G | 0.93548 | A | 0.06452 | 4 | 0.12903 |
| 1086 | S1_145912029 | 1 | 145912029 | 119 | C | T | C | 0.93548 | T | 0.06452 | 4 | 0.12903 |
| 1087 | S1_145912094 | 1 | 145912094 | 65 | C | T | C | 0.79032 | T | 0.20968 | 7 | 0.22581 |
| 1088 | S1_146019858 | 1 | 146019858 | 107764 | G | A | G | 0.59677 | A | 0.40323 | 13 | 0.41935 |
| 1089 | S1_146024566 | 1 | 146024566 | 4708 | C | T | C | 0.91935 | T | 0.08065 | 5 | 0.16129 |
| 1090 | S1_146565151 | 1 | 146565151 | 540585 | T | C | T | 0.85484 | C | 0.14516 | 9 | 0.29032 |
| 1091 | S1_146565206 | 1 | 146565206 | 55 | C | T | C | 0.90323 | T | 0.09677 | 4 | 0.12903 |
| 1092 | S1_146565244 | 1 | 146565244 | 38 | G | A | G | 0.54839 | A | 0.45161 | 10 | 0.32258 |
| 1093 | S1_146565248 | 1 | 146565248 | 4 | T | C | T | 0.54839 | C | 0.45161 | 10 | 0.32258 |
| 1094 | S1_146565271 | 1 | 146565271 | 23 | C | T | C | 0.54839 | T | 0.45161 | 10 | 0.32258 |
| 1095 | S1_146565281 | 1 | 146565281 | 10 | A | G | A | 0.90323 | G | 0.09677 | 6 | 0.19355 |
| 1096 | S1_146565292 | 1 | 146565292 | 11 | G | A | G | 0.85484 | A | 0.14516 | 9 | 0.29032 |
| 1097 | S1_146565332 | 1 | 146565332 | 40 | A | G | A | 0.54839 | G | 0.45161 | 10 | 0.32258 |
| 1098 | S1_146998877 | 1 | 146998877 | 433545 | G | A | G | 0.93548 | A | 0.06452 | 2 | 0.06452 |
| 1099 | S1_147187714 | 1 | 147187714 | 188837 | T | C | T | 0.74194 | C | 0.25806 | 14 | 0.45161 |
| 1100 | S1_147187774 | 1 | 147187774 | 60 | C | T | C | 0.93548 | T | 0.06452 | 4 | 0.12903 |
| 1101 | S1_147187837 | 1 | 147187837 | 63 | C | G | C | 0.91935 | G | 0.08065 | 3 | 0.09677 |
| 1102 | S1_147322106 | 1 | 147322106 | 134269 | A | C | A | 0.90323 | C | 0.09677 | 6 | 0.19355 |
| 1103 | S1_147427987 | 1 | 147427987 | 105881 | A | G | A | 0.91935 | G | 0.08065 | 5 | 0.16129 |
| 1104 | S1_147428029 | 1 | 147428029 | 42 | G | A | G | 0.91935 | A | 0.08065 | 5 | 0.16129 |
| 1105 | S1_147428050 | 1 | 147428050 | 21 | T | C | T | 0.91935 | C | 0.08065 | 5 | 0.16129 |
| 1106 | S1_147428146 | 1 | 147428146 | 96 | G | A | G | 0.91935 | A | 0.08065 | 5 | 0.16129 |
| 1107 | S1_147428169 | 1 | 147428169 | 23 | T | C | T | 0.91935 | C | 0.08065 | 5 | 0.16129 |
| 1108 | S1_147428202 | 1 | 147428202 | 33 | G | A | G | 0.91935 | A | 0.08065 | 5 | 0.16129 |
| 1109 | S1_147428212 | 1 | 147428212 | 10 | T | G | T | 0.64516 | G | 0.35484 | 14 | 0.45161 |
| 1110 | S1_147846123 | 1 | 147846123 | 417911 | G | T | G | 0.87097 | T | 0.12903 | 6 | 0.19355 |
| 1111 | S1_147846289 | 1 | 147846289 | 166 | G | A | G | 0.8871 | A | 0.1129 | 7 | 0.22581 |
| 1112 | S1_148087365 | 1 | 148087365 | 241076 | T | C | T | 0.77419 | C | 0.22581 | 14 | 0.45161 |
| 1113 | S1_148366820 | 1 | 148366820 | 279455 | G | A | G | 0.87097 | A | 0.12903 | 4 | 0.12903 |
| 1114 | S1_148634102 | 1 | 148634102 | 267282 | T | C | T | 0.91935 | C | 0.08065 | 5 | 0.16129 |
| 1115 | S1_148634114 | 1 | 148634114 | 12 | C | T | C | 0.8871 | T | 0.1129 | 7 | 0.22581 |
| 1116 | S1_148634221 | 1 | 148634221 | 107 | G | A | G | 0.83871 | A | 0.16129 | 10 | 0.32258 |
| 1117 | S1_148634272 | 1 | 148634272 | 51 | A | G | A | 0.80645 | G | 0.19355 | 12 | 0.3871 |
| 1118 | S1_148779765 | 1 | 148779765 | 145493 | C | G | C | 0.95161 | G | 0.04839 | 3 | 0.09677 |
| 1119 | S1_148779952 | 1 | 148779952 | 187 | T | A | T | 0.91935 | A | 0.08065 | 5 | 0.16129 |
| 1120 | S1_149004721 | 1 | 149004721 | 224769 | G | A | G | 0.95161 | A | 0.04839 | 3 | 0.09677 |
| 1121 | S1_149004749 | 1 | 149004749 | 28 | G | A | G | 0.95161 | A | 0.04839 | 3 | 0.09677 |
| 1122 | S1_149004907 | 1 | 149004907 | 158 | A | G | A | 0.90323 | G | 0.09677 | 6 | 0.19355 |
| 1123 | S1_149320618 | 1 | 149320618 | 315711 | C | G | C | 0.87097 | G | 0.12903 | 8 | 0.25806 |
| 1124 | S1_149532371 | 1 | 149532371 | 211753 | G | A | G | 0.91935 | A | 0.08065 | 5 | 0.16129 |
| 1125 | S1_149547053 | 1 | 149547053 | 14682 | A | G | A | 0.5 | G | 0.5 | 17 | 0.54839 |
| 1126 | S1_149686772 | 1 | 149686772 | 139719 | A | C | A | 0.91935 | C | 0.08065 | 5 | 0.16129 |
| 1127 | S1_149686967 | 1 | 149686967 | 195 | T | C | T | 0.91935 | C | 0.08065 | 5 | 0.16129 |
| 1128 | S1_150204128 | 1 | 150204128 | 517161 | T | A | A | 0.53226 | T | 0.46774 | 21 | 0.67742 |
| 1129 | S1_150204239 | 1 | 150204239 | 111 | C | A | C | 0.93548 | A | 0.06452 | 4 | 0.12903 |
| 1130 | S1_150457670 | 1 | 150457670 | 253431 | C | T | C | 0.64516 | T | 0.35484 | 10 | 0.32258 |
| 1131 | S1_150457671 | 1 | 150457671 | 1 | A | G | A | 0.64516 | G | 0.35484 | 10 | 0.32258 |
| 1132 | S1_151116074 | 1 | 151116074 | 658403 | C | T | T | 0.53226 | C | 0.46774 | 15 | 0.48387 |

| Site number | SNP name | Chromosome | Physical position | Physical distance from the previous SNP | Reference allele | Alternative allele | Major allele | Major allele frequency | Minor allele | Minor allele frequency | Number heterozygous | Heterozygosity |
|---|---|---|---|---|---|---|---|---|---|---|---|---|
| 1133 | S1_151116084 | 1 | 151116084 | 10 | C | T | C | 0.80645 | T | 0.19355 | 12 | 0.3871 |
| 1134 | S1_151340829 | 1 | 151340829 | 224745 | A | G | A | 0.95161 | G | 0.04839 | 3 | 0.09677 |
| 1135 | S1_151701660 | 1 | 151701660 | 360831 | T | C | C | 0.80645 | T | 0.19355 | 12 | 0.3871 |
| 1136 | S1_151701661 | 1 | 151701661 | 1 | G | A | G | 0.80645 | A | 0.19355 | 12 | 0.3871 |
| 1137 | S1_151701667 | 1 | 151701667 | 6 | T | C | T | 0.85484 | C | 0.14516 | 9 | 0.29032 |
| 1138 | S1_151701668 | 1 | 151701668 | 1 | A | T | A | 0.85484 | T | 0.14516 | 9 | 0.29032 |
| 1139 | S1_151819374 | 1 | 151819374 | 117706 | A | G | G | 0.54839 | A | 0.45161 | 20 | 0.64516 |
| 1140 | S1_151819446 | 1 | 151819446 | 72 | A | G | G | 0.54839 | A | 0.45161 | 20 | 0.64516 |
| 1141 | S1_152172527 | 1 | 152172527 | 353081 | G | A | G | 0.8871 | A | 0.1129 | 7 | 0.22581 |
| 1142 | S1_152172611 | 1 | 152172611 | 84 | G | A | G | 0.80645 | A | 0.19355 | 8 | 0.25806 |
| 1143 | S1_152184582 | 1 | 152184582 | 11971 | T | C | T | 0.53226 | C | 0.46774 | 15 | 0.48387 |
| 1144 | S1_152310318 | 1 | 152310318 | 125736 | G | A | G | 0.8871 | A | 0.1129 | 7 | 0.22581 |
| 1145 | S1_152345590 | 1 | 152345590 | 35272 | T | C | C | 0.80645 | T | 0.19355 | 12 | 0.3871 |
| 1146 | S1_152345651 | 1 | 152345651 | 61 | T | C | C | 0.80645 | T | 0.19355 | 12 | 0.3871 |
| 1147 | S1_152523431 | 1 | 152523431 | 177780 | C | T | C | 0.79032 | T | 0.20968 | 11 | 0.35484 |
| 1148 | S1_152706050 | 1 | 152706050 | 182619 | T | C | T | 0.6129 | C | 0.3871 | 14 | 0.45161 |
| 1149 | S1_152775529 | 1 | 152775529 | 69479 | T | C | C | 0.70968 | T | 0.29032 | 14 | 0.45161 |
| 1150 | S1_152775530 | 1 | 152775530 | 1 | G | A | G | 0.82258 | A | 0.17742 | 9 | 0.29032 |
| 1151 | S1_152775606 | 1 | 152775606 | 76 | G | A | G | 0.95161 | A | 0.04839 | 3 | 0.09677 |
| 1152 | S1_152922405 | 1 | 152922405 | 146799 | A | C | C | 0.62903 | A | 0.37097 | 17 | 0.54839 |
| 1153 | S1_152922539 | 1 | 152922539 | 134 | C | T | C | 0.75806 | T | 0.24194 | 11 | 0.35484 |
| 1154 | S1_153045130 | 1 | 153045130 | 122591 | C | T | C | 0.77419 | T | 0.22581 | 10 | 0.32258 |
| 1155 | S1_153109282 | 1 | 153109282 | 64152 | T | C | C | 0.91935 | T | 0.08065 | 5 | 0.16129 |
| 1156 | S1_153109399 | 1 | 153109399 | 117 | G | T | G | 0.72581 | T | 0.27419 | 11 | 0.35484 |
| 1157 | S1_153109400 | 1 | 153109400 | 1 | T | A | T | 0.70968 | A | 0.29032 | 12 | 0.3871 |
| 1158 | S1_153154748 | 1 | 153154748 | 45348 | T | G | T | 0.93548 | G | 0.06452 | 4 | 0.12903 |
| 1159 | S1_153154807 | 1 | 153154807 | 59 | T | G | T | 0.95161 | G | 0.04839 | 3 | 0.09677 |
| 1160 | S1_153256713 | 1 | 153256713 | 101906 | C | T | C | 0.75806 | T | 0.24194 | 9 | 0.29032 |
| 1161 | S1_153291563 | 1 | 153291563 | 34850 | T | C | T | 0.82258 | C | 0.17742 | 9 | 0.29032 |
| 1162 | S1_153291641 | 1 | 153291641 | 78 | G | C | G | 0.82258 | C | 0.17742 | 9 | 0.29032 |
| 1163 | S1_153291662 | 1 | 153291662 | 21 | C | G | C | 0.87097 | G | 0.12903 | 8 | 0.25806 |
| 1164 | S1_153518909 | 1 | 153518909 | 227247 | G | T | G | 0.77419 | T | 0.22581 | 10 | 0.32258 |
| 1165 | S1_153518956 | 1 | 153518956 | 47 | T | C | C | 0.53226 | T | 0.46774 | 15 | 0.48387 |
| 1166 | S1_153519000 | 1 | 153519000 | 44 | A | G | G | 0.77419 | A | 0.22581 | 10 | 0.32258 |
| 1167 | S1_153519036 | 1 | 153519036 | 36 | A | G | G | 0.53226 | A | 0.46774 | 15 | 0.48387 |
| 1168 | S1_153546462 | 1 | 153546462 | 27426 | C | T | C | 0.95161 | T | 0.04839 | 3 | 0.09677 |
| 1169 | S1_153546477 | 1 | 153546477 | 15 | A | G | A | 0.69355 | G | 0.30645 | 15 | 0.48387 |
| 1170 | S1_153546494 | 1 | 153546494 | 17 | A | G | A | 0.69355 | G | 0.30645 | 15 | 0.48387 |
| 1171 | S1_153546497 | 1 | 153546497 | 3 | G | A | G | 0.90323 | A | 0.09677 | 6 | 0.19355 |
| 1172 | S1_153546560 | 1 | 153546560 | 63 | C | T | C | 0.59677 | T | 0.40323 | 17 | 0.54839 |
| 1173 | S1_153546664 | 1 | 153546664 | 104 | T | C | T | 0.69355 | C | 0.30645 | 15 | 0.48387 |
| 1174 | S1_153546673 | 1 | 153546673 | 9 | A | G | A | 0.69355 | G | 0.30645 | 15 | 0.48387 |
| 1175 | S1_153546679 | 1 | 153546679 | 6 | T | A | T | 0.69355 | A | 0.30645 | 15 | 0.48387 |
| 1176 | S1_153546682 | 1 | 153546682 | 3 | A | T | A | 0.87097 | T | 0.12903 | 6 | 0.19355 |
| 1177 | S1_153881140 | 1 | 153881140 | 334458 | A | G | A | 0.75806 | G | 0.24194 | 11 | 0.35484 |
| 1178 | S1_153881251 | 1 | 153881251 | 111 | A | G | A | 0.91935 | G | 0.08065 | 5 | 0.16129 |
| 1179 | S1_153881292 | 1 | 153881292 | 41 | G | A | G | 0.75806 | A | 0.24194 | 11 | 0.35484 |
| 1180 | S1_153926916 | 1 | 153926916 | 45624 | A | C | C | 0.58065 | A | 0.41935 | 12 | 0.3871 |
| 1181 | S1_153987130 | 1 | 153987130 | 60214 | C | G | G | 0.79032 | C | 0.20968 | 9 | 0.29032 |
| 1182 | S1_153987138 | 1 | 153987138 | 8 | C | T | T | 0.67742 | C | 0.32258 | 10 | 0.32258 |
| 1183 | S1_153987177 | 1 | 153987177 | 39 | T | C | T | 0.90323 | C | 0.09677 | 6 | 0.19355 |
| 1184 | S1_153990920 | 1 | 153990920 | 3743 | C | T | C | 0.90323 | T | 0.09677 | 6 | 0.19355 |
| 1185 | S1_154499287 | 1 | 154499287 | 508367 | G | C | G | 0.82258 | C | 0.17742 | 11 | 0.35484 |
| 1186 | S1_154499433 | 1 | 154499433 | 146 | A | G | A | 0.82258 | G | 0.17742 | 11 | 0.35484 |

| Site number | SNP name | Chromosome | Physical position | Physical distance from the previous SNP | Reference allele | Alternative allele | Major allele | Major allele frequency | Minor allele | Minor allele frequency | Number heterozygous | Heterozygosity |
|---|---|---|---|---|---|---|---|---|---|---|---|---|
| 1187 | S1_154499452 | 1 | 154499452 | 19 | C | T | C | 0.82258 | T | 0.17742 | 11 | 0.35484 |
| 1188 | S1_154499494 | 1 | 154499494 | 42 | C | T | C | 0.83871 | T | 0.16129 | 8 | 0.25806 |
| 1189 | S1_154561510 | 1 | 154561510 | 62016 | T | C | T | 0.51613 | C | 0.48387 | 20 | 0.64516 |
| 1190 | S1_154561618 | 1 | 154561618 | 108 | T | C | T | 0.51613 | C | 0.48387 | 20 | 0.64516 |
| 1191 | S1_154561661 | 1 | 154561661 | 43 | C | T | C | 0.51613 | T | 0.48387 | 20 | 0.64516 |
| 1192 | S1_154561680 | 1 | 154561680 | 19 | A | G | A | 0.51613 | G | 0.48387 | 20 | 0.64516 |
| 1193 | S1_154595039 | 1 | 154595039 | 33359 | G | A | A | 0.72581 | G | 0.27419 | 15 | 0.48387 |
| 1194 | S1_154727941 | 1 | 154727941 | 132902 | T | C | T | 0.58065 | C | 0.41935 | 14 | 0.45161 |
| 1195 | S1_154727966 | 1 | 154727966 | 25 | G | A | G | 0.58065 | A | 0.41935 | 14 | 0.45161 |
| 1196 | S1_154798578 | 1 | 154798578 | 70612 | C | T | C | 0.66129 | T | 0.33871 | 13 | 0.41935 |
| 1197 | S1_154798704 | 1 | 154798704 | 126 | G | T | T | 0.54839 | G | 0.45161 | 16 | 0.51613 |
| 1198 | S1_154798718 | 1 | 154798718 | 14 | C | T | C | 0.87097 | T | 0.12903 | 8 | 0.25806 |
| 1199 | S1_154798805 | 1 | 154798805 | 87 | T | C | T | 0.66129 | C | 0.33871 | 13 | 0.41935 |
| 1200 | S1_154803853 | 1 | 154803853 | 5048 | T | C | C | 0.95161 | T | 0.04839 | 3 | 0.09677 |
| 1201 | S1_154952355 | 1 | 154952355 | 148502 | T | C | C | 0.64516 | T | 0.35484 | 16 | 0.51613 |
| 1202 | S1_154961287 | 1 | 154961287 | 8932 | G | T | G | 0.93548 | T | 0.06452 | 2 | 0.06452 |
| 1203 | S1_155272634 | 1 | 155272634 | 311347 | T | C | T | 0.70968 | C | 0.29032 | 14 | 0.45161 |
| 1204 | S1_155272675 | 1 | 155272675 | 41 | T | C | T | 0.69355 | C | 0.30645 | 13 | 0.41935 |
| 1205 | S1_155272728 | 1 | 155272728 | 53 | C | T | C | 0.79032 | T | 0.20968 | 11 | 0.35484 |
| 1206 | S1_155272781 | 1 | 155272781 | 53 | G | A | A | 0.5 | G | 0.5 | 17 | 0.54839 |
| 1207 | S1_155321600 | 1 | 155321600 | 48819 | A | G | A | 0.53226 | G | 0.46774 | 17 | 0.54839 |
| 1208 | S1_155321628 | 1 | 155321628 | 28 | C | T | C | 0.79032 | T | 0.20968 | 13 | 0.41935 |
| 1209 | S1_155321678 | 1 | 155321678 | 50 | G | T | G | 0.77419 | T | 0.22581 | 12 | 0.3871 |
| 1210 | S1_155674654 | 1 | 155674654 | 352976 | T | G | G | 0.82258 | T | 0.17742 | 9 | 0.29032 |
| 1211 | S1_155674686 | 1 | 155674686 | 32 | C | T | T | 0.82258 | C | 0.17742 | 9 | 0.29032 |
| 1212 | S1_155674687 | 1 | 155674687 | 1 | A | G | G | 0.82258 | A | 0.17742 | 9 | 0.29032 |
| 1213 | S1_155674741 | 1 | 155674741 | 54 | A | G | G | 0.82258 | A | 0.17742 | 9 | 0.29032 |
| 1214 | S1_155674777 | 1 | 155674777 | 36 | T | G | G | 0.82258 | T | 0.17742 | 9 | 0.29032 |
| 1215 | S1_155674804 | 1 | 155674804 | 27 | T | C | C | 0.82258 | T | 0.17742 | 9 | 0.29032 |
| 1216 | S1_155759728 | 1 | 155759728 | 84924 | T | A | T | 0.91935 | A | 0.08065 | 5 | 0.16129 |
| 1217 | S1_155759732 | 1 | 155759732 | 4 | T | C | T | 0.91935 | C | 0.08065 | 5 | 0.16129 |
| 1218 | S1_156016880 | 1 | 156016880 | 257148 | A | G | G | 0.70968 | A | 0.29032 | 12 | 0.3871 |
| 1219 | S1_156016965 | 1 | 156016965 | 85 | A | T | A | 0.95161 | T | 0.04839 | 3 | 0.09677 |
| 1220 | S1_156017013 | 1 | 156017013 | 48 | C | T | C | 0.82258 | T | 0.17742 | 9 | 0.29032 |
| 1221 | S1_156146556 | 1 | 156146556 | 129543 | G | A | G | 0.53226 | A | 0.46774 | 17 | 0.54839 |
| 1222 | S1_156146607 | 1 | 156146607 | 51 | C | T | C | 0.95161 | T | 0.04839 | 3 | 0.09677 |
| 1223 | S1_156507420 | 1 | 156507420 | 360813 | G | T | G | 0.87097 | T | 0.12903 | 8 | 0.25806 |
| 1224 | S1_156507475 | 1 | 156507475 | 55 | T | C | T | 0.6129 | C | 0.3871 | 18 | 0.58065 |
| 1225 | S1_156507489 | 1 | 156507489 | 14 | G | A | G | 0.87097 | A | 0.12903 | 8 | 0.25806 |
| 1226 | S1_156507592 | 1 | 156507592 | 103 | G | A | G | 0.90323 | A | 0.09677 | 6 | 0.19355 |
| 1227 | S1_156518667 | 1 | 156518667 | 11075 | C | T | C | 0.8871 | T | 0.1129 | 7 | 0.22581 |
| 1228 | S1_156518764 | 1 | 156518764 | 97 | G | A | A | 0.56452 | G | 0.43548 | 13 | 0.41935 |
| 1229 | S1_156518786 | 1 | 156518786 | 22 | C | T | T | 0.80645 | C | 0.19355 | 8 | 0.25806 |
| 1230 | S1_156518804 | 1 | 156518804 | 18 | A | G | G | 0.56452 | A | 0.43548 | 13 | 0.41935 |
| 1231 | S1_156621463 | 1 | 156621463 | 102659 | C | T | C | 0.64516 | T | 0.35484 | 16 | 0.51613 |
| 1232 | S1_156621581 | 1 | 156621581 | 118 | C | G | C | 0.95161 | G | 0.04839 | 3 | 0.09677 |
| 1233 | S1_156885497 | 1 | 156885497 | 263916 | A | C | A | 0.70968 | C | 0.29032 | 14 | 0.45161 |
| 1234 | S1_156885498 | 1 | 156885498 | 1 | G | A | G | 0.70968 | A | 0.29032 | 14 | 0.45161 |
| 1235 | S1_156885571 | 1 | 156885571 | 73 | A | T | A | 0.66129 | T | 0.33871 | 15 | 0.48387 |
| 1236 | S1_156885573 | 1 | 156885573 | 2 | C | G | C | 0.80645 | G | 0.19355 | 10 | 0.32258 |
| 1237 | S1_156885607 | 1 | 156885607 | 34 | G | A | G | 0.87097 | A | 0.12903 | 8 | 0.25806 |
| 1238 | S1_156885616 | 1 | 156885616 | 9 | C | T | C | 0.82258 | T | 0.17742 | 9 | 0.29032 |
| 1239 | S1_156885716 | 1 | 156885716 | 100 | G | A | G | 0.93548 | A | 0.06452 | 4 | 0.12903 |
| 1240 | S1_156928752 | 1 | 156928752 | 43036 | G | A | G | 0.83871 | A | 0.16129 | 8 | 0.25806 |

| Site number | SNP name | Chromosome | Physical position | Physical distance from the previous SNP | Reference allele | Alternative allele | Major allele | Major allele frequency | Minor allele | Minor allele frequency | Number heterozygous | Heterozygosity |
|---|---|---|---|---|---|---|---|---|---|---|---|---|
| 1241 | S2_179076 | 2 | 179076 | 0 | C | G | C | 0.72581 | G | 0.27419 | 11 | 0.35484 |
| 1242 | S2_324842 | 2 | 324842 | 145766 | C | T | T | 0.54839 | C | 0.45161 | 16 | 0.51613 |
| 1243 | S2_324863 | 2 | 324863 | 21 | G | A | G | 0.8871 | A | 0.1129 | 7 | 0.22581 |
| 1244 | S2_324923 | 2 | 324923 | 60 | C | A | C | 0.91935 | A | 0.08065 | 5 | 0.16129 |
| 1245 | S2_472312 | 2 | 472312 | 147389 | C | G | G | 0.51613 | C | 0.48387 | 16 | 0.51613 |
| 1246 | S2_497141 | 2 | 497141 | 24829 | T | G | T | 0.66129 | G | 0.33871 | 13 | 0.41935 |
| 1247 | S2_497281 | 2 | 497281 | 140 | T | C | T | 0.95161 | C | 0.04839 | 3 | 0.09677 |
| 1248 | S2_606672 | 2 | 606672 | 109391 | G | A | A | 0.53226 | G | 0.46774 | 13 | 0.41935 |
| 1249 | S2_606709 | 2 | 606709 | 37 | C | A | A | 0.53226 | C | 0.46774 | 13 | 0.41935 |
| 1250 | S2_606774 | 2 | 606774 | 65 | C | T | C | 0.91935 | T | 0.08065 | 5 | 0.16129 |
| 1251 | S2_606783 | 2 | 606783 | 9 | C | T | C | 0.64516 | T | 0.35484 | 14 | 0.45161 |
| 1252 | S2_606787 | 2 | 606787 | 4 | T | C | C | 0.56452 | T | 0.43548 | 13 | 0.41935 |
| 1253 | S2_1353761 | 2 | 1353761 | 746974 | C | T | C | 0.69355 | T | 0.30645 | 15 | 0.48387 |
| 1254 | S2_1428467 | 2 | 1428467 | 74706 | C | T | T | 0.59677 | C | 0.40323 | 11 | 0.35484 |
| 1255 | S2_1704503 | 2 | 1704503 | 276036 | C | T | C | 0.95161 | T | 0.04839 | 3 | 0.09677 |
| 1256 | S2_1704525 | 2 | 1704525 | 22 | C | T | C | 0.91935 | T | 0.08065 | 5 | 0.16129 |
| 1257 | S2_1704542 | 2 | 1704542 | 17 | G | A | G | 0.91935 | A | 0.08065 | 3 | 0.09677 |
| 1258 | S2_1704706 | 2 | 1704706 | 164 | A | G | G | 0.93548 | A | 0.06452 | 4 | 0.12903 |
| 1259 | S2_2155863 | 2 | 2155863 | 451157 | C | T | C | 0.90323 | T | 0.09677 | 6 | 0.19355 |
| 1260 | S2_2333666 | 2 | 2333666 | 177803 | T | C | T | 0.8871 | C | 0.1129 | 7 | 0.22581 |
| 1261 | S2_2357958 | 2 | 2357958 | 24292 | G | A | G | 0.90323 | A | 0.09677 | 6 | 0.19355 |
| 1262 | S2_2617381 | 2 | 2617381 | 259423 | T | C | T | 0.85484 | C | 0.14516 | 9 | 0.29032 |
| 1263 | S2_2617541 | 2 | 2617541 | 160 | G | T | G | 0.75806 | T | 0.24194 | 9 | 0.29032 |
| 1264 | S2_2617563 | 2 | 2617563 | 22 | A | G | A | 0.83871 | G | 0.16129 | 10 | 0.32258 |
| 1265 | S2_3687082 | 2 | 3687082 | 1069519 | C | T | C | 0.75806 | T | 0.24194 | 11 | 0.35484 |
| 1266 | S2_3687187 | 2 | 3687187 | 105 | G | A | G | 0.6129 | A | 0.3871 | 16 | 0.51613 |
| 1267 | S2_3930941 | 2 | 3930941 | 243754 | C | T | C | 0.93548 | T | 0.06452 | 4 | 0.12903 |
| 1268 | S2_4018910 | 2 | 4018910 | 87969 | T | C | C | 0.69355 | T | 0.30645 | 15 | 0.48387 |
| 1269 | S2_4062046 | 2 | 4062046 | 43136 | G | C | G | 0.77419 | C | 0.22581 | 12 | 0.3871 |
| 1270 | S2_4181359 | 2 | 4181359 | 119313 | C | T | C | 0.74194 | T | 0.25806 | 14 | 0.45161 |
| 1271 | S2_4215670 | 2 | 4215670 | 34311 | C | T | C | 0.91935 | T | 0.08065 | 5 | 0.16129 |
| 1272 | S2_4215671 | 2 | 4215671 | 1 | A | G | G | 0.91935 | A | 0.08065 | 5 | 0.16129 |
| 1273 | S2_4779704 | 2 | 4779704 | 564033 | T | C | C | 0.93548 | T | 0.06452 | 4 | 0.12903 |
| 1274 | S2_4779715 | 2 | 4779715 | 11 | C | T | C | 0.77419 | T | 0.22581 | 10 | 0.32258 |
| 1275 | S2_5281243 | 2 | 5281243 | 501528 | T | C | C | 0.79032 | T | 0.20968 | 9 | 0.29032 |
| 1276 | S2_5281244 | 2 | 5281244 | 1 | G | A | G | 0.93548 | A | 0.06452 | 4 | 0.12903 |
| 1277 | S2_5450157 | 2 | 5450157 | 168913 | G | A | G | 0.74194 | A | 0.25806 | 10 | 0.32258 |
| 1278 | S2_5521640 | 2 | 5521640 | 71483 | T | C | T | 0.90323 | C | 0.09677 | 6 | 0.19355 |
| 1279 | S2_5521676 | 2 | 5521676 | 36 | C | G | C | 0.83871 | G | 0.16129 | 8 | 0.25806 |
| 1280 | S2_5521678 | 2 | 5521678 | 2 | G | A | G | 0.87097 | A | 0.12903 | 6 | 0.19355 |
| 1281 | S2_5521725 | 2 | 5521725 | 47 | T | C | T | 0.93548 | C | 0.06452 | 4 | 0.12903 |
| 1282 | S2_5812050 | 2 | 5812050 | 290325 | A | T | A | 0.90323 | T | 0.09677 | 6 | 0.19355 |
| 1283 | S2_5886625 | 2 | 5886625 | 74575 | T | C | T | 0.70968 | C | 0.29032 | 12 | 0.3871 |
| 1284 | S2_5902959 | 2 | 5902959 | 16334 | G | A | G | 0.75806 | A | 0.24194 | 11 | 0.35484 |
| 1285 | S2_5903064 | 2 | 5903064 | 105 | G | C | C | 0.66129 | G | 0.33871 | 11 | 0.35484 |
| 1286 | S2_5992148 | 2 | 5992148 | 89084 | T | C | C | 0.8871 | T | 0.1129 | 5 | 0.16129 |
| 1287 | S2_5992275 | 2 | 5992275 | 127 | T | C | T | 0.93548 | C | 0.06452 | 4 | 0.12903 |
| 1288 | S2_6034243 | 2 | 6034243 | 41968 | T | A | T | 0.83871 | A | 0.16129 | 10 | 0.32258 |
| 1289 | S2_6034289 | 2 | 6034289 | 46 | C | A | C | 0.6129 | A | 0.3871 | 16 | 0.51613 |
| 1290 | S2_6034407 | 2 | 6034407 | 118 | G | A | G | 0.8871 | A | 0.1129 | 7 | 0.22581 |
| 1291 | S2_6034431 | 2 | 6034431 | 24 | A | G | A | 0.62903 | G | 0.37097 | 17 | 0.54839 |
| 1292 | S2_6246800 | 2 | 6246800 | 212369 | C | G | C | 0.8871 | G | 0.1129 | 7 | 0.22581 |
| 1293 | S2_6246807 | 2 | 6246807 | 7 | T | C | C | 0.56452 | T | 0.43548 | 15 | 0.48387 |
| 1294 | S2_6392298 | 2 | 6392298 | 145491 | T | C | T | 0.8871 | C | 0.1129 | 7 | 0.22581 |

| Site number | SNP name | Chromosome | Physical position | Physical distance from the previous SNP | Reference allele | Alternative allele | Major allele | Major allele frequency | Minor allele | Minor allele frequency | Number heterozygous | Heterozygosity |
|---|---|---|---|---|---|---|---|---|---|---|---|---|
| 1295 | S2_6392491 | 2 | 6392491 | 193 | G | C | G | 0.72581 | C | 0.27419 | 15 | 0.48387 |
| 1296 | S2_6712177 | 2 | 6712177 | 319686 | G | C | G | 0.83871 | C | 0.16129 | 8 | 0.25806 |
| 1297 | S2_6712233 | 2 | 6712233 | 56 | T | A | T | 0.83871 | A | 0.16129 | 8 | 0.25806 |
| 1298 | S2_6712334 | 2 | 6712334 | 101 | G | A | G | 0.70968 | A | 0.29032 | 14 | 0.45161 |
| 1299 | S2_7052523 | 2 | 7052523 | 340189 | T | C | T | 0.91935 | C | 0.08065 | 5 | 0.16129 |
| 1300 | S2_7052565 | 2 | 7052565 | 42 | A | C | A | 0.91935 | C | 0.08065 | 5 | 0.16129 |
| 1301 | S2_7052618 | 2 | 7052618 | 53 | A | G | A | 0.5 | G | 0.5 | 17 | 0.54839 |
| 1302 | S2_7102017 | 2 | 7102017 | 49399 | A | G | A | 0.77419 | G | 0.22581 | 14 | 0.45161 |
| 1303 | S2_7102106 | 2 | 7102106 | 89 | T | C | C | 0.90323 | T | 0.09677 | 4 | 0.12903 |
| 1304 | S2_7486757 | 2 | 7486757 | 384651 | G | A | G | 0.77419 | A | 0.22581 | 14 | 0.45161 |
| 1305 | S2_7486799 | 2 | 7486799 | 42 | A | G | A | 0.8871 | G | 0.1129 | 7 | 0.22581 |
| 1306 | S2_7585896 | 2 | 7585896 | 99097 | A | G | A | 0.95161 | G | 0.04839 | 3 | 0.09677 |
| 1307 | S2_7586089 | 2 | 7586089 | 193 | T | C | T | 0.54839 | C | 0.45161 | 14 | 0.45161 |
| 1308 | S2_7640157 | 2 | 7640157 | 54068 | A | C | A | 0.75806 | C | 0.24194 | 15 | 0.48387 |
| 1309 | S2_7719352 | 2 | 7719352 | 79195 | C | T | T | 0.83871 | C | 0.16129 | 8 | 0.25806 |
| 1310 | S2_8239727 | 2 | 8239727 | 520375 | G | A | G | 0.91935 | A | 0.08065 | 3 | 0.09677 |
| 1311 | S2_8239756 | 2 | 8239756 | 29 | G | A | G | 0.8871 | A | 0.1129 | 7 | 0.22581 |
| 1312 | S2_8239887 | 2 | 8239887 | 131 | C | T | C | 0.8871 | T | 0.1129 | 7 | 0.22581 |
| 1313 | S2_8239902 | 2 | 8239902 | 15 | A | C | A | 0.77419 | C | 0.22581 | 14 | 0.45161 |
| 1314 | S2_8239911 | 2 | 8239911 | 9 | A | G | A | 0.62903 | G | 0.37097 | 13 | 0.41935 |
| 1315 | S2_8970857 | 2 | 8970857 | 730946 | G | A | G | 0.85484 | A | 0.14516 | 9 | 0.29032 |
| 1316 | S2_8995157 | 2 | 8995157 | 24300 | G | A | G | 0.91935 | A | 0.08065 | 5 | 0.16129 |
| 1317 | S2_8995326 | 2 | 8995326 | 169 | G | A | G | 0.79032 | A | 0.20968 | 13 | 0.41935 |
| 1318 | S2_9815164 | 2 | 9815164 | 819838 | C | T | C | 0.91935 | T | 0.08065 | 3 | 0.09677 |
| 1319 | S2_9917445 | 2 | 9917445 | 102281 | C | T | C | 0.95161 | T | 0.04839 | 3 | 0.09677 |
| 1320 | S2_10219355 | 2 | 10219355 | 301910 | T | C | C | 0.8871 | T | 0.1129 | 5 | 0.16129 |
| 1321 | S2_10219420 | 2 | 10219420 | 65 | T | C | C | 0.74194 | T | 0.25806 | 12 | 0.3871 |
| 1322 | S2_10927076 | 2 | 10927076 | 707656 | T | C | T | 0.80645 | C | 0.19355 | 6 | 0.19355 |
| 1323 | S2_10950777 | 2 | 10950777 | 23701 | T | A | T | 0.95161 | A | 0.04839 | 3 | 0.09677 |
| 1324 | S2_11305144 | 2 | 11305144 | 354367 | C | T | C | 0.93548 | T | 0.06452 | 4 | 0.12903 |
| 1325 | S2_11305145 | 2 | 11305145 | 1 | G | A | G | 0.8871 | A | 0.1129 | 7 | 0.22581 |
| 1326 | S2_11305161 | 2 | 11305161 | 16 | C | T | C | 0.62903 | T | 0.37097 | 17 | 0.54839 |
| 1327 | S2_11305207 | 2 | 11305207 | 46 | C | T | C | 0.72581 | T | 0.27419 | 13 | 0.41935 |
| 1328 | S2_11305208 | 2 | 11305208 | 1 | G | A | G | 0.66129 | A | 0.33871 | 17 | 0.54839 |
| 1329 | S2_11422602 | 2 | 11422602 | 117394 | T | C | T | 0.90323 | C | 0.09677 | 4 | 0.12903 |
| 1330 | S2_11422651 | 2 | 11422651 | 49 | C | A | C | 0.93548 | A | 0.06452 | 4 | 0.12903 |
| 1331 | S2_11618102 | 2 | 11618102 | 195451 | T | C | C | 0.51613 | T | 0.48387 | 18 | 0.58065 |
| 1332 | S2_11618209 | 2 | 11618209 | 107 | A | G | A | 0.87097 | G | 0.12903 | 8 | 0.25806 |
| 1333 | S2_11618232 | 2 | 11618232 | 23 | C | A | C | 0.87097 | A | 0.12903 | 8 | 0.25806 |
| 1334 | S2_11618278 | 2 | 11618278 | 46 | A | G | A | 0.87097 | G | 0.12903 | 8 | 0.25806 |
| 1335 | S2_11618283 | 2 | 11618283 | 5 | G | T | G | 0.82258 | T | 0.17742 | 7 | 0.22581 |
| 1336 | S2_11618303 | 2 | 11618303 | 20 | C | T | C | 0.56452 | T | 0.43548 | 15 | 0.48387 |
| 1337 | S2_12029147 | 2 | 12029147 | 410844 | C | T | C | 0.90323 | T | 0.09677 | 6 | 0.19355 |
| 1338 | S2_12094245 | 2 | 12094245 | 65098 | A | G | A | 0.8871 | G | 0.1129 | 7 | 0.22581 |
| 1339 | S2_12094266 | 2 | 12094266 | 21 | G | A | A | 0.83871 | G | 0.16129 | 6 | 0.19355 |
| 1340 | S2_12094422 | 2 | 12094422 | 156 | A | G | A | 0.83871 | G | 0.16129 | 6 | 0.19355 |
| 1341 | S2_12677702 | 2 | 12677702 | 583280 | C | T | C | 0.82258 | T | 0.17742 | 9 | 0.29032 |
| 1342 | S2_12677734 | 2 | 12677734 | 32 | C | T | C | 0.66129 | T | 0.33871 | 15 | 0.48387 |
| 1343 | S2_12691157 | 2 | 12691157 | 13423 | T | A | A | 0.58065 | T | 0.41935 | 18 | 0.58065 |
| 1344 | S2_12691264 | 2 | 12691264 | 107 | G | A | A | 0.64516 | G | 0.35484 | 16 | 0.51613 |
| 1345 | S2_12691335 | 2 | 12691335 | 71 | C | T | T | 0.58065 | C | 0.41935 | 18 | 0.58065 |
| 1346 | S2_12691377 | 2 | 12691377 | 42 | C | T | C | 0.93548 | T | 0.06452 | 4 | 0.12903 |
| 1347 | S2_12904648 | 2 | 12904648 | 213271 | G | T | G | 0.93548 | T | 0.06452 | 4 | 0.12903 |
| 1348 | S2_12904784 | 2 | 12904784 | 136 | A | G | A | 0.93548 | G | 0.06452 | 4 | 0.12903 |

| Site number | SNP name | Chromosome | Physical position | Physical distance from the previous SNP | Reference allele | Alternative allele | Major allele | Major allele frequency | Minor allele | Minor allele frequency | Number heterozygous | Heterozygosity |
|---|---|---|---|---|---|---|---|---|---|---|---|---|
| 1349 | S2_13199478 | 2 | 13199478 | 294694 | G | A | G | 0.59677 | A | 0.40323 | 15 | 0.48387 |
| 1350 | S2_13199490 | 2 | 13199490 | 12 | A | G | G | 0.82258 | A | 0.17742 | 11 | 0.35484 |
| 1351 | S2_13289460 | 2 | 13289460 | 89970 | T | C | C | 0.64516 | T | 0.35484 | 10 | 0.32258 |
| 1352 | S2_13572975 | 2 | 13572975 | 283515 | G | C | C | 0.70968 | G | 0.29032 | 12 | 0.3871 |
| 1353 | S2_13573004 | 2 | 13573004 | 29 | A | C | A | 0.72581 | C | 0.27419 | 11 | 0.35484 |
| 1354 | S2_13573029 | 2 | 13573029 | 25 | A | G | A | 0.72581 | G | 0.27419 | 11 | 0.35484 |
| 1355 | S2_13573067 | 2 | 13573067 | 38 | G | A | G | 0.72581 | A | 0.27419 | 11 | 0.35484 |
| 1356 | S2_13594944 | 2 | 13594944 | 21877 | G | T | G | 0.74194 | T | 0.25806 | 10 | 0.32258 |
| 1357 | S2_13887058 | 2 | 13887058 | 292114 | C | T | C | 0.95161 | T | 0.04839 | 3 | 0.09677 |
| 1358 | S2_13887059 | 2 | 13887059 | 1 | G | T | G | 0.91935 | T | 0.08065 | 3 | 0.09677 |
| 1359 | S2_13887063 | 2 | 13887063 | 4 | A | G | A | 0.59677 | G | 0.40323 | 21 | 0.67742 |
| 1360 | S2_13887092 | 2 | 13887092 | 29 | T | C | T | 0.51613 | C | 0.48387 | 20 | 0.64516 |
| 1361 | S2_13887202 | 2 | 13887202 | 110 | C | T | C | 0.69355 | T | 0.30645 | 17 | 0.54839 |
| 1362 | S2_13887241 | 2 | 13887241 | 39 | T | C | T | 0.70968 | C | 0.29032 | 16 | 0.51613 |
| 1363 | S2_14551237 | 2 | 14551237 | 663996 | A | G | A | 0.59677 | G | 0.40323 | 19 | 0.6129 |
| 1364 | S2_14645952 | 2 | 14645952 | 94715 | A | C | A | 0.95161 | C | 0.04839 | 3 | 0.09677 |
| 1365 | S2_14652701 | 2 | 14652701 | 6749 | T | G | T | 0.95161 | G | 0.04839 | 3 | 0.09677 |
| 1366 | S2_15414046 | 2 | 15414046 | 761345 | G | A | G | 0.83871 | A | 0.16129 | 8 | 0.25806 |
| 1367 | S2_15483303 | 2 | 15483303 | 69257 | C | T | C | 0.95161 | T | 0.04839 | 3 | 0.09677 |
| 1368 | S2_15690751 | 2 | 15690751 | 207448 | C | T | C | 0.90323 | T | 0.09677 | 4 | 0.12903 |
| 1369 | S2_15918351 | 2 | 15918351 | 227600 | C | A | C | 0.70968 | A | 0.29032 | 14 | 0.45161 |
| 1370 | S2_16263205 | 2 | 16263205 | 344854 | G | A | G | 0.90323 | A | 0.09677 | 6 | 0.19355 |
| 1371 | S2_16881708 | 2 | 16881708 | 618503 | G | A | G | 0.74194 | A | 0.25806 | 10 | 0.32258 |
| 1372 | S2_17008290 | 2 | 17008290 | 126582 | G | A | G | 0.95161 | A | 0.04839 | 3 | 0.09677 |
| 1373 | S2_17717676 | 2 | 17717676 | 709386 | T | C | T | 0.83871 | C | 0.16129 | 8 | 0.25806 |
| 1374 | S2_17717684 | 2 | 17717684 | 8 | G | C | G | 0.95161 | C | 0.04839 | 3 | 0.09677 |
| 1375 | S2_18186934 | 2 | 18186934 | 469250 | A | G | G | 0.87097 | A | 0.12903 | 8 | 0.25806 |
| 1376 | S2_18186939 | 2 | 18186939 | 5 | A | G | A | 0.90323 | G | 0.09677 | 6 | 0.19355 |
| 1377 | S2_18186957 | 2 | 18186957 | 18 | C | T | C | 0.90323 | T | 0.09677 | 6 | 0.19355 |
| 1378 | S2_18186967 | 2 | 18186967 | 10 | T | C | T | 0.90323 | C | 0.09677 | 6 | 0.19355 |
| 1379 | S2_18186975 | 2 | 18186975 | 8 | C | T | T | 0.87097 | C | 0.12903 | 8 | 0.25806 |
| 1380 | S2_18186986 | 2 | 18186986 | 11 | T | C | T | 0.90323 | C | 0.09677 | 6 | 0.19355 |
| 1381 | S2_18187003 | 2 | 18187003 | 17 | A | G | A | 0.90323 | G | 0.09677 | 6 | 0.19355 |
| 1382 | S2_18187017 | 2 | 18187017 | 14 | G | C | G | 0.90323 | C | 0.09677 | 6 | 0.19355 |
| 1383 | S2_18278094 | 2 | 18278094 | 91077 | C | T | C | 0.69355 | T | 0.30645 | 13 | 0.41935 |
| 1384 | S2_18278169 | 2 | 18278169 | 75 | G | A | A | 0.54839 | G | 0.45161 | 18 | 0.58065 |
| 1385 | S2_18278245 | 2 | 18278245 | 76 | T | C | C | 0.54839 | T | 0.45161 | 18 | 0.58065 |
| 1386 | S2_18278264 | 2 | 18278264 | 19 | T | C | C | 0.54839 | T | 0.45161 | 18 | 0.58065 |
| 1387 | S2_18332453 | 2 | 18332453 | 54189 | C | T | C | 0.83871 | T | 0.16129 | 8 | 0.25806 |
| 1388 | S2_18332498 | 2 | 18332498 | 45 | A | C | A | 0.90323 | C | 0.09677 | 4 | 0.12903 |
| 1389 | S2_18466584 | 2 | 18466584 | 134086 | G | T | G | 0.90323 | T | 0.09677 | 6 | 0.19355 |
| 1390 | S2_18466654 | 2 | 18466654 | 70 | A | G | A | 0.83871 | G | 0.16129 | 8 | 0.25806 |
| 1391 | S2_18485167 | 2 | 18485167 | 18513 | A | T | A | 0.83871 | T | 0.16129 | 8 | 0.25806 |
| 1392 | S2_18723801 | 2 | 18723801 | 238634 | T | C | C | 0.83871 | T | 0.16129 | 8 | 0.25806 |
| 1393 | S2_18734144 | 2 | 18734144 | 10343 | G | A | G | 0.8871 | A | 0.1129 | 5 | 0.16129 |
| 1394 | S2_18734272 | 2 | 18734272 | 128 | A | G | A | 0.93548 | G | 0.06452 | 2 | 0.06452 |
| 1395 | S2_18734331 | 2 | 18734331 | 59 | A | G | A | 0.82258 | G | 0.17742 | 7 | 0.22581 |
| 1396 | S2_18769910 | 2 | 18769910 | 35579 | G | T | G | 0.87097 | T | 0.12903 | 8 | 0.25806 |
| 1397 | S2_18769976 | 2 | 18769976 | 66 | T | C | C | 0.91935 | T | 0.08065 | 5 | 0.16129 |
| 1398 | S2_18778245 | 2 | 18778245 | 8269 | A | T | A | 0.91935 | T | 0.08065 | 5 | 0.16129 |
| 1399 | S2_18778297 | 2 | 18778297 | 52 | A | G | A | 0.91935 | G | 0.08065 | 5 | 0.16129 |
| 1400 | S2_18778328 | 2 | 18778328 | 31 | A | G | G | 0.93548 | A | 0.06452 | 4 | 0.12903 |
| 1401 | S2_18778372 | 2 | 18778372 | 44 | G | T | G | 0.90323 | T | 0.09677 | 6 | 0.19355 |
| 1402 | S2_18822630 | 2 | 18822630 | 44258 | G | A | G | 0.82258 | A | 0.17742 | 11 | 0.35484 |

| Site number | SNP name | Chromosome | Physical position | Physical distance from the previous SNP | Reference allele | Alternative allele | Major allele | Major allele frequency | Minor allele | Minor allele frequency | Number heterozygous | Heterozygosity |
|---|---|---|---|---|---|---|---|---|---|---|---|---|
| 1403 | S2_18822647 | 2 | 18822647 | 17 | G | A | G | 0.87097 | A | 0.12903 | 6 | 0.19355 |
| 1404 | S2_18904298 | 2 | 18904298 | 81651 | T | C | T | 0.91935 | C | 0.08065 | 5 | 0.16129 |
| 1405 | S2_18904354 | 2 | 18904354 | 56 | A | G | A | 0.91935 | G | 0.08065 | 5 | 0.16129 |
| 1406 | S2_18904461 | 2 | 18904461 | 107 | C | G | C | 0.51613 | G | 0.48387 | 16 | 0.51613 |
| 1407 | S2_18904503 | 2 | 18904503 | 42 | T | C | C | 0.6129 | T | 0.3871 | 20 | 0.64516 |
| 1408 | S2_18904517 | 2 | 18904517 | 14 | A | G | A | 0.95161 | G | 0.04839 | 3 | 0.09677 |
| 1409 | S2_19093456 | 2 | 19093456 | 188939 | C | T | C | 0.62903 | T | 0.37097 | 17 | 0.54839 |
| 1410 | S2_19826647 | 2 | 19826647 | 733191 | G | A | G | 0.75806 | A | 0.24194 | 15 | 0.48387 |
| 1411 | S2_19826655 | 2 | 19826655 | 8 | G | A | G | 0.74194 | A | 0.25806 | 16 | 0.51613 |
| 1412 | S2_19826711 | 2 | 19826711 | 56 | G | T | G | 0.74194 | T | 0.25806 | 16 | 0.51613 |
| 1413 | S2_19826717 | 2 | 19826717 | 6 | A | G | G | 0.70968 | A | 0.29032 | 18 | 0.58065 |
| 1414 | S2_20329601 | 2 | 20329601 | 502884 | A | G | A | 0.83871 | G | 0.16129 | 6 | 0.19355 |
| 1415 | S2_20622167 | 2 | 20622167 | 292566 | A | C | A | 0.54839 | C | 0.45161 | 14 | 0.45161 |
| 1416 | S2_20622382 | 2 | 20622382 | 215 | C | T | C | 0.8871 | T | 0.1129 | 7 | 0.22581 |
| 1417 | S2_20622393 | 2 | 20622393 | 11 | A | G | A | 0.54839 | G | 0.45161 | 14 | 0.45161 |
| 1418 | S2_20643544 | 2 | 20643544 | 21151 | C | A | C | 0.95161 | A | 0.04839 | 3 | 0.09677 |
| 1419 | S2_20643549 | 2 | 20643549 | 5 | C | T | C | 0.5 | T | 0.5 | 13 | 0.41935 |
| 1420 | S2_21432950 | 2 | 21432950 | 789401 | G | A | A | 0.80645 | G | 0.19355 | 10 | 0.32258 |
| 1421 | S2_21432962 | 2 | 21432962 | 12 | G | A | G | 0.93548 | A | 0.06452 | 4 | 0.12903 |
| 1422 | S2_21433012 | 2 | 21433012 | 50 | T | A | T | 0.93548 | A | 0.06452 | 4 | 0.12903 |
| 1423 | S2_21484021 | 2 | 21484021 | 51009 | C | T | T | 0.80645 | C | 0.19355 | 10 | 0.32258 |
| 1424 | S2_21484215 | 2 | 21484215 | 194 | C | T | C | 0.93548 | T | 0.06452 | 4 | 0.12903 |
| 1425 | S2_21484220 | 2 | 21484220 | 5 | G | A | G | 0.80645 | A | 0.19355 | 12 | 0.3871 |
| 1426 | S2_21785944 | 2 | 21785944 | 301724 | A | G | G | 0.80645 | A | 0.19355 | 8 | 0.25806 |
| 1427 | S2_22039707 | 2 | 22039707 | 253763 | C | T | C | 0.83871 | T | 0.16129 | 10 | 0.32258 |
| 1428 | S2_22039760 | 2 | 22039760 | 53 | A | C | C | 0.85484 | A | 0.14516 | 7 | 0.22581 |
| 1429 | S2_22522401 | 2 | 22522401 | 482641 | A | G | A | 0.93548 | G | 0.06452 | 4 | 0.12903 |
| 1430 | S2_22522545 | 2 | 22522545 | 144 | C | T | C | 0.8871 | T | 0.1129 | 5 | 0.16129 |
| 1431 | S2_22764195 | 2 | 22764195 | 241650 | A | G | A | 0.8871 | G | 0.1129 | 7 | 0.22581 |
| 1432 | S2_22764321 | 2 | 22764321 | 126 | T | C | T | 0.8871 | C | 0.1129 | 7 | 0.22581 |
| 1433 | S2_22821573 | 2 | 22821573 | 57252 | A | C | A | 0.66129 | C | 0.33871 | 13 | 0.41935 |
| 1434 | S2_23039561 | 2 | 23039561 | 217988 | C | T | C | 0.90323 | T | 0.09677 | 6 | 0.19355 |
| 1435 | S2_23490175 | 2 | 23490175 | 450614 | G | C | C | 0.85484 | G | 0.14516 | 7 | 0.22581 |
| 1436 | S2_23554358 | 2 | 23554358 | 64183 | C | A | C | 0.8871 | A | 0.1129 | 7 | 0.22581 |
| 1437 | S2_23623986 | 2 | 23623986 | 69628 | A | G | G | 0.91935 | A | 0.08065 | 5 | 0.16129 |
| 1438 | S2_23624014 | 2 | 23624014 | 28 | A | G | A | 0.85484 | G | 0.14516 | 9 | 0.29032 |
| 1439 | S2_23728370 | 2 | 23728370 | 104356 | C | T | C | 0.85484 | T | 0.14516 | 9 | 0.29032 |
| 1440 | S2_23728433 | 2 | 23728433 | 63 | G | T | G | 0.67742 | T | 0.32258 | 12 | 0.3871 |
| 1441 | S2_23728467 | 2 | 23728467 | 34 | A | G | A | 0.85484 | G | 0.14516 | 9 | 0.29032 |
| 1442 | S2_23881308 | 2 | 23881308 | 152841 | C | T | T | 0.82258 | C | 0.17742 | 9 | 0.29032 |
| 1443 | S2_23881371 | 2 | 23881371 | 63 | A | C | A | 0.91935 | C | 0.08065 | 5 | 0.16129 |
| 1444 | S2_23881476 | 2 | 23881476 | 105 | G | A | G | 0.8871 | A | 0.1129 | 5 | 0.16129 |
| 1445 | S2_23974797 | 2 | 23974797 | 93321 | G | T | G | 0.80645 | T | 0.19355 | 10 | 0.32258 |
| 1446 | S2_23974817 | 2 | 23974817 | 20 | C | T | C | 0.79032 | T | 0.20968 | 11 | 0.35484 |
| 1447 | S2_23974841 | 2 | 23974841 | 24 | G | A | A | 0.75806 | G | 0.24194 | 11 | 0.35484 |
| 1448 | S2_23974888 | 2 | 23974888 | 47 | C | T | C | 0.90323 | T | 0.09677 | 6 | 0.19355 |
| 1449 | S2_23974896 | 2 | 23974896 | 8 | C | T | C | 0.79032 | T | 0.20968 | 11 | 0.35484 |
| 1450 | S2_23974958 | 2 | 23974958 | 62 | T | C | T | 0.90323 | C | 0.09677 | 6 | 0.19355 |
| 1451 | S2_23974966 | 2 | 23974966 | 8 | G | A | G | 0.91935 | A | 0.08065 | 5 | 0.16129 |
| 1452 | S2_23974988 | 2 | 23974988 | 22 | C | T | T | 0.59677 | C | 0.40323 | 15 | 0.48387 |
| 1453 | S2_23974989 | 2 | 23974989 | 1 | A | G | G | 0.59677 | A | 0.40323 | 15 | 0.48387 |
| 1454 | S2_23974998 | 2 | 23974998 | 9 | C | T | T | 0.59677 | C | 0.40323 | 15 | 0.48387 |
| 1455 | S2_24067772 | 2 | 24067772 | 92774 | G | A | G | 0.53226 | A | 0.46774 | 19 | 0.6129 |
| 1456 | S2_24067799 | 2 | 24067799 | 27 | C | T | T | 0.79032 | C | 0.20968 | 13 | 0.41935 |

| Site number | SNP name | Chromosome | Physical position | Physical distance from the previous SNP | Reference allele | Alternative allele | Major allele | Major allele frequency | Minor allele | Minor allele frequency | Number heterozygous | Heterozygosity |
|---|---|---|---|---|---|---|---|---|---|---|---|---|
| 1457 | S2_24067840 | 2 | 24067840 | 41 | T | C | T | 0.83871 | C | 0.16129 | 10 | 0.32258 |
| 1458 | S2_24067849 | 2 | 24067849 | 9 | C | T | C | 0.6129 | T | 0.3871 | 16 | 0.51613 |
| 1459 | S2_24067918 | 2 | 24067918 | 69 | G | A | G | 0.70968 | A | 0.29032 | 12 | 0.3871 |
| 1460 | S2_24644077 | 2 | 24644077 | 576159 | A | G | A | 0.91935 | G | 0.08065 | 3 | 0.09677 |
| 1461 | S2_24644176 | 2 | 24644176 | 99 | A | G | A | 0.51613 | G | 0.48387 | 12 | 0.3871 |
| 1462 | S2_24644196 | 2 | 24644196 | 20 | C | T | C | 0.93548 | T | 0.06452 | 4 | 0.12903 |
| 1463 | S2_24909773 | 2 | 24909773 | 265577 | A | G | G | 0.74194 | A | 0.25806 | 14 | 0.45161 |
| 1464 | S2_24909842 | 2 | 24909842 | 69 | G | A | G | 0.8871 | A | 0.1129 | 7 | 0.22581 |
| 1465 | S2_25057850 | 2 | 25057850 | 148008 | G | A | G | 0.93548 | A | 0.06452 | 2 | 0.06452 |
| 1466 | S2_25058065 | 2 | 25058065 | 215 | A | C | A | 0.91935 | C | 0.08065 | 3 | 0.09677 |
| 1467 | S2_26463247 | 2 | 26463247 | 1405182 | T | C | C | 0.80645 | T | 0.19355 | 12 | 0.3871 |
| 1468 | S2_26463397 | 2 | 26463397 | 150 | C | T | C | 0.95161 | T | 0.04839 | 1 | 0.03226 |
| 1469 | S2_26781872 | 2 | 26781872 | 318475 | G | A | G | 0.93548 | A | 0.06452 | 4 | 0.12903 |
| 1470 | S2_26792764 | 2 | 26792764 | 10892 | G | A | G | 0.93548 | A | 0.06452 | 4 | 0.12903 |
| 1471 | S2_26792805 | 2 | 26792805 | 41 | T | C | T | 0.93548 | C | 0.06452 | 4 | 0.12903 |
| 1472 | S2_26792933 | 2 | 26792933 | 128 | G | A | G | 0.93548 | A | 0.06452 | 4 | 0.12903 |
| 1473 | S2_26792947 | 2 | 26792947 | 14 | G | A | G | 0.87097 | A | 0.12903 | 8 | 0.25806 |
| 1474 | S2_27220506 | 2 | 27220506 | 427559 | A | G | A | 0.64516 | G | 0.35484 | 16 | 0.51613 |
| 1475 | S2_27220533 | 2 | 27220533 | 27 | G | C | G | 0.79032 | C | 0.20968 | 11 | 0.35484 |
| 1476 | S2_27220612 | 2 | 27220612 | 79 | G | A | G | 0.87097 | A | 0.12903 | 8 | 0.25806 |
| 1477 | S2_27220613 | 2 | 27220613 | 1 | A | G | A | 0.87097 | G | 0.12903 | 8 | 0.25806 |
| 1478 | S2_27761070 | 2 | 27761070 | 540457 | T | C | T | 0.90323 | C | 0.09677 | 6 | 0.19355 |
| 1479 | S2_27761303 | 2 | 27761303 | 233 | G | A | G | 0.93548 | A | 0.06452 | 2 | 0.06452 |
| 1480 | S2_28001978 | 2 | 28001978 | 240675 | C | T | C | 0.80645 | T | 0.19355 | 10 | 0.32258 |
| 1481 | S2_28002110 | 2 | 28002110 | 132 | C | T | C | 0.80645 | T | 0.19355 | 10 | 0.32258 |
| 1482 | S2_28068960 | 2 | 28068960 | 66850 | G | A | G | 0.93548 | A | 0.06452 | 4 | 0.12903 |
| 1483 | S2_28068970 | 2 | 28068970 | 10 | C | T | C | 0.87097 | T | 0.12903 | 6 | 0.19355 |
| 1484 | S2_28069001 | 2 | 28069001 | 31 | G | A | G | 0.95161 | A | 0.04839 | 3 | 0.09677 |
| 1485 | S2_28069013 | 2 | 28069013 | 12 | T | C | C | 0.90323 | T | 0.09677 | 4 | 0.12903 |
| 1486 | S2_28069039 | 2 | 28069039 | 26 | C | T | C | 0.95161 | T | 0.04839 | 3 | 0.09677 |
| 1487 | S2_28247359 | 2 | 28247359 | 178320 | G | A | G | 0.51613 | A | 0.48387 | 18 | 0.58065 |
| 1488 | S2_28247399 | 2 | 28247399 | 40 | G | A | G | 0.56452 | A | 0.43548 | 17 | 0.54839 |
| 1489 | S2_28535788 | 2 | 28535788 | 288389 | T | C | C | 0.95161 | T | 0.04839 | 3 | 0.09677 |
| 1490 | S2_28535877 | 2 | 28535877 | 89 | C | T | C | 0.8871 | T | 0.1129 | 5 | 0.16129 |
| 1491 | S2_28536277 | 2 | 28536277 | 400 | G | A | G | 0.93548 | A | 0.06452 | 4 | 0.12903 |
| 1492 | S2_28536307 | 2 | 28536307 | 30 | G | A | G | 0.93548 | A | 0.06452 | 2 | 0.06452 |
| 1493 | S2_28536376 | 2 | 28536376 | 69 | T | C | C | 0.93548 | T | 0.06452 | 4 | 0.12903 |
| 1494 | S2_28737820 | 2 | 28737820 | 201444 | C | T | C | 0.91935 | T | 0.08065 | 5 | 0.16129 |
| 1495 | S2_29401288 | 2 | 29401288 | 663468 | A | G | A | 0.93548 | G | 0.06452 | 4 | 0.12903 |
| 1496 | S2_29654129 | 2 | 29654129 | 252841 | A | G | A | 0.91935 | G | 0.08065 | 5 | 0.16129 |
| 1497 | S2_29654325 | 2 | 29654325 | 196 | G | A | G | 0.77419 | A | 0.22581 | 8 | 0.25806 |
| 1498 | S2_29654352 | 2 | 29654352 | 27 | C | G | C | 0.79032 | G | 0.20968 | 11 | 0.35484 |
| 1499 | S2_29727215 | 2 | 29727215 | 72863 | C | T | C | 0.8871 | T | 0.1129 | 7 | 0.22581 |
| 1500 | S2_29727262 | 2 | 29727262 | 47 | G | T | G | 0.8871 | T | 0.1129 | 7 | 0.22581 |
| 1501 | S2_30008434 | 2 | 30008434 | 281172 | C | T | C | 0.79032 | T | 0.20968 | 13 | 0.41935 |
| 1502 | S2_30229539 | 2 | 30229539 | 221105 | T | C | T | 0.85484 | C | 0.14516 | 7 | 0.22581 |
| 1503 | S2_30229540 | 2 | 30229540 | 1 | G | T | G | 0.85484 | T | 0.14516 | 7 | 0.22581 |
| 1504 | S2_30229640 | 2 | 30229640 | 100 | T | C | T | 0.87097 | C | 0.12903 | 6 | 0.19355 |
| 1505 | S2_30289092 | 2 | 30289092 | 59452 | T | C | T | 0.80645 | C | 0.19355 | 8 | 0.25806 |
| 1506 | S2_30289117 | 2 | 30289117 | 25 | T | A | A | 0.75806 | T | 0.24194 | 9 | 0.29032 |
| 1507 | S2_30289124 | 2 | 30289124 | 7 | A | G | A | 0.90323 | G | 0.09677 | 4 | 0.12903 |
| 1508 | S2_30289171 | 2 | 30289171 | 47 | C | T | C | 0.93548 | T | 0.06452 | 4 | 0.12903 |
| 1509 | S2_30289221 | 2 | 30289221 | 50 | A | G | A | 0.77419 | G | 0.22581 | 14 | 0.45161 |
| 1510 | S2_30289228 | 2 | 30289228 | 7 | C | T | C | 0.95161 | T | 0.04839 | 3 | 0.09677 |

| Site number | SNP name | Chromosome | Physical position | Physical distance from the previous SNP | Reference allele | Alternative allele | Major allele | Major allele frequency | Minor allele | Minor allele frequency | Number heterozygous | Heterozygosity |
|---|---|---|---|---|---|---|---|---|---|---|---|---|
| 1511 | S2_30289229 | 2 | 30289229 | 1 | T | G | T | 0.75806 | G | 0.24194 | 9 | 0.29032 |
| 1512 | S2_30687989 | 2 | 30687989 | 398760 | C | T | C | 0.93548 | T | 0.06452 | 4 | 0.12903 |
| 1513 | S2_30799444 | 2 | 30799444 | 111455 | C | T | C | 0.6129 | T | 0.3871 | 14 | 0.45161 |
| 1514 | S2_30799621 | 2 | 30799621 | 177 | T | C | T | 0.69355 | C | 0.30645 | 13 | 0.41935 |
| 1515 | S2_31046029 | 2 | 31046029 | 246408 | G | A | G | 0.80645 | A | 0.19355 | 10 | 0.32258 |
| 1516 | S2_31046142 | 2 | 31046142 | 113 | A | C | C | 0.83871 | A | 0.16129 | 10 | 0.32258 |
| 1517 | S2_31143689 | 2 | 31143689 | 97547 | C | T | C | 0.95161 | T | 0.04839 | 3 | 0.09677 |
| 1518 | S2_31143708 | 2 | 31143708 | 19 | C | T | C | 0.85484 | T | 0.14516 | 9 | 0.29032 |
| 1519 | S2_31415113 | 2 | 31415113 | 271405 | C | T | C | 0.95161 | T | 0.04839 | 3 | 0.09677 |
| 1520 | S2_31415152 | 2 | 31415152 | 39 | C | A | C | 0.91935 | A | 0.08065 | 5 | 0.16129 |
| 1521 | S2_31455443 | 2 | 31455443 | 40291 | G | A | G | 0.67742 | A | 0.32258 | 16 | 0.51613 |
| 1522 | S2_31455568 | 2 | 31455568 | 125 | C | T | C | 0.93548 | T | 0.06452 | 4 | 0.12903 |
| 1523 | S2_31455579 | 2 | 31455579 | 11 | G | A | G | 0.82258 | A | 0.17742 | 9 | 0.29032 |
| 1524 | S2_31530021 | 2 | 31530021 | 74442 | C | T | C | 0.87097 | T | 0.12903 | 6 | 0.19355 |
| 1525 | S2_31530052 | 2 | 31530052 | 31 | T | C | T | 0.80645 | C | 0.19355 | 10 | 0.32258 |
| 1526 | S2_31530114 | 2 | 31530114 | 62 | C | T | C | 0.80645 | T | 0.19355 | 10 | 0.32258 |
| 1527 | S2_31560928 | 2 | 31560928 | 30814 | G | A | G | 0.80645 | A | 0.19355 | 12 | 0.3871 |
| 1528 | S2_31561069 | 2 | 31561069 | 141 | G | A | G | 0.80645 | A | 0.19355 | 12 | 0.3871 |
| 1529 | S2_31561070 | 2 | 31561070 | 1 | T | C | T | 0.90323 | C | 0.09677 | 6 | 0.19355 |
| 1530 | S2_31747753 | 2 | 31747753 | 186683 | G | A | G | 0.66129 | A | 0.33871 | 17 | 0.54839 |
| 1531 | S2_31929051 | 2 | 31929051 | 181298 | T | C | T | 0.85484 | C | 0.14516 | 9 | 0.29032 |
| 1532 | S2_32473317 | 2 | 32473317 | 544266 | A | G | A | 0.87097 | G | 0.12903 | 8 | 0.25806 |
| 1533 | S2_32473508 | 2 | 32473508 | 191 | T | C | T | 0.82258 | C | 0.17742 | 11 | 0.35484 |
| 1534 | S2_33001783 | 2 | 33001783 | 528275 | G | C | G | 0.8871 | C | 0.1129 | 7 | 0.22581 |
| 1535 | S2_33001938 | 2 | 33001938 | 155 | C | T | C | 0.91935 | T | 0.08065 | 5 | 0.16129 |
| 1536 | S2_33001969 | 2 | 33001969 | 31 | C | T | C | 0.8871 | T | 0.1129 | 7 | 0.22581 |
| 1537 | S2_33079623 | 2 | 33079623 | 77654 | C | T | C | 0.87097 | T | 0.12903 | 6 | 0.19355 |
| 1538 | S2_33079663 | 2 | 33079663 | 40 | A | G | A | 0.93548 | G | 0.06452 | 4 | 0.12903 |
| 1539 | S2_33079778 | 2 | 33079778 | 115 | G | A | A | 0.66129 | G | 0.33871 | 13 | 0.41935 |
| 1540 | S2_33079791 | 2 | 33079791 | 13 | G | A | A | 0.69355 | G | 0.30645 | 11 | 0.35484 |
| 1541 | S2_33079806 | 2 | 33079806 | 15 | C | T | C | 0.93548 | T | 0.06452 | 4 | 0.12903 |
| 1542 | S2_33079832 | 2 | 33079832 | 26 | C | T | T | 0.6129 | C | 0.3871 | 16 | 0.51613 |
| 1543 | S2_34005523 | 2 | 34005523 | 925691 | A | G | A | 0.70968 | G | 0.29032 | 14 | 0.45161 |
| 1544 | S2_34005552 | 2 | 34005552 | 29 | C | G | C | 0.95161 | G | 0.04839 | 3 | 0.09677 |
| 1545 | S2_34005632 | 2 | 34005632 | 80 | C | T | C | 0.90323 | T | 0.09677 | 6 | 0.19355 |
| 1546 | S2_34214271 | 2 | 34214271 | 208639 | C | G | C | 0.62903 | G | 0.37097 | 17 | 0.54839 |
| 1547 | S2_34214397 | 2 | 34214397 | 126 | T | C | T | 0.62903 | C | 0.37097 | 17 | 0.54839 |
| 1548 | S2_34214463 | 2 | 34214463 | 66 | T | C | T | 0.83871 | C | 0.16129 | 10 | 0.32258 |
| 1549 | S2_34752150 | 2 | 34752150 | 537687 | G | A | G | 0.8871 | A | 0.1129 | 7 | 0.22581 |
| 1550 | S2_34752152 | 2 | 34752152 | 2 | G | T | G | 0.70968 | T | 0.29032 | 14 | 0.45161 |
| 1551 | S2_34772040 | 2 | 34772040 | 19888 | G | A | G | 0.85484 | A | 0.14516 | 9 | 0.29032 |
| 1552 | S2_34951968 | 2 | 34951968 | 179928 | G | A | G | 0.87097 | A | 0.12903 | 8 | 0.25806 |
| 1553 | S2_35071519 | 2 | 35071519 | 119551 | G | A | G | 0.87097 | A | 0.12903 | 8 | 0.25806 |
| 1554 | S2_35207965 | 2 | 35207965 | 136446 | G | T | T | 0.80645 | G | 0.19355 | 10 | 0.32258 |
| 1555 | S2_35293297 | 2 | 35293297 | 85332 | G | A | G | 0.95161 | A | 0.04839 | 3 | 0.09677 |
| 1556 | S2_35293410 | 2 | 35293410 | 113 | C | T | C | 0.8871 | T | 0.1129 | 5 | 0.16129 |
| 1557 | S2_36092613 | 2 | 36092613 | 799203 | T | C | C | 0.64516 | T | 0.35484 | 12 | 0.3871 |
| 1558 | S2_36214831 | 2 | 36214831 | 122218 | C | T | C | 0.91935 | T | 0.08065 | 5 | 0.16129 |
| 1559 | S2_36214873 | 2 | 36214873 | 42 | C | A | A | 0.53226 | C | 0.46774 | 15 | 0.48387 |
| 1560 | S2_36214874 | 2 | 36214874 | 1 | G | C | C | 0.53226 | G | 0.46774 | 15 | 0.48387 |
| 1561 | S2_36214889 | 2 | 36214889 | 15 | T | C | T | 0.93548 | C | 0.06452 | 4 | 0.12903 |
| 1562 | S2_36214930 | 2 | 36214930 | 41 | C | G | C | 0.93548 | G | 0.06452 | 4 | 0.12903 |
| 1563 | S2_36214950 | 2 | 36214950 | 20 | C | A | C | 0.93548 | A | 0.06452 | 4 | 0.12903 |
| 1564 | S2_36250481 | 2 | 36250481 | 35531 | A | G | A | 0.83871 | G | 0.16129 | 6 | 0.19355 |

| Site number | SNP name | Chromosome | Physical position | Physical distance from the previous SNP | Reference allele | Alternative allele | Major allele | Major allele frequency | Minor allele | Minor allele frequency | Number heterozygous | Heterozygosity |
|---|---|---|---|---|---|---|---|---|---|---|---|---|
| 1565 | S2_36672654 | 2 | 36672654 | 422173 | C | T | T | 0.80645 | C | 0.19355 | 10 | 0.32258 |
| 1566 | S2_36672775 | 2 | 36672775 | 121 | C | T | C | 0.69355 | T | 0.30645 | 13 | 0.41935 |
| 1567 | S2_36813908 | 2 | 36813908 | 141133 | C | T | C | 0.91935 | T | 0.08065 | 5 | 0.16129 |
| 1568 | S2_37235167 | 2 | 37235167 | 421259 | G | T | T | 0.54839 | G | 0.45161 | 14 | 0.45161 |
| 1569 | S2_37235200 | 2 | 37235200 | 33 | T | C | T | 0.83871 | C | 0.16129 | 10 | 0.32258 |
| 1570 | S2_37235251 | 2 | 37235251 | 51 | T | C | C | 0.70968 | T | 0.29032 | 12 | 0.3871 |
| 1571 | S2_37235260 | 2 | 37235260 | 9 | A | T | A | 0.80645 | T | 0.19355 | 10 | 0.32258 |
| 1572 | S2_37235364 | 2 | 37235364 | 104 | C | A | C | 0.8871 | A | 0.1129 | 7 | 0.22581 |
| 1573 | S2_37235400 | 2 | 37235400 | 36 | C | T | T | 0.64516 | C | 0.35484 | 12 | 0.3871 |
| 1574 | S2_37235405 | 2 | 37235405 | 5 | T | G | T | 0.8871 | G | 0.1129 | 7 | 0.22581 |
| 1575 | S2_38047673 | 2 | 38047673 | 812268 | T | G | G | 0.6129 | T | 0.3871 | 16 | 0.51613 |
| 1576 | S2_38513499 | 2 | 38513499 | 465826 | G | A | G | 0.91935 | A | 0.08065 | 5 | 0.16129 |
| 1577 | S2_38784404 | 2 | 38784404 | 270905 | T | C | T | 0.58065 | C | 0.41935 | 20 | 0.64516 |
| 1578 | S2_38784516 | 2 | 38784516 | 112 | G | A | G | 0.62903 | A | 0.37097 | 19 | 0.6129 |
| 1579 | S2_39090188 | 2 | 39090188 | 305672 | C | T | C | 0.51613 | T | 0.48387 | 22 | 0.70968 |
| 1580 | S2_39382635 | 2 | 39382635 | 292447 | A | G | A | 0.95161 | G | 0.04839 | 3 | 0.09677 |
| 1581 | S2_39458821 | 2 | 39458821 | 76186 | G | A | G | 0.93548 | A | 0.06452 | 4 | 0.12903 |
| 1582 | S2_39458844 | 2 | 39458844 | 23 | G | A | G | 0.80645 | A | 0.19355 | 12 | 0.3871 |
| 1583 | S2_39458851 | 2 | 39458851 | 7 | G | A | G | 0.80645 | A | 0.19355 | 12 | 0.3871 |
| 1584 | S2_39645281 | 2 | 39645281 | 186430 | G | A | G | 0.90323 | A | 0.09677 | 6 | 0.19355 |
| 1585 | S2_39831277 | 2 | 39831277 | 185996 | G | A | G | 0.95161 | A | 0.04839 | 3 | 0.09677 |
| 1586 | S2_40910533 | 2 | 40910533 | 1079256 | C | T | C | 0.93548 | T | 0.06452 | 4 | 0.12903 |
| 1587 | S2_41107202 | 2 | 41107202 | 196669 | G | A | G | 0.85484 | A | 0.14516 | 7 | 0.22581 |
| 1588 | S2_41107322 | 2 | 41107322 | 120 | G | A | G | 0.85484 | A | 0.14516 | 7 | 0.22581 |
| 1589 | S2_41727328 | 2 | 41727328 | 620006 | G | A | G | 0.93548 | A | 0.06452 | 4 | 0.12903 |
| 1590 | S2_41776208 | 2 | 41776208 | 48880 | G | A | G | 0.90323 | A | 0.09677 | 6 | 0.19355 |
| 1591 | S2_41776251 | 2 | 41776251 | 43 | C | T | C | 0.85484 | T | 0.14516 | 7 | 0.22581 |
| 1592 | S2_42181683 | 2 | 42181683 | 405432 | A | G | A | 0.87097 | G | 0.12903 | 6 | 0.19355 |
| 1593 | S2_42181752 | 2 | 42181752 | 69 | T | C | T | 0.83871 | C | 0.16129 | 6 | 0.19355 |
| 1594 | S2_42181759 | 2 | 42181759 | 7 | A | G | A | 0.91935 | G | 0.08065 | 3 | 0.09677 |
| 1595 | S2_42181832 | 2 | 42181832 | 73 | G | C | G | 0.8871 | C | 0.1129 | 5 | 0.16129 |
| 1596 | S2_42181852 | 2 | 42181852 | 20 | C | T | C | 0.95161 | T | 0.04839 | 3 | 0.09677 |
| 1597 | S2_42434791 | 2 | 42434791 | 252939 | C | T | C | 0.56452 | T | 0.43548 | 17 | 0.54839 |
| 1598 | S2_42434972 | 2 | 42434972 | 181 | C | T | C | 0.56452 | T | 0.43548 | 17 | 0.54839 |
| 1599 | S2_42824714 | 2 | 42824714 | 389742 | A | G | G | 0.67742 | A | 0.32258 | 12 | 0.3871 |
| 1600 | S2_42824898 | 2 | 42824898 | 184 | T | C | C | 0.77419 | T | 0.22581 | 10 | 0.32258 |
| 1601 | S2_43071389 | 2 | 43071389 | 246491 | G | A | G | 0.80645 | A | 0.19355 | 8 | 0.25806 |
| 1602 | S2_43071393 | 2 | 43071393 | 4 | T | C | T | 0.80645 | C | 0.19355 | 8 | 0.25806 |
| 1603 | S2_43346120 | 2 | 43346120 | 274727 | C | T | C | 0.95161 | T | 0.04839 | 3 | 0.09677 |
| 1604 | S2_44710915 | 2 | 44710915 | 1364795 | T | G | T | 0.95161 | G | 0.04839 | 3 | 0.09677 |
| 1605 | S2_44831872 | 2 | 44831872 | 120957 | T | C | C | 0.77419 | T | 0.22581 | 14 | 0.45161 |
| 1606 | S2_44856244 | 2 | 44856244 | 24372 | C | T | C | 0.95161 | T | 0.04839 | 3 | 0.09677 |
| 1607 | S2_45769538 | 2 | 45769538 | 913294 | A | G | G | 0.67742 | A | 0.32258 | 14 | 0.45161 |
| 1608 | S2_46338544 | 2 | 46338544 | 569006 | C | T | C | 0.90323 | T | 0.09677 | 6 | 0.19355 |
| 1609 | S2_46338651 | 2 | 46338651 | 107 | G | A | G | 0.91935 | A | 0.08065 | 5 | 0.16129 |
| 1610 | S2_46483313 | 2 | 46483313 | 144662 | A | G | A | 0.8871 | G | 0.1129 | 7 | 0.22581 |
| 1611 | S2_46483349 | 2 | 46483349 | 36 | G | A | G | 0.91935 | A | 0.08065 | 3 | 0.09677 |
| 1612 | S2_46483449 | 2 | 46483449 | 100 | T | G | T | 0.91935 | G | 0.08065 | 3 | 0.09677 |
| 1613 | S2_46549589 | 2 | 46549589 | 66140 | C | A | C | 0.91935 | A | 0.08065 | 5 | 0.16129 |
| 1614 | S2_46549675 | 2 | 46549675 | 86 | G | C | G | 0.54839 | C | 0.45161 | 14 | 0.45161 |
| 1615 | S2_46549712 | 2 | 46549712 | 37 | C | T | C | 0.74194 | T | 0.25806 | 14 | 0.45161 |
| 1616 | S2_46814458 | 2 | 46814458 | 264746 | A | G | G | 0.87097 | A | 0.12903 | 8 | 0.25806 |
| 1617 | S2_46814479 | 2 | 46814479 | 21 | G | C | C | 0.87097 | G | 0.12903 | 8 | 0.25806 |
| 1618 | S2_46814564 | 2 | 46814564 | 85 | G | A | A | 0.85484 | G | 0.14516 | 9 | 0.29032 |

| Site number | SNP name | Chromosome | Physical position | Physical distance from the previous SNP | Reference allele | Alternative allele | Major allele | Major allele frequency | Minor allele | Minor allele frequency | Number heterozygous | Heterozygosity |
|---|---|---|---|---|---|---|---|---|---|---|---|---|
| 1619 | S2_46928259 | 2 | 46928259 | 113695 | C | T | C | 0.93548 | T | 0.06452 | 4 | 0.12903 |
| 1620 | S2_47416697 | 2 | 47416697 | 488438 | A | G | A | 0.87097 | G | 0.12903 | 6 | 0.19355 |
| 1621 | S2_47416791 | 2 | 47416791 | 94 | G | A | G | 0.83871 | A | 0.16129 | 10 | 0.32258 |
| 1622 | S2_47781763 | 2 | 47781763 | 364972 | A | G | A | 0.5 | G | 0.5 | 19 | 0.6129 |
| 1623 | S2_47781871 | 2 | 47781871 | 108 | G | A | G | 0.91935 | A | 0.08065 | 5 | 0.16129 |
| 1624 | S2_47781958 | 2 | 47781958 | 87 | G | A | G | 0.6129 | A | 0.3871 | 12 | 0.3871 |
| 1625 | S2_47996869 | 2 | 47996869 | 214911 | A | G | G | 0.59677 | A | 0.40323 | 15 | 0.48387 |
| 1626 | S2_48004038 | 2 | 48004038 | 7169 | C | T | C | 0.93548 | T | 0.06452 | 4 | 0.12903 |
| 1627 | S2_48336130 | 2 | 48336130 | 332092 | T | C | C | 0.79032 | T | 0.20968 | 11 | 0.35484 |
| 1628 | S2_48881530 | 2 | 48881530 | 545400 | A | G | G | 0.93548 | A | 0.06452 | 4 | 0.12903 |
| 1629 | S2_49319784 | 2 | 49319784 | 438254 | A | G | G | 0.67742 | A | 0.32258 | 14 | 0.45161 |
| 1630 | S2_49720287 | 2 | 49720287 | 400503 | A | G | G | 0.87097 | A | 0.12903 | 6 | 0.19355 |
| 1631 | S2_50210421 | 2 | 50210421 | 490134 | A | G | G | 0.72581 | A | 0.27419 | 13 | 0.41935 |
| 1632 | S2_50443931 | 2 | 50443931 | 233510 | G | A | G | 0.91935 | A | 0.08065 | 5 | 0.16129 |
| 1633 | S2_50652179 | 2 | 50652179 | 208248 | G | A | G | 0.95161 | A | 0.04839 | 3 | 0.09677 |
| 1634 | S2_50652302 | 2 | 50652302 | 123 | G | A | G | 0.56452 | A | 0.43548 | 13 | 0.41935 |
| 1635 | S2_50652388 | 2 | 50652388 | 86 | G | A | A | 0.72581 | G | 0.27419 | 9 | 0.29032 |
| 1636 | S2_50723770 | 2 | 50723770 | 71382 | A | G | G | 0.83871 | A | 0.16129 | 8 | 0.25806 |
| 1637 | S2_50723791 | 2 | 50723791 | 21 | A | G | G | 0.83871 | A | 0.16129 | 8 | 0.25806 |
| 1638 | S2_50723841 | 2 | 50723841 | 50 | T | C | C | 0.83871 | T | 0.16129 | 8 | 0.25806 |
| 1639 | S2_50723900 | 2 | 50723900 | 59 | C | T | T | 0.83871 | C | 0.16129 | 8 | 0.25806 |
| 1640 | S2_50723979 | 2 | 50723979 | 79 | C | T | T | 0.83871 | C | 0.16129 | 8 | 0.25806 |
| 1641 | S2_50723995 | 2 | 50723995 | 16 | C | T | C | 0.56452 | T | 0.43548 | 17 | 0.54839 |
| 1642 | S2_51521093 | 2 | 51521093 | 797098 | T | G | T | 0.56452 | G | 0.43548 | 13 | 0.41935 |
| 1643 | S2_51521177 | 2 | 51521177 | 84 | G | C | G | 0.93548 | C | 0.06452 | 4 | 0.12903 |
| 1644 | S2_51918200 | 2 | 51918200 | 397023 | A | G | A | 0.59677 | G | 0.40323 | 15 | 0.48387 |
| 1645 | S2_51918240 | 2 | 51918240 | 40 | C | A | C | 0.79032 | A | 0.20968 | 11 | 0.35484 |
| 1646 | S2_51918289 | 2 | 51918289 | 49 | T | C | T | 0.79032 | C | 0.20968 | 11 | 0.35484 |
| 1647 | S2_51918297 | 2 | 51918297 | 8 | T | A | T | 0.79032 | A | 0.20968 | 11 | 0.35484 |
| 1648 | S2_53320578 | 2 | 53320578 | 1402281 | C | T | C | 0.95161 | T | 0.04839 | 3 | 0.09677 |
| 1649 | S2_53320621 | 2 | 53320621 | 43 | G | A | G | 0.8871 | A | 0.1129 | 5 | 0.16129 |
| 1650 | S2_54793114 | 2 | 54793114 | 1472493 | C | T | T | 0.54839 | C | 0.45161 | 14 | 0.45161 |
| 1651 | S2_54793310 | 2 | 54793310 | 196 | C | T | C | 0.74194 | T | 0.25806 | 14 | 0.45161 |
| 1652 | S2_55504986 | 2 | 55504986 | 711676 | A | C | A | 0.90323 | C | 0.09677 | 6 | 0.19355 |
| 1653 | S2_55505025 | 2 | 55505025 | 39 | C | T | C | 0.93548 | T | 0.06452 | 4 | 0.12903 |
| 1654 | S2_55544167 | 2 | 55544167 | 39142 | C | T | C | 0.79032 | T | 0.20968 | 9 | 0.29032 |
| 1655 | S2_55544309 | 2 | 55544309 | 142 | G | A | G | 0.74194 | A | 0.25806 | 12 | 0.3871 |
| 1656 | S2_55839353 | 2 | 55839353 | 295044 | T | C | C | 0.67742 | T | 0.32258 | 14 | 0.45161 |
| 1657 | S2_55839442 | 2 | 55839442 | 89 | A | T | A | 0.82258 | T | 0.17742 | 9 | 0.29032 |
| 1658 | S2_56417316 | 2 | 56417316 | 577874 | A | G | A | 0.87097 | G | 0.12903 | 8 | 0.25806 |
| 1659 | S2_56417366 | 2 | 56417366 | 50 | T | G | T | 0.70968 | G | 0.29032 | 18 | 0.58065 |
| 1660 | S2_56417408 | 2 | 56417408 | 42 | C | T | C | 0.70968 | T | 0.29032 | 18 | 0.58065 |
| 1661 | S2_56661623 | 2 | 56661623 | 244215 | A | C | C | 0.77419 | A | 0.22581 | 10 | 0.32258 |
| 1662 | S2_57053333 | 2 | 57053333 | 391710 | C | T | C | 0.93548 | T | 0.06452 | 4 | 0.12903 |
| 1663 | S2_57053419 | 2 | 57053419 | 86 | G | T | T | 0.74194 | G | 0.25806 | 14 | 0.45161 |
| 1664 | S2_57053504 | 2 | 57053504 | 85 | T | G | T | 0.69355 | G | 0.30645 | 17 | 0.54839 |
| 1665 | S2_57326234 | 2 | 57326234 | 272730 | A | G | A | 0.83871 | G | 0.16129 | 10 | 0.32258 |
| 1666 | S2_57605372 | 2 | 57605372 | 279138 | G | A | G | 0.93548 | A | 0.06452 | 4 | 0.12903 |
| 1667 | S2_58675766 | 2 | 58675766 | 1070394 | C | A | C | 0.91935 | A | 0.08065 | 3 | 0.09677 |
| 1668 | S2_59133186 | 2 | 59133186 | 457420 | A | G | G | 0.83871 | A | 0.16129 | 8 | 0.25806 |
| 1669 | S2_59667332 | 2 | 59667332 | 534146 | G | A | G | 0.58065 | A | 0.41935 | 14 | 0.45161 |
| 1670 | S2_59667406 | 2 | 59667406 | 74 | C | A | C | 0.58065 | A | 0.41935 | 14 | 0.45161 |
| 1671 | S2_59723375 | 2 | 59723375 | 55969 | G | C | C | 0.56452 | G | 0.43548 | 19 | 0.6129 |
| 1672 | S2_59723473 | 2 | 59723473 | 98 | G | A | G | 0.77419 | A | 0.22581 | 10 | 0.32258 |

| Site number | SNP name | Chromosome | Physical position | Physical distance from the previous SNP | Reference allele | Alternative allele | Major allele | Major allele frequency | Minor allele | Minor allele frequency | Number heterozygous | Heterozygosity |
|---|---|---|---|---|---|---|---|---|---|---|---|---|
| 1673 | S2_59723605 | 2 | 59723605 | 132 | T | C | T | 0.66129 | C | 0.33871 | 11 | 0.35484 |
| 1674 | S2_59739380 | 2 | 59739380 | 15775 | A | G | G | 0.66129 | A | 0.33871 | 15 | 0.48387 |
| 1675 | S2_59739398 | 2 | 59739398 | 18 | G | A | G | 0.79032 | A | 0.20968 | 7 | 0.22581 |
| 1676 | S2_59798749 | 2 | 59798749 | 59351 | T | C | C | 0.56452 | T | 0.43548 | 17 | 0.54839 |
| 1677 | S2_60095611 | 2 | 60095611 | 296862 | C | T | T | 0.79032 | C | 0.20968 | 7 | 0.22581 |
| 1678 | S2_60095613 | 2 | 60095613 | 2 | A | C | C | 0.79032 | A | 0.20968 | 7 | 0.22581 |
| 1679 | S2_60137777 | 2 | 60137777 | 42164 | T | C | T | 0.93548 | C | 0.06452 | 4 | 0.12903 |
| 1680 | S2_61376591 | 2 | 61376591 | 1238814 | C | G | G | 0.66129 | C | 0.33871 | 15 | 0.48387 |
| 1681 | S2_61644570 | 2 | 61644570 | 267979 | A | G | A | 0.64516 | G | 0.35484 | 14 | 0.45161 |
| 1682 | S2_61801184 | 2 | 61801184 | 156614 | A | T | A | 0.95161 | T | 0.04839 | 3 | 0.09677 |
| 1683 | S2_61993658 | 2 | 61993658 | 192474 | T | C | C | 0.67742 | T | 0.32258 | 16 | 0.51613 |
| 1684 | S2_61993695 | 2 | 61993695 | 37 | T | C | C | 0.67742 | T | 0.32258 | 16 | 0.51613 |
| 1685 | S2_62168666 | 2 | 62168666 | 174971 | C | A | C | 0.82258 | A | 0.17742 | 11 | 0.35484 |
| 1686 | S2_62168737 | 2 | 62168737 | 71 | C | T | C | 0.93548 | T | 0.06452 | 4 | 0.12903 |
| 1687 | S2_62168765 | 2 | 62168765 | 28 | G | A | G | 0.59677 | A | 0.40323 | 15 | 0.48387 |
| 1688 | S2_62168770 | 2 | 62168770 | 5 | G | A | G | 0.8871 | A | 0.1129 | 7 | 0.22581 |
| 1689 | S2_62168879 | 2 | 62168879 | 109 | A | G | A | 0.90323 | G | 0.09677 | 6 | 0.19355 |
| 1690 | S2_62198706 | 2 | 62198706 | 29827 | A | G | A | 0.85484 | G | 0.14516 | 5 | 0.16129 |
| 1691 | S2_62414798 | 2 | 62414798 | 216092 | C | T | C | 0.93548 | T | 0.06452 | 4 | 0.12903 |
| 1692 | S2_62463184 | 2 | 62463184 | 48386 | G | A | G | 0.62903 | A | 0.37097 | 13 | 0.41935 |
| 1693 | S2_62463246 | 2 | 62463246 | 62 | G | A | G | 0.62903 | A | 0.37097 | 13 | 0.41935 |
| 1694 | S2_62985560 | 2 | 62985560 | 522314 | T | C | T | 0.83871 | C | 0.16129 | 8 | 0.25806 |
| 1695 | S2_63523598 | 2 | 63523598 | 538038 | A | G | G | 0.53226 | A | 0.46774 | 15 | 0.48387 |
| 1696 | S2_63523608 | 2 | 63523608 | 10 | C | T | C | 0.85484 | T | 0.14516 | 7 | 0.22581 |
| 1697 | S2_63523688 | 2 | 63523688 | 80 | T | C | C | 0.53226 | T | 0.46774 | 15 | 0.48387 |
| 1698 | S2_64013686 | 2 | 64013686 | 489998 | C | T | C | 0.58065 | T | 0.41935 | 18 | 0.58065 |
| 1699 | S2_64013736 | 2 | 64013736 | 50 | C | T | C | 0.58065 | T | 0.41935 | 18 | 0.58065 |
| 1700 | S2_64013790 | 2 | 64013790 | 54 | C | T | C | 0.85484 | T | 0.14516 | 7 | 0.22581 |
| 1701 | S2_64013835 | 2 | 64013835 | 45 | A | G | A | 0.58065 | G | 0.41935 | 18 | 0.58065 |
| 1702 | S2_64051264 | 2 | 64051264 | 37429 | C | T | C | 0.93548 | T | 0.06452 | 4 | 0.12903 |
| 1703 | S2_64051305 | 2 | 64051305 | 41 | C | T | C | 0.93548 | T | 0.06452 | 4 | 0.12903 |
| 1704 | S2_64607433 | 2 | 64607433 | 556128 | G | C | C | 0.51613 | G | 0.48387 | 16 | 0.51613 |
| 1705 | S2_64771390 | 2 | 64771390 | 163957 | G | A | A | 0.56452 | G | 0.43548 | 19 | 0.6129 |
| 1706 | S2_64771394 | 2 | 64771394 | 4 | T | C | C | 0.54839 | T | 0.45161 | 18 | 0.58065 |
| 1707 | S2_64771399 | 2 | 64771399 | 5 | A | T | T | 0.56452 | A | 0.43548 | 19 | 0.6129 |
| 1708 | S2_64771411 | 2 | 64771411 | 12 | C | T | C | 0.91935 | T | 0.08065 | 5 | 0.16129 |
| 1709 | S2_64771433 | 2 | 64771433 | 22 | G | A | G | 0.91935 | A | 0.08065 | 5 | 0.16129 |
| 1710 | S2_64771448 | 2 | 64771448 | 15 | G | A | A | 0.56452 | G | 0.43548 | 19 | 0.6129 |
| 1711 | S2_64771576 | 2 | 64771576 | 128 | G | A | A | 0.56452 | G | 0.43548 | 19 | 0.6129 |
| 1712 | S2_64771581 | 2 | 64771581 | 5 | G | A | A | 0.56452 | G | 0.43548 | 19 | 0.6129 |
| 1713 | S2_64771609 | 2 | 64771609 | 28 | C | T | T | 0.56452 | C | 0.43548 | 19 | 0.6129 |
| 1714 | S2_65465494 | 2 | 65465494 | 693885 | C | T | C | 0.82258 | T | 0.17742 | 9 | 0.29032 |
| 1715 | S2_65700897 | 2 | 65700897 | 235403 | T | C | C | 0.93548 | T | 0.06452 | 4 | 0.12903 |
| 1716 | S2_65783053 | 2 | 65783053 | 82156 | G | A | G | 0.79032 | A | 0.20968 | 11 | 0.35484 |
| 1717 | S2_65783080 | 2 | 65783080 | 27 | C | T | C | 0.93548 | T | 0.06452 | 4 | 0.12903 |
| 1718 | S2_65783081 | 2 | 65783081 | 1 | G | A | G | 0.8871 | A | 0.1129 | 7 | 0.22581 |
| 1719 | S2_65783124 | 2 | 65783124 | 43 | A | G | A | 0.91935 | G | 0.08065 | 5 | 0.16129 |
| 1720 | S2_65851920 | 2 | 65851920 | 68796 | C | T | C | 0.85484 | T | 0.14516 | 9 | 0.29032 |
| 1721 | S2_66294393 | 2 | 66294393 | 442473 | C | T | C | 0.82258 | T | 0.17742 | 7 | 0.22581 |
| 1722 | S2_66413775 | 2 | 66413775 | 119382 | A | G | A | 0.95161 | G | 0.04839 | 3 | 0.09677 |
| 1723 | S2_66614041 | 2 | 66614041 | 200266 | T | C | C | 0.82258 | T | 0.17742 | 11 | 0.35484 |
| 1724 | S2_66831731 | 2 | 66831731 | 217690 | C | T | C | 0.93548 | T | 0.06452 | 4 | 0.12903 |
| 1725 | S2_66831732 | 2 | 66831732 | 1 | C | G | C | 0.8871 | G | 0.1129 | 7 | 0.22581 |
| 1726 | S2_66883332 | 2 | 66883332 | 51600 | C | T | C | 0.80645 | T | 0.19355 | 12 | 0.3871 |

| Site number | SNP name | Chromosome | Physical position | Physical distance from the previous SNP | Reference allele | Alternative allele | Major allele | Major allele frequency | Minor allele | Minor allele frequency | Number heterozygous | Heterozygosity |
|---|---|---|---|---|---|---|---|---|---|---|---|---|
| 1727 | S2_67634348 | 2 | 67634348 | 751016 | T | C | T | 0.64516 | C | 0.35484 | 14 | 0.45161 |
| 1728 | S2_67634356 | 2 | 67634356 | 8 | C | A | C | 0.77419 | A | 0.22581 | 12 | 0.3871 |
| 1729 | S2_68567730 | 2 | 68567730 | 933374 | G | A | G | 0.91935 | A | 0.08065 | 5 | 0.16129 |
| 1730 | S2_68567736 | 2 | 68567736 | 6 | G | A | G | 0.80645 | A | 0.19355 | 8 | 0.25806 |
| 1731 | S2_68567809 | 2 | 68567809 | 73 | C | T | C | 0.80645 | T | 0.19355 | 8 | 0.25806 |
| 1732 | S2_68567827 | 2 | 68567827 | 18 | G | A | G | 0.80645 | A | 0.19355 | 12 | 0.3871 |
| 1733 | S2_68567832 | 2 | 68567832 | 5 | T | C | T | 0.93548 | C | 0.06452 | 4 | 0.12903 |
| 1734 | S2_68567860 | 2 | 68567860 | 28 | A | C | A | 0.51613 | C | 0.48387 | 18 | 0.58065 |
| 1735 | S2_68567902 | 2 | 68567902 | 42 | T | C | T | 0.80645 | C | 0.19355 | 8 | 0.25806 |
| 1736 | S2_68742292 | 2 | 68742292 | 174390 | G | A | G | 0.82258 | A | 0.17742 | 9 | 0.29032 |
| 1737 | S2_69024133 | 2 | 69024133 | 281841 | G | T | G | 0.66129 | T | 0.33871 | 13 | 0.41935 |
| 1738 | S2_70719897 | 2 | 70719897 | 1695764 | G | A | G | 0.85484 | A | 0.14516 | 7 | 0.22581 |
| 1739 | S2_70720014 | 2 | 70720014 | 117 | G | A | G | 0.91935 | A | 0.08065 | 5 | 0.16129 |
| 1740 | S2_71807582 | 2 | 71807582 | 1087568 | G | A | G | 0.83871 | A | 0.16129 | 10 | 0.32258 |
| 1741 | S2_71807712 | 2 | 71807712 | 130 | G | C | G | 0.70968 | C | 0.29032 | 14 | 0.45161 |
| 1742 | S2_72368617 | 2 | 72368617 | 560905 | A | G | A | 0.95161 | G | 0.04839 | 3 | 0.09677 |
| 1743 | S2_72368792 | 2 | 72368792 | 175 | A | G | A | 0.93548 | G | 0.06452 | 4 | 0.12903 |
| 1744 | S2_73272921 | 2 | 73272921 | 904129 | C | G | C | 0.91935 | G | 0.08065 | 5 | 0.16129 |
| 1745 | S2_73273017 | 2 | 73273017 | 96 | G | A | G | 0.8871 | A | 0.1129 | 5 | 0.16129 |
| 1746 | S2_73273041 | 2 | 73273041 | 24 | C | A | C | 0.87097 | A | 0.12903 | 8 | 0.25806 |
| 1747 | S2_73600828 | 2 | 73600828 | 327787 | T | C | T | 0.90323 | C | 0.09677 | 6 | 0.19355 |
| 1748 | S2_73600836 | 2 | 73600836 | 8 | G | A | G | 0.53226 | A | 0.46774 | 17 | 0.54839 |
| 1749 | S2_73600852 | 2 | 73600852 | 16 | A | G | G | 0.58065 | A | 0.41935 | 18 | 0.58065 |
| 1750 | S2_73600921 | 2 | 73600921 | 69 | A | C | A | 0.8871 | C | 0.1129 | 7 | 0.22581 |
| 1751 | S2_73600925 | 2 | 73600925 | 4 | C | A | C | 0.8871 | A | 0.1129 | 7 | 0.22581 |
| 1752 | S2_73600958 | 2 | 73600958 | 33 | A | G | G | 0.87097 | A | 0.12903 | 8 | 0.25806 |
| 1753 | S2_73622070 | 2 | 73622070 | 21112 | G | A | G | 0.91935 | A | 0.08065 | 5 | 0.16129 |
| 1754 | S2_73622087 | 2 | 73622087 | 17 | A | G | A | 0.95161 | G | 0.04839 | 3 | 0.09677 |
| 1755 | S2_73622146 | 2 | 73622146 | 59 | C | G | C | 0.95161 | G | 0.04839 | 3 | 0.09677 |
| 1756 | S2_73947529 | 2 | 73947529 | 325383 | G | A | G | 0.87097 | A | 0.12903 | 8 | 0.25806 |
| 1757 | S2_73959640 | 2 | 73959640 | 12111 | G | A | G | 0.93548 | A | 0.06452 | 4 | 0.12903 |
| 1758 | S2_73959686 | 2 | 73959686 | 46 | A | G | A | 0.70968 | G | 0.29032 | 12 | 0.3871 |
| 1759 | S2_74294529 | 2 | 74294529 | 334843 | C | T | C | 0.8871 | T | 0.1129 | 7 | 0.22581 |
| 1760 | S2_74294635 | 2 | 74294635 | 106 | G | A | G | 0.91935 | A | 0.08065 | 5 | 0.16129 |
| 1761 | S2_74443389 | 2 | 74443389 | 148754 | A | G | A | 0.93548 | G | 0.06452 | 4 | 0.12903 |
| 1762 | S2_74443541 | 2 | 74443541 | 152 | G | A | G | 0.93548 | A | 0.06452 | 4 | 0.12903 |
| 1763 | S2_75068087 | 2 | 75068087 | 624546 | T | C | T | 0.93548 | C | 0.06452 | 2 | 0.06452 |
| 1764 | S2_75526265 | 2 | 75526265 | 458178 | T | C | C | 0.56452 | T | 0.43548 | 13 | 0.41935 |
| 1765 | S2_75664715 | 2 | 75664715 | 138450 | A | C | C | 0.95161 | A | 0.04839 | 3 | 0.09677 |
| 1766 | S2_76068726 | 2 | 76068726 | 404011 | T | C | T | 0.67742 | C | 0.32258 | 14 | 0.45161 |
| 1767 | S2_76068736 | 2 | 76068736 | 10 | G | A | G | 0.67742 | A | 0.32258 | 16 | 0.51613 |
| 1768 | S2_76425649 | 2 | 76425649 | 356913 | A | G | A | 0.59677 | G | 0.40323 | 17 | 0.54839 |
| 1769 | S2_76553723 | 2 | 76553723 | 128074 | A | G | A | 0.87097 | G | 0.12903 | 6 | 0.19355 |
| 1770 | S2_76553798 | 2 | 76553798 | 75 | A | G | A | 0.93548 | G | 0.06452 | 4 | 0.12903 |
| 1771 | S2_76553854 | 2 | 76553854 | 56 | T | C | C | 0.67742 | T | 0.32258 | 10 | 0.32258 |
| 1772 | S2_76553915 | 2 | 76553915 | 61 | C | T | C | 0.91935 | T | 0.08065 | 5 | 0.16129 |
| 1773 | S2_76737725 | 2 | 76737725 | 183810 | G | A | G | 0.95161 | A | 0.04839 | 3 | 0.09677 |
| 1774 | S2_76737734 | 2 | 76737734 | 9 | C | T | C | 0.80645 | T | 0.19355 | 12 | 0.3871 |
| 1775 | S2_76737750 | 2 | 76737750 | 16 | G | T | G | 0.80645 | T | 0.19355 | 12 | 0.3871 |
| 1776 | S2_76737802 | 2 | 76737802 | 52 | G | A | G | 0.8871 | A | 0.1129 | 7 | 0.22581 |
| 1777 | S2_76737809 | 2 | 76737809 | 7 | G | A | A | 0.87097 | G | 0.12903 | 6 | 0.19355 |
| 1778 | S2_76737833 | 2 | 76737833 | 24 | C | T | C | 0.77419 | T | 0.22581 | 12 | 0.3871 |
| 1779 | S2_76737879 | 2 | 76737879 | 46 | C | G | C | 0.8871 | G | 0.1129 | 7 | 0.22581 |
| 1780 | S2_76737882 | 2 | 76737882 | 3 | T | G | T | 0.80645 | G | 0.19355 | 12 | 0.3871 |

| Site number | SNP name | Chromosome | Physical position | Physical distance from the previous SNP | Reference allele | Alternative allele | Major allele | Major allele frequency | Minor allele | Minor allele frequency | Number heterozygous | Heterozygosity |
|---|---|---|---|---|---|---|---|---|---|---|---|---|
| 1781 | S2_77108634 | 2 | 77108634 | 370752 | A | G | G | 0.69355 | A | 0.30645 | 11 | 0.35484 |
| 1782 | S2_77108661 | 2 | 77108661 | 27 | C | T | C | 0.83871 | T | 0.16129 | 8 | 0.25806 |
| 1783 | S2_77593632 | 2 | 77593632 | 484971 | G | A | G | 0.91935 | A | 0.08065 | 5 | 0.16129 |
| 1784 | S2_78148608 | 2 | 78148608 | 554976 | G | T | G | 0.91935 | T | 0.08065 | 5 | 0.16129 |
| 1785 | S2_78190032 | 2 | 78190032 | 41424 | C | T | T | 0.59677 | C | 0.40323 | 21 | 0.67742 |
| 1786 | S2_79010450 | 2 | 79010450 | 820418 | G | A | G | 0.83871 | A | 0.16129 | 8 | 0.25806 |
| 1787 | S2_79010557 | 2 | 79010557 | 107 | G | A | G | 0.64516 | A | 0.35484 | 16 | 0.51613 |
| 1788 | S2_79010609 | 2 | 79010609 | 52 | A | C | A | 0.85484 | C | 0.14516 | 9 | 0.29032 |
| 1789 | S2_79666520 | 2 | 79666520 | 655911 | A | G | A | 0.90323 | G | 0.09677 | 6 | 0.19355 |
| 1790 | S2_80164430 | 2 | 80164430 | 497910 | C | T | C | 0.83871 | T | 0.16129 | 6 | 0.19355 |
| 1791 | S2_80409865 | 2 | 80409865 | 245435 | G | T | G | 0.66129 | T | 0.33871 | 13 | 0.41935 |
| 1792 | S2_80649657 | 2 | 80649657 | 239792 | C | T | C | 0.95161 | T | 0.04839 | 3 | 0.09677 |
| 1793 | S2_80649744 | 2 | 80649744 | 87 | G | A | G | 0.93548 | A | 0.06452 | 4 | 0.12903 |
| 1794 | S2_80649761 | 2 | 80649761 | 17 | G | A | G | 0.51613 | A | 0.48387 | 18 | 0.58065 |
| 1795 | S2_80649815 | 2 | 80649815 | 54 | T | A | T | 0.87097 | A | 0.12903 | 8 | 0.25806 |
| 1796 | S2_80649817 | 2 | 80649817 | 2 | T | A | T | 0.87097 | A | 0.12903 | 8 | 0.25806 |
| 1797 | S2_81199071 | 2 | 81199071 | 549254 | G | A | G | 0.91935 | A | 0.08065 | 5 | 0.16129 |
| 1798 | S2_81199146 | 2 | 81199146 | 75 | G | A | G | 0.95161 | A | 0.04839 | 3 | 0.09677 |
| 1799 | S2_81199251 | 2 | 81199251 | 105 | A | G | A | 0.74194 | G | 0.25806 | 14 | 0.45161 |
| 1800 | S2_81304551 | 2 | 81304551 | 105300 | C | T | C | 0.93548 | T | 0.06452 | 4 | 0.12903 |
| 1801 | S2_81550432 | 2 | 81550432 | 245881 | T | C | T | 0.69355 | C | 0.30645 | 17 | 0.54839 |
| 1802 | S2_81550680 | 2 | 81550680 | 248 | G | C | G | 0.75806 | C | 0.24194 | 11 | 0.35484 |
| 1803 | S2_82574576 | 2 | 82574576 | 1023896 | A | G | G | 0.87097 | A | 0.12903 | 8 | 0.25806 |
| 1804 | S2_82574649 | 2 | 82574649 | 73 | T | A | T | 0.91935 | A | 0.08065 | 5 | 0.16129 |
| 1805 | S2_82757518 | 2 | 82757518 | 182869 | G | A | G | 0.82258 | A | 0.17742 | 11 | 0.35484 |
| 1806 | S2_82757576 | 2 | 82757576 | 58 | C | T | T | 0.53226 | C | 0.46774 | 15 | 0.48387 |
| 1807 | S2_82757583 | 2 | 82757583 | 7 | C | T | C | 0.95161 | T | 0.04839 | 3 | 0.09677 |
| 1808 | S2_83455282 | 2 | 83455282 | 697699 | G | T | G | 0.8871 | T | 0.1129 | 7 | 0.22581 |
| 1809 | S2_83675425 | 2 | 83675425 | 220143 | C | T | T | 0.62903 | C | 0.37097 | 17 | 0.54839 |
| 1810 | S2_83675486 | 2 | 83675486 | 61 | T | C | C | 0.62903 | T | 0.37097 | 17 | 0.54839 |
| 1811 | S2_84043499 | 2 | 84043499 | 368013 | C | T | C | 0.74194 | T | 0.25806 | 14 | 0.45161 |
| 1812 | S2_84043542 | 2 | 84043542 | 43 | G | T | G | 0.74194 | T | 0.25806 | 14 | 0.45161 |
| 1813 | S2_84473586 | 2 | 84473586 | 430044 | A | G | A | 0.66129 | G | 0.33871 | 17 | 0.54839 |
| 1814 | S2_84473633 | 2 | 84473633 | 47 | G | A | G | 0.87097 | A | 0.12903 | 8 | 0.25806 |
| 1815 | S2_84473649 | 2 | 84473649 | 16 | C | A | C | 0.93548 | A | 0.06452 | 4 | 0.12903 |
| 1816 | S2_84473711 | 2 | 84473711 | 62 | C | A | C | 0.87097 | A | 0.12903 | 8 | 0.25806 |
| 1817 | S2_84473716 | 2 | 84473716 | 5 | C | A | C | 0.80645 | A | 0.19355 | 10 | 0.32258 |
| 1818 | S2_84473722 | 2 | 84473722 | 6 | A | G | A | 0.59677 | G | 0.40323 | 15 | 0.48387 |
| 1819 | S2_84473732 | 2 | 84473732 | 10 | G | T | T | 0.54839 | G | 0.45161 | 16 | 0.51613 |
| 1820 | S2_85504215 | 2 | 85504215 | 1030483 | C | T | C | 0.6129 | T | 0.3871 | 16 | 0.51613 |
| 1821 | S2_85504313 | 2 | 85504313 | 98 | G | A | G | 0.6129 | A | 0.3871 | 16 | 0.51613 |
| 1822 | S2_85539752 | 2 | 85539752 | 35439 | A | T | T | 0.75806 | A | 0.24194 | 11 | 0.35484 |
| 1823 | S2_85539820 | 2 | 85539820 | 68 | C | T | T | 0.77419 | C | 0.22581 | 10 | 0.32258 |
| 1824 | S2_85811899 | 2 | 85811899 | 272079 | G | A | A | 0.51613 | G | 0.48387 | 14 | 0.45161 |
| 1825 | S2_86356092 | 2 | 86356092 | 544193 | T | C | T | 0.93548 | C | 0.06452 | 4 | 0.12903 |
| 1826 | S2_86356172 | 2 | 86356172 | 80 | C | T | C | 0.93548 | T | 0.06452 | 4 | 0.12903 |
| 1827 | S2_86356202 | 2 | 86356202 | 30 | C | T | C | 0.87097 | T | 0.12903 | 8 | 0.25806 |
| 1828 | S2_86356203 | 2 | 86356203 | 1 | A | C | A | 0.8871 | C | 0.1129 | 5 | 0.16129 |
| 1829 | S2_86356225 | 2 | 86356225 | 22 | G | A | G | 0.93548 | A | 0.06452 | 4 | 0.12903 |
| 1830 | S2_87345293 | 2 | 87345293 | 989068 | G | A | G | 0.75806 | A | 0.24194 | 15 | 0.48387 |
| 1831 | S2_87345313 | 2 | 87345313 | 20 | A | T | A | 0.93548 | T | 0.06452 | 4 | 0.12903 |
| 1832 | S2_87345452 | 2 | 87345452 | 139 | T | G | T | 0.90323 | G | 0.09677 | 4 | 0.12903 |
| 1833 | S2_87536672 | 2 | 87536672 | 191220 | C | T | T | 0.91935 | C | 0.08065 | 5 | 0.16129 |
| 1834 | S2_87852890 | 2 | 87852890 | 316218 | C | T | C | 0.95161 | T | 0.04839 | 3 | 0.09677 |

| Site number | SNP name | Chromosome | Physical position | Physical distance from the previous SNP | Reference allele | Alternative allele | Major allele | Major allele frequency | Minor allele | Minor allele frequency | Number heterozygous | Heterozygosity |
|---|---|---|---|---|---|---|---|---|---|---|---|---|
| 1835 | S2_87852910 | 2 | 87852910 | 20 | T | A | T | 0.82258 | A | 0.17742 | 9 | 0.29032 |
| 1836 | S2_89105732 | 2 | 89105732 | 1252822 | G | A | G | 0.91935 | A | 0.08065 | 5 | 0.16129 |
| 1837 | S2_89105768 | 2 | 89105768 | 36 | C | T | C | 0.93548 | T | 0.06452 | 4 | 0.12903 |
| 1838 | S2_89166943 | 2 | 89166943 | 61175 | G | A | A | 0.85484 | G | 0.14516 | 7 | 0.22581 |
| 1839 | S2_89166988 | 2 | 89166988 | 45 | G | C | C | 0.85484 | G | 0.14516 | 7 | 0.22581 |
| 1840 | S2_89167037 | 2 | 89167037 | 49 | C | T | T | 0.77419 | C | 0.22581 | 10 | 0.32258 |
| 1841 | S2_89167103 | 2 | 89167103 | 66 | C | T | C | 0.93548 | T | 0.06452 | 4 | 0.12903 |
| 1842 | S2_89167104 | 2 | 89167104 | 1 | A | G | A | 0.93548 | G | 0.06452 | 4 | 0.12903 |
| 1843 | S2_89247596 | 2 | 89247596 | 80492 | T | C | C | 0.82258 | T | 0.17742 | 9 | 0.29032 |
| 1844 | S2_89561635 | 2 | 89561635 | 314039 | G | T | G | 0.95161 | T | 0.04839 | 3 | 0.09677 |
| 1845 | S2_89561645 | 2 | 89561645 | 10 | G | A | G | 0.90323 | A | 0.09677 | 6 | 0.19355 |
| 1846 | S2_89561756 | 2 | 89561756 | 111 | G | A | G | 0.91935 | A | 0.08065 | 5 | 0.16129 |
| 1847 | S2_89680523 | 2 | 89680523 | 118767 | C | T | T | 0.80645 | C | 0.19355 | 10 | 0.32258 |
| 1848 | S2_89680536 | 2 | 89680536 | 13 | G | C | G | 0.90323 | C | 0.09677 | 4 | 0.12903 |
| 1849 | S2_89680579 | 2 | 89680579 | 43 | C | T | C | 0.80645 | T | 0.19355 | 10 | 0.32258 |
| 1850 | S2_89680690 | 2 | 89680690 | 111 | T | C | T | 0.90323 | C | 0.09677 | 6 | 0.19355 |
| 1851 | S2_89865126 | 2 | 89865126 | 184436 | G | A | G | 0.75806 | A | 0.24194 | 11 | 0.35484 |
| 1852 | S2_89865223 | 2 | 89865223 | 97 | G | A | G | 0.95161 | A | 0.04839 | 3 | 0.09677 |
| 1853 | S2_89976220 | 2 | 89976220 | 110997 | G | C | C | 0.90323 | G | 0.09677 | 6 | 0.19355 |
| 1854 | S2_89976224 | 2 | 89976224 | 4 | C | T | C | 0.90323 | T | 0.09677 | 6 | 0.19355 |
| 1855 | S2_89976282 | 2 | 89976282 | 58 | G | C | C | 0.90323 | G | 0.09677 | 6 | 0.19355 |
| 1856 | S2_89976310 | 2 | 89976310 | 28 | A | G | A | 0.75806 | G | 0.24194 | 11 | 0.35484 |
| 1857 | S2_90134218 | 2 | 90134218 | 157908 | C | T | C | 0.80645 | T | 0.19355 | 10 | 0.32258 |
| 1858 | S2_90164133 | 2 | 90164133 | 29915 | C | G | C | 0.74194 | G | 0.25806 | 14 | 0.45161 |
| 1859 | S2_90253199 | 2 | 90253199 | 89066 | G | A | G | 0.58065 | A | 0.41935 | 16 | 0.51613 |
| 1860 | S2_90253235 | 2 | 90253235 | 36 | G | A | G | 0.8871 | A | 0.1129 | 5 | 0.16129 |
| 1861 | S2_90253351 | 2 | 90253351 | 116 | C | T | C | 0.93548 | T | 0.06452 | 4 | 0.12903 |
| 1862 | S2_90469428 | 2 | 90469428 | 216077 | C | T | C | 0.93548 | T | 0.06452 | 4 | 0.12903 |
| 1863 | S2_90489796 | 2 | 90489796 | 20368 | C | T | C | 0.83871 | T | 0.16129 | 8 | 0.25806 |
| 1864 | S2_90489824 | 2 | 90489824 | 28 | T | G | G | 0.93548 | T | 0.06452 | 4 | 0.12903 |
| 1865 | S2_90489856 | 2 | 90489856 | 32 | A | G | A | 0.74194 | G | 0.25806 | 12 | 0.3871 |
| 1866 | S2_90489860 | 2 | 90489860 | 4 | G | A | G | 0.82258 | A | 0.17742 | 9 | 0.29032 |
| 1867 | S2_91255622 | 2 | 91255622 | 765762 | C | T | C | 0.90323 | T | 0.09677 | 4 | 0.12903 |
| 1868 | S2_91488120 | 2 | 91488120 | 232498 | G | A | A | 0.6129 | G | 0.3871 | 12 | 0.3871 |
| 1869 | S2_91488130 | 2 | 91488130 | 10 | A | G | G | 0.6129 | A | 0.3871 | 12 | 0.3871 |
| 1870 | S2_91488313 | 2 | 91488313 | 183 | C | T | C | 0.95161 | T | 0.04839 | 3 | 0.09677 |
| 1871 | S2_91488321 | 2 | 91488321 | 8 | C | G | C | 0.83871 | G | 0.16129 | 8 | 0.25806 |
| 1872 | S2_91772735 | 2 | 91772735 | 284414 | C | G | C | 0.91935 | G | 0.08065 | 3 | 0.09677 |
| 1873 | S2_91772838 | 2 | 91772838 | 103 | G | C | G | 0.87097 | C | 0.12903 | 8 | 0.25806 |
| 1874 | S2_92175996 | 2 | 92175996 | 403158 | G | A | G | 0.77419 | A | 0.22581 | 12 | 0.3871 |
| 1875 | S2_92176020 | 2 | 92176020 | 24 | T | C | T | 0.8871 | C | 0.1129 | 7 | 0.22581 |
| 1876 | S2_92176113 | 2 | 92176113 | 93 | C | T | C | 0.77419 | T | 0.22581 | 12 | 0.3871 |
| 1877 | S2_92426803 | 2 | 92426803 | 250690 | G | T | G | 0.93548 | T | 0.06452 | 4 | 0.12903 |
| 1878 | S2_92426947 | 2 | 92426947 | 144 | C | G | C | 0.85484 | G | 0.14516 | 7 | 0.22581 |
| 1879 | S2_92426967 | 2 | 92426967 | 20 | C | T | C | 0.54839 | T | 0.45161 | 18 | 0.58065 |
| 1880 | S2_92594641 | 2 | 92594641 | 167674 | C | T | C | 0.90323 | T | 0.09677 | 6 | 0.19355 |
| 1881 | S2_93269617 | 2 | 93269617 | 674976 | G | C | G | 0.85484 | C | 0.14516 | 9 | 0.29032 |
| 1882 | S2_93269688 | 2 | 93269688 | 71 | C | G | C | 0.80645 | G | 0.19355 | 12 | 0.3871 |
| 1883 | S2_93269864 | 2 | 93269864 | 176 | C | T | C | 0.90323 | T | 0.09677 | 4 | 0.12903 |
| 1884 | S2_96232300 | 2 | 96232300 | 2962436 | A | G | A | 0.87097 | G | 0.12903 | 8 | 0.25806 |
| 1885 | S2_96232470 | 2 | 96232470 | 170 | A | G | A | 0.93548 | G | 0.06452 | 4 | 0.12903 |
| 1886 | S2_96312700 | 2 | 96312700 | 80230 | G | A | G | 0.93548 | A | 0.06452 | 4 | 0.12903 |
| 1887 | S2_96694791 | 2 | 96694791 | 382091 | C | T | C | 0.85484 | T | 0.14516 | 5 | 0.16129 |
| 1888 | S2_96940297 | 2 | 96940297 | 245506 | T | C | T | 0.91935 | C | 0.08065 | 5 | 0.16129 |

| Site number | SNP name | Chromosome | Physical position | Physical distance from the previous SNP | Reference allele | Alternative allele | Major allele | Major allele frequency | Minor allele | Minor allele frequency | Number heterozygous | Heterozygosity |
|---|---|---|---|---|---|---|---|---|---|---|---|---|
| 1889 | S2_96940453 | 2 | 96940453 | 156 | C | T | C | 0.90323 | T | 0.09677 | 4 | 0.12903 |
| 1890 | S2_97039230 | 2 | 97039230 | 98777 | G | A | G | 0.59677 | A | 0.40323 | 15 | 0.48387 |
| 1891 | S2_97039258 | 2 | 97039258 | 28 | C | T | C | 0.93548 | T | 0.06452 | 4 | 0.12903 |
| 1892 | S2_97958942 | 2 | 97958942 | 919684 | G | A | G | 0.93548 | A | 0.06452 | 4 | 0.12903 |
| 1893 | S2_98352905 | 2 | 98352905 | 393963 | G | A | G | 0.93548 | A | 0.06452 | 4 | 0.12903 |
| 1894 | S2_98353036 | 2 | 98353036 | 131 | C | T | C | 0.91935 | T | 0.08065 | 5 | 0.16129 |
| 1895 | S2_98476000 | 2 | 98476000 | 122964 | G | A | A | 0.90323 | G | 0.09677 | 6 | 0.19355 |
| 1896 | S2_98498439 | 2 | 98498439 | 22439 | G | C | C | 0.75806 | G | 0.24194 | 9 | 0.29032 |
| 1897 | S2_98618784 | 2 | 98618784 | 120345 | A | G | G | 0.66129 | A | 0.33871 | 13 | 0.41935 |
| 1898 | S2_98618810 | 2 | 98618810 | 26 | G | A | G | 0.79032 | A | 0.20968 | 11 | 0.35484 |
| 1899 | S2_98618827 | 2 | 98618827 | 17 | G | C | G | 0.66129 | C | 0.33871 | 13 | 0.41935 |
| 1900 | S2_98774064 | 2 | 98774064 | 155237 | G | A | G | 0.75806 | A | 0.24194 | 9 | 0.29032 |
| 1901 | S2_98795155 | 2 | 98795155 | 21091 | G | A | G | 0.93548 | A | 0.06452 | 4 | 0.12903 |
| 1902 | S2_99085386 | 2 | 99085386 | 290231 | G | A | G | 0.74194 | A | 0.25806 | 10 | 0.32258 |
| 1903 | S2_99085438 | 2 | 99085438 | 52 | A | G | A | 0.93548 | G | 0.06452 | 2 | 0.06452 |
| 1904 | S2_99085473 | 2 | 99085473 | 35 | C | T | C | 0.83871 | T | 0.16129 | 8 | 0.25806 |
| 1905 | S2_99688552 | 2 | 99688552 | 603079 | T | C | T | 0.93548 | C | 0.06452 | 4 | 0.12903 |
| 1906 | S2_99688631 | 2 | 99688631 | 79 | A | G | A | 0.79032 | G | 0.20968 | 11 | 0.35484 |
| 1907 | S2_100172226 | 2 | 100172226 | 483595 | A | G | A | 0.54839 | G | 0.45161 | 16 | 0.51613 |
| 1908 | S2_100172267 | 2 | 100172267 | 41 | G | A | G | 0.93548 | A | 0.06452 | 4 | 0.12903 |
| 1909 | S2_100298658 | 2 | 100298658 | 126391 | G | A | G | 0.95161 | A | 0.04839 | 3 | 0.09677 |
| 1910 | S2_100465301 | 2 | 100465301 | 166643 | G | A | A | 0.90323 | G | 0.09677 | 6 | 0.19355 |
| 1911 | S2_100465407 | 2 | 100465407 | 106 | T | G | T | 0.8871 | G | 0.1129 | 5 | 0.16129 |
| 1912 | S2_101488060 | 2 | 101488060 | 1022653 | C | G | C | 0.74194 | G | 0.25806 | 10 | 0.32258 |
| 1913 | S2_101835203 | 2 | 101835203 | 347143 | T | C | C | 0.87097 | T | 0.12903 | 8 | 0.25806 |
| 1914 | S2_101835292 | 2 | 101835292 | 89 | C | T | C | 0.72581 | T | 0.27419 | 15 | 0.48387 |
| 1915 | S2_101835361 | 2 | 101835361 | 69 | G | A | G | 0.93548 | A | 0.06452 | 4 | 0.12903 |
| 1916 | S2_102239312 | 2 | 102239312 | 403951 | A | G | G | 0.87097 | A | 0.12903 | 6 | 0.19355 |
| 1917 | S2_102239470 | 2 | 102239470 | 158 | T | C | T | 0.85484 | C | 0.14516 | 9 | 0.29032 |
| 1918 | S2_102239549 | 2 | 102239549 | 79 | C | T | C | 0.82258 | T | 0.17742 | 7 | 0.22581 |
| 1919 | S2_102319729 | 2 | 102319729 | 80180 | T | C | T | 0.59677 | C | 0.40323 | 21 | 0.67742 |
| 1920 | S2_103611801 | 2 | 103611801 | 1292072 | C | G | C | 0.90323 | G | 0.09677 | 6 | 0.19355 |
| 1921 | S2_104394972 | 2 | 104394972 | 783171 | T | C | T | 0.95161 | C | 0.04839 | 3 | 0.09677 |
| 1922 | S2_104395103 | 2 | 104395103 | 131 | G | A | A | 0.66129 | G | 0.33871 | 13 | 0.41935 |
| 1923 | S2_104463021 | 2 | 104463021 | 67918 | C | T | C | 0.95161 | T | 0.04839 | 3 | 0.09677 |
| 1924 | S2_104463114 | 2 | 104463114 | 93 | T | C | T | 0.95161 | C | 0.04839 | 3 | 0.09677 |
| 1925 | S2_104463202 | 2 | 104463202 | 88 | T | A | T | 0.83871 | A | 0.16129 | 8 | 0.25806 |
| 1926 | S2_104519699 | 2 | 104519699 | 56497 | T | C | T | 0.8871 | C | 0.1129 | 7 | 0.22581 |
| 1927 | S2_104798893 | 2 | 104798893 | 279194 | G | C | G | 0.91935 | C | 0.08065 | 5 | 0.16129 |
| 1928 | S2_105105999 | 2 | 105105999 | 307106 | G | A | G | 0.85484 | A | 0.14516 | 9 | 0.29032 |
| 1929 | S2_105106012 | 2 | 105106012 | 13 | G | A | G | 0.85484 | A | 0.14516 | 9 | 0.29032 |
| 1930 | S2_105198170 | 2 | 105198170 | 92158 | C | T | C | 0.91935 | T | 0.08065 | 5 | 0.16129 |
| 1931 | S2_105277213 | 2 | 105277213 | 79043 | T | C | T | 0.91935 | C | 0.08065 | 5 | 0.16129 |
| 1932 | S2_105635291 | 2 | 105635291 | 358078 | G | A | A | 0.82258 | G | 0.17742 | 7 | 0.22581 |
| 1933 | S2_105859630 | 2 | 105859630 | 224339 | T | C | T | 0.91935 | C | 0.08065 | 5 | 0.16129 |
| 1934 | S2_106362254 | 2 | 106362254 | 502624 | G | A | A | 0.64516 | G | 0.35484 | 16 | 0.51613 |
| 1935 | S2_106362324 | 2 | 106362324 | 70 | A | G | G | 0.62903 | A | 0.37097 | 17 | 0.54839 |
| 1936 | S2_106485233 | 2 | 106485233 | 122909 | A | G | G | 0.70968 | A | 0.29032 | 14 | 0.45161 |
| 1937 | S2_106485256 | 2 | 106485256 | 23 | T | C | C | 0.70968 | T | 0.29032 | 14 | 0.45161 |
| 1938 | S2_106485274 | 2 | 106485274 | 18 | A | G | A | 0.6129 | G | 0.3871 | 14 | 0.45161 |
| 1939 | S2_106485298 | 2 | 106485298 | 24 | G | A | A | 0.72581 | G | 0.27419 | 15 | 0.48387 |
| 1940 | S2_106485395 | 2 | 106485395 | 97 | G | A | A | 0.70968 | G | 0.29032 | 14 | 0.45161 |
| 1941 | S2_106485465 | 2 | 106485465 | 70 | T | A | A | 0.70968 | T | 0.29032 | 14 | 0.45161 |
| 1942 | S2_106485472 | 2 | 106485472 | 7 | T | C | T | 0.91935 | C | 0.08065 | 5 | 0.16129 |

| Site number | SNP name | Chromosome | Physical position | Physical distance from the previous SNP | Reference allele | Alternative allele | Major allele | Major allele frequency | Minor allele | Minor allele frequency | Number heterozygous | Heterozygosity |
|---|---|---|---|---|---|---|---|---|---|---|---|---|
| 1943 | S2_107303575 | 2 | 107303575 | 818103 | T | C | T | 0.93548 | C | 0.06452 | 4 | 0.12903 |
| 1944 | S2_107303675 | 2 | 107303675 | 100 | T | C | T | 0.85484 | C | 0.14516 | 9 | 0.29032 |
| 1945 | S2_107303683 | 2 | 107303683 | 8 | G | A | G | 0.85484 | A | 0.14516 | 9 | 0.29032 |
| 1946 | S2_107303707 | 2 | 107303707 | 24 | C | T | C | 0.93548 | T | 0.06452 | 4 | 0.12903 |
| 1947 | S2_107303772 | 2 | 107303772 | 65 | G | A | G | 0.90323 | A | 0.09677 | 4 | 0.12903 |
| 1948 | S2_107303792 | 2 | 107303792 | 20 | T | C | C | 0.80645 | T | 0.19355 | 10 | 0.32258 |
| 1949 | S2_107738117 | 2 | 107738117 | 434325 | A | G | G | 0.77419 | A | 0.22581 | 12 | 0.3871 |
| 1950 | S2_107889894 | 2 | 107889894 | 151777 | T | C | T | 0.91935 | C | 0.08065 | 5 | 0.16129 |
| 1951 | S2_107889936 | 2 | 107889936 | 42 | G | A | G | 0.85484 | A | 0.14516 | 7 | 0.22581 |
| 1952 | S2_107890012 | 2 | 107890012 | 76 | T | C | C | 0.80645 | T | 0.19355 | 8 | 0.25806 |
| 1953 | S2_107890013 | 2 | 107890013 | 1 | G | A | G | 0.79032 | A | 0.20968 | 9 | 0.29032 |
| 1954 | S2_107890074 | 2 | 107890074 | 61 | C | T | C | 0.77419 | T | 0.22581 | 10 | 0.32258 |
| 1955 | S2_107890097 | 2 | 107890097 | 23 | C | G | C | 0.64516 | G | 0.35484 | 12 | 0.3871 |
| 1956 | S2_108376129 | 2 | 108376129 | 486032 | T | C | C | 0.70968 | T | 0.29032 | 12 | 0.3871 |
| 1957 | S2_108434414 | 2 | 108434414 | 58285 | A | C | C | 0.56452 | A | 0.43548 | 17 | 0.54839 |
| 1958 | S2_108434420 | 2 | 108434420 | 6 | C | T | C | 0.90323 | T | 0.09677 | 6 | 0.19355 |
| 1959 | S2_108793031 | 2 | 108793031 | 358611 | A | G | G | 0.58065 | A | 0.41935 | 14 | 0.45161 |
| 1960 | S2_108793073 | 2 | 108793073 | 42 | G | A | G | 0.91935 | A | 0.08065 | 5 | 0.16129 |
| 1961 | S2_108839287 | 2 | 108839287 | 46214 | C | A | C | 0.93548 | A | 0.06452 | 4 | 0.12903 |
| 1962 | S2_108839307 | 2 | 108839307 | 20 | T | G | G | 0.64516 | T | 0.35484 | 12 | 0.3871 |
| 1963 | S2_108839371 | 2 | 108839371 | 64 | G | T | G | 0.5 | T | 0.5 | 15 | 0.48387 |
| 1964 | S2_109494744 | 2 | 109494744 | 655373 | C | A | C | 0.93548 | A | 0.06452 | 4 | 0.12903 |
| 1965 | S2_109635051 | 2 | 109635051 | 140307 | G | A | G | 0.90323 | A | 0.09677 | 4 | 0.12903 |
| 1966 | S2_109667396 | 2 | 109667396 | 32345 | C | T | C | 0.8871 | T | 0.1129 | 7 | 0.22581 |
| 1967 | S2_109667463 | 2 | 109667463 | 67 | T | G | G | 0.6129 | T | 0.3871 | 14 | 0.45161 |
| 1968 | S2_109667534 | 2 | 109667534 | 71 | G | A | G | 0.8871 | A | 0.1129 | 7 | 0.22581 |
| 1969 | S2_109667570 | 2 | 109667570 | 36 | T | C | T | 0.8871 | C | 0.1129 | 7 | 0.22581 |
| 1970 | S2_109667575 | 2 | 109667575 | 5 | G | A | G | 0.95161 | A | 0.04839 | 1 | 0.03226 |
| 1971 | S2_110328549 | 2 | 110328549 | 660974 | C | G | C | 0.77419 | G | 0.22581 | 10 | 0.32258 |
| 1972 | S2_110328667 | 2 | 110328667 | 118 | A | G | A | 0.67742 | G | 0.32258 | 12 | 0.3871 |
| 1973 | S2_110328701 | 2 | 110328701 | 34 | G | A | G | 0.70968 | A | 0.29032 | 10 | 0.32258 |
| 1974 | S2_110365250 | 2 | 110365250 | 36549 | G | A | G | 0.83871 | A | 0.16129 | 8 | 0.25806 |
| 1975 | S2_110472523 | 2 | 110472523 | 107273 | G | T | G | 0.91935 | T | 0.08065 | 5 | 0.16129 |
| 1976 | S2_110547020 | 2 | 110547020 | 74497 | G | T | G | 0.95161 | T | 0.04839 | 3 | 0.09677 |
| 1977 | S2_110547024 | 2 | 110547024 | 4 | G | C | C | 0.56452 | G | 0.43548 | 13 | 0.41935 |
| 1978 | S2_110781429 | 2 | 110781429 | 234405 | G | A | G | 0.8871 | A | 0.1129 | 5 | 0.16129 |
| 1979 | S2_110781437 | 2 | 110781437 | 8 | C | T | T | 0.8871 | C | 0.1129 | 5 | 0.16129 |
| 1980 | S2_110781456 | 2 | 110781456 | 19 | C | T | C | 0.91935 | T | 0.08065 | 3 | 0.09677 |
| 1981 | S2_110781534 | 2 | 110781534 | 78 | G | A | G | 0.8871 | A | 0.1129 | 5 | 0.16129 |
| 1982 | S2_111148354 | 2 | 111148354 | 366820 | C | A | C | 0.64516 | A | 0.35484 | 16 | 0.51613 |
| 1983 | S2_111148360 | 2 | 111148360 | 6 | A | T | T | 0.80645 | A | 0.19355 | 12 | 0.3871 |
| 1984 | S2_111390459 | 2 | 111390459 | 242099 | C | T | C | 0.75806 | T | 0.24194 | 13 | 0.41935 |
| 1985 | S2_111390517 | 2 | 111390517 | 58 | T | C | T | 0.74194 | C | 0.25806 | 16 | 0.51613 |
| 1986 | S2_111391607 | 2 | 111391607 | 1090 | G | A | A | 0.51613 | G | 0.48387 | 16 | 0.51613 |
| 1987 | S2_111483801 | 2 | 111483801 | 92194 | T | A | T | 0.83871 | A | 0.16129 | 8 | 0.25806 |
| 1988 | S2_111483834 | 2 | 111483834 | 33 | G | A | G | 0.90323 | A | 0.09677 | 6 | 0.19355 |
| 1989 | S2_111483861 | 2 | 111483861 | 27 | G | T | G | 0.82258 | T | 0.17742 | 11 | 0.35484 |
| 1990 | S2_111483910 | 2 | 111483910 | 49 | C | T | C | 0.82258 | T | 0.17742 | 7 | 0.22581 |
| 1991 | S2_113146452 | 2 | 113146452 | 1662542 | A | G | A | 0.77419 | G | 0.22581 | 14 | 0.45161 |
| 1992 | S2_113235318 | 2 | 113235318 | 88866 | C | T | C | 0.90323 | T | 0.09677 | 6 | 0.19355 |
| 1993 | S2_114002607 | 2 | 114002607 | 767289 | C | A | A | 0.64516 | C | 0.35484 | 10 | 0.32258 |
| 1994 | S2_114190202 | 2 | 114190202 | 187595 | G | A | G | 0.85484 | A | 0.14516 | 5 | 0.16129 |
| 1995 | S2_114458563 | 2 | 114458563 | 268361 | A | G | A | 0.85484 | G | 0.14516 | 7 | 0.22581 |
| 1996 | S2_114484525 | 2 | 114484525 | 25962 | G | A | G | 0.93548 | A | 0.06452 | 4 | 0.12903 |

| Site number | SNP name | Chromosome | Physical position | Physical distance from the previous SNP | Reference allele | Alternative allele | Major allele | Major allele frequency | Minor allele | Minor allele frequency | Number heterozygous | Heterozygosity |
|---|---|---|---|---|---|---|---|---|---|---|---|---|
| 1997 | S2_114877356 | 2 | 114877356 | 392831 | A | G | A | 0.85484 | G | 0.14516 | 7 | 0.22581 |
| 1998 | S2_114877411 | 2 | 114877411 | 55 | G | A | G | 0.85484 | A | 0.14516 | 7 | 0.22581 |
| 1999 | S2_114877453 | 2 | 114877453 | 42 | C | A | C | 0.80645 | A | 0.19355 | 8 | 0.25806 |
| 2000 | S2_114911886 | 2 | 114911886 | 34433 | A | G | A | 0.69355 | G | 0.30645 | 15 | 0.48387 |
| 2001 | S2_114912079 | 2 | 114912079 | 193 | T | C | T | 0.70968 | C | 0.29032 | 12 | 0.3871 |
| 2002 | S2_115902644 | 2 | 115902644 | 990565 | C | T | C | 0.87097 | T | 0.12903 | 6 | 0.19355 |
| 2003 | S2_115902775 | 2 | 115902775 | 131 | G | A | G | 0.67742 | A | 0.32258 | 16 | 0.51613 |
| 2004 | S2_115982025 | 2 | 115982025 | 79250 | A | G | A | 0.8871 | G | 0.1129 | 5 | 0.16129 |
| 2005 | S2_115982128 | 2 | 115982128 | 103 | C | T | C | 0.82258 | T | 0.17742 | 9 | 0.29032 |
| 2006 | S2_115994061 | 2 | 115994061 | 11933 | C | T | C | 0.91935 | T | 0.08065 | 5 | 0.16129 |
| 2007 | S2_116066005 | 2 | 116066005 | 71944 | A | G | G | 0.8871 | A | 0.1129 | 5 | 0.16129 |
| 2008 | S2_116345517 | 2 | 116345517 | 279512 | T | C | T | 0.90323 | C | 0.09677 | 6 | 0.19355 |
| 2009 | S2_116571493 | 2 | 116571493 | 225976 | G | A | G | 0.8871 | A | 0.1129 | 7 | 0.22581 |
| 2010 | S2_116571662 | 2 | 116571662 | 169 | A | G | G | 0.74194 | A | 0.25806 | 14 | 0.45161 |
| 2011 | S2_116805975 | 2 | 116805975 | 234313 | T | C | C | 0.79032 | T | 0.20968 | 9 | 0.29032 |
| 2012 | S2_116806065 | 2 | 116806065 | 90 | G | C | G | 0.95161 | C | 0.04839 | 3 | 0.09677 |
| 2013 | S2_116806125 | 2 | 116806125 | 60 | C | T | C | 0.91935 | T | 0.08065 | 5 | 0.16129 |
| 2014 | S2_116881639 | 2 | 116881639 | 75514 | C | T | C | 0.93548 | T | 0.06452 | 4 | 0.12903 |
| 2015 | S2_116990187 | 2 | 116990187 | 108548 | G | A | G | 0.72581 | A | 0.27419 | 11 | 0.35484 |
| 2016 | S2_117508720 | 2 | 117508720 | 518533 | C | T | C | 0.95161 | T | 0.04839 | 3 | 0.09677 |
| 2017 | S2_117508769 | 2 | 117508769 | 49 | C | T | C | 0.91935 | T | 0.08065 | 5 | 0.16129 |
| 2018 | S2_117508770 | 2 | 117508770 | 1 | G | A | G | 0.93548 | A | 0.06452 | 4 | 0.12903 |
| 2019 | S2_117508842 | 2 | 117508842 | 72 | G | A | G | 0.85484 | A | 0.14516 | 9 | 0.29032 |
| 2020 | S2_117528516 | 2 | 117528516 | 19674 | T | C | T | 0.67742 | C | 0.32258 | 12 | 0.3871 |
| 2021 | S2_117528655 | 2 | 117528655 | 139 | A | G | A | 0.93548 | G | 0.06452 | 4 | 0.12903 |
| 2022 | S2_117677292 | 2 | 117677292 | 148637 | A | G | A | 0.72581 | G | 0.27419 | 11 | 0.35484 |
| 2023 | S2_117677296 | 2 | 117677296 | 4 | A | G | G | 0.72581 | A | 0.27419 | 11 | 0.35484 |
| 2024 | S2_117677302 | 2 | 117677302 | 6 | A | C | C | 0.91935 | A | 0.08065 | 5 | 0.16129 |
| 2025 | S2_117677354 | 2 | 117677354 | 52 | G | A | G | 0.79032 | A | 0.20968 | 13 | 0.41935 |
| 2026 | S2_117677400 | 2 | 117677400 | 46 | T | A | A | 0.69355 | T | 0.30645 | 13 | 0.41935 |
| 2027 | S2_117677470 | 2 | 117677470 | 70 | A | G | A | 0.8871 | G | 0.1129 | 7 | 0.22581 |
| 2028 | S2_118128291 | 2 | 118128291 | 450821 | C | T | C | 0.93548 | T | 0.06452 | 4 | 0.12903 |
| 2029 | S2_118128293 | 2 | 118128293 | 2 | T | C | T | 0.93548 | C | 0.06452 | 4 | 0.12903 |
| 2030 | S2_118191186 | 2 | 118191186 | 62893 | A | G | G | 0.77419 | A | 0.22581 | 10 | 0.32258 |
| 2031 | S2_118194434 | 2 | 118194434 | 3248 | A | G | A | 0.69355 | G | 0.30645 | 15 | 0.48387 |
| 2032 | S2_118194509 | 2 | 118194509 | 75 | C | T | T | 0.82258 | C | 0.17742 | 9 | 0.29032 |
| 2033 | S2_118204723 | 2 | 118204723 | 10214 | A | G | G | 0.8871 | A | 0.1129 | 7 | 0.22581 |
| 2034 | S2_118204726 | 2 | 118204726 | 3 | A | G | G | 0.83871 | A | 0.16129 | 6 | 0.19355 |
| 2035 | S2_118204793 | 2 | 118204793 | 67 | C | T | C | 0.62903 | T | 0.37097 | 11 | 0.35484 |
| 2036 | S2_118823125 | 2 | 118823125 | 618332 | C | A | A | 0.58065 | C | 0.41935 | 10 | 0.32258 |
| 2037 | S2_120634393 | 2 | 120634393 | 1811268 | G | A | G | 0.85484 | A | 0.14516 | 7 | 0.22581 |
| 2038 | S2_120634520 | 2 | 120634520 | 127 | G | C | G | 0.95161 | C | 0.04839 | 1 | 0.03226 |
| 2039 | S2_120680750 | 2 | 120680750 | 46230 | G | A | A | 0.54839 | G | 0.45161 | 16 | 0.51613 |
| 2040 | S2_120680760 | 2 | 120680760 | 10 | G | A | G | 0.93548 | A | 0.06452 | 4 | 0.12903 |
| 2041 | S2_120691048 | 2 | 120691048 | 10288 | C | T | C | 0.8871 | T | 0.1129 | 5 | 0.16129 |
| 2042 | S2_120691059 | 2 | 120691059 | 11 | C | T | C | 0.8871 | T | 0.1129 | 5 | 0.16129 |
| 2043 | S2_120691080 | 2 | 120691080 | 21 | C | T | C | 0.93548 | T | 0.06452 | 4 | 0.12903 |
| 2044 | S2_120691096 | 2 | 120691096 | 16 | C | G | C | 0.93548 | G | 0.06452 | 4 | 0.12903 |
| 2045 | S2_120691137 | 2 | 120691137 | 41 | G | A | G | 0.93548 | A | 0.06452 | 4 | 0.12903 |
| 2046 | S2_120691145 | 2 | 120691145 | 8 | C | T | C | 0.95161 | T | 0.04839 | 3 | 0.09677 |
| 2047 | S2_120838723 | 2 | 120838723 | 147578 | A | C | A | 0.82258 | C | 0.17742 | 7 | 0.22581 |
| 2048 | S2_120838793 | 2 | 120838793 | 70 | G | A | G | 0.51613 | A | 0.48387 | 20 | 0.64516 |
| 2049 | S2_120838843 | 2 | 120838843 | 50 | G | A | G | 0.82258 | A | 0.17742 | 7 | 0.22581 |
| 2050 | S2_120868146 | 2 | 120868146 | 29303 | C | T | C | 0.70968 | T | 0.29032 | 12 | 0.3871 |

| Site number | SNP name | Chromosome | Physical position | Physical distance from the previous SNP | Reference allele | Alternative allele | Major allele | Major allele frequency | Minor allele | Minor allele frequency | Number heterozygous | Heterozygosity |
|---|---|---|---|---|---|---|---|---|---|---|---|---|
| 2051 | S2_120868223 | 2 | 120868223 | 77 | C | A | C | 0.93548 | A | 0.06452 | 4 | 0.12903 |
| 2052 | S2_121317910 | 2 | 121317910 | 449687 | T | G | T | 0.8871 | G | 0.1129 | 7 | 0.22581 |
| 2053 | S2_121317943 | 2 | 121317943 | 33 | G | A | G | 0.95161 | A | 0.04839 | 3 | 0.09677 |
| 2054 | S2_121318068 | 2 | 121318068 | 125 | C | T | C | 0.77419 | T | 0.22581 | 10 | 0.32258 |
| 2055 | S2_121755505 | 2 | 121755505 | 437437 | G | T | T | 0.90323 | G | 0.09677 | 6 | 0.19355 |
| 2056 | S2_121810335 | 2 | 121810335 | 54830 | A | G | A | 0.91935 | G | 0.08065 | 5 | 0.16129 |
| 2057 | S2_121810418 | 2 | 121810418 | 83 | C | T | C | 0.83871 | T | 0.16129 | 10 | 0.32258 |
| 2058 | S2_121810477 | 2 | 121810477 | 59 | G | A | G | 0.87097 | A | 0.12903 | 6 | 0.19355 |
| 2059 | S2_121849642 | 2 | 121849642 | 39165 | C | A | C | 0.62903 | A | 0.37097 | 17 | 0.54839 |
| 2060 | S2_121849770 | 2 | 121849770 | 128 | A | G | G | 0.91935 | A | 0.08065 | 5 | 0.16129 |
| 2061 | S2_121849850 | 2 | 121849850 | 80 | C | T | C | 0.62903 | T | 0.37097 | 17 | 0.54839 |
| 2062 | S2_121981162 | 2 | 121981162 | 131312 | T | C | C | 0.87097 | T | 0.12903 | 8 | 0.25806 |
| 2063 | S2_121981188 | 2 | 121981188 | 26 | T | G | T | 0.69355 | G | 0.30645 | 13 | 0.41935 |
| 2064 | S2_121981400 | 2 | 121981400 | 212 | G | A | G | 0.91935 | A | 0.08065 | 3 | 0.09677 |
| 2065 | S2_122324995 | 2 | 122324995 | 343595 | G | T | G | 0.91935 | T | 0.08065 | 3 | 0.09677 |
| 2066 | S2_122546706 | 2 | 122546706 | 221711 | T | C | T | 0.90323 | C | 0.09677 | 4 | 0.12903 |
| 2067 | S2_122546745 | 2 | 122546745 | 39 | T | C | T | 0.62903 | C | 0.37097 | 13 | 0.41935 |
| 2068 | S2_122546747 | 2 | 122546747 | 2 | T | C | T | 0.62903 | C | 0.37097 | 13 | 0.41935 |
| 2069 | S2_122546786 | 2 | 122546786 | 39 | T | C | T | 0.79032 | C | 0.20968 | 9 | 0.29032 |
| 2070 | S2_122546817 | 2 | 122546817 | 31 | G | A | G | 0.90323 | A | 0.09677 | 4 | 0.12903 |
| 2071 | S2_122546823 | 2 | 122546823 | 6 | A | G | A | 0.90323 | G | 0.09677 | 4 | 0.12903 |
| 2072 | S2_122546853 | 2 | 122546853 | 30 | T | C | T | 0.62903 | C | 0.37097 | 13 | 0.41935 |
| 2073 | S2_123201959 | 2 | 123201959 | 655106 | G | A | G | 0.6129 | A | 0.3871 | 18 | 0.58065 |
| 2074 | S2_123201968 | 2 | 123201968 | 9 | A | G | A | 0.67742 | G | 0.32258 | 14 | 0.45161 |
| 2075 | S2_123202055 | 2 | 123202055 | 87 | C | T | C | 0.77419 | T | 0.22581 | 12 | 0.3871 |
| 2076 | S2_123309384 | 2 | 123309384 | 107329 | T | G | T | 0.95161 | G | 0.04839 | 3 | 0.09677 |
| 2077 | S2_123401598 | 2 | 123401598 | 92214 | G | A | G | 0.90323 | A | 0.09677 | 4 | 0.12903 |
| 2078 | S2_123401612 | 2 | 123401612 | 14 | C | T | C | 0.80645 | T | 0.19355 | 8 | 0.25806 |
| 2079 | S2_123401717 | 2 | 123401717 | 105 | G | A | G | 0.70968 | A | 0.29032 | 10 | 0.32258 |
| 2080 | S2_124578420 | 2 | 124578420 | 1176703 | T | C | T | 0.74194 | C | 0.25806 | 12 | 0.3871 |
| 2081 | S2_124578427 | 2 | 124578427 | 7 | T | C | C | 0.93548 | T | 0.06452 | 4 | 0.12903 |
| 2082 | S2_124578513 | 2 | 124578513 | 86 | G | C | G | 0.74194 | C | 0.25806 | 12 | 0.3871 |
| 2083 | S2_124578535 | 2 | 124578535 | 22 | A | G | A | 0.66129 | G | 0.33871 | 13 | 0.41935 |
| 2084 | S2_124578548 | 2 | 124578548 | 13 | T | C | C | 0.95161 | T | 0.04839 | 1 | 0.03226 |
| 2085 | S2_124578562 | 2 | 124578562 | 14 | T | G | T | 0.66129 | G | 0.33871 | 13 | 0.41935 |
| 2086 | S2_125494159 | 2 | 125494159 | 915597 | G | A | G | 0.83871 | A | 0.16129 | 6 | 0.19355 |
| 2087 | S2_125494217 | 2 | 125494217 | 58 | G | A | G | 0.93548 | A | 0.06452 | 4 | 0.12903 |
| 2088 | S2_125494246 | 2 | 125494246 | 29 | G | A | G | 0.79032 | A | 0.20968 | 13 | 0.41935 |
| 2089 | S2_126282640 | 2 | 126282640 | 788394 | A | T | A | 0.93548 | T | 0.06452 | 4 | 0.12903 |
| 2090 | S2_126282693 | 2 | 126282693 | 53 | G | A | G | 0.93548 | A | 0.06452 | 2 | 0.06452 |
| 2091 | S2_126282717 | 2 | 126282717 | 24 | G | A | G | 0.90323 | A | 0.09677 | 4 | 0.12903 |
| 2092 | S2_126550894 | 2 | 126550894 | 268177 | G | A | G | 0.95161 | A | 0.04839 | 3 | 0.09677 |
| 2093 | S2_126550990 | 2 | 126550990 | 96 | C | T | C | 0.95161 | T | 0.04839 | 3 | 0.09677 |
| 2094 | S2_126551032 | 2 | 126551032 | 42 | A | G | A | 0.59677 | G | 0.40323 | 17 | 0.54839 |
| 2095 | S2_126551059 | 2 | 126551059 | 27 | G | A | G | 0.90323 | A | 0.09677 | 6 | 0.19355 |
| 2096 | S2_126676302 | 2 | 126676302 | 125243 | A | G | G | 0.82258 | A | 0.17742 | 9 | 0.29032 |
| 2097 | S2_126676381 | 2 | 126676381 | 79 | C | T | C | 0.95161 | T | 0.04839 | 3 | 0.09677 |
| 2098 | S2_126676491 | 2 | 126676491 | 110 | C | T | C | 0.95161 | T | 0.04839 | 3 | 0.09677 |
| 2099 | S2_126735228 | 2 | 126735228 | 58737 | G | T | T | 0.90323 | G | 0.09677 | 6 | 0.19355 |
| 2100 | S2_126735268 | 2 | 126735268 | 40 | A | G | G | 0.85484 | A | 0.14516 | 9 | 0.29032 |
| 2101 | S2_126735434 | 2 | 126735434 | 166 | A | G | A | 0.80645 | G | 0.19355 | 12 | 0.3871 |
| 2102 | S2_126821632 | 2 | 126821632 | 86198 | A | T | T | 0.58065 | A | 0.41935 | 16 | 0.51613 |
| 2103 | S2_126821656 | 2 | 126821656 | 24 | A | G | A | 0.59677 | G | 0.40323 | 13 | 0.41935 |
| 2104 | S2_126821662 | 2 | 126821662 | 6 | G | A | G | 0.72581 | A | 0.27419 | 11 | 0.35484 |

| Site number | SNP name | Chromosome | Physical position | Physical distance from the previous SNP | Reference allele | Alternative allele | Major allele | Major allele frequency | Minor allele | Minor allele frequency | Number heterozygous | Heterozygosity |
|---|---|---|---|---|---|---|---|---|---|---|---|---|
| 2105 | S2_127684127 | 2 | 127684127 | 862465 | G | C | G | 0.77419 | C | 0.22581 | 12 | 0.3871 |
| 2106 | S2_128509166 | 2 | 128509166 | 825039 | A | T | A | 0.93548 | T | 0.06452 | 4 | 0.12903 |
| 2107 | S2_128509167 | 2 | 128509167 | 1 | T | A | T | 0.93548 | A | 0.06452 | 4 | 0.12903 |
| 2108 | S2_128928231 | 2 | 128928231 | 419064 | G | A | G | 0.93548 | A | 0.06452 | 4 | 0.12903 |
| 2109 | S2_128928232 | 2 | 128928232 | 1 | C | T | C | 0.8871 | T | 0.1129 | 7 | 0.22581 |
| 2110 | S2_129442690 | 2 | 129442690 | 514458 | G | A | G | 0.93548 | A | 0.06452 | 2 | 0.06452 |
| 2111 | S2_130373221 | 2 | 130373221 | 930531 | C | A | C | 0.85484 | A | 0.14516 | 9 | 0.29032 |
| 2112 | S2_130373241 | 2 | 130373241 | 20 | G | A | G | 0.83871 | A | 0.16129 | 8 | 0.25806 |
| 2113 | S2_130373308 | 2 | 130373308 | 67 | A | C | A | 0.85484 | C | 0.14516 | 9 | 0.29032 |
| 2114 | S2_130481471 | 2 | 130481471 | 108163 | C | G | C | 0.95161 | G | 0.04839 | 3 | 0.09677 |
| 2115 | S2_130514512 | 2 | 130514512 | 33041 | G | A | A | 0.6129 | G | 0.3871 | 16 | 0.51613 |
| 2116 | S2_130514542 | 2 | 130514542 | 30 | T | C | C | 0.8871 | T | 0.1129 | 5 | 0.16129 |
| 2117 | S2_130514554 | 2 | 130514554 | 12 | A | G | G | 0.8871 | A | 0.1129 | 5 | 0.16129 |
| 2118 | S2_130514559 | 2 | 130514559 | 5 | T | C | C | 0.8871 | T | 0.1129 | 5 | 0.16129 |
| 2119 | S2_130514567 | 2 | 130514567 | 8 | A | G | G | 0.8871 | A | 0.1129 | 5 | 0.16129 |
| 2120 | S2_130514600 | 2 | 130514600 | 33 | C | A | C | 0.8871 | A | 0.1129 | 5 | 0.16129 |
| 2121 | S2_130514601 | 2 | 130514601 | 1 | G | A | G | 0.90323 | A | 0.09677 | 6 | 0.19355 |
| 2122 | S2_130514606 | 2 | 130514606 | 5 | C | A | C | 0.8871 | A | 0.1129 | 5 | 0.16129 |
| 2123 | S2_130514618 | 2 | 130514618 | 12 | T | C | T | 0.72581 | C | 0.27419 | 11 | 0.35484 |
| 2124 | S2_130514619 | 2 | 130514619 | 1 | G | A | G | 0.72581 | A | 0.27419 | 11 | 0.35484 |
| 2125 | S2_130514661 | 2 | 130514661 | 42 | A | C | C | 0.6129 | A | 0.3871 | 16 | 0.51613 |
| 2126 | S2_130514677 | 2 | 130514677 | 16 | C | T | T | 0.6129 | C | 0.3871 | 16 | 0.51613 |
| 2127 | S2_130590966 | 2 | 130590966 | 76289 | A | C | C | 0.53226 | A | 0.46774 | 15 | 0.48387 |
| 2128 | S2_130591153 | 2 | 130591153 | 187 | A | G | A | 0.5 | G | 0.5 | 15 | 0.48387 |
| 2129 | S2_130948442 | 2 | 130948442 | 357289 | G | A | G | 0.87097 | A | 0.12903 | 8 | 0.25806 |
| 2130 | S2_131144221 | 2 | 131144221 | 195779 | C | T | C | 0.69355 | T | 0.30645 | 11 | 0.35484 |
| 2131 | S2_131144376 | 2 | 131144376 | 155 | G | A | G | 0.95161 | A | 0.04839 | 3 | 0.09677 |
| 2132 | S2_131144405 | 2 | 131144405 | 29 | T | A | T | 0.95161 | A | 0.04839 | 3 | 0.09677 |
| 2133 | S2_131144406 | 2 | 131144406 | 1 | A | C | A | 0.6129 | C | 0.3871 | 12 | 0.3871 |
| 2134 | S2_131170966 | 2 | 131170966 | 26560 | G | A | A | 0.64516 | G | 0.35484 | 12 | 0.3871 |
| 2135 | S2_131260825 | 2 | 131260825 | 89859 | T | C | C | 0.67742 | T | 0.32258 | 10 | 0.32258 |
| 2136 | S2_131260855 | 2 | 131260855 | 30 | T | C | C | 0.67742 | T | 0.32258 | 10 | 0.32258 |
| 2137 | S2_131260915 | 2 | 131260915 | 60 | A | G | G | 0.69355 | A | 0.30645 | 11 | 0.35484 |
| 2138 | S2_131260935 | 2 | 131260935 | 20 | T | C | C | 0.67742 | T | 0.32258 | 10 | 0.32258 |
| 2139 | S2_131260945 | 2 | 131260945 | 10 | A | C | C | 0.67742 | A | 0.32258 | 10 | 0.32258 |
| 2140 | S2_131260973 | 2 | 131260973 | 28 | G | C | C | 0.67742 | G | 0.32258 | 10 | 0.32258 |
| 2141 | S2_131260978 | 2 | 131260978 | 5 | T | C | C | 0.67742 | T | 0.32258 | 10 | 0.32258 |
| 2142 | S2_131584423 | 2 | 131584423 | 323445 | T | C | T | 0.70968 | C | 0.29032 | 12 | 0.3871 |
| 2143 | S2_131584560 | 2 | 131584560 | 137 | G | A | G | 0.90323 | A | 0.09677 | 6 | 0.19355 |
| 2144 | S2_131584564 | 2 | 131584564 | 4 | C | G | C | 0.95161 | G | 0.04839 | 1 | 0.03226 |
| 2145 | S2_131667747 | 2 | 131667747 | 83183 | T | C | C | 0.53226 | T | 0.46774 | 13 | 0.41935 |
| 2146 | S2_131667818 | 2 | 131667818 | 71 | C | T | T | 0.54839 | C | 0.45161 | 14 | 0.45161 |
| 2147 | S2_131667858 | 2 | 131667858 | 40 | T | C | C | 0.79032 | T | 0.20968 | 9 | 0.29032 |
| 2148 | S2_131667899 | 2 | 131667899 | 41 | G | A | G | 0.90323 | A | 0.09677 | 4 | 0.12903 |
| 2149 | S2_132142634 | 2 | 132142634 | 474735 | A | G | A | 0.90323 | G | 0.09677 | 6 | 0.19355 |
| 2150 | S2_132142715 | 2 | 132142715 | 81 | G | A | G | 0.67742 | A | 0.32258 | 12 | 0.3871 |
| 2151 | S2_132176762 | 2 | 132176762 | 34047 | A | G | A | 0.75806 | G | 0.24194 | 11 | 0.35484 |
| 2152 | S2_132176861 | 2 | 132176861 | 99 | C | T | C | 0.74194 | T | 0.25806 | 12 | 0.3871 |
| 2153 | S2_132341892 | 2 | 132341892 | 165031 | T | G | T | 0.56452 | G | 0.43548 | 15 | 0.48387 |
| 2154 | S2_132341899 | 2 | 132341899 | 7 | C | T | C | 0.56452 | T | 0.43548 | 15 | 0.48387 |
| 2155 | S2_132342093 | 2 | 132342093 | 194 | G | T | G | 0.95161 | T | 0.04839 | 3 | 0.09677 |
| 2156 | S2_132535153 | 2 | 132535153 | 193060 | A | G | A | 0.59677 | G | 0.40323 | 15 | 0.48387 |
| 2157 | S2_132535224 | 2 | 132535224 | 71 | T | A | T | 0.72581 | A | 0.27419 | 13 | 0.41935 |
| 2158 | S2_132831488 | 2 | 132831488 | 296264 | T | C | T | 0.8871 | C | 0.1129 | 7 | 0.22581 |

| Site number | SNP name | Chromosome | Physical position | Physical distance from the previous SNP | Reference allele | Alternative allele | Major allele | Major allele frequency | Minor allele | Minor allele frequency | Number heterozygous | Heterozygosity |
|---|---|---|---|---|---|---|---|---|---|---|---|---|
| 2159 | S2_132831558 | 2 | 132831558 | 70 | G | A | G | 0.87097 | A | 0.12903 | 8 | 0.25806 |
| 2160 | S2_132831578 | 2 | 132831578 | 20 | C | T | C | 0.87097 | T | 0.12903 | 8 | 0.25806 |
| 2161 | S2_134021214 | 2 | 134021214 | 1189636 | C | T | C | 0.91935 | T | 0.08065 | 5 | 0.16129 |
| 2162 | S2_134021280 | 2 | 134021280 | 66 | T | G | T | 0.93548 | G | 0.06452 | 4 | 0.12903 |
| 2163 | S2_134021298 | 2 | 134021298 | 18 | G | T | G | 0.51613 | T | 0.48387 | 14 | 0.45161 |
| 2164 | S2_134021325 | 2 | 134021325 | 27 | G | A | G | 0.51613 | A | 0.48387 | 14 | 0.45161 |
| 2165 | S2_134306819 | 2 | 134306819 | 285494 | C | T | C | 0.8871 | T | 0.1129 | 5 | 0.16129 |
| 2166 | S2_134306857 | 2 | 134306857 | 38 | G | A | G | 0.8871 | A | 0.1129 | 5 | 0.16129 |
| 2167 | S2_134306954 | 2 | 134306954 | 97 | C | T | C | 0.95161 | T | 0.04839 | 3 | 0.09677 |
| 2168 | S2_134438459 | 2 | 134438459 | 131505 | A | G | A | 0.91935 | G | 0.08065 | 5 | 0.16129 |
| 2169 | S2_134438534 | 2 | 134438534 | 75 | C | T | C | 0.93548 | T | 0.06452 | 4 | 0.12903 |
| 2170 | S2_134438599 | 2 | 134438599 | 65 | C | T | C | 0.80645 | T | 0.19355 | 12 | 0.3871 |
| 2171 | S2_134491607 | 2 | 134491607 | 53008 | G | A | G | 0.8871 | A | 0.1129 | 5 | 0.16129 |
| 2172 | S2_134920010 | 2 | 134920010 | 428403 | C | T | C | 0.82258 | T | 0.17742 | 11 | 0.35484 |
| 2173 | S2_134920078 | 2 | 134920078 | 68 | T | C | T | 0.79032 | C | 0.20968 | 9 | 0.29032 |
| 2174 | S2_134920108 | 2 | 134920108 | 30 | T | G | G | 0.74194 | T | 0.25806 | 12 | 0.3871 |
| 2175 | S2_134920112 | 2 | 134920112 | 4 | C | T | T | 0.66129 | C | 0.33871 | 17 | 0.54839 |
| 2176 | S2_134920233 | 2 | 134920233 | 121 | G | T | G | 0.74194 | T | 0.25806 | 12 | 0.3871 |
| 2177 | S2_135450278 | 2 | 135450278 | 530045 | G | A | G | 0.85484 | A | 0.14516 | 7 | 0.22581 |
| 2178 | S2_135450485 | 2 | 135450485 | 207 | C | T | C | 0.90323 | T | 0.09677 | 6 | 0.19355 |
| 2179 | S2_135728549 | 2 | 135728549 | 278064 | G | A | G | 0.95161 | A | 0.04839 | 3 | 0.09677 |
| 2180 | S2_136438651 | 2 | 136438651 | 710102 | G | A | G | 0.95161 | A | 0.04839 | 1 | 0.03226 |
| 2181 | S2_136438725 | 2 | 136438725 | 74 | C | T | T | 0.66129 | C | 0.33871 | 11 | 0.35484 |
| 2182 | S2_136474235 | 2 | 136474235 | 35510 | A | G | A | 0.95161 | G | 0.04839 | 1 | 0.03226 |
| 2183 | S3_127902 | 3 | 127902 | 0 | T | G | T | 0.95161 | G | 0.04839 | 3 | 0.09677 |
| 2184 | S3_127908 | 3 | 127908 | 6 | C | T | C | 0.87097 | T | 0.12903 | 8 | 0.25806 |
| 2185 | S3_127928 | 3 | 127928 | 20 | G | A | G | 0.95161 | A | 0.04839 | 3 | 0.09677 |
| 2186 | S3_181853 | 3 | 181853 | 53925 | T | C | C | 0.93548 | T | 0.06452 | 4 | 0.12903 |
| 2187 | S3_267801 | 3 | 267801 | 85948 | A | C | A | 0.93548 | C | 0.06452 | 4 | 0.12903 |
| 2188 | S3_267859 | 3 | 267859 | 58 | G | A | G | 0.72581 | A | 0.27419 | 17 | 0.54839 |
| 2189 | S3_267931 | 3 | 267931 | 72 | A | C | A | 0.87097 | C | 0.12903 | 8 | 0.25806 |
| 2190 | S3_267958 | 3 | 267958 | 27 | G | A | G | 0.85484 | A | 0.14516 | 9 | 0.29032 |
| 2191 | S3_267965 | 3 | 267965 | 7 | A | G | A | 0.90323 | G | 0.09677 | 6 | 0.19355 |
| 2192 | S3_267966 | 3 | 267966 | 1 | G | A | G | 0.8871 | A | 0.1129 | 7 | 0.22581 |
| 2193 | S3_267976 | 3 | 267976 | 10 | G | T | G | 0.8871 | T | 0.1129 | 7 | 0.22581 |
| 2194 | S3_456167 | 3 | 456167 | 188191 | A | G | A | 0.69355 | G | 0.30645 | 15 | 0.48387 |
| 2195 | S3_456226 | 3 | 456226 | 59 | T | C | T | 0.80645 | C | 0.19355 | 12 | 0.3871 |
| 2196 | S3_754483 | 3 | 754483 | 298257 | C | T | T | 0.74194 | C | 0.25806 | 10 | 0.32258 |
| 2197 | S3_1327114 | 3 | 1327114 | 572631 | G | C | C | 0.82258 | G | 0.17742 | 11 | 0.35484 |
| 2198 | S3_1410290 | 3 | 1410290 | 83176 | T | C | T | 0.77419 | C | 0.22581 | 10 | 0.32258 |
| 2199 | S3_2351477 | 3 | 2351477 | 941187 | T | C | T | 0.91935 | C | 0.08065 | 5 | 0.16129 |
| 2200 | S3_2351489 | 3 | 2351489 | 12 | T | C | C | 0.87097 | T | 0.12903 | 6 | 0.19355 |
| 2201 | S3_2840399 | 3 | 2840399 | 488910 | T | G | T | 0.70968 | G | 0.29032 | 12 | 0.3871 |
| 2202 | S3_2840428 | 3 | 2840428 | 29 | C | T | C | 0.95161 | T | 0.04839 | 3 | 0.09677 |
| 2203 | S3_2870370 | 3 | 2870370 | 29942 | G | A | G | 0.87097 | A | 0.12903 | 8 | 0.25806 |
| 2204 | S3_3076280 | 3 | 3076280 | 205910 | A | G | A | 0.77419 | G | 0.22581 | 12 | 0.3871 |
| 2205 | S3_3111521 | 3 | 3111521 | 35241 | A | G | A | 0.93548 | G | 0.06452 | 4 | 0.12903 |
| 2206 | S3_3111554 | 3 | 3111554 | 33 | G | C | G | 0.79032 | C | 0.20968 | 9 | 0.29032 |
| 2207 | S3_3111570 | 3 | 3111570 | 16 | G | A | G | 0.53226 | A | 0.46774 | 15 | 0.48387 |
| 2208 | S3_3111599 | 3 | 3111599 | 29 | G | A | G | 0.93548 | A | 0.06452 | 4 | 0.12903 |
| 2209 | S3_3111626 | 3 | 3111626 | 27 | T | C | T | 0.93548 | C | 0.06452 | 4 | 0.12903 |
| 2210 | S3_3111628 | 3 | 3111628 | 2 | A | G | A | 0.93548 | G | 0.06452 | 4 | 0.12903 |
| 2211 | S3_3111629 | 3 | 3111629 | 1 | C | G | C | 0.93548 | G | 0.06452 | 4 | 0.12903 |
| 2212 | S3_3111761 | 3 | 3111761 | 132 | G | A | G | 0.8871 | A | 0.1129 | 7 | 0.22581 |

| Site number | SNP name | Chromosome | Physical position | Physical distance from the previous SNP | Reference allele | Alternative allele | Major allele | Major allele frequency | Minor allele | Minor allele frequency | Number heterozygous | Heterozygosity |
|---|---|---|---|---|---|---|---|---|---|---|---|---|
| 2213 | S3_3325037 | 3 | 3325037 | 213276 | G | A | G | 0.77419 | A | 0.22581 | 10 | 0.32258 |
| 2214 | S3_3536340 | 3 | 3536340 | 211303 | G | A | G | 0.93548 | A | 0.06452 | 4 | 0.12903 |
| 2215 | S3_3629696 | 3 | 3629696 | 93356 | C | A | C | 0.95161 | A | 0.04839 | 3 | 0.09677 |
| 2216 | S3_3629850 | 3 | 3629850 | 154 | C | G | C | 0.53226 | G | 0.46774 | 15 | 0.48387 |
| 2217 | S3_3872498 | 3 | 3872498 | 242648 | C | T | C | 0.90323 | T | 0.09677 | 6 | 0.19355 |
| 2218 | S3_4064435 | 3 | 4064435 | 191937 | G | T | G | 0.87097 | T | 0.12903 | 4 | 0.12903 |
| 2219 | S3_4085523 | 3 | 4085523 | 21088 | C | T | T | 0.91935 | C | 0.08065 | 5 | 0.16129 |
| 2220 | S3_4085562 | 3 | 4085562 | 39 | C | T | C | 0.62903 | T | 0.37097 | 19 | 0.6129 |
| 2221 | S3_4131199 | 3 | 4131199 | 45637 | A | C | C | 0.91935 | A | 0.08065 | 5 | 0.16129 |
| 2222 | S3_4131351 | 3 | 4131351 | 152 | C | T | T | 0.56452 | C | 0.43548 | 17 | 0.54839 |
| 2223 | S3_4225803 | 3 | 4225803 | 94452 | G | A | G | 0.90323 | A | 0.09677 | 6 | 0.19355 |
| 2224 | S3_4225855 | 3 | 4225855 | 52 | G | A | G | 0.90323 | A | 0.09677 | 6 | 0.19355 |
| 2225 | S3_4367869 | 3 | 4367869 | 142014 | T | C | T | 0.85484 | C | 0.14516 | 7 | 0.22581 |
| 2226 | S3_4367874 | 3 | 4367874 | 5 | G | A | G | 0.8871 | A | 0.1129 | 7 | 0.22581 |
| 2227 | S3_4367890 | 3 | 4367890 | 16 | G | A | G | 0.90323 | A | 0.09677 | 6 | 0.19355 |
| 2228 | S3_4367989 | 3 | 4367989 | 99 | C | T | C | 0.90323 | T | 0.09677 | 6 | 0.19355 |
| 2229 | S3_4423311 | 3 | 4423311 | 55322 | G | C | C | 0.70968 | G | 0.29032 | 14 | 0.45161 |
| 2230 | S3_4423320 | 3 | 4423320 | 9 | T | C | T | 0.80645 | C | 0.19355 | 10 | 0.32258 |
| 2231 | S3_4529522 | 3 | 4529522 | 106202 | G | A | G | 0.56452 | A | 0.43548 | 15 | 0.48387 |
| 2232 | S3_4682765 | 3 | 4682765 | 153243 | A | G | G | 0.91935 | A | 0.08065 | 3 | 0.09677 |
| 2233 | S3_4682783 | 3 | 4682783 | 18 | G | T | G | 0.70968 | T | 0.29032 | 10 | 0.32258 |
| 2234 | S3_4682814 | 3 | 4682814 | 31 | T | C | C | 0.54839 | T | 0.45161 | 16 | 0.51613 |
| 2235 | S3_4713695 | 3 | 4713695 | 30881 | T | C | T | 0.72581 | C | 0.27419 | 9 | 0.29032 |
| 2236 | S3_4713822 | 3 | 4713822 | 127 | T | C | C | 0.74194 | T | 0.25806 | 10 | 0.32258 |
| 2237 | S3_4754956 | 3 | 4754956 | 41134 | G | A | G | 0.8871 | A | 0.1129 | 5 | 0.16129 |
| 2238 | S3_4755112 | 3 | 4755112 | 156 | C | G | C | 0.93548 | G | 0.06452 | 4 | 0.12903 |
| 2239 | S3_4809243 | 3 | 4809243 | 54131 | G | A | G | 0.90323 | A | 0.09677 | 4 | 0.12903 |
| 2240 | S3_4809267 | 3 | 4809267 | 24 | G | A | G | 0.91935 | A | 0.08065 | 3 | 0.09677 |
| 2241 | S3_4836937 | 3 | 4836937 | 27670 | G | A | G | 0.59677 | A | 0.40323 | 15 | 0.48387 |
| 2242 | S3_5176069 | 3 | 5176069 | 339132 | C | A | C | 0.6129 | A | 0.3871 | 12 | 0.3871 |
| 2243 | S3_5176243 | 3 | 5176243 | 174 | C | T | C | 0.5 | T | 0.5 | 13 | 0.41935 |
| 2244 | S3_5203292 | 3 | 5203292 | 27049 | G | A | G | 0.51613 | A | 0.48387 | 14 | 0.45161 |
| 2245 | S3_5305960 | 3 | 5305960 | 102668 | A | G | A | 0.93548 | G | 0.06452 | 4 | 0.12903 |
| 2246 | S3_5306037 | 3 | 5306037 | 77 | G | A | G | 0.85484 | A | 0.14516 | 7 | 0.22581 |
| 2247 | S3_5306047 | 3 | 5306047 | 10 | G | A | A | 0.69355 | G | 0.30645 | 15 | 0.48387 |
| 2248 | S3_5306115 | 3 | 5306115 | 68 | A | G | G | 0.67742 | A | 0.32258 | 16 | 0.51613 |
| 2249 | S3_5411281 | 3 | 5411281 | 105166 | G | A | A | 0.8871 | G | 0.1129 | 7 | 0.22581 |
| 2250 | S3_5411355 | 3 | 5411355 | 74 | G | A | G | 0.85484 | A | 0.14516 | 7 | 0.22581 |
| 2251 | S3_5411397 | 3 | 5411397 | 42 | T | C | C | 0.8871 | T | 0.1129 | 7 | 0.22581 |
| 2252 | S3_5414746 | 3 | 5414746 | 3349 | G | T | T | 0.58065 | G | 0.41935 | 18 | 0.58065 |
| 2253 | S3_5414754 | 3 | 5414754 | 8 | G | A | G | 0.93548 | A | 0.06452 | 4 | 0.12903 |
| 2254 | S3_5421915 | 3 | 5421915 | 7161 | G | A | G | 0.58065 | A | 0.41935 | 16 | 0.51613 |
| 2255 | S3_5421967 | 3 | 5421967 | 52 | T | G | T | 0.87097 | G | 0.12903 | 8 | 0.25806 |
| 2256 | S3_5422039 | 3 | 5422039 | 72 | G | A | G | 0.93548 | A | 0.06452 | 4 | 0.12903 |
| 2257 | S3_5422065 | 3 | 5422065 | 26 | G | C | G | 0.67742 | C | 0.32258 | 18 | 0.58065 |
| 2258 | S3_5422087 | 3 | 5422087 | 22 | C | T | C | 0.93548 | T | 0.06452 | 4 | 0.12903 |
| 2259 | S3_5422088 | 3 | 5422088 | 1 | G | A | G | 0.74194 | A | 0.25806 | 16 | 0.51613 |
| 2260 | S3_5422103 | 3 | 5422103 | 15 | G | A | G | 0.91935 | A | 0.08065 | 5 | 0.16129 |
| 2261 | S3_5422118 | 3 | 5422118 | 15 | G | A | G | 0.93548 | A | 0.06452 | 4 | 0.12903 |
| 2262 | S3_5563303 | 3 | 5563303 | 141185 | C | T | C | 0.91935 | T | 0.08065 | 5 | 0.16129 |
| 2263 | S3_6169419 | 3 | 6169419 | 606116 | C | T | C | 0.91935 | T | 0.08065 | 3 | 0.09677 |
| 2264 | S3_6169556 | 3 | 6169556 | 137 | T | C | T | 0.85484 | C | 0.14516 | 9 | 0.29032 |
| 2265 | S3_6522390 | 3 | 6522390 | 352834 | A | G | A | 0.90323 | G | 0.09677 | 6 | 0.19355 |
| 2266 | S3_6522485 | 3 | 6522485 | 95 | T | C | T | 0.95161 | C | 0.04839 | 3 | 0.09677 |

| Site number | SNP name | Chromosome | Physical position | Physical distance from the previous SNP | Reference allele | Alternative allele | Major allele | Major allele frequency | Minor allele | Minor allele frequency | Number heterozygous | Heterozygosity |
|---|---|---|---|---|---|---|---|---|---|---|---|---|
| 2267 | S3_6957674 | 3 | 6957674 | 435189 | A | G | A | 0.93548 | G | 0.06452 | 4 | 0.12903 |
| 2268 | S3_7185537 | 3 | 7185537 | 227863 | T | C | C | 0.75806 | T | 0.24194 | 11 | 0.35484 |
| 2269 | S3_7185621 | 3 | 7185621 | 84 | A | G | G | 0.75806 | A | 0.24194 | 11 | 0.35484 |
| 2270 | S3_7185648 | 3 | 7185648 | 27 | G | A | G | 0.87097 | A | 0.12903 | 6 | 0.19355 |
| 2271 | S3_7526783 | 3 | 7526783 | 341135 | A | G | A | 0.91935 | G | 0.08065 | 5 | 0.16129 |
| 2272 | S3_7729418 | 3 | 7729418 | 202635 | C | A | C | 0.93548 | A | 0.06452 | 4 | 0.12903 |
| 2273 | S3_8080548 | 3 | 8080548 | 351130 | A | T | A | 0.53226 | T | 0.46774 | 13 | 0.41935 |
| 2274 | S3_8080571 | 3 | 8080571 | 23 | G | A | G | 0.95161 | A | 0.04839 | 3 | 0.09677 |
| 2275 | S3_8080573 | 3 | 8080573 | 2 | T | C | T | 0.58065 | C | 0.41935 | 10 | 0.32258 |
| 2276 | S3_8168952 | 3 | 8168952 | 88379 | G | A | A | 0.62903 | G | 0.37097 | 13 | 0.41935 |
| 2277 | S3_8169016 | 3 | 8169016 | 64 | C | T | C | 0.87097 | T | 0.12903 | 6 | 0.19355 |
| 2278 | S3_8361873 | 3 | 8361873 | 192857 | A | G | A | 0.90323 | G | 0.09677 | 4 | 0.12903 |
| 2279 | S3_8381096 | 3 | 8381096 | 19223 | C | A | C | 0.69355 | A | 0.30645 | 11 | 0.35484 |
| 2280 | S3_8381157 | 3 | 8381157 | 61 | A | C | C | 0.59677 | A | 0.40323 | 15 | 0.48387 |
| 2281 | S3_8407135 | 3 | 8407135 | 25978 | C | T | C | 0.66129 | T | 0.33871 | 13 | 0.41935 |
| 2282 | S3_8407143 | 3 | 8407143 | 8 | C | T | T | 0.72581 | C | 0.27419 | 11 | 0.35484 |
| 2283 | S3_8407150 | 3 | 8407150 | 7 | C | T | T | 0.64516 | C | 0.35484 | 14 | 0.45161 |
| 2284 | S3_8574105 | 3 | 8574105 | 166955 | T | C | C | 0.77419 | T | 0.22581 | 10 | 0.32258 |
| 2285 | S3_8574114 | 3 | 8574114 | 9 | G | A | A | 0.51613 | G | 0.48387 | 14 | 0.45161 |
| 2286 | S3_8829627 | 3 | 8829627 | 255513 | C | T | C | 0.90323 | T | 0.09677 | 6 | 0.19355 |
| 2287 | S3_8829650 | 3 | 8829650 | 23 | T | C | T | 0.70968 | C | 0.29032 | 10 | 0.32258 |
| 2288 | S3_8829825 | 3 | 8829825 | 175 | G | A | G | 0.74194 | A | 0.25806 | 8 | 0.25806 |
| 2289 | S3_8876250 | 3 | 8876250 | 46425 | G | A | G | 0.72581 | A | 0.27419 | 15 | 0.48387 |
| 2290 | S3_8876311 | 3 | 8876311 | 61 | C | T | C | 0.80645 | T | 0.19355 | 10 | 0.32258 |
| 2291 | S3_8876344 | 3 | 8876344 | 33 | A | G | A | 0.62903 | G | 0.37097 | 19 | 0.6129 |
| 2292 | S3_8876379 | 3 | 8876379 | 35 | C | T | C | 0.8871 | T | 0.1129 | 5 | 0.16129 |
| 2293 | S3_8876386 | 3 | 8876386 | 7 | C | T | C | 0.72581 | T | 0.27419 | 15 | 0.48387 |
| 2294 | S3_8876420 | 3 | 8876420 | 34 | C | T | C | 0.93548 | T | 0.06452 | 2 | 0.06452 |
| 2295 | S3_8876421 | 3 | 8876421 | 1 | G | A | G | 0.72581 | A | 0.27419 | 15 | 0.48387 |
| 2296 | S3_8941966 | 3 | 8941966 | 65545 | A | G | G | 0.72581 | A | 0.27419 | 15 | 0.48387 |
| 2297 | S3_8942089 | 3 | 8942089 | 123 | G | A | G | 0.56452 | A | 0.43548 | 13 | 0.41935 |
| 2298 | S3_9096662 | 3 | 9096662 | 154573 | G | A | A | 0.74194 | G | 0.25806 | 10 | 0.32258 |
| 2299 | S3_9547009 | 3 | 9547009 | 450347 | C | T | C | 0.83871 | T | 0.16129 | 8 | 0.25806 |
| 2300 | S3_9547079 | 3 | 9547079 | 70 | G | A | G | 0.83871 | A | 0.16129 | 8 | 0.25806 |
| 2301 | S3_9547087 | 3 | 9547087 | 8 | G | T | T | 0.62903 | G | 0.37097 | 17 | 0.54839 |
| 2302 | S3_9669336 | 3 | 9669336 | 122249 | C | T | T | 0.75806 | C | 0.24194 | 11 | 0.35484 |
| 2303 | S3_9669404 | 3 | 9669404 | 68 | G | C | G | 0.83871 | C | 0.16129 | 8 | 0.25806 |
| 2304 | S3_9669454 | 3 | 9669454 | 50 | C | G | G | 0.93548 | C | 0.06452 | 4 | 0.12903 |
| 2305 | S3_9669481 | 3 | 9669481 | 27 | C | G | C | 0.8871 | G | 0.1129 | 5 | 0.16129 |
| 2306 | S3_9799314 | 3 | 9799314 | 129833 | T | G | T | 0.87097 | G | 0.12903 | 6 | 0.19355 |
| 2307 | S3_10189934 | 3 | 10189934 | 390620 | G | A | G | 0.74194 | A | 0.25806 | 12 | 0.3871 |
| 2308 | S3_10190054 | 3 | 10190054 | 120 | G | A | G | 0.54839 | A | 0.45161 | 12 | 0.3871 |
| 2309 | S3_10239337 | 3 | 10239337 | 49283 | A | C | C | 0.91935 | A | 0.08065 | 3 | 0.09677 |
| 2310 | S3_10420804 | 3 | 10420804 | 181467 | A | G | A | 0.69355 | G | 0.30645 | 13 | 0.41935 |
| 2311 | S3_10503065 | 3 | 10503065 | 82261 | G | A | G | 0.59677 | A | 0.40323 | 17 | 0.54839 |
| 2312 | S3_10503119 | 3 | 10503119 | 54 | A | T | A | 0.8871 | T | 0.1129 | 5 | 0.16129 |
| 2313 | S3_10789647 | 3 | 10789647 | 286528 | A | G | A | 0.82258 | G | 0.17742 | 11 | 0.35484 |
| 2314 | S3_10858570 | 3 | 10858570 | 68923 | G | C | G | 0.74194 | C | 0.25806 | 14 | 0.45161 |
| 2315 | S3_10858741 | 3 | 10858741 | 171 | G | A | G | 0.66129 | A | 0.33871 | 13 | 0.41935 |
| 2316 | S3_10865746 | 3 | 10865746 | 7005 | A | G | A | 0.72581 | G | 0.27419 | 15 | 0.48387 |
| 2317 | S3_11206541 | 3 | 11206541 | 340795 | T | C | C | 0.90323 | T | 0.09677 | 6 | 0.19355 |
| 2318 | S3_11232264 | 3 | 11232264 | 25723 | G | C | G | 0.95161 | C | 0.04839 | 3 | 0.09677 |
| 2319 | S3_11232289 | 3 | 11232289 | 25 | G | C | G | 0.95161 | C | 0.04839 | 3 | 0.09677 |
| 2320 | S3_11282986 | 3 | 11282986 | 50697 | A | G | A | 0.67742 | G | 0.32258 | 14 | 0.45161 |

| Site number | SNP name | Chromosome | Physical position | Physical distance from the previous SNP | Reference allele | Alternative allele | Major allele | Major allele frequency | Minor allele | Minor allele frequency | Number heterozygous | Heterozygosity |
|---|---|---|---|---|---|---|---|---|---|---|---|---|
| 2321 | S3_11283011 | 3 | 11283011 | 25 | C | T | C | 0.67742 | T | 0.32258 | 14 | 0.45161 |
| 2322 | S3_11283125 | 3 | 11283125 | 114 | T | C | T | 0.64516 | C | 0.35484 | 16 | 0.51613 |
| 2323 | S3_11626074 | 3 | 11626074 | 342949 | C | T | T | 0.51613 | C | 0.48387 | 18 | 0.58065 |
| 2324 | S3_11626087 | 3 | 11626087 | 13 | C | T | T | 0.51613 | C | 0.48387 | 18 | 0.58065 |
| 2325 | S3_11626111 | 3 | 11626111 | 24 | T | C | T | 0.54839 | C | 0.45161 | 18 | 0.58065 |
| 2326 | S3_11654958 | 3 | 11654958 | 28847 | T | C | C | 0.72581 | T | 0.27419 | 15 | 0.48387 |
| 2327 | S3_11655142 | 3 | 11655142 | 184 | C | T | C | 0.90323 | T | 0.09677 | 6 | 0.19355 |
| 2328 | S3_11765691 | 3 | 11765691 | 110549 | C | G | C | 0.91935 | G | 0.08065 | 5 | 0.16129 |
| 2329 | S3_11765725 | 3 | 11765725 | 34 | C | T | C | 0.87097 | T | 0.12903 | 8 | 0.25806 |
| 2330 | S3_11765792 | 3 | 11765792 | 67 | T | C | C | 0.77419 | T | 0.22581 | 12 | 0.3871 |
| 2331 | S3_11765797 | 3 | 11765797 | 5 | T | C | C | 0.77419 | T | 0.22581 | 12 | 0.3871 |
| 2332 | S3_11842317 | 3 | 11842317 | 76520 | A | C | A | 0.82258 | C | 0.17742 | 11 | 0.35484 |
| 2333 | S3_11842362 | 3 | 11842362 | 45 | G | A | G | 0.82258 | A | 0.17742 | 9 | 0.29032 |
| 2334 | S3_11842370 | 3 | 11842370 | 8 | A | T | A | 0.74194 | T | 0.25806 | 14 | 0.45161 |
| 2335 | S3_11842379 | 3 | 11842379 | 9 | C | G | C | 0.90323 | G | 0.09677 | 6 | 0.19355 |
| 2336 | S3_11842400 | 3 | 11842400 | 21 | G | T | G | 0.91935 | T | 0.08065 | 5 | 0.16129 |
| 2337 | S3_11842424 | 3 | 11842424 | 24 | C | T | C | 0.72581 | T | 0.27419 | 11 | 0.35484 |
| 2338 | S3_11842540 | 3 | 11842540 | 116 | T | G | G | 0.79032 | T | 0.20968 | 9 | 0.29032 |
| 2339 | S3_12060662 | 3 | 12060662 | 218122 | A | G | A | 0.82258 | G | 0.17742 | 7 | 0.22581 |
| 2340 | S3_12060675 | 3 | 12060675 | 13 | A | G | A | 0.82258 | G | 0.17742 | 7 | 0.22581 |
| 2341 | S3_12060791 | 3 | 12060791 | 116 | C | T | C | 0.8871 | T | 0.1129 | 5 | 0.16129 |
| 2342 | S3_12060823 | 3 | 12060823 | 32 | T | C | T | 0.77419 | C | 0.22581 | 12 | 0.3871 |
| 2343 | S3_12139285 | 3 | 12139285 | 78462 | G | A | G | 0.74194 | A | 0.25806 | 14 | 0.45161 |
| 2344 | S3_12139286 | 3 | 12139286 | 1 | C | G | C | 0.74194 | G | 0.25806 | 14 | 0.45161 |
| 2345 | S3_12139419 | 3 | 12139419 | 133 | G | A | G | 0.66129 | A | 0.33871 | 19 | 0.6129 |
| 2346 | S3_12338426 | 3 | 12338426 | 199007 | A | G | A | 0.87097 | G | 0.12903 | 8 | 0.25806 |
| 2347 | S3_12338446 | 3 | 12338446 | 20 | A | G | A | 0.90323 | G | 0.09677 | 6 | 0.19355 |
| 2348 | S3_12338496 | 3 | 12338496 | 50 | G | A | G | 0.77419 | A | 0.22581 | 14 | 0.45161 |
| 2349 | S3_12541608 | 3 | 12541608 | 203112 | T | A | T | 0.85484 | A | 0.14516 | 9 | 0.29032 |
| 2350 | S3_12997347 | 3 | 12997347 | 455739 | C | T | C | 0.93548 | T | 0.06452 | 4 | 0.12903 |
| 2351 | S3_12997356 | 3 | 12997356 | 9 | G | A | A | 0.66129 | G | 0.33871 | 19 | 0.6129 |
| 2352 | S3_12997476 | 3 | 12997476 | 120 | T | C | C | 0.66129 | T | 0.33871 | 19 | 0.6129 |
| 2353 | S3_12997532 | 3 | 12997532 | 56 | G | A | A | 0.66129 | G | 0.33871 | 19 | 0.6129 |
| 2354 | S3_13109264 | 3 | 13109264 | 111732 | C | T | C | 0.95161 | T | 0.04839 | 3 | 0.09677 |
| 2355 | S3_13109320 | 3 | 13109320 | 56 | G | A | G | 0.91935 | A | 0.08065 | 5 | 0.16129 |
| 2356 | S3_13305440 | 3 | 13305440 | 196120 | A | G | G | 0.58065 | A | 0.41935 | 16 | 0.51613 |
| 2357 | S3_13305478 | 3 | 13305478 | 38 | A | T | A | 0.77419 | T | 0.22581 | 12 | 0.3871 |
| 2358 | S3_13398947 | 3 | 13398947 | 93469 | T | C | T | 0.90323 | C | 0.09677 | 6 | 0.19355 |
| 2359 | S3_13443692 | 3 | 13443692 | 44745 | A | G | G | 0.85484 | A | 0.14516 | 9 | 0.29032 |
| 2360 | S3_13443738 | 3 | 13443738 | 46 | T | C | T | 0.95161 | C | 0.04839 | 3 | 0.09677 |
| 2361 | S3_13443866 | 3 | 13443866 | 128 | C | A | C | 0.8871 | A | 0.1129 | 7 | 0.22581 |
| 2362 | S3_13443867 | 3 | 13443867 | 1 | G | A | G | 0.56452 | A | 0.43548 | 19 | 0.6129 |
| 2363 | S3_13638069 | 3 | 13638069 | 194202 | G | A | G | 0.87097 | A | 0.12903 | 6 | 0.19355 |
| 2364 | S3_13720417 | 3 | 13720417 | 82348 | G | A | G | 0.91935 | A | 0.08065 | 5 | 0.16129 |
| 2365 | S3_13720432 | 3 | 13720432 | 15 | C | T | C | 0.6129 | T | 0.3871 | 16 | 0.51613 |
| 2366 | S3_14065651 | 3 | 14065651 | 345219 | C | T | C | 0.87097 | T | 0.12903 | 8 | 0.25806 |
| 2367 | S3_14381856 | 3 | 14381856 | 316205 | G | A | G | 0.79032 | A | 0.20968 | 11 | 0.35484 |
| 2368 | S3_14384483 | 3 | 14384483 | 2627 | G | A | G | 0.70968 | A | 0.29032 | 14 | 0.45161 |
| 2369 | S3_14436612 | 3 | 14436612 | 52129 | G | A | G | 0.91935 | A | 0.08065 | 5 | 0.16129 |
| 2370 | S3_14510320 | 3 | 14510320 | 73708 | G | A | G | 0.93548 | A | 0.06452 | 4 | 0.12903 |
| 2371 | S3_14510366 | 3 | 14510366 | 46 | C | T | C | 0.75806 | T | 0.24194 | 15 | 0.48387 |
| 2372 | S3_14510379 | 3 | 14510379 | 13 | C | T | C | 0.93548 | T | 0.06452 | 4 | 0.12903 |
| 2373 | S3_14510446 | 3 | 14510446 | 67 | C | T | C | 0.91935 | T | 0.08065 | 5 | 0.16129 |
| 2374 | S3_14510470 | 3 | 14510470 | 24 | C | T | C | 0.93548 | T | 0.06452 | 4 | 0.12903 |

| Site number | SNP name | Chromosome | Physical position | Physical distance from the previous SNP | Reference allele | Alternative allele | Major allele | Major allele frequency | Minor allele | Minor allele frequency | Number heterozygous | Heterozygosity |
|---|---|---|---|---|---|---|---|---|---|---|---|---|
| 2375 | S3_14763774 | 3 | 14763774 | 253304 | A | G | A | 0.93548 | G | 0.06452 | 4 | 0.12903 |
| 2376 | S3_14763933 | 3 | 14763933 | 159 | A | G | A | 0.80645 | G | 0.19355 | 12 | 0.3871 |
| 2377 | S3_14984867 | 3 | 14984867 | 220934 | T | A | T | 0.80645 | A | 0.19355 | 10 | 0.32258 |
| 2378 | S3_15179287 | 3 | 15179287 | 194420 | A | G | A | 0.95161 | G | 0.04839 | 3 | 0.09677 |
| 2379 | S3_15179330 | 3 | 15179330 | 43 | C | T | C | 0.80645 | T | 0.19355 | 8 | 0.25806 |
| 2380 | S3_15179387 | 3 | 15179387 | 57 | G | A | G | 0.95161 | A | 0.04839 | 3 | 0.09677 |
| 2381 | S3_15179460 | 3 | 15179460 | 73 | C | G | C | 0.83871 | G | 0.16129 | 10 | 0.32258 |
| 2382 | S3_15227308 | 3 | 15227308 | 47848 | T | C | T | 0.75806 | C | 0.24194 | 11 | 0.35484 |
| 2383 | S3_15227345 | 3 | 15227345 | 37 | C | T | C | 0.90323 | T | 0.09677 | 6 | 0.19355 |
| 2384 | S3_15227464 | 3 | 15227464 | 119 | G | A | G | 0.90323 | A | 0.09677 | 6 | 0.19355 |
| 2385 | S3_15460177 | 3 | 15460177 | 232713 | G | C | G | 0.82258 | C | 0.17742 | 9 | 0.29032 |
| 2386 | S3_15834360 | 3 | 15834360 | 374183 | C | T | C | 0.67742 | T | 0.32258 | 12 | 0.3871 |
| 2387 | S3_15834471 | 3 | 15834471 | 111 | C | T | C | 0.8871 | T | 0.1129 | 5 | 0.16129 |
| 2388 | S3_16163287 | 3 | 16163287 | 328816 | T | C | T | 0.66129 | C | 0.33871 | 13 | 0.41935 |
| 2389 | S3_16399655 | 3 | 16399655 | 236368 | G | C | G | 0.95161 | C | 0.04839 | 3 | 0.09677 |
| 2390 | S3_16399663 | 3 | 16399663 | 8 | G | A | G | 0.79032 | A | 0.20968 | 11 | 0.35484 |
| 2391 | S3_16399721 | 3 | 16399721 | 58 | G | A | G | 0.95161 | A | 0.04839 | 3 | 0.09677 |
| 2392 | S3_16399724 | 3 | 16399724 | 3 | A | G | G | 0.70968 | A | 0.29032 | 14 | 0.45161 |
| 2393 | S3_16399761 | 3 | 16399761 | 37 | A | G | A | 0.80645 | G | 0.19355 | 10 | 0.32258 |
| 2394 | S3_16430617 | 3 | 16430617 | 30856 | G | A | G | 0.95161 | A | 0.04839 | 3 | 0.09677 |
| 2395 | S3_16430682 | 3 | 16430682 | 65 | G | A | G | 0.59677 | A | 0.40323 | 15 | 0.48387 |
| 2396 | S3_17384187 | 3 | 17384187 | 953505 | C | T | C | 0.69355 | T | 0.30645 | 13 | 0.41935 |
| 2397 | S3_17493877 | 3 | 17493877 | 109690 | T | C | T | 0.79032 | C | 0.20968 | 11 | 0.35484 |
| 2398 | S3_17562015 | 3 | 17562015 | 68138 | T | A | T | 0.66129 | A | 0.33871 | 17 | 0.54839 |
| 2399 | S3_17562088 | 3 | 17562088 | 73 | C | T | C | 0.74194 | T | 0.25806 | 14 | 0.45161 |
| 2400 | S3_17562098 | 3 | 17562098 | 10 | C | T | C | 0.66129 | T | 0.33871 | 17 | 0.54839 |
| 2401 | S3_18218215 | 3 | 18218215 | 656117 | T | G | T | 0.93548 | G | 0.06452 | 4 | 0.12903 |
| 2402 | S3_18218321 | 3 | 18218321 | 106 | A | G | A | 0.95161 | G | 0.04839 | 3 | 0.09677 |
| 2403 | S3_18448814 | 3 | 18448814 | 230493 | G | A | G | 0.74194 | A | 0.25806 | 16 | 0.51613 |
| 2404 | S3_18448914 | 3 | 18448914 | 100 | T | G | T | 0.87097 | G | 0.12903 | 8 | 0.25806 |
| 2405 | S3_18511498 | 3 | 18511498 | 62584 | C | T | C | 0.67742 | T | 0.32258 | 16 | 0.51613 |
| 2406 | S3_18511593 | 3 | 18511593 | 95 | C | T | C | 0.67742 | T | 0.32258 | 16 | 0.51613 |
| 2407 | S3_19052573 | 3 | 19052573 | 540980 | G | C | C | 0.62903 | G | 0.37097 | 11 | 0.35484 |
| 2408 | S3_19664839 | 3 | 19664839 | 612266 | T | C | T | 0.75806 | C | 0.24194 | 15 | 0.48387 |
| 2409 | S3_19665015 | 3 | 19665015 | 176 | T | C | C | 0.91935 | T | 0.08065 | 3 | 0.09677 |
| 2410 | S3_19665050 | 3 | 19665050 | 35 | T | C | C | 0.91935 | T | 0.08065 | 3 | 0.09677 |
| 2411 | S3_19665072 | 3 | 19665072 | 22 | A | G | G | 0.91935 | A | 0.08065 | 3 | 0.09677 |
| 2412 | S3_19713450 | 3 | 19713450 | 48378 | C | T | C | 0.95161 | T | 0.04839 | 3 | 0.09677 |
| 2413 | S3_19934883 | 3 | 19934883 | 221433 | T | C | T | 0.77419 | C | 0.22581 | 10 | 0.32258 |
| 2414 | S3_19934892 | 3 | 19934892 | 9 | A | G | A | 0.83871 | G | 0.16129 | 10 | 0.32258 |
| 2415 | S3_19934905 | 3 | 19934905 | 13 | A | G | A | 0.79032 | G | 0.20968 | 9 | 0.29032 |
| 2416 | S3_19934990 | 3 | 19934990 | 85 | C | T | T | 0.80645 | C | 0.19355 | 10 | 0.32258 |
| 2417 | S3_19935018 | 3 | 19935018 | 28 | C | T | C | 0.79032 | T | 0.20968 | 9 | 0.29032 |
| 2418 | S3_19935064 | 3 | 19935064 | 46 | C | T | C | 0.83871 | T | 0.16129 | 10 | 0.32258 |
| 2419 | S3_19935100 | 3 | 19935100 | 36 | C | A | C | 0.83871 | A | 0.16129 | 10 | 0.32258 |
| 2420 | S3_20051299 | 3 | 20051299 | 116199 | C | G | G | 0.91935 | C | 0.08065 | 5 | 0.16129 |
| 2421 | S3_20051354 | 3 | 20051354 | 55 | G | A | G | 0.90323 | A | 0.09677 | 4 | 0.12903 |
| 2422 | S3_20267892 | 3 | 20267892 | 216538 | A | G | A | 0.66129 | G | 0.33871 | 15 | 0.48387 |
| 2423 | S3_20518737 | 3 | 20518737 | 250845 | G | A | G | 0.77419 | A | 0.22581 | 10 | 0.32258 |
| 2424 | S3_20518761 | 3 | 20518761 | 24 | C | G | G | 0.70968 | C | 0.29032 | 14 | 0.45161 |
| 2425 | S3_20619832 | 3 | 20619832 | 101071 | A | G | A | 0.69355 | G | 0.30645 | 15 | 0.48387 |
| 2426 | S3_20693640 | 3 | 20693640 | 73808 | C | G | C | 0.8871 | G | 0.1129 | 7 | 0.22581 |
| 2427 | S3_20816654 | 3 | 20816654 | 123014 | C | T | C | 0.74194 | T | 0.25806 | 12 | 0.3871 |
| 2428 | S3_20874280 | 3 | 20874280 | 57626 | C | T | C | 0.87097 | T | 0.12903 | 8 | 0.25806 |

| Site number | SNP name | Chromosome | Physical position | Physical distance from the previous SNP | Reference allele | Alternative allele | Major allele | Major allele frequency | Minor allele | Minor allele frequency | Number heterozygous | Heterozygosity |
|---|---|---|---|---|---|---|---|---|---|---|---|---|
| 2429 | S3_21254039 | 3 | 21254039 | 379759 | C | T | C | 0.95161 | T | 0.04839 | 3 | 0.09677 |
| 2430 | S3_21304420 | 3 | 21304420 | 50381 | C | T | C | 0.85484 | T | 0.14516 | 9 | 0.29032 |
| 2431 | S3_21304439 | 3 | 21304439 | 19 | G | T | G | 0.95161 | T | 0.04839 | 3 | 0.09677 |
| 2432 | S3_21428079 | 3 | 21428079 | 123640 | C | T | C | 0.91935 | T | 0.08065 | 3 | 0.09677 |
| 2433 | S3_21428121 | 3 | 21428121 | 42 | C | T | C | 0.87097 | T | 0.12903 | 6 | 0.19355 |
| 2434 | S3_21435291 | 3 | 21435291 | 7170 | A | C | A | 0.90323 | C | 0.09677 | 4 | 0.12903 |
| 2435 | S3_21435341 | 3 | 21435341 | 50 | A | G | A | 0.90323 | G | 0.09677 | 4 | 0.12903 |
| 2436 | S3_21435374 | 3 | 21435374 | 33 | C | T | C | 0.74194 | T | 0.25806 | 14 | 0.45161 |
| 2437 | S3_21435375 | 3 | 21435375 | 1 | T | G | G | 0.93548 | T | 0.06452 | 4 | 0.12903 |
| 2438 | S3_21435399 | 3 | 21435399 | 24 | C | G | C | 0.77419 | G | 0.22581 | 12 | 0.3871 |
| 2439 | S3_21435481 | 3 | 21435481 | 82 | A | G | G | 0.72581 | A | 0.27419 | 13 | 0.41935 |
| 2440 | S3_21435482 | 3 | 21435482 | 1 | C | T | C | 0.75806 | T | 0.24194 | 11 | 0.35484 |
| 2441 | S3_22262152 | 3 | 22262152 | 826670 | T | G | T | 0.95161 | G | 0.04839 | 3 | 0.09677 |
| 2442 | S3_22404854 | 3 | 22404854 | 142702 | C | T | C | 0.77419 | T | 0.22581 | 10 | 0.32258 |
| 2443 | S3_22467708 | 3 | 22467708 | 62854 | G | A | G | 0.64516 | A | 0.35484 | 14 | 0.45161 |
| 2444 | S3_23070779 | 3 | 23070779 | 603071 | C | T | C | 0.8871 | T | 0.1129 | 5 | 0.16129 |
| 2445 | S3_23070824 | 3 | 23070824 | 45 | C | T | T | 0.79032 | C | 0.20968 | 13 | 0.41935 |
| 2446 | S3_23124186 | 3 | 23124186 | 53362 | T | C | T | 0.83871 | C | 0.16129 | 10 | 0.32258 |
| 2447 | S3_23124189 | 3 | 23124189 | 3 | C | T | C | 0.87097 | T | 0.12903 | 8 | 0.25806 |
| 2448 | S3_23135508 | 3 | 23135508 | 11319 | A | G | A | 0.90323 | G | 0.09677 | 6 | 0.19355 |
| 2449 | S3_23135547 | 3 | 23135547 | 39 | T | C | T | 0.53226 | C | 0.46774 | 19 | 0.6129 |
| 2450 | S3_23135555 | 3 | 23135555 | 8 | C | T | C | 0.91935 | T | 0.08065 | 5 | 0.16129 |
| 2451 | S3_23210748 | 3 | 23210748 | 75193 | A | G | A | 0.64516 | G | 0.35484 | 18 | 0.58065 |
| 2452 | S3_23210801 | 3 | 23210801 | 53 | A | G | A | 0.66129 | G | 0.33871 | 17 | 0.54839 |
| 2453 | S3_23210811 | 3 | 23210811 | 10 | G | A | G | 0.90323 | A | 0.09677 | 6 | 0.19355 |
| 2454 | S3_23210826 | 3 | 23210826 | 15 | T | C | T | 0.66129 | C | 0.33871 | 17 | 0.54839 |
| 2455 | S3_23224522 | 3 | 23224522 | 13696 | C | T | C | 0.70968 | T | 0.29032 | 12 | 0.3871 |
| 2456 | S3_23273473 | 3 | 23273473 | 48951 | A | C | A | 0.87097 | C | 0.12903 | 6 | 0.19355 |
| 2457 | S3_24586958 | 3 | 24586958 | 1313485 | G | A | G | 0.93548 | A | 0.06452 | 4 | 0.12903 |
| 2458 | S3_24840890 | 3 | 24840890 | 253932 | G | A | A | 0.8871 | G | 0.1129 | 5 | 0.16129 |
| 2459 | S3_24992200 | 3 | 24992200 | 151310 | G | A | A | 0.90323 | G | 0.09677 | 6 | 0.19355 |
| 2460 | S3_25577944 | 3 | 25577944 | 585744 | C | T | C | 0.77419 | T | 0.22581 | 14 | 0.45161 |
| 2461 | S3_26445276 | 3 | 26445276 | 867332 | G | T | G | 0.51613 | T | 0.48387 | 18 | 0.58065 |
| 2462 | S3_26445278 | 3 | 26445278 | 2 | A | C | A | 0.58065 | C | 0.41935 | 16 | 0.51613 |
| 2463 | S3_26465377 | 3 | 26465377 | 20099 | G | T | T | 0.90323 | G | 0.09677 | 6 | 0.19355 |
| 2464 | S3_26465463 | 3 | 26465463 | 86 | C | T | C | 0.80645 | T | 0.19355 | 10 | 0.32258 |
| 2465 | S3_26465483 | 3 | 26465483 | 20 | C | T | C | 0.85484 | T | 0.14516 | 9 | 0.29032 |
| 2466 | S3_26484149 | 3 | 26484149 | 18666 | A | G | G | 0.74194 | A | 0.25806 | 14 | 0.45161 |
| 2467 | S3_26484181 | 3 | 26484181 | 32 | C | T | T | 0.70968 | C | 0.29032 | 16 | 0.51613 |
| 2468 | S3_26484317 | 3 | 26484317 | 136 | C | T | T | 0.74194 | C | 0.25806 | 14 | 0.45161 |
| 2469 | S3_26484318 | 3 | 26484318 | 1 | A | G | G | 0.74194 | A | 0.25806 | 14 | 0.45161 |
| 2470 | S3_26509474 | 3 | 26509474 | 25156 | G | A | G | 0.82258 | A | 0.17742 | 11 | 0.35484 |
| 2471 | S3_26597387 | 3 | 26597387 | 87913 | C | T | C | 0.93548 | T | 0.06452 | 4 | 0.12903 |
| 2472 | S3_26597468 | 3 | 26597468 | 81 | G | A | A | 0.77419 | G | 0.22581 | 8 | 0.25806 |
| 2473 | S3_26605059 | 3 | 26605059 | 7591 | G | A | G | 0.95161 | A | 0.04839 | 3 | 0.09677 |
| 2474 | S3_26605073 | 3 | 26605073 | 14 | G | T | G | 0.93548 | T | 0.06452 | 4 | 0.12903 |
| 2475 | S3_26605074 | 3 | 26605074 | 1 | T | C | T | 0.93548 | C | 0.06452 | 4 | 0.12903 |
| 2476 | S3_26796948 | 3 | 26796948 | 191874 | G | T | G | 0.87097 | T | 0.12903 | 8 | 0.25806 |
| 2477 | S3_26797098 | 3 | 26797098 | 150 | G | A | G | 0.87097 | A | 0.12903 | 8 | 0.25806 |
| 2478 | S3_27082709 | 3 | 27082709 | 285611 | G | A | G | 0.95161 | A | 0.04839 | 3 | 0.09677 |
| 2479 | S3_27082726 | 3 | 27082726 | 17 | A | G | A | 0.87097 | G | 0.12903 | 8 | 0.25806 |
| 2480 | S3_27146419 | 3 | 27146419 | 63693 | A | G | A | 0.85484 | G | 0.14516 | 7 | 0.22581 |
| 2481 | S3_27182928 | 3 | 27182928 | 36509 | C | T | C | 0.80645 | T | 0.19355 | 10 | 0.32258 |
| 2482 | S3_27207651 | 3 | 27207651 | 24723 | C | T | C | 0.91935 | T | 0.08065 | 3 | 0.09677 |

| Site number | SNP name | Chromosome | Physical position | Physical distance from the previous SNP | Reference allele | Alternative allele | Major allele | Major allele frequency | Minor allele | Minor allele frequency | Number heterozygous | Heterozygosity |
|---|---|---|---|---|---|---|---|---|---|---|---|---|
| 2483 | S3_27325464 | 3 | 27325464 | 117813 | G | A | G | 0.95161 | A | 0.04839 | 3 | 0.09677 |
| 2484 | S3_27325565 | 3 | 27325565 | 101 | A | G | A | 0.95161 | G | 0.04839 | 3 | 0.09677 |
| 2485 | S3_27620707 | 3 | 27620707 | 295142 | G | A | A | 0.80645 | G | 0.19355 | 12 | 0.3871 |
| 2486 | S3_27966091 | 3 | 27966091 | 345384 | G | T | T | 0.62903 | G | 0.37097 | 19 | 0.6129 |
| 2487 | S3_27966149 | 3 | 27966149 | 58 | G | A | G | 0.79032 | A | 0.20968 | 13 | 0.41935 |
| 2488 | S3_28589920 | 3 | 28589920 | 623771 | G | A | G | 0.87097 | A | 0.12903 | 6 | 0.19355 |
| 2489 | S3_28595768 | 3 | 28595768 | 5848 | T | C | T | 0.83871 | C | 0.16129 | 8 | 0.25806 |
| 2490 | S3_28806163 | 3 | 28806163 | 210395 | C | T | T | 0.8871 | C | 0.1129 | 7 | 0.22581 |
| 2491 | S3_28806170 | 3 | 28806170 | 7 | G | A | G | 0.82258 | A | 0.17742 | 11 | 0.35484 |
| 2492 | S3_28806293 | 3 | 28806293 | 123 | C | T | C | 0.95161 | T | 0.04839 | 3 | 0.09677 |
| 2493 | S3_29286447 | 3 | 29286447 | 480154 | C | G | C | 0.66129 | G | 0.33871 | 13 | 0.41935 |
| 2494 | S3_29286462 | 3 | 29286462 | 15 | C | T | C | 0.74194 | T | 0.25806 | 12 | 0.3871 |
| 2495 | S3_29462483 | 3 | 29462483 | 176021 | T | C | T | 0.90323 | C | 0.09677 | 4 | 0.12903 |
| 2496 | S3_29495066 | 3 | 29495066 | 32583 | C | T | C | 0.82258 | T | 0.17742 | 11 | 0.35484 |
| 2497 | S3_29495166 | 3 | 29495166 | 100 | C | T | C | 0.70968 | T | 0.29032 | 10 | 0.32258 |
| 2498 | S3_29495184 | 3 | 29495184 | 18 | C | T | C | 0.83871 | T | 0.16129 | 10 | 0.32258 |
| 2499 | S3_30261412 | 3 | 30261412 | 766228 | A | G | A | 0.93548 | G | 0.06452 | 4 | 0.12903 |
| 2500 | S3_30261420 | 3 | 30261420 | 8 | C | T | T | 0.82258 | C | 0.17742 | 11 | 0.35484 |
| 2501 | S3_30819478 | 3 | 30819478 | 558058 | T | C | T | 0.8871 | C | 0.1129 | 5 | 0.16129 |
| 2502 | S3_30894303 | 3 | 30894303 | 74825 | G | T | G | 0.91935 | T | 0.08065 | 5 | 0.16129 |
| 2503 | S3_30983289 | 3 | 30983289 | 88986 | G | A | G | 0.91935 | A | 0.08065 | 5 | 0.16129 |
| 2504 | S3_30983377 | 3 | 30983377 | 88 | C | T | C | 0.93548 | T | 0.06452 | 4 | 0.12903 |
| 2505 | S3_31154294 | 3 | 31154294 | 170917 | T | A | T | 0.93548 | A | 0.06452 | 4 | 0.12903 |
| 2506 | S3_31902058 | 3 | 31902058 | 747764 | C | A | C | 0.8871 | A | 0.1129 | 7 | 0.22581 |
| 2507 | S3_31902086 | 3 | 31902086 | 28 | T | G | G | 0.62903 | T | 0.37097 | 13 | 0.41935 |
| 2508 | S3_32122540 | 3 | 32122540 | 220454 | T | C | C | 0.77419 | T | 0.22581 | 14 | 0.45161 |
| 2509 | S3_32122580 | 3 | 32122580 | 40 | G | A | G | 0.82258 | A | 0.17742 | 9 | 0.29032 |
| 2510 | S3_32629654 | 3 | 32629654 | 507074 | G | A | G | 0.85484 | A | 0.14516 | 9 | 0.29032 |
| 2511 | S3_32629865 | 3 | 32629865 | 211 | C | A | C | 0.91935 | A | 0.08065 | 5 | 0.16129 |
| 2512 | S3_32724860 | 3 | 32724860 | 94995 | G | A | G | 0.64516 | A | 0.35484 | 14 | 0.45161 |
| 2513 | S3_33508315 | 3 | 33508315 | 783455 | A | G | A | 0.87097 | G | 0.12903 | 6 | 0.19355 |
| 2514 | S3_33699738 | 3 | 33699738 | 191423 | C | T | C | 0.8871 | T | 0.1129 | 7 | 0.22581 |
| 2515 | S3_33809756 | 3 | 33809756 | 110018 | G | A | G | 0.91935 | A | 0.08065 | 5 | 0.16129 |
| 2516 | S3_33809758 | 3 | 33809758 | 2 | G | T | G | 0.90323 | T | 0.09677 | 6 | 0.19355 |
| 2517 | S3_33874584 | 3 | 33874584 | 64826 | G | A | G | 0.91935 | A | 0.08065 | 5 | 0.16129 |
| 2518 | S3_34228615 | 3 | 34228615 | 354031 | T | C | C | 0.51613 | T | 0.48387 | 14 | 0.45161 |
| 2519 | S3_34228642 | 3 | 34228642 | 27 | C | G | C | 0.90323 | G | 0.09677 | 6 | 0.19355 |
| 2520 | S3_34228738 | 3 | 34228738 | 96 | T | G | T | 0.85484 | G | 0.14516 | 7 | 0.22581 |
| 2521 | S3_34228742 | 3 | 34228742 | 4 | T | A | A | 0.5 | T | 0.5 | 15 | 0.48387 |
| 2522 | S3_37441131 | 3 | 37441131 | 3212389 | G | A | G | 0.74194 | A | 0.25806 | 12 | 0.3871 |
| 2523 | S3_37441262 | 3 | 37441262 | 131 | G | C | C | 0.51613 | G | 0.48387 | 12 | 0.3871 |
| 2524 | S3_37447628 | 3 | 37447628 | 6366 | A | G | G | 0.87097 | A | 0.12903 | 8 | 0.25806 |
| 2525 | S3_37447735 | 3 | 37447735 | 107 | G | C | G | 0.93548 | C | 0.06452 | 4 | 0.12903 |
| 2526 | S3_38098518 | 3 | 38098518 | 650783 | G | A | G | 0.53226 | A | 0.46774 | 19 | 0.6129 |
| 2527 | S3_38098558 | 3 | 38098558 | 40 | G | C | G | 0.90323 | C | 0.09677 | 6 | 0.19355 |
| 2528 | S3_38262578 | 3 | 38262578 | 164020 | C | T | C | 0.95161 | T | 0.04839 | 3 | 0.09677 |
| 2529 | S3_38262615 | 3 | 38262615 | 37 | G | A | G | 0.80645 | A | 0.19355 | 8 | 0.25806 |
| 2530 | S3_38262724 | 3 | 38262724 | 109 | G | A | G | 0.93548 | A | 0.06452 | 4 | 0.12903 |
| 2531 | S3_38625781 | 3 | 38625781 | 363057 | T | A | T | 0.95161 | A | 0.04839 | 3 | 0.09677 |
| 2532 | S3_38625894 | 3 | 38625894 | 113 | C | T | C | 0.75806 | T | 0.24194 | 9 | 0.29032 |
| 2533 | S3_38625940 | 3 | 38625940 | 46 | T | A | T | 0.82258 | A | 0.17742 | 11 | 0.35484 |
| 2534 | S3_39072976 | 3 | 39072976 | 447036 | C | T | C | 0.87097 | T | 0.12903 | 6 | 0.19355 |
| 2535 | S3_39589942 | 3 | 39589942 | 516966 | G | T | G | 0.80645 | T | 0.19355 | 10 | 0.32258 |
| 2536 | S3_39589951 | 3 | 39589951 | 9 | C | A | C | 0.56452 | A | 0.43548 | 11 | 0.35484 |

| Site number | SNP name | Chromosome | Physical position | Physical distance from the previous SNP | Reference allele | Alternative allele | Major allele | Major allele frequency | Minor allele | Minor allele frequency | Number heterozygous | Heterozygosity |
|---|---|---|---|---|---|---|---|---|---|---|---|---|
| 2537 | S3_39589960 | 3 | 39589960 | 9 | G | A | G | 0.93548 | A | 0.06452 | 2 | 0.06452 |
| 2538 | S3_39589965 | 3 | 39589965 | 5 | G | A | G | 0.77419 | A | 0.22581 | 12 | 0.3871 |
| 2539 | S3_39616334 | 3 | 39616334 | 26369 | C | G | G | 0.80645 | C | 0.19355 | 10 | 0.32258 |
| 2540 | S3_39616583 | 3 | 39616583 | 249 | T | C | C | 0.5 | T | 0.5 | 15 | 0.48387 |
| 2541 | S3_39783922 | 3 | 39783922 | 167339 | G | A | G | 0.95161 | A | 0.04839 | 3 | 0.09677 |
| 2542 | S3_39918597 | 3 | 39918597 | 134675 | C | T | T | 0.70968 | C | 0.29032 | 12 | 0.3871 |
| 2543 | S3_40555522 | 3 | 40555522 | 636925 | G | A | G | 0.59677 | A | 0.40323 | 15 | 0.48387 |
| 2544 | S3_40555669 | 3 | 40555669 | 147 | T | G | G | 0.95161 | T | 0.04839 | 3 | 0.09677 |
| 2545 | S3_40555717 | 3 | 40555717 | 48 | T | C | C | 0.95161 | T | 0.04839 | 3 | 0.09677 |
| 2546 | S3_40939027 | 3 | 40939027 | 383310 | G | C | G | 0.77419 | C | 0.22581 | 12 | 0.3871 |
| 2547 | S3_40939105 | 3 | 40939105 | 78 | T | C | T | 0.80645 | C | 0.19355 | 12 | 0.3871 |
| 2548 | S3_40939121 | 3 | 40939121 | 16 | G | T | G | 0.91935 | T | 0.08065 | 3 | 0.09677 |
| 2549 | S3_40939240 | 3 | 40939240 | 119 | C | T | C | 0.80645 | T | 0.19355 | 10 | 0.32258 |
| 2550 | S3_41236255 | 3 | 41236255 | 297015 | C | T | T | 0.87097 | C | 0.12903 | 8 | 0.25806 |
| 2551 | S3_41236291 | 3 | 41236291 | 36 | G | A | G | 0.75806 | A | 0.24194 | 13 | 0.41935 |
| 2552 | S3_41300816 | 3 | 41300816 | 64525 | T | C | T | 0.93548 | C | 0.06452 | 4 | 0.12903 |
| 2553 | S3_41300884 | 3 | 41300884 | 68 | C | T | C | 0.53226 | T | 0.46774 | 11 | 0.35484 |
| 2554 | S3_41300967 | 3 | 41300967 | 83 | A | T | A | 0.53226 | T | 0.46774 | 11 | 0.35484 |
| 2555 | S3_41900357 | 3 | 41900357 | 599390 | G | A | G | 0.72581 | A | 0.27419 | 15 | 0.48387 |
| 2556 | S3_41929546 | 3 | 41929546 | 29189 | C | G | G | 0.53226 | C | 0.46774 | 19 | 0.6129 |
| 2557 | S3_42085750 | 3 | 42085750 | 156204 | C | T | T | 0.74194 | C | 0.25806 | 14 | 0.45161 |
| 2558 | S3_42085877 | 3 | 42085877 | 127 | A | G | A | 0.74194 | G | 0.25806 | 14 | 0.45161 |
| 2559 | S3_42689336 | 3 | 42689336 | 603459 | T | C | C | 0.59677 | T | 0.40323 | 17 | 0.54839 |
| 2560 | S3_42958774 | 3 | 42958774 | 269438 | C | T | C | 0.90323 | T | 0.09677 | 6 | 0.19355 |
| 2561 | S3_42958792 | 3 | 42958792 | 18 | A | G | A | 0.80645 | G | 0.19355 | 10 | 0.32258 |
| 2562 | S3_42958894 | 3 | 42958894 | 102 | A | G | A | 0.74194 | G | 0.25806 | 14 | 0.45161 |
| 2563 | S3_42958957 | 3 | 42958957 | 63 | T | C | C | 0.8871 | T | 0.1129 | 7 | 0.22581 |
| 2564 | S3_42958961 | 3 | 42958961 | 4 | A | G | A | 0.87097 | G | 0.12903 | 8 | 0.25806 |
| 2565 | S3_42958981 | 3 | 42958981 | 20 | T | C | T | 0.90323 | C | 0.09677 | 6 | 0.19355 |
| 2566 | S3_43129493 | 3 | 43129493 | 170512 | T | C | T | 0.95161 | C | 0.04839 | 3 | 0.09677 |
| 2567 | S3_43129494 | 3 | 43129494 | 1 | G | T | G | 0.95161 | T | 0.04839 | 3 | 0.09677 |
| 2568 | S3_43129497 | 3 | 43129497 | 3 | A | T | A | 0.95161 | T | 0.04839 | 3 | 0.09677 |
| 2569 | S3_43792208 | 3 | 43792208 | 662711 | T | C | T | 0.90323 | C | 0.09677 | 4 | 0.12903 |
| 2570 | S3_44698435 | 3 | 44698435 | 906227 | G | A | G | 0.91935 | A | 0.08065 | 5 | 0.16129 |
| 2571 | S3_44698462 | 3 | 44698462 | 27 | T | A | T | 0.91935 | A | 0.08065 | 5 | 0.16129 |
| 2572 | S3_44698542 | 3 | 44698542 | 80 | G | T | G | 0.91935 | T | 0.08065 | 5 | 0.16129 |
| 2573 | S3_44698604 | 3 | 44698604 | 62 | G | A | G | 0.91935 | A | 0.08065 | 5 | 0.16129 |
| 2574 | S3_44941840 | 3 | 44941840 | 243236 | C | T | C | 0.69355 | T | 0.30645 | 9 | 0.29032 |
| 2575 | S3_44941905 | 3 | 44941905 | 65 | A | G | G | 0.75806 | A | 0.24194 | 9 | 0.29032 |
| 2576 | S3_45307800 | 3 | 45307800 | 365895 | A | G | G | 0.93548 | A | 0.06452 | 4 | 0.12903 |
| 2577 | S3_45307817 | 3 | 45307817 | 17 | C | T | C | 0.90323 | T | 0.09677 | 4 | 0.12903 |
| 2578 | S3_45307819 | 3 | 45307819 | 2 | G | C | G | 0.90323 | C | 0.09677 | 4 | 0.12903 |
| 2579 | S3_45474569 | 3 | 45474569 | 166750 | G | C | G | 0.74194 | C | 0.25806 | 14 | 0.45161 |
| 2580 | S3_45474610 | 3 | 45474610 | 41 | G | T | T | 0.77419 | G | 0.22581 | 14 | 0.45161 |
| 2581 | S3_45724853 | 3 | 45724853 | 250243 | C | T | C | 0.80645 | T | 0.19355 | 8 | 0.25806 |
| 2582 | S3_45724854 | 3 | 45724854 | 1 | A | G | G | 0.62903 | A | 0.37097 | 11 | 0.35484 |
| 2583 | S3_45724865 | 3 | 45724865 | 11 | T | C | T | 0.82258 | C | 0.17742 | 7 | 0.22581 |
| 2584 | S3_45724912 | 3 | 45724912 | 47 | A | G | A | 0.82258 | G | 0.17742 | 7 | 0.22581 |
| 2585 | S3_45724966 | 3 | 45724966 | 54 | C | T | C | 0.90323 | T | 0.09677 | 6 | 0.19355 |
| 2586 | S3_45725039 | 3 | 45725039 | 73 | A | G | A | 0.82258 | G | 0.17742 | 7 | 0.22581 |
| 2587 | S3_45989273 | 3 | 45989273 | 264234 | C | T | C | 0.91935 | T | 0.08065 | 5 | 0.16129 |
| 2588 | S3_46153784 | 3 | 46153784 | 164511 | A | G | A | 0.77419 | G | 0.22581 | 8 | 0.25806 |
| 2589 | S3_46482785 | 3 | 46482785 | 329001 | C | T | C | 0.80645 | T | 0.19355 | 10 | 0.32258 |
| 2590 | S3_46482835 | 3 | 46482835 | 50 | C | G | C | 0.95161 | G | 0.04839 | 3 | 0.09677 |

| Site number | SNP name | Chromosome | Physical position | Physical distance from the previous SNP | Reference allele | Alternative allele | Major allele | Major allele frequency | Minor allele | Minor allele frequency | Number heterozygous | Heterozygosity |
|---|---|---|---|---|---|---|---|---|---|---|---|---|
| 2591 | S3_46482883 | 3 | 46482883 | 48 | G | A | A | 0.69355 | G | 0.30645 | 9 | 0.29032 |
| 2592 | S3_46482885 | 3 | 46482885 | 2 | A | G | A | 0.95161 | G | 0.04839 | 3 | 0.09677 |
| 2593 | S3_47088257 | 3 | 47088257 | 605372 | C | G | C | 0.51613 | G | 0.48387 | 16 | 0.51613 |
| 2594 | S3_47088288 | 3 | 47088288 | 31 | G | A | G | 0.91935 | A | 0.08065 | 5 | 0.16129 |
| 2595 | S3_47088378 | 3 | 47088378 | 90 | C | T | C | 0.85484 | T | 0.14516 | 7 | 0.22581 |
| 2596 | S3_49871576 | 3 | 49871576 | 2783198 | T | C | T | 0.66129 | C | 0.33871 | 15 | 0.48387 |
| 2597 | S3_49871642 | 3 | 49871642 | 66 | T | C | T | 0.80645 | C | 0.19355 | 12 | 0.3871 |
| 2598 | S3_50282588 | 3 | 50282588 | 410946 | A | G | G | 0.8871 | A | 0.1129 | 7 | 0.22581 |
| 2599 | S3_50282602 | 3 | 50282602 | 14 | A | T | T | 0.53226 | A | 0.46774 | 15 | 0.48387 |
| 2600 | S3_50282616 | 3 | 50282616 | 14 | G | A | A | 0.8871 | G | 0.1129 | 7 | 0.22581 |
| 2601 | S3_50282676 | 3 | 50282676 | 60 | C | T | T | 0.8871 | C | 0.1129 | 7 | 0.22581 |
| 2602 | S3_50282682 | 3 | 50282682 | 6 | C | T | T | 0.8871 | C | 0.1129 | 7 | 0.22581 |
| 2603 | S3_50282683 | 3 | 50282683 | 1 | T | G | G | 0.8871 | T | 0.1129 | 7 | 0.22581 |
| 2604 | S3_50282737 | 3 | 50282737 | 54 | G | C | C | 0.8871 | G | 0.1129 | 7 | 0.22581 |
| 2605 | S3_50528660 | 3 | 50528660 | 245923 | G | A | G | 0.87097 | A | 0.12903 | 8 | 0.25806 |
| 2606 | S3_50528818 | 3 | 50528818 | 158 | C | T | C | 0.87097 | T | 0.12903 | 8 | 0.25806 |
| 2607 | S3_51280704 | 3 | 51280704 | 751886 | C | T | C | 0.93548 | T | 0.06452 | 4 | 0.12903 |
| 2608 | S3_51433022 | 3 | 51433022 | 152318 | C | T | C | 0.93548 | T | 0.06452 | 2 | 0.06452 |
| 2609 | S3_51433094 | 3 | 51433094 | 72 | A | C | A | 0.95161 | C | 0.04839 | 3 | 0.09677 |
| 2610 | S3_51433189 | 3 | 51433189 | 95 | A | T | A | 0.8871 | T | 0.1129 | 7 | 0.22581 |
| 2611 | S3_51555348 | 3 | 51555348 | 122159 | C | T | C | 0.87097 | T | 0.12903 | 6 | 0.19355 |
| 2612 | S3_51555426 | 3 | 51555426 | 78 | C | T | C | 0.95161 | T | 0.04839 | 3 | 0.09677 |
| 2613 | S3_51555464 | 3 | 51555464 | 38 | C | T | C | 0.56452 | T | 0.43548 | 11 | 0.35484 |
| 2614 | S3_51555465 | 3 | 51555465 | 1 | C | T | C | 0.93548 | T | 0.06452 | 4 | 0.12903 |
| 2615 | S3_51681998 | 3 | 51681998 | 126533 | C | T | C | 0.77419 | T | 0.22581 | 14 | 0.45161 |
| 2616 | S3_51682035 | 3 | 51682035 | 37 | A | G | A | 0.90323 | G | 0.09677 | 6 | 0.19355 |
| 2617 | S3_52106735 | 3 | 52106735 | 424700 | C | T | C | 0.83871 | T | 0.16129 | 10 | 0.32258 |
| 2618 | S3_52519831 | 3 | 52519831 | 413096 | T | C | C | 0.91935 | T | 0.08065 | 5 | 0.16129 |
| 2619 | S3_52519929 | 3 | 52519929 | 98 | T | A | T | 0.90323 | A | 0.09677 | 4 | 0.12903 |
| 2620 | S3_52597175 | 3 | 52597175 | 77246 | C | T | C | 0.8871 | T | 0.1129 | 5 | 0.16129 |
| 2621 | S3_52941079 | 3 | 52941079 | 343904 | T | C | T | 0.82258 | C | 0.17742 | 7 | 0.22581 |
| 2622 | S3_53056696 | 3 | 53056696 | 115617 | T | C | T | 0.91935 | C | 0.08065 | 3 | 0.09677 |
| 2623 | S3_53056760 | 3 | 53056760 | 64 | A | G | A | 0.67742 | G | 0.32258 | 12 | 0.3871 |
| 2624 | S3_53293943 | 3 | 53293943 | 237183 | G | A | G | 0.93548 | A | 0.06452 | 4 | 0.12903 |
| 2625 | S3_53294140 | 3 | 53294140 | 197 | C | T | C | 0.91935 | T | 0.08065 | 5 | 0.16129 |
| 2626 | S3_53477730 | 3 | 53477730 | 183590 | G | A | G | 0.95161 | A | 0.04839 | 3 | 0.09677 |
| 2627 | S3_53477806 | 3 | 53477806 | 76 | G | A | G | 0.93548 | A | 0.06452 | 4 | 0.12903 |
| 2628 | S3_53477819 | 3 | 53477819 | 13 | C | A | C | 0.91935 | A | 0.08065 | 3 | 0.09677 |
| 2629 | S3_53477953 | 3 | 53477953 | 134 | C | A | C | 0.95161 | A | 0.04839 | 3 | 0.09677 |
| 2630 | S3_53555826 | 3 | 53555826 | 77873 | A | T | A | 0.66129 | T | 0.33871 | 15 | 0.48387 |
| 2631 | S3_53555872 | 3 | 53555872 | 46 | A | T | A | 0.87097 | T | 0.12903 | 8 | 0.25806 |
| 2632 | S3_53555960 | 3 | 53555960 | 88 | G | A | G | 0.6129 | A | 0.3871 | 18 | 0.58065 |
| 2633 | S3_53711128 | 3 | 53711128 | 155168 | G | A | G | 0.51613 | A | 0.48387 | 12 | 0.3871 |
| 2634 | S3_54045393 | 3 | 54045393 | 334265 | T | C | T | 0.64516 | C | 0.35484 | 12 | 0.3871 |
| 2635 | S3_54045397 | 3 | 54045397 | 4 | C | T | C | 0.64516 | T | 0.35484 | 12 | 0.3871 |
| 2636 | S3_54045476 | 3 | 54045476 | 79 | T | A | T | 0.64516 | A | 0.35484 | 12 | 0.3871 |
| 2637 | S3_54724714 | 3 | 54724714 | 679238 | C | T | T | 0.6129 | C | 0.3871 | 12 | 0.3871 |
| 2638 | S3_54724796 | 3 | 54724796 | 82 | C | T | C | 0.91935 | T | 0.08065 | 5 | 0.16129 |
| 2639 | S3_55025292 | 3 | 55025292 | 300496 | C | T | C | 0.93548 | T | 0.06452 | 4 | 0.12903 |
| 2640 | S3_55425480 | 3 | 55425480 | 400188 | G | A | G | 0.79032 | A | 0.20968 | 11 | 0.35484 |
| 2641 | S3_55948435 | 3 | 55948435 | 522955 | G | A | G | 0.91935 | A | 0.08065 | 5 | 0.16129 |
| 2642 | S3_55948454 | 3 | 55948454 | 19 | G | C | G | 0.93548 | C | 0.06452 | 4 | 0.12903 |
| 2643 | S3_55948524 | 3 | 55948524 | 70 | C | T | C | 0.66129 | T | 0.33871 | 15 | 0.48387 |
| 2644 | S3_55955895 | 3 | 55955895 | 7371 | G | A | G | 0.93548 | A | 0.06452 | 4 | 0.12903 |

| Site number | SNP name | Chromosome | Physical position | Physical distance from the previous SNP | Reference allele | Alternative allele | Major allele | Major allele frequency | Minor allele | Minor allele frequency | Number heterozygous | Heterozygosity |
|---|---|---|---|---|---|---|---|---|---|---|---|---|
| 2645 | S3_55955911 | 3 | 55955911 | 16 | T | C | C | 0.80645 | T | 0.19355 | 6 | 0.19355 |
| 2646 | S3_56610911 | 3 | 56610911 | 655000 | G | A | G | 0.90323 | A | 0.09677 | 4 | 0.12903 |
| 2647 | S3_56610942 | 3 | 56610942 | 31 | T | A | A | 0.62903 | T | 0.37097 | 17 | 0.54839 |
| 2648 | S3_56611128 | 3 | 56611128 | 186 | G | A | G | 0.85484 | A | 0.14516 | 7 | 0.22581 |
| 2649 | S3_56785259 | 3 | 56785259 | 174131 | C | G | C | 0.90323 | G | 0.09677 | 6 | 0.19355 |
| 2650 | S3_56810529 | 3 | 56810529 | 25270 | A | T | A | 0.51613 | T | 0.48387 | 16 | 0.51613 |
| 2651 | S3_56810673 | 3 | 56810673 | 144 | G | A | A | 0.5 | G | 0.5 | 15 | 0.48387 |
| 2652 | S3_57169916 | 3 | 57169916 | 359243 | C | T | C | 0.95161 | T | 0.04839 | 3 | 0.09677 |
| 2653 | S3_57169927 | 3 | 57169927 | 11 | T | C | T | 0.64516 | C | 0.35484 | 18 | 0.58065 |
| 2654 | S3_57169947 | 3 | 57169947 | 20 | T | A | T | 0.95161 | A | 0.04839 | 3 | 0.09677 |
| 2655 | S3_57169979 | 3 | 57169979 | 32 | G | C | G | 0.87097 | C | 0.12903 | 6 | 0.19355 |
| 2656 | S3_58022676 | 3 | 58022676 | 852697 | A | G | G | 0.93548 | A | 0.06452 | 4 | 0.12903 |
| 2657 | S3_58022682 | 3 | 58022682 | 6 | T | C | T | 0.80645 | C | 0.19355 | 12 | 0.3871 |
| 2658 | S3_58022705 | 3 | 58022705 | 23 | T | C | C | 0.6129 | T | 0.3871 | 18 | 0.58065 |
| 2659 | S3_58022842 | 3 | 58022842 | 137 | C | T | C | 0.91935 | T | 0.08065 | 3 | 0.09677 |
| 2660 | S3_58022858 | 3 | 58022858 | 16 | C | T | T | 0.93548 | C | 0.06452 | 4 | 0.12903 |
| 2661 | S3_58451790 | 3 | 58451790 | 428932 | T | C | T | 0.56452 | C | 0.43548 | 15 | 0.48387 |
| 2662 | S3_58451813 | 3 | 58451813 | 23 | G | A | G | 0.82258 | A | 0.17742 | 11 | 0.35484 |
| 2663 | S3_59483284 | 3 | 59483284 | 1031471 | A | G | A | 0.93548 | G | 0.06452 | 4 | 0.12903 |
| 2664 | S3_59483477 | 3 | 59483477 | 193 | G | A | G | 0.91935 | A | 0.08065 | 5 | 0.16129 |
| 2665 | S3_59725759 | 3 | 59725759 | 242282 | G | A | A | 0.58065 | G | 0.41935 | 18 | 0.58065 |
| 2666 | S3_59725765 | 3 | 59725765 | 6 | G | A | G | 0.95161 | A | 0.04839 | 3 | 0.09677 |
| 2667 | S3_60374210 | 3 | 60374210 | 648445 | G | C | G | 0.54839 | C | 0.45161 | 16 | 0.51613 |
| 2668 | S3_60374379 | 3 | 60374379 | 169 | A | G | A | 0.54839 | G | 0.45161 | 16 | 0.51613 |
| 2669 | S3_60748058 | 3 | 60748058 | 373679 | T | C | T | 0.79032 | C | 0.20968 | 13 | 0.41935 |
| 2670 | S3_60748083 | 3 | 60748083 | 25 | C | T | C | 0.6129 | T | 0.3871 | 20 | 0.64516 |
| 2671 | S3_60748105 | 3 | 60748105 | 22 | T | G | T | 0.8871 | G | 0.1129 | 7 | 0.22581 |
| 2672 | S3_60748120 | 3 | 60748120 | 15 | G | A | G | 0.6129 | A | 0.3871 | 20 | 0.64516 |
| 2673 | S3_60748171 | 3 | 60748171 | 51 | G | A | G | 0.93548 | A | 0.06452 | 4 | 0.12903 |
| 2674 | S3_60748231 | 3 | 60748231 | 60 | T | C | T | 0.83871 | C | 0.16129 | 8 | 0.25806 |
| 2675 | S3_61906529 | 3 | 61906529 | 1158298 | G | A | G | 0.75806 | A | 0.24194 | 11 | 0.35484 |
| 2676 | S3_61906712 | 3 | 61906712 | 183 | T | C | T | 0.87097 | C | 0.12903 | 6 | 0.19355 |
| 2677 | S3_62068222 | 3 | 62068222 | 161510 | T | A | T | 0.87097 | A | 0.12903 | 6 | 0.19355 |
| 2678 | S3_62068239 | 3 | 62068239 | 17 | G | A | G | 0.93548 | A | 0.06452 | 4 | 0.12903 |
| 2679 | S3_62068411 | 3 | 62068411 | 172 | C | G | C | 0.53226 | G | 0.46774 | 13 | 0.41935 |
| 2680 | S3_62068413 | 3 | 62068413 | 2 | G | A | G | 0.95161 | A | 0.04839 | 3 | 0.09677 |
| 2681 | S3_63567004 | 3 | 63567004 | 1498591 | T | G | T | 0.79032 | G | 0.20968 | 13 | 0.41935 |
| 2682 | S3_63567035 | 3 | 63567035 | 31 | T | G | T | 0.74194 | G | 0.25806 | 14 | 0.45161 |
| 2683 | S3_63567099 | 3 | 63567099 | 64 | G | A | G | 0.79032 | A | 0.20968 | 13 | 0.41935 |
| 2684 | S3_63567103 | 3 | 63567103 | 4 | G | C | G | 0.90323 | C | 0.09677 | 4 | 0.12903 |
| 2685 | S3_63567136 | 3 | 63567136 | 33 | A | G | A | 0.79032 | G | 0.20968 | 13 | 0.41935 |
| 2686 | S3_63567181 | 3 | 63567181 | 45 | G | C | G | 0.67742 | C | 0.32258 | 14 | 0.45161 |
| 2687 | S3_63567184 | 3 | 63567184 | 3 | T | C | T | 0.79032 | C | 0.20968 | 13 | 0.41935 |
| 2688 | S3_63977646 | 3 | 63977646 | 410462 | A | G | A | 0.95161 | G | 0.04839 | 3 | 0.09677 |
| 2689 | S3_64006993 | 3 | 64006993 | 29347 | G | T | G | 0.90323 | T | 0.09677 | 6 | 0.19355 |
| 2690 | S3_64007096 | 3 | 64007096 | 103 | C | T | C | 0.62903 | T | 0.37097 | 9 | 0.29032 |
| 2691 | S3_64665900 | 3 | 64665900 | 658804 | C | G | G | 0.51613 | C | 0.48387 | 14 | 0.45161 |
| 2692 | S3_64665990 | 3 | 64665990 | 90 | A | G | G | 0.79032 | A | 0.20968 | 11 | 0.35484 |
| 2693 | S3_64666111 | 3 | 64666111 | 121 | G | A | A | 0.51613 | G | 0.48387 | 14 | 0.45161 |
| 2694 | S3_65278971 | 3 | 65278971 | 612860 | T | C | C | 0.91935 | T | 0.08065 | 5 | 0.16129 |
| 2695 | S3_65479246 | 3 | 65479246 | 200275 | A | G | G | 0.8871 | A | 0.1129 | 7 | 0.22581 |
| 2696 | S3_66266394 | 3 | 66266394 | 787148 | T | C | T | 0.85484 | C | 0.14516 | 7 | 0.22581 |
| 2697 | S3_66266459 | 3 | 66266459 | 65 | G | C | G | 0.53226 | C | 0.46774 | 17 | 0.54839 |
| 2698 | S3_66347341 | 3 | 66347341 | 80882 | C | G | C | 0.74194 | G | 0.25806 | 16 | 0.51613 |

| Site number | SNP name | Chromosome | Physical position | Physical distance from the previous SNP | Reference allele | Alternative allele | Major allele | Major allele frequency | Minor allele | Minor allele frequency | Number heterozygous | Heterozygosity |
|---|---|---|---|---|---|---|---|---|---|---|---|---|
| 2699 | S3_66347351 | 3 | 66347351 | 10 | G | A | G | 0.93548 | A | 0.06452 | 4 | 0.12903 |
| 2700 | S3_66347374 | 3 | 66347374 | 23 | C | T | C | 0.91935 | T | 0.08065 | 5 | 0.16129 |
| 2701 | S3_66347456 | 3 | 66347456 | 82 | A | C | A | 0.85484 | C | 0.14516 | 9 | 0.29032 |
| 2702 | S3_66348807 | 3 | 66348807 | 1351 | C | T | C | 0.91935 | T | 0.08065 | 5 | 0.16129 |
| 2703 | S3_66348841 | 3 | 66348841 | 34 | C | T | C | 0.87097 | T | 0.12903 | 8 | 0.25806 |
| 2704 | S3_66353126 | 3 | 66353126 | 4285 | A | G | A | 0.77419 | G | 0.22581 | 14 | 0.45161 |
| 2705 | S3_66381359 | 3 | 66381359 | 28233 | G | C | G | 0.77419 | C | 0.22581 | 14 | 0.45161 |
| 2706 | S3_66381386 | 3 | 66381386 | 27 | T | C | T | 0.72581 | C | 0.27419 | 17 | 0.54839 |
| 2707 | S3_66381387 | 3 | 66381387 | 1 | G | A | G | 0.87097 | A | 0.12903 | 8 | 0.25806 |
| 2708 | S3_66381408 | 3 | 66381408 | 21 | C | T | C | 0.74194 | T | 0.25806 | 16 | 0.51613 |
| 2709 | S3_66381440 | 3 | 66381440 | 32 | C | T | C | 0.72581 | T | 0.27419 | 17 | 0.54839 |
| 2710 | S3_66381442 | 3 | 66381442 | 2 | G | A | G | 0.72581 | A | 0.27419 | 17 | 0.54839 |
| 2711 | S3_66381444 | 3 | 66381444 | 2 | C | A | C | 0.72581 | A | 0.27419 | 17 | 0.54839 |
| 2712 | S3_66381452 | 3 | 66381452 | 8 | A | G | A | 0.74194 | G | 0.25806 | 16 | 0.51613 |
| 2713 | S3_66381475 | 3 | 66381475 | 23 | G | A | G | 0.80645 | A | 0.19355 | 12 | 0.3871 |
| 2714 | S3_66381481 | 3 | 66381481 | 6 | G | A | G | 0.72581 | A | 0.27419 | 17 | 0.54839 |
| 2715 | S3_66381483 | 3 | 66381483 | 2 | A | G | A | 0.72581 | G | 0.27419 | 17 | 0.54839 |
| 2716 | S3_66381527 | 3 | 66381527 | 44 | G | A | G | 0.93548 | A | 0.06452 | 4 | 0.12903 |
| 2717 | S3_66381532 | 3 | 66381532 | 5 | T | C | T | 0.72581 | C | 0.27419 | 17 | 0.54839 |
| 2718 | S3_66381533 | 3 | 66381533 | 1 | G | A | G | 0.72581 | A | 0.27419 | 17 | 0.54839 |
| 2719 | S3_66762056 | 3 | 66762056 | 380523 | G | C | G | 0.72581 | C | 0.27419 | 17 | 0.54839 |
| 2720 | S3_66762085 | 3 | 66762085 | 29 | G | C | G | 0.72581 | C | 0.27419 | 17 | 0.54839 |
| 2721 | S3_66762116 | 3 | 66762116 | 31 | G | A | G | 0.72581 | A | 0.27419 | 17 | 0.54839 |
| 2722 | S3_66762149 | 3 | 66762149 | 33 | A | G | A | 0.74194 | G | 0.25806 | 16 | 0.51613 |
| 2723 | S3_66762209 | 3 | 66762209 | 60 | A | G | A | 0.72581 | G | 0.27419 | 17 | 0.54839 |
| 2724 | S3_66762272 | 3 | 66762272 | 63 | C | T | C | 0.72581 | T | 0.27419 | 17 | 0.54839 |
| 2725 | S3_67141618 | 3 | 67141618 | 379346 | A | G | G | 0.93548 | A | 0.06452 | 4 | 0.12903 |
| 2726 | S3_67338629 | 3 | 67338629 | 197011 | G | C | G | 0.80645 | C | 0.19355 | 8 | 0.25806 |
| 2727 | S3_67406472 | 3 | 67406472 | 67843 | C | G | G | 0.95161 | C | 0.04839 | 3 | 0.09677 |
| 2728 | S3_67406510 | 3 | 67406510 | 38 | T | C | C | 0.95161 | T | 0.04839 | 3 | 0.09677 |
| 2729 | S3_67406549 | 3 | 67406549 | 39 | C | T | T | 0.95161 | C | 0.04839 | 3 | 0.09677 |
| 2730 | S3_67860459 | 3 | 67860459 | 453910 | A | G | A | 0.90323 | G | 0.09677 | 6 | 0.19355 |
| 2731 | S3_67860524 | 3 | 67860524 | 65 | G | A | G | 0.95161 | A | 0.04839 | 3 | 0.09677 |
| 2732 | S3_67860609 | 3 | 67860609 | 85 | A | C | A | 0.8871 | C | 0.1129 | 5 | 0.16129 |
| 2733 | S3_68041644 | 3 | 68041644 | 181035 | C | T | C | 0.91935 | T | 0.08065 | 5 | 0.16129 |
| 2734 | S3_68172359 | 3 | 68172359 | 130715 | T | C | T | 0.95161 | C | 0.04839 | 3 | 0.09677 |
| 2735 | S3_68172427 | 3 | 68172427 | 68 | A | C | C | 0.56452 | A | 0.43548 | 19 | 0.6129 |
| 2736 | S3_69320278 | 3 | 69320278 | 1147851 | A | G | G | 0.77419 | A | 0.22581 | 12 | 0.3871 |
| 2737 | S3_69606369 | 3 | 69606369 | 286091 | C | T | C | 0.62903 | T | 0.37097 | 13 | 0.41935 |
| 2738 | S3_69656971 | 3 | 69656971 | 50602 | T | C | T | 0.95161 | C | 0.04839 | 3 | 0.09677 |
| 2739 | S3_71405090 | 3 | 71405090 | 1748119 | C | T | C | 0.69355 | T | 0.30645 | 15 | 0.48387 |
| 2740 | S3_71405124 | 3 | 71405124 | 34 | T | C | C | 0.69355 | T | 0.30645 | 13 | 0.41935 |
| 2741 | S3_71872225 | 3 | 71872225 | 467101 | C | G | C | 0.95161 | G | 0.04839 | 3 | 0.09677 |
| 2742 | S3_71872415 | 3 | 71872415 | 190 | T | C | C | 0.6129 | T | 0.3871 | 18 | 0.58065 |
| 2743 | S3_72148373 | 3 | 72148373 | 275958 | C | G | C | 0.8871 | G | 0.1129 | 7 | 0.22581 |
| 2744 | S3_72516391 | 3 | 72516391 | 368018 | G | A | G | 0.80645 | A | 0.19355 | 12 | 0.3871 |
| 2745 | S3_72529381 | 3 | 72529381 | 12990 | C | T | C | 0.70968 | T | 0.29032 | 12 | 0.3871 |
| 2746 | S3_72529387 | 3 | 72529387 | 6 | T | C | T | 0.70968 | C | 0.29032 | 12 | 0.3871 |
| 2747 | S3_72738189 | 3 | 72738189 | 208802 | C | A | C | 0.87097 | A | 0.12903 | 8 | 0.25806 |
| 2748 | S3_73349171 | 3 | 73349171 | 610982 | C | A | A | 0.83871 | C | 0.16129 | 10 | 0.32258 |
| 2749 | S3_73349177 | 3 | 73349177 | 6 | T | C | T | 0.74194 | C | 0.25806 | 14 | 0.45161 |
| 2750 | S3_73463422 | 3 | 73463422 | 114245 | A | G | A | 0.87097 | G | 0.12903 | 8 | 0.25806 |
| 2751 | S3_73491907 | 3 | 73491907 | 28485 | C | G | C | 0.56452 | G | 0.43548 | 13 | 0.41935 |
| 2752 | S3_73960250 | 3 | 73960250 | 468343 | C | G | C | 0.77419 | G | 0.22581 | 10 | 0.32258 |

| Site number | SNP name | Chromosome | Physical position | Physical distance from the previous SNP | Reference allele | Alternative allele | Major allele | Major allele frequency | Minor allele | Minor allele frequency | Number heterozygous | Heterozygosity |
|---|---|---|---|---|---|---|---|---|---|---|---|---|
| 2753 | S3_73960270 | 3 | 73960270 | 20 | C | G | C | 0.53226 | G | 0.46774 | 19 | 0.6129 |
| 2754 | S3_74024972 | 3 | 74024972 | 64702 | T | C | T | 0.80645 | C | 0.19355 | 8 | 0.25806 |
| 2755 | S3_74025094 | 3 | 74025094 | 122 | G | A | G | 0.69355 | A | 0.30645 | 13 | 0.41935 |
| 2756 | S3_74298176 | 3 | 74298176 | 273082 | G | C | G | 0.75806 | C | 0.24194 | 11 | 0.35484 |
| 2757 | S3_74298296 | 3 | 74298296 | 120 | T | C | T | 0.75806 | C | 0.24194 | 11 | 0.35484 |
| 2758 | S3_74310460 | 3 | 74310460 | 12164 | C | T | C | 0.67742 | T | 0.32258 | 10 | 0.32258 |
| 2759 | S3_74310513 | 3 | 74310513 | 53 | T | C | T | 0.95161 | C | 0.04839 | 3 | 0.09677 |
| 2760 | S3_74676012 | 3 | 74676012 | 365499 | T | A | T | 0.87097 | A | 0.12903 | 6 | 0.19355 |
| 2761 | S3_76349545 | 3 | 76349545 | 1673533 | C | T | C | 0.90323 | T | 0.09677 | 4 | 0.12903 |
| 2762 | S3_76349780 | 3 | 76349780 | 235 | A | G | A | 0.90323 | G | 0.09677 | 6 | 0.19355 |
| 2763 | S3_76894511 | 3 | 76894511 | 544731 | T | A | T | 0.82258 | A | 0.17742 | 9 | 0.29032 |
| 2764 | S3_77143168 | 3 | 77143168 | 248657 | T | C | T | 0.95161 | C | 0.04839 | 3 | 0.09677 |
| 2765 | S3_77356235 | 3 | 77356235 | 213067 | C | T | C | 0.59677 | T | 0.40323 | 19 | 0.6129 |
| 2766 | S3_77356244 | 3 | 77356244 | 9 | C | T | C | 0.54839 | T | 0.45161 | 18 | 0.58065 |
| 2767 | S3_77451217 | 3 | 77451217 | 94973 | C | T | C | 0.91935 | T | 0.08065 | 5 | 0.16129 |
| 2768 | S3_77451236 | 3 | 77451236 | 19 | C | T | C | 0.93548 | T | 0.06452 | 4 | 0.12903 |
| 2769 | S3_77451437 | 3 | 77451437 | 201 | T | C | T | 0.93548 | C | 0.06452 | 4 | 0.12903 |
| 2770 | S3_77455653 | 3 | 77455653 | 4216 | G | C | G | 0.91935 | C | 0.08065 | 3 | 0.09677 |
| 2771 | S3_77455752 | 3 | 77455752 | 99 | C | T | C | 0.91935 | T | 0.08065 | 3 | 0.09677 |
| 2772 | S3_77584689 | 3 | 77584689 | 128937 | T | G | G | 0.6129 | T | 0.3871 | 14 | 0.45161 |
| 2773 | S3_77925398 | 3 | 77925398 | 340709 | T | C | T | 0.95161 | C | 0.04839 | 3 | 0.09677 |
| 2774 | S3_77925461 | 3 | 77925461 | 63 | G | A | G | 0.85484 | A | 0.14516 | 7 | 0.22581 |
| 2775 | S3_77925492 | 3 | 77925492 | 31 | G | A | A | 0.53226 | G | 0.46774 | 15 | 0.48387 |
| 2776 | S3_77925518 | 3 | 77925518 | 26 | C | T | C | 0.51613 | T | 0.48387 | 18 | 0.58065 |
| 2777 | S3_77925561 | 3 | 77925561 | 43 | A | G | A | 0.95161 | G | 0.04839 | 3 | 0.09677 |
| 2778 | S3_77925643 | 3 | 77925643 | 82 | T | C | T | 0.95161 | C | 0.04839 | 3 | 0.09677 |
| 2779 | S3_79368264 | 3 | 79368264 | 1442621 | T | C | C | 0.66129 | T | 0.33871 | 15 | 0.48387 |
| 2780 | S3_79368284 | 3 | 79368284 | 20 | G | A | G | 0.79032 | A | 0.20968 | 11 | 0.35484 |
| 2781 | S3_79368302 | 3 | 79368302 | 18 | C | T | C | 0.53226 | T | 0.46774 | 21 | 0.67742 |
| 2782 | S3_80256396 | 3 | 80256396 | 888094 | G | A | G | 0.80645 | A | 0.19355 | 10 | 0.32258 |
| 2783 | S3_80256428 | 3 | 80256428 | 32 | A | C | A | 0.79032 | C | 0.20968 | 11 | 0.35484 |
| 2784 | S3_80256504 | 3 | 80256504 | 76 | T | C | T | 0.77419 | C | 0.22581 | 12 | 0.3871 |
| 2785 | S3_80256578 | 3 | 80256578 | 74 | C | A | C | 0.69355 | A | 0.30645 | 11 | 0.35484 |
| 2786 | S3_80956729 | 3 | 80956729 | 700151 | C | T | C | 0.87097 | T | 0.12903 | 8 | 0.25806 |
| 2787 | S3_80956774 | 3 | 80956774 | 45 | C | T | C | 0.87097 | T | 0.12903 | 6 | 0.19355 |
| 2788 | S3_81099157 | 3 | 81099157 | 142383 | T | C | C | 0.72581 | T | 0.27419 | 11 | 0.35484 |
| 2789 | S3_81099295 | 3 | 81099295 | 138 | A | G | A | 0.75806 | G | 0.24194 | 11 | 0.35484 |
| 2790 | S3_81099339 | 3 | 81099339 | 44 | C | A | C | 0.8871 | A | 0.1129 | 7 | 0.22581 |
| 2791 | S3_81099380 | 3 | 81099380 | 41 | G | C | G | 0.80645 | C | 0.19355 | 12 | 0.3871 |
| 2792 | S3_81166089 | 3 | 81166089 | 66709 | C | T | C | 0.91935 | T | 0.08065 | 5 | 0.16129 |
| 2793 | S3_81707909 | 3 | 81707909 | 541820 | A | G | A | 0.74194 | G | 0.25806 | 16 | 0.51613 |
| 2794 | S3_81707950 | 3 | 81707950 | 41 | G | A | G | 0.82258 | A | 0.17742 | 11 | 0.35484 |
| 2795 | S3_81707981 | 3 | 81707981 | 31 | A | T | A | 0.79032 | T | 0.20968 | 13 | 0.41935 |
| 2796 | S3_81785608 | 3 | 81785608 | 77627 | T | C | T | 0.74194 | C | 0.25806 | 12 | 0.3871 |
| 2797 | S3_81785617 | 3 | 81785617 | 9 | G | T | G | 0.83871 | T | 0.16129 | 10 | 0.32258 |
| 2798 | S3_81785642 | 3 | 81785642 | 25 | A | G | A | 0.90323 | G | 0.09677 | 6 | 0.19355 |
| 2799 | S3_81973471 | 3 | 81973471 | 187829 | A | G | A | 0.53226 | G | 0.46774 | 13 | 0.41935 |
| 2800 | S3_81973473 | 3 | 81973473 | 2 | G | C | C | 0.83871 | G | 0.16129 | 10 | 0.32258 |
| 2801 | S3_82006462 | 3 | 82006462 | 32989 | T | C | C | 0.62903 | T | 0.37097 | 13 | 0.41935 |
| 2802 | S3_82006466 | 3 | 82006466 | 4 | C | T | C | 0.80645 | T | 0.19355 | 8 | 0.25806 |
| 2803 | S3_82588416 | 3 | 82588416 | 581950 | A | G | A | 0.8871 | G | 0.1129 | 7 | 0.22581 |
| 2804 | S3_82588434 | 3 | 82588434 | 18 | C | T | C | 0.82258 | T | 0.17742 | 7 | 0.22581 |
| 2805 | S3_82588489 | 3 | 82588489 | 55 | C | T | C | 0.67742 | T | 0.32258 | 14 | 0.45161 |
| 2806 | S3_82588517 | 3 | 82588517 | 28 | T | C | T | 0.80645 | C | 0.19355 | 8 | 0.25806 |

| Site number | SNP name | Chromosome | Physical position | Physical distance from the previous SNP | Reference allele | Alternative allele | Major allele | Major allele frequency | Minor allele | Minor allele frequency | Number heterozygous | Heterozygosity |
|---|---|---|---|---|---|---|---|---|---|---|---|---|
| 2807 | S3_84321722 | 3 | 84321722 | 1733205 | C | G | C | 0.95161 | G | 0.04839 | 3 | 0.09677 |
| 2808 | S3_84910234 | 3 | 84910234 | 588512 | G | A | G | 0.82258 | A | 0.17742 | 9 | 0.29032 |
| 2809 | S3_84910405 | 3 | 84910405 | 171 | C | T | C | 0.95161 | T | 0.04839 | 3 | 0.09677 |
| 2810 | S3_84941743 | 3 | 84941743 | 31338 | T | A | T | 0.79032 | A | 0.20968 | 11 | 0.35484 |
| 2811 | S3_84941770 | 3 | 84941770 | 27 | T | C | T | 0.79032 | C | 0.20968 | 11 | 0.35484 |
| 2812 | S3_85450678 | 3 | 85450678 | 508908 | C | T | C | 0.90323 | T | 0.09677 | 6 | 0.19355 |
| 2813 | S3_86079941 | 3 | 86079941 | 629263 | G | A | G | 0.83871 | A | 0.16129 | 8 | 0.25806 |
| 2814 | S3_86080020 | 3 | 86080020 | 79 | A | G | A | 0.80645 | G | 0.19355 | 10 | 0.32258 |
| 2815 | S3_86436108 | 3 | 86436108 | 356088 | A | G | A | 0.53226 | G | 0.46774 | 15 | 0.48387 |
| 2816 | S3_86436154 | 3 | 86436154 | 46 | G | A | G | 0.93548 | A | 0.06452 | 4 | 0.12903 |
| 2817 | S3_86629308 | 3 | 86629308 | 193154 | T | C | T | 0.80645 | C | 0.19355 | 8 | 0.25806 |
| 2818 | S3_86629322 | 3 | 86629322 | 14 | A | C | A | 0.69355 | C | 0.30645 | 13 | 0.41935 |
| 2819 | S3_86629450 | 3 | 86629450 | 128 | C | G | C | 0.93548 | G | 0.06452 | 4 | 0.12903 |
| 2820 | S3_86786050 | 3 | 86786050 | 156600 | T | G | T | 0.8871 | G | 0.1129 | 7 | 0.22581 |
| 2821 | S3_86786275 | 3 | 86786275 | 225 | G | A | G | 0.77419 | A | 0.22581 | 14 | 0.45161 |
| 2822 | S3_86881997 | 3 | 86881997 | 95722 | C | T | T | 0.80645 | C | 0.19355 | 10 | 0.32258 |
| 2823 | S3_87413500 | 3 | 87413500 | 531503 | C | T | C | 0.69355 | T | 0.30645 | 13 | 0.41935 |
| 2824 | S3_87413516 | 3 | 87413516 | 16 | T | G | T | 0.72581 | G | 0.27419 | 13 | 0.41935 |
| 2825 | S3_87413619 | 3 | 87413619 | 103 | T | C | T | 0.62903 | C | 0.37097 | 17 | 0.54839 |
| 2826 | S3_87413690 | 3 | 87413690 | 71 | T | C | T | 0.80645 | C | 0.19355 | 12 | 0.3871 |
| 2827 | S3_88788894 | 3 | 88788894 | 1375204 | C | T | T | 0.51613 | C | 0.48387 | 14 | 0.45161 |
| 2828 | S3_88788935 | 3 | 88788935 | 41 | C | A | A | 0.53226 | C | 0.46774 | 13 | 0.41935 |
| 2829 | S3_88788961 | 3 | 88788961 | 26 | G | A | A | 0.53226 | G | 0.46774 | 13 | 0.41935 |
| 2830 | S3_88789008 | 3 | 88789008 | 47 | C | T | C | 0.53226 | T | 0.46774 | 15 | 0.48387 |
| 2831 | S3_88789050 | 3 | 88789050 | 42 | G | A | A | 0.53226 | G | 0.46774 | 13 | 0.41935 |
| 2832 | S3_88789080 | 3 | 88789080 | 30 | G | T | T | 0.53226 | G | 0.46774 | 13 | 0.41935 |
| 2833 | S3_89076379 | 3 | 89076379 | 287299 | C | T | C | 0.91935 | T | 0.08065 | 5 | 0.16129 |
| 2834 | S3_89076383 | 3 | 89076383 | 4 | C | A | C | 0.91935 | A | 0.08065 | 5 | 0.16129 |
| 2835 | S3_89159579 | 3 | 89159579 | 83196 | C | T | T | 0.95161 | C | 0.04839 | 3 | 0.09677 |
| 2836 | S3_89437466 | 3 | 89437466 | 277887 | C | T | C | 0.70968 | T | 0.29032 | 12 | 0.3871 |
| 2837 | S3_89883832 | 3 | 89883832 | 446366 | C | T | C | 0.5 | T | 0.5 | 13 | 0.41935 |
| 2838 | S3_89996904 | 3 | 89996904 | 113072 | C | G | C | 0.79032 | G | 0.20968 | 11 | 0.35484 |
| 2839 | S3_89997027 | 3 | 89997027 | 123 | G | A | G | 0.79032 | A | 0.20968 | 11 | 0.35484 |
| 2840 | S3_90056264 | 3 | 90056264 | 59237 | T | C | C | 0.74194 | T | 0.25806 | 14 | 0.45161 |
| 2841 | S3_90216097 | 3 | 90216097 | 159833 | T | C | T | 0.91935 | C | 0.08065 | 3 | 0.09677 |
| 2842 | S3_90629088 | 3 | 90629088 | 412991 | T | C | C | 0.72581 | T | 0.27419 | 11 | 0.35484 |
| 2843 | S3_90629090 | 3 | 90629090 | 2 | G | T | G | 0.85484 | T | 0.14516 | 7 | 0.22581 |
| 2844 | S3_90629285 | 3 | 90629285 | 195 | A | G | A | 0.83871 | G | 0.16129 | 10 | 0.32258 |
| 2845 | S3_91082563 | 3 | 91082563 | 453278 | T | C | T | 0.85484 | C | 0.14516 | 7 | 0.22581 |
| 2846 | S3_91082598 | 3 | 91082598 | 35 | A | C | A | 0.85484 | C | 0.14516 | 7 | 0.22581 |
| 2847 | S3_91082643 | 3 | 91082643 | 45 | G | A | G | 0.79032 | A | 0.20968 | 11 | 0.35484 |
| 2848 | S3_91088726 | 3 | 91088726 | 6083 | T | A | T | 0.82258 | A | 0.17742 | 11 | 0.35484 |
| 2849 | S3_91088864 | 3 | 91088864 | 138 | C | T | C | 0.90323 | T | 0.09677 | 4 | 0.12903 |
| 2850 | S3_91859794 | 3 | 91859794 | 770930 | C | T | C | 0.91935 | T | 0.08065 | 5 | 0.16129 |
| 2851 | S3_92140665 | 3 | 92140665 | 280871 | C | A | C | 0.64516 | A | 0.35484 | 12 | 0.3871 |
| 2852 | S3_92230828 | 3 | 92230828 | 90163 | C | T | C | 0.93548 | T | 0.06452 | 2 | 0.06452 |
| 2853 | S3_92406301 | 3 | 92406301 | 175473 | C | T | C | 0.87097 | T | 0.12903 | 8 | 0.25806 |
| 2854 | S3_92406395 | 3 | 92406395 | 94 | T | C | C | 0.85484 | T | 0.14516 | 9 | 0.29032 |
| 2855 | S3_92767758 | 3 | 92767758 | 361363 | G | A | G | 0.80645 | A | 0.19355 | 12 | 0.3871 |
| 2856 | S3_92767852 | 3 | 92767852 | 94 | G | A | G | 0.8871 | A | 0.1129 | 5 | 0.16129 |
| 2857 | S3_92767948 | 3 | 92767948 | 96 | G | A | A | 0.79032 | G | 0.20968 | 9 | 0.29032 |
| 2858 | S3_92770187 | 3 | 92770187 | 2239 | C | T | C | 0.90323 | T | 0.09677 | 4 | 0.12903 |
| 2859 | S3_92770379 | 3 | 92770379 | 192 | A | G | A | 0.59677 | G | 0.40323 | 13 | 0.41935 |
| 2860 | S3_92811172 | 3 | 92811172 | 40793 | T | C | C | 0.6129 | T | 0.3871 | 14 | 0.45161 |

| Site number | SNP name | Chromosome | Physical position | Physical distance from the previous SNP | Reference allele | Alternative allele | Major allele | Major allele frequency | Minor allele | Minor allele frequency | Number heterozygous | Heterozygosity |
|---|---|---|---|---|---|---|---|---|---|---|---|---|
| 2861 | S3_92811181 | 3 | 92811181 | 9 | A | G | G | 0.6129 | A | 0.3871 | 14 | 0.45161 |
| 2862 | S3_92811210 | 3 | 92811210 | 29 | T | C | C | 0.6129 | T | 0.3871 | 14 | 0.45161 |
| 2863 | S3_92811279 | 3 | 92811279 | 69 | A | G | A | 0.93548 | G | 0.06452 | 2 | 0.06452 |
| 2864 | S3_92811304 | 3 | 92811304 | 25 | T | C | T | 0.93548 | C | 0.06452 | 4 | 0.12903 |
| 2865 | S3_92811321 | 3 | 92811321 | 17 | T | C | C | 0.62903 | T | 0.37097 | 13 | 0.41935 |
| 2866 | S3_92812980 | 3 | 92812980 | 1659 | G | A | G | 0.79032 | A | 0.20968 | 11 | 0.35484 |
| 2867 | S3_92813027 | 3 | 92813027 | 47 | T | C | C | 0.6129 | T | 0.3871 | 14 | 0.45161 |
| 2868 | S3_92813088 | 3 | 92813088 | 61 | A | G | A | 0.6129 | G | 0.3871 | 14 | 0.45161 |
| 2869 | S3_92813091 | 3 | 92813091 | 3 | T | C | T | 0.6129 | C | 0.3871 | 14 | 0.45161 |
| 2870 | S3_92813164 | 3 | 92813164 | 73 | T | C | T | 0.90323 | C | 0.09677 | 6 | 0.19355 |
| 2871 | S3_92813168 | 3 | 92813168 | 4 | T | C | C | 0.6129 | T | 0.3871 | 14 | 0.45161 |
| 2872 | S3_92813178 | 3 | 92813178 | 10 | A | G | G | 0.6129 | A | 0.3871 | 14 | 0.45161 |
| 2873 | S3_92850089 | 3 | 92850089 | 36911 | A | G | A | 0.5 | G | 0.5 | 15 | 0.48387 |
| 2874 | S3_92850200 | 3 | 92850200 | 111 | C | T | C | 0.93548 | T | 0.06452 | 4 | 0.12903 |
| 2875 | S3_92850233 | 3 | 92850233 | 33 | T | C | C | 0.90323 | T | 0.09677 | 4 | 0.12903 |
| 2876 | S3_92850274 | 3 | 92850274 | 41 | C | T | C | 0.91935 | T | 0.08065 | 5 | 0.16129 |
| 2877 | S3_93115457 | 3 | 93115457 | 265183 | C | T | C | 0.85484 | T | 0.14516 | 9 | 0.29032 |
| 2878 | S3_93115513 | 3 | 93115513 | 56 | A | C | A | 0.66129 | C | 0.33871 | 13 | 0.41935 |
| 2879 | S3_93115514 | 3 | 93115514 | 1 | T | C | T | 0.66129 | C | 0.33871 | 13 | 0.41935 |
| 2880 | S3_93115544 | 3 | 93115544 | 30 | A | C | A | 0.90323 | C | 0.09677 | 6 | 0.19355 |
| 2881 | S3_93115570 | 3 | 93115570 | 26 | T | C | T | 0.66129 | C | 0.33871 | 13 | 0.41935 |
| 2882 | S3_93274794 | 3 | 93274794 | 159224 | C | T | C | 0.77419 | T | 0.22581 | 10 | 0.32258 |
| 2883 | S3_93317417 | 3 | 93317417 | 42623 | G | A | G | 0.85484 | A | 0.14516 | 7 | 0.22581 |
| 2884 | S3_94215783 | 3 | 94215783 | 898366 | T | G | T | 0.67742 | G | 0.32258 | 14 | 0.45161 |
| 2885 | S3_94411896 | 3 | 94411896 | 196113 | G | A | G | 0.8871 | A | 0.1129 | 7 | 0.22581 |
| 2886 | S3_94477522 | 3 | 94477522 | 65626 | G | A | G | 0.83871 | A | 0.16129 | 8 | 0.25806 |
| 2887 | S3_94477634 | 3 | 94477634 | 112 | A | G | A | 0.83871 | G | 0.16129 | 8 | 0.25806 |
| 2888 | S3_95125709 | 3 | 95125709 | 648075 | G | A | G | 0.93548 | A | 0.06452 | 4 | 0.12903 |
| 2889 | S3_95265915 | 3 | 95265915 | 140206 | G | A | G | 0.95161 | A | 0.04839 | 3 | 0.09677 |
| 2890 | S3_95285940 | 3 | 95285940 | 20025 | A | T | A | 0.93548 | T | 0.06452 | 2 | 0.06452 |
| 2891 | S3_95400578 | 3 | 95400578 | 114638 | C | T | C | 0.77419 | T | 0.22581 | 12 | 0.3871 |
| 2892 | S3_95400601 | 3 | 95400601 | 23 | G | A | G | 0.95161 | A | 0.04839 | 3 | 0.09677 |
| 2893 | S3_95930417 | 3 | 95930417 | 529816 | G | A | G | 0.90323 | A | 0.09677 | 6 | 0.19355 |
| 2894 | S3_96211856 | 3 | 96211856 | 281439 | T | A | T | 0.67742 | A | 0.32258 | 14 | 0.45161 |
| 2895 | S3_96334526 | 3 | 96334526 | 122670 | G | A | A | 0.59677 | G | 0.40323 | 19 | 0.6129 |
| 2896 | S3_96462580 | 3 | 96462580 | 128054 | T | C | T | 0.64516 | C | 0.35484 | 18 | 0.58065 |
| 2897 | S3_96462674 | 3 | 96462674 | 94 | T | A | T | 0.82258 | A | 0.17742 | 11 | 0.35484 |
| 2898 | S3_97119216 | 3 | 97119216 | 656542 | G | A | G | 0.70968 | A | 0.29032 | 14 | 0.45161 |
| 2899 | S3_97387391 | 3 | 97387391 | 268175 | A | G | G | 0.75806 | A | 0.24194 | 13 | 0.41935 |
| 2900 | S3_97387470 | 3 | 97387470 | 79 | A | G | G | 0.74194 | A | 0.25806 | 14 | 0.45161 |
| 2901 | S3_97387568 | 3 | 97387568 | 98 | G | A | G | 0.74194 | A | 0.25806 | 8 | 0.25806 |
| 2902 | S3_97591371 | 3 | 97591371 | 203803 | G | A | G | 0.51613 | A | 0.48387 | 16 | 0.51613 |
| 2903 | S3_97591395 | 3 | 97591395 | 24 | T | C | T | 0.51613 | C | 0.48387 | 16 | 0.51613 |
| 2904 | S3_97591521 | 3 | 97591521 | 126 | C | T | C | 0.82258 | T | 0.17742 | 9 | 0.29032 |
| 2905 | S3_97894142 | 3 | 97894142 | 302621 | G | T | G | 0.8871 | T | 0.1129 | 7 | 0.22581 |
| 2906 | S3_97894338 | 3 | 97894338 | 196 | G | T | G | 0.8871 | T | 0.1129 | 7 | 0.22581 |
| 2907 | S3_98429771 | 3 | 98429771 | 535433 | A | C | A | 0.77419 | C | 0.22581 | 10 | 0.32258 |
| 2908 | S3_98429772 | 3 | 98429772 | 1 | A | T | A | 0.77419 | T | 0.22581 | 10 | 0.32258 |
| 2909 | S3_98453133 | 3 | 98453133 | 23361 | G | A | G | 0.75806 | A | 0.24194 | 11 | 0.35484 |
| 2910 | S3_98580261 | 3 | 98580261 | 127128 | A | G | A | 0.8871 | G | 0.1129 | 7 | 0.22581 |
| 2911 | S3_98580271 | 3 | 98580271 | 10 | C | T | C | 0.95161 | T | 0.04839 | 3 | 0.09677 |
| 2912 | S3_98627826 | 3 | 98627826 | 47555 | G | A | A | 0.79032 | G | 0.20968 | 11 | 0.35484 |
| 2913 | S3_98627877 | 3 | 98627877 | 51 | C | T | C | 0.90323 | T | 0.09677 | 4 | 0.12903 |
| 2914 | S3_98764373 | 3 | 98764373 | 136496 | A | G | A | 0.91935 | G | 0.08065 | 5 | 0.16129 |

| Site number | SNP name | Chromosome | Physical position | Physical distance from the previous SNP | Reference allele | Alternative allele | Major allele | Major allele frequency | Minor allele | Minor allele frequency | Number heterozygous | Heterozygosity |
|---|---|---|---|---|---|---|---|---|---|---|---|---|
| 2915 | S3_100151793 | 3 | 100151793 | 1387420 | T | C | T | 0.58065 | C | 0.41935 | 14 | 0.45161 |
| 2916 | S3_100327071 | 3 | 100327071 | 175278 | T | C | T | 0.83871 | C | 0.16129 | 6 | 0.19355 |
| 2917 | S3_100342973 | 3 | 100342973 | 15902 | C | G | C | 0.62903 | G | 0.37097 | 19 | 0.6129 |
| 2918 | S3_100577734 | 3 | 100577734 | 234761 | T | C | T | 0.74194 | C | 0.25806 | 12 | 0.3871 |
| 2919 | S3_100853064 | 3 | 100853064 | 275330 | G | A | G | 0.91935 | A | 0.08065 | 3 | 0.09677 |
| 2920 | S3_100853198 | 3 | 100853198 | 134 | C | T | C | 0.91935 | T | 0.08065 | 3 | 0.09677 |
| 2921 | S3_100853220 | 3 | 100853220 | 22 | A | G | G | 0.93548 | A | 0.06452 | 4 | 0.12903 |
| 2922 | S3_100964905 | 3 | 100964905 | 111685 | C | T | T | 0.83871 | C | 0.16129 | 8 | 0.25806 |
| 2923 | S3_101379658 | 3 | 101379658 | 414753 | A | G | A | 0.87097 | G | 0.12903 | 8 | 0.25806 |
| 2924 | S3_101379799 | 3 | 101379799 | 141 | T | C | T | 0.93548 | C | 0.06452 | 4 | 0.12903 |
| 2925 | S3_101402665 | 3 | 101402665 | 22866 | A | T | A | 0.8871 | T | 0.1129 | 7 | 0.22581 |
| 2926 | S3_101402667 | 3 | 101402667 | 2 | A | G | A | 0.66129 | G | 0.33871 | 15 | 0.48387 |
| 2927 | S3_101402711 | 3 | 101402711 | 44 | A | G | A | 0.51613 | G | 0.48387 | 16 | 0.51613 |
| 2928 | S3_101552999 | 3 | 101552999 | 150288 | G | A | G | 0.77419 | A | 0.22581 | 14 | 0.45161 |
| 2929 | S3_101621708 | 3 | 101621708 | 68709 | G | T | G | 0.90323 | T | 0.09677 | 6 | 0.19355 |
| 2930 | S3_101621715 | 3 | 101621715 | 7 | G | A | G | 0.8871 | A | 0.1129 | 7 | 0.22581 |
| 2931 | S3_101629501 | 3 | 101629501 | 7786 | C | A | C | 0.90323 | A | 0.09677 | 6 | 0.19355 |
| 2932 | S3_101813818 | 3 | 101813818 | 184317 | A | G | A | 0.90323 | G | 0.09677 | 6 | 0.19355 |
| 2933 | S3_101813821 | 3 | 101813821 | 3 | G | T | G | 0.90323 | T | 0.09677 | 6 | 0.19355 |
| 2934 | S3_101813923 | 3 | 101813923 | 102 | C | T | C | 0.90323 | T | 0.09677 | 6 | 0.19355 |
| 2935 | S3_101813957 | 3 | 101813957 | 34 | T | C | T | 0.90323 | C | 0.09677 | 6 | 0.19355 |
| 2936 | S3_101813961 | 3 | 101813961 | 4 | C | G | C | 0.90323 | G | 0.09677 | 6 | 0.19355 |
| 2937 | S3_102399307 | 3 | 102399307 | 585346 | C | T | T | 0.87097 | C | 0.12903 | 8 | 0.25806 |
| 2938 | S3_102399349 | 3 | 102399349 | 42 | C | T | C | 0.82258 | T | 0.17742 | 9 | 0.29032 |
| 2939 | S3_102399474 | 3 | 102399474 | 125 | C | G | C | 0.87097 | G | 0.12903 | 8 | 0.25806 |
| 2940 | S3_102655420 | 3 | 102655420 | 255946 | C | A | C | 0.93548 | A | 0.06452 | 4 | 0.12903 |
| 2941 | S3_102710076 | 3 | 102710076 | 54656 | A | G | A | 0.95161 | G | 0.04839 | 3 | 0.09677 |
| 2942 | S3_102710188 | 3 | 102710188 | 112 | C | T | C | 0.95161 | T | 0.04839 | 3 | 0.09677 |
| 2943 | S3_102773961 | 3 | 102773961 | 63773 | A | G | A | 0.95161 | G | 0.04839 | 3 | 0.09677 |
| 2944 | S3_102774040 | 3 | 102774040 | 79 | C | A | C | 0.93548 | A | 0.06452 | 4 | 0.12903 |
| 2945 | S3_102774108 | 3 | 102774108 | 68 | A | G | G | 0.90323 | A | 0.09677 | 6 | 0.19355 |
| 2946 | S3_102789810 | 3 | 102789810 | 15702 | G | T | G | 0.83871 | T | 0.16129 | 8 | 0.25806 |
| 2947 | S3_102919761 | 3 | 102919761 | 129951 | A | C | A | 0.93548 | C | 0.06452 | 4 | 0.12903 |
| 2948 | S3_103066948 | 3 | 103066948 | 147187 | C | T | C | 0.90323 | T | 0.09677 | 6 | 0.19355 |
| 2949 | S3_103096787 | 3 | 103096787 | 29839 | A | C | A | 0.90323 | C | 0.09677 | 6 | 0.19355 |
| 2950 | S3_103096836 | 3 | 103096836 | 49 | C | T | C | 0.90323 | T | 0.09677 | 6 | 0.19355 |
| 2951 | S3_103096941 | 3 | 103096941 | 105 | G | A | G | 0.90323 | A | 0.09677 | 6 | 0.19355 |
| 2952 | S3_103096982 | 3 | 103096982 | 41 | A | G | A | 0.95161 | G | 0.04839 | 3 | 0.09677 |
| 2953 | S3_103097677 | 3 | 103097677 | 695 | G | A | G | 0.8871 | A | 0.1129 | 7 | 0.22581 |
| 2954 | S3_103097795 | 3 | 103097795 | 118 | A | C | A | 0.95161 | C | 0.04839 | 3 | 0.09677 |
| 2955 | S3_103097888 | 3 | 103097888 | 93 | C | T | C | 0.93548 | T | 0.06452 | 4 | 0.12903 |
| 2956 | S3_103479401 | 3 | 103479401 | 381513 | G | A | G | 0.95161 | A | 0.04839 | 3 | 0.09677 |
| 2957 | S3_103479442 | 3 | 103479442 | 41 | G | A | G | 0.95161 | A | 0.04839 | 3 | 0.09677 |
| 2958 | S3_103539168 | 3 | 103539168 | 59726 | C | T | T | 0.54839 | C | 0.45161 | 16 | 0.51613 |
| 2959 | S3_103732200 | 3 | 103732200 | 193032 | A | G | A | 0.82258 | G | 0.17742 | 7 | 0.22581 |
| 2960 | S3_103991341 | 3 | 103991341 | 259141 | G | A | A | 0.51613 | G | 0.48387 | 20 | 0.64516 |
| 2961 | S3_103991462 | 3 | 103991462 | 121 | G | T | G | 0.91935 | T | 0.08065 | 5 | 0.16129 |
| 2962 | S3_104029649 | 3 | 104029649 | 38187 | G | A | G | 0.90323 | A | 0.09677 | 6 | 0.19355 |
| 2963 | S3_104029757 | 3 | 104029757 | 108 | C | G | C | 0.93548 | G | 0.06452 | 4 | 0.12903 |
| 2964 | S3_104105475 | 3 | 104105475 | 75718 | G | T | G | 0.74194 | T | 0.25806 | 12 | 0.3871 |
| 2965 | S3_104436353 | 3 | 104436353 | 330878 | G | A | G | 0.93548 | A | 0.06452 | 4 | 0.12903 |
| 2966 | S3_104436394 | 3 | 104436394 | 41 | A | G | A | 0.70968 | G | 0.29032 | 14 | 0.45161 |
| 2967 | S3_104475816 | 3 | 104475816 | 39422 | C | T | T | 0.59677 | C | 0.40323 | 17 | 0.54839 |
| 2968 | S3_105463187 | 3 | 105463187 | 987371 | C | G | C | 0.91935 | G | 0.08065 | 5 | 0.16129 |

| Site number | SNP name | Chromosome | Physical position | Physical distance from the previous SNP | Reference allele | Alternative allele | Major allele | Major allele frequency | Minor allele | Minor allele frequency | Number heterozygous | Heterozygosity |
|---|---|---|---|---|---|---|---|---|---|---|---|---|
| 2969 | S3_105463188 | 3 | 105463188 | 1 | G | A | G | 0.6129 | A | 0.3871 | 14 | 0.45161 |
| 2970 | S3_105751963 | 3 | 105751963 | 288775 | T | A | A | 0.93548 | T | 0.06452 | 4 | 0.12903 |
| 2971 | S3_105752083 | 3 | 105752083 | 120 | C | T | C | 0.87097 | T | 0.12903 | 6 | 0.19355 |
| 2972 | S3_105752115 | 3 | 105752115 | 32 | G | A | A | 0.93548 | G | 0.06452 | 4 | 0.12903 |
| 2973 | S3_106274913 | 3 | 106274913 | 522798 | C | T | C | 0.87097 | T | 0.12903 | 8 | 0.25806 |
| 2974 | S3_106274964 | 3 | 106274964 | 51 | C | T | C | 0.82258 | T | 0.17742 | 7 | 0.22581 |
| 2975 | S3_106274972 | 3 | 106274972 | 8 | A | G | G | 0.69355 | A | 0.30645 | 11 | 0.35484 |
| 2976 | S3_106794871 | 3 | 106794871 | 519899 | A | C | A | 0.72581 | C | 0.27419 | 17 | 0.54839 |
| 2977 | S3_106794915 | 3 | 106794915 | 44 | T | C | C | 0.93548 | T | 0.06452 | 4 | 0.12903 |
| 2978 | S3_106795003 | 3 | 106795003 | 88 | T | C | T | 0.93548 | C | 0.06452 | 4 | 0.12903 |
| 2979 | S3_106795082 | 3 | 106795082 | 79 | C | T | C | 0.75806 | T | 0.24194 | 15 | 0.48387 |
| 2980 | S3_106795117 | 3 | 106795117 | 35 | A | C | A | 0.77419 | C | 0.22581 | 14 | 0.45161 |
| 2981 | S3_106970793 | 3 | 106970793 | 175676 | C | T | C | 0.54839 | T | 0.45161 | 12 | 0.3871 |
| 2982 | S3_107884825 | 3 | 107884825 | 914032 | T | C | C | 0.85484 | T | 0.14516 | 9 | 0.29032 |
| 2983 | S3_107884839 | 3 | 107884839 | 14 | C | T | C | 0.93548 | T | 0.06452 | 4 | 0.12903 |
| 2984 | S3_107884898 | 3 | 107884898 | 59 | C | T | C | 0.93548 | T | 0.06452 | 4 | 0.12903 |
| 2985 | S3_107884928 | 3 | 107884928 | 30 | G | A | G | 0.75806 | A | 0.24194 | 11 | 0.35484 |
| 2986 | S3_107885003 | 3 | 107885003 | 75 | G | A | G | 0.74194 | A | 0.25806 | 12 | 0.3871 |
| 2987 | S3_107885004 | 3 | 107885004 | 1 | C | T | C | 0.75806 | T | 0.24194 | 11 | 0.35484 |
| 2988 | S3_109436902 | 3 | 109436902 | 1551898 | T | C | T | 0.54839 | C | 0.45161 | 16 | 0.51613 |
| 2989 | S3_109756883 | 3 | 109756883 | 319981 | A | G | G | 0.70968 | A | 0.29032 | 14 | 0.45161 |
| 2990 | S3_109756886 | 3 | 109756886 | 3 | C | T | C | 0.74194 | T | 0.25806 | 12 | 0.3871 |
| 2991 | S3_109756888 | 3 | 109756888 | 2 | C | T | C | 0.74194 | T | 0.25806 | 12 | 0.3871 |
| 2992 | S3_109756891 | 3 | 109756891 | 3 | C | T | C | 0.74194 | T | 0.25806 | 12 | 0.3871 |
| 2993 | S3_109756958 | 3 | 109756958 | 67 | G | T | G | 0.74194 | T | 0.25806 | 12 | 0.3871 |
| 2994 | S3_109757033 | 3 | 109757033 | 75 | G | A | G | 0.93548 | A | 0.06452 | 4 | 0.12903 |
| 2995 | S3_109757036 | 3 | 109757036 | 3 | G | C | C | 0.56452 | G | 0.43548 | 15 | 0.48387 |
| 2996 | S3_109757090 | 3 | 109757090 | 54 | C | T | T | 0.56452 | C | 0.43548 | 15 | 0.48387 |
| 2997 | S3_109911000 | 3 | 109911000 | 153910 | G | A | G | 0.64516 | A | 0.35484 | 16 | 0.51613 |
| 2998 | S3_109911055 | 3 | 109911055 | 55 | G | A | G | 0.69355 | A | 0.30645 | 15 | 0.48387 |
| 2999 | S3_109911110 | 3 | 109911110 | 55 | C | T | C | 0.93548 | T | 0.06452 | 4 | 0.12903 |
| 3000 | S3_110190101 | 3 | 110190101 | 278991 | A | T | A | 0.82258 | T | 0.17742 | 9 | 0.29032 |
| 3001 | S3_110190141 | 3 | 110190141 | 40 | T | A | T | 0.91935 | A | 0.08065 | 5 | 0.16129 |
| 3002 | S3_110190215 | 3 | 110190215 | 74 | G | A | G | 0.91935 | A | 0.08065 | 5 | 0.16129 |
| 3003 | S3_110190329 | 3 | 110190329 | 114 | T | C | T | 0.91935 | C | 0.08065 | 5 | 0.16129 |
| 3004 | S3_110190330 | 3 | 110190330 | 1 | G | A | G | 0.91935 | A | 0.08065 | 5 | 0.16129 |
| 3005 | S3_110565553 | 3 | 110565553 | 375223 | T | C | T | 0.87097 | C | 0.12903 | 8 | 0.25806 |
| 3006 | S3_111005349 | 3 | 111005349 | 439796 | G | C | C | 0.91935 | G | 0.08065 | 3 | 0.09677 |
| 3007 | S3_111264183 | 3 | 111264183 | 258834 | G | A | G | 0.91935 | A | 0.08065 | 5 | 0.16129 |
| 3008 | S3_112050500 | 3 | 112050500 | 786317 | T | C | T | 0.75806 | C | 0.24194 | 11 | 0.35484 |
| 3009 | S3_112050671 | 3 | 112050671 | 171 | C | A | A | 0.82258 | C | 0.17742 | 11 | 0.35484 |
| 3010 | S3_112818200 | 3 | 112818200 | 767529 | C | T | C | 0.66129 | T | 0.33871 | 11 | 0.35484 |
| 3011 | S3_112818263 | 3 | 112818263 | 63 | A | G | A | 0.8871 | G | 0.1129 | 7 | 0.22581 |
| 3012 | S3_112890236 | 3 | 112890236 | 71973 | A | G | G | 0.56452 | A | 0.43548 | 13 | 0.41935 |
| 3013 | S3_112890238 | 3 | 112890238 | 2 | A | G | A | 0.74194 | G | 0.25806 | 12 | 0.3871 |
| 3014 | S3_112890242 | 3 | 112890242 | 4 | G | T | G | 0.93548 | T | 0.06452 | 2 | 0.06452 |
| 3015 | S3_112890246 | 3 | 112890246 | 4 | G | A | G | 0.93548 | A | 0.06452 | 2 | 0.06452 |
| 3016 | S3_112923912 | 3 | 112923912 | 33666 | T | C | T | 0.93548 | C | 0.06452 | 4 | 0.12903 |
| 3017 | S3_112923915 | 3 | 112923915 | 3 | C | T | C | 0.93548 | T | 0.06452 | 4 | 0.12903 |
| 3018 | S3_112972558 | 3 | 112972558 | 48643 | T | C | C | 0.80645 | T | 0.19355 | 8 | 0.25806 |
| 3019 | S3_112972596 | 3 | 112972596 | 38 | G | A | G | 0.95161 | A | 0.04839 | 3 | 0.09677 |
| 3020 | S3_112972620 | 3 | 112972620 | 24 | A | G | A | 0.85484 | G | 0.14516 | 5 | 0.16129 |
| 3021 | S3_112972659 | 3 | 112972659 | 39 | A | G | A | 0.85484 | G | 0.14516 | 5 | 0.16129 |
| 3022 | S3_113249541 | 3 | 113249541 | 276882 | G | C | G | 0.90323 | C | 0.09677 | 6 | 0.19355 |

| Site number | SNP name | Chromosome | Physical position | Physical distance from the previous SNP | Reference allele | Alternative allele | Major allele | Major allele frequency | Minor allele | Minor allele frequency | Number heterozygous | Heterozygosity |
|---|---|---|---|---|---|---|---|---|---|---|---|---|
| 3023 | S3_113249622 | 3 | 113249622 | 81 | G | A | G | 0.93548 | A | 0.06452 | 2 | 0.06452 |
| 3024 | S3_113249660 | 3 | 113249660 | 38 | G | A | G | 0.79032 | A | 0.20968 | 11 | 0.35484 |
| 3025 | S3_114042970 | 3 | 114042970 | 793310 | C | T | C | 0.90323 | T | 0.09677 | 6 | 0.19355 |
| 3026 | S3_114043124 | 3 | 114043124 | 154 | C | T | C | 0.93548 | T | 0.06452 | 4 | 0.12903 |
| 3027 | S3_114043170 | 3 | 114043170 | 46 | T | C | T | 0.82258 | C | 0.17742 | 9 | 0.29032 |
| 3028 | S3_114334614 | 3 | 114334614 | 291444 | G | C | G | 0.87097 | C | 0.12903 | 8 | 0.25806 |
| 3029 | S3_114381274 | 3 | 114381274 | 46660 | A | G | G | 0.75806 | A | 0.24194 | 11 | 0.35484 |
| 3030 | S3_114381445 | 3 | 114381445 | 171 | A | G | G | 0.93548 | A | 0.06452 | 4 | 0.12903 |
| 3031 | S3_115273690 | 3 | 115273690 | 892245 | A | C | A | 0.91935 | C | 0.08065 | 5 | 0.16129 |
| 3032 | S3_115294524 | 3 | 115294524 | 20834 | T | G | G | 0.82258 | T | 0.17742 | 11 | 0.35484 |
| 3033 | S3_115416302 | 3 | 115416302 | 121778 | T | A | T | 0.93548 | A | 0.06452 | 4 | 0.12903 |
| 3034 | S3_115847187 | 3 | 115847187 | 430885 | A | G | A | 0.72581 | G | 0.27419 | 9 | 0.29032 |
| 3035 | S3_116098213 | 3 | 116098213 | 251026 | G | A | G | 0.80645 | A | 0.19355 | 8 | 0.25806 |
| 3036 | S3_116219366 | 3 | 116219366 | 121153 | C | T | C | 0.90323 | T | 0.09677 | 4 | 0.12903 |
| 3037 | S3_116219384 | 3 | 116219384 | 18 | G | A | G | 0.75806 | A | 0.24194 | 11 | 0.35484 |
| 3038 | S3_116219431 | 3 | 116219431 | 47 | T | C | T | 0.83871 | C | 0.16129 | 8 | 0.25806 |
| 3039 | S3_116239470 | 3 | 116239470 | 20039 | A | G | A | 0.85484 | G | 0.14516 | 7 | 0.22581 |
| 3040 | S3_116239473 | 3 | 116239473 | 3 | C | T | C | 0.90323 | T | 0.09677 | 6 | 0.19355 |
| 3041 | S3_116239530 | 3 | 116239530 | 57 | G | T | G | 0.90323 | T | 0.09677 | 6 | 0.19355 |
| 3042 | S3_116239548 | 3 | 116239548 | 18 | A | G | A | 0.95161 | G | 0.04839 | 3 | 0.09677 |
| 3043 | S3_116417001 | 3 | 116417001 | 177453 | C | G | C | 0.77419 | G | 0.22581 | 12 | 0.3871 |
| 3044 | S3_116562463 | 3 | 116562463 | 145462 | T | C | T | 0.95161 | C | 0.04839 | 3 | 0.09677 |
| 3045 | S3_116562489 | 3 | 116562489 | 26 | G | A | A | 0.77419 | G | 0.22581 | 14 | 0.45161 |
| 3046 | S3_116562689 | 3 | 116562689 | 200 | G | A | G | 0.85484 | A | 0.14516 | 7 | 0.22581 |
| 3047 | S3_116744696 | 3 | 116744696 | 182007 | G | A | G | 0.82258 | A | 0.17742 | 7 | 0.22581 |
| 3048 | S3_116781857 | 3 | 116781857 | 37161 | T | A | T | 0.82258 | A | 0.17742 | 9 | 0.29032 |
| 3049 | S3_116781864 | 3 | 116781864 | 7 | C | T | C | 0.85484 | T | 0.14516 | 7 | 0.22581 |
| 3050 | S3_116781923 | 3 | 116781923 | 59 | G | A | G | 0.85484 | A | 0.14516 | 7 | 0.22581 |
| 3051 | S3_116782038 | 3 | 116782038 | 115 | G | A | G | 0.69355 | A | 0.30645 | 13 | 0.41935 |
| 3052 | S3_116833346 | 3 | 116833346 | 51308 | A | G | G | 0.66129 | A | 0.33871 | 15 | 0.48387 |
| 3053 | S3_116833431 | 3 | 116833431 | 85 | G | A | G | 0.80645 | A | 0.19355 | 10 | 0.32258 |
| 3054 | S3_116833448 | 3 | 116833448 | 17 | A | G | A | 0.80645 | G | 0.19355 | 10 | 0.32258 |
| 3055 | S3_116833482 | 3 | 116833482 | 34 | C | T | C | 0.85484 | T | 0.14516 | 7 | 0.22581 |
| 3056 | S3_116833509 | 3 | 116833509 | 27 | A | G | A | 0.80645 | G | 0.19355 | 10 | 0.32258 |
| 3057 | S3_116916865 | 3 | 116916865 | 83356 | G | A | G | 0.90323 | A | 0.09677 | 6 | 0.19355 |
| 3058 | S3_116916974 | 3 | 116916974 | 109 | A | C | A | 0.54839 | C | 0.45161 | 14 | 0.45161 |
| 3059 | S3_117150888 | 3 | 117150888 | 233914 | G | A | G | 0.82258 | A | 0.17742 | 11 | 0.35484 |
| 3060 | S3_117379481 | 3 | 117379481 | 228593 | A | G | A | 0.95161 | G | 0.04839 | 3 | 0.09677 |
| 3061 | S3_117379519 | 3 | 117379519 | 38 | C | T | C | 0.95161 | T | 0.04839 | 3 | 0.09677 |
| 3062 | S3_118524425 | 3 | 118524425 | 1144906 | A | G | G | 0.93548 | A | 0.06452 | 4 | 0.12903 |
| 3063 | S3_118668435 | 3 | 118668435 | 144010 | T | C | T | 0.85484 | C | 0.14516 | 9 | 0.29032 |
| 3064 | S3_118668439 | 3 | 118668439 | 4 | A | G | A | 0.8871 | G | 0.1129 | 7 | 0.22581 |
| 3065 | S3_118668521 | 3 | 118668521 | 82 | A | G | A | 0.90323 | G | 0.09677 | 6 | 0.19355 |
| 3066 | S3_118729174 | 3 | 118729174 | 60653 | G | A | A | 0.95161 | G | 0.04839 | 3 | 0.09677 |
| 3067 | S3_118833706 | 3 | 118833706 | 104532 | C | T | C | 0.51613 | T | 0.48387 | 10 | 0.32258 |
| 3068 | S3_118833828 | 3 | 118833828 | 122 | G | A | G | 0.62903 | A | 0.37097 | 17 | 0.54839 |
| 3069 | S3_118859839 | 3 | 118859839 | 26011 | C | T | C | 0.54839 | T | 0.45161 | 14 | 0.45161 |
| 3070 | S3_118859849 | 3 | 118859849 | 10 | G | T | G | 0.91935 | T | 0.08065 | 5 | 0.16129 |
| 3071 | S4_332753 | 4 | 332753 | 0 | G | A | G | 0.69355 | A | 0.30645 | 11 | 0.35484 |
| 3072 | S4_550205 | 4 | 550205 | 217452 | T | C | T | 0.91935 | C | 0.08065 | 5 | 0.16129 |
| 3073 | S4_550293 | 4 | 550293 | 88 | G | C | G | 0.90323 | C | 0.09677 | 6 | 0.19355 |
| 3074 | S4_550357 | 4 | 550357 | 64 | A | G | A | 0.90323 | G | 0.09677 | 6 | 0.19355 |
| 3075 | S4_550371 | 4 | 550371 | 14 | G | A | G | 0.91935 | A | 0.08065 | 3 | 0.09677 |
| 3076 | S4_550403 | 4 | 550403 | 32 | C | T | C | 0.93548 | T | 0.06452 | 4 | 0.12903 |

| Site number | SNP name | Chromosome | Physical position | Physical distance from the previous SNP | Reference allele | Alternative allele | Major allele | Major allele frequency | Minor allele | Minor allele frequency | Number heterozygous | Heterozygosity |
|---|---|---|---|---|---|---|---|---|---|---|---|---|
| 3077 | S4_957798 | 4 | 957798 | 407395 | A | G | A | 0.62903 | G | 0.37097 | 15 | 0.48387 |
| 3078 | S4_1773855 | 4 | 1773855 | 816057 | A | G | A | 0.56452 | G | 0.43548 | 17 | 0.54839 |
| 3079 | S4_1884843 | 4 | 1884843 | 110988 | C | T | C | 0.85484 | T | 0.14516 | 9 | 0.29032 |
| 3080 | S4_1971517 | 4 | 1971517 | 86674 | C | T | C | 0.85484 | T | 0.14516 | 5 | 0.16129 |
| 3081 | S4_2104176 | 4 | 2104176 | 132659 | G | A | G | 0.90323 | A | 0.09677 | 4 | 0.12903 |
| 3082 | S4_2122830 | 4 | 2122830 | 18654 | G | A | G | 0.91935 | A | 0.08065 | 5 | 0.16129 |
| 3083 | S4_2123053 | 4 | 2123053 | 223 | G | A | G | 0.91935 | A | 0.08065 | 5 | 0.16129 |
| 3084 | S4_2126702 | 4 | 2126702 | 3649 | C | A | C | 0.91935 | A | 0.08065 | 5 | 0.16129 |
| 3085 | S4_2229434 | 4 | 2229434 | 102732 | G | A | G | 0.67742 | A | 0.32258 | 14 | 0.45161 |
| 3086 | S4_2310811 | 4 | 2310811 | 81377 | A | C | A | 0.70968 | C | 0.29032 | 14 | 0.45161 |
| 3087 | S4_2310829 | 4 | 2310829 | 18 | G | A | A | 0.79032 | G | 0.20968 | 11 | 0.35484 |
| 3088 | S4_2310834 | 4 | 2310834 | 5 | A | G | A | 0.75806 | G | 0.24194 | 9 | 0.29032 |
| 3089 | S4_2381747 | 4 | 2381747 | 70913 | G | A | G | 0.93548 | A | 0.06452 | 4 | 0.12903 |
| 3090 | S4_2381908 | 4 | 2381908 | 161 | G | A | G | 0.8871 | A | 0.1129 | 5 | 0.16129 |
| 3091 | S4_2548978 | 4 | 2548978 | 167070 | C | T | C | 0.91935 | T | 0.08065 | 3 | 0.09677 |
| 3092 | S4_2548979 | 4 | 2548979 | 1 | G | A | G | 0.83871 | A | 0.16129 | 8 | 0.25806 |
| 3093 | S4_2549047 | 4 | 2549047 | 68 | T | C | T | 0.80645 | C | 0.19355 | 6 | 0.19355 |
| 3094 | S4_2615469 | 4 | 2615469 | 66422 | C | T | C | 0.82258 | T | 0.17742 | 9 | 0.29032 |
| 3095 | S4_2615606 | 4 | 2615606 | 137 | G | T | G | 0.69355 | T | 0.30645 | 13 | 0.41935 |
| 3096 | S4_2615621 | 4 | 2615621 | 15 | C | G | C | 0.83871 | G | 0.16129 | 10 | 0.32258 |
| 3097 | S4_2718967 | 4 | 2718967 | 103346 | T | C | T | 0.90323 | C | 0.09677 | 6 | 0.19355 |
| 3098 | S4_2719003 | 4 | 2719003 | 36 | A | G | G | 0.67742 | A | 0.32258 | 18 | 0.58065 |
| 3099 | S4_2910204 | 4 | 2910204 | 191201 | C | A | A | 0.56452 | C | 0.43548 | 15 | 0.48387 |
| 3100 | S4_3032914 | 4 | 3032914 | 122710 | C | T | C | 0.93548 | T | 0.06452 | 2 | 0.06452 |
| 3101 | S4_3033029 | 4 | 3033029 | 115 | G | A | G | 0.66129 | A | 0.33871 | 13 | 0.41935 |
| 3102 | S4_3038486 | 4 | 3038486 | 5457 | A | G | A | 0.59677 | G | 0.40323 | 11 | 0.35484 |
| 3103 | S4_3038493 | 4 | 3038493 | 7 | T | C | T | 0.59677 | C | 0.40323 | 11 | 0.35484 |
| 3104 | S4_3132833 | 4 | 3132833 | 94340 | A | G | G | 0.90323 | A | 0.09677 | 4 | 0.12903 |
| 3105 | S4_3132834 | 4 | 3132834 | 1 | T | C | C | 0.90323 | T | 0.09677 | 4 | 0.12903 |
| 3106 | S4_3132841 | 4 | 3132841 | 7 | T | C | C | 0.90323 | T | 0.09677 | 4 | 0.12903 |
| 3107 | S4_3132948 | 4 | 3132948 | 107 | T | C | C | 0.90323 | T | 0.09677 | 4 | 0.12903 |
| 3108 | S4_3132990 | 4 | 3132990 | 42 | T | C | C | 0.90323 | T | 0.09677 | 4 | 0.12903 |
| 3109 | S4_3137638 | 4 | 3137638 | 4648 | C | T | T | 0.90323 | C | 0.09677 | 4 | 0.12903 |
| 3110 | S4_3137686 | 4 | 3137686 | 48 | G | A | A | 0.8871 | G | 0.1129 | 5 | 0.16129 |
| 3111 | S4_3144006 | 4 | 3144006 | 6320 | G | A | A | 0.64516 | G | 0.35484 | 16 | 0.51613 |
| 3112 | S4_3144015 | 4 | 3144015 | 9 | T | C | T | 0.93548 | C | 0.06452 | 4 | 0.12903 |
| 3113 | S4_3144075 | 4 | 3144075 | 60 | G | A | G | 0.70968 | A | 0.29032 | 12 | 0.3871 |
| 3114 | S4_3144087 | 4 | 3144087 | 12 | G | A | G | 0.80645 | A | 0.19355 | 12 | 0.3871 |
| 3115 | S4_3144141 | 4 | 3144141 | 54 | A | G | A | 0.93548 | G | 0.06452 | 4 | 0.12903 |
| 3116 | S4_3144235 | 4 | 3144235 | 94 | G | A | G | 0.59677 | A | 0.40323 | 11 | 0.35484 |
| 3117 | S4_3146580 | 4 | 3146580 | 2345 | G | A | G | 0.59677 | A | 0.40323 | 11 | 0.35484 |
| 3118 | S4_3164400 | 4 | 3164400 | 17820 | T | C | C | 0.66129 | T | 0.33871 | 19 | 0.6129 |
| 3119 | S4_3164413 | 4 | 3164413 | 13 | G | A | A | 0.66129 | G | 0.33871 | 19 | 0.6129 |
| 3120 | S4_3164525 | 4 | 3164525 | 112 | A | G | G | 0.66129 | A | 0.33871 | 19 | 0.6129 |
| 3121 | S4_3317993 | 4 | 3317993 | 153468 | G | A | G | 0.67742 | A | 0.32258 | 14 | 0.45161 |
| 3122 | S4_3318005 | 4 | 3318005 | 12 | G | A | A | 0.56452 | G | 0.43548 | 11 | 0.35484 |
| 3123 | S4_3318022 | 4 | 3318022 | 17 | C | T | C | 0.93548 | T | 0.06452 | 4 | 0.12903 |
| 3124 | S4_3318023 | 4 | 3318023 | 1 | A | G | G | 0.87097 | A | 0.12903 | 4 | 0.12903 |
| 3125 | S4_3318065 | 4 | 3318065 | 42 | A | G | G | 0.58065 | A | 0.41935 | 12 | 0.3871 |
| 3126 | S4_3318098 | 4 | 3318098 | 33 | T | C | T | 0.67742 | C | 0.32258 | 14 | 0.45161 |
| 3127 | S4_3318106 | 4 | 3318106 | 8 | T | C | C | 0.8871 | T | 0.1129 | 7 | 0.22581 |
| 3128 | S4_3327615 | 4 | 3327615 | 9509 | T | C | T | 0.54839 | C | 0.45161 | 12 | 0.3871 |
| 3129 | S4_3327707 | 4 | 3327707 | 92 | G | A | G | 0.91935 | A | 0.08065 | 3 | 0.09677 |
| 3130 | S4_3327801 | 4 | 3327801 | 94 | A | G | A | 0.58065 | G | 0.41935 | 12 | 0.3871 |

| Site number | SNP name | Chromosome | Physical position | Physical distance from the previous SNP | Reference allele | Alternative allele | Major allele | Major allele frequency | Minor allele | Minor allele frequency | Number heterozygous | Heterozygosity |
|---|---|---|---|---|---|---|---|---|---|---|---|---|
| 3131 | S4_3542635 | 4 | 3542635 | 214834 | C | T | C | 0.87097 | T | 0.12903 | 8 | 0.25806 |
| 3132 | S4_3542780 | 4 | 3542780 | 145 | C | T | C | 0.90323 | T | 0.09677 | 6 | 0.19355 |
| 3133 | S4_3553323 | 4 | 3553323 | 10543 | A | G | G | 0.66129 | A | 0.33871 | 13 | 0.41935 |
| 3134 | S4_3553488 | 4 | 3553488 | 165 | C | A | C | 0.85484 | A | 0.14516 | 9 | 0.29032 |
| 3135 | S4_3731279 | 4 | 3731279 | 177791 | A | G | G | 0.75806 | A | 0.24194 | 11 | 0.35484 |
| 3136 | S4_3731282 | 4 | 3731282 | 3 | G | A | G | 0.90323 | A | 0.09677 | 6 | 0.19355 |
| 3137 | S4_3731332 | 4 | 3731332 | 50 | C | T | C | 0.93548 | T | 0.06452 | 4 | 0.12903 |
| 3138 | S4_3731333 | 4 | 3731333 | 1 | A | G | G | 0.87097 | A | 0.12903 | 6 | 0.19355 |
| 3139 | S4_3731363 | 4 | 3731363 | 30 | C | T | C | 0.5 | T | 0.5 | 19 | 0.6129 |
| 3140 | S4_3731387 | 4 | 3731387 | 24 | G | A | G | 0.93548 | A | 0.06452 | 4 | 0.12903 |
| 3141 | S4_3731399 | 4 | 3731399 | 12 | C | T | T | 0.72581 | C | 0.27419 | 11 | 0.35484 |
| 3142 | S4_3731406 | 4 | 3731406 | 7 | T | G | G | 0.75806 | T | 0.24194 | 11 | 0.35484 |
| 3143 | S4_3731409 | 4 | 3731409 | 3 | T | C | C | 0.75806 | T | 0.24194 | 11 | 0.35484 |
| 3144 | S4_3889929 | 4 | 3889929 | 158520 | C | T | T | 0.59677 | C | 0.40323 | 15 | 0.48387 |
| 3145 | S4_3889932 | 4 | 3889932 | 3 | C | T | T | 0.59677 | C | 0.40323 | 15 | 0.48387 |
| 3146 | S4_3932485 | 4 | 3932485 | 42553 | C | G | C | 0.95161 | G | 0.04839 | 3 | 0.09677 |
| 3147 | S4_3932554 | 4 | 3932554 | 69 | T | C | T | 0.74194 | C | 0.25806 | 14 | 0.45161 |
| 3148 | S4_3962015 | 4 | 3962015 | 29461 | T | C | C | 0.79032 | T | 0.20968 | 9 | 0.29032 |
| 3149 | S4_3962127 | 4 | 3962127 | 112 | C | T | C | 0.93548 | T | 0.06452 | 2 | 0.06452 |
| 3150 | S4_4065131 | 4 | 4065131 | 103004 | G | A | G | 0.90323 | A | 0.09677 | 6 | 0.19355 |
| 3151 | S4_4323393 | 4 | 4323393 | 258262 | C | T | T | 0.62903 | C | 0.37097 | 19 | 0.6129 |
| 3152 | S4_4677728 | 4 | 4677728 | 354335 | A | G | A | 0.87097 | G | 0.12903 | 8 | 0.25806 |
| 3153 | S4_4678249 | 4 | 4678249 | 521 | C | T | C | 0.95161 | T | 0.04839 | 1 | 0.03226 |
| 3154 | S4_4678305 | 4 | 4678305 | 56 | A | C | A | 0.66129 | C | 0.33871 | 17 | 0.54839 |
| 3155 | S4_4734187 | 4 | 4734187 | 55882 | C | T | C | 0.62903 | T | 0.37097 | 17 | 0.54839 |
| 3156 | S4_4734196 | 4 | 4734196 | 9 | A | G | A | 0.82258 | G | 0.17742 | 7 | 0.22581 |
| 3157 | S4_4734239 | 4 | 4734239 | 43 | G | A | G | 0.91935 | A | 0.08065 | 3 | 0.09677 |
| 3158 | S4_4766333 | 4 | 4766333 | 32094 | T | C | C | 0.67742 | T | 0.32258 | 12 | 0.3871 |
| 3159 | S4_4766354 | 4 | 4766354 | 21 | G | A | A | 0.66129 | G | 0.33871 | 13 | 0.41935 |
| 3160 | S4_4766405 | 4 | 4766405 | 51 | C | G | G | 0.67742 | C | 0.32258 | 12 | 0.3871 |
| 3161 | S4_4868387 | 4 | 4868387 | 101982 | G | C | G | 0.82258 | C | 0.17742 | 9 | 0.29032 |
| 3162 | S4_5457966 | 4 | 5457966 | 589579 | T | C | T | 0.77419 | C | 0.22581 | 10 | 0.32258 |
| 3163 | S4_5462442 | 4 | 5462442 | 4476 | G | A | G | 0.90323 | A | 0.09677 | 6 | 0.19355 |
| 3164 | S4_5462486 | 4 | 5462486 | 44 | G | A | G | 0.77419 | A | 0.22581 | 12 | 0.3871 |
| 3165 | S4_5475647 | 4 | 5475647 | 13161 | T | C | T | 0.91935 | C | 0.08065 | 5 | 0.16129 |
| 3166 | S4_5475698 | 4 | 5475698 | 51 | G | A | G | 0.90323 | A | 0.09677 | 6 | 0.19355 |
| 3167 | S4_5924070 | 4 | 5924070 | 448372 | G | T | G | 0.80645 | T | 0.19355 | 10 | 0.32258 |
| 3168 | S4_5949748 | 4 | 5949748 | 25678 | T | C | T | 0.6129 | C | 0.3871 | 14 | 0.45161 |
| 3169 | S4_6359543 | 4 | 6359543 | 409795 | G | A | A | 0.8871 | G | 0.1129 | 5 | 0.16129 |
| 3170 | S4_6359554 | 4 | 6359554 | 11 | C | G | C | 0.95161 | G | 0.04839 | 3 | 0.09677 |
| 3171 | S4_6359563 | 4 | 6359563 | 9 | G | T | G | 0.95161 | T | 0.04839 | 3 | 0.09677 |
| 3172 | S4_6359637 | 4 | 6359637 | 74 | G | A | G | 0.95161 | A | 0.04839 | 3 | 0.09677 |
| 3173 | S4_6416274 | 4 | 6416274 | 56637 | A | G | G | 0.58065 | A | 0.41935 | 18 | 0.58065 |
| 3174 | S4_6416378 | 4 | 6416378 | 104 | T | C | C | 0.58065 | T | 0.41935 | 18 | 0.58065 |
| 3175 | S4_6416382 | 4 | 6416382 | 4 | T | C | C | 0.58065 | T | 0.41935 | 18 | 0.58065 |
| 3176 | S4_6416401 | 4 | 6416401 | 19 | C | T | C | 0.67742 | T | 0.32258 | 12 | 0.3871 |
| 3177 | S4_6416503 | 4 | 6416503 | 102 | G | A | A | 0.58065 | G | 0.41935 | 18 | 0.58065 |
| 3178 | S4_6418478 | 4 | 6418478 | 1975 | T | C | C | 0.58065 | T | 0.41935 | 18 | 0.58065 |
| 3179 | S4_6418507 | 4 | 6418507 | 29 | G | A | A | 0.59677 | G | 0.40323 | 17 | 0.54839 |
| 3180 | S4_6418545 | 4 | 6418545 | 38 | A | G | G | 0.90323 | A | 0.09677 | 6 | 0.19355 |
| 3181 | S4_6418606 | 4 | 6418606 | 61 | G | A | G | 0.69355 | A | 0.30645 | 17 | 0.54839 |
| 3182 | S4_6483409 | 4 | 6483409 | 64803 | G | A | A | 0.85484 | G | 0.14516 | 7 | 0.22581 |
| 3183 | S4_6483457 | 4 | 6483457 | 48 | G | A | G | 0.66129 | A | 0.33871 | 11 | 0.35484 |
| 3184 | S4_6483539 | 4 | 6483539 | 82 | C | G | C | 0.66129 | G | 0.33871 | 11 | 0.35484 |

| Site number | SNP name | Chromosome | Physical position | Physical distance from the previous SNP | Reference allele | Alternative allele | Major allele | Major allele frequency | Minor allele | Minor allele frequency | Number heterozygous | Heterozygosity |
|---|---|---|---|---|---|---|---|---|---|---|---|---|
| 3185 | S4_6555208 | 4 | 6555208 | 71669 | G | A | G | 0.93548 | A | 0.06452 | 4 | 0.12903 |
| 3186 | S4_6555304 | 4 | 6555304 | 96 | G | A | G | 0.8871 | A | 0.1129 | 7 | 0.22581 |
| 3187 | S4_6555345 | 4 | 6555345 | 41 | C | T | T | 0.82258 | C | 0.17742 | 11 | 0.35484 |
| 3188 | S4_6604655 | 4 | 6604655 | 49310 | C | T | C | 0.77419 | T | 0.22581 | 14 | 0.45161 |
| 3189 | S4_6604656 | 4 | 6604656 | 1 | A | G | A | 0.75806 | G | 0.24194 | 15 | 0.48387 |
| 3190 | S4_6604684 | 4 | 6604684 | 28 | C | T | C | 0.72581 | T | 0.27419 | 17 | 0.54839 |
| 3191 | S4_6604769 | 4 | 6604769 | 85 | A | G | A | 0.85484 | G | 0.14516 | 9 | 0.29032 |
| 3192 | S4_7012587 | 4 | 7012587 | 407818 | A | G | A | 0.80645 | G | 0.19355 | 12 | 0.3871 |
| 3193 | S4_7012744 | 4 | 7012744 | 157 | T | C | T | 0.85484 | C | 0.14516 | 9 | 0.29032 |
| 3194 | S4_7012767 | 4 | 7012767 | 23 | T | C | C | 0.82258 | T | 0.17742 | 11 | 0.35484 |
| 3195 | S4_7285090 | 4 | 7285090 | 272323 | A | C | C | 0.72581 | A | 0.27419 | 11 | 0.35484 |
| 3196 | S4_7285096 | 4 | 7285096 | 6 | C | G | G | 0.72581 | C | 0.27419 | 11 | 0.35484 |
| 3197 | S4_7285228 | 4 | 7285228 | 132 | C | T | C | 0.87097 | T | 0.12903 | 8 | 0.25806 |
| 3198 | S4_7295670 | 4 | 7295670 | 10442 | G | T | G | 0.67742 | T | 0.32258 | 16 | 0.51613 |
| 3199 | S4_7295757 | 4 | 7295757 | 87 | G | C | G | 0.67742 | C | 0.32258 | 16 | 0.51613 |
| 3200 | S4_7665928 | 4 | 7665928 | 370171 | A | G | A | 0.91935 | G | 0.08065 | 3 | 0.09677 |
| 3201 | S4_7665963 | 4 | 7665963 | 35 | A | G | A | 0.93548 | G | 0.06452 | 4 | 0.12903 |
| 3202 | S4_7665980 | 4 | 7665980 | 17 | G | A | G | 0.75806 | A | 0.24194 | 15 | 0.48387 |
| 3203 | S4_7666040 | 4 | 7666040 | 60 | G | A | G | 0.80645 | A | 0.19355 | 10 | 0.32258 |
| 3204 | S4_7666117 | 4 | 7666117 | 77 | C | G | C | 0.82258 | G | 0.17742 | 9 | 0.29032 |
| 3205 | S4_8261072 | 4 | 8261072 | 594955 | C | T | C | 0.66129 | T | 0.33871 | 13 | 0.41935 |
| 3206 | S4_8261252 | 4 | 8261252 | 180 | G | A | G | 0.90323 | A | 0.09677 | 6 | 0.19355 |
| 3207 | S4_8272642 | 4 | 8272642 | 11390 | A | G | A | 0.79032 | G | 0.20968 | 9 | 0.29032 |
| 3208 | S4_8272668 | 4 | 8272668 | 26 | C | T | C | 0.80645 | T | 0.19355 | 8 | 0.25806 |
| 3209 | S4_8272690 | 4 | 8272690 | 22 | C | T | C | 0.8871 | T | 0.1129 | 5 | 0.16129 |
| 3210 | S4_8272692 | 4 | 8272692 | 2 | C | A | C | 0.67742 | A | 0.32258 | 12 | 0.3871 |
| 3211 | S4_8344747 | 4 | 8344747 | 72055 | A | C | A | 0.83871 | C | 0.16129 | 8 | 0.25806 |
| 3212 | S4_8344818 | 4 | 8344818 | 71 | C | T | T | 0.72581 | C | 0.27419 | 13 | 0.41935 |
| 3213 | S4_8344820 | 4 | 8344820 | 2 | G | T | T | 0.72581 | G | 0.27419 | 13 | 0.41935 |
| 3214 | S4_8437965 | 4 | 8437965 | 93145 | C | T | C | 0.87097 | T | 0.12903 | 8 | 0.25806 |
| 3215 | S4_8665025 | 4 | 8665025 | 227060 | C | T | C | 0.91935 | T | 0.08065 | 5 | 0.16129 |
| 3216 | S4_9036144 | 4 | 9036144 | 371119 | A | G | G | 0.74194 | A | 0.25806 | 14 | 0.45161 |
| 3217 | S4_9057019 | 4 | 9057019 | 20875 | T | C | T | 0.74194 | C | 0.25806 | 14 | 0.45161 |
| 3218 | S4_9057191 | 4 | 9057191 | 172 | C | A | C | 0.90323 | A | 0.09677 | 4 | 0.12903 |
| 3219 | S4_9057243 | 4 | 9057243 | 52 | C | T | T | 0.53226 | C | 0.46774 | 19 | 0.6129 |
| 3220 | S4_9061568 | 4 | 9061568 | 4325 | T | C | C | 0.5 | T | 0.5 | 13 | 0.41935 |
| 3221 | S4_9061643 | 4 | 9061643 | 75 | G | T | G | 0.87097 | T | 0.12903 | 6 | 0.19355 |
| 3222 | S4_9076394 | 4 | 9076394 | 14751 | C | T | C | 0.93548 | T | 0.06452 | 2 | 0.06452 |
| 3223 | S4_9123754 | 4 | 9123754 | 47360 | G | A | G | 0.95161 | A | 0.04839 | 3 | 0.09677 |
| 3224 | S4_9123959 | 4 | 9123959 | 205 | T | C | T | 0.95161 | C | 0.04839 | 3 | 0.09677 |
| 3225 | S4_9209296 | 4 | 9209296 | 85337 | T | G | T | 0.69355 | G | 0.30645 | 11 | 0.35484 |
| 3226 | S4_9465838 | 4 | 9465838 | 256542 | A | G | G | 0.70968 | A | 0.29032 | 8 | 0.25806 |
| 3227 | S4_9465905 | 4 | 9465905 | 67 | T | C | T | 0.90323 | C | 0.09677 | 4 | 0.12903 |
| 3228 | S4_9466041 | 4 | 9466041 | 136 | C | A | A | 0.70968 | C | 0.29032 | 8 | 0.25806 |
| 3229 | S4_9466045 | 4 | 9466045 | 4 | C | A | C | 0.90323 | A | 0.09677 | 4 | 0.12903 |
| 3230 | S4_9466060 | 4 | 9466060 | 15 | C | T | C | 0.90323 | T | 0.09677 | 4 | 0.12903 |
| 3231 | S4_9998131 | 4 | 9998131 | 532071 | T | C | C | 0.85484 | T | 0.14516 | 9 | 0.29032 |
| 3232 | S4_9998149 | 4 | 9998149 | 18 | A | C | C | 0.85484 | A | 0.14516 | 9 | 0.29032 |
| 3233 | S4_9998168 | 4 | 9998168 | 19 | T | C | C | 0.85484 | T | 0.14516 | 9 | 0.29032 |
| 3234 | S4_10090677 | 4 | 10090677 | 92509 | C | T | T | 0.58065 | C | 0.41935 | 10 | 0.32258 |
| 3235 | S4_10090725 | 4 | 10090725 | 48 | G | A | G | 0.91935 | A | 0.08065 | 5 | 0.16129 |
| 3236 | S4_10090730 | 4 | 10090730 | 5 | A | G | A | 0.93548 | G | 0.06452 | 4 | 0.12903 |
| 3237 | S4_10090766 | 4 | 10090766 | 36 | T | C | T | 0.93548 | C | 0.06452 | 4 | 0.12903 |
| 3238 | S4_10431684 | 4 | 10431684 | 340918 | C | T | C | 0.80645 | T | 0.19355 | 8 | 0.25806 |

| Site number | SNP name | Chromosome | Physical position | Physical distance from the previous SNP | Reference allele | Alternative allele | Major allele | Major allele frequency | Minor allele | Minor allele frequency | Number heterozygous | Heterozygosity |
|---|---|---|---|---|---|---|---|---|---|---|---|---|
| 3239 | S4_10431719 | 4 | 10431719 | 35 | C | A | C | 0.93548 | A | 0.06452 | 2 | 0.06452 |
| 3240 | S4_10431774 | 4 | 10431774 | 55 | T | C | T | 0.79032 | C | 0.20968 | 9 | 0.29032 |
| 3241 | S4_10946063 | 4 | 10946063 | 514289 | C | T | T | 0.91935 | C | 0.08065 | 5 | 0.16129 |
| 3242 | S4_10946089 | 4 | 10946089 | 26 | A | C | A | 0.95161 | C | 0.04839 | 3 | 0.09677 |
| 3243 | S4_10946161 | 4 | 10946161 | 72 | G | A | G | 0.95161 | A | 0.04839 | 3 | 0.09677 |
| 3244 | S4_10946167 | 4 | 10946167 | 6 | A | G | G | 0.91935 | A | 0.08065 | 5 | 0.16129 |
| 3245 | S4_10946173 | 4 | 10946173 | 6 | G | A | G | 0.95161 | A | 0.04839 | 3 | 0.09677 |
| 3246 | S4_10946232 | 4 | 10946232 | 59 | G | T | T | 0.95161 | G | 0.04839 | 3 | 0.09677 |
| 3247 | S4_11460901 | 4 | 11460901 | 514669 | C | T | T | 0.85484 | C | 0.14516 | 9 | 0.29032 |
| 3248 | S4_12067017 | 4 | 12067017 | 606116 | A | G | G | 0.6129 | A | 0.3871 | 12 | 0.3871 |
| 3249 | S4_12067063 | 4 | 12067063 | 46 | C | A | C | 0.90323 | A | 0.09677 | 6 | 0.19355 |
| 3250 | S4_12067216 | 4 | 12067216 | 153 | A | C | C | 0.6129 | A | 0.3871 | 12 | 0.3871 |
| 3251 | S4_12405829 | 4 | 12405829 | 338613 | G | A | G | 0.51613 | A | 0.48387 | 14 | 0.45161 |
| 3252 | S4_12405971 | 4 | 12405971 | 142 | T | C | T | 0.53226 | C | 0.46774 | 13 | 0.41935 |
| 3253 | S4_12489314 | 4 | 12489314 | 83343 | A | G | A | 0.90323 | G | 0.09677 | 4 | 0.12903 |
| 3254 | S4_12489349 | 4 | 12489349 | 35 | G | A | G | 0.8871 | A | 0.1129 | 7 | 0.22581 |
| 3255 | S4_12840495 | 4 | 12840495 | 351146 | G | T | G | 0.91935 | T | 0.08065 | 5 | 0.16129 |
| 3256 | S4_12840544 | 4 | 12840544 | 49 | C | A | A | 0.54839 | C | 0.45161 | 16 | 0.51613 |
| 3257 | S4_12840579 | 4 | 12840579 | 35 | T | C | T | 0.66129 | C | 0.33871 | 11 | 0.35484 |
| 3258 | S4_12840602 | 4 | 12840602 | 23 | A | G | A | 0.75806 | G | 0.24194 | 13 | 0.41935 |
| 3259 | S4_13178182 | 4 | 13178182 | 337580 | T | G | T | 0.93548 | G | 0.06452 | 4 | 0.12903 |
| 3260 | S4_13202582 | 4 | 13202582 | 24400 | C | G | C | 0.8871 | G | 0.1129 | 7 | 0.22581 |
| 3261 | S4_13202583 | 4 | 13202583 | 1 | C | G | G | 0.62903 | C | 0.37097 | 15 | 0.48387 |
| 3262 | S4_13202596 | 4 | 13202596 | 13 | T | C | T | 0.6129 | C | 0.3871 | 14 | 0.45161 |
| 3263 | S4_13202795 | 4 | 13202795 | 199 | T | C | C | 0.62903 | T | 0.37097 | 15 | 0.48387 |
| 3264 | S4_13483516 | 4 | 13483516 | 280721 | A | G | A | 0.91935 | G | 0.08065 | 5 | 0.16129 |
| 3265 | S4_13483585 | 4 | 13483585 | 69 | C | T | C | 0.90323 | T | 0.09677 | 6 | 0.19355 |
| 3266 | S4_13483704 | 4 | 13483704 | 119 | A | G | A | 0.90323 | G | 0.09677 | 6 | 0.19355 |
| 3267 | S4_13483714 | 4 | 13483714 | 10 | G | A | G | 0.90323 | A | 0.09677 | 6 | 0.19355 |
| 3268 | S4_13508829 | 4 | 13508829 | 25115 | C | G | C | 0.62903 | G | 0.37097 | 13 | 0.41935 |
| 3269 | S4_13508866 | 4 | 13508866 | 37 | C | T | C | 0.85484 | T | 0.14516 | 9 | 0.29032 |
| 3270 | S4_13540891 | 4 | 13540891 | 32025 | G | T | G | 0.66129 | T | 0.33871 | 17 | 0.54839 |
| 3271 | S4_13541067 | 4 | 13541067 | 176 | C | T | C | 0.79032 | T | 0.20968 | 11 | 0.35484 |
| 3272 | S4_13593604 | 4 | 13593604 | 52537 | A | G | A | 0.6129 | G | 0.3871 | 14 | 0.45161 |
| 3273 | S4_13593673 | 4 | 13593673 | 69 | A | T | T | 0.79032 | A | 0.20968 | 11 | 0.35484 |
| 3274 | S4_13866232 | 4 | 13866232 | 272559 | C | T | C | 0.77419 | T | 0.22581 | 10 | 0.32258 |
| 3275 | S4_13866419 | 4 | 13866419 | 187 | G | A | A | 0.69355 | G | 0.30645 | 13 | 0.41935 |
| 3276 | S4_14335047 | 4 | 14335047 | 468628 | C | T | C | 0.83871 | T | 0.16129 | 10 | 0.32258 |
| 3277 | S4_14335130 | 4 | 14335130 | 83 | C | T | C | 0.62903 | T | 0.37097 | 17 | 0.54839 |
| 3278 | S4_14335146 | 4 | 14335146 | 16 | G | A | G | 0.87097 | A | 0.12903 | 8 | 0.25806 |
| 3279 | S4_14335216 | 4 | 14335216 | 70 | T | A | T | 0.82258 | A | 0.17742 | 11 | 0.35484 |
| 3280 | S4_14411378 | 4 | 14411378 | 76162 | T | C | T | 0.80645 | C | 0.19355 | 8 | 0.25806 |
| 3281 | S4_14411411 | 4 | 14411411 | 33 | C | T | C | 0.80645 | T | 0.19355 | 8 | 0.25806 |
| 3282 | S4_14413531 | 4 | 14413531 | 2120 | C | A | A | 0.91935 | C | 0.08065 | 5 | 0.16129 |
| 3283 | S4_14413538 | 4 | 14413538 | 7 | G | T | T | 0.91935 | G | 0.08065 | 5 | 0.16129 |
| 3284 | S4_14629729 | 4 | 14629729 | 216191 | T | C | C | 0.8871 | T | 0.1129 | 7 | 0.22581 |
| 3285 | S4_14629821 | 4 | 14629821 | 92 | A | G | A | 0.8871 | G | 0.1129 | 7 | 0.22581 |
| 3286 | S4_14629858 | 4 | 14629858 | 37 | C | T | C | 0.90323 | T | 0.09677 | 6 | 0.19355 |
| 3287 | S4_14629917 | 4 | 14629917 | 59 | A | G | G | 0.74194 | A | 0.25806 | 12 | 0.3871 |
| 3288 | S4_14629966 | 4 | 14629966 | 49 | A | G | A | 0.95161 | G | 0.04839 | 3 | 0.09677 |
| 3289 | S4_14837959 | 4 | 14837959 | 207993 | A | G | G | 0.83871 | A | 0.16129 | 6 | 0.19355 |
| 3290 | S4_14838017 | 4 | 14838017 | 58 | G | A | G | 0.58065 | A | 0.41935 | 10 | 0.32258 |
| 3291 | S4_14838059 | 4 | 14838059 | 42 | A | G | A | 0.58065 | G | 0.41935 | 10 | 0.32258 |
| 3292 | S4_14838060 | 4 | 14838060 | 1 | G | C | G | 0.58065 | C | 0.41935 | 10 | 0.32258 |

| Site number | SNP name | Chromosome | Physical position | Physical distance from the previous SNP | Reference allele | Alternative allele | Major allele | Major allele frequency | Minor allele | Minor allele frequency | Number heterozygous | Heterozygosity |
|---|---|---|---|---|---|---|---|---|---|---|---|---|
| 3293 | S4_15112716 | 4 | 15112716 | 274656 | G | A | A | 0.56452 | G | 0.43548 | 13 | 0.41935 |
| 3294 | S4_15112723 | 4 | 15112723 | 7 | T | C | C | 0.59677 | T | 0.40323 | 13 | 0.41935 |
| 3295 | S4_15206069 | 4 | 15206069 | 93346 | G | A | G | 0.90323 | A | 0.09677 | 6 | 0.19355 |
| 3296 | S4_15206080 | 4 | 15206080 | 11 | A | G | A | 0.53226 | G | 0.46774 | 15 | 0.48387 |
| 3297 | S4_15206113 | 4 | 15206113 | 33 | A | G | A | 0.53226 | G | 0.46774 | 15 | 0.48387 |
| 3298 | S4_15206234 | 4 | 15206234 | 121 | T | A | T | 0.93548 | A | 0.06452 | 2 | 0.06452 |
| 3299 | S4_15206248 | 4 | 15206248 | 14 | C | T | C | 0.62903 | T | 0.37097 | 15 | 0.48387 |
| 3300 | S4_15590327 | 4 | 15590327 | 384079 | T | C | C | 0.83871 | T | 0.16129 | 8 | 0.25806 |
| 3301 | S4_15590368 | 4 | 15590368 | 41 | G | T | G | 0.79032 | T | 0.20968 | 11 | 0.35484 |
| 3302 | S4_15590404 | 4 | 15590404 | 36 | G | C | G | 0.87097 | C | 0.12903 | 8 | 0.25806 |
| 3303 | S4_15590446 | 4 | 15590446 | 42 | C | G | C | 0.93548 | G | 0.06452 | 4 | 0.12903 |
| 3304 | S4_15590514 | 4 | 15590514 | 68 | G | A | G | 0.69355 | A | 0.30645 | 13 | 0.41935 |
| 3305 | S4_15590520 | 4 | 15590520 | 6 | T | C | C | 0.75806 | T | 0.24194 | 13 | 0.41935 |
| 3306 | S4_15885592 | 4 | 15885592 | 295072 | C | G | G | 0.93548 | C | 0.06452 | 4 | 0.12903 |
| 3307 | S4_15885626 | 4 | 15885626 | 34 | G | T | T | 0.93548 | G | 0.06452 | 4 | 0.12903 |
| 3308 | S4_16429767 | 4 | 16429767 | 544141 | G | A | G | 0.8871 | A | 0.1129 | 7 | 0.22581 |
| 3309 | S4_16664249 | 4 | 16664249 | 234482 | C | T | C | 0.83871 | T | 0.16129 | 6 | 0.19355 |
| 3310 | S4_16811353 | 4 | 16811353 | 147104 | C | T | C | 0.80645 | T | 0.19355 | 10 | 0.32258 |
| 3311 | S4_16811358 | 4 | 16811358 | 5 | T | C | T | 0.83871 | C | 0.16129 | 6 | 0.19355 |
| 3312 | S4_16843470 | 4 | 16843470 | 32112 | G | C | G | 0.77419 | C | 0.22581 | 8 | 0.25806 |
| 3313 | S4_16913157 | 4 | 16913157 | 69687 | A | G | G | 0.72581 | A | 0.27419 | 9 | 0.29032 |
| 3314 | S4_17211832 | 4 | 17211832 | 298675 | A | G | G | 0.70968 | A | 0.29032 | 16 | 0.51613 |
| 3315 | S4_17272348 | 4 | 17272348 | 60516 | G | A | G | 0.87097 | A | 0.12903 | 8 | 0.25806 |
| 3316 | S4_17400350 | 4 | 17400350 | 128002 | G | A | G | 0.91935 | A | 0.08065 | 5 | 0.16129 |
| 3317 | S4_17400381 | 4 | 17400381 | 31 | C | G | C | 0.85484 | G | 0.14516 | 9 | 0.29032 |
| 3318 | S4_17531840 | 4 | 17531840 | 131459 | T | G | T | 0.80645 | G | 0.19355 | 8 | 0.25806 |
| 3319 | S4_17531889 | 4 | 17531889 | 49 | T | C | T | 0.90323 | C | 0.09677 | 4 | 0.12903 |
| 3320 | S4_17686414 | 4 | 17686414 | 154525 | G | C | C | 0.83871 | G | 0.16129 | 10 | 0.32258 |
| 3321 | S4_17779718 | 4 | 17779718 | 93304 | A | G | G | 0.51613 | A | 0.48387 | 16 | 0.51613 |
| 3322 | S4_17779719 | 4 | 17779719 | 1 | C | T | C | 0.75806 | T | 0.24194 | 13 | 0.41935 |
| 3323 | S4_17779796 | 4 | 17779796 | 77 | T | C | C | 0.69355 | T | 0.30645 | 15 | 0.48387 |
| 3324 | S4_18280102 | 4 | 18280102 | 500306 | T | C | T | 0.87097 | C | 0.12903 | 8 | 0.25806 |
| 3325 | S4_18280162 | 4 | 18280162 | 60 | G | A | G | 0.87097 | A | 0.12903 | 8 | 0.25806 |
| 3326 | S4_18280170 | 4 | 18280170 | 8 | C | T | T | 0.90323 | C | 0.09677 | 6 | 0.19355 |
| 3327 | S4_18336440 | 4 | 18336440 | 56270 | A | C | A | 0.77419 | C | 0.22581 | 12 | 0.3871 |
| 3328 | S4_18336475 | 4 | 18336475 | 35 | A | G | A | 0.93548 | G | 0.06452 | 4 | 0.12903 |
| 3329 | S4_18336544 | 4 | 18336544 | 69 | T | C | T | 0.51613 | C | 0.48387 | 16 | 0.51613 |
| 3330 | S4_18336594 | 4 | 18336594 | 50 | G | A | G | 0.83871 | A | 0.16129 | 10 | 0.32258 |
| 3331 | S4_18362385 | 4 | 18362385 | 25791 | G | T | T | 0.82258 | G | 0.17742 | 11 | 0.35484 |
| 3332 | S4_18362512 | 4 | 18362512 | 127 | A | G | A | 0.67742 | G | 0.32258 | 14 | 0.45161 |
| 3333 | S4_18414842 | 4 | 18414842 | 52330 | C | T | C | 0.85484 | T | 0.14516 | 5 | 0.16129 |
| 3334 | S4_18415005 | 4 | 18415005 | 163 | C | T | T | 0.66129 | C | 0.33871 | 11 | 0.35484 |
| 3335 | S4_18415048 | 4 | 18415048 | 43 | A | T | A | 0.5 | T | 0.5 | 13 | 0.41935 |
| 3336 | S4_18892556 | 4 | 18892556 | 477508 | C | T | C | 0.74194 | T | 0.25806 | 16 | 0.51613 |
| 3337 | S4_18892622 | 4 | 18892622 | 66 | C | T | T | 0.91935 | C | 0.08065 | 5 | 0.16129 |
| 3338 | S4_19002480 | 4 | 19002480 | 109858 | G | A | G | 0.91935 | A | 0.08065 | 3 | 0.09677 |
| 3339 | S4_19261949 | 4 | 19261949 | 259469 | C | T | C | 0.75806 | T | 0.24194 | 13 | 0.41935 |
| 3340 | S4_19262030 | 4 | 19262030 | 81 | A | T | A | 0.93548 | T | 0.06452 | 4 | 0.12903 |
| 3341 | S4_20068134 | 4 | 20068134 | 806104 | A | G | A | 0.58065 | G | 0.41935 | 16 | 0.51613 |
| 3342 | S4_20291760 | 4 | 20291760 | 223626 | C | G | C | 0.93548 | G | 0.06452 | 4 | 0.12903 |
| 3343 | S4_20430555 | 4 | 20430555 | 138795 | C | T | C | 0.93548 | T | 0.06452 | 4 | 0.12903 |
| 3344 | S4_20430616 | 4 | 20430616 | 61 | G | A | G | 0.53226 | A | 0.46774 | 17 | 0.54839 |
| 3345 | S4_20430688 | 4 | 20430688 | 72 | A | G | A | 0.70968 | G | 0.29032 | 12 | 0.3871 |
| 3346 | S4_20430715 | 4 | 20430715 | 27 | T | C | T | 0.53226 | C | 0.46774 | 17 | 0.54839 |

| Site number | SNP name | Chromosome | Physical position | Physical distance from the previous SNP | Reference allele | Alternative allele | Major allele | Major allele frequency | Minor allele | Minor allele frequency | Number heterozygous | Heterozygosity |
|---|---|---|---|---|---|---|---|---|---|---|---|---|
| 3347 | S4_20430734 | 4 | 20430734 | 19 | C | T | C | 0.53226 | T | 0.46774 | 17 | 0.54839 |
| 3348 | S4_20430760 | 4 | 20430760 | 26 | G | A | G | 0.93548 | A | 0.06452 | 4 | 0.12903 |
| 3349 | S4_20536838 | 4 | 20536838 | 106078 | A | G | A | 0.75806 | G | 0.24194 | 9 | 0.29032 |
| 3350 | S4_20686677 | 4 | 20686677 | 149839 | A | G | G | 0.75806 | A | 0.24194 | 11 | 0.35484 |
| 3351 | S4_20686685 | 4 | 20686685 | 8 | G | A | G | 0.69355 | A | 0.30645 | 15 | 0.48387 |
| 3352 | S4_20686892 | 4 | 20686892 | 207 | C | T | T | 0.75806 | C | 0.24194 | 11 | 0.35484 |
| 3353 | S4_20686909 | 4 | 20686909 | 17 | G | A | A | 0.75806 | G | 0.24194 | 11 | 0.35484 |
| 3354 | S4_20988390 | 4 | 20988390 | 301481 | C | T | T | 0.69355 | C | 0.30645 | 17 | 0.54839 |
| 3355 | S4_20988484 | 4 | 20988484 | 94 | C | T | C | 0.91935 | T | 0.08065 | 5 | 0.16129 |
| 3356 | S4_20988544 | 4 | 20988544 | 60 | A | G | G | 0.69355 | A | 0.30645 | 17 | 0.54839 |
| 3357 | S4_21206512 | 4 | 21206512 | 217968 | T | C | T | 0.93548 | C | 0.06452 | 2 | 0.06452 |
| 3358 | S4_21206686 | 4 | 21206686 | 174 | G | C | G | 0.51613 | C | 0.48387 | 16 | 0.51613 |
| 3359 | S4_21528303 | 4 | 21528303 | 321617 | G | A | G | 0.74194 | A | 0.25806 | 8 | 0.25806 |
| 3360 | S4_21528379 | 4 | 21528379 | 76 | T | C | T | 0.91935 | C | 0.08065 | 3 | 0.09677 |
| 3361 | S4_21528387 | 4 | 21528387 | 8 | T | C | T | 0.75806 | C | 0.24194 | 9 | 0.29032 |
| 3362 | S4_21528437 | 4 | 21528437 | 50 | T | C | T | 0.75806 | C | 0.24194 | 9 | 0.29032 |
| 3363 | S4_21528453 | 4 | 21528453 | 16 | A | C | A | 0.75806 | C | 0.24194 | 9 | 0.29032 |
| 3364 | S4_21528503 | 4 | 21528503 | 50 | T | C | T | 0.75806 | C | 0.24194 | 9 | 0.29032 |
| 3365 | S4_21528524 | 4 | 21528524 | 21 | G | T | G | 0.75806 | T | 0.24194 | 9 | 0.29032 |
| 3366 | S4_21768328 | 4 | 21768328 | 239804 | G | A | A | 0.58065 | G | 0.41935 | 14 | 0.45161 |
| 3367 | S4_22046949 | 4 | 22046949 | 278621 | C | T | T | 0.69355 | C | 0.30645 | 13 | 0.41935 |
| 3368 | S4_22088902 | 4 | 22088902 | 41953 | A | T | A | 0.91935 | T | 0.08065 | 5 | 0.16129 |
| 3369 | S4_22088954 | 4 | 22088954 | 52 | G | A | G | 0.79032 | A | 0.20968 | 13 | 0.41935 |
| 3370 | S4_22098129 | 4 | 22098129 | 9175 | G | A | A | 0.85484 | G | 0.14516 | 7 | 0.22581 |
| 3371 | S4_22098202 | 4 | 22098202 | 73 | C | G | G | 0.75806 | C | 0.24194 | 9 | 0.29032 |
| 3372 | S4_22142183 | 4 | 22142183 | 43981 | G | T | T | 0.85484 | G | 0.14516 | 5 | 0.16129 |
| 3373 | S4_22142236 | 4 | 22142236 | 53 | T | C | T | 0.75806 | C | 0.24194 | 9 | 0.29032 |
| 3374 | S4_22142253 | 4 | 22142253 | 17 | A | T | T | 0.64516 | A | 0.35484 | 14 | 0.45161 |
| 3375 | S4_22142254 | 4 | 22142254 | 1 | C | T | T | 0.64516 | C | 0.35484 | 14 | 0.45161 |
| 3376 | S4_22287770 | 4 | 22287770 | 145516 | C | T | C | 0.93548 | T | 0.06452 | 4 | 0.12903 |
| 3377 | S4_22287886 | 4 | 22287886 | 116 | T | C | T | 0.66129 | C | 0.33871 | 13 | 0.41935 |
| 3378 | S4_22466854 | 4 | 22466854 | 178968 | C | G | G | 0.59677 | C | 0.40323 | 13 | 0.41935 |
| 3379 | S4_22466935 | 4 | 22466935 | 81 | C | T | T | 0.6129 | C | 0.3871 | 12 | 0.3871 |
| 3380 | S4_22467024 | 4 | 22467024 | 89 | T | C | C | 0.6129 | T | 0.3871 | 12 | 0.3871 |
| 3381 | S4_22467077 | 4 | 22467077 | 53 | G | T | T | 0.6129 | G | 0.3871 | 12 | 0.3871 |
| 3382 | S4_22467101 | 4 | 22467101 | 24 | T | C | C | 0.6129 | T | 0.3871 | 12 | 0.3871 |
| 3383 | S4_22467103 | 4 | 22467103 | 2 | A | C | C | 0.6129 | A | 0.3871 | 12 | 0.3871 |
| 3384 | S4_23228219 | 4 | 23228219 | 761116 | G | T | G | 0.95161 | T | 0.04839 | 3 | 0.09677 |
| 3385 | S4_23228222 | 4 | 23228222 | 3 | G | A | G | 0.8871 | A | 0.1129 | 5 | 0.16129 |
| 3386 | S4_23228419 | 4 | 23228419 | 197 | C | T | C | 0.83871 | T | 0.16129 | 10 | 0.32258 |
| 3387 | S4_23303952 | 4 | 23303952 | 75533 | G | A | A | 0.5 | G | 0.5 | 11 | 0.35484 |
| 3388 | S4_23622863 | 4 | 23622863 | 318911 | G | A | G | 0.8871 | A | 0.1129 | 7 | 0.22581 |
| 3389 | S4_23938508 | 4 | 23938508 | 315645 | A | G | G | 0.64516 | A | 0.35484 | 18 | 0.58065 |
| 3390 | S4_23945555 | 4 | 23945555 | 7047 | C | T | C | 0.90323 | T | 0.09677 | 6 | 0.19355 |
| 3391 | S4_23945587 | 4 | 23945587 | 32 | C | T | C | 0.8871 | T | 0.1129 | 7 | 0.22581 |
| 3392 | S4_23945687 | 4 | 23945687 | 100 | A | G | A | 0.74194 | G | 0.25806 | 10 | 0.32258 |
| 3393 | S4_24051495 | 4 | 24051495 | 105808 | C | T | C | 0.93548 | T | 0.06452 | 2 | 0.06452 |
| 3394 | S4_24051518 | 4 | 24051518 | 23 | G | A | G | 0.93548 | A | 0.06452 | 2 | 0.06452 |
| 3395 | S4_24051531 | 4 | 24051531 | 13 | T | C | T | 0.8871 | C | 0.1129 | 7 | 0.22581 |
| 3396 | S4_24051536 | 4 | 24051536 | 5 | G | T | G | 0.93548 | T | 0.06452 | 2 | 0.06452 |
| 3397 | S4_24051538 | 4 | 24051538 | 2 | A | G | A | 0.8871 | G | 0.1129 | 7 | 0.22581 |
| 3398 | S4_24051600 | 4 | 24051600 | 62 | G | T | G | 0.93548 | T | 0.06452 | 2 | 0.06452 |
| 3399 | S4_24070379 | 4 | 24070379 | 18779 | G | T | G | 0.59677 | T | 0.40323 | 15 | 0.48387 |
| 3400 | S4_24070412 | 4 | 24070412 | 33 | A | G | G | 0.69355 | A | 0.30645 | 13 | 0.41935 |

| Site number | SNP name | Chromosome | Physical position | Physical distance from the previous SNP | Reference allele | Alternative allele | Major allele | Major allele frequency | Minor allele | Minor allele frequency | Number heterozygous | Heterozygosity |
|---|---|---|---|---|---|---|---|---|---|---|---|---|
| 3401 | S4_24070509 | 4 | 24070509 | 97 | G | A | G | 0.70968 | A | 0.29032 | 12 | 0.3871 |
| 3402 | S4_24119912 | 4 | 24119912 | 49403 | T | G | T | 0.95161 | G | 0.04839 | 3 | 0.09677 |
| 3403 | S4_24119963 | 4 | 24119963 | 51 | G | A | G | 0.95161 | A | 0.04839 | 3 | 0.09677 |
| 3404 | S4_24141094 | 4 | 24141094 | 21131 | C | T | C | 0.82258 | T | 0.17742 | 9 | 0.29032 |
| 3405 | S4_24141119 | 4 | 24141119 | 25 | T | G | T | 0.51613 | G | 0.48387 | 18 | 0.58065 |
| 3406 | S4_24141186 | 4 | 24141186 | 67 | G | A | G | 0.83871 | A | 0.16129 | 10 | 0.32258 |
| 3407 | S4_24298301 | 4 | 24298301 | 157115 | G | A | G | 0.87097 | A | 0.12903 | 8 | 0.25806 |
| 3408 | S4_24298334 | 4 | 24298334 | 33 | A | C | C | 0.75806 | A | 0.24194 | 13 | 0.41935 |
| 3409 | S4_24361238 | 4 | 24361238 | 62904 | G | A | G | 0.87097 | A | 0.12903 | 8 | 0.25806 |
| 3410 | S4_24361308 | 4 | 24361308 | 70 | C | T | C | 0.95161 | T | 0.04839 | 3 | 0.09677 |
| 3411 | S4_24361353 | 4 | 24361353 | 45 | T | A | T | 0.62903 | A | 0.37097 | 15 | 0.48387 |
| 3412 | S4_24626590 | 4 | 24626590 | 265237 | C | A | C | 0.51613 | A | 0.48387 | 10 | 0.32258 |
| 3413 | S4_24626630 | 4 | 24626630 | 40 | C | G | C | 0.51613 | G | 0.48387 | 10 | 0.32258 |
| 3414 | S4_24631227 | 4 | 24631227 | 4597 | T | C | C | 0.62903 | T | 0.37097 | 15 | 0.48387 |
| 3415 | S4_24631251 | 4 | 24631251 | 24 | G | A | G | 0.91935 | A | 0.08065 | 3 | 0.09677 |
| 3416 | S4_24631404 | 4 | 24631404 | 153 | G | A | G | 0.91935 | A | 0.08065 | 3 | 0.09677 |
| 3417 | S4_24631428 | 4 | 24631428 | 24 | C | A | C | 0.91935 | A | 0.08065 | 3 | 0.09677 |
| 3418 | S4_24666030 | 4 | 24666030 | 34602 | A | G | A | 0.90323 | G | 0.09677 | 4 | 0.12903 |
| 3419 | S4_24846858 | 4 | 24846858 | 180828 | G | A | A | 0.90323 | G | 0.09677 | 6 | 0.19355 |
| 3420 | S4_24846953 | 4 | 24846953 | 95 | G | A | G | 0.95161 | A | 0.04839 | 3 | 0.09677 |
| 3421 | S4_25130442 | 4 | 25130442 | 283489 | G | A | G | 0.90323 | A | 0.09677 | 4 | 0.12903 |
| 3422 | S4_25677142 | 4 | 25677142 | 546700 | G | A | A | 0.62903 | G | 0.37097 | 15 | 0.48387 |
| 3423 | S4_25677282 | 4 | 25677282 | 140 | G | A | G | 0.95161 | A | 0.04839 | 1 | 0.03226 |
| 3424 | S4_25958985 | 4 | 25958985 | 281703 | G | A | G | 0.93548 | A | 0.06452 | 2 | 0.06452 |
| 3425 | S4_27264004 | 4 | 27264004 | 1305019 | G | A | G | 0.74194 | A | 0.25806 | 12 | 0.3871 |
| 3426 | S4_27664288 | 4 | 27664288 | 400284 | C | T | T | 0.66129 | C | 0.33871 | 17 | 0.54839 |
| 3427 | S4_27753805 | 4 | 27753805 | 89517 | G | A | A | 0.90323 | G | 0.09677 | 6 | 0.19355 |
| 3428 | S4_27795417 | 4 | 27795417 | 41612 | G | C | G | 0.79032 | C | 0.20968 | 9 | 0.29032 |
| 3429 | S4_27943876 | 4 | 27943876 | 148459 | G | A | G | 0.79032 | A | 0.20968 | 11 | 0.35484 |
| 3430 | S4_28017137 | 4 | 28017137 | 73261 | C | T | C | 0.75806 | T | 0.24194 | 13 | 0.41935 |
| 3431 | S4_28017234 | 4 | 28017234 | 97 | G | A | G | 0.90323 | A | 0.09677 | 6 | 0.19355 |
| 3432 | S4_28017250 | 4 | 28017250 | 16 | G | A | G | 0.90323 | A | 0.09677 | 6 | 0.19355 |
| 3433 | S4_28017356 | 4 | 28017356 | 106 | T | A | A | 0.67742 | T | 0.32258 | 12 | 0.3871 |
| 3434 | S4_29212650 | 4 | 29212650 | 1195294 | T | A | T | 0.51613 | A | 0.48387 | 16 | 0.51613 |
| 3435 | S4_29212697 | 4 | 29212697 | 47 | G | A | A | 0.8871 | G | 0.1129 | 5 | 0.16129 |
| 3436 | S4_29212795 | 4 | 29212795 | 98 | C | T | C | 0.53226 | T | 0.46774 | 15 | 0.48387 |
| 3437 | S4_29212883 | 4 | 29212883 | 88 | C | T | T | 0.67742 | C | 0.32258 | 14 | 0.45161 |
| 3438 | S4_29212994 | 4 | 29212994 | 111 | T | C | T | 0.58065 | C | 0.41935 | 14 | 0.45161 |
| 3439 | S4_29263068 | 4 | 29263068 | 50074 | T | C | T | 0.75806 | C | 0.24194 | 13 | 0.41935 |
| 3440 | S4_30145500 | 4 | 30145500 | 882432 | C | T | C | 0.74194 | T | 0.25806 | 10 | 0.32258 |
| 3441 | S4_30657336 | 4 | 30657336 | 511836 | A | C | A | 0.90323 | C | 0.09677 | 6 | 0.19355 |
| 3442 | S4_30657551 | 4 | 30657551 | 215 | T | C | C | 0.62903 | T | 0.37097 | 17 | 0.54839 |
| 3443 | S4_31167679 | 4 | 31167679 | 510128 | C | T | C | 0.67742 | T | 0.32258 | 16 | 0.51613 |
| 3444 | S4_31167704 | 4 | 31167704 | 25 | T | A | A | 0.75806 | T | 0.24194 | 11 | 0.35484 |
| 3445 | S4_31755198 | 4 | 31755198 | 587494 | T | C | C | 0.80645 | T | 0.19355 | 10 | 0.32258 |
| 3446 | S4_31755229 | 4 | 31755229 | 31 | C | T | T | 0.80645 | C | 0.19355 | 10 | 0.32258 |
| 3447 | S4_31755250 | 4 | 31755250 | 21 | G | A | G | 0.83871 | A | 0.16129 | 10 | 0.32258 |
| 3448 | S4_31755264 | 4 | 31755264 | 14 | G | A | A | 0.80645 | G | 0.19355 | 10 | 0.32258 |
| 3449 | S4_31755269 | 4 | 31755269 | 5 | A | G | A | 0.64516 | G | 0.35484 | 16 | 0.51613 |
| 3450 | S4_31755288 | 4 | 31755288 | 19 | T | A | T | 0.64516 | A | 0.35484 | 16 | 0.51613 |
| 3451 | S4_31755290 | 4 | 31755290 | 2 | C | G | C | 0.64516 | G | 0.35484 | 16 | 0.51613 |
| 3452 | S4_31755330 | 4 | 31755330 | 40 | T | C | C | 0.80645 | T | 0.19355 | 10 | 0.32258 |
| 3453 | S4_31755346 | 4 | 31755346 | 16 | T | C | C | 0.80645 | T | 0.19355 | 10 | 0.32258 |
| 3454 | S4_31755365 | 4 | 31755365 | 19 | T | G | G | 0.80645 | T | 0.19355 | 10 | 0.32258 |

| Site number | SNP name | Chromosome | Physical position | Physical distance from the previous SNP | Reference allele | Alternative allele | Major allele | Major allele frequency | Minor allele | Minor allele frequency | Number heterozygous | Heterozygosity |
|---|---|---|---|---|---|---|---|---|---|---|---|---|
| 3455 | S4_31755388 | 4 | 31755388 | 23 | A | G | G | 0.80645 | A | 0.19355 | 10 | 0.32258 |
| 3456 | S4_31850202 | 4 | 31850202 | 94814 | A | G | A | 0.91935 | G | 0.08065 | 3 | 0.09677 |
| 3457 | S4_31850247 | 4 | 31850247 | 45 | A | C | A | 0.74194 | C | 0.25806 | 14 | 0.45161 |
| 3458 | S4_31850400 | 4 | 31850400 | 153 | G | A | G | 0.91935 | A | 0.08065 | 3 | 0.09677 |
| 3459 | S4_32274298 | 4 | 32274298 | 423898 | G | A | G | 0.85484 | A | 0.14516 | 9 | 0.29032 |
| 3460 | S4_32358338 | 4 | 32358338 | 84040 | C | T | T | 0.91935 | C | 0.08065 | 5 | 0.16129 |
| 3461 | S4_32358427 | 4 | 32358427 | 89 | A | C | C | 0.91935 | A | 0.08065 | 5 | 0.16129 |
| 3462 | S4_32358480 | 4 | 32358480 | 53 | T | G | G | 0.91935 | T | 0.08065 | 5 | 0.16129 |
| 3463 | S4_32696876 | 4 | 32696876 | 338396 | A | G | G | 0.77419 | A | 0.22581 | 10 | 0.32258 |
| 3464 | S4_32918945 | 4 | 32918945 | 222069 | G | C | G | 0.6129 | C | 0.3871 | 18 | 0.58065 |
| 3465 | S4_32918965 | 4 | 32918965 | 20 | C | T | C | 0.82258 | T | 0.17742 | 11 | 0.35484 |
| 3466 | S4_32919142 | 4 | 32919142 | 177 | T | C | T | 0.6129 | C | 0.3871 | 18 | 0.58065 |
| 3467 | S4_32994750 | 4 | 32994750 | 75608 | T | C | T | 0.8871 | C | 0.1129 | 7 | 0.22581 |
| 3468 | S4_32994778 | 4 | 32994778 | 28 | A | G | G | 0.58065 | A | 0.41935 | 16 | 0.51613 |
| 3469 | S4_32994839 | 4 | 32994839 | 61 | T | G | T | 0.6129 | G | 0.3871 | 16 | 0.51613 |
| 3470 | S4_32994935 | 4 | 32994935 | 96 | G | A | G | 0.93548 | A | 0.06452 | 4 | 0.12903 |
| 3471 | S4_33051024 | 4 | 33051024 | 56089 | G | A | G | 0.8871 | A | 0.1129 | 7 | 0.22581 |
| 3472 | S4_33051157 | 4 | 33051157 | 133 | G | A | G | 0.72581 | A | 0.27419 | 13 | 0.41935 |
| 3473 | S4_33255707 | 4 | 33255707 | 204550 | T | A | A | 0.62903 | T | 0.37097 | 13 | 0.41935 |
| 3474 | S4_33667132 | 4 | 33667132 | 411425 | A | G | A | 0.69355 | G | 0.30645 | 15 | 0.48387 |
| 3475 | S4_34364905 | 4 | 34364905 | 697773 | T | C | T | 0.77419 | C | 0.22581 | 12 | 0.3871 |
| 3476 | S4_34364924 | 4 | 34364924 | 19 | A | G | G | 0.8871 | A | 0.1129 | 7 | 0.22581 |
| 3477 | S4_34545085 | 4 | 34545085 | 180161 | T | C | C | 0.74194 | T | 0.25806 | 12 | 0.3871 |
| 3478 | S4_34545087 | 4 | 34545087 | 2 | C | G | C | 0.91935 | G | 0.08065 | 5 | 0.16129 |
| 3479 | S4_34545154 | 4 | 34545154 | 67 | A | G | G | 0.91935 | A | 0.08065 | 3 | 0.09677 |
| 3480 | S4_36342374 | 4 | 36342374 | 1797220 | C | T | C | 0.93548 | T | 0.06452 | 4 | 0.12903 |
| 3481 | S4_36342538 | 4 | 36342538 | 164 | C | T | C | 0.82258 | T | 0.17742 | 9 | 0.29032 |
| 3482 | S4_37022328 | 4 | 37022328 | 679790 | G | T | T | 0.90323 | G | 0.09677 | 6 | 0.19355 |
| 3483 | S4_37022456 | 4 | 37022456 | 128 | C | T | C | 0.91935 | T | 0.08065 | 5 | 0.16129 |
| 3484 | S4_37457309 | 4 | 37457309 | 434853 | C | T | C | 0.8871 | T | 0.1129 | 7 | 0.22581 |
| 3485 | S4_37880839 | 4 | 37880839 | 423530 | C | T | C | 0.90323 | T | 0.09677 | 6 | 0.19355 |
| 3486 | S4_38103898 | 4 | 38103898 | 223059 | G | T | G | 0.77419 | T | 0.22581 | 10 | 0.32258 |
| 3487 | S4_38217615 | 4 | 38217615 | 113717 | C | T | C | 0.93548 | T | 0.06452 | 4 | 0.12903 |
| 3488 | S4_38522133 | 4 | 38522133 | 304518 | G | T | G | 0.59677 | T | 0.40323 | 11 | 0.35484 |
| 3489 | S4_38653478 | 4 | 38653478 | 131345 | C | T | C | 0.75806 | T | 0.24194 | 11 | 0.35484 |
| 3490 | S4_38653487 | 4 | 38653487 | 9 | G | A | G | 0.67742 | A | 0.32258 | 14 | 0.45161 |
| 3491 | S4_39727748 | 4 | 39727748 | 1074261 | G | C | G | 0.87097 | C | 0.12903 | 8 | 0.25806 |
| 3492 | S4_39727796 | 4 | 39727796 | 48 | A | T | A | 0.64516 | T | 0.35484 | 16 | 0.51613 |
| 3493 | S4_39727839 | 4 | 39727839 | 43 | A | C | A | 0.83871 | C | 0.16129 | 10 | 0.32258 |
| 3494 | S4_39981299 | 4 | 39981299 | 253460 | C | T | C | 0.58065 | T | 0.41935 | 14 | 0.45161 |
| 3495 | S4_40654623 | 4 | 40654623 | 673324 | C | T | C | 0.74194 | T | 0.25806 | 10 | 0.32258 |
| 3496 | S4_40911307 | 4 | 40911307 | 256684 | C | T | C | 0.87097 | T | 0.12903 | 6 | 0.19355 |
| 3497 | S4_41075934 | 4 | 41075934 | 164627 | G | A | G | 0.91935 | A | 0.08065 | 5 | 0.16129 |
| 3498 | S4_41076020 | 4 | 41076020 | 86 | C | T | C | 0.91935 | T | 0.08065 | 5 | 0.16129 |
| 3499 | S4_41141177 | 4 | 41141177 | 65157 | A | C | A | 0.79032 | C | 0.20968 | 11 | 0.35484 |
| 3500 | S4_41141247 | 4 | 41141247 | 70 | G | A | G | 0.93548 | A | 0.06452 | 4 | 0.12903 |
| 3501 | S4_41587123 | 4 | 41587123 | 445876 | T | G | T | 0.77419 | G | 0.22581 | 12 | 0.3871 |
| 3502 | S4_42509850 | 4 | 42509850 | 922727 | T | C | C | 0.8871 | T | 0.1129 | 5 | 0.16129 |
| 3503 | S4_42509949 | 4 | 42509949 | 99 | A | G | G | 0.8871 | A | 0.1129 | 5 | 0.16129 |
| 3504 | S4_42509962 | 4 | 42509962 | 13 | T | C | C | 0.8871 | T | 0.1129 | 5 | 0.16129 |
| 3505 | S4_43232383 | 4 | 43232383 | 722421 | G | A | G | 0.90323 | A | 0.09677 | 6 | 0.19355 |
| 3506 | S4_44282402 | 4 | 44282402 | 1050019 | C | T | C | 0.87097 | T | 0.12903 | 6 | 0.19355 |
| 3507 | S4_44282404 | 4 | 44282404 | 2 | C | T | C | 0.93548 | T | 0.06452 | 4 | 0.12903 |
| 3508 | S4_45267241 | 4 | 45267241 | 984837 | T | C | T | 0.67742 | C | 0.32258 | 10 | 0.32258 |

| Site number | SNP name | Chromosome | Physical position | Physical distance from the previous SNP | Reference allele | Alternative allele | Major allele | Major allele frequency | Minor allele | Minor allele frequency | Number heterozygous | Heterozygosity |
|---|---|---|---|---|---|---|---|---|---|---|---|---|
| 3509 | S4_45289206 | 4 | 45289206 | 21965 | G | A | A | 0.53226 | G | 0.46774 | 15 | 0.48387 |
| 3510 | S4_45289217 | 4 | 45289217 | 11 | G | C | G | 0.91935 | C | 0.08065 | 5 | 0.16129 |
| 3511 | S4_45289281 | 4 | 45289281 | 64 | G | A | G | 0.91935 | A | 0.08065 | 5 | 0.16129 |
| 3512 | S4_45458615 | 4 | 45458615 | 169334 | A | G | A | 0.64516 | G | 0.35484 | 14 | 0.45161 |
| 3513 | S4_45513620 | 4 | 45513620 | 55005 | C | T | C | 0.91935 | T | 0.08065 | 5 | 0.16129 |
| 3514 | S4_45721939 | 4 | 45721939 | 208319 | G | A | G | 0.95161 | A | 0.04839 | 3 | 0.09677 |
| 3515 | S4_45722130 | 4 | 45722130 | 191 | C | T | C | 0.95161 | T | 0.04839 | 3 | 0.09677 |
| 3516 | S4_45966822 | 4 | 45966822 | 244692 | C | A | C | 0.95161 | A | 0.04839 | 3 | 0.09677 |
| 3517 | S4_45966827 | 4 | 45966827 | 5 | G | A | G | 0.93548 | A | 0.06452 | 4 | 0.12903 |
| 3518 | S4_45966897 | 4 | 45966897 | 70 | A | G | A | 0.93548 | G | 0.06452 | 4 | 0.12903 |
| 3519 | S4_46293607 | 4 | 46293607 | 326710 | G | A | G | 0.62903 | A | 0.37097 | 13 | 0.41935 |
| 3520 | S4_46293800 | 4 | 46293800 | 193 | C | T | C | 0.54839 | T | 0.45161 | 16 | 0.51613 |
| 3521 | S4_46293824 | 4 | 46293824 | 24 | G | A | G | 0.85484 | A | 0.14516 | 7 | 0.22581 |
| 3522 | S4_46824682 | 4 | 46824682 | 530858 | A | G | A | 0.83871 | G | 0.16129 | 8 | 0.25806 |
| 3523 | S4_46951360 | 4 | 46951360 | 126678 | A | G | G | 0.56452 | A | 0.43548 | 17 | 0.54839 |
| 3524 | S4_46951419 | 4 | 46951419 | 59 | C | T | C | 0.54839 | T | 0.45161 | 14 | 0.45161 |
| 3525 | S4_46951529 | 4 | 46951529 | 110 | G | A | G | 0.8871 | A | 0.1129 | 7 | 0.22581 |
| 3526 | S4_46951556 | 4 | 46951556 | 27 | C | T | C | 0.8871 | T | 0.1129 | 7 | 0.22581 |
| 3527 | S4_47689279 | 4 | 47689279 | 737723 | T | C | T | 0.85484 | C | 0.14516 | 9 | 0.29032 |
| 3528 | S4_47851222 | 4 | 47851222 | 161943 | C | T | C | 0.90323 | T | 0.09677 | 6 | 0.19355 |
| 3529 | S4_47898220 | 4 | 47898220 | 46998 | C | T | C | 0.87097 | T | 0.12903 | 8 | 0.25806 |
| 3530 | S4_47898380 | 4 | 47898380 | 160 | T | C | T | 0.56452 | C | 0.43548 | 15 | 0.48387 |
| 3531 | S4_48180452 | 4 | 48180452 | 282072 | G | A | A | 0.59677 | G | 0.40323 | 11 | 0.35484 |
| 3532 | S4_48451185 | 4 | 48451185 | 270733 | T | C | T | 0.79032 | C | 0.20968 | 11 | 0.35484 |
| 3533 | S4_48542183 | 4 | 48542183 | 90998 | T | C | C | 0.53226 | T | 0.46774 | 11 | 0.35484 |
| 3534 | S4_48542354 | 4 | 48542354 | 171 | A | G | A | 0.70968 | G | 0.29032 | 10 | 0.32258 |
| 3535 | S4_48760768 | 4 | 48760768 | 218414 | G | A | G | 0.90323 | A | 0.09677 | 6 | 0.19355 |
| 3536 | S4_49486570 | 4 | 49486570 | 725802 | C | T | C | 0.93548 | T | 0.06452 | 4 | 0.12903 |
| 3537 | S4_49486681 | 4 | 49486681 | 111 | G | A | G | 0.91935 | A | 0.08065 | 5 | 0.16129 |
| 3538 | S4_49582862 | 4 | 49582862 | 96181 | C | T | C | 0.90323 | T | 0.09677 | 6 | 0.19355 |
| 3539 | S4_49582903 | 4 | 49582903 | 41 | G | A | G | 0.75806 | A | 0.24194 | 11 | 0.35484 |
| 3540 | S4_50353262 | 4 | 50353262 | 770359 | G | T | G | 0.93548 | T | 0.06452 | 4 | 0.12903 |
| 3541 | S4_50353362 | 4 | 50353362 | 100 | A | G | A | 0.64516 | G | 0.35484 | 14 | 0.45161 |
| 3542 | S4_50385704 | 4 | 50385704 | 32342 | G | A | G | 0.95161 | A | 0.04839 | 3 | 0.09677 |
| 3543 | S4_50570703 | 4 | 50570703 | 184999 | G | T | G | 0.80645 | T | 0.19355 | 8 | 0.25806 |
| 3544 | S4_50570916 | 4 | 50570916 | 213 | C | G | C | 0.93548 | G | 0.06452 | 4 | 0.12903 |
| 3545 | S4_50854430 | 4 | 50854430 | 283514 | T | C | T | 0.77419 | C | 0.22581 | 10 | 0.32258 |
| 3546 | S4_50854438 | 4 | 50854438 | 8 | A | G | A | 0.93548 | G | 0.06452 | 4 | 0.12903 |
| 3547 | S4_50854513 | 4 | 50854513 | 75 | G | A | G | 0.80645 | A | 0.19355 | 10 | 0.32258 |
| 3548 | S4_50854579 | 4 | 50854579 | 66 | C | T | C | 0.74194 | T | 0.25806 | 14 | 0.45161 |
| 3549 | S4_50858578 | 4 | 50858578 | 3999 | G | T | G | 0.8871 | T | 0.1129 | 7 | 0.22581 |
| 3550 | S4_50858633 | 4 | 50858633 | 55 | C | A | C | 0.87097 | A | 0.12903 | 6 | 0.19355 |
| 3551 | S4_50858663 | 4 | 50858663 | 30 | G | A | G | 0.90323 | A | 0.09677 | 4 | 0.12903 |
| 3552 | S4_50873759 | 4 | 50873759 | 15096 | T | A | T | 0.80645 | A | 0.19355 | 8 | 0.25806 |
| 3553 | S4_50873779 | 4 | 50873779 | 20 | A | G | A | 0.82258 | G | 0.17742 | 9 | 0.29032 |
| 3554 | S4_50873801 | 4 | 50873801 | 22 | G | C | G | 0.90323 | C | 0.09677 | 6 | 0.19355 |
| 3555 | S4_50873831 | 4 | 50873831 | 30 | G | A | G | 0.82258 | A | 0.17742 | 9 | 0.29032 |
| 3556 | S4_50948344 | 4 | 50948344 | 74513 | C | T | C | 0.90323 | T | 0.09677 | 6 | 0.19355 |
| 3557 | S4_51011146 | 4 | 51011146 | 62802 | G | C | G | 0.8871 | C | 0.1129 | 5 | 0.16129 |
| 3558 | S4_51214200 | 4 | 51214200 | 203054 | G | A | G | 0.93548 | A | 0.06452 | 4 | 0.12903 |
| 3559 | S4_51623161 | 4 | 51623161 | 408961 | C | T | C | 0.83871 | T | 0.16129 | 8 | 0.25806 |
| 3560 | S4_51786665 | 4 | 51786665 | 163504 | A | G | A | 0.87097 | G | 0.12903 | 8 | 0.25806 |
| 3561 | S4_51811224 | 4 | 51811224 | 24559 | C | T | C | 0.79032 | T | 0.20968 | 11 | 0.35484 |
| 3562 | S4_51837499 | 4 | 51837499 | 26275 | C | T | C | 0.82258 | T | 0.17742 | 9 | 0.29032 |

| Site number | SNP name | Chromosome | Physical position | Physical distance from the previous SNP | Reference allele | Alternative allele | Major allele | Major allele frequency | Minor allele | Minor allele frequency | Number heterozygous | Heterozygosity |
|---|---|---|---|---|---|---|---|---|---|---|---|---|
| 3563 | S4_51874707 | 4 | 51874707 | 37208 | C | T | C | 0.70968 | T | 0.29032 | 14 | 0.45161 |
| 3564 | S4_52026285 | 4 | 52026285 | 151578 | C | T | C | 0.69355 | T | 0.30645 | 9 | 0.29032 |
| 3565 | S4_52026286 | 4 | 52026286 | 1 | G | T | G | 0.85484 | T | 0.14516 | 5 | 0.16129 |
| 3566 | S4_52026291 | 4 | 52026291 | 5 | C | T | C | 0.79032 | T | 0.20968 | 7 | 0.22581 |
| 3567 | S4_52026314 | 4 | 52026314 | 23 | G | A | G | 0.91935 | A | 0.08065 | 5 | 0.16129 |
| 3568 | S4_52247217 | 4 | 52247217 | 220903 | G | A | A | 0.62903 | G | 0.37097 | 13 | 0.41935 |
| 3569 | S4_52247224 | 4 | 52247224 | 7 | G | A | G | 0.95161 | A | 0.04839 | 3 | 0.09677 |
| 3570 | S4_52664848 | 4 | 52664848 | 417624 | G | A | G | 0.95161 | A | 0.04839 | 3 | 0.09677 |
| 3571 | S4_52664905 | 4 | 52664905 | 57 | G | A | G | 0.91935 | A | 0.08065 | 5 | 0.16129 |
| 3572 | S4_52997977 | 4 | 52997977 | 333072 | A | G | A | 0.83871 | G | 0.16129 | 6 | 0.19355 |
| 3573 | S4_53151975 | 4 | 53151975 | 153998 | C | A | C | 0.8871 | A | 0.1129 | 5 | 0.16129 |
| 3574 | S4_53152002 | 4 | 53152002 | 27 | T | C | T | 0.58065 | C | 0.41935 | 14 | 0.45161 |
| 3575 | S4_53152038 | 4 | 53152038 | 36 | A | G | G | 0.53226 | A | 0.46774 | 13 | 0.41935 |
| 3576 | S4_53494012 | 4 | 53494012 | 341974 | C | T | C | 0.87097 | T | 0.12903 | 6 | 0.19355 |
| 3577 | S4_53494136 | 4 | 53494136 | 124 | T | A | T | 0.53226 | A | 0.46774 | 13 | 0.41935 |
| 3578 | S4_53494247 | 4 | 53494247 | 111 | A | G | A | 0.74194 | G | 0.25806 | 10 | 0.32258 |
| 3579 | S4_53591634 | 4 | 53591634 | 97387 | G | T | G | 0.93548 | T | 0.06452 | 4 | 0.12903 |
| 3580 | S4_53591693 | 4 | 53591693 | 59 | T | C | T | 0.93548 | C | 0.06452 | 4 | 0.12903 |
| 3581 | S4_53675490 | 4 | 53675490 | 83797 | C | T | T | 0.91935 | C | 0.08065 | 5 | 0.16129 |
| 3582 | S4_53675660 | 4 | 53675660 | 170 | C | T | C | 0.95161 | T | 0.04839 | 3 | 0.09677 |
| 3583 | S4_53912122 | 4 | 53912122 | 236462 | C | T | C | 0.93548 | T | 0.06452 | 4 | 0.12903 |
| 3584 | S4_54208431 | 4 | 54208431 | 296309 | G | T | G | 0.79032 | T | 0.20968 | 9 | 0.29032 |
| 3585 | S4_54208603 | 4 | 54208603 | 172 | C | T | C | 0.80645 | T | 0.19355 | 8 | 0.25806 |
| 3586 | S4_54415344 | 4 | 54415344 | 206741 | G | A | G | 0.80645 | A | 0.19355 | 8 | 0.25806 |
| 3587 | S4_54586268 | 4 | 54586268 | 170924 | C | T | C | 0.75806 | T | 0.24194 | 13 | 0.41935 |
| 3588 | S4_54586455 | 4 | 54586455 | 187 | G | C | G | 0.8871 | C | 0.1129 | 7 | 0.22581 |
| 3589 | S4_54681368 | 4 | 54681368 | 94913 | C | A | C | 0.58065 | A | 0.41935 | 12 | 0.3871 |
| 3590 | S4_54681407 | 4 | 54681407 | 39 | C | T | C | 0.93548 | T | 0.06452 | 4 | 0.12903 |
| 3591 | S4_54681408 | 4 | 54681408 | 1 | C | T | C | 0.62903 | T | 0.37097 | 9 | 0.29032 |
| 3592 | S4_54895827 | 4 | 54895827 | 214419 | G | A | G | 0.80645 | A | 0.19355 | 10 | 0.32258 |
| 3593 | S4_54895889 | 4 | 54895889 | 62 | T | C | T | 0.80645 | C | 0.19355 | 10 | 0.32258 |
| 3594 | S4_54895976 | 4 | 54895976 | 87 | G | A | G | 0.91935 | A | 0.08065 | 5 | 0.16129 |
| 3595 | S4_54895992 | 4 | 54895992 | 16 | A | G | A | 0.80645 | G | 0.19355 | 10 | 0.32258 |
| 3596 | S4_54896003 | 4 | 54896003 | 11 | G | A | G | 0.83871 | A | 0.16129 | 10 | 0.32258 |
| 3597 | S4_54896031 | 4 | 54896031 | 28 | G | C | G | 0.91935 | C | 0.08065 | 5 | 0.16129 |
| 3598 | S4_55196728 | 4 | 55196728 | 300697 | G | A | G | 0.54839 | A | 0.45161 | 16 | 0.51613 |
| 3599 | S4_55308348 | 4 | 55308348 | 111620 | A | G | A | 0.93548 | G | 0.06452 | 4 | 0.12903 |
| 3600 | S4_55308403 | 4 | 55308403 | 55 | G | A | G | 0.93548 | A | 0.06452 | 4 | 0.12903 |
| 3601 | S4_55308440 | 4 | 55308440 | 37 | C | T | C | 0.8871 | T | 0.1129 | 5 | 0.16129 |
| 3602 | S4_55308488 | 4 | 55308488 | 48 | C | T | C | 0.83871 | T | 0.16129 | 8 | 0.25806 |
| 3603 | S4_55623544 | 4 | 55623544 | 315056 | T | C | T | 0.8871 | C | 0.1129 | 7 | 0.22581 |
| 3604 | S4_55623563 | 4 | 55623563 | 19 | C | T | C | 0.91935 | T | 0.08065 | 5 | 0.16129 |
| 3605 | S4_55623668 | 4 | 55623668 | 105 | A | G | G | 0.83871 | A | 0.16129 | 8 | 0.25806 |
| 3606 | S4_55623729 | 4 | 55623729 | 61 | G | A | G | 0.91935 | A | 0.08065 | 5 | 0.16129 |
| 3607 | S4_55623752 | 4 | 55623752 | 23 | A | G | A | 0.72581 | G | 0.27419 | 13 | 0.41935 |
| 3608 | S4_56108856 | 4 | 56108856 | 485104 | A | G | G | 0.72581 | A | 0.27419 | 13 | 0.41935 |
| 3609 | S4_56108897 | 4 | 56108897 | 41 | G | A | G | 0.91935 | A | 0.08065 | 3 | 0.09677 |
| 3610 | S4_56159344 | 4 | 56159344 | 50447 | C | A | A | 0.75806 | C | 0.24194 | 11 | 0.35484 |
| 3611 | S4_56159356 | 4 | 56159356 | 12 | A | G | G | 0.75806 | A | 0.24194 | 11 | 0.35484 |
| 3612 | S4_56159489 | 4 | 56159489 | 133 | T | C | C | 0.75806 | T | 0.24194 | 11 | 0.35484 |
| 3613 | S4_56159497 | 4 | 56159497 | 8 | G | A | G | 0.95161 | A | 0.04839 | 3 | 0.09677 |
| 3614 | S4_56411446 | 4 | 56411446 | 251949 | A | G | A | 0.67742 | G | 0.32258 | 16 | 0.51613 |
| 3615 | S4_56818310 | 4 | 56818310 | 406864 | A | G | A | 0.93548 | G | 0.06452 | 4 | 0.12903 |
| 3616 | S4_56893575 | 4 | 56893575 | 75265 | C | T | T | 0.75806 | C | 0.24194 | 11 | 0.35484 |

| Site number | SNP name | Chromosome | Physical position | Physical distance from the previous SNP | Reference allele | Alternative allele | Major allele | Major allele frequency | Minor allele | Minor allele frequency | Number heterozygous | Heterozygosity |
|---|---|---|---|---|---|---|---|---|---|---|---|---|
| 3617 | S4_56893653 | 4 | 56893653 | 78 | C | T | T | 0.82258 | C | 0.17742 | 9 | 0.29032 |
| 3618 | S4_56893659 | 4 | 56893659 | 6 | T | C | C | 0.82258 | T | 0.17742 | 9 | 0.29032 |
| 3619 | S4_57916356 | 4 | 57916356 | 1022697 | G | A | G | 0.91935 | A | 0.08065 | 5 | 0.16129 |
| 3620 | S4_57916489 | 4 | 57916489 | 133 | T | C | T | 0.72581 | C | 0.27419 | 13 | 0.41935 |
| 3621 | S4_58151051 | 4 | 58151051 | 234562 | T | C | T | 0.58065 | C | 0.41935 | 14 | 0.45161 |
| 3622 | S4_58266908 | 4 | 58266908 | 115857 | T | G | T | 0.93548 | G | 0.06452 | 4 | 0.12903 |
| 3623 | S4_58266916 | 4 | 58266916 | 8 | G | A | G | 0.95161 | A | 0.04839 | 3 | 0.09677 |
| 3624 | S4_58266954 | 4 | 58266954 | 38 | T | C | T | 0.91935 | C | 0.08065 | 3 | 0.09677 |
| 3625 | S4_58267051 | 4 | 58267051 | 97 | G | A | G | 0.77419 | A | 0.22581 | 10 | 0.32258 |
| 3626 | S4_58270077 | 4 | 58270077 | 3026 | C | T | C | 0.8871 | T | 0.1129 | 7 | 0.22581 |
| 3627 | S4_58270166 | 4 | 58270166 | 89 | G | A | G | 0.53226 | A | 0.46774 | 13 | 0.41935 |
| 3628 | S4_58270181 | 4 | 58270181 | 15 | C | G | C | 0.51613 | G | 0.48387 | 18 | 0.58065 |
| 3629 | S4_58270250 | 4 | 58270250 | 69 | G | A | G | 0.91935 | A | 0.08065 | 3 | 0.09677 |
| 3630 | S4_58270252 | 4 | 58270252 | 2 | A | G | G | 0.70968 | A | 0.29032 | 16 | 0.51613 |
| 3631 | S4_58270253 | 4 | 58270253 | 1 | T | A | T | 0.91935 | A | 0.08065 | 3 | 0.09677 |
| 3632 | S4_58270283 | 4 | 58270283 | 30 | C | T | C | 0.91935 | T | 0.08065 | 3 | 0.09677 |
| 3633 | S4_58475176 | 4 | 58475176 | 204893 | C | T | C | 0.93548 | T | 0.06452 | 4 | 0.12903 |
| 3634 | S4_58475253 | 4 | 58475253 | 77 | G | A | G | 0.93548 | A | 0.06452 | 4 | 0.12903 |
| 3635 | S4_58477755 | 4 | 58477755 | 2502 | G | A | G | 0.8871 | A | 0.1129 | 7 | 0.22581 |
| 3636 | S4_58477779 | 4 | 58477779 | 24 | C | T | C | 0.95161 | T | 0.04839 | 3 | 0.09677 |
| 3637 | S4_58477799 | 4 | 58477799 | 20 | G | T | G | 0.93548 | T | 0.06452 | 4 | 0.12903 |
| 3638 | S4_58562765 | 4 | 58562765 | 84966 | C | T | C | 0.91935 | T | 0.08065 | 5 | 0.16129 |
| 3639 | S4_58562807 | 4 | 58562807 | 42 | G | A | G | 0.6129 | A | 0.3871 | 14 | 0.45161 |
| 3640 | S4_58562848 | 4 | 58562848 | 41 | T | A | T | 0.6129 | A | 0.3871 | 14 | 0.45161 |
| 3641 | S4_58562874 | 4 | 58562874 | 26 | G | A | G | 0.91935 | A | 0.08065 | 5 | 0.16129 |
| 3642 | S4_58562931 | 4 | 58562931 | 57 | G | A | G | 0.6129 | A | 0.3871 | 14 | 0.45161 |
| 3643 | S4_58562932 | 4 | 58562932 | 1 | T | C | T | 0.93548 | C | 0.06452 | 4 | 0.12903 |
| 3644 | S4_58768361 | 4 | 58768361 | 205429 | A | G | A | 0.91935 | G | 0.08065 | 5 | 0.16129 |
| 3645 | S4_59178460 | 4 | 59178460 | 410099 | C | A | C | 0.90323 | A | 0.09677 | 6 | 0.19355 |
| 3646 | S4_59868526 | 4 | 59868526 | 690066 | T | C | C | 0.64516 | T | 0.35484 | 14 | 0.45161 |
| 3647 | S4_59868617 | 4 | 59868617 | 91 | G | A | G | 0.66129 | A | 0.33871 | 15 | 0.48387 |
| 3648 | S4_59868688 | 4 | 59868688 | 71 | G | T | G | 0.93548 | T | 0.06452 | 4 | 0.12903 |
| 3649 | S4_59982757 | 4 | 59982757 | 114069 | A | C | A | 0.91935 | C | 0.08065 | 5 | 0.16129 |
| 3650 | S4_60316665 | 4 | 60316665 | 333908 | C | T | C | 0.91935 | T | 0.08065 | 5 | 0.16129 |
| 3651 | S4_60916629 | 4 | 60916629 | 599964 | T | G | G | 0.51613 | T | 0.48387 | 12 | 0.3871 |
| 3652 | S4_60916674 | 4 | 60916674 | 45 | T | C | T | 0.74194 | C | 0.25806 | 12 | 0.3871 |
| 3653 | S4_60916704 | 4 | 60916704 | 30 | A | G | G | 0.83871 | A | 0.16129 | 10 | 0.32258 |
| 3654 | S4_60916786 | 4 | 60916786 | 82 | C | G | C | 0.51613 | G | 0.48387 | 14 | 0.45161 |
| 3655 | S4_61009111 | 4 | 61009111 | 92325 | G | A | A | 0.79032 | G | 0.20968 | 7 | 0.22581 |
| 3656 | S4_61621642 | 4 | 61621642 | 612531 | A | G | A | 0.69355 | G | 0.30645 | 11 | 0.35484 |
| 3657 | S4_62014691 | 4 | 62014691 | 393049 | C | T | C | 0.95161 | T | 0.04839 | 3 | 0.09677 |
| 3658 | S4_62014780 | 4 | 62014780 | 89 | A | G | G | 0.8871 | A | 0.1129 | 5 | 0.16129 |
| 3659 | S4_62014884 | 4 | 62014884 | 104 | G | A | G | 0.95161 | A | 0.04839 | 3 | 0.09677 |
| 3660 | S4_62014901 | 4 | 62014901 | 17 | G | A | G | 0.93548 | A | 0.06452 | 4 | 0.12903 |
| 3661 | S4_62359050 | 4 | 62359050 | 344149 | T | A | T | 0.93548 | A | 0.06452 | 4 | 0.12903 |
| 3662 | S4_62548550 | 4 | 62548550 | 189500 | T | C | T | 0.72581 | C | 0.27419 | 15 | 0.48387 |
| 3663 | S4_62548555 | 4 | 62548555 | 5 | C | T | C | 0.91935 | T | 0.08065 | 5 | 0.16129 |
| 3664 | S4_62548607 | 4 | 62548607 | 52 | C | T | C | 0.72581 | T | 0.27419 | 13 | 0.41935 |
| 3665 | S4_63129100 | 4 | 63129100 | 580493 | C | T | C | 0.75806 | T | 0.24194 | 13 | 0.41935 |
| 3666 | S4_63187626 | 4 | 63187626 | 58526 | T | A | A | 0.79032 | T | 0.20968 | 11 | 0.35484 |
| 3667 | S4_63187658 | 4 | 63187658 | 32 | G | A | G | 0.87097 | A | 0.12903 | 6 | 0.19355 |
| 3668 | S4_63187666 | 4 | 63187666 | 8 | G | A | G | 0.51613 | A | 0.48387 | 18 | 0.58065 |
| 3669 | S4_63187831 | 4 | 63187831 | 165 | G | A | G | 0.8871 | A | 0.1129 | 5 | 0.16129 |
| 3670 | S4_63983231 | 4 | 63983231 | 795400 | T | C | C | 0.90323 | T | 0.09677 | 6 | 0.19355 |

| Site number | SNP name | Chromosome | Physical position | Physical distance from the previous SNP | Reference allele | Alternative allele | Major allele | Major allele frequency | Minor allele | Minor allele frequency | Number heterozygous | Heterozygosity |
|---|---|---|---|---|---|---|---|---|---|---|---|---|
| 3671 | S4_64693185 | 4 | 64693185 | 709954 | C | T | T | 0.80645 | C | 0.19355 | 12 | 0.3871 |
| 3672 | S4_64693192 | 4 | 64693192 | 7 | G | T | G | 0.82258 | T | 0.17742 | 11 | 0.35484 |
| 3673 | S4_65963824 | 4 | 65963824 | 1270632 | G | A | G | 0.93548 | A | 0.06452 | 4 | 0.12903 |
| 3674 | S4_65963924 | 4 | 65963924 | 100 | G | A | G | 0.8871 | A | 0.1129 | 5 | 0.16129 |
| 3675 | S4_66303616 | 4 | 66303616 | 339692 | G | A | G | 0.95161 | A | 0.04839 | 1 | 0.03226 |
| 3676 | S4_66832442 | 4 | 66832442 | 528826 | A | T | A | 0.85484 | T | 0.14516 | 9 | 0.29032 |
| 3677 | S4_66832528 | 4 | 66832528 | 86 | G | A | G | 0.93548 | A | 0.06452 | 4 | 0.12903 |
| 3678 | S4_68177833 | 4 | 68177833 | 1345305 | G | T | G | 0.82258 | T | 0.17742 | 9 | 0.29032 |
| 3679 | S4_68597426 | 4 | 68597426 | 419593 | C | T | C | 0.90323 | T | 0.09677 | 6 | 0.19355 |
| 3680 | S4_68937421 | 4 | 68937421 | 339995 | G | A | A | 0.5 | G | 0.5 | 17 | 0.54839 |
| 3681 | S4_69456051 | 4 | 69456051 | 518630 | G | A | G | 0.83871 | A | 0.16129 | 6 | 0.19355 |
| 3682 | S4_70053646 | 4 | 70053646 | 597595 | G | T | G | 0.93548 | T | 0.06452 | 4 | 0.12903 |
| 3683 | S4_70249683 | 4 | 70249683 | 196037 | T | C | T | 0.83871 | C | 0.16129 | 8 | 0.25806 |
| 3684 | S4_70249728 | 4 | 70249728 | 45 | A | G | A | 0.51613 | G | 0.48387 | 16 | 0.51613 |
| 3685 | S4_70249763 | 4 | 70249763 | 35 | G | C | C | 0.5 | G | 0.5 | 15 | 0.48387 |
| 3686 | S4_70285499 | 4 | 70285499 | 35736 | G | A | G | 0.90323 | A | 0.09677 | 6 | 0.19355 |
| 3687 | S4_70285696 | 4 | 70285696 | 197 | C | T | C | 0.87097 | T | 0.12903 | 8 | 0.25806 |
| 3688 | S4_70896533 | 4 | 70896533 | 610837 | A | G | A | 0.8871 | G | 0.1129 | 5 | 0.16129 |
| 3689 | S4_70896627 | 4 | 70896627 | 94 | C | T | C | 0.93548 | T | 0.06452 | 4 | 0.12903 |
| 3690 | S4_71131188 | 4 | 71131188 | 234561 | G | A | A | 0.79032 | G | 0.20968 | 7 | 0.22581 |
| 3691 | S4_71280744 | 4 | 71280744 | 149556 | C | T | C | 0.72581 | T | 0.27419 | 11 | 0.35484 |
| 3692 | S4_71352421 | 4 | 71352421 | 71677 | T | C | T | 0.83871 | C | 0.16129 | 8 | 0.25806 |
| 3693 | S4_71352435 | 4 | 71352435 | 14 | C | T | C | 0.83871 | T | 0.16129 | 8 | 0.25806 |
| 3694 | S4_71352450 | 4 | 71352450 | 15 | C | G | G | 0.53226 | C | 0.46774 | 17 | 0.54839 |
| 3695 | S4_71352614 | 4 | 71352614 | 164 | C | T | C | 0.90323 | T | 0.09677 | 6 | 0.19355 |
| 3696 | S4_72409226 | 4 | 72409226 | 1056612 | C | T | T | 0.51613 | C | 0.48387 | 12 | 0.3871 |
| 3697 | S4_72409250 | 4 | 72409250 | 24 | T | G | G | 0.83871 | T | 0.16129 | 10 | 0.32258 |
| 3698 | S4_72409272 | 4 | 72409272 | 22 | C | T | C | 0.95161 | T | 0.04839 | 3 | 0.09677 |
| 3699 | S4_72774538 | 4 | 72774538 | 365266 | C | T | C | 0.90323 | T | 0.09677 | 4 | 0.12903 |
| 3700 | S4_72774557 | 4 | 72774557 | 19 | A | G | A | 0.59677 | G | 0.40323 | 15 | 0.48387 |
| 3701 | S4_72774615 | 4 | 72774615 | 58 | C | G | C | 0.83871 | G | 0.16129 | 8 | 0.25806 |
| 3702 | S4_72823922 | 4 | 72823922 | 49307 | A | G | G | 0.82258 | A | 0.17742 | 9 | 0.29032 |
| 3703 | S4_73423710 | 4 | 73423710 | 599788 | G | T | G | 0.91935 | T | 0.08065 | 5 | 0.16129 |
| 3704 | S4_73688602 | 4 | 73688602 | 264892 | C | T | C | 0.72581 | T | 0.27419 | 13 | 0.41935 |
| 3705 | S4_73688614 | 4 | 73688614 | 12 | T | C | C | 0.67742 | T | 0.32258 | 14 | 0.45161 |
| 3706 | S4_73902323 | 4 | 73902323 | 213709 | G | T | G | 0.70968 | T | 0.29032 | 8 | 0.25806 |
| 3707 | S4_73907477 | 4 | 73907477 | 5154 | G | C | C | 0.79032 | G | 0.20968 | 9 | 0.29032 |
| 3708 | S4_73907557 | 4 | 73907557 | 80 | A | G | A | 0.53226 | G | 0.46774 | 13 | 0.41935 |
| 3709 | S4_74188638 | 4 | 74188638 | 281081 | A | T | A | 0.91935 | T | 0.08065 | 3 | 0.09677 |
| 3710 | S4_74286102 | 4 | 74286102 | 97464 | C | T | C | 0.87097 | T | 0.12903 | 6 | 0.19355 |
| 3711 | S4_74333476 | 4 | 74333476 | 47374 | A | T | T | 0.6129 | A | 0.3871 | 10 | 0.32258 |
| 3712 | S4_74745227 | 4 | 74745227 | 411751 | A | G | A | 0.8871 | G | 0.1129 | 7 | 0.22581 |
| 3713 | S4_74745407 | 4 | 74745407 | 180 | C | T | C | 0.93548 | T | 0.06452 | 4 | 0.12903 |
| 3714 | S4_74807689 | 4 | 74807689 | 62282 | G | A | G | 0.8871 | A | 0.1129 | 7 | 0.22581 |
| 3715 | S4_76123298 | 4 | 76123298 | 1315609 | C | T | T | 0.6129 | C | 0.3871 | 14 | 0.45161 |
| 3716 | S4_76123335 | 4 | 76123335 | 37 | G | A | G | 0.83871 | A | 0.16129 | 6 | 0.19355 |
| 3717 | S4_76509665 | 4 | 76509665 | 386330 | T | C | T | 0.80645 | C | 0.19355 | 8 | 0.25806 |
| 3718 | S4_76509738 | 4 | 76509738 | 73 | G | A | G | 0.90323 | A | 0.09677 | 6 | 0.19355 |
| 3719 | S4_76509815 | 4 | 76509815 | 77 | G | A | G | 0.90323 | A | 0.09677 | 6 | 0.19355 |
| 3720 | S4_77096600 | 4 | 77096600 | 586785 | T | C | T | 0.6129 | C | 0.3871 | 14 | 0.45161 |
| 3721 | S4_77096720 | 4 | 77096720 | 120 | C | T | C | 0.91935 | T | 0.08065 | 5 | 0.16129 |
| 3722 | S4_77327185 | 4 | 77327185 | 230465 | C | A | A | 0.62903 | C | 0.37097 | 9 | 0.29032 |
| 3723 | S4_77886218 | 4 | 77886218 | 559033 | A | C | A | 0.51613 | C | 0.48387 | 16 | 0.51613 |
| 3724 | S4_79196623 | 4 | 79196623 | 1310405 | G | A | G | 0.80645 | A | 0.19355 | 10 | 0.32258 |

| Site number | SNP name | Chromosome | Physical position | Physical distance from the previous SNP | Reference allele | Alternative allele | Major allele | Major allele frequency | Minor allele | Minor allele frequency | Number heterozygous | Heterozygosity |
|---|---|---|---|---|---|---|---|---|---|---|---|---|
| 3725 | S4_79646173 | 4 | 79646173 | 449550 | A | G | G | 0.83871 | A | 0.16129 | 10 | 0.32258 |
| 3726 | S4_79646179 | 4 | 79646179 | 6 | A | G | G | 0.83871 | A | 0.16129 | 10 | 0.32258 |
| 3727 | S4_79646224 | 4 | 79646224 | 45 | C | T | C | 0.8871 | T | 0.1129 | 7 | 0.22581 |
| 3728 | S4_80216547 | 4 | 80216547 | 570323 | T | C | T | 0.79032 | C | 0.20968 | 11 | 0.35484 |
| 3729 | S4_80216548 | 4 | 80216548 | 1 | G | A | G | 0.79032 | A | 0.20968 | 11 | 0.35484 |
| 3730 | S4_80663909 | 4 | 80663909 | 447361 | A | G | G | 0.69355 | A | 0.30645 | 13 | 0.41935 |
| 3731 | S4_80664005 | 4 | 80664005 | 96 | A | G | G | 0.69355 | A | 0.30645 | 13 | 0.41935 |
| 3732 | S4_80664022 | 4 | 80664022 | 17 | G | A | G | 0.93548 | A | 0.06452 | 4 | 0.12903 |
| 3733 | S4_80664038 | 4 | 80664038 | 16 | A | G | G | 0.69355 | A | 0.30645 | 13 | 0.41935 |
| 3734 | S4_80770072 | 4 | 80770072 | 106034 | G | A | G | 0.72581 | A | 0.27419 | 9 | 0.29032 |
| 3735 | S4_81001117 | 4 | 81001117 | 231045 | T | C | T | 0.93548 | C | 0.06452 | 4 | 0.12903 |
| 3736 | S4_81001123 | 4 | 81001123 | 6 | C | T | C | 0.93548 | T | 0.06452 | 4 | 0.12903 |
| 3737 | S4_81227091 | 4 | 81227091 | 225968 | G | C | C | 0.87097 | G | 0.12903 | 8 | 0.25806 |
| 3738 | S4_81227223 | 4 | 81227223 | 132 | C | T | C | 0.93548 | T | 0.06452 | 4 | 0.12903 |
| 3739 | S4_81412544 | 4 | 81412544 | 185321 | G | A | G | 0.95161 | A | 0.04839 | 3 | 0.09677 |
| 3740 | S4_81737378 | 4 | 81737378 | 324834 | C | T | C | 0.90323 | T | 0.09677 | 6 | 0.19355 |
| 3741 | S4_82445900 | 4 | 82445900 | 708522 | G | A | G | 0.64516 | A | 0.35484 | 14 | 0.45161 |
| 3742 | S4_82445946 | 4 | 82445946 | 46 | G | A | G | 0.90323 | A | 0.09677 | 4 | 0.12903 |
| 3743 | S4_82459576 | 4 | 82459576 | 13630 | G | A | G | 0.8871 | A | 0.1129 | 5 | 0.16129 |
| 3744 | S4_82459694 | 4 | 82459694 | 118 | T | A | T | 0.74194 | A | 0.25806 | 8 | 0.25806 |
| 3745 | S4_85462895 | 4 | 85462895 | 3003201 | C | T | C | 0.82258 | T | 0.17742 | 9 | 0.29032 |
| 3746 | S4_85462983 | 4 | 85462983 | 88 | G | T | G | 0.67742 | T | 0.32258 | 14 | 0.45161 |
| 3747 | S4_85585998 | 4 | 85585998 | 123015 | C | T | C | 0.74194 | T | 0.25806 | 14 | 0.45161 |
| 3748 | S4_86405830 | 4 | 86405830 | 819832 | G | A | G | 0.77419 | A | 0.22581 | 10 | 0.32258 |
| 3749 | S4_86405896 | 4 | 86405896 | 66 | T | A | T | 0.82258 | A | 0.17742 | 9 | 0.29032 |
| 3750 | S4_86684572 | 4 | 86684572 | 278676 | T | G | T | 0.83871 | G | 0.16129 | 8 | 0.25806 |
| 3751 | S4_87036951 | 4 | 87036951 | 352379 | C | T | C | 0.91935 | T | 0.08065 | 5 | 0.16129 |
| 3752 | S4_87309104 | 4 | 87309104 | 272153 | A | G | A | 0.91935 | G | 0.08065 | 3 | 0.09677 |
| 3753 | S4_87309221 | 4 | 87309221 | 117 | G | A | G | 0.69355 | A | 0.30645 | 17 | 0.54839 |
| 3754 | S4_87535161 | 4 | 87535161 | 225940 | T | C | C | 0.79032 | T | 0.20968 | 9 | 0.29032 |
| 3755 | S4_87535196 | 4 | 87535196 | 35 | T | C | C | 0.93548 | T | 0.06452 | 4 | 0.12903 |
| 3756 | S4_87535224 | 4 | 87535224 | 28 | C | T | C | 0.8871 | T | 0.1129 | 7 | 0.22581 |
| 3757 | S4_87535227 | 4 | 87535227 | 3 | G | A | G | 0.8871 | A | 0.1129 | 7 | 0.22581 |
| 3758 | S4_87535300 | 4 | 87535300 | 73 | C | T | T | 0.90323 | C | 0.09677 | 6 | 0.19355 |
| 3759 | S4_87535310 | 4 | 87535310 | 10 | A | G | G | 0.93548 | A | 0.06452 | 4 | 0.12903 |
| 3760 | S4_87535357 | 4 | 87535357 | 47 | C | T | C | 0.8871 | T | 0.1129 | 7 | 0.22581 |
| 3761 | S4_87535362 | 4 | 87535362 | 5 | G | A | G | 0.8871 | A | 0.1129 | 7 | 0.22581 |
| 3762 | S4_87639693 | 4 | 87639693 | 104331 | A | T | A | 0.8871 | T | 0.1129 | 7 | 0.22581 |
| 3763 | S4_87639794 | 4 | 87639794 | 101 | G | A | G | 0.83871 | A | 0.16129 | 8 | 0.25806 |
| 3764 | S4_87639855 | 4 | 87639855 | 61 | T | C | T | 0.8871 | C | 0.1129 | 7 | 0.22581 |
| 3765 | S4_87770561 | 4 | 87770561 | 130706 | C | T | C | 0.77419 | T | 0.22581 | 8 | 0.25806 |
| 3766 | S4_87770629 | 4 | 87770629 | 68 | T | G | T | 0.95161 | G | 0.04839 | 3 | 0.09677 |
| 3767 | S4_87907836 | 4 | 87907836 | 137207 | G | C | G | 0.90323 | C | 0.09677 | 6 | 0.19355 |
| 3768 | S4_87907893 | 4 | 87907893 | 57 | C | A | C | 0.90323 | A | 0.09677 | 6 | 0.19355 |
| 3769 | S4_87907937 | 4 | 87907937 | 44 | C | A | C | 0.90323 | A | 0.09677 | 6 | 0.19355 |
| 3770 | S4_87907977 | 4 | 87907977 | 40 | C | T | C | 0.90323 | T | 0.09677 | 6 | 0.19355 |
| 3771 | S4_87907998 | 4 | 87907998 | 21 | G | T | G | 0.90323 | T | 0.09677 | 6 | 0.19355 |
| 3772 | S4_87917331 | 4 | 87917331 | 9333 | T | A | T | 0.85484 | A | 0.14516 | 9 | 0.29032 |
| 3773 | S4_87917427 | 4 | 87917427 | 96 | C | T | C | 0.80645 | T | 0.19355 | 12 | 0.3871 |
| 3774 | S4_87917541 | 4 | 87917541 | 114 | G | A | G | 0.80645 | A | 0.19355 | 12 | 0.3871 |
| 3775 | S4_88365671 | 4 | 88365671 | 448130 | C | T | C | 0.75806 | T | 0.24194 | 11 | 0.35484 |
| 3776 | S4_88378334 | 4 | 88378334 | 12663 | G | A | G | 0.77419 | A | 0.22581 | 12 | 0.3871 |
| 3777 | S4_88400095 | 4 | 88400095 | 21761 | A | C | A | 0.79032 | C | 0.20968 | 9 | 0.29032 |
| 3778 | S4_88542353 | 4 | 88542353 | 142258 | A | G | A | 0.93548 | G | 0.06452 | 4 | 0.12903 |

| Site number | SNP name | Chromosome | Physical position | Physical distance from the previous SNP | Reference allele | Alternative allele | Major allele | Major allele frequency | Minor allele | Minor allele frequency | Number heterozygous | Heterozygosity |
|---|---|---|---|---|---|---|---|---|---|---|---|---|
| 3779 | S4_88542502 | 4 | 88542502 | 149 | G | A | G | 0.93548 | A | 0.06452 | 4 | 0.12903 |
| 3780 | S4_88542540 | 4 | 88542540 | 38 | G | A | G | 0.87097 | A | 0.12903 | 8 | 0.25806 |
| 3781 | S4_89625861 | 4 | 89625861 | 1083321 | T | C | T | 0.91935 | C | 0.08065 | 3 | 0.09677 |
| 3782 | S4_89672288 | 4 | 89672288 | 46427 | G | A | G | 0.95161 | A | 0.04839 | 3 | 0.09677 |
| 3783 | S4_90389236 | 4 | 90389236 | 716948 | T | C | T | 0.64516 | C | 0.35484 | 14 | 0.45161 |
| 3784 | S4_90479573 | 4 | 90479573 | 90337 | T | C | C | 0.77419 | T | 0.22581 | 12 | 0.3871 |
| 3785 | S4_90504082 | 4 | 90504082 | 24509 | T | G | G | 0.75806 | T | 0.24194 | 13 | 0.41935 |
| 3786 | S4_90504119 | 4 | 90504119 | 37 | G | A | G | 0.6129 | A | 0.3871 | 16 | 0.51613 |
| 3787 | S4_90504120 | 4 | 90504120 | 1 | C | G | G | 0.87097 | C | 0.12903 | 8 | 0.25806 |
| 3788 | S4_91695100 | 4 | 91695100 | 1190980 | G | A | G | 0.66129 | A | 0.33871 | 13 | 0.41935 |
| 3789 | S4_93089809 | 4 | 93089809 | 1394709 | C | T | C | 0.93548 | T | 0.06452 | 4 | 0.12903 |
| 3790 | S4_94028541 | 4 | 94028541 | 938732 | C | T | C | 0.91935 | T | 0.08065 | 5 | 0.16129 |
| 3791 | S4_94708060 | 4 | 94708060 | 679519 | C | T | C | 0.93548 | T | 0.06452 | 4 | 0.12903 |
| 3792 | S4_95060200 | 4 | 95060200 | 352140 | A | T | A | 0.95161 | T | 0.04839 | 3 | 0.09677 |
| 3793 | S4_95236211 | 4 | 95236211 | 176011 | A | G | A | 0.85484 | G | 0.14516 | 9 | 0.29032 |
| 3794 | S4_95236214 | 4 | 95236214 | 3 | A | G | A | 0.85484 | G | 0.14516 | 9 | 0.29032 |
| 3795 | S4_95236225 | 4 | 95236225 | 11 | T | G | T | 0.90323 | G | 0.09677 | 6 | 0.19355 |
| 3796 | S4_95395338 | 4 | 95395338 | 159113 | G | A | G | 0.6129 | A | 0.3871 | 12 | 0.3871 |
| 3797 | S4_95602008 | 4 | 95602008 | 206670 | C | T | C | 0.66129 | T | 0.33871 | 9 | 0.29032 |
| 3798 | S4_96010580 | 4 | 96010580 | 408572 | A | G | G | 0.64516 | A | 0.35484 | 12 | 0.3871 |
| 3799 | S4_96010723 | 4 | 96010723 | 143 | C | T | C | 0.91935 | T | 0.08065 | 3 | 0.09677 |
| 3800 | S4_96357183 | 4 | 96357183 | 346460 | G | A | G | 0.8871 | A | 0.1129 | 7 | 0.22581 |
| 3801 | S4_97507346 | 4 | 97507346 | 1150163 | G | A | G | 0.64516 | A | 0.35484 | 18 | 0.58065 |
| 3802 | S4_97865606 | 4 | 97865606 | 358260 | C | T | C | 0.95161 | T | 0.04839 | 1 | 0.03226 |
| 3803 | S4_97865642 | 4 | 97865642 | 36 | G | A | A | 0.53226 | G | 0.46774 | 15 | 0.48387 |
| 3804 | S4_97865643 | 4 | 97865643 | 1 | C | T | C | 0.91935 | T | 0.08065 | 5 | 0.16129 |
| 3805 | S4_97865752 | 4 | 97865752 | 109 | C | T | C | 0.95161 | T | 0.04839 | 1 | 0.03226 |
| 3806 | S4_98079027 | 4 | 98079027 | 213275 | G | A | G | 0.85484 | A | 0.14516 | 5 | 0.16129 |
| 3807 | S4_99259187 | 4 | 99259187 | 1180160 | G | C | G | 0.91935 | C | 0.08065 | 5 | 0.16129 |
| 3808 | S4_99259195 | 4 | 99259195 | 8 | T | C | T | 0.91935 | C | 0.08065 | 5 | 0.16129 |
| 3809 | S4_99259357 | 4 | 99259357 | 162 | A | G | A | 0.8871 | G | 0.1129 | 5 | 0.16129 |
| 3810 | S4_99259377 | 4 | 99259377 | 20 | T | C | T | 0.91935 | C | 0.08065 | 5 | 0.16129 |
| 3811 | S4_99483387 | 4 | 99483387 | 224010 | C | T | C | 0.62903 | T | 0.37097 | 15 | 0.48387 |
| 3812 | S4_99483485 | 4 | 99483485 | 98 | T | C | C | 0.66129 | T | 0.33871 | 9 | 0.29032 |
| 3813 | S4_99760679 | 4 | 99760679 | 277194 | C | T | C | 0.90323 | T | 0.09677 | 4 | 0.12903 |
| 3814 | S4_101234681 | 4 | 101234681 | 1474002 | A | C | A | 0.72581 | C | 0.27419 | 11 | 0.35484 |
| 3815 | S4_101234683 | 4 | 101234683 | 2 | G | A | G | 0.90323 | A | 0.09677 | 4 | 0.12903 |
| 3816 | S4_101328369 | 4 | 101328369 | 93686 | A | G | A | 0.91935 | G | 0.08065 | 5 | 0.16129 |
| 3817 | S4_101559053 | 4 | 101559053 | 230684 | C | G | C | 0.87097 | G | 0.12903 | 8 | 0.25806 |
| 3818 | S4_101658361 | 4 | 101658361 | 99308 | G | A | G | 0.93548 | A | 0.06452 | 2 | 0.06452 |
| 3819 | S4_101658561 | 4 | 101658561 | 200 | C | T | T | 0.87097 | C | 0.12903 | 4 | 0.12903 |
| 3820 | S4_101658591 | 4 | 101658591 | 30 | G | A | G | 0.70968 | A | 0.29032 | 12 | 0.3871 |
| 3821 | S4_101674453 | 4 | 101674453 | 15862 | A | G | G | 0.8871 | A | 0.1129 | 7 | 0.22581 |
| 3822 | S4_101674483 | 4 | 101674483 | 30 | A | G | A | 0.85484 | G | 0.14516 | 9 | 0.29032 |
| 3823 | S4_101674539 | 4 | 101674539 | 56 | C | T | C | 0.93548 | T | 0.06452 | 2 | 0.06452 |
| 3824 | S4_101674574 | 4 | 101674574 | 35 | G | A | A | 0.8871 | G | 0.1129 | 7 | 0.22581 |
| 3825 | S4_101947747 | 4 | 101947747 | 273173 | T | C | C | 0.85484 | T | 0.14516 | 7 | 0.22581 |
| 3826 | S4_101947758 | 4 | 101947758 | 11 | G | A | G | 0.67742 | A | 0.32258 | 12 | 0.3871 |
| 3827 | S4_101947764 | 4 | 101947764 | 6 | G | A | A | 0.59677 | G | 0.40323 | 11 | 0.35484 |
| 3828 | S4_101947838 | 4 | 101947838 | 74 | G | A | G | 0.77419 | A | 0.22581 | 8 | 0.25806 |
| 3829 | S4_101947865 | 4 | 101947865 | 27 | C | G | C | 0.72581 | G | 0.27419 | 9 | 0.29032 |
| 3830 | S4_101947907 | 4 | 101947907 | 42 | G | A | G | 0.83871 | A | 0.16129 | 6 | 0.19355 |
| 3831 | S4_102786161 | 4 | 102786161 | 838254 | A | C | A | 0.6129 | C | 0.3871 | 10 | 0.32258 |
| 3832 | S4_102786213 | 4 | 102786213 | 52 | G | C | C | 0.79032 | G | 0.20968 | 9 | 0.29032 |

| Site number | SNP name | Chromosome | Physical position | Physical distance from the previous SNP | Reference allele | Alternative allele | Major allele | Major allele frequency | Minor allele | Minor allele frequency | Number heterozygous | Heterozygosity |
|---|---|---|---|---|---|---|---|---|---|---|---|---|
| 3833 | S4_103942625 | 4 | 103942625 | 1156412 | C | T | C | 0.95161 | T | 0.04839 | 3 | 0.09677 |
| 3834 | S4_103942631 | 4 | 103942631 | 6 | C | T | C | 0.51613 | T | 0.48387 | 14 | 0.45161 |
| 3835 | S4_104399330 | 4 | 104399330 | 456699 | G | A | G | 0.74194 | A | 0.25806 | 8 | 0.25806 |
| 3836 | S4_104399537 | 4 | 104399537 | 207 | A | G | G | 0.79032 | A | 0.20968 | 11 | 0.35484 |
| 3837 | S4_104882400 | 4 | 104882400 | 482863 | A | T | A | 0.90323 | T | 0.09677 | 6 | 0.19355 |
| 3838 | S4_104882415 | 4 | 104882415 | 15 | C | T | C | 0.93548 | T | 0.06452 | 4 | 0.12903 |
| 3839 | S4_105136145 | 4 | 105136145 | 253730 | G | A | G | 0.90323 | A | 0.09677 | 6 | 0.19355 |
| 3840 | S4_105894685 | 4 | 105894685 | 758540 | T | C | T | 0.90323 | C | 0.09677 | 4 | 0.12903 |
| 3841 | S4_106123408 | 4 | 106123408 | 228723 | T | C | T | 0.95161 | C | 0.04839 | 3 | 0.09677 |
| 3842 | S4_106318823 | 4 | 106318823 | 195415 | C | A | A | 0.69355 | C | 0.30645 | 9 | 0.29032 |
| 3843 | S4_106447379 | 4 | 106447379 | 128556 | A | G | A | 0.93548 | G | 0.06452 | 4 | 0.12903 |
| 3844 | S4_106892977 | 4 | 106892977 | 445598 | G | A | A | 0.5 | G | 0.5 | 13 | 0.41935 |
| 3845 | S4_106893075 | 4 | 106893075 | 98 | T | C | T | 0.87097 | C | 0.12903 | 4 | 0.12903 |
| 3846 | S4_106893083 | 4 | 106893083 | 8 | T | A | T | 0.87097 | A | 0.12903 | 4 | 0.12903 |
| 3847 | S4_106893096 | 4 | 106893096 | 13 | A | G | A | 0.87097 | G | 0.12903 | 4 | 0.12903 |
| 3848 | S4_106899370 | 4 | 106899370 | 6274 | G | C | G | 0.93548 | C | 0.06452 | 2 | 0.06452 |
| 3849 | S4_107189356 | 4 | 107189356 | 289986 | G | A | G | 0.82258 | A | 0.17742 | 9 | 0.29032 |
| 3850 | S4_107189526 | 4 | 107189526 | 170 | C | T | C | 0.82258 | T | 0.17742 | 9 | 0.29032 |
| 3851 | S4_107248027 | 4 | 107248027 | 58501 | C | T | C | 0.90323 | T | 0.09677 | 4 | 0.12903 |
| 3852 | S4_107248188 | 4 | 107248188 | 161 | A | G | G | 0.87097 | A | 0.12903 | 4 | 0.12903 |
| 3853 | S4_107264372 | 4 | 107264372 | 16184 | A | G | A | 0.54839 | G | 0.45161 | 10 | 0.32258 |
| 3854 | S4_107857017 | 4 | 107857017 | 592645 | C | T | C | 0.91935 | T | 0.08065 | 5 | 0.16129 |
| 3855 | S4_107889935 | 4 | 107889935 | 32918 | T | A | T | 0.93548 | A | 0.06452 | 4 | 0.12903 |
| 3856 | S4_108111281 | 4 | 108111281 | 221346 | C | T | C | 0.80645 | T | 0.19355 | 6 | 0.19355 |
| 3857 | S4_108111322 | 4 | 108111322 | 41 | C | T | C | 0.64516 | T | 0.35484 | 12 | 0.3871 |
| 3858 | S4_108461459 | 4 | 108461459 | 350137 | G | A | G | 0.91935 | A | 0.08065 | 5 | 0.16129 |
| 3859 | S4_108588371 | 4 | 108588371 | 126912 | G | A | A | 0.51613 | G | 0.48387 | 18 | 0.58065 |
| 3860 | S4_108998184 | 4 | 108998184 | 409813 | G | A | A | 0.77419 | G | 0.22581 | 10 | 0.32258 |
| 3861 | S4_109166889 | 4 | 109166889 | 168705 | G | A | G | 0.93548 | A | 0.06452 | 4 | 0.12903 |
| 3862 | S4_109683176 | 4 | 109683176 | 516287 | A | T | T | 0.66129 | A | 0.33871 | 13 | 0.41935 |
| 3863 | S4_110576377 | 4 | 110576377 | 893201 | G | A | G | 0.87097 | A | 0.12903 | 6 | 0.19355 |
| 3864 | S4_110576382 | 4 | 110576382 | 5 | A | C | A | 0.91935 | C | 0.08065 | 5 | 0.16129 |
| 3865 | S4_110603389 | 4 | 110603389 | 27007 | A | G | G | 0.56452 | A | 0.43548 | 15 | 0.48387 |
| 3866 | S4_110877659 | 4 | 110877659 | 274270 | A | G | A | 0.6129 | G | 0.3871 | 10 | 0.32258 |
| 3867 | S4_111499281 | 4 | 111499281 | 621622 | A | T | T | 0.70968 | A | 0.29032 | 12 | 0.3871 |
| 3868 | S4_111744367 | 4 | 111744367 | 245086 | T | C | T | 0.91935 | C | 0.08065 | 5 | 0.16129 |
| 3869 | S4_111744388 | 4 | 111744388 | 21 | A | C | A | 0.91935 | C | 0.08065 | 5 | 0.16129 |
| 3870 | S4_111744392 | 4 | 111744392 | 4 | G | C | G | 0.93548 | C | 0.06452 | 4 | 0.12903 |
| 3871 | S4_111744463 | 4 | 111744463 | 71 | C | G | C | 0.91935 | G | 0.08065 | 5 | 0.16129 |
| 3872 | S4_111744549 | 4 | 111744549 | 86 | T | A | T | 0.79032 | A | 0.20968 | 13 | 0.41935 |
| 3873 | S4_111887372 | 4 | 111887372 | 142823 | G | A | G | 0.87097 | A | 0.12903 | 4 | 0.12903 |
| 3874 | S4_111887476 | 4 | 111887476 | 104 | C | G | C | 0.66129 | G | 0.33871 | 13 | 0.41935 |
| 3875 | S4_112047308 | 4 | 112047308 | 159832 | G | A | G | 0.83871 | A | 0.16129 | 8 | 0.25806 |
| 3876 | S4_112127978 | 4 | 112127978 | 80670 | G | A | G | 0.93548 | A | 0.06452 | 4 | 0.12903 |
| 3877 | S4_112128063 | 4 | 112128063 | 85 | T | C | C | 0.77419 | T | 0.22581 | 12 | 0.3871 |
| 3878 | S4_112128097 | 4 | 112128097 | 34 | T | C | T | 0.6129 | C | 0.3871 | 14 | 0.45161 |
| 3879 | S4_112128115 | 4 | 112128115 | 18 | G | A | G | 0.93548 | A | 0.06452 | 4 | 0.12903 |
| 3880 | S4_112164502 | 4 | 112164502 | 36387 | G | A | G | 0.79032 | A | 0.20968 | 11 | 0.35484 |
| 3881 | S4_112164671 | 4 | 112164671 | 169 | C | T | C | 0.91935 | T | 0.08065 | 5 | 0.16129 |
| 3882 | S4_113014678 | 4 | 113014678 | 850007 | C | T | C | 0.90323 | T | 0.09677 | 4 | 0.12903 |
| 3883 | S4_113014756 | 4 | 113014756 | 78 | T | C | C | 0.69355 | T | 0.30645 | 9 | 0.29032 |
| 3884 | S4_113026223 | 4 | 113026223 | 11467 | G | A | A | 0.5 | G | 0.5 | 13 | 0.41935 |
| 3885 | S4_113026458 | 4 | 113026458 | 235 | G | A | G | 0.80645 | A | 0.19355 | 6 | 0.19355 |
| 3886 | S4_113026462 | 4 | 113026462 | 4 | T | C | T | 0.83871 | C | 0.16129 | 6 | 0.19355 |

| Site number | SNP name | Chromosome | Physical position | Physical distance from the previous SNP | Reference allele | Alternative allele | Major allele | Major allele frequency | Minor allele | Minor allele frequency | Number heterozygous | Heterozygosity |
|---|---|---|---|---|---|---|---|---|---|---|---|---|
| 3887 | S4_113101412 | 4 | 113101412 | 74950 | G | A | G | 0.93548 | A | 0.06452 | 2 | 0.06452 |
| 3888 | S4_113101437 | 4 | 113101437 | 25 | A | G | A | 0.93548 | G | 0.06452 | 2 | 0.06452 |
| 3889 | S4_113101531 | 4 | 113101531 | 94 | C | T | C | 0.90323 | T | 0.09677 | 4 | 0.12903 |
| 3890 | S4_113101564 | 4 | 113101564 | 33 | A | G | A | 0.93548 | G | 0.06452 | 2 | 0.06452 |
| 3891 | S4_113101565 | 4 | 113101565 | 1 | T | A | T | 0.93548 | A | 0.06452 | 2 | 0.06452 |
| 3892 | S4_113101569 | 4 | 113101569 | 4 | G | A | G | 0.93548 | A | 0.06452 | 2 | 0.06452 |
| 3893 | S4_113101605 | 4 | 113101605 | 36 | T | C | T | 0.93548 | C | 0.06452 | 2 | 0.06452 |
| 3894 | S4_113305977 | 4 | 113305977 | 204372 | G | A | G | 0.79032 | A | 0.20968 | 9 | 0.29032 |
| 3895 | S4_114277304 | 4 | 114277304 | 971327 | T | C | C | 0.95161 | T | 0.04839 | 3 | 0.09677 |
| 3896 | S4_114360307 | 4 | 114360307 | 83003 | C | T | T | 0.87097 | C | 0.12903 | 6 | 0.19355 |
| 3897 | S4_114360348 | 4 | 114360348 | 41 | A | G | A | 0.8871 | G | 0.1129 | 5 | 0.16129 |
| 3898 | S4_114360360 | 4 | 114360360 | 12 | T | C | T | 0.8871 | C | 0.1129 | 5 | 0.16129 |
| 3899 | S4_114630853 | 4 | 114630853 | 270493 | G | A | G | 0.82258 | A | 0.17742 | 9 | 0.29032 |
| 3900 | S4_114631081 | 4 | 114631081 | 228 | C | A | C | 0.8871 | A | 0.1129 | 7 | 0.22581 |
| 3901 | S4_114905121 | 4 | 114905121 | 274040 | G | C | G | 0.66129 | C | 0.33871 | 15 | 0.48387 |
| 3902 | S4_114905232 | 4 | 114905232 | 111 | T | C | T | 0.79032 | C | 0.20968 | 11 | 0.35484 |
| 3903 | S4_114949560 | 4 | 114949560 | 44328 | G | T | G | 0.87097 | T | 0.12903 | 8 | 0.25806 |
| 3904 | S4_115014928 | 4 | 115014928 | 65368 | G | C | G | 0.87097 | C | 0.12903 | 8 | 0.25806 |
| 3905 | S4_115015035 | 4 | 115015035 | 107 | A | T | A | 0.87097 | T | 0.12903 | 6 | 0.19355 |
| 3906 | S4_115015047 | 4 | 115015047 | 12 | G | T | T | 0.91935 | G | 0.08065 | 5 | 0.16129 |
| 3907 | S4_115315788 | 4 | 115315788 | 300741 | C | T | C | 0.93548 | T | 0.06452 | 4 | 0.12903 |
| 3908 | S4_115641327 | 4 | 115641327 | 325539 | A | G | G | 0.53226 | A | 0.46774 | 17 | 0.54839 |
| 3909 | S4_115684050 | 4 | 115684050 | 42723 | G | A | G | 0.59677 | A | 0.40323 | 15 | 0.48387 |
| 3910 | S4_115684056 | 4 | 115684056 | 6 | G | A | G | 0.74194 | A | 0.25806 | 12 | 0.3871 |
| 3911 | S4_115684099 | 4 | 115684099 | 43 | A | G | G | 0.56452 | A | 0.43548 | 17 | 0.54839 |
| 3912 | S4_115684128 | 4 | 115684128 | 29 | G | T | G | 0.69355 | T | 0.30645 | 15 | 0.48387 |
| 3913 | S4_115813385 | 4 | 115813385 | 129257 | T | G | T | 0.8871 | G | 0.1129 | 7 | 0.22581 |
| 3914 | S4_116091209 | 4 | 116091209 | 277824 | G | A | G | 0.77419 | A | 0.22581 | 10 | 0.32258 |
| 3915 | S4_116854796 | 4 | 116854796 | 763587 | A | G | A | 0.6129 | G | 0.3871 | 12 | 0.3871 |
| 3916 | S4_117498689 | 4 | 117498689 | 643893 | G | A | G | 0.91935 | A | 0.08065 | 3 | 0.09677 |
| 3917 | S4_117498691 | 4 | 117498691 | 2 | C | T | C | 0.83871 | T | 0.16129 | 10 | 0.32258 |
| 3918 | S4_118416671 | 4 | 118416671 | 917980 | T | G | G | 0.91935 | T | 0.08065 | 5 | 0.16129 |
| 3919 | S4_118416759 | 4 | 118416759 | 88 | A | G | G | 0.95161 | A | 0.04839 | 3 | 0.09677 |
| 3920 | S4_118459116 | 4 | 118459116 | 42357 | T | C | T | 0.83871 | C | 0.16129 | 8 | 0.25806 |
| 3921 | S4_118756812 | 4 | 118756812 | 297696 | A | T | A | 0.90323 | T | 0.09677 | 6 | 0.19355 |
| 3922 | S4_118756890 | 4 | 118756890 | 78 | T | G | T | 0.90323 | G | 0.09677 | 6 | 0.19355 |
| 3923 | S4_118756919 | 4 | 118756919 | 29 | T | C | C | 0.90323 | T | 0.09677 | 6 | 0.19355 |
| 3924 | S4_118915477 | 4 | 118915477 | 158558 | T | C | T | 0.79032 | C | 0.20968 | 11 | 0.35484 |
| 3925 | S4_118915488 | 4 | 118915488 | 11 | G | T | G | 0.93548 | T | 0.06452 | 4 | 0.12903 |
| 3926 | S4_118915514 | 4 | 118915514 | 26 | C | G | C | 0.75806 | G | 0.24194 | 11 | 0.35484 |
| 3927 | S4_118915531 | 4 | 118915531 | 17 | G | A | G | 0.95161 | A | 0.04839 | 1 | 0.03226 |
| 3928 | S4_119161331 | 4 | 119161331 | 245800 | A | T | A | 0.95161 | T | 0.04839 | 3 | 0.09677 |
| 3929 | S4_119161363 | 4 | 119161363 | 32 | T | G | T | 0.79032 | G | 0.20968 | 11 | 0.35484 |
| 3930 | S4_119161373 | 4 | 119161373 | 10 | C | G | C | 0.91935 | G | 0.08065 | 3 | 0.09677 |
| 3931 | S4_119161387 | 4 | 119161387 | 14 | G | T | G | 0.91935 | T | 0.08065 | 3 | 0.09677 |
| 3932 | S4_119256305 | 4 | 119256305 | 94918 | G | A | G | 0.93548 | A | 0.06452 | 4 | 0.12903 |
| 3933 | S4_119967647 | 4 | 119967647 | 711342 | C | T | C | 0.87097 | T | 0.12903 | 6 | 0.19355 |
| 3934 | S4_120231291 | 4 | 120231291 | 263644 | C | T | C | 0.93548 | T | 0.06452 | 4 | 0.12903 |
| 3935 | S4_120231292 | 4 | 120231292 | 1 | G | A | A | 0.51613 | G | 0.48387 | 16 | 0.51613 |
| 3936 | S4_120231323 | 4 | 120231323 | 31 | C | T | T | 0.51613 | C | 0.48387 | 16 | 0.51613 |
| 3937 | S4_120231345 | 4 | 120231345 | 22 | C | T | C | 0.8871 | T | 0.1129 | 7 | 0.22581 |
| 3938 | S4_120231385 | 4 | 120231385 | 40 | A | G | A | 0.93548 | G | 0.06452 | 4 | 0.12903 |
| 3939 | S4_120231448 | 4 | 120231448 | 63 | A | G | G | 0.85484 | A | 0.14516 | 7 | 0.22581 |
| 3940 | S4_120643909 | 4 | 120643909 | 412461 | T | C | T | 0.53226 | C | 0.46774 | 17 | 0.54839 |

| Site number | SNP name | Chromosome | Physical position | Physical distance from the previous SNP | Reference allele | Alternative allele | Major allele | Major allele frequency | Minor allele | Minor allele frequency | Number heterozygous | Heterozygosity |
|---|---|---|---|---|---|---|---|---|---|---|---|---|
| 3941 | S4_120643969 | 4 | 120643969 | 60 | A | T | A | 0.93548 | T | 0.06452 | 2 | 0.06452 |
| 3942 | S4_120644141 | 4 | 120644141 | 172 | T | C | T | 0.53226 | C | 0.46774 | 17 | 0.54839 |
| 3943 | S5_88181 | 5 | 88181 | 0 | C | T | C | 0.90323 | T | 0.09677 | 6 | 0.19355 |
| 3944 | S5_395056 | 5 | 395056 | 306875 | A | T | A | 0.91935 | T | 0.08065 | 3 | 0.09677 |
| 3945 | S5_395199 | 5 | 395199 | 143 | G | A | G | 0.74194 | A | 0.25806 | 12 | 0.3871 |
| 3946 | S5_758804 | 5 | 758804 | 363605 | C | T | T | 0.75806 | C | 0.24194 | 15 | 0.48387 |
| 3947 | S5_758810 | 5 | 758810 | 6 | T | C | T | 0.8871 | C | 0.1129 | 7 | 0.22581 |
| 3948 | S5_758945 | 5 | 758945 | 135 | G | T | G | 0.90323 | T | 0.09677 | 6 | 0.19355 |
| 3949 | S5_758956 | 5 | 758956 | 11 | G | T | T | 0.80645 | G | 0.19355 | 12 | 0.3871 |
| 3950 | S5_758969 | 5 | 758969 | 13 | A | G | A | 0.8871 | G | 0.1129 | 5 | 0.16129 |
| 3951 | S5_758977 | 5 | 758977 | 8 | C | A | C | 0.8871 | A | 0.1129 | 5 | 0.16129 |
| 3952 | S5_758991 | 5 | 758991 | 14 | G | A | G | 0.67742 | A | 0.32258 | 14 | 0.45161 |
| 3953 | S5_758995 | 5 | 758995 | 4 | G | A | G | 0.91935 | A | 0.08065 | 5 | 0.16129 |
| 3954 | S5_1156031 | 5 | 1156031 | 397036 | G | T | G | 0.53226 | T | 0.46774 | 19 | 0.6129 |
| 3955 | S5_1337384 | 5 | 1337384 | 181353 | A | G | A | 0.80645 | G | 0.19355 | 10 | 0.32258 |
| 3956 | S5_1527592 | 5 | 1527592 | 190208 | G | C | G | 0.91935 | C | 0.08065 | 5 | 0.16129 |
| 3957 | S5_1527605 | 5 | 1527605 | 13 | T | G | T | 0.91935 | G | 0.08065 | 5 | 0.16129 |
| 3958 | S5_1527637 | 5 | 1527637 | 32 | G | A | G | 0.91935 | A | 0.08065 | 5 | 0.16129 |
| 3959 | S5_1527683 | 5 | 1527683 | 46 | G | A | G | 0.8871 | A | 0.1129 | 7 | 0.22581 |
| 3960 | S5_1527730 | 5 | 1527730 | 47 | G | A | G | 0.91935 | A | 0.08065 | 5 | 0.16129 |
| 3961 | S5_1527732 | 5 | 1527732 | 2 | C | T | C | 0.91935 | T | 0.08065 | 5 | 0.16129 |
| 3962 | S5_1527757 | 5 | 1527757 | 25 | C | T | C | 0.66129 | T | 0.33871 | 15 | 0.48387 |
| 3963 | S5_1675155 | 5 | 1675155 | 147398 | G | A | G | 0.93548 | A | 0.06452 | 4 | 0.12903 |
| 3964 | S5_2176791 | 5 | 2176791 | 501636 | G | A | G | 0.51613 | A | 0.48387 | 18 | 0.58065 |
| 3965 | S5_2176862 | 5 | 2176862 | 71 | C | T | C | 0.59677 | T | 0.40323 | 17 | 0.54839 |
| 3966 | S5_2203818 | 5 | 2203818 | 26956 | C | T | C | 0.93548 | T | 0.06452 | 4 | 0.12903 |
| 3967 | S5_2484128 | 5 | 2484128 | 280310 | C | T | C | 0.91935 | T | 0.08065 | 5 | 0.16129 |
| 3968 | S5_2484260 | 5 | 2484260 | 132 | A | T | A | 0.90323 | T | 0.09677 | 6 | 0.19355 |
| 3969 | S5_2736119 | 5 | 2736119 | 251859 | G | A | G | 0.72581 | A | 0.27419 | 11 | 0.35484 |
| 3970 | S5_2736124 | 5 | 2736124 | 5 | C | T | C | 0.74194 | T | 0.25806 | 10 | 0.32258 |
| 3971 | S5_2736157 | 5 | 2736157 | 33 | T | C | T | 0.83871 | C | 0.16129 | 10 | 0.32258 |
| 3972 | S5_2736159 | 5 | 2736159 | 2 | C | A | C | 0.95161 | A | 0.04839 | 3 | 0.09677 |
| 3973 | S5_2736160 | 5 | 2736160 | 1 | C | T | C | 0.93548 | T | 0.06452 | 4 | 0.12903 |
| 3974 | S5_2736167 | 5 | 2736167 | 7 | C | T | C | 0.80645 | T | 0.19355 | 10 | 0.32258 |
| 3975 | S5_2736278 | 5 | 2736278 | 111 | C | T | C | 0.93548 | T | 0.06452 | 4 | 0.12903 |
| 3976 | S5_3621007 | 5 | 3621007 | 884729 | A | G | A | 0.90323 | G | 0.09677 | 4 | 0.12903 |
| 3977 | S5_3621016 | 5 | 3621016 | 9 | A | G | A | 0.91935 | G | 0.08065 | 5 | 0.16129 |
| 3978 | S5_3899194 | 5 | 3899194 | 278178 | G | C | G | 0.82258 | C | 0.17742 | 9 | 0.29032 |
| 3979 | S5_3899280 | 5 | 3899280 | 86 | G | A | G | 0.59677 | A | 0.40323 | 19 | 0.6129 |
| 3980 | S5_3899321 | 5 | 3899321 | 41 | C | T | C | 0.59677 | T | 0.40323 | 19 | 0.6129 |
| 3981 | S5_3899328 | 5 | 3899328 | 7 | T | C | C | 0.59677 | T | 0.40323 | 21 | 0.67742 |
| 3982 | S5_4826855 | 5 | 4826855 | 927527 | A | G | A | 0.5 | G | 0.5 | 15 | 0.48387 |
| 3983 | S5_4827039 | 5 | 4827039 | 184 | C | G | C | 0.54839 | G | 0.45161 | 14 | 0.45161 |
| 3984 | S5_5197438 | 5 | 5197438 | 370399 | T | C | C | 0.75806 | T | 0.24194 | 15 | 0.48387 |
| 3985 | S5_5197625 | 5 | 5197625 | 187 | A | G | A | 0.70968 | G | 0.29032 | 14 | 0.45161 |
| 3986 | S5_5574394 | 5 | 5574394 | 376769 | A | G | G | 0.75806 | A | 0.24194 | 11 | 0.35484 |
| 3987 | S5_5574484 | 5 | 5574484 | 90 | A | G | G | 0.91935 | A | 0.08065 | 5 | 0.16129 |
| 3988 | S5_5574498 | 5 | 5574498 | 14 | C | T | C | 0.95161 | T | 0.04839 | 3 | 0.09677 |
| 3989 | S5_5574556 | 5 | 5574556 | 58 | T | A | A | 0.91935 | T | 0.08065 | 5 | 0.16129 |
| 3990 | S5_5574637 | 5 | 5574637 | 81 | T | C | T | 0.90323 | C | 0.09677 | 6 | 0.19355 |
| 3991 | S5_5837670 | 5 | 5837670 | 263033 | A | G | A | 0.90323 | G | 0.09677 | 6 | 0.19355 |
| 3992 | S5_5837801 | 5 | 5837801 | 131 | T | C | T | 0.72581 | C | 0.27419 | 15 | 0.48387 |
| 3993 | S5_5837802 | 5 | 5837802 | 1 | C | G | C | 0.72581 | G | 0.27419 | 15 | 0.48387 |
| 3994 | S5_5837817 | 5 | 5837817 | 15 | T | C | T | 0.64516 | C | 0.35484 | 16 | 0.51613 |

| Site number | SNP name | Chromosome | Physical position | Physical distance from the previous SNP | Reference allele | Alternative allele | Major allele | Major allele frequency | Minor allele | Minor allele frequency | Number heterozygous | Heterozygosity |
|---|---|---|---|---|---|---|---|---|---|---|---|---|
| 3995 | S5_5837838 | 5 | 5837838 | 21 | A | G | A | 0.93548 | G | 0.06452 | 4 | 0.12903 |
| 3996 | S5_6071482 | 5 | 6071482 | 233644 | A | G | A | 0.85484 | G | 0.14516 | 9 | 0.29032 |
| 3997 | S5_6280793 | 5 | 6280793 | 209311 | A | G | A | 0.93548 | G | 0.06452 | 4 | 0.12903 |
| 3998 | S5_6280860 | 5 | 6280860 | 67 | C | T | C | 0.93548 | T | 0.06452 | 4 | 0.12903 |
| 3999 | S5_6707194 | 5 | 6707194 | 426334 | C | T | C | 0.85484 | T | 0.14516 | 7 | 0.22581 |
| 4000 | S5_6707292 | 5 | 6707292 | 98 | T | C | T | 0.75806 | C | 0.24194 | 13 | 0.41935 |
| 4001 | S5_6707296 | 5 | 6707296 | 4 | C | T | C | 0.69355 | T | 0.30645 | 9 | 0.29032 |
| 4002 | S5_6781800 | 5 | 6781800 | 74504 | G | T | G | 0.80645 | T | 0.19355 | 10 | 0.32258 |
| 4003 | S5_6967352 | 5 | 6967352 | 185552 | T | C | T | 0.91935 | C | 0.08065 | 5 | 0.16129 |
| 4004 | S5_6967437 | 5 | 6967437 | 85 | T | C | T | 0.91935 | C | 0.08065 | 5 | 0.16129 |
| 4005 | S5_6967491 | 5 | 6967491 | 54 | G | A | G | 0.70968 | A | 0.29032 | 12 | 0.3871 |
| 4006 | S5_6967518 | 5 | 6967518 | 27 | T | C | T | 0.80645 | C | 0.19355 | 10 | 0.32258 |
| 4007 | S5_7379718 | 5 | 7379718 | 412200 | G | T | G | 0.54839 | T | 0.45161 | 18 | 0.58065 |
| 4008 | S5_7379784 | 5 | 7379784 | 66 | T | A | T | 0.91935 | A | 0.08065 | 5 | 0.16129 |
| 4009 | S5_7379822 | 5 | 7379822 | 38 | T | C | T | 0.51613 | C | 0.48387 | 18 | 0.58065 |
| 4010 | S5_7379842 | 5 | 7379842 | 20 | G | A | A | 0.51613 | G | 0.48387 | 16 | 0.51613 |
| 4011 | S5_7440891 | 5 | 7440891 | 61049 | C | T | C | 0.51613 | T | 0.48387 | 12 | 0.3871 |
| 4012 | S5_7441002 | 5 | 7441002 | 111 | A | G | A | 0.51613 | G | 0.48387 | 12 | 0.3871 |
| 4013 | S5_7441020 | 5 | 7441020 | 18 | G | T | T | 0.51613 | G | 0.48387 | 12 | 0.3871 |
| 4014 | S5_7537304 | 5 | 7537304 | 96284 | G | A | G | 0.85484 | A | 0.14516 | 9 | 0.29032 |
| 4015 | S5_8118379 | 5 | 8118379 | 581075 | T | C | T | 0.74194 | C | 0.25806 | 8 | 0.25806 |
| 4016 | S5_8118495 | 5 | 8118495 | 116 | T | A | T | 0.90323 | A | 0.09677 | 6 | 0.19355 |
| 4017 | S5_8229960 | 5 | 8229960 | 111465 | T | C | C | 0.59677 | T | 0.40323 | 15 | 0.48387 |
| 4018 | S5_8989444 | 5 | 8989444 | 759484 | C | G | C | 0.75806 | G | 0.24194 | 11 | 0.35484 |
| 4019 | S5_8989497 | 5 | 8989497 | 53 | A | G | G | 0.53226 | A | 0.46774 | 19 | 0.6129 |
| 4020 | S5_8989510 | 5 | 8989510 | 13 | G | A | G | 0.87097 | A | 0.12903 | 8 | 0.25806 |
| 4021 | S5_9016204 | 5 | 9016204 | 26694 | A | G | A | 0.87097 | G | 0.12903 | 8 | 0.25806 |
| 4022 | S5_9016391 | 5 | 9016391 | 187 | T | C | T | 0.90323 | C | 0.09677 | 6 | 0.19355 |
| 4023 | S5_9767949 | 5 | 9767949 | 751558 | T | G | T | 0.85484 | G | 0.14516 | 7 | 0.22581 |
| 4024 | S5_9767984 | 5 | 9767984 | 35 | A | G | A | 0.85484 | G | 0.14516 | 7 | 0.22581 |
| 4025 | S5_9767989 | 5 | 9767989 | 5 | C | T | C | 0.93548 | T | 0.06452 | 4 | 0.12903 |
| 4026 | S5_10260134 | 5 | 10260134 | 492145 | C | T | C | 0.8871 | T | 0.1129 | 7 | 0.22581 |
| 4027 | S5_10260143 | 5 | 10260143 | 9 | C | T | C | 0.8871 | T | 0.1129 | 7 | 0.22581 |
| 4028 | S5_10260144 | 5 | 10260144 | 1 | A | G | A | 0.8871 | G | 0.1129 | 7 | 0.22581 |
| 4029 | S5_10260215 | 5 | 10260215 | 71 | G | A | G | 0.8871 | A | 0.1129 | 7 | 0.22581 |
| 4030 | S5_10260301 | 5 | 10260301 | 86 | C | T | C | 0.8871 | T | 0.1129 | 7 | 0.22581 |
| 4031 | S5_10483644 | 5 | 10483644 | 223343 | C | T | C | 0.82258 | T | 0.17742 | 11 | 0.35484 |
| 4032 | S5_10541102 | 5 | 10541102 | 57458 | C | A | C | 0.54839 | A | 0.45161 | 12 | 0.3871 |
| 4033 | S5_10541119 | 5 | 10541119 | 17 | A | G | A | 0.67742 | G | 0.32258 | 16 | 0.51613 |
| 4034 | S5_10541143 | 5 | 10541143 | 24 | C | T | C | 0.80645 | T | 0.19355 | 10 | 0.32258 |
| 4035 | S5_10541227 | 5 | 10541227 | 84 | C | T | C | 0.58065 | T | 0.41935 | 18 | 0.58065 |
| 4036 | S5_10541268 | 5 | 10541268 | 41 | G | A | G | 0.91935 | A | 0.08065 | 5 | 0.16129 |
| 4037 | S5_10548748 | 5 | 10548748 | 7480 | C | T | C | 0.80645 | T | 0.19355 | 12 | 0.3871 |
| 4038 | S5_11189630 | 5 | 11189630 | 640882 | C | T | C | 0.93548 | T | 0.06452 | 4 | 0.12903 |
| 4039 | S5_11189700 | 5 | 11189700 | 70 | T | G | T | 0.74194 | G | 0.25806 | 12 | 0.3871 |
| 4040 | S5_11189796 | 5 | 11189796 | 96 | G | A | G | 0.8871 | A | 0.1129 | 5 | 0.16129 |
| 4041 | S5_11835247 | 5 | 11835247 | 645451 | G | A | G | 0.64516 | A | 0.35484 | 16 | 0.51613 |
| 4042 | S5_11835292 | 5 | 11835292 | 45 | G | A | G | 0.90323 | A | 0.09677 | 6 | 0.19355 |
| 4043 | S5_11835387 | 5 | 11835387 | 95 | T | G | T | 0.66129 | G | 0.33871 | 15 | 0.48387 |
| 4044 | S5_11835406 | 5 | 11835406 | 19 | T | C | T | 0.6129 | C | 0.3871 | 18 | 0.58065 |
| 4045 | S5_11835418 | 5 | 11835418 | 12 | C | T | C | 0.6129 | T | 0.3871 | 18 | 0.58065 |
| 4046 | S5_12073813 | 5 | 12073813 | 238395 | A | G | A | 0.75806 | G | 0.24194 | 11 | 0.35484 |
| 4047 | S5_12073968 | 5 | 12073968 | 155 | C | T | C | 0.91935 | T | 0.08065 | 3 | 0.09677 |
| 4048 | S5_12073995 | 5 | 12073995 | 27 | G | A | G | 0.85484 | A | 0.14516 | 5 | 0.16129 |

| Site number | SNP name | Chromosome | Physical position | Physical distance from the previous SNP | Reference allele | Alternative allele | Major allele | Major allele frequency | Minor allele | Minor allele frequency | Number heterozygous | Heterozygosity |
|---|---|---|---|---|---|---|---|---|---|---|---|---|
| 4049 | S5_12387615 | 5 | 12387615 | 313620 | C | T | T | 0.87097 | C | 0.12903 | 8 | 0.25806 |
| 4050 | S5_12598845 | 5 | 12598845 | 211230 | A | G | G | 0.95161 | A | 0.04839 | 3 | 0.09677 |
| 4051 | S5_12599046 | 5 | 12599046 | 201 | A | G | A | 0.79032 | G | 0.20968 | 9 | 0.29032 |
| 4052 | S5_12791904 | 5 | 12791904 | 192858 | A | T | A | 0.93548 | T | 0.06452 | 4 | 0.12903 |
| 4053 | S5_13387506 | 5 | 13387506 | 595602 | G | C | G | 0.8871 | C | 0.1129 | 7 | 0.22581 |
| 4054 | S5_13387549 | 5 | 13387549 | 43 | T | C | C | 0.6129 | T | 0.3871 | 18 | 0.58065 |
| 4055 | S5_13748920 | 5 | 13748920 | 361371 | C | T | T | 0.59677 | C | 0.40323 | 15 | 0.48387 |
| 4056 | S5_13805778 | 5 | 13805778 | 56858 | A | G | A | 0.5 | G | 0.5 | 13 | 0.41935 |
| 4057 | S5_14404620 | 5 | 14404620 | 598842 | T | G | T | 0.75806 | G | 0.24194 | 11 | 0.35484 |
| 4058 | S5_14770981 | 5 | 14770981 | 366361 | C | T | T | 0.58065 | C | 0.41935 | 16 | 0.51613 |
| 4059 | S5_15043179 | 5 | 15043179 | 272198 | A | G | A | 0.8871 | G | 0.1129 | 7 | 0.22581 |
| 4060 | S5_15043215 | 5 | 15043215 | 36 | G | A | G | 0.93548 | A | 0.06452 | 4 | 0.12903 |
| 4061 | S5_15091389 | 5 | 15091389 | 48174 | G | A | G | 0.82258 | A | 0.17742 | 9 | 0.29032 |
| 4062 | S5_15091398 | 5 | 15091398 | 9 | G | A | G | 0.80645 | A | 0.19355 | 10 | 0.32258 |
| 4063 | S5_15491509 | 5 | 15491509 | 400111 | A | G | A | 0.56452 | G | 0.43548 | 19 | 0.6129 |
| 4064 | S5_15832231 | 5 | 15832231 | 340722 | G | T | G | 0.58065 | T | 0.41935 | 14 | 0.45161 |
| 4065 | S5_15855509 | 5 | 15855509 | 23278 | C | T | C | 0.69355 | T | 0.30645 | 15 | 0.48387 |
| 4066 | S5_15855649 | 5 | 15855649 | 140 | C | A | C | 0.95161 | A | 0.04839 | 3 | 0.09677 |
| 4067 | S5_16989456 | 5 | 16989456 | 1133807 | A | C | A | 0.69355 | C | 0.30645 | 15 | 0.48387 |
| 4068 | S5_16989544 | 5 | 16989544 | 88 | G | A | G | 0.93548 | A | 0.06452 | 2 | 0.06452 |
| 4069 | S5_16989575 | 5 | 16989575 | 31 | T | C | T | 0.90323 | C | 0.09677 | 6 | 0.19355 |
| 4070 | S5_16989676 | 5 | 16989676 | 101 | G | A | G | 0.93548 | A | 0.06452 | 4 | 0.12903 |
| 4071 | S5_17090222 | 5 | 17090222 | 100546 | A | C | A | 0.58065 | C | 0.41935 | 16 | 0.51613 |
| 4072 | S5_17090394 | 5 | 17090394 | 172 | A | C | A | 0.54839 | C | 0.45161 | 16 | 0.51613 |
| 4073 | S5_17284085 | 5 | 17284085 | 193691 | T | C | T | 0.67742 | C | 0.32258 | 16 | 0.51613 |
| 4074 | S5_17284116 | 5 | 17284116 | 31 | T | G | T | 0.70968 | G | 0.29032 | 18 | 0.58065 |
| 4075 | S5_17856380 | 5 | 17856380 | 572264 | A | G | A | 0.95161 | G | 0.04839 | 3 | 0.09677 |
| 4076 | S5_17856402 | 5 | 17856402 | 22 | T | C | T | 0.72581 | C | 0.27419 | 13 | 0.41935 |
| 4077 | S5_17856544 | 5 | 17856544 | 142 | A | G | G | 0.91935 | A | 0.08065 | 5 | 0.16129 |
| 4078 | S5_17856550 | 5 | 17856550 | 6 | A | G | A | 0.75806 | G | 0.24194 | 11 | 0.35484 |
| 4079 | S5_17856576 | 5 | 17856576 | 26 | T | C | T | 0.72581 | C | 0.27419 | 13 | 0.41935 |
| 4080 | S5_17865660 | 5 | 17865660 | 9084 | C | A | C | 0.70968 | A | 0.29032 | 14 | 0.45161 |
| 4081 | S5_17865668 | 5 | 17865668 | 8 | T | A | T | 0.75806 | A | 0.24194 | 13 | 0.41935 |
| 4082 | S5_17865708 | 5 | 17865708 | 40 | G | T | G | 0.75806 | T | 0.24194 | 13 | 0.41935 |
| 4083 | S5_17865731 | 5 | 17865731 | 23 | T | G | T | 0.75806 | G | 0.24194 | 13 | 0.41935 |
| 4084 | S5_17865732 | 5 | 17865732 | 1 | A | G | A | 0.75806 | G | 0.24194 | 13 | 0.41935 |
| 4085 | S5_17865737 | 5 | 17865737 | 5 | G | A | G | 0.90323 | A | 0.09677 | 6 | 0.19355 |
| 4086 | S5_17865771 | 5 | 17865771 | 34 | G | A | A | 0.91935 | G | 0.08065 | 5 | 0.16129 |
| 4087 | S5_17865786 | 5 | 17865786 | 15 | G | A | G | 0.75806 | A | 0.24194 | 13 | 0.41935 |
| 4088 | S5_17865804 | 5 | 17865804 | 18 | C | A | C | 0.75806 | A | 0.24194 | 13 | 0.41935 |
| 4089 | S5_18354801 | 5 | 18354801 | 488997 | A | G | A | 0.64516 | G | 0.35484 | 14 | 0.45161 |
| 4090 | S5_18354821 | 5 | 18354821 | 20 | A | G | A | 0.64516 | G | 0.35484 | 14 | 0.45161 |
| 4091 | S5_18354940 | 5 | 18354940 | 119 | G | A | G | 0.64516 | A | 0.35484 | 14 | 0.45161 |
| 4092 | S5_18354953 | 5 | 18354953 | 13 | G | A | G | 0.64516 | A | 0.35484 | 14 | 0.45161 |
| 4093 | S5_18467256 | 5 | 18467256 | 112303 | T | C | T | 0.95161 | C | 0.04839 | 3 | 0.09677 |
| 4094 | S5_18537170 | 5 | 18537170 | 69914 | C | T | C | 0.82258 | T | 0.17742 | 11 | 0.35484 |
| 4095 | S5_19254728 | 5 | 19254728 | 717558 | T | C | T | 0.95161 | C | 0.04839 | 3 | 0.09677 |
| 4096 | S5_19254741 | 5 | 19254741 | 13 | A | C | A | 0.93548 | C | 0.06452 | 4 | 0.12903 |
| 4097 | S5_19254806 | 5 | 19254806 | 65 | C | G | G | 0.56452 | C | 0.43548 | 13 | 0.41935 |
| 4098 | S5_21713907 | 5 | 21713907 | 2459101 | A | G | A | 0.69355 | G | 0.30645 | 11 | 0.35484 |
| 4099 | S5_21714080 | 5 | 21714080 | 173 | C | T | C | 0.70968 | T | 0.29032 | 10 | 0.32258 |
| 4100 | S5_21786910 | 5 | 21786910 | 72830 | G | A | G | 0.87097 | A | 0.12903 | 8 | 0.25806 |
| 4101 | S5_22008815 | 5 | 22008815 | 221905 | C | T | C | 0.82258 | T | 0.17742 | 7 | 0.22581 |
| 4102 | S5_22008975 | 5 | 22008975 | 160 | C | T | C | 0.87097 | T | 0.12903 | 8 | 0.25806 |

| Site number | SNP name | Chromosome | Physical position | Physical distance from the previous SNP | Reference allele | Alternative allele | Major allele | Major allele frequency | Minor allele | Minor allele frequency | Number heterozygous | Heterozygosity |
|---|---|---|---|---|---|---|---|---|---|---|---|---|
| 4103 | S5_22205692 | 5 | 22205692 | 196717 | T | C | T | 0.72581 | C | 0.27419 | 15 | 0.48387 |
| 4104 | S5_22277234 | 5 | 22277234 | 71542 | T | C | T | 0.62903 | C | 0.37097 | 17 | 0.54839 |
| 4105 | S5_22277237 | 5 | 22277237 | 3 | G | T | G | 0.69355 | T | 0.30645 | 15 | 0.48387 |
| 4106 | S5_22279711 | 5 | 22279711 | 2474 | C | T | C | 0.80645 | T | 0.19355 | 12 | 0.3871 |
| 4107 | S5_22279757 | 5 | 22279757 | 46 | A | G | A | 0.69355 | G | 0.30645 | 13 | 0.41935 |
| 4108 | S5_22459910 | 5 | 22459910 | 180153 | C | T | C | 0.69355 | T | 0.30645 | 15 | 0.48387 |
| 4109 | S5_22460024 | 5 | 22460024 | 114 | C | G | C | 0.93548 | G | 0.06452 | 4 | 0.12903 |
| 4110 | S5_22850252 | 5 | 22850252 | 390228 | T | C | T | 0.77419 | C | 0.22581 | 10 | 0.32258 |
| 4111 | S5_22854078 | 5 | 22854078 | 3826 | T | C | C | 0.59677 | T | 0.40323 | 15 | 0.48387 |
| 4112 | S5_22910761 | 5 | 22910761 | 56683 | G | A | G | 0.59677 | A | 0.40323 | 15 | 0.48387 |
| 4113 | S5_23114711 | 5 | 23114711 | 203950 | A | G | A | 0.6129 | G | 0.3871 | 20 | 0.64516 |
| 4114 | S5_23114734 | 5 | 23114734 | 23 | T | C | T | 0.69355 | C | 0.30645 | 17 | 0.54839 |
| 4115 | S5_23171215 | 5 | 23171215 | 56481 | C | T | C | 0.93548 | T | 0.06452 | 4 | 0.12903 |
| 4116 | S5_23186793 | 5 | 23186793 | 15578 | C | T | C | 0.85484 | T | 0.14516 | 7 | 0.22581 |
| 4117 | S5_23186881 | 5 | 23186881 | 88 | C | T | C | 0.80645 | T | 0.19355 | 8 | 0.25806 |
| 4118 | S5_23605834 | 5 | 23605834 | 418953 | C | A | C | 0.80645 | A | 0.19355 | 10 | 0.32258 |
| 4119 | S5_23605846 | 5 | 23605846 | 12 | C | T | C | 0.95161 | T | 0.04839 | 3 | 0.09677 |
| 4120 | S5_23605892 | 5 | 23605892 | 46 | C | T | C | 0.90323 | T | 0.09677 | 6 | 0.19355 |
| 4121 | S5_23605897 | 5 | 23605897 | 5 | C | T | C | 0.93548 | T | 0.06452 | 4 | 0.12903 |
| 4122 | S5_23782184 | 5 | 23782184 | 176287 | A | G | A | 0.56452 | G | 0.43548 | 17 | 0.54839 |
| 4123 | S5_23782341 | 5 | 23782341 | 157 | T | C | T | 0.95161 | C | 0.04839 | 3 | 0.09677 |
| 4124 | S5_24249119 | 5 | 24249119 | 466778 | A | G | G | 0.66129 | A | 0.33871 | 13 | 0.41935 |
| 4125 | S5_24285670 | 5 | 24285670 | 36551 | T | G | T | 0.83871 | G | 0.16129 | 10 | 0.32258 |
| 4126 | S5_24285703 | 5 | 24285703 | 33 | G | A | G | 0.83871 | A | 0.16129 | 10 | 0.32258 |
| 4127 | S5_24390835 | 5 | 24390835 | 105132 | G | A | G | 0.82258 | A | 0.17742 | 9 | 0.29032 |
| 4128 | S5_24390838 | 5 | 24390838 | 3 | G | A | G | 0.8871 | A | 0.1129 | 7 | 0.22581 |
| 4129 | S5_24390922 | 5 | 24390922 | 84 | G | T | T | 0.62903 | G | 0.37097 | 19 | 0.6129 |
| 4130 | S5_24586716 | 5 | 24586716 | 195794 | C | T | C | 0.80645 | T | 0.19355 | 10 | 0.32258 |
| 4131 | S5_24615706 | 5 | 24615706 | 28990 | C | T | C | 0.93548 | T | 0.06452 | 4 | 0.12903 |
| 4132 | S5_24791703 | 5 | 24791703 | 175997 | G | A | G | 0.75806 | A | 0.24194 | 13 | 0.41935 |
| 4133 | S5_25181972 | 5 | 25181972 | 390269 | T | C | T | 0.75806 | C | 0.24194 | 11 | 0.35484 |
| 4134 | S5_25181981 | 5 | 25181981 | 9 | G | A | G | 0.69355 | A | 0.30645 | 17 | 0.54839 |
| 4135 | S5_25182032 | 5 | 25182032 | 51 | G | A | G | 0.87097 | A | 0.12903 | 8 | 0.25806 |
| 4136 | S5_25306004 | 5 | 25306004 | 123972 | C | G | C | 0.5 | G | 0.5 | 15 | 0.48387 |
| 4137 | S5_25306043 | 5 | 25306043 | 39 | T | C | T | 0.87097 | C | 0.12903 | 8 | 0.25806 |
| 4138 | S5_25306071 | 5 | 25306071 | 28 | T | C | T | 0.87097 | C | 0.12903 | 8 | 0.25806 |
| 4139 | S5_25306074 | 5 | 25306074 | 3 | A | G | A | 0.87097 | G | 0.12903 | 8 | 0.25806 |
| 4140 | S5_25354744 | 5 | 25354744 | 48670 | G | A | G | 0.69355 | A | 0.30645 | 11 | 0.35484 |
| 4141 | S5_25623197 | 5 | 25623197 | 268453 | T | C | C | 0.56452 | T | 0.43548 | 17 | 0.54839 |
| 4142 | S5_26126783 | 5 | 26126783 | 503586 | G | A | A | 0.87097 | G | 0.12903 | 8 | 0.25806 |
| 4143 | S5_26659338 | 5 | 26659338 | 532555 | T | C | T | 0.82258 | C | 0.17742 | 7 | 0.22581 |
| 4144 | S5_26740644 | 5 | 26740644 | 81306 | A | G | G | 0.77419 | A | 0.22581 | 8 | 0.25806 |
| 4145 | S5_26950855 | 5 | 26950855 | 210211 | T | C | T | 0.75806 | C | 0.24194 | 11 | 0.35484 |
| 4146 | S5_26994117 | 5 | 26994117 | 43262 | G | C | G | 0.87097 | C | 0.12903 | 8 | 0.25806 |
| 4147 | S5_26994159 | 5 | 26994159 | 42 | G | A | G | 0.59677 | A | 0.40323 | 17 | 0.54839 |
| 4148 | S5_26994184 | 5 | 26994184 | 25 | A | C | A | 0.59677 | C | 0.40323 | 17 | 0.54839 |
| 4149 | S5_26994198 | 5 | 26994198 | 14 | T | A | A | 0.56452 | T | 0.43548 | 17 | 0.54839 |
| 4150 | S5_27136872 | 5 | 27136872 | 142674 | C | T | C | 0.83871 | T | 0.16129 | 10 | 0.32258 |
| 4151 | S5_27136882 | 5 | 27136882 | 10 | T | C | T | 0.83871 | C | 0.16129 | 10 | 0.32258 |
| 4152 | S5_27136909 | 5 | 27136909 | 27 | C | G | C | 0.54839 | G | 0.45161 | 14 | 0.45161 |
| 4153 | S5_27136912 | 5 | 27136912 | 3 | G | T | G | 0.54839 | T | 0.45161 | 14 | 0.45161 |
| 4154 | S5_27136933 | 5 | 27136933 | 21 | A | G | A | 0.54839 | G | 0.45161 | 14 | 0.45161 |
| 4155 | S5_27137022 | 5 | 27137022 | 89 | C | T | C | 0.95161 | T | 0.04839 | 3 | 0.09677 |
| 4156 | S5_27173622 | 5 | 27173622 | 36600 | G | C | C | 0.80645 | G | 0.19355 | 12 | 0.3871 |

| Site number | SNP name | Chromosome | Physical position | Physical distance from the previous SNP | Reference allele | Alternative allele | Major allele | Major allele frequency | Minor allele | Minor allele frequency | Number heterozygous | Heterozygosity |
|---|---|---|---|---|---|---|---|---|---|---|---|---|
| 4157 | S5_27173644 | 5 | 27173644 | 22 | G | A | A | 0.80645 | G | 0.19355 | 12 | 0.3871 |
| 4158 | S5_27173733 | 5 | 27173733 | 89 | C | A | C | 0.80645 | A | 0.19355 | 12 | 0.3871 |
| 4159 | S5_27173734 | 5 | 27173734 | 1 | G | A | G | 0.95161 | A | 0.04839 | 3 | 0.09677 |
| 4160 | S5_27173770 | 5 | 27173770 | 36 | G | C | C | 0.80645 | G | 0.19355 | 12 | 0.3871 |
| 4161 | S5_27507093 | 5 | 27507093 | 333323 | T | C | T | 0.93548 | C | 0.06452 | 2 | 0.06452 |
| 4162 | S5_27507103 | 5 | 27507103 | 10 | T | A | T | 0.93548 | A | 0.06452 | 2 | 0.06452 |
| 4163 | S5_27507119 | 5 | 27507119 | 16 | C | A | A | 0.53226 | C | 0.46774 | 17 | 0.54839 |
| 4164 | S5_27593880 | 5 | 27593880 | 86761 | C | T | C | 0.72581 | T | 0.27419 | 11 | 0.35484 |
| 4165 | S5_27602079 | 5 | 27602079 | 8199 | C | T | C | 0.93548 | T | 0.06452 | 2 | 0.06452 |
| 4166 | S5_27602204 | 5 | 27602204 | 125 | T | C | C | 0.79032 | T | 0.20968 | 13 | 0.41935 |
| 4167 | S5_28655775 | 5 | 28655775 | 1053571 | G | A | G | 0.87097 | A | 0.12903 | 8 | 0.25806 |
| 4168 | S5_28655818 | 5 | 28655818 | 43 | G | A | G | 0.75806 | A | 0.24194 | 15 | 0.48387 |
| 4169 | S5_28745768 | 5 | 28745768 | 89950 | G | A | G | 0.67742 | A | 0.32258 | 14 | 0.45161 |
| 4170 | S5_28864228 | 5 | 28864228 | 118460 | A | G | G | 0.8871 | A | 0.1129 | 5 | 0.16129 |
| 4171 | S5_28944795 | 5 | 28944795 | 80567 | T | C | T | 0.66129 | C | 0.33871 | 17 | 0.54839 |
| 4172 | S5_29149531 | 5 | 29149531 | 204736 | A | G | A | 0.95161 | G | 0.04839 | 3 | 0.09677 |
| 4173 | S5_29149543 | 5 | 29149543 | 12 | A | G | G | 0.79032 | A | 0.20968 | 11 | 0.35484 |
| 4174 | S5_29149606 | 5 | 29149606 | 63 | A | G | A | 0.66129 | G | 0.33871 | 17 | 0.54839 |
| 4175 | S5_30255704 | 5 | 30255704 | 1106098 | T | G | T | 0.93548 | G | 0.06452 | 4 | 0.12903 |
| 4176 | S5_30272474 | 5 | 30272474 | 16770 | T | G | T | 0.93548 | G | 0.06452 | 4 | 0.12903 |
| 4177 | S5_30308618 | 5 | 30308618 | 36144 | A | G | G | 0.6129 | A | 0.3871 | 12 | 0.3871 |
| 4178 | S5_30308637 | 5 | 30308637 | 19 | A | G | A | 0.93548 | G | 0.06452 | 4 | 0.12903 |
| 4179 | S5_30612833 | 5 | 30612833 | 304196 | G | A | A | 0.67742 | G | 0.32258 | 18 | 0.58065 |
| 4180 | S5_31820501 | 5 | 31820501 | 1207668 | T | G | G | 0.93548 | T | 0.06452 | 4 | 0.12903 |
| 4181 | S5_32001277 | 5 | 32001277 | 180776 | A | G | A | 0.93548 | G | 0.06452 | 4 | 0.12903 |
| 4182 | S5_32001326 | 5 | 32001326 | 49 | T | C | T | 0.59677 | C | 0.40323 | 19 | 0.6129 |
| 4183 | S5_32426357 | 5 | 32426357 | 425031 | G | A | A | 0.56452 | G | 0.43548 | 15 | 0.48387 |
| 4184 | S5_32426425 | 5 | 32426425 | 68 | A | G | G | 0.56452 | A | 0.43548 | 15 | 0.48387 |
| 4185 | S5_32948568 | 5 | 32948568 | 522143 | A | G | A | 0.95161 | G | 0.04839 | 3 | 0.09677 |
| 4186 | S5_32948675 | 5 | 32948675 | 107 | G | C | G | 0.59677 | C | 0.40323 | 15 | 0.48387 |
| 4187 | S5_33391287 | 5 | 33391287 | 442612 | A | C | A | 0.93548 | C | 0.06452 | 4 | 0.12903 |
| 4188 | S5_34760653 | 5 | 34760653 | 1369366 | G | A | G | 0.95161 | A | 0.04839 | 3 | 0.09677 |
| 4189 | S5_34760698 | 5 | 34760698 | 45 | C | A | C | 0.95161 | A | 0.04839 | 3 | 0.09677 |
| 4190 | S5_34760719 | 5 | 34760719 | 21 | A | G | A | 0.95161 | G | 0.04839 | 3 | 0.09677 |
| 4191 | S5_34804804 | 5 | 34804804 | 44085 | T | C | T | 0.95161 | C | 0.04839 | 3 | 0.09677 |
| 4192 | S5_34804870 | 5 | 34804870 | 66 | A | G | A | 0.69355 | G | 0.30645 | 15 | 0.48387 |
| 4193 | S5_34804914 | 5 | 34804914 | 44 | G | A | G | 0.64516 | A | 0.35484 | 14 | 0.45161 |
| 4194 | S5_34804935 | 5 | 34804935 | 21 | A | C | A | 0.80645 | C | 0.19355 | 8 | 0.25806 |
| 4195 | S5_35267326 | 5 | 35267326 | 462391 | T | G | T | 0.95161 | G | 0.04839 | 3 | 0.09677 |
| 4196 | S5_35495490 | 5 | 35495490 | 228164 | C | T | C | 0.83871 | T | 0.16129 | 10 | 0.32258 |
| 4197 | S5_35495523 | 5 | 35495523 | 33 | C | G | C | 0.80645 | G | 0.19355 | 10 | 0.32258 |
| 4198 | S5_35681572 | 5 | 35681572 | 186049 | A | G | A | 0.56452 | G | 0.43548 | 13 | 0.41935 |
| 4199 | S5_35681616 | 5 | 35681616 | 44 | T | C | T | 0.54839 | C | 0.45161 | 14 | 0.45161 |
| 4200 | S5_35681627 | 5 | 35681627 | 11 | C | T | C | 0.95161 | T | 0.04839 | 3 | 0.09677 |
| 4201 | S5_35681799 | 5 | 35681799 | 172 | G | A | G | 0.95161 | A | 0.04839 | 3 | 0.09677 |
| 4202 | S5_35714076 | 5 | 35714076 | 32277 | A | C | C | 0.54839 | A | 0.45161 | 14 | 0.45161 |
| 4203 | S5_35714081 | 5 | 35714081 | 5 | G | T | G | 0.72581 | T | 0.27419 | 15 | 0.48387 |
| 4204 | S5_35714110 | 5 | 35714110 | 29 | G | A | G | 0.72581 | A | 0.27419 | 15 | 0.48387 |
| 4205 | S5_35714125 | 5 | 35714125 | 15 | C | T | C | 0.51613 | T | 0.48387 | 14 | 0.45161 |
| 4206 | S5_35714126 | 5 | 35714126 | 1 | T | C | C | 0.75806 | T | 0.24194 | 13 | 0.41935 |
| 4207 | S5_35714169 | 5 | 35714169 | 43 | C | T | C | 0.5 | T | 0.5 | 15 | 0.48387 |
| 4208 | S5_35714231 | 5 | 35714231 | 62 | G | A | G | 0.72581 | A | 0.27419 | 15 | 0.48387 |
| 4209 | S5_35714252 | 5 | 35714252 | 21 | C | T | C | 0.8871 | T | 0.1129 | 5 | 0.16129 |
| 4210 | S5_36170153 | 5 | 36170153 | 455901 | G | A | G | 0.69355 | A | 0.30645 | 13 | 0.41935 |

| Site number | SNP name | Chromosome | Physical position | Physical distance from the previous SNP | Reference allele | Alternative allele | Major allele | Major allele frequency | Minor allele | Minor allele frequency | Number heterozygous | Heterozygosity |
|---|---|---|---|---|---|---|---|---|---|---|---|---|
| 4211 | S5_36170175 | 5 | 36170175 | 22 | A | G | A | 0.69355 | G | 0.30645 | 13 | 0.41935 |
| 4212 | S5_36392425 | 5 | 36392425 | 222250 | T | C | T | 0.91935 | C | 0.08065 | 5 | 0.16129 |
| 4213 | S5_36392452 | 5 | 36392452 | 27 | C | A | C | 0.85484 | A | 0.14516 | 9 | 0.29032 |
| 4214 | S5_36948730 | 5 | 36948730 | 556278 | C | T | C | 0.70968 | T | 0.29032 | 16 | 0.51613 |
| 4215 | S5_37139442 | 5 | 37139442 | 190712 | T | A | T | 0.54839 | A | 0.45161 | 12 | 0.3871 |
| 4216 | S5_37139559 | 5 | 37139559 | 117 | A | G | G | 0.91935 | A | 0.08065 | 5 | 0.16129 |
| 4217 | S5_38678888 | 5 | 38678888 | 1539329 | T | G | T | 0.90323 | G | 0.09677 | 6 | 0.19355 |
| 4218 | S5_38809240 | 5 | 38809240 | 130352 | G | A | G | 0.90323 | A | 0.09677 | 6 | 0.19355 |
| 4219 | S5_38809319 | 5 | 38809319 | 79 | G | C | G | 0.8871 | C | 0.1129 | 7 | 0.22581 |
| 4220 | S5_38811977 | 5 | 38811977 | 2658 | A | G | A | 0.95161 | G | 0.04839 | 3 | 0.09677 |
| 4221 | S5_39269807 | 5 | 39269807 | 457830 | T | C | T | 0.87097 | C | 0.12903 | 6 | 0.19355 |
| 4222 | S5_39269817 | 5 | 39269817 | 10 | G | A | A | 0.6129 | G | 0.3871 | 16 | 0.51613 |
| 4223 | S5_39269931 | 5 | 39269931 | 114 | C | G | G | 0.6129 | C | 0.3871 | 16 | 0.51613 |
| 4224 | S5_39270002 | 5 | 39270002 | 71 | T | C | C | 0.6129 | T | 0.3871 | 16 | 0.51613 |
| 4225 | S5_39270008 | 5 | 39270008 | 6 | G | A | A | 0.6129 | G | 0.3871 | 16 | 0.51613 |
| 4226 | S5_39270024 | 5 | 39270024 | 16 | C | T | C | 0.91935 | T | 0.08065 | 5 | 0.16129 |
| 4227 | S5_39734388 | 5 | 39734388 | 464364 | G | C | G | 0.82258 | C | 0.17742 | 7 | 0.22581 |
| 4228 | S5_39734454 | 5 | 39734454 | 66 | T | C | T | 0.82258 | C | 0.17742 | 7 | 0.22581 |
| 4229 | S5_39734565 | 5 | 39734565 | 111 | A | G | A | 0.82258 | G | 0.17742 | 7 | 0.22581 |
| 4230 | S5_39948564 | 5 | 39948564 | 213999 | G | A | G | 0.87097 | A | 0.12903 | 4 | 0.12903 |
| 4231 | S5_39948703 | 5 | 39948703 | 139 | G | T | G | 0.51613 | T | 0.48387 | 22 | 0.70968 |
| 4232 | S5_39948732 | 5 | 39948732 | 29 | T | C | C | 0.87097 | T | 0.12903 | 8 | 0.25806 |
| 4233 | S5_40075971 | 5 | 40075971 | 127239 | G | A | G | 0.62903 | A | 0.37097 | 17 | 0.54839 |
| 4234 | S5_40075998 | 5 | 40075998 | 27 | G | A | G | 0.75806 | A | 0.24194 | 13 | 0.41935 |
| 4235 | S5_40368194 | 5 | 40368194 | 292196 | G | A | G | 0.95161 | A | 0.04839 | 3 | 0.09677 |
| 4236 | S5_40368292 | 5 | 40368292 | 98 | A | G | G | 0.80645 | A | 0.19355 | 8 | 0.25806 |
| 4237 | S5_40368321 | 5 | 40368321 | 29 | C | T | C | 0.56452 | T | 0.43548 | 19 | 0.6129 |
| 4238 | S5_40368354 | 5 | 40368354 | 33 | C | T | C | 0.59677 | T | 0.40323 | 17 | 0.54839 |
| 4239 | S5_42313442 | 5 | 42313442 | 1945088 | C | T | C | 0.90323 | T | 0.09677 | 4 | 0.12903 |
| 4240 | S5_42313488 | 5 | 42313488 | 46 | C | T | C | 0.93548 | T | 0.06452 | 4 | 0.12903 |
| 4241 | S5_42492259 | 5 | 42492259 | 178771 | C | T | C | 0.8871 | T | 0.1129 | 7 | 0.22581 |
| 4242 | S5_43030412 | 5 | 43030412 | 538153 | C | G | C | 0.51613 | G | 0.48387 | 14 | 0.45161 |
| 4243 | S5_43030414 | 5 | 43030414 | 2 | A | G | A | 0.54839 | G | 0.45161 | 18 | 0.58065 |
| 4244 | S5_43030445 | 5 | 43030445 | 31 | T | C | T | 0.53226 | C | 0.46774 | 15 | 0.48387 |
| 4245 | S5_43340218 | 5 | 43340218 | 309773 | T | A | T | 0.93548 | A | 0.06452 | 4 | 0.12903 |
| 4246 | S5_43340377 | 5 | 43340377 | 159 | G | A | G | 0.93548 | A | 0.06452 | 4 | 0.12903 |
| 4247 | S5_43981965 | 5 | 43981965 | 641588 | A | G | A | 0.74194 | G | 0.25806 | 8 | 0.25806 |
| 4248 | S5_43982112 | 5 | 43982112 | 147 | G | A | A | 0.90323 | G | 0.09677 | 6 | 0.19355 |
| 4249 | S5_43982137 | 5 | 43982137 | 25 | A | G | G | 0.90323 | A | 0.09677 | 6 | 0.19355 |
| 4250 | S5_44316408 | 5 | 44316408 | 334271 | A | G | A | 0.62903 | G | 0.37097 | 13 | 0.41935 |
| 4251 | S5_44396791 | 5 | 44396791 | 80383 | C | T | C | 0.90323 | T | 0.09677 | 4 | 0.12903 |
| 4252 | S5_44396877 | 5 | 44396877 | 86 | A | T | A | 0.8871 | T | 0.1129 | 5 | 0.16129 |
| 4253 | S5_44752456 | 5 | 44752456 | 355579 | T | C | T | 0.59677 | C | 0.40323 | 13 | 0.41935 |
| 4254 | S5_44891774 | 5 | 44891774 | 139318 | G | A | G | 0.93548 | A | 0.06452 | 4 | 0.12903 |
| 4255 | S5_45492961 | 5 | 45492961 | 601187 | C | A | C | 0.90323 | A | 0.09677 | 6 | 0.19355 |
| 4256 | S5_45579211 | 5 | 45579211 | 86250 | G | A | G | 0.64516 | A | 0.35484 | 12 | 0.3871 |
| 4257 | S5_45579365 | 5 | 45579365 | 154 | G | A | G | 0.90323 | A | 0.09677 | 6 | 0.19355 |
| 4258 | S5_45944073 | 5 | 45944073 | 364708 | C | T | C | 0.62903 | T | 0.37097 | 11 | 0.35484 |
| 4259 | S5_45944104 | 5 | 45944104 | 31 | A | G | A | 0.8871 | G | 0.1129 | 7 | 0.22581 |
| 4260 | S5_46156698 | 5 | 46156698 | 212594 | C | A | C | 0.82258 | A | 0.17742 | 11 | 0.35484 |
| 4261 | S5_46292990 | 5 | 46292990 | 136292 | G | A | G | 0.90323 | A | 0.09677 | 6 | 0.19355 |
| 4262 | S5_46325892 | 5 | 46325892 | 32902 | C | G | G | 0.56452 | C | 0.43548 | 19 | 0.6129 |
| 4263 | S5_46325894 | 5 | 46325894 | 2 | A | G | A | 0.66129 | G | 0.33871 | 17 | 0.54839 |
| 4264 | S5_46325913 | 5 | 46325913 | 19 | T | C | T | 0.69355 | C | 0.30645 | 15 | 0.48387 |

| Site number | SNP name | Chromosome | Physical position | Physical distance from the previous SNP | Reference allele | Alternative allele | Major allele | Major allele frequency | Minor allele | Minor allele frequency | Number heterozygous | Heterozygosity |
|---|---|---|---|---|---|---|---|---|---|---|---|---|
| 4265 | S5_46325935 | 5 | 46325935 | 22 | C | T | C | 0.69355 | T | 0.30645 | 15 | 0.48387 |
| 4266 | S5_46325950 | 5 | 46325950 | 15 | T | C | T | 0.69355 | C | 0.30645 | 15 | 0.48387 |
| 4267 | S5_46325952 | 5 | 46325952 | 2 | C | T | C | 0.70968 | T | 0.29032 | 14 | 0.45161 |
| 4268 | S5_46326057 | 5 | 46326057 | 105 | T | C | C | 0.91935 | T | 0.08065 | 3 | 0.09677 |
| 4269 | S5_46440131 | 5 | 46440131 | 114074 | G | A | G | 0.74194 | A | 0.25806 | 10 | 0.32258 |
| 4270 | S5_46440188 | 5 | 46440188 | 57 | G | C | G | 0.87097 | C | 0.12903 | 8 | 0.25806 |
| 4271 | S5_46464242 | 5 | 46464242 | 24054 | A | G | A | 0.74194 | G | 0.25806 | 10 | 0.32258 |
| 4272 | S5_46528383 | 5 | 46528383 | 64141 | A | G | A | 0.75806 | G | 0.24194 | 13 | 0.41935 |
| 4273 | S5_46528397 | 5 | 46528397 | 14 | C | A | C | 0.8871 | A | 0.1129 | 7 | 0.22581 |
| 4274 | S5_46528472 | 5 | 46528472 | 75 | G | A | G | 0.87097 | A | 0.12903 | 6 | 0.19355 |
| 4275 | S5_46672625 | 5 | 46672625 | 144153 | C | T | C | 0.85484 | T | 0.14516 | 7 | 0.22581 |
| 4276 | S5_46672841 | 5 | 46672841 | 216 | T | G | T | 0.93548 | G | 0.06452 | 4 | 0.12903 |
| 4277 | S5_47769425 | 5 | 47769425 | 1096584 | A | G | A | 0.6129 | G | 0.3871 | 12 | 0.3871 |
| 4278 | S5_48062818 | 5 | 48062818 | 293393 | A | G | G | 0.75806 | A | 0.24194 | 9 | 0.29032 |
| 4279 | S5_48062846 | 5 | 48062846 | 28 | G | C | C | 0.74194 | G | 0.25806 | 10 | 0.32258 |
| 4280 | S5_48062870 | 5 | 48062870 | 24 | G | A | A | 0.74194 | G | 0.25806 | 10 | 0.32258 |
| 4281 | S5_48062875 | 5 | 48062875 | 5 | T | G | T | 0.93548 | G | 0.06452 | 4 | 0.12903 |
| 4282 | S5_48062988 | 5 | 48062988 | 113 | A | G | A | 0.74194 | G | 0.25806 | 8 | 0.25806 |
| 4283 | S5_48063868 | 5 | 48063868 | 880 | C | T | C | 0.90323 | T | 0.09677 | 6 | 0.19355 |
| 4284 | S5_48071355 | 5 | 48071355 | 7487 | T | C | C | 0.75806 | T | 0.24194 | 11 | 0.35484 |
| 4285 | S5_48071403 | 5 | 48071403 | 48 | C | T | T | 0.74194 | C | 0.25806 | 10 | 0.32258 |
| 4286 | S5_48195769 | 5 | 48195769 | 124366 | C | G | C | 0.85484 | G | 0.14516 | 9 | 0.29032 |
| 4287 | S5_48195893 | 5 | 48195893 | 124 | A | G | A | 0.67742 | G | 0.32258 | 12 | 0.3871 |
| 4288 | S5_48195925 | 5 | 48195925 | 32 | G | T | G | 0.85484 | T | 0.14516 | 9 | 0.29032 |
| 4289 | S5_48195952 | 5 | 48195952 | 27 | G | A | G | 0.82258 | A | 0.17742 | 7 | 0.22581 |
| 4290 | S5_48195968 | 5 | 48195968 | 16 | A | T | A | 0.85484 | T | 0.14516 | 9 | 0.29032 |
| 4291 | S5_48612154 | 5 | 48612154 | 416186 | T | G | T | 0.95161 | G | 0.04839 | 3 | 0.09677 |
| 4292 | S5_48612201 | 5 | 48612201 | 47 | G | C | G | 0.80645 | C | 0.19355 | 8 | 0.25806 |
| 4293 | S5_48612211 | 5 | 48612211 | 10 | A | C | C | 0.66129 | A | 0.33871 | 9 | 0.29032 |
| 4294 | S5_48612306 | 5 | 48612306 | 95 | G | C | G | 0.91935 | C | 0.08065 | 5 | 0.16129 |
| 4295 | S5_50971102 | 5 | 50971102 | 2358796 | C | G | C | 0.8871 | G | 0.1129 | 5 | 0.16129 |
| 4296 | S5_50971189 | 5 | 50971189 | 87 | A | T | A | 0.87097 | T | 0.12903 | 6 | 0.19355 |
| 4297 | S5_50971239 | 5 | 50971239 | 50 | T | G | T | 0.90323 | G | 0.09677 | 4 | 0.12903 |
| 4298 | S5_51024445 | 5 | 51024445 | 53206 | G | A | G | 0.66129 | A | 0.33871 | 11 | 0.35484 |
| 4299 | S5_51071647 | 5 | 51071647 | 47202 | T | C | T | 0.91935 | C | 0.08065 | 5 | 0.16129 |
| 4300 | S5_51071682 | 5 | 51071682 | 35 | A | G | A | 0.95161 | G | 0.04839 | 3 | 0.09677 |
| 4301 | S5_51071777 | 5 | 51071777 | 95 | A | G | A | 0.95161 | G | 0.04839 | 3 | 0.09677 |
| 4302 | S5_51424308 | 5 | 51424308 | 352531 | C | G | C | 0.75806 | G | 0.24194 | 15 | 0.48387 |
| 4303 | S5_51424486 | 5 | 51424486 | 178 | A | G | A | 0.90323 | G | 0.09677 | 6 | 0.19355 |
| 4304 | S5_52074067 | 5 | 52074067 | 649581 | G | C | G | 0.83871 | C | 0.16129 | 10 | 0.32258 |
| 4305 | S5_52074077 | 5 | 52074077 | 10 | T | C | T | 0.79032 | C | 0.20968 | 13 | 0.41935 |
| 4306 | S5_52915074 | 5 | 52915074 | 840997 | G | A | G | 0.53226 | A | 0.46774 | 13 | 0.41935 |
| 4307 | S5_53262580 | 5 | 53262580 | 347506 | T | C | C | 0.66129 | T | 0.33871 | 17 | 0.54839 |
| 4308 | S5_54251803 | 5 | 54251803 | 989223 | A | G | G | 0.59677 | A | 0.40323 | 17 | 0.54839 |
| 4309 | S5_55045915 | 5 | 55045915 | 794112 | G | A | G | 0.64516 | A | 0.35484 | 14 | 0.45161 |
| 4310 | S5_55390939 | 5 | 55390939 | 345024 | C | A | C | 0.54839 | A | 0.45161 | 12 | 0.3871 |
| 4311 | S5_55406886 | 5 | 55406886 | 15947 | T | C | T | 0.53226 | C | 0.46774 | 13 | 0.41935 |
| 4312 | S5_55942778 | 5 | 55942778 | 535892 | G | A | G | 0.90323 | A | 0.09677 | 6 | 0.19355 |
| 4313 | S5_55942892 | 5 | 55942892 | 114 | C | T | C | 0.93548 | T | 0.06452 | 4 | 0.12903 |
| 4314 | S5_56012904 | 5 | 56012904 | 70012 | T | C | T | 0.58065 | C | 0.41935 | 16 | 0.51613 |
| 4315 | S5_56012985 | 5 | 56012985 | 81 | C | T | C | 0.66129 | T | 0.33871 | 13 | 0.41935 |
| 4316 | S5_56246578 | 5 | 56246578 | 233593 | A | G | A | 0.87097 | G | 0.12903 | 6 | 0.19355 |
| 4317 | S5_56358688 | 5 | 56358688 | 112110 | C | T | C | 0.8871 | T | 0.1129 | 7 | 0.22581 |
| 4318 | S5_56615523 | 5 | 56615523 | 256835 | A | G | A | 0.91935 | G | 0.08065 | 5 | 0.16129 |

| Site number | SNP name | Chromosome | Physical position | Physical distance from the previous SNP | Reference allele | Alternative allele | Major allele | Major allele frequency | Minor allele | Minor allele frequency | Number heterozygous | Heterozygosity |
|---|---|---|---|---|---|---|---|---|---|---|---|---|
| 4319 | S5_56615672 | 5 | 56615672 | 149 | A | C | A | 0.83871 | C | 0.16129 | 8 | 0.25806 |
| 4320 | S5_56648460 | 5 | 56648460 | 32788 | C | T | C | 0.93548 | T | 0.06452 | 4 | 0.12903 |
| 4321 | S5_56648517 | 5 | 56648517 | 57 | C | T | C | 0.93548 | T | 0.06452 | 4 | 0.12903 |
| 4322 | S5_58179856 | 5 | 58179856 | 1531339 | A | C | A | 0.74194 | C | 0.25806 | 12 | 0.3871 |
| 4323 | S5_58643984 | 5 | 58643984 | 464128 | A | G | A | 0.95161 | G | 0.04839 | 3 | 0.09677 |
| 4324 | S5_58932086 | 5 | 58932086 | 288102 | G | A | G | 0.77419 | A | 0.22581 | 10 | 0.32258 |
| 4325 | S5_58976928 | 5 | 58976928 | 44842 | T | A | A | 0.87097 | T | 0.12903 | 6 | 0.19355 |
| 4326 | S5_59179626 | 5 | 59179626 | 202698 | A | C | C | 0.56452 | A | 0.43548 | 15 | 0.48387 |
| 4327 | S5_59179683 | 5 | 59179683 | 57 | A | G | A | 0.95161 | G | 0.04839 | 3 | 0.09677 |
| 4328 | S5_59557730 | 5 | 59557730 | 378047 | G | A | G | 0.54839 | A | 0.45161 | 12 | 0.3871 |
| 4329 | S5_59686721 | 5 | 59686721 | 128991 | G | A | G | 0.93548 | A | 0.06452 | 4 | 0.12903 |
| 4330 | S5_60105121 | 5 | 60105121 | 418400 | C | G | C | 0.79032 | G | 0.20968 | 9 | 0.29032 |
| 4331 | S5_60105311 | 5 | 60105311 | 190 | G | C | G | 0.70968 | C | 0.29032 | 12 | 0.3871 |
| 4332 | S5_60314398 | 5 | 60314398 | 209087 | A | G | A | 0.58065 | G | 0.41935 | 16 | 0.51613 |
| 4333 | S5_60314503 | 5 | 60314503 | 105 | C | T | C | 0.82258 | T | 0.17742 | 11 | 0.35484 |
| 4334 | S5_60326401 | 5 | 60326401 | 11898 | G | A | G | 0.93548 | A | 0.06452 | 4 | 0.12903 |
| 4335 | S5_60448945 | 5 | 60448945 | 122544 | C | T | C | 0.6129 | T | 0.3871 | 12 | 0.3871 |
| 4336 | S5_60449011 | 5 | 60449011 | 66 | C | T | C | 0.87097 | T | 0.12903 | 6 | 0.19355 |
| 4337 | S5_60469356 | 5 | 60469356 | 20345 | C | G | C | 0.59677 | G | 0.40323 | 13 | 0.41935 |
| 4338 | S5_60469446 | 5 | 60469446 | 90 | A | G | G | 0.59677 | A | 0.40323 | 15 | 0.48387 |
| 4339 | S5_60469525 | 5 | 60469525 | 79 | C | A | C | 0.59677 | A | 0.40323 | 13 | 0.41935 |
| 4340 | S5_60511517 | 5 | 60511517 | 41992 | C | T | C | 0.93548 | T | 0.06452 | 4 | 0.12903 |
| 4341 | S5_60511591 | 5 | 60511591 | 74 | T | C | T | 0.51613 | C | 0.48387 | 14 | 0.45161 |
| 4342 | S5_60560476 | 5 | 60560476 | 48885 | C | T | C | 0.74194 | T | 0.25806 | 10 | 0.32258 |
| 4343 | S5_60560477 | 5 | 60560477 | 1 | G | A | G | 0.93548 | A | 0.06452 | 4 | 0.12903 |
| 4344 | S5_60560506 | 5 | 60560506 | 29 | G | A | G | 0.74194 | A | 0.25806 | 10 | 0.32258 |
| 4345 | S5_60560625 | 5 | 60560625 | 119 | C | T | C | 0.82258 | T | 0.17742 | 9 | 0.29032 |
| 4346 | S5_61071339 | 5 | 61071339 | 510714 | A | G | A | 0.87097 | G | 0.12903 | 4 | 0.12903 |
| 4347 | S5_61071399 | 5 | 61071399 | 60 | G | C | C | 0.77419 | G | 0.22581 | 14 | 0.45161 |
| 4348 | S5_61251477 | 5 | 61251477 | 180078 | C | T | C | 0.95161 | T | 0.04839 | 3 | 0.09677 |
| 4349 | S5_61628606 | 5 | 61628606 | 377129 | T | C | T | 0.58065 | C | 0.41935 | 14 | 0.45161 |
| 4350 | S5_61935181 | 5 | 61935181 | 306575 | C | T | C | 0.95161 | T | 0.04839 | 3 | 0.09677 |
| 4351 | S5_61935379 | 5 | 61935379 | 198 | T | C | T | 0.85484 | C | 0.14516 | 7 | 0.22581 |
| 4352 | S5_61935400 | 5 | 61935400 | 21 | A | G | A | 0.91935 | G | 0.08065 | 5 | 0.16129 |
| 4353 | S5_63000047 | 5 | 63000047 | 1064647 | G | A | A | 0.74194 | G | 0.25806 | 12 | 0.3871 |
| 4354 | S5_63124927 | 5 | 63124927 | 124880 | C | T | C | 0.95161 | T | 0.04839 | 3 | 0.09677 |
| 4355 | S5_63125009 | 5 | 63125009 | 82 | A | G | A | 0.95161 | G | 0.04839 | 3 | 0.09677 |
| 4356 | S5_63156106 | 5 | 63156106 | 31097 | T | C | T | 0.95161 | C | 0.04839 | 3 | 0.09677 |
| 4357 | S5_63156107 | 5 | 63156107 | 1 | G | A | G | 0.85484 | A | 0.14516 | 7 | 0.22581 |
| 4358 | S5_63269492 | 5 | 63269492 | 113385 | C | A | A | 0.67742 | C | 0.32258 | 12 | 0.3871 |
| 4359 | S5_63269506 | 5 | 63269506 | 14 | C | T | T | 0.67742 | C | 0.32258 | 12 | 0.3871 |
| 4360 | S5_63269512 | 5 | 63269512 | 6 | A | G | G | 0.67742 | A | 0.32258 | 12 | 0.3871 |
| 4361 | S5_63269594 | 5 | 63269594 | 82 | G | A | A | 0.67742 | G | 0.32258 | 12 | 0.3871 |
| 4362 | S5_63269621 | 5 | 63269621 | 27 | G | A | A | 0.67742 | G | 0.32258 | 12 | 0.3871 |
| 4363 | S5_63269646 | 5 | 63269646 | 25 | C | T | T | 0.67742 | C | 0.32258 | 12 | 0.3871 |
| 4364 | S5_63291060 | 5 | 63291060 | 21414 | G | A | A | 0.85484 | G | 0.14516 | 5 | 0.16129 |
| 4365 | S5_63291170 | 5 | 63291170 | 110 | T | C | T | 0.8871 | C | 0.1129 | 5 | 0.16129 |
| 4366 | S5_63327550 | 5 | 63327550 | 36380 | C | T | T | 0.58065 | C | 0.41935 | 18 | 0.58065 |
| 4367 | S5_63327551 | 5 | 63327551 | 1 | A | G | G | 0.87097 | A | 0.12903 | 8 | 0.25806 |
| 4368 | S5_63327727 | 5 | 63327727 | 176 | G | T | T | 0.58065 | G | 0.41935 | 18 | 0.58065 |
| 4369 | S5_63327792 | 5 | 63327792 | 65 | A | C | A | 0.59677 | C | 0.40323 | 15 | 0.48387 |
| 4370 | S5_63355696 | 5 | 63355696 | 27904 | T | C | T | 0.64516 | C | 0.35484 | 16 | 0.51613 |
| 4371 | S5_63355782 | 5 | 63355782 | 86 | G | T | G | 0.90323 | T | 0.09677 | 6 | 0.19355 |
| 4372 | S5_63429242 | 5 | 63429242 | 73460 | C | T | C | 0.90323 | T | 0.09677 | 4 | 0.12903 |

| Site number | SNP name | Chromosome | Physical position | Physical distance from the previous SNP | Reference allele | Alternative allele | Major allele | Major allele frequency | Minor allele | Minor allele frequency | Number heterozygous | Heterozygosity |
|---|---|---|---|---|---|---|---|---|---|---|---|---|
| 4373 | S5_63429266 | 5 | 63429266 | 24 | A | G | A | 0.90323 | G | 0.09677 | 6 | 0.19355 |
| 4374 | S5_63495682 | 5 | 63495682 | 66416 | A | G | A | 0.93548 | G | 0.06452 | 4 | 0.12903 |
| 4375 | S5_63495701 | 5 | 63495701 | 19 | C | A | C | 0.82258 | A | 0.17742 | 9 | 0.29032 |
| 4376 | S5_63686744 | 5 | 63686744 | 191043 | C | T | T | 0.79032 | C | 0.20968 | 13 | 0.41935 |
| 4377 | S5_63832199 | 5 | 63832199 | 145455 | A | G | A | 0.77419 | G | 0.22581 | 10 | 0.32258 |
| 4378 | S5_64005692 | 5 | 64005692 | 173493 | C | T | C | 0.95161 | T | 0.04839 | 3 | 0.09677 |
| 4379 | S5_64005793 | 5 | 64005793 | 101 | G | T | G | 0.93548 | T | 0.06452 | 4 | 0.12903 |
| 4380 | S5_64321662 | 5 | 64321662 | 315869 | A | G | A | 0.85484 | G | 0.14516 | 7 | 0.22581 |
| 4381 | S5_64321809 | 5 | 64321809 | 147 | A | C | A | 0.59677 | C | 0.40323 | 17 | 0.54839 |
| 4382 | S5_64368193 | 5 | 64368193 | 46384 | A | G | G | 0.53226 | A | 0.46774 | 15 | 0.48387 |
| 4383 | S5_65260939 | 5 | 65260939 | 892746 | G | A | G | 0.90323 | A | 0.09677 | 6 | 0.19355 |
| 4384 | S5_65260982 | 5 | 65260982 | 43 | C | T | C | 0.95161 | T | 0.04839 | 3 | 0.09677 |
| 4385 | S5_65502634 | 5 | 65502634 | 241652 | C | A | C | 0.91935 | A | 0.08065 | 5 | 0.16129 |
| 4386 | S5_65502635 | 5 | 65502635 | 1 | C | T | C | 0.91935 | T | 0.08065 | 5 | 0.16129 |
| 4387 | S5_65502723 | 5 | 65502723 | 88 | G | A | G | 0.85484 | A | 0.14516 | 7 | 0.22581 |
| 4388 | S5_65917904 | 5 | 65917904 | 415181 | T | G | T | 0.82258 | G | 0.17742 | 11 | 0.35484 |
| 4389 | S5_65918019 | 5 | 65918019 | 115 | T | C | T | 0.82258 | C | 0.17742 | 11 | 0.35484 |
| 4390 | S5_65965496 | 5 | 65965496 | 47477 | C | T | C | 0.91935 | T | 0.08065 | 5 | 0.16129 |
| 4391 | S5_65965607 | 5 | 65965607 | 111 | G | A | A | 0.80645 | G | 0.19355 | 8 | 0.25806 |
| 4392 | S5_66506512 | 5 | 66506512 | 540905 | G | T | G | 0.91935 | T | 0.08065 | 5 | 0.16129 |
| 4393 | S5_66851967 | 5 | 66851967 | 345455 | A | G | A | 0.69355 | G | 0.30645 | 13 | 0.41935 |
| 4394 | S5_66852086 | 5 | 66852086 | 119 | G | A | G | 0.69355 | A | 0.30645 | 13 | 0.41935 |
| 4395 | S5_66901281 | 5 | 66901281 | 49195 | A | T | A | 0.5 | T | 0.5 | 15 | 0.48387 |
| 4396 | S5_66901390 | 5 | 66901390 | 109 | C | T | C | 0.5 | T | 0.5 | 15 | 0.48387 |
| 4397 | S5_66901417 | 5 | 66901417 | 27 | A | G | A | 0.56452 | G | 0.43548 | 15 | 0.48387 |
| 4398 | S5_66901428 | 5 | 66901428 | 11 | C | T | C | 0.51613 | T | 0.48387 | 14 | 0.45161 |
| 4399 | S5_66901513 | 5 | 66901513 | 85 | C | T | C | 0.5 | T | 0.5 | 15 | 0.48387 |
| 4400 | S5_66995438 | 5 | 66995438 | 93925 | T | C | T | 0.8871 | C | 0.1129 | 7 | 0.22581 |
| 4401 | S5_67030731 | 5 | 67030731 | 35293 | C | G | C | 0.8871 | G | 0.1129 | 7 | 0.22581 |
| 4402 | S5_67030855 | 5 | 67030855 | 124 | A | T | A | 0.87097 | T | 0.12903 | 8 | 0.25806 |
| 4403 | S5_67030857 | 5 | 67030857 | 2 | C | T | C | 0.79032 | T | 0.20968 | 11 | 0.35484 |
| 4404 | S5_67030860 | 5 | 67030860 | 3 | C | A | C | 0.95161 | A | 0.04839 | 3 | 0.09677 |
| 4405 | S5_67032569 | 5 | 67032569 | 1709 | A | C | A | 0.80645 | C | 0.19355 | 8 | 0.25806 |
| 4406 | S5_67189786 | 5 | 67189786 | 157217 | A | G | G | 0.67742 | A | 0.32258 | 10 | 0.32258 |
| 4407 | S5_67190006 | 5 | 67190006 | 220 | G | A | G | 0.91935 | A | 0.08065 | 3 | 0.09677 |
| 4408 | S5_67361056 | 5 | 67361056 | 171050 | G | A | G | 0.95161 | A | 0.04839 | 3 | 0.09677 |
| 4409 | S5_67589773 | 5 | 67589773 | 228717 | C | T | C | 0.93548 | T | 0.06452 | 4 | 0.12903 |
| 4410 | S5_67594187 | 5 | 67594187 | 4414 | T | C | T | 0.54839 | C | 0.45161 | 14 | 0.45161 |
| 4411 | S5_67782913 | 5 | 67782913 | 188726 | T | C | T | 0.77419 | C | 0.22581 | 12 | 0.3871 |
| 4412 | S5_67783061 | 5 | 67783061 | 148 | G | A | G | 0.77419 | A | 0.22581 | 12 | 0.3871 |
| 4413 | S5_67834953 | 5 | 67834953 | 51892 | T | G | T | 0.64516 | G | 0.35484 | 14 | 0.45161 |
| 4414 | S5_67835033 | 5 | 67835033 | 80 | T | A | A | 0.85484 | T | 0.14516 | 7 | 0.22581 |
| 4415 | S5_67835039 | 5 | 67835039 | 6 | A | G | A | 0.64516 | G | 0.35484 | 14 | 0.45161 |
| 4416 | S5_68107051 | 5 | 68107051 | 272012 | G | A | G | 0.8871 | A | 0.1129 | 7 | 0.22581 |
| 4417 | S5_68107062 | 5 | 68107062 | 11 | T | C | T | 0.8871 | C | 0.1129 | 7 | 0.22581 |
| 4418 | S5_68107160 | 5 | 68107160 | 98 | A | T | A | 0.62903 | T | 0.37097 | 17 | 0.54839 |
| 4419 | S5_69209364 | 5 | 69209364 | 1102204 | G | A | G | 0.8871 | A | 0.1129 | 7 | 0.22581 |
| 4420 | S5_69209416 | 5 | 69209416 | 52 | C | T | C | 0.95161 | T | 0.04839 | 3 | 0.09677 |
| 4421 | S5_69209458 | 5 | 69209458 | 42 | G | A | G | 0.8871 | A | 0.1129 | 7 | 0.22581 |
| 4422 | S5_69613376 | 5 | 69613376 | 403918 | G | T | G | 0.91935 | T | 0.08065 | 5 | 0.16129 |
| 4423 | S5_69613393 | 5 | 69613393 | 17 | A | C | C | 0.93548 | A | 0.06452 | 4 | 0.12903 |
| 4424 | S5_70277950 | 5 | 70277950 | 664557 | C | G | C | 0.75806 | G | 0.24194 | 13 | 0.41935 |
| 4425 | S5_70278109 | 5 | 70278109 | 159 | G | A | G | 0.87097 | A | 0.12903 | 6 | 0.19355 |
| 4426 | S5_70413594 | 5 | 70413594 | 135485 | A | T | T | 0.83871 | A | 0.16129 | 10 | 0.32258 |

| Site number | SNP name | Chromosome | Physical position | Physical distance from the previous SNP | Reference allele | Alternative allele | Major allele | Major allele frequency | Minor allele | Minor allele frequency | Number heterozygous | Heterozygosity |
|---|---|---|---|---|---|---|---|---|---|---|---|---|
| 4427 | S5_70413600 | 5 | 70413600 | 6 | T | C | C | 0.83871 | T | 0.16129 | 10 | 0.32258 |
| 4428 | S5_70413718 | 5 | 70413718 | 118 | A | G | A | 0.72581 | G | 0.27419 | 13 | 0.41935 |
| 4429 | S5_70413802 | 5 | 70413802 | 84 | T | C | T | 0.74194 | C | 0.25806 | 12 | 0.3871 |
| 4430 | S5_70781675 | 5 | 70781675 | 367873 | G | A | G | 0.53226 | A | 0.46774 | 21 | 0.67742 |
| 4431 | S5_70781699 | 5 | 70781699 | 24 | C | A | C | 0.90323 | A | 0.09677 | 6 | 0.19355 |
| 4432 | S5_70810913 | 5 | 70810913 | 29214 | G | T | G | 0.83871 | T | 0.16129 | 10 | 0.32258 |
| 4433 | S5_70811001 | 5 | 70811001 | 88 | C | T | T | 0.64516 | C | 0.35484 | 16 | 0.51613 |
| 4434 | S5_70905353 | 5 | 70905353 | 94352 | C | T | C | 0.95161 | T | 0.04839 | 3 | 0.09677 |
| 4435 | S5_70905456 | 5 | 70905456 | 103 | C | T | C | 0.93548 | T | 0.06452 | 4 | 0.12903 |
| 4436 | S5_70921256 | 5 | 70921256 | 15800 | T | C | T | 0.93548 | C | 0.06452 | 4 | 0.12903 |
| 4437 | S5_70921429 | 5 | 70921429 | 173 | A | C | A | 0.74194 | C | 0.25806 | 12 | 0.3871 |
| 4438 | S5_71115883 | 5 | 71115883 | 194454 | C | T | C | 0.87097 | T | 0.12903 | 8 | 0.25806 |
| 4439 | S5_71115895 | 5 | 71115895 | 12 | A | G | A | 0.85484 | G | 0.14516 | 9 | 0.29032 |
| 4440 | S5_71116000 | 5 | 71116000 | 105 | T | C | T | 0.56452 | C | 0.43548 | 11 | 0.35484 |
| 4441 | S5_71116026 | 5 | 71116026 | 26 | C | T | C | 0.87097 | T | 0.12903 | 8 | 0.25806 |
| 4442 | S5_71116032 | 5 | 71116032 | 6 | T | C | T | 0.93548 | C | 0.06452 | 4 | 0.12903 |
| 4443 | S5_71459158 | 5 | 71459158 | 343126 | A | T | A | 0.90323 | T | 0.09677 | 6 | 0.19355 |
| 4444 | S5_71459186 | 5 | 71459186 | 28 | G | A | G | 0.90323 | A | 0.09677 | 6 | 0.19355 |
| 4445 | S5_71944271 | 5 | 71944271 | 485085 | C | A | C | 0.93548 | A | 0.06452 | 4 | 0.12903 |
| 4446 | S5_71944461 | 5 | 71944461 | 190 | T | C | T | 0.83871 | C | 0.16129 | 10 | 0.32258 |
| 4447 | S5_72300448 | 5 | 72300448 | 355987 | C | T | C | 0.85484 | T | 0.14516 | 5 | 0.16129 |
| 4448 | S5_72300488 | 5 | 72300488 | 40 | A | G | A | 0.85484 | G | 0.14516 | 5 | 0.16129 |
| 4449 | S5_72300555 | 5 | 72300555 | 67 | T | C | T | 0.70968 | C | 0.29032 | 14 | 0.45161 |
| 4450 | S5_72690701 | 5 | 72690701 | 390146 | C | T | C | 0.85484 | T | 0.14516 | 9 | 0.29032 |
| 4451 | S5_72690732 | 5 | 72690732 | 31 | C | T | C | 0.82258 | T | 0.17742 | 11 | 0.35484 |
| 4452 | S5_72844936 | 5 | 72844936 | 154204 | T | C | C | 0.91935 | T | 0.08065 | 5 | 0.16129 |
| 4453 | S5_72844963 | 5 | 72844963 | 27 | G | C | C | 0.53226 | G | 0.46774 | 17 | 0.54839 |
| 4454 | S5_72844980 | 5 | 72844980 | 17 | T | C | T | 0.79032 | C | 0.20968 | 11 | 0.35484 |
| 4455 | S5_72909259 | 5 | 72909259 | 64279 | T | C | C | 0.93548 | T | 0.06452 | 4 | 0.12903 |
| 4456 | S5_72909419 | 5 | 72909419 | 160 | C | T | T | 0.93548 | C | 0.06452 | 4 | 0.12903 |
| 4457 | S5_73231106 | 5 | 73231106 | 321687 | A | G | A | 0.54839 | G | 0.45161 | 10 | 0.32258 |
| 4458 | S5_73231239 | 5 | 73231239 | 133 | G | A | G | 0.54839 | A | 0.45161 | 10 | 0.32258 |
| 4459 | S5_73231250 | 5 | 73231250 | 11 | A | C | A | 0.53226 | C | 0.46774 | 11 | 0.35484 |
| 4460 | S5_73347424 | 5 | 73347424 | 116174 | C | T | C | 0.82258 | T | 0.17742 | 7 | 0.22581 |
| 4461 | S5_73347432 | 5 | 73347432 | 8 | A | G | A | 0.91935 | G | 0.08065 | 5 | 0.16129 |
| 4462 | S5_73347487 | 5 | 73347487 | 55 | C | A | C | 0.83871 | A | 0.16129 | 8 | 0.25806 |
| 4463 | S5_73347522 | 5 | 73347522 | 35 | C | T | C | 0.83871 | T | 0.16129 | 8 | 0.25806 |
| 4464 | S5_73347529 | 5 | 73347529 | 7 | A | C | A | 0.75806 | C | 0.24194 | 13 | 0.41935 |
| 4465 | S5_73347530 | 5 | 73347530 | 1 | A | C | A | 0.83871 | C | 0.16129 | 8 | 0.25806 |
| 4466 | S5_73347543 | 5 | 73347543 | 13 | A | G | A | 0.83871 | G | 0.16129 | 8 | 0.25806 |
| 4467 | S5_73347658 | 5 | 73347658 | 115 | G | A | G | 0.83871 | A | 0.16129 | 8 | 0.25806 |
| 4468 | S5_73620670 | 5 | 73620670 | 273012 | C | T | C | 0.82258 | T | 0.17742 | 11 | 0.35484 |
| 4469 | S5_73709643 | 5 | 73709643 | 88973 | T | C | C | 0.67742 | T | 0.32258 | 14 | 0.45161 |
| 4470 | S5_74020827 | 5 | 74020827 | 311184 | C | A | C | 0.91935 | A | 0.08065 | 5 | 0.16129 |
| 4471 | S5_74220894 | 5 | 74220894 | 200067 | G | A | G | 0.74194 | A | 0.25806 | 14 | 0.45161 |
| 4472 | S5_74220912 | 5 | 74220912 | 18 | T | C | T | 0.80645 | C | 0.19355 | 10 | 0.32258 |
| 4473 | S5_74220959 | 5 | 74220959 | 47 | G | A | G | 0.93548 | A | 0.06452 | 4 | 0.12903 |
| 4474 | S5_74220963 | 5 | 74220963 | 4 | G | A | G | 0.80645 | A | 0.19355 | 10 | 0.32258 |
| 4475 | S5_74905793 | 5 | 74905793 | 684830 | T | C | T | 0.82258 | C | 0.17742 | 9 | 0.29032 |
| 4476 | S5_74905795 | 5 | 74905795 | 2 | C | T | C | 0.77419 | T | 0.22581 | 10 | 0.32258 |
| 4477 | S5_74905796 | 5 | 74905796 | 1 | C | T | C | 0.87097 | T | 0.12903 | 4 | 0.12903 |
| 4478 | S5_74905923 | 5 | 74905923 | 127 | C | T | C | 0.77419 | T | 0.22581 | 10 | 0.32258 |
| 4479 | S5_74906005 | 5 | 74906005 | 82 | C | T | C | 0.87097 | T | 0.12903 | 4 | 0.12903 |
| 4480 | S5_74906018 | 5 | 74906018 | 13 | A | G | A | 0.87097 | G | 0.12903 | 4 | 0.12903 |

| Site number | SNP name | Chromosome | Physical position | Physical distance from the previous SNP | Reference allele | Alternative allele | Major allele | Major allele frequency | Minor allele | Minor allele frequency | Number heterozygous | Heterozygosity |
|---|---|---|---|---|---|---|---|---|---|---|---|---|
| 4481 | S5_74911131 | 5 | 74911131 | 5113 | C | G | C | 0.91935 | G | 0.08065 | 5 | 0.16129 |
| 4482 | S5_74911221 | 5 | 74911221 | 90 | A | T | A | 0.95161 | T | 0.04839 | 3 | 0.09677 |
| 4483 | S5_74911280 | 5 | 74911280 | 59 | A | G | A | 0.95161 | G | 0.04839 | 3 | 0.09677 |
| 4484 | S5_75592027 | 5 | 75592027 | 680747 | G | T | G | 0.95161 | T | 0.04839 | 3 | 0.09677 |
| 4485 | S5_75592105 | 5 | 75592105 | 78 | C | T | C | 0.95161 | T | 0.04839 | 3 | 0.09677 |
| 4486 | S5_75940803 | 5 | 75940803 | 348698 | G | C | C | 0.93548 | G | 0.06452 | 4 | 0.12903 |
| 4487 | S5_75940916 | 5 | 75940916 | 113 | C | T | C | 0.72581 | T | 0.27419 | 13 | 0.41935 |
| 4488 | S5_76050332 | 5 | 76050332 | 109416 | G | T | G | 0.91935 | T | 0.08065 | 5 | 0.16129 |
| 4489 | S5_76101757 | 5 | 76101757 | 51425 | G | A | G | 0.95161 | A | 0.04839 | 3 | 0.09677 |
| 4490 | S5_76138268 | 5 | 76138268 | 36511 | G | A | G | 0.54839 | A | 0.45161 | 16 | 0.51613 |
| 4491 | S5_76138313 | 5 | 76138313 | 45 | T | G | G | 0.85484 | T | 0.14516 | 9 | 0.29032 |
| 4492 | S5_76351054 | 5 | 76351054 | 212741 | T | C | C | 0.79032 | T | 0.20968 | 13 | 0.41935 |
| 4493 | S5_76351128 | 5 | 76351128 | 74 | C | T | C | 0.95161 | T | 0.04839 | 3 | 0.09677 |
| 4494 | S5_77088121 | 5 | 77088121 | 736993 | A | G | A | 0.74194 | G | 0.25806 | 10 | 0.32258 |
| 4495 | S5_77088204 | 5 | 77088204 | 83 | A | G | A | 0.74194 | G | 0.25806 | 10 | 0.32258 |
| 4496 | S5_80055952 | 5 | 80055952 | 2967748 | G | A | G | 0.93548 | A | 0.06452 | 4 | 0.12903 |
| 4497 | S5_80055986 | 5 | 80055986 | 34 | A | G | A | 0.93548 | G | 0.06452 | 4 | 0.12903 |
| 4498 | S5_80382490 | 5 | 80382490 | 326504 | A | C | A | 0.70968 | C | 0.29032 | 16 | 0.51613 |
| 4499 | S5_80796582 | 5 | 80796582 | 414092 | T | C | T | 0.51613 | C | 0.48387 | 20 | 0.64516 |
| 4500 | S5_80796609 | 5 | 80796609 | 27 | G | C | G | 0.51613 | C | 0.48387 | 20 | 0.64516 |
| 4501 | S5_80796655 | 5 | 80796655 | 46 | T | C | T | 0.79032 | C | 0.20968 | 9 | 0.29032 |
| 4502 | S5_80864148 | 5 | 80864148 | 67493 | C | G | G | 0.95161 | C | 0.04839 | 3 | 0.09677 |
| 4503 | S5_80864201 | 5 | 80864201 | 53 | A | G | G | 0.95161 | A | 0.04839 | 3 | 0.09677 |
| 4504 | S5_80864225 | 5 | 80864225 | 24 | C | T | C | 0.95161 | T | 0.04839 | 3 | 0.09677 |
| 4505 | S5_80864255 | 5 | 80864255 | 30 | A | G | A | 0.70968 | G | 0.29032 | 12 | 0.3871 |
| 4506 | S5_80864264 | 5 | 80864264 | 9 | A | C | A | 0.83871 | C | 0.16129 | 6 | 0.19355 |
| 4507 | S5_80864308 | 5 | 80864308 | 44 | A | G | A | 0.93548 | G | 0.06452 | 4 | 0.12903 |
| 4508 | S5_80864324 | 5 | 80864324 | 16 | T | C | T | 0.93548 | C | 0.06452 | 4 | 0.12903 |
| 4509 | S5_81100929 | 5 | 81100929 | 236605 | C | T | T | 0.87097 | C | 0.12903 | 8 | 0.25806 |
| 4510 | S5_81100935 | 5 | 81100935 | 6 | G | T | T | 0.87097 | G | 0.12903 | 8 | 0.25806 |
| 4511 | S5_81339411 | 5 | 81339411 | 238476 | C | T | T | 0.62903 | C | 0.37097 | 13 | 0.41935 |
| 4512 | S5_81339575 | 5 | 81339575 | 164 | C | T | C | 0.93548 | T | 0.06452 | 4 | 0.12903 |
| 4513 | S5_81532870 | 5 | 81532870 | 193295 | G | A | A | 0.6129 | G | 0.3871 | 16 | 0.51613 |
| 4514 | S5_81785319 | 5 | 81785319 | 252449 | G | A | G | 0.93548 | A | 0.06452 | 4 | 0.12903 |
| 4515 | S5_81865152 | 5 | 81865152 | 79833 | A | T | A | 0.56452 | T | 0.43548 | 17 | 0.54839 |
| 4516 | S5_81865175 | 5 | 81865175 | 23 | T | C | C | 0.6129 | T | 0.3871 | 16 | 0.51613 |
| 4517 | S5_81865187 | 5 | 81865187 | 12 | C | G | C | 0.82258 | G | 0.17742 | 9 | 0.29032 |
| 4518 | S5_81865191 | 5 | 81865191 | 4 | T | A | T | 0.82258 | A | 0.17742 | 9 | 0.29032 |
| 4519 | S5_81946802 | 5 | 81946802 | 81611 | G | A | G | 0.83871 | A | 0.16129 | 8 | 0.25806 |
| 4520 | S5_81946980 | 5 | 81946980 | 178 | G | T | G | 0.54839 | T | 0.45161 | 18 | 0.58065 |
| 4521 | S5_82023821 | 5 | 82023821 | 76841 | C | T | C | 0.54839 | T | 0.45161 | 16 | 0.51613 |
| 4522 | S5_82158161 | 5 | 82158161 | 134340 | C | T | C | 0.77419 | T | 0.22581 | 14 | 0.45161 |
| 4523 | S5_82158331 | 5 | 82158331 | 170 | T | G | T | 0.75806 | G | 0.24194 | 15 | 0.48387 |
| 4524 | S5_82194213 | 5 | 82194213 | 35882 | G | A | G | 0.87097 | A | 0.12903 | 8 | 0.25806 |
| 4525 | S5_82194234 | 5 | 82194234 | 21 | C | T | C | 0.91935 | T | 0.08065 | 5 | 0.16129 |
| 4526 | S5_82194237 | 5 | 82194237 | 3 | A | G | G | 0.82258 | A | 0.17742 | 11 | 0.35484 |
| 4527 | S5_82194301 | 5 | 82194301 | 64 | T | C | T | 0.79032 | C | 0.20968 | 7 | 0.22581 |
| 4528 | S5_82194414 | 5 | 82194414 | 113 | C | T | C | 0.59677 | T | 0.40323 | 11 | 0.35484 |
| 4529 | S5_82232189 | 5 | 82232189 | 37775 | G | A | G | 0.66129 | A | 0.33871 | 15 | 0.48387 |
| 4530 | S5_82232285 | 5 | 82232285 | 96 | G | A | G | 0.91935 | A | 0.08065 | 5 | 0.16129 |
| 4531 | S5_82232292 | 5 | 82232292 | 7 | C | G | C | 0.80645 | G | 0.19355 | 10 | 0.32258 |
| 4532 | S5_82291166 | 5 | 82291166 | 58874 | C | T | C | 0.90323 | T | 0.09677 | 4 | 0.12903 |
| 4533 | S5_82291182 | 5 | 82291182 | 16 | C | T | C | 0.93548 | T | 0.06452 | 4 | 0.12903 |
| 4534 | S5_82291240 | 5 | 82291240 | 58 | A | G | A | 0.8871 | G | 0.1129 | 7 | 0.22581 |

| Site number | SNP name | Chromosome | Physical position | Physical distance from the previous SNP | Reference allele | Alternative allele | Major allele | Major allele frequency | Minor allele | Minor allele frequency | Number heterozygous | Heterozygosity |
|---|---|---|---|---|---|---|---|---|---|---|---|---|
| 4535 | S5_82291361 | 5 | 82291361 | 121 | A | G | A | 0.91935 | G | 0.08065 | 5 | 0.16129 |
| 4536 | S5_82291383 | 5 | 82291383 | 22 | T | C | T | 0.91935 | C | 0.08065 | 5 | 0.16129 |
| 4537 | S5_82745270 | 5 | 82745270 | 453887 | G | T | G | 0.91935 | T | 0.08065 | 5 | 0.16129 |
| 4538 | S5_82745419 | 5 | 82745419 | 149 | T | C | T | 0.83871 | C | 0.16129 | 8 | 0.25806 |
| 4539 | S5_82745441 | 5 | 82745441 | 22 | A | G | A | 0.51613 | G | 0.48387 | 16 | 0.51613 |
| 4540 | S5_82888721 | 5 | 82888721 | 143280 | C | T | C | 0.58065 | T | 0.41935 | 10 | 0.32258 |
| 4541 | S5_82888813 | 5 | 82888813 | 92 | A | G | A | 0.95161 | G | 0.04839 | 3 | 0.09677 |
| 4542 | S5_82897552 | 5 | 82897552 | 8739 | C | T | C | 0.93548 | T | 0.06452 | 4 | 0.12903 |
| 4543 | S5_82897655 | 5 | 82897655 | 103 | G | A | G | 0.79032 | A | 0.20968 | 11 | 0.35484 |
| 4544 | S5_82897682 | 5 | 82897682 | 27 | T | C | T | 0.85484 | C | 0.14516 | 9 | 0.29032 |
| 4545 | S5_82897721 | 5 | 82897721 | 39 | C | T | C | 0.91935 | T | 0.08065 | 3 | 0.09677 |
| 4546 | S5_82897730 | 5 | 82897730 | 9 | T | C | T | 0.72581 | C | 0.27419 | 13 | 0.41935 |
| 4547 | S5_83128393 | 5 | 83128393 | 230663 | G | A | G | 0.72581 | A | 0.27419 | 11 | 0.35484 |
| 4548 | S5_83128473 | 5 | 83128473 | 80 | G | A | A | 0.74194 | G | 0.25806 | 10 | 0.32258 |
| 4549 | S5_83654916 | 5 | 83654916 | 526443 | T | C | T | 0.51613 | C | 0.48387 | 12 | 0.3871 |
| 4550 | S5_84279277 | 5 | 84279277 | 624361 | T | G | T | 0.77419 | G | 0.22581 | 12 | 0.3871 |
| 4551 | S5_84294126 | 5 | 84294126 | 14849 | G | A | G | 0.90323 | A | 0.09677 | 4 | 0.12903 |
| 4552 | S5_84679759 | 5 | 84679759 | 385633 | T | A | T | 0.93548 | A | 0.06452 | 4 | 0.12903 |
| 4553 | S5_84679934 | 5 | 84679934 | 175 | T | C | C | 0.67742 | T | 0.32258 | 12 | 0.3871 |
| 4554 | S5_84864298 | 5 | 84864298 | 184364 | T | C | C | 0.79032 | T | 0.20968 | 11 | 0.35484 |
| 4555 | S5_85485896 | 5 | 85485896 | 621598 | T | C | T | 0.77419 | C | 0.22581 | 10 | 0.32258 |
| 4556 | S5_85485926 | 5 | 85485926 | 30 | A | G | A | 0.83871 | G | 0.16129 | 6 | 0.19355 |
| 4557 | S5_86056539 | 5 | 86056539 | 570613 | G | A | A | 0.72581 | G | 0.27419 | 15 | 0.48387 |
| 4558 | S5_86170545 | 5 | 86170545 | 114006 | G | A | G | 0.91935 | A | 0.08065 | 5 | 0.16129 |
| 4559 | S5_86578854 | 5 | 86578854 | 408309 | A | G | G | 0.75806 | A | 0.24194 | 11 | 0.35484 |
| 4560 | S5_86660725 | 5 | 86660725 | 81871 | G | C | G | 0.74194 | C | 0.25806 | 12 | 0.3871 |
| 4561 | S5_87014401 | 5 | 87014401 | 353676 | T | C | T | 0.87097 | C | 0.12903 | 6 | 0.19355 |
| 4562 | S5_87103638 | 5 | 87103638 | 89237 | A | G | A | 0.8871 | G | 0.1129 | 7 | 0.22581 |
| 4563 | S5_87103813 | 5 | 87103813 | 175 | C | T | C | 0.80645 | T | 0.19355 | 8 | 0.25806 |
| 4564 | S5_87103834 | 5 | 87103834 | 21 | T | G | T | 0.67742 | G | 0.32258 | 12 | 0.3871 |
| 4565 | S5_87965410 | 5 | 87965410 | 861576 | A | G | A | 0.87097 | G | 0.12903 | 8 | 0.25806 |
| 4566 | S5_87965418 | 5 | 87965418 | 8 | C | T | C | 0.87097 | T | 0.12903 | 8 | 0.25806 |
| 4567 | S5_88052337 | 5 | 88052337 | 86919 | A | G | A | 0.82258 | G | 0.17742 | 9 | 0.29032 |
| 4568 | S5_88052397 | 5 | 88052397 | 60 | G | A | A | 0.58065 | G | 0.41935 | 12 | 0.3871 |
| 4569 | S5_88114913 | 5 | 88114913 | 62516 | T | C | T | 0.90323 | C | 0.09677 | 6 | 0.19355 |
| 4570 | S5_88752436 | 5 | 88752436 | 637523 | T | C | T | 0.95161 | C | 0.04839 | 3 | 0.09677 |
| 4571 | S5_88752564 | 5 | 88752564 | 128 | C | T | C | 0.8871 | T | 0.1129 | 5 | 0.16129 |
| 4572 | S5_89231364 | 5 | 89231364 | 478800 | T | G | T | 0.85484 | G | 0.14516 | 7 | 0.22581 |
| 4573 | S5_89231458 | 5 | 89231458 | 94 | C | T | C | 0.77419 | T | 0.22581 | 12 | 0.3871 |
| 4574 | S5_89231520 | 5 | 89231520 | 62 | C | T | C | 0.91935 | T | 0.08065 | 5 | 0.16129 |
| 4575 | S5_89231521 | 5 | 89231521 | 1 | G | A | G | 0.87097 | A | 0.12903 | 8 | 0.25806 |
| 4576 | S5_89335693 | 5 | 89335693 | 104172 | G | C | G | 0.74194 | C | 0.25806 | 10 | 0.32258 |
| 4577 | S5_89335807 | 5 | 89335807 | 114 | T | C | T | 0.74194 | C | 0.25806 | 10 | 0.32258 |
| 4578 | S5_89335818 | 5 | 89335818 | 11 | T | C | T | 0.90323 | C | 0.09677 | 6 | 0.19355 |
| 4579 | S5_89335874 | 5 | 89335874 | 56 | C | T | C | 0.90323 | T | 0.09677 | 6 | 0.19355 |
| 4580 | S5_89343316 | 5 | 89343316 | 7442 | T | A | T | 0.85484 | A | 0.14516 | 9 | 0.29032 |
| 4581 | S5_89343333 | 5 | 89343333 | 17 | C | T | C | 0.93548 | T | 0.06452 | 2 | 0.06452 |
| 4582 | S5_89343377 | 5 | 89343377 | 44 | C | T | C | 0.90323 | T | 0.09677 | 6 | 0.19355 |
| 4583 | S5_89343424 | 5 | 89343424 | 47 | T | C | T | 0.93548 | C | 0.06452 | 4 | 0.12903 |
| 4584 | S5_89343435 | 5 | 89343435 | 11 | C | T | C | 0.79032 | T | 0.20968 | 11 | 0.35484 |
| 4585 | S5_89343509 | 5 | 89343509 | 74 | G | T | G | 0.90323 | T | 0.09677 | 6 | 0.19355 |
| 4586 | S5_89343523 | 5 | 89343523 | 14 | G | A | G | 0.90323 | A | 0.09677 | 6 | 0.19355 |
| 4587 | S5_90360890 | 5 | 90360890 | 1017367 | C | T | C | 0.8871 | T | 0.1129 | 7 | 0.22581 |
| 4588 | S5_90870633 | 5 | 90870633 | 509743 | A | C | A | 0.59677 | C | 0.40323 | 13 | 0.41935 |

| Site number | SNP name | Chromosome | Physical position | Physical distance from the previous SNP | Reference allele | Alternative allele | Major allele | Major allele frequency | Minor allele | Minor allele frequency | Number heterozygous | Heterozygosity |
|---|---|---|---|---|---|---|---|---|---|---|---|---|
| 4589 | S5_90870645 | 5 | 90870645 | 12 | A | G | A | 0.91935 | G | 0.08065 | 5 | 0.16129 |
| 4590 | S5_90942037 | 5 | 90942037 | 71392 | G | A | G | 0.93548 | A | 0.06452 | 4 | 0.12903 |
| 4591 | S5_90942074 | 5 | 90942074 | 37 | C | G | C | 0.70968 | G | 0.29032 | 12 | 0.3871 |
| 4592 | S5_91105546 | 5 | 91105546 | 163472 | C | T | C | 0.87097 | T | 0.12903 | 6 | 0.19355 |
| 4593 | S5_91105575 | 5 | 91105575 | 29 | C | G | C | 0.95161 | G | 0.04839 | 3 | 0.09677 |
| 4594 | S5_91679169 | 5 | 91679169 | 573594 | C | T | C | 0.95161 | T | 0.04839 | 3 | 0.09677 |
| 4595 | S5_91679184 | 5 | 91679184 | 15 | A | G | A | 0.95161 | G | 0.04839 | 3 | 0.09677 |
| 4596 | S5_91762018 | 5 | 91762018 | 82834 | C | T | C | 0.95161 | T | 0.04839 | 3 | 0.09677 |
| 4597 | S5_91762145 | 5 | 91762145 | 127 | C | T | C | 0.8871 | T | 0.1129 | 7 | 0.22581 |
| 4598 | S5_91834210 | 5 | 91834210 | 72065 | A | G | A | 0.79032 | G | 0.20968 | 7 | 0.22581 |
| 4599 | S5_91834324 | 5 | 91834324 | 114 | G | A | G | 0.64516 | A | 0.35484 | 10 | 0.32258 |
| 4600 | S5_91834433 | 5 | 91834433 | 109 | T | C | T | 0.72581 | C | 0.27419 | 9 | 0.29032 |
| 4601 | S5_91909586 | 5 | 91909586 | 75153 | G | A | G | 0.83871 | A | 0.16129 | 10 | 0.32258 |
| 4602 | S5_92064909 | 5 | 92064909 | 155323 | G | A | G | 0.95161 | A | 0.04839 | 3 | 0.09677 |
| 4603 | S5_92064913 | 5 | 92064913 | 4 | G | A | A | 0.95161 | G | 0.04839 | 3 | 0.09677 |
| 4604 | S5_92238050 | 5 | 92238050 | 173137 | T | C | T | 0.95161 | C | 0.04839 | 3 | 0.09677 |
| 4605 | S5_92238069 | 5 | 92238069 | 19 | G | C | C | 0.53226 | G | 0.46774 | 17 | 0.54839 |
| 4606 | S5_92238076 | 5 | 92238076 | 7 | C | T | C | 0.91935 | T | 0.08065 | 3 | 0.09677 |
| 4607 | S5_92316526 | 5 | 92316526 | 78450 | G | A | G | 0.91935 | A | 0.08065 | 5 | 0.16129 |
| 4608 | S5_92316599 | 5 | 92316599 | 73 | G | T | G | 0.85484 | T | 0.14516 | 9 | 0.29032 |
| 4609 | S5_92710179 | 5 | 92710179 | 393580 | G | A | G | 0.82258 | A | 0.17742 | 7 | 0.22581 |
| 4610 | S5_93178764 | 5 | 93178764 | 468585 | G | A | G | 0.8871 | A | 0.1129 | 5 | 0.16129 |
| 4611 | S5_93178779 | 5 | 93178779 | 15 | G | A | G | 0.8871 | A | 0.1129 | 7 | 0.22581 |
| 4612 | S5_93178887 | 5 | 93178887 | 108 | G | A | G | 0.75806 | A | 0.24194 | 13 | 0.41935 |
| 4613 | S5_93893240 | 5 | 93893240 | 714353 | T | C | T | 0.8871 | C | 0.1129 | 7 | 0.22581 |
| 4614 | S5_94133797 | 5 | 94133797 | 240557 | C | T | C | 0.91935 | T | 0.08065 | 3 | 0.09677 |
| 4615 | S5_94466618 | 5 | 94466618 | 332821 | C | T | C | 0.74194 | T | 0.25806 | 12 | 0.3871 |
| 4616 | S5_94516704 | 5 | 94516704 | 50086 | C | T | C | 0.95161 | T | 0.04839 | 1 | 0.03226 |
| 4617 | S5_94516731 | 5 | 94516731 | 27 | C | T | C | 0.90323 | T | 0.09677 | 6 | 0.19355 |
| 4618 | S5_94516826 | 5 | 94516826 | 95 | G | A | G | 0.95161 | A | 0.04839 | 1 | 0.03226 |
| 4619 | S5_94516891 | 5 | 94516891 | 65 | G | A | A | 0.56452 | G | 0.43548 | 13 | 0.41935 |
| 4620 | S5_94819732 | 5 | 94819732 | 302841 | C | T | C | 0.85484 | T | 0.14516 | 9 | 0.29032 |
| 4621 | S5_94907743 | 5 | 94907743 | 88011 | G | A | G | 0.8871 | A | 0.1129 | 7 | 0.22581 |
| 4622 | S5_94907769 | 5 | 94907769 | 26 | A | T | A | 0.90323 | T | 0.09677 | 4 | 0.12903 |
| 4623 | S5_94907849 | 5 | 94907849 | 80 | A | G | A | 0.93548 | G | 0.06452 | 4 | 0.12903 |
| 4624 | S5_95193196 | 5 | 95193196 | 285347 | G | T | G | 0.91935 | T | 0.08065 | 5 | 0.16129 |
| 4625 | S5_95193197 | 5 | 95193197 | 1 | G | A | A | 0.51613 | G | 0.48387 | 14 | 0.45161 |
| 4626 | S5_95193205 | 5 | 95193205 | 8 | G | A | G | 0.90323 | A | 0.09677 | 6 | 0.19355 |
| 4627 | S5_95193268 | 5 | 95193268 | 63 | T | G | T | 0.90323 | G | 0.09677 | 6 | 0.19355 |
| 4628 | S5_95193319 | 5 | 95193319 | 51 | G | A | G | 0.90323 | A | 0.09677 | 6 | 0.19355 |
| 4629 | S5_95193362 | 5 | 95193362 | 43 | C | T | C | 0.90323 | T | 0.09677 | 6 | 0.19355 |
| 4630 | S5_95193413 | 5 | 95193413 | 51 | T | C | T | 0.90323 | C | 0.09677 | 6 | 0.19355 |
| 4631 | S5_95193429 | 5 | 95193429 | 16 | T | C | T | 0.82258 | C | 0.17742 | 11 | 0.35484 |
| 4632 | S5_95270922 | 5 | 95270922 | 77493 | C | T | C | 0.77419 | T | 0.22581 | 12 | 0.3871 |
| 4633 | S5_95369863 | 5 | 95369863 | 98941 | T | C | C | 0.83871 | T | 0.16129 | 8 | 0.25806 |
| 4634 | S5_95369974 | 5 | 95369974 | 111 | G | A | A | 0.75806 | G | 0.24194 | 11 | 0.35484 |
| 4635 | S5_95547264 | 5 | 95547264 | 177290 | A | G | A | 0.79032 | G | 0.20968 | 13 | 0.41935 |
| 4636 | S5_95547323 | 5 | 95547323 | 59 | G | A | G | 0.95161 | A | 0.04839 | 3 | 0.09677 |
| 4637 | S5_95547360 | 5 | 95547360 | 37 | G | A | G | 0.79032 | A | 0.20968 | 13 | 0.41935 |
| 4638 | S5_95629594 | 5 | 95629594 | 82234 | C | T | C | 0.93548 | T | 0.06452 | 4 | 0.12903 |
| 4639 | S5_95629595 | 5 | 95629595 | 1 | G | T | G | 0.93548 | T | 0.06452 | 4 | 0.12903 |
| 4640 | S5_95927449 | 5 | 95927449 | 297854 | C | T | C | 0.90323 | T | 0.09677 | 6 | 0.19355 |
| 4641 | S5_96070300 | 5 | 96070300 | 142851 | G | A | G | 0.77419 | A | 0.22581 | 12 | 0.3871 |
| 4642 | S5_96070307 | 5 | 96070307 | 7 | C | A | A | 0.8871 | C | 0.1129 | 7 | 0.22581 |

| Site number | SNP name | Chromosome | Physical position | Physical distance from the previous SNP | Reference allele | Alternative allele | Major allele | Major allele frequency | Minor allele | Minor allele frequency | Number heterozygous | Heterozygosity |
|---|---|---|---|---|---|---|---|---|---|---|---|---|
| 4643 | S5_96070332 | 5 | 96070332 | 25 | T | C | C | 0.8871 | T | 0.1129 | 7 | 0.22581 |
| 4644 | S5_96199838 | 5 | 96199838 | 129506 | C | T | T | 0.74194 | C | 0.25806 | 12 | 0.3871 |
| 4645 | S5_96200041 | 5 | 96200041 | 203 | T | C | T | 0.95161 | C | 0.04839 | 3 | 0.09677 |
| 4646 | S5_96499637 | 5 | 96499637 | 299596 | G | A | G | 0.93548 | A | 0.06452 | 4 | 0.12903 |
| 4647 | S5_96594040 | 5 | 96594040 | 94403 | G | A | G | 0.87097 | A | 0.12903 | 6 | 0.19355 |
| 4648 | S5_96594069 | 5 | 96594069 | 29 | T | C | T | 0.93548 | C | 0.06452 | 4 | 0.12903 |
| 4649 | S5_96713057 | 5 | 96713057 | 118988 | T | C | T | 0.91935 | C | 0.08065 | 5 | 0.16129 |
| 4650 | S5_96713226 | 5 | 96713226 | 169 | T | G | T | 0.79032 | G | 0.20968 | 7 | 0.22581 |
| 4651 | S5_96771004 | 5 | 96771004 | 57778 | G | C | G | 0.64516 | C | 0.35484 | 12 | 0.3871 |
| 4652 | S5_96852348 | 5 | 96852348 | 81344 | T | C | T | 0.69355 | C | 0.30645 | 11 | 0.35484 |
| 4653 | S5_96935684 | 5 | 96935684 | 83336 | A | C | A | 0.95161 | C | 0.04839 | 3 | 0.09677 |
| 4654 | S5_96935832 | 5 | 96935832 | 148 | C | T | C | 0.77419 | T | 0.22581 | 8 | 0.25806 |
| 4655 | S5_97141118 | 5 | 97141118 | 205286 | T | C | T | 0.95161 | C | 0.04839 | 3 | 0.09677 |
| 4656 | S5_97141174 | 5 | 97141174 | 56 | T | A | A | 0.67742 | T | 0.32258 | 12 | 0.3871 |
| 4657 | S5_97141189 | 5 | 97141189 | 15 | C | G | C | 0.91935 | G | 0.08065 | 3 | 0.09677 |
| 4658 | S5_97141227 | 5 | 97141227 | 38 | C | G | C | 0.95161 | G | 0.04839 | 3 | 0.09677 |
| 4659 | S5_97141265 | 5 | 97141265 | 38 | T | C | C | 0.67742 | T | 0.32258 | 12 | 0.3871 |
| 4660 | S5_97141280 | 5 | 97141280 | 15 | C | T | C | 0.64516 | T | 0.35484 | 12 | 0.3871 |
| 4661 | S5_97510939 | 5 | 97510939 | 369659 | C | T | T | 0.66129 | C | 0.33871 | 11 | 0.35484 |
| 4662 | S5_97510940 | 5 | 97510940 | 1 | A | G | G | 0.66129 | A | 0.33871 | 11 | 0.35484 |
| 4663 | S5_97510958 | 5 | 97510958 | 18 | G | A | G | 0.74194 | A | 0.25806 | 10 | 0.32258 |
| 4664 | S5_97510962 | 5 | 97510962 | 4 | T | C | C | 0.91935 | T | 0.08065 | 5 | 0.16129 |
| 4665 | S5_97511119 | 5 | 97511119 | 157 | C | T | T | 0.66129 | C | 0.33871 | 11 | 0.35484 |
| 4666 | S5_98451100 | 5 | 98451100 | 939981 | C | G | G | 0.53226 | C | 0.46774 | 13 | 0.41935 |
| 4667 | S5_98451117 | 5 | 98451117 | 17 | G | A | A | 0.53226 | G | 0.46774 | 13 | 0.41935 |
| 4668 | S5_98451235 | 5 | 98451235 | 118 | G | A | A | 0.53226 | G | 0.46774 | 13 | 0.41935 |
| 4669 | S5_98451302 | 5 | 98451302 | 67 | C | T | C | 0.77419 | T | 0.22581 | 14 | 0.45161 |
| 4670 | S5_98891691 | 5 | 98891691 | 440389 | C | A | C | 0.93548 | A | 0.06452 | 2 | 0.06452 |
| 4671 | S5_98891782 | 5 | 98891782 | 91 | G | A | G | 0.95161 | A | 0.04839 | 3 | 0.09677 |
| 4672 | S5_99204227 | 5 | 99204227 | 312445 | G | A | G | 0.82258 | A | 0.17742 | 11 | 0.35484 |
| 4673 | S5_99204235 | 5 | 99204235 | 8 | A | T | A | 0.82258 | T | 0.17742 | 11 | 0.35484 |
| 4674 | S5_99204281 | 5 | 99204281 | 46 | T | C | T | 0.82258 | C | 0.17742 | 11 | 0.35484 |
| 4675 | S5_99204288 | 5 | 99204288 | 7 | C | T | C | 0.82258 | T | 0.17742 | 11 | 0.35484 |
| 4676 | S5_99204297 | 5 | 99204297 | 9 | T | C | T | 0.82258 | C | 0.17742 | 11 | 0.35484 |
| 4677 | S5_99204364 | 5 | 99204364 | 67 | C | A | C | 0.80645 | A | 0.19355 | 12 | 0.3871 |
| 4678 | S5_99204393 | 5 | 99204393 | 29 | C | T | C | 0.82258 | T | 0.17742 | 11 | 0.35484 |
| 4679 | S5_99208708 | 5 | 99208708 | 4315 | A | G | A | 0.95161 | G | 0.04839 | 3 | 0.09677 |
| 4680 | S5_99208801 | 5 | 99208801 | 93 | G | A | G | 0.80645 | A | 0.19355 | 12 | 0.3871 |
| 4681 | S5_99208810 | 5 | 99208810 | 9 | T | A | T | 0.95161 | A | 0.04839 | 3 | 0.09677 |
| 4682 | S5_99272203 | 5 | 99272203 | 63393 | G | A | G | 0.85484 | A | 0.14516 | 9 | 0.29032 |
| 4683 | S5_99272283 | 5 | 99272283 | 80 | T | C | T | 0.83871 | C | 0.16129 | 10 | 0.32258 |
| 4684 | S5_99272320 | 5 | 99272320 | 37 | C | T | C | 0.90323 | T | 0.09677 | 6 | 0.19355 |
| 4685 | S5_99367987 | 5 | 99367987 | 95667 | G | A | G | 0.95161 | A | 0.04839 | 3 | 0.09677 |
| 4686 | S5_99368017 | 5 | 99368017 | 30 | T | C | C | 0.67742 | T | 0.32258 | 18 | 0.58065 |
| 4687 | S5_99368064 | 5 | 99368064 | 47 | C | T | C | 0.91935 | T | 0.08065 | 3 | 0.09677 |
| 4688 | S5_99368126 | 5 | 99368126 | 62 | C | T | T | 0.59677 | C | 0.40323 | 17 | 0.54839 |
| 4689 | S5_99368129 | 5 | 99368129 | 3 | A | G | G | 0.67742 | A | 0.32258 | 18 | 0.58065 |
| 4690 | S5_99368148 | 5 | 99368148 | 19 | G | A | G | 0.59677 | A | 0.40323 | 17 | 0.54839 |
| 4691 | S5_99383570 | 5 | 99383570 | 15422 | T | C | T | 0.83871 | C | 0.16129 | 6 | 0.19355 |
| 4692 | S5_99383627 | 5 | 99383627 | 57 | T | A | A | 0.77419 | T | 0.22581 | 10 | 0.32258 |
| 4693 | S5_99383662 | 5 | 99383662 | 35 | G | A | G | 0.8871 | A | 0.1129 | 5 | 0.16129 |
| 4694 | S5_99383694 | 5 | 99383694 | 32 | T | C | C | 0.77419 | T | 0.22581 | 10 | 0.32258 |
| 4695 | S5_99383713 | 5 | 99383713 | 19 | G | T | T | 0.59677 | G | 0.40323 | 13 | 0.41935 |
| 4696 | S5_99383810 | 5 | 99383810 | 97 | G | A | G | 0.8871 | A | 0.1129 | 5 | 0.16129 |

| Site number | SNP name | Chromosome | Physical position | Physical distance from the previous SNP | Reference allele | Alternative allele | Major allele | Major allele frequency | Minor allele | Minor allele frequency | Number heterozygous | Heterozygosity |
|---|---|---|---|---|---|---|---|---|---|---|---|---|
| 4697 | S5_100224716 | 5 | 100224716 | 840906 | C | G | G | 0.66129 | C | 0.33871 | 13 | 0.41935 |
| 4698 | S5_100224758 | 5 | 100224758 | 42 | C | T | T | 0.51613 | C | 0.48387 | 14 | 0.45161 |
| 4699 | S5_100224781 | 5 | 100224781 | 23 | G | C | G | 0.85484 | C | 0.14516 | 7 | 0.22581 |
| 4700 | S5_100224783 | 5 | 100224783 | 2 | T | C | C | 0.51613 | T | 0.48387 | 14 | 0.45161 |
| 4701 | S5_100224934 | 5 | 100224934 | 151 | C | T | C | 0.59677 | T | 0.40323 | 15 | 0.48387 |
| 4702 | S5_100267685 | 5 | 100267685 | 42751 | G | A | G | 0.64516 | A | 0.35484 | 16 | 0.51613 |
| 4703 | S5_100669159 | 5 | 100669159 | 401474 | C | T | C | 0.90323 | T | 0.09677 | 6 | 0.19355 |
| 4704 | S5_100905989 | 5 | 100905989 | 236830 | A | C | A | 0.82258 | C | 0.17742 | 11 | 0.35484 |
| 4705 | S5_100906002 | 5 | 100906002 | 13 | T | C | T | 0.93548 | C | 0.06452 | 4 | 0.12903 |
| 4706 | S5_100906031 | 5 | 100906031 | 29 | C | T | C | 0.80645 | T | 0.19355 | 12 | 0.3871 |
| 4707 | S5_101029438 | 5 | 101029438 | 123407 | G | T | G | 0.95161 | T | 0.04839 | 3 | 0.09677 |
| 4708 | S5_101029521 | 5 | 101029521 | 83 | A | C | A | 0.75806 | C | 0.24194 | 15 | 0.48387 |
| 4709 | S5_101029522 | 5 | 101029522 | 1 | G | A | G | 0.75806 | A | 0.24194 | 15 | 0.48387 |
| 4710 | S5_101127858 | 5 | 101127858 | 98336 | G | A | G | 0.8871 | A | 0.1129 | 7 | 0.22581 |
| 4711 | S5_101127860 | 5 | 101127860 | 2 | C | A | C | 0.8871 | A | 0.1129 | 7 | 0.22581 |
| 4712 | S5_101168795 | 5 | 101168795 | 40935 | G | A | G | 0.82258 | A | 0.17742 | 9 | 0.29032 |
| 4713 | S5_101168845 | 5 | 101168845 | 50 | G | A | A | 0.75806 | G | 0.24194 | 11 | 0.35484 |
| 4714 | S5_101168993 | 5 | 101168993 | 148 | C | T | C | 0.90323 | T | 0.09677 | 4 | 0.12903 |
| 4715 | S5_101177901 | 5 | 101177901 | 8908 | T | C | C | 0.51613 | T | 0.48387 | 20 | 0.64516 |
| 4716 | S5_101177958 | 5 | 101177958 | 57 | A | G | G | 0.8871 | A | 0.1129 | 7 | 0.22581 |
| 4717 | S5_101177963 | 5 | 101177963 | 5 | T | C | C | 0.53226 | T | 0.46774 | 17 | 0.54839 |
| 4718 | S5_101178009 | 5 | 101178009 | 46 | A | G | G | 0.77419 | A | 0.22581 | 10 | 0.32258 |
| 4719 | S5_101375831 | 5 | 101375831 | 197822 | A | C | A | 0.5 | C | 0.5 | 19 | 0.6129 |
| 4720 | S5_101530184 | 5 | 101530184 | 154353 | C | T | C | 0.90323 | T | 0.09677 | 6 | 0.19355 |
| 4721 | S5_101530225 | 5 | 101530225 | 41 | C | T | T | 0.70968 | C | 0.29032 | 16 | 0.51613 |
| 4722 | S5_101807198 | 5 | 101807198 | 276973 | G | A | G | 0.93548 | A | 0.06452 | 4 | 0.12903 |
| 4723 | S5_101807200 | 5 | 101807200 | 2 | G | T | G | 0.93548 | T | 0.06452 | 4 | 0.12903 |
| 4724 | S5_101807249 | 5 | 101807249 | 49 | T | G | T | 0.95161 | G | 0.04839 | 3 | 0.09677 |
| 4725 | S5_101807279 | 5 | 101807279 | 30 | C | G | C | 0.93548 | G | 0.06452 | 4 | 0.12903 |
| 4726 | S5_101807330 | 5 | 101807330 | 51 | A | G | A | 0.85484 | G | 0.14516 | 9 | 0.29032 |
| 4727 | S5_101807387 | 5 | 101807387 | 57 | G | A | G | 0.93548 | A | 0.06452 | 4 | 0.12903 |
| 4728 | S5_101925964 | 5 | 101925964 | 118577 | C | T | C | 0.8871 | T | 0.1129 | 7 | 0.22581 |
| 4729 | S5_101926099 | 5 | 101926099 | 135 | T | C | T | 0.90323 | C | 0.09677 | 6 | 0.19355 |
| 4730 | S5_102090191 | 5 | 102090191 | 164092 | C | A | C | 0.91935 | A | 0.08065 | 5 | 0.16129 |
| 4731 | S5_102090348 | 5 | 102090348 | 157 | T | C | T | 0.51613 | C | 0.48387 | 12 | 0.3871 |
| 4732 | S5_102090349 | 5 | 102090349 | 1 | G | A | G | 0.91935 | A | 0.08065 | 5 | 0.16129 |
| 4733 | S5_102213139 | 5 | 102213139 | 122790 | G | C | G | 0.85484 | C | 0.14516 | 7 | 0.22581 |
| 4734 | S5_102213194 | 5 | 102213194 | 55 | G | A | A | 0.70968 | G | 0.29032 | 14 | 0.45161 |
| 4735 | S5_102541974 | 5 | 102541974 | 328780 | T | C | T | 0.87097 | C | 0.12903 | 8 | 0.25806 |
| 4736 | S5_102882955 | 5 | 102882955 | 340981 | A | G | A | 0.95161 | G | 0.04839 | 3 | 0.09677 |
| 4737 | S5_102883014 | 5 | 102883014 | 59 | T | C | T | 0.79032 | C | 0.20968 | 13 | 0.41935 |
| 4738 | S5_102935630 | 5 | 102935630 | 52616 | G | T | G | 0.80645 | T | 0.19355 | 6 | 0.19355 |
| 4739 | S5_102969948 | 5 | 102969948 | 34318 | T | C | T | 0.90323 | C | 0.09677 | 6 | 0.19355 |
| 4740 | S5_103114494 | 5 | 103114494 | 144546 | C | T | C | 0.93548 | T | 0.06452 | 4 | 0.12903 |
| 4741 | S5_103114654 | 5 | 103114654 | 160 | C | T | C | 0.90323 | T | 0.09677 | 6 | 0.19355 |
| 4742 | S5_103205286 | 5 | 103205286 | 90632 | G | T | G | 0.93548 | T | 0.06452 | 4 | 0.12903 |
| 4743 | S5_103205346 | 5 | 103205346 | 60 | C | T | C | 0.93548 | T | 0.06452 | 4 | 0.12903 |
| 4744 | S5_103205388 | 5 | 103205388 | 42 | G | A | G | 0.90323 | A | 0.09677 | 6 | 0.19355 |
| 4745 | S5_103573506 | 5 | 103573506 | 368118 | G | A | G | 0.93548 | A | 0.06452 | 4 | 0.12903 |
| 4746 | S5_103573547 | 5 | 103573547 | 41 | T | A | T | 0.93548 | A | 0.06452 | 4 | 0.12903 |
| 4747 | S5_103782427 | 5 | 103782427 | 208880 | G | A | G | 0.93548 | A | 0.06452 | 4 | 0.12903 |
| 4748 | S5_104001577 | 5 | 104001577 | 219150 | G | A | G | 0.79032 | A | 0.20968 | 13 | 0.41935 |
| 4749 | S5_104001784 | 5 | 104001784 | 207 | C | T | C | 0.85484 | T | 0.14516 | 7 | 0.22581 |
| 4750 | S5_104028805 | 5 | 104028805 | 27021 | G | C | G | 0.93548 | C | 0.06452 | 4 | 0.12903 |

| Site number | SNP name | Chromosome | Physical position | Physical distance from the previous SNP | Reference allele | Alternative allele | Major allele | Major allele frequency | Minor allele | Minor allele frequency | Number heterozygous | Heterozygosity |
|---|---|---|---|---|---|---|---|---|---|---|---|---|
| 4751 | S5_104077579 | 5 | 104077579 | 48774 | A | G | A | 0.90323 | G | 0.09677 | 4 | 0.12903 |
| 4752 | S5_104346906 | 5 | 104346906 | 269327 | C | T | C | 0.83871 | T | 0.16129 | 8 | 0.25806 |
| 4753 | S5_104346970 | 5 | 104346970 | 64 | G | T | G | 0.59677 | T | 0.40323 | 13 | 0.41935 |
| 4754 | S5_104346982 | 5 | 104346982 | 12 | C | A | C | 0.59677 | A | 0.40323 | 13 | 0.41935 |
| 4755 | S5_104346991 | 5 | 104346991 | 9 | G | A | G | 0.59677 | A | 0.40323 | 13 | 0.41935 |
| 4756 | S5_104346992 | 5 | 104346992 | 1 | A | G | A | 0.59677 | G | 0.40323 | 13 | 0.41935 |
| 4757 | S5_104347007 | 5 | 104347007 | 15 | A | G | A | 0.59677 | G | 0.40323 | 13 | 0.41935 |
| 4758 | S5_104347012 | 5 | 104347012 | 5 | C | T | C | 0.59677 | T | 0.40323 | 13 | 0.41935 |
| 4759 | S5_104347096 | 5 | 104347096 | 84 | C | T | T | 0.56452 | C | 0.43548 | 15 | 0.48387 |
| 4760 | S5_104475100 | 5 | 104475100 | 128004 | A | G | A | 0.85484 | G | 0.14516 | 7 | 0.22581 |
| 4761 | S5_104475192 | 5 | 104475192 | 92 | G | A | A | 0.75806 | G | 0.24194 | 13 | 0.41935 |
| 4762 | S5_104475248 | 5 | 104475248 | 56 | T | C | T | 0.83871 | C | 0.16129 | 8 | 0.25806 |
| 4763 | S5_104565889 | 5 | 104565889 | 90641 | T | C | T | 0.62903 | C | 0.37097 | 13 | 0.41935 |
| 4764 | S5_104565946 | 5 | 104565946 | 57 | C | T | C | 0.62903 | T | 0.37097 | 13 | 0.41935 |
| 4765 | S5_104566010 | 5 | 104566010 | 64 | A | T | A | 0.62903 | T | 0.37097 | 13 | 0.41935 |
| 4766 | S5_104864938 | 5 | 104864938 | 298928 | C | T | C | 0.87097 | T | 0.12903 | 8 | 0.25806 |
| 4767 | S5_104940549 | 5 | 104940549 | 75611 | C | T | C | 0.80645 | T | 0.19355 | 10 | 0.32258 |
| 4768 | S5_105007950 | 5 | 105007950 | 67401 | C | T | C | 0.95161 | T | 0.04839 | 3 | 0.09677 |
| 4769 | S5_105008014 | 5 | 105008014 | 64 | G | T | G | 0.75806 | T | 0.24194 | 15 | 0.48387 |
| 4770 | S5_105230689 | 5 | 105230689 | 222675 | C | T | C | 0.90323 | T | 0.09677 | 4 | 0.12903 |
| 4771 | S5_105230845 | 5 | 105230845 | 156 | A | G | A | 0.90323 | G | 0.09677 | 4 | 0.12903 |
| 4772 | S5_105367862 | 5 | 105367862 | 137017 | C | A | A | 0.90323 | C | 0.09677 | 4 | 0.12903 |
| 4773 | S5_105368009 | 5 | 105368009 | 147 | G | A | G | 0.8871 | A | 0.1129 | 5 | 0.16129 |
| 4774 | S5_105368016 | 5 | 105368016 | 7 | T | C | T | 0.90323 | C | 0.09677 | 4 | 0.12903 |
| 4775 | S5_105368030 | 5 | 105368030 | 14 | T | A | T | 0.83871 | A | 0.16129 | 6 | 0.19355 |
| 4776 | S5_105416121 | 5 | 105416121 | 48091 | C | T | C | 0.6129 | T | 0.3871 | 16 | 0.51613 |
| 4777 | S5_105416122 | 5 | 105416122 | 1 | G | A | G | 0.93548 | A | 0.06452 | 4 | 0.12903 |
| 4778 | S5_105524945 | 5 | 105524945 | 108823 | A | G | A | 0.70968 | G | 0.29032 | 14 | 0.45161 |
| 4779 | S5_105524961 | 5 | 105524961 | 16 | A | T | A | 0.8871 | T | 0.1129 | 5 | 0.16129 |
| 4780 | S5_105564110 | 5 | 105564110 | 39149 | C | T | C | 0.93548 | T | 0.06452 | 4 | 0.12903 |
| 4781 | S5_105799242 | 5 | 105799242 | 235132 | G | T | G | 0.93548 | T | 0.06452 | 4 | 0.12903 |
| 4782 | S5_105799273 | 5 | 105799273 | 31 | C | T | C | 0.58065 | T | 0.41935 | 14 | 0.45161 |
| 4783 | S5_106148206 | 5 | 106148206 | 348933 | C | T | C | 0.64516 | T | 0.35484 | 12 | 0.3871 |
| 4784 | S5_106148327 | 5 | 106148327 | 121 | T | C | T | 0.91935 | C | 0.08065 | 5 | 0.16129 |
| 4785 | S5_106283430 | 5 | 106283430 | 135103 | A | G | A | 0.83871 | G | 0.16129 | 8 | 0.25806 |
| 4786 | S5_106360011 | 5 | 106360011 | 76581 | G | A | G | 0.95161 | A | 0.04839 | 3 | 0.09677 |
| 4787 | S5_106360148 | 5 | 106360148 | 137 | A | T | A | 0.91935 | T | 0.08065 | 5 | 0.16129 |
| 4788 | S5_106383277 | 5 | 106383277 | 23129 | G | A | G | 0.90323 | A | 0.09677 | 6 | 0.19355 |
| 4789 | S5_106611643 | 5 | 106611643 | 228366 | G | A | A | 0.93548 | G | 0.06452 | 4 | 0.12903 |
| 4790 | S5_106686758 | 5 | 106686758 | 75115 | G | C | G | 0.85484 | C | 0.14516 | 9 | 0.29032 |
| 4791 | S5_106686840 | 5 | 106686840 | 82 | C | T | C | 0.93548 | T | 0.06452 | 4 | 0.12903 |
| 4792 | S5_106732856 | 5 | 106732856 | 46016 | C | T | C | 0.91935 | T | 0.08065 | 5 | 0.16129 |
| 4793 | S5_106733055 | 5 | 106733055 | 199 | A | G | A | 0.90323 | G | 0.09677 | 6 | 0.19355 |
| 4794 | S5_106761059 | 5 | 106761059 | 28004 | C | T | C | 0.87097 | T | 0.12903 | 8 | 0.25806 |
| 4795 | S5_106761092 | 5 | 106761092 | 33 | G | A | G | 0.83871 | A | 0.16129 | 10 | 0.32258 |
| 4796 | S5_106806646 | 5 | 106806646 | 45554 | C | T | C | 0.75806 | T | 0.24194 | 11 | 0.35484 |
| 4797 | S5_106806748 | 5 | 106806748 | 102 | G | A | G | 0.8871 | A | 0.1129 | 5 | 0.16129 |
| 4798 | S5_106817656 | 5 | 106817656 | 10908 | G | T | G | 0.95161 | T | 0.04839 | 3 | 0.09677 |
| 4799 | S5_106817696 | 5 | 106817696 | 40 | G | A | G | 0.95161 | A | 0.04839 | 3 | 0.09677 |
| 4800 | S5_106817705 | 5 | 106817705 | 9 | G | A | G | 0.95161 | A | 0.04839 | 3 | 0.09677 |
| 4801 | S5_106817711 | 5 | 106817711 | 6 | G | A | G | 0.95161 | A | 0.04839 | 3 | 0.09677 |
| 4802 | S5_106897663 | 5 | 106897663 | 79952 | A | T | A | 0.74194 | T | 0.25806 | 12 | 0.3871 |
| 4803 | S5_106897784 | 5 | 106897784 | 121 | G | A | G | 0.91935 | A | 0.08065 | 3 | 0.09677 |
| 4804 | S5_106897806 | 5 | 106897806 | 22 | C | T | C | 0.90323 | T | 0.09677 | 6 | 0.19355 |

| Site number | SNP name | Chromosome | Physical position | Physical distance from the previous SNP | Reference allele | Alternative allele | Major allele | Major allele frequency | Minor allele | Minor allele frequency | Number heterozygous | Heterozygosity |
|---|---|---|---|---|---|---|---|---|---|---|---|---|
| 4805 | S5_106948869 | 5 | 106948869 | 51063 | T | G | T | 0.90323 | G | 0.09677 | 6 | 0.19355 |
| 4806 | S5_106949093 | 5 | 106949093 | 224 | A | G | A | 0.74194 | G | 0.25806 | 12 | 0.3871 |
| 4807 | S5_106975786 | 5 | 106975786 | 26693 | G | A | G | 0.91935 | A | 0.08065 | 3 | 0.09677 |
| 4808 | S5_107059379 | 5 | 107059379 | 83593 | G | A | G | 0.79032 | A | 0.20968 | 9 | 0.29032 |
| 4809 | S5_107059581 | 5 | 107059581 | 202 | G | A | G | 0.90323 | A | 0.09677 | 4 | 0.12903 |
| 4810 | S5_107086590 | 5 | 107086590 | 27009 | G | A | G | 0.83871 | A | 0.16129 | 10 | 0.32258 |
| 4811 | S5_107157059 | 5 | 107157059 | 70469 | C | T | C | 0.59677 | T | 0.40323 | 19 | 0.6129 |
| 4812 | S5_107157079 | 5 | 107157079 | 20 | A | G | A | 0.75806 | G | 0.24194 | 9 | 0.29032 |
| 4813 | S5_107643304 | 5 | 107643304 | 486225 | T | C | C | 0.87097 | T | 0.12903 | 8 | 0.25806 |
| 4814 | S5_107726516 | 5 | 107726516 | 83212 | G | A | A | 0.58065 | G | 0.41935 | 16 | 0.51613 |
| 4815 | S5_107916448 | 5 | 107916448 | 189932 | G | T | G | 0.8871 | T | 0.1129 | 7 | 0.22581 |
| 4816 | S5_107963829 | 5 | 107963829 | 47381 | G | T | G | 0.53226 | T | 0.46774 | 17 | 0.54839 |
| 4817 | S5_107963975 | 5 | 107963975 | 146 | A | G | A | 0.53226 | G | 0.46774 | 17 | 0.54839 |
| 4818 | S5_107989527 | 5 | 107989527 | 25552 | G | A | A | 0.54839 | G | 0.45161 | 18 | 0.58065 |
| 4819 | S5_107989580 | 5 | 107989580 | 53 | C | T | C | 0.90323 | T | 0.09677 | 6 | 0.19355 |
| 4820 | S5_108231011 | 5 | 108231011 | 241431 | G | T | G | 0.91935 | T | 0.08065 | 5 | 0.16129 |
| 4821 | S5_108454279 | 5 | 108454279 | 223268 | C | A | C | 0.90323 | A | 0.09677 | 6 | 0.19355 |
| 4822 | S5_108465299 | 5 | 108465299 | 11020 | T | C | T | 0.82258 | C | 0.17742 | 9 | 0.29032 |
| 4823 | S5_108659229 | 5 | 108659229 | 193930 | C | T | C | 0.95161 | T | 0.04839 | 3 | 0.09677 |
| 4824 | S5_108659369 | 5 | 108659369 | 140 | A | C | A | 0.6129 | C | 0.3871 | 16 | 0.51613 |
| 4825 | S5_108659404 | 5 | 108659404 | 35 | A | G | A | 0.62903 | G | 0.37097 | 15 | 0.48387 |
| 4826 | S5_108812135 | 5 | 108812135 | 152731 | T | C | C | 0.90323 | T | 0.09677 | 6 | 0.19355 |
| 4827 | S5_108812203 | 5 | 108812203 | 68 | C | T | T | 0.83871 | C | 0.16129 | 8 | 0.25806 |
| 4828 | S5_108812216 | 5 | 108812216 | 13 | C | T | T | 0.90323 | C | 0.09677 | 6 | 0.19355 |
| 4829 | S5_108812269 | 5 | 108812269 | 53 | C | T | C | 0.95161 | T | 0.04839 | 3 | 0.09677 |
| 4830 | S5_108812332 | 5 | 108812332 | 63 | C | T | C | 0.53226 | T | 0.46774 | 17 | 0.54839 |
| 4831 | S5_109741242 | 5 | 109741242 | 928910 | A | G | A | 0.72581 | G | 0.27419 | 13 | 0.41935 |
| 4832 | S5_109741302 | 5 | 109741302 | 60 | C | T | C | 0.91935 | T | 0.08065 | 5 | 0.16129 |
| 4833 | S5_109741405 | 5 | 109741405 | 103 | C | A | C | 0.91935 | A | 0.08065 | 5 | 0.16129 |
| 4834 | S5_109890495 | 5 | 109890495 | 149090 | G | A | G | 0.8871 | A | 0.1129 | 7 | 0.22581 |
| 4835 | S5_110005260 | 5 | 110005260 | 114765 | T | C | T | 0.53226 | C | 0.46774 | 15 | 0.48387 |
| 4836 | S5_110005303 | 5 | 110005303 | 43 | T | C | T | 0.53226 | C | 0.46774 | 15 | 0.48387 |
| 4837 | S5_110215504 | 5 | 110215504 | 210201 | A | G | A | 0.74194 | G | 0.25806 | 14 | 0.45161 |
| 4838 | S5_110215702 | 5 | 110215702 | 198 | C | T | C | 0.75806 | T | 0.24194 | 13 | 0.41935 |
| 4839 | S5_110279846 | 5 | 110279846 | 64144 | C | T | C | 0.95161 | T | 0.04839 | 3 | 0.09677 |
| 4840 | S5_110279971 | 5 | 110279971 | 125 | G | A | G | 0.85484 | A | 0.14516 | 9 | 0.29032 |
| 4841 | S5_110280094 | 5 | 110280094 | 123 | C | A | C | 0.95161 | A | 0.04839 | 3 | 0.09677 |
| 4842 | S5_110280095 | 5 | 110280095 | 1 | G | A | G | 0.85484 | A | 0.14516 | 9 | 0.29032 |
| 4843 | S5_111100633 | 5 | 111100633 | 820538 | A | G | A | 0.51613 | G | 0.48387 | 18 | 0.58065 |
| 4844 | S5_112147118 | 5 | 112147118 | 1046485 | T | C | C | 0.87097 | T | 0.12903 | 8 | 0.25806 |
| 4845 | S5_112520004 | 5 | 112520004 | 372886 | G | A | G | 0.80645 | A | 0.19355 | 8 | 0.25806 |
| 4846 | S5_112520139 | 5 | 112520139 | 135 | A | C | C | 0.66129 | A | 0.33871 | 13 | 0.41935 |
| 4847 | S5_112520172 | 5 | 112520172 | 33 | A | G | A | 0.72581 | G | 0.27419 | 11 | 0.35484 |
| 4848 | S5_112571237 | 5 | 112571237 | 51065 | T | C | T | 0.75806 | C | 0.24194 | 11 | 0.35484 |
| 4849 | S5_112571243 | 5 | 112571243 | 6 | G | A | G | 0.77419 | A | 0.22581 | 10 | 0.32258 |
| 4850 | S5_112571258 | 5 | 112571258 | 15 | T | C | T | 0.77419 | C | 0.22581 | 10 | 0.32258 |
| 4851 | S5_112618299 | 5 | 112618299 | 47041 | A | G | A | 0.83871 | G | 0.16129 | 10 | 0.32258 |
| 4852 | S5_112618352 | 5 | 112618352 | 53 | C | G | C | 0.95161 | G | 0.04839 | 3 | 0.09677 |
| 4853 | S5_112660398 | 5 | 112660398 | 42046 | T | A | T | 0.83871 | A | 0.16129 | 8 | 0.25806 |
| 4854 | S5_112660429 | 5 | 112660429 | 31 | G | C | G | 0.91935 | C | 0.08065 | 5 | 0.16129 |
| 4855 | S5_112716873 | 5 | 112716873 | 56444 | A | C | A | 0.74194 | C | 0.25806 | 12 | 0.3871 |
| 4856 | S5_112717005 | 5 | 112717005 | 132 | G | A | G | 0.91935 | A | 0.08065 | 5 | 0.16129 |
| 4857 | S5_112739193 | 5 | 112739193 | 22188 | T | C | C | 0.75806 | T | 0.24194 | 13 | 0.41935 |
| 4858 | S5_112853814 | 5 | 112853814 | 114621 | G | A | G | 0.95161 | A | 0.04839 | 3 | 0.09677 |

| Site number | SNP name | Chromosome | Physical position | Physical distance from the previous SNP | Reference allele | Alternative allele | Major allele | Major allele frequency | Minor allele | Minor allele frequency | Number heterozygous | Heterozygosity |
|---|---|---|---|---|---|---|---|---|---|---|---|---|
| 4859 | S5_113053027 | 5 | 113053027 | 199213 | C | T | T | 0.59677 | C | 0.40323 | 13 | 0.41935 |
| 4860 | S5_113338178 | 5 | 113338178 | 285151 | T | C | T | 0.75806 | C | 0.24194 | 15 | 0.48387 |
| 4861 | S5_113338269 | 5 | 113338269 | 91 | A | C | A | 0.91935 | C | 0.08065 | 5 | 0.16129 |
| 4862 | S5_113338371 | 5 | 113338371 | 102 | T | C | T | 0.87097 | C | 0.12903 | 6 | 0.19355 |
| 4863 | S5_113338374 | 5 | 113338374 | 3 | C | T | C | 0.90323 | T | 0.09677 | 6 | 0.19355 |
| 4864 | S5_113338388 | 5 | 113338388 | 14 | G | A | G | 0.87097 | A | 0.12903 | 6 | 0.19355 |
| 4865 | S5_113349910 | 5 | 113349910 | 11522 | A | C | C | 0.70968 | A | 0.29032 | 12 | 0.3871 |
| 4866 | S5_113479382 | 5 | 113479382 | 129472 | C | T | C | 0.82258 | T | 0.17742 | 11 | 0.35484 |
| 4867 | S5_113479393 | 5 | 113479393 | 11 | T | C | T | 0.70968 | C | 0.29032 | 14 | 0.45161 |
| 4868 | S5_113479529 | 5 | 113479529 | 136 | A | C | A | 0.70968 | C | 0.29032 | 14 | 0.45161 |
| 4869 | S5_113713282 | 5 | 113713282 | 233753 | C | T | C | 0.87097 | T | 0.12903 | 8 | 0.25806 |
| 4870 | S5_113713341 | 5 | 113713341 | 59 | C | T | C | 0.95161 | T | 0.04839 | 3 | 0.09677 |
| 4871 | S5_113713421 | 5 | 113713421 | 80 | C | T | C | 0.87097 | T | 0.12903 | 8 | 0.25806 |
| 4872 | S5_113713440 | 5 | 113713440 | 19 | C | T | C | 0.87097 | T | 0.12903 | 8 | 0.25806 |
| 4873 | S5_113713465 | 5 | 113713465 | 25 | C | G | C | 0.87097 | G | 0.12903 | 8 | 0.25806 |
| 4874 | S5_113713470 | 5 | 113713470 | 5 | C | T | C | 0.87097 | T | 0.12903 | 8 | 0.25806 |
| 4875 | S5_113713478 | 5 | 113713478 | 8 | T | C | T | 0.87097 | C | 0.12903 | 8 | 0.25806 |
| 4876 | S5_113839273 | 5 | 113839273 | 125795 | C | G | C | 0.70968 | G | 0.29032 | 14 | 0.45161 |
| 4877 | S5_113839281 | 5 | 113839281 | 8 | C | T | C | 0.75806 | T | 0.24194 | 9 | 0.29032 |
| 4878 | S5_114247833 | 5 | 114247833 | 408552 | T | C | T | 0.56452 | C | 0.43548 | 11 | 0.35484 |
| 4879 | S5_114247845 | 5 | 114247845 | 12 | G | C | G | 0.54839 | C | 0.45161 | 12 | 0.3871 |
| 4880 | S5_114248039 | 5 | 114248039 | 194 | A | G | A | 0.90323 | G | 0.09677 | 4 | 0.12903 |
| 4881 | S5_114295840 | 5 | 114295840 | 47801 | G | T | G | 0.95161 | T | 0.04839 | 3 | 0.09677 |
| 4882 | S5_114295923 | 5 | 114295923 | 83 | A | G | A | 0.70968 | G | 0.29032 | 14 | 0.45161 |
| 4883 | S5_114295995 | 5 | 114295995 | 72 | T | C | T | 0.70968 | C | 0.29032 | 14 | 0.45161 |
| 4884 | S5_114440816 | 5 | 114440816 | 144821 | A | G | A | 0.8871 | G | 0.1129 | 5 | 0.16129 |
| 4885 | S5_114440883 | 5 | 114440883 | 67 | A | G | A | 0.87097 | G | 0.12903 | 6 | 0.19355 |
| 4886 | S5_114440897 | 5 | 114440897 | 14 | T | C | T | 0.79032 | C | 0.20968 | 11 | 0.35484 |
| 4887 | S5_114639511 | 5 | 114639511 | 198614 | C | T | C | 0.80645 | T | 0.19355 | 12 | 0.3871 |
| 4888 | S5_114639516 | 5 | 114639516 | 5 | G | T | G | 0.91935 | T | 0.08065 | 5 | 0.16129 |
| 4889 | S5_114815057 | 5 | 114815057 | 175541 | T | C | T | 0.91935 | C | 0.08065 | 5 | 0.16129 |
| 4890 | S5_115097357 | 5 | 115097357 | 282300 | A | G | G | 0.82258 | A | 0.17742 | 11 | 0.35484 |
| 4891 | S5_115097403 | 5 | 115097403 | 46 | G | A | G | 0.82258 | A | 0.17742 | 11 | 0.35484 |
| 4892 | S5_115435486 | 5 | 115435486 | 338083 | T | C | T | 0.93548 | C | 0.06452 | 4 | 0.12903 |
| 4893 | S5_115435532 | 5 | 115435532 | 46 | C | A | A | 0.93548 | C | 0.06452 | 4 | 0.12903 |
| 4894 | S5_115435541 | 5 | 115435541 | 9 | A | G | A | 0.93548 | G | 0.06452 | 4 | 0.12903 |
| 4895 | S5_116138085 | 5 | 116138085 | 702544 | C | G | C | 0.62903 | G | 0.37097 | 13 | 0.41935 |
| 4896 | S5_116138187 | 5 | 116138187 | 102 | C | T | C | 0.80645 | T | 0.19355 | 12 | 0.3871 |
| 4897 | S5_116138239 | 5 | 116138239 | 52 | T | C | T | 0.91935 | C | 0.08065 | 3 | 0.09677 |
| 4898 | S5_116138242 | 5 | 116138242 | 3 | A | G | A | 0.91935 | G | 0.08065 | 3 | 0.09677 |
| 4899 | S5_116138247 | 5 | 116138247 | 5 | T | C | T | 0.91935 | C | 0.08065 | 3 | 0.09677 |
| 4900 | S5_116138259 | 5 | 116138259 | 12 | C | T | C | 0.91935 | T | 0.08065 | 3 | 0.09677 |
| 4901 | S5_116138274 | 5 | 116138274 | 15 | G | T | G | 0.59677 | T | 0.40323 | 17 | 0.54839 |
| 4902 | S5_116138287 | 5 | 116138287 | 13 | G | A | A | 0.5 | G | 0.5 | 17 | 0.54839 |
| 4903 | S5_116261969 | 5 | 116261969 | 123682 | T | C | C | 0.91935 | T | 0.08065 | 5 | 0.16129 |
| 4904 | S5_116356031 | 5 | 116356031 | 94062 | T | C | C | 0.58065 | T | 0.41935 | 14 | 0.45161 |
| 4905 | S5_116356047 | 5 | 116356047 | 16 | C | T | C | 0.95161 | T | 0.04839 | 3 | 0.09677 |
| 4906 | S5_116356198 | 5 | 116356198 | 151 | G | C | G | 0.77419 | C | 0.22581 | 8 | 0.25806 |
| 4907 | S5_116356240 | 5 | 116356240 | 42 | C | T | C | 0.74194 | T | 0.25806 | 14 | 0.45161 |
| 4908 | S5_116529058 | 5 | 116529058 | 172818 | T | C | T | 0.93548 | C | 0.06452 | 4 | 0.12903 |
| 4909 | S5_116529121 | 5 | 116529121 | 63 | C | G | C | 0.93548 | G | 0.06452 | 4 | 0.12903 |
| 4910 | S5_116529171 | 5 | 116529171 | 50 | G | A | G | 0.93548 | A | 0.06452 | 4 | 0.12903 |
| 4911 | S5_116661649 | 5 | 116661649 | 132478 | G | T | T | 0.66129 | G | 0.33871 | 17 | 0.54839 |
| 4912 | S5_116661814 | 5 | 116661814 | 165 | G | A | A | 0.66129 | G | 0.33871 | 17 | 0.54839 |

| Site number | SNP name | Chromosome | Physical position | Physical distance from the previous SNP | Reference allele | Alternative allele | Major allele | Major allele frequency | Minor allele | Minor allele frequency | Number heterozygous | Heterozygosity |
|---|---|---|---|---|---|---|---|---|---|---|---|---|
| 4913 | S5_116903618 | 5 | 116903618 | 241804 | G | A | G | 0.93548 | A | 0.06452 | 4 | 0.12903 |
| 4914 | S5_116903682 | 5 | 116903682 | 64 | A | G | G | 0.87097 | A | 0.12903 | 4 | 0.12903 |
| 4915 | S5_116903702 | 5 | 116903702 | 20 | C | G | G | 0.82258 | C | 0.17742 | 7 | 0.22581 |
| 4916 | S5_117027792 | 5 | 117027792 | 124090 | C | T | C | 0.74194 | T | 0.25806 | 12 | 0.3871 |
| 4917 | S5_117027871 | 5 | 117027871 | 79 | T | G | T | 0.80645 | G | 0.19355 | 10 | 0.32258 |
| 4918 | S5_117422341 | 5 | 117422341 | 394470 | C | T | C | 0.82258 | T | 0.17742 | 11 | 0.35484 |
| 4919 | S5_117422486 | 5 | 117422486 | 145 | G | A | G | 0.95161 | A | 0.04839 | 3 | 0.09677 |
| 4920 | S5_117474758 | 5 | 117474758 | 52272 | C | T | C | 0.77419 | T | 0.22581 | 8 | 0.25806 |
| 4921 | S5_117630947 | 5 | 117630947 | 156189 | T | C | C | 0.5 | T | 0.5 | 11 | 0.35484 |
| 4922 | S5_117631102 | 5 | 117631102 | 155 | A | G | G | 0.91935 | A | 0.08065 | 5 | 0.16129 |
| 4923 | S5_118106379 | 5 | 118106379 | 475277 | A | G | G | 0.8871 | A | 0.1129 | 7 | 0.22581 |
| 4924 | S5_118106473 | 5 | 118106473 | 94 | A | G | A | 0.85484 | G | 0.14516 | 7 | 0.22581 |
| 4925 | S5_118106504 | 5 | 118106504 | 31 | C | T | C | 0.93548 | T | 0.06452 | 4 | 0.12903 |
| 4926 | S5_118106563 | 5 | 118106563 | 59 | G | A | G | 0.95161 | A | 0.04839 | 3 | 0.09677 |
| 4927 | S6_1146904 | 6 | 1146904 | 0 | A | C | A | 0.64516 | C | 0.35484 | 14 | 0.45161 |
| 4928 | S6_1146914 | 6 | 1146914 | 10 | T | C | C | 0.5 | T | 0.5 | 13 | 0.41935 |
| 4929 | S6_1146922 | 6 | 1146922 | 8 | C | T | C | 0.77419 | T | 0.22581 | 14 | 0.45161 |
| 4930 | S6_1146962 | 6 | 1146962 | 40 | C | T | C | 0.90323 | T | 0.09677 | 6 | 0.19355 |
| 4931 | S6_1555208 | 6 | 1555208 | 408246 | G | A | A | 0.87097 | G | 0.12903 | 6 | 0.19355 |
| 4932 | S6_1555270 | 6 | 1555270 | 62 | C | A | C | 0.95161 | A | 0.04839 | 3 | 0.09677 |
| 4933 | S6_1555313 | 6 | 1555313 | 43 | A | T | T | 0.87097 | A | 0.12903 | 6 | 0.19355 |
| 4934 | S6_3424630 | 6 | 3424630 | 1869317 | T | C | T | 0.69355 | C | 0.30645 | 11 | 0.35484 |
| 4935 | S6_3424707 | 6 | 3424707 | 77 | G | A | G | 0.93548 | A | 0.06452 | 4 | 0.12903 |
| 4936 | S6_3424782 | 6 | 3424782 | 75 | C | T | C | 0.64516 | T | 0.35484 | 12 | 0.3871 |
| 4937 | S6_3424814 | 6 | 3424814 | 32 | C | T | T | 0.77419 | C | 0.22581 | 12 | 0.3871 |
| 4938 | S6_3938041 | 6 | 3938041 | 513227 | C | T | T | 0.64516 | C | 0.35484 | 10 | 0.32258 |
| 4939 | S6_3938123 | 6 | 3938123 | 82 | A | G | G | 0.80645 | A | 0.19355 | 6 | 0.19355 |
| 4940 | S6_3938137 | 6 | 3938137 | 14 | C | T | C | 0.54839 | T | 0.45161 | 10 | 0.32258 |
| 4941 | S6_3938218 | 6 | 3938218 | 81 | C | T | C | 0.82258 | T | 0.17742 | 7 | 0.22581 |
| 4942 | S6_3938231 | 6 | 3938231 | 13 | C | T | T | 0.87097 | C | 0.12903 | 6 | 0.19355 |
| 4943 | S6_3938247 | 6 | 3938247 | 16 | C | T | C | 0.85484 | T | 0.14516 | 7 | 0.22581 |
| 4944 | S6_4176338 | 6 | 4176338 | 238091 | C | T | C | 0.90323 | T | 0.09677 | 6 | 0.19355 |
| 4945 | S6_4348989 | 6 | 4348989 | 172651 | G | C | G | 0.77419 | C | 0.22581 | 8 | 0.25806 |
| 4946 | S6_4348997 | 6 | 4348997 | 8 | C | T | C | 0.80645 | T | 0.19355 | 8 | 0.25806 |
| 4947 | S6_4349157 | 6 | 4349157 | 160 | A | G | A | 0.66129 | G | 0.33871 | 15 | 0.48387 |
| 4948 | S6_4349183 | 6 | 4349183 | 26 | T | G | T | 0.91935 | G | 0.08065 | 5 | 0.16129 |
| 4949 | S6_4723318 | 6 | 4723318 | 374135 | C | T | T | 0.83871 | C | 0.16129 | 8 | 0.25806 |
| 4950 | S6_4723461 | 6 | 4723461 | 143 | C | T | C | 0.90323 | T | 0.09677 | 6 | 0.19355 |
| 4951 | S6_4782517 | 6 | 4782517 | 59056 | G | A | G | 0.82258 | A | 0.17742 | 9 | 0.29032 |
| 4952 | S6_4782527 | 6 | 4782527 | 10 | C | T | T | 0.95161 | C | 0.04839 | 3 | 0.09677 |
| 4953 | S6_5205596 | 6 | 5205596 | 423069 | C | T | C | 0.91935 | T | 0.08065 | 5 | 0.16129 |
| 4954 | S6_5237852 | 6 | 5237852 | 32256 | T | C | T | 0.53226 | C | 0.46774 | 13 | 0.41935 |
| 4955 | S6_5237886 | 6 | 5237886 | 34 | A | G | G | 0.95161 | A | 0.04839 | 3 | 0.09677 |
| 4956 | S6_5238011 | 6 | 5238011 | 125 | G | A | G | 0.95161 | A | 0.04839 | 3 | 0.09677 |
| 4957 | S6_5238057 | 6 | 5238057 | 46 | T | C | C | 0.95161 | T | 0.04839 | 3 | 0.09677 |
| 4958 | S6_5465902 | 6 | 5465902 | 227845 | C | T | C | 0.87097 | T | 0.12903 | 4 | 0.12903 |
| 4959 | S6_5465972 | 6 | 5465972 | 70 | G | A | G | 0.93548 | A | 0.06452 | 4 | 0.12903 |
| 4960 | S6_5466041 | 6 | 5466041 | 69 | A | G | G | 0.83871 | A | 0.16129 | 6 | 0.19355 |
| 4961 | S6_5556448 | 6 | 5556448 | 90407 | T | C | T | 0.91935 | C | 0.08065 | 5 | 0.16129 |
| 4962 | S6_6260592 | 6 | 6260592 | 704144 | G | A | A | 0.90323 | G | 0.09677 | 6 | 0.19355 |
| 4963 | S6_6309743 | 6 | 6309743 | 49151 | C | T | C | 0.91935 | T | 0.08065 | 5 | 0.16129 |
| 4964 | S6_6475241 | 6 | 6475241 | 165498 | C | T | C | 0.62903 | T | 0.37097 | 13 | 0.41935 |
| 4965 | S6_7011349 | 6 | 7011349 | 536108 | C | T | T | 0.51613 | C | 0.48387 | 18 | 0.58065 |
| 4966 | S6_7011411 | 6 | 7011411 | 62 | G | T | G | 0.95161 | T | 0.04839 | 3 | 0.09677 |

| Site number | SNP name | Chromosome | Physical position | Physical distance from the previous SNP | Reference allele | Alternative allele | Major allele | Major allele frequency | Minor allele | Minor allele frequency | Number heterozygous | Heterozygosity |
|---|---|---|---|---|---|---|---|---|---|---|---|---|
| 4967 | S6_7587556 | 6 | 7587556 | 576145 | G | A | A | 0.56452 | G | 0.43548 | 11 | 0.35484 |
| 4968 | S6_7946369 | 6 | 7946369 | 358813 | C | T | C | 0.87097 | T | 0.12903 | 8 | 0.25806 |
| 4969 | S6_8155801 | 6 | 8155801 | 209432 | T | G | T | 0.82258 | G | 0.17742 | 9 | 0.29032 |
| 4970 | S6_8155868 | 6 | 8155868 | 67 | A | G | G | 0.74194 | A | 0.25806 | 10 | 0.32258 |
| 4971 | S6_8155905 | 6 | 8155905 | 37 | A | C | A | 0.82258 | C | 0.17742 | 9 | 0.29032 |
| 4972 | S6_8155988 | 6 | 8155988 | 83 | T | C | C | 0.8871 | T | 0.1129 | 7 | 0.22581 |
| 4973 | S6_9263396 | 6 | 9263396 | 1107408 | G | T | G | 0.69355 | T | 0.30645 | 11 | 0.35484 |
| 4974 | S6_9263413 | 6 | 9263413 | 17 | T | C | T | 0.70968 | C | 0.29032 | 12 | 0.3871 |
| 4975 | S6_9263460 | 6 | 9263460 | 47 | T | C | C | 0.59677 | T | 0.40323 | 17 | 0.54839 |
| 4976 | S6_9263498 | 6 | 9263498 | 38 | A | C | C | 0.59677 | A | 0.40323 | 17 | 0.54839 |
| 4977 | S6_9263559 | 6 | 9263559 | 61 | C | G | G | 0.59677 | C | 0.40323 | 17 | 0.54839 |
| 4978 | S6_9281027 | 6 | 9281027 | 17468 | G | A | G | 0.69355 | A | 0.30645 | 13 | 0.41935 |
| 4979 | S6_9281061 | 6 | 9281061 | 34 | A | C | A | 0.70968 | C | 0.29032 | 14 | 0.45161 |
| 4980 | S6_9325250 | 6 | 9325250 | 44189 | G | A | G | 0.75806 | A | 0.24194 | 11 | 0.35484 |
| 4981 | S6_9325260 | 6 | 9325260 | 10 | T | C | T | 0.66129 | C | 0.33871 | 15 | 0.48387 |
| 4982 | S6_9325388 | 6 | 9325388 | 128 | T | G | G | 0.80645 | T | 0.19355 | 8 | 0.25806 |
| 4983 | S6_9325473 | 6 | 9325473 | 85 | G | C | G | 0.72581 | C | 0.27419 | 13 | 0.41935 |
| 4984 | S6_10790302 | 6 | 10790302 | 1464829 | G | A | A | 0.56452 | G | 0.43548 | 15 | 0.48387 |
| 4985 | S6_11285496 | 6 | 11285496 | 495194 | T | A | T | 0.77419 | A | 0.22581 | 12 | 0.3871 |
| 4986 | S6_11285704 | 6 | 11285704 | 208 | G | T | T | 0.95161 | G | 0.04839 | 3 | 0.09677 |
| 4987 | S6_11312075 | 6 | 11312075 | 26371 | A | G | A | 0.8871 | G | 0.1129 | 5 | 0.16129 |
| 4988 | S6_11654367 | 6 | 11654367 | 342292 | C | T | C | 0.82258 | T | 0.17742 | 9 | 0.29032 |
| 4989 | S6_11654439 | 6 | 11654439 | 72 | G | C | G | 0.62903 | C | 0.37097 | 13 | 0.41935 |
| 4990 | S6_11654519 | 6 | 11654519 | 80 | T | G | T | 0.83871 | G | 0.16129 | 6 | 0.19355 |
| 4991 | S6_11654524 | 6 | 11654524 | 5 | C | T | C | 0.91935 | T | 0.08065 | 5 | 0.16129 |
| 4992 | S6_11742219 | 6 | 11742219 | 87695 | C | T | T | 0.58065 | C | 0.41935 | 20 | 0.64516 |
| 4993 | S6_11742230 | 6 | 11742230 | 11 | A | C | A | 0.87097 | C | 0.12903 | 8 | 0.25806 |
| 4994 | S6_11742260 | 6 | 11742260 | 30 | A | G | A | 0.87097 | G | 0.12903 | 8 | 0.25806 |
| 4995 | S6_11742307 | 6 | 11742307 | 47 | C | G | C | 0.79032 | G | 0.20968 | 9 | 0.29032 |
| 4996 | S6_11742397 | 6 | 11742397 | 90 | C | A | C | 0.77419 | A | 0.22581 | 10 | 0.32258 |
| 4997 | S6_12291325 | 6 | 12291325 | 548928 | C | T | C | 0.83871 | T | 0.16129 | 8 | 0.25806 |
| 4998 | S6_12309564 | 6 | 12309564 | 18239 | G | A | G | 0.74194 | A | 0.25806 | 16 | 0.51613 |
| 4999 | S6_12309719 | 6 | 12309719 | 155 | G | T | G | 0.74194 | T | 0.25806 | 16 | 0.51613 |
| 5000 | S6_12806636 | 6 | 12806636 | 496917 | A | T | A | 0.77419 | T | 0.22581 | 10 | 0.32258 |
| 5001 | S6_13450200 | 6 | 13450200 | 643564 | A | G | G | 0.90323 | A | 0.09677 | 4 | 0.12903 |
| 5002 | S6_14105232 | 6 | 14105232 | 655032 | A | G | A | 0.93548 | G | 0.06452 | 4 | 0.12903 |
| 5003 | S6_14105248 | 6 | 14105248 | 16 | G | C | C | 0.51613 | G | 0.48387 | 18 | 0.58065 |
| 5004 | S6_14709862 | 6 | 14709862 | 604614 | G | A | A | 0.59677 | G | 0.40323 | 15 | 0.48387 |
| 5005 | S6_14709867 | 6 | 14709867 | 5 | T | C | C | 0.59677 | T | 0.40323 | 15 | 0.48387 |
| 5006 | S6_14709901 | 6 | 14709901 | 34 | T | G | T | 0.74194 | G | 0.25806 | 12 | 0.3871 |
| 5007 | S6_14709941 | 6 | 14709941 | 40 | C | A | C | 0.79032 | A | 0.20968 | 11 | 0.35484 |
| 5008 | S6_14709950 | 6 | 14709950 | 9 | G | A | G | 0.82258 | A | 0.17742 | 9 | 0.29032 |
| 5009 | S6_14710020 | 6 | 14710020 | 70 | C | A | C | 0.67742 | A | 0.32258 | 12 | 0.3871 |
| 5010 | S6_15238049 | 6 | 15238049 | 528029 | T | C | T | 0.95161 | C | 0.04839 | 3 | 0.09677 |
| 5011 | S6_15238063 | 6 | 15238063 | 14 | G | A | G | 0.95161 | A | 0.04839 | 3 | 0.09677 |
| 5012 | S6_15238174 | 6 | 15238174 | 111 | G | C | G | 0.90323 | C | 0.09677 | 4 | 0.12903 |
| 5013 | S6_15238193 | 6 | 15238193 | 19 | T | G | T | 0.87097 | G | 0.12903 | 8 | 0.25806 |
| 5014 | S6_15238198 | 6 | 15238198 | 5 | A | G | A | 0.62903 | G | 0.37097 | 15 | 0.48387 |
| 5015 | S6_15238257 | 6 | 15238257 | 59 | A | G | A | 0.87097 | G | 0.12903 | 8 | 0.25806 |
| 5016 | S6_15329724 | 6 | 15329724 | 91467 | A | G | G | 0.85484 | A | 0.14516 | 9 | 0.29032 |
| 5017 | S6_15329852 | 6 | 15329852 | 128 | G | A | G | 0.90323 | A | 0.09677 | 6 | 0.19355 |
| 5018 | S6_15417800 | 6 | 15417800 | 87948 | G | A | G | 0.93548 | A | 0.06452 | 4 | 0.12903 |
| 5019 | S6_15417981 | 6 | 15417981 | 181 | G | A | G | 0.95161 | A | 0.04839 | 3 | 0.09677 |
| 5020 | S6_16068547 | 6 | 16068547 | 650566 | G | A | G | 0.91935 | A | 0.08065 | 5 | 0.16129 |

| Site number | SNP name | Chromosome | Physical position | Physical distance from the previous SNP | Reference allele | Alternative allele | Major allele | Major allele frequency | Minor allele | Minor allele frequency | Number heterozygous | Heterozygosity |
|---|---|---|---|---|---|---|---|---|---|---|---|---|
| 5021 | S6_16068548 | 6 | 16068548 | 1 | C | G | C | 0.91935 | G | 0.08065 | 5 | 0.16129 |
| 5022 | S6_16068558 | 6 | 16068558 | 10 | C | T | C | 0.91935 | T | 0.08065 | 5 | 0.16129 |
| 5023 | S6_16068725 | 6 | 16068725 | 167 | T | C | C | 0.80645 | T | 0.19355 | 10 | 0.32258 |
| 5024 | S6_16068790 | 6 | 16068790 | 65 | A | G | A | 0.91935 | G | 0.08065 | 5 | 0.16129 |
| 5025 | S6_16360718 | 6 | 16360718 | 291928 | A | G | A | 0.56452 | G | 0.43548 | 15 | 0.48387 |
| 5026 | S6_16360784 | 6 | 16360784 | 66 | A | G | A | 0.93548 | G | 0.06452 | 4 | 0.12903 |
| 5027 | S6_16710810 | 6 | 16710810 | 350026 | G | A | G | 0.93548 | A | 0.06452 | 4 | 0.12903 |
| 5028 | S6_16710911 | 6 | 16710911 | 101 | A | G | A | 0.91935 | G | 0.08065 | 5 | 0.16129 |
| 5029 | S6_17039031 | 6 | 17039031 | 328120 | G | A | G | 0.91935 | A | 0.08065 | 5 | 0.16129 |
| 5030 | S6_17039103 | 6 | 17039103 | 72 | C | T | C | 0.93548 | T | 0.06452 | 4 | 0.12903 |
| 5031 | S6_17039168 | 6 | 17039168 | 65 | G | T | G | 0.93548 | T | 0.06452 | 4 | 0.12903 |
| 5032 | S6_17039181 | 6 | 17039181 | 13 | G | A | G | 0.93548 | A | 0.06452 | 4 | 0.12903 |
| 5033 | S6_17039202 | 6 | 17039202 | 21 | T | C | C | 0.8871 | T | 0.1129 | 5 | 0.16129 |
| 5034 | S6_17732145 | 6 | 17732145 | 692943 | C | T | C | 0.90323 | T | 0.09677 | 6 | 0.19355 |
| 5035 | S6_17732192 | 6 | 17732192 | 47 | G | A | G | 0.87097 | A | 0.12903 | 8 | 0.25806 |
| 5036 | S6_17815477 | 6 | 17815477 | 83285 | G | T | G | 0.91935 | T | 0.08065 | 5 | 0.16129 |
| 5037 | S6_17815558 | 6 | 17815558 | 81 | T | C | C | 0.8871 | T | 0.1129 | 7 | 0.22581 |
| 5038 | S6_17815692 | 6 | 17815692 | 134 | T | C | T | 0.79032 | C | 0.20968 | 9 | 0.29032 |
| 5039 | S6_17885196 | 6 | 17885196 | 69504 | C | G | C | 0.91935 | G | 0.08065 | 5 | 0.16129 |
| 5040 | S6_17885241 | 6 | 17885241 | 45 | G | A | G | 0.87097 | A | 0.12903 | 6 | 0.19355 |
| 5041 | S6_17903482 | 6 | 17903482 | 18241 | A | G | A | 0.6129 | G | 0.3871 | 12 | 0.3871 |
| 5042 | S6_17903500 | 6 | 17903500 | 18 | C | T | C | 0.66129 | T | 0.33871 | 13 | 0.41935 |
| 5043 | S6_17903559 | 6 | 17903559 | 59 | A | C | A | 0.6129 | C | 0.3871 | 12 | 0.3871 |
| 5044 | S6_17903630 | 6 | 17903630 | 71 | A | G | A | 0.6129 | G | 0.3871 | 12 | 0.3871 |
| 5045 | S6_17926018 | 6 | 17926018 | 22388 | T | C | C | 0.54839 | T | 0.45161 | 16 | 0.51613 |
| 5046 | S6_17926192 | 6 | 17926192 | 174 | A | T | A | 0.54839 | T | 0.45161 | 14 | 0.45161 |
| 5047 | S6_17988318 | 6 | 17988318 | 62126 | A | G | A | 0.90323 | G | 0.09677 | 6 | 0.19355 |
| 5048 | S6_18109014 | 6 | 18109014 | 120696 | C | G | C | 0.85484 | G | 0.14516 | 9 | 0.29032 |
| 5049 | S6_18109044 | 6 | 18109044 | 30 | A | G | A | 0.5 | G | 0.5 | 17 | 0.54839 |
| 5050 | S6_18109103 | 6 | 18109103 | 59 | G | A | A | 0.58065 | G | 0.41935 | 14 | 0.45161 |
| 5051 | S6_18109112 | 6 | 18109112 | 9 | A | G | G | 0.58065 | A | 0.41935 | 14 | 0.45161 |
| 5052 | S6_18109134 | 6 | 18109134 | 22 | A | G | A | 0.83871 | G | 0.16129 | 8 | 0.25806 |
| 5053 | S6_18109200 | 6 | 18109200 | 66 | G | T | G | 0.90323 | T | 0.09677 | 6 | 0.19355 |
| 5054 | S6_18109225 | 6 | 18109225 | 25 | A | G | A | 0.90323 | G | 0.09677 | 6 | 0.19355 |
| 5055 | S6_18295860 | 6 | 18295860 | 186635 | T | C | T | 0.91935 | C | 0.08065 | 5 | 0.16129 |
| 5056 | S6_18835655 | 6 | 18835655 | 539795 | C | T | T | 0.69355 | C | 0.30645 | 13 | 0.41935 |
| 5057 | S6_18835656 | 6 | 18835656 | 1 | T | G | G | 0.70968 | T | 0.29032 | 12 | 0.3871 |
| 5058 | S6_19668602 | 6 | 19668602 | 832946 | T | A | T | 0.83871 | A | 0.16129 | 10 | 0.32258 |
| 5059 | S6_19926100 | 6 | 19926100 | 257498 | C | T | C | 0.90323 | T | 0.09677 | 4 | 0.12903 |
| 5060 | S6_19926203 | 6 | 19926203 | 103 | G | A | G | 0.51613 | A | 0.48387 | 18 | 0.58065 |
| 5061 | S6_19926215 | 6 | 19926215 | 12 | G | C | C | 0.62903 | G | 0.37097 | 15 | 0.48387 |
| 5062 | S6_19926223 | 6 | 19926223 | 8 | C | G | G | 0.59677 | C | 0.40323 | 13 | 0.41935 |
| 5063 | S6_19926271 | 6 | 19926271 | 48 | G | A | G | 0.93548 | A | 0.06452 | 4 | 0.12903 |
| 5064 | S6_19926307 | 6 | 19926307 | 36 | A | G | A | 0.56452 | G | 0.43548 | 19 | 0.6129 |
| 5065 | S6_20166807 | 6 | 20166807 | 240500 | C | A | C | 0.93548 | A | 0.06452 | 4 | 0.12903 |
| 5066 | S6_20166816 | 6 | 20166816 | 9 | G | A | G | 0.93548 | A | 0.06452 | 4 | 0.12903 |
| 5067 | S6_20540955 | 6 | 20540955 | 374139 | G | A | G | 0.93548 | A | 0.06452 | 4 | 0.12903 |
| 5068 | S6_20540960 | 6 | 20540960 | 5 | C | T | C | 0.95161 | T | 0.04839 | 3 | 0.09677 |
| 5069 | S6_20995835 | 6 | 20995835 | 454875 | A | G | A | 0.77419 | G | 0.22581 | 8 | 0.25806 |
| 5070 | S6_20995855 | 6 | 20995855 | 20 | G | A | G | 0.75806 | A | 0.24194 | 9 | 0.29032 |
| 5071 | S6_21318853 | 6 | 21318853 | 322998 | A | G | A | 0.90323 | G | 0.09677 | 6 | 0.19355 |
| 5072 | S6_21318964 | 6 | 21318964 | 111 | C | A | C | 0.8871 | A | 0.1129 | 7 | 0.22581 |
| 5073 | S6_21318981 | 6 | 21318981 | 17 | A | T | A | 0.8871 | T | 0.1129 | 7 | 0.22581 |
| 5074 | S6_21362246 | 6 | 21362246 | 43265 | C | T | C | 0.85484 | T | 0.14516 | 7 | 0.22581 |

| Site number | SNP name | Chromosome | Physical position | Physical distance from the previous SNP | Reference allele | Alternative allele | Major allele | Major allele frequency | Minor allele | Minor allele frequency | Number heterozygous | Heterozygosity |
|---|---|---|---|---|---|---|---|---|---|---|---|---|
| 5075 | S6_21362272 | 6 | 21362272 | 26 | A | T | A | 0.85484 | T | 0.14516 | 7 | 0.22581 |
| 5076 | S6_21362290 | 6 | 21362290 | 18 | G | A | G | 0.85484 | A | 0.14516 | 7 | 0.22581 |
| 5077 | S6_21362429 | 6 | 21362429 | 139 | C | T | C | 0.85484 | T | 0.14516 | 7 | 0.22581 |
| 5078 | S6_21925649 | 6 | 21925649 | 563220 | T | G | T | 0.77419 | G | 0.22581 | 12 | 0.3871 |
| 5079 | S6_21925674 | 6 | 21925674 | 25 | G | C | G | 0.77419 | C | 0.22581 | 12 | 0.3871 |
| 5080 | S6_21925692 | 6 | 21925692 | 18 | G | C | G | 0.77419 | C | 0.22581 | 12 | 0.3871 |
| 5081 | S6_21925727 | 6 | 21925727 | 35 | G | A | G | 0.77419 | A | 0.22581 | 12 | 0.3871 |
| 5082 | S6_21925765 | 6 | 21925765 | 38 | A | G | A | 0.77419 | G | 0.22581 | 12 | 0.3871 |
| 5083 | S6_21925768 | 6 | 21925768 | 3 | T | C | T | 0.77419 | C | 0.22581 | 12 | 0.3871 |
| 5084 | S6_22248195 | 6 | 22248195 | 322427 | T | C | T | 0.77419 | C | 0.22581 | 12 | 0.3871 |
| 5085 | S6_23168231 | 6 | 23168231 | 920036 | C | G | C | 0.6129 | G | 0.3871 | 12 | 0.3871 |
| 5086 | S6_23168253 | 6 | 23168253 | 22 | C | T | C | 0.85484 | T | 0.14516 | 7 | 0.22581 |
| 5087 | S6_23168346 | 6 | 23168346 | 93 | G | A | G | 0.85484 | A | 0.14516 | 7 | 0.22581 |
| 5088 | S6_23175958 | 6 | 23175958 | 7612 | A | G | A | 0.56452 | G | 0.43548 | 17 | 0.54839 |
| 5089 | S6_23175959 | 6 | 23175959 | 1 | G | C | G | 0.56452 | C | 0.43548 | 17 | 0.54839 |
| 5090 | S6_23380487 | 6 | 23380487 | 204528 | G | A | G | 0.91935 | A | 0.08065 | 5 | 0.16129 |
| 5091 | S6_23380577 | 6 | 23380577 | 90 | A | G | A | 0.6129 | G | 0.3871 | 18 | 0.58065 |
| 5092 | S6_23380641 | 6 | 23380641 | 64 | C | T | C | 0.80645 | T | 0.19355 | 12 | 0.3871 |
| 5093 | S6_23380655 | 6 | 23380655 | 14 | C | G | C | 0.80645 | G | 0.19355 | 12 | 0.3871 |
| 5094 | S6_23380668 | 6 | 23380668 | 13 | T | C | T | 0.6129 | C | 0.3871 | 18 | 0.58065 |
| 5095 | S6_23753802 | 6 | 23753802 | 373134 | G | T | G | 0.91935 | T | 0.08065 | 5 | 0.16129 |
| 5096 | S6_23753808 | 6 | 23753808 | 6 | A | G | G | 0.58065 | A | 0.41935 | 18 | 0.58065 |
| 5097 | S6_23874150 | 6 | 23874150 | 120342 | A | G | A | 0.83871 | G | 0.16129 | 8 | 0.25806 |
| 5098 | S6_23874270 | 6 | 23874270 | 120 | T | A | T | 0.8871 | A | 0.1129 | 7 | 0.22581 |
| 5099 | S6_23874276 | 6 | 23874276 | 6 | A | G | A | 0.8871 | G | 0.1129 | 7 | 0.22581 |
| 5100 | S6_23874322 | 6 | 23874322 | 46 | C | T | C | 0.8871 | T | 0.1129 | 7 | 0.22581 |
| 5101 | S6_24553700 | 6 | 24553700 | 679378 | G | T | G | 0.95161 | T | 0.04839 | 3 | 0.09677 |
| 5102 | S6_24553934 | 6 | 24553934 | 234 | A | G | A | 0.95161 | G | 0.04839 | 3 | 0.09677 |
| 5103 | S6_24694166 | 6 | 24694166 | 140232 | G | A | G | 0.91935 | A | 0.08065 | 5 | 0.16129 |
| 5104 | S6_24694196 | 6 | 24694196 | 30 | G | A | G | 0.93548 | A | 0.06452 | 4 | 0.12903 |
| 5105 | S6_24857073 | 6 | 24857073 | 162877 | G | A | G | 0.95161 | A | 0.04839 | 3 | 0.09677 |
| 5106 | S6_24857104 | 6 | 24857104 | 31 | G | A | A | 0.66129 | G | 0.33871 | 15 | 0.48387 |
| 5107 | S6_24857168 | 6 | 24857168 | 64 | C | T | C | 0.87097 | T | 0.12903 | 8 | 0.25806 |
| 5108 | S6_24857223 | 6 | 24857223 | 55 | T | C | C | 0.79032 | T | 0.20968 | 11 | 0.35484 |
| 5109 | S6_25100509 | 6 | 25100509 | 243286 | A | G | A | 0.53226 | G | 0.46774 | 15 | 0.48387 |
| 5110 | S6_25504812 | 6 | 25504812 | 404303 | G | A | G | 0.93548 | A | 0.06452 | 4 | 0.12903 |
| 5111 | S6_25505009 | 6 | 25505009 | 197 | T | C | C | 0.79032 | T | 0.20968 | 9 | 0.29032 |
| 5112 | S6_25556721 | 6 | 25556721 | 51712 | G | A | G | 0.91935 | A | 0.08065 | 5 | 0.16129 |
| 5113 | S6_25556820 | 6 | 25556820 | 99 | T | C | T | 0.8871 | C | 0.1129 | 7 | 0.22581 |
| 5114 | S6_26031099 | 6 | 26031099 | 474279 | C | G | G | 0.66129 | C | 0.33871 | 13 | 0.41935 |
| 5115 | S6_26031147 | 6 | 26031147 | 48 | C | T | T | 0.95161 | C | 0.04839 | 3 | 0.09677 |
| 5116 | S6_26106792 | 6 | 26106792 | 75645 | A | G | A | 0.91935 | G | 0.08065 | 5 | 0.16129 |
| 5117 | S6_26106901 | 6 | 26106901 | 109 | G | A | G | 0.91935 | A | 0.08065 | 5 | 0.16129 |
| 5118 | S6_26235800 | 6 | 26235800 | 128899 | G | T | G | 0.8871 | T | 0.1129 | 7 | 0.22581 |
| 5119 | S6_26235988 | 6 | 26235988 | 188 | G | A | G | 0.8871 | A | 0.1129 | 7 | 0.22581 |
| 5120 | S6_26402085 | 6 | 26402085 | 166097 | G | A | G | 0.72581 | A | 0.27419 | 13 | 0.41935 |
| 5121 | S6_26402091 | 6 | 26402091 | 6 | T | G | T | 0.62903 | G | 0.37097 | 17 | 0.54839 |
| 5122 | S6_26773435 | 6 | 26773435 | 371344 | C | T | C | 0.93548 | T | 0.06452 | 4 | 0.12903 |
| 5123 | S6_27051920 | 6 | 27051920 | 278485 | C | T | T | 0.64516 | C | 0.35484 | 18 | 0.58065 |
| 5124 | S6_27179871 | 6 | 27179871 | 127951 | T | C | T | 0.91935 | C | 0.08065 | 5 | 0.16129 |
| 5125 | S6_27179917 | 6 | 27179917 | 46 | C | A | C | 0.91935 | A | 0.08065 | 5 | 0.16129 |
| 5126 | S6_27753640 | 6 | 27753640 | 573723 | C | T | C | 0.74194 | T | 0.25806 | 10 | 0.32258 |
| 5127 | S6_28345349 | 6 | 28345349 | 591709 | T | A | A | 0.80645 | T | 0.19355 | 10 | 0.32258 |
| 5128 | S6_28345462 | 6 | 28345462 | 113 | A | T | T | 0.87097 | A | 0.12903 | 6 | 0.19355 |

| Site number | SNP name | Chromosome | Physical position | Physical distance from the previous SNP | Reference allele | Alternative allele | Major allele | Major allele frequency | Minor allele | Minor allele frequency | Number heterozygous | Heterozygosity |
|---|---|---|---|---|---|---|---|---|---|---|---|---|
| 5129 | S6_28742351 | 6 | 28742351 | 396889 | C | T | C | 0.75806 | T | 0.24194 | 13 | 0.41935 |
| 5130 | S6_29742007 | 6 | 29742007 | 999656 | G | A | G | 0.74194 | A | 0.25806 | 8 | 0.25806 |
| 5131 | S6_30463709 | 6 | 30463709 | 721702 | C | T | C | 0.91935 | T | 0.08065 | 5 | 0.16129 |
| 5132 | S6_30463864 | 6 | 30463864 | 155 | G | C | C | 0.66129 | G | 0.33871 | 17 | 0.54839 |
| 5133 | S6_30545637 | 6 | 30545637 | 81773 | G | T | G | 0.80645 | T | 0.19355 | 8 | 0.25806 |
| 5134 | S6_30545729 | 6 | 30545729 | 92 | C | T | C | 0.91935 | T | 0.08065 | 5 | 0.16129 |
| 5135 | S6_30545772 | 6 | 30545772 | 43 | C | G | C | 0.67742 | G | 0.32258 | 10 | 0.32258 |
| 5136 | S6_30655419 | 6 | 30655419 | 109647 | T | C | T | 0.83871 | C | 0.16129 | 8 | 0.25806 |
| 5137 | S6_30717409 | 6 | 30717409 | 61990 | G | A | G | 0.93548 | A | 0.06452 | 4 | 0.12903 |
| 5138 | S6_30717464 | 6 | 30717464 | 55 | C | T | C | 0.91935 | T | 0.08065 | 5 | 0.16129 |
| 5139 | S6_30717496 | 6 | 30717496 | 32 | A | G | A | 0.93548 | G | 0.06452 | 4 | 0.12903 |
| 5140 | S6_30717579 | 6 | 30717579 | 83 | A | G | A | 0.6129 | G | 0.3871 | 16 | 0.51613 |
| 5141 | S6_31032420 | 6 | 31032420 | 314841 | C | T | C | 0.83871 | T | 0.16129 | 6 | 0.19355 |
| 5142 | S6_32623758 | 6 | 32623758 | 1591338 | T | C | T | 0.95161 | C | 0.04839 | 1 | 0.03226 |
| 5143 | S6_33993780 | 6 | 33993780 | 1370022 | G | T | T | 0.56452 | G | 0.43548 | 19 | 0.6129 |
| 5144 | S6_33993844 | 6 | 33993844 | 64 | G | A | G | 0.8871 | A | 0.1129 | 7 | 0.22581 |
| 5145 | S6_34207578 | 6 | 34207578 | 213734 | C | T | C | 0.95161 | T | 0.04839 | 3 | 0.09677 |
| 5146 | S6_34442490 | 6 | 34442490 | 234912 | C | T | C | 0.90323 | T | 0.09677 | 6 | 0.19355 |
| 5147 | S6_35492144 | 6 | 35492144 | 1049654 | G | A | G | 0.85484 | A | 0.14516 | 9 | 0.29032 |
| 5148 | S6_35492215 | 6 | 35492215 | 71 | C | T | T | 0.53226 | C | 0.46774 | 17 | 0.54839 |
| 5149 | S6_35609453 | 6 | 35609453 | 117238 | A | G | A | 0.95161 | G | 0.04839 | 3 | 0.09677 |
| 5150 | S6_35859527 | 6 | 35859527 | 250074 | T | C | T | 0.85484 | C | 0.14516 | 9 | 0.29032 |
| 5151 | S6_35859556 | 6 | 35859556 | 29 | C | T | C | 0.79032 | T | 0.20968 | 11 | 0.35484 |
| 5152 | S6_35859594 | 6 | 35859594 | 38 | G | C | G | 0.93548 | C | 0.06452 | 4 | 0.12903 |
| 5153 | S6_35859745 | 6 | 35859745 | 151 | T | C | T | 0.79032 | C | 0.20968 | 11 | 0.35484 |
| 5154 | S6_35910908 | 6 | 35910908 | 51163 | T | G | T | 0.59677 | G | 0.40323 | 17 | 0.54839 |
| 5155 | S6_35911012 | 6 | 35911012 | 104 | G | A | G | 0.87097 | A | 0.12903 | 8 | 0.25806 |
| 5156 | S6_35937863 | 6 | 35937863 | 26851 | A | T | T | 0.53226 | A | 0.46774 | 17 | 0.54839 |
| 5157 | S6_35967976 | 6 | 35967976 | 30113 | C | A | C | 0.95161 | A | 0.04839 | 3 | 0.09677 |
| 5158 | S6_35968198 | 6 | 35968198 | 222 | A | G | G | 0.79032 | A | 0.20968 | 11 | 0.35484 |
| 5159 | S6_36267836 | 6 | 36267836 | 299638 | C | G | G | 0.59677 | C | 0.40323 | 15 | 0.48387 |
| 5160 | S6_36490627 | 6 | 36490627 | 222791 | G | A | G | 0.93548 | A | 0.06452 | 4 | 0.12903 |
| 5161 | S6_36524823 | 6 | 36524823 | 34196 | G | A | G | 0.79032 | A | 0.20968 | 11 | 0.35484 |
| 5162 | S6_36597668 | 6 | 36597668 | 72845 | T | C | T | 0.69355 | C | 0.30645 | 13 | 0.41935 |
| 5163 | S6_36597826 | 6 | 36597826 | 158 | T | C | T | 0.6129 | C | 0.3871 | 16 | 0.51613 |
| 5164 | S6_36650684 | 6 | 36650684 | 52858 | T | C | T | 0.91935 | C | 0.08065 | 5 | 0.16129 |
| 5165 | S6_36650695 | 6 | 36650695 | 11 | G | A | G | 0.90323 | A | 0.09677 | 6 | 0.19355 |
| 5166 | S6_36692867 | 6 | 36692867 | 42172 | T | A | T | 0.82258 | A | 0.17742 | 9 | 0.29032 |
| 5167 | S6_36842423 | 6 | 36842423 | 149556 | C | T | C | 0.77419 | T | 0.22581 | 10 | 0.32258 |
| 5168 | S6_37416051 | 6 | 37416051 | 573628 | G | A | G | 0.93548 | A | 0.06452 | 4 | 0.12903 |
| 5169 | S6_37416055 | 6 | 37416055 | 4 | G | A | G | 0.59677 | A | 0.40323 | 15 | 0.48387 |
| 5170 | S6_37819437 | 6 | 37819437 | 403382 | A | G | G | 0.95161 | A | 0.04839 | 3 | 0.09677 |
| 5171 | S6_38669148 | 6 | 38669148 | 849711 | T | C | C | 0.83871 | T | 0.16129 | 10 | 0.32258 |
| 5172 | S6_38669156 | 6 | 38669156 | 8 | C | T | C | 0.58065 | T | 0.41935 | 16 | 0.51613 |
| 5173 | S6_42339490 | 6 | 42339490 | 3670334 | A | G | A | 0.83871 | G | 0.16129 | 6 | 0.19355 |
| 5174 | S6_42339683 | 6 | 42339683 | 193 | T | C | T | 0.85484 | C | 0.14516 | 5 | 0.16129 |
| 5175 | S6_42339697 | 6 | 42339697 | 14 | T | C | T | 0.85484 | C | 0.14516 | 5 | 0.16129 |
| 5176 | S6_42524861 | 6 | 42524861 | 185164 | G | T | G | 0.72581 | T | 0.27419 | 11 | 0.35484 |
| 5177 | S6_42524867 | 6 | 42524867 | 6 | C | A | A | 0.82258 | C | 0.17742 | 7 | 0.22581 |
| 5178 | S6_42524982 | 6 | 42524982 | 115 | G | C | G | 0.85484 | C | 0.14516 | 7 | 0.22581 |
| 5179 | S6_42524985 | 6 | 42524985 | 3 | G | A | G | 0.85484 | A | 0.14516 | 7 | 0.22581 |
| 5180 | S6_42552251 | 6 | 42552251 | 27266 | C | T | C | 0.77419 | T | 0.22581 | 10 | 0.32258 |
| 5181 | S6_42552260 | 6 | 42552260 | 9 | A | G | G | 0.93548 | A | 0.06452 | 4 | 0.12903 |
| 5182 | S6_42552315 | 6 | 42552315 | 55 | C | T | C | 0.90323 | T | 0.09677 | 6 | 0.19355 |

| Site number | SNP name | Chromosome | Physical position | Physical distance from the previous SNP | Reference allele | Alternative allele | Major allele | Major allele frequency | Minor allele | Minor allele frequency | Number heterozygous | Heterozygosity |
|---|---|---|---|---|---|---|---|---|---|---|---|---|
| 5183 | S6_42702717 | 6 | 42702717 | 150402 | T | C | T | 0.91935 | C | 0.08065 | 5 | 0.16129 |
| 5184 | S6_42757963 | 6 | 42757963 | 55246 | G | C | G | 0.72581 | C | 0.27419 | 13 | 0.41935 |
| 5185 | S6_42758057 | 6 | 42758057 | 94 | A | G | A | 0.70968 | G | 0.29032 | 12 | 0.3871 |
| 5186 | S6_43401688 | 6 | 43401688 | 643631 | A | G | A | 0.93548 | G | 0.06452 | 4 | 0.12903 |
| 5187 | S6_43652448 | 6 | 43652448 | 250760 | C | T | C | 0.75806 | T | 0.24194 | 13 | 0.41935 |
| 5188 | S6_43704092 | 6 | 43704092 | 51644 | T | A | T | 0.93548 | A | 0.06452 | 4 | 0.12903 |
| 5189 | S6_44028167 | 6 | 44028167 | 324075 | T | C | T | 0.90323 | C | 0.09677 | 6 | 0.19355 |
| 5190 | S6_44028187 | 6 | 44028187 | 20 | G | A | G | 0.56452 | A | 0.43548 | 15 | 0.48387 |
| 5191 | S6_44028208 | 6 | 44028208 | 21 | G | A | G | 0.95161 | A | 0.04839 | 3 | 0.09677 |
| 5192 | S6_44050520 | 6 | 44050520 | 22312 | T | C | C | 0.53226 | T | 0.46774 | 11 | 0.35484 |
| 5193 | S6_44272316 | 6 | 44272316 | 221796 | A | C | A | 0.91935 | C | 0.08065 | 3 | 0.09677 |
| 5194 | S6_44272429 | 6 | 44272429 | 113 | A | G | G | 0.66129 | A | 0.33871 | 11 | 0.35484 |
| 5195 | S6_44272543 | 6 | 44272543 | 114 | G | T | G | 0.95161 | T | 0.04839 | 3 | 0.09677 |
| 5196 | S6_44272547 | 6 | 44272547 | 4 | C | T | T | 0.66129 | C | 0.33871 | 11 | 0.35484 |
| 5197 | S6_44719929 | 6 | 44719929 | 447382 | G | C | G | 0.79032 | C | 0.20968 | 7 | 0.22581 |
| 5198 | S6_44719968 | 6 | 44719968 | 39 | C | T | C | 0.87097 | T | 0.12903 | 8 | 0.25806 |
| 5199 | S6_44719969 | 6 | 44719969 | 1 | G | A | A | 0.59677 | G | 0.40323 | 17 | 0.54839 |
| 5200 | S6_44791330 | 6 | 44791330 | 71361 | C | T | C | 0.64516 | T | 0.35484 | 14 | 0.45161 |
| 5201 | S6_44791362 | 6 | 44791362 | 32 | C | A | C | 0.91935 | A | 0.08065 | 5 | 0.16129 |
| 5202 | S6_45041398 | 6 | 45041398 | 250036 | C | T | C | 0.77419 | T | 0.22581 | 8 | 0.25806 |
| 5203 | S6_45041460 | 6 | 45041460 | 62 | C | A | A | 0.59677 | C | 0.40323 | 13 | 0.41935 |
| 5204 | S6_45128745 | 6 | 45128745 | 87285 | G | C | C | 0.95161 | G | 0.04839 | 3 | 0.09677 |
| 5205 | S6_45851712 | 6 | 45851712 | 722967 | G | A | G | 0.79032 | A | 0.20968 | 7 | 0.22581 |
| 5206 | S6_45851732 | 6 | 45851732 | 20 | G | A | G | 0.79032 | A | 0.20968 | 7 | 0.22581 |
| 5207 | S6_45851771 | 6 | 45851771 | 39 | G | A | G | 0.79032 | A | 0.20968 | 11 | 0.35484 |
| 5208 | S6_45851831 | 6 | 45851831 | 60 | C | T | C | 0.77419 | T | 0.22581 | 12 | 0.3871 |
| 5209 | S6_46076256 | 6 | 46076256 | 224425 | T | C | T | 0.85484 | C | 0.14516 | 7 | 0.22581 |
| 5210 | S6_46088910 | 6 | 46088910 | 12654 | C | G | C | 0.93548 | G | 0.06452 | 2 | 0.06452 |
| 5211 | S6_47239582 | 6 | 47239582 | 1150672 | A | G | A | 0.5 | G | 0.5 | 13 | 0.41935 |
| 5212 | S6_47239734 | 6 | 47239734 | 152 | G | A | G | 0.8871 | A | 0.1129 | 5 | 0.16129 |
| 5213 | S6_47266865 | 6 | 47266865 | 27131 | G | A | G | 0.58065 | A | 0.41935 | 14 | 0.45161 |
| 5214 | S6_47266935 | 6 | 47266935 | 70 | G | A | G | 0.95161 | A | 0.04839 | 3 | 0.09677 |
| 5215 | S6_47491938 | 6 | 47491938 | 225003 | G | A | G | 0.82258 | A | 0.17742 | 9 | 0.29032 |
| 5216 | S6_47491954 | 6 | 47491954 | 16 | A | G | A | 0.51613 | G | 0.48387 | 14 | 0.45161 |
| 5217 | S6_47491978 | 6 | 47491978 | 24 | G | A | G | 0.69355 | A | 0.30645 | 13 | 0.41935 |
| 5218 | S6_47492123 | 6 | 47492123 | 145 | C | T | C | 0.82258 | T | 0.17742 | 9 | 0.29032 |
| 5219 | S6_47744251 | 6 | 47744251 | 252128 | C | T | C | 0.91935 | T | 0.08065 | 3 | 0.09677 |
| 5220 | S6_47744325 | 6 | 47744325 | 74 | A | C | A | 0.66129 | C | 0.33871 | 13 | 0.41935 |
| 5221 | S6_47744417 | 6 | 47744417 | 92 | C | G | C | 0.95161 | G | 0.04839 | 3 | 0.09677 |
| 5222 | S6_48195346 | 6 | 48195346 | 450929 | C | T | C | 0.82258 | T | 0.17742 | 7 | 0.22581 |
| 5223 | S6_48195518 | 6 | 48195518 | 172 | T | C | T | 0.95161 | C | 0.04839 | 3 | 0.09677 |
| 5224 | S6_48430092 | 6 | 48430092 | 234574 | T | G | T | 0.91935 | G | 0.08065 | 5 | 0.16129 |
| 5225 | S6_48452732 | 6 | 48452732 | 22640 | G | A | A | 0.83871 | G | 0.16129 | 8 | 0.25806 |
| 5226 | S6_48452777 | 6 | 48452777 | 45 | C | T | C | 0.66129 | T | 0.33871 | 11 | 0.35484 |
| 5227 | S6_48452856 | 6 | 48452856 | 79 | G | A | G | 0.91935 | A | 0.08065 | 5 | 0.16129 |
| 5228 | S6_48452882 | 6 | 48452882 | 26 | G | A | G | 0.66129 | A | 0.33871 | 11 | 0.35484 |
| 5229 | S6_48452883 | 6 | 48452883 | 1 | C | A | C | 0.67742 | A | 0.32258 | 12 | 0.3871 |
| 5230 | S6_48852076 | 6 | 48852076 | 399193 | G | A | G | 0.85484 | A | 0.14516 | 9 | 0.29032 |
| 5231 | S6_48852120 | 6 | 48852120 | 44 | A | G | A | 0.83871 | G | 0.16129 | 10 | 0.32258 |
| 5232 | S6_49389380 | 6 | 49389380 | 537260 | G | A | G | 0.56452 | A | 0.43548 | 17 | 0.54839 |
| 5233 | S6_49389442 | 6 | 49389442 | 62 | G | T | G | 0.74194 | T | 0.25806 | 10 | 0.32258 |
| 5234 | S6_49517267 | 6 | 49517267 | 127825 | G | A | G | 0.91935 | A | 0.08065 | 5 | 0.16129 |
| 5235 | S6_50083976 | 6 | 50083976 | 566709 | G | A | G | 0.93548 | A | 0.06452 | 4 | 0.12903 |
| 5236 | S6_50400218 | 6 | 50400218 | 316242 | C | T | C | 0.95161 | T | 0.04839 | 3 | 0.09677 |

| Site number | SNP name | Chromosome | Physical position | Physical distance from the previous SNP | Reference allele | Alternative allele | Major allele | Major allele frequency | Minor allele | Minor allele frequency | Number heterozygous | Heterozygosity |
|---|---|---|---|---|---|---|---|---|---|---|---|---|
| 5237 | S6_50400219 | 6 | 50400219 | 1 | G | A | G | 0.51613 | A | 0.48387 | 18 | 0.58065 |
| 5238 | S6_50581949 | 6 | 50581949 | 181730 | C | G | G | 0.56452 | C | 0.43548 | 17 | 0.54839 |
| 5239 | S6_50581975 | 6 | 50581975 | 26 | G | A | A | 0.51613 | G | 0.48387 | 16 | 0.51613 |
| 5240 | S6_50582102 | 6 | 50582102 | 127 | G | A | A | 0.56452 | G | 0.43548 | 17 | 0.54839 |
| 5241 | S6_50582108 | 6 | 50582108 | 6 | A | G | G | 0.85484 | A | 0.14516 | 5 | 0.16129 |
| 5242 | S6_50582114 | 6 | 50582114 | 6 | A | G | A | 0.56452 | G | 0.43548 | 17 | 0.54839 |
| 5243 | S6_51537474 | 6 | 51537474 | 955360 | G | A | G | 0.54839 | A | 0.45161 | 20 | 0.64516 |
| 5244 | S6_51537525 | 6 | 51537525 | 51 | T | C | T | 0.93548 | C | 0.06452 | 2 | 0.06452 |
| 5245 | S6_51537642 | 6 | 51537642 | 117 | T | C | C | 0.75806 | T | 0.24194 | 13 | 0.41935 |
| 5246 | S6_51662703 | 6 | 51662703 | 125061 | G | T | G | 0.91935 | T | 0.08065 | 5 | 0.16129 |
| 5247 | S6_51662814 | 6 | 51662814 | 111 | C | G | C | 0.66129 | G | 0.33871 | 11 | 0.35484 |
| 5248 | S6_52732103 | 6 | 52732103 | 1069289 | A | G | G | 0.58065 | A | 0.41935 | 14 | 0.45161 |
| 5249 | S6_52919625 | 6 | 52919625 | 187522 | C | T | T | 0.93548 | C | 0.06452 | 4 | 0.12903 |
| 5250 | S6_53761217 | 6 | 53761217 | 841592 | T | C | C | 0.58065 | T | 0.41935 | 12 | 0.3871 |
| 5251 | S6_53761355 | 6 | 53761355 | 138 | G | A | G | 0.80645 | A | 0.19355 | 8 | 0.25806 |
| 5252 | S6_53853882 | 6 | 53853882 | 92527 | G | A | G | 0.8871 | A | 0.1129 | 5 | 0.16129 |
| 5253 | S6_54471104 | 6 | 54471104 | 617222 | A | T | A | 0.85484 | T | 0.14516 | 9 | 0.29032 |
| 5254 | S6_54471130 | 6 | 54471130 | 26 | A | T | A | 0.64516 | T | 0.35484 | 14 | 0.45161 |
| 5255 | S6_54471182 | 6 | 54471182 | 52 | C | A | C | 0.64516 | A | 0.35484 | 14 | 0.45161 |
| 5256 | S6_54471184 | 6 | 54471184 | 2 | G | A | G | 0.64516 | A | 0.35484 | 14 | 0.45161 |
| 5257 | S6_54471244 | 6 | 54471244 | 60 | G | A | G | 0.80645 | A | 0.19355 | 10 | 0.32258 |
| 5258 | S6_54471317 | 6 | 54471317 | 73 | C | G | C | 0.64516 | G | 0.35484 | 14 | 0.45161 |
| 5259 | S6_54471327 | 6 | 54471327 | 10 | C | T | C | 0.87097 | T | 0.12903 | 8 | 0.25806 |
| 5260 | S6_54471335 | 6 | 54471335 | 8 | T | C | T | 0.64516 | C | 0.35484 | 14 | 0.45161 |
| 5261 | S6_54682643 | 6 | 54682643 | 211308 | A | C | C | 0.70968 | A | 0.29032 | 16 | 0.51613 |
| 5262 | S6_55118116 | 6 | 55118116 | 435473 | G | A | G | 0.91935 | A | 0.08065 | 5 | 0.16129 |
| 5263 | S6_55118118 | 6 | 55118118 | 2 | G | A | G | 0.91935 | A | 0.08065 | 5 | 0.16129 |
| 5264 | S6_55118126 | 6 | 55118126 | 8 | C | T | C | 0.95161 | T | 0.04839 | 3 | 0.09677 |
| 5265 | S6_55512339 | 6 | 55512339 | 394213 | C | T | C | 0.79032 | T | 0.20968 | 11 | 0.35484 |
| 5266 | S6_55585753 | 6 | 55585753 | 73414 | A | T | A | 0.93548 | T | 0.06452 | 2 | 0.06452 |
| 5267 | S6_55585971 | 6 | 55585971 | 218 | G | A | G | 0.69355 | A | 0.30645 | 11 | 0.35484 |
| 5268 | S6_55596449 | 6 | 55596449 | 10478 | G | A | A | 0.56452 | G | 0.43548 | 11 | 0.35484 |
| 5269 | S6_55596471 | 6 | 55596471 | 22 | A | G | G | 0.75806 | A | 0.24194 | 9 | 0.29032 |
| 5270 | S6_55596563 | 6 | 55596563 | 92 | A | G | G | 0.56452 | A | 0.43548 | 11 | 0.35484 |
| 5271 | S6_55695127 | 6 | 55695127 | 98564 | C | T | C | 0.95161 | T | 0.04839 | 3 | 0.09677 |
| 5272 | S6_55695153 | 6 | 55695153 | 26 | T | C | C | 0.56452 | T | 0.43548 | 17 | 0.54839 |
| 5273 | S6_55695188 | 6 | 55695188 | 35 | C | G | C | 0.90323 | G | 0.09677 | 6 | 0.19355 |
| 5274 | S6_56765193 | 6 | 56765193 | 1070005 | C | T | C | 0.67742 | T | 0.32258 | 16 | 0.51613 |
| 5275 | S6_56765241 | 6 | 56765241 | 48 | C | G | C | 0.91935 | G | 0.08065 | 3 | 0.09677 |
| 5276 | S6_56765319 | 6 | 56765319 | 78 | G | A | G | 0.64516 | A | 0.35484 | 16 | 0.51613 |
| 5277 | S6_56809498 | 6 | 56809498 | 44179 | C | T | C | 0.64516 | T | 0.35484 | 12 | 0.3871 |
| 5278 | S6_56809641 | 6 | 56809641 | 143 | G | A | G | 0.91935 | A | 0.08065 | 5 | 0.16129 |
| 5279 | S6_56962130 | 6 | 56962130 | 152489 | C | T | C | 0.66129 | T | 0.33871 | 19 | 0.6129 |
| 5280 | S6_57229904 | 6 | 57229904 | 267774 | A | G | G | 0.87097 | A | 0.12903 | 8 | 0.25806 |
| 5281 | S6_57229925 | 6 | 57229925 | 21 | A | G | A | 0.80645 | G | 0.19355 | 10 | 0.32258 |
| 5282 | S6_57346026 | 6 | 57346026 | 116101 | G | A | G | 0.90323 | A | 0.09677 | 6 | 0.19355 |
| 5283 | S6_57571704 | 6 | 57571704 | 225678 | G | A | G | 0.64516 | A | 0.35484 | 14 | 0.45161 |
| 5284 | S6_57571763 | 6 | 57571763 | 59 | G | A | G | 0.77419 | A | 0.22581 | 12 | 0.3871 |
| 5285 | S6_57571802 | 6 | 57571802 | 39 | C | T | C | 0.8871 | T | 0.1129 | 7 | 0.22581 |
| 5286 | S6_57571814 | 6 | 57571814 | 12 | T | G | T | 0.6129 | G | 0.3871 | 14 | 0.45161 |
| 5287 | S6_58156270 | 6 | 58156270 | 584456 | C | T | C | 0.93548 | T | 0.06452 | 4 | 0.12903 |
| 5288 | S6_58226161 | 6 | 58226161 | 69891 | T | G | T | 0.95161 | G | 0.04839 | 3 | 0.09677 |
| 5289 | S6_59006559 | 6 | 59006559 | 780398 | G | A | A | 0.79032 | G | 0.20968 | 9 | 0.29032 |
| 5290 | S6_59006589 | 6 | 59006589 | 30 | C | A | C | 0.93548 | A | 0.06452 | 4 | 0.12903 |

| Site number | SNP name | Chromosome | Physical position | Physical distance from the previous SNP | Reference allele | Alternative allele | Major allele | Major allele frequency | Minor allele | Minor allele frequency | Number heterozygous | Heterozygosity |
|---|---|---|---|---|---|---|---|---|---|---|---|---|
| 5291 | S6_59006619 | 6 | 59006619 | 30 | T | C | T | 0.93548 | C | 0.06452 | 4 | 0.12903 |
| 5292 | S6_59151946 | 6 | 59151946 | 145327 | G | A | G | 0.69355 | A | 0.30645 | 15 | 0.48387 |
| 5293 | S6_59152125 | 6 | 59152125 | 179 | C | T | C | 0.69355 | T | 0.30645 | 15 | 0.48387 |
| 5294 | S6_59152135 | 6 | 59152135 | 10 | A | G | A | 0.69355 | G | 0.30645 | 15 | 0.48387 |
| 5295 | S6_59212612 | 6 | 59212612 | 60477 | A | G | A | 0.64516 | G | 0.35484 | 14 | 0.45161 |
| 5296 | S6_59212829 | 6 | 59212829 | 217 | G | T | G | 0.95161 | T | 0.04839 | 3 | 0.09677 |
| 5297 | S6_59753619 | 6 | 59753619 | 540790 | T | C | C | 0.85484 | T | 0.14516 | 5 | 0.16129 |
| 5298 | S6_59753633 | 6 | 59753633 | 14 | C | T | C | 0.85484 | T | 0.14516 | 9 | 0.29032 |
| 5299 | S6_59753731 | 6 | 59753731 | 98 | G | A | A | 0.87097 | G | 0.12903 | 6 | 0.19355 |
| 5300 | S6_60089297 | 6 | 60089297 | 335566 | C | T | C | 0.58065 | T | 0.41935 | 12 | 0.3871 |
| 5301 | S6_60089373 | 6 | 60089373 | 76 | A | G | A | 0.95161 | G | 0.04839 | 3 | 0.09677 |
| 5302 | S6_60089387 | 6 | 60089387 | 14 | C | T | C | 0.67742 | T | 0.32258 | 14 | 0.45161 |
| 5303 | S6_60599450 | 6 | 60599450 | 510063 | G | A | G | 0.75806 | A | 0.24194 | 15 | 0.48387 |
| 5304 | S6_60599517 | 6 | 60599517 | 67 | A | C | C | 0.67742 | A | 0.32258 | 12 | 0.3871 |
| 5305 | S6_60599587 | 6 | 60599587 | 70 | C | A | C | 0.69355 | A | 0.30645 | 13 | 0.41935 |
| 5306 | S6_60965030 | 6 | 60965030 | 365443 | A | G | A | 0.66129 | G | 0.33871 | 15 | 0.48387 |
| 5307 | S6_61357855 | 6 | 61357855 | 392825 | G | A | G | 0.95161 | A | 0.04839 | 3 | 0.09677 |
| 5308 | S6_62268592 | 6 | 62268592 | 910737 | A | C | A | 0.62903 | C | 0.37097 | 13 | 0.41935 |
| 5309 | S6_62268654 | 6 | 62268654 | 62 | C | T | T | 0.83871 | C | 0.16129 | 8 | 0.25806 |
| 5310 | S6_62425932 | 6 | 62425932 | 157278 | G | C | G | 0.79032 | C | 0.20968 | 9 | 0.29032 |
| 5311 | S6_62425958 | 6 | 62425958 | 26 | C | T | C | 0.8871 | T | 0.1129 | 7 | 0.22581 |
| 5312 | S6_62426045 | 6 | 62426045 | 87 | G | A | G | 0.79032 | A | 0.20968 | 9 | 0.29032 |
| 5313 | S6_62484146 | 6 | 62484146 | 58101 | A | G | G | 0.77419 | A | 0.22581 | 10 | 0.32258 |
| 5314 | S6_62484191 | 6 | 62484191 | 45 | T | C | T | 0.77419 | C | 0.22581 | 10 | 0.32258 |
| 5315 | S6_62484214 | 6 | 62484214 | 23 | T | C | T | 0.77419 | C | 0.22581 | 10 | 0.32258 |
| 5316 | S6_62484244 | 6 | 62484244 | 30 | T | C | T | 0.91935 | C | 0.08065 | 5 | 0.16129 |
| 5317 | S6_62484248 | 6 | 62484248 | 4 | T | A | T | 0.90323 | A | 0.09677 | 6 | 0.19355 |
| 5318 | S6_62484283 | 6 | 62484283 | 35 | T | C | T | 0.77419 | C | 0.22581 | 10 | 0.32258 |
| 5319 | S6_62569393 | 6 | 62569393 | 85110 | C | T | C | 0.93548 | T | 0.06452 | 4 | 0.12903 |
| 5320 | S6_62569399 | 6 | 62569399 | 6 | T | C | T | 0.93548 | C | 0.06452 | 4 | 0.12903 |
| 5321 | S6_62569501 | 6 | 62569501 | 102 | A | G | A | 0.64516 | G | 0.35484 | 16 | 0.51613 |
| 5322 | S6_63037511 | 6 | 63037511 | 468010 | G | A | G | 0.77419 | A | 0.22581 | 10 | 0.32258 |
| 5323 | S6_63037585 | 6 | 63037585 | 74 | A | T | A | 0.6129 | T | 0.3871 | 18 | 0.58065 |
| 5324 | S6_63037655 | 6 | 63037655 | 70 | C | T | C | 0.69355 | T | 0.30645 | 11 | 0.35484 |
| 5325 | S6_63037685 | 6 | 63037685 | 30 | A | T | A | 0.75806 | T | 0.24194 | 11 | 0.35484 |
| 5326 | S6_63623975 | 6 | 63623975 | 586290 | C | G | C | 0.90323 | G | 0.09677 | 4 | 0.12903 |
| 5327 | S6_63624014 | 6 | 63624014 | 39 | C | T | C | 0.59677 | T | 0.40323 | 13 | 0.41935 |
| 5328 | S6_63808569 | 6 | 63808569 | 184555 | G | A | G | 0.80645 | A | 0.19355 | 12 | 0.3871 |
| 5329 | S6_63808578 | 6 | 63808578 | 9 | T | C | C | 0.87097 | T | 0.12903 | 6 | 0.19355 |
| 5330 | S6_63808611 | 6 | 63808611 | 33 | G | T | G | 0.72581 | T | 0.27419 | 13 | 0.41935 |
| 5331 | S6_63852998 | 6 | 63852998 | 44387 | A | G | G | 0.62903 | A | 0.37097 | 15 | 0.48387 |
| 5332 | S6_63853054 | 6 | 63853054 | 56 | G | A | G | 0.93548 | A | 0.06452 | 2 | 0.06452 |
| 5333 | S6_63853076 | 6 | 63853076 | 22 | C | G | C | 0.93548 | G | 0.06452 | 4 | 0.12903 |
| 5334 | S6_63853077 | 6 | 63853077 | 1 | T | G | T | 0.62903 | G | 0.37097 | 15 | 0.48387 |
| 5335 | S6_63853112 | 6 | 63853112 | 35 | A | C | A | 0.90323 | C | 0.09677 | 6 | 0.19355 |
| 5336 | S6_64282935 | 6 | 64282935 | 429823 | G | A | G | 0.95161 | A | 0.04839 | 3 | 0.09677 |
| 5337 | S6_64384604 | 6 | 64384604 | 101669 | A | G | G | 0.75806 | A | 0.24194 | 15 | 0.48387 |
| 5338 | S6_64384642 | 6 | 64384642 | 38 | C | G | C | 0.75806 | G | 0.24194 | 15 | 0.48387 |
| 5339 | S6_64384651 | 6 | 64384651 | 9 | G | A | A | 0.6129 | G | 0.3871 | 14 | 0.45161 |
| 5340 | S6_64669787 | 6 | 64669787 | 285136 | G | A | G | 0.90323 | A | 0.09677 | 6 | 0.19355 |
| 5341 | S6_64669829 | 6 | 64669829 | 42 | G | A | G | 0.90323 | A | 0.09677 | 6 | 0.19355 |
| 5342 | S6_64669873 | 6 | 64669873 | 44 | A | G | G | 0.83871 | A | 0.16129 | 6 | 0.19355 |
| 5343 | S6_64669927 | 6 | 64669927 | 54 | T | C | C | 0.74194 | T | 0.25806 | 10 | 0.32258 |
| 5344 | S6_64669997 | 6 | 64669997 | 70 | T | C | T | 0.90323 | C | 0.09677 | 6 | 0.19355 |

| Site number | SNP name | Chromosome | Physical position | Physical distance from the previous SNP | Reference allele | Alternative allele | Major allele | Major allele frequency | Minor allele | Minor allele frequency | Number heterozygous | Heterozygosity |
|---|---|---|---|---|---|---|---|---|---|---|---|---|
| 5345 | S6_65176358 | 6 | 65176358 | 506361 | T | C | T | 0.95161 | C | 0.04839 | 3 | 0.09677 |
| 5346 | S6_65176567 | 6 | 65176567 | 209 | T | C | T | 0.80645 | C | 0.19355 | 8 | 0.25806 |
| 5347 | S6_66921237 | 6 | 66921237 | 1744670 | A | G | A | 0.93548 | G | 0.06452 | 4 | 0.12903 |
| 5348 | S6_66921278 | 6 | 66921278 | 41 | C | T | C | 0.62903 | T | 0.37097 | 19 | 0.6129 |
| 5349 | S6_66921327 | 6 | 66921327 | 49 | C | T | C | 0.82258 | T | 0.17742 | 11 | 0.35484 |
| 5350 | S6_66921411 | 6 | 66921411 | 84 | T | A | T | 0.62903 | A | 0.37097 | 19 | 0.6129 |
| 5351 | S6_67544674 | 6 | 67544674 | 623263 | C | T | C | 0.83871 | T | 0.16129 | 8 | 0.25806 |
| 5352 | S6_67544702 | 6 | 67544702 | 28 | A | G | G | 0.62903 | A | 0.37097 | 15 | 0.48387 |
| 5353 | S6_68047482 | 6 | 68047482 | 502780 | A | G | G | 0.74194 | A | 0.25806 | 10 | 0.32258 |
| 5354 | S6_68122286 | 6 | 68122286 | 74804 | C | T | C | 0.72581 | T | 0.27419 | 9 | 0.29032 |
| 5355 | S6_68122431 | 6 | 68122431 | 145 | T | C | T | 0.83871 | C | 0.16129 | 8 | 0.25806 |
| 5356 | S6_68239207 | 6 | 68239207 | 116776 | G | A | G | 0.85484 | A | 0.14516 | 7 | 0.22581 |
| 5357 | S6_68595876 | 6 | 68595876 | 356669 | C | A | C | 0.82258 | A | 0.17742 | 9 | 0.29032 |
| 5358 | S6_68595942 | 6 | 68595942 | 66 | G | A | A | 0.54839 | G | 0.45161 | 16 | 0.51613 |
| 5359 | S6_68861147 | 6 | 68861147 | 265205 | T | G | G | 0.87097 | T | 0.12903 | 6 | 0.19355 |
| 5360 | S6_68861304 | 6 | 68861304 | 157 | G | A | G | 0.91935 | A | 0.08065 | 5 | 0.16129 |
| 5361 | S6_68969552 | 6 | 68969552 | 108248 | A | G | A | 0.67742 | G | 0.32258 | 14 | 0.45161 |
| 5362 | S6_68969598 | 6 | 68969598 | 46 | A | C | A | 0.67742 | C | 0.32258 | 14 | 0.45161 |
| 5363 | S6_68969613 | 6 | 68969613 | 15 | T | C | T | 0.67742 | C | 0.32258 | 14 | 0.45161 |
| 5364 | S6_68969679 | 6 | 68969679 | 66 | A | G | A | 0.67742 | G | 0.32258 | 14 | 0.45161 |
| 5365 | S6_68969689 | 6 | 68969689 | 10 | A | G | A | 0.67742 | G | 0.32258 | 14 | 0.45161 |
| 5366 | S6_68969735 | 6 | 68969735 | 46 | C | T | C | 0.67742 | T | 0.32258 | 14 | 0.45161 |
| 5367 | S6_68974354 | 6 | 68974354 | 4619 | C | G | C | 0.83871 | G | 0.16129 | 10 | 0.32258 |
| 5368 | S6_69067139 | 6 | 69067139 | 92785 | C | T | C | 0.93548 | T | 0.06452 | 4 | 0.12903 |
| 5369 | S6_69257031 | 6 | 69257031 | 189892 | C | T | C | 0.91935 | T | 0.08065 | 5 | 0.16129 |
| 5370 | S6_69258862 | 6 | 69258862 | 1831 | C | T | C | 0.5 | T | 0.5 | 17 | 0.54839 |
| 5371 | S6_69366710 | 6 | 69366710 | 107848 | C | T | T | 0.90323 | C | 0.09677 | 4 | 0.12903 |
| 5372 | S6_69420152 | 6 | 69420152 | 53442 | G | A | G | 0.87097 | A | 0.12903 | 6 | 0.19355 |
| 5373 | S6_69611551 | 6 | 69611551 | 191399 | C | T | C | 0.64516 | T | 0.35484 | 12 | 0.3871 |
| 5374 | S6_69611621 | 6 | 69611621 | 70 | T | C | T | 0.64516 | C | 0.35484 | 12 | 0.3871 |
| 5375 | S6_69611645 | 6 | 69611645 | 24 | G | A | G | 0.64516 | A | 0.35484 | 12 | 0.3871 |
| 5376 | S6_69611682 | 6 | 69611682 | 37 | C | T | T | 0.53226 | C | 0.46774 | 15 | 0.48387 |
| 5377 | S6_69611697 | 6 | 69611697 | 15 | C | T | C | 0.85484 | T | 0.14516 | 9 | 0.29032 |
| 5378 | S6_69929318 | 6 | 69929318 | 317621 | G | A | G | 0.8871 | A | 0.1129 | 7 | 0.22581 |
| 5379 | S6_70307748 | 6 | 70307748 | 378430 | G | T | G | 0.70968 | T | 0.29032 | 10 | 0.32258 |
| 5380 | S6_70307882 | 6 | 70307882 | 134 | T | C | C | 0.51613 | T | 0.48387 | 14 | 0.45161 |
| 5381 | S6_70317139 | 6 | 70317139 | 9257 | G | A | G | 0.79032 | A | 0.20968 | 9 | 0.29032 |
| 5382 | S6_70398234 | 6 | 70398234 | 81095 | T | G | T | 0.53226 | G | 0.46774 | 13 | 0.41935 |
| 5383 | S6_70398310 | 6 | 70398310 | 76 | T | G | G | 0.74194 | T | 0.25806 | 10 | 0.32258 |
| 5384 | S6_70398418 | 6 | 70398418 | 108 | G | A | G | 0.93548 | A | 0.06452 | 4 | 0.12903 |
| 5385 | S6_70763802 | 6 | 70763802 | 365384 | C | A | A | 0.72581 | C | 0.27419 | 11 | 0.35484 |
| 5386 | S6_70965203 | 6 | 70965203 | 201401 | C | T | C | 0.85484 | T | 0.14516 | 5 | 0.16129 |
| 5387 | S6_71098627 | 6 | 71098627 | 133424 | G | A | G | 0.8871 | A | 0.1129 | 5 | 0.16129 |
| 5388 | S6_71114156 | 6 | 71114156 | 15529 | A | G | A | 0.8871 | G | 0.1129 | 5 | 0.16129 |
| 5389 | S6_71114260 | 6 | 71114260 | 104 | A | G | A | 0.93548 | G | 0.06452 | 4 | 0.12903 |
| 5390 | S6_71114374 | 6 | 71114374 | 114 | A | G | A | 0.80645 | G | 0.19355 | 12 | 0.3871 |
| 5391 | S6_71117080 | 6 | 71117080 | 2706 | T | C | T | 0.69355 | C | 0.30645 | 13 | 0.41935 |
| 5392 | S6_71117159 | 6 | 71117159 | 79 | G | A | G | 0.77419 | A | 0.22581 | 10 | 0.32258 |
| 5393 | S6_71135980 | 6 | 71135980 | 18821 | G | A | G | 0.93548 | A | 0.06452 | 4 | 0.12903 |
| 5394 | S6_71136031 | 6 | 71136031 | 51 | C | T | C | 0.87097 | T | 0.12903 | 6 | 0.19355 |
| 5395 | S6_71177538 | 6 | 71177538 | 41507 | T | C | T | 0.95161 | C | 0.04839 | 3 | 0.09677 |
| 5396 | S6_71362648 | 6 | 71362648 | 185110 | C | T | T | 0.56452 | C | 0.43548 | 15 | 0.48387 |
| 5397 | S6_71362808 | 6 | 71362808 | 160 | G | A | G | 0.91935 | A | 0.08065 | 5 | 0.16129 |
| 5398 | S6_71815011 | 6 | 71815011 | 452203 | G | A | G | 0.95161 | A | 0.04839 | 1 | 0.03226 |

| Site number | SNP name | Chromosome | Physical position | Physical distance from the previous SNP | Reference allele | Alternative allele | Major allele | Major allele frequency | Minor allele | Minor allele frequency | Number heterozygous | Heterozygosity |
|---|---|---|---|---|---|---|---|---|---|---|---|---|
| 5399 | S6_71815077 | 6 | 71815077 | 66 | G | A | G | 0.77419 | A | 0.22581 | 12 | 0.3871 |
| 5400 | S6_72059643 | 6 | 72059643 | 244566 | A | G | A | 0.82258 | G | 0.17742 | 11 | 0.35484 |
| 5401 | S6_72099298 | 6 | 72099298 | 39655 | C | T | C | 0.8871 | T | 0.1129 | 7 | 0.22581 |
| 5402 | S6_72155109 | 6 | 72155109 | 55811 | G | A | G | 0.82258 | A | 0.17742 | 11 | 0.35484 |
| 5403 | S6_72191348 | 6 | 72191348 | 36239 | G | A | G | 0.82258 | A | 0.17742 | 11 | 0.35484 |
| 5404 | S6_72191430 | 6 | 72191430 | 82 | G | A | A | 0.53226 | G | 0.46774 | 11 | 0.35484 |
| 5405 | S6_72197926 | 6 | 72197926 | 6496 | A | T | A | 0.53226 | T | 0.46774 | 21 | 0.67742 |
| 5406 | S6_72211382 | 6 | 72211382 | 13456 | A | G | A | 0.75806 | G | 0.24194 | 13 | 0.41935 |
| 5407 | S6_72211456 | 6 | 72211456 | 74 | G | T | G | 0.67742 | T | 0.32258 | 14 | 0.45161 |
| 5408 | S6_72285204 | 6 | 72285204 | 73748 | T | C | T | 0.79032 | C | 0.20968 | 13 | 0.41935 |
| 5409 | S6_72385038 | 6 | 72385038 | 99834 | G | T | G | 0.95161 | T | 0.04839 | 3 | 0.09677 |
| 5410 | S6_72504117 | 6 | 72504117 | 119079 | A | G | A | 0.80645 | G | 0.19355 | 10 | 0.32258 |
| 5411 | S6_72504139 | 6 | 72504139 | 22 | C | T | C | 0.79032 | T | 0.20968 | 13 | 0.41935 |
| 5412 | S6_72504157 | 6 | 72504157 | 18 | A | G | A | 0.90323 | G | 0.09677 | 6 | 0.19355 |
| 5413 | S6_72504168 | 6 | 72504168 | 11 | A | G | A | 0.90323 | G | 0.09677 | 6 | 0.19355 |
| 5414 | S6_72504182 | 6 | 72504182 | 14 | C | T | C | 0.90323 | T | 0.09677 | 6 | 0.19355 |
| 5415 | S6_72504183 | 6 | 72504183 | 1 | G | A | G | 0.90323 | A | 0.09677 | 6 | 0.19355 |
| 5416 | S6_72560752 | 6 | 72560752 | 56569 | C | A | C | 0.8871 | A | 0.1129 | 7 | 0.22581 |
| 5417 | S6_72560848 | 6 | 72560848 | 96 | C | T | C | 0.69355 | T | 0.30645 | 15 | 0.48387 |
| 5418 | S6_72695408 | 6 | 72695408 | 134560 | G | C | G | 0.69355 | C | 0.30645 | 13 | 0.41935 |
| 5419 | S6_73114342 | 6 | 73114342 | 418934 | G | T | G | 0.79032 | T | 0.20968 | 11 | 0.35484 |
| 5420 | S6_73855418 | 6 | 73855418 | 741076 | G | A | G | 0.83871 | A | 0.16129 | 10 | 0.32258 |
| 5421 | S6_73855445 | 6 | 73855445 | 27 | G | A | G | 0.91935 | A | 0.08065 | 5 | 0.16129 |
| 5422 | S6_73855496 | 6 | 73855496 | 51 | G | A | G | 0.90323 | A | 0.09677 | 6 | 0.19355 |
| 5423 | S6_73855567 | 6 | 73855567 | 71 | G | A | G | 0.85484 | A | 0.14516 | 5 | 0.16129 |
| 5424 | S6_74134768 | 6 | 74134768 | 279201 | A | G | A | 0.90323 | G | 0.09677 | 4 | 0.12903 |
| 5425 | S6_74134791 | 6 | 74134791 | 23 | T | C | C | 0.74194 | T | 0.25806 | 10 | 0.32258 |
| 5426 | S6_74134829 | 6 | 74134829 | 38 | C | T | C | 0.90323 | T | 0.09677 | 6 | 0.19355 |
| 5427 | S6_74134916 | 6 | 74134916 | 87 | A | G | A | 0.66129 | G | 0.33871 | 15 | 0.48387 |
| 5428 | S6_74134921 | 6 | 74134921 | 5 | C | T | C | 0.75806 | T | 0.24194 | 13 | 0.41935 |
| 5429 | S6_74134927 | 6 | 74134927 | 6 | G | A | G | 0.87097 | A | 0.12903 | 8 | 0.25806 |
| 5430 | S6_74671154 | 6 | 74671154 | 536227 | A | G | A | 0.69355 | G | 0.30645 | 17 | 0.54839 |
| 5431 | S6_74672371 | 6 | 74672371 | 1217 | A | C | A | 0.64516 | C | 0.35484 | 18 | 0.58065 |
| 5432 | S6_74672573 | 6 | 74672573 | 202 | T | C | T | 0.64516 | C | 0.35484 | 18 | 0.58065 |
| 5433 | S6_76037742 | 6 | 76037742 | 1365169 | T | A | T | 0.83871 | A | 0.16129 | 8 | 0.25806 |
| 5434 | S6_76037875 | 6 | 76037875 | 133 | G | A | G | 0.74194 | A | 0.25806 | 16 | 0.51613 |
| 5435 | S6_76224780 | 6 | 76224780 | 186905 | C | G | C | 0.93548 | G | 0.06452 | 4 | 0.12903 |
| 5436 | S6_76224822 | 6 | 76224822 | 42 | T | C | T | 0.62903 | C | 0.37097 | 17 | 0.54839 |
| 5437 | S6_76441928 | 6 | 76441928 | 217106 | T | C | C | 0.8871 | T | 0.1129 | 5 | 0.16129 |
| 5438 | S6_76775526 | 6 | 76775526 | 333598 | A | G | G | 0.56452 | A | 0.43548 | 19 | 0.6129 |
| 5439 | S6_77301109 | 6 | 77301109 | 525583 | G | A | G | 0.95161 | A | 0.04839 | 3 | 0.09677 |
| 5440 | S6_77301148 | 6 | 77301148 | 39 | A | T | A | 0.93548 | T | 0.06452 | 4 | 0.12903 |
| 5441 | S6_77301203 | 6 | 77301203 | 55 | T | C | T | 0.93548 | C | 0.06452 | 4 | 0.12903 |
| 5442 | S6_77813743 | 6 | 77813743 | 512540 | T | A | T | 0.74194 | A | 0.25806 | 12 | 0.3871 |
| 5443 | S6_77813783 | 6 | 77813783 | 40 | A | G | A | 0.93548 | G | 0.06452 | 4 | 0.12903 |
| 5444 | S6_79149340 | 6 | 79149340 | 1335557 | G | A | G | 0.82258 | A | 0.17742 | 9 | 0.29032 |
| 5445 | S6_79149400 | 6 | 79149400 | 60 | G | A | G | 0.87097 | A | 0.12903 | 4 | 0.12903 |
| 5446 | S6_79149478 | 6 | 79149478 | 78 | G | A | G | 0.79032 | A | 0.20968 | 9 | 0.29032 |
| 5447 | S6_80355114 | 6 | 80355114 | 1205636 | T | C | C | 0.74194 | T | 0.25806 | 14 | 0.45161 |
| 5448 | S6_80355152 | 6 | 80355152 | 38 | T | C | C | 0.74194 | T | 0.25806 | 14 | 0.45161 |
| 5449 | S6_80355206 | 6 | 80355206 | 54 | G | T | T | 0.74194 | G | 0.25806 | 14 | 0.45161 |
| 5450 | S6_80355208 | 6 | 80355208 | 2 | C | T | T | 0.74194 | C | 0.25806 | 14 | 0.45161 |
| 5451 | S6_80355282 | 6 | 80355282 | 74 | G | A | G | 0.90323 | A | 0.09677 | 4 | 0.12903 |
| 5452 | S6_81369204 | 6 | 81369204 | 1013922 | C | T | C | 0.70968 | T | 0.29032 | 10 | 0.32258 |

| Site number | SNP name | Chromosome | Physical position | Physical distance from the previous SNP | Reference allele | Alternative allele | Major allele | Major allele frequency | Minor allele | Minor allele frequency | Number heterozygous | Heterozygosity |
|---|---|---|---|---|---|---|---|---|---|---|---|---|
| 5453 | S6_81369239 | 6 | 81369239 | 35 | G | C | G | 0.82258 | C | 0.17742 | 9 | 0.29032 |
| 5454 | S6_81778862 | 6 | 81778862 | 409623 | T | C | C | 0.56452 | T | 0.43548 | 19 | 0.6129 |
| 5455 | S6_81840902 | 6 | 81840902 | 62040 | T | C | T | 0.67742 | C | 0.32258 | 18 | 0.58065 |
| 5456 | S6_81840910 | 6 | 81840910 | 8 | C | T | C | 0.80645 | T | 0.19355 | 10 | 0.32258 |
| 5457 | S6_82774032 | 6 | 82774032 | 933122 | A | G | A | 0.69355 | G | 0.30645 | 15 | 0.48387 |
| 5458 | S6_82967334 | 6 | 82967334 | 193302 | G | A | G | 0.90323 | A | 0.09677 | 4 | 0.12903 |
| 5459 | S6_82967457 | 6 | 82967457 | 123 | G | A | G | 0.77419 | A | 0.22581 | 10 | 0.32258 |
| 5460 | S6_82967477 | 6 | 82967477 | 20 | C | T | C | 0.90323 | T | 0.09677 | 6 | 0.19355 |
| 5461 | S6_83097879 | 6 | 83097879 | 130402 | A | T | A | 0.8871 | T | 0.1129 | 7 | 0.22581 |
| 5462 | S6_84159714 | 6 | 84159714 | 1061835 | T | A | T | 0.90323 | A | 0.09677 | 6 | 0.19355 |
| 5463 | S6_84385161 | 6 | 84385161 | 225447 | A | C | A | 0.95161 | C | 0.04839 | 3 | 0.09677 |
| 5464 | S6_84385356 | 6 | 84385356 | 195 | C | T | C | 0.85484 | T | 0.14516 | 7 | 0.22581 |
| 5465 | S6_84385357 | 6 | 84385357 | 1 | G | A | G | 0.95161 | A | 0.04839 | 3 | 0.09677 |
| 5466 | S6_85795479 | 6 | 85795479 | 1410122 | G | T | G | 0.8871 | T | 0.1129 | 7 | 0.22581 |
| 5467 | S6_86239407 | 6 | 86239407 | 443928 | G | A | G | 0.77419 | A | 0.22581 | 14 | 0.45161 |
| 5468 | S6_86239461 | 6 | 86239461 | 54 | A | G | A | 0.77419 | G | 0.22581 | 8 | 0.25806 |
| 5469 | S6_87619941 | 6 | 87619941 | 1380480 | A | G | G | 0.56452 | A | 0.43548 | 13 | 0.41935 |
| 5470 | S6_87973760 | 6 | 87973760 | 353819 | C | T | C | 0.85484 | T | 0.14516 | 7 | 0.22581 |
| 5471 | S6_87973764 | 6 | 87973764 | 4 | G | T | G | 0.62903 | T | 0.37097 | 11 | 0.35484 |
| 5472 | S6_87976975 | 6 | 87976975 | 3211 | G | A | A | 0.64516 | G | 0.35484 | 14 | 0.45161 |
| 5473 | S6_88554688 | 6 | 88554688 | 577713 | A | G | G | 0.77419 | A | 0.22581 | 12 | 0.3871 |
| 5474 | S6_88599459 | 6 | 88599459 | 44771 | C | T | C | 0.93548 | T | 0.06452 | 4 | 0.12903 |
| 5475 | S6_88599519 | 6 | 88599519 | 60 | A | G | A | 0.90323 | G | 0.09677 | 6 | 0.19355 |
| 5476 | S6_88988978 | 6 | 88988978 | 389459 | T | C | C | 0.87097 | T | 0.12903 | 8 | 0.25806 |
| 5477 | S6_88989099 | 6 | 88989099 | 121 | T | C | C | 0.87097 | T | 0.12903 | 8 | 0.25806 |
| 5478 | S6_88989147 | 6 | 88989147 | 48 | G | A | A | 0.8871 | G | 0.1129 | 7 | 0.22581 |
| 5479 | S6_88989187 | 6 | 88989187 | 40 | T | C | C | 0.87097 | T | 0.12903 | 8 | 0.25806 |
| 5480 | S6_89017941 | 6 | 89017941 | 28754 | C | A | C | 0.67742 | A | 0.32258 | 18 | 0.58065 |
| 5481 | S6_89017955 | 6 | 89017955 | 14 | T | C | T | 0.95161 | C | 0.04839 | 3 | 0.09677 |
| 5482 | S6_89018014 | 6 | 89018014 | 59 | G | A | G | 0.58065 | A | 0.41935 | 14 | 0.45161 |
| 5483 | S6_89477511 | 6 | 89477511 | 459497 | T | G | T | 0.95161 | G | 0.04839 | 3 | 0.09677 |
| 5484 | S6_89477627 | 6 | 89477627 | 116 | G | A | G | 0.91935 | A | 0.08065 | 3 | 0.09677 |
| 5485 | S6_89524518 | 6 | 89524518 | 46891 | A | T | A | 0.72581 | T | 0.27419 | 11 | 0.35484 |
| 5486 | S6_89524519 | 6 | 89524519 | 1 | A | C | A | 0.72581 | C | 0.27419 | 11 | 0.35484 |
| 5487 | S6_89524548 | 6 | 89524548 | 29 | T | C | T | 0.91935 | C | 0.08065 | 5 | 0.16129 |
| 5488 | S6_89524582 | 6 | 89524582 | 34 | G | A | G | 0.82258 | A | 0.17742 | 7 | 0.22581 |
| 5489 | S6_89524590 | 6 | 89524590 | 8 | G | A | G | 0.90323 | A | 0.09677 | 4 | 0.12903 |
| 5490 | S6_90109260 | 6 | 90109260 | 584670 | C | T | C | 0.93548 | T | 0.06452 | 4 | 0.12903 |
| 5491 | S6_90109299 | 6 | 90109299 | 39 | G | A | G | 0.8871 | A | 0.1129 | 7 | 0.22581 |
| 5492 | S6_90146841 | 6 | 90146841 | 37542 | T | C | T | 0.64516 | C | 0.35484 | 14 | 0.45161 |
| 5493 | S6_90146848 | 6 | 90146848 | 7 | T | C | C | 0.70968 | T | 0.29032 | 14 | 0.45161 |
| 5494 | S6_90146943 | 6 | 90146943 | 95 | C | T | C | 0.87097 | T | 0.12903 | 4 | 0.12903 |
| 5495 | S6_90169692 | 6 | 90169692 | 22749 | G | A | A | 0.83871 | G | 0.16129 | 6 | 0.19355 |
| 5496 | S6_90169708 | 6 | 90169708 | 16 | T | C | C | 0.83871 | T | 0.16129 | 6 | 0.19355 |
| 5497 | S6_90169725 | 6 | 90169725 | 17 | G | A | A | 0.83871 | G | 0.16129 | 6 | 0.19355 |
| 5498 | S6_90169863 | 6 | 90169863 | 138 | C | T | T | 0.83871 | C | 0.16129 | 6 | 0.19355 |
| 5499 | S6_90322383 | 6 | 90322383 | 152520 | T | C | T | 0.54839 | C | 0.45161 | 16 | 0.51613 |
| 5500 | S6_90322420 | 6 | 90322420 | 37 | C | T | C | 0.83871 | T | 0.16129 | 8 | 0.25806 |
| 5501 | S6_90322439 | 6 | 90322439 | 19 | G | A | A | 0.51613 | G | 0.48387 | 18 | 0.58065 |
| 5502 | S6_90322491 | 6 | 90322491 | 52 | C | T | C | 0.91935 | T | 0.08065 | 5 | 0.16129 |
| 5503 | S6_90436312 | 6 | 90436312 | 113821 | G | A | G | 0.8871 | A | 0.1129 | 7 | 0.22581 |
| 5504 | S6_90587859 | 6 | 90587859 | 151547 | T | C | T | 0.90323 | C | 0.09677 | 6 | 0.19355 |
| 5505 | S6_90598905 | 6 | 90598905 | 11046 | C | T | C | 0.64516 | T | 0.35484 | 12 | 0.3871 |
| 5506 | S6_90599011 | 6 | 90599011 | 106 | A | T | A | 0.90323 | T | 0.09677 | 6 | 0.19355 |

| Site number | SNP name | Chromosome | Physical position | Physical distance from the previous SNP | Reference allele | Alternative allele | Major allele | Major allele frequency | Minor allele | Minor allele frequency | Number heterozygous | Heterozygosity |
|---|---|---|---|---|---|---|---|---|---|---|---|---|
| 5507 | S6_90741244 | 6 | 90741244 | 142233 | A | G | A | 0.90323 | G | 0.09677 | 6 | 0.19355 |
| 5508 | S6_90741296 | 6 | 90741296 | 52 | C | T | C | 0.87097 | T | 0.12903 | 6 | 0.19355 |
| 5509 | S6_90741324 | 6 | 90741324 | 28 | A | G | G | 0.69355 | A | 0.30645 | 13 | 0.41935 |
| 5510 | S6_90929666 | 6 | 90929666 | 188342 | A | G | A | 0.64516 | G | 0.35484 | 14 | 0.45161 |
| 5511 | S6_90929695 | 6 | 90929695 | 29 | C | T | C | 0.95161 | T | 0.04839 | 3 | 0.09677 |
| 5512 | S6_91201395 | 6 | 91201395 | 271700 | T | G | T | 0.72581 | G | 0.27419 | 11 | 0.35484 |
| 5513 | S6_91808895 | 6 | 91808895 | 607500 | C | A | C | 0.93548 | A | 0.06452 | 4 | 0.12903 |
| 5514 | S6_91808950 | 6 | 91808950 | 55 | C | T | C | 0.83871 | T | 0.16129 | 6 | 0.19355 |
| 5515 | S6_91952554 | 6 | 91952554 | 143604 | G | A | G | 0.85484 | A | 0.14516 | 5 | 0.16129 |
| 5516 | S6_92865093 | 6 | 92865093 | 912539 | G | A | G | 0.77419 | A | 0.22581 | 8 | 0.25806 |
| 5517 | S6_92865177 | 6 | 92865177 | 84 | A | C | C | 0.87097 | A | 0.12903 | 8 | 0.25806 |
| 5518 | S6_93201483 | 6 | 93201483 | 336306 | C | T | T | 0.69355 | C | 0.30645 | 9 | 0.29032 |
| 5519 | S6_93645126 | 6 | 93645126 | 443643 | C | T | C | 0.72581 | T | 0.27419 | 13 | 0.41935 |
| 5520 | S6_93853894 | 6 | 93853894 | 208768 | C | G | C | 0.93548 | G | 0.06452 | 4 | 0.12903 |
| 5521 | S6_95102253 | 6 | 95102253 | 1248359 | G | C | G | 0.80645 | C | 0.19355 | 8 | 0.25806 |
| 5522 | S6_95102262 | 6 | 95102262 | 9 | A | G | G | 0.56452 | A | 0.43548 | 17 | 0.54839 |
| 5523 | S6_95102312 | 6 | 95102312 | 50 | A | G | G | 0.54839 | A | 0.45161 | 16 | 0.51613 |
| 5524 | S6_95102367 | 6 | 95102367 | 55 | C | G | C | 0.93548 | G | 0.06452 | 4 | 0.12903 |
| 5525 | S6_95222839 | 6 | 95222839 | 120472 | A | C | A | 0.70968 | C | 0.29032 | 14 | 0.45161 |
| 5526 | S6_95222857 | 6 | 95222857 | 18 | C | T | C | 0.93548 | T | 0.06452 | 4 | 0.12903 |
| 5527 | S6_95222938 | 6 | 95222938 | 81 | G | A | G | 0.87097 | A | 0.12903 | 6 | 0.19355 |
| 5528 | S6_95222943 | 6 | 95222943 | 5 | C | T | C | 0.87097 | T | 0.12903 | 6 | 0.19355 |
| 5529 | S6_95222986 | 6 | 95222986 | 43 | C | T | C | 0.70968 | T | 0.29032 | 14 | 0.45161 |
| 5530 | S6_95223014 | 6 | 95223014 | 28 | G | A | G | 0.93548 | A | 0.06452 | 4 | 0.12903 |
| 5531 | S6_95223033 | 6 | 95223033 | 19 | C | T | C | 0.93548 | T | 0.06452 | 4 | 0.12903 |
| 5532 | S6_95225326 | 6 | 95225326 | 2293 | A | G | G | 0.53226 | A | 0.46774 | 13 | 0.41935 |
| 5533 | S6_95225453 | 6 | 95225453 | 127 | T | C | T | 0.95161 | C | 0.04839 | 3 | 0.09677 |
| 5534 | S6_95760042 | 6 | 95760042 | 534589 | T | C | T | 0.83871 | C | 0.16129 | 8 | 0.25806 |
| 5535 | S6_95947282 | 6 | 95947282 | 187240 | G | C | C | 0.87097 | G | 0.12903 | 4 | 0.12903 |
| 5536 | S6_95947287 | 6 | 95947287 | 5 | A | T | A | 0.93548 | T | 0.06452 | 4 | 0.12903 |
| 5537 | S6_95954366 | 6 | 95954366 | 7079 | C | T | C | 0.85484 | T | 0.14516 | 9 | 0.29032 |
| 5538 | S6_95954372 | 6 | 95954372 | 6 | C | T | C | 0.74194 | T | 0.25806 | 16 | 0.51613 |
| 5539 | S6_95954395 | 6 | 95954395 | 23 | C | T | C | 0.79032 | T | 0.20968 | 11 | 0.35484 |
| 5540 | S6_96004080 | 6 | 96004080 | 49685 | A | G | G | 0.79032 | A | 0.20968 | 7 | 0.22581 |
| 5541 | S6_96170317 | 6 | 96170317 | 166237 | A | G | A | 0.64516 | G | 0.35484 | 14 | 0.45161 |
| 5542 | S6_96170434 | 6 | 96170434 | 117 | A | G | G | 0.70968 | A | 0.29032 | 8 | 0.25806 |
| 5543 | S6_96170443 | 6 | 96170443 | 9 | T | C | C | 0.82258 | T | 0.17742 | 9 | 0.29032 |
| 5544 | S6_96170477 | 6 | 96170477 | 34 | G | A | A | 0.59677 | G | 0.40323 | 15 | 0.48387 |
| 5545 | S6_96265065 | 6 | 96265065 | 94588 | T | C | T | 0.91935 | C | 0.08065 | 3 | 0.09677 |
| 5546 | S6_96265075 | 6 | 96265075 | 10 | T | C | T | 0.82258 | C | 0.17742 | 9 | 0.29032 |
| 5547 | S6_96547960 | 6 | 96547960 | 282885 | C | T | C | 0.87097 | T | 0.12903 | 8 | 0.25806 |
| 5548 | S6_96553674 | 6 | 96553674 | 5714 | C | G | C | 0.87097 | G | 0.12903 | 8 | 0.25806 |
| 5549 | S6_96651349 | 6 | 96651349 | 97675 | G | A | G | 0.74194 | A | 0.25806 | 12 | 0.3871 |
| 5550 | S6_96693979 | 6 | 96693979 | 42630 | G | C | G | 0.85484 | C | 0.14516 | 7 | 0.22581 |
| 5551 | S6_96710458 | 6 | 96710458 | 16479 | C | T | C | 0.93548 | T | 0.06452 | 4 | 0.12903 |
| 5552 | S6_96710496 | 6 | 96710496 | 38 | A | G | A | 0.90323 | G | 0.09677 | 4 | 0.12903 |
| 5553 | S6_96892049 | 6 | 96892049 | 181553 | G | A | G | 0.85484 | A | 0.14516 | 9 | 0.29032 |
| 5554 | S6_96892174 | 6 | 96892174 | 125 | T | C | T | 0.95161 | C | 0.04839 | 3 | 0.09677 |
| 5555 | S6_96892184 | 6 | 96892184 | 10 | G | C | G | 0.59677 | C | 0.40323 | 19 | 0.6129 |
| 5556 | S6_96892195 | 6 | 96892195 | 11 | G | A | G | 0.95161 | A | 0.04839 | 3 | 0.09677 |
| 5557 | S6_96892198 | 6 | 96892198 | 3 | A | G | A | 0.95161 | G | 0.04839 | 3 | 0.09677 |
| 5558 | S6_96892211 | 6 | 96892211 | 13 | C | T | C | 0.59677 | T | 0.40323 | 19 | 0.6129 |
| 5559 | S6_97140260 | 6 | 97140260 | 248049 | C | A | C | 0.64516 | A | 0.35484 | 16 | 0.51613 |
| 5560 | S6_97204650 | 6 | 97204650 | 64390 | C | T | C | 0.77419 | T | 0.22581 | 12 | 0.3871 |

| Site number | SNP name | Chromosome | Physical position | Physical distance from the previous SNP | Reference allele | Alternative allele | Major allele | Major allele frequency | Minor allele | Minor allele frequency | Number heterozygous | Heterozygosity |
|---|---|---|---|---|---|---|---|---|---|---|---|---|
| 5561 | S6_97204667 | 6 | 97204667 | 17 | G | A | G | 0.93548 | A | 0.06452 | 4 | 0.12903 |
| 5562 | S6_97204701 | 6 | 97204701 | 34 | A | C | A | 0.93548 | C | 0.06452 | 4 | 0.12903 |
| 5563 | S6_97204758 | 6 | 97204758 | 57 | G | A | G | 0.93548 | A | 0.06452 | 4 | 0.12903 |
| 5564 | S6_97204803 | 6 | 97204803 | 45 | T | G | T | 0.64516 | G | 0.35484 | 14 | 0.45161 |
| 5565 | S6_97204806 | 6 | 97204806 | 3 | C | T | C | 0.77419 | T | 0.22581 | 12 | 0.3871 |
| 5566 | S6_97287756 | 6 | 97287756 | 82950 | G | A | G | 0.83871 | A | 0.16129 | 8 | 0.25806 |
| 5567 | S6_97287758 | 6 | 97287758 | 2 | G | C | C | 0.95161 | G | 0.04839 | 3 | 0.09677 |
| 5568 | S6_97287761 | 6 | 97287761 | 3 | C | T | C | 0.69355 | T | 0.30645 | 13 | 0.41935 |
| 5569 | S6_97287855 | 6 | 97287855 | 94 | T | C | T | 0.93548 | C | 0.06452 | 4 | 0.12903 |
| 5570 | S6_97287856 | 6 | 97287856 | 1 | G | C | G | 0.93548 | C | 0.06452 | 4 | 0.12903 |
| 5571 | S6_97287890 | 6 | 97287890 | 34 | A | G | G | 0.8871 | A | 0.1129 | 7 | 0.22581 |
| 5572 | S6_97901292 | 6 | 97901292 | 613402 | C | T | T | 0.91935 | C | 0.08065 | 5 | 0.16129 |
| 5573 | S6_97901407 | 6 | 97901407 | 115 | A | G | G | 0.51613 | A | 0.48387 | 12 | 0.3871 |
| 5574 | S6_97901415 | 6 | 97901415 | 8 | C | T | C | 0.87097 | T | 0.12903 | 8 | 0.25806 |
| 5575 | S6_98006061 | 6 | 98006061 | 104646 | C | T | C | 0.91935 | T | 0.08065 | 5 | 0.16129 |
| 5576 | S6_98218289 | 6 | 98218289 | 212228 | G | C | C | 0.83871 | G | 0.16129 | 6 | 0.19355 |
| 5577 | S6_98243614 | 6 | 98243614 | 25325 | C | T | T | 0.64516 | C | 0.35484 | 14 | 0.45161 |
| 5578 | S6_98302745 | 6 | 98302745 | 59131 | G | C | G | 0.80645 | C | 0.19355 | 10 | 0.32258 |
| 5579 | S6_98302785 | 6 | 98302785 | 40 | C | T | C | 0.85484 | T | 0.14516 | 7 | 0.22581 |
| 5580 | S6_98337064 | 6 | 98337064 | 34279 | C | T | C | 0.64516 | T | 0.35484 | 14 | 0.45161 |
| 5581 | S6_98337099 | 6 | 98337099 | 35 | C | T | C | 0.67742 | T | 0.32258 | 10 | 0.32258 |
| 5582 | S6_98337200 | 6 | 98337200 | 101 | G | A | G | 0.93548 | A | 0.06452 | 4 | 0.12903 |
| 5583 | S6_98337204 | 6 | 98337204 | 4 | C | A | A | 0.53226 | C | 0.46774 | 15 | 0.48387 |
| 5584 | S6_98337220 | 6 | 98337220 | 16 | T | C | C | 0.75806 | T | 0.24194 | 9 | 0.29032 |
| 5585 | S6_98337230 | 6 | 98337230 | 10 | T | C | T | 0.87097 | C | 0.12903 | 6 | 0.19355 |
| 5586 | S6_98337281 | 6 | 98337281 | 51 | G | A | G | 0.91935 | A | 0.08065 | 5 | 0.16129 |
| 5587 | S6_98437406 | 6 | 98437406 | 100125 | G | C | C | 0.59677 | G | 0.40323 | 11 | 0.35484 |
| 5588 | S6_98437434 | 6 | 98437434 | 28 | G | A | G | 0.70968 | A | 0.29032 | 12 | 0.3871 |
| 5589 | S6_98437458 | 6 | 98437458 | 24 | A | G | G | 0.66129 | A | 0.33871 | 13 | 0.41935 |
| 5590 | S6_98437538 | 6 | 98437538 | 80 | C | G | G | 0.66129 | C | 0.33871 | 13 | 0.41935 |
| 5591 | S6_98437595 | 6 | 98437595 | 57 | C | T | C | 0.70968 | T | 0.29032 | 14 | 0.45161 |
| 5592 | S6_98527405 | 6 | 98527405 | 89810 | T | C | T | 0.91935 | C | 0.08065 | 5 | 0.16129 |
| 5593 | S6_98527470 | 6 | 98527470 | 65 | C | T | C | 0.91935 | T | 0.08065 | 3 | 0.09677 |
| 5594 | S6_98527578 | 6 | 98527578 | 108 | G | A | A | 0.91935 | G | 0.08065 | 5 | 0.16129 |
| 5595 | S6_98527608 | 6 | 98527608 | 30 | C | T | C | 0.87097 | T | 0.12903 | 8 | 0.25806 |
| 5596 | S6_98527617 | 6 | 98527617 | 9 | C | T | C | 0.93548 | T | 0.06452 | 4 | 0.12903 |
| 5597 | S6_98547049 | 6 | 98547049 | 19432 | C | T | T | 0.80645 | C | 0.19355 | 8 | 0.25806 |
| 5598 | S6_98547051 | 6 | 98547051 | 2 | T | C | C | 0.80645 | T | 0.19355 | 8 | 0.25806 |
| 5599 | S6_98547101 | 6 | 98547101 | 50 | C | T | T | 0.62903 | C | 0.37097 | 11 | 0.35484 |
| 5600 | S6_98547164 | 6 | 98547164 | 63 | A | G | G | 0.80645 | A | 0.19355 | 8 | 0.25806 |
| 5601 | S6_98547169 | 6 | 98547169 | 5 | G | A | G | 0.8871 | A | 0.1129 | 5 | 0.16129 |
| 5602 | S6_98547176 | 6 | 98547176 | 7 | A | G | A | 0.90323 | G | 0.09677 | 6 | 0.19355 |
| 5603 | S6_98697471 | 6 | 98697471 | 150295 | T | A | T | 0.91935 | A | 0.08065 | 5 | 0.16129 |
| 5604 | S6_98697541 | 6 | 98697541 | 70 | C | T | C | 0.66129 | T | 0.33871 | 15 | 0.48387 |
| 5605 | S6_99579924 | 6 | 99579924 | 882383 | C | G | G | 0.69355 | C | 0.30645 | 17 | 0.54839 |
| 5606 | S6_99611983 | 6 | 99611983 | 32059 | G | T | G | 0.85484 | T | 0.14516 | 9 | 0.29032 |
| 5607 | S6_99810422 | 6 | 99810422 | 198439 | G | T | G | 0.8871 | T | 0.1129 | 7 | 0.22581 |
| 5608 | S6_99810443 | 6 | 99810443 | 21 | A | G | A | 0.91935 | G | 0.08065 | 5 | 0.16129 |
| 5609 | S6_99810449 | 6 | 99810449 | 6 | C | A | C | 0.93548 | A | 0.06452 | 4 | 0.12903 |
| 5610 | S6_99810560 | 6 | 99810560 | 111 | C | T | C | 0.54839 | T | 0.45161 | 22 | 0.70968 |
| 5611 | S6_100123069 | 6 | 100123069 | 312509 | T | C | T | 0.95161 | C | 0.04839 | 3 | 0.09677 |
| 5612 | S6_100475944 | 6 | 100475944 | 352875 | T | C | T | 0.80645 | C | 0.19355 | 10 | 0.32258 |
| 5613 | S6_100476133 | 6 | 100476133 | 189 | C | A | C | 0.91935 | A | 0.08065 | 5 | 0.16129 |
| 5614 | S6_100618479 | 6 | 100618479 | 142346 | C | G | C | 0.93548 | G | 0.06452 | 4 | 0.12903 |

| Site number | SNP name | Chromosome | Physical position | Physical distance from the previous SNP | Reference allele | Alternative allele | Major allele | Major allele frequency | Minor allele | Minor allele frequency | Number heterozygous | Heterozygosity |
|---|---|---|---|---|---|---|---|---|---|---|---|---|
| 5615 | S6_101456904 | 6 | 101456904 | 838425 | G | T | G | 0.90323 | T | 0.09677 | 4 | 0.12903 |
| 5616 | S6_101678182 | 6 | 101678182 | 221278 | T | G | T | 0.85484 | G | 0.14516 | 7 | 0.22581 |
| 5617 | S6_101710723 | 6 | 101710723 | 32541 | T | A | T | 0.82258 | A | 0.17742 | 9 | 0.29032 |
| 5618 | S6_101710737 | 6 | 101710737 | 14 | T | C | C | 0.74194 | T | 0.25806 | 14 | 0.45161 |
| 5619 | S6_101710759 | 6 | 101710759 | 22 | A | G | A | 0.93548 | G | 0.06452 | 4 | 0.12903 |
| 5620 | S6_101851312 | 6 | 101851312 | 140553 | T | C | T | 0.95161 | C | 0.04839 | 3 | 0.09677 |
| 5621 | S6_101892238 | 6 | 101892238 | 40926 | A | C | A | 0.87097 | C | 0.12903 | 6 | 0.19355 |
| 5622 | S6_102483710 | 6 | 102483710 | 591472 | C | A | C | 0.79032 | A | 0.20968 | 11 | 0.35484 |
| 5623 | S6_102483711 | 6 | 102483711 | 1 | G | A | A | 0.51613 | G | 0.48387 | 16 | 0.51613 |
| 5624 | S6_102483720 | 6 | 102483720 | 9 | G | A | G | 0.90323 | A | 0.09677 | 6 | 0.19355 |
| 5625 | S6_102483830 | 6 | 102483830 | 110 | G | A | G | 0.83871 | A | 0.16129 | 10 | 0.32258 |
| 5626 | S6_102586230 | 6 | 102586230 | 102400 | G | A | G | 0.83871 | A | 0.16129 | 8 | 0.25806 |
| 5627 | S6_103138921 | 6 | 103138921 | 552691 | G | T | G | 0.90323 | T | 0.09677 | 6 | 0.19355 |
| 5628 | S6_103138924 | 6 | 103138924 | 3 | A | G | A | 0.90323 | G | 0.09677 | 6 | 0.19355 |
| 5629 | S6_103138955 | 6 | 103138955 | 31 | T | C | T | 0.90323 | C | 0.09677 | 6 | 0.19355 |
| 5630 | S6_103138962 | 6 | 103138962 | 7 | G | A | G | 0.90323 | A | 0.09677 | 6 | 0.19355 |
| 5631 | S6_103139090 | 6 | 103139090 | 128 | A | G | A | 0.90323 | G | 0.09677 | 6 | 0.19355 |
| 5632 | S6_103170834 | 6 | 103170834 | 31744 | T | C | T | 0.82258 | C | 0.17742 | 11 | 0.35484 |
| 5633 | S6_103213574 | 6 | 103213574 | 42740 | G | A | A | 0.74194 | G | 0.25806 | 14 | 0.45161 |
| 5634 | S6_103213604 | 6 | 103213604 | 30 | T | C | C | 0.90323 | T | 0.09677 | 4 | 0.12903 |
| 5635 | S6_103213635 | 6 | 103213635 | 31 | G | C | C | 0.75806 | G | 0.24194 | 13 | 0.41935 |
| 5636 | S6_103213707 | 6 | 103213707 | 72 | G | A | G | 0.87097 | A | 0.12903 | 6 | 0.19355 |
| 5637 | S6_103355046 | 6 | 103355046 | 141339 | G | T | G | 0.95161 | T | 0.04839 | 3 | 0.09677 |
| 5638 | S6_103355081 | 6 | 103355081 | 35 | G | A | G | 0.79032 | A | 0.20968 | 13 | 0.41935 |
| 5639 | S6_103452405 | 6 | 103452405 | 97324 | C | T | C | 0.93548 | T | 0.06452 | 4 | 0.12903 |
| 5640 | S6_103452420 | 6 | 103452420 | 15 | C | G | C | 0.82258 | G | 0.17742 | 7 | 0.22581 |
| 5641 | S6_103452480 | 6 | 103452480 | 60 | A | G | G | 0.69355 | A | 0.30645 | 15 | 0.48387 |
| 5642 | S6_103452518 | 6 | 103452518 | 38 | T | G | T | 0.91935 | G | 0.08065 | 3 | 0.09677 |
| 5643 | S6_103452657 | 6 | 103452657 | 139 | T | C | T | 0.66129 | C | 0.33871 | 13 | 0.41935 |
| 5644 | S6_103465503 | 6 | 103465503 | 12846 | G | T | G | 0.83871 | T | 0.16129 | 8 | 0.25806 |
| 5645 | S6_103465509 | 6 | 103465509 | 6 | T | C | T | 0.93548 | C | 0.06452 | 4 | 0.12903 |
| 5646 | S6_103465545 | 6 | 103465545 | 36 | A | C | A | 0.67742 | C | 0.32258 | 14 | 0.45161 |
| 5647 | S6_103465602 | 6 | 103465602 | 57 | T | C | T | 0.72581 | C | 0.27419 | 13 | 0.41935 |
| 5648 | S6_103465607 | 6 | 103465607 | 5 | C | T | C | 0.64516 | T | 0.35484 | 12 | 0.3871 |
| 5649 | S6_103694015 | 6 | 103694015 | 228408 | T | C | T | 0.82258 | C | 0.17742 | 7 | 0.22581 |
| 5650 | S6_103694025 | 6 | 103694025 | 10 | T | C | C | 0.74194 | T | 0.25806 | 10 | 0.32258 |
| 5651 | S6_103694033 | 6 | 103694033 | 8 | C | T | C | 0.80645 | T | 0.19355 | 8 | 0.25806 |
| 5652 | S6_103694034 | 6 | 103694034 | 1 | G | A | G | 0.8871 | A | 0.1129 | 7 | 0.22581 |
| 5653 | S6_103694065 | 6 | 103694065 | 31 | A | G | G | 0.64516 | A | 0.35484 | 12 | 0.3871 |
| 5654 | S6_103694147 | 6 | 103694147 | 82 | G | C | C | 0.74194 | G | 0.25806 | 10 | 0.32258 |
| 5655 | S6_103975858 | 6 | 103975858 | 281711 | C | T | C | 0.95161 | T | 0.04839 | 3 | 0.09677 |
| 5656 | S6_104090683 | 6 | 104090683 | 114825 | C | T | C | 0.85484 | T | 0.14516 | 9 | 0.29032 |
| 5657 | S6_104090866 | 6 | 104090866 | 183 | A | G | A | 0.93548 | G | 0.06452 | 4 | 0.12903 |
| 5658 | S6_104090884 | 6 | 104090884 | 18 | A | G | G | 0.58065 | A | 0.41935 | 16 | 0.51613 |
| 5659 | S6_104098562 | 6 | 104098562 | 7678 | G | A | G | 0.6129 | A | 0.3871 | 16 | 0.51613 |
| 5660 | S6_104106851 | 6 | 104106851 | 8289 | C | T | C | 0.64516 | T | 0.35484 | 14 | 0.45161 |
| 5661 | S6_104317985 | 6 | 104317985 | 211134 | G | C | G | 0.59677 | C | 0.40323 | 15 | 0.48387 |
| 5662 | S6_104317990 | 6 | 104317990 | 5 | C | T | C | 0.59677 | T | 0.40323 | 15 | 0.48387 |
| 5663 | S6_104318078 | 6 | 104318078 | 88 | A | G | A | 0.59677 | G | 0.40323 | 15 | 0.48387 |
| 5664 | S6_104318183 | 6 | 104318183 | 105 | A | G | A | 0.59677 | G | 0.40323 | 15 | 0.48387 |
| 5665 | S6_104558192 | 6 | 104558192 | 240009 | C | T | C | 0.64516 | T | 0.35484 | 14 | 0.45161 |
| 5666 | S6_104558203 | 6 | 104558203 | 11 | A | G | G | 0.64516 | A | 0.35484 | 16 | 0.51613 |
| 5667 | S6_104840820 | 6 | 104840820 | 282617 | T | C | C | 0.8871 | T | 0.1129 | 5 | 0.16129 |
| 5668 | S6_105077113 | 6 | 105077113 | 236293 | G | A | G | 0.93548 | A | 0.06452 | 4 | 0.12903 |

| Site number | SNP name | Chromosome | Physical position | Physical distance from the previous SNP | Reference allele | Alternative allele | Major allele | Major allele frequency | Minor allele | Minor allele frequency | Number heterozygous | Heterozygosity |
|---|---|---|---|---|---|---|---|---|---|---|---|---|
| 5669 | S6_105079086 | 6 | 105079086 | 1973 | C | T | T | 0.8871 | C | 0.1129 | 7 | 0.22581 |
| 5670 | S6_105079256 | 6 | 105079256 | 170 | T | G | T | 0.64516 | G | 0.35484 | 18 | 0.58065 |
| 5671 | S6_105345055 | 6 | 105345055 | 265799 | A | G | A | 0.85484 | G | 0.14516 | 7 | 0.22581 |
| 5672 | S6_105345057 | 6 | 105345057 | 2 | G | A | G | 0.83871 | A | 0.16129 | 8 | 0.25806 |
| 5673 | S6_105468473 | 6 | 105468473 | 123416 | C | T | C | 0.54839 | T | 0.45161 | 12 | 0.3871 |
| 5674 | S6_105468489 | 6 | 105468489 | 16 | T | G | G | 0.8871 | T | 0.1129 | 7 | 0.22581 |
| 5675 | S6_105468500 | 6 | 105468500 | 11 | C | T | C | 0.54839 | T | 0.45161 | 12 | 0.3871 |
| 5676 | S6_105468510 | 6 | 105468510 | 10 | C | G | C | 0.54839 | G | 0.45161 | 12 | 0.3871 |
| 5677 | S6_105468513 | 6 | 105468513 | 3 | T | C | T | 0.54839 | C | 0.45161 | 12 | 0.3871 |
| 5678 | S6_105468543 | 6 | 105468543 | 30 | G | A | G | 0.54839 | A | 0.45161 | 12 | 0.3871 |
| 5679 | S6_105468547 | 6 | 105468547 | 4 | A | G | A | 0.56452 | G | 0.43548 | 15 | 0.48387 |
| 5680 | S6_105468604 | 6 | 105468604 | 57 | T | G | G | 0.58065 | T | 0.41935 | 14 | 0.45161 |
| 5681 | S6_105468611 | 6 | 105468611 | 7 | C | T | C | 0.87097 | T | 0.12903 | 8 | 0.25806 |
| 5682 | S6_105468678 | 6 | 105468678 | 67 | T | C | T | 0.54839 | C | 0.45161 | 12 | 0.3871 |
| 5683 | S6_105468721 | 6 | 105468721 | 43 | C | A | A | 0.91935 | C | 0.08065 | 5 | 0.16129 |
| 5684 | S6_105513411 | 6 | 105513411 | 44690 | C | T | C | 0.95161 | T | 0.04839 | 3 | 0.09677 |
| 5685 | S6_105513496 | 6 | 105513496 | 85 | A | G | A | 0.87097 | G | 0.12903 | 4 | 0.12903 |
| 5686 | S6_105550559 | 6 | 105550559 | 37063 | C | T | C | 0.91935 | T | 0.08065 | 3 | 0.09677 |
| 5687 | S6_105550702 | 6 | 105550702 | 143 | C | T | C | 0.95161 | T | 0.04839 | 3 | 0.09677 |
| 5688 | S6_105985765 | 6 | 105985765 | 435063 | G | A | G | 0.93548 | A | 0.06452 | 4 | 0.12903 |
| 5689 | S6_105985870 | 6 | 105985870 | 105 | G | A | G | 0.93548 | A | 0.06452 | 4 | 0.12903 |
| 5690 | S6_105985926 | 6 | 105985926 | 56 | C | T | C | 0.93548 | T | 0.06452 | 4 | 0.12903 |
| 5691 | S6_105985955 | 6 | 105985955 | 29 | T | C | T | 0.93548 | C | 0.06452 | 4 | 0.12903 |
| 5692 | S6_105985957 | 6 | 105985957 | 2 | C | A | C | 0.90323 | A | 0.09677 | 4 | 0.12903 |
| 5693 | S6_106009264 | 6 | 106009264 | 23307 | G | A | A | 0.58065 | G | 0.41935 | 20 | 0.64516 |
| 5694 | S6_106323029 | 6 | 106323029 | 313765 | A | C | A | 0.95161 | C | 0.04839 | 3 | 0.09677 |
| 5695 | S6_106423668 | 6 | 106423668 | 100639 | C | T | C | 0.8871 | T | 0.1129 | 5 | 0.16129 |
| 5696 | S6_106639426 | 6 | 106639426 | 215758 | C | T | C | 0.91935 | T | 0.08065 | 3 | 0.09677 |
| 5697 | S6_106639451 | 6 | 106639451 | 25 | C | T | C | 0.93548 | T | 0.06452 | 4 | 0.12903 |
| 5698 | S6_106639653 | 6 | 106639653 | 202 | C | T | C | 0.91935 | T | 0.08065 | 3 | 0.09677 |
| 5699 | S6_106748130 | 6 | 106748130 | 108477 | G | A | G | 0.80645 | A | 0.19355 | 10 | 0.32258 |
| 5700 | S6_106748131 | 6 | 106748131 | 1 | T | C | T | 0.72581 | C | 0.27419 | 9 | 0.29032 |
| 5701 | S6_106748356 | 6 | 106748356 | 225 | C | T | C | 0.8871 | T | 0.1129 | 5 | 0.16129 |
| 5702 | S6_106748977 | 6 | 106748977 | 621 | T | A | A | 0.75806 | T | 0.24194 | 13 | 0.41935 |
| 5703 | S6_106749051 | 6 | 106749051 | 74 | G | A | G | 0.80645 | A | 0.19355 | 10 | 0.32258 |
| 5704 | S6_106749078 | 6 | 106749078 | 27 | C | T | C | 0.8871 | T | 0.1129 | 5 | 0.16129 |
| 5705 | S6_106749160 | 6 | 106749160 | 82 | T | C | T | 0.85484 | C | 0.14516 | 7 | 0.22581 |
| 5706 | S6_106749169 | 6 | 106749169 | 9 | G | C | G | 0.8871 | C | 0.1129 | 5 | 0.16129 |
| 5707 | S6_106749191 | 6 | 106749191 | 22 | G | A | G | 0.75806 | A | 0.24194 | 13 | 0.41935 |
| 5708 | S6_107210713 | 6 | 107210713 | 461522 | C | T | C | 0.82258 | T | 0.17742 | 9 | 0.29032 |
| 5709 | S6_107210760 | 6 | 107210760 | 47 | T | A | T | 0.82258 | A | 0.17742 | 9 | 0.29032 |
| 5710 | S6_107547476 | 6 | 107547476 | 336716 | T | C | C | 0.80645 | T | 0.19355 | 10 | 0.32258 |
| 5711 | S6_107547606 | 6 | 107547606 | 130 | C | A | C | 0.87097 | A | 0.12903 | 8 | 0.25806 |
| 5712 | S6_107959798 | 6 | 107959798 | 412192 | G | A | G | 0.80645 | A | 0.19355 | 12 | 0.3871 |
| 5713 | S6_107959818 | 6 | 107959818 | 20 | C | T | C | 0.80645 | T | 0.19355 | 10 | 0.32258 |
| 5714 | S6_107959826 | 6 | 107959826 | 8 | T | C | T | 0.95161 | C | 0.04839 | 3 | 0.09677 |
| 5715 | S6_107959877 | 6 | 107959877 | 51 | C | G | C | 0.91935 | G | 0.08065 | 3 | 0.09677 |
| 5716 | S6_108042662 | 6 | 108042662 | 82785 | C | T | C | 0.85484 | T | 0.14516 | 9 | 0.29032 |
| 5717 | S6_108042783 | 6 | 108042783 | 121 | C | T | C | 0.80645 | T | 0.19355 | 10 | 0.32258 |
| 5718 | S6_108042804 | 6 | 108042804 | 21 | A | G | A | 0.80645 | G | 0.19355 | 10 | 0.32258 |
| 5719 | S6_108042824 | 6 | 108042824 | 20 | G | T | G | 0.83871 | T | 0.16129 | 8 | 0.25806 |
| 5720 | S6_108042829 | 6 | 108042829 | 5 | G | T | G | 0.80645 | T | 0.19355 | 8 | 0.25806 |
| 5721 | S6_108042834 | 6 | 108042834 | 5 | T | G | T | 0.79032 | G | 0.20968 | 9 | 0.29032 |
| 5722 | S6_108455164 | 6 | 108455164 | 412330 | A | G | A | 0.95161 | G | 0.04839 | 3 | 0.09677 |

| Site number | SNP name | Chromosome | Physical position | Physical distance from the previous SNP | Reference allele | Alternative allele | Major allele | Major allele frequency | Minor allele | Minor allele frequency | Number heterozygous | Heterozygosity |
|---|---|---|---|---|---|---|---|---|---|---|---|---|
| 5723 | S6_108455182 | 6 | 108455182 | 18 | G | A | G | 0.95161 | A | 0.04839 | 3 | 0.09677 |
| 5724 | S6_109254842 | 6 | 109254842 | 799660 | A | G | G | 0.72581 | A | 0.27419 | 15 | 0.48387 |
| 5725 | S6_109380993 | 6 | 109380993 | 126151 | C | T | C | 0.69355 | T | 0.30645 | 13 | 0.41935 |
| 5726 | S6_109436505 | 6 | 109436505 | 55512 | T | G | T | 0.95161 | G | 0.04839 | 3 | 0.09677 |
| 5727 | S6_109635685 | 6 | 109635685 | 199180 | C | A | C | 0.53226 | A | 0.46774 | 17 | 0.54839 |
| 5728 | S6_109635744 | 6 | 109635744 | 59 | G | A | A | 0.5 | G | 0.5 | 19 | 0.6129 |
| 5729 | S6_110003242 | 6 | 110003242 | 367498 | A | C | A | 0.62903 | C | 0.37097 | 15 | 0.48387 |
| 5730 | S6_110495861 | 6 | 110495861 | 492619 | T | C | T | 0.90323 | C | 0.09677 | 6 | 0.19355 |
| 5731 | S6_110597818 | 6 | 110597818 | 101957 | G | C | C | 0.53226 | G | 0.46774 | 15 | 0.48387 |
| 5732 | S6_110597860 | 6 | 110597860 | 42 | C | T | C | 0.95161 | T | 0.04839 | 3 | 0.09677 |
| 5733 | S6_110597862 | 6 | 110597862 | 2 | C | A | A | 0.53226 | C | 0.46774 | 15 | 0.48387 |
| 5734 | S6_110597868 | 6 | 110597868 | 6 | G | A | G | 0.93548 | A | 0.06452 | 4 | 0.12903 |
| 5735 | S6_110597882 | 6 | 110597882 | 14 | T | C | C | 0.58065 | T | 0.41935 | 16 | 0.51613 |
| 5736 | S6_110663569 | 6 | 110663569 | 65687 | G | A | G | 0.70968 | A | 0.29032 | 12 | 0.3871 |
| 5737 | S6_110972607 | 6 | 110972607 | 309038 | G | A | G | 0.95161 | A | 0.04839 | 3 | 0.09677 |
| 5738 | S6_111014673 | 6 | 111014673 | 42066 | G | C | G | 0.80645 | C | 0.19355 | 10 | 0.32258 |
| 5739 | S6_111014698 | 6 | 111014698 | 25 | T | A | A | 0.90323 | T | 0.09677 | 6 | 0.19355 |
| 5740 | S6_111014739 | 6 | 111014739 | 41 | G | T | T | 0.90323 | G | 0.09677 | 6 | 0.19355 |
| 5741 | S6_111014741 | 6 | 111014741 | 2 | G | C | G | 0.85484 | C | 0.14516 | 7 | 0.22581 |
| 5742 | S6_111014890 | 6 | 111014890 | 149 | T | C | C | 0.90323 | T | 0.09677 | 6 | 0.19355 |
| 5743 | S6_111037897 | 6 | 111037897 | 23007 | C | A | C | 0.93548 | A | 0.06452 | 2 | 0.06452 |
| 5744 | S6_111037974 | 6 | 111037974 | 77 | A | G | G | 0.51613 | A | 0.48387 | 14 | 0.45161 |
| 5745 | S6_111174734 | 6 | 111174734 | 136760 | C | T | C | 0.95161 | T | 0.04839 | 1 | 0.03226 |
| 5746 | S6_111174818 | 6 | 111174818 | 84 | A | G | A | 0.51613 | G | 0.48387 | 14 | 0.45161 |
| 5747 | S6_111174863 | 6 | 111174863 | 45 | G | A | G | 0.74194 | A | 0.25806 | 12 | 0.3871 |
| 5748 | S6_111305014 | 6 | 111305014 | 130151 | G | A | G | 0.77419 | A | 0.22581 | 12 | 0.3871 |
| 5749 | S6_111305090 | 6 | 111305090 | 76 | C | T | C | 0.93548 | T | 0.06452 | 2 | 0.06452 |
| 5750 | S6_111952446 | 6 | 111952446 | 647356 | G | C | G | 0.6129 | C | 0.3871 | 14 | 0.45161 |
| 5751 | S6_112000521 | 6 | 112000521 | 48075 | G | A | G | 0.93548 | A | 0.06452 | 4 | 0.12903 |
| 5752 | S6_112000617 | 6 | 112000617 | 96 | C | T | C | 0.91935 | T | 0.08065 | 5 | 0.16129 |
| 5753 | S6_112089187 | 6 | 112089187 | 88570 | C | T | C | 0.95161 | T | 0.04839 | 3 | 0.09677 |
| 5754 | S6_112121635 | 6 | 112121635 | 32448 | C | T | C | 0.70968 | T | 0.29032 | 12 | 0.3871 |
| 5755 | S6_112200574 | 6 | 112200574 | 78939 | C | T | C | 0.95161 | T | 0.04839 | 1 | 0.03226 |
| 5756 | S6_112200620 | 6 | 112200620 | 46 | G | C | G | 0.93548 | C | 0.06452 | 4 | 0.12903 |
| 5757 | S6_112200663 | 6 | 112200663 | 43 | C | T | C | 0.90323 | T | 0.09677 | 4 | 0.12903 |
| 5758 | S6_112200783 | 6 | 112200783 | 120 | G | T | T | 0.66129 | G | 0.33871 | 17 | 0.54839 |
| 5759 | S6_112298108 | 6 | 112298108 | 97325 | A | G | A | 0.83871 | G | 0.16129 | 8 | 0.25806 |
| 5760 | S6_112298112 | 6 | 112298112 | 4 | A | G | A | 0.83871 | G | 0.16129 | 8 | 0.25806 |
| 5761 | S6_112298113 | 6 | 112298113 | 1 | A | C | A | 0.83871 | C | 0.16129 | 8 | 0.25806 |
| 5762 | S6_112298185 | 6 | 112298185 | 72 | G | A | G | 0.74194 | A | 0.25806 | 8 | 0.25806 |
| 5763 | S6_112308126 | 6 | 112308126 | 9941 | G | C | G | 0.93548 | C | 0.06452 | 4 | 0.12903 |
| 5764 | S6_112308149 | 6 | 112308149 | 23 | T | G | T | 0.93548 | G | 0.06452 | 4 | 0.12903 |
| 5765 | S6_112308164 | 6 | 112308164 | 15 | T | C | T | 0.93548 | C | 0.06452 | 4 | 0.12903 |
| 5766 | S6_112308193 | 6 | 112308193 | 29 | C | T | C | 0.82258 | T | 0.17742 | 7 | 0.22581 |
| 5767 | S6_112308280 | 6 | 112308280 | 87 | G | A | G | 0.87097 | A | 0.12903 | 6 | 0.19355 |
| 5768 | S6_112364446 | 6 | 112364446 | 56166 | G | A | G | 0.91935 | A | 0.08065 | 3 | 0.09677 |
| 5769 | S6_112364469 | 6 | 112364469 | 23 | T | C | T | 0.90323 | C | 0.09677 | 6 | 0.19355 |
| 5770 | S6_112468480 | 6 | 112468480 | 104011 | A | G | A | 0.93548 | G | 0.06452 | 4 | 0.12903 |
| 5771 | S6_112468622 | 6 | 112468622 | 142 | C | T | C | 0.87097 | T | 0.12903 | 8 | 0.25806 |
| 5772 | S6_112604879 | 6 | 112604879 | 136257 | T | G | T | 0.79032 | G | 0.20968 | 11 | 0.35484 |
| 5773 | S6_112604900 | 6 | 112604900 | 21 | G | A | G | 0.79032 | A | 0.20968 | 11 | 0.35484 |
| 5774 | S6_112604967 | 6 | 112604967 | 67 | A | G | A | 0.79032 | G | 0.20968 | 11 | 0.35484 |
| 5775 | S6_112604981 | 6 | 112604981 | 14 | C | T | C | 0.79032 | T | 0.20968 | 11 | 0.35484 |
| 5776 | S6_112605071 | 6 | 112605071 | 90 | A | G | A | 0.79032 | G | 0.20968 | 11 | 0.35484 |

| Site number | SNP name | Chromosome | Physical position | Physical distance from the previous SNP | Reference allele | Alternative allele | Major allele | Major allele frequency | Minor allele | Minor allele frequency | Number heterozygous | Heterozygosity |
|---|---|---|---|---|---|---|---|---|---|---|---|---|
| 5777 | S6_112605075 | 6 | 112605075 | 4 | A | G | A | 0.79032 | G | 0.20968 | 11 | 0.35484 |
| 5778 | S6_112605104 | 6 | 112605104 | 29 | T | C | T | 0.79032 | C | 0.20968 | 11 | 0.35484 |
| 5779 | S6_112647891 | 6 | 112647891 | 42787 | G | A | G | 0.67742 | A | 0.32258 | 12 | 0.3871 |
| 5780 | S6_112647980 | 6 | 112647980 | 89 | C | T | C | 0.90323 | T | 0.09677 | 6 | 0.19355 |
| 5781 | S6_112691646 | 6 | 112691646 | 43666 | G | A | G | 0.93548 | A | 0.06452 | 4 | 0.12903 |
| 5782 | S6_112691818 | 6 | 112691818 | 172 | G | T | T | 0.8871 | G | 0.1129 | 7 | 0.22581 |
| 5783 | S6_112724607 | 6 | 112724607 | 32789 | T | C | T | 0.77419 | C | 0.22581 | 10 | 0.32258 |
| 5784 | S6_113359093 | 6 | 113359093 | 634486 | T | C | T | 0.93548 | C | 0.06452 | 4 | 0.12903 |
| 5785 | S6_113359278 | 6 | 113359278 | 185 | A | G | A | 0.93548 | G | 0.06452 | 4 | 0.12903 |
| 5786 | S6_113614144 | 6 | 113614144 | 254866 | C | T | C | 0.80645 | T | 0.19355 | 10 | 0.32258 |
| 5787 | S6_113614175 | 6 | 113614175 | 31 | T | C | T | 0.74194 | C | 0.25806 | 12 | 0.3871 |
| 5788 | S6_114211477 | 6 | 114211477 | 597302 | G | A | G | 0.80645 | A | 0.19355 | 10 | 0.32258 |
| 5789 | S6_114321047 | 6 | 114321047 | 109570 | G | T | G | 0.74194 | T | 0.25806 | 14 | 0.45161 |
| 5790 | S6_114321136 | 6 | 114321136 | 89 | T | C | T | 0.74194 | C | 0.25806 | 14 | 0.45161 |
| 5791 | S6_114414154 | 6 | 114414154 | 93018 | C | T | C | 0.82258 | T | 0.17742 | 9 | 0.29032 |
| 5792 | S6_114613275 | 6 | 114613275 | 199121 | A | C | A | 0.64516 | C | 0.35484 | 16 | 0.51613 |
| 5793 | S6_114613281 | 6 | 114613281 | 6 | C | A | C | 0.95161 | A | 0.04839 | 3 | 0.09677 |
| 5794 | S6_114613351 | 6 | 114613351 | 70 | T | C | T | 0.64516 | C | 0.35484 | 16 | 0.51613 |
| 5795 | S6_114613360 | 6 | 114613360 | 9 | G | A | G | 0.59677 | A | 0.40323 | 19 | 0.6129 |
| 5796 | S6_115093744 | 6 | 115093744 | 480384 | G | A | G | 0.75806 | A | 0.24194 | 13 | 0.41935 |
| 5797 | S6_115093878 | 6 | 115093878 | 134 | C | T | C | 0.87097 | T | 0.12903 | 8 | 0.25806 |
| 5798 | S6_115093911 | 6 | 115093911 | 33 | G | A | G | 0.66129 | A | 0.33871 | 9 | 0.29032 |
| 5799 | S6_115093916 | 6 | 115093916 | 5 | G | A | G | 0.91935 | A | 0.08065 | 5 | 0.16129 |
| 5800 | S6_115093944 | 6 | 115093944 | 28 | C | T | C | 0.93548 | T | 0.06452 | 4 | 0.12903 |
| 5801 | S6_115922508 | 6 | 115922508 | 828564 | G | C | G | 0.83871 | C | 0.16129 | 10 | 0.32258 |
| 5802 | S6_116047930 | 6 | 116047930 | 125422 | C | T | C | 0.93548 | T | 0.06452 | 4 | 0.12903 |
| 5803 | S6_116204202 | 6 | 116204202 | 156272 | A | C | A | 0.91935 | C | 0.08065 | 5 | 0.16129 |
| 5804 | S6_116204290 | 6 | 116204290 | 88 | A | G | G | 0.58065 | A | 0.41935 | 16 | 0.51613 |
| 5805 | S6_116254119 | 6 | 116254119 | 49829 | C | T | C | 0.93548 | T | 0.06452 | 2 | 0.06452 |
| 5806 | S6_116254133 | 6 | 116254133 | 14 | A | C | A | 0.90323 | C | 0.09677 | 4 | 0.12903 |
| 5807 | S6_116267646 | 6 | 116267646 | 13513 | A | C | A | 0.95161 | C | 0.04839 | 1 | 0.03226 |
| 5808 | S6_116532541 | 6 | 116532541 | 264895 | T | C | T | 0.91935 | C | 0.08065 | 5 | 0.16129 |
| 5809 | S6_116532618 | 6 | 116532618 | 77 | T | C | T | 0.79032 | C | 0.20968 | 11 | 0.35484 |
| 5810 | S6_116532627 | 6 | 116532627 | 9 | A | T | T | 0.91935 | A | 0.08065 | 3 | 0.09677 |
| 5811 | S6_116532632 | 6 | 116532632 | 5 | A | G | A | 0.91935 | G | 0.08065 | 3 | 0.09677 |
| 5812 | S6_116532656 | 6 | 116532656 | 24 | G | A | G | 0.8871 | A | 0.1129 | 7 | 0.22581 |
| 5813 | S6_116854893 | 6 | 116854893 | 322237 | T | A | T | 0.8871 | A | 0.1129 | 7 | 0.22581 |
| 5814 | S6_117073102 | 6 | 117073102 | 218209 | G | A | G | 0.74194 | A | 0.25806 | 16 | 0.51613 |
| 5815 | S6_117557119 | 6 | 117557119 | 484017 | C | A | C | 0.67742 | A | 0.32258 | 16 | 0.51613 |
| 5816 | S7_328852 | 7 | 328852 | 0 | C | G | C | 0.95161 | G | 0.04839 | 3 | 0.09677 |
| 5817 | S7_328870 | 7 | 328870 | 18 | C | T | C | 0.95161 | T | 0.04839 | 3 | 0.09677 |
| 5818 | S7_419131 | 7 | 419131 | 90261 | C | T | C | 0.85484 | T | 0.14516 | 9 | 0.29032 |
| 5819 | S7_429123 | 7 | 429123 | 9992 | G | T | G | 0.93548 | T | 0.06452 | 4 | 0.12903 |
| 5820 | S7_537902 | 7 | 537902 | 108779 | C | T | C | 0.91935 | T | 0.08065 | 5 | 0.16129 |
| 5821 | S7_724475 | 7 | 724475 | 186573 | A | G | A | 0.8871 | G | 0.1129 | 7 | 0.22581 |
| 5822 | S7_905986 | 7 | 905986 | 181511 | T | C | T | 0.93548 | C | 0.06452 | 4 | 0.12903 |
| 5823 | S7_906073 | 7 | 906073 | 87 | G | A | G | 0.93548 | A | 0.06452 | 4 | 0.12903 |
| 5824 | S7_906081 | 7 | 906081 | 8 | T | C | T | 0.51613 | C | 0.48387 | 12 | 0.3871 |
| 5825 | S7_906086 | 7 | 906086 | 5 | G | A | G | 0.93548 | A | 0.06452 | 4 | 0.12903 |
| 5826 | S7_906088 | 7 | 906088 | 2 | G | A | G | 0.93548 | A | 0.06452 | 4 | 0.12903 |
| 5827 | S7_906093 | 7 | 906093 | 5 | G | A | G | 0.85484 | A | 0.14516 | 7 | 0.22581 |
| 5828 | S7_906168 | 7 | 906168 | 75 | G | C | G | 0.93548 | C | 0.06452 | 4 | 0.12903 |
| 5829 | S7_906186 | 7 | 906186 | 18 | G | A | G | 0.93548 | A | 0.06452 | 4 | 0.12903 |
| 5830 | S7_1117815 | 7 | 1117815 | 211629 | G | A | G | 0.66129 | A | 0.33871 | 15 | 0.48387 |

| Site number | SNP name | Chromosome | Physical position | Physical distance from the previous SNP | Reference allele | Alternative allele | Major allele | Major allele frequency | Minor allele | Minor allele frequency | Number heterozygous | Heterozygosity |
|---|---|---|---|---|---|---|---|---|---|---|---|---|
| 5831 | S7_1267488 | 7 | 1267488 | 149673 | G | A | G | 0.83871 | A | 0.16129 | 8 | 0.25806 |
| 5832 | S7_1292283 | 7 | 1292283 | 24795 | A | G | A | 0.91935 | G | 0.08065 | 5 | 0.16129 |
| 5833 | S7_1292405 | 7 | 1292405 | 122 | A | C | A | 0.83871 | C | 0.16129 | 10 | 0.32258 |
| 5834 | S7_1292414 | 7 | 1292414 | 9 | G | T | G | 0.83871 | T | 0.16129 | 10 | 0.32258 |
| 5835 | S7_1292415 | 7 | 1292415 | 1 | T | G | T | 0.82258 | G | 0.17742 | 11 | 0.35484 |
| 5836 | S7_1292419 | 7 | 1292419 | 4 | A | G | A | 0.83871 | G | 0.16129 | 10 | 0.32258 |
| 5837 | S7_1474765 | 7 | 1474765 | 182346 | A | G | G | 0.69355 | A | 0.30645 | 15 | 0.48387 |
| 5838 | S7_1474887 | 7 | 1474887 | 122 | C | A | C | 0.83871 | A | 0.16129 | 8 | 0.25806 |
| 5839 | S7_1474896 | 7 | 1474896 | 9 | G | C | G | 0.8871 | C | 0.1129 | 7 | 0.22581 |
| 5840 | S7_1474930 | 7 | 1474930 | 34 | A | T | A | 0.83871 | T | 0.16129 | 8 | 0.25806 |
| 5841 | S7_1474957 | 7 | 1474957 | 27 | A | G | A | 0.83871 | G | 0.16129 | 8 | 0.25806 |
| 5842 | S7_1739696 | 7 | 1739696 | 264739 | A | G | A | 0.91935 | G | 0.08065 | 5 | 0.16129 |
| 5843 | S7_1739732 | 7 | 1739732 | 36 | T | A | A | 0.83871 | T | 0.16129 | 10 | 0.32258 |
| 5844 | S7_1739739 | 7 | 1739739 | 7 | T | C | T | 0.51613 | C | 0.48387 | 20 | 0.64516 |
| 5845 | S7_1739863 | 7 | 1739863 | 124 | G | A | G | 0.91935 | A | 0.08065 | 5 | 0.16129 |
| 5846 | S7_1803115 | 7 | 1803115 | 63252 | T | C | T | 0.95161 | C | 0.04839 | 3 | 0.09677 |
| 5847 | S7_1803145 | 7 | 1803145 | 30 | C | G | C | 0.95161 | G | 0.04839 | 3 | 0.09677 |
| 5848 | S7_1803318 | 7 | 1803318 | 173 | T | C | T | 0.79032 | C | 0.20968 | 11 | 0.35484 |
| 5849 | S7_1875999 | 7 | 1875999 | 72681 | C | T | C | 0.87097 | T | 0.12903 | 8 | 0.25806 |
| 5850 | S7_1876047 | 7 | 1876047 | 48 | C | T | C | 0.90323 | T | 0.09677 | 4 | 0.12903 |
| 5851 | S7_1989125 | 7 | 1989125 | 113078 | C | T | C | 0.95161 | T | 0.04839 | 1 | 0.03226 |
| 5852 | S7_2350656 | 7 | 2350656 | 361531 | G | A | G | 0.82258 | A | 0.17742 | 7 | 0.22581 |
| 5853 | S7_2350710 | 7 | 2350710 | 54 | T | C | T | 0.51613 | C | 0.48387 | 14 | 0.45161 |
| 5854 | S7_2579911 | 7 | 2579911 | 229201 | C | T | C | 0.87097 | T | 0.12903 | 8 | 0.25806 |
| 5855 | S7_2579982 | 7 | 2579982 | 71 | T | C | C | 0.87097 | T | 0.12903 | 6 | 0.19355 |
| 5856 | S7_2864490 | 7 | 2864490 | 284508 | G | A | G | 0.90323 | A | 0.09677 | 4 | 0.12903 |
| 5857 | S7_2864505 | 7 | 2864505 | 15 | G | T | G | 0.70968 | T | 0.29032 | 10 | 0.32258 |
| 5858 | S7_2864531 | 7 | 2864531 | 26 | C | T | C | 0.95161 | T | 0.04839 | 1 | 0.03226 |
| 5859 | S7_3102292 | 7 | 3102292 | 237761 | A | G | A | 0.91935 | G | 0.08065 | 5 | 0.16129 |
| 5860 | S7_3102397 | 7 | 3102397 | 105 | A | G | A | 0.79032 | G | 0.20968 | 13 | 0.41935 |
| 5861 | S7_3542196 | 7 | 3542196 | 439799 | G | A | G | 0.77419 | A | 0.22581 | 12 | 0.3871 |
| 5862 | S7_3574627 | 7 | 3574627 | 32431 | C | T | C | 0.79032 | T | 0.20968 | 7 | 0.22581 |
| 5863 | S7_3574673 | 7 | 3574673 | 46 | C | T | C | 0.85484 | T | 0.14516 | 5 | 0.16129 |
| 5864 | S7_3574713 | 7 | 3574713 | 40 | A | T | A | 0.80645 | T | 0.19355 | 12 | 0.3871 |
| 5865 | S7_3831322 | 7 | 3831322 | 256609 | C | G | G | 0.54839 | C | 0.45161 | 16 | 0.51613 |
| 5866 | S7_3912101 | 7 | 3912101 | 80779 | A | G | A | 0.85484 | G | 0.14516 | 9 | 0.29032 |
| 5867 | S7_4133234 | 7 | 4133234 | 221133 | T | A | T | 0.82258 | A | 0.17742 | 9 | 0.29032 |
| 5868 | S7_4133235 | 7 | 4133235 | 1 | A | T | A | 0.82258 | T | 0.17742 | 9 | 0.29032 |
| 5869 | S7_4221138 | 7 | 4221138 | 87903 | C | T | C | 0.87097 | T | 0.12903 | 8 | 0.25806 |
| 5870 | S7_4221285 | 7 | 4221285 | 147 | A | G | G | 0.82258 | A | 0.17742 | 9 | 0.29032 |
| 5871 | S7_4543076 | 7 | 4543076 | 321791 | C | T | C | 0.87097 | T | 0.12903 | 8 | 0.25806 |
| 5872 | S7_4688217 | 7 | 4688217 | 145141 | A | C | C | 0.67742 | A | 0.32258 | 14 | 0.45161 |
| 5873 | S7_4756628 | 7 | 4756628 | 68411 | T | C | T | 0.82258 | C | 0.17742 | 9 | 0.29032 |
| 5874 | S7_5032503 | 7 | 5032503 | 275875 | C | A | C | 0.87097 | A | 0.12903 | 6 | 0.19355 |
| 5875 | S7_5032540 | 7 | 5032540 | 37 | G | A | G | 0.87097 | A | 0.12903 | 6 | 0.19355 |
| 5876 | S7_5032599 | 7 | 5032599 | 59 | T | C | T | 0.87097 | C | 0.12903 | 6 | 0.19355 |
| 5877 | S7_5032640 | 7 | 5032640 | 41 | A | G | A | 0.87097 | G | 0.12903 | 6 | 0.19355 |
| 5878 | S7_5032672 | 7 | 5032672 | 32 | T | C | T | 0.87097 | C | 0.12903 | 6 | 0.19355 |
| 5879 | S7_5066559 | 7 | 5066559 | 33887 | G | C | G | 0.8871 | C | 0.1129 | 5 | 0.16129 |
| 5880 | S7_5353194 | 7 | 5353194 | 286635 | G | A | G | 0.91935 | A | 0.08065 | 5 | 0.16129 |
| 5881 | S7_5512556 | 7 | 5512556 | 159362 | A | C | A | 0.93548 | C | 0.06452 | 4 | 0.12903 |
| 5882 | S7_7606444 | 7 | 7606444 | 2093888 | C | T | C | 0.90323 | T | 0.09677 | 6 | 0.19355 |
| 5883 | S7_7793181 | 7 | 7793181 | 186737 | T | C | T | 0.95161 | C | 0.04839 | 3 | 0.09677 |
| 5884 | S7_8649342 | 7 | 8649342 | 856161 | T | C | C | 0.69355 | T | 0.30645 | 11 | 0.35484 |

| Site number | SNP name | Chromosome | Physical position | Physical distance from the previous SNP | Reference allele | Alternative allele | Major allele | Major allele frequency | Minor allele | Minor allele frequency | Number heterozygous | Heterozygosity |
|---|---|---|---|---|---|---|---|---|---|---|---|---|
| 5885 | S7_8649491 | 7 | 8649491 | 149 | A | G | G | 0.54839 | A | 0.45161 | 16 | 0.51613 |
| 5886 | S7_9000113 | 7 | 9000113 | 350622 | G | A | G | 0.85484 | A | 0.14516 | 9 | 0.29032 |
| 5887 | S7_9000118 | 7 | 9000118 | 5 | T | C | T | 0.64516 | C | 0.35484 | 16 | 0.51613 |
| 5888 | S7_9000121 | 7 | 9000121 | 3 | G | A | G | 0.64516 | A | 0.35484 | 16 | 0.51613 |
| 5889 | S7_9000143 | 7 | 9000143 | 22 | G | T | T | 0.59677 | G | 0.40323 | 17 | 0.54839 |
| 5890 | S7_9000196 | 7 | 9000196 | 53 | A | G | A | 0.64516 | G | 0.35484 | 16 | 0.51613 |
| 5891 | S7_9000207 | 7 | 9000207 | 11 | T | C | T | 0.64516 | C | 0.35484 | 16 | 0.51613 |
| 5892 | S7_9000217 | 7 | 9000217 | 10 | T | C | T | 0.64516 | C | 0.35484 | 16 | 0.51613 |
| 5893 | S7_9000242 | 7 | 9000242 | 25 | A | T | A | 0.64516 | T | 0.35484 | 16 | 0.51613 |
| 5894 | S7_9000287 | 7 | 9000287 | 45 | C | T | C | 0.8871 | T | 0.1129 | 5 | 0.16129 |
| 5895 | S7_9000299 | 7 | 9000299 | 12 | G | C | G | 0.64516 | C | 0.35484 | 16 | 0.51613 |
| 5896 | S7_9322719 | 7 | 9322719 | 322420 | C | T | T | 0.51613 | C | 0.48387 | 14 | 0.45161 |
| 5897 | S7_9322804 | 7 | 9322804 | 85 | C | G | C | 0.91935 | G | 0.08065 | 3 | 0.09677 |
| 5898 | S7_9322871 | 7 | 9322871 | 67 | G | A | G | 0.72581 | A | 0.27419 | 13 | 0.41935 |
| 5899 | S7_9342405 | 7 | 9342405 | 19534 | C | T | C | 0.91935 | T | 0.08065 | 5 | 0.16129 |
| 5900 | S7_9342531 | 7 | 9342531 | 126 | T | C | T | 0.90323 | C | 0.09677 | 6 | 0.19355 |
| 5901 | S7_9913002 | 7 | 9913002 | 570471 | G | A | G | 0.83871 | A | 0.16129 | 8 | 0.25806 |
| 5902 | S7_9913063 | 7 | 9913063 | 61 | C | A | C | 0.93548 | A | 0.06452 | 4 | 0.12903 |
| 5903 | S7_9913081 | 7 | 9913081 | 18 | T | G | T | 0.8871 | G | 0.1129 | 7 | 0.22581 |
| 5904 | S7_9913208 | 7 | 9913208 | 127 | T | C | T | 0.70968 | C | 0.29032 | 12 | 0.3871 |
| 5905 | S7_9938564 | 7 | 9938564 | 25356 | T | C | T | 0.93548 | C | 0.06452 | 4 | 0.12903 |
| 5906 | S7_9938629 | 7 | 9938629 | 65 | C | A | C | 0.79032 | A | 0.20968 | 9 | 0.29032 |
| 5907 | S7_10525192 | 7 | 10525192 | 586563 | G | A | G | 0.91935 | A | 0.08065 | 5 | 0.16129 |
| 5908 | S7_10585995 | 7 | 10585995 | 60803 | T | C | C | 0.69355 | T | 0.30645 | 15 | 0.48387 |
| 5909 | S7_10586100 | 7 | 10586100 | 105 | T | G | T | 0.95161 | G | 0.04839 | 3 | 0.09677 |
| 5910 | S7_10586184 | 7 | 10586184 | 84 | G | A | G | 0.95161 | A | 0.04839 | 3 | 0.09677 |
| 5911 | S7_10754505 | 7 | 10754505 | 168321 | G | A | G | 0.80645 | A | 0.19355 | 12 | 0.3871 |
| 5912 | S7_10754536 | 7 | 10754536 | 31 | C | T | C | 0.95161 | T | 0.04839 | 3 | 0.09677 |
| 5913 | S7_10914165 | 7 | 10914165 | 159629 | G | A | G | 0.91935 | A | 0.08065 | 5 | 0.16129 |
| 5914 | S7_10914319 | 7 | 10914319 | 154 | C | T | C | 0.8871 | T | 0.1129 | 5 | 0.16129 |
| 5915 | S7_11130241 | 7 | 11130241 | 215922 | T | C | T | 0.8871 | C | 0.1129 | 7 | 0.22581 |
| 5916 | S7_11130253 | 7 | 11130253 | 12 | T | A | T | 0.95161 | A | 0.04839 | 3 | 0.09677 |
| 5917 | S7_11196296 | 7 | 11196296 | 66043 | G | A | G | 0.90323 | A | 0.09677 | 6 | 0.19355 |
| 5918 | S7_11196345 | 7 | 11196345 | 49 | G | A | G | 0.90323 | A | 0.09677 | 6 | 0.19355 |
| 5919 | S7_11196387 | 7 | 11196387 | 42 | G | A | G | 0.95161 | A | 0.04839 | 3 | 0.09677 |
| 5920 | S7_12167347 | 7 | 12167347 | 970960 | C | T | C | 0.91935 | T | 0.08065 | 5 | 0.16129 |
| 5921 | S7_12167360 | 7 | 12167360 | 13 | G | C | C | 0.87097 | G | 0.12903 | 8 | 0.25806 |
| 5922 | S7_12167433 | 7 | 12167433 | 73 | A | G | G | 0.87097 | A | 0.12903 | 8 | 0.25806 |
| 5923 | S7_12167457 | 7 | 12167457 | 24 | C | A | C | 0.85484 | A | 0.14516 | 7 | 0.22581 |
| 5924 | S7_12167544 | 7 | 12167544 | 87 | T | C | T | 0.8871 | C | 0.1129 | 5 | 0.16129 |
| 5925 | S7_12167559 | 7 | 12167559 | 15 | C | T | T | 0.83871 | C | 0.16129 | 8 | 0.25806 |
| 5926 | S7_12608048 | 7 | 12608048 | 440489 | G | T | T | 0.66129 | G | 0.33871 | 11 | 0.35484 |
| 5927 | S7_12608176 | 7 | 12608176 | 128 | C | T | C | 0.95161 | T | 0.04839 | 3 | 0.09677 |
| 5928 | S7_12892526 | 7 | 12892526 | 284350 | G | C | C | 0.6129 | G | 0.3871 | 12 | 0.3871 |
| 5929 | S7_12892611 | 7 | 12892611 | 85 | C | T | C | 0.79032 | T | 0.20968 | 13 | 0.41935 |
| 5930 | S7_12892680 | 7 | 12892680 | 69 | T | C | T | 0.53226 | C | 0.46774 | 19 | 0.6129 |
| 5931 | S7_13033635 | 7 | 13033635 | 140955 | T | C | C | 0.53226 | T | 0.46774 | 13 | 0.41935 |
| 5932 | S7_13033681 | 7 | 13033681 | 46 | C | T | T | 0.53226 | C | 0.46774 | 13 | 0.41935 |
| 5933 | S7_13033736 | 7 | 13033736 | 55 | G | A | A | 0.93548 | G | 0.06452 | 4 | 0.12903 |
| 5934 | S7_13033744 | 7 | 13033744 | 8 | G | A | A | 0.93548 | G | 0.06452 | 4 | 0.12903 |
| 5935 | S7_13033831 | 7 | 13033831 | 87 | A | G | G | 0.54839 | A | 0.45161 | 14 | 0.45161 |
| 5936 | S7_13033841 | 7 | 13033841 | 10 | G | A | G | 0.80645 | A | 0.19355 | 8 | 0.25806 |
| 5937 | S7_13033845 | 7 | 13033845 | 4 | A | G | G | 0.53226 | A | 0.46774 | 13 | 0.41935 |
| 5938 | S7_13033851 | 7 | 13033851 | 6 | T | A | T | 0.83871 | A | 0.16129 | 8 | 0.25806 |

| Site number | SNP name | Chromosome | Physical position | Physical distance from the previous SNP | Reference allele | Alternative allele | Major allele | Major allele frequency | Minor allele | Minor allele frequency | Number heterozygous | Heterozygosity |
|---|---|---|---|---|---|---|---|---|---|---|---|---|
| 5939 | S7_13858120 | 7 | 13858120 | 824269 | G | A | G | 0.8871 | A | 0.1129 | 5 | 0.16129 |
| 5940 | S7_13858218 | 7 | 13858218 | 98 | G | A | G | 0.8871 | A | 0.1129 | 5 | 0.16129 |
| 5941 | S7_13858239 | 7 | 13858239 | 21 | G | C | G | 0.8871 | C | 0.1129 | 5 | 0.16129 |
| 5942 | S7_13955592 | 7 | 13955592 | 97353 | C | T | C | 0.85484 | T | 0.14516 | 7 | 0.22581 |
| 5943 | S7_13955608 | 7 | 13955608 | 16 | G | C | C | 0.90323 | G | 0.09677 | 6 | 0.19355 |
| 5944 | S7_13999444 | 7 | 13999444 | 43836 | A | G | A | 0.93548 | G | 0.06452 | 4 | 0.12903 |
| 5945 | S7_13999541 | 7 | 13999541 | 97 | G | A | G | 0.75806 | A | 0.24194 | 11 | 0.35484 |
| 5946 | S7_13999649 | 7 | 13999649 | 108 | G | C | G | 0.72581 | C | 0.27419 | 13 | 0.41935 |
| 5947 | S7_14008612 | 7 | 14008612 | 8963 | C | A | C | 0.8871 | A | 0.1129 | 5 | 0.16129 |
| 5948 | S7_14571473 | 7 | 14571473 | 562861 | A | T | A | 0.8871 | T | 0.1129 | 5 | 0.16129 |
| 5949 | S7_14571508 | 7 | 14571508 | 35 | G | A | G | 0.59677 | A | 0.40323 | 15 | 0.48387 |
| 5950 | S7_14571575 | 7 | 14571575 | 67 | C | T | C | 0.59677 | T | 0.40323 | 15 | 0.48387 |
| 5951 | S7_15569901 | 7 | 15569901 | 998326 | C | T | C | 0.90323 | T | 0.09677 | 6 | 0.19355 |
| 5952 | S7_15569902 | 7 | 15569902 | 1 | G | A | G | 0.77419 | A | 0.22581 | 10 | 0.32258 |
| 5953 | S7_15862978 | 7 | 15862978 | 293076 | T | C | T | 0.59677 | C | 0.40323 | 11 | 0.35484 |
| 5954 | S7_15862979 | 7 | 15862979 | 1 | G | A | G | 0.91935 | A | 0.08065 | 5 | 0.16129 |
| 5955 | S7_16011383 | 7 | 16011383 | 148404 | G | A | G | 0.85484 | A | 0.14516 | 5 | 0.16129 |
| 5956 | S7_16011389 | 7 | 16011389 | 6 | G | C | G | 0.91935 | C | 0.08065 | 5 | 0.16129 |
| 5957 | S7_16011402 | 7 | 16011402 | 13 | A | G | A | 0.79032 | G | 0.20968 | 11 | 0.35484 |
| 5958 | S7_16011538 | 7 | 16011538 | 136 | A | G | G | 0.80645 | A | 0.19355 | 10 | 0.32258 |
| 5959 | S7_16011568 | 7 | 16011568 | 30 | T | C | T | 0.95161 | C | 0.04839 | 3 | 0.09677 |
| 5960 | S7_16461347 | 7 | 16461347 | 449779 | G | A | G | 0.85484 | A | 0.14516 | 7 | 0.22581 |
| 5961 | S7_16624834 | 7 | 16624834 | 163487 | C | A | C | 0.6129 | A | 0.3871 | 14 | 0.45161 |
| 5962 | S7_16625046 | 7 | 16625046 | 212 | C | A | C | 0.90323 | A | 0.09677 | 6 | 0.19355 |
| 5963 | S7_17344830 | 7 | 17344830 | 719784 | C | T | C | 0.91935 | T | 0.08065 | 5 | 0.16129 |
| 5964 | S7_17463555 | 7 | 17463555 | 118725 | G | C | G | 0.91935 | C | 0.08065 | 5 | 0.16129 |
| 5965 | S7_17659491 | 7 | 17659491 | 195936 | C | G | C | 0.82258 | G | 0.17742 | 7 | 0.22581 |
| 5966 | S7_17876477 | 7 | 17876477 | 216986 | G | A | G | 0.95161 | A | 0.04839 | 3 | 0.09677 |
| 5967 | S7_17876631 | 7 | 17876631 | 154 | C | T | C | 0.91935 | T | 0.08065 | 5 | 0.16129 |
| 5968 | S7_18284899 | 7 | 18284899 | 408268 | C | T | C | 0.80645 | T | 0.19355 | 8 | 0.25806 |
| 5969 | S7_18624053 | 7 | 18624053 | 339154 | T | G | T | 0.90323 | G | 0.09677 | 6 | 0.19355 |
| 5970 | S7_18624060 | 7 | 18624060 | 7 | G | C | C | 0.53226 | G | 0.46774 | 17 | 0.54839 |
| 5971 | S7_18806290 | 7 | 18806290 | 182230 | C | T | C | 0.95161 | T | 0.04839 | 3 | 0.09677 |
| 5972 | S7_18806377 | 7 | 18806377 | 87 | C | T | C | 0.91935 | T | 0.08065 | 5 | 0.16129 |
| 5973 | S7_19307151 | 7 | 19307151 | 500774 | T | C | T | 0.79032 | C | 0.20968 | 9 | 0.29032 |
| 5974 | S7_19731510 | 7 | 19731510 | 424359 | G | A | G | 0.93548 | A | 0.06452 | 4 | 0.12903 |
| 5975 | S7_20336418 | 7 | 20336418 | 604908 | A | C | A | 0.80645 | C | 0.19355 | 6 | 0.19355 |
| 5976 | S7_20336547 | 7 | 20336547 | 129 | G | A | G | 0.93548 | A | 0.06452 | 4 | 0.12903 |
| 5977 | S7_20336587 | 7 | 20336587 | 40 | C | T | C | 0.90323 | T | 0.09677 | 6 | 0.19355 |
| 5978 | S7_20477521 | 7 | 20477521 | 140934 | T | C | T | 0.83871 | C | 0.16129 | 8 | 0.25806 |
| 5979 | S7_20477605 | 7 | 20477605 | 84 | T | C | T | 0.79032 | C | 0.20968 | 9 | 0.29032 |
| 5980 | S7_21489789 | 7 | 21489789 | 1012184 | G | A | G | 0.75806 | A | 0.24194 | 9 | 0.29032 |
| 5981 | S7_21550520 | 7 | 21550520 | 60731 | C | G | C | 0.95161 | G | 0.04839 | 3 | 0.09677 |
| 5982 | S7_21550553 | 7 | 21550553 | 33 | T | C | T | 0.91935 | C | 0.08065 | 5 | 0.16129 |
| 5983 | S7_22149731 | 7 | 22149731 | 599178 | G | A | G | 0.87097 | A | 0.12903 | 6 | 0.19355 |
| 5984 | S7_22859443 | 7 | 22859443 | 709712 | T | G | T | 0.6129 | G | 0.3871 | 14 | 0.45161 |
| 5985 | S7_23400449 | 7 | 23400449 | 541006 | G | A | G | 0.95161 | A | 0.04839 | 3 | 0.09677 |
| 5986 | S7_23400539 | 7 | 23400539 | 90 | T | C | C | 0.95161 | T | 0.04839 | 3 | 0.09677 |
| 5987 | S7_23400542 | 7 | 23400542 | 3 | G | A | G | 0.87097 | A | 0.12903 | 4 | 0.12903 |
| 5988 | S7_23633512 | 7 | 23633512 | 232970 | C | A | C | 0.85484 | A | 0.14516 | 7 | 0.22581 |
| 5989 | S7_23633516 | 7 | 23633516 | 4 | C | T | C | 0.93548 | T | 0.06452 | 4 | 0.12903 |
| 5990 | S7_23715636 | 7 | 23715636 | 82120 | C | T | C | 0.87097 | T | 0.12903 | 8 | 0.25806 |
| 5991 | S7_23715643 | 7 | 23715643 | 7 | G | A | G | 0.83871 | A | 0.16129 | 6 | 0.19355 |
| 5992 | S7_24103729 | 7 | 24103729 | 388086 | T | C | T | 0.87097 | C | 0.12903 | 4 | 0.12903 |

| Site number | SNP name | Chromosome | Physical position | Physical distance from the previous SNP | Reference allele | Alternative allele | Major allele | Major allele frequency | Minor allele | Minor allele frequency | Number heterozygous | Heterozygosity |
|---|---|---|---|---|---|---|---|---|---|---|---|---|
| 5993 | S7_24103934 | 7 | 24103934 | 205 | G | C | G | 0.8871 | C | 0.1129 | 5 | 0.16129 |
| 5994 | S7_24385638 | 7 | 24385638 | 281704 | G | A | G | 0.95161 | A | 0.04839 | 3 | 0.09677 |
| 5995 | S7_24385686 | 7 | 24385686 | 48 | T | C | C | 0.82258 | T | 0.17742 | 11 | 0.35484 |
| 5996 | S7_24385806 | 7 | 24385806 | 120 | C | G | G | 0.77419 | C | 0.22581 | 12 | 0.3871 |
| 5997 | S7_24385861 | 7 | 24385861 | 55 | C | T | T | 0.82258 | C | 0.17742 | 11 | 0.35484 |
| 5998 | S7_24688882 | 7 | 24688882 | 303021 | G | T | G | 0.93548 | T | 0.06452 | 4 | 0.12903 |
| 5999 | S7_25122301 | 7 | 25122301 | 433419 | C | T | T | 0.67742 | C | 0.32258 | 14 | 0.45161 |
| 6000 | S7_25122321 | 7 | 25122321 | 20 | T | C | C | 0.74194 | T | 0.25806 | 8 | 0.25806 |
| 6001 | S7_25122402 | 7 | 25122402 | 81 | G | A | G | 0.70968 | A | 0.29032 | 12 | 0.3871 |
| 6002 | S7_25122432 | 7 | 25122432 | 30 | C | T | T | 0.67742 | C | 0.32258 | 14 | 0.45161 |
| 6003 | S7_26156578 | 7 | 26156578 | 1034146 | C | T | C | 0.87097 | T | 0.12903 | 6 | 0.19355 |
| 6004 | S7_26943711 | 7 | 26943711 | 787133 | C | T | C | 0.93548 | T | 0.06452 | 4 | 0.12903 |
| 6005 | S7_27024475 | 7 | 27024475 | 80764 | C | T | C | 0.85484 | T | 0.14516 | 9 | 0.29032 |
| 6006 | S7_27128171 | 7 | 27128171 | 103696 | C | T | C | 0.8871 | T | 0.1129 | 5 | 0.16129 |
| 6007 | S7_27128206 | 7 | 27128206 | 35 | A | G | A | 0.91935 | G | 0.08065 | 3 | 0.09677 |
| 6008 | S7_27668506 | 7 | 27668506 | 540300 | C | T | C | 0.91935 | T | 0.08065 | 3 | 0.09677 |
| 6009 | S7_27927760 | 7 | 27927760 | 259254 | C | T | C | 0.8871 | T | 0.1129 | 5 | 0.16129 |
| 6010 | S7_27927877 | 7 | 27927877 | 117 | C | T | C | 0.83871 | T | 0.16129 | 6 | 0.19355 |
| 6011 | S7_27949167 | 7 | 27949167 | 21290 | G | A | G | 0.6129 | A | 0.3871 | 16 | 0.51613 |
| 6012 | S7_27949232 | 7 | 27949232 | 65 | C | T | C | 0.6129 | T | 0.3871 | 16 | 0.51613 |
| 6013 | S7_27976324 | 7 | 27976324 | 27092 | C | T | C | 0.82258 | T | 0.17742 | 11 | 0.35484 |
| 6014 | S7_27976329 | 7 | 27976329 | 5 | G | A | G | 0.82258 | A | 0.17742 | 11 | 0.35484 |
| 6015 | S7_27976368 | 7 | 27976368 | 39 | A | C | A | 0.82258 | C | 0.17742 | 11 | 0.35484 |
| 6016 | S7_28152140 | 7 | 28152140 | 175772 | C | T | C | 0.77419 | T | 0.22581 | 14 | 0.45161 |
| 6017 | S7_28152156 | 7 | 28152156 | 16 | C | T | C | 0.64516 | T | 0.35484 | 16 | 0.51613 |
| 6018 | S7_28222466 | 7 | 28222466 | 70310 | T | C | T | 0.72581 | C | 0.27419 | 15 | 0.48387 |
| 6019 | S7_28876413 | 7 | 28876413 | 653947 | C | T | C | 0.54839 | T | 0.45161 | 18 | 0.58065 |
| 6020 | S7_30137704 | 7 | 30137704 | 1261291 | G | A | A | 0.66129 | G | 0.33871 | 19 | 0.6129 |
| 6021 | S7_30137723 | 7 | 30137723 | 19 | G | A | A | 0.66129 | G | 0.33871 | 19 | 0.6129 |
| 6022 | S7_30137763 | 7 | 30137763 | 40 | C | T | T | 0.69355 | C | 0.30645 | 17 | 0.54839 |
| 6023 | S7_30198588 | 7 | 30198588 | 60825 | A | G | A | 0.64516 | G | 0.35484 | 18 | 0.58065 |
| 6024 | S7_30198704 | 7 | 30198704 | 116 | A | G | A | 0.59677 | G | 0.40323 | 17 | 0.54839 |
| 6025 | S7_30628342 | 7 | 30628342 | 429638 | G | A | G | 0.8871 | A | 0.1129 | 5 | 0.16129 |
| 6026 | S7_30628353 | 7 | 30628353 | 11 | A | G | A | 0.8871 | G | 0.1129 | 5 | 0.16129 |
| 6027 | S7_30628455 | 7 | 30628455 | 102 | G | C | G | 0.8871 | C | 0.1129 | 5 | 0.16129 |
| 6028 | S7_30775852 | 7 | 30775852 | 147397 | G | C | G | 0.87097 | C | 0.12903 | 8 | 0.25806 |
| 6029 | S7_30785966 | 7 | 30785966 | 10114 | A | G | G | 0.72581 | A | 0.27419 | 9 | 0.29032 |
| 6030 | S7_31963683 | 7 | 31963683 | 1177717 | T | C | T | 0.85484 | C | 0.14516 | 9 | 0.29032 |
| 6031 | S7_32258934 | 7 | 32258934 | 295251 | A | G | A | 0.59677 | G | 0.40323 | 21 | 0.67742 |
| 6032 | S7_32477961 | 7 | 32477961 | 219027 | C | T | C | 0.67742 | T | 0.32258 | 16 | 0.51613 |
| 6033 | S7_32478038 | 7 | 32478038 | 77 | A | T | A | 0.64516 | T | 0.35484 | 18 | 0.58065 |
| 6034 | S7_32478162 | 7 | 32478162 | 124 | G | C | C | 0.5 | G | 0.5 | 17 | 0.54839 |
| 6035 | S7_33132239 | 7 | 33132239 | 654077 | C | T | C | 0.59677 | T | 0.40323 | 17 | 0.54839 |
| 6036 | S7_33141464 | 7 | 33141464 | 9225 | T | C | T | 0.70968 | C | 0.29032 | 12 | 0.3871 |
| 6037 | S7_33741401 | 7 | 33741401 | 599937 | A | C | A | 0.90323 | C | 0.09677 | 4 | 0.12903 |
| 6038 | S7_33880309 | 7 | 33880309 | 138908 | G | A | G | 0.8871 | A | 0.1129 | 5 | 0.16129 |
| 6039 | S7_33880328 | 7 | 33880328 | 19 | A | T | A | 0.90323 | T | 0.09677 | 6 | 0.19355 |
| 6040 | S7_33880357 | 7 | 33880357 | 29 | G | A | G | 0.8871 | A | 0.1129 | 7 | 0.22581 |
| 6041 | S7_34490930 | 7 | 34490930 | 610573 | C | G | C | 0.8871 | G | 0.1129 | 7 | 0.22581 |
| 6042 | S7_34960101 | 7 | 34960101 | 469171 | C | T | C | 0.91935 | T | 0.08065 | 5 | 0.16129 |
| 6043 | S7_34960235 | 7 | 34960235 | 134 | G | T | G | 0.95161 | T | 0.04839 | 3 | 0.09677 |
| 6044 | S7_34960243 | 7 | 34960243 | 8 | G | A | G | 0.93548 | A | 0.06452 | 4 | 0.12903 |
| 6045 | S7_34960258 | 7 | 34960258 | 15 | T | A | T | 0.93548 | A | 0.06452 | 4 | 0.12903 |
| 6046 | S7_34960301 | 7 | 34960301 | 43 | G | A | G | 0.64516 | A | 0.35484 | 12 | 0.3871 |

| Site number | SNP name | Chromosome | Physical position | Physical distance from the previous SNP | Reference allele | Alternative allele | Major allele | Major allele frequency | Minor allele | Minor allele frequency | Number heterozygous | Heterozygosity |
|---|---|---|---|---|---|---|---|---|---|---|---|---|
| 6047 | S7_34960327 | 7 | 34960327 | 26 | A | G | A | 0.93548 | G | 0.06452 | 4 | 0.12903 |
| 6048 | S7_35454611 | 7 | 35454611 | 494284 | G | A | A | 0.83871 | G | 0.16129 | 10 | 0.32258 |
| 6049 | S7_35454671 | 7 | 35454671 | 60 | A | G | A | 0.56452 | G | 0.43548 | 19 | 0.6129 |
| 6050 | S7_35454787 | 7 | 35454787 | 116 | A | G | A | 0.75806 | G | 0.24194 | 13 | 0.41935 |
| 6051 | S7_35498368 | 7 | 35498368 | 43581 | C | G | G | 0.54839 | C | 0.45161 | 18 | 0.58065 |
| 6052 | S7_35498537 | 7 | 35498537 | 169 | T | C | T | 0.74194 | C | 0.25806 | 16 | 0.51613 |
| 6053 | S7_35949415 | 7 | 35949415 | 450878 | A | G | A | 0.77419 | G | 0.22581 | 10 | 0.32258 |
| 6054 | S7_35949458 | 7 | 35949458 | 43 | A | C | A | 0.62903 | C | 0.37097 | 11 | 0.35484 |
| 6055 | S7_35949474 | 7 | 35949474 | 16 | T | A | T | 0.87097 | A | 0.12903 | 8 | 0.25806 |
| 6056 | S7_35949490 | 7 | 35949490 | 16 | C | T | C | 0.90323 | T | 0.09677 | 6 | 0.19355 |
| 6057 | S7_35949523 | 7 | 35949523 | 33 | C | T | C | 0.77419 | T | 0.22581 | 10 | 0.32258 |
| 6058 | S7_35949635 | 7 | 35949635 | 112 | G | A | G | 0.87097 | A | 0.12903 | 8 | 0.25806 |
| 6059 | S7_35949655 | 7 | 35949655 | 20 | A | G | A | 0.8871 | G | 0.1129 | 5 | 0.16129 |
| 6060 | S7_36216398 | 7 | 36216398 | 266743 | T | C | C | 0.93548 | T | 0.06452 | 4 | 0.12903 |
| 6061 | S7_36216406 | 7 | 36216406 | 8 | A | G | A | 0.91935 | G | 0.08065 | 5 | 0.16129 |
| 6062 | S7_36216599 | 7 | 36216599 | 193 | G | A | G | 0.74194 | A | 0.25806 | 10 | 0.32258 |
| 6063 | S7_36216612 | 7 | 36216612 | 13 | C | T | C | 0.72581 | T | 0.27419 | 11 | 0.35484 |
| 6064 | S7_36216615 | 7 | 36216615 | 3 | G | A | G | 0.74194 | A | 0.25806 | 10 | 0.32258 |
| 6065 | S7_37430268 | 7 | 37430268 | 1213653 | T | G | T | 0.83871 | G | 0.16129 | 8 | 0.25806 |
| 6066 | S7_37430273 | 7 | 37430273 | 5 | A | G | A | 0.79032 | G | 0.20968 | 9 | 0.29032 |
| 6067 | S7_37430279 | 7 | 37430279 | 6 | G | C | G | 0.79032 | C | 0.20968 | 9 | 0.29032 |
| 6068 | S7_37430469 | 7 | 37430469 | 190 | C | T | C | 0.93548 | T | 0.06452 | 2 | 0.06452 |
| 6069 | S7_38261742 | 7 | 38261742 | 831273 | T | G | G | 0.53226 | T | 0.46774 | 17 | 0.54839 |
| 6070 | S7_38261754 | 7 | 38261754 | 12 | C | T | C | 0.56452 | T | 0.43548 | 15 | 0.48387 |
| 6071 | S7_38261769 | 7 | 38261769 | 15 | G | T | G | 0.91935 | T | 0.08065 | 5 | 0.16129 |
| 6072 | S7_38261770 | 7 | 38261770 | 1 | G | C | G | 0.91935 | C | 0.08065 | 5 | 0.16129 |
| 6073 | S7_38574904 | 7 | 38574904 | 313134 | T | C | T | 0.59677 | C | 0.40323 | 17 | 0.54839 |
| 6074 | S7_39187532 | 7 | 39187532 | 612628 | C | T | T | 0.70968 | C | 0.29032 | 8 | 0.25806 |
| 6075 | S7_39239330 | 7 | 39239330 | 51798 | A | G | A | 0.54839 | G | 0.45161 | 14 | 0.45161 |
| 6076 | S7_39346844 | 7 | 39346844 | 107514 | T | C | T | 0.90323 | C | 0.09677 | 6 | 0.19355 |
| 6077 | S7_39346984 | 7 | 39346984 | 140 | T | C | T | 0.90323 | C | 0.09677 | 6 | 0.19355 |
| 6078 | S7_39466950 | 7 | 39466950 | 119966 | G | A | G | 0.90323 | A | 0.09677 | 6 | 0.19355 |
| 6079 | S7_39466980 | 7 | 39466980 | 30 | T | A | T | 0.90323 | A | 0.09677 | 6 | 0.19355 |
| 6080 | S7_39467117 | 7 | 39467117 | 137 | G | A | G | 0.90323 | A | 0.09677 | 6 | 0.19355 |
| 6081 | S7_39601061 | 7 | 39601061 | 133944 | G | A | G | 0.66129 | A | 0.33871 | 17 | 0.54839 |
| 6082 | S7_39601080 | 7 | 39601080 | 19 | C | T | C | 0.91935 | T | 0.08065 | 5 | 0.16129 |
| 6083 | S7_39601121 | 7 | 39601121 | 41 | G | A | G | 0.91935 | A | 0.08065 | 5 | 0.16129 |
| 6084 | S7_39601142 | 7 | 39601142 | 21 | T | A | T | 0.59677 | A | 0.40323 | 13 | 0.41935 |
| 6085 | S7_39601191 | 7 | 39601191 | 49 | T | C | T | 0.66129 | C | 0.33871 | 17 | 0.54839 |
| 6086 | S7_39977061 | 7 | 39977061 | 375870 | A | G | G | 0.93548 | A | 0.06452 | 4 | 0.12903 |
| 6087 | S7_39977102 | 7 | 39977102 | 41 | A | C | A | 0.90323 | C | 0.09677 | 6 | 0.19355 |
| 6088 | S7_40264842 | 7 | 40264842 | 287740 | G | A | G | 0.90323 | A | 0.09677 | 6 | 0.19355 |
| 6089 | S7_40264887 | 7 | 40264887 | 45 | G | A | G | 0.90323 | A | 0.09677 | 6 | 0.19355 |
| 6090 | S7_40754166 | 7 | 40754166 | 489279 | G | A | G | 0.79032 | A | 0.20968 | 7 | 0.22581 |
| 6091 | S7_40754206 | 7 | 40754206 | 40 | A | C | A | 0.90323 | C | 0.09677 | 6 | 0.19355 |
| 6092 | S7_40754250 | 7 | 40754250 | 44 | T | C | C | 0.64516 | T | 0.35484 | 14 | 0.45161 |
| 6093 | S7_41125857 | 7 | 41125857 | 371607 | A | T | A | 0.64516 | T | 0.35484 | 16 | 0.51613 |
| 6094 | S7_41125969 | 7 | 41125969 | 112 | A | G | A | 0.85484 | G | 0.14516 | 7 | 0.22581 |
| 6095 | S7_41540865 | 7 | 41540865 | 414896 | A | C | C | 0.80645 | A | 0.19355 | 10 | 0.32258 |
| 6096 | S7_41541026 | 7 | 41541026 | 161 | C | A | C | 0.83871 | A | 0.16129 | 10 | 0.32258 |
| 6097 | S7_41609476 | 7 | 41609476 | 68450 | T | C | T | 0.87097 | C | 0.12903 | 8 | 0.25806 |
| 6098 | S7_41609582 | 7 | 41609582 | 106 | C | T | C | 0.91935 | T | 0.08065 | 5 | 0.16129 |
| 6099 | S7_42624867 | 7 | 42624867 | 1015285 | C | T | C | 0.90323 | T | 0.09677 | 6 | 0.19355 |
| 6100 | S7_42624888 | 7 | 42624888 | 21 | G | A | G | 0.87097 | A | 0.12903 | 6 | 0.19355 |

| Site number | SNP name | Chromosome | Physical position | Physical distance from the previous SNP | Reference allele | Alternative allele | Major allele | Major allele frequency | Minor allele | Minor allele frequency | Number heterozygous | Heterozygosity |
|---|---|---|---|---|---|---|---|---|---|---|---|---|
| 6101 | S7_42625079 | 7 | 42625079 | 191 | G | A | G | 0.87097 | A | 0.12903 | 6 | 0.19355 |
| 6102 | S7_42702266 | 7 | 42702266 | 77187 | C | T | T | 0.6129 | C | 0.3871 | 12 | 0.3871 |
| 6103 | S7_42702509 | 7 | 42702509 | 243 | C | T | T | 0.93548 | C | 0.06452 | 4 | 0.12903 |
| 6104 | S7_43142596 | 7 | 43142596 | 440087 | G | A | G | 0.85484 | A | 0.14516 | 7 | 0.22581 |
| 6105 | S7_43142680 | 7 | 43142680 | 84 | A | G | A | 0.85484 | G | 0.14516 | 7 | 0.22581 |
| 6106 | S7_43273609 | 7 | 43273609 | 130929 | C | T | T | 0.72581 | C | 0.27419 | 11 | 0.35484 |
| 6107 | S7_43273654 | 7 | 43273654 | 45 | G | A | A | 0.72581 | G | 0.27419 | 11 | 0.35484 |
| 6108 | S7_43418693 | 7 | 43418693 | 145039 | G | A | G | 0.93548 | A | 0.06452 | 4 | 0.12903 |
| 6109 | S7_43418753 | 7 | 43418753 | 60 | A | G | G | 0.93548 | A | 0.06452 | 4 | 0.12903 |
| 6110 | S7_43418764 | 7 | 43418764 | 11 | T | C | C | 0.66129 | T | 0.33871 | 9 | 0.29032 |
| 6111 | S7_43418828 | 7 | 43418828 | 64 | G | A | G | 0.62903 | A | 0.37097 | 13 | 0.41935 |
| 6112 | S7_43418875 | 7 | 43418875 | 47 | C | G | C | 0.95161 | G | 0.04839 | 3 | 0.09677 |
| 6113 | S7_43418910 | 7 | 43418910 | 35 | A | G | G | 0.64516 | A | 0.35484 | 10 | 0.32258 |
| 6114 | S7_43753090 | 7 | 43753090 | 334180 | T | C | T | 0.74194 | C | 0.25806 | 14 | 0.45161 |
| 6115 | S7_43753237 | 7 | 43753237 | 147 | G | A | G | 0.74194 | A | 0.25806 | 14 | 0.45161 |
| 6116 | S7_44176073 | 7 | 44176073 | 422836 | G | C | G | 0.93548 | C | 0.06452 | 2 | 0.06452 |
| 6117 | S7_44633032 | 7 | 44633032 | 456959 | A | T | A | 0.90323 | T | 0.09677 | 4 | 0.12903 |
| 6118 | S7_44633094 | 7 | 44633094 | 62 | C | T | C | 0.8871 | T | 0.1129 | 5 | 0.16129 |
| 6119 | S7_44714379 | 7 | 44714379 | 81285 | C | T | C | 0.93548 | T | 0.06452 | 4 | 0.12903 |
| 6120 | S7_44714551 | 7 | 44714551 | 172 | C | A | C | 0.66129 | A | 0.33871 | 13 | 0.41935 |
| 6121 | S7_44721665 | 7 | 44721665 | 7114 | A | C | A | 0.95161 | C | 0.04839 | 3 | 0.09677 |
| 6122 | S7_45074370 | 7 | 45074370 | 352705 | C | T | C | 0.95161 | T | 0.04839 | 1 | 0.03226 |
| 6123 | S7_45074562 | 7 | 45074562 | 192 | A | G | A | 0.85484 | G | 0.14516 | 9 | 0.29032 |
| 6124 | S7_45304046 | 7 | 45304046 | 229484 | A | G | A | 0.91935 | G | 0.08065 | 3 | 0.09677 |
| 6125 | S7_45372015 | 7 | 45372015 | 67969 | C | A | A | 0.56452 | C | 0.43548 | 13 | 0.41935 |
| 6126 | S7_45372046 | 7 | 45372046 | 31 | C | T | C | 0.8871 | T | 0.1129 | 7 | 0.22581 |
| 6127 | S7_45385455 | 7 | 45385455 | 13409 | T | C | T | 0.56452 | C | 0.43548 | 13 | 0.41935 |
| 6128 | S7_45616656 | 7 | 45616656 | 231201 | G | A | G | 0.93548 | A | 0.06452 | 2 | 0.06452 |
| 6129 | S7_45616810 | 7 | 45616810 | 154 | G | A | G | 0.95161 | A | 0.04839 | 3 | 0.09677 |
| 6130 | S7_47267037 | 7 | 47267037 | 1650227 | A | C | C | 0.95161 | A | 0.04839 | 3 | 0.09677 |
| 6131 | S7_47353097 | 7 | 47353097 | 86060 | C | T | T | 0.59677 | C | 0.40323 | 17 | 0.54839 |
| 6132 | S7_47353151 | 7 | 47353151 | 54 | G | C | G | 0.59677 | C | 0.40323 | 17 | 0.54839 |
| 6133 | S7_47420627 | 7 | 47420627 | 67476 | T | C | T | 0.85484 | C | 0.14516 | 9 | 0.29032 |
| 6134 | S7_47471961 | 7 | 47471961 | 51334 | C | T | C | 0.95161 | T | 0.04839 | 1 | 0.03226 |
| 6135 | S7_47472061 | 7 | 47472061 | 100 | G | A | G | 0.8871 | A | 0.1129 | 7 | 0.22581 |
| 6136 | S7_48049287 | 7 | 48049287 | 577226 | C | T | C | 0.93548 | T | 0.06452 | 4 | 0.12903 |
| 6137 | S7_48140346 | 7 | 48140346 | 91059 | C | T | C | 0.95161 | T | 0.04839 | 3 | 0.09677 |
| 6138 | S7_48243199 | 7 | 48243199 | 102853 | C | A | C | 0.93548 | A | 0.06452 | 4 | 0.12903 |
| 6139 | S7_48243231 | 7 | 48243231 | 32 | C | T | C | 0.80645 | T | 0.19355 | 10 | 0.32258 |
| 6140 | S7_48243315 | 7 | 48243315 | 84 | G | A | G | 0.80645 | A | 0.19355 | 10 | 0.32258 |
| 6141 | S7_49016098 | 7 | 49016098 | 772783 | G | A | G | 0.95161 | A | 0.04839 | 3 | 0.09677 |
| 6142 | S7_49103633 | 7 | 49103633 | 87535 | C | A | A | 0.64516 | C | 0.35484 | 16 | 0.51613 |
| 6143 | S7_49494312 | 7 | 49494312 | 390679 | T | C | T | 0.82258 | C | 0.17742 | 9 | 0.29032 |
| 6144 | S7_49494341 | 7 | 49494341 | 29 | C | T | C | 0.72581 | T | 0.27419 | 13 | 0.41935 |
| 6145 | S7_49494446 | 7 | 49494446 | 105 | C | A | C | 0.85484 | A | 0.14516 | 9 | 0.29032 |
| 6146 | S7_49494492 | 7 | 49494492 | 46 | A | G | A | 0.70968 | G | 0.29032 | 14 | 0.45161 |
| 6147 | S7_49494502 | 7 | 49494502 | 10 | A | C | A | 0.82258 | C | 0.17742 | 9 | 0.29032 |
| 6148 | S7_49604959 | 7 | 49604959 | 110457 | T | C | T | 0.82258 | C | 0.17742 | 9 | 0.29032 |
| 6149 | S7_49784029 | 7 | 49784029 | 179070 | T | C | T | 0.64516 | C | 0.35484 | 14 | 0.45161 |
| 6150 | S7_49784240 | 7 | 49784240 | 211 | G | A | G | 0.82258 | A | 0.17742 | 9 | 0.29032 |
| 6151 | S7_49811902 | 7 | 49811902 | 27662 | C | T | T | 0.67742 | C | 0.32258 | 14 | 0.45161 |
| 6152 | S7_49916093 | 7 | 49916093 | 104191 | C | T | T | 0.6129 | C | 0.3871 | 12 | 0.3871 |
| 6153 | S7_50064122 | 7 | 50064122 | 148029 | C | G | C | 0.70968 | G | 0.29032 | 14 | 0.45161 |
| 6154 | S7_50064183 | 7 | 50064183 | 61 | G | A | G | 0.70968 | A | 0.29032 | 14 | 0.45161 |

| Site number | SNP name | Chromosome | Physical position | Physical distance from the previous SNP | Reference allele | Alternative allele | Major allele | Major allele frequency | Minor allele | Minor allele frequency | Number heterozygous | Heterozygosity |
|---|---|---|---|---|---|---|---|---|---|---|---|---|
| 6155 | S7_50223532 | 7 | 50223532 | 159349 | G | A | G | 0.53226 | A | 0.46774 | 17 | 0.54839 |
| 6156 | S7_50223610 | 7 | 50223610 | 78 | G | T | G | 0.93548 | T | 0.06452 | 4 | 0.12903 |
| 6157 | S7_50320871 | 7 | 50320871 | 97261 | A | C | A | 0.77419 | C | 0.22581 | 8 | 0.25806 |
| 6158 | S7_50321006 | 7 | 50321006 | 135 | C | T | C | 0.83871 | T | 0.16129 | 8 | 0.25806 |
| 6159 | S7_50321018 | 7 | 50321018 | 12 | C | T | C | 0.83871 | T | 0.16129 | 8 | 0.25806 |
| 6160 | S7_50321023 | 7 | 50321023 | 5 | A | G | A | 0.83871 | G | 0.16129 | 8 | 0.25806 |
| 6161 | S7_50627753 | 7 | 50627753 | 306730 | A | G | A | 0.90323 | G | 0.09677 | 4 | 0.12903 |
| 6162 | S7_50826195 | 7 | 50826195 | 198442 | G | A | G | 0.70968 | A | 0.29032 | 14 | 0.45161 |
| 6163 | S7_50826324 | 7 | 50826324 | 129 | T | C | C | 0.90323 | T | 0.09677 | 6 | 0.19355 |
| 6164 | S7_50826399 | 7 | 50826399 | 75 | C | T | C | 0.8871 | T | 0.1129 | 5 | 0.16129 |
| 6165 | S7_50915766 | 7 | 50915766 | 89367 | G | A | G | 0.70968 | A | 0.29032 | 12 | 0.3871 |
| 6166 | S7_50915989 | 7 | 50915989 | 223 | C | T | C | 0.95161 | T | 0.04839 | 3 | 0.09677 |
| 6167 | S7_51520603 | 7 | 51520603 | 604614 | C | A | C | 0.87097 | A | 0.12903 | 6 | 0.19355 |
| 6168 | S7_51815036 | 7 | 51815036 | 294433 | T | G | T | 0.80645 | G | 0.19355 | 6 | 0.19355 |
| 6169 | S7_51863539 | 7 | 51863539 | 48503 | A | G | A | 0.72581 | G | 0.27419 | 13 | 0.41935 |
| 6170 | S7_52067701 | 7 | 52067701 | 204162 | G | A | G | 0.90323 | A | 0.09677 | 6 | 0.19355 |
| 6171 | S7_52067792 | 7 | 52067792 | 91 | A | G | A | 0.56452 | G | 0.43548 | 13 | 0.41935 |
| 6172 | S7_52067872 | 7 | 52067872 | 80 | G | A | G | 0.85484 | A | 0.14516 | 7 | 0.22581 |
| 6173 | S7_52237118 | 7 | 52237118 | 169246 | G | T | G | 0.5 | T | 0.5 | 17 | 0.54839 |
| 6174 | S7_52511563 | 7 | 52511563 | 274445 | A | C | A | 0.69355 | C | 0.30645 | 11 | 0.35484 |
| 6175 | S7_52511667 | 7 | 52511667 | 104 | T | C | T | 0.93548 | C | 0.06452 | 4 | 0.12903 |
| 6176 | S7_52539708 | 7 | 52539708 | 28041 | G | A | G | 0.83871 | A | 0.16129 | 10 | 0.32258 |
| 6177 | S7_52539742 | 7 | 52539742 | 34 | C | G | C | 0.95161 | G | 0.04839 | 3 | 0.09677 |
| 6178 | S7_52539746 | 7 | 52539746 | 4 | C | T | T | 0.87097 | C | 0.12903 | 6 | 0.19355 |
| 6179 | S7_52539753 | 7 | 52539753 | 7 | G | A | G | 0.90323 | A | 0.09677 | 6 | 0.19355 |
| 6180 | S7_52539767 | 7 | 52539767 | 14 | T | A | A | 0.6129 | T | 0.3871 | 14 | 0.45161 |
| 6181 | S7_52539778 | 7 | 52539778 | 11 | G | C | G | 0.83871 | C | 0.16129 | 10 | 0.32258 |
| 6182 | S7_52539779 | 7 | 52539779 | 1 | T | C | T | 0.83871 | C | 0.16129 | 10 | 0.32258 |
| 6183 | S7_52539780 | 7 | 52539780 | 1 | G | A | G | 0.83871 | A | 0.16129 | 10 | 0.32258 |
| 6184 | S7_52539876 | 7 | 52539876 | 96 | G | A | G | 0.93548 | A | 0.06452 | 4 | 0.12903 |
| 6185 | S7_52539916 | 7 | 52539916 | 40 | C | T | C | 0.83871 | T | 0.16129 | 8 | 0.25806 |
| 6186 | S7_52539925 | 7 | 52539925 | 9 | C | T | C | 0.93548 | T | 0.06452 | 4 | 0.12903 |
| 6187 | S7_52623578 | 7 | 52623578 | 83653 | C | T | T | 0.83871 | C | 0.16129 | 10 | 0.32258 |
| 6188 | S7_52623604 | 7 | 52623604 | 26 | A | G | G | 0.83871 | A | 0.16129 | 10 | 0.32258 |
| 6189 | S7_52623680 | 7 | 52623680 | 76 | G | A | A | 0.83871 | G | 0.16129 | 10 | 0.32258 |
| 6190 | S7_53359300 | 7 | 53359300 | 735620 | C | T | T | 0.70968 | C | 0.29032 | 12 | 0.3871 |
| 6191 | S7_53359306 | 7 | 53359306 | 6 | G | A | A | 0.70968 | G | 0.29032 | 12 | 0.3871 |
| 6192 | S7_53363304 | 7 | 53363304 | 3998 | T | A | T | 0.90323 | A | 0.09677 | 6 | 0.19355 |
| 6193 | S7_53370356 | 7 | 53370356 | 7052 | G | A | G | 0.93548 | A | 0.06452 | 4 | 0.12903 |
| 6194 | S7_53430964 | 7 | 53430964 | 60608 | A | G | A | 0.51613 | G | 0.48387 | 14 | 0.45161 |
| 6195 | S7_53431004 | 7 | 53431004 | 40 | C | T | C | 0.72581 | T | 0.27419 | 11 | 0.35484 |
| 6196 | S7_53662959 | 7 | 53662959 | 231955 | A | C | A | 0.85484 | C | 0.14516 | 9 | 0.29032 |
| 6197 | S7_53786240 | 7 | 53786240 | 123281 | T | C | C | 0.51613 | T | 0.48387 | 12 | 0.3871 |
| 6198 | S7_53786266 | 7 | 53786266 | 26 | A | T | A | 0.80645 | T | 0.19355 | 8 | 0.25806 |
| 6199 | S7_53786313 | 7 | 53786313 | 47 | C | A | C | 0.79032 | A | 0.20968 | 11 | 0.35484 |
| 6200 | S7_53786365 | 7 | 53786365 | 52 | G | A | G | 0.80645 | A | 0.19355 | 8 | 0.25806 |
| 6201 | S7_53786428 | 7 | 53786428 | 63 | T | C | C | 0.51613 | T | 0.48387 | 12 | 0.3871 |
| 6202 | S7_53942718 | 7 | 53942718 | 156290 | C | T | T | 0.74194 | C | 0.25806 | 14 | 0.45161 |
| 6203 | S7_53942724 | 7 | 53942724 | 6 | T | C | C | 0.75806 | T | 0.24194 | 13 | 0.41935 |
| 6204 | S7_53942788 | 7 | 53942788 | 64 | T | C | T | 0.90323 | C | 0.09677 | 6 | 0.19355 |
| 6205 | S7_54191191 | 7 | 54191191 | 248403 | A | G | A | 0.8871 | G | 0.1129 | 7 | 0.22581 |
| 6206 | S7_54191314 | 7 | 54191314 | 123 | A | T | T | 0.62903 | A | 0.37097 | 13 | 0.41935 |
| 6207 | S7_54191317 | 7 | 54191317 | 3 | A | G | G | 0.62903 | A | 0.37097 | 13 | 0.41935 |
| 6208 | S7_55868846 | 7 | 55868846 | 1677529 | G | A | G | 0.87097 | A | 0.12903 | 6 | 0.19355 |

| Site number | SNP name | Chromosome | Physical position | Physical distance from the previous SNP | Reference allele | Alternative allele | Major allele | Major allele frequency | Minor allele | Minor allele frequency | Number heterozygous | Heterozygosity |
|---|---|---|---|---|---|---|---|---|---|---|---|---|
| 6209 | S7_55868906 | 7 | 55868906 | 60 | A | G | A | 0.91935 | G | 0.08065 | 5 | 0.16129 |
| 6210 | S7_55868915 | 7 | 55868915 | 9 | G | A | G | 0.67742 | A | 0.32258 | 16 | 0.51613 |
| 6211 | S7_55868934 | 7 | 55868934 | 19 | C | T | C | 0.69355 | T | 0.30645 | 9 | 0.29032 |
| 6212 | S7_55993073 | 7 | 55993073 | 124139 | T | C | C | 0.91935 | T | 0.08065 | 5 | 0.16129 |
| 6213 | S7_55993180 | 7 | 55993180 | 107 | C | T | C | 0.93548 | T | 0.06452 | 4 | 0.12903 |
| 6214 | S7_56241486 | 7 | 56241486 | 248306 | C | T | C | 0.74194 | T | 0.25806 | 14 | 0.45161 |
| 6215 | S7_56281863 | 7 | 56281863 | 40377 | G | A | G | 0.91935 | A | 0.08065 | 5 | 0.16129 |
| 6216 | S7_56432408 | 7 | 56432408 | 150545 | C | A | C | 0.74194 | A | 0.25806 | 16 | 0.51613 |
| 6217 | S7_57788660 | 7 | 57788660 | 1356252 | G | C | G | 0.83871 | C | 0.16129 | 10 | 0.32258 |
| 6218 | S7_58124557 | 7 | 58124557 | 335897 | G | C | G | 0.90323 | C | 0.09677 | 6 | 0.19355 |
| 6219 | S7_58124651 | 7 | 58124651 | 94 | T | C | T | 0.87097 | C | 0.12903 | 4 | 0.12903 |
| 6220 | S7_58311924 | 7 | 58311924 | 187273 | A | T | A | 0.72581 | T | 0.27419 | 13 | 0.41935 |
| 6221 | S7_58311984 | 7 | 58311984 | 60 | G | A | G | 0.90323 | A | 0.09677 | 6 | 0.19355 |
| 6222 | S7_58312003 | 7 | 58312003 | 19 | C | T | C | 0.72581 | T | 0.27419 | 13 | 0.41935 |
| 6223 | S7_58416679 | 7 | 58416679 | 104676 | T | C | T | 0.90323 | C | 0.09677 | 4 | 0.12903 |
| 6224 | S7_58534859 | 7 | 58534859 | 118180 | C | T | T | 0.90323 | C | 0.09677 | 6 | 0.19355 |
| 6225 | S7_58534875 | 7 | 58534875 | 16 | C | G | C | 0.69355 | G | 0.30645 | 15 | 0.48387 |
| 6226 | S7_58534932 | 7 | 58534932 | 57 | T | G | G | 0.8871 | T | 0.1129 | 7 | 0.22581 |
| 6227 | S7_59146054 | 7 | 59146054 | 611122 | A | C | A | 0.93548 | C | 0.06452 | 4 | 0.12903 |
| 6228 | S7_59146184 | 7 | 59146184 | 130 | G | A | G | 0.91935 | A | 0.08065 | 5 | 0.16129 |
| 6229 | S7_59146232 | 7 | 59146232 | 48 | A | G | A | 0.79032 | G | 0.20968 | 9 | 0.29032 |
| 6230 | S7_59545082 | 7 | 59545082 | 398850 | T | C | C | 0.90323 | T | 0.09677 | 4 | 0.12903 |
| 6231 | S7_60268804 | 7 | 60268804 | 723722 | C | T | T | 0.66129 | C | 0.33871 | 17 | 0.54839 |
| 6232 | S7_60424441 | 7 | 60424441 | 155637 | G | A | G | 0.8871 | A | 0.1129 | 7 | 0.22581 |
| 6233 | S7_60424518 | 7 | 60424518 | 77 | G | A | G | 0.80645 | A | 0.19355 | 8 | 0.25806 |
| 6234 | S7_60424552 | 7 | 60424552 | 34 | C | G | G | 0.87097 | C | 0.12903 | 8 | 0.25806 |
| 6235 | S7_60424586 | 7 | 60424586 | 34 | C | T | C | 0.8871 | T | 0.1129 | 7 | 0.22581 |
| 6236 | S7_60840990 | 7 | 60840990 | 416404 | C | T | C | 0.93548 | T | 0.06452 | 4 | 0.12903 |
| 6237 | S7_60881047 | 7 | 60881047 | 40057 | G | A | G | 0.90323 | A | 0.09677 | 6 | 0.19355 |
| 6238 | S7_61850855 | 7 | 61850855 | 969808 | A | G | G | 0.85484 | A | 0.14516 | 9 | 0.29032 |
| 6239 | S7_62264476 | 7 | 62264476 | 413621 | T | C | C | 0.6129 | T | 0.3871 | 20 | 0.64516 |
| 6240 | S7_62264481 | 7 | 62264481 | 5 | C | T | C | 0.93548 | T | 0.06452 | 4 | 0.12903 |
| 6241 | S7_62312047 | 7 | 62312047 | 47566 | A | G | G | 0.95161 | A | 0.04839 | 3 | 0.09677 |
| 6242 | S7_62529055 | 7 | 62529055 | 217008 | C | T | C | 0.8871 | T | 0.1129 | 7 | 0.22581 |
| 6243 | S7_63027576 | 7 | 63027576 | 498521 | C | T | C | 0.85484 | T | 0.14516 | 7 | 0.22581 |
| 6244 | S7_63027624 | 7 | 63027624 | 48 | C | T | C | 0.79032 | T | 0.20968 | 9 | 0.29032 |
| 6245 | S7_63063313 | 7 | 63063313 | 35689 | G | A | G | 0.82258 | A | 0.17742 | 11 | 0.35484 |
| 6246 | S7_63288750 | 7 | 63288750 | 225437 | T | C | T | 0.83871 | C | 0.16129 | 10 | 0.32258 |
| 6247 | S7_63382260 | 7 | 63382260 | 93510 | C | A | C | 0.95161 | A | 0.04839 | 3 | 0.09677 |
| 6248 | S7_63436934 | 7 | 63436934 | 54674 | C | A | A | 0.58065 | C | 0.41935 | 20 | 0.64516 |
| 6249 | S7_63675505 | 7 | 63675505 | 238571 | C | T | C | 0.56452 | T | 0.43548 | 13 | 0.41935 |
| 6250 | S7_64252872 | 7 | 64252872 | 577367 | A | G | A | 0.6129 | G | 0.3871 | 20 | 0.64516 |
| 6251 | S7_64252988 | 7 | 64252988 | 116 | C | A | C | 0.8871 | A | 0.1129 | 7 | 0.22581 |
| 6252 | S7_64253005 | 7 | 64253005 | 17 | C | G | C | 0.93548 | G | 0.06452 | 4 | 0.12903 |
| 6253 | S7_64253078 | 7 | 64253078 | 73 | A | G | A | 0.75806 | G | 0.24194 | 15 | 0.48387 |
| 6254 | S7_64384046 | 7 | 64384046 | 130968 | C | T | C | 0.93548 | T | 0.06452 | 4 | 0.12903 |
| 6255 | S7_64384156 | 7 | 64384156 | 110 | C | T | C | 0.8871 | T | 0.1129 | 7 | 0.22581 |
| 6256 | S7_64384166 | 7 | 64384166 | 10 | G | A | G | 0.80645 | A | 0.19355 | 12 | 0.3871 |
| 6257 | S7_64853494 | 7 | 64853494 | 469328 | C | T | C | 0.90323 | T | 0.09677 | 6 | 0.19355 |
| 6258 | S7_64931233 | 7 | 64931233 | 77739 | A | C | A | 0.90323 | C | 0.09677 | 6 | 0.19355 |
| 6259 | S7_64931251 | 7 | 64931251 | 18 | A | G | G | 0.56452 | A | 0.43548 | 11 | 0.35484 |
| 6260 | S7_64931375 | 7 | 64931375 | 124 | A | G | A | 0.90323 | G | 0.09677 | 6 | 0.19355 |
| 6261 | S7_65366555 | 7 | 65366555 | 435180 | G | A | G | 0.87097 | A | 0.12903 | 8 | 0.25806 |
| 6262 | S7_65366567 | 7 | 65366567 | 12 | G | A | G | 0.79032 | A | 0.20968 | 11 | 0.35484 |

| Site number | SNP name | Chromosome | Physical position | Physical distance from the previous SNP | Reference allele | Alternative allele | Major allele | Major allele frequency | Minor allele | Minor allele frequency | Number heterozygous | Heterozygosity |
|---|---|---|---|---|---|---|---|---|---|---|---|---|
| 6263 | S7_65594644 | 7 | 65594644 | 228077 | G | A | G | 0.85484 | A | 0.14516 | 9 | 0.29032 |
| 6264 | S7_65594724 | 7 | 65594724 | 80 | G | A | G | 0.91935 | A | 0.08065 | 5 | 0.16129 |
| 6265 | S7_65931887 | 7 | 65931887 | 337163 | T | C | T | 0.72581 | C | 0.27419 | 15 | 0.48387 |
| 6266 | S7_65956561 | 7 | 65956561 | 24674 | T | C | T | 0.77419 | C | 0.22581 | 12 | 0.3871 |
| 6267 | S7_66093665 | 7 | 66093665 | 137104 | G | T | G | 0.93548 | T | 0.06452 | 4 | 0.12903 |
| 6268 | S7_66382140 | 7 | 66382140 | 288475 | C | T | C | 0.54839 | T | 0.45161 | 12 | 0.3871 |
| 6269 | S7_66747333 | 7 | 66747333 | 365193 | G | A | G | 0.80645 | A | 0.19355 | 8 | 0.25806 |
| 6270 | S7_67081114 | 7 | 67081114 | 333781 | G | A | G | 0.80645 | A | 0.19355 | 8 | 0.25806 |
| 6271 | S7_67334135 | 7 | 67334135 | 253021 | C | A | C | 0.72581 | A | 0.27419 | 15 | 0.48387 |
| 6272 | S7_67652845 | 7 | 67652845 | 318710 | A | G | G | 0.72581 | A | 0.27419 | 11 | 0.35484 |
| 6273 | S7_67935329 | 7 | 67935329 | 282484 | A | G | A | 0.90323 | G | 0.09677 | 6 | 0.19355 |
| 6274 | S7_67935419 | 7 | 67935419 | 90 | T | C | T | 0.93548 | C | 0.06452 | 4 | 0.12903 |
| 6275 | S7_67935472 | 7 | 67935472 | 53 | G | A | G | 0.90323 | A | 0.09677 | 6 | 0.19355 |
| 6276 | S7_67965807 | 7 | 67965807 | 30335 | C | T | C | 0.91935 | T | 0.08065 | 3 | 0.09677 |
| 6277 | S7_68608196 | 7 | 68608196 | 642389 | A | G | A | 0.74194 | G | 0.25806 | 12 | 0.3871 |
| 6278 | S7_68666316 | 7 | 68666316 | 58120 | C | T | C | 0.93548 | T | 0.06452 | 4 | 0.12903 |
| 6279 | S7_68666404 | 7 | 68666404 | 88 | G | T | G | 0.64516 | T | 0.35484 | 18 | 0.58065 |
| 6280 | S7_68666514 | 7 | 68666514 | 110 | T | C | T | 0.93548 | C | 0.06452 | 4 | 0.12903 |
| 6281 | S7_69051843 | 7 | 69051843 | 385329 | G | A | G | 0.53226 | A | 0.46774 | 13 | 0.41935 |
| 6282 | S7_69051850 | 7 | 69051850 | 7 | C | T | C | 0.85484 | T | 0.14516 | 7 | 0.22581 |
| 6283 | S7_69051939 | 7 | 69051939 | 89 | G | A | G | 0.53226 | A | 0.46774 | 13 | 0.41935 |
| 6284 | S7_69051956 | 7 | 69051956 | 17 | C | A | C | 0.93548 | A | 0.06452 | 4 | 0.12903 |
| 6285 | S7_69051957 | 7 | 69051957 | 1 | C | A | C | 0.53226 | A | 0.46774 | 13 | 0.41935 |
| 6286 | S7_69051973 | 7 | 69051973 | 16 | T | C | T | 0.75806 | C | 0.24194 | 13 | 0.41935 |
| 6287 | S7_69051979 | 7 | 69051979 | 6 | G | A | G | 0.82258 | A | 0.17742 | 9 | 0.29032 |
| 6288 | S7_69052010 | 7 | 69052010 | 31 | A | T | A | 0.54839 | T | 0.45161 | 12 | 0.3871 |
| 6289 | S7_69052068 | 7 | 69052068 | 58 | C | T | C | 0.80645 | T | 0.19355 | 10 | 0.32258 |
| 6290 | S7_69729324 | 7 | 69729324 | 677256 | T | C | T | 0.95161 | C | 0.04839 | 3 | 0.09677 |
| 6291 | S7_69729328 | 7 | 69729328 | 4 | T | C | T | 0.79032 | C | 0.20968 | 9 | 0.29032 |
| 6292 | S7_69729398 | 7 | 69729398 | 70 | A | T | A | 0.93548 | T | 0.06452 | 4 | 0.12903 |
| 6293 | S7_69735486 | 7 | 69735486 | 6088 | C | T | C | 0.59677 | T | 0.40323 | 17 | 0.54839 |
| 6294 | S7_69735499 | 7 | 69735499 | 13 | C | T | T | 0.8871 | C | 0.1129 | 5 | 0.16129 |
| 6295 | S7_72026305 | 7 | 72026305 | 2290806 | A | G | G | 0.85484 | A | 0.14516 | 7 | 0.22581 |
| 6296 | S7_72026325 | 7 | 72026325 | 20 | G | A | G | 0.87097 | A | 0.12903 | 8 | 0.25806 |
| 6297 | S7_72454217 | 7 | 72454217 | 427892 | G | A | G | 0.87097 | A | 0.12903 | 6 | 0.19355 |
| 6298 | S7_72454228 | 7 | 72454228 | 11 | C | A | C | 0.90323 | A | 0.09677 | 6 | 0.19355 |
| 6299 | S7_72476831 | 7 | 72476831 | 22603 | C | T | C | 0.95161 | T | 0.04839 | 3 | 0.09677 |
| 6300 | S7_72585397 | 7 | 72585397 | 108566 | T | C | T | 0.87097 | C | 0.12903 | 8 | 0.25806 |
| 6301 | S7_72585419 | 7 | 72585419 | 22 | C | A | C | 0.91935 | A | 0.08065 | 5 | 0.16129 |
| 6302 | S7_73204677 | 7 | 73204677 | 619258 | G | A | G | 0.95161 | A | 0.04839 | 3 | 0.09677 |
| 6303 | S7_73365575 | 7 | 73365575 | 160898 | G | A | G | 0.8871 | A | 0.1129 | 7 | 0.22581 |
| 6304 | S7_73365696 | 7 | 73365696 | 121 | G | C | G | 0.87097 | C | 0.12903 | 8 | 0.25806 |
| 6305 | S7_73370009 | 7 | 73370009 | 4313 | G | A | G | 0.87097 | A | 0.12903 | 8 | 0.25806 |
| 6306 | S7_74879620 | 7 | 74879620 | 1509611 | T | C | T | 0.69355 | C | 0.30645 | 13 | 0.41935 |
| 6307 | S7_74879669 | 7 | 74879669 | 49 | G | C | C | 0.67742 | G | 0.32258 | 14 | 0.45161 |
| 6308 | S7_74879730 | 7 | 74879730 | 61 | C | T | C | 0.87097 | T | 0.12903 | 6 | 0.19355 |
| 6309 | S7_74879824 | 7 | 74879824 | 94 | T | A | T | 0.53226 | A | 0.46774 | 15 | 0.48387 |
| 6310 | S7_75430556 | 7 | 75430556 | 550732 | T | C | T | 0.93548 | C | 0.06452 | 2 | 0.06452 |
| 6311 | S7_75522583 | 7 | 75522583 | 92027 | T | C | T | 0.95161 | C | 0.04839 | 3 | 0.09677 |
| 6312 | S7_75522734 | 7 | 75522734 | 151 | A | G | A | 0.8871 | G | 0.1129 | 5 | 0.16129 |
| 6313 | S7_75522809 | 7 | 75522809 | 75 | G | A | G | 0.95161 | A | 0.04839 | 3 | 0.09677 |
| 6314 | S7_76388458 | 7 | 76388458 | 865649 | C | T | C | 0.91935 | T | 0.08065 | 5 | 0.16129 |
| 6315 | S7_77197667 | 7 | 77197667 | 809209 | G | A | G | 0.95161 | A | 0.04839 | 3 | 0.09677 |
| 6316 | S7_77197716 | 7 | 77197716 | 49 | G | A | G | 0.80645 | A | 0.19355 | 12 | 0.3871 |

| Site number | SNP name | Chromosome | Physical position | Physical distance from the previous SNP | Reference allele | Alternative allele | Major allele | Major allele frequency | Minor allele | Minor allele frequency | Number heterozygous | Heterozygosity |
|---|---|---|---|---|---|---|---|---|---|---|---|---|
| 6317 | S7_77197754 | 7 | 77197754 | 38 | T | C | T | 0.95161 | C | 0.04839 | 3 | 0.09677 |
| 6318 | S7_77197775 | 7 | 77197775 | 21 | G | C | G | 0.93548 | C | 0.06452 | 4 | 0.12903 |
| 6319 | S7_77790424 | 7 | 77790424 | 592649 | C | G | C | 0.87097 | G | 0.12903 | 8 | 0.25806 |
| 6320 | S7_78838851 | 7 | 78838851 | 1048427 | A | G | A | 0.82258 | G | 0.17742 | 7 | 0.22581 |
| 6321 | S7_78838866 | 7 | 78838866 | 15 | C | G | C | 0.69355 | G | 0.30645 | 11 | 0.35484 |
| 6322 | S7_79571662 | 7 | 79571662 | 732796 | A | T | A | 0.85484 | T | 0.14516 | 5 | 0.16129 |
| 6323 | S7_80533783 | 7 | 80533783 | 962121 | C | T | C | 0.80645 | T | 0.19355 | 10 | 0.32258 |
| 6324 | S7_80732634 | 7 | 80732634 | 198851 | G | A | G | 0.83871 | A | 0.16129 | 10 | 0.32258 |
| 6325 | S7_80732647 | 7 | 80732647 | 13 | T | A | T | 0.83871 | A | 0.16129 | 8 | 0.25806 |
| 6326 | S7_80732787 | 7 | 80732787 | 140 | C | T | C | 0.53226 | T | 0.46774 | 15 | 0.48387 |
| 6327 | S7_80732808 | 7 | 80732808 | 21 | T | G | G | 0.77419 | T | 0.22581 | 10 | 0.32258 |
| 6328 | S7_81072226 | 7 | 81072226 | 339418 | T | G | T | 0.83871 | G | 0.16129 | 10 | 0.32258 |
| 6329 | S7_81217859 | 7 | 81217859 | 145633 | C | T | C | 0.90323 | T | 0.09677 | 4 | 0.12903 |
| 6330 | S7_81484732 | 7 | 81484732 | 266873 | T | G | G | 0.8871 | T | 0.1129 | 5 | 0.16129 |
| 6331 | S7_81484799 | 7 | 81484799 | 67 | G | A | A | 0.8871 | G | 0.1129 | 5 | 0.16129 |
| 6332 | S7_82675797 | 7 | 82675797 | 1190998 | G | C | G | 0.95161 | C | 0.04839 | 3 | 0.09677 |
| 6333 | S7_83002364 | 7 | 83002364 | 326567 | A | C | A | 0.95161 | C | 0.04839 | 3 | 0.09677 |
| 6334 | S7_83976859 | 7 | 83976859 | 974495 | G | A | G | 0.87097 | A | 0.12903 | 6 | 0.19355 |
| 6335 | S7_83976898 | 7 | 83976898 | 39 | C | T | C | 0.87097 | T | 0.12903 | 6 | 0.19355 |
| 6336 | S7_84595008 | 7 | 84595008 | 618110 | C | G | C | 0.95161 | G | 0.04839 | 3 | 0.09677 |
| 6337 | S7_84648052 | 7 | 84648052 | 53044 | C | T | T | 0.64516 | C | 0.35484 | 18 | 0.58065 |
| 6338 | S7_84648209 | 7 | 84648209 | 157 | T | C | C | 0.82258 | T | 0.17742 | 11 | 0.35484 |
| 6339 | S7_84670068 | 7 | 84670068 | 21859 | A | C | A | 0.53226 | C | 0.46774 | 17 | 0.54839 |
| 6340 | S7_85004748 | 7 | 85004748 | 334680 | T | C | T | 0.87097 | C | 0.12903 | 6 | 0.19355 |
| 6341 | S7_85004761 | 7 | 85004761 | 13 | C | T | C | 0.90323 | T | 0.09677 | 6 | 0.19355 |
| 6342 | S7_85004894 | 7 | 85004894 | 133 | C | T | C | 0.90323 | T | 0.09677 | 6 | 0.19355 |
| 6343 | S7_85092625 | 7 | 85092625 | 87731 | T | C | T | 0.95161 | C | 0.04839 | 3 | 0.09677 |
| 6344 | S7_85593823 | 7 | 85593823 | 501198 | A | C | A | 0.93548 | C | 0.06452 | 4 | 0.12903 |
| 6345 | S7_85686437 | 7 | 85686437 | 92614 | A | G | A | 0.90323 | G | 0.09677 | 6 | 0.19355 |
| 6346 | S7_85732104 | 7 | 85732104 | 45667 | C | T | C | 0.95161 | T | 0.04839 | 3 | 0.09677 |
| 6347 | S7_85732178 | 7 | 85732178 | 74 | T | C | C | 0.93548 | T | 0.06452 | 4 | 0.12903 |
| 6348 | S7_85732296 | 7 | 85732296 | 118 | A | T | T | 0.53226 | A | 0.46774 | 19 | 0.6129 |
| 6349 | S7_86263040 | 7 | 86263040 | 530744 | C | A | C | 0.95161 | A | 0.04839 | 3 | 0.09677 |
| 6350 | S7_86263262 | 7 | 86263262 | 222 | C | T | T | 0.91935 | C | 0.08065 | 5 | 0.16129 |
| 6351 | S7_86263263 | 7 | 86263263 | 1 | T | G | G | 0.91935 | T | 0.08065 | 5 | 0.16129 |
| 6352 | S7_88524531 | 7 | 88524531 | 2261268 | A | G | G | 0.54839 | A | 0.45161 | 20 | 0.64516 |
| 6353 | S7_88524713 | 7 | 88524713 | 182 | C | T | T | 0.69355 | C | 0.30645 | 11 | 0.35484 |
| 6354 | S7_88524717 | 7 | 88524717 | 4 | T | C | T | 0.80645 | C | 0.19355 | 10 | 0.32258 |
| 6355 | S7_88524739 | 7 | 88524739 | 22 | T | A | A | 0.62903 | T | 0.37097 | 15 | 0.48387 |
| 6356 | S7_88652485 | 7 | 88652485 | 127746 | T | C | T | 0.69355 | C | 0.30645 | 15 | 0.48387 |
| 6357 | S7_90041757 | 7 | 90041757 | 1389272 | T | A | T | 0.91935 | A | 0.08065 | 3 | 0.09677 |
| 6358 | S7_91058845 | 7 | 91058845 | 1017088 | G | A | G | 0.95161 | A | 0.04839 | 3 | 0.09677 |
| 6359 | S7_91058948 | 7 | 91058948 | 103 | G | A | G | 0.95161 | A | 0.04839 | 3 | 0.09677 |
| 6360 | S7_91125460 | 7 | 91125460 | 66512 | A | G | A | 0.5 | G | 0.5 | 11 | 0.35484 |
| 6361 | S7_91317350 | 7 | 91317350 | 191890 | C | T | C | 0.87097 | T | 0.12903 | 6 | 0.19355 |
| 6362 | S7_91406139 | 7 | 91406139 | 88789 | C | T | C | 0.93548 | T | 0.06452 | 2 | 0.06452 |
| 6363 | S7_91406169 | 7 | 91406169 | 30 | G | T | G | 0.93548 | T | 0.06452 | 2 | 0.06452 |
| 6364 | S7_91406193 | 7 | 91406193 | 24 | G | A | G | 0.66129 | A | 0.33871 | 11 | 0.35484 |
| 6365 | S7_91406232 | 7 | 91406232 | 39 | C | T | C | 0.91935 | T | 0.08065 | 5 | 0.16129 |
| 6366 | S7_91500483 | 7 | 91500483 | 94251 | C | T | C | 0.70968 | T | 0.29032 | 8 | 0.25806 |
| 6367 | S7_91596062 | 7 | 91596062 | 95579 | G | A | G | 0.56452 | A | 0.43548 | 17 | 0.54839 |
| 6368 | S7_91596068 | 7 | 91596068 | 6 | G | A | G | 0.56452 | A | 0.43548 | 17 | 0.54839 |
| 6369 | S7_91596226 | 7 | 91596226 | 158 | G | C | G | 0.82258 | C | 0.17742 | 11 | 0.35484 |
| 6370 | S7_91596251 | 7 | 91596251 | 25 | T | C | T | 0.56452 | C | 0.43548 | 17 | 0.54839 |

| Site number | SNP name | Chromosome | Physical position | Physical distance from the previous SNP | Reference allele | Alternative allele | Major allele | Major allele frequency | Minor allele | Minor allele frequency | Number heterozygous | Heterozygosity |
|---|---|---|---|---|---|---|---|---|---|---|---|---|
| 6371 | S7_91641750 | 7 | 91641750 | 45499 | G | A | G | 0.53226 | A | 0.46774 | 17 | 0.54839 |
| 6372 | S7_91641789 | 7 | 91641789 | 39 | C | A | C | 0.95161 | A | 0.04839 | 3 | 0.09677 |
| 6373 | S7_91829659 | 7 | 91829659 | 187870 | G | A | G | 0.8871 | A | 0.1129 | 5 | 0.16129 |
| 6374 | S7_91829800 | 7 | 91829800 | 141 | G | C | G | 0.87097 | C | 0.12903 | 6 | 0.19355 |
| 6375 | S7_92075357 | 7 | 92075357 | 245557 | A | G | A | 0.85484 | G | 0.14516 | 9 | 0.29032 |
| 6376 | S7_92178058 | 7 | 92178058 | 102701 | G | A | G | 0.82258 | A | 0.17742 | 9 | 0.29032 |
| 6377 | S7_92178085 | 7 | 92178085 | 27 | C | T | C | 0.93548 | T | 0.06452 | 4 | 0.12903 |
| 6378 | S7_92461485 | 7 | 92461485 | 283400 | C | A | C | 0.93548 | A | 0.06452 | 4 | 0.12903 |
| 6379 | S7_92473457 | 7 | 92473457 | 11972 | G | C | G | 0.69355 | C | 0.30645 | 11 | 0.35484 |
| 6380 | S7_92473489 | 7 | 92473489 | 32 | T | C | C | 0.90323 | T | 0.09677 | 6 | 0.19355 |
| 6381 | S7_92473495 | 7 | 92473495 | 6 | C | T | C | 0.69355 | T | 0.30645 | 11 | 0.35484 |
| 6382 | S7_92683919 | 7 | 92683919 | 210424 | A | G | A | 0.85484 | G | 0.14516 | 9 | 0.29032 |
| 6383 | S7_92683938 | 7 | 92683938 | 19 | T | C | T | 0.85484 | C | 0.14516 | 9 | 0.29032 |
| 6384 | S7_92683956 | 7 | 92683956 | 18 | G | A | G | 0.87097 | A | 0.12903 | 8 | 0.25806 |
| 6385 | S7_92683975 | 7 | 92683975 | 19 | G | A | G | 0.83871 | A | 0.16129 | 10 | 0.32258 |
| 6386 | S7_92684020 | 7 | 92684020 | 45 | T | C | T | 0.87097 | C | 0.12903 | 8 | 0.25806 |
| 6387 | S7_92684035 | 7 | 92684035 | 15 | T | C | T | 0.87097 | C | 0.12903 | 8 | 0.25806 |
| 6388 | S7_92684042 | 7 | 92684042 | 7 | C | T | C | 0.77419 | T | 0.22581 | 14 | 0.45161 |
| 6389 | S7_92684044 | 7 | 92684044 | 2 | A | G | A | 0.70968 | G | 0.29032 | 18 | 0.58065 |
| 6390 | S7_92684048 | 7 | 92684048 | 4 | T | C | T | 0.87097 | C | 0.12903 | 8 | 0.25806 |
| 6391 | S7_92684049 | 7 | 92684049 | 1 | A | G | A | 0.87097 | G | 0.12903 | 8 | 0.25806 |
| 6392 | S7_92684076 | 7 | 92684076 | 27 | G | C | G | 0.87097 | C | 0.12903 | 8 | 0.25806 |
| 6393 | S7_92688804 | 7 | 92688804 | 4728 | T | G | T | 0.77419 | G | 0.22581 | 14 | 0.45161 |
| 6394 | S7_92688806 | 7 | 92688806 | 2 | G | A | G | 0.87097 | A | 0.12903 | 8 | 0.25806 |
| 6395 | S7_92698067 | 7 | 92698067 | 9261 | C | T | C | 0.83871 | T | 0.16129 | 10 | 0.32258 |
| 6396 | S7_92698082 | 7 | 92698082 | 15 | C | G | C | 0.93548 | G | 0.06452 | 4 | 0.12903 |
| 6397 | S7_92993382 | 7 | 92993382 | 295300 | G | A | G | 0.79032 | A | 0.20968 | 11 | 0.35484 |
| 6398 | S7_92993383 | 7 | 92993383 | 1 | T | C | T | 0.79032 | C | 0.20968 | 11 | 0.35484 |
| 6399 | S7_92993386 | 7 | 92993386 | 3 | G | C | G | 0.79032 | C | 0.20968 | 11 | 0.35484 |
| 6400 | S7_92993387 | 7 | 92993387 | 1 | G | A | G | 0.79032 | A | 0.20968 | 11 | 0.35484 |
| 6401 | S7_92993449 | 7 | 92993449 | 62 | C | G | C | 0.82258 | G | 0.17742 | 9 | 0.29032 |
| 6402 | S7_92993490 | 7 | 92993490 | 41 | T | G | T | 0.79032 | G | 0.20968 | 11 | 0.35484 |
| 6403 | S7_92993496 | 7 | 92993496 | 6 | G | A | G | 0.77419 | A | 0.22581 | 12 | 0.3871 |
| 6404 | S7_92993588 | 7 | 92993588 | 92 | A | G | A | 0.79032 | G | 0.20968 | 11 | 0.35484 |
| 6405 | S7_93099270 | 7 | 93099270 | 105682 | G | A | G | 0.95161 | A | 0.04839 | 3 | 0.09677 |
| 6406 | S7_93099386 | 7 | 93099386 | 116 | G | A | G | 0.95161 | A | 0.04839 | 3 | 0.09677 |
| 6407 | S7_93202326 | 7 | 93202326 | 102940 | G | A | G | 0.93548 | A | 0.06452 | 4 | 0.12903 |
| 6408 | S7_93369473 | 7 | 93369473 | 167147 | G | A | G | 0.80645 | A | 0.19355 | 10 | 0.32258 |
| 6409 | S7_93369561 | 7 | 93369561 | 88 | G | C | G | 0.66129 | C | 0.33871 | 13 | 0.41935 |
| 6410 | S7_93509049 | 7 | 93509049 | 139488 | A | G | A | 0.74194 | G | 0.25806 | 14 | 0.45161 |
| 6411 | S7_93509222 | 7 | 93509222 | 173 | G | A | G | 0.62903 | A | 0.37097 | 17 | 0.54839 |
| 6412 | S7_93509229 | 7 | 93509229 | 7 | A | G | A | 0.90323 | G | 0.09677 | 6 | 0.19355 |
| 6413 | S7_93509239 | 7 | 93509239 | 10 | T | C | C | 0.53226 | T | 0.46774 | 13 | 0.41935 |
| 6414 | S7_93509245 | 7 | 93509245 | 6 | C | T | C | 0.83871 | T | 0.16129 | 8 | 0.25806 |
| 6415 | S7_93567862 | 7 | 93567862 | 58617 | G | A | G | 0.95161 | A | 0.04839 | 3 | 0.09677 |
| 6416 | S7_94094939 | 7 | 94094939 | 527077 | G | A | A | 0.51613 | G | 0.48387 | 16 | 0.51613 |
| 6417 | S7_94094961 | 7 | 94094961 | 22 | C | T | C | 0.5 | T | 0.5 | 17 | 0.54839 |
| 6418 | S7_94100459 | 7 | 94100459 | 5498 | C | A | C | 0.64516 | A | 0.35484 | 14 | 0.45161 |
| 6419 | S7_94354951 | 7 | 94354951 | 254492 | G | A | A | 0.8871 | G | 0.1129 | 5 | 0.16129 |
| 6420 | S7_94547770 | 7 | 94547770 | 192819 | A | G | G | 0.74194 | A | 0.25806 | 12 | 0.3871 |
| 6421 | S7_94547772 | 7 | 94547772 | 2 | A | G | A | 0.85484 | G | 0.14516 | 9 | 0.29032 |
| 6422 | S7_94584715 | 7 | 94584715 | 36943 | G | A | G | 0.90323 | A | 0.09677 | 6 | 0.19355 |
| 6423 | S7_94584722 | 7 | 94584722 | 7 | A | G | G | 0.66129 | A | 0.33871 | 17 | 0.54839 |
| 6424 | S7_95369867 | 7 | 95369867 | 785145 | C | T | C | 0.93548 | T | 0.06452 | 4 | 0.12903 |

| Site number | SNP name | Chromosome | Physical position | Physical distance from the previous SNP | Reference allele | Alternative allele | Major allele | Major allele frequency | Minor allele | Minor allele frequency | Number heterozygous | Heterozygosity |
|---|---|---|---|---|---|---|---|---|---|---|---|---|
| 6425 | S7_95369901 | 7 | 95369901 | 34 | G | A | G | 0.69355 | A | 0.30645 | 11 | 0.35484 |
| 6426 | S7_96136416 | 7 | 96136416 | 766515 | C | T | C | 0.66129 | T | 0.33871 | 15 | 0.48387 |
| 6427 | S7_96136432 | 7 | 96136432 | 16 | T | C | T | 0.93548 | C | 0.06452 | 4 | 0.12903 |
| 6428 | S7_96136498 | 7 | 96136498 | 66 | T | G | T | 0.75806 | G | 0.24194 | 13 | 0.41935 |
| 6429 | S7_96136501 | 7 | 96136501 | 3 | T | C | T | 0.8871 | C | 0.1129 | 5 | 0.16129 |
| 6430 | S7_96136627 | 7 | 96136627 | 126 | T | C | T | 0.93548 | C | 0.06452 | 4 | 0.12903 |
| 6431 | S7_96154918 | 7 | 96154918 | 18291 | G | A | A | 0.53226 | G | 0.46774 | 19 | 0.6129 |
| 6432 | S7_97055405 | 7 | 97055405 | 900487 | T | C | C | 0.95161 | T | 0.04839 | 3 | 0.09677 |
| 6433 | S7_97059003 | 7 | 97059003 | 3598 | A | G | A | 0.85484 | G | 0.14516 | 5 | 0.16129 |
| 6434 | S7_97156896 | 7 | 97156896 | 97893 | T | C | T | 0.91935 | C | 0.08065 | 5 | 0.16129 |
| 6435 | S7_97236562 | 7 | 97236562 | 79666 | G | A | G | 0.93548 | A | 0.06452 | 4 | 0.12903 |
| 6436 | S7_97303663 | 7 | 97303663 | 67101 | T | G | T | 0.53226 | G | 0.46774 | 13 | 0.41935 |
| 6437 | S7_97303701 | 7 | 97303701 | 38 | G | T | G | 0.51613 | T | 0.48387 | 14 | 0.45161 |
| 6438 | S7_97303732 | 7 | 97303732 | 31 | A | C | A | 0.51613 | C | 0.48387 | 14 | 0.45161 |
| 6439 | S7_97303759 | 7 | 97303759 | 27 | C | T | C | 0.51613 | T | 0.48387 | 14 | 0.45161 |
| 6440 | S7_97328674 | 7 | 97328674 | 24915 | A | G | A | 0.66129 | G | 0.33871 | 13 | 0.41935 |
| 6441 | S7_97328726 | 7 | 97328726 | 52 | C | T | C | 0.77419 | T | 0.22581 | 8 | 0.25806 |
| 6442 | S7_97398429 | 7 | 97398429 | 69703 | T | C | T | 0.95161 | C | 0.04839 | 3 | 0.09677 |
| 6443 | S7_97439416 | 7 | 97439416 | 40987 | C | T | C | 0.79032 | T | 0.20968 | 13 | 0.41935 |
| 6444 | S7_97439438 | 7 | 97439438 | 22 | G | T | G | 0.85484 | T | 0.14516 | 9 | 0.29032 |
| 6445 | S7_97439442 | 7 | 97439442 | 4 | T | C | T | 0.87097 | C | 0.12903 | 8 | 0.25806 |
| 6446 | S7_97439506 | 7 | 97439506 | 64 | T | C | T | 0.87097 | C | 0.12903 | 8 | 0.25806 |
| 6447 | S7_97439583 | 7 | 97439583 | 77 | T | C | C | 0.75806 | T | 0.24194 | 15 | 0.48387 |
| 6448 | S7_97589129 | 7 | 97589129 | 149546 | C | T | C | 0.75806 | T | 0.24194 | 13 | 0.41935 |
| 6449 | S7_97589141 | 7 | 97589141 | 12 | C | G | C | 0.77419 | G | 0.22581 | 8 | 0.25806 |
| 6450 | S7_97589163 | 7 | 97589163 | 22 | G | A | G | 0.74194 | A | 0.25806 | 12 | 0.3871 |
| 6451 | S7_97589189 | 7 | 97589189 | 26 | G | A | G | 0.74194 | A | 0.25806 | 12 | 0.3871 |
| 6452 | S7_97589242 | 7 | 97589242 | 53 | G | A | G | 0.74194 | A | 0.25806 | 12 | 0.3871 |
| 6453 | S7_97589323 | 7 | 97589323 | 81 | C | T | C | 0.79032 | T | 0.20968 | 11 | 0.35484 |
| 6454 | S7_97589336 | 7 | 97589336 | 13 | A | G | A | 0.74194 | G | 0.25806 | 12 | 0.3871 |
| 6455 | S7_97589354 | 7 | 97589354 | 18 | T | C | T | 0.74194 | C | 0.25806 | 12 | 0.3871 |
| 6456 | S7_97589356 | 7 | 97589356 | 2 | T | C | T | 0.74194 | C | 0.25806 | 12 | 0.3871 |
| 6457 | S7_97605276 | 7 | 97605276 | 15920 | C | T | T | 0.51613 | C | 0.48387 | 18 | 0.58065 |
| 6458 | S7_97605462 | 7 | 97605462 | 186 | T | A | A | 0.80645 | T | 0.19355 | 8 | 0.25806 |
| 6459 | S7_97668827 | 7 | 97668827 | 63365 | T | C | C | 0.69355 | T | 0.30645 | 11 | 0.35484 |
| 6460 | S7_97668894 | 7 | 97668894 | 67 | T | A | T | 0.87097 | A | 0.12903 | 8 | 0.25806 |
| 6461 | S7_97669015 | 7 | 97669015 | 121 | C | T | C | 0.90323 | T | 0.09677 | 6 | 0.19355 |
| 6462 | S7_97730878 | 7 | 97730878 | 61863 | A | C | A | 0.70968 | C | 0.29032 | 10 | 0.32258 |
| 6463 | S7_97730960 | 7 | 97730960 | 82 | A | G | G | 0.56452 | A | 0.43548 | 17 | 0.54839 |
| 6464 | S7_97730966 | 7 | 97730966 | 6 | T | C | C | 0.77419 | T | 0.22581 | 10 | 0.32258 |
| 6465 | S7_97730997 | 7 | 97730997 | 31 | T | G | T | 0.77419 | G | 0.22581 | 12 | 0.3871 |
| 6466 | S7_97731112 | 7 | 97731112 | 115 | G | A | A | 0.69355 | G | 0.30645 | 13 | 0.41935 |
| 6467 | S7_97775111 | 7 | 97775111 | 43999 | G | T | G | 0.93548 | T | 0.06452 | 2 | 0.06452 |
| 6468 | S7_97826919 | 7 | 97826919 | 51808 | G | A | G | 0.85484 | A | 0.14516 | 5 | 0.16129 |
| 6469 | S7_97964270 | 7 | 97964270 | 137351 | A | G | G | 0.91935 | A | 0.08065 | 5 | 0.16129 |
| 6470 | S7_98000184 | 7 | 98000184 | 35914 | A | T | A | 0.75806 | T | 0.24194 | 9 | 0.29032 |
| 6471 | S7_98000260 | 7 | 98000260 | 76 | T | C | T | 0.70968 | C | 0.29032 | 12 | 0.3871 |
| 6472 | S7_98000326 | 7 | 98000326 | 66 | C | T | C | 0.91935 | T | 0.08065 | 5 | 0.16129 |
| 6473 | S7_98000355 | 7 | 98000355 | 29 | G | C | G | 0.75806 | C | 0.24194 | 9 | 0.29032 |
| 6474 | S7_98287600 | 7 | 98287600 | 287245 | A | C | A | 0.74194 | C | 0.25806 | 10 | 0.32258 |
| 6475 | S7_98287696 | 7 | 98287696 | 96 | C | T | C | 0.67742 | T | 0.32258 | 10 | 0.32258 |
| 6476 | S7_98287711 | 7 | 98287711 | 15 | C | T | C | 0.85484 | T | 0.14516 | 7 | 0.22581 |
| 6477 | S7_98353812 | 7 | 98353812 | 66101 | T | C | T | 0.91935 | C | 0.08065 | 5 | 0.16129 |
| 6478 | S7_98417807 | 7 | 98417807 | 63995 | T | G | T | 0.91935 | G | 0.08065 | 5 | 0.16129 |

| Site number | SNP name | Chromosome | Physical position | Physical distance from the previous SNP | Reference allele | Alternative allele | Major allele | Major allele frequency | Minor allele | Minor allele frequency | Number heterozygous | Heterozygosity |
|---|---|---|---|---|---|---|---|---|---|---|---|---|
| 6479 | S7_98417975 | 7 | 98417975 | 168 | T | C | C | 0.67742 | T | 0.32258 | 12 | 0.3871 |
| 6480 | S7_98445590 | 7 | 98445590 | 27615 | T | C | C | 0.64516 | T | 0.35484 | 18 | 0.58065 |
| 6481 | S7_98657019 | 7 | 98657019 | 211429 | T | C | C | 0.83871 | T | 0.16129 | 6 | 0.19355 |
| 6482 | S7_98657030 | 7 | 98657030 | 11 | G | A | A | 0.53226 | G | 0.46774 | 21 | 0.67742 |
| 6483 | S7_98657079 | 7 | 98657079 | 49 | C | T | C | 0.54839 | T | 0.45161 | 16 | 0.51613 |
| 6484 | S7_98657104 | 7 | 98657104 | 25 | T | A | T | 0.95161 | A | 0.04839 | 3 | 0.09677 |
| 6485 | S7_98657105 | 7 | 98657105 | 1 | C | G | G | 0.53226 | C | 0.46774 | 21 | 0.67742 |
| 6486 | S7_98657153 | 7 | 98657153 | 48 | C | T | T | 0.53226 | C | 0.46774 | 21 | 0.67742 |
| 6487 | S7_98657159 | 7 | 98657159 | 6 | C | T | T | 0.53226 | C | 0.46774 | 21 | 0.67742 |
| 6488 | S7_99100751 | 7 | 99100751 | 443592 | T | A | A | 0.62903 | T | 0.37097 | 17 | 0.54839 |
| 6489 | S7_99100772 | 7 | 99100772 | 21 | A | G | G | 0.62903 | A | 0.37097 | 17 | 0.54839 |
| 6490 | S7_99100779 | 7 | 99100779 | 7 | G | A | G | 0.8871 | A | 0.1129 | 7 | 0.22581 |
| 6491 | S7_99100782 | 7 | 99100782 | 3 | G | A | A | 0.54839 | G | 0.45161 | 20 | 0.64516 |
| 6492 | S7_99100787 | 7 | 99100787 | 5 | C | A | A | 0.62903 | C | 0.37097 | 17 | 0.54839 |
| 6493 | S7_99100797 | 7 | 99100797 | 10 | C | T | T | 0.62903 | C | 0.37097 | 17 | 0.54839 |
| 6494 | S7_99100806 | 7 | 99100806 | 9 | T | G | G | 0.62903 | T | 0.37097 | 17 | 0.54839 |
| 6495 | S7_99100946 | 7 | 99100946 | 140 | A | T | A | 0.80645 | T | 0.19355 | 12 | 0.3871 |
| 6496 | S7_99544880 | 7 | 99544880 | 443934 | C | T | C | 0.95161 | T | 0.04839 | 3 | 0.09677 |
| 6497 | S7_100124002 | 7 | 100124002 | 579122 | G | A | G | 0.91935 | A | 0.08065 | 5 | 0.16129 |
| 6498 | S7_100124022 | 7 | 100124022 | 20 | G | A | G | 0.91935 | A | 0.08065 | 5 | 0.16129 |
| 6499 | S7_100124031 | 7 | 100124031 | 9 | T | C | C | 0.58065 | T | 0.41935 | 16 | 0.51613 |
| 6500 | S7_100124097 | 7 | 100124097 | 66 | G | A | G | 0.93548 | A | 0.06452 | 4 | 0.12903 |
| 6501 | S7_100124164 | 7 | 100124164 | 67 | T | A | T | 0.95161 | A | 0.04839 | 3 | 0.09677 |
| 6502 | S7_100124181 | 7 | 100124181 | 17 | T | G | T | 0.51613 | G | 0.48387 | 18 | 0.58065 |
| 6503 | S7_100502137 | 7 | 100502137 | 377956 | A | G | A | 0.93548 | G | 0.06452 | 4 | 0.12903 |
| 6504 | S7_100502138 | 7 | 100502138 | 1 | T | G | T | 0.69355 | G | 0.30645 | 17 | 0.54839 |
| 6505 | S7_100707761 | 7 | 100707761 | 205623 | A | G | G | 0.75806 | A | 0.24194 | 9 | 0.29032 |
| 6506 | S7_100707895 | 7 | 100707895 | 134 | C | T | T | 0.77419 | C | 0.22581 | 10 | 0.32258 |
| 6507 | S7_101082869 | 7 | 101082869 | 374974 | G | C | G | 0.72581 | C | 0.27419 | 11 | 0.35484 |
| 6508 | S7_101082913 | 7 | 101082913 | 44 | G | A | G | 0.72581 | A | 0.27419 | 11 | 0.35484 |
| 6509 | S7_101082944 | 7 | 101082944 | 31 | A | G | A | 0.59677 | G | 0.40323 | 13 | 0.41935 |
| 6510 | S7_101082948 | 7 | 101082948 | 4 | T | A | T | 0.59677 | A | 0.40323 | 13 | 0.41935 |
| 6511 | S7_101087685 | 7 | 101087685 | 4737 | C | T | C | 0.64516 | T | 0.35484 | 14 | 0.45161 |
| 6512 | S7_101087757 | 7 | 101087757 | 72 | C | T | C | 0.5 | T | 0.5 | 19 | 0.6129 |
| 6513 | S7_101087787 | 7 | 101087787 | 30 | G | A | G | 0.93548 | A | 0.06452 | 4 | 0.12903 |
| 6514 | S7_101087895 | 7 | 101087895 | 108 | G | A | G | 0.62903 | A | 0.37097 | 13 | 0.41935 |
| 6515 | S7_101091413 | 7 | 101091413 | 3518 | G | A | G | 0.93548 | A | 0.06452 | 4 | 0.12903 |
| 6516 | S7_101243491 | 7 | 101243491 | 152078 | C | T | T | 0.87097 | C | 0.12903 | 6 | 0.19355 |
| 6517 | S7_101448399 | 7 | 101448399 | 204908 | T | C | T | 0.90323 | C | 0.09677 | 4 | 0.12903 |
| 6518 | S7_101448416 | 7 | 101448416 | 17 | T | C | T | 0.80645 | C | 0.19355 | 10 | 0.32258 |
| 6519 | S7_101589809 | 7 | 101589809 | 141393 | G | A | G | 0.83871 | A | 0.16129 | 6 | 0.19355 |
| 6520 | S7_101589860 | 7 | 101589860 | 51 | C | A | C | 0.82258 | A | 0.17742 | 7 | 0.22581 |
| 6521 | S7_101690408 | 7 | 101690408 | 100548 | C | A | C | 0.83871 | A | 0.16129 | 10 | 0.32258 |
| 6522 | S7_101690415 | 7 | 101690415 | 7 | G | T | G | 0.83871 | T | 0.16129 | 10 | 0.32258 |
| 6523 | S7_101690555 | 7 | 101690555 | 140 | A | G | A | 0.83871 | G | 0.16129 | 10 | 0.32258 |
| 6524 | S7_101817467 | 7 | 101817467 | 126912 | T | C | T | 0.67742 | C | 0.32258 | 12 | 0.3871 |
| 6525 | S7_101817528 | 7 | 101817528 | 61 | T | C | T | 0.72581 | C | 0.27419 | 11 | 0.35484 |
| 6526 | S7_101817540 | 7 | 101817540 | 12 | A | G | A | 0.67742 | G | 0.32258 | 12 | 0.3871 |
| 6527 | S7_101817633 | 7 | 101817633 | 93 | T | C | T | 0.77419 | C | 0.22581 | 10 | 0.32258 |
| 6528 | S7_101869847 | 7 | 101869847 | 52214 | G | A | G | 0.80645 | A | 0.19355 | 10 | 0.32258 |
| 6529 | S7_101914936 | 7 | 101914936 | 45089 | A | G | G | 0.72581 | A | 0.27419 | 15 | 0.48387 |
| 6530 | S7_101914947 | 7 | 101914947 | 11 | C | T | C | 0.93548 | T | 0.06452 | 4 | 0.12903 |
| 6531 | S7_101979037 | 7 | 101979037 | 64090 | C | G | G | 0.51613 | C | 0.48387 | 14 | 0.45161 |
| 6532 | S7_101979072 | 7 | 101979072 | 35 | C | G | C | 0.67742 | G | 0.32258 | 12 | 0.3871 |

| Site number | SNP name | Chromosome | Physical position | Physical distance from the previous SNP | Reference allele | Alternative allele | Major allele | Major allele frequency | Minor allele | Minor allele frequency | Number heterozygous | Heterozygosity |
|---|---|---|---|---|---|---|---|---|---|---|---|---|
| 6533 | S7_101979172 | 7 | 101979172 | 100 | C | T | C | 0.90323 | T | 0.09677 | 6 | 0.19355 |
| 6534 | S7_101979234 | 7 | 101979234 | 62 | A | G | G | 0.64516 | A | 0.35484 | 14 | 0.45161 |
| 6535 | S7_101979273 | 7 | 101979273 | 39 | G | T | T | 0.51613 | G | 0.48387 | 14 | 0.45161 |
| 6536 | S7_102262848 | 7 | 102262848 | 283575 | G | A | G | 0.90323 | A | 0.09677 | 6 | 0.19355 |
| 6537 | S7_102262962 | 7 | 102262962 | 114 | C | T | C | 0.95161 | T | 0.04839 | 3 | 0.09677 |
| 6538 | S7_102262969 | 7 | 102262969 | 7 | C | T | C | 0.91935 | T | 0.08065 | 3 | 0.09677 |
| 6539 | S7_102455778 | 7 | 102455778 | 192809 | T | C | T | 0.58065 | C | 0.41935 | 12 | 0.3871 |
| 6540 | S7_102556126 | 7 | 102556126 | 100348 | G | T | G | 0.77419 | T | 0.22581 | 10 | 0.32258 |
| 6541 | S7_102791652 | 7 | 102791652 | 235526 | C | A | C | 0.80645 | A | 0.19355 | 8 | 0.25806 |
| 6542 | S7_102826847 | 7 | 102826847 | 35195 | C | T | C | 0.83871 | T | 0.16129 | 8 | 0.25806 |
| 6543 | S7_102826927 | 7 | 102826927 | 80 | G | A | G | 0.93548 | A | 0.06452 | 4 | 0.12903 |
| 6544 | S7_102827060 | 7 | 102827060 | 133 | C | T | C | 0.66129 | T | 0.33871 | 11 | 0.35484 |
| 6545 | S7_103123457 | 7 | 103123457 | 296397 | C | T | C | 0.91935 | T | 0.08065 | 5 | 0.16129 |
| 6546 | S7_103338161 | 7 | 103338161 | 214704 | G | T | T | 0.6129 | G | 0.3871 | 12 | 0.3871 |
| 6547 | S7_103338238 | 7 | 103338238 | 77 | T | C | T | 0.87097 | C | 0.12903 | 6 | 0.19355 |
| 6548 | S7_103338294 | 7 | 103338294 | 56 | G | C | G | 0.64516 | C | 0.35484 | 12 | 0.3871 |
| 6549 | S7_103422936 | 7 | 103422936 | 84642 | A | G | A | 0.90323 | G | 0.09677 | 6 | 0.19355 |
| 6550 | S7_103422941 | 7 | 103422941 | 5 | C | T | C | 0.93548 | T | 0.06452 | 4 | 0.12903 |
| 6551 | S7_103923611 | 7 | 103923611 | 500670 | T | A | T | 0.93548 | A | 0.06452 | 4 | 0.12903 |
| 6552 | S7_104253602 | 7 | 104253602 | 329991 | T | C | T | 0.83871 | C | 0.16129 | 8 | 0.25806 |
| 6553 | S7_104253681 | 7 | 104253681 | 79 | G | T | G | 0.79032 | T | 0.20968 | 7 | 0.22581 |
| 6554 | S7_104292654 | 7 | 104292654 | 38973 | C | T | C | 0.62903 | T | 0.37097 | 15 | 0.48387 |
| 6555 | S7_104489545 | 7 | 104489545 | 196891 | C | T | C | 0.79032 | T | 0.20968 | 7 | 0.22581 |
| 6556 | S7_104489599 | 7 | 104489599 | 54 | G | A | A | 0.6129 | G | 0.3871 | 14 | 0.45161 |
| 6557 | S7_104489604 | 7 | 104489604 | 5 | G | A | G | 0.91935 | A | 0.08065 | 5 | 0.16129 |
| 6558 | S7_104489681 | 7 | 104489681 | 77 | C | T | C | 0.79032 | T | 0.20968 | 7 | 0.22581 |
| 6559 | S7_104980033 | 7 | 104980033 | 490352 | A | C | C | 0.69355 | A | 0.30645 | 11 | 0.35484 |
| 6560 | S7_104980190 | 7 | 104980190 | 157 | C | T | C | 0.91935 | T | 0.08065 | 5 | 0.16129 |
| 6561 | S7_105034592 | 7 | 105034592 | 54402 | A | G | A | 0.95161 | G | 0.04839 | 3 | 0.09677 |
| 6562 | S7_105336734 | 7 | 105336734 | 302142 | C | T | C | 0.82258 | T | 0.17742 | 11 | 0.35484 |
| 6563 | S7_105336826 | 7 | 105336826 | 92 | C | T | C | 0.79032 | T | 0.20968 | 9 | 0.29032 |
| 6564 | S7_105336851 | 7 | 105336851 | 25 | C | G | C | 0.58065 | G | 0.41935 | 16 | 0.51613 |
| 6565 | S7_105337693 | 7 | 105337693 | 842 | G | A | G | 0.90323 | A | 0.09677 | 6 | 0.19355 |
| 6566 | S7_105337885 | 7 | 105337885 | 192 | A | T | T | 0.83871 | A | 0.16129 | 8 | 0.25806 |
| 6567 | S7_105802177 | 7 | 105802177 | 464292 | C | T | C | 0.93548 | T | 0.06452 | 4 | 0.12903 |
| 6568 | S7_105802271 | 7 | 105802271 | 94 | T | G | T | 0.58065 | G | 0.41935 | 16 | 0.51613 |
| 6569 | S7_106104807 | 7 | 106104807 | 302536 | T | C | T | 0.93548 | C | 0.06452 | 2 | 0.06452 |
| 6570 | S7_106104846 | 7 | 106104846 | 39 | A | G | G | 0.58065 | A | 0.41935 | 16 | 0.51613 |
| 6571 | S7_106104855 | 7 | 106104855 | 9 | T | C | C | 0.62903 | T | 0.37097 | 15 | 0.48387 |
| 6572 | S7_106104922 | 7 | 106104922 | 67 | T | C | T | 0.72581 | C | 0.27419 | 13 | 0.41935 |
| 6573 | S7_106104940 | 7 | 106104940 | 18 | C | T | C | 0.80645 | T | 0.19355 | 10 | 0.32258 |
| 6574 | S7_106104970 | 7 | 106104970 | 30 | T | C | T | 0.62903 | C | 0.37097 | 9 | 0.29032 |
| 6575 | S7_106104996 | 7 | 106104996 | 26 | A | G | A | 0.56452 | G | 0.43548 | 15 | 0.48387 |
| 6576 | S7_106250922 | 7 | 106250922 | 145926 | G | A | G | 0.93548 | A | 0.06452 | 4 | 0.12903 |
| 6577 | S7_106251033 | 7 | 106251033 | 111 | C | A | C | 0.67742 | A | 0.32258 | 12 | 0.3871 |
| 6578 | S7_106251043 | 7 | 106251043 | 10 | A | G | A | 0.8871 | G | 0.1129 | 7 | 0.22581 |
| 6579 | S7_106299903 | 7 | 106299903 | 48860 | C | T | T | 0.53226 | C | 0.46774 | 17 | 0.54839 |
| 6580 | S7_106299937 | 7 | 106299937 | 34 | G | A | G | 0.91935 | A | 0.08065 | 5 | 0.16129 |
| 6581 | S7_106338580 | 7 | 106338580 | 38643 | A | G | A | 0.95161 | G | 0.04839 | 1 | 0.03226 |
| 6582 | S7_106616619 | 7 | 106616619 | 278039 | G | A | G | 0.79032 | A | 0.20968 | 9 | 0.29032 |
| 6583 | S7_106616769 | 7 | 106616769 | 150 | T | C | T | 0.79032 | C | 0.20968 | 9 | 0.29032 |
| 6584 | S7_106616781 | 7 | 106616781 | 12 | G | A | G | 0.79032 | A | 0.20968 | 9 | 0.29032 |
| 6585 | S7_106744431 | 7 | 106744431 | 127650 | C | T | T | 0.64516 | C | 0.35484 | 12 | 0.3871 |
| 6586 | S7_106744490 | 7 | 106744490 | 59 | T | C | T | 0.64516 | C | 0.35484 | 12 | 0.3871 |

| Site number | SNP name | Chromosome | Physical position | Physical distance from the previous SNP | Reference allele | Alternative allele | Major allele | Major allele frequency | Minor allele | Minor allele frequency | Number heterozygous | Heterozygosity |
|---|---|---|---|---|---|---|---|---|---|---|---|---|
| 6587 | S7_107119963 | 7 | 107119963 | 375473 | A | C | A | 0.8871 | C | 0.1129 | 7 | 0.22581 |
| 6588 | S7_107132862 | 7 | 107132862 | 12899 | C | T | C | 0.8871 | T | 0.1129 | 7 | 0.22581 |
| 6589 | S7_107132954 | 7 | 107132954 | 92 | G | A | G | 0.58065 | A | 0.41935 | 12 | 0.3871 |
| 6590 | S7_107276273 | 7 | 107276273 | 143319 | T | C | T | 0.75806 | C | 0.24194 | 15 | 0.48387 |
| 6591 | S7_107276278 | 7 | 107276278 | 5 | T | C | T | 0.74194 | C | 0.25806 | 16 | 0.51613 |
| 6592 | S7_107276367 | 7 | 107276367 | 89 | C | T | C | 0.74194 | T | 0.25806 | 16 | 0.51613 |
| 6593 | S7_107276387 | 7 | 107276387 | 20 | C | T | C | 0.90323 | T | 0.09677 | 6 | 0.19355 |
| 6594 | S7_107398188 | 7 | 107398188 | 121801 | G | C | G | 0.90323 | C | 0.09677 | 6 | 0.19355 |
| 6595 | S7_107398358 | 7 | 107398358 | 170 | G | A | G | 0.93548 | A | 0.06452 | 4 | 0.12903 |
| 6596 | S7_107503172 | 7 | 107503172 | 104814 | A | C | C | 0.83871 | A | 0.16129 | 8 | 0.25806 |
| 6597 | S7_107503222 | 7 | 107503222 | 50 | C | T | C | 0.85484 | T | 0.14516 | 7 | 0.22581 |
| 6598 | S7_107503337 | 7 | 107503337 | 115 | A | G | A | 0.74194 | G | 0.25806 | 14 | 0.45161 |
| 6599 | S7_107529483 | 7 | 107529483 | 26146 | G | A | G | 0.85484 | A | 0.14516 | 7 | 0.22581 |
| 6600 | S7_107529484 | 7 | 107529484 | 1 | T | C | T | 0.83871 | C | 0.16129 | 6 | 0.19355 |
| 6601 | S7_107529509 | 7 | 107529509 | 25 | C | A | C | 0.93548 | A | 0.06452 | 4 | 0.12903 |
| 6602 | S7_107529550 | 7 | 107529550 | 41 | C | T | C | 0.90323 | T | 0.09677 | 6 | 0.19355 |
| 6603 | S7_107529635 | 7 | 107529635 | 85 | G | A | G | 0.95161 | A | 0.04839 | 3 | 0.09677 |
| 6604 | S7_107622416 | 7 | 107622416 | 92781 | A | G | A | 0.91935 | G | 0.08065 | 5 | 0.16129 |
| 6605 | S7_107622464 | 7 | 107622464 | 48 | A | G | A | 0.91935 | G | 0.08065 | 5 | 0.16129 |
| 6606 | S7_107622510 | 7 | 107622510 | 46 | G | A | G | 0.91935 | A | 0.08065 | 5 | 0.16129 |
| 6607 | S7_107622512 | 7 | 107622512 | 2 | C | G | C | 0.91935 | G | 0.08065 | 5 | 0.16129 |
| 6608 | S7_107622514 | 7 | 107622514 | 2 | C | T | C | 0.91935 | T | 0.08065 | 5 | 0.16129 |
| 6609 | S7_107622564 | 7 | 107622564 | 50 | C | T | C | 0.91935 | T | 0.08065 | 5 | 0.16129 |
| 6610 | S7_107835273 | 7 | 107835273 | 212709 | C | A | A | 0.95161 | C | 0.04839 | 3 | 0.09677 |
| 6611 | S7_107835369 | 7 | 107835369 | 96 | A | G | A | 0.75806 | G | 0.24194 | 9 | 0.29032 |
| 6612 | S7_107835470 | 7 | 107835470 | 101 | A | C | A | 0.75806 | C | 0.24194 | 9 | 0.29032 |
| 6613 | S7_107835475 | 7 | 107835475 | 5 | A | G | G | 0.95161 | A | 0.04839 | 3 | 0.09677 |
| 6614 | S7_107835505 | 7 | 107835505 | 30 | C | G | G | 0.95161 | C | 0.04839 | 3 | 0.09677 |
| 6615 | S7_107865811 | 7 | 107865811 | 30306 | C | A | C | 0.77419 | A | 0.22581 | 12 | 0.3871 |
| 6616 | S7_107865888 | 7 | 107865888 | 77 | C | T | C | 0.85484 | T | 0.14516 | 9 | 0.29032 |
| 6617 | S7_107865919 | 7 | 107865919 | 31 | G | A | G | 0.93548 | A | 0.06452 | 4 | 0.12903 |
| 6618 | S7_107865940 | 7 | 107865940 | 21 | C | T | C | 0.85484 | T | 0.14516 | 9 | 0.29032 |
| 6619 | S7_107947690 | 7 | 107947690 | 81750 | C | T | C | 0.91935 | T | 0.08065 | 5 | 0.16129 |
| 6620 | S7_107947696 | 7 | 107947696 | 6 | A | T | T | 0.53226 | A | 0.46774 | 17 | 0.54839 |
| 6621 | S7_107947812 | 7 | 107947812 | 116 | G | A | G | 0.93548 | A | 0.06452 | 4 | 0.12903 |
| 6622 | S8_700180 | 8 | 700180 | 0 | C | T | C | 0.90323 | T | 0.09677 | 4 | 0.12903 |
| 6623 | S8_713526 | 8 | 713526 | 13346 | C | T | C | 0.91935 | T | 0.08065 | 5 | 0.16129 |
| 6624 | S8_713631 | 8 | 713631 | 105 | T | G | T | 0.93548 | G | 0.06452 | 4 | 0.12903 |
| 6625 | S8_953786 | 8 | 953786 | 240155 | G | A | G | 0.83871 | A | 0.16129 | 8 | 0.25806 |
| 6626 | S8_1019147 | 8 | 1019147 | 65361 | G | A | G | 0.87097 | A | 0.12903 | 4 | 0.12903 |
| 6627 | S8_1221057 | 8 | 1221057 | 201910 | T | C | T | 0.54839 | C | 0.45161 | 12 | 0.3871 |
| 6628 | S8_1436848 | 8 | 1436848 | 215791 | A | G | G | 0.59677 | A | 0.40323 | 13 | 0.41935 |
| 6629 | S8_1436869 | 8 | 1436869 | 21 | A | G | A | 0.93548 | G | 0.06452 | 4 | 0.12903 |
| 6630 | S8_1694147 | 8 | 1694147 | 257278 | C | T | C | 0.91935 | T | 0.08065 | 5 | 0.16129 |
| 6631 | S8_1694296 | 8 | 1694296 | 149 | G | C | G | 0.91935 | C | 0.08065 | 5 | 0.16129 |
| 6632 | S8_1694387 | 8 | 1694387 | 91 | G | C | G | 0.70968 | C | 0.29032 | 14 | 0.45161 |
| 6633 | S8_1753462 | 8 | 1753462 | 59075 | T | C | C | 0.79032 | T | 0.20968 | 13 | 0.41935 |
| 6634 | S8_1753592 | 8 | 1753592 | 130 | T | C | T | 0.80645 | C | 0.19355 | 12 | 0.3871 |
| 6635 | S8_1759437 | 8 | 1759437 | 5845 | T | C | T | 0.8871 | C | 0.1129 | 7 | 0.22581 |
| 6636 | S8_1759485 | 8 | 1759485 | 48 | C | T | C | 0.79032 | T | 0.20968 | 13 | 0.41935 |
| 6637 | S8_1759581 | 8 | 1759581 | 96 | G | T | G | 0.90323 | T | 0.09677 | 6 | 0.19355 |
| 6638 | S8_1823008 | 8 | 1823008 | 63427 | A | G | G | 0.83871 | A | 0.16129 | 10 | 0.32258 |
| 6639 | S8_1823010 | 8 | 1823010 | 2 | G | A | G | 0.8871 | A | 0.1129 | 7 | 0.22581 |
| 6640 | S8_1823011 | 8 | 1823011 | 1 | C | T | C | 0.85484 | T | 0.14516 | 9 | 0.29032 |

| Site number | SNP name | Chromosome | Physical position | Physical distance from the previous SNP | Reference allele | Alternative allele | Major allele | Major allele frequency | Minor allele | Minor allele frequency | Number heterozygous | Heterozygosity |
|---|---|---|---|---|---|---|---|---|---|---|---|---|
| 6641 | S8_1823051 | 8 | 1823051 | 40 | G | C | G | 0.93548 | C | 0.06452 | 2 | 0.06452 |
| 6642 | S8_1823153 | 8 | 1823153 | 102 | A | G | A | 0.93548 | G | 0.06452 | 2 | 0.06452 |
| 6643 | S8_1903054 | 8 | 1903054 | 79901 | C | T | C | 0.87097 | T | 0.12903 | 6 | 0.19355 |
| 6644 | S8_1903139 | 8 | 1903139 | 85 | C | T | C | 0.91935 | T | 0.08065 | 3 | 0.09677 |
| 6645 | S8_2149718 | 8 | 2149718 | 246579 | T | C | C | 0.59677 | T | 0.40323 | 11 | 0.35484 |
| 6646 | S8_2563103 | 8 | 2563103 | 413385 | A | G | A | 0.95161 | G | 0.04839 | 3 | 0.09677 |
| 6647 | S8_2962417 | 8 | 2962417 | 399314 | G | A | G | 0.62903 | A | 0.37097 | 15 | 0.48387 |
| 6648 | S8_4155497 | 8 | 4155497 | 1193080 | A | G | A | 0.79032 | G | 0.20968 | 11 | 0.35484 |
| 6649 | S8_4155540 | 8 | 4155540 | 43 | C | A | C | 0.87097 | A | 0.12903 | 8 | 0.25806 |
| 6650 | S8_4155641 | 8 | 4155641 | 101 | T | G | T | 0.87097 | G | 0.12903 | 8 | 0.25806 |
| 6651 | S8_4155646 | 8 | 4155646 | 5 | A | G | A | 0.79032 | G | 0.20968 | 11 | 0.35484 |
| 6652 | S8_4386793 | 8 | 4386793 | 231147 | T | C | T | 0.66129 | C | 0.33871 | 13 | 0.41935 |
| 6653 | S8_4743279 | 8 | 4743279 | 356486 | G | A | A | 0.56452 | G | 0.43548 | 19 | 0.6129 |
| 6654 | S8_4759997 | 8 | 4759997 | 16718 | G | C | G | 0.75806 | C | 0.24194 | 15 | 0.48387 |
| 6655 | S8_4760107 | 8 | 4760107 | 110 | G | A | G | 0.79032 | A | 0.20968 | 11 | 0.35484 |
| 6656 | S8_4760140 | 8 | 4760140 | 33 | G | A | G | 0.90323 | A | 0.09677 | 6 | 0.19355 |
| 6657 | S8_4760168 | 8 | 4760168 | 28 | G | A | G | 0.80645 | A | 0.19355 | 12 | 0.3871 |
| 6658 | S8_4760237 | 8 | 4760237 | 69 | T | C | T | 0.75806 | C | 0.24194 | 15 | 0.48387 |
| 6659 | S8_4760246 | 8 | 4760246 | 9 | T | C | T | 0.75806 | C | 0.24194 | 15 | 0.48387 |
| 6660 | S8_4789558 | 8 | 4789558 | 29312 | A | T | T | 0.82258 | A | 0.17742 | 11 | 0.35484 |
| 6661 | S8_4789702 | 8 | 4789702 | 144 | A | G | A | 0.53226 | G | 0.46774 | 19 | 0.6129 |
| 6662 | S8_4886152 | 8 | 4886152 | 96450 | C | T | T | 0.72581 | C | 0.27419 | 13 | 0.41935 |
| 6663 | S8_4886231 | 8 | 4886231 | 79 | G | T | G | 0.83871 | T | 0.16129 | 10 | 0.32258 |
| 6664 | S8_4886332 | 8 | 4886332 | 101 | G | C | G | 0.8871 | C | 0.1129 | 7 | 0.22581 |
| 6665 | S8_4897736 | 8 | 4897736 | 11404 | A | G | A | 0.87097 | G | 0.12903 | 8 | 0.25806 |
| 6666 | S8_4897770 | 8 | 4897770 | 34 | T | C | T | 0.8871 | C | 0.1129 | 7 | 0.22581 |
| 6667 | S8_5050622 | 8 | 5050622 | 152852 | T | C | T | 0.64516 | C | 0.35484 | 16 | 0.51613 |
| 6668 | S8_5050647 | 8 | 5050647 | 25 | T | C | T | 0.90323 | C | 0.09677 | 6 | 0.19355 |
| 6669 | S8_5064895 | 8 | 5064895 | 14248 | C | T | C | 0.83871 | T | 0.16129 | 6 | 0.19355 |
| 6670 | S8_5064914 | 8 | 5064914 | 19 | G | A | G | 0.75806 | A | 0.24194 | 9 | 0.29032 |
| 6671 | S8_5064929 | 8 | 5064929 | 15 | T | C | T | 0.53226 | C | 0.46774 | 15 | 0.48387 |
| 6672 | S8_5064958 | 8 | 5064958 | 29 | G | C | G | 0.53226 | C | 0.46774 | 15 | 0.48387 |
| 6673 | S8_5065039 | 8 | 5065039 | 81 | C | T | C | 0.67742 | T | 0.32258 | 14 | 0.45161 |
| 6674 | S8_5119615 | 8 | 5119615 | 54576 | T | C | C | 0.72581 | T | 0.27419 | 15 | 0.48387 |
| 6675 | S8_5119618 | 8 | 5119618 | 3 | G | A | G | 0.91935 | A | 0.08065 | 5 | 0.16129 |
| 6676 | S8_5119636 | 8 | 5119636 | 18 | C | T | C | 0.8871 | T | 0.1129 | 7 | 0.22581 |
| 6677 | S8_5119765 | 8 | 5119765 | 129 | C | T | C | 0.91935 | T | 0.08065 | 5 | 0.16129 |
| 6678 | S8_5119775 | 8 | 5119775 | 10 | A | C | A | 0.91935 | C | 0.08065 | 5 | 0.16129 |
| 6679 | S8_5195397 | 8 | 5195397 | 75622 | A | G | A | 0.91935 | G | 0.08065 | 5 | 0.16129 |
| 6680 | S8_5195412 | 8 | 5195412 | 15 | G | A | G | 0.85484 | A | 0.14516 | 7 | 0.22581 |
| 6681 | S8_5195532 | 8 | 5195532 | 120 | T | C | T | 0.93548 | C | 0.06452 | 4 | 0.12903 |
| 6682 | S8_5195583 | 8 | 5195583 | 51 | C | G | G | 0.72581 | C | 0.27419 | 11 | 0.35484 |
| 6683 | S8_5253076 | 8 | 5253076 | 57493 | A | G | A | 0.74194 | G | 0.25806 | 12 | 0.3871 |
| 6684 | S8_5253145 | 8 | 5253145 | 69 | T | G | T | 0.70968 | G | 0.29032 | 10 | 0.32258 |
| 6685 | S8_5253270 | 8 | 5253270 | 125 | C | T | C | 0.93548 | T | 0.06452 | 4 | 0.12903 |
| 6686 | S8_5372650 | 8 | 5372650 | 119380 | T | A | T | 0.82258 | A | 0.17742 | 11 | 0.35484 |
| 6687 | S8_5438914 | 8 | 5438914 | 66264 | T | C | T | 0.87097 | C | 0.12903 | 8 | 0.25806 |
| 6688 | S8_5438992 | 8 | 5438992 | 78 | T | A | T | 0.93548 | A | 0.06452 | 4 | 0.12903 |
| 6689 | S8_5439086 | 8 | 5439086 | 94 | G | T | G | 0.80645 | T | 0.19355 | 12 | 0.3871 |
| 6690 | S8_5439100 | 8 | 5439100 | 14 | A | G | A | 0.64516 | G | 0.35484 | 20 | 0.64516 |
| 6691 | S8_5458975 | 8 | 5458975 | 19875 | C | T | C | 0.95161 | T | 0.04839 | 3 | 0.09677 |
| 6692 | S8_5459075 | 8 | 5459075 | 100 | T | G | T | 0.66129 | G | 0.33871 | 17 | 0.54839 |
| 6693 | S8_5459121 | 8 | 5459121 | 46 | A | G | G | 0.69355 | A | 0.30645 | 17 | 0.54839 |
| 6694 | S8_5583802 | 8 | 5583802 | 124681 | C | T | C | 0.79032 | T | 0.20968 | 13 | 0.41935 |

| Site number | SNP name | Chromosome | Physical position | Physical distance from the previous SNP | Reference allele | Alternative allele | Major allele | Major allele frequency | Minor allele | Minor allele frequency | Number heterozygous | Heterozygosity |
|---|---|---|---|---|---|---|---|---|---|---|---|---|
| 6695 | S8_5583859 | 8 | 5583859 | 57 | C | T | C | 0.56452 | T | 0.43548 | 21 | 0.67742 |
| 6696 | S8_5758410 | 8 | 5758410 | 174551 | A | G | A | 0.75806 | G | 0.24194 | 11 | 0.35484 |
| 6697 | S8_5790536 | 8 | 5790536 | 32126 | C | T | C | 0.58065 | T | 0.41935 | 16 | 0.51613 |
| 6698 | S8_5790720 | 8 | 5790720 | 184 | G | A | G | 0.95161 | A | 0.04839 | 3 | 0.09677 |
| 6699 | S8_6556677 | 8 | 6556677 | 765957 | C | T | C | 0.69355 | T | 0.30645 | 15 | 0.48387 |
| 6700 | S8_6556725 | 8 | 6556725 | 48 | G | A | G | 0.90323 | A | 0.09677 | 6 | 0.19355 |
| 6701 | S8_6556813 | 8 | 6556813 | 88 | C | T | C | 0.70968 | T | 0.29032 | 12 | 0.3871 |
| 6702 | S8_6556852 | 8 | 6556852 | 39 | G | T | G | 0.69355 | T | 0.30645 | 15 | 0.48387 |
| 6703 | S8_6556865 | 8 | 6556865 | 13 | T | A | T | 0.93548 | A | 0.06452 | 4 | 0.12903 |
| 6704 | S8_6556896 | 8 | 6556896 | 31 | C | T | C | 0.69355 | T | 0.30645 | 15 | 0.48387 |
| 6705 | S8_6813471 | 8 | 6813471 | 256575 | T | C | T | 0.87097 | C | 0.12903 | 8 | 0.25806 |
| 6706 | S8_6813549 | 8 | 6813549 | 78 | C | T | C | 0.91935 | T | 0.08065 | 5 | 0.16129 |
| 6707 | S8_6880878 | 8 | 6880878 | 67329 | G | A | G | 0.93548 | A | 0.06452 | 4 | 0.12903 |
| 6708 | S8_6881097 | 8 | 6881097 | 219 | C | A | C | 0.8871 | A | 0.1129 | 7 | 0.22581 |
| 6709 | S8_7068606 | 8 | 7068606 | 187509 | G | A | G | 0.69355 | A | 0.30645 | 17 | 0.54839 |
| 6710 | S8_7384392 | 8 | 7384392 | 315786 | T | A | T | 0.91935 | A | 0.08065 | 5 | 0.16129 |
| 6711 | S8_7384551 | 8 | 7384551 | 159 | G | A | G | 0.77419 | A | 0.22581 | 10 | 0.32258 |
| 6712 | S8_7384560 | 8 | 7384560 | 9 | G | A | G | 0.90323 | A | 0.09677 | 6 | 0.19355 |
| 6713 | S8_7431770 | 8 | 7431770 | 47210 | C | T | C | 0.93548 | T | 0.06452 | 4 | 0.12903 |
| 6714 | S8_7431780 | 8 | 7431780 | 10 | G | A | G | 0.87097 | A | 0.12903 | 8 | 0.25806 |
| 6715 | S8_7431809 | 8 | 7431809 | 29 | C | T | C | 0.93548 | T | 0.06452 | 4 | 0.12903 |
| 6716 | S8_7533737 | 8 | 7533737 | 101928 | G | A | G | 0.91935 | A | 0.08065 | 5 | 0.16129 |
| 6717 | S8_7533768 | 8 | 7533768 | 31 | G | A | G | 0.66129 | A | 0.33871 | 13 | 0.41935 |
| 6718 | S8_7533799 | 8 | 7533799 | 31 | A | G | A | 0.77419 | G | 0.22581 | 10 | 0.32258 |
| 6719 | S8_7641557 | 8 | 7641557 | 107758 | T | C | T | 0.56452 | C | 0.43548 | 13 | 0.41935 |
| 6720 | S8_7641690 | 8 | 7641690 | 133 | A | G | G | 0.85484 | A | 0.14516 | 7 | 0.22581 |
| 6721 | S8_7641728 | 8 | 7641728 | 38 | C | T | C | 0.56452 | T | 0.43548 | 13 | 0.41935 |
| 6722 | S8_7641736 | 8 | 7641736 | 8 | G | A | A | 0.85484 | G | 0.14516 | 7 | 0.22581 |
| 6723 | S8_7641740 | 8 | 7641740 | 4 | C | T | C | 0.95161 | T | 0.04839 | 3 | 0.09677 |
| 6724 | S8_7641776 | 8 | 7641776 | 36 | C | T | C | 0.91935 | T | 0.08065 | 5 | 0.16129 |
| 6725 | S8_7703528 | 8 | 7703528 | 61752 | A | G | G | 0.59677 | A | 0.40323 | 15 | 0.48387 |
| 6726 | S8_7966959 | 8 | 7966959 | 263431 | G | C | C | 0.91935 | G | 0.08065 | 5 | 0.16129 |
| 6727 | S8_7967036 | 8 | 7967036 | 77 | A | C | A | 0.66129 | C | 0.33871 | 11 | 0.35484 |
| 6728 | S8_8167034 | 8 | 8167034 | 199998 | G | A | G | 0.91935 | A | 0.08065 | 5 | 0.16129 |
| 6729 | S8_8285531 | 8 | 8285531 | 118497 | G | A | G | 0.90323 | A | 0.09677 | 6 | 0.19355 |
| 6730 | S8_8285552 | 8 | 8285552 | 21 | T | C | T | 0.75806 | C | 0.24194 | 11 | 0.35484 |
| 6731 | S8_8652489 | 8 | 8652489 | 366937 | T | C | T | 0.83871 | C | 0.16129 | 8 | 0.25806 |
| 6732 | S8_8652490 | 8 | 8652490 | 1 | G | A | G | 0.83871 | A | 0.16129 | 8 | 0.25806 |
| 6733 | S8_8652612 | 8 | 8652612 | 122 | C | T | C | 0.95161 | T | 0.04839 | 3 | 0.09677 |
| 6734 | S8_8652660 | 8 | 8652660 | 48 | G | A | G | 0.83871 | A | 0.16129 | 8 | 0.25806 |
| 6735 | S8_8717564 | 8 | 8717564 | 64904 | T | A | T | 0.91935 | A | 0.08065 | 3 | 0.09677 |
| 6736 | S8_9061580 | 8 | 9061580 | 344016 | T | C | T | 0.82258 | C | 0.17742 | 9 | 0.29032 |
| 6737 | S8_9061600 | 8 | 9061600 | 20 | A | G | G | 0.66129 | A | 0.33871 | 17 | 0.54839 |
| 6738 | S8_9061613 | 8 | 9061613 | 13 | A | G | G | 0.66129 | A | 0.33871 | 17 | 0.54839 |
| 6739 | S8_9061645 | 8 | 9061645 | 32 | G | A | G | 0.95161 | A | 0.04839 | 3 | 0.09677 |
| 6740 | S8_9061652 | 8 | 9061652 | 7 | G | A | G | 0.90323 | A | 0.09677 | 6 | 0.19355 |
| 6741 | S8_9061758 | 8 | 9061758 | 106 | C | T | C | 0.87097 | T | 0.12903 | 8 | 0.25806 |
| 6742 | S8_9061761 | 8 | 9061761 | 3 | C | T | C | 0.80645 | T | 0.19355 | 10 | 0.32258 |
| 6743 | S8_9061805 | 8 | 9061805 | 44 | A | G | A | 0.83871 | G | 0.16129 | 8 | 0.25806 |
| 6744 | S8_9061822 | 8 | 9061822 | 17 | G | A | G | 0.90323 | A | 0.09677 | 6 | 0.19355 |
| 6745 | S8_9062400 | 8 | 9062400 | 578 | T | C | C | 0.67742 | T | 0.32258 | 16 | 0.51613 |
| 6746 | S8_9062494 | 8 | 9062494 | 94 | C | T | T | 0.54839 | C | 0.45161 | 18 | 0.58065 |
| 6747 | S8_9911657 | 8 | 9911657 | 849163 | G | T | G | 0.82258 | T | 0.17742 | 9 | 0.29032 |
| 6748 | S8_9911787 | 8 | 9911787 | 130 | A | C | A | 0.82258 | C | 0.17742 | 9 | 0.29032 |

| Site number | SNP name | Chromosome | Physical position | Physical distance from the previous SNP | Reference allele | Alternative allele | Major allele | Major allele frequency | Minor allele | Minor allele frequency | Number heterozygous | Heterozygosity |
|---|---|---|---|---|---|---|---|---|---|---|---|---|
| 6749 | S8_9911824 | 8 | 9911824 | 37 | G | A | G | 0.82258 | A | 0.17742 | 9 | 0.29032 |
| 6750 | S8_10164511 | 8 | 10164511 | 252687 | G | A | G | 0.93548 | A | 0.06452 | 4 | 0.12903 |
| 6751 | S8_10219580 | 8 | 10219580 | 55069 | T | C | T | 0.90323 | C | 0.09677 | 6 | 0.19355 |
| 6752 | S8_10283744 | 8 | 10283744 | 64164 | G | C | C | 0.51613 | G | 0.48387 | 14 | 0.45161 |
| 6753 | S8_10283824 | 8 | 10283824 | 80 | A | G | G | 0.69355 | A | 0.30645 | 13 | 0.41935 |
| 6754 | S8_10283894 | 8 | 10283894 | 70 | A | G | G | 0.51613 | A | 0.48387 | 12 | 0.3871 |
| 6755 | S8_10295543 | 8 | 10295543 | 11649 | A | T | A | 0.82258 | T | 0.17742 | 9 | 0.29032 |
| 6756 | S8_10295643 | 8 | 10295643 | 100 | T | C | T | 0.8871 | C | 0.1129 | 7 | 0.22581 |
| 6757 | S8_10360985 | 8 | 10360985 | 65342 | G | A | A | 0.5 | G | 0.5 | 15 | 0.48387 |
| 6758 | S8_10361015 | 8 | 10361015 | 30 | C | T | C | 0.85484 | T | 0.14516 | 7 | 0.22581 |
| 6759 | S8_10361065 | 8 | 10361065 | 50 | C | G | C | 0.74194 | G | 0.25806 | 14 | 0.45161 |
| 6760 | S8_10361098 | 8 | 10361098 | 33 | T | C | T | 0.90323 | C | 0.09677 | 6 | 0.19355 |
| 6761 | S8_10361143 | 8 | 10361143 | 45 | A | G | A | 0.83871 | G | 0.16129 | 8 | 0.25806 |
| 6762 | S8_10494381 | 8 | 10494381 | 133238 | G | A | G | 0.79032 | A | 0.20968 | 11 | 0.35484 |
| 6763 | S8_10552733 | 8 | 10552733 | 58352 | C | T | C | 0.93548 | T | 0.06452 | 4 | 0.12903 |
| 6764 | S8_10552751 | 8 | 10552751 | 18 | C | T | C | 0.82258 | T | 0.17742 | 9 | 0.29032 |
| 6765 | S8_10552786 | 8 | 10552786 | 35 | A | G | A | 0.59677 | G | 0.40323 | 17 | 0.54839 |
| 6766 | S8_10572160 | 8 | 10572160 | 19374 | A | G | A | 0.54839 | G | 0.45161 | 22 | 0.70968 |
| 6767 | S8_10572180 | 8 | 10572180 | 20 | T | C | T | 0.85484 | C | 0.14516 | 7 | 0.22581 |
| 6768 | S8_10572183 | 8 | 10572183 | 3 | G | A | G | 0.93548 | A | 0.06452 | 4 | 0.12903 |
| 6769 | S8_10572293 | 8 | 10572293 | 110 | A | C | A | 0.56452 | C | 0.43548 | 21 | 0.67742 |
| 6770 | S8_10677679 | 8 | 10677679 | 105386 | C | A | A | 0.75806 | C | 0.24194 | 13 | 0.41935 |
| 6771 | S8_10677688 | 8 | 10677688 | 9 | G | A | G | 0.91935 | A | 0.08065 | 5 | 0.16129 |
| 6772 | S8_10721940 | 8 | 10721940 | 44252 | G | A | G | 0.64516 | A | 0.35484 | 18 | 0.58065 |
| 6773 | S8_10721975 | 8 | 10721975 | 35 | T | G | T | 0.95161 | G | 0.04839 | 3 | 0.09677 |
| 6774 | S8_10721988 | 8 | 10721988 | 13 | A | G | A | 0.79032 | G | 0.20968 | 11 | 0.35484 |
| 6775 | S8_10722082 | 8 | 10722082 | 94 | C | T | C | 0.95161 | T | 0.04839 | 3 | 0.09677 |
| 6776 | S8_10781772 | 8 | 10781772 | 59690 | C | T | C | 0.90323 | T | 0.09677 | 6 | 0.19355 |
| 6777 | S8_10781963 | 8 | 10781963 | 191 | A | G | A | 0.93548 | G | 0.06452 | 4 | 0.12903 |
| 6778 | S8_10781968 | 8 | 10781968 | 5 | A | G | A | 0.59677 | G | 0.40323 | 17 | 0.54839 |
| 6779 | S8_10781974 | 8 | 10781974 | 6 | G | A | G | 0.58065 | A | 0.41935 | 16 | 0.51613 |
| 6780 | S8_11259453 | 8 | 11259453 | 477479 | C | T | T | 0.83871 | C | 0.16129 | 8 | 0.25806 |
| 6781 | S8_11345080 | 8 | 11345080 | 85627 | T | G | T | 0.83871 | G | 0.16129 | 10 | 0.32258 |
| 6782 | S8_11345116 | 8 | 11345116 | 36 | T | C | C | 0.80645 | T | 0.19355 | 10 | 0.32258 |
| 6783 | S8_11345123 | 8 | 11345123 | 7 | G | A | G | 0.93548 | A | 0.06452 | 4 | 0.12903 |
| 6784 | S8_11345197 | 8 | 11345197 | 74 | G | A | A | 0.74194 | G | 0.25806 | 14 | 0.45161 |
| 6785 | S8_11345285 | 8 | 11345285 | 88 | C | G | C | 0.64516 | G | 0.35484 | 14 | 0.45161 |
| 6786 | S8_11815731 | 8 | 11815731 | 470446 | G | A | G | 0.74194 | A | 0.25806 | 12 | 0.3871 |
| 6787 | S8_11815757 | 8 | 11815757 | 26 | G | A | G | 0.93548 | A | 0.06452 | 4 | 0.12903 |
| 6788 | S8_12405719 | 8 | 12405719 | 589962 | C | T | C | 0.6129 | T | 0.3871 | 12 | 0.3871 |
| 6789 | S8_12405897 | 8 | 12405897 | 178 | C | A | C | 0.8871 | A | 0.1129 | 7 | 0.22581 |
| 6790 | S8_12405925 | 8 | 12405925 | 28 | G | A | G | 0.77419 | A | 0.22581 | 14 | 0.45161 |
| 6791 | S8_12742574 | 8 | 12742574 | 336649 | C | T | C | 0.93548 | T | 0.06452 | 4 | 0.12903 |
| 6792 | S8_12742794 | 8 | 12742794 | 220 | G | A | G | 0.75806 | A | 0.24194 | 9 | 0.29032 |
| 6793 | S8_13037309 | 8 | 13037309 | 294515 | C | T | C | 0.90323 | T | 0.09677 | 6 | 0.19355 |
| 6794 | S8_13037339 | 8 | 13037339 | 30 | C | A | C | 0.93548 | A | 0.06452 | 4 | 0.12903 |
| 6795 | S8_13037354 | 8 | 13037354 | 15 | C | A | C | 0.95161 | A | 0.04839 | 3 | 0.09677 |
| 6796 | S8_13037363 | 8 | 13037363 | 9 | T | C | T | 0.80645 | C | 0.19355 | 12 | 0.3871 |
| 6797 | S8_13037390 | 8 | 13037390 | 27 | A | G | G | 0.72581 | A | 0.27419 | 15 | 0.48387 |
| 6798 | S8_13037528 | 8 | 13037528 | 138 | C | T | C | 0.90323 | T | 0.09677 | 6 | 0.19355 |
| 6799 | S8_13037529 | 8 | 13037529 | 1 | G | A | G | 0.75806 | A | 0.24194 | 15 | 0.48387 |
| 6800 | S8_13196686 | 8 | 13196686 | 159157 | G | C | G | 0.91935 | C | 0.08065 | 5 | 0.16129 |
| 6801 | S8_13725398 | 8 | 13725398 | 528712 | G | A | G | 0.82258 | A | 0.17742 | 9 | 0.29032 |
| 6802 | S8_14325887 | 8 | 14325887 | 600489 | A | G | A | 0.8871 | G | 0.1129 | 7 | 0.22581 |

| Site number | SNP name | Chromosome | Physical position | Physical distance from the previous SNP | Reference allele | Alternative allele | Major allele | Major allele frequency | Minor allele | Minor allele frequency | Number heterozygous | Heterozygosity |
|---|---|---|---|---|---|---|---|---|---|---|---|---|
| 6803 | S8_15288360 | 8 | 15288360 | 962473 | C | G | C | 0.82258 | G | 0.17742 | 9 | 0.29032 |
| 6804 | S8_15288437 | 8 | 15288437 | 77 | G | A | G | 0.93548 | A | 0.06452 | 4 | 0.12903 |
| 6805 | S8_15298230 | 8 | 15298230 | 9793 | C | T | C | 0.87097 | T | 0.12903 | 8 | 0.25806 |
| 6806 | S8_15420815 | 8 | 15420815 | 122585 | C | T | C | 0.90323 | T | 0.09677 | 6 | 0.19355 |
| 6807 | S8_15873482 | 8 | 15873482 | 452667 | C | T | T | 0.83871 | C | 0.16129 | 8 | 0.25806 |
| 6808 | S8_16225324 | 8 | 16225324 | 351842 | G | A | G | 0.75806 | A | 0.24194 | 13 | 0.41935 |
| 6809 | S8_16225360 | 8 | 16225360 | 36 | A | G | A | 0.8871 | G | 0.1129 | 7 | 0.22581 |
| 6810 | S8_16372277 | 8 | 16372277 | 146917 | T | G | T | 0.95161 | G | 0.04839 | 3 | 0.09677 |
| 6811 | S8_16372294 | 8 | 16372294 | 17 | G | A | A | 0.59677 | G | 0.40323 | 15 | 0.48387 |
| 6812 | S8_16372339 | 8 | 16372339 | 45 | G | A | G | 0.95161 | A | 0.04839 | 3 | 0.09677 |
| 6813 | S8_16483051 | 8 | 16483051 | 110712 | G | A | G | 0.80645 | A | 0.19355 | 10 | 0.32258 |
| 6814 | S8_16483065 | 8 | 16483065 | 14 | A | G | A | 0.75806 | G | 0.24194 | 13 | 0.41935 |
| 6815 | S8_16734419 | 8 | 16734419 | 251354 | A | G | A | 0.87097 | G | 0.12903 | 8 | 0.25806 |
| 6816 | S8_16734470 | 8 | 16734470 | 51 | T | C | T | 0.85484 | C | 0.14516 | 9 | 0.29032 |
| 6817 | S8_16857565 | 8 | 16857565 | 123095 | G | A | G | 0.66129 | A | 0.33871 | 11 | 0.35484 |
| 6818 | S8_16857633 | 8 | 16857633 | 68 | C | T | C | 0.66129 | T | 0.33871 | 11 | 0.35484 |
| 6819 | S8_16857654 | 8 | 16857654 | 21 | G | A | G | 0.70968 | A | 0.29032 | 14 | 0.45161 |
| 6820 | S8_16857686 | 8 | 16857686 | 32 | G | A | G | 0.93548 | A | 0.06452 | 2 | 0.06452 |
| 6821 | S8_16857738 | 8 | 16857738 | 52 | A | G | A | 0.77419 | G | 0.22581 | 12 | 0.3871 |
| 6822 | S8_16857767 | 8 | 16857767 | 29 | T | A | T | 0.70968 | A | 0.29032 | 14 | 0.45161 |
| 6823 | S8_16857775 | 8 | 16857775 | 8 | C | T | C | 0.93548 | T | 0.06452 | 2 | 0.06452 |
| 6824 | S8_16884233 | 8 | 16884233 | 26458 | T | A | T | 0.93548 | A | 0.06452 | 4 | 0.12903 |
| 6825 | S8_16884280 | 8 | 16884280 | 47 | G | T | T | 0.93548 | G | 0.06452 | 4 | 0.12903 |
| 6826 | S8_16884352 | 8 | 16884352 | 72 | C | T | C | 0.95161 | T | 0.04839 | 3 | 0.09677 |
| 6827 | S8_16962939 | 8 | 16962939 | 78587 | A | G | G | 0.54839 | A | 0.45161 | 18 | 0.58065 |
| 6828 | S8_16963113 | 8 | 16963113 | 174 | G | A | G | 0.82258 | A | 0.17742 | 11 | 0.35484 |
| 6829 | S8_16963119 | 8 | 16963119 | 6 | G | C | G | 0.54839 | C | 0.45161 | 20 | 0.64516 |
| 6830 | S8_16963141 | 8 | 16963141 | 22 | G | A | G | 0.90323 | A | 0.09677 | 4 | 0.12903 |
| 6831 | S8_16991838 | 8 | 16991838 | 28697 | C | T | C | 0.54839 | T | 0.45161 | 12 | 0.3871 |
| 6832 | S8_16991896 | 8 | 16991896 | 58 | T | C | C | 0.77419 | T | 0.22581 | 12 | 0.3871 |
| 6833 | S8_16992022 | 8 | 16992022 | 126 | A | G | G | 0.54839 | A | 0.45161 | 12 | 0.3871 |
| 6834 | S8_17021216 | 8 | 17021216 | 29194 | G | A | G | 0.62903 | A | 0.37097 | 15 | 0.48387 |
| 6835 | S8_17108747 | 8 | 17108747 | 87531 | A | C | C | 0.69355 | A | 0.30645 | 15 | 0.48387 |
| 6836 | S8_17180602 | 8 | 17180602 | 71855 | A | G | G | 0.85484 | A | 0.14516 | 9 | 0.29032 |
| 6837 | S8_17180616 | 8 | 17180616 | 14 | G | C | C | 0.56452 | G | 0.43548 | 17 | 0.54839 |
| 6838 | S8_17462741 | 8 | 17462741 | 282125 | C | T | T | 0.62903 | C | 0.37097 | 17 | 0.54839 |
| 6839 | S8_17462806 | 8 | 17462806 | 65 | T | A | T | 0.64516 | A | 0.35484 | 16 | 0.51613 |
| 6840 | S8_17785384 | 8 | 17785384 | 322578 | C | A | C | 0.79032 | A | 0.20968 | 13 | 0.41935 |
| 6841 | S8_17785401 | 8 | 17785401 | 17 | G | T | G | 0.51613 | T | 0.48387 | 12 | 0.3871 |
| 6842 | S8_17798287 | 8 | 17798287 | 12886 | C | T | C | 0.8871 | T | 0.1129 | 5 | 0.16129 |
| 6843 | S8_17798294 | 8 | 17798294 | 7 | G | A | G | 0.79032 | A | 0.20968 | 9 | 0.29032 |
| 6844 | S8_17798301 | 8 | 17798301 | 7 | T | G | G | 0.8871 | T | 0.1129 | 5 | 0.16129 |
| 6845 | S8_17798349 | 8 | 17798349 | 48 | G | A | G | 0.8871 | A | 0.1129 | 5 | 0.16129 |
| 6846 | S8_17798397 | 8 | 17798397 | 48 | T | G | G | 0.8871 | T | 0.1129 | 5 | 0.16129 |
| 6847 | S8_18184317 | 8 | 18184317 | 385920 | C | T | T | 0.51613 | C | 0.48387 | 16 | 0.51613 |
| 6848 | S8_18184462 | 8 | 18184462 | 145 | G | A | A | 0.51613 | G | 0.48387 | 16 | 0.51613 |
| 6849 | S8_18184472 | 8 | 18184472 | 10 | T | C | C | 0.51613 | T | 0.48387 | 16 | 0.51613 |
| 6850 | S8_18184476 | 8 | 18184476 | 4 | T | C | C | 0.51613 | T | 0.48387 | 16 | 0.51613 |
| 6851 | S8_18232423 | 8 | 18232423 | 47947 | G | A | G | 0.95161 | A | 0.04839 | 3 | 0.09677 |
| 6852 | S8_18561831 | 8 | 18561831 | 329408 | C | T | C | 0.82258 | T | 0.17742 | 11 | 0.35484 |
| 6853 | S8_18561839 | 8 | 18561839 | 8 | C | T | C | 0.80645 | T | 0.19355 | 12 | 0.3871 |
| 6854 | S8_18562005 | 8 | 18562005 | 166 | T | C | C | 0.59677 | T | 0.40323 | 21 | 0.67742 |
| 6855 | S8_18604672 | 8 | 18604672 | 42667 | T | G | T | 0.91935 | G | 0.08065 | 3 | 0.09677 |
| 6856 | S8_18647006 | 8 | 18647006 | 42334 | C | A | C | 0.95161 | A | 0.04839 | 3 | 0.09677 |

| Site number | SNP name | Chromosome | Physical position | Physical distance from the previous SNP | Reference allele | Alternative allele | Major allele | Major allele frequency | Minor allele | Minor allele frequency | Number heterozygous | Heterozygosity |
|---|---|---|---|---|---|---|---|---|---|---|---|---|
| 6857 | S8_18647022 | 8 | 18647022 | 16 | G | A | G | 0.91935 | A | 0.08065 | 3 | 0.09677 |
| 6858 | S8_19814116 | 8 | 19814116 | 1167094 | A | G | G | 0.59677 | A | 0.40323 | 15 | 0.48387 |
| 6859 | S8_19832967 | 8 | 19832967 | 18851 | G | A | A | 0.59677 | G | 0.40323 | 17 | 0.54839 |
| 6860 | S8_21177076 | 8 | 21177076 | 1344109 | G | A | G | 0.79032 | A | 0.20968 | 11 | 0.35484 |
| 6861 | S8_21210980 | 8 | 21210980 | 33904 | T | A | T | 0.85484 | A | 0.14516 | 7 | 0.22581 |
| 6862 | S8_21350499 | 8 | 21350499 | 139519 | C | A | A | 0.56452 | C | 0.43548 | 15 | 0.48387 |
| 6863 | S8_21350602 | 8 | 21350602 | 103 | A | T | T | 0.56452 | A | 0.43548 | 15 | 0.48387 |
| 6864 | S8_21922973 | 8 | 21922973 | 572371 | G | T | G | 0.56452 | T | 0.43548 | 13 | 0.41935 |
| 6865 | S8_22299678 | 8 | 22299678 | 376705 | C | T | C | 0.72581 | T | 0.27419 | 13 | 0.41935 |
| 6866 | S8_22569787 | 8 | 22569787 | 270109 | C | T | C | 0.8871 | T | 0.1129 | 5 | 0.16129 |
| 6867 | S8_22714733 | 8 | 22714733 | 144946 | C | T | T | 0.66129 | C | 0.33871 | 15 | 0.48387 |
| 6868 | S8_22780420 | 8 | 22780420 | 65687 | T | G | T | 0.82258 | G | 0.17742 | 11 | 0.35484 |
| 6869 | S8_22780448 | 8 | 22780448 | 28 | G | C | G | 0.75806 | C | 0.24194 | 15 | 0.48387 |
| 6870 | S8_22780488 | 8 | 22780488 | 40 | T | C | T | 0.95161 | C | 0.04839 | 3 | 0.09677 |
| 6871 | S8_22780510 | 8 | 22780510 | 22 | T | C | T | 0.93548 | C | 0.06452 | 4 | 0.12903 |
| 6872 | S8_22780546 | 8 | 22780546 | 36 | T | C | T | 0.85484 | C | 0.14516 | 9 | 0.29032 |
| 6873 | S8_22780552 | 8 | 22780552 | 6 | A | G | A | 0.95161 | G | 0.04839 | 3 | 0.09677 |
| 6874 | S8_22780555 | 8 | 22780555 | 3 | T | C | T | 0.95161 | C | 0.04839 | 3 | 0.09677 |
| 6875 | S8_22780565 | 8 | 22780565 | 10 | C | G | C | 0.77419 | G | 0.22581 | 14 | 0.45161 |
| 6876 | S8_22780623 | 8 | 22780623 | 58 | C | G | C | 0.67742 | G | 0.32258 | 18 | 0.58065 |
| 6877 | S8_22781283 | 8 | 22781283 | 660 | T | C | T | 0.74194 | C | 0.25806 | 16 | 0.51613 |
| 6878 | S8_22781465 | 8 | 22781465 | 182 | T | C | T | 0.83871 | C | 0.16129 | 10 | 0.32258 |
| 6879 | S8_22781525 | 8 | 22781525 | 60 | T | C | T | 0.91935 | C | 0.08065 | 5 | 0.16129 |
| 6880 | S8_22786635 | 8 | 22786635 | 5110 | A | C | A | 0.95161 | C | 0.04839 | 3 | 0.09677 |
| 6881 | S8_22786650 | 8 | 22786650 | 15 | A | C | A | 0.80645 | C | 0.19355 | 12 | 0.3871 |
| 6882 | S8_22786658 | 8 | 22786658 | 8 | A | C | A | 0.80645 | C | 0.19355 | 12 | 0.3871 |
| 6883 | S8_22786676 | 8 | 22786676 | 18 | T | C | T | 0.80645 | C | 0.19355 | 12 | 0.3871 |
| 6884 | S8_22790058 | 8 | 22790058 | 3382 | G | C | G | 0.85484 | C | 0.14516 | 9 | 0.29032 |
| 6885 | S8_22790060 | 8 | 22790060 | 2 | G | T | G | 0.82258 | T | 0.17742 | 7 | 0.22581 |
| 6886 | S8_22790072 | 8 | 22790072 | 12 | G | A | G | 0.59677 | A | 0.40323 | 21 | 0.67742 |
| 6887 | S8_22790165 | 8 | 22790165 | 93 | G | C | G | 0.87097 | C | 0.12903 | 8 | 0.25806 |
| 6888 | S8_23090309 | 8 | 23090309 | 300144 | A | C | A | 0.87097 | C | 0.12903 | 8 | 0.25806 |
| 6889 | S8_23090394 | 8 | 23090394 | 85 | C | G | C | 0.87097 | G | 0.12903 | 8 | 0.25806 |
| 6890 | S8_23090489 | 8 | 23090489 | 95 | C | A | C | 0.77419 | A | 0.22581 | 14 | 0.45161 |
| 6891 | S8_23090492 | 8 | 23090492 | 3 | G | T | G | 0.77419 | T | 0.22581 | 14 | 0.45161 |
| 6892 | S8_23094214 | 8 | 23094214 | 3722 | C | A | C | 0.80645 | A | 0.19355 | 12 | 0.3871 |
| 6893 | S8_23094341 | 8 | 23094341 | 127 | C | T | C | 0.77419 | T | 0.22581 | 14 | 0.45161 |
| 6894 | S8_23094399 | 8 | 23094399 | 58 | T | C | T | 0.77419 | C | 0.22581 | 14 | 0.45161 |
| 6895 | S8_23123640 | 8 | 23123640 | 29241 | A | G | G | 0.83871 | A | 0.16129 | 10 | 0.32258 |
| 6896 | S8_23123697 | 8 | 23123697 | 57 | C | T | C | 0.95161 | T | 0.04839 | 3 | 0.09677 |
| 6897 | S8_23123853 | 8 | 23123853 | 156 | C | A | C | 0.77419 | A | 0.22581 | 14 | 0.45161 |
| 6898 | S8_23123855 | 8 | 23123855 | 2 | A | G | A | 0.77419 | G | 0.22581 | 14 | 0.45161 |
| 6899 | S8_23151255 | 8 | 23151255 | 27400 | T | A | T | 0.90323 | A | 0.09677 | 6 | 0.19355 |
| 6900 | S8_23151395 | 8 | 23151395 | 140 | G | A | G | 0.85484 | A | 0.14516 | 9 | 0.29032 |
| 6901 | S8_23151494 | 8 | 23151494 | 99 | C | T | C | 0.93548 | T | 0.06452 | 4 | 0.12903 |
| 6902 | S8_23279588 | 8 | 23279588 | 128094 | C | T | C | 0.8871 | T | 0.1129 | 5 | 0.16129 |
| 6903 | S8_23350078 | 8 | 23350078 | 70490 | C | T | T | 0.64516 | C | 0.35484 | 14 | 0.45161 |
| 6904 | S8_23350177 | 8 | 23350177 | 99 | C | T | T | 0.64516 | C | 0.35484 | 14 | 0.45161 |
| 6905 | S8_23350198 | 8 | 23350198 | 21 | G | A | G | 0.95161 | A | 0.04839 | 3 | 0.09677 |
| 6906 | S8_23350221 | 8 | 23350221 | 23 | C | T | C | 0.67742 | T | 0.32258 | 12 | 0.3871 |
| 6907 | S8_23350264 | 8 | 23350264 | 43 | C | G | G | 0.64516 | C | 0.35484 | 14 | 0.45161 |
| 6908 | S8_23499443 | 8 | 23499443 | 149179 | T | G | T | 0.6129 | G | 0.3871 | 14 | 0.45161 |
| 6909 | S8_23660893 | 8 | 23660893 | 161450 | T | A | T | 0.56452 | A | 0.43548 | 17 | 0.54839 |
| 6910 | S8_23763054 | 8 | 23763054 | 102161 | C | G | C | 0.93548 | G | 0.06452 | 4 | 0.12903 |

| Site number | SNP name | Chromosome | Physical position | Physical distance from the previous SNP | Reference allele | Alternative allele | Major allele | Major allele frequency | Minor allele | Minor allele frequency | Number heterozygous | Heterozygosity |
|---|---|---|---|---|---|---|---|---|---|---|---|---|
| 6911 | S8_23887964 | 8 | 23887964 | 124910 | G | C | G | 0.54839 | C | 0.45161 | 14 | 0.45161 |
| 6912 | S8_24222579 | 8 | 24222579 | 334615 | G | A | G | 0.91935 | A | 0.08065 | 5 | 0.16129 |
| 6913 | S8_24222613 | 8 | 24222613 | 34 | T | G | T | 0.95161 | G | 0.04839 | 3 | 0.09677 |
| 6914 | S8_24222762 | 8 | 24222762 | 149 | T | C | T | 0.91935 | C | 0.08065 | 5 | 0.16129 |
| 6915 | S8_24458586 | 8 | 24458586 | 235824 | T | C | T | 0.79032 | C | 0.20968 | 13 | 0.41935 |
| 6916 | S8_24494645 | 8 | 24494645 | 36059 | A | G | G | 0.75806 | A | 0.24194 | 11 | 0.35484 |
| 6917 | S8_24605029 | 8 | 24605029 | 110384 | G | A | G | 0.95161 | A | 0.04839 | 3 | 0.09677 |
| 6918 | S8_24605187 | 8 | 24605187 | 158 | C | T | C | 0.91935 | T | 0.08065 | 5 | 0.16129 |
| 6919 | S8_24837565 | 8 | 24837565 | 232378 | C | T | C | 0.83871 | T | 0.16129 | 10 | 0.32258 |
| 6920 | S8_24952866 | 8 | 24952866 | 115301 | T | C | T | 0.93548 | C | 0.06452 | 4 | 0.12903 |
| 6921 | S8_24952914 | 8 | 24952914 | 48 | C | G | C | 0.56452 | G | 0.43548 | 13 | 0.41935 |
| 6922 | S8_24953005 | 8 | 24953005 | 91 | C | A | C | 0.64516 | A | 0.35484 | 16 | 0.51613 |
| 6923 | S8_24953012 | 8 | 24953012 | 7 | T | G | T | 0.67742 | G | 0.32258 | 12 | 0.3871 |
| 6924 | S8_25188270 | 8 | 25188270 | 235258 | G | C | C | 0.59677 | G | 0.40323 | 17 | 0.54839 |
| 6925 | S8_25274229 | 8 | 25274229 | 85959 | C | T | C | 0.95161 | T | 0.04839 | 3 | 0.09677 |
| 6926 | S8_25598639 | 8 | 25598639 | 324410 | T | C | T | 0.91935 | C | 0.08065 | 5 | 0.16129 |
| 6927 | S8_25598759 | 8 | 25598759 | 120 | C | T | C | 0.91935 | T | 0.08065 | 5 | 0.16129 |
| 6928 | S8_26139089 | 8 | 26139089 | 540330 | G | C | G | 0.77419 | C | 0.22581 | 12 | 0.3871 |
| 6929 | S8_26262290 | 8 | 26262290 | 123201 | G | A | G | 0.75806 | A | 0.24194 | 13 | 0.41935 |
| 6930 | S8_26262351 | 8 | 26262351 | 61 | T | C | T | 0.83871 | C | 0.16129 | 8 | 0.25806 |
| 6931 | S8_26262376 | 8 | 26262376 | 25 | A | G | A | 0.69355 | G | 0.30645 | 15 | 0.48387 |
| 6932 | S8_26320695 | 8 | 26320695 | 58319 | A | G | G | 0.80645 | A | 0.19355 | 10 | 0.32258 |
| 6933 | S8_26619881 | 8 | 26619881 | 299186 | T | C | T | 0.83871 | C | 0.16129 | 8 | 0.25806 |
| 6934 | S8_26619972 | 8 | 26619972 | 91 | G | A | G | 0.87097 | A | 0.12903 | 8 | 0.25806 |
| 6935 | S8_26770082 | 8 | 26770082 | 150110 | C | T | C | 0.90323 | T | 0.09677 | 6 | 0.19355 |
| 6936 | S8_26848182 | 8 | 26848182 | 78100 | G | A | G | 0.66129 | A | 0.33871 | 17 | 0.54839 |
| 6937 | S8_26848342 | 8 | 26848342 | 160 | G | A | A | 0.54839 | G | 0.45161 | 16 | 0.51613 |
| 6938 | S8_27104959 | 8 | 27104959 | 256617 | C | T | C | 0.75806 | T | 0.24194 | 13 | 0.41935 |
| 6939 | S8_27216857 | 8 | 27216857 | 111898 | A | G | G | 0.51613 | A | 0.48387 | 16 | 0.51613 |
| 6940 | S8_27938761 | 8 | 27938761 | 721904 | C | T | T | 0.62903 | C | 0.37097 | 13 | 0.41935 |
| 6941 | S8_27938771 | 8 | 27938771 | 10 | T | C | T | 0.80645 | C | 0.19355 | 6 | 0.19355 |
| 6942 | S8_27938808 | 8 | 27938808 | 37 | C | T | C | 0.82258 | T | 0.17742 | 11 | 0.35484 |
| 6943 | S8_28632330 | 8 | 28632330 | 693522 | G | A | G | 0.8871 | A | 0.1129 | 7 | 0.22581 |
| 6944 | S8_28815891 | 8 | 28815891 | 183561 | C | T | C | 0.75806 | T | 0.24194 | 11 | 0.35484 |
| 6945 | S8_28954747 | 8 | 28954747 | 138856 | T | C | T | 0.90323 | C | 0.09677 | 6 | 0.19355 |
| 6946 | S8_28954759 | 8 | 28954759 | 12 | A | G | A | 0.90323 | G | 0.09677 | 6 | 0.19355 |
| 6947 | S8_28954822 | 8 | 28954822 | 63 | T | C | T | 0.74194 | C | 0.25806 | 8 | 0.25806 |
| 6948 | S8_29228480 | 8 | 29228480 | 273658 | G | T | T | 0.90323 | G | 0.09677 | 6 | 0.19355 |
| 6949 | S8_29228578 | 8 | 29228578 | 98 | G | T | G | 0.95161 | T | 0.04839 | 3 | 0.09677 |
| 6950 | S8_30179670 | 8 | 30179670 | 951092 | C | T | C | 0.93548 | T | 0.06452 | 4 | 0.12903 |
| 6951 | S8_30629856 | 8 | 30629856 | 450186 | G | A | G | 0.93548 | A | 0.06452 | 4 | 0.12903 |
| 6952 | S8_31485618 | 8 | 31485618 | 855762 | C | T | C | 0.95161 | T | 0.04839 | 3 | 0.09677 |
| 6953 | S8_31485759 | 8 | 31485759 | 141 | C | T | C | 0.95161 | T | 0.04839 | 3 | 0.09677 |
| 6954 | S8_31485769 | 8 | 31485769 | 10 | C | T | C | 0.95161 | T | 0.04839 | 3 | 0.09677 |
| 6955 | S8_31485783 | 8 | 31485783 | 14 | T | A | T | 0.90323 | A | 0.09677 | 6 | 0.19355 |
| 6956 | S8_32486050 | 8 | 32486050 | 1000267 | C | T | T | 0.87097 | C | 0.12903 | 6 | 0.19355 |
| 6957 | S8_32486284 | 8 | 32486284 | 234 | C | T | C | 0.93548 | T | 0.06452 | 4 | 0.12903 |
| 6958 | S8_32660587 | 8 | 32660587 | 174303 | G | T | G | 0.95161 | T | 0.04839 | 1 | 0.03226 |
| 6959 | S8_32660706 | 8 | 32660706 | 119 | T | C | T | 0.93548 | C | 0.06452 | 4 | 0.12903 |
| 6960 | S8_33043600 | 8 | 33043600 | 382894 | T | C | T | 0.91935 | C | 0.08065 | 5 | 0.16129 |
| 6961 | S8_33043602 | 8 | 33043602 | 2 | G | A | G | 0.69355 | A | 0.30645 | 17 | 0.54839 |
| 6962 | S8_33043609 | 8 | 33043609 | 7 | C | T | C | 0.91935 | T | 0.08065 | 5 | 0.16129 |
| 6963 | S8_33737546 | 8 | 33737546 | 693937 | T | C | T | 0.51613 | C | 0.48387 | 14 | 0.45161 |
| 6964 | S8_34029967 | 8 | 34029967 | 292421 | A | T | A | 0.95161 | T | 0.04839 | 3 | 0.09677 |

| Site number | SNP name | Chromosome | Physical position | Physical distance from the previous SNP | Reference allele | Alternative allele | Major allele | Major allele frequency | Minor allele | Minor allele frequency | Number heterozygous | Heterozygosity |
|---|---|---|---|---|---|---|---|---|---|---|---|---|
| 6965 | S8_35081616 | 8 | 35081616 | 1051649 | T | C | C | 0.83871 | T | 0.16129 | 10 | 0.32258 |
| 6966 | S8_35654388 | 8 | 35654388 | 572772 | A | G | A | 0.58065 | G | 0.41935 | 14 | 0.45161 |
| 6967 | S8_35654396 | 8 | 35654396 | 8 | C | T | C | 0.56452 | T | 0.43548 | 13 | 0.41935 |
| 6968 | S8_36034278 | 8 | 36034278 | 379882 | T | C | C | 0.82258 | T | 0.17742 | 9 | 0.29032 |
| 6969 | S8_36087228 | 8 | 36087228 | 52950 | T | G | T | 0.72581 | G | 0.27419 | 11 | 0.35484 |
| 6970 | S8_37176384 | 8 | 37176384 | 1089156 | G | A | G | 0.66129 | A | 0.33871 | 11 | 0.35484 |
| 6971 | S8_37266083 | 8 | 37266083 | 89699 | G | A | G | 0.83871 | A | 0.16129 | 10 | 0.32258 |
| 6972 | S8_37266219 | 8 | 37266219 | 136 | C | T | C | 0.91935 | T | 0.08065 | 3 | 0.09677 |
| 6973 | S8_37266320 | 8 | 37266320 | 101 | A | G | G | 0.91935 | A | 0.08065 | 3 | 0.09677 |
| 6974 | S8_37493158 | 8 | 37493158 | 226838 | G | A | G | 0.69355 | A | 0.30645 | 15 | 0.48387 |
| 6975 | S8_37493277 | 8 | 37493277 | 119 | G | C | G | 0.72581 | C | 0.27419 | 15 | 0.48387 |
| 6976 | S8_37593632 | 8 | 37593632 | 100355 | T | C | T | 0.90323 | C | 0.09677 | 6 | 0.19355 |
| 6977 | S8_37593687 | 8 | 37593687 | 55 | G | A | A | 0.70968 | G | 0.29032 | 14 | 0.45161 |
| 6978 | S8_37593743 | 8 | 37593743 | 56 | A | G | G | 0.87097 | A | 0.12903 | 4 | 0.12903 |
| 6979 | S8_38073041 | 8 | 38073041 | 479298 | G | A | G | 0.93548 | A | 0.06452 | 4 | 0.12903 |
| 6980 | S8_38073071 | 8 | 38073071 | 30 | G | A | G | 0.69355 | A | 0.30645 | 17 | 0.54839 |
| 6981 | S8_38073092 | 8 | 38073092 | 21 | C | T | C | 0.67742 | T | 0.32258 | 16 | 0.51613 |
| 6982 | S8_38073211 | 8 | 38073211 | 119 | T | G | G | 0.62903 | T | 0.37097 | 15 | 0.48387 |
| 6983 | S8_38073237 | 8 | 38073237 | 26 | A | G | G | 0.62903 | A | 0.37097 | 15 | 0.48387 |
| 6984 | S8_38073244 | 8 | 38073244 | 7 | T | C | T | 0.69355 | C | 0.30645 | 17 | 0.54839 |
| 6985 | S8_38174413 | 8 | 38174413 | 101169 | G | A | G | 0.69355 | A | 0.30645 | 13 | 0.41935 |
| 6986 | S8_38174430 | 8 | 38174430 | 17 | C | T | C | 0.69355 | T | 0.30645 | 13 | 0.41935 |
| 6987 | S8_38291744 | 8 | 38291744 | 117314 | T | A | T | 0.91935 | A | 0.08065 | 5 | 0.16129 |
| 6988 | S8_38291802 | 8 | 38291802 | 58 | G | A | G | 0.91935 | A | 0.08065 | 5 | 0.16129 |
| 6989 | S8_38698373 | 8 | 38698373 | 406571 | G | A | G | 0.77419 | A | 0.22581 | 12 | 0.3871 |
| 6990 | S8_39514433 | 8 | 39514433 | 816060 | T | C | T | 0.85484 | C | 0.14516 | 7 | 0.22581 |
| 6991 | S8_39527850 | 8 | 39527850 | 13417 | G | A | G | 0.67742 | A | 0.32258 | 16 | 0.51613 |
| 6992 | S8_39930544 | 8 | 39930544 | 402694 | A | G | A | 0.82258 | G | 0.17742 | 11 | 0.35484 |
| 6993 | S8_40517932 | 8 | 40517932 | 587388 | T | C | C | 0.8871 | T | 0.1129 | 5 | 0.16129 |
| 6994 | S8_40518019 | 8 | 40518019 | 87 | A | G | G | 0.8871 | A | 0.1129 | 5 | 0.16129 |
| 6995 | S8_40925602 | 8 | 40925602 | 407583 | G | T | G | 0.8871 | T | 0.1129 | 7 | 0.22581 |
| 6996 | S8_41142382 | 8 | 41142382 | 216780 | A | G | G | 0.70968 | A | 0.29032 | 12 | 0.3871 |
| 6997 | S8_41172763 | 8 | 41172763 | 30381 | C | G | C | 0.91935 | G | 0.08065 | 5 | 0.16129 |
| 6998 | S8_41319848 | 8 | 41319848 | 147085 | T | C | T | 0.70968 | C | 0.29032 | 12 | 0.3871 |
| 6999 | S8_41369710 | 8 | 41369710 | 49862 | T | C | C | 0.83871 | T | 0.16129 | 8 | 0.25806 |
| 7000 | S8_41369729 | 8 | 41369729 | 19 | A | G | G | 0.62903 | A | 0.37097 | 15 | 0.48387 |
| 7001 | S8_41456706 | 8 | 41456706 | 86977 | G | A | G | 0.93548 | A | 0.06452 | 4 | 0.12903 |
| 7002 | S8_41790205 | 8 | 41790205 | 333499 | G | A | G | 0.72581 | A | 0.27419 | 13 | 0.41935 |
| 7003 | S8_41874196 | 8 | 41874196 | 83991 | C | G | G | 0.51613 | C | 0.48387 | 18 | 0.58065 |
| 7004 | S8_42268055 | 8 | 42268055 | 393859 | C | G | G | 0.83871 | C | 0.16129 | 8 | 0.25806 |
| 7005 | S8_42268074 | 8 | 42268074 | 19 | A | G | G | 0.83871 | A | 0.16129 | 8 | 0.25806 |
| 7006 | S8_42268133 | 8 | 42268133 | 59 | C | T | T | 0.67742 | C | 0.32258 | 14 | 0.45161 |
| 7007 | S8_42883110 | 8 | 42883110 | 614977 | C | G | C | 0.5 | G | 0.5 | 17 | 0.54839 |
| 7008 | S8_42883206 | 8 | 42883206 | 96 | G | C | G | 0.93548 | C | 0.06452 | 4 | 0.12903 |
| 7009 | S8_42883247 | 8 | 42883247 | 41 | G | C | G | 0.95161 | C | 0.04839 | 3 | 0.09677 |
| 7010 | S8_43350398 | 8 | 43350398 | 467151 | C | T | T | 0.8871 | C | 0.1129 | 5 | 0.16129 |
| 7011 | S8_43354446 | 8 | 43354446 | 4048 | C | G | C | 0.69355 | G | 0.30645 | 11 | 0.35484 |
| 7012 | S8_43354448 | 8 | 43354448 | 2 | A | G | G | 0.53226 | A | 0.46774 | 13 | 0.41935 |
| 7013 | S8_43629427 | 8 | 43629427 | 274979 | C | T | C | 0.95161 | T | 0.04839 | 3 | 0.09677 |
| 7014 | S8_43984114 | 8 | 43984114 | 354687 | C | T | C | 0.91935 | T | 0.08065 | 5 | 0.16129 |
| 7015 | S8_44392664 | 8 | 44392664 | 408550 | A | G | A | 0.83871 | G | 0.16129 | 8 | 0.25806 |
| 7016 | S8_44766777 | 8 | 44766777 | 374113 | T | G | G | 0.70968 | T | 0.29032 | 16 | 0.51613 |
| 7017 | S8_44766796 | 8 | 44766796 | 19 | G | T | T | 0.70968 | G | 0.29032 | 16 | 0.51613 |
| 7018 | S8_44766891 | 8 | 44766891 | 95 | T | C | C | 0.69355 | T | 0.30645 | 15 | 0.48387 |

| Site number | SNP name | Chromosome | Physical position | Physical distance from the previous SNP | Reference allele | Alternative allele | Major allele | Major allele frequency | Minor allele | Minor allele frequency | Number heterozygous | Heterozygosity |
|---|---|---|---|---|---|---|---|---|---|---|---|---|
| 7019 | S8_44875727 | 8 | 44875727 | 108836 | A | C | C | 0.70968 | A | 0.29032 | 14 | 0.45161 |
| 7020 | S8_45159085 | 8 | 45159085 | 283358 | C | T | T | 0.67742 | C | 0.32258 | 14 | 0.45161 |
| 7021 | S8_45159104 | 8 | 45159104 | 19 | T | G | T | 0.6129 | G | 0.3871 | 14 | 0.45161 |
| 7022 | S8_45159142 | 8 | 45159142 | 38 | C | T | C | 0.70968 | T | 0.29032 | 14 | 0.45161 |
| 7023 | S8_45159156 | 8 | 45159156 | 14 | A | G | A | 0.6129 | G | 0.3871 | 14 | 0.45161 |
| 7024 | S8_46429073 | 8 | 46429073 | 1269917 | G | A | G | 0.95161 | A | 0.04839 | 3 | 0.09677 |
| 7025 | S8_46677209 | 8 | 46677209 | 248136 | A | G | A | 0.6129 | G | 0.3871 | 18 | 0.58065 |
| 7026 | S8_46677234 | 8 | 46677234 | 25 | G | A | G | 0.93548 | A | 0.06452 | 4 | 0.12903 |
| 7027 | S8_46783990 | 8 | 46783990 | 106756 | A | G | A | 0.58065 | G | 0.41935 | 20 | 0.64516 |
| 7028 | S8_46784018 | 8 | 46784018 | 28 | A | G | A | 0.58065 | G | 0.41935 | 20 | 0.64516 |
| 7029 | S8_46784119 | 8 | 46784119 | 101 | A | T | A | 0.58065 | T | 0.41935 | 20 | 0.64516 |
| 7030 | S8_46784121 | 8 | 46784121 | 2 | C | T | C | 0.58065 | T | 0.41935 | 20 | 0.64516 |
| 7031 | S8_46784146 | 8 | 46784146 | 25 | G | A | G | 0.58065 | A | 0.41935 | 20 | 0.64516 |
| 7032 | S8_46784174 | 8 | 46784174 | 28 | A | T | A | 0.54839 | T | 0.45161 | 20 | 0.64516 |
| 7033 | S8_46784175 | 8 | 46784175 | 1 | T | C | T | 0.54839 | C | 0.45161 | 20 | 0.64516 |
| 7034 | S8_47321603 | 8 | 47321603 | 537428 | A | T | T | 0.66129 | A | 0.33871 | 13 | 0.41935 |
| 7035 | S8_48432418 | 8 | 48432418 | 1110815 | C | G | C | 0.64516 | G | 0.35484 | 16 | 0.51613 |
| 7036 | S8_48432441 | 8 | 48432441 | 23 | A | G | G | 0.91935 | A | 0.08065 | 5 | 0.16129 |
| 7037 | S8_48432495 | 8 | 48432495 | 54 | C | T | T | 0.91935 | C | 0.08065 | 5 | 0.16129 |
| 7038 | S8_48432515 | 8 | 48432515 | 20 | C | T | T | 0.8871 | C | 0.1129 | 7 | 0.22581 |
| 7039 | S8_48971365 | 8 | 48971365 | 538850 | A | C | A | 0.53226 | C | 0.46774 | 17 | 0.54839 |
| 7040 | S8_48971415 | 8 | 48971415 | 50 | T | C | T | 0.90323 | C | 0.09677 | 6 | 0.19355 |
| 7041 | S8_49120937 | 8 | 49120937 | 149522 | G | T | T | 0.6129 | G | 0.3871 | 14 | 0.45161 |
| 7042 | S8_49120979 | 8 | 49120979 | 42 | G | A | G | 0.79032 | A | 0.20968 | 9 | 0.29032 |
| 7043 | S8_49129156 | 8 | 49129156 | 8177 | A | C | A | 0.79032 | C | 0.20968 | 9 | 0.29032 |
| 7044 | S8_49445911 | 8 | 49445911 | 316755 | G | A | G | 0.91935 | A | 0.08065 | 5 | 0.16129 |
| 7045 | S8_50089076 | 8 | 50089076 | 643165 | A | G | G | 0.95161 | A | 0.04839 | 3 | 0.09677 |
| 7046 | S8_50485473 | 8 | 50485473 | 396397 | G | A | G | 0.8871 | A | 0.1129 | 7 | 0.22581 |
| 7047 | S8_50553196 | 8 | 50553196 | 67723 | G | A | A | 0.51613 | G | 0.48387 | 20 | 0.64516 |
| 7048 | S8_50553211 | 8 | 50553211 | 15 | A | G | G | 0.51613 | A | 0.48387 | 20 | 0.64516 |
| 7049 | S8_50553229 | 8 | 50553229 | 18 | G | A | G | 0.8871 | A | 0.1129 | 7 | 0.22581 |
| 7050 | S8_50553236 | 8 | 50553236 | 7 | G | T | T | 0.83871 | G | 0.16129 | 6 | 0.19355 |
| 7051 | S8_50553308 | 8 | 50553308 | 72 | G | A | G | 0.58065 | A | 0.41935 | 18 | 0.58065 |
| 7052 | S8_50553333 | 8 | 50553333 | 25 | T | C | C | 0.5 | T | 0.5 | 17 | 0.54839 |
| 7053 | S8_51404150 | 8 | 51404150 | 850817 | G | A | A | 0.69355 | G | 0.30645 | 11 | 0.35484 |
| 7054 | S8_51915608 | 8 | 51915608 | 511458 | G | C | G | 0.66129 | C | 0.33871 | 15 | 0.48387 |
| 7055 | S8_51915623 | 8 | 51915623 | 15 | A | G | A | 0.53226 | G | 0.46774 | 13 | 0.41935 |
| 7056 | S8_51915720 | 8 | 51915720 | 97 | A | G | A | 0.58065 | G | 0.41935 | 12 | 0.3871 |
| 7057 | S8_51915758 | 8 | 51915758 | 38 | C | T | C | 0.91935 | T | 0.08065 | 3 | 0.09677 |
| 7058 | S8_51915763 | 8 | 51915763 | 5 | T | G | T | 0.91935 | G | 0.08065 | 3 | 0.09677 |
| 7059 | S8_52475160 | 8 | 52475160 | 559397 | G | A | G | 0.70968 | A | 0.29032 | 8 | 0.25806 |
| 7060 | S8_52475253 | 8 | 52475253 | 93 | C | T | C | 0.8871 | T | 0.1129 | 7 | 0.22581 |
| 7061 | S8_52475283 | 8 | 52475283 | 30 | G | C | G | 0.85484 | C | 0.14516 | 9 | 0.29032 |
| 7062 | S8_52475295 | 8 | 52475295 | 12 | A | G | A | 0.80645 | G | 0.19355 | 10 | 0.32258 |
| 7063 | S8_52475332 | 8 | 52475332 | 37 | G | T | G | 0.85484 | T | 0.14516 | 9 | 0.29032 |
| 7064 | S8_53473874 | 8 | 53473874 | 998542 | C | T | C | 0.80645 | T | 0.19355 | 6 | 0.19355 |
| 7065 | S8_53474102 | 8 | 53474102 | 228 | A | T | A | 0.80645 | T | 0.19355 | 6 | 0.19355 |
| 7066 | S8_53474118 | 8 | 53474118 | 16 | T | C | T | 0.80645 | C | 0.19355 | 6 | 0.19355 |
| 7067 | S8_54035697 | 8 | 54035697 | 561579 | A | T | A | 0.72581 | T | 0.27419 | 13 | 0.41935 |
| 7068 | S8_54433387 | 8 | 54433387 | 397690 | A | C | A | 0.93548 | C | 0.06452 | 4 | 0.12903 |
| 7069 | S8_54433474 | 8 | 54433474 | 87 | A | G | A | 0.93548 | G | 0.06452 | 4 | 0.12903 |
| 7070 | S8_54550519 | 8 | 54550519 | 117045 | C | T | C | 0.91935 | T | 0.08065 | 5 | 0.16129 |
| 7071 | S8_54632373 | 8 | 54632373 | 81854 | C | T | T | 0.62903 | C | 0.37097 | 19 | 0.6129 |
| 7072 | S8_54632573 | 8 | 54632573 | 200 | T | C | C | 0.62903 | T | 0.37097 | 19 | 0.6129 |

| Site number | SNP name | Chromosome | Physical position | Physical distance from the previous SNP | Reference allele | Alternative allele | Major allele | Major allele frequency | Minor allele | Minor allele frequency | Number heterozygous | Heterozygosity |
|---|---|---|---|---|---|---|---|---|---|---|---|---|
| 7073 | S8_55494933 | 8 | 55494933 | 862360 | A | G | A | 0.91935 | G | 0.08065 | 5 | 0.16129 |
| 7074 | S8_55495085 | 8 | 55495085 | 152 | G | A | G | 0.8871 | A | 0.1129 | 7 | 0.22581 |
| 7075 | S8_55787205 | 8 | 55787205 | 292120 | G | A | G | 0.90323 | A | 0.09677 | 6 | 0.19355 |
| 7076 | S8_56048985 | 8 | 56048985 | 261780 | C | T | C | 0.90323 | T | 0.09677 | 6 | 0.19355 |
| 7077 | S8_56049101 | 8 | 56049101 | 116 | C | T | C | 0.8871 | T | 0.1129 | 5 | 0.16129 |
| 7078 | S8_56445633 | 8 | 56445633 | 396532 | T | C | T | 0.62903 | C | 0.37097 | 11 | 0.35484 |
| 7079 | S8_56445687 | 8 | 56445687 | 54 | C | T | T | 0.93548 | C | 0.06452 | 2 | 0.06452 |
| 7080 | S8_56445797 | 8 | 56445797 | 110 | T | G | T | 0.83871 | G | 0.16129 | 10 | 0.32258 |
| 7081 | S8_56467455 | 8 | 56467455 | 21658 | T | G | T | 0.51613 | G | 0.48387 | 16 | 0.51613 |
| 7082 | S8_56467539 | 8 | 56467539 | 84 | T | A | T | 0.69355 | A | 0.30645 | 17 | 0.54839 |
| 7083 | S8_56467575 | 8 | 56467575 | 36 | T | G | G | 0.93548 | T | 0.06452 | 4 | 0.12903 |
| 7084 | S8_56671098 | 8 | 56671098 | 203523 | G | C | G | 0.77419 | C | 0.22581 | 12 | 0.3871 |
| 7085 | S8_56671275 | 8 | 56671275 | 177 | G | C | G | 0.74194 | C | 0.25806 | 16 | 0.51613 |
| 7086 | S8_56671277 | 8 | 56671277 | 2 | T | C | C | 0.5 | T | 0.5 | 19 | 0.6129 |
| 7087 | S8_57134603 | 8 | 57134603 | 463326 | T | C | T | 0.95161 | C | 0.04839 | 3 | 0.09677 |
| 7088 | S8_57134613 | 8 | 57134613 | 10 | C | A | C | 0.69355 | A | 0.30645 | 17 | 0.54839 |
| 7089 | S8_57544617 | 8 | 57544617 | 410004 | C | G | C | 0.90323 | G | 0.09677 | 6 | 0.19355 |
| 7090 | S8_57544630 | 8 | 57544630 | 13 | C | T | C | 0.82258 | T | 0.17742 | 11 | 0.35484 |
| 7091 | S8_58955970 | 8 | 58955970 | 1411340 | G | A | G | 0.90323 | A | 0.09677 | 6 | 0.19355 |
| 7092 | S8_59363989 | 8 | 59363989 | 408019 | T | C | T | 0.64516 | C | 0.35484 | 16 | 0.51613 |
| 7093 | S8_59777667 | 8 | 59777667 | 413678 | G | A | G | 0.74194 | A | 0.25806 | 10 | 0.32258 |
| 7094 | S8_59782262 | 8 | 59782262 | 4595 | C | A | C | 0.91935 | A | 0.08065 | 5 | 0.16129 |
| 7095 | S8_60435541 | 8 | 60435541 | 653279 | T | C | T | 0.82258 | C | 0.17742 | 9 | 0.29032 |
| 7096 | S8_60435677 | 8 | 60435677 | 136 | T | C | T | 0.82258 | C | 0.17742 | 9 | 0.29032 |
| 7097 | S8_60961704 | 8 | 60961704 | 526027 | T | C | T | 0.8871 | C | 0.1129 | 7 | 0.22581 |
| 7098 | S8_61366309 | 8 | 61366309 | 404605 | G | C | G | 0.77419 | C | 0.22581 | 10 | 0.32258 |
| 7099 | S8_61366329 | 8 | 61366329 | 20 | G | T | T | 0.51613 | G | 0.48387 | 16 | 0.51613 |
| 7100 | S8_61670829 | 8 | 61670829 | 304500 | C | T | C | 0.87097 | T | 0.12903 | 4 | 0.12903 |
| 7101 | S8_61676108 | 8 | 61676108 | 5279 | G | A | G | 0.93548 | A | 0.06452 | 4 | 0.12903 |
| 7102 | S8_61756070 | 8 | 61756070 | 79962 | G | T | G | 0.91935 | T | 0.08065 | 5 | 0.16129 |
| 7103 | S8_62325935 | 8 | 62325935 | 569865 | G | A | G | 0.93548 | A | 0.06452 | 4 | 0.12903 |
| 7104 | S8_62326082 | 8 | 62326082 | 147 | A | G | A | 0.82258 | G | 0.17742 | 9 | 0.29032 |
| 7105 | S8_62409330 | 8 | 62409330 | 83248 | T | C | T | 0.91935 | C | 0.08065 | 5 | 0.16129 |
| 7106 | S8_62422719 | 8 | 62422719 | 13389 | A | C | A | 0.67742 | C | 0.32258 | 18 | 0.58065 |
| 7107 | S8_62787934 | 8 | 62787934 | 365215 | C | T | C | 0.91935 | T | 0.08065 | 5 | 0.16129 |
| 7108 | S8_62866802 | 8 | 62866802 | 78868 | C | T | C | 0.79032 | T | 0.20968 | 13 | 0.41935 |
| 7109 | S8_63254810 | 8 | 63254810 | 388008 | C | T | T | 0.74194 | C | 0.25806 | 8 | 0.25806 |
| 7110 | S8_63625286 | 8 | 63625286 | 370476 | G | A | G | 0.90323 | A | 0.09677 | 6 | 0.19355 |
| 7111 | S8_63625370 | 8 | 63625370 | 84 | A | G | G | 0.79032 | A | 0.20968 | 7 | 0.22581 |
| 7112 | S8_64054788 | 8 | 64054788 | 429418 | C | G | C | 0.54839 | G | 0.45161 | 20 | 0.64516 |
| 7113 | S8_64054789 | 8 | 64054789 | 1 | G | A | G | 0.95161 | A | 0.04839 | 3 | 0.09677 |
| 7114 | S8_64168941 | 8 | 64168941 | 114152 | G | A | G | 0.59677 | A | 0.40323 | 15 | 0.48387 |
| 7115 | S8_64401569 | 8 | 64401569 | 232628 | A | T | T | 0.79032 | A | 0.20968 | 13 | 0.41935 |
| 7116 | S8_64796070 | 8 | 64796070 | 394501 | G | A | G | 0.83871 | A | 0.16129 | 10 | 0.32258 |
| 7117 | S8_64796201 | 8 | 64796201 | 131 | A | T | A | 0.93548 | T | 0.06452 | 4 | 0.12903 |
| 7118 | S8_64796240 | 8 | 64796240 | 39 | G | C | C | 0.56452 | G | 0.43548 | 17 | 0.54839 |
| 7119 | S8_64823208 | 8 | 64823208 | 26968 | C | T | C | 0.74194 | T | 0.25806 | 12 | 0.3871 |
| 7120 | S8_64859097 | 8 | 64859097 | 35889 | T | A | T | 0.56452 | A | 0.43548 | 17 | 0.54839 |
| 7121 | S8_64859198 | 8 | 64859198 | 101 | G | A | G | 0.54839 | A | 0.45161 | 18 | 0.58065 |
| 7122 | S8_65012015 | 8 | 65012015 | 152817 | T | C | C | 0.54839 | T | 0.45161 | 16 | 0.51613 |
| 7123 | S8_65012203 | 8 | 65012203 | 188 | A | G | G | 0.93548 | A | 0.06452 | 2 | 0.06452 |
| 7124 | S8_65081410 | 8 | 65081410 | 69207 | C | T | C | 0.87097 | T | 0.12903 | 8 | 0.25806 |
| 7125 | S8_65081581 | 8 | 65081581 | 171 | G | A | G | 0.91935 | A | 0.08065 | 5 | 0.16129 |
| 7126 | S8_65370175 | 8 | 65370175 | 288594 | G | A | A | 0.74194 | G | 0.25806 | 12 | 0.3871 |

| Site number | SNP name | Chromosome | Physical position | Physical distance from the previous SNP | Reference allele | Alternative allele | Major allele | Major allele frequency | Minor allele | Minor allele frequency | Number heterozygous | Heterozygosity |
|---|---|---|---|---|---|---|---|---|---|---|---|---|
| 7127 | S8_65458226 | 8 | 65458226 | 88051 | A | G | A | 0.95161 | G | 0.04839 | 3 | 0.09677 |
| 7128 | S8_65712310 | 8 | 65712310 | 254084 | G | A | G | 0.83871 | A | 0.16129 | 10 | 0.32258 |
| 7129 | S8_66180677 | 8 | 66180677 | 468367 | A | C | A | 0.80645 | C | 0.19355 | 6 | 0.19355 |
| 7130 | S8_66180811 | 8 | 66180811 | 134 | G | A | G | 0.91935 | A | 0.08065 | 3 | 0.09677 |
| 7131 | S8_66180868 | 8 | 66180868 | 57 | T | C | T | 0.95161 | C | 0.04839 | 3 | 0.09677 |
| 7132 | S8_66942371 | 8 | 66942371 | 761503 | A | G | A | 0.58065 | G | 0.41935 | 12 | 0.3871 |
| 7133 | S8_66942530 | 8 | 66942530 | 159 | C | T | C | 0.85484 | T | 0.14516 | 7 | 0.22581 |
| 7134 | S8_66942550 | 8 | 66942550 | 20 | C | T | C | 0.91935 | T | 0.08065 | 3 | 0.09677 |
| 7135 | S8_67033040 | 8 | 67033040 | 90490 | C | A | C | 0.8871 | A | 0.1129 | 5 | 0.16129 |
| 7136 | S8_67063741 | 8 | 67063741 | 30701 | T | C | C | 0.56452 | T | 0.43548 | 13 | 0.41935 |
| 7137 | S8_67063806 | 8 | 67063806 | 65 | A | G | A | 0.74194 | G | 0.25806 | 14 | 0.45161 |
| 7138 | S8_67288440 | 8 | 67288440 | 224634 | T | C | T | 0.83871 | C | 0.16129 | 8 | 0.25806 |
| 7139 | S8_67288558 | 8 | 67288558 | 118 | A | G | A | 0.82258 | G | 0.17742 | 9 | 0.29032 |
| 7140 | S8_67527837 | 8 | 67527837 | 239279 | G | A | G | 0.66129 | A | 0.33871 | 11 | 0.35484 |
| 7141 | S8_67721439 | 8 | 67721439 | 193602 | A | T | A | 0.87097 | T | 0.12903 | 6 | 0.19355 |
| 7142 | S8_67721484 | 8 | 67721484 | 45 | T | C | C | 0.91935 | T | 0.08065 | 3 | 0.09677 |
| 7143 | S8_67953625 | 8 | 67953625 | 232141 | C | T | C | 0.87097 | T | 0.12903 | 4 | 0.12903 |
| 7144 | S8_67953626 | 8 | 67953626 | 1 | A | G | G | 0.93548 | A | 0.06452 | 2 | 0.06452 |
| 7145 | S8_68239920 | 8 | 68239920 | 286294 | G | A | G | 0.87097 | A | 0.12903 | 6 | 0.19355 |
| 7146 | S8_68240009 | 8 | 68240009 | 89 | T | C | C | 0.79032 | T | 0.20968 | 9 | 0.29032 |
| 7147 | S8_68240023 | 8 | 68240023 | 14 | T | A | A | 0.79032 | T | 0.20968 | 9 | 0.29032 |
| 7148 | S8_68410505 | 8 | 68410505 | 170482 | C | A | C | 0.93548 | A | 0.06452 | 4 | 0.12903 |
| 7149 | S8_68569249 | 8 | 68569249 | 158744 | A | G | A | 0.74194 | G | 0.25806 | 14 | 0.45161 |
| 7150 | S8_68569311 | 8 | 68569311 | 62 | C | T | C | 0.79032 | T | 0.20968 | 11 | 0.35484 |
| 7151 | S8_68569313 | 8 | 68569313 | 2 | T | C | T | 0.93548 | C | 0.06452 | 4 | 0.12903 |
| 7152 | S8_68755328 | 8 | 68755328 | 186015 | G | A | G | 0.91935 | A | 0.08065 | 3 | 0.09677 |
| 7153 | S8_69233851 | 8 | 69233851 | 478523 | C | T | C | 0.93548 | T | 0.06452 | 4 | 0.12903 |
| 7154 | S8_69233910 | 8 | 69233910 | 59 | C | T | T | 0.66129 | C | 0.33871 | 9 | 0.29032 |
| 7155 | S8_69409087 | 8 | 69409087 | 175177 | G | A | A | 0.72581 | G | 0.27419 | 11 | 0.35484 |
| 7156 | S8_69851210 | 8 | 69851210 | 442123 | C | A | C | 0.6129 | A | 0.3871 | 10 | 0.32258 |
| 7157 | S8_69851218 | 8 | 69851218 | 8 | C | A | C | 0.6129 | A | 0.3871 | 10 | 0.32258 |
| 7158 | S8_69851267 | 8 | 69851267 | 49 | C | T | C | 0.91935 | T | 0.08065 | 5 | 0.16129 |
| 7159 | S8_69851274 | 8 | 69851274 | 7 | C | T | C | 0.6129 | T | 0.3871 | 10 | 0.32258 |
| 7160 | S8_69851316 | 8 | 69851316 | 42 | G | A | G | 0.6129 | A | 0.3871 | 10 | 0.32258 |
| 7161 | S8_69910174 | 8 | 69910174 | 58858 | C | T | C | 0.91935 | T | 0.08065 | 5 | 0.16129 |
| 7162 | S8_70009286 | 8 | 70009286 | 99112 | G | T | G | 0.8871 | T | 0.1129 | 7 | 0.22581 |
| 7163 | S8_70127110 | 8 | 70127110 | 117824 | G | A | A | 0.53226 | G | 0.46774 | 13 | 0.41935 |
| 7164 | S8_70127213 | 8 | 70127213 | 103 | C | T | C | 0.58065 | T | 0.41935 | 14 | 0.45161 |
| 7165 | S8_70127245 | 8 | 70127245 | 32 | A | G | A | 0.74194 | G | 0.25806 | 8 | 0.25806 |
| 7166 | S8_70566081 | 8 | 70566081 | 438836 | C | T | C | 0.8871 | T | 0.1129 | 7 | 0.22581 |
| 7167 | S8_70700868 | 8 | 70700868 | 134787 | G | A | G | 0.93548 | A | 0.06452 | 2 | 0.06452 |
| 7168 | S8_70710789 | 8 | 70710789 | 9921 | G | A | G | 0.70968 | A | 0.29032 | 10 | 0.32258 |
| 7169 | S8_70720002 | 8 | 70720002 | 9213 | C | A | C | 0.83871 | A | 0.16129 | 6 | 0.19355 |
| 7170 | S8_70720163 | 8 | 70720163 | 161 | T | C | T | 0.83871 | C | 0.16129 | 6 | 0.19355 |
| 7171 | S8_70830773 | 8 | 70830773 | 110610 | C | T | C | 0.67742 | T | 0.32258 | 18 | 0.58065 |
| 7172 | S8_70854331 | 8 | 70854331 | 23558 | A | G | A | 0.70968 | G | 0.29032 | 14 | 0.45161 |
| 7173 | S8_70920001 | 8 | 70920001 | 65670 | G | A | G | 0.95161 | A | 0.04839 | 3 | 0.09677 |
| 7174 | S8_70920017 | 8 | 70920017 | 16 | G | A | G | 0.93548 | A | 0.06452 | 4 | 0.12903 |
| 7175 | S8_71263049 | 8 | 71263049 | 343032 | A | G | A | 0.82258 | G | 0.17742 | 7 | 0.22581 |
| 7176 | S8_71263195 | 8 | 71263195 | 146 | G | A | G | 0.79032 | A | 0.20968 | 9 | 0.29032 |
| 7177 | S8_71263212 | 8 | 71263212 | 17 | C | T | T | 0.67742 | C | 0.32258 | 10 | 0.32258 |
| 7178 | S8_71263257 | 8 | 71263257 | 45 | T | C | T | 0.53226 | C | 0.46774 | 17 | 0.54839 |
| 7179 | S8_72175274 | 8 | 72175274 | 912017 | C | T | C | 0.70968 | T | 0.29032 | 10 | 0.32258 |
| 7180 | S8_72175493 | 8 | 72175493 | 219 | A | G | A | 0.70968 | G | 0.29032 | 10 | 0.32258 |

| Site number | SNP name | Chromosome | Physical position | Physical distance from the previous SNP | Reference allele | Alternative allele | Major allele | Major allele frequency | Minor allele | Minor allele frequency | Number heterozygous | Heterozygosity |
|---|---|---|---|---|---|---|---|---|---|---|---|---|
| 7181 | S8_72443083 | 8 | 72443083 | 267590 | G | A | G | 0.95161 | A | 0.04839 | 3 | 0.09677 |
| 7182 | S8_72443092 | 8 | 72443092 | 9 | G | T | G | 0.72581 | T | 0.27419 | 9 | 0.29032 |
| 7183 | S8_72443131 | 8 | 72443131 | 39 | C | T | C | 0.83871 | T | 0.16129 | 10 | 0.32258 |
| 7184 | S8_72443260 | 8 | 72443260 | 129 | T | C | T | 0.83871 | C | 0.16129 | 10 | 0.32258 |
| 7185 | S8_72443261 | 8 | 72443261 | 1 | G | A | G | 0.83871 | A | 0.16129 | 10 | 0.32258 |
| 7186 | S8_72443277 | 8 | 72443277 | 16 | G | A | G | 0.83871 | A | 0.16129 | 10 | 0.32258 |
| 7187 | S8_72646201 | 8 | 72646201 | 202924 | G | A | G | 0.87097 | A | 0.12903 | 6 | 0.19355 |
| 7188 | S8_72749925 | 8 | 72749925 | 103724 | A | C | A | 0.85484 | C | 0.14516 | 9 | 0.29032 |
| 7189 | S8_73115253 | 8 | 73115253 | 365328 | G | A | G | 0.82258 | A | 0.17742 | 9 | 0.29032 |
| 7190 | S8_73127861 | 8 | 73127861 | 12608 | T | C | T | 0.70968 | C | 0.29032 | 12 | 0.3871 |
| 7191 | S8_73136610 | 8 | 73136610 | 8749 | C | T | C | 0.83871 | T | 0.16129 | 8 | 0.25806 |
| 7192 | S8_73136667 | 8 | 73136667 | 57 | A | T | A | 0.91935 | T | 0.08065 | 3 | 0.09677 |
| 7193 | S8_73313398 | 8 | 73313398 | 176731 | G | T | G | 0.85484 | T | 0.14516 | 9 | 0.29032 |
| 7194 | S8_73907983 | 8 | 73907983 | 594585 | T | A | T | 0.95161 | A | 0.04839 | 1 | 0.03226 |
| 7195 | S8_74117478 | 8 | 74117478 | 209495 | G | A | G | 0.87097 | A | 0.12903 | 6 | 0.19355 |
| 7196 | S8_75273565 | 8 | 75273565 | 1156087 | A | G | G | 0.74194 | A | 0.25806 | 12 | 0.3871 |
| 7197 | S8_75561316 | 8 | 75561316 | 287751 | C | T | C | 0.93548 | T | 0.06452 | 4 | 0.12903 |
| 7198 | S8_75810058 | 8 | 75810058 | 248742 | C | T | C | 0.91935 | T | 0.08065 | 3 | 0.09677 |
| 7199 | S8_76247251 | 8 | 76247251 | 437193 | C | T | C | 0.82258 | T | 0.17742 | 9 | 0.29032 |
| 7200 | S8_76460651 | 8 | 76460651 | 213400 | G | T | T | 0.62903 | G | 0.37097 | 13 | 0.41935 |
| 7201 | S8_76849441 | 8 | 76849441 | 388790 | C | T | C | 0.70968 | T | 0.29032 | 8 | 0.25806 |
| 7202 | S8_76934422 | 8 | 76934422 | 84981 | T | C | T | 0.79032 | C | 0.20968 | 11 | 0.35484 |
| 7203 | S8_76934516 | 8 | 76934516 | 94 | C | T | T | 0.51613 | C | 0.48387 | 16 | 0.51613 |
| 7204 | S8_77006899 | 8 | 77006899 | 72383 | C | T | T | 0.69355 | C | 0.30645 | 11 | 0.35484 |
| 7205 | S8_77044443 | 8 | 77044443 | 37544 | C | T | C | 0.91935 | T | 0.08065 | 3 | 0.09677 |
| 7206 | S8_79088745 | 8 | 79088745 | 2044302 | T | C | C | 0.75806 | T | 0.24194 | 9 | 0.29032 |
| 7207 | S8_79088783 | 8 | 79088783 | 38 | T | C | T | 0.93548 | C | 0.06452 | 4 | 0.12903 |
| 7208 | S8_79088888 | 8 | 79088888 | 105 | T | C | C | 0.75806 | T | 0.24194 | 9 | 0.29032 |
| 7209 | S8_79088951 | 8 | 79088951 | 63 | C | T | C | 0.95161 | T | 0.04839 | 3 | 0.09677 |
| 7210 | S8_79299054 | 8 | 79299054 | 210103 | G | A | G | 0.93548 | A | 0.06452 | 4 | 0.12903 |
| 7211 | S8_79299137 | 8 | 79299137 | 83 | A | G | A | 0.91935 | G | 0.08065 | 5 | 0.16129 |
| 7212 | S8_79630052 | 8 | 79630052 | 330915 | G | C | G | 0.58065 | C | 0.41935 | 18 | 0.58065 |
| 7213 | S8_79711608 | 8 | 79711608 | 81556 | G | A | G | 0.95161 | A | 0.04839 | 3 | 0.09677 |
| 7214 | S8_79711617 | 8 | 79711617 | 9 | G | A | G | 0.85484 | A | 0.14516 | 7 | 0.22581 |
| 7215 | S8_79711784 | 8 | 79711784 | 167 | T | A | T | 0.95161 | A | 0.04839 | 3 | 0.09677 |
| 7216 | S8_79855646 | 8 | 79855646 | 143862 | C | T | C | 0.90323 | T | 0.09677 | 6 | 0.19355 |
| 7217 | S8_79898131 | 8 | 79898131 | 42485 | C | T | C | 0.95161 | T | 0.04839 | 3 | 0.09677 |
| 7218 | S8_80419126 | 8 | 80419126 | 520995 | C | G | C | 0.6129 | G | 0.3871 | 14 | 0.45161 |
| 7219 | S8_81325731 | 8 | 81325731 | 906605 | G | A | G | 0.95161 | A | 0.04839 | 3 | 0.09677 |
| 7220 | S8_81389917 | 8 | 81389917 | 64186 | A | G | A | 0.58065 | G | 0.41935 | 18 | 0.58065 |
| 7221 | S8_81389941 | 8 | 81389941 | 24 | T | G | T | 0.91935 | G | 0.08065 | 5 | 0.16129 |
| 7222 | S8_81389966 | 8 | 81389966 | 25 | C | T | C | 0.58065 | T | 0.41935 | 18 | 0.58065 |
| 7223 | S8_81390038 | 8 | 81390038 | 72 | C | T | T | 0.53226 | C | 0.46774 | 17 | 0.54839 |
| 7224 | S8_82555220 | 8 | 82555220 | 1165182 | A | G | A | 0.90323 | G | 0.09677 | 6 | 0.19355 |
| 7225 | S8_82706535 | 8 | 82706535 | 151315 | G | A | G | 0.82258 | A | 0.17742 | 7 | 0.22581 |
| 7226 | S8_82883178 | 8 | 82883178 | 176643 | A | T | A | 0.58065 | T | 0.41935 | 14 | 0.45161 |
| 7227 | S8_82969412 | 8 | 82969412 | 86234 | T | C | T | 0.74194 | C | 0.25806 | 14 | 0.45161 |
| 7228 | S8_84096636 | 8 | 84096636 | 1127224 | G | A | A | 0.66129 | G | 0.33871 | 15 | 0.48387 |
| 7229 | S8_84096682 | 8 | 84096682 | 46 | T | G | G | 0.93548 | T | 0.06452 | 4 | 0.12903 |
| 7230 | S8_84411170 | 8 | 84411170 | 314488 | A | T | A | 0.90323 | T | 0.09677 | 6 | 0.19355 |
| 7231 | S8_84711488 | 8 | 84711488 | 300318 | A | G | A | 0.5 | G | 0.5 | 17 | 0.54839 |
| 7232 | S8_84711584 | 8 | 84711584 | 96 | A | G | A | 0.5 | G | 0.5 | 17 | 0.54839 |
| 7233 | S8_84711597 | 8 | 84711597 | 13 | A | G | A | 0.5 | G | 0.5 | 17 | 0.54839 |
| 7234 | S8_84804092 | 8 | 84804092 | 92495 | T | C | C | 0.62903 | T | 0.37097 | 13 | 0.41935 |

| Site number | SNP name | Chromosome | Physical position | Physical distance from the previous SNP | Reference allele | Alternative allele | Major allele | Major allele frequency | Minor allele | Minor allele frequency | Number heterozygous | Heterozygosity |
|---|---|---|---|---|---|---|---|---|---|---|---|---|
| 7235 | S8_85456142 | 8 | 85456142 | 652050 | C | T | C | 0.95161 | T | 0.04839 | 3 | 0.09677 |
| 7236 | S8_85705160 | 8 | 85705160 | 249018 | C | G | C | 0.8871 | G | 0.1129 | 7 | 0.22581 |
| 7237 | S8_85793793 | 8 | 85793793 | 88633 | C | T | C | 0.83871 | T | 0.16129 | 8 | 0.25806 |
| 7238 | S8_85793869 | 8 | 85793869 | 76 | G | C | G | 0.95161 | C | 0.04839 | 3 | 0.09677 |
| 7239 | S8_85916317 | 8 | 85916317 | 122448 | T | C | C | 0.91935 | T | 0.08065 | 5 | 0.16129 |
| 7240 | S8_85916388 | 8 | 85916388 | 71 | C | T | C | 0.87097 | T | 0.12903 | 6 | 0.19355 |
| 7241 | S8_86706350 | 8 | 86706350 | 789962 | C | T | C | 0.80645 | T | 0.19355 | 8 | 0.25806 |
| 7242 | S8_86706366 | 8 | 86706366 | 16 | G | A | G | 0.80645 | A | 0.19355 | 8 | 0.25806 |
| 7243 | S8_86706401 | 8 | 86706401 | 35 | A | G | A | 0.93548 | G | 0.06452 | 4 | 0.12903 |
| 7244 | S8_86706417 | 8 | 86706417 | 16 | G | A | G | 0.93548 | A | 0.06452 | 4 | 0.12903 |
| 7245 | S8_86706471 | 8 | 86706471 | 54 | T | C | T | 0.51613 | C | 0.48387 | 16 | 0.51613 |
| 7246 | S8_86706478 | 8 | 86706478 | 7 | A | G | A | 0.93548 | G | 0.06452 | 4 | 0.12903 |
| 7247 | S8_86809953 | 8 | 86809953 | 103475 | T | C | T | 0.74194 | C | 0.25806 | 14 | 0.45161 |
| 7248 | S8_86883213 | 8 | 86883213 | 73260 | C | T | C | 0.85484 | T | 0.14516 | 7 | 0.22581 |
| 7249 | S8_87074920 | 8 | 87074920 | 191707 | C | T | C | 0.91935 | T | 0.08065 | 5 | 0.16129 |
| 7250 | S8_87075003 | 8 | 87075003 | 83 | G | C | G | 0.66129 | C | 0.33871 | 17 | 0.54839 |
| 7251 | S8_87561640 | 8 | 87561640 | 486637 | G | T | G | 0.59677 | T | 0.40323 | 17 | 0.54839 |
| 7252 | S8_87966883 | 8 | 87966883 | 405243 | G | C | G | 0.83871 | C | 0.16129 | 10 | 0.32258 |
| 7253 | S8_87966925 | 8 | 87966925 | 42 | G | A | G | 0.77419 | A | 0.22581 | 12 | 0.3871 |
| 7254 | S8_87966993 | 8 | 87966993 | 68 | C | T | C | 0.77419 | T | 0.22581 | 12 | 0.3871 |
| 7255 | S8_88156343 | 8 | 88156343 | 189350 | C | T | C | 0.58065 | T | 0.41935 | 18 | 0.58065 |
| 7256 | S8_88156374 | 8 | 88156374 | 31 | G | A | G | 0.72581 | A | 0.27419 | 9 | 0.29032 |
| 7257 | S8_88739050 | 8 | 88739050 | 582676 | A | G | A | 0.79032 | G | 0.20968 | 9 | 0.29032 |
| 7258 | S8_88739083 | 8 | 88739083 | 33 | C | T | T | 0.54839 | C | 0.45161 | 14 | 0.45161 |
| 7259 | S8_88947345 | 8 | 88947345 | 208262 | A | G | A | 0.93548 | G | 0.06452 | 4 | 0.12903 |
| 7260 | S8_88947429 | 8 | 88947429 | 84 | G | A | G | 0.93548 | A | 0.06452 | 4 | 0.12903 |
| 7261 | S8_88947435 | 8 | 88947435 | 6 | C | T | C | 0.74194 | T | 0.25806 | 12 | 0.3871 |
| 7262 | S8_88947436 | 8 | 88947436 | 1 | G | A | G | 0.93548 | A | 0.06452 | 4 | 0.12903 |
| 7263 | S8_88947512 | 8 | 88947512 | 76 | C | T | C | 0.93548 | T | 0.06452 | 4 | 0.12903 |
| 7264 | S8_89357061 | 8 | 89357061 | 409549 | T | C | T | 0.70968 | C | 0.29032 | 12 | 0.3871 |
| 7265 | S8_89357141 | 8 | 89357141 | 80 | A | G | G | 0.70968 | A | 0.29032 | 14 | 0.45161 |
| 7266 | S8_89446046 | 8 | 89446046 | 88905 | C | T | C | 0.93548 | T | 0.06452 | 2 | 0.06452 |
| 7267 | S8_89465843 | 8 | 89465843 | 19797 | T | C | T | 0.70968 | C | 0.29032 | 10 | 0.32258 |
| 7268 | S8_89466033 | 8 | 89466033 | 190 | G | A | G | 0.70968 | A | 0.29032 | 10 | 0.32258 |
| 7269 | S8_89638695 | 8 | 89638695 | 172662 | G | T | G | 0.85484 | T | 0.14516 | 9 | 0.29032 |
| 7270 | S8_90627889 | 8 | 90627889 | 989194 | T | G | T | 0.95161 | G | 0.04839 | 3 | 0.09677 |
| 7271 | S8_90627949 | 8 | 90627949 | 60 | C | T | C | 0.95161 | T | 0.04839 | 3 | 0.09677 |
| 7272 | S8_90628002 | 8 | 90628002 | 53 | C | A | C | 0.95161 | A | 0.04839 | 3 | 0.09677 |
| 7273 | S8_90628039 | 8 | 90628039 | 37 | G | A | G | 0.74194 | A | 0.25806 | 10 | 0.32258 |
| 7274 | S8_90628052 | 8 | 90628052 | 13 | C | T | C | 0.87097 | T | 0.12903 | 6 | 0.19355 |
| 7275 | S8_90628069 | 8 | 90628069 | 17 | C | T | C | 0.95161 | T | 0.04839 | 3 | 0.09677 |
| 7276 | S8_91077762 | 8 | 91077762 | 449693 | G | A | G | 0.95161 | A | 0.04839 | 3 | 0.09677 |
| 7277 | S8_91077805 | 8 | 91077805 | 43 | T | C | C | 0.70968 | T | 0.29032 | 16 | 0.51613 |
| 7278 | S8_91077860 | 8 | 91077860 | 55 | G | T | G | 0.83871 | T | 0.16129 | 10 | 0.32258 |
| 7279 | S8_91077863 | 8 | 91077863 | 3 | C | T | T | 0.70968 | C | 0.29032 | 16 | 0.51613 |
| 7280 | S8_91077894 | 8 | 91077894 | 31 | C | T | C | 0.66129 | T | 0.33871 | 15 | 0.48387 |
| 7281 | S8_91077895 | 8 | 91077895 | 1 | G | A | G | 0.6129 | A | 0.3871 | 14 | 0.45161 |
| 7282 | S8_91232005 | 8 | 91232005 | 154110 | G | A | A | 0.95161 | G | 0.04839 | 3 | 0.09677 |
| 7283 | S8_91232069 | 8 | 91232069 | 64 | C | T | C | 0.95161 | T | 0.04839 | 3 | 0.09677 |
| 7284 | S8_91232071 | 8 | 91232071 | 2 | C | T | C | 0.93548 | T | 0.06452 | 4 | 0.12903 |
| 7285 | S8_91232088 | 8 | 91232088 | 17 | C | T | C | 0.80645 | T | 0.19355 | 10 | 0.32258 |
| 7286 | S8_92564124 | 8 | 92564124 | 1332036 | A | G | G | 0.83871 | A | 0.16129 | 10 | 0.32258 |
| 7287 | S8_93084492 | 8 | 93084492 | 520368 | C | T | C | 0.74194 | T | 0.25806 | 8 | 0.25806 |
| 7288 | S8_93084504 | 8 | 93084504 | 12 | A | C | C | 0.69355 | A | 0.30645 | 11 | 0.35484 |

| Site number | SNP name | Chromosome | Physical position | Physical distance from the previous SNP | Reference allele | Alternative allele | Major allele | Major allele frequency | Minor allele | Minor allele frequency | Number heterozygous | Heterozygosity |
|---|---|---|---|---|---|---|---|---|---|---|---|---|
| 7289 | S8_93248296 | 8 | 93248296 | 163792 | A | G | G | 0.69355 | A | 0.30645 | 13 | 0.41935 |
| 7290 | S8_94140493 | 8 | 94140493 | 892197 | G | A | G | 0.95161 | A | 0.04839 | 3 | 0.09677 |
| 7291 | S8_94140610 | 8 | 94140610 | 117 | T | C | T | 0.85484 | C | 0.14516 | 7 | 0.22581 |
| 7292 | S8_94140616 | 8 | 94140616 | 6 | C | T | C | 0.6129 | T | 0.3871 | 12 | 0.3871 |
| 7293 | S8_94518860 | 8 | 94518860 | 378244 | C | T | C | 0.91935 | T | 0.08065 | 5 | 0.16129 |
| 7294 | S8_94519021 | 8 | 94519021 | 161 | C | T | T | 0.77419 | C | 0.22581 | 8 | 0.25806 |
| 7295 | S8_94519438 | 8 | 94519438 | 417 | G | T | G | 0.87097 | T | 0.12903 | 8 | 0.25806 |
| 7296 | S8_95593666 | 8 | 95593666 | 1074228 | A | G | A | 0.95161 | G | 0.04839 | 3 | 0.09677 |
| 7297 | S8_95593780 | 8 | 95593780 | 114 | A | G | A | 0.85484 | G | 0.14516 | 9 | 0.29032 |
| 7298 | S8_95593796 | 8 | 95593796 | 16 | T | G | T | 0.67742 | G | 0.32258 | 16 | 0.51613 |
| 7299 | S8_95753462 | 8 | 95753462 | 159666 | T | A | T | 0.80645 | A | 0.19355 | 12 | 0.3871 |
| 7300 | S8_95753475 | 8 | 95753475 | 13 | A | G | A | 0.70968 | G | 0.29032 | 12 | 0.3871 |
| 7301 | S8_95753521 | 8 | 95753521 | 46 | A | G | A | 0.70968 | G | 0.29032 | 12 | 0.3871 |
| 7302 | S8_95753532 | 8 | 95753532 | 11 | G | A | G | 0.79032 | A | 0.20968 | 9 | 0.29032 |
| 7303 | S8_95836799 | 8 | 95836799 | 83267 | G | A | G | 0.91935 | A | 0.08065 | 5 | 0.16129 |
| 7304 | S8_95978629 | 8 | 95978629 | 141830 | G | C | G | 0.80645 | C | 0.19355 | 8 | 0.25806 |
| 7305 | S8_95978798 | 8 | 95978798 | 169 | A | G | A | 0.66129 | G | 0.33871 | 13 | 0.41935 |
| 7306 | S8_95978861 | 8 | 95978861 | 63 | A | T | A | 0.62903 | T | 0.37097 | 17 | 0.54839 |
| 7307 | S8_95978877 | 8 | 95978877 | 16 | A | C | A | 0.62903 | C | 0.37097 | 17 | 0.54839 |
| 7308 | S8_95978950 | 8 | 95978950 | 73 | G | C | G | 0.62903 | C | 0.37097 | 17 | 0.54839 |
| 7309 | S8_95979056 | 8 | 95979056 | 106 | A | G | A | 0.8871 | G | 0.1129 | 7 | 0.22581 |
| 7310 | S8_96603957 | 8 | 96603957 | 624901 | A | T | A | 0.59677 | T | 0.40323 | 15 | 0.48387 |
| 7311 | S8_97338092 | 8 | 97338092 | 734135 | T | C | T | 0.93548 | C | 0.06452 | 4 | 0.12903 |
| 7312 | S8_97480570 | 8 | 97480570 | 142478 | G | A | G | 0.91935 | A | 0.08065 | 5 | 0.16129 |
| 7313 | S8_97480661 | 8 | 97480661 | 91 | A | G | A | 0.95161 | G | 0.04839 | 3 | 0.09677 |
| 7314 | S8_97498218 | 8 | 97498218 | 17557 | C | T | T | 0.53226 | C | 0.46774 | 17 | 0.54839 |
| 7315 | S8_98447945 | 8 | 98447945 | 949727 | G | A | G | 0.51613 | A | 0.48387 | 16 | 0.51613 |
| 7316 | S8_98447978 | 8 | 98447978 | 33 | T | A | A | 0.5 | T | 0.5 | 17 | 0.54839 |
| 7317 | S8_98447982 | 8 | 98447982 | 4 | A | G | A | 0.5 | G | 0.5 | 17 | 0.54839 |
| 7318 | S8_98447986 | 8 | 98447986 | 4 | C | G | C | 0.90323 | G | 0.09677 | 6 | 0.19355 |
| 7319 | S8_98448090 | 8 | 98448090 | 104 | T | C | C | 0.66129 | T | 0.33871 | 11 | 0.35484 |
| 7320 | S8_98448112 | 8 | 98448112 | 22 | C | T | T | 0.64516 | C | 0.35484 | 12 | 0.3871 |
| 7321 | S8_98448120 | 8 | 98448120 | 8 | G | T | T | 0.66129 | G | 0.33871 | 11 | 0.35484 |
| 7322 | S8_98816782 | 8 | 98816782 | 368662 | A | G | A | 0.77419 | G | 0.22581 | 12 | 0.3871 |
| 7323 | S8_99414374 | 8 | 99414374 | 597592 | G | T | G | 0.85484 | T | 0.14516 | 5 | 0.16129 |
| 7324 | S8_99414387 | 8 | 99414387 | 13 | C | T | C | 0.85484 | T | 0.14516 | 5 | 0.16129 |
| 7325 | S8_99414396 | 8 | 99414396 | 9 | G | T | G | 0.93548 | T | 0.06452 | 4 | 0.12903 |
| 7326 | S8_99518384 | 8 | 99518384 | 103988 | A | C | A | 0.93548 | C | 0.06452 | 4 | 0.12903 |
| 7327 | S8_99518399 | 8 | 99518399 | 15 | A | G | A | 0.93548 | G | 0.06452 | 4 | 0.12903 |
| 7328 | S8_99518554 | 8 | 99518554 | 155 | T | C | T | 0.91935 | C | 0.08065 | 5 | 0.16129 |
| 7329 | S8_99518613 | 8 | 99518613 | 59 | C | T | C | 0.93548 | T | 0.06452 | 4 | 0.12903 |
| 7330 | S8_99640334 | 8 | 99640334 | 121721 | C | T | C | 0.64516 | T | 0.35484 | 10 | 0.32258 |
| 7331 | S8_99640453 | 8 | 99640453 | 119 | T | G | T | 0.83871 | G | 0.16129 | 8 | 0.25806 |
| 7332 | S8_100765512 | 8 | 100765512 | 1125059 | C | T | C | 0.91935 | T | 0.08065 | 5 | 0.16129 |
| 7333 | S8_101193775 | 8 | 101193775 | 428263 | A | G | A | 0.95161 | G | 0.04839 | 3 | 0.09677 |
| 7334 | S8_101254788 | 8 | 101254788 | 61013 | G | A | G | 0.8871 | A | 0.1129 | 5 | 0.16129 |
| 7335 | S8_101467282 | 8 | 101467282 | 212494 | G | A | G | 0.91935 | A | 0.08065 | 3 | 0.09677 |
| 7336 | S8_101738338 | 8 | 101738338 | 271056 | G | A | G | 0.91935 | A | 0.08065 | 5 | 0.16129 |
| 7337 | S8_101738411 | 8 | 101738411 | 73 | C | T | T | 0.77419 | C | 0.22581 | 14 | 0.45161 |
| 7338 | S8_101778338 | 8 | 101778338 | 39927 | C | T | C | 0.91935 | T | 0.08065 | 3 | 0.09677 |
| 7339 | S8_101901663 | 8 | 101901663 | 123325 | C | T | C | 0.95161 | T | 0.04839 | 1 | 0.03226 |
| 7340 | S8_101901825 | 8 | 101901825 | 162 | C | T | C | 0.95161 | T | 0.04839 | 3 | 0.09677 |
| 7341 | S8_102446582 | 8 | 102446582 | 544757 | G | A | G | 0.90323 | A | 0.09677 | 6 | 0.19355 |
| 7342 | S8_102446667 | 8 | 102446667 | 85 | C | T | C | 0.85484 | T | 0.14516 | 7 | 0.22581 |

| Site number | SNP name | Chromosome | Physical position | Physical distance from the previous SNP | Reference allele | Alternative allele | Major allele | Major allele frequency | Minor allele | Minor allele frequency | Number heterozygous | Heterozygosity |
|---|---|---|---|---|---|---|---|---|---|---|---|---|
| 7343 | S8_102491163 | 8 | 102491163 | 44496 | G | A | G | 0.95161 | A | 0.04839 | 1 | 0.03226 |
| 7344 | S8_102491270 | 8 | 102491270 | 107 | G | C | C | 0.54839 | G | 0.45161 | 16 | 0.51613 |
| 7345 | S8_102588822 | 8 | 102588822 | 97552 | G | C | G | 0.80645 | C | 0.19355 | 10 | 0.32258 |
| 7346 | S8_102588851 | 8 | 102588851 | 29 | G | A | G | 0.72581 | A | 0.27419 | 13 | 0.41935 |
| 7347 | S8_102730547 | 8 | 102730547 | 141696 | C | G | C | 0.62903 | G | 0.37097 | 15 | 0.48387 |
| 7348 | S8_103304237 | 8 | 103304237 | 573690 | G | A | G | 0.85484 | A | 0.14516 | 7 | 0.22581 |
| 7349 | S8_103304244 | 8 | 103304244 | 7 | A | G | A | 0.91935 | G | 0.08065 | 5 | 0.16129 |
| 7350 | S8_103304269 | 8 | 103304269 | 25 | C | T | C | 0.91935 | T | 0.08065 | 5 | 0.16129 |
| 7351 | S8_103304342 | 8 | 103304342 | 73 | A | G | A | 0.85484 | G | 0.14516 | 7 | 0.22581 |
| 7352 | S8_103406602 | 8 | 103406602 | 102260 | G | A | G | 0.91935 | A | 0.08065 | 3 | 0.09677 |
| 7353 | S8_103423242 | 8 | 103423242 | 16640 | T | A | T | 0.82258 | A | 0.17742 | 9 | 0.29032 |
| 7354 | S8_103423464 | 8 | 103423464 | 222 | A | C | A | 0.95161 | C | 0.04839 | 3 | 0.09677 |
| 7355 | S8_103455482 | 8 | 103455482 | 32018 | G | A | G | 0.91935 | A | 0.08065 | 3 | 0.09677 |
| 7356 | S8_103455496 | 8 | 103455496 | 14 | A | G | A | 0.8871 | G | 0.1129 | 7 | 0.22581 |
| 7357 | S8_103455547 | 8 | 103455547 | 51 | A | T | A | 0.91935 | T | 0.08065 | 3 | 0.09677 |
| 7358 | S8_103494041 | 8 | 103494041 | 38494 | C | T | C | 0.91935 | T | 0.08065 | 3 | 0.09677 |
| 7359 | S8_103922565 | 8 | 103922565 | 428524 | G | A | G | 0.72581 | A | 0.27419 | 13 | 0.41935 |
| 7360 | S8_103922753 | 8 | 103922753 | 188 | G | A | G | 0.72581 | A | 0.27419 | 13 | 0.41935 |
| 7361 | S8_103975094 | 8 | 103975094 | 52341 | T | C | T | 0.66129 | C | 0.33871 | 15 | 0.48387 |
| 7362 | S8_103975115 | 8 | 103975115 | 21 | G | T | G | 0.91935 | T | 0.08065 | 3 | 0.09677 |
| 7363 | S8_103975188 | 8 | 103975188 | 73 | G | A | G | 0.91935 | A | 0.08065 | 5 | 0.16129 |
| 7364 | S8_104166895 | 8 | 104166895 | 191707 | C | T | T | 0.58065 | C | 0.41935 | 14 | 0.45161 |
| 7365 | S8_104349965 | 8 | 104349965 | 183070 | G | A | G | 0.79032 | A | 0.20968 | 11 | 0.35484 |
| 7366 | S8_104350007 | 8 | 104350007 | 42 | A | G | A | 0.72581 | G | 0.27419 | 9 | 0.29032 |
| 7367 | S8_104350029 | 8 | 104350029 | 22 | C | T | C | 0.74194 | T | 0.25806 | 8 | 0.25806 |
| 7368 | S8_104350048 | 8 | 104350048 | 19 | T | C | T | 0.51613 | C | 0.48387 | 16 | 0.51613 |
| 7369 | S8_104350128 | 8 | 104350128 | 80 | C | A | C | 0.72581 | A | 0.27419 | 9 | 0.29032 |
| 7370 | S8_104733417 | 8 | 104733417 | 383289 | G | A | G | 0.83871 | A | 0.16129 | 6 | 0.19355 |
| 7371 | S8_105048959 | 8 | 105048959 | 315542 | A | C | A | 0.93548 | C | 0.06452 | 4 | 0.12903 |
| 7372 | S8_105107669 | 8 | 105107669 | 58710 | T | C | C | 0.93548 | T | 0.06452 | 4 | 0.12903 |
| 7373 | S8_105177442 | 8 | 105177442 | 69773 | A | G | A | 0.93548 | G | 0.06452 | 4 | 0.12903 |
| 7374 | S8_105177598 | 8 | 105177598 | 156 | C | T | C | 0.90323 | T | 0.09677 | 6 | 0.19355 |
| 7375 | S8_105793554 | 8 | 105793554 | 615956 | G | C | G | 0.87097 | C | 0.12903 | 4 | 0.12903 |
| 7376 | S8_105793570 | 8 | 105793570 | 16 | C | G | C | 0.87097 | G | 0.12903 | 4 | 0.12903 |
| 7377 | S8_105793572 | 8 | 105793572 | 2 | G | A | G | 0.54839 | A | 0.45161 | 14 | 0.45161 |
| 7378 | S8_105793576 | 8 | 105793576 | 4 | G | A | G | 0.93548 | A | 0.06452 | 4 | 0.12903 |
| 7379 | S8_105793695 | 8 | 105793695 | 119 | A | G | A | 0.53226 | G | 0.46774 | 15 | 0.48387 |
| 7380 | S8_105793782 | 8 | 105793782 | 87 | C | T | C | 0.67742 | T | 0.32258 | 14 | 0.45161 |
| 7381 | S8_105800823 | 8 | 105800823 | 7041 | A | G | G | 0.93548 | A | 0.06452 | 4 | 0.12903 |
| 7382 | S8_105800996 | 8 | 105800996 | 173 | G | A | G | 0.87097 | A | 0.12903 | 4 | 0.12903 |
| 7383 | S8_105865520 | 8 | 105865520 | 64524 | C | A | C | 0.75806 | A | 0.24194 | 11 | 0.35484 |
| 7384 | S8_105865639 | 8 | 105865639 | 119 | T | C | T | 0.75806 | C | 0.24194 | 11 | 0.35484 |
| 7385 | S8_105869710 | 8 | 105869710 | 4071 | G | T | G | 0.87097 | T | 0.12903 | 4 | 0.12903 |
| 7386 | S8_105869764 | 8 | 105869764 | 54 | G | A | G | 0.75806 | A | 0.24194 | 9 | 0.29032 |
| 7387 | S8_105869819 | 8 | 105869819 | 55 | T | C | C | 0.64516 | T | 0.35484 | 12 | 0.3871 |
| 7388 | S8_105869849 | 8 | 105869849 | 30 | C | G | G | 0.51613 | C | 0.48387 | 14 | 0.45161 |
| 7389 | S8_105996284 | 8 | 105996284 | 126435 | G | C | G | 0.6129 | C | 0.3871 | 10 | 0.32258 |
| 7390 | S8_105996309 | 8 | 105996309 | 25 | C | T | C | 0.6129 | T | 0.3871 | 10 | 0.32258 |
| 7391 | S8_105996317 | 8 | 105996317 | 8 | A | G | A | 0.6129 | G | 0.3871 | 10 | 0.32258 |
| 7392 | S8_105996331 | 8 | 105996331 | 14 | A | G | A | 0.6129 | G | 0.3871 | 10 | 0.32258 |
| 7393 | S8_105996337 | 8 | 105996337 | 6 | A | G | A | 0.6129 | G | 0.3871 | 10 | 0.32258 |
| 7394 | S8_105996338 | 8 | 105996338 | 1 | G | T | G | 0.6129 | T | 0.3871 | 10 | 0.32258 |
| 7395 | S8_106417868 | 8 | 106417868 | 421530 | G | C | G | 0.91935 | C | 0.08065 | 5 | 0.16129 |
| 7396 | S8_106417903 | 8 | 106417903 | 35 | C | A | C | 0.62903 | A | 0.37097 | 9 | 0.29032 |

| Site number | SNP name | Chromosome | Physical position | Physical distance from the previous SNP | Reference allele | Alternative allele | Major allele | Major allele frequency | Minor allele | Minor allele frequency | Number heterozygous | Heterozygosity |
|---|---|---|---|---|---|---|---|---|---|---|---|---|
| 7397 | S8_106418006 | 8 | 106418006 | 103 | C | T | T | 0.53226 | C | 0.46774 | 11 | 0.35484 |
| 7398 | S8_106530888 | 8 | 106530888 | 112882 | T | C | T | 0.93548 | C | 0.06452 | 4 | 0.12903 |
| 7399 | S8_106664404 | 8 | 106664404 | 133516 | C | T | C | 0.53226 | T | 0.46774 | 13 | 0.41935 |
| 7400 | S8_106664444 | 8 | 106664444 | 40 | G | A | G | 0.83871 | A | 0.16129 | 10 | 0.32258 |
| 7401 | S8_106664582 | 8 | 106664582 | 138 | C | T | C | 0.91935 | T | 0.08065 | 5 | 0.16129 |
| 7402 | S8_106672346 | 8 | 106672346 | 7764 | G | C | G | 0.75806 | C | 0.24194 | 13 | 0.41935 |
| 7403 | S8_106672365 | 8 | 106672365 | 19 | T | C | T | 0.75806 | C | 0.24194 | 11 | 0.35484 |
| 7404 | S8_106782621 | 8 | 106782621 | 110256 | A | C | A | 0.69355 | C | 0.30645 | 13 | 0.41935 |
| 7405 | S8_106932649 | 8 | 106932649 | 150028 | A | G | A | 0.91935 | G | 0.08065 | 3 | 0.09677 |
| 7406 | S8_106958605 | 8 | 106958605 | 25956 | C | T | C | 0.5 | T | 0.5 | 13 | 0.41935 |
| 7407 | S8_106958612 | 8 | 106958612 | 7 | A | G | G | 0.58065 | A | 0.41935 | 16 | 0.51613 |
| 7408 | S8_107042648 | 8 | 107042648 | 84036 | G | A | G | 0.69355 | A | 0.30645 | 15 | 0.48387 |
| 7409 | S8_107042716 | 8 | 107042716 | 68 | C | T | C | 0.59677 | T | 0.40323 | 17 | 0.54839 |
| 7410 | S8_107042749 | 8 | 107042749 | 33 | G | A | A | 0.80645 | G | 0.19355 | 6 | 0.19355 |
| 7411 | S8_107433491 | 8 | 107433491 | 390742 | G | A | G | 0.82258 | A | 0.17742 | 7 | 0.22581 |
| 7412 | S8_107433554 | 8 | 107433554 | 63 | T | C | C | 0.79032 | T | 0.20968 | 9 | 0.29032 |
| 7413 | S8_107433626 | 8 | 107433626 | 72 | T | C | C | 0.93548 | T | 0.06452 | 4 | 0.12903 |
| 7414 | S8_107702059 | 8 | 107702059 | 268433 | C | T | C | 0.93548 | T | 0.06452 | 4 | 0.12903 |
| 7415 | S8_107877644 | 8 | 107877644 | 175585 | T | G | T | 0.90323 | G | 0.09677 | 6 | 0.19355 |
| 7416 | S8_108484425 | 8 | 108484425 | 606781 | C | A | C | 0.91935 | A | 0.08065 | 3 | 0.09677 |
| 7417 | S8_108779452 | 8 | 108779452 | 295027 | G | A | A | 0.51613 | G | 0.48387 | 16 | 0.51613 |
| 7418 | S8_108779461 | 8 | 108779461 | 9 | A | G | G | 0.51613 | A | 0.48387 | 16 | 0.51613 |
| 7419 | S8_108779544 | 8 | 108779544 | 83 | C | T | C | 0.91935 | T | 0.08065 | 5 | 0.16129 |
| 7420 | S8_108779619 | 8 | 108779619 | 75 | G | A | G | 0.95161 | A | 0.04839 | 3 | 0.09677 |
| 7421 | S8_108779635 | 8 | 108779635 | 16 | G | A | G | 0.90323 | A | 0.09677 | 4 | 0.12903 |
| 7422 | S8_108788040 | 8 | 108788040 | 8405 | A | G | G | 0.56452 | A | 0.43548 | 13 | 0.41935 |
| 7423 | S8_108788055 | 8 | 108788055 | 15 | C | T | T | 0.70968 | C | 0.29032 | 14 | 0.45161 |
| 7424 | S8_108788080 | 8 | 108788080 | 25 | G | A | G | 0.87097 | A | 0.12903 | 6 | 0.19355 |
| 7425 | S8_109057686 | 8 | 109057686 | 269606 | T | A | T | 0.77419 | A | 0.22581 | 8 | 0.25806 |
| 7426 | S8_109057770 | 8 | 109057770 | 84 | G | A | G | 0.95161 | A | 0.04839 | 3 | 0.09677 |
| 7427 | S8_109057777 | 8 | 109057777 | 7 | T | A | A | 0.62903 | T | 0.37097 | 13 | 0.41935 |
| 7428 | S8_109057779 | 8 | 109057779 | 2 | T | C | C | 0.62903 | T | 0.37097 | 13 | 0.41935 |
| 7429 | S8_109057788 | 8 | 109057788 | 9 | A | G | G | 0.6129 | A | 0.3871 | 14 | 0.45161 |
| 7430 | S8_109057806 | 8 | 109057806 | 18 | T | C | C | 0.6129 | T | 0.3871 | 14 | 0.45161 |
| 7431 | S8_109057808 | 8 | 109057808 | 2 | T | C | C | 0.6129 | T | 0.3871 | 14 | 0.45161 |
| 7432 | S8_109057823 | 8 | 109057823 | 15 | C | T | T | 0.62903 | C | 0.37097 | 13 | 0.41935 |
| 7433 | S8_109057862 | 8 | 109057862 | 39 | T | A | T | 0.77419 | A | 0.22581 | 8 | 0.25806 |
| 7434 | S8_109057892 | 8 | 109057892 | 30 | G | A | G | 0.95161 | A | 0.04839 | 3 | 0.09677 |
| 7435 | S8_109319192 | 8 | 109319192 | 261300 | G | A | G | 0.90323 | A | 0.09677 | 6 | 0.19355 |
| 7436 | S8_109545739 | 8 | 109545739 | 226547 | A | G | A | 0.93548 | G | 0.06452 | 4 | 0.12903 |
| 7437 | S8_109545748 | 8 | 109545748 | 9 | C | T | T | 0.66129 | C | 0.33871 | 11 | 0.35484 |
| 7438 | S8_109545857 | 8 | 109545857 | 109 | G | T | G | 0.95161 | T | 0.04839 | 3 | 0.09677 |
| 7439 | S8_110022033 | 8 | 110022033 | 476176 | C | T | C | 0.91935 | T | 0.08065 | 5 | 0.16129 |
| 7440 | S8_110022091 | 8 | 110022091 | 58 | C | T | C | 0.77419 | T | 0.22581 | 8 | 0.25806 |
| 7441 | S8_110194470 | 8 | 110194470 | 172379 | G | A | G | 0.6129 | A | 0.3871 | 12 | 0.3871 |
| 7442 | S8_110707405 | 8 | 110707405 | 512935 | A | T | A | 0.93548 | T | 0.06452 | 4 | 0.12903 |
| 7443 | S8_110792175 | 8 | 110792175 | 84770 | C | T | C | 0.93548 | T | 0.06452 | 4 | 0.12903 |
| 7444 | S8_110792241 | 8 | 110792241 | 66 | G | T | G | 0.93548 | T | 0.06452 | 4 | 0.12903 |
| 7445 | S8_110970104 | 8 | 110970104 | 177863 | G | A | G | 0.93548 | A | 0.06452 | 4 | 0.12903 |
| 7446 | S8_111038190 | 8 | 111038190 | 68086 | T | C | T | 0.72581 | C | 0.27419 | 11 | 0.35484 |
| 7447 | S8_111038205 | 8 | 111038205 | 15 | C | G | G | 0.53226 | C | 0.46774 | 15 | 0.48387 |
| 7448 | S8_111038214 | 8 | 111038214 | 9 | G | A | A | 0.53226 | G | 0.46774 | 15 | 0.48387 |
| 7449 | S8_111038310 | 8 | 111038310 | 96 | A | G | G | 0.72581 | A | 0.27419 | 13 | 0.41935 |
| 7450 | S8_111598049 | 8 | 111598049 | 559739 | G | C | G | 0.67742 | C | 0.32258 | 14 | 0.45161 |

| Site number | SNP name | Chromosome | Physical position | Physical distance from the previous SNP | Reference allele | Alternative allele | Major allele | Major allele frequency | Minor allele | Minor allele frequency | Number heterozygous | Heterozygosity |
|---|---|---|---|---|---|---|---|---|---|---|---|---|
| 7451 | S8_111598189 | 8 | 111598189 | 140 | C | T | C | 0.51613 | T | 0.48387 | 10 | 0.32258 |
| 7452 | S8_111682055 | 8 | 111682055 | 83866 | T | C | T | 0.79032 | C | 0.20968 | 11 | 0.35484 |
| 7453 | S8_111682208 | 8 | 111682208 | 153 | A | G | A | 0.79032 | G | 0.20968 | 11 | 0.35484 |
| 7454 | S8_111803829 | 8 | 111803829 | 121621 | T | C | T | 0.74194 | C | 0.25806 | 12 | 0.3871 |
| 7455 | S8_111827239 | 8 | 111827239 | 23410 | T | C | T | 0.53226 | C | 0.46774 | 19 | 0.6129 |
| 7456 | S8_111827433 | 8 | 111827433 | 194 | G | A | G | 0.93548 | A | 0.06452 | 4 | 0.12903 |
| 7457 | S8_112569438 | 8 | 112569438 | 742005 | G | A | G | 0.93548 | A | 0.06452 | 4 | 0.12903 |
| 7458 | S9_152229 | 9 | 152229 | 0 | C | T | C | 0.79032 | T | 0.20968 | 9 | 0.29032 |
| 7459 | S9_422091 | 9 | 422091 | 269862 | T | C | T | 0.91935 | C | 0.08065 | 5 | 0.16129 |
| 7460 | S9_590978 | 9 | 590978 | 168887 | G | A | G | 0.79032 | A | 0.20968 | 11 | 0.35484 |
| 7461 | S9_591095 | 9 | 591095 | 117 | G | A | A | 0.53226 | G | 0.46774 | 19 | 0.6129 |
| 7462 | S9_591108 | 9 | 591108 | 13 | G | C | C | 0.53226 | G | 0.46774 | 19 | 0.6129 |
| 7463 | S9_808463 | 9 | 808463 | 217355 | A | G | G | 0.95161 | A | 0.04839 | 3 | 0.09677 |
| 7464 | S9_1051421 | 9 | 1051421 | 242958 | T | C | C | 0.6129 | T | 0.3871 | 12 | 0.3871 |
| 7465 | S9_1051582 | 9 | 1051582 | 161 | T | A | T | 0.59677 | A | 0.40323 | 15 | 0.48387 |
| 7466 | S9_1124087 | 9 | 1124087 | 72505 | G | A | G | 0.90323 | A | 0.09677 | 6 | 0.19355 |
| 7467 | S9_1124284 | 9 | 1124284 | 197 | C | T | C | 0.77419 | T | 0.22581 | 14 | 0.45161 |
| 7468 | S9_1844425 | 9 | 1844425 | 720141 | G | A | G | 0.75806 | A | 0.24194 | 15 | 0.48387 |
| 7469 | S9_1844578 | 9 | 1844578 | 153 | G | T | G | 0.90323 | T | 0.09677 | 6 | 0.19355 |
| 7470 | S9_1870662 | 9 | 1870662 | 26084 | G | A | G | 0.82258 | A | 0.17742 | 7 | 0.22581 |
| 7471 | S9_1932658 | 9 | 1932658 | 61996 | G | A | G | 0.80645 | A | 0.19355 | 10 | 0.32258 |
| 7472 | S9_1958147 | 9 | 1958147 | 25489 | G | C | G | 0.62903 | C | 0.37097 | 13 | 0.41935 |
| 7473 | S9_1958252 | 9 | 1958252 | 105 | G | A | G | 0.62903 | A | 0.37097 | 13 | 0.41935 |
| 7474 | S9_2005734 | 9 | 2005734 | 47482 | C | A | C | 0.90323 | A | 0.09677 | 6 | 0.19355 |
| 7475 | S9_2086207 | 9 | 2086207 | 80473 | C | G | G | 0.80645 | C | 0.19355 | 12 | 0.3871 |
| 7476 | S9_2093064 | 9 | 2093064 | 6857 | C | T | C | 0.87097 | T | 0.12903 | 4 | 0.12903 |
| 7477 | S9_2093123 | 9 | 2093123 | 59 | C | T | C | 0.6129 | T | 0.3871 | 14 | 0.45161 |
| 7478 | S9_2093152 | 9 | 2093152 | 29 | A | G | A | 0.83871 | G | 0.16129 | 6 | 0.19355 |
| 7479 | S9_2093201 | 9 | 2093201 | 49 | C | T | C | 0.6129 | T | 0.3871 | 12 | 0.3871 |
| 7480 | S9_2631491 | 9 | 2631491 | 538290 | A | G | A | 0.66129 | G | 0.33871 | 13 | 0.41935 |
| 7481 | S9_2631505 | 9 | 2631505 | 14 | T | G | T | 0.66129 | G | 0.33871 | 13 | 0.41935 |
| 7482 | S9_2631506 | 9 | 2631506 | 1 | A | G | A | 0.66129 | G | 0.33871 | 13 | 0.41935 |
| 7483 | S9_2631514 | 9 | 2631514 | 8 | A | T | A | 0.53226 | T | 0.46774 | 13 | 0.41935 |
| 7484 | S9_2631533 | 9 | 2631533 | 19 | A | G | A | 0.66129 | G | 0.33871 | 13 | 0.41935 |
| 7485 | S9_2631578 | 9 | 2631578 | 45 | T | A | T | 0.69355 | A | 0.30645 | 15 | 0.48387 |
| 7486 | S9_2631629 | 9 | 2631629 | 51 | T | G | T | 0.87097 | G | 0.12903 | 4 | 0.12903 |
| 7487 | S9_2631634 | 9 | 2631634 | 5 | G | A | G | 0.66129 | A | 0.33871 | 13 | 0.41935 |
| 7488 | S9_3434102 | 9 | 3434102 | 802468 | G | T | G | 0.83871 | T | 0.16129 | 8 | 0.25806 |
| 7489 | S9_3434323 | 9 | 3434323 | 221 | T | C | T | 0.67742 | C | 0.32258 | 14 | 0.45161 |
| 7490 | S9_3499881 | 9 | 3499881 | 65558 | T | C | T | 0.70968 | C | 0.29032 | 10 | 0.32258 |
| 7491 | S9_3499882 | 9 | 3499882 | 1 | A | G | A | 0.95161 | G | 0.04839 | 3 | 0.09677 |
| 7492 | S9_3499944 | 9 | 3499944 | 62 | G | C | G | 0.95161 | C | 0.04839 | 3 | 0.09677 |
| 7493 | S9_3499995 | 9 | 3499995 | 51 | T | G | T | 0.95161 | G | 0.04839 | 3 | 0.09677 |
| 7494 | S9_3500012 | 9 | 3500012 | 17 | C | T | C | 0.95161 | T | 0.04839 | 3 | 0.09677 |
| 7495 | S9_3837763 | 9 | 3837763 | 337751 | A | G | A | 0.82258 | G | 0.17742 | 7 | 0.22581 |
| 7496 | S9_3837792 | 9 | 3837792 | 29 | G | A | G | 0.82258 | A | 0.17742 | 7 | 0.22581 |
| 7497 | S9_3837821 | 9 | 3837821 | 29 | C | T | C | 0.93548 | T | 0.06452 | 4 | 0.12903 |
| 7498 | S9_3837835 | 9 | 3837835 | 14 | A | G | G | 0.79032 | A | 0.20968 | 11 | 0.35484 |
| 7499 | S9_3837862 | 9 | 3837862 | 27 | G | A | G | 0.95161 | A | 0.04839 | 3 | 0.09677 |
| 7500 | S9_4841664 | 9 | 4841664 | 1003802 | G | A | G | 0.87097 | A | 0.12903 | 8 | 0.25806 |
| 7501 | S9_4841728 | 9 | 4841728 | 64 | T | G | T | 0.77419 | G | 0.22581 | 14 | 0.45161 |
| 7502 | S9_4841796 | 9 | 4841796 | 68 | T | C | T | 0.77419 | C | 0.22581 | 12 | 0.3871 |
| 7503 | S9_5063893 | 9 | 5063893 | 222097 | C | A | C | 0.93548 | A | 0.06452 | 4 | 0.12903 |
| 7504 | S9_5064053 | 9 | 5064053 | 160 | T | A | T | 0.93548 | A | 0.06452 | 4 | 0.12903 |

| Site number | SNP name | Chromosome | Physical position | Physical distance from the previous SNP | Reference allele | Alternative allele | Major allele | Major allele frequency | Minor allele | Minor allele frequency | Number heterozygous | Heterozygosity |
|---|---|---|---|---|---|---|---|---|---|---|---|---|
| 7505 | S9_5064115 | 9 | 5064115 | 62 | T | C | T | 0.93548 | C | 0.06452 | 4 | 0.12903 |
| 7506 | S9_5064131 | 9 | 5064131 | 16 | T | C | C | 0.8871 | T | 0.1129 | 7 | 0.22581 |
| 7507 | S9_5408996 | 9 | 5408996 | 344865 | C | G | C | 0.72581 | G | 0.27419 | 13 | 0.41935 |
| 7508 | S9_5409132 | 9 | 5409132 | 136 | C | T | C | 0.91935 | T | 0.08065 | 5 | 0.16129 |
| 7509 | S9_5724823 | 9 | 5724823 | 315691 | G | C | G | 0.91935 | C | 0.08065 | 5 | 0.16129 |
| 7510 | S9_5724959 | 9 | 5724959 | 136 | A | G | G | 0.82258 | A | 0.17742 | 11 | 0.35484 |
| 7511 | S9_6088047 | 9 | 6088047 | 363088 | A | C | A | 0.91935 | C | 0.08065 | 5 | 0.16129 |
| 7512 | S9_6180720 | 9 | 6180720 | 92673 | A | G | G | 0.83871 | A | 0.16129 | 10 | 0.32258 |
| 7513 | S9_6180754 | 9 | 6180754 | 34 | T | C | T | 0.93548 | C | 0.06452 | 4 | 0.12903 |
| 7514 | S9_6218441 | 9 | 6218441 | 37687 | A | T | A | 0.72581 | T | 0.27419 | 13 | 0.41935 |
| 7515 | S9_6218449 | 9 | 6218449 | 8 | G | A | G | 0.72581 | A | 0.27419 | 13 | 0.41935 |
| 7516 | S9_6218472 | 9 | 6218472 | 23 | A | G | A | 0.72581 | G | 0.27419 | 13 | 0.41935 |
| 7517 | S9_6355654 | 9 | 6355654 | 137182 | G | A | G | 0.87097 | A | 0.12903 | 8 | 0.25806 |
| 7518 | S9_6460679 | 9 | 6460679 | 105025 | T | G | T | 0.58065 | G | 0.41935 | 12 | 0.3871 |
| 7519 | S9_6460704 | 9 | 6460704 | 25 | T | C | T | 0.87097 | C | 0.12903 | 8 | 0.25806 |
| 7520 | S9_6460754 | 9 | 6460754 | 50 | G | A | G | 0.56452 | A | 0.43548 | 13 | 0.41935 |
| 7521 | S9_6471374 | 9 | 6471374 | 10620 | T | C | T | 0.58065 | C | 0.41935 | 12 | 0.3871 |
| 7522 | S9_6471390 | 9 | 6471390 | 16 | C | G | C | 0.91935 | G | 0.08065 | 5 | 0.16129 |
| 7523 | S9_6471457 | 9 | 6471457 | 67 | C | T | C | 0.91935 | T | 0.08065 | 3 | 0.09677 |
| 7524 | S9_6471494 | 9 | 6471494 | 37 | A | G | A | 0.58065 | G | 0.41935 | 12 | 0.3871 |
| 7525 | S9_6678254 | 9 | 6678254 | 206760 | G | C | G | 0.95161 | C | 0.04839 | 3 | 0.09677 |
| 7526 | S9_6678272 | 9 | 6678272 | 18 | G | A | G | 0.93548 | A | 0.06452 | 4 | 0.12903 |
| 7527 | S9_6807451 | 9 | 6807451 | 129179 | T | C | C | 0.62903 | T | 0.37097 | 17 | 0.54839 |
| 7528 | S9_6807452 | 9 | 6807452 | 1 | C | T | T | 0.62903 | C | 0.37097 | 17 | 0.54839 |
| 7529 | S9_6807455 | 9 | 6807455 | 3 | C | T | C | 0.95161 | T | 0.04839 | 3 | 0.09677 |
| 7530 | S9_6807493 | 9 | 6807493 | 38 | T | C | T | 0.70968 | C | 0.29032 | 16 | 0.51613 |
| 7531 | S9_6807508 | 9 | 6807508 | 15 | A | C | A | 0.72581 | C | 0.27419 | 13 | 0.41935 |
| 7532 | S9_6835393 | 9 | 6835393 | 27885 | A | G | A | 0.67742 | G | 0.32258 | 14 | 0.45161 |
| 7533 | S9_6835408 | 9 | 6835408 | 15 | C | T | C | 0.72581 | T | 0.27419 | 11 | 0.35484 |
| 7534 | S9_6846603 | 9 | 6846603 | 11195 | T | C | C | 0.79032 | T | 0.20968 | 9 | 0.29032 |
| 7535 | S9_7084765 | 9 | 7084765 | 238162 | G | A | G | 0.83871 | A | 0.16129 | 8 | 0.25806 |
| 7536 | S9_7084808 | 9 | 7084808 | 43 | G | A | G | 0.91935 | A | 0.08065 | 5 | 0.16129 |
| 7537 | S9_7084875 | 9 | 7084875 | 67 | C | T | C | 0.83871 | T | 0.16129 | 10 | 0.32258 |
| 7538 | S9_7302817 | 9 | 7302817 | 217942 | C | T | C | 0.90323 | T | 0.09677 | 6 | 0.19355 |
| 7539 | S9_8618670 | 9 | 8618670 | 1315853 | G | C | C | 0.72581 | G | 0.27419 | 9 | 0.29032 |
| 7540 | S9_8994056 | 9 | 8994056 | 375386 | C | T | C | 0.93548 | T | 0.06452 | 4 | 0.12903 |
| 7541 | S9_9004303 | 9 | 9004303 | 10247 | A | G | A | 0.91935 | G | 0.08065 | 5 | 0.16129 |
| 7542 | S9_9284622 | 9 | 9284622 | 280319 | C | T | C | 0.79032 | T | 0.20968 | 11 | 0.35484 |
| 7543 | S9_9513065 | 9 | 9513065 | 228443 | G | A | G | 0.75806 | A | 0.24194 | 11 | 0.35484 |
| 7544 | S9_9579694 | 9 | 9579694 | 66629 | T | C | T | 0.64516 | C | 0.35484 | 14 | 0.45161 |
| 7545 | S9_9579761 | 9 | 9579761 | 67 | G | A | G | 0.93548 | A | 0.06452 | 4 | 0.12903 |
| 7546 | S9_9579765 | 9 | 9579765 | 4 | T | A | T | 0.91935 | A | 0.08065 | 5 | 0.16129 |
| 7547 | S9_9602658 | 9 | 9602658 | 22893 | T | G | T | 0.79032 | G | 0.20968 | 7 | 0.22581 |
| 7548 | S9_9602691 | 9 | 9602691 | 33 | C | T | C | 0.79032 | T | 0.20968 | 7 | 0.22581 |
| 7549 | S9_9602719 | 9 | 9602719 | 28 | G | A | G | 0.6129 | A | 0.3871 | 14 | 0.45161 |
| 7550 | S9_9602786 | 9 | 9602786 | 67 | G | A | G | 0.79032 | A | 0.20968 | 7 | 0.22581 |
| 7551 | S9_9602790 | 9 | 9602790 | 4 | G | T | G | 0.79032 | T | 0.20968 | 7 | 0.22581 |
| 7552 | S9_9602800 | 9 | 9602800 | 10 | T | A | T | 0.79032 | A | 0.20968 | 7 | 0.22581 |
| 7553 | S9_9602822 | 9 | 9602822 | 22 | G | A | G | 0.91935 | A | 0.08065 | 3 | 0.09677 |
| 7554 | S9_9966143 | 9 | 9966143 | 363321 | T | C | T | 0.77419 | C | 0.22581 | 12 | 0.3871 |
| 7555 | S9_9966212 | 9 | 9966212 | 69 | C | T | C | 0.91935 | T | 0.08065 | 3 | 0.09677 |
| 7556 | S9_9966311 | 9 | 9966311 | 99 | C | T | C | 0.85484 | T | 0.14516 | 7 | 0.22581 |
| 7557 | S9_10194778 | 9 | 10194778 | 228467 | C | T | C | 0.74194 | T | 0.25806 | 12 | 0.3871 |
| 7558 | S9_10194814 | 9 | 10194814 | 36 | G | A | G | 0.85484 | A | 0.14516 | 5 | 0.16129 |

| Site number | SNP name | Chromosome | Physical position | Physical distance from the previous SNP | Reference allele | Alternative allele | Major allele | Major allele frequency | Minor allele | Minor allele frequency | Number heterozygous | Heterozygosity |
|---|---|---|---|---|---|---|---|---|---|---|---|---|
| 7559 | S9_10194884 | 9 | 10194884 | 70 | C | A | A | 0.82258 | C | 0.17742 | 7 | 0.22581 |
| 7560 | S9_10219153 | 9 | 10219153 | 24269 | A | C | A | 0.90323 | C | 0.09677 | 6 | 0.19355 |
| 7561 | S9_10219170 | 9 | 10219170 | 17 | A | C | A | 0.59677 | C | 0.40323 | 11 | 0.35484 |
| 7562 | S9_10219267 | 9 | 10219267 | 97 | C | T | C | 0.67742 | T | 0.32258 | 16 | 0.51613 |
| 7563 | S9_10274356 | 9 | 10274356 | 55089 | A | G | A | 0.82258 | G | 0.17742 | 7 | 0.22581 |
| 7564 | S9_10274500 | 9 | 10274500 | 144 | C | G | G | 0.58065 | C | 0.41935 | 12 | 0.3871 |
| 7565 | S9_10274539 | 9 | 10274539 | 39 | T | C | T | 0.8871 | C | 0.1129 | 7 | 0.22581 |
| 7566 | S9_10360796 | 9 | 10360796 | 86257 | G | A | G | 0.67742 | A | 0.32258 | 12 | 0.3871 |
| 7567 | S9_10420053 | 9 | 10420053 | 59257 | C | T | T | 0.53226 | C | 0.46774 | 11 | 0.35484 |
| 7568 | S9_10420074 | 9 | 10420074 | 21 | C | T | C | 0.77419 | T | 0.22581 | 8 | 0.25806 |
| 7569 | S9_10631763 | 9 | 10631763 | 211689 | C | T | C | 0.75806 | T | 0.24194 | 11 | 0.35484 |
| 7570 | S9_10631875 | 9 | 10631875 | 112 | G | A | G | 0.8871 | A | 0.1129 | 5 | 0.16129 |
| 7571 | S9_10661442 | 9 | 10661442 | 29567 | C | T | C | 0.80645 | T | 0.19355 | 8 | 0.25806 |
| 7572 | S9_10661554 | 9 | 10661554 | 112 | G | A | A | 0.93548 | G | 0.06452 | 4 | 0.12903 |
| 7573 | S9_10661566 | 9 | 10661566 | 12 | G | A | G | 0.93548 | A | 0.06452 | 4 | 0.12903 |
| 7574 | S9_10661570 | 9 | 10661570 | 4 | A | G | A | 0.74194 | G | 0.25806 | 10 | 0.32258 |
| 7575 | S9_11006851 | 9 | 11006851 | 345281 | T | C | T | 0.87097 | C | 0.12903 | 8 | 0.25806 |
| 7576 | S9_11006906 | 9 | 11006906 | 55 | A | G | A | 0.74194 | G | 0.25806 | 12 | 0.3871 |
| 7577 | S9_11102259 | 9 | 11102259 | 95353 | G | A | G | 0.6129 | A | 0.3871 | 14 | 0.45161 |
| 7578 | S9_11362282 | 9 | 11362282 | 260023 | T | C | T | 0.72581 | C | 0.27419 | 11 | 0.35484 |
| 7579 | S9_11379500 | 9 | 11379500 | 17218 | T | C | T | 0.8871 | C | 0.1129 | 7 | 0.22581 |
| 7580 | S9_12275252 | 9 | 12275252 | 895752 | G | A | G | 0.62903 | A | 0.37097 | 13 | 0.41935 |
| 7581 | S9_12275365 | 9 | 12275365 | 113 | G | A | G | 0.93548 | A | 0.06452 | 4 | 0.12903 |
| 7582 | S9_12275393 | 9 | 12275393 | 28 | A | G | A | 0.62903 | G | 0.37097 | 13 | 0.41935 |
| 7583 | S9_12571092 | 9 | 12571092 | 295699 | C | T | C | 0.59677 | T | 0.40323 | 11 | 0.35484 |
| 7584 | S9_12616785 | 9 | 12616785 | 45693 | C | T | T | 0.75806 | C | 0.24194 | 11 | 0.35484 |
| 7585 | S9_13011199 | 9 | 13011199 | 394414 | A | G | A | 0.74194 | G | 0.25806 | 14 | 0.45161 |
| 7586 | S9_13011285 | 9 | 13011285 | 86 | C | T | C | 0.95161 | T | 0.04839 | 3 | 0.09677 |
| 7587 | S9_13034355 | 9 | 13034355 | 23070 | T | C | T | 0.90323 | C | 0.09677 | 6 | 0.19355 |
| 7588 | S9_13189547 | 9 | 13189547 | 155192 | G | T | G | 0.75806 | T | 0.24194 | 9 | 0.29032 |
| 7589 | S9_13189638 | 9 | 13189638 | 91 | A | G | A | 0.83871 | G | 0.16129 | 8 | 0.25806 |
| 7590 | S9_13247320 | 9 | 13247320 | 57682 | C | T | C | 0.91935 | T | 0.08065 | 5 | 0.16129 |
| 7591 | S9_13493852 | 9 | 13493852 | 246532 | T | C | T | 0.95161 | C | 0.04839 | 3 | 0.09677 |
| 7592 | S9_13655862 | 9 | 13655862 | 162010 | C | G | C | 0.69355 | G | 0.30645 | 17 | 0.54839 |
| 7593 | S9_13907713 | 9 | 13907713 | 251851 | C | T | C | 0.91935 | T | 0.08065 | 5 | 0.16129 |
| 7594 | S9_13907714 | 9 | 13907714 | 1 | G | A | G | 0.95161 | A | 0.04839 | 3 | 0.09677 |
| 7595 | S9_13907737 | 9 | 13907737 | 23 | T | G | T | 0.77419 | G | 0.22581 | 12 | 0.3871 |
| 7596 | S9_13907811 | 9 | 13907811 | 74 | G | C | G | 0.95161 | C | 0.04839 | 3 | 0.09677 |
| 7597 | S9_13907834 | 9 | 13907834 | 23 | A | C | A | 0.91935 | C | 0.08065 | 5 | 0.16129 |
| 7598 | S9_13907927 | 9 | 13907927 | 93 | A | G | A | 0.77419 | G | 0.22581 | 12 | 0.3871 |
| 7599 | S9_14035656 | 9 | 14035656 | 127729 | A | T | T | 0.64516 | A | 0.35484 | 12 | 0.3871 |
| 7600 | S9_14035694 | 9 | 14035694 | 38 | C | T | C | 0.83871 | T | 0.16129 | 8 | 0.25806 |
| 7601 | S9_14035717 | 9 | 14035717 | 23 | C | T | C | 0.59677 | T | 0.40323 | 13 | 0.41935 |
| 7602 | S9_14035752 | 9 | 14035752 | 35 | C | G | C | 0.8871 | G | 0.1129 | 5 | 0.16129 |
| 7603 | S9_14426431 | 9 | 14426431 | 390679 | T | C | T | 0.93548 | C | 0.06452 | 4 | 0.12903 |
| 7604 | S9_14780260 | 9 | 14780260 | 353829 | G | A | G | 0.91935 | A | 0.08065 | 5 | 0.16129 |
| 7605 | S9_14780285 | 9 | 14780285 | 25 | G | A | G | 0.75806 | A | 0.24194 | 9 | 0.29032 |
| 7606 | S9_14780287 | 9 | 14780287 | 2 | T | C | T | 0.93548 | C | 0.06452 | 4 | 0.12903 |
| 7607 | S9_14780357 | 9 | 14780357 | 70 | A | G | A | 0.77419 | G | 0.22581 | 8 | 0.25806 |
| 7608 | S9_15190952 | 9 | 15190952 | 410595 | A | G | G | 0.83871 | A | 0.16129 | 8 | 0.25806 |
| 7609 | S9_15264589 | 9 | 15264589 | 73637 | A | G | G | 0.67742 | A | 0.32258 | 14 | 0.45161 |
| 7610 | S9_15264607 | 9 | 15264607 | 18 | G | A | G | 0.91935 | A | 0.08065 | 5 | 0.16129 |
| 7611 | S9_15353216 | 9 | 15353216 | 88609 | A | G | A | 0.54839 | G | 0.45161 | 14 | 0.45161 |
| 7612 | S9_17067095 | 9 | 17067095 | 1713879 | G | C | G | 0.91935 | C | 0.08065 | 5 | 0.16129 |

| Site number | SNP name | Chromosome | Physical position | Physical distance from the previous SNP | Reference allele | Alternative allele | Major allele | Major allele frequency | Minor allele | Minor allele frequency | Number heterozygous | Heterozygosity |
|---|---|---|---|---|---|---|---|---|---|---|---|---|
| 7613 | S9_17067096 | 9 | 17067096 | 1 | G | A | G | 0.91935 | A | 0.08065 | 5 | 0.16129 |
| 7614 | S9_17067118 | 9 | 17067118 | 22 | G | A | A | 0.90323 | G | 0.09677 | 4 | 0.12903 |
| 7615 | S9_17067317 | 9 | 17067317 | 199 | G | T | G | 0.91935 | T | 0.08065 | 5 | 0.16129 |
| 7616 | S9_17638519 | 9 | 17638519 | 571202 | G | A | G | 0.95161 | A | 0.04839 | 3 | 0.09677 |
| 7617 | S9_17638669 | 9 | 17638669 | 150 | C | T | T | 0.87097 | C | 0.12903 | 6 | 0.19355 |
| 7618 | S9_17638713 | 9 | 17638713 | 44 | A | G | A | 0.93548 | G | 0.06452 | 4 | 0.12903 |
| 7619 | S9_17638715 | 9 | 17638715 | 2 | G | C | G | 0.51613 | C | 0.48387 | 14 | 0.45161 |
| 7620 | S9_18219317 | 9 | 18219317 | 580602 | C | G | C | 0.59677 | G | 0.40323 | 15 | 0.48387 |
| 7621 | S9_18219334 | 9 | 18219334 | 17 | G | A | G | 0.59677 | A | 0.40323 | 15 | 0.48387 |
| 7622 | S9_18219369 | 9 | 18219369 | 35 | A | G | A | 0.59677 | G | 0.40323 | 15 | 0.48387 |
| 7623 | S9_18219389 | 9 | 18219389 | 20 | C | T | C | 0.95161 | T | 0.04839 | 3 | 0.09677 |
| 7624 | S9_18219390 | 9 | 18219390 | 1 | G | A | G | 0.59677 | A | 0.40323 | 15 | 0.48387 |
| 7625 | S9_18253759 | 9 | 18253759 | 34369 | C | A | C | 0.70968 | A | 0.29032 | 12 | 0.3871 |
| 7626 | S9_18253913 | 9 | 18253913 | 154 | T | C | T | 0.93548 | C | 0.06452 | 4 | 0.12903 |
| 7627 | S9_18422407 | 9 | 18422407 | 168494 | C | G | C | 0.74194 | G | 0.25806 | 10 | 0.32258 |
| 7628 | S9_18422507 | 9 | 18422507 | 100 | G | A | G | 0.66129 | A | 0.33871 | 11 | 0.35484 |
| 7629 | S9_18917454 | 9 | 18917454 | 494947 | C | G | C | 0.90323 | G | 0.09677 | 6 | 0.19355 |
| 7630 | S9_18917455 | 9 | 18917455 | 1 | T | C | T | 0.93548 | C | 0.06452 | 4 | 0.12903 |
| 7631 | S9_18917482 | 9 | 18917482 | 27 | G | A | G | 0.95161 | A | 0.04839 | 3 | 0.09677 |
| 7632 | S9_19482046 | 9 | 19482046 | 564564 | G | A | G | 0.83871 | A | 0.16129 | 6 | 0.19355 |
| 7633 | S9_19494694 | 9 | 19494694 | 12648 | G | A | G | 0.95161 | A | 0.04839 | 3 | 0.09677 |
| 7634 | S9_19494748 | 9 | 19494748 | 54 | A | G | G | 0.87097 | A | 0.12903 | 6 | 0.19355 |
| 7635 | S9_19494784 | 9 | 19494784 | 36 | C | T | T | 0.82258 | C | 0.17742 | 9 | 0.29032 |
| 7636 | S9_19494791 | 9 | 19494791 | 7 | C | T | T | 0.82258 | C | 0.17742 | 9 | 0.29032 |
| 7637 | S9_19494792 | 9 | 19494792 | 1 | A | G | G | 0.8871 | A | 0.1129 | 5 | 0.16129 |
| 7638 | S9_19494859 | 9 | 19494859 | 67 | G | A | G | 0.74194 | A | 0.25806 | 12 | 0.3871 |
| 7639 | S9_19494867 | 9 | 19494867 | 8 | G | A | A | 0.75806 | G | 0.24194 | 13 | 0.41935 |
| 7640 | S9_19494873 | 9 | 19494873 | 6 | G | A | A | 0.80645 | G | 0.19355 | 10 | 0.32258 |
| 7641 | S9_19494877 | 9 | 19494877 | 4 | C | T | T | 0.80645 | C | 0.19355 | 10 | 0.32258 |
| 7642 | S9_19494931 | 9 | 19494931 | 54 | A | C | A | 0.8871 | C | 0.1129 | 5 | 0.16129 |
| 7643 | S9_19601618 | 9 | 19601618 | 106687 | G | A | G | 0.95161 | A | 0.04839 | 3 | 0.09677 |
| 7644 | S9_19646540 | 9 | 19646540 | 44922 | C | T | T | 0.6129 | C | 0.3871 | 18 | 0.58065 |
| 7645 | S9_20242358 | 9 | 20242358 | 595818 | G | A | G | 0.8871 | A | 0.1129 | 7 | 0.22581 |
| 7646 | S9_20242388 | 9 | 20242388 | 30 | A | G | G | 0.85484 | A | 0.14516 | 9 | 0.29032 |
| 7647 | S9_20242425 | 9 | 20242425 | 37 | C | T | C | 0.8871 | T | 0.1129 | 7 | 0.22581 |
| 7648 | S9_20274088 | 9 | 20274088 | 31663 | T | C | C | 0.85484 | T | 0.14516 | 7 | 0.22581 |
| 7649 | S9_20274204 | 9 | 20274204 | 116 | A | C | A | 0.70968 | C | 0.29032 | 10 | 0.32258 |
| 7650 | S9_20274256 | 9 | 20274256 | 52 | C | A | A | 0.58065 | C | 0.41935 | 14 | 0.45161 |
| 7651 | S9_20312707 | 9 | 20312707 | 38451 | A | G | A | 0.82258 | G | 0.17742 | 11 | 0.35484 |
| 7652 | S9_20312761 | 9 | 20312761 | 54 | G | A | G | 0.64516 | A | 0.35484 | 12 | 0.3871 |
| 7653 | S9_20312766 | 9 | 20312766 | 5 | G | A | G | 0.95161 | A | 0.04839 | 3 | 0.09677 |
| 7654 | S9_20312813 | 9 | 20312813 | 47 | A | G | A | 0.95161 | G | 0.04839 | 3 | 0.09677 |
| 7655 | S9_20312873 | 9 | 20312873 | 60 | G | A | G | 0.95161 | A | 0.04839 | 3 | 0.09677 |
| 7656 | S9_20312893 | 9 | 20312893 | 20 | G | A | G | 0.82258 | A | 0.17742 | 11 | 0.35484 |
| 7657 | S9_21041514 | 9 | 21041514 | 728621 | G | C | C | 0.8871 | G | 0.1129 | 7 | 0.22581 |
| 7658 | S9_21042027 | 9 | 21042027 | 513 | T | C | C | 0.8871 | T | 0.1129 | 7 | 0.22581 |
| 7659 | S9_21114777 | 9 | 21114777 | 72750 | C | T | C | 0.91935 | T | 0.08065 | 3 | 0.09677 |
| 7660 | S9_21272248 | 9 | 21272248 | 157471 | C | G | C | 0.77419 | G | 0.22581 | 12 | 0.3871 |
| 7661 | S9_21272345 | 9 | 21272345 | 97 | A | T | A | 0.77419 | T | 0.22581 | 12 | 0.3871 |
| 7662 | S9_21272360 | 9 | 21272360 | 15 | C | T | C | 0.95161 | T | 0.04839 | 3 | 0.09677 |
| 7663 | S9_21649812 | 9 | 21649812 | 377452 | C | T | C | 0.93548 | T | 0.06452 | 4 | 0.12903 |
| 7664 | S9_21649835 | 9 | 21649835 | 23 | C | T | C | 0.87097 | T | 0.12903 | 8 | 0.25806 |
| 7665 | S9_21780742 | 9 | 21780742 | 130907 | A | G | A | 0.64516 | G | 0.35484 | 14 | 0.45161 |
| 7666 | S9_21780913 | 9 | 21780913 | 171 | C | T | C | 0.93548 | T | 0.06452 | 2 | 0.06452 |

| Site number | SNP name | Chromosome | Physical position | Physical distance from the previous SNP | Reference allele | Alternative allele | Major allele | Major allele frequency | Minor allele | Minor allele frequency | Number heterozygous | Heterozygosity |
|---|---|---|---|---|---|---|---|---|---|---|---|---|
| 7667 | S9_21852708 | 9 | 21852708 | 71795 | T | G | T | 0.93548 | G | 0.06452 | 4 | 0.12903 |
| 7668 | S9_21860021 | 9 | 21860021 | 7313 | C | T | C | 0.95161 | T | 0.04839 | 3 | 0.09677 |
| 7669 | S9_21860026 | 9 | 21860026 | 5 | G | A | G | 0.6129 | A | 0.3871 | 20 | 0.64516 |
| 7670 | S9_21894367 | 9 | 21894367 | 34341 | G | A | G | 0.93548 | A | 0.06452 | 4 | 0.12903 |
| 7671 | S9_21928477 | 9 | 21928477 | 34110 | C | T | C | 0.93548 | T | 0.06452 | 4 | 0.12903 |
| 7672 | S9_21928575 | 9 | 21928575 | 98 | T | C | T | 0.58065 | C | 0.41935 | 12 | 0.3871 |
| 7673 | S9_21928576 | 9 | 21928576 | 1 | G | A | G | 0.58065 | A | 0.41935 | 12 | 0.3871 |
| 7674 | S9_21928580 | 9 | 21928580 | 4 | A | C | A | 0.59677 | C | 0.40323 | 13 | 0.41935 |
| 7675 | S9_21928586 | 9 | 21928586 | 6 | G | A | G | 0.58065 | A | 0.41935 | 12 | 0.3871 |
| 7676 | S9_21928597 | 9 | 21928597 | 11 | C | T | C | 0.59677 | T | 0.40323 | 13 | 0.41935 |
| 7677 | S9_21928659 | 9 | 21928659 | 62 | G | C | G | 0.58065 | C | 0.41935 | 12 | 0.3871 |
| 7678 | S9_22291899 | 9 | 22291899 | 363240 | T | A | T | 0.93548 | A | 0.06452 | 4 | 0.12903 |
| 7679 | S9_22291921 | 9 | 22291921 | 22 | G | A | G | 0.91935 | A | 0.08065 | 5 | 0.16129 |
| 7680 | S9_22291930 | 9 | 22291930 | 9 | A | G | A | 0.93548 | G | 0.06452 | 4 | 0.12903 |
| 7681 | S9_22291937 | 9 | 22291937 | 7 | A | G | A | 0.93548 | G | 0.06452 | 4 | 0.12903 |
| 7682 | S9_22291964 | 9 | 22291964 | 27 | C | G | C | 0.95161 | G | 0.04839 | 3 | 0.09677 |
| 7683 | S9_23651890 | 9 | 23651890 | 1359926 | G | A | G | 0.80645 | A | 0.19355 | 10 | 0.32258 |
| 7684 | S9_23765195 | 9 | 23765195 | 113305 | G | T | T | 0.79032 | G | 0.20968 | 11 | 0.35484 |
| 7685 | S9_25578801 | 9 | 25578801 | 1813606 | C | T | C | 0.79032 | T | 0.20968 | 13 | 0.41935 |
| 7686 | S9_25782483 | 9 | 25782483 | 203682 | A | G | G | 0.74194 | A | 0.25806 | 12 | 0.3871 |
| 7687 | S9_25782659 | 9 | 25782659 | 176 | C | A | C | 0.54839 | A | 0.45161 | 18 | 0.58065 |
| 7688 | S9_25979214 | 9 | 25979214 | 196555 | G | A | G | 0.87097 | A | 0.12903 | 4 | 0.12903 |
| 7689 | S9_25979237 | 9 | 25979237 | 23 | G | A | G | 0.90323 | A | 0.09677 | 6 | 0.19355 |
| 7690 | S9_25979285 | 9 | 25979285 | 48 | C | G | C | 0.8871 | G | 0.1129 | 5 | 0.16129 |
| 7691 | S9_25979419 | 9 | 25979419 | 134 | T | C | C | 0.64516 | T | 0.35484 | 14 | 0.45161 |
| 7692 | S9_25979434 | 9 | 25979434 | 15 | A | G | A | 0.90323 | G | 0.09677 | 6 | 0.19355 |
| 7693 | S9_26602931 | 9 | 26602931 | 623497 | A | C | C | 0.83871 | A | 0.16129 | 8 | 0.25806 |
| 7694 | S9_26962047 | 9 | 26962047 | 359116 | C | T | C | 0.74194 | T | 0.25806 | 14 | 0.45161 |
| 7695 | S9_27292122 | 9 | 27292122 | 330075 | G | A | G | 0.90323 | A | 0.09677 | 4 | 0.12903 |
| 7696 | S9_27618257 | 9 | 27618257 | 326135 | A | G | G | 0.56452 | A | 0.43548 | 13 | 0.41935 |
| 7697 | S9_27632736 | 9 | 27632736 | 14479 | C | G | C | 0.90323 | G | 0.09677 | 4 | 0.12903 |
| 7698 | S9_28649697 | 9 | 28649697 | 1016961 | C | T | C | 0.66129 | T | 0.33871 | 11 | 0.35484 |
| 7699 | S9_28982099 | 9 | 28982099 | 332402 | T | C | C | 0.90323 | T | 0.09677 | 6 | 0.19355 |
| 7700 | S9_28982203 | 9 | 28982203 | 104 | T | C | C | 0.90323 | T | 0.09677 | 6 | 0.19355 |
| 7701 | S9_28982222 | 9 | 28982222 | 19 | C | T | C | 0.90323 | T | 0.09677 | 6 | 0.19355 |
| 7702 | S9_29211080 | 9 | 29211080 | 228858 | A | G | A | 0.64516 | G | 0.35484 | 10 | 0.32258 |
| 7703 | S9_29211153 | 9 | 29211153 | 73 | G | A | G | 0.87097 | A | 0.12903 | 4 | 0.12903 |
| 7704 | S9_29632777 | 9 | 29632777 | 421624 | T | C | T | 0.90323 | C | 0.09677 | 6 | 0.19355 |
| 7705 | S9_30254232 | 9 | 30254232 | 621455 | C | T | C | 0.93548 | T | 0.06452 | 4 | 0.12903 |
| 7706 | S9_30453825 | 9 | 30453825 | 199593 | C | T | C | 0.87097 | T | 0.12903 | 8 | 0.25806 |
| 7707 | S9_30697216 | 9 | 30697216 | 243391 | T | C | T | 0.85484 | C | 0.14516 | 9 | 0.29032 |
| 7708 | S9_30697270 | 9 | 30697270 | 54 | A | G | G | 0.6129 | A | 0.3871 | 12 | 0.3871 |
| 7709 | S9_31176605 | 9 | 31176605 | 479335 | G | A | G | 0.8871 | A | 0.1129 | 7 | 0.22581 |
| 7710 | S9_31420699 | 9 | 31420699 | 244094 | T | C | C | 0.82258 | T | 0.17742 | 7 | 0.22581 |
| 7711 | S9_32297082 | 9 | 32297082 | 876383 | C | G | C | 0.77419 | G | 0.22581 | 12 | 0.3871 |
| 7712 | S9_32297125 | 9 | 32297125 | 43 | C | T | C | 0.77419 | T | 0.22581 | 12 | 0.3871 |
| 7713 | S9_32390808 | 9 | 32390808 | 93683 | C | T | C | 0.80645 | T | 0.19355 | 10 | 0.32258 |
| 7714 | S9_32390833 | 9 | 32390833 | 25 | G | A | G | 0.79032 | A | 0.20968 | 13 | 0.41935 |
| 7715 | S9_32562317 | 9 | 32562317 | 171484 | A | T | T | 0.80645 | A | 0.19355 | 12 | 0.3871 |
| 7716 | S9_32761915 | 9 | 32761915 | 199598 | G | A | G | 0.75806 | A | 0.24194 | 11 | 0.35484 |
| 7717 | S9_33113578 | 9 | 33113578 | 351663 | A | C | A | 0.67742 | C | 0.32258 | 16 | 0.51613 |
| 7718 | S9_33113595 | 9 | 33113595 | 17 | T | G | T | 0.74194 | G | 0.25806 | 12 | 0.3871 |
| 7719 | S9_33113650 | 9 | 33113650 | 55 | T | C | T | 0.82258 | C | 0.17742 | 9 | 0.29032 |
| 7720 | S9_33113672 | 9 | 33113672 | 22 | T | C | T | 0.87097 | C | 0.12903 | 6 | 0.19355 |

| Site number | SNP name | Chromosome | Physical position | Physical distance from the previous SNP | Reference allele | Alternative allele | Major allele | Major allele frequency | Minor allele | Minor allele frequency | Number heterozygous | Heterozygosity |
|---|---|---|---|---|---|---|---|---|---|---|---|---|
| 7721 | S9_33113678 | 9 | 33113678 | 6 | C | T | T | 0.53226 | C | 0.46774 | 11 | 0.35484 |
| 7722 | S9_33113696 | 9 | 33113696 | 18 | T | C | T | 0.8871 | C | 0.1129 | 5 | 0.16129 |
| 7723 | S9_33113703 | 9 | 33113703 | 7 | T | C | T | 0.62903 | C | 0.37097 | 9 | 0.29032 |
| 7724 | S9_33113746 | 9 | 33113746 | 43 | G | A | G | 0.56452 | A | 0.43548 | 15 | 0.48387 |
| 7725 | S9_33692889 | 9 | 33692889 | 579143 | C | G | G | 0.54839 | C | 0.45161 | 20 | 0.64516 |
| 7726 | S9_33692962 | 9 | 33692962 | 73 | G | C | C | 0.54839 | G | 0.45161 | 20 | 0.64516 |
| 7727 | S9_33693004 | 9 | 33693004 | 42 | T | C | C | 0.56452 | T | 0.43548 | 19 | 0.6129 |
| 7728 | S9_33693068 | 9 | 33693068 | 64 | G | A | A | 0.58065 | G | 0.41935 | 20 | 0.64516 |
| 7729 | S9_35110031 | 9 | 35110031 | 1416963 | T | C | C | 0.85484 | T | 0.14516 | 9 | 0.29032 |
| 7730 | S9_35424046 | 9 | 35424046 | 314015 | C | T | C | 0.8871 | T | 0.1129 | 5 | 0.16129 |
| 7731 | S9_36162181 | 9 | 36162181 | 738135 | G | A | G | 0.70968 | A | 0.29032 | 12 | 0.3871 |
| 7732 | S9_36199269 | 9 | 36199269 | 37088 | T | C | C | 0.58065 | T | 0.41935 | 16 | 0.51613 |
| 7733 | S9_36199351 | 9 | 36199351 | 82 | A | G | A | 0.8871 | G | 0.1129 | 7 | 0.22581 |
| 7734 | S9_36713585 | 9 | 36713585 | 514234 | G | C | G | 0.80645 | C | 0.19355 | 12 | 0.3871 |
| 7735 | S9_36965639 | 9 | 36965639 | 252054 | T | C | T | 0.80645 | C | 0.19355 | 8 | 0.25806 |
| 7736 | S9_37051191 | 9 | 37051191 | 85552 | G | A | G | 0.91935 | A | 0.08065 | 5 | 0.16129 |
| 7737 | S9_37690300 | 9 | 37690300 | 639109 | C | T | C | 0.83871 | T | 0.16129 | 10 | 0.32258 |
| 7738 | S9_37773438 | 9 | 37773438 | 83138 | C | T | C | 0.95161 | T | 0.04839 | 3 | 0.09677 |
| 7739 | S9_37922597 | 9 | 37922597 | 149159 | C | G | C | 0.74194 | G | 0.25806 | 14 | 0.45161 |
| 7740 | S9_39332376 | 9 | 39332376 | 1409779 | C | T | T | 0.85484 | C | 0.14516 | 7 | 0.22581 |
| 7741 | S9_39841188 | 9 | 39841188 | 508812 | G | A | G | 0.70968 | A | 0.29032 | 14 | 0.45161 |
| 7742 | S9_39841244 | 9 | 39841244 | 56 | C | T | C | 0.74194 | T | 0.25806 | 8 | 0.25806 |
| 7743 | S9_40182880 | 9 | 40182880 | 341636 | T | C | T | 0.80645 | C | 0.19355 | 6 | 0.19355 |
| 7744 | S9_40182962 | 9 | 40182962 | 82 | G | A | G | 0.79032 | A | 0.20968 | 7 | 0.22581 |
| 7745 | S9_42310977 | 9 | 42310977 | 2128015 | G | T | T | 0.58065 | G | 0.41935 | 20 | 0.64516 |
| 7746 | S9_42311000 | 9 | 42311000 | 23 | C | T | T | 0.95161 | C | 0.04839 | 3 | 0.09677 |
| 7747 | S9_42311075 | 9 | 42311075 | 75 | T | C | C | 0.95161 | T | 0.04839 | 3 | 0.09677 |
| 7748 | S9_42311131 | 9 | 42311131 | 56 | T | C | T | 0.8871 | C | 0.1129 | 7 | 0.22581 |
| 7749 | S9_42510228 | 9 | 42510228 | 199097 | C | T | T | 0.79032 | C | 0.20968 | 11 | 0.35484 |
| 7750 | S9_42510306 | 9 | 42510306 | 78 | A | T | T | 0.79032 | A | 0.20968 | 11 | 0.35484 |
| 7751 | S9_42510322 | 9 | 42510322 | 16 | A | G | G | 0.79032 | A | 0.20968 | 11 | 0.35484 |
| 7752 | S9_42510375 | 9 | 42510375 | 53 | G | T | G | 0.93548 | T | 0.06452 | 4 | 0.12903 |
| 7753 | S9_42510435 | 9 | 42510435 | 60 | T | C | C | 0.8871 | T | 0.1129 | 5 | 0.16129 |
| 7754 | S9_42510442 | 9 | 42510442 | 7 | C | T | T | 0.79032 | C | 0.20968 | 11 | 0.35484 |
| 7755 | S9_43564531 | 9 | 43564531 | 1054089 | A | C | A | 0.90323 | C | 0.09677 | 6 | 0.19355 |
| 7756 | S9_43564622 | 9 | 43564622 | 91 | T | C | C | 0.53226 | T | 0.46774 | 17 | 0.54839 |
| 7757 | S9_44780500 | 9 | 44780500 | 1215878 | T | A | T | 0.66129 | A | 0.33871 | 11 | 0.35484 |
| 7758 | S9_45365341 | 9 | 45365341 | 584841 | T | C | T | 0.91935 | C | 0.08065 | 3 | 0.09677 |
| 7759 | S9_45365350 | 9 | 45365350 | 9 | G | A | G | 0.82258 | A | 0.17742 | 11 | 0.35484 |
| 7760 | S9_45365403 | 9 | 45365403 | 53 | A | G | A | 0.95161 | G | 0.04839 | 3 | 0.09677 |
| 7761 | S9_45365407 | 9 | 45365407 | 4 | G | A | G | 0.95161 | A | 0.04839 | 3 | 0.09677 |
| 7762 | S9_45365456 | 9 | 45365456 | 49 | G | A | G | 0.6129 | A | 0.3871 | 16 | 0.51613 |
| 7763 | S9_45365524 | 9 | 45365524 | 68 | C | T | C | 0.77419 | T | 0.22581 | 10 | 0.32258 |
| 7764 | S9_45621966 | 9 | 45621966 | 256442 | T | G | T | 0.69355 | G | 0.30645 | 9 | 0.29032 |
| 7765 | S9_45622033 | 9 | 45622033 | 67 | C | G | C | 0.69355 | G | 0.30645 | 9 | 0.29032 |
| 7766 | S9_45629764 | 9 | 45629764 | 7731 | G | A | G | 0.93548 | A | 0.06452 | 4 | 0.12903 |
| 7767 | S9_45629882 | 9 | 45629882 | 118 | C | A | C | 0.79032 | A | 0.20968 | 9 | 0.29032 |
| 7768 | S9_46389260 | 9 | 46389260 | 759378 | A | C | A | 0.51613 | C | 0.48387 | 18 | 0.58065 |
| 7769 | S9_46389478 | 9 | 46389478 | 218 | A | G | A | 0.77419 | G | 0.22581 | 10 | 0.32258 |
| 7770 | S9_46398712 | 9 | 46398712 | 9234 | G | A | G | 0.8871 | A | 0.1129 | 7 | 0.22581 |
| 7771 | S9_46895743 | 9 | 46895743 | 497031 | C | A | C | 0.77419 | A | 0.22581 | 12 | 0.3871 |
| 7772 | S9_47428550 | 9 | 47428550 | 532807 | C | A | C | 0.58065 | A | 0.41935 | 16 | 0.51613 |
| 7773 | S9_47428650 | 9 | 47428650 | 100 | G | A | G | 0.77419 | A | 0.22581 | 8 | 0.25806 |
| 7774 | S9_47596813 | 9 | 47596813 | 168163 | G | A | G | 0.80645 | A | 0.19355 | 10 | 0.32258 |

| Site number | SNP name | Chromosome | Physical position | Physical distance from the previous SNP | Reference allele | Alternative allele | Major allele | Major allele frequency | Minor allele | Minor allele frequency | Number heterozygous | Heterozygosity |
|---|---|---|---|---|---|---|---|---|---|---|---|---|
| 7775 | S9_47596850 | 9 | 47596850 | 37 | T | G | T | 0.53226 | G | 0.46774 | 15 | 0.48387 |
| 7776 | S9_47596891 | 9 | 47596891 | 41 | C | T | C | 0.90323 | T | 0.09677 | 6 | 0.19355 |
| 7777 | S9_47596892 | 9 | 47596892 | 1 | G | A | G | 0.8871 | A | 0.1129 | 7 | 0.22581 |
| 7778 | S9_47720786 | 9 | 47720786 | 123894 | A | G | G | 0.62903 | A | 0.37097 | 17 | 0.54839 |
| 7779 | S9_47747290 | 9 | 47747290 | 26504 | G | A | G | 0.90323 | A | 0.09677 | 6 | 0.19355 |
| 7780 | S9_47809296 | 9 | 47809296 | 62006 | G | A | G | 0.90323 | A | 0.09677 | 6 | 0.19355 |
| 7781 | S9_47809330 | 9 | 47809330 | 34 | T | C | T | 0.85484 | C | 0.14516 | 9 | 0.29032 |
| 7782 | S9_47958081 | 9 | 47958081 | 148751 | T | A | T | 0.62903 | A | 0.37097 | 15 | 0.48387 |
| 7783 | S9_47958147 | 9 | 47958147 | 66 | T | C | C | 0.59677 | T | 0.40323 | 15 | 0.48387 |
| 7784 | S9_47997244 | 9 | 47997244 | 39097 | T | C | C | 0.72581 | T | 0.27419 | 15 | 0.48387 |
| 7785 | S9_47997255 | 9 | 47997255 | 11 | G | A | A | 0.72581 | G | 0.27419 | 15 | 0.48387 |
| 7786 | S9_48525211 | 9 | 48525211 | 527956 | G | A | G | 0.91935 | A | 0.08065 | 5 | 0.16129 |
| 7787 | S9_48525214 | 9 | 48525214 | 3 | T | C | T | 0.90323 | C | 0.09677 | 6 | 0.19355 |
| 7788 | S9_49631669 | 9 | 49631669 | 1106455 | T | A | A | 0.85484 | T | 0.14516 | 7 | 0.22581 |
| 7789 | S9_49649620 | 9 | 49649620 | 17951 | G | A | G | 0.75806 | A | 0.24194 | 11 | 0.35484 |
| 7790 | S9_49779250 | 9 | 49779250 | 129630 | G | A | G | 0.85484 | A | 0.14516 | 7 | 0.22581 |
| 7791 | S9_49804021 | 9 | 49804021 | 24771 | C | G | C | 0.8871 | G | 0.1129 | 5 | 0.16129 |
| 7792 | S9_49857327 | 9 | 49857327 | 53306 | T | C | C | 0.90323 | T | 0.09677 | 6 | 0.19355 |
| 7793 | S9_50026813 | 9 | 50026813 | 169486 | C | T | C | 0.8871 | T | 0.1129 | 5 | 0.16129 |
| 7794 | S9_50026856 | 9 | 50026856 | 43 | G | A | G | 0.93548 | A | 0.06452 | 4 | 0.12903 |
| 7795 | S9_50026985 | 9 | 50026985 | 129 | A | G | A | 0.90323 | G | 0.09677 | 6 | 0.19355 |
| 7796 | S9_50026992 | 9 | 50026992 | 7 | A | G | A | 0.95161 | G | 0.04839 | 3 | 0.09677 |
| 7797 | S9_50388556 | 9 | 50388556 | 361564 | C | T | C | 0.70968 | T | 0.29032 | 14 | 0.45161 |
| 7798 | S9_50388571 | 9 | 50388571 | 15 | C | G | C | 0.90323 | G | 0.09677 | 6 | 0.19355 |
| 7799 | S9_50388637 | 9 | 50388637 | 66 | T | C | T | 0.93548 | C | 0.06452 | 4 | 0.12903 |
| 7800 | S9_51000183 | 9 | 51000183 | 611546 | C | T | T | 0.74194 | C | 0.25806 | 16 | 0.51613 |
| 7801 | S9_51000323 | 9 | 51000323 | 140 | G | C | C | 0.74194 | G | 0.25806 | 16 | 0.51613 |
| 7802 | S9_51000349 | 9 | 51000349 | 26 | A | C | C | 0.87097 | A | 0.12903 | 8 | 0.25806 |
| 7803 | S9_51398998 | 9 | 51398998 | 398649 | T | C | T | 0.87097 | C | 0.12903 | 8 | 0.25806 |
| 7804 | S9_51399003 | 9 | 51399003 | 5 | A | C | A | 0.87097 | C | 0.12903 | 8 | 0.25806 |
| 7805 | S9_51399069 | 9 | 51399069 | 66 | A | G | G | 0.75806 | A | 0.24194 | 9 | 0.29032 |
| 7806 | S9_51399075 | 9 | 51399075 | 6 | G | A | G | 0.8871 | A | 0.1129 | 7 | 0.22581 |
| 7807 | S9_51892680 | 9 | 51892680 | 493605 | T | C | T | 0.82258 | C | 0.17742 | 7 | 0.22581 |
| 7808 | S9_51967755 | 9 | 51967755 | 75075 | G | A | G | 0.95161 | A | 0.04839 | 3 | 0.09677 |
| 7809 | S9_52327263 | 9 | 52327263 | 359508 | C | T | C | 0.8871 | T | 0.1129 | 7 | 0.22581 |
| 7810 | S9_52711526 | 9 | 52711526 | 384263 | G | A | G | 0.82258 | A | 0.17742 | 11 | 0.35484 |
| 7811 | S9_53104135 | 9 | 53104135 | 392609 | T | C | T | 0.93548 | C | 0.06452 | 4 | 0.12903 |
| 7812 | S9_53758811 | 9 | 53758811 | 654676 | A | T | A | 0.77419 | T | 0.22581 | 10 | 0.32258 |
| 7813 | S9_54631963 | 9 | 54631963 | 873152 | C | T | T | 0.79032 | C | 0.20968 | 13 | 0.41935 |
| 7814 | S9_54935461 | 9 | 54935461 | 303498 | T | C | T | 0.91935 | C | 0.08065 | 5 | 0.16129 |
| 7815 | S9_55067359 | 9 | 55067359 | 131898 | C | T | C | 0.59677 | T | 0.40323 | 17 | 0.54839 |
| 7816 | S9_55149885 | 9 | 55149885 | 82526 | G | A | G | 0.95161 | A | 0.04839 | 3 | 0.09677 |
| 7817 | S9_55496110 | 9 | 55496110 | 346225 | T | C | T | 0.93548 | C | 0.06452 | 4 | 0.12903 |
| 7818 | S9_55496238 | 9 | 55496238 | 128 | C | G | C | 0.91935 | G | 0.08065 | 5 | 0.16129 |
| 7819 | S9_55496241 | 9 | 55496241 | 3 | T | C | C | 0.54839 | T | 0.45161 | 14 | 0.45161 |
| 7820 | S9_55704843 | 9 | 55704843 | 208602 | C | T | C | 0.82258 | T | 0.17742 | 7 | 0.22581 |
| 7821 | S9_55947736 | 9 | 55947736 | 242893 | G | A | G | 0.85484 | A | 0.14516 | 5 | 0.16129 |
| 7822 | S9_55947742 | 9 | 55947742 | 6 | G | C | C | 0.80645 | G | 0.19355 | 6 | 0.19355 |
| 7823 | S9_55947836 | 9 | 55947836 | 94 | T | C | T | 0.59677 | C | 0.40323 | 13 | 0.41935 |
| 7824 | S9_56475459 | 9 | 56475459 | 527623 | T | C | T | 0.77419 | C | 0.22581 | 10 | 0.32258 |
| 7825 | S9_57041048 | 9 | 57041048 | 565589 | C | T | C | 0.53226 | T | 0.46774 | 17 | 0.54839 |
| 7826 | S9_57279485 | 9 | 57279485 | 238437 | C | T | C | 0.69355 | T | 0.30645 | 13 | 0.41935 |
| 7827 | S9_57279513 | 9 | 57279513 | 28 | C | A | C | 0.77419 | A | 0.22581 | 10 | 0.32258 |
| 7828 | S9_57279518 | 9 | 57279518 | 5 | A | G | A | 0.93548 | G | 0.06452 | 4 | 0.12903 |

| Site number | SNP name | Chromosome | Physical position | Physical distance from the previous SNP | Reference allele | Alternative allele | Major allele | Major allele frequency | Minor allele | Minor allele frequency | Number heterozygous | Heterozygosity |
|---|---|---|---|---|---|---|---|---|---|---|---|---|
| 7829 | S9_57279570 | 9 | 57279570 | 52 | A | G | A | 0.77419 | G | 0.22581 | 10 | 0.32258 |
| 7830 | S9_57312382 | 9 | 57312382 | 32812 | T | A | T | 0.95161 | A | 0.04839 | 3 | 0.09677 |
| 7831 | S9_57485623 | 9 | 57485623 | 173241 | C | A | C | 0.93548 | A | 0.06452 | 4 | 0.12903 |
| 7832 | S9_57485624 | 9 | 57485624 | 1 | A | G | A | 0.70968 | G | 0.29032 | 12 | 0.3871 |
| 7833 | S9_57583912 | 9 | 57583912 | 98288 | T | C | T | 0.90323 | C | 0.09677 | 6 | 0.19355 |
| 7834 | S9_57583946 | 9 | 57583946 | 34 | C | T | C | 0.87097 | T | 0.12903 | 6 | 0.19355 |
| 7835 | S9_57583950 | 9 | 57583950 | 4 | A | G | A | 0.90323 | G | 0.09677 | 6 | 0.19355 |
| 7836 | S9_57584012 | 9 | 57584012 | 62 | C | T | C | 0.90323 | T | 0.09677 | 6 | 0.19355 |
| 7837 | S9_57584048 | 9 | 57584048 | 36 | G | A | G | 0.74194 | A | 0.25806 | 12 | 0.3871 |
| 7838 | S9_57745853 | 9 | 57745853 | 161805 | T | C | T | 0.56452 | C | 0.43548 | 15 | 0.48387 |
| 7839 | S9_57745865 | 9 | 57745865 | 12 | A | G | A | 0.95161 | G | 0.04839 | 3 | 0.09677 |
| 7840 | S9_57745920 | 9 | 57745920 | 55 | G | A | G | 0.56452 | A | 0.43548 | 15 | 0.48387 |
| 7841 | S9_59056303 | 9 | 59056303 | 1310383 | T | C | C | 0.87097 | T | 0.12903 | 8 | 0.25806 |
| 7842 | S9_59206427 | 9 | 59206427 | 150124 | T | C | C | 0.74194 | T | 0.25806 | 12 | 0.3871 |
| 7843 | S9_59206463 | 9 | 59206463 | 36 | A | G | A | 0.87097 | G | 0.12903 | 8 | 0.25806 |
| 7844 | S9_59710082 | 9 | 59710082 | 503619 | G | T | G | 0.82258 | T | 0.17742 | 7 | 0.22581 |
| 7845 | S9_59710126 | 9 | 59710126 | 44 | G | T | G | 0.82258 | T | 0.17742 | 7 | 0.22581 |
| 7846 | S9_59710127 | 9 | 59710127 | 1 | A | G | A | 0.95161 | G | 0.04839 | 3 | 0.09677 |
| 7847 | S9_59710313 | 9 | 59710313 | 186 | G | A | G | 0.82258 | A | 0.17742 | 7 | 0.22581 |
| 7848 | S9_59714064 | 9 | 59714064 | 3751 | C | A | C | 0.95161 | A | 0.04839 | 3 | 0.09677 |
| 7849 | S9_60073123 | 9 | 60073123 | 359059 | G | C | G | 0.91935 | C | 0.08065 | 5 | 0.16129 |
| 7850 | S9_60073124 | 9 | 60073124 | 1 | C | T | C | 0.91935 | T | 0.08065 | 5 | 0.16129 |
| 7851 | S9_60073126 | 9 | 60073126 | 2 | C | T | T | 0.66129 | C | 0.33871 | 11 | 0.35484 |
| 7852 | S9_60073266 | 9 | 60073266 | 140 | A | C | A | 0.77419 | C | 0.22581 | 10 | 0.32258 |
| 7853 | S9_60128448 | 9 | 60128448 | 55182 | A | G | A | 0.70968 | G | 0.29032 | 14 | 0.45161 |
| 7854 | S9_60128498 | 9 | 60128498 | 50 | C | A | C | 0.67742 | A | 0.32258 | 14 | 0.45161 |
| 7855 | S9_60140919 | 9 | 60140919 | 12421 | G | A | G | 0.79032 | A | 0.20968 | 11 | 0.35484 |
| 7856 | S9_60140976 | 9 | 60140976 | 57 | G | A | G | 0.91935 | A | 0.08065 | 5 | 0.16129 |
| 7857 | S9_60141044 | 9 | 60141044 | 68 | C | T | C | 0.93548 | T | 0.06452 | 4 | 0.12903 |
| 7858 | S9_60146947 | 9 | 60146947 | 5903 | C | T | C | 0.95161 | T | 0.04839 | 3 | 0.09677 |
| 7859 | S9_60146973 | 9 | 60146973 | 26 | C | A | C | 0.72581 | A | 0.27419 | 13 | 0.41935 |
| 7860 | S9_60146991 | 9 | 60146991 | 18 | T | G | T | 0.72581 | G | 0.27419 | 13 | 0.41935 |
| 7861 | S9_60347167 | 9 | 60347167 | 200176 | C | T | C | 0.91935 | T | 0.08065 | 5 | 0.16129 |
| 7862 | S9_60347273 | 9 | 60347273 | 106 | G | A | G | 0.90323 | A | 0.09677 | 6 | 0.19355 |
| 7863 | S9_61037155 | 9 | 61037155 | 689882 | A | C | A | 0.79032 | C | 0.20968 | 9 | 0.29032 |
| 7864 | S9_61037179 | 9 | 61037179 | 24 | T | C | T | 0.69355 | C | 0.30645 | 11 | 0.35484 |
| 7865 | S9_61037195 | 9 | 61037195 | 16 | C | G | C | 0.93548 | G | 0.06452 | 4 | 0.12903 |
| 7866 | S9_61037202 | 9 | 61037202 | 7 | G | C | C | 0.93548 | G | 0.06452 | 4 | 0.12903 |
| 7867 | S9_61037210 | 9 | 61037210 | 8 | T | G | T | 0.79032 | G | 0.20968 | 9 | 0.29032 |
| 7868 | S9_61037223 | 9 | 61037223 | 13 | A | C | A | 0.87097 | C | 0.12903 | 8 | 0.25806 |
| 7869 | S9_61366531 | 9 | 61366531 | 329308 | A | G | G | 0.8871 | A | 0.1129 | 7 | 0.22581 |
| 7870 | S9_61366699 | 9 | 61366699 | 168 | A | G | G | 0.51613 | A | 0.48387 | 10 | 0.32258 |
| 7871 | S9_61366701 | 9 | 61366701 | 2 | G | T | T | 0.51613 | G | 0.48387 | 10 | 0.32258 |
| 7872 | S9_61685433 | 9 | 61685433 | 318732 | T | G | T | 0.64516 | G | 0.35484 | 18 | 0.58065 |
| 7873 | S9_61685541 | 9 | 61685541 | 108 | T | G | T | 0.82258 | G | 0.17742 | 7 | 0.22581 |
| 7874 | S9_61851657 | 9 | 61851657 | 166116 | T | C | T | 0.64516 | C | 0.35484 | 10 | 0.32258 |
| 7875 | S9_61928568 | 9 | 61928568 | 76911 | C | T | C | 0.91935 | T | 0.08065 | 5 | 0.16129 |
| 7876 | S9_61990089 | 9 | 61990089 | 61521 | C | T | C | 0.6129 | T | 0.3871 | 16 | 0.51613 |
| 7877 | S9_61995273 | 9 | 61995273 | 5184 | G | A | A | 0.75806 | G | 0.24194 | 15 | 0.48387 |
| 7878 | S9_62676273 | 9 | 62676273 | 681000 | G | A | G | 0.56452 | A | 0.43548 | 13 | 0.41935 |
| 7879 | S9_62676318 | 9 | 62676318 | 45 | C | T | T | 0.93548 | C | 0.06452 | 4 | 0.12903 |
| 7880 | S9_62676384 | 9 | 62676384 | 66 | T | G | G | 0.93548 | T | 0.06452 | 4 | 0.12903 |
| 7881 | S9_62772424 | 9 | 62772424 | 96040 | G | T | G | 0.90323 | T | 0.09677 | 6 | 0.19355 |
| 7882 | S9_63120407 | 9 | 63120407 | 347983 | C | T | C | 0.87097 | T | 0.12903 | 6 | 0.19355 |

| Site number | SNP name | Chromosome | Physical position | Physical distance from the previous SNP | Reference allele | Alternative allele | Major allele | Major allele frequency | Minor allele | Minor allele frequency | Number heterozygous | Heterozygosity |
|---|---|---|---|---|---|---|---|---|---|---|---|---|
| 7883 | S9_63120545 | 9 | 63120545 | 138 | T | C | T | 0.90323 | C | 0.09677 | 6 | 0.19355 |
| 7884 | S9_63120553 | 9 | 63120553 | 8 | C | T | C | 0.90323 | T | 0.09677 | 6 | 0.19355 |
| 7885 | S9_63451790 | 9 | 63451790 | 331237 | C | T | C | 0.93548 | T | 0.06452 | 4 | 0.12903 |
| 7886 | S9_63451883 | 9 | 63451883 | 93 | C | T | C | 0.93548 | T | 0.06452 | 4 | 0.12903 |
| 7887 | S9_63451956 | 9 | 63451956 | 73 | C | A | A | 0.54839 | C | 0.45161 | 20 | 0.64516 |
| 7888 | S9_63451982 | 9 | 63451982 | 26 | C | T | C | 0.93548 | T | 0.06452 | 4 | 0.12903 |
| 7889 | S9_63629279 | 9 | 63629279 | 177297 | C | T | C | 0.93548 | T | 0.06452 | 4 | 0.12903 |
| 7890 | S9_63870481 | 9 | 63870481 | 241202 | G | A | G | 0.85484 | A | 0.14516 | 7 | 0.22581 |
| 7891 | S9_65143365 | 9 | 65143365 | 1272884 | G | A | A | 0.90323 | G | 0.09677 | 6 | 0.19355 |
| 7892 | S9_65143422 | 9 | 65143422 | 57 | A | G | G | 0.75806 | A | 0.24194 | 9 | 0.29032 |
| 7893 | S9_65143583 | 9 | 65143583 | 161 | T | A | A | 0.90323 | T | 0.09677 | 6 | 0.19355 |
| 7894 | S9_65193649 | 9 | 65193649 | 50066 | G | A | A | 0.8871 | G | 0.1129 | 7 | 0.22581 |
| 7895 | S9_65306497 | 9 | 65306497 | 112848 | A | G | A | 0.69355 | G | 0.30645 | 17 | 0.54839 |
| 7896 | S9_65306565 | 9 | 65306565 | 68 | A | G | A | 0.74194 | G | 0.25806 | 14 | 0.45161 |
| 7897 | S9_66182774 | 9 | 66182774 | 876209 | C | T | C | 0.93548 | T | 0.06452 | 4 | 0.12903 |
| 7898 | S9_66753899 | 9 | 66753899 | 571125 | G | A | A | 0.67742 | G | 0.32258 | 16 | 0.51613 |
| 7899 | S9_66765070 | 9 | 66765070 | 11171 | C | A | C | 0.87097 | A | 0.12903 | 4 | 0.12903 |
| 7900 | S9_66909951 | 9 | 66909951 | 144881 | T | C | T | 0.8871 | C | 0.1129 | 7 | 0.22581 |
| 7901 | S9_66943270 | 9 | 66943270 | 33319 | A | G | A | 0.83871 | G | 0.16129 | 10 | 0.32258 |
| 7902 | S9_67100955 | 9 | 67100955 | 157685 | C | T | T | 0.74194 | C | 0.25806 | 14 | 0.45161 |
| 7903 | S9_67101007 | 9 | 67101007 | 52 | C | T | T | 0.59677 | C | 0.40323 | 15 | 0.48387 |
| 7904 | S9_67235031 | 9 | 67235031 | 134024 | T | C | C | 0.53226 | T | 0.46774 | 15 | 0.48387 |
| 7905 | S9_67690740 | 9 | 67690740 | 455709 | A | G | G | 0.54839 | A | 0.45161 | 14 | 0.45161 |
| 7906 | S9_67786977 | 9 | 67786977 | 96237 | G | A | G | 0.74194 | A | 0.25806 | 12 | 0.3871 |
| 7907 | S9_67787078 | 9 | 67787078 | 101 | G | C | G | 0.74194 | C | 0.25806 | 12 | 0.3871 |
| 7908 | S9_67787091 | 9 | 67787091 | 13 | C | T | C | 0.74194 | T | 0.25806 | 12 | 0.3871 |
| 7909 | S9_67787093 | 9 | 67787093 | 2 | G | A | G | 0.74194 | A | 0.25806 | 12 | 0.3871 |
| 7910 | S9_67948879 | 9 | 67948879 | 161786 | C | T | C | 0.95161 | T | 0.04839 | 3 | 0.09677 |
| 7911 | S9_68014040 | 9 | 68014040 | 65161 | G | A | G | 0.8871 | A | 0.1129 | 7 | 0.22581 |
| 7912 | S9_68052918 | 9 | 68052918 | 38878 | T | C | T | 0.90323 | C | 0.09677 | 6 | 0.19355 |
| 7913 | S9_68052959 | 9 | 68052959 | 41 | T | C | T | 0.91935 | C | 0.08065 | 5 | 0.16129 |
| 7914 | S9_68286688 | 9 | 68286688 | 233729 | C | G | C | 0.93548 | G | 0.06452 | 4 | 0.12903 |
| 7915 | S9_68286758 | 9 | 68286758 | 70 | T | C | T | 0.59677 | C | 0.40323 | 15 | 0.48387 |
| 7916 | S9_68880502 | 9 | 68880502 | 593744 | G | C | G | 0.85484 | C | 0.14516 | 7 | 0.22581 |
| 7917 | S9_68880519 | 9 | 68880519 | 17 | A | C | A | 0.54839 | C | 0.45161 | 16 | 0.51613 |
| 7918 | S9_69241856 | 9 | 69241856 | 361337 | G | A | A | 0.69355 | G | 0.30645 | 13 | 0.41935 |
| 7919 | S9_69241985 | 9 | 69241985 | 129 | T | C | C | 0.8871 | T | 0.1129 | 5 | 0.16129 |
| 7920 | S9_69241988 | 9 | 69241988 | 3 | T | A | A | 0.8871 | T | 0.1129 | 5 | 0.16129 |
| 7921 | S9_70183685 | 9 | 70183685 | 941697 | A | G | G | 0.6129 | A | 0.3871 | 14 | 0.45161 |
| 7922 | S9_70720075 | 9 | 70720075 | 536390 | G | A | G | 0.91935 | A | 0.08065 | 5 | 0.16129 |
| 7923 | S9_70992255 | 9 | 70992255 | 272180 | C | T | C | 0.77419 | T | 0.22581 | 8 | 0.25806 |
| 7924 | S9_70992300 | 9 | 70992300 | 45 | G | A | G | 0.8871 | A | 0.1129 | 7 | 0.22581 |
| 7925 | S9_70992418 | 9 | 70992418 | 118 | T | C | T | 0.51613 | C | 0.48387 | 16 | 0.51613 |
| 7926 | S9_71101291 | 9 | 71101291 | 108873 | C | T | C | 0.95161 | T | 0.04839 | 3 | 0.09677 |
| 7927 | S9_71418853 | 9 | 71418853 | 317562 | C | T | C | 0.80645 | T | 0.19355 | 12 | 0.3871 |
| 7928 | S9_71598427 | 9 | 71598427 | 179574 | T | C | T | 0.58065 | C | 0.41935 | 18 | 0.58065 |
| 7929 | S9_71598579 | 9 | 71598579 | 152 | G | A | G | 0.74194 | A | 0.25806 | 14 | 0.45161 |
| 7930 | S9_71881994 | 9 | 71881994 | 283415 | C | T | C | 0.66129 | T | 0.33871 | 13 | 0.41935 |
| 7931 | S9_72405132 | 9 | 72405132 | 523138 | C | T | C | 0.5 | T | 0.5 | 19 | 0.6129 |
| 7932 | S9_72405133 | 9 | 72405133 | 1 | A | G | A | 0.5 | G | 0.5 | 17 | 0.54839 |
| 7933 | S9_72405144 | 9 | 72405144 | 11 | G | A | G | 0.51613 | A | 0.48387 | 18 | 0.58065 |
| 7934 | S9_72405148 | 9 | 72405148 | 4 | G | A | G | 0.8871 | A | 0.1129 | 7 | 0.22581 |
| 7935 | S9_72405352 | 9 | 72405352 | 204 | T | C | T | 0.51613 | C | 0.48387 | 18 | 0.58065 |
| 7936 | S9_72506194 | 9 | 72506194 | 100842 | T | C | C | 0.67742 | T | 0.32258 | 12 | 0.3871 |

| Site number | SNP name | Chromosome | Physical position | Physical distance from the previous SNP | Reference allele | Alternative allele | Major allele | Major allele frequency | Minor allele | Minor allele frequency | Number heterozygous | Heterozygosity |
|---|---|---|---|---|---|---|---|---|---|---|---|---|
| 7937 | S9_72506207 | 9 | 72506207 | 13 | A | G | A | 0.91935 | G | 0.08065 | 5 | 0.16129 |
| 7938 | S9_72506248 | 9 | 72506248 | 41 | C | T | C | 0.90323 | T | 0.09677 | 6 | 0.19355 |
| 7939 | S9_72641426 | 9 | 72641426 | 135178 | T | C | T | 0.54839 | C | 0.45161 | 14 | 0.45161 |
| 7940 | S9_72641471 | 9 | 72641471 | 45 | G | A | G | 0.91935 | A | 0.08065 | 5 | 0.16129 |
| 7941 | S9_72641512 | 9 | 72641512 | 41 | T | C | T | 0.54839 | C | 0.45161 | 14 | 0.45161 |
| 7942 | S9_72736292 | 9 | 72736292 | 94780 | G | A | G | 0.93548 | A | 0.06452 | 4 | 0.12903 |
| 7943 | S9_72736392 | 9 | 72736392 | 100 | T | G | G | 0.87097 | T | 0.12903 | 8 | 0.25806 |
| 7944 | S9_72981358 | 9 | 72981358 | 244966 | T | C | T | 0.75806 | C | 0.24194 | 13 | 0.41935 |
| 7945 | S9_73253005 | 9 | 73253005 | 271647 | A | G | G | 0.82258 | A | 0.17742 | 9 | 0.29032 |
| 7946 | S9_73253193 | 9 | 73253193 | 188 | G | T | G | 0.87097 | T | 0.12903 | 8 | 0.25806 |
| 7947 | S9_73330273 | 9 | 73330273 | 77080 | G | A | G | 0.59677 | A | 0.40323 | 17 | 0.54839 |
| 7948 | S9_74080073 | 9 | 74080073 | 749800 | C | T | C | 0.91935 | T | 0.08065 | 5 | 0.16129 |
| 7949 | S9_74086858 | 9 | 74086858 | 6785 | G | A | G | 0.82258 | A | 0.17742 | 11 | 0.35484 |
| 7950 | S9_74111832 | 9 | 74111832 | 24974 | C | G | C | 0.93548 | G | 0.06452 | 4 | 0.12903 |
| 7951 | S9_74668275 | 9 | 74668275 | 556443 | G | A | G | 0.83871 | A | 0.16129 | 10 | 0.32258 |
| 7952 | S9_74758604 | 9 | 74758604 | 90329 | G | A | G | 0.85484 | A | 0.14516 | 7 | 0.22581 |
| 7953 | S9_74769248 | 9 | 74769248 | 10644 | G | A | G | 0.91935 | A | 0.08065 | 5 | 0.16129 |
| 7954 | S9_74769272 | 9 | 74769272 | 24 | C | T | C | 0.91935 | T | 0.08065 | 5 | 0.16129 |
| 7955 | S9_74769373 | 9 | 74769373 | 101 | C | A | C | 0.91935 | A | 0.08065 | 5 | 0.16129 |
| 7956 | S9_74769389 | 9 | 74769389 | 16 | C | T | C | 0.95161 | T | 0.04839 | 3 | 0.09677 |
| 7957 | S9_74769413 | 9 | 74769413 | 24 | A | G | A | 0.91935 | G | 0.08065 | 5 | 0.16129 |
| 7958 | S9_74769432 | 9 | 74769432 | 19 | G | T | G | 0.93548 | T | 0.06452 | 4 | 0.12903 |
| 7959 | S9_75065390 | 9 | 75065390 | 295958 | T | C | T | 0.8871 | C | 0.1129 | 7 | 0.22581 |
| 7960 | S9_75201848 | 9 | 75201848 | 136458 | C | T | C | 0.58065 | T | 0.41935 | 16 | 0.51613 |
| 7961 | S9_75201852 | 9 | 75201852 | 4 | T | C | T | 0.58065 | C | 0.41935 | 16 | 0.51613 |
| 7962 | S9_75201909 | 9 | 75201909 | 57 | G | A | G | 0.85484 | A | 0.14516 | 7 | 0.22581 |
| 7963 | S9_75201926 | 9 | 75201926 | 17 | T | C | C | 0.91935 | T | 0.08065 | 5 | 0.16129 |
| 7964 | S9_75201965 | 9 | 75201965 | 39 | C | T | C | 0.58065 | T | 0.41935 | 16 | 0.51613 |
| 7965 | S9_76706524 | 9 | 76706524 | 1504559 | T | C | T | 0.90323 | C | 0.09677 | 4 | 0.12903 |
| 7966 | S9_76814284 | 9 | 76814284 | 107760 | G | A | G | 0.62903 | A | 0.37097 | 13 | 0.41935 |
| 7967 | S9_76843547 | 9 | 76843547 | 29263 | G | A | G | 0.8871 | A | 0.1129 | 7 | 0.22581 |
| 7968 | S9_77026835 | 9 | 77026835 | 183288 | G | A | G | 0.75806 | A | 0.24194 | 9 | 0.29032 |
| 7969 | S9_77026848 | 9 | 77026848 | 13 | G | C | C | 0.6129 | G | 0.3871 | 16 | 0.51613 |
| 7970 | S9_77026893 | 9 | 77026893 | 45 | G | C | G | 0.80645 | C | 0.19355 | 10 | 0.32258 |
| 7971 | S9_77846775 | 9 | 77846775 | 819882 | C | A | A | 0.64516 | C | 0.35484 | 16 | 0.51613 |
| 7972 | S9_77846792 | 9 | 77846792 | 17 | C | T | T | 0.64516 | C | 0.35484 | 16 | 0.51613 |
| 7973 | S9_77846798 | 9 | 77846798 | 6 | G | A | A | 0.64516 | G | 0.35484 | 16 | 0.51613 |
| 7974 | S9_77846801 | 9 | 77846801 | 3 | C | T | T | 0.64516 | C | 0.35484 | 16 | 0.51613 |
| 7975 | S9_77846825 | 9 | 77846825 | 24 | G | A | A | 0.64516 | G | 0.35484 | 16 | 0.51613 |
| 7976 | S9_77846963 | 9 | 77846963 | 138 | T | A | A | 0.64516 | T | 0.35484 | 16 | 0.51613 |
| 7977 | S9_77846985 | 9 | 77846985 | 22 | T | G | T | 0.91935 | G | 0.08065 | 5 | 0.16129 |
| 7978 | S9_77874824 | 9 | 77874824 | 27839 | C | T | C | 0.87097 | T | 0.12903 | 8 | 0.25806 |
| 7979 | S9_77907043 | 9 | 77907043 | 32219 | T | C | C | 0.70968 | T | 0.29032 | 16 | 0.51613 |
| 7980 | S9_77907057 | 9 | 77907057 | 14 | T | C | C | 0.70968 | T | 0.29032 | 16 | 0.51613 |
| 7981 | S9_77907201 | 9 | 77907201 | 144 | T | C | C | 0.90323 | T | 0.09677 | 6 | 0.19355 |
| 7982 | S9_77907264 | 9 | 77907264 | 63 | G | A | G | 0.93548 | A | 0.06452 | 4 | 0.12903 |
| 7983 | S9_78019120 | 9 | 78019120 | 111856 | A | T | A | 0.80645 | T | 0.19355 | 10 | 0.32258 |
| 7984 | S9_78378899 | 9 | 78378899 | 359779 | G | T | G | 0.85484 | T | 0.14516 | 9 | 0.29032 |
| 7985 | S9_78818240 | 9 | 78818240 | 439341 | C | T | C | 0.75806 | T | 0.24194 | 15 | 0.48387 |
| 7986 | S9_78818921 | 9 | 78818921 | 681 | T | C | T | 0.51613 | C | 0.48387 | 22 | 0.70968 |
| 7987 | S9_78819100 | 9 | 78819100 | 179 | G | T | G | 0.75806 | T | 0.24194 | 15 | 0.48387 |
| 7988 | S9_78890563 | 9 | 78890563 | 71463 | C | G | C | 0.56452 | G | 0.43548 | 13 | 0.41935 |
| 7989 | S9_78890628 | 9 | 78890628 | 65 | T | C | T | 0.51613 | C | 0.48387 | 10 | 0.32258 |
| 7990 | S9_78949227 | 9 | 78949227 | 58599 | T | C | C | 0.74194 | T | 0.25806 | 10 | 0.32258 |

| Site number | SNP name | Chromosome | Physical position | Physical distance from the previous SNP | Reference allele | Alternative allele | Major allele | Major allele frequency | Minor allele | Minor allele frequency | Number heterozygous | Heterozygosity |
|---|---|---|---|---|---|---|---|---|---|---|---|---|
| 7991 | S9_78949244 | 9 | 78949244 | 17 | T | C | C | 0.64516 | T | 0.35484 | 12 | 0.3871 |
| 7992 | S9_78949294 | 9 | 78949294 | 50 | C | A | C | 0.90323 | A | 0.09677 | 6 | 0.19355 |
| 7993 | S9_79052049 | 9 | 79052049 | 102755 | A | C | A | 0.75806 | C | 0.24194 | 11 | 0.35484 |
| 7994 | S9_79052094 | 9 | 79052094 | 45 | A | G | G | 0.6129 | A | 0.3871 | 14 | 0.45161 |
| 7995 | S9_79052154 | 9 | 79052154 | 60 | A | G | A | 0.95161 | G | 0.04839 | 3 | 0.09677 |
| 7996 | S9_79052237 | 9 | 79052237 | 83 | A | C | A | 0.54839 | C | 0.45161 | 16 | 0.51613 |
| 7997 | S9_79110946 | 9 | 79110946 | 58709 | G | A | G | 0.93548 | A | 0.06452 | 2 | 0.06452 |
| 7998 | S9_79189010 | 9 | 79189010 | 78064 | G | A | G | 0.8871 | A | 0.1129 | 7 | 0.22581 |
| 7999 | S9_79189055 | 9 | 79189055 | 45 | G | A | G | 0.93548 | A | 0.06452 | 4 | 0.12903 |
| 8000 | S9_79189057 | 9 | 79189057 | 2 | A | G | A | 0.8871 | G | 0.1129 | 7 | 0.22581 |
| 8001 | S9_79189140 | 9 | 79189140 | 83 | T | C | T | 0.8871 | C | 0.1129 | 7 | 0.22581 |
| 8002 | S9_79189142 | 9 | 79189142 | 2 | T | C | T | 0.91935 | C | 0.08065 | 5 | 0.16129 |
| 8003 | S9_79347322 | 9 | 79347322 | 158180 | G | A | G | 0.8871 | A | 0.1129 | 7 | 0.22581 |
| 8004 | S9_79347331 | 9 | 79347331 | 9 | C | G | C | 0.8871 | G | 0.1129 | 7 | 0.22581 |
| 8005 | S9_79347337 | 9 | 79347337 | 6 | G | A | G | 0.80645 | A | 0.19355 | 12 | 0.3871 |
| 8006 | S9_79347383 | 9 | 79347383 | 46 | A | C | A | 0.70968 | C | 0.29032 | 14 | 0.45161 |
| 8007 | S9_79347484 | 9 | 79347484 | 101 | T | C | T | 0.91935 | C | 0.08065 | 3 | 0.09677 |
| 8008 | S9_79347504 | 9 | 79347504 | 20 | C | T | C | 0.80645 | T | 0.19355 | 12 | 0.3871 |
| 8009 | S9_79347510 | 9 | 79347510 | 6 | C | T | C | 0.8871 | T | 0.1129 | 7 | 0.22581 |
| 8010 | S9_79486691 | 9 | 79486691 | 139181 | C | A | A | 0.69355 | C | 0.30645 | 13 | 0.41935 |
| 8011 | S9_80672432 | 9 | 80672432 | 1185741 | A | T | A | 0.93548 | T | 0.06452 | 4 | 0.12903 |
| 8012 | S9_80837226 | 9 | 80837226 | 164794 | A | G | A | 0.58065 | G | 0.41935 | 18 | 0.58065 |
| 8013 | S9_80837238 | 9 | 80837238 | 12 | T | C | T | 0.91935 | C | 0.08065 | 3 | 0.09677 |
| 8014 | S9_81076222 | 9 | 81076222 | 238984 | G | A | G | 0.72581 | A | 0.27419 | 13 | 0.41935 |
| 8015 | S9_81302538 | 9 | 81302538 | 226316 | G | A | G | 0.93548 | A | 0.06452 | 4 | 0.12903 |
| 8016 | S9_81302567 | 9 | 81302567 | 29 | C | T | C | 0.8871 | T | 0.1129 | 7 | 0.22581 |
| 8017 | S9_81459185 | 9 | 81459185 | 156618 | G | T | G | 0.87097 | T | 0.12903 | 6 | 0.19355 |
| 8018 | S9_81459260 | 9 | 81459260 | 75 | C | T | C | 0.6129 | T | 0.3871 | 16 | 0.51613 |
| 8019 | S9_81778588 | 9 | 81778588 | 319328 | C | A | C | 0.95161 | A | 0.04839 | 1 | 0.03226 |
| 8020 | S9_81798879 | 9 | 81798879 | 20291 | C | T | C | 0.83871 | T | 0.16129 | 6 | 0.19355 |
| 8021 | S9_81798880 | 9 | 81798880 | 1 | G | A | G | 0.85484 | A | 0.14516 | 9 | 0.29032 |
| 8022 | S9_81861447 | 9 | 81861447 | 62567 | G | C | G | 0.95161 | C | 0.04839 | 3 | 0.09677 |
| 8023 | S9_81861549 | 9 | 81861549 | 102 | T | C | C | 0.59677 | T | 0.40323 | 13 | 0.41935 |
| 8024 | S9_81939359 | 9 | 81939359 | 77810 | C | G | C | 0.6129 | G | 0.3871 | 14 | 0.45161 |
| 8025 | S9_82055427 | 9 | 82055427 | 116068 | G | A | G | 0.93548 | A | 0.06452 | 4 | 0.12903 |
| 8026 | S9_82292552 | 9 | 82292552 | 237125 | C | T | C | 0.95161 | T | 0.04839 | 3 | 0.09677 |
| 8027 | S9_82292615 | 9 | 82292615 | 63 | G | A | G | 0.90323 | A | 0.09677 | 6 | 0.19355 |
| 8028 | S9_82292662 | 9 | 82292662 | 47 | G | A | G | 0.58065 | A | 0.41935 | 10 | 0.32258 |
| 8029 | S9_82900687 | 9 | 82900687 | 608025 | C | T | C | 0.74194 | T | 0.25806 | 14 | 0.45161 |
| 8030 | S9_83201264 | 9 | 83201264 | 300577 | T | C | T | 0.82258 | C | 0.17742 | 11 | 0.35484 |
| 8031 | S9_83201462 | 9 | 83201462 | 198 | G | A | G | 0.82258 | A | 0.17742 | 11 | 0.35484 |
| 8032 | S9_83373285 | 9 | 83373285 | 171823 | T | C | T | 0.51613 | C | 0.48387 | 12 | 0.3871 |
| 8033 | S9_83848300 | 9 | 83848300 | 475015 | G | A | G | 0.77419 | A | 0.22581 | 14 | 0.45161 |
| 8034 | S9_83848313 | 9 | 83848313 | 13 | C | A | A | 0.82258 | C | 0.17742 | 11 | 0.35484 |
| 8035 | S9_83922921 | 9 | 83922921 | 74608 | C | G | C | 0.95161 | G | 0.04839 | 3 | 0.09677 |
| 8036 | S9_84136645 | 9 | 84136645 | 213724 | C | T | T | 0.66129 | C | 0.33871 | 13 | 0.41935 |
| 8037 | S9_84136782 | 9 | 84136782 | 137 | T | C | T | 0.90323 | C | 0.09677 | 6 | 0.19355 |
| 8038 | S9_84144023 | 9 | 84144023 | 7241 | T | C | T | 0.77419 | C | 0.22581 | 8 | 0.25806 |
| 8039 | S9_84144035 | 9 | 84144035 | 12 | C | T | C | 0.93548 | T | 0.06452 | 4 | 0.12903 |
| 8040 | S9_84144208 | 9 | 84144208 | 173 | C | T | C | 0.93548 | T | 0.06452 | 4 | 0.12903 |
| 8041 | S9_84203635 | 9 | 84203635 | 59427 | G | C | G | 0.8871 | C | 0.1129 | 7 | 0.22581 |
| 8042 | S9_84336608 | 9 | 84336608 | 132973 | G | A | G | 0.83871 | A | 0.16129 | 10 | 0.32258 |
| 8043 | S9_84560266 | 9 | 84560266 | 223658 | C | T | T | 0.62903 | C | 0.37097 | 15 | 0.48387 |
| 8044 | S9_84560275 | 9 | 84560275 | 9 | C | A | C | 0.59677 | A | 0.40323 | 13 | 0.41935 |

| Site number | SNP name | Chromosome | Physical position | Physical distance from the previous SNP | Reference allele | Alternative allele | Major allele | Major allele frequency | Minor allele | Minor allele frequency | Number heterozygous | Heterozygosity |
|---|---|---|---|---|---|---|---|---|---|---|---|---|
| 8045 | S9_84792588 | 9 | 84792588 | 232313 | T | C | T | 0.79032 | C | 0.20968 | 13 | 0.41935 |
| 8046 | S9_84792623 | 9 | 84792623 | 35 | T | G | G | 0.70968 | T | 0.29032 | 14 | 0.45161 |
| 8047 | S9_84792635 | 9 | 84792635 | 12 | C | T | C | 0.93548 | T | 0.06452 | 4 | 0.12903 |
| 8048 | S9_84792637 | 9 | 84792637 | 2 | C | T | C | 0.79032 | T | 0.20968 | 13 | 0.41935 |
| 8049 | S9_84792699 | 9 | 84792699 | 62 | A | G | G | 0.70968 | A | 0.29032 | 14 | 0.45161 |
| 8050 | S9_85022259 | 9 | 85022259 | 229560 | C | T | C | 0.83871 | T | 0.16129 | 10 | 0.32258 |
| 8051 | S9_85022468 | 9 | 85022468 | 209 | A | G | A | 0.93548 | G | 0.06452 | 4 | 0.12903 |
| 8052 | S9_85235923 | 9 | 85235923 | 213455 | C | T | C | 0.87097 | T | 0.12903 | 8 | 0.25806 |
| 8053 | S9_85235949 | 9 | 85235949 | 26 | T | C | T | 0.90323 | C | 0.09677 | 6 | 0.19355 |
| 8054 | S9_85236030 | 9 | 85236030 | 81 | T | C | T | 0.85484 | C | 0.14516 | 7 | 0.22581 |
| 8055 | S9_85236031 | 9 | 85236031 | 1 | G | A | G | 0.91935 | A | 0.08065 | 3 | 0.09677 |
| 8056 | S9_85236036 | 9 | 85236036 | 5 | G | A | G | 0.85484 | A | 0.14516 | 9 | 0.29032 |
| 8057 | S9_85236042 | 9 | 85236042 | 6 | C | T | C | 0.91935 | T | 0.08065 | 3 | 0.09677 |
| 8058 | S9_85236112 | 9 | 85236112 | 70 | A | G | G | 0.80645 | A | 0.19355 | 12 | 0.3871 |
| 8059 | S9_85368050 | 9 | 85368050 | 131938 | A | G | A | 0.87097 | G | 0.12903 | 8 | 0.25806 |
| 8060 | S9_85368080 | 9 | 85368080 | 30 | C | T | C | 0.8871 | T | 0.1129 | 7 | 0.22581 |
| 8061 | S9_85538113 | 9 | 85538113 | 170033 | A | G | A | 0.90323 | G | 0.09677 | 6 | 0.19355 |
| 8062 | S9_85805403 | 9 | 85805403 | 267290 | G | C | G | 0.90323 | C | 0.09677 | 6 | 0.19355 |
| 8063 | S9_86254585 | 9 | 86254585 | 449182 | T | C | C | 0.95161 | T | 0.04839 | 3 | 0.09677 |
| 8064 | S9_86506975 | 9 | 86506975 | 252390 | A | G | A | 0.87097 | G | 0.12903 | 8 | 0.25806 |
| 8065 | S9_86507052 | 9 | 86507052 | 77 | C | T | C | 0.93548 | T | 0.06452 | 4 | 0.12903 |
| 8066 | S9_86507101 | 9 | 86507101 | 49 | C | T | C | 0.64516 | T | 0.35484 | 12 | 0.3871 |
| 8067 | S9_86513157 | 9 | 86513157 | 6056 | C | T | C | 0.62903 | T | 0.37097 | 11 | 0.35484 |
| 8068 | S9_86803390 | 9 | 86803390 | 290233 | G | A | G | 0.83871 | A | 0.16129 | 8 | 0.25806 |
| 8069 | S9_86803521 | 9 | 86803521 | 131 | G | A | G | 0.83871 | A | 0.16129 | 8 | 0.25806 |
| 8070 | S9_86846259 | 9 | 86846259 | 42738 | T | C | C | 0.6129 | T | 0.3871 | 14 | 0.45161 |
| 8071 | S9_86949725 | 9 | 86949725 | 103466 | T | C | T | 0.93548 | C | 0.06452 | 4 | 0.12903 |
| 8072 | S9_86949775 | 9 | 86949775 | 50 | C | T | C | 0.70968 | T | 0.29032 | 16 | 0.51613 |
| 8073 | S9_86949789 | 9 | 86949789 | 14 | C | A | C | 0.77419 | A | 0.22581 | 8 | 0.25806 |
| 8074 | S9_86949827 | 9 | 86949827 | 38 | A | T | A | 0.93548 | T | 0.06452 | 4 | 0.12903 |
| 8075 | S9_87025219 | 9 | 87025219 | 75392 | T | C | C | 0.93548 | T | 0.06452 | 4 | 0.12903 |
| 8076 | S9_87086320 | 9 | 87086320 | 61101 | G | A | G | 0.77419 | A | 0.22581 | 14 | 0.45161 |
| 8077 | S9_87638412 | 9 | 87638412 | 552092 | A | G | G | 0.8871 | A | 0.1129 | 7 | 0.22581 |
| 8078 | S9_87638448 | 9 | 87638448 | 36 | C | T | C | 0.8871 | T | 0.1129 | 7 | 0.22581 |
| 8079 | S9_87638464 | 9 | 87638464 | 16 | A | G | A | 0.8871 | G | 0.1129 | 7 | 0.22581 |
| 8080 | S9_87638616 | 9 | 87638616 | 152 | T | C | T | 0.67742 | C | 0.32258 | 10 | 0.32258 |
| 8081 | S9_87638617 | 9 | 87638617 | 1 | G | A | G | 0.87097 | A | 0.12903 | 8 | 0.25806 |
| 8082 | S9_87638641 | 9 | 87638641 | 24 | C | T | C | 0.54839 | T | 0.45161 | 16 | 0.51613 |
| 8083 | S9_87638649 | 9 | 87638649 | 8 | G | A | G | 0.54839 | A | 0.45161 | 16 | 0.51613 |
| 8084 | S9_87664015 | 9 | 87664015 | 25366 | C | T | C | 0.85484 | T | 0.14516 | 7 | 0.22581 |
| 8085 | S9_87664108 | 9 | 87664108 | 93 | T | C | C | 0.74194 | T | 0.25806 | 14 | 0.45161 |
| 8086 | S9_87897828 | 9 | 87897828 | 233720 | T | A | T | 0.72581 | A | 0.27419 | 17 | 0.54839 |
| 8087 | S9_88329604 | 9 | 88329604 | 431776 | C | T | C | 0.90323 | T | 0.09677 | 6 | 0.19355 |
| 8088 | S9_88329679 | 9 | 88329679 | 75 | A | C | A | 0.95161 | C | 0.04839 | 3 | 0.09677 |
| 8089 | S9_88470295 | 9 | 88470295 | 140616 | T | G | T | 0.87097 | G | 0.12903 | 8 | 0.25806 |
| 8090 | S9_88470348 | 9 | 88470348 | 53 | G | T | G | 0.90323 | T | 0.09677 | 6 | 0.19355 |
| 8091 | S9_88605527 | 9 | 88605527 | 135179 | G | A | G | 0.79032 | A | 0.20968 | 9 | 0.29032 |
| 8092 | S9_88616746 | 9 | 88616746 | 11219 | A | G | A | 0.82258 | G | 0.17742 | 11 | 0.35484 |
| 8093 | S9_88620427 | 9 | 88620427 | 3681 | G | C | G | 0.64516 | C | 0.35484 | 16 | 0.51613 |
| 8094 | S9_88725730 | 9 | 88725730 | 105303 | C | A | A | 0.90323 | C | 0.09677 | 6 | 0.19355 |
| 8095 | S9_88751318 | 9 | 88751318 | 25588 | G | A | G | 0.67742 | A | 0.32258 | 14 | 0.45161 |
| 8096 | S9_88751444 | 9 | 88751444 | 126 | C | T | C | 0.69355 | T | 0.30645 | 13 | 0.41935 |
| 8097 | S9_88751449 | 9 | 88751449 | 5 | A | C | A | 0.90323 | C | 0.09677 | 6 | 0.19355 |
| 8098 | S9_88948344 | 9 | 88948344 | 196895 | A | C | A | 0.80645 | C | 0.19355 | 10 | 0.32258 |

| Site number | SNP name | Chromosome | Physical position | Physical distance from the previous SNP | Reference allele | Alternative allele | Major allele | Major allele frequency | Minor allele | Minor allele frequency | Number heterozygous | Heterozygosity |
|---|---|---|---|---|---|---|---|---|---|---|---|---|
| 8099 | S9_88948345 | 9 | 88948345 | 1 | C | G | C | 0.80645 | G | 0.19355 | 10 | 0.32258 |
| 8100 | S9_88948358 | 9 | 88948358 | 13 | C | G | C | 0.80645 | G | 0.19355 | 10 | 0.32258 |
| 8101 | S9_88948383 | 9 | 88948383 | 25 | G | A | G | 0.95161 | A | 0.04839 | 3 | 0.09677 |
| 8102 | S9_88948393 | 9 | 88948393 | 10 | C | T | C | 0.85484 | T | 0.14516 | 9 | 0.29032 |
| 8103 | S9_88948498 | 9 | 88948498 | 105 | C | T | C | 0.79032 | T | 0.20968 | 9 | 0.29032 |
| 8104 | S9_89021691 | 9 | 89021691 | 73193 | G | A | G | 0.93548 | A | 0.06452 | 4 | 0.12903 |
| 8105 | S9_89042032 | 9 | 89042032 | 20341 | T | C | C | 0.90323 | T | 0.09677 | 6 | 0.19355 |
| 8106 | S9_89042125 | 9 | 89042125 | 93 | C | T | C | 0.79032 | T | 0.20968 | 9 | 0.29032 |
| 8107 | S9_89053479 | 9 | 89053479 | 11354 | C | T | C | 0.8871 | T | 0.1129 | 7 | 0.22581 |
| 8108 | S9_89138029 | 9 | 89138029 | 84550 | A | G | G | 0.82258 | A | 0.17742 | 11 | 0.35484 |
| 8109 | S9_89138071 | 9 | 89138071 | 42 | C | T | C | 0.90323 | T | 0.09677 | 6 | 0.19355 |
| 8110 | S9_89138095 | 9 | 89138095 | 24 | G | A | G | 0.90323 | A | 0.09677 | 6 | 0.19355 |
| 8111 | S9_89138178 | 9 | 89138178 | 83 | G | C | G | 0.75806 | C | 0.24194 | 11 | 0.35484 |
| 8112 | S9_89218754 | 9 | 89218754 | 80576 | T | C | C | 0.91935 | T | 0.08065 | 5 | 0.16129 |
| 8113 | S9_89421583 | 9 | 89421583 | 202829 | C | T | T | 0.58065 | C | 0.41935 | 14 | 0.45161 |
| 8114 | S9_89421719 | 9 | 89421719 | 136 | C | T | C | 0.66129 | T | 0.33871 | 13 | 0.41935 |
| 8115 | S9_89421739 | 9 | 89421739 | 20 | G | A | G | 0.77419 | A | 0.22581 | 10 | 0.32258 |
| 8116 | S9_89518586 | 9 | 89518586 | 96847 | G | A | G | 0.64516 | A | 0.35484 | 16 | 0.51613 |
| 8117 | S9_89518600 | 9 | 89518600 | 14 | A | G | A | 0.79032 | G | 0.20968 | 11 | 0.35484 |
| 8118 | S9_89518642 | 9 | 89518642 | 42 | A | G | A | 0.64516 | G | 0.35484 | 16 | 0.51613 |
| 8119 | S9_89546488 | 9 | 89546488 | 27846 | G | T | G | 0.5 | T | 0.5 | 15 | 0.48387 |
| 8120 | S9_89546516 | 9 | 89546516 | 28 | T | C | C | 0.51613 | T | 0.48387 | 14 | 0.45161 |
| 8121 | S9_89546520 | 9 | 89546520 | 4 | C | T | C | 0.5 | T | 0.5 | 15 | 0.48387 |
| 8122 | S9_89546525 | 9 | 89546525 | 5 | A | G | A | 0.5 | G | 0.5 | 15 | 0.48387 |
| 8123 | S9_89546546 | 9 | 89546546 | 21 | A | C | A | 0.5 | C | 0.5 | 15 | 0.48387 |
| 8124 | S9_89546635 | 9 | 89546635 | 89 | C | T | C | 0.75806 | T | 0.24194 | 15 | 0.48387 |
| 8125 | S9_89546647 | 9 | 89546647 | 12 | C | T | C | 0.5 | T | 0.5 | 15 | 0.48387 |
| 8126 | S9_89548882 | 9 | 89548882 | 2235 | A | G | A | 0.79032 | G | 0.20968 | 11 | 0.35484 |
| 8127 | S9_89548988 | 9 | 89548988 | 106 | G | T | T | 0.53226 | G | 0.46774 | 13 | 0.41935 |
| 8128 | S9_89549072 | 9 | 89549072 | 84 | C | T | T | 0.53226 | C | 0.46774 | 13 | 0.41935 |
| 8129 | S9_89768281 | 9 | 89768281 | 219209 | A | C | A | 0.54839 | C | 0.45161 | 16 | 0.51613 |
| 8130 | S9_89768370 | 9 | 89768370 | 89 | C | T | C | 0.79032 | T | 0.20968 | 13 | 0.41935 |
| 8131 | S9_90285055 | 9 | 90285055 | 516685 | G | A | G | 0.83871 | A | 0.16129 | 10 | 0.32258 |
| 8132 | S9_90344513 | 9 | 90344513 | 59458 | T | G | T | 0.93548 | G | 0.06452 | 4 | 0.12903 |
| 8133 | S9_90528346 | 9 | 90528346 | 183833 | G | T | G | 0.56452 | T | 0.43548 | 11 | 0.35484 |
| 8134 | S9_90528348 | 9 | 90528348 | 2 | G | A | G | 0.79032 | A | 0.20968 | 11 | 0.35484 |
| 8135 | S9_90971996 | 9 | 90971996 | 443648 | T | C | T | 0.93548 | C | 0.06452 | 4 | 0.12903 |
| 8136 | S9_90972026 | 9 | 90972026 | 30 | A | T | A | 0.72581 | T | 0.27419 | 9 | 0.29032 |
| 8137 | S9_90972182 | 9 | 90972182 | 156 | A | C | A | 0.72581 | C | 0.27419 | 9 | 0.29032 |
| 8138 | S9_91181817 | 9 | 91181817 | 209635 | T | C | T | 0.90323 | C | 0.09677 | 6 | 0.19355 |
| 8139 | S9_91378678 | 9 | 91378678 | 196861 | G | T | G | 0.75806 | T | 0.24194 | 9 | 0.29032 |
| 8140 | S9_91378722 | 9 | 91378722 | 44 | C | T | C | 0.90323 | T | 0.09677 | 6 | 0.19355 |
| 8141 | S10_320869 | 10 | 320869 | 0 | G | A | G | 0.91935 | A | 0.08065 | 5 | 0.16129 |
| 8142 | S10_570683 | 10 | 570683 | 249814 | A | G | A | 0.90323 | G | 0.09677 | 6 | 0.19355 |
| 8143 | S10_570830 | 10 | 570830 | 147 | C | T | C | 0.90323 | T | 0.09677 | 6 | 0.19355 |
| 8144 | S10_838874 | 10 | 838874 | 268044 | C | T | C | 0.91935 | T | 0.08065 | 5 | 0.16129 |
| 8145 | S10_838937 | 10 | 838937 | 63 | C | T | C | 0.93548 | T | 0.06452 | 4 | 0.12903 |
| 8146 | S10_838992 | 10 | 838992 | 55 | C | G | C | 0.82258 | G | 0.17742 | 9 | 0.29032 |
| 8147 | S10_1164475 | 10 | 1164475 | 325483 | G | C | G | 0.80645 | C | 0.19355 | 8 | 0.25806 |
| 8148 | S10_1182393 | 10 | 1182393 | 17918 | C | T | C | 0.91935 | T | 0.08065 | 5 | 0.16129 |
| 8149 | S10_1182395 | 10 | 1182395 | 2 | G | A | G | 0.91935 | A | 0.08065 | 5 | 0.16129 |
| 8150 | S10_1545105 | 10 | 1545105 | 362710 | A | G | G | 0.93548 | A | 0.06452 | 2 | 0.06452 |
| 8151 | S10_1817513 | 10 | 1817513 | 272408 | C | T | C | 0.8871 | T | 0.1129 | 5 | 0.16129 |
| 8152 | S10_1817516 | 10 | 1817516 | 3 | A | T | A | 0.8871 | T | 0.1129 | 5 | 0.16129 |

| Site number | SNP name | Chromosome | Physical position | Physical distance from the previous SNP | Reference allele | Alternative allele | Major allele | Major allele frequency | Minor allele | Minor allele frequency | Number heterozygous | Heterozygosity |
|---|---|---|---|---|---|---|---|---|---|---|---|---|
| 8153 | S10_1817546 | 10 | 1817546 | 30 | G | A | G | 0.8871 | A | 0.1129 | 5 | 0.16129 |
| 8154 | S10_1958919 | 10 | 1958919 | 141373 | A | C | C | 0.66129 | A | 0.33871 | 15 | 0.48387 |
| 8155 | S10_1966472 | 10 | 1966472 | 7553 | G | T | T | 0.74194 | G | 0.25806 | 12 | 0.3871 |
| 8156 | S10_2020606 | 10 | 2020606 | 54134 | A | G | A | 0.83871 | G | 0.16129 | 8 | 0.25806 |
| 8157 | S10_2189953 | 10 | 2189953 | 169347 | G | T | G | 0.93548 | T | 0.06452 | 4 | 0.12903 |
| 8158 | S10_2190038 | 10 | 2190038 | 85 | C | T | C | 0.90323 | T | 0.09677 | 4 | 0.12903 |
| 8159 | S10_2190061 | 10 | 2190061 | 23 | C | T | C | 0.95161 | T | 0.04839 | 3 | 0.09677 |
| 8160 | S10_2190086 | 10 | 2190086 | 25 | C | T | C | 0.85484 | T | 0.14516 | 7 | 0.22581 |
| 8161 | S10_2290751 | 10 | 2290751 | 100665 | G | A | G | 0.83871 | A | 0.16129 | 10 | 0.32258 |
| 8162 | S10_2290925 | 10 | 2290925 | 174 | A | G | A | 0.83871 | G | 0.16129 | 10 | 0.32258 |
| 8163 | S10_2619454 | 10 | 2619454 | 328529 | A | G | G | 0.93548 | A | 0.06452 | 2 | 0.06452 |
| 8164 | S10_2622401 | 10 | 2622401 | 2947 | G | A | G | 0.83871 | A | 0.16129 | 8 | 0.25806 |
| 8165 | S10_2622497 | 10 | 2622497 | 96 | C | A | C | 0.82258 | A | 0.17742 | 11 | 0.35484 |
| 8166 | S10_2628603 | 10 | 2628603 | 6106 | T | A | T | 0.62903 | A | 0.37097 | 13 | 0.41935 |
| 8167 | S10_2628642 | 10 | 2628642 | 39 | A | G | A | 0.64516 | G | 0.35484 | 14 | 0.45161 |
| 8168 | S10_2628721 | 10 | 2628721 | 79 | G | C | G | 0.91935 | C | 0.08065 | 5 | 0.16129 |
| 8169 | S10_2628735 | 10 | 2628735 | 14 | A | G | A | 0.70968 | G | 0.29032 | 12 | 0.3871 |
| 8170 | S10_2628820 | 10 | 2628820 | 85 | T | G | T | 0.91935 | G | 0.08065 | 5 | 0.16129 |
| 8171 | S10_2697174 | 10 | 2697174 | 68354 | C | T | C | 0.8871 | T | 0.1129 | 5 | 0.16129 |
| 8172 | S10_2697582 | 10 | 2697582 | 408 | G | A | G | 0.8871 | A | 0.1129 | 7 | 0.22581 |
| 8173 | S10_2697594 | 10 | 2697594 | 12 | G | C | G | 0.85484 | C | 0.14516 | 7 | 0.22581 |
| 8174 | S10_2866797 | 10 | 2866797 | 169203 | A | G | G | 0.83871 | A | 0.16129 | 10 | 0.32258 |
| 8175 | S10_3435872 | 10 | 3435872 | 569075 | C | T | T | 0.51613 | C | 0.48387 | 22 | 0.70968 |
| 8176 | S10_3435948 | 10 | 3435948 | 76 | A | G | G | 0.53226 | A | 0.46774 | 21 | 0.67742 |
| 8177 | S10_3512727 | 10 | 3512727 | 76779 | T | C | T | 0.95161 | C | 0.04839 | 3 | 0.09677 |
| 8178 | S10_3512814 | 10 | 3512814 | 87 | G | C | G | 0.83871 | C | 0.16129 | 6 | 0.19355 |
| 8179 | S10_3512823 | 10 | 3512823 | 9 | G | C | C | 0.53226 | G | 0.46774 | 13 | 0.41935 |
| 8180 | S10_3512893 | 10 | 3512893 | 70 | T | C | T | 0.62903 | C | 0.37097 | 15 | 0.48387 |
| 8181 | S10_3527626 | 10 | 3527626 | 14733 | A | G | A | 0.93548 | G | 0.06452 | 4 | 0.12903 |
| 8182 | S10_3527778 | 10 | 3527778 | 152 | G | A | G | 0.77419 | A | 0.22581 | 10 | 0.32258 |
| 8183 | S10_3527787 | 10 | 3527787 | 9 | G | A | G | 0.77419 | A | 0.22581 | 10 | 0.32258 |
| 8184 | S10_3582775 | 10 | 3582775 | 54988 | G | A | G | 0.8871 | A | 0.1129 | 7 | 0.22581 |
| 8185 | S10_3582884 | 10 | 3582884 | 109 | C | T | C | 0.8871 | T | 0.1129 | 5 | 0.16129 |
| 8186 | S10_3583643 | 10 | 3583643 | 759 | C | T | T | 0.59677 | C | 0.40323 | 17 | 0.54839 |
| 8187 | S10_3583874 | 10 | 3583874 | 231 | T | G | T | 0.91935 | G | 0.08065 | 5 | 0.16129 |
| 8188 | S10_3659797 | 10 | 3659797 | 75923 | A | G | A | 0.69355 | G | 0.30645 | 17 | 0.54839 |
| 8189 | S10_3659822 | 10 | 3659822 | 25 | C | T | C | 0.64516 | T | 0.35484 | 18 | 0.58065 |
| 8190 | S10_3659905 | 10 | 3659905 | 83 | T | C | C | 0.95161 | T | 0.04839 | 3 | 0.09677 |
| 8191 | S10_3708041 | 10 | 3708041 | 48136 | G | A | G | 0.80645 | A | 0.19355 | 8 | 0.25806 |
| 8192 | S10_3933555 | 10 | 3933555 | 225514 | C | T | C | 0.95161 | T | 0.04839 | 3 | 0.09677 |
| 8193 | S10_3933602 | 10 | 3933602 | 47 | G | C | G | 0.95161 | C | 0.04839 | 3 | 0.09677 |
| 8194 | S10_3933613 | 10 | 3933613 | 11 | C | T | C | 0.82258 | T | 0.17742 | 9 | 0.29032 |
| 8195 | S10_4325811 | 10 | 4325811 | 392198 | G | A | G | 0.8871 | A | 0.1129 | 5 | 0.16129 |
| 8196 | S10_4334680 | 10 | 4334680 | 8869 | C | T | C | 0.67742 | T | 0.32258 | 16 | 0.51613 |
| 8197 | S10_4334826 | 10 | 4334826 | 146 | C | T | C | 0.69355 | T | 0.30645 | 15 | 0.48387 |
| 8198 | S10_4334827 | 10 | 4334827 | 1 | G | A | G | 0.70968 | A | 0.29032 | 14 | 0.45161 |
| 8199 | S10_4936730 | 10 | 4936730 | 601903 | C | T | C | 0.90323 | T | 0.09677 | 6 | 0.19355 |
| 8200 | S10_4936734 | 10 | 4936734 | 4 | C | T | C | 0.83871 | T | 0.16129 | 8 | 0.25806 |
| 8201 | S10_4936757 | 10 | 4936757 | 23 | C | T | C | 0.91935 | T | 0.08065 | 5 | 0.16129 |
| 8202 | S10_4936927 | 10 | 4936927 | 170 | C | T | T | 0.90323 | C | 0.09677 | 6 | 0.19355 |
| 8203 | S10_5929583 | 10 | 5929583 | 992656 | A | T | A | 0.91935 | T | 0.08065 | 5 | 0.16129 |
| 8204 | S10_5929654 | 10 | 5929654 | 71 | A | G | A | 0.67742 | G | 0.32258 | 12 | 0.3871 |
| 8205 | S10_5929758 | 10 | 5929758 | 104 | A | G | G | 0.66129 | A | 0.33871 | 17 | 0.54839 |
| 8206 | S10_6102072 | 10 | 6102072 | 172314 | A | G | A | 0.64516 | G | 0.35484 | 14 | 0.45161 |

| Site number | SNP name | Chromosome | Physical position | Physical distance from the previous SNP | Reference allele | Alternative allele | Major allele | Major allele frequency | Minor allele | Minor allele frequency | Number heterozygous | Heterozygosity |
|---|---|---|---|---|---|---|---|---|---|---|---|---|
| 8207 | S10_6262075 | 10 | 6262075 | 160003 | C | T | C | 0.91935 | T | 0.08065 | 5 | 0.16129 |
| 8208 | S10_6262221 | 10 | 6262221 | 146 | T | C | T | 0.77419 | C | 0.22581 | 8 | 0.25806 |
| 8209 | S10_6724551 | 10 | 6724551 | 462330 | C | T | C | 0.91935 | T | 0.08065 | 5 | 0.16129 |
| 8210 | S10_6920728 | 10 | 6920728 | 196177 | A | G | A | 0.95161 | G | 0.04839 | 3 | 0.09677 |
| 8211 | S10_6920739 | 10 | 6920739 | 11 | G | A | A | 0.6129 | G | 0.3871 | 16 | 0.51613 |
| 8212 | S10_6920889 | 10 | 6920889 | 150 | G | A | G | 0.91935 | A | 0.08065 | 5 | 0.16129 |
| 8213 | S10_7104986 | 10 | 7104986 | 184097 | T | C | C | 0.91935 | T | 0.08065 | 5 | 0.16129 |
| 8214 | S10_7965963 | 10 | 7965963 | 860977 | G | A | A | 0.51613 | G | 0.48387 | 16 | 0.51613 |
| 8215 | S10_7966039 | 10 | 7966039 | 76 | G | A | G | 0.51613 | A | 0.48387 | 16 | 0.51613 |
| 8216 | S10_8262694 | 10 | 8262694 | 296655 | C | T | C | 0.93548 | T | 0.06452 | 4 | 0.12903 |
| 8217 | S10_8262813 | 10 | 8262813 | 119 | G | A | G | 0.91935 | A | 0.08065 | 5 | 0.16129 |
| 8218 | S10_8327912 | 10 | 8327912 | 65099 | G | T | T | 0.67742 | G | 0.32258 | 14 | 0.45161 |
| 8219 | S10_8446377 | 10 | 8446377 | 118465 | C | T | C | 0.79032 | T | 0.20968 | 13 | 0.41935 |
| 8220 | S10_8636590 | 10 | 8636590 | 190213 | T | C | T | 0.93548 | C | 0.06452 | 2 | 0.06452 |
| 8221 | S10_8788110 | 10 | 8788110 | 151520 | G | A | G | 0.87097 | A | 0.12903 | 8 | 0.25806 |
| 8222 | S10_8788176 | 10 | 8788176 | 66 | C | G | C | 0.87097 | G | 0.12903 | 8 | 0.25806 |
| 8223 | S10_8788209 | 10 | 8788209 | 33 | G | A | G | 0.87097 | A | 0.12903 | 8 | 0.25806 |
| 8224 | S10_9628406 | 10 | 9628406 | 840197 | A | G | A | 0.59677 | G | 0.40323 | 13 | 0.41935 |
| 8225 | S10_9628465 | 10 | 9628465 | 59 | C | T | C | 0.72581 | T | 0.27419 | 9 | 0.29032 |
| 8226 | S10_9628467 | 10 | 9628467 | 2 | T | C | T | 0.70968 | C | 0.29032 | 10 | 0.32258 |
| 8227 | S10_9628504 | 10 | 9628504 | 37 | G | A | G | 0.8871 | A | 0.1129 | 7 | 0.22581 |
| 8228 | S10_9628561 | 10 | 9628561 | 57 | T | C | C | 0.66129 | T | 0.33871 | 13 | 0.41935 |
| 8229 | S10_9628623 | 10 | 9628623 | 62 | G | A | G | 0.93548 | A | 0.06452 | 4 | 0.12903 |
| 8230 | S10_9886321 | 10 | 9886321 | 257698 | C | T | C | 0.80645 | T | 0.19355 | 8 | 0.25806 |
| 8231 | S10_9886359 | 10 | 9886359 | 38 | G | A | G | 0.80645 | A | 0.19355 | 8 | 0.25806 |
| 8232 | S10_10233760 | 10 | 10233760 | 347401 | A | G | A | 0.82258 | G | 0.17742 | 9 | 0.29032 |
| 8233 | S10_10233785 | 10 | 10233785 | 25 | A | G | A | 0.59677 | G | 0.40323 | 15 | 0.48387 |
| 8234 | S10_10233878 | 10 | 10233878 | 93 | G | C | G | 0.59677 | C | 0.40323 | 15 | 0.48387 |
| 8235 | S10_10464666 | 10 | 10464666 | 230788 | A | C | A | 0.90323 | C | 0.09677 | 6 | 0.19355 |
| 8236 | S10_10464684 | 10 | 10464684 | 18 | A | G | A | 0.90323 | G | 0.09677 | 6 | 0.19355 |
| 8237 | S10_10464693 | 10 | 10464693 | 9 | C | T | C | 0.90323 | T | 0.09677 | 6 | 0.19355 |
| 8238 | S10_10464694 | 10 | 10464694 | 1 | A | G | A | 0.90323 | G | 0.09677 | 6 | 0.19355 |
| 8239 | S10_10464715 | 10 | 10464715 | 21 | G | A | G | 0.90323 | A | 0.09677 | 6 | 0.19355 |
| 8240 | S10_10464804 | 10 | 10464804 | 89 | T | C | T | 0.90323 | C | 0.09677 | 6 | 0.19355 |
| 8241 | S10_10464823 | 10 | 10464823 | 19 | G | A | G | 0.90323 | A | 0.09677 | 6 | 0.19355 |
| 8242 | S10_10464848 | 10 | 10464848 | 25 | T | C | C | 0.70968 | T | 0.29032 | 10 | 0.32258 |
| 8243 | S10_10517840 | 10 | 10517840 | 52992 | C | T | C | 0.90323 | T | 0.09677 | 4 | 0.12903 |
| 8244 | S10_10568776 | 10 | 10568776 | 50936 | A | C | A | 0.93548 | C | 0.06452 | 4 | 0.12903 |
| 8245 | S10_10730248 | 10 | 10730248 | 161472 | G | A | G | 0.87097 | A | 0.12903 | 6 | 0.19355 |
| 8246 | S10_11119347 | 10 | 11119347 | 389099 | C | T | C | 0.91935 | T | 0.08065 | 5 | 0.16129 |
| 8247 | S10_11427621 | 10 | 11427621 | 308274 | T | A | A | 0.59677 | T | 0.40323 | 15 | 0.48387 |
| 8248 | S10_11427664 | 10 | 11427664 | 43 | T | C | C | 0.62903 | T | 0.37097 | 13 | 0.41935 |
| 8249 | S10_11427702 | 10 | 11427702 | 38 | G | A | G | 0.62903 | A | 0.37097 | 13 | 0.41935 |
| 8250 | S10_11427771 | 10 | 11427771 | 69 | A | G | G | 0.62903 | A | 0.37097 | 13 | 0.41935 |
| 8251 | S10_12373503 | 10 | 12373503 | 945732 | G | C | G | 0.72581 | C | 0.27419 | 11 | 0.35484 |
| 8252 | S10_12373669 | 10 | 12373669 | 166 | G | A | G | 0.8871 | A | 0.1129 | 7 | 0.22581 |
| 8253 | S10_12413632 | 10 | 12413632 | 39963 | G | A | G | 0.82258 | A | 0.17742 | 9 | 0.29032 |
| 8254 | S10_12413757 | 10 | 12413757 | 125 | C | T | C | 0.93548 | T | 0.06452 | 4 | 0.12903 |
| 8255 | S10_12858317 | 10 | 12858317 | 444560 | G | A | G | 0.75806 | A | 0.24194 | 13 | 0.41935 |
| 8256 | S10_13084541 | 10 | 13084541 | 226224 | C | G | G | 0.6129 | C | 0.3871 | 18 | 0.58065 |
| 8257 | S10_13084563 | 10 | 13084563 | 22 | A | G | G | 0.58065 | A | 0.41935 | 20 | 0.64516 |
| 8258 | S10_13084581 | 10 | 13084581 | 18 | G | A | A | 0.58065 | G | 0.41935 | 20 | 0.64516 |
| 8259 | S10_13109947 | 10 | 13109947 | 25366 | A | G | A | 0.66129 | G | 0.33871 | 13 | 0.41935 |
| 8260 | S10_13275439 | 10 | 13275439 | 165492 | G | A | G | 0.90323 | A | 0.09677 | 6 | 0.19355 |

| Site number | SNP name | Chromosome | Physical position | Physical distance from the previous SNP | Reference allele | Alternative allele | Major allele | Major allele frequency | Minor allele | Minor allele frequency | Number heterozygous | Heterozygosity |
|---|---|---|---|---|---|---|---|---|---|---|---|---|
| 8261 | S10_13275486 | 10 | 13275486 | 47 | T | C | T | 0.77419 | C | 0.22581 | 12 | 0.3871 |
| 8262 | S10_13275651 | 10 | 13275651 | 165 | G | A | A | 0.53226 | G | 0.46774 | 15 | 0.48387 |
| 8263 | S10_13600097 | 10 | 13600097 | 324446 | T | C | C | 0.91935 | T | 0.08065 | 5 | 0.16129 |
| 8264 | S10_13600282 | 10 | 13600282 | 185 | G | T | G | 0.62903 | T | 0.37097 | 11 | 0.35484 |
| 8265 | S10_13718808 | 10 | 13718808 | 118526 | G | A | G | 0.64516 | A | 0.35484 | 12 | 0.3871 |
| 8266 | S10_13718851 | 10 | 13718851 | 43 | G | A | G | 0.56452 | A | 0.43548 | 13 | 0.41935 |
| 8267 | S10_13718880 | 10 | 13718880 | 29 | T | C | T | 0.6129 | C | 0.3871 | 14 | 0.45161 |
| 8268 | S10_13771830 | 10 | 13771830 | 52950 | C | T | C | 0.75806 | T | 0.24194 | 13 | 0.41935 |
| 8269 | S10_13771950 | 10 | 13771950 | 120 | C | T | C | 0.85484 | T | 0.14516 | 7 | 0.22581 |
| 8270 | S10_13772017 | 10 | 13772017 | 67 | C | T | C | 0.8871 | T | 0.1129 | 7 | 0.22581 |
| 8271 | S10_13822431 | 10 | 13822431 | 50414 | G | A | G | 0.90323 | A | 0.09677 | 6 | 0.19355 |
| 8272 | S10_13850418 | 10 | 13850418 | 27987 | A | G | A | 0.51613 | G | 0.48387 | 18 | 0.58065 |
| 8273 | S10_13850431 | 10 | 13850431 | 13 | A | G | A | 0.51613 | G | 0.48387 | 18 | 0.58065 |
| 8274 | S10_13850485 | 10 | 13850485 | 54 | G | A | G | 0.51613 | A | 0.48387 | 18 | 0.58065 |
| 8275 | S10_13904130 | 10 | 13904130 | 53645 | G | C | G | 0.77419 | C | 0.22581 | 10 | 0.32258 |
| 8276 | S10_13904272 | 10 | 13904272 | 142 | C | T | C | 0.53226 | T | 0.46774 | 17 | 0.54839 |
| 8277 | S10_13958400 | 10 | 13958400 | 54128 | G | A | G | 0.74194 | A | 0.25806 | 12 | 0.3871 |
| 8278 | S10_13958480 | 10 | 13958480 | 80 | C | T | T | 0.80645 | C | 0.19355 | 6 | 0.19355 |
| 8279 | S10_13958490 | 10 | 13958490 | 10 | A | G | A | 0.74194 | G | 0.25806 | 12 | 0.3871 |
| 8280 | S10_13958491 | 10 | 13958491 | 1 | C | G | G | 0.80645 | C | 0.19355 | 6 | 0.19355 |
| 8281 | S10_14018167 | 10 | 14018167 | 59676 | T | C | T | 0.87097 | C | 0.12903 | 6 | 0.19355 |
| 8282 | S10_14018180 | 10 | 14018180 | 13 | T | C | T | 0.87097 | C | 0.12903 | 6 | 0.19355 |
| 8283 | S10_14018226 | 10 | 14018226 | 46 | G | A | G | 0.8871 | A | 0.1129 | 5 | 0.16129 |
| 8284 | S10_14018307 | 10 | 14018307 | 81 | A | G | A | 0.8871 | G | 0.1129 | 5 | 0.16129 |
| 8285 | S10_14018308 | 10 | 14018308 | 1 | C | G | C | 0.8871 | G | 0.1129 | 5 | 0.16129 |
| 8286 | S10_14018310 | 10 | 14018310 | 2 | T | G | T | 0.8871 | G | 0.1129 | 5 | 0.16129 |
| 8287 | S10_14018346 | 10 | 14018346 | 36 | C | T | C | 0.8871 | T | 0.1129 | 5 | 0.16129 |
| 8288 | S10_14018349 | 10 | 14018349 | 3 | C | T | C | 0.87097 | T | 0.12903 | 6 | 0.19355 |
| 8289 | S10_14018356 | 10 | 14018356 | 7 | A | G | A | 0.87097 | G | 0.12903 | 6 | 0.19355 |
| 8290 | S10_14018367 | 10 | 14018367 | 11 | T | C | T | 0.87097 | C | 0.12903 | 6 | 0.19355 |
| 8291 | S10_14018374 | 10 | 14018374 | 7 | C | A | C | 0.87097 | A | 0.12903 | 6 | 0.19355 |
| 8292 | S10_14246648 | 10 | 14246648 | 228274 | G | A | A | 0.64516 | G | 0.35484 | 16 | 0.51613 |
| 8293 | S10_14246774 | 10 | 14246774 | 126 | A | G | A | 0.56452 | G | 0.43548 | 17 | 0.54839 |
| 8294 | S10_14455515 | 10 | 14455515 | 208741 | A | G | G | 0.8871 | A | 0.1129 | 7 | 0.22581 |
| 8295 | S10_14455584 | 10 | 14455584 | 69 | C | T | C | 0.93548 | T | 0.06452 | 4 | 0.12903 |
| 8296 | S10_14678073 | 10 | 14678073 | 222489 | G | A | G | 0.64516 | A | 0.35484 | 12 | 0.3871 |
| 8297 | S10_14678270 | 10 | 14678270 | 197 | A | G | A | 0.67742 | G | 0.32258 | 12 | 0.3871 |
| 8298 | S10_14709716 | 10 | 14709716 | 31446 | G | A | G | 0.93548 | A | 0.06452 | 4 | 0.12903 |
| 8299 | S10_14709880 | 10 | 14709880 | 164 | G | A | G | 0.95161 | A | 0.04839 | 3 | 0.09677 |
| 8300 | S10_14983225 | 10 | 14983225 | 273345 | G | A | G | 0.67742 | A | 0.32258 | 10 | 0.32258 |
| 8301 | S10_15135100 | 10 | 15135100 | 151875 | T | C | T | 0.8871 | C | 0.1129 | 5 | 0.16129 |
| 8302 | S10_15240328 | 10 | 15240328 | 105228 | C | T | C | 0.80645 | T | 0.19355 | 8 | 0.25806 |
| 8303 | S10_15240524 | 10 | 15240524 | 196 | C | T | C | 0.82258 | T | 0.17742 | 9 | 0.29032 |
| 8304 | S10_15343541 | 10 | 15343541 | 103017 | T | C | T | 0.95161 | C | 0.04839 | 3 | 0.09677 |
| 8305 | S10_15343732 | 10 | 15343732 | 191 | A | G | G | 0.91935 | A | 0.08065 | 5 | 0.16129 |
| 8306 | S10_15343737 | 10 | 15343737 | 5 | T | C | C | 0.91935 | T | 0.08065 | 5 | 0.16129 |
| 8307 | S10_15482511 | 10 | 15482511 | 138774 | T | C | C | 0.58065 | T | 0.41935 | 16 | 0.51613 |
| 8308 | S10_15482532 | 10 | 15482532 | 21 | A | G | G | 0.58065 | A | 0.41935 | 16 | 0.51613 |
| 8309 | S10_15482654 | 10 | 15482654 | 122 | G | T | G | 0.93548 | T | 0.06452 | 4 | 0.12903 |
| 8310 | S10_15482746 | 10 | 15482746 | 92 | A | G | A | 0.93548 | G | 0.06452 | 4 | 0.12903 |
| 8311 | S10_16014455 | 10 | 16014455 | 531709 | G | A | G | 0.56452 | A | 0.43548 | 17 | 0.54839 |
| 8312 | S10_16019808 | 10 | 16019808 | 5353 | T | C | C | 0.54839 | T | 0.45161 | 18 | 0.58065 |
| 8313 | S10_16099989 | 10 | 16099989 | 80181 | C | G | C | 0.80645 | G | 0.19355 | 10 | 0.32258 |
| 8314 | S10_16276282 | 10 | 16276282 | 176293 | G | A | G | 0.80645 | A | 0.19355 | 12 | 0.3871 |

| Site number | SNP name | Chromosome | Physical position | Physical distance from the previous SNP | Reference allele | Alternative allele | Major allele | Major allele frequency | Minor allele | Minor allele frequency | Number heterozygous | Heterozygosity |
|---|---|---|---|---|---|---|---|---|---|---|---|---|
| 8315 | S10_16372838 | 10 | 16372838 | 96556 | C | T | C | 0.93548 | T | 0.06452 | 4 | 0.12903 |
| 8316 | S10_16612214 | 10 | 16612214 | 239376 | C | G | C | 0.93548 | G | 0.06452 | 4 | 0.12903 |
| 8317 | S10_16612310 | 10 | 16612310 | 96 | T | C | T | 0.75806 | C | 0.24194 | 15 | 0.48387 |
| 8318 | S10_16612311 | 10 | 16612311 | 1 | A | G | A | 0.77419 | G | 0.22581 | 14 | 0.45161 |
| 8319 | S10_16612322 | 10 | 16612322 | 11 | C | G | C | 0.77419 | G | 0.22581 | 14 | 0.45161 |
| 8320 | S10_16612335 | 10 | 16612335 | 13 | G | A | G | 0.77419 | A | 0.22581 | 14 | 0.45161 |
| 8321 | S10_16612347 | 10 | 16612347 | 12 | A | G | A | 0.74194 | G | 0.25806 | 16 | 0.51613 |
| 8322 | S10_16612385 | 10 | 16612385 | 38 | C | G | C | 0.77419 | G | 0.22581 | 14 | 0.45161 |
| 8323 | S10_16612406 | 10 | 16612406 | 21 | C | T | C | 0.79032 | T | 0.20968 | 13 | 0.41935 |
| 8324 | S10_16731884 | 10 | 16731884 | 119478 | T | C | C | 0.72581 | T | 0.27419 | 15 | 0.48387 |
| 8325 | S10_16731905 | 10 | 16731905 | 21 | G | A | G | 0.59677 | A | 0.40323 | 15 | 0.48387 |
| 8326 | S10_16934087 | 10 | 16934087 | 202182 | G | T | G | 0.70968 | T | 0.29032 | 8 | 0.25806 |
| 8327 | S10_16934088 | 10 | 16934088 | 1 | G | T | G | 0.70968 | T | 0.29032 | 8 | 0.25806 |
| 8328 | S10_16945914 | 10 | 16945914 | 11826 | G | C | G | 0.90323 | C | 0.09677 | 6 | 0.19355 |
| 8329 | S10_16972134 | 10 | 16972134 | 26220 | A | G | G | 0.72581 | A | 0.27419 | 13 | 0.41935 |
| 8330 | S10_17065882 | 10 | 17065882 | 93748 | C | A | C | 0.95161 | A | 0.04839 | 3 | 0.09677 |
| 8331 | S10_17106556 | 10 | 17106556 | 40674 | G | T | G | 0.95161 | T | 0.04839 | 3 | 0.09677 |
| 8332 | S10_17212285 | 10 | 17212285 | 105729 | T | C | C | 0.56452 | T | 0.43548 | 13 | 0.41935 |
| 8333 | S10_17212322 | 10 | 17212322 | 37 | C | A | A | 0.56452 | C | 0.43548 | 13 | 0.41935 |
| 8334 | S10_17637951 | 10 | 17637951 | 425629 | A | G | A | 0.83871 | G | 0.16129 | 8 | 0.25806 |
| 8335 | S10_17638113 | 10 | 17638113 | 162 | A | G | A | 0.95161 | G | 0.04839 | 3 | 0.09677 |
| 8336 | S10_18368697 | 10 | 18368697 | 730584 | A | G | A | 0.91935 | G | 0.08065 | 5 | 0.16129 |
| 8337 | S10_18446851 | 10 | 18446851 | 78154 | A | G | A | 0.8871 | G | 0.1129 | 5 | 0.16129 |
| 8338 | S10_18446927 | 10 | 18446927 | 76 | T | A | T | 0.8871 | A | 0.1129 | 5 | 0.16129 |
| 8339 | S10_18601101 | 10 | 18601101 | 154174 | T | A | A | 0.95161 | T | 0.04839 | 1 | 0.03226 |
| 8340 | S10_18601248 | 10 | 18601248 | 147 | T | A | T | 0.87097 | A | 0.12903 | 6 | 0.19355 |
| 8341 | S10_18616621 | 10 | 18616621 | 15373 | G | A | G | 0.91935 | A | 0.08065 | 5 | 0.16129 |
| 8342 | S10_18616679 | 10 | 18616679 | 58 | C | G | C | 0.82258 | G | 0.17742 | 9 | 0.29032 |
| 8343 | S10_18616684 | 10 | 18616684 | 5 | C | T | C | 0.77419 | T | 0.22581 | 12 | 0.3871 |
| 8344 | S10_18616751 | 10 | 18616751 | 67 | C | T | C | 0.82258 | T | 0.17742 | 7 | 0.22581 |
| 8345 | S10_18616800 | 10 | 18616800 | 49 | G | T | G | 0.91935 | T | 0.08065 | 5 | 0.16129 |
| 8346 | S10_18909052 | 10 | 18909052 | 292252 | G | A | G | 0.93548 | A | 0.06452 | 4 | 0.12903 |
| 8347 | S10_19122933 | 10 | 19122933 | 213881 | G | A | G | 0.67742 | A | 0.32258 | 16 | 0.51613 |
| 8348 | S10_19122951 | 10 | 19122951 | 18 | C | T | C | 0.67742 | T | 0.32258 | 16 | 0.51613 |
| 8349 | S10_19122958 | 10 | 19122958 | 7 | T | C | T | 0.69355 | C | 0.30645 | 15 | 0.48387 |
| 8350 | S10_19123048 | 10 | 19123048 | 90 | C | T | C | 0.67742 | T | 0.32258 | 16 | 0.51613 |
| 8351 | S10_19123059 | 10 | 19123059 | 11 | G | A | G | 0.83871 | A | 0.16129 | 8 | 0.25806 |
| 8352 | S10_19448923 | 10 | 19448923 | 325864 | G | A | G | 0.74194 | A | 0.25806 | 12 | 0.3871 |
| 8353 | S10_19448989 | 10 | 19448989 | 66 | T | C | T | 0.90323 | C | 0.09677 | 4 | 0.12903 |
| 8354 | S10_19756042 | 10 | 19756042 | 307053 | C | G | C | 0.75806 | G | 0.24194 | 9 | 0.29032 |
| 8355 | S10_19756089 | 10 | 19756089 | 47 | A | G | G | 0.90323 | A | 0.09677 | 6 | 0.19355 |
| 8356 | S10_19780505 | 10 | 19780505 | 24416 | C | T | T | 0.82258 | C | 0.17742 | 9 | 0.29032 |
| 8357 | S10_19881180 | 10 | 19881180 | 100675 | C | A | C | 0.93548 | A | 0.06452 | 4 | 0.12903 |
| 8358 | S10_19881252 | 10 | 19881252 | 72 | C | T | T | 0.75806 | C | 0.24194 | 11 | 0.35484 |
| 8359 | S10_20141504 | 10 | 20141504 | 260252 | C | T | C | 0.90323 | T | 0.09677 | 4 | 0.12903 |
| 8360 | S10_20141575 | 10 | 20141575 | 71 | C | T | C | 0.8871 | T | 0.1129 | 7 | 0.22581 |
| 8361 | S10_20195720 | 10 | 20195720 | 54145 | G | C | G | 0.67742 | C | 0.32258 | 12 | 0.3871 |
| 8362 | S10_20208855 | 10 | 20208855 | 13135 | T | C | T | 0.77419 | C | 0.22581 | 10 | 0.32258 |
| 8363 | S10_20208861 | 10 | 20208861 | 6 | A | G | G | 0.74194 | A | 0.25806 | 12 | 0.3871 |
| 8364 | S10_20333106 | 10 | 20333106 | 124245 | G | A | G | 0.93548 | A | 0.06452 | 4 | 0.12903 |
| 8365 | S10_20333110 | 10 | 20333110 | 4 | G | A | G | 0.93548 | A | 0.06452 | 4 | 0.12903 |
| 8366 | S10_21009549 | 10 | 21009549 | 676439 | A | G | A | 0.85484 | G | 0.14516 | 7 | 0.22581 |
| 8367 | S10_21085161 | 10 | 21085161 | 75612 | G | A | G | 0.91935 | A | 0.08065 | 5 | 0.16129 |
| 8368 | S10_21085428 | 10 | 21085428 | 267 | C | T | C | 0.80645 | T | 0.19355 | 10 | 0.32258 |

| Site number | SNP name | Chromosome | Physical position | Physical distance from the previous SNP | Reference allele | Alternative allele | Major allele | Major allele frequency | Minor allele | Minor allele frequency | Number heterozygous | Heterozygosity |
|---|---|---|---|---|---|---|---|---|---|---|---|---|
| 8369 | S10_21085442 | 10 | 21085442 | 14 | G | A | G | 0.90323 | A | 0.09677 | 4 | 0.12903 |
| 8370 | S10_21085453 | 10 | 21085453 | 11 | T | C | T | 0.66129 | C | 0.33871 | 13 | 0.41935 |
| 8371 | S10_21085480 | 10 | 21085480 | 27 | G | A | G | 0.87097 | A | 0.12903 | 4 | 0.12903 |
| 8372 | S10_21131409 | 10 | 21131409 | 45929 | C | T | C | 0.85484 | T | 0.14516 | 9 | 0.29032 |
| 8373 | S10_21131495 | 10 | 21131495 | 86 | G | T | G | 0.85484 | T | 0.14516 | 9 | 0.29032 |
| 8374 | S10_21153371 | 10 | 21153371 | 21876 | A | G | A | 0.72581 | G | 0.27419 | 13 | 0.41935 |
| 8375 | S10_21153549 | 10 | 21153549 | 178 | A | G | A | 0.66129 | G | 0.33871 | 17 | 0.54839 |
| 8376 | S10_21175757 | 10 | 21175757 | 22208 | C | G | C | 0.95161 | G | 0.04839 | 3 | 0.09677 |
| 8377 | S10_21175854 | 10 | 21175854 | 97 | G | A | G | 0.6129 | A | 0.3871 | 12 | 0.3871 |
| 8378 | S10_21175914 | 10 | 21175914 | 60 | C | G | C | 0.82258 | G | 0.17742 | 7 | 0.22581 |
| 8379 | S10_21525088 | 10 | 21525088 | 349174 | T | C | C | 0.95161 | T | 0.04839 | 3 | 0.09677 |
| 8380 | S10_21684885 | 10 | 21684885 | 159797 | C | T | C | 0.90323 | T | 0.09677 | 4 | 0.12903 |
| 8381 | S10_21720258 | 10 | 21720258 | 35373 | C | G | C | 0.51613 | G | 0.48387 | 16 | 0.51613 |
| 8382 | S10_21766659 | 10 | 21766659 | 46401 | G | A | A | 0.5 | G | 0.5 | 13 | 0.41935 |
| 8383 | S10_21820654 | 10 | 21820654 | 53995 | G | C | G | 0.87097 | C | 0.12903 | 8 | 0.25806 |
| 8384 | S10_22180364 | 10 | 22180364 | 359710 | T | A | T | 0.91935 | A | 0.08065 | 3 | 0.09677 |
| 8385 | S10_22183377 | 10 | 22183377 | 3013 | T | C | T | 0.95161 | C | 0.04839 | 3 | 0.09677 |
| 8386 | S10_22320181 | 10 | 22320181 | 136804 | T | A | A | 0.67742 | T | 0.32258 | 12 | 0.3871 |
| 8387 | S10_22624411 | 10 | 22624411 | 304230 | C | T | T | 0.74194 | C | 0.25806 | 10 | 0.32258 |
| 8388 | S10_22624460 | 10 | 22624460 | 49 | G | C | G | 0.90323 | C | 0.09677 | 6 | 0.19355 |
| 8389 | S10_23500247 | 10 | 23500247 | 875787 | G | A | G | 0.69355 | A | 0.30645 | 15 | 0.48387 |
| 8390 | S10_23500253 | 10 | 23500253 | 6 | G | A | G | 0.70968 | A | 0.29032 | 10 | 0.32258 |
| 8391 | S10_23900545 | 10 | 23900545 | 400292 | C | T | C | 0.91935 | T | 0.08065 | 5 | 0.16129 |
| 8392 | S10_24442210 | 10 | 24442210 | 541665 | C | T | C | 0.95161 | T | 0.04839 | 3 | 0.09677 |
| 8393 | S10_24442217 | 10 | 24442217 | 7 | G | A | G | 0.91935 | A | 0.08065 | 5 | 0.16129 |
| 8394 | S10_24442231 | 10 | 24442231 | 14 | T | C | T | 0.93548 | C | 0.06452 | 4 | 0.12903 |
| 8395 | S10_24442445 | 10 | 24442445 | 214 | A | T | A | 0.58065 | T | 0.41935 | 18 | 0.58065 |
| 8396 | S10_25012211 | 10 | 25012211 | 569766 | G | A | A | 0.6129 | G | 0.3871 | 10 | 0.32258 |
| 8397 | S10_25072250 | 10 | 25072250 | 60039 | C | T | C | 0.85484 | T | 0.14516 | 7 | 0.22581 |
| 8398 | S10_25072313 | 10 | 25072313 | 63 | G | A | G | 0.83871 | A | 0.16129 | 8 | 0.25806 |
| 8399 | S10_25183310 | 10 | 25183310 | 110997 | C | A | A | 0.93548 | C | 0.06452 | 4 | 0.12903 |
| 8400 | S10_25337975 | 10 | 25337975 | 154665 | G | A | G | 0.85484 | A | 0.14516 | 5 | 0.16129 |
| 8401 | S10_25338021 | 10 | 25338021 | 46 | T | G | G | 0.56452 | T | 0.43548 | 15 | 0.48387 |
| 8402 | S10_26535784 | 10 | 26535784 | 1197763 | A | T | A | 0.85484 | T | 0.14516 | 7 | 0.22581 |
| 8403 | S10_26535875 | 10 | 26535875 | 91 | G | A | G | 0.95161 | A | 0.04839 | 3 | 0.09677 |
| 8404 | S10_26569475 | 10 | 26569475 | 33600 | T | C | T | 0.83871 | C | 0.16129 | 8 | 0.25806 |
| 8405 | S10_26569560 | 10 | 26569560 | 85 | T | C | T | 0.91935 | C | 0.08065 | 5 | 0.16129 |
| 8406 | S10_26569576 | 10 | 26569576 | 16 | G | C | G | 0.8871 | C | 0.1129 | 7 | 0.22581 |
| 8407 | S10_26601140 | 10 | 26601140 | 31564 | T | C | T | 0.58065 | C | 0.41935 | 10 | 0.32258 |
| 8408 | S10_27106513 | 10 | 27106513 | 505373 | A | C | A | 0.87097 | C | 0.12903 | 8 | 0.25806 |
| 8409 | S10_27106722 | 10 | 27106722 | 209 | A | T | A | 0.83871 | T | 0.16129 | 8 | 0.25806 |
| 8410 | S10_27185605 | 10 | 27185605 | 78883 | A | G | A | 0.95161 | G | 0.04839 | 3 | 0.09677 |
| 8411 | S10_27339744 | 10 | 27339744 | 154139 | A | G | A | 0.93548 | G | 0.06452 | 2 | 0.06452 |
| 8412 | S10_27531396 | 10 | 27531396 | 191652 | C | T | C | 0.69355 | T | 0.30645 | 9 | 0.29032 |
| 8413 | S10_27564368 | 10 | 27564368 | 32972 | G | T | T | 0.58065 | G | 0.41935 | 18 | 0.58065 |
| 8414 | S10_27564457 | 10 | 27564457 | 89 | C | T | C | 0.95161 | T | 0.04839 | 3 | 0.09677 |
| 8415 | S10_27564470 | 10 | 27564470 | 13 | G | A | A | 0.58065 | G | 0.41935 | 18 | 0.58065 |
| 8416 | S10_27728131 | 10 | 27728131 | 163661 | C | T | C | 0.95161 | T | 0.04839 | 3 | 0.09677 |
| 8417 | S10_27734731 | 10 | 27734731 | 6600 | C | T | C | 0.8871 | T | 0.1129 | 5 | 0.16129 |
| 8418 | S10_27932332 | 10 | 27932332 | 197601 | C | T | C | 0.93548 | T | 0.06452 | 4 | 0.12903 |
| 8419 | S10_27932343 | 10 | 27932343 | 11 | G | A | G | 0.90323 | A | 0.09677 | 6 | 0.19355 |
| 8420 | S10_27979792 | 10 | 27979792 | 47449 | C | A | C | 0.70968 | A | 0.29032 | 8 | 0.25806 |
| 8421 | S10_27979804 | 10 | 27979804 | 12 | A | G | A | 0.64516 | G | 0.35484 | 14 | 0.45161 |
| 8422 | S10_28373910 | 10 | 28373910 | 394106 | A | G | G | 0.8871 | A | 0.1129 | 7 | 0.22581 |

| Site number | SNP name | Chromosome | Physical position | Physical distance from the previous SNP | Reference allele | Alternative allele | Major allele | Major allele frequency | Minor allele | Minor allele frequency | Number heterozygous | Heterozygosity |
|---|---|---|---|---|---|---|---|---|---|---|---|---|
| 8423 | S10_28373953 | 10 | 28373953 | 43 | A | G | A | 0.91935 | G | 0.08065 | 5 | 0.16129 |
| 8424 | S10_28374046 | 10 | 28374046 | 93 | G | T | G | 0.90323 | T | 0.09677 | 6 | 0.19355 |
| 8425 | S10_28374108 | 10 | 28374108 | 62 | A | G | A | 0.90323 | G | 0.09677 | 6 | 0.19355 |
| 8426 | S10_28710896 | 10 | 28710896 | 336788 | A | G | A | 0.95161 | G | 0.04839 | 3 | 0.09677 |
| 8427 | S10_29013896 | 10 | 29013896 | 303000 | C | T | T | 0.64516 | C | 0.35484 | 10 | 0.32258 |
| 8428 | S10_29228602 | 10 | 29228602 | 214706 | T | A | A | 0.53226 | T | 0.46774 | 15 | 0.48387 |
| 8429 | S10_29228605 | 10 | 29228605 | 3 | A | C | C | 0.53226 | A | 0.46774 | 15 | 0.48387 |
| 8430 | S10_29228614 | 10 | 29228614 | 9 | T | C | C | 0.53226 | T | 0.46774 | 15 | 0.48387 |
| 8431 | S10_29228635 | 10 | 29228635 | 21 | G | C | C | 0.53226 | G | 0.46774 | 15 | 0.48387 |
| 8432 | S10_29626154 | 10 | 29626154 | 397519 | C | A | C | 0.93548 | A | 0.06452 | 4 | 0.12903 |
| 8433 | S10_29626319 | 10 | 29626319 | 165 | C | T | C | 0.91935 | T | 0.08065 | 5 | 0.16129 |
| 8434 | S10_29996563 | 10 | 29996563 | 370244 | C | T | T | 0.90323 | C | 0.09677 | 6 | 0.19355 |
| 8435 | S10_30137000 | 10 | 30137000 | 140437 | A | G | G | 0.56452 | A | 0.43548 | 13 | 0.41935 |
| 8436 | S10_30214399 | 10 | 30214399 | 77399 | G | A | G | 0.6129 | A | 0.3871 | 12 | 0.3871 |
| 8437 | S10_30214402 | 10 | 30214402 | 3 | T | C | T | 0.90323 | C | 0.09677 | 4 | 0.12903 |
| 8438 | S10_30214449 | 10 | 30214449 | 47 | G | A | G | 0.95161 | A | 0.04839 | 3 | 0.09677 |
| 8439 | S10_30642544 | 10 | 30642544 | 428095 | T | A | T | 0.93548 | A | 0.06452 | 4 | 0.12903 |
| 8440 | S10_30642589 | 10 | 30642589 | 45 | A | T | A | 0.93548 | T | 0.06452 | 4 | 0.12903 |
| 8441 | S10_30642703 | 10 | 30642703 | 114 | C | A | C | 0.69355 | A | 0.30645 | 15 | 0.48387 |
| 8442 | S10_30802382 | 10 | 30802382 | 159679 | C | T | C | 0.91935 | T | 0.08065 | 5 | 0.16129 |
| 8443 | S10_30802452 | 10 | 30802452 | 70 | G | A | G | 0.70968 | A | 0.29032 | 18 | 0.58065 |
| 8444 | S10_30802453 | 10 | 30802453 | 1 | C | T | C | 0.70968 | T | 0.29032 | 18 | 0.58065 |
| 8445 | S10_30802495 | 10 | 30802495 | 42 | G | T | G | 0.70968 | T | 0.29032 | 18 | 0.58065 |
| 8446 | S10_30856500 | 10 | 30856500 | 54005 | G | T | G | 0.72581 | T | 0.27419 | 11 | 0.35484 |
| 8447 | S10_30856609 | 10 | 30856609 | 109 | A | G | G | 0.72581 | A | 0.27419 | 11 | 0.35484 |
| 8448 | S10_31504143 | 10 | 31504143 | 647534 | C | T | C | 0.90323 | T | 0.09677 | 4 | 0.12903 |
| 8449 | S10_31504349 | 10 | 31504349 | 206 | T | C | T | 0.91935 | C | 0.08065 | 3 | 0.09677 |
| 8450 | S10_31558441 | 10 | 31558441 | 54092 | C | A | C | 0.83871 | A | 0.16129 | 6 | 0.19355 |
| 8451 | S10_31740713 | 10 | 31740713 | 182272 | G | C | G | 0.8871 | C | 0.1129 | 5 | 0.16129 |
| 8452 | S10_31740774 | 10 | 31740774 | 61 | T | C | T | 0.95161 | C | 0.04839 | 3 | 0.09677 |
| 8453 | S10_31740797 | 10 | 31740797 | 23 | G | A | A | 0.58065 | G | 0.41935 | 14 | 0.45161 |
| 8454 | S10_31740808 | 10 | 31740808 | 11 | G | A | A | 0.6129 | G | 0.3871 | 14 | 0.45161 |
| 8455 | S10_31740900 | 10 | 31740900 | 92 | C | G | G | 0.69355 | C | 0.30645 | 13 | 0.41935 |
| 8456 | S10_31744366 | 10 | 31744366 | 3466 | G | C | G | 0.8871 | C | 0.1129 | 5 | 0.16129 |
| 8457 | S10_32042887 | 10 | 32042887 | 298521 | C | T | C | 0.8871 | T | 0.1129 | 7 | 0.22581 |
| 8458 | S10_32060426 | 10 | 32060426 | 17539 | G | T | G | 0.90323 | T | 0.09677 | 4 | 0.12903 |
| 8459 | S10_32060454 | 10 | 32060454 | 28 | G | A | G | 0.8871 | A | 0.1129 | 7 | 0.22581 |
| 8460 | S10_32276705 | 10 | 32276705 | 216251 | A | G | A | 0.70968 | G | 0.29032 | 10 | 0.32258 |
| 8461 | S10_32587388 | 10 | 32587388 | 310683 | C | T | C | 0.90323 | T | 0.09677 | 4 | 0.12903 |
| 8462 | S10_32587403 | 10 | 32587403 | 15 | G | A | A | 0.91935 | G | 0.08065 | 5 | 0.16129 |
| 8463 | S10_32850083 | 10 | 32850083 | 262680 | C | G | C | 0.91935 | G | 0.08065 | 5 | 0.16129 |
| 8464 | S10_32850181 | 10 | 32850181 | 98 | T | C | T | 0.90323 | C | 0.09677 | 4 | 0.12903 |
| 8465 | S10_32931606 | 10 | 32931606 | 81425 | G | A | G | 0.67742 | A | 0.32258 | 14 | 0.45161 |
| 8466 | S10_32931608 | 10 | 32931608 | 2 | T | C | T | 0.58065 | C | 0.41935 | 14 | 0.45161 |
| 8467 | S10_32931641 | 10 | 32931641 | 33 | T | C | T | 0.67742 | C | 0.32258 | 14 | 0.45161 |
| 8468 | S10_32964305 | 10 | 32964305 | 32664 | A | G | G | 0.51613 | A | 0.48387 | 16 | 0.51613 |
| 8469 | S10_32964337 | 10 | 32964337 | 32 | C | T | T | 0.51613 | C | 0.48387 | 16 | 0.51613 |
| 8470 | S10_32964342 | 10 | 32964342 | 5 | G | C | C | 0.51613 | G | 0.48387 | 16 | 0.51613 |
| 8471 | S10_32964372 | 10 | 32964372 | 30 | G | A | A | 0.51613 | G | 0.48387 | 16 | 0.51613 |
| 8472 | S10_32964403 | 10 | 32964403 | 31 | A | G | G | 0.51613 | A | 0.48387 | 16 | 0.51613 |
| 8473 | S10_32964440 | 10 | 32964440 | 37 | C | T | T | 0.51613 | C | 0.48387 | 16 | 0.51613 |
| 8474 | S10_32964466 | 10 | 32964466 | 26 | C | T | T | 0.51613 | C | 0.48387 | 16 | 0.51613 |
| 8475 | S10_32964481 | 10 | 32964481 | 15 | G | A | A | 0.51613 | G | 0.48387 | 16 | 0.51613 |
| 8476 | S10_32964491 | 10 | 32964491 | 10 | A | C | C | 0.51613 | A | 0.48387 | 16 | 0.51613 |

| Site number | SNP name | Chromosome | Physical position | Physical distance from the previous SNP | Reference allele | Alternative allele | Major allele | Major allele frequency | Minor allele | Minor allele frequency | Number heterozygous | Heterozygosity |
|---|---|---|---|---|---|---|---|---|---|---|---|---|
| 8477 | S10_32964492 | 10 | 32964492 | 1 | G | A | A | 0.51613 | G | 0.48387 | 16 | 0.51613 |
| 8478 | S10_32964500 | 10 | 32964500 | 8 | C | T | C | 0.93548 | T | 0.06452 | 4 | 0.12903 |
| 8479 | S10_32964502 | 10 | 32964502 | 2 | T | C | C | 0.51613 | T | 0.48387 | 16 | 0.51613 |
| 8480 | S10_32964531 | 10 | 32964531 | 29 | A | T | T | 0.51613 | A | 0.48387 | 16 | 0.51613 |
| 8481 | S10_33003872 | 10 | 33003872 | 39341 | G | A | A | 0.53226 | G | 0.46774 | 15 | 0.48387 |
| 8482 | S10_33221641 | 10 | 33221641 | 217769 | T | G | T | 0.74194 | G | 0.25806 | 12 | 0.3871 |
| 8483 | S10_33221679 | 10 | 33221679 | 38 | C | T | C | 0.75806 | T | 0.24194 | 11 | 0.35484 |
| 8484 | S10_33221766 | 10 | 33221766 | 87 | G | T | G | 0.62903 | T | 0.37097 | 11 | 0.35484 |
| 8485 | S10_33451963 | 10 | 33451963 | 230197 | G | A | G | 0.95161 | A | 0.04839 | 3 | 0.09677 |
| 8486 | S10_33451994 | 10 | 33451994 | 31 | C | T | C | 0.82258 | T | 0.17742 | 7 | 0.22581 |
| 8487 | S10_33451995 | 10 | 33451995 | 1 | G | A | G | 0.95161 | A | 0.04839 | 3 | 0.09677 |
| 8488 | S10_33452066 | 10 | 33452066 | 71 | C | G | G | 0.74194 | C | 0.25806 | 12 | 0.3871 |
| 8489 | S10_33793800 | 10 | 33793800 | 341734 | T | C | C | 0.8871 | T | 0.1129 | 7 | 0.22581 |
| 8490 | S10_33835017 | 10 | 33835017 | 41217 | G | A | G | 0.56452 | A | 0.43548 | 17 | 0.54839 |
| 8491 | S10_33835135 | 10 | 33835135 | 118 | G | A | G | 0.95161 | A | 0.04839 | 3 | 0.09677 |
| 8492 | S10_33835213 | 10 | 33835213 | 78 | G | A | G | 0.87097 | A | 0.12903 | 8 | 0.25806 |
| 8493 | S10_33840033 | 10 | 33840033 | 4820 | G | A | G | 0.8871 | A | 0.1129 | 7 | 0.22581 |
| 8494 | S10_34087530 | 10 | 34087530 | 247497 | G | C | C | 0.80645 | G | 0.19355 | 12 | 0.3871 |
| 8495 | S10_34269890 | 10 | 34269890 | 182360 | A | G | A | 0.80645 | G | 0.19355 | 8 | 0.25806 |
| 8496 | S10_34832816 | 10 | 34832816 | 562926 | A | T | A | 0.95161 | T | 0.04839 | 3 | 0.09677 |
| 8497 | S10_34832844 | 10 | 34832844 | 28 | C | A | C | 0.69355 | A | 0.30645 | 15 | 0.48387 |
| 8498 | S10_34832847 | 10 | 34832847 | 3 | T | C | T | 0.90323 | C | 0.09677 | 6 | 0.19355 |
| 8499 | S10_34973471 | 10 | 34973471 | 140624 | T | C | T | 0.53226 | C | 0.46774 | 21 | 0.67742 |
| 8500 | S10_34973481 | 10 | 34973481 | 10 | G | A | G | 0.69355 | A | 0.30645 | 13 | 0.41935 |
| 8501 | S10_34973492 | 10 | 34973492 | 11 | C | G | C | 0.53226 | G | 0.46774 | 21 | 0.67742 |
| 8502 | S10_34973526 | 10 | 34973526 | 34 | A | G | A | 0.53226 | G | 0.46774 | 21 | 0.67742 |
| 8503 | S10_34973550 | 10 | 34973550 | 24 | A | G | A | 0.53226 | G | 0.46774 | 21 | 0.67742 |
| 8504 | S10_34973614 | 10 | 34973614 | 64 | A | G | A | 0.53226 | G | 0.46774 | 21 | 0.67742 |
| 8505 | S10_34973621 | 10 | 34973621 | 7 | T | A | T | 0.53226 | A | 0.46774 | 21 | 0.67742 |
| 8506 | S10_35604627 | 10 | 35604627 | 631006 | C | T | C | 0.79032 | T | 0.20968 | 13 | 0.41935 |
| 8507 | S10_35902458 | 10 | 35902458 | 297831 | G | C | G | 0.87097 | C | 0.12903 | 6 | 0.19355 |
| 8508 | S10_36476027 | 10 | 36476027 | 573569 | G | A | G | 0.93548 | A | 0.06452 | 2 | 0.06452 |
| 8509 | S10_36655768 | 10 | 36655768 | 179741 | C | T | C | 0.87097 | T | 0.12903 | 6 | 0.19355 |
| 8510 | S10_36655871 | 10 | 36655871 | 103 | C | T | C | 0.53226 | T | 0.46774 | 15 | 0.48387 |
| 8511 | S10_36655906 | 10 | 36655906 | 35 | C | G | C | 0.87097 | G | 0.12903 | 6 | 0.19355 |
| 8512 | S10_36849584 | 10 | 36849584 | 193678 | G | T | T | 0.56452 | G | 0.43548 | 13 | 0.41935 |
| 8513 | S10_36849754 | 10 | 36849754 | 170 | A | G | G | 0.64516 | A | 0.35484 | 14 | 0.45161 |
| 8514 | S10_37584369 | 10 | 37584369 | 734615 | C | A | C | 0.93548 | A | 0.06452 | 4 | 0.12903 |
| 8515 | S10_37971330 | 10 | 37971330 | 386961 | C | T | C | 0.87097 | T | 0.12903 | 8 | 0.25806 |
| 8516 | S10_38189693 | 10 | 38189693 | 218363 | T | G | G | 0.6129 | T | 0.3871 | 14 | 0.45161 |
| 8517 | S10_38189727 | 10 | 38189727 | 34 | G | T | T | 0.59677 | G | 0.40323 | 15 | 0.48387 |
| 8518 | S10_38386079 | 10 | 38386079 | 196352 | A | G | G | 0.91935 | A | 0.08065 | 3 | 0.09677 |
| 8519 | S10_38386177 | 10 | 38386177 | 98 | T | C | T | 0.8871 | C | 0.1129 | 5 | 0.16129 |
| 8520 | S10_38502727 | 10 | 38502727 | 116550 | A | G | A | 0.59677 | G | 0.40323 | 13 | 0.41935 |
| 8521 | S10_39240873 | 10 | 39240873 | 738146 | T | C | C | 0.91935 | T | 0.08065 | 5 | 0.16129 |
| 8522 | S10_39240936 | 10 | 39240936 | 63 | C | T | C | 0.95161 | T | 0.04839 | 3 | 0.09677 |
| 8523 | S10_39240955 | 10 | 39240955 | 19 | G | A | G | 0.95161 | A | 0.04839 | 3 | 0.09677 |
| 8524 | S10_39577272 | 10 | 39577272 | 336317 | C | T | C | 0.95161 | T | 0.04839 | 3 | 0.09677 |
| 8525 | S10_39577297 | 10 | 39577297 | 25 | T | G | T | 0.87097 | G | 0.12903 | 6 | 0.19355 |
| 8526 | S10_39963357 | 10 | 39963357 | 386060 | G | A | G | 0.79032 | A | 0.20968 | 7 | 0.22581 |
| 8527 | S10_39963416 | 10 | 39963416 | 59 | G | A | G | 0.93548 | A | 0.06452 | 4 | 0.12903 |
| 8528 | S10_39963436 | 10 | 39963436 | 20 | C | T | C | 0.95161 | T | 0.04839 | 3 | 0.09677 |
| 8529 | S10_40060693 | 10 | 40060693 | 97257 | C | T | C | 0.83871 | T | 0.16129 | 8 | 0.25806 |
| 8530 | S10_40060767 | 10 | 40060767 | 74 | C | G | G | 0.74194 | C | 0.25806 | 12 | 0.3871 |

| Site number | SNP name | Chromosome | Physical position | Physical distance from the previous SNP | Reference allele | Alternative allele | Major allele | Major allele frequency | Minor allele | Minor allele frequency | Number heterozygous | Heterozygosity |
|---|---|---|---|---|---|---|---|---|---|---|---|---|
| 8531 | S10_40297504 | 10 | 40297504 | 236737 | T | C | T | 0.93548 | C | 0.06452 | 4 | 0.12903 |
| 8532 | S10_40297530 | 10 | 40297530 | 26 | G | A | G | 0.93548 | A | 0.06452 | 4 | 0.12903 |
| 8533 | S10_40413691 | 10 | 40413691 | 116161 | C | T | C | 0.95161 | T | 0.04839 | 3 | 0.09677 |
| 8534 | S10_40413697 | 10 | 40413697 | 6 | C | T | C | 0.80645 | T | 0.19355 | 8 | 0.25806 |
| 8535 | S10_40413699 | 10 | 40413699 | 2 | G | A | G | 0.59677 | A | 0.40323 | 11 | 0.35484 |
| 8536 | S10_40413721 | 10 | 40413721 | 22 | G | A | G | 0.87097 | A | 0.12903 | 6 | 0.19355 |
| 8537 | S10_40413850 | 10 | 40413850 | 129 | G | C | G | 0.87097 | C | 0.12903 | 6 | 0.19355 |
| 8538 | S10_40413865 | 10 | 40413865 | 15 | C | T | C | 0.91935 | T | 0.08065 | 5 | 0.16129 |
| 8539 | S10_40633451 | 10 | 40633451 | 219586 | C | T | C | 0.8871 | T | 0.1129 | 7 | 0.22581 |
| 8540 | S10_41331776 | 10 | 41331776 | 698325 | C | T | C | 0.95161 | T | 0.04839 | 3 | 0.09677 |
| 8541 | S10_41454858 | 10 | 41454858 | 123082 | C | T | C | 0.95161 | T | 0.04839 | 3 | 0.09677 |
| 8542 | S10_41454863 | 10 | 41454863 | 5 | C | T | C | 0.95161 | T | 0.04839 | 3 | 0.09677 |
| 8543 | S10_41454882 | 10 | 41454882 | 19 | C | T | C | 0.93548 | T | 0.06452 | 4 | 0.12903 |
| 8544 | S10_41454904 | 10 | 41454904 | 22 | A | C | A | 0.83871 | C | 0.16129 | 10 | 0.32258 |
| 8545 | S10_41493707 | 10 | 41493707 | 38803 | G | A | G | 0.83871 | A | 0.16129 | 8 | 0.25806 |
| 8546 | S10_41493744 | 10 | 41493744 | 37 | G | A | G | 0.87097 | A | 0.12903 | 6 | 0.19355 |
| 8547 | S10_41493856 | 10 | 41493856 | 112 | T | C | T | 0.83871 | C | 0.16129 | 8 | 0.25806 |
| 8548 | S10_41804103 | 10 | 41804103 | 310247 | T | C | T | 0.91935 | C | 0.08065 | 5 | 0.16129 |
| 8549 | S10_43096474 | 10 | 43096474 | 1292371 | G | A | G | 0.80645 | A | 0.19355 | 8 | 0.25806 |
| 8550 | S10_43212228 | 10 | 43212228 | 115754 | G | A | G | 0.95161 | A | 0.04839 | 3 | 0.09677 |
| 8551 | S10_43487588 | 10 | 43487588 | 275360 | T | C | T | 0.93548 | C | 0.06452 | 4 | 0.12903 |
| 8552 | S10_43487647 | 10 | 43487647 | 59 | C | T | C | 0.95161 | T | 0.04839 | 3 | 0.09677 |
| 8553 | S10_43553944 | 10 | 43553944 | 66297 | A | G | G | 0.72581 | A | 0.27419 | 11 | 0.35484 |
| 8554 | S10_43554119 | 10 | 43554119 | 175 | T | C | T | 0.77419 | C | 0.22581 | 10 | 0.32258 |
| 8555 | S10_44645172 | 10 | 44645172 | 1091053 | G | A | G | 0.80645 | A | 0.19355 | 12 | 0.3871 |
| 8556 | S10_44717699 | 10 | 44717699 | 72527 | G | T | G | 0.95161 | T | 0.04839 | 1 | 0.03226 |
| 8557 | S10_44717748 | 10 | 44717748 | 49 | A | G | A | 0.59677 | G | 0.40323 | 11 | 0.35484 |
| 8558 | S10_44717749 | 10 | 44717749 | 1 | C | T | C | 0.80645 | T | 0.19355 | 8 | 0.25806 |
| 8559 | S10_45937527 | 10 | 45937527 | 1219778 | C | T | C | 0.91935 | T | 0.08065 | 5 | 0.16129 |
| 8560 | S10_45937528 | 10 | 45937528 | 1 | G | A | G | 0.87097 | A | 0.12903 | 6 | 0.19355 |
| 8561 | S10_45937543 | 10 | 45937543 | 15 | C | T | C | 0.91935 | T | 0.08065 | 5 | 0.16129 |
| 8562 | S10_46837177 | 10 | 46837177 | 899634 | T | C | C | 0.80645 | T | 0.19355 | 8 | 0.25806 |
| 8563 | S10_47184976 | 10 | 47184976 | 347799 | G | C | C | 0.58065 | G | 0.41935 | 12 | 0.3871 |
| 8564 | S10_47184997 | 10 | 47184997 | 21 | G | A | G | 0.95161 | A | 0.04839 | 3 | 0.09677 |
| 8565 | S10_47185051 | 10 | 47185051 | 54 | G | A | G | 0.80645 | A | 0.19355 | 8 | 0.25806 |
| 8566 | S10_47343103 | 10 | 47343103 | 158052 | T | C | C | 0.54839 | T | 0.45161 | 18 | 0.58065 |
| 8567 | S10_47343173 | 10 | 47343173 | 70 | A | G | A | 0.90323 | G | 0.09677 | 6 | 0.19355 |
| 8568 | S10_47343261 | 10 | 47343261 | 88 | A | C | C | 0.53226 | A | 0.46774 | 17 | 0.54839 |
| 8569 | S10_47816062 | 10 | 47816062 | 472801 | G | C | C | 0.51613 | G | 0.48387 | 16 | 0.51613 |
| 8570 | S10_47816126 | 10 | 47816126 | 64 | T | C | C | 0.93548 | T | 0.06452 | 4 | 0.12903 |
| 8571 | S10_47816144 | 10 | 47816144 | 18 | G | A | A | 0.91935 | G | 0.08065 | 5 | 0.16129 |
| 8572 | S10_47872772 | 10 | 47872772 | 56628 | C | T | C | 0.6129 | T | 0.3871 | 18 | 0.58065 |
| 8573 | S10_47872808 | 10 | 47872808 | 36 | C | T | C | 0.85484 | T | 0.14516 | 9 | 0.29032 |
| 8574 | S10_47872818 | 10 | 47872818 | 10 | C | A | C | 0.85484 | A | 0.14516 | 9 | 0.29032 |
| 8575 | S10_47872826 | 10 | 47872826 | 8 | C | T | T | 0.77419 | C | 0.22581 | 10 | 0.32258 |
| 8576 | S10_48780053 | 10 | 48780053 | 907227 | T | C | T | 0.74194 | C | 0.25806 | 10 | 0.32258 |
| 8577 | S10_48847192 | 10 | 48847192 | 67139 | T | C | T | 0.70968 | C | 0.29032 | 12 | 0.3871 |
| 8578 | S10_48847210 | 10 | 48847210 | 18 | G | A | G | 0.83871 | A | 0.16129 | 8 | 0.25806 |
| 8579 | S10_49362156 | 10 | 49362156 | 514946 | T | C | C | 0.69355 | T | 0.30645 | 17 | 0.54839 |
| 8580 | S10_50225823 | 10 | 50225823 | 863667 | C | T | C | 0.8871 | T | 0.1129 | 5 | 0.16129 |
| 8581 | S10_50225850 | 10 | 50225850 | 27 | G | A | G | 0.91935 | A | 0.08065 | 5 | 0.16129 |
| 8582 | S10_50313616 | 10 | 50313616 | 87766 | C | G | G | 0.54839 | C | 0.45161 | 18 | 0.58065 |
| 8583 | S10_50313639 | 10 | 50313639 | 23 | G | A | A | 0.53226 | G | 0.46774 | 19 | 0.6129 |
| 8584 | S10_50313652 | 10 | 50313652 | 13 | T | C | C | 0.53226 | T | 0.46774 | 19 | 0.6129 |

| Site number | SNP name | Chromosome | Physical position | Physical distance from the previous SNP | Reference allele | Alternative allele | Major allele | Major allele frequency | Minor allele | Minor allele frequency | Number heterozygous | Heterozygosity |
|---|---|---|---|---|---|---|---|---|---|---|---|---|
| 8585 | S10_50405837 | 10 | 50405837 | 92185 | T | C | T | 0.83871 | C | 0.16129 | 10 | 0.32258 |
| 8586 | S10_50620301 | 10 | 50620301 | 214464 | A | G | A | 0.93548 | G | 0.06452 | 4 | 0.12903 |
| 8587 | S10_51115686 | 10 | 51115686 | 495385 | A | T | A | 0.74194 | T | 0.25806 | 12 | 0.3871 |
| 8588 | S10_51235581 | 10 | 51235581 | 119895 | T | C | C | 0.91935 | T | 0.08065 | 5 | 0.16129 |
| 8589 | S10_51235722 | 10 | 51235722 | 141 | T | C | C | 0.91935 | T | 0.08065 | 5 | 0.16129 |
| 8590 | S10_51354333 | 10 | 51354333 | 118611 | T | C | C | 0.66129 | T | 0.33871 | 13 | 0.41935 |
| 8591 | S10_51983528 | 10 | 51983528 | 629195 | T | A | A | 0.53226 | T | 0.46774 | 15 | 0.48387 |
| 8592 | S10_52540838 | 10 | 52540838 | 557310 | C | T | C | 0.93548 | T | 0.06452 | 2 | 0.06452 |
| 8593 | S10_52659825 | 10 | 52659825 | 118987 | C | T | C | 0.72581 | T | 0.27419 | 13 | 0.41935 |
| 8594 | S10_52659827 | 10 | 52659827 | 2 | A | G | A | 0.90323 | G | 0.09677 | 6 | 0.19355 |
| 8595 | S10_52659904 | 10 | 52659904 | 77 | A | G | G | 0.79032 | A | 0.20968 | 13 | 0.41935 |
| 8596 | S10_52659920 | 10 | 52659920 | 16 | C | T | C | 0.91935 | T | 0.08065 | 5 | 0.16129 |
| 8597 | S10_52684867 | 10 | 52684867 | 24947 | C | T | C | 0.95161 | T | 0.04839 | 3 | 0.09677 |
| 8598 | S10_52684875 | 10 | 52684875 | 8 | G | A | G | 0.79032 | A | 0.20968 | 13 | 0.41935 |
| 8599 | S10_52684882 | 10 | 52684882 | 7 | G | A | A | 0.8871 | G | 0.1129 | 7 | 0.22581 |
| 8600 | S10_52684908 | 10 | 52684908 | 26 | G | A | G | 0.79032 | A | 0.20968 | 13 | 0.41935 |
| 8601 | S10_52684919 | 10 | 52684919 | 11 | C | A | C | 0.79032 | A | 0.20968 | 13 | 0.41935 |
| 8602 | S10_52685041 | 10 | 52685041 | 122 | C | G | C | 0.79032 | G | 0.20968 | 13 | 0.41935 |
| 8603 | S10_52999868 | 10 | 52999868 | 314827 | C | A | C | 0.51613 | A | 0.48387 | 22 | 0.70968 |
| 8604 | S10_52999886 | 10 | 52999886 | 18 | C | T | C | 0.54839 | T | 0.45161 | 22 | 0.70968 |
| 8605 | S10_52999887 | 10 | 52999887 | 1 | G | A | G | 0.87097 | A | 0.12903 | 8 | 0.25806 |
| 8606 | S10_53041074 | 10 | 53041074 | 41187 | T | G | G | 0.80645 | T | 0.19355 | 12 | 0.3871 |
| 8607 | S10_53041126 | 10 | 53041126 | 52 | G | A | G | 0.90323 | A | 0.09677 | 6 | 0.19355 |
| 8608 | S10_53041222 | 10 | 53041222 | 96 | G | A | G | 0.82258 | A | 0.17742 | 11 | 0.35484 |
| 8609 | S10_53041242 | 10 | 53041242 | 20 | G | A | G | 0.87097 | A | 0.12903 | 8 | 0.25806 |
| 8610 | S10_53158549 | 10 | 53158549 | 117307 | G | A | G | 0.80645 | A | 0.19355 | 12 | 0.3871 |
| 8611 | S10_53164408 | 10 | 53164408 | 5859 | A | C | C | 0.95161 | A | 0.04839 | 1 | 0.03226 |
| 8612 | S10_53164495 | 10 | 53164495 | 87 | G | A | G | 0.83871 | A | 0.16129 | 10 | 0.32258 |
| 8613 | S10_53205598 | 10 | 53205598 | 41103 | G | A | G | 0.8871 | A | 0.1129 | 5 | 0.16129 |
| 8614 | S10_53205616 | 10 | 53205616 | 18 | A | T | T | 0.74194 | A | 0.25806 | 12 | 0.3871 |
| 8615 | S10_53205775 | 10 | 53205775 | 159 | C | T | C | 0.66129 | T | 0.33871 | 13 | 0.41935 |
| 8616 | S10_53205799 | 10 | 53205799 | 24 | A | C | C | 0.72581 | A | 0.27419 | 11 | 0.35484 |
| 8617 | S10_54195360 | 10 | 54195360 | 989561 | C | T | C | 0.8871 | T | 0.1129 | 5 | 0.16129 |
| 8618 | S10_54195583 | 10 | 54195583 | 223 | C | T | C | 0.83871 | T | 0.16129 | 8 | 0.25806 |
| 8619 | S10_54570231 | 10 | 54570231 | 374648 | C | T | C | 0.93548 | T | 0.06452 | 4 | 0.12903 |
| 8620 | S10_54570237 | 10 | 54570237 | 6 | A | G | A | 0.75806 | G | 0.24194 | 13 | 0.41935 |
| 8621 | S10_54570274 | 10 | 54570274 | 37 | C | T | T | 0.53226 | C | 0.46774 | 17 | 0.54839 |
| 8622 | S10_54570374 | 10 | 54570374 | 100 | C | T | C | 0.93548 | T | 0.06452 | 4 | 0.12903 |
| 8623 | S10_54836362 | 10 | 54836362 | 265988 | A | G | A | 0.95161 | G | 0.04839 | 3 | 0.09677 |
| 8624 | S10_54836462 | 10 | 54836462 | 100 | G | A | G | 0.67742 | A | 0.32258 | 14 | 0.45161 |
| 8625 | S10_55171932 | 10 | 55171932 | 335470 | G | A | G | 0.85484 | A | 0.14516 | 7 | 0.22581 |
| 8626 | S10_55405234 | 10 | 55405234 | 233302 | C | G | C | 0.77419 | G | 0.22581 | 12 | 0.3871 |
| 8627 | S10_55405240 | 10 | 55405240 | 6 | G | A | G | 0.91935 | A | 0.08065 | 5 | 0.16129 |
| 8628 | S10_55405247 | 10 | 55405247 | 7 | C | T | C | 0.64516 | T | 0.35484 | 16 | 0.51613 |
| 8629 | S10_55405249 | 10 | 55405249 | 2 | G | C | G | 0.91935 | C | 0.08065 | 5 | 0.16129 |
| 8630 | S10_55405345 | 10 | 55405345 | 96 | C | T | C | 0.82258 | T | 0.17742 | 9 | 0.29032 |
| 8631 | S10_55538762 | 10 | 55538762 | 133417 | C | G | C | 0.82258 | G | 0.17742 | 7 | 0.22581 |
| 8632 | S10_55641828 | 10 | 55641828 | 103066 | T | G | T | 0.80645 | G | 0.19355 | 10 | 0.32258 |
| 8633 | S10_55692937 | 10 | 55692937 | 51109 | T | C | C | 0.8871 | T | 0.1129 | 7 | 0.22581 |
| 8634 | S10_55759494 | 10 | 55759494 | 66557 | C | T | C | 0.75806 | T | 0.24194 | 13 | 0.41935 |
| 8635 | S10_55759539 | 10 | 55759539 | 45 | C | T | C | 0.79032 | T | 0.20968 | 11 | 0.35484 |
| 8636 | S10_56107506 | 10 | 56107506 | 347967 | C | T | C | 0.95161 | T | 0.04839 | 3 | 0.09677 |
| 8637 | S10_56775499 | 10 | 56775499 | 667993 | C | T | C | 0.95161 | T | 0.04839 | 3 | 0.09677 |
| 8638 | S10_57069989 | 10 | 57069989 | 294490 | G | A | G | 0.95161 | A | 0.04839 | 3 | 0.09677 |

| Site number | SNP name | Chromosome | Physical position | Physical distance from the previous SNP | Reference allele | Alternative allele | Major allele | Major allele frequency | Minor allele | Minor allele frequency | Number heterozygous | Heterozygosity |
|---|---|---|---|---|---|---|---|---|---|---|---|---|
| 8639 | S10_57070113 | 10 | 57070113 | 124 | T | C | C | 0.62903 | T | 0.37097 | 13 | 0.41935 |
| 8640 | S10_57070191 | 10 | 57070191 | 78 | T | C | T | 0.74194 | C | 0.25806 | 10 | 0.32258 |
| 8641 | S10_57114899 | 10 | 57114899 | 44708 | C | T | T | 0.70968 | C | 0.29032 | 14 | 0.45161 |
| 8642 | S10_57114989 | 10 | 57114989 | 90 | G | A | G | 0.91935 | A | 0.08065 | 5 | 0.16129 |
| 8643 | S10_57606621 | 10 | 57606621 | 491632 | A | G | A | 0.87097 | G | 0.12903 | 8 | 0.25806 |
| 8644 | S10_57606836 | 10 | 57606836 | 215 | G | A | G | 0.90323 | A | 0.09677 | 6 | 0.19355 |
| 8645 | S10_57844075 | 10 | 57844075 | 237239 | C | T | C | 0.95161 | T | 0.04839 | 3 | 0.09677 |
| 8646 | S10_57966415 | 10 | 57966415 | 122340 | T | C | T | 0.87097 | C | 0.12903 | 6 | 0.19355 |
| 8647 | S10_57979716 | 10 | 57979716 | 13301 | G | A | A | 0.83871 | G | 0.16129 | 10 | 0.32258 |
| 8648 | S10_58213407 | 10 | 58213407 | 233691 | G | A | G | 0.8871 | A | 0.1129 | 7 | 0.22581 |
| 8649 | S10_58409357 | 10 | 58409357 | 195950 | G | A | G | 0.80645 | A | 0.19355 | 10 | 0.32258 |
| 8650 | S10_58409421 | 10 | 58409421 | 64 | A | C | A | 0.82258 | C | 0.17742 | 9 | 0.29032 |
| 8651 | S10_58409467 | 10 | 58409467 | 46 | T | C | T | 0.82258 | C | 0.17742 | 9 | 0.29032 |
| 8652 | S10_58409499 | 10 | 58409499 | 32 | G | A | G | 0.82258 | A | 0.17742 | 9 | 0.29032 |
| 8653 | S10_58495627 | 10 | 58495627 | 86128 | T | C | T | 0.8871 | C | 0.1129 | 7 | 0.22581 |
| 8654 | S10_58495717 | 10 | 58495717 | 90 | C | A | C | 0.8871 | A | 0.1129 | 7 | 0.22581 |
| 8655 | S10_58495843 | 10 | 58495843 | 126 | G | T | G | 0.8871 | T | 0.1129 | 7 | 0.22581 |
| 8656 | S10_58683287 | 10 | 58683287 | 187444 | G | A | G | 0.58065 | A | 0.41935 | 10 | 0.32258 |
| 8657 | S10_58683329 | 10 | 58683329 | 42 | T | C | T | 0.58065 | C | 0.41935 | 10 | 0.32258 |
| 8658 | S10_58693613 | 10 | 58693613 | 10284 | G | A | G | 0.74194 | A | 0.25806 | 10 | 0.32258 |
| 8659 | S10_58916576 | 10 | 58916576 | 222963 | C | T | C | 0.95161 | T | 0.04839 | 3 | 0.09677 |
| 8660 | S10_58916649 | 10 | 58916649 | 73 | T | C | T | 0.91935 | C | 0.08065 | 5 | 0.16129 |
| 8661 | S10_58916712 | 10 | 58916712 | 63 | C | T | C | 0.91935 | T | 0.08065 | 5 | 0.16129 |
| 8662 | S10_59081215 | 10 | 59081215 | 164503 | A | G | G | 0.67742 | A | 0.32258 | 16 | 0.51613 |
| 8663 | S10_59081231 | 10 | 59081231 | 16 | A | G | G | 0.67742 | A | 0.32258 | 16 | 0.51613 |
| 8664 | S10_59233284 | 10 | 59233284 | 152053 | A | G | A | 0.90323 | G | 0.09677 | 6 | 0.19355 |
| 8665 | S10_59233294 | 10 | 59233294 | 10 | C | T | C | 0.51613 | T | 0.48387 | 14 | 0.45161 |
| 8666 | S10_59233338 | 10 | 59233338 | 44 | A | C | A | 0.93548 | C | 0.06452 | 4 | 0.12903 |
| 8667 | S10_59233417 | 10 | 59233417 | 79 | T | A | T | 0.90323 | A | 0.09677 | 6 | 0.19355 |
| 8668 | S10_60047643 | 10 | 60047643 | 814226 | G | T | G | 0.91935 | T | 0.08065 | 5 | 0.16129 |
| 8669 | S10_60047745 | 10 | 60047745 | 102 | A | C | C | 0.91935 | A | 0.08065 | 5 | 0.16129 |
| 8670 | S10_60047798 | 10 | 60047798 | 53 | T | C | T | 0.95161 | C | 0.04839 | 3 | 0.09677 |
| 8671 | S10_60124610 | 10 | 60124610 | 76812 | G | A | G | 0.70968 | A | 0.29032 | 12 | 0.3871 |
| 8672 | S10_60124617 | 10 | 60124617 | 7 | A | G | A | 0.90323 | G | 0.09677 | 6 | 0.19355 |
| 8673 | S10_60506568 | 10 | 60506568 | 381951 | G | A | G | 0.82258 | A | 0.17742 | 9 | 0.29032 |
| 8674 | S10_60736747 | 10 | 60736747 | 230179 | T | C | T | 0.85484 | C | 0.14516 | 9 | 0.29032 |
| 8675 | S10_60748190 | 10 | 60748190 | 11443 | C | T | C | 0.95161 | T | 0.04839 | 3 | 0.09677 |
| 8676 | S10_60810359 | 10 | 60810359 | 62169 | C | T | C | 0.93548 | T | 0.06452 | 4 | 0.12903 |
| 8677 | S10_60948958 | 10 | 60948958 | 138599 | G | A | G | 0.70968 | A | 0.29032 | 14 | 0.45161 |
| 8678 | S10_60949091 | 10 | 60949091 | 133 | C | A | C | 0.90323 | A | 0.09677 | 6 | 0.19355 |
| 8679 | S10_61011932 | 10 | 61011932 | 62841 | T | C | T | 0.79032 | C | 0.20968 | 11 | 0.35484 |
| 8680 | S10_61011965 | 10 | 61011965 | 33 | T | C | T | 0.8871 | C | 0.1129 | 5 | 0.16129 |
| 8681 | S10_61012104 | 10 | 61012104 | 139 | A | C | A | 0.79032 | C | 0.20968 | 11 | 0.35484 |
| 8682 | S10_62912217 | 10 | 62912217 | 1900113 | G | A | G | 0.95161 | A | 0.04839 | 3 | 0.09677 |
| 8683 | S10_63288127 | 10 | 63288127 | 375910 | T | C | T | 0.95161 | C | 0.04839 | 1 | 0.03226 |
| 8684 | S10_63288153 | 10 | 63288153 | 26 | G | T | G | 0.80645 | T | 0.19355 | 10 | 0.32258 |
| 8685 | S10_63288218 | 10 | 63288218 | 65 | T | C | T | 0.80645 | C | 0.19355 | 10 | 0.32258 |
| 8686 | S10_63288228 | 10 | 63288228 | 10 | C | A | C | 0.93548 | A | 0.06452 | 4 | 0.12903 |
| 8687 | S10_63288253 | 10 | 63288253 | 25 | C | T | C | 0.87097 | T | 0.12903 | 8 | 0.25806 |
| 8688 | S10_63288283 | 10 | 63288283 | 30 | A | C | C | 0.74194 | A | 0.25806 | 12 | 0.3871 |
| 8689 | S10_63951369 | 10 | 63951369 | 663086 | T | C | T | 0.8871 | C | 0.1129 | 7 | 0.22581 |
| 8690 | S10_63951426 | 10 | 63951426 | 57 | C | T | C | 0.85484 | T | 0.14516 | 9 | 0.29032 |
| 8691 | S10_63951443 | 10 | 63951443 | 17 | G | A | G | 0.8871 | A | 0.1129 | 7 | 0.22581 |
| 8692 | S10_64413875 | 10 | 64413875 | 462432 | A | G | G | 0.93548 | A | 0.06452 | 4 | 0.12903 |

| Site number | SNP name | Chromosome | Physical position | Physical distance from the previous SNP | Reference allele | Alternative allele | Major allele | Major allele frequency | Minor allele | Minor allele frequency | Number heterozygous | Heterozygosity |
|---|---|---|---|---|---|---|---|---|---|---|---|---|
| 8693 | S10_64582510 | 10 | 64582510 | 168635 | G | A | G | 0.82258 | A | 0.17742 | 9 | 0.29032 |
| 8694 | S10_64582587 | 10 | 64582587 | 77 | C | T | C | 0.82258 | T | 0.17742 | 9 | 0.29032 |
| 8695 | S10_64582603 | 10 | 64582603 | 16 | C | T | C | 0.77419 | T | 0.22581 | 12 | 0.3871 |
| 8696 | S10_65358957 | 10 | 65358957 | 776354 | G | T | G | 0.90323 | T | 0.09677 | 6 | 0.19355 |
| 8697 | S10_65359008 | 10 | 65359008 | 51 | G | A | G | 0.95161 | A | 0.04839 | 3 | 0.09677 |
| 8698 | S10_65412212 | 10 | 65412212 | 53204 | C | T | C | 0.91935 | T | 0.08065 | 3 | 0.09677 |
| 8699 | S10_65412226 | 10 | 65412226 | 14 | T | C | T | 0.91935 | C | 0.08065 | 3 | 0.09677 |
| 8700 | S10_65412369 | 10 | 65412369 | 143 | G | A | G | 0.8871 | A | 0.1129 | 7 | 0.22581 |
| 8701 | S10_66231050 | 10 | 66231050 | 818681 | A | G | A | 0.51613 | G | 0.48387 | 16 | 0.51613 |
| 8702 | S10_66573795 | 10 | 66573795 | 342745 | G | A | G | 0.90323 | A | 0.09677 | 6 | 0.19355 |
| 8703 | S10_66649485 | 10 | 66649485 | 75690 | A | G | A | 0.56452 | G | 0.43548 | 19 | 0.6129 |
| 8704 | S10_66722406 | 10 | 66722406 | 72921 | T | C | T | 0.95161 | C | 0.04839 | 3 | 0.09677 |
| 8705 | S10_66785060 | 10 | 66785060 | 62654 | G | A | G | 0.87097 | A | 0.12903 | 6 | 0.19355 |
| 8706 | S10_66909440 | 10 | 66909440 | 124380 | C | A | C | 0.79032 | A | 0.20968 | 13 | 0.41935 |
| 8707 | S10_66945012 | 10 | 66945012 | 35572 | G | A | G | 0.58065 | A | 0.41935 | 18 | 0.58065 |
| 8708 | S10_66945084 | 10 | 66945084 | 72 | G | C | C | 0.5 | G | 0.5 | 15 | 0.48387 |
| 8709 | S10_66945212 | 10 | 66945212 | 128 | C | A | C | 0.93548 | A | 0.06452 | 4 | 0.12903 |
| 8710 | S10_67432757 | 10 | 67432757 | 487545 | A | G | A | 0.91935 | G | 0.08065 | 5 | 0.16129 |
| 8711 | S10_67619320 | 10 | 67619320 | 186563 | T | C | T | 0.83871 | C | 0.16129 | 10 | 0.32258 |
| 8712 | S10_67753221 | 10 | 67753221 | 133901 | G | C | G | 0.8871 | C | 0.1129 | 7 | 0.22581 |
| 8713 | S10_67753246 | 10 | 67753246 | 25 | G | A | G | 0.70968 | A | 0.29032 | 12 | 0.3871 |
| 8714 | S10_67753279 | 10 | 67753279 | 33 | A | G | A | 0.83871 | G | 0.16129 | 10 | 0.32258 |
| 8715 | S10_67792638 | 10 | 67792638 | 39359 | T | C | C | 0.95161 | T | 0.04839 | 3 | 0.09677 |
| 8716 | S10_67792800 | 10 | 67792800 | 162 | G | A | G | 0.70968 | A | 0.29032 | 16 | 0.51613 |
| 8717 | S10_67911596 | 10 | 67911596 | 118796 | G | A | G | 0.95161 | A | 0.04839 | 3 | 0.09677 |
| 8718 | S10_68111350 | 10 | 68111350 | 199754 | T | A | A | 0.51613 | T | 0.48387 | 12 | 0.3871 |
| 8719 | S10_68111454 | 10 | 68111454 | 104 | C | T | T | 0.56452 | C | 0.43548 | 13 | 0.41935 |
| 8720 | S10_68111471 | 10 | 68111471 | 17 | C | T | C | 0.5 | T | 0.5 | 13 | 0.41935 |
| 8721 | S10_68983420 | 10 | 68983420 | 871949 | C | T | C | 0.85484 | T | 0.14516 | 7 | 0.22581 |
| 8722 | S10_69136476 | 10 | 69136476 | 153056 | T | C | T | 0.83871 | C | 0.16129 | 10 | 0.32258 |
| 8723 | S10_69136690 | 10 | 69136690 | 214 | C | A | C | 0.85484 | A | 0.14516 | 9 | 0.29032 |
| 8724 | S10_69446709 | 10 | 69446709 | 310019 | G | A | G | 0.95161 | A | 0.04839 | 3 | 0.09677 |
| 8725 | S10_69446814 | 10 | 69446814 | 105 | G | A | G | 0.91935 | A | 0.08065 | 5 | 0.16129 |
| 8726 | S10_69446862 | 10 | 69446862 | 48 | T | C | T | 0.91935 | C | 0.08065 | 5 | 0.16129 |
| 8727 | S10_69712637 | 10 | 69712637 | 265775 | G | A | G | 0.91935 | A | 0.08065 | 5 | 0.16129 |
| 8728 | S10_69712656 | 10 | 69712656 | 19 | C | T | C | 0.91935 | T | 0.08065 | 5 | 0.16129 |
| 8729 | S10_69712657 | 10 | 69712657 | 1 | A | G | G | 0.64516 | A | 0.35484 | 14 | 0.45161 |
| 8730 | S10_69712723 | 10 | 69712723 | 66 | C | T | C | 0.83871 | T | 0.16129 | 8 | 0.25806 |
| 8731 | S10_70166721 | 10 | 70166721 | 453998 | C | T | T | 0.64516 | C | 0.35484 | 14 | 0.45161 |
| 8732 | S10_70166722 | 10 | 70166722 | 1 | G | A | G | 0.95161 | A | 0.04839 | 3 | 0.09677 |
| 8733 | S10_71213590 | 10 | 71213590 | 1046868 | G | A | G | 0.93548 | A | 0.06452 | 4 | 0.12903 |
| 8734 | S10_71213643 | 10 | 71213643 | 53 | G | A | G | 0.91935 | A | 0.08065 | 5 | 0.16129 |
| 8735 | S10_72727207 | 10 | 72727207 | 1513564 | G | A | G | 0.93548 | A | 0.06452 | 4 | 0.12903 |
| 8736 | S10_72757841 | 10 | 72757841 | 30634 | A | G | A | 0.77419 | G | 0.22581 | 12 | 0.3871 |
| 8737 | S10_73141503 | 10 | 73141503 | 383662 | C | T | C | 0.69355 | T | 0.30645 | 11 | 0.35484 |
| 8738 | S10_73653665 | 10 | 73653665 | 512162 | G | C | G | 0.75806 | C | 0.24194 | 11 | 0.35484 |
| 8739 | S10_73653871 | 10 | 73653871 | 206 | A | G | A | 0.93548 | G | 0.06452 | 4 | 0.12903 |
| 8740 | S10_73722893 | 10 | 73722893 | 69022 | C | A | C | 0.95161 | A | 0.04839 | 3 | 0.09677 |
| 8741 | S10_73722902 | 10 | 73722902 | 9 | C | T | T | 0.59677 | C | 0.40323 | 11 | 0.35484 |
| 8742 | S10_73722946 | 10 | 73722946 | 44 | C | T | T | 0.59677 | C | 0.40323 | 11 | 0.35484 |
| 8743 | S10_73722981 | 10 | 73722981 | 35 | C | T | C | 0.95161 | T | 0.04839 | 3 | 0.09677 |
| 8744 | S10_74094089 | 10 | 74094089 | 371108 | G | A | G | 0.70968 | A | 0.29032 | 12 | 0.3871 |
| 8745 | S10_74234446 | 10 | 74234446 | 140357 | G | A | G | 0.66129 | A | 0.33871 | 19 | 0.6129 |
| 8746 | S10_74326047 | 10 | 74326047 | 91601 | T | C | C | 0.69355 | T | 0.30645 | 11 | 0.35484 |

| Site number | SNP name | Chromosome | Physical position | Physical distance from the previous SNP | Reference allele | Alternative allele | Major allele | Major allele frequency | Minor allele | Minor allele frequency | Number heterozygous | Heterozygosity |
|---|---|---|---|---|---|---|---|---|---|---|---|---|
| 8747 | S10_74383568 | 10 | 74383568 | 57521 | T | G | T | 0.90323 | G | 0.09677 | 6 | 0.19355 |
| 8748 | S10_74430819 | 10 | 74430819 | 47251 | G | A | G | 0.74194 | A | 0.25806 | 14 | 0.45161 |
| 8749 | S10_74738381 | 10 | 74738381 | 307562 | G | A | G | 0.91935 | A | 0.08065 | 5 | 0.16129 |
| 8750 | S10_74769243 | 10 | 74769243 | 30862 | A | G | A | 0.6129 | G | 0.3871 | 14 | 0.45161 |
| 8751 | S10_74769267 | 10 | 74769267 | 24 | G | A | G | 0.6129 | A | 0.3871 | 14 | 0.45161 |
| 8752 | S10_74769304 | 10 | 74769304 | 37 | G | A | G | 0.95161 | A | 0.04839 | 3 | 0.09677 |
| 8753 | S10_75731454 | 10 | 75731454 | 962150 | T | C | T | 0.79032 | C | 0.20968 | 11 | 0.35484 |
| 8754 | S10_75731500 | 10 | 75731500 | 46 | C | T | C | 0.70968 | T | 0.29032 | 16 | 0.51613 |
| 8755 | S10_75731580 | 10 | 75731580 | 80 | C | T | T | 0.51613 | C | 0.48387 | 14 | 0.45161 |
| 8756 | S10_76029433 | 10 | 76029433 | 297853 | T | C | C | 0.95161 | T | 0.04839 | 3 | 0.09677 |
| 8757 | S10_76029551 | 10 | 76029551 | 118 | G | A | A | 0.54839 | G | 0.45161 | 16 | 0.51613 |
| 8758 | S10_76029595 | 10 | 76029595 | 44 | A | G | G | 0.72581 | A | 0.27419 | 15 | 0.48387 |
| 8759 | S10_76029600 | 10 | 76029600 | 5 | G | A | A | 0.85484 | G | 0.14516 | 7 | 0.22581 |
| 8760 | S10_76219464 | 10 | 76219464 | 189864 | G | C | C | 0.87097 | G | 0.12903 | 6 | 0.19355 |
| 8761 | S10_76219497 | 10 | 76219497 | 33 | G | C | G | 0.85484 | C | 0.14516 | 7 | 0.22581 |
| 8762 | S10_77411009 | 10 | 77411009 | 1191512 | T | C | T | 0.72581 | C | 0.27419 | 17 | 0.54839 |
| 8763 | S10_77411048 | 10 | 77411048 | 39 | G | A | G | 0.91935 | A | 0.08065 | 5 | 0.16129 |
| 8764 | S10_77411073 | 10 | 77411073 | 25 | A | G | A | 0.90323 | G | 0.09677 | 6 | 0.19355 |
| 8765 | S10_77411078 | 10 | 77411078 | 5 | G | A | G | 0.75806 | A | 0.24194 | 11 | 0.35484 |
| 8766 | S10_77411143 | 10 | 77411143 | 65 | G | T | G | 0.8871 | T | 0.1129 | 7 | 0.22581 |
| 8767 | S10_77543419 | 10 | 77543419 | 132276 | G | A | G | 0.90323 | A | 0.09677 | 6 | 0.19355 |
| 8768 | S10_77543428 | 10 | 77543428 | 9 | T | C | C | 0.75806 | T | 0.24194 | 15 | 0.48387 |
| 8769 | S10_77543626 | 10 | 77543626 | 198 | G | A | G | 0.93548 | A | 0.06452 | 4 | 0.12903 |
| 8770 | S10_77563920 | 10 | 77563920 | 20294 | C | A | C | 0.53226 | A | 0.46774 | 19 | 0.6129 |
| 8771 | S10_77564015 | 10 | 77564015 | 95 | C | T | C | 0.91935 | T | 0.08065 | 5 | 0.16129 |
| 8772 | S10_77924760 | 10 | 77924760 | 360745 | G | C | G | 0.91935 | C | 0.08065 | 5 | 0.16129 |
| 8773 | S10_77924956 | 10 | 77924956 | 196 | C | T | C | 0.87097 | T | 0.12903 | 8 | 0.25806 |
| 8774 | S10_77953427 | 10 | 77953427 | 28471 | C | T | C | 0.87097 | T | 0.12903 | 8 | 0.25806 |
| 8775 | S10_77953432 | 10 | 77953432 | 5 | A | T | A | 0.8871 | T | 0.1129 | 7 | 0.22581 |
| 8776 | S10_77953572 | 10 | 77953572 | 140 | C | T | C | 0.8871 | T | 0.1129 | 7 | 0.22581 |
| 8777 | S10_78068432 | 10 | 78068432 | 114860 | G | C | G | 0.74194 | C | 0.25806 | 14 | 0.45161 |
| 8778 | S10_78068496 | 10 | 78068496 | 64 | C | G | C | 0.93548 | G | 0.06452 | 4 | 0.12903 |
| 8779 | S10_78103536 | 10 | 78103536 | 35040 | G | A | G | 0.80645 | A | 0.19355 | 12 | 0.3871 |
| 8780 | S10_78103571 | 10 | 78103571 | 35 | G | T | G | 0.83871 | T | 0.16129 | 10 | 0.32258 |
| 8781 | S10_78103616 | 10 | 78103616 | 45 | A | G | A | 0.90323 | G | 0.09677 | 4 | 0.12903 |
| 8782 | S10_78135175 | 10 | 78135175 | 31559 | C | T | C | 0.93548 | T | 0.06452 | 4 | 0.12903 |
| 8783 | S10_78135188 | 10 | 78135188 | 13 | G | A | G | 0.87097 | A | 0.12903 | 8 | 0.25806 |
| 8784 | S10_78135207 | 10 | 78135207 | 19 | G | C | G | 0.58065 | C | 0.41935 | 18 | 0.58065 |
| 8785 | S10_78135222 | 10 | 78135222 | 15 | C | T | C | 0.8871 | T | 0.1129 | 7 | 0.22581 |
| 8786 | S10_78135273 | 10 | 78135273 | 51 | C | T | T | 0.56452 | C | 0.43548 | 15 | 0.48387 |
| 8787 | S10_78135280 | 10 | 78135280 | 7 | C | G | G | 0.51613 | C | 0.48387 | 10 | 0.32258 |
| 8788 | S10_78135287 | 10 | 78135287 | 7 | G | A | A | 0.53226 | G | 0.46774 | 13 | 0.41935 |
| 8789 | S10_78135319 | 10 | 78135319 | 32 | G | A | G | 0.80645 | A | 0.19355 | 10 | 0.32258 |
| 8790 | S10_78135381 | 10 | 78135381 | 62 | T | C | C | 0.53226 | T | 0.46774 | 17 | 0.54839 |
| 8791 | S10_78383768 | 10 | 78383768 | 248387 | C | T | C | 0.72581 | T | 0.27419 | 17 | 0.54839 |
| 8792 | S10_78457254 | 10 | 78457254 | 73486 | T | C | T | 0.85484 | C | 0.14516 | 5 | 0.16129 |
| 8793 | S10_78457286 | 10 | 78457286 | 32 | G | C | G | 0.59677 | C | 0.40323 | 17 | 0.54839 |
| 8794 | S10_78457298 | 10 | 78457298 | 12 | A | C | A | 0.95161 | C | 0.04839 | 3 | 0.09677 |
| 8795 | S10_78457301 | 10 | 78457301 | 3 | T | C | T | 0.79032 | C | 0.20968 | 13 | 0.41935 |
| 8796 | S10_78457352 | 10 | 78457352 | 51 | C | T | T | 0.75806 | C | 0.24194 | 15 | 0.48387 |
| 8797 | S10_78457359 | 10 | 78457359 | 7 | C | G | G | 0.8871 | C | 0.1129 | 7 | 0.22581 |
| 8798 | S10_78457366 | 10 | 78457366 | 7 | G | A | A | 0.8871 | G | 0.1129 | 7 | 0.22581 |
| 8799 | S10_78457398 | 10 | 78457398 | 32 | A | G | A | 0.64516 | G | 0.35484 | 16 | 0.51613 |
| 8800 | S10_78472731 | 10 | 78472731 | 15333 | G | A | G | 0.91935 | A | 0.08065 | 5 | 0.16129 |

| Site number | SNP name | Chromosome | Physical position | Physical distance from the previous SNP | Reference allele | Alternative allele | Major allele | Major allele frequency | Minor allele | Minor allele frequency | Number heterozygous | Heterozygosity |
|---|---|---|---|---|---|---|---|---|---|---|---|---|
| 8801 | S10_78472739 | 10 | 78472739 | 8 | C | T | C | 0.83871 | T | 0.16129 | 10 | 0.32258 |
| 8802 | S10_78472802 | 10 | 78472802 | 63 | C | T | C | 0.75806 | T | 0.24194 | 15 | 0.48387 |
| 8803 | S10_78533581 | 10 | 78533581 | 60779 | G | T | G | 0.91935 | T | 0.08065 | 5 | 0.16129 |
| 8804 | S10_78533591 | 10 | 78533591 | 10 | T | C | T | 0.93548 | C | 0.06452 | 4 | 0.12903 |
| 8805 | S10_78533592 | 10 | 78533592 | 1 | T | G | T | 0.91935 | G | 0.08065 | 5 | 0.16129 |
| 8806 | S10_78533669 | 10 | 78533669 | 77 | C | T | C | 0.90323 | T | 0.09677 | 6 | 0.19355 |
| 8807 | S10_78533727 | 10 | 78533727 | 58 | C | T | C | 0.77419 | T | 0.22581 | 14 | 0.45161 |
| 8808 | S10_78533741 | 10 | 78533741 | 14 | T | C | T | 0.80645 | C | 0.19355 | 12 | 0.3871 |
| 8809 | S10_78533742 | 10 | 78533742 | 1 | G | A | G | 0.90323 | A | 0.09677 | 6 | 0.19355 |
| 8810 | S10_78533753 | 10 | 78533753 | 11 | G | T | G | 0.90323 | T | 0.09677 | 6 | 0.19355 |
| 8811 | S10_78533771 | 10 | 78533771 | 18 | G | A | G | 0.90323 | A | 0.09677 | 6 | 0.19355 |
| 8812 | S10_78533789 | 10 | 78533789 | 18 | A | G | A | 0.87097 | G | 0.12903 | 8 | 0.25806 |
| 8813 | S10_78533793 | 10 | 78533793 | 4 | C | A | C | 0.87097 | A | 0.12903 | 8 | 0.25806 |
| 8814 | S10_78577304 | 10 | 78577304 | 43511 | G | A | G | 0.75806 | A | 0.24194 | 15 | 0.48387 |
| 8815 | S10_78577425 | 10 | 78577425 | 121 | G | C | G | 0.70968 | C | 0.29032 | 18 | 0.58065 |
| 8816 | S10_78906839 | 10 | 78906839 | 329414 | C | T | C | 0.85484 | T | 0.14516 | 9 | 0.29032 |
| 8817 | S10_78907033 | 10 | 78907033 | 194 | G | A | G | 0.75806 | A | 0.24194 | 11 | 0.35484 |
| 8818 | S10_79357131 | 10 | 79357131 | 450098 | G | A | G | 0.90323 | A | 0.09677 | 4 | 0.12903 |
| 8819 | S10_79776913 | 10 | 79776913 | 419782 | C | A | C | 0.90323 | A | 0.09677 | 6 | 0.19355 |
| 8820 | S10_79857228 | 10 | 79857228 | 80315 | C | T | C | 0.90323 | T | 0.09677 | 6 | 0.19355 |
| 8821 | S10_80001087 | 10 | 80001087 | 143859 | G | A | G | 0.91935 | A | 0.08065 | 3 | 0.09677 |
| 8822 | S10_80146706 | 10 | 80146706 | 145619 | G | A | G | 0.70968 | A | 0.29032 | 12 | 0.3871 |
| 8823 | S10_80756836 | 10 | 80756836 | 610130 | G | A | G | 0.69355 | A | 0.30645 | 13 | 0.41935 |
| 8824 | S10_80756923 | 10 | 80756923 | 87 | C | T | C | 0.70968 | T | 0.29032 | 12 | 0.3871 |
| 8825 | S10_80796433 | 10 | 80796433 | 39510 | C | T | C | 0.74194 | T | 0.25806 | 10 | 0.32258 |
| 8826 | S10_80796541 | 10 | 80796541 | 108 | T | C | C | 0.80645 | T | 0.19355 | 10 | 0.32258 |
| 8827 | S10_80796596 | 10 | 80796596 | 55 | A | G | G | 0.80645 | A | 0.19355 | 10 | 0.32258 |
| 8828 | S10_81148857 | 10 | 81148857 | 352261 | G | A | G | 0.62903 | A | 0.37097 | 13 | 0.41935 |
| 8829 | S10_81295027 | 10 | 81295027 | 146170 | C | T | C | 0.59677 | T | 0.40323 | 15 | 0.48387 |
| 8830 | S10_81295043 | 10 | 81295043 | 16 | T | A | T | 0.58065 | A | 0.41935 | 14 | 0.45161 |
| 8831 | S10_82067538 | 10 | 82067538 | 772495 | C | T | C | 0.69355 | T | 0.30645 | 13 | 0.41935 |
| 8832 | S10_82247030 | 10 | 82247030 | 179492 | G | A | G | 0.83871 | A | 0.16129 | 8 | 0.25806 |
| 8833 | S10_82535848 | 10 | 82535848 | 288818 | A | T | A | 0.75806 | T | 0.24194 | 13 | 0.41935 |
| 8834 | S10_82535922 | 10 | 82535922 | 74 | C | T | C | 0.93548 | T | 0.06452 | 4 | 0.12903 |
| 8835 | S10_82535982 | 10 | 82535982 | 60 | G | A | G | 0.75806 | A | 0.24194 | 13 | 0.41935 |
| 8836 | S10_82655400 | 10 | 82655400 | 119418 | C | T | C | 0.87097 | T | 0.12903 | 8 | 0.25806 |
| 8837 | S10_82655419 | 10 | 82655419 | 19 | G | A | A | 0.62903 | G | 0.37097 | 13 | 0.41935 |
| 8838 | S10_82655509 | 10 | 82655509 | 90 | A | G | G | 0.62903 | A | 0.37097 | 13 | 0.41935 |
| 8839 | S10_82655548 | 10 | 82655548 | 39 | G | A | A | 0.62903 | G | 0.37097 | 13 | 0.41935 |
| 8840 | S10_82655553 | 10 | 82655553 | 5 | A | G | G | 0.58065 | A | 0.41935 | 16 | 0.51613 |
| 8841 | S10_82699556 | 10 | 82699556 | 44003 | A | G | A | 0.70968 | G | 0.29032 | 12 | 0.3871 |
| 8842 | S10_82699605 | 10 | 82699605 | 49 | C | G | C | 0.93548 | G | 0.06452 | 4 | 0.12903 |
| 8843 | S10_82749555 | 10 | 82749555 | 49950 | A | C | A | 0.95161 | C | 0.04839 | 3 | 0.09677 |
| 8844 | S10_82800653 | 10 | 82800653 | 51098 | G | A | G | 0.83871 | A | 0.16129 | 6 | 0.19355 |
| 8845 | S10_82800663 | 10 | 82800663 | 10 | T | C | T | 0.83871 | C | 0.16129 | 6 | 0.19355 |
| 8846 | S10_82974169 | 10 | 82974169 | 173506 | T | A | T | 0.80645 | A | 0.19355 | 12 | 0.3871 |
| 8847 | S10_82974175 | 10 | 82974175 | 6 | G | T | G | 0.70968 | T | 0.29032 | 8 | 0.25806 |
| 8848 | S10_83053121 | 10 | 83053121 | 78946 | C | T | C | 0.74194 | T | 0.25806 | 12 | 0.3871 |
| 8849 | S10_83084193 | 10 | 83084193 | 31072 | C | T | C | 0.62903 | T | 0.37097 | 17 | 0.54839 |
| 8850 | S10_83113526 | 10 | 83113526 | 29333 | C | T | T | 0.56452 | C | 0.43548 | 17 | 0.54839 |
| 8851 | S10_83213072 | 10 | 83213072 | 99546 | C | T | T | 0.90323 | C | 0.09677 | 4 | 0.12903 |
| 8852 | S10_83213095 | 10 | 83213095 | 23 | A | C | A | 0.90323 | C | 0.09677 | 4 | 0.12903 |
| 8853 | S10_83213205 | 10 | 83213205 | 110 | A | G | A | 0.74194 | G | 0.25806 | 14 | 0.45161 |
| 8854 | S10_83353495 | 10 | 83353495 | 140290 | T | C | T | 0.8871 | C | 0.1129 | 5 | 0.16129 |

| Site number | SNP name | Chromosome | Physical position | Physical distance from the previous SNP | Reference allele | Alternative allele | Major allele | Major allele frequency | Minor allele | Minor allele frequency | Number heterozygous | Heterozygosity |
|---|---|---|---|---|---|---|---|---|---|---|---|---|
| 8855 | S10_83494894 | 10 | 83494894 | 141399 | T | A | T | 0.85484 | A | 0.14516 | 7 | 0.22581 |
| 8856 | S10_83495027 | 10 | 83495027 | 133 | A | G | A | 0.91935 | G | 0.08065 | 5 | 0.16129 |
| 8857 | S10_83495029 | 10 | 83495029 | 2 | T | G | T | 0.82258 | G | 0.17742 | 7 | 0.22581 |
| 8858 | S10_83495089 | 10 | 83495089 | 60 | A | G | A | 0.91935 | G | 0.08065 | 5 | 0.16129 |
| 8859 | S10_83820429 | 10 | 83820429 | 325340 | A | C | A | 0.95161 | C | 0.04839 | 3 | 0.09677 |
| 8860 | S10_84178536 | 10 | 84178536 | 358107 | G | A | G | 0.77419 | A | 0.22581 | 10 | 0.32258 |
| 8861 | S10_84178538 | 10 | 84178538 | 2 | G | A | G | 0.77419 | A | 0.22581 | 10 | 0.32258 |
| 8862 | S10_84322142 | 10 | 84322142 | 143604 | A | G | A | 0.93548 | G | 0.06452 | 4 | 0.12903 |
| 8863 | S10_84322182 | 10 | 84322182 | 40 | C | T | C | 0.67742 | T | 0.32258 | 10 | 0.32258 |
| 8864 | S10_84322183 | 10 | 84322183 | 1 | G | A | G | 0.91935 | A | 0.08065 | 5 | 0.16129 |
| 8865 | S10_84700938 | 10 | 84700938 | 378755 | T | C | C | 0.90323 | T | 0.09677 | 6 | 0.19355 |
| 8866 | S10_84700949 | 10 | 84700949 | 11 | C | T | C | 0.95161 | T | 0.04839 | 3 | 0.09677 |
| 8867 | S10_84869716 | 10 | 84869716 | 168767 | C | T | T | 0.67742 | C | 0.32258 | 10 | 0.32258 |
| 8868 | S10_84872672 | 10 | 84872672 | 2956 | G | A | G | 0.91935 | A | 0.08065 | 5 | 0.16129 |
| 8869 | S10_84872674 | 10 | 84872674 | 2 | G | A | G | 0.80645 | A | 0.19355 | 10 | 0.32258 |
| 8870 | S10_85024241 | 10 | 85024241 | 151567 | C | A | C | 0.75806 | A | 0.24194 | 9 | 0.29032 |
| 8871 | S10_85024266 | 10 | 85024266 | 25 | T | C | T | 0.93548 | C | 0.06452 | 4 | 0.12903 |
| 8872 | S10_85093194 | 10 | 85093194 | 68928 | G | A | G | 0.93548 | A | 0.06452 | 2 | 0.06452 |
| 8873 | S10_85093229 | 10 | 85093229 | 35 | G | A | G | 0.93548 | A | 0.06452 | 2 | 0.06452 |
| 8874 | S10_85093251 | 10 | 85093251 | 22 | A | G | G | 0.6129 | A | 0.3871 | 16 | 0.51613 |
| 8875 | S10_85093314 | 10 | 85093314 | 63 | G | A | G | 0.87097 | A | 0.12903 | 6 | 0.19355 |
| 8876 | S10_85093516 | 10 | 85093516 | 202 | G | A | G | 0.80645 | A | 0.19355 | 10 | 0.32258 |
| 8877 | S10_85257567 | 10 | 85257567 | 164051 | C | T | C | 0.83871 | T | 0.16129 | 10 | 0.32258 |
| 8878 | S10_85335107 | 10 | 85335107 | 77540 | G | T | G | 0.56452 | T | 0.43548 | 13 | 0.41935 |
| 8879 | S10_85335126 | 10 | 85335126 | 19 | A | G | A | 0.56452 | G | 0.43548 | 13 | 0.41935 |
| 8880 | S10_85335230 | 10 | 85335230 | 104 | A | T | T | 0.95161 | A | 0.04839 | 3 | 0.09677 |
| 8881 | S10_85335240 | 10 | 85335240 | 10 | G | A | A | 0.54839 | G | 0.45161 | 12 | 0.3871 |
| 8882 | S10_85335243 | 10 | 85335243 | 3 | G | A | A | 0.56452 | G | 0.43548 | 13 | 0.41935 |
| 8883 | S10_85335294 | 10 | 85335294 | 51 | A | G | A | 0.69355 | G | 0.30645 | 15 | 0.48387 |
| 8884 | S10_85335314 | 10 | 85335314 | 20 | T | A | T | 0.95161 | A | 0.04839 | 3 | 0.09677 |
| 8885 | S10_85366571 | 10 | 85366571 | 31257 | G | C | C | 0.64516 | G | 0.35484 | 10 | 0.32258 |
| 8886 | S10_85366632 | 10 | 85366632 | 61 | C | T | C | 0.82258 | T | 0.17742 | 7 | 0.22581 |
| 8887 | S10_85749638 | 10 | 85749638 | 383006 | C | T | C | 0.95161 | T | 0.04839 | 3 | 0.09677 |
| 8888 | S10_85754135 | 10 | 85754135 | 4497 | C | T | C | 0.91935 | T | 0.08065 | 5 | 0.16129 |
| 8889 | S10_85754256 | 10 | 85754256 | 121 | G | A | G | 0.85484 | A | 0.14516 | 9 | 0.29032 |
| 8890 | S10_85754320 | 10 | 85754320 | 64 | A | G | A | 0.85484 | G | 0.14516 | 9 | 0.29032 |
| 8891 | S10_85881373 | 10 | 85881373 | 127053 | C | T | C | 0.70968 | T | 0.29032 | 14 | 0.45161 |
| 8892 | S10_86039924 | 10 | 86039924 | 158551 | T | C | C | 0.83871 | T | 0.16129 | 10 | 0.32258 |
| 8893 | S10_86040039 | 10 | 86040039 | 115 | C | T | C | 0.93548 | T | 0.06452 | 4 | 0.12903 |
| 8894 | S10_86391214 | 10 | 86391214 | 351175 | A | C | A | 0.8871 | C | 0.1129 | 7 | 0.22581 |
| 8895 | S10_86391222 | 10 | 86391222 | 8 | C | T | C | 0.90323 | T | 0.09677 | 6 | 0.19355 |
| 8896 | S10_86391283 | 10 | 86391283 | 61 | T | C | T | 0.83871 | C | 0.16129 | 8 | 0.25806 |
| 8897 | S10_86580655 | 10 | 86580655 | 189372 | A | G | A | 0.83871 | G | 0.16129 | 10 | 0.32258 |
| 8898 | S10_86580756 | 10 | 86580756 | 101 | T | C | T | 0.80645 | C | 0.19355 | 12 | 0.3871 |
| 8899 | S10_86580851 | 10 | 86580851 | 95 | T | G | T | 0.83871 | G | 0.16129 | 10 | 0.32258 |
| 8900 | S10_86868107 | 10 | 86868107 | 287256 | C | T | C | 0.87097 | T | 0.12903 | 8 | 0.25806 |
| 8901 | S10_86868212 | 10 | 86868212 | 105 | C | T | T | 0.59677 | C | 0.40323 | 17 | 0.54839 |
| 8902 | S10_87023400 | 10 | 87023400 | 155188 | C | A | C | 0.93548 | A | 0.06452 | 4 | 0.12903 |
| 8903 | S10_87514900 | 10 | 87514900 | 491500 | G | A | G | 0.80645 | A | 0.19355 | 10 | 0.32258 |
| 8904 | S10_87515012 | 10 | 87515012 | 112 | T | C | C | 0.85484 | T | 0.14516 | 9 | 0.29032 |
| 8905 | S10_87614938 | 10 | 87614938 | 99926 | C | T | C | 0.91935 | T | 0.08065 | 5 | 0.16129 |
| 8906 | S10_87615086 | 10 | 87615086 | 148 | T | C | T | 0.80645 | C | 0.19355 | 12 | 0.3871 |
| 8907 | S10_87783716 | 10 | 87783716 | 168630 | T | C | C | 0.83871 | T | 0.16129 | 8 | 0.25806 |
| 8908 | S10_87783812 | 10 | 87783812 | 96 | G | A | G | 0.87097 | A | 0.12903 | 8 | 0.25806 |

| Site number | SNP name | Chromosome | Physical position | Physical distance from the previous SNP | Reference allele | Alternative allele | Major allele | Major allele frequency | Minor allele | Minor allele frequency | Number heterozygous | Heterozygosity |
|---|---|---|---|---|---|---|---|---|---|---|---|---|
| 8909 | S10_87903428 | 10 | 87903428 | 119616 | A | C | C | 0.51613 | A | 0.48387 | 16 | 0.51613 |
| 8910 | S10_88007029 | 10 | 88007029 | 103601 | C | T | T | 0.51613 | C | 0.48387 | 20 | 0.64516 |
| 8911 | S10_88007042 | 10 | 88007042 | 13 | T | C | T | 0.91935 | C | 0.08065 | 5 | 0.16129 |
| 8912 | S10_88156736 | 10 | 88156736 | 149694 | A | G | G | 0.85484 | A | 0.14516 | 9 | 0.29032 |
| 8913 | S10_88156785 | 10 | 88156785 | 49 | G | A | G | 0.91935 | A | 0.08065 | 5 | 0.16129 |
| 8914 | S10_88156793 | 10 | 88156793 | 8 | C | T | C | 0.8871 | T | 0.1129 | 7 | 0.22581 |
| 8915 | S10_88156883 | 10 | 88156883 | 90 | C | T | C | 0.90323 | T | 0.09677 | 6 | 0.19355 |
| 8916 | S10_88369645 | 10 | 88369645 | 212762 | G | A | G | 0.6129 | A | 0.3871 | 10 | 0.32258 |
| 8917 | S10_88369672 | 10 | 88369672 | 27 | G | T | T | 0.80645 | G | 0.19355 | 10 | 0.32258 |
| 8918 | S10_88444613 | 10 | 88444613 | 74941 | C | T | C | 0.91935 | T | 0.08065 | 5 | 0.16129 |
| 8919 | S10_88444731 | 10 | 88444731 | 118 | G | A | G | 0.77419 | A | 0.22581 | 8 | 0.25806 |
| 8920 | S10_88697606 | 10 | 88697606 | 252875 | A | G | A | 0.69355 | G | 0.30645 | 15 | 0.48387 |
| 8921 | S10_88697704 | 10 | 88697704 | 98 | G | A | G | 0.69355 | A | 0.30645 | 15 | 0.48387 |
| 8922 | S10_88894209 | 10 | 88894209 | 196505 | G | A | G | 0.87097 | A | 0.12903 | 8 | 0.25806 |
| 8923 | S10_88926986 | 10 | 88926986 | 32777 | A | G | G | 0.77419 | A | 0.22581 | 12 | 0.3871 |
| 8924 | S10_88927122 | 10 | 88927122 | 136 | G | C | C | 0.79032 | G | 0.20968 | 11 | 0.35484 |
| 8925 | S10_89019079 | 10 | 89019079 | 91957 | G | A | G | 0.95161 | A | 0.04839 | 3 | 0.09677 |
| 8926 | S10_89019177 | 10 | 89019177 | 98 | A | T | A | 0.85484 | T | 0.14516 | 7 | 0.22581 |
| 8927 | S10_89019212 | 10 | 89019212 | 35 | T | C | T | 0.72581 | C | 0.27419 | 15 | 0.48387 |
| 8928 | S10_89019224 | 10 | 89019224 | 12 | C | T | C | 0.85484 | T | 0.14516 | 7 | 0.22581 |
| 8929 | S10_89920932 | 10 | 89920932 | 901708 | C | T | C | 0.51613 | T | 0.48387 | 16 | 0.51613 |
| 8930 | S10_89926889 | 10 | 89926889 | 5957 | T | C | C | 0.5 | T | 0.5 | 13 | 0.41935 |
| 8931 | S10_89926989 | 10 | 89926989 | 100 | A | G | A | 0.66129 | G | 0.33871 | 11 | 0.35484 |
| 8932 | S10_89927003 | 10 | 89927003 | 14 | G | A | G | 0.66129 | A | 0.33871 | 11 | 0.35484 |
| 8933 | S10_89927033 | 10 | 89927033 | 30 | G | A | G | 0.90323 | A | 0.09677 | 4 | 0.12903 |
| 8934 | S10_89927039 | 10 | 89927039 | 6 | G | A | G | 0.90323 | A | 0.09677 | 4 | 0.12903 |
| 8935 | S10_89927050 | 10 | 89927050 | 11 | T | C | T | 0.59677 | C | 0.40323 | 13 | 0.41935 |
| 8936 | S10_89963689 | 10 | 89963689 | 36639 | C | T | C | 0.90323 | T | 0.09677 | 6 | 0.19355 |
| 8937 | S10_89963727 | 10 | 89963727 | 38 | C | G | C | 0.59677 | G | 0.40323 | 15 | 0.48387 |
| 8938 | S10_89963759 | 10 | 89963759 | 32 | A | G | A | 0.72581 | G | 0.27419 | 11 | 0.35484 |
| 8939 | S10_89963792 | 10 | 89963792 | 33 | T | C | C | 0.69355 | T | 0.30645 | 11 | 0.35484 |
| 8940 | S10_89963806 | 10 | 89963806 | 14 | C | T | C | 0.59677 | T | 0.40323 | 15 | 0.48387 |
| 8941 | S10_89963807 | 10 | 89963807 | 1 | A | G | G | 0.69355 | A | 0.30645 | 11 | 0.35484 |
| 8942 | S10_89963843 | 10 | 89963843 | 36 | C | T | C | 0.90323 | T | 0.09677 | 6 | 0.19355 |
| 8943 | S10_90076956 | 10 | 90076956 | 113113 | T | G | T | 0.54839 | G | 0.45161 | 12 | 0.3871 |
| 8944 | S10_90077014 | 10 | 90077014 | 58 | G | A | G | 0.91935 | A | 0.08065 | 5 | 0.16129 |
| 8945 | S10_90077087 | 10 | 90077087 | 73 | C | T | T | 0.72581 | C | 0.27419 | 9 | 0.29032 |
| 8946 | S10_90187113 | 10 | 90187113 | 110026 | A | C | A | 0.8871 | C | 0.1129 | 7 | 0.22581 |
| 8947 | S10_90391097 | 10 | 90391097 | 203984 | T | C | T | 0.79032 | C | 0.20968 | 11 | 0.35484 |
| 8948 | S10_90723262 | 10 | 90723262 | 332165 | C | A | C | 0.87097 | A | 0.12903 | 6 | 0.19355 |
| 8949 | S10_90723372 | 10 | 90723372 | 110 | C | T | T | 0.74194 | C | 0.25806 | 12 | 0.3871 |
| 8950 | S10_90723472 | 10 | 90723472 | 100 | G | A | G | 0.54839 | A | 0.45161 | 14 | 0.45161 |
| 8951 | S10_90766484 | 10 | 90766484 | 43012 | G | A | G | 0.91935 | A | 0.08065 | 5 | 0.16129 |
| 8952 | S10_91200064 | 10 | 91200064 | 433580 | A | C | A | 0.83871 | C | 0.16129 | 8 | 0.25806 |
| 8953 | S10_91200071 | 10 | 91200071 | 7 | G | T | G | 0.77419 | T | 0.22581 | 10 | 0.32258 |
| 8954 | S10_91200102 | 10 | 91200102 | 31 | G | C | G | 0.93548 | C | 0.06452 | 4 | 0.12903 |
| 8955 | S10_91200110 | 10 | 91200110 | 8 | C | A | C | 0.70968 | A | 0.29032 | 12 | 0.3871 |
| 8956 | S10_91200152 | 10 | 91200152 | 42 | T | C | T | 0.64516 | C | 0.35484 | 10 | 0.32258 |
| 8957 | S10_91200162 | 10 | 91200162 | 10 | G | A | G | 0.67742 | A | 0.32258 | 10 | 0.32258 |
| 8958 | S10_91200177 | 10 | 91200177 | 15 | T | C | T | 0.83871 | C | 0.16129 | 8 | 0.25806 |
| 8959 | S10_91200211 | 10 | 91200211 | 34 | T | G | T | 0.85484 | G | 0.14516 | 9 | 0.29032 |
| 8960 | S10_91200238 | 10 | 91200238 | 27 | G | A | G | 0.87097 | A | 0.12903 | 8 | 0.25806 |
| 8961 | S10_91241208 | 10 | 91241208 | 40970 | G | A | G | 0.90323 | A | 0.09677 | 6 | 0.19355 |
| 8962 | S10_91241219 | 10 | 91241219 | 11 | T | C | T | 0.93548 | C | 0.06452 | 4 | 0.12903 |

| Site number | SNP name | Chromosome | Physical position | Physical distance from the previous SNP | Reference allele | Alternative allele | Major allele | Major allele frequency | Minor allele | Minor allele frequency | Number heterozygous | Heterozygosity |
|---|---|---|---|---|---|---|---|---|---|---|---|---|
| 8963 | S10_91241429 | 10 | 91241429 | 210 | A | T | A | 0.93548 | T | 0.06452 | 4 | 0.12903 |
| 8964 | S10_91241455 | 10 | 91241455 | 26 | A | G | G | 0.8871 | A | 0.1129 | 5 | 0.16129 |
| 8965 | S10_91594156 | 10 | 91594156 | 352701 | G | A | G | 0.8871 | A | 0.1129 | 7 | 0.22581 |
| 8966 | S10_91594160 | 10 | 91594160 | 4 | C | G | C | 0.87097 | G | 0.12903 | 8 | 0.25806 |
| 8967 | S10_91594267 | 10 | 91594267 | 107 | G | T | G | 0.70968 | T | 0.29032 | 8 | 0.25806 |
| 8968 | S10_91594289 | 10 | 91594289 | 22 | G | A | G | 0.90323 | A | 0.09677 | 6 | 0.19355 |
| 8969 | S10_92390180 | 10 | 92390180 | 795891 | A | G | G | 0.53226 | A | 0.46774 | 17 | 0.54839 |
| 8970 | S10_92390239 | 10 | 92390239 | 59 | C | A | C | 0.72581 | A | 0.27419 | 17 | 0.54839 |
| 8971 | S10_92424623 | 10 | 92424623 | 34384 | G | C | G | 0.69355 | C | 0.30645 | 17 | 0.54839 |
| 8972 | S10_92424685 | 10 | 92424685 | 62 | T | C | T | 0.83871 | C | 0.16129 | 10 | 0.32258 |
| 8973 | S10_92649137 | 10 | 92649137 | 224452 | G | A | G | 0.91935 | A | 0.08065 | 5 | 0.16129 |
| 8974 | S10_92649223 | 10 | 92649223 | 86 | G | A | G | 0.91935 | A | 0.08065 | 5 | 0.16129 |
| 8975 | S10_92649274 | 10 | 92649274 | 51 | C | T | C | 0.74194 | T | 0.25806 | 14 | 0.45161 |
| 8976 | S10_92649284 | 10 | 92649284 | 10 | A | G | A | 0.80645 | G | 0.19355 | 10 | 0.32258 |
| 8977 | S10_92702681 | 10 | 92702681 | 53397 | A | G | G | 0.83871 | A | 0.16129 | 10 | 0.32258 |
| 8978 | S10_92714858 | 10 | 92714858 | 12177 | T | C | T | 0.80645 | C | 0.19355 | 10 | 0.32258 |
| 8979 | S10_92714921 | 10 | 92714921 | 63 | T | C | T | 0.8871 | C | 0.1129 | 7 | 0.22581 |
| 8980 | S10_92771265 | 10 | 92771265 | 56344 | G | A | G | 0.87097 | A | 0.12903 | 6 | 0.19355 |
| 8981 | S10_92771433 | 10 | 92771433 | 168 | T | C | T | 0.80645 | C | 0.19355 | 6 | 0.19355 |
| 8982 | S10_93035736 | 10 | 93035736 | 264303 | T | A | T | 0.93548 | A | 0.06452 | 4 | 0.12903 |
| 8983 | S10_93155024 | 10 | 93155024 | 119288 | T | C | C | 0.74194 | T | 0.25806 | 12 | 0.3871 |
| 8984 | S10_93155083 | 10 | 93155083 | 59 | A | G | A | 0.67742 | G | 0.32258 | 16 | 0.51613 |
| 8985 | S10_93303415 | 10 | 93303415 | 148332 | G | T | G | 0.90323 | T | 0.09677 | 6 | 0.19355 |
| 8986 | S10_93303421 | 10 | 93303421 | 6 | G | C | G | 0.8871 | C | 0.1129 | 7 | 0.22581 |
| 8987 | S10_93303428 | 10 | 93303428 | 7 | C | T | C | 0.6129 | T | 0.3871 | 20 | 0.64516 |
| 8988 | S10_93303491 | 10 | 93303491 | 63 | C | T | C | 0.91935 | T | 0.08065 | 5 | 0.16129 |
| 8989 | S10_93594114 | 10 | 93594114 | 290623 | G | C | G | 0.87097 | C | 0.12903 | 8 | 0.25806 |
| 8990 | S10_93594178 | 10 | 93594178 | 64 | A | G | A | 0.91935 | G | 0.08065 | 5 | 0.16129 |
| 8991 | S10_93594203 | 10 | 93594203 | 25 | G | A | G | 0.8871 | A | 0.1129 | 7 | 0.22581 |
| 8992 | S10_93594246 | 10 | 93594246 | 43 | T | C | T | 0.91935 | C | 0.08065 | 5 | 0.16129 |
| 8993 | S10_93594292 | 10 | 93594292 | 46 | T | A | T | 0.95161 | A | 0.04839 | 3 | 0.09677 |
| 8994 | S10_93868183 | 10 | 93868183 | 273891 | C | T | C | 0.91935 | T | 0.08065 | 3 | 0.09677 |
| 8995 | S10_93868192 | 10 | 93868192 | 9 | C | T | C | 0.82258 | T | 0.17742 | 9 | 0.29032 |
| 8996 | S10_93973336 | 10 | 93973336 | 105144 | G | A | G | 0.82258 | A | 0.17742 | 11 | 0.35484 |
| 8997 | S10_93973354 | 10 | 93973354 | 18 | C | T | T | 0.54839 | C | 0.45161 | 18 | 0.58065 |
| 8998 | S10_93973439 | 10 | 93973439 | 85 | G | A | G | 0.8871 | A | 0.1129 | 7 | 0.22581 |
| 8999 | S10_93973478 | 10 | 93973478 | 39 | A | G | A | 0.93548 | G | 0.06452 | 4 | 0.12903 |
| 9000 | S10_93973491 | 10 | 93973491 | 13 | C | T | C | 0.82258 | T | 0.17742 | 11 | 0.35484 |
| 9001 | S10_93973518 | 10 | 93973518 | 27 | C | T | T | 0.54839 | C | 0.45161 | 18 | 0.58065 |
| 9002 | S10_94581673 | 10 | 94581673 | 608155 | G | A | A | 0.51613 | G | 0.48387 | 20 | 0.64516 |
| 9003 | S10_94581807 | 10 | 94581807 | 134 | C | T | C | 0.90323 | T | 0.09677 | 6 | 0.19355 |
| 9004 | S10_94756514 | 10 | 94756514 | 174707 | A | G | G | 0.62903 | A | 0.37097 | 19 | 0.6129 |
| 9005 | S10_94756567 | 10 | 94756567 | 53 | G | A | G | 0.72581 | A | 0.27419 | 11 | 0.35484 |
| 9006 | S10_94756576 | 10 | 94756576 | 9 | G | A | A | 0.59677 | G | 0.40323 | 19 | 0.6129 |
| 9007 | S10_94756633 | 10 | 94756633 | 57 | T | G | G | 0.62903 | T | 0.37097 | 19 | 0.6129 |
| 9008 | S10_94756639 | 10 | 94756639 | 6 | G | T | T | 0.62903 | G | 0.37097 | 19 | 0.6129 |
| 9009 | S10_94825954 | 10 | 94825954 | 69315 | A | G | A | 0.95161 | G | 0.04839 | 3 | 0.09677 |
| 9010 | S10_95040458 | 10 | 95040458 | 214504 | C | T | C | 0.75806 | T | 0.24194 | 13 | 0.41935 |
| 9011 | S10_95273130 | 10 | 95273130 | 232672 | G | A | G | 0.83871 | A | 0.16129 | 8 | 0.25806 |
| 9012 | S10_95273145 | 10 | 95273145 | 15 | G | A | G | 0.83871 | A | 0.16129 | 8 | 0.25806 |
| 9013 | S10_95273168 | 10 | 95273168 | 23 | G | A | G | 0.83871 | A | 0.16129 | 8 | 0.25806 |
| 9014 | S10_95273201 | 10 | 95273201 | 33 | C | A | C | 0.85484 | A | 0.14516 | 9 | 0.29032 |
| 9015 | S10_95273273 | 10 | 95273273 | 72 | A | C | A | 0.75806 | C | 0.24194 | 9 | 0.29032 |
| 9016 | S10_95273321 | 10 | 95273321 | 48 | G | A | G | 0.83871 | A | 0.16129 | 8 | 0.25806 |

| Site number | SNP name | Chromosome | Physical position | Physical distance from the previous SNP | Reference allele | Alternative allele | Major allele | Major allele frequency | Minor allele | Minor allele frequency | Number heterozygous | Heterozygosity |
|---|---|---|---|---|---|---|---|---|---|---|---|---|
| 9017 | S10_95301522 | 10 | 95301522 | 28201 | A | G | A | 0.5 | G | 0.5 | 15 | 0.48387 |
| 9018 | S10_95301601 | 10 | 95301601 | 79 | G | A | G | 0.91935 | A | 0.08065 | 5 | 0.16129 |
| 9019 | S10_95301687 | 10 | 95301687 | 86 | A | G | A | 0.5 | G | 0.5 | 15 | 0.48387 |
| 9020 | S10_95556949 | 10 | 95556949 | 255262 | T | A | T | 0.90323 | A | 0.09677 | 6 | 0.19355 |
| 9021 | S10_95556951 | 10 | 95556951 | 2 | T | C | C | 0.62903 | T | 0.37097 | 13 | 0.41935 |
| 9022 | S10_95556958 | 10 | 95556958 | 7 | T | C | C | 0.75806 | T | 0.24194 | 13 | 0.41935 |
| 9023 | S10_95556967 | 10 | 95556967 | 9 | A | T | T | 0.75806 | A | 0.24194 | 13 | 0.41935 |
| 9024 | S10_95556991 | 10 | 95556991 | 24 | C | T | C | 0.6129 | T | 0.3871 | 12 | 0.3871 |
| 9025 | S10_95557002 | 10 | 95557002 | 11 | T | G | G | 0.62903 | T | 0.37097 | 13 | 0.41935 |
| 9026 | S10_95557008 | 10 | 95557008 | 6 | G | T | T | 0.62903 | G | 0.37097 | 13 | 0.41935 |
| 9027 | S10_95557021 | 10 | 95557021 | 13 | T | A | A | 0.62903 | T | 0.37097 | 13 | 0.41935 |
| 9028 | S10_95557121 | 10 | 95557121 | 100 | C | A | A | 0.75806 | C | 0.24194 | 13 | 0.41935 |
| 9029 | S10_95594926 | 10 | 95594926 | 37805 | C | T | C | 0.83871 | T | 0.16129 | 10 | 0.32258 |
| 9030 | S10_95594951 | 10 | 95594951 | 25 | T | C | C | 0.79032 | T | 0.20968 | 13 | 0.41935 |
| 9031 | S10_95594958 | 10 | 95594958 | 7 | T | C | T | 0.82258 | C | 0.17742 | 7 | 0.22581 |
| 9032 | S10_95615136 | 10 | 95615136 | 20178 | G | A | G | 0.80645 | A | 0.19355 | 10 | 0.32258 |
| 9033 | S10_95750033 | 10 | 95750033 | 134897 | C | T | C | 0.87097 | T | 0.12903 | 8 | 0.25806 |
| 9034 | S10_95750063 | 10 | 95750063 | 30 | A | G | A | 0.95161 | G | 0.04839 | 3 | 0.09677 |
| 9035 | S10_95750096 | 10 | 95750096 | 33 | T | C | T | 0.95161 | C | 0.04839 | 3 | 0.09677 |
| 9036 | S10_95812896 | 10 | 95812896 | 62800 | A | G | G | 0.83871 | A | 0.16129 | 8 | 0.25806 |
| 9037 | S10_95867428 | 10 | 95867428 | 54532 | G | A | G | 0.77419 | A | 0.22581 | 14 | 0.45161 |
| 9038 | S10_95867449 | 10 | 95867449 | 21 | G | A | G | 0.83871 | A | 0.16129 | 8 | 0.25806 |
| 9039 | S10_95867561 | 10 | 95867561 | 112 | C | T | C | 0.91935 | T | 0.08065 | 5 | 0.16129 |
| 9040 | S10_95867562 | 10 | 95867562 | 1 | G | A | G | 0.77419 | A | 0.22581 | 14 | 0.45161 |
| 9041 | S10_96242769 | 10 | 96242769 | 375207 | C | T | C | 0.90323 | T | 0.09677 | 6 | 0.19355 |
| 9042 | S10_96242770 | 10 | 96242770 | 1 | A | T | A | 0.70968 | T | 0.29032 | 14 | 0.45161 |
| 9043 | S10_96242995 | 10 | 96242995 | 225 | A | G | A | 0.79032 | G | 0.20968 | 9 | 0.29032 |
| 9044 | S10_96365519 | 10 | 96365519 | 122524 | A | C | C | 0.54839 | A | 0.45161 | 18 | 0.58065 |
| 9045 | S10_96365728 | 10 | 96365728 | 209 | C | G | C | 0.91935 | G | 0.08065 | 5 | 0.16129 |
| 9046 | S10_96461937 | 10 | 96461937 | 96209 | C | A | C | 0.95161 | A | 0.04839 | 3 | 0.09677 |
| 9047 | S10_96484875 | 10 | 96484875 | 22938 | C | T | C | 0.95161 | T | 0.04839 | 3 | 0.09677 |
| 9048 | S10_96484936 | 10 | 96484936 | 61 | T | C | T | 0.80645 | C | 0.19355 | 10 | 0.32258 |
| 9049 | S10_96484977 | 10 | 96484977 | 41 | T | C | T | 0.95161 | C | 0.04839 | 3 | 0.09677 |
| 9050 | S10_96484983 | 10 | 96484983 | 6 | C | T | C | 0.95161 | T | 0.04839 | 3 | 0.09677 |
| 9051 | S10_96484984 | 10 | 96484984 | 1 | A | G | A | 0.95161 | G | 0.04839 | 3 | 0.09677 |
| 9052 | S10_96484986 | 10 | 96484986 | 2 | G | A | G | 0.95161 | A | 0.04839 | 3 | 0.09677 |
| 9053 | S10_96485069 | 10 | 96485069 | 83 | A | G | A | 0.95161 | G | 0.04839 | 3 | 0.09677 |
| 9054 | S10_96485108 | 10 | 96485108 | 39 | C | T | C | 0.95161 | T | 0.04839 | 3 | 0.09677 |
| 9055 | S10_96485119 | 10 | 96485119 | 11 | A | C | A | 0.95161 | C | 0.04839 | 3 | 0.09677 |
| 9056 | S10_96527413 | 10 | 96527413 | 42294 | C | T | C | 0.51613 | T | 0.48387 | 14 | 0.45161 |
| 9057 | S10_96527421 | 10 | 96527421 | 8 | C | T | C | 0.79032 | T | 0.20968 | 9 | 0.29032 |
| 9058 | S10_96670958 | 10 | 96670958 | 143537 | C | T | C | 0.69355 | T | 0.30645 | 15 | 0.48387 |
| 9059 | S10_96670970 | 10 | 96670970 | 12 | G | A | G | 0.93548 | A | 0.06452 | 4 | 0.12903 |
| 9060 | S10_96671039 | 10 | 96671039 | 69 | T | C | T | 0.69355 | C | 0.30645 | 15 | 0.48387 |
| 9061 | S10_96671103 | 10 | 96671103 | 64 | A | G | A | 0.80645 | G | 0.19355 | 10 | 0.32258 |
| 9062 | S10_96671110 | 10 | 96671110 | 7 | C | G | C | 0.91935 | G | 0.08065 | 5 | 0.16129 |
| 9063 | S10_96671120 | 10 | 96671120 | 10 | C | T | C | 0.91935 | T | 0.08065 | 5 | 0.16129 |
| 9064 | S10_96671136 | 10 | 96671136 | 16 | C | G | C | 0.69355 | G | 0.30645 | 15 | 0.48387 |
| 9065 | S10_96671142 | 10 | 96671142 | 6 | A | G | A | 0.95161 | G | 0.04839 | 3 | 0.09677 |
| 9066 | S10_96671163 | 10 | 96671163 | 21 | G | T | G | 0.69355 | T | 0.30645 | 15 | 0.48387 |
| 9067 | S10_97055543 | 10 | 97055543 | 384380 | T | C | T | 0.82258 | C | 0.17742 | 9 | 0.29032 |
| 9068 | S10_97055566 | 10 | 97055566 | 23 | T | C | T | 0.85484 | C | 0.14516 | 9 | 0.29032 |
| 9069 | S10_97055767 | 10 | 97055767 | 201 | G | C | C | 0.53226 | G | 0.46774 | 19 | 0.6129 |
| 9070 | S10_97056419 | 10 | 97056419 | 652 | C | T | C | 0.91935 | T | 0.08065 | 5 | 0.16129 |

| Site number | SNP name | Chromosome | Physical position | Physical distance from the previous SNP | Reference allele | Alternative allele | Major allele | Major allele frequency | Minor allele | Minor allele frequency | Number heterozygous | Heterozygosity |
|---|---|---|---|---|---|---|---|---|---|---|---|---|
| 9071 | S10_97056546 | 10 | 97056546 | 127 | T | C | T | 0.53226 | C | 0.46774 | 17 | 0.54839 |
| 9072 | S10_97206423 | 10 | 97206423 | 149877 | G | T | G | 0.90323 | T | 0.09677 | 6 | 0.19355 |
| 9073 | S10_97206576 | 10 | 97206576 | 153 | G | A | G | 0.95161 | A | 0.04839 | 3 | 0.09677 |
| 9074 | S10_97206618 | 10 | 97206618 | 42 | G | A | G | 0.80645 | A | 0.19355 | 12 | 0.3871 |
| 9075 | S10_97236013 | 10 | 97236013 | 29395 | G | A | G | 0.72581 | A | 0.27419 | 13 | 0.41935 |
| 9076 | S10_97328599 | 10 | 97328599 | 92586 | T | C | T | 0.59677 | C | 0.40323 | 15 | 0.48387 |
| 9077 | S10_97481052 | 10 | 97481052 | 152453 | G | A | G | 0.64516 | A | 0.35484 | 14 | 0.45161 |
| 9078 | S10_97481070 | 10 | 97481070 | 18 | C | T | C | 0.83871 | T | 0.16129 | 10 | 0.32258 |
| 9079 | S10_97481102 | 10 | 97481102 | 32 | C | T | C | 0.95161 | T | 0.04839 | 3 | 0.09677 |
| 9080 | S10_97481283 | 10 | 97481283 | 181 | A | G | A | 0.59677 | G | 0.40323 | 15 | 0.48387 |
| 9081 | S10_97518053 | 10 | 97518053 | 36770 | C | G | C | 0.80645 | G | 0.19355 | 10 | 0.32258 |
| 9082 | S10_97698799 | 10 | 97698799 | 180746 | C | T | C | 0.53226 | T | 0.46774 | 15 | 0.48387 |
| 9083 | S10_97698803 | 10 | 97698803 | 4 | G | A | G | 0.90323 | A | 0.09677 | 6 | 0.19355 |
| 9084 | S10_97698818 | 10 | 97698818 | 15 | G | C | C | 0.5 | G | 0.5 | 13 | 0.41935 |
| 9085 | S10_97720753 | 10 | 97720753 | 21935 | G | A | G | 0.93548 | A | 0.06452 | 4 | 0.12903 |
| 9086 | S10_97720768 | 10 | 97720768 | 15 | C | A | C | 0.93548 | A | 0.06452 | 4 | 0.12903 |
| 9087 | S10_97720844 | 10 | 97720844 | 76 | C | A | C | 0.93548 | A | 0.06452 | 4 | 0.12903 |
| 9088 | S10_97720862 | 10 | 97720862 | 18 | C | T | T | 0.8871 | C | 0.1129 | 5 | 0.16129 |
| 9089 | S10_97720890 | 10 | 97720890 | 28 | C | T | C | 0.67742 | T | 0.32258 | 16 | 0.51613 |
| 9090 | S10_97720974 | 10 | 97720974 | 84 | C | A | C | 0.93548 | A | 0.06452 | 4 | 0.12903 |
| 9091 | S10_98030907 | 10 | 98030907 | 309933 | T | C | T | 0.66129 | C | 0.33871 | 13 | 0.41935 |
| 9092 | S10_98030943 | 10 | 98030943 | 36 | C | T | C | 0.85484 | T | 0.14516 | 9 | 0.29032 |
| 9093 | S10_98031029 | 10 | 98031029 | 86 | A | G | A | 0.85484 | G | 0.14516 | 9 | 0.29032 |
| 9094 | S10_98031068 | 10 | 98031068 | 39 | G | A | G | 0.83871 | A | 0.16129 | 10 | 0.32258 |
| 9095 | S10_98652703 | 10 | 98652703 | 621635 | T | C | T | 0.80645 | C | 0.19355 | 10 | 0.32258 |
| 9096 | S10_98652835 | 10 | 98652835 | 132 | G | A | G | 0.80645 | A | 0.19355 | 10 | 0.32258 |
| 9097 | S10_99081063 | 10 | 99081063 | 428228 | G | A | A | 0.66129 | G | 0.33871 | 13 | 0.41935 |
| 9098 | S10_99123563 | 10 | 99123563 | 42500 | C | T | C | 0.91935 | T | 0.08065 | 5 | 0.16129 |
| 9099 | S10_99218048 | 10 | 99218048 | 94485 | G | A | G | 0.83871 | A | 0.16129 | 10 | 0.32258 |
| 9100 | S10_99222973 | 10 | 99222973 | 4925 | A | G | A | 0.95161 | G | 0.04839 | 3 | 0.09677 |
| 9101 | S10_99675051 | 10 | 99675051 | 452078 | C | G | C | 0.8871 | G | 0.1129 | 7 | 0.22581 |
| 9102 | S10_99675090 | 10 | 99675090 | 39 | T | C | T | 0.87097 | C | 0.12903 | 4 | 0.12903 |
| 9103 | S10_99687213 | 10 | 99687213 | 12123 | G | A | G | 0.91935 | A | 0.08065 | 5 | 0.16129 |
| 9104 | S10_99687214 | 10 | 99687214 | 1 | T | C | T | 0.8871 | C | 0.1129 | 7 | 0.22581 |
| 9105 | S10_99687216 | 10 | 99687216 | 2 | A | G | A | 0.95161 | G | 0.04839 | 3 | 0.09677 |
| 9106 | S10_99687236 | 10 | 99687236 | 20 | T | C | T | 0.83871 | C | 0.16129 | 6 | 0.19355 |
| 9107 | S10_99687258 | 10 | 99687258 | 22 | G | T | G | 0.95161 | T | 0.04839 | 3 | 0.09677 |
| 9108 | S10_99687279 | 10 | 99687279 | 21 | T | C | T | 0.8871 | C | 0.1129 | 7 | 0.22581 |
| 9109 | S10_99687286 | 10 | 99687286 | 7 | G | A | G | 0.95161 | A | 0.04839 | 3 | 0.09677 |
| 9110 | S10_99687340 | 10 | 99687340 | 54 | A | G | A | 0.53226 | G | 0.46774 | 17 | 0.54839 |
| 9111 | S10_100315828 | 10 | 100315828 | 628488 | A | G | A | 0.69355 | G | 0.30645 | 13 | 0.41935 |
| 9112 | S10_100315966 | 10 | 100315966 | 138 | T | A | T | 0.69355 | A | 0.30645 | 13 | 0.41935 |
| 9113 | S10_100343585 | 10 | 100343585 | 27619 | G | A | G | 0.90323 | A | 0.09677 | 6 | 0.19355 |
| 9114 | S10_100445695 | 10 | 100445695 | 102110 | A | G | G | 0.77419 | A | 0.22581 | 14 | 0.45161 |
| 9115 | S10_100607256 | 10 | 100607256 | 161561 | C | T | C | 0.95161 | T | 0.04839 | 3 | 0.09677 |
| 9116 | S11_97671 | 11 | 97671 | 0 | G | A | G | 0.62903 | A | 0.37097 | 15 | 0.48387 |
| 9117 | S11_212317 | 11 | 212317 | 114646 | C | T | T | 0.83871 | C | 0.16129 | 10 | 0.32258 |
| 9118 | S11_212491 | 11 | 212491 | 174 | C | G | C | 0.64516 | G | 0.35484 | 14 | 0.45161 |
| 9119 | S11_619992 | 11 | 619992 | 407501 | C | G | C | 0.90323 | G | 0.09677 | 6 | 0.19355 |
| 9120 | S11_620028 | 11 | 620028 | 36 | C | T | T | 0.77419 | C | 0.22581 | 10 | 0.32258 |
| 9121 | S11_639606 | 11 | 639606 | 19578 | C | T | C | 0.95161 | T | 0.04839 | 3 | 0.09677 |
| 9122 | S11_766447 | 11 | 766447 | 126841 | T | G | G | 0.59677 | T | 0.40323 | 11 | 0.35484 |
| 9123 | S11_1046082 | 11 | 1046082 | 279635 | C | A | C | 0.62903 | A | 0.37097 | 13 | 0.41935 |
| 9124 | S11_1086008 | 11 | 1086008 | 39926 | C | T | C | 0.80645 | T | 0.19355 | 12 | 0.3871 |

| Site number | SNP name | Chromosome | Physical position | Physical distance from the previous SNP | Reference allele | Alternative allele | Major allele | Major allele frequency | Minor allele | Minor allele frequency | Number heterozygous | Heterozygosity |
|---|---|---|---|---|---|---|---|---|---|---|---|---|
| 9125 | S11_1127051 | 11 | 1127051 | 41043 | C | T | C | 0.82258 | T | 0.17742 | 9 | 0.29032 |
| 9126 | S11_1554606 | 11 | 1554606 | 427555 | C | T | C | 0.8871 | T | 0.1129 | 7 | 0.22581 |
| 9127 | S11_1648877 | 11 | 1648877 | 94271 | G | A | G | 0.69355 | A | 0.30645 | 17 | 0.54839 |
| 9128 | S11_1679405 | 11 | 1679405 | 30528 | C | T | C | 0.66129 | T | 0.33871 | 13 | 0.41935 |
| 9129 | S11_1679441 | 11 | 1679441 | 36 | G | A | G | 0.66129 | A | 0.33871 | 13 | 0.41935 |
| 9130 | S11_1693332 | 11 | 1693332 | 13891 | A | C | A | 0.83871 | C | 0.16129 | 8 | 0.25806 |
| 9131 | S11_1693373 | 11 | 1693373 | 41 | A | G | G | 0.93548 | A | 0.06452 | 4 | 0.12903 |
| 9132 | S11_2555064 | 11 | 2555064 | 861691 | G | A | G | 0.91935 | A | 0.08065 | 5 | 0.16129 |
| 9133 | S11_2555106 | 11 | 2555106 | 42 | A | G | A | 0.79032 | G | 0.20968 | 13 | 0.41935 |
| 9134 | S11_2555222 | 11 | 2555222 | 116 | C | T | C | 0.79032 | T | 0.20968 | 13 | 0.41935 |
| 9135 | S11_2555238 | 11 | 2555238 | 16 | G | A | G | 0.77419 | A | 0.22581 | 12 | 0.3871 |
| 9136 | S11_2555278 | 11 | 2555278 | 40 | G | A | G | 0.80645 | A | 0.19355 | 12 | 0.3871 |
| 9137 | S11_2555286 | 11 | 2555286 | 8 | T | G | T | 0.75806 | G | 0.24194 | 15 | 0.48387 |
| 9138 | S11_2778270 | 11 | 2778270 | 222984 | C | A | C | 0.8871 | A | 0.1129 | 7 | 0.22581 |
| 9139 | S11_2778286 | 11 | 2778286 | 16 | G | A | G | 0.6129 | A | 0.3871 | 16 | 0.51613 |
| 9140 | S11_2778290 | 11 | 2778290 | 4 | G | A | A | 0.79032 | G | 0.20968 | 11 | 0.35484 |
| 9141 | S11_2778300 | 11 | 2778300 | 10 | T | C | T | 0.8871 | C | 0.1129 | 7 | 0.22581 |
| 9142 | S11_2963811 | 11 | 2963811 | 185511 | G | A | G | 0.93548 | A | 0.06452 | 4 | 0.12903 |
| 9143 | S11_3268627 | 11 | 3268627 | 304816 | C | T | T | 0.74194 | C | 0.25806 | 8 | 0.25806 |
| 9144 | S11_3268797 | 11 | 3268797 | 170 | A | G | G | 0.85484 | A | 0.14516 | 7 | 0.22581 |
| 9145 | S11_3402467 | 11 | 3402467 | 133670 | A | C | A | 0.79032 | C | 0.20968 | 7 | 0.22581 |
| 9146 | S11_3435153 | 11 | 3435153 | 32686 | T | C | C | 0.83871 | T | 0.16129 | 10 | 0.32258 |
| 9147 | S11_4026606 | 11 | 4026606 | 591453 | T | C | T | 0.74194 | C | 0.25806 | 14 | 0.45161 |
| 9148 | S11_4026665 | 11 | 4026665 | 59 | G | A | A | 0.67742 | G | 0.32258 | 14 | 0.45161 |
| 9149 | S11_4050045 | 11 | 4050045 | 23380 | C | T | C | 0.93548 | T | 0.06452 | 4 | 0.12903 |
| 9150 | S11_4333804 | 11 | 4333804 | 283759 | G | A | A | 0.8871 | G | 0.1129 | 5 | 0.16129 |
| 9151 | S11_4333807 | 11 | 4333807 | 3 | C | T | C | 0.91935 | T | 0.08065 | 3 | 0.09677 |
| 9152 | S11_4333833 | 11 | 4333833 | 26 | C | T | C | 0.72581 | T | 0.27419 | 15 | 0.48387 |
| 9153 | S11_4389414 | 11 | 4389414 | 55581 | T | C | T | 0.56452 | C | 0.43548 | 13 | 0.41935 |
| 9154 | S11_4437635 | 11 | 4437635 | 48221 | T | C | C | 0.59677 | T | 0.40323 | 13 | 0.41935 |
| 9155 | S11_4830066 | 11 | 4830066 | 392431 | G | A | A | 0.69355 | G | 0.30645 | 13 | 0.41935 |
| 9156 | S11_4830232 | 11 | 4830232 | 166 | C | T | C | 0.83871 | T | 0.16129 | 10 | 0.32258 |
| 9157 | S11_4830259 | 11 | 4830259 | 27 | C | A | A | 0.69355 | C | 0.30645 | 13 | 0.41935 |
| 9158 | S11_4830319 | 11 | 4830319 | 60 | G | A | G | 0.87097 | A | 0.12903 | 8 | 0.25806 |
| 9159 | S11_4964852 | 11 | 4964852 | 134533 | A | C | C | 0.69355 | A | 0.30645 | 11 | 0.35484 |
| 9160 | S11_4964878 | 11 | 4964878 | 26 | T | C | C | 0.69355 | T | 0.30645 | 11 | 0.35484 |
| 9161 | S11_4969174 | 11 | 4969174 | 4296 | A | C | A | 0.80645 | C | 0.19355 | 6 | 0.19355 |
| 9162 | S11_4969185 | 11 | 4969185 | 11 | T | C | T | 0.93548 | C | 0.06452 | 2 | 0.06452 |
| 9163 | S11_5027214 | 11 | 5027214 | 58029 | T | G | G | 0.8871 | T | 0.1129 | 7 | 0.22581 |
| 9164 | S11_5027446 | 11 | 5027446 | 232 | G | A | G | 0.59677 | A | 0.40323 | 19 | 0.6129 |
| 9165 | S11_5254645 | 11 | 5254645 | 227199 | A | G | A | 0.83871 | G | 0.16129 | 8 | 0.25806 |
| 9166 | S11_5254807 | 11 | 5254807 | 162 | G | A | G | 0.95161 | A | 0.04839 | 3 | 0.09677 |
| 9167 | S11_5254857 | 11 | 5254857 | 50 | A | G | A | 0.85484 | G | 0.14516 | 7 | 0.22581 |
| 9168 | S11_5254858 | 11 | 5254858 | 1 | C | T | C | 0.95161 | T | 0.04839 | 3 | 0.09677 |
| 9169 | S11_5395701 | 11 | 5395701 | 140843 | G | A | G | 0.75806 | A | 0.24194 | 9 | 0.29032 |
| 9170 | S11_5395733 | 11 | 5395733 | 32 | C | T | C | 0.83871 | T | 0.16129 | 6 | 0.19355 |
| 9171 | S11_5395761 | 11 | 5395761 | 28 | T | C | T | 0.85484 | C | 0.14516 | 7 | 0.22581 |
| 9172 | S11_5452858 | 11 | 5452858 | 57097 | T | C | T | 0.75806 | C | 0.24194 | 11 | 0.35484 |
| 9173 | S11_5452891 | 11 | 5452891 | 33 | A | G | A | 0.87097 | G | 0.12903 | 8 | 0.25806 |
| 9174 | S11_5505278 | 11 | 5505278 | 52387 | C | A | C | 0.72581 | A | 0.27419 | 11 | 0.35484 |
| 9175 | S11_5505299 | 11 | 5505299 | 21 | C | T | C | 0.72581 | T | 0.27419 | 11 | 0.35484 |
| 9176 | S11_5505387 | 11 | 5505387 | 88 | C | T | C | 0.72581 | T | 0.27419 | 11 | 0.35484 |
| 9177 | S11_5505431 | 11 | 5505431 | 44 | C | T | C | 0.72581 | T | 0.27419 | 11 | 0.35484 |
| 9178 | S11_5505445 | 11 | 5505445 | 14 | C | T | C | 0.72581 | T | 0.27419 | 11 | 0.35484 |

| Site number | SNP name | Chromosome | Physical position | Physical distance from the previous SNP | Reference allele | Alternative allele | Major allele | Major allele frequency | Minor allele | Minor allele frequency | Number heterozygous | Heterozygosity |
|---|---|---|---|---|---|---|---|---|---|---|---|---|
| 9179 | S11_5505446 | 11 | 5505446 | 1 | A | G | A | 0.72581 | G | 0.27419 | 11 | 0.35484 |
| 9180 | S11_5505454 | 11 | 5505454 | 8 | G | A | G | 0.72581 | A | 0.27419 | 11 | 0.35484 |
| 9181 | S11_5505492 | 11 | 5505492 | 38 | C | A | C | 0.72581 | A | 0.27419 | 11 | 0.35484 |
| 9182 | S11_5510910 | 11 | 5510910 | 5418 | G | A | G | 0.70968 | A | 0.29032 | 16 | 0.51613 |
| 9183 | S11_5511006 | 11 | 5511006 | 96 | G | A | G | 0.72581 | A | 0.27419 | 13 | 0.41935 |
| 9184 | S11_6036210 | 11 | 6036210 | 525204 | C | T | C | 0.53226 | T | 0.46774 | 15 | 0.48387 |
| 9185 | S11_6036276 | 11 | 6036276 | 66 | A | G | A | 0.83871 | G | 0.16129 | 10 | 0.32258 |
| 9186 | S11_6059938 | 11 | 6059938 | 23662 | G | A | G | 0.91935 | A | 0.08065 | 5 | 0.16129 |
| 9187 | S11_6060114 | 11 | 6060114 | 176 | G | A | G | 0.91935 | A | 0.08065 | 5 | 0.16129 |
| 9188 | S11_6299086 | 11 | 6299086 | 238972 | C | T | C | 0.91935 | T | 0.08065 | 5 | 0.16129 |
| 9189 | S11_6299115 | 11 | 6299115 | 29 | T | C | T | 0.80645 | C | 0.19355 | 10 | 0.32258 |
| 9190 | S11_6389403 | 11 | 6389403 | 90288 | T | A | A | 0.85484 | T | 0.14516 | 9 | 0.29032 |
| 9191 | S11_6389411 | 11 | 6389411 | 8 | G | T | T | 0.54839 | G | 0.45161 | 20 | 0.64516 |
| 9192 | S11_6389429 | 11 | 6389429 | 18 | G | A | G | 0.87097 | A | 0.12903 | 8 | 0.25806 |
| 9193 | S11_6389440 | 11 | 6389440 | 11 | T | C | T | 0.90323 | C | 0.09677 | 4 | 0.12903 |
| 9194 | S11_6389492 | 11 | 6389492 | 52 | T | C | T | 0.91935 | C | 0.08065 | 5 | 0.16129 |
| 9195 | S11_6902730 | 11 | 6902730 | 513238 | A | G | A | 0.8871 | G | 0.1129 | 7 | 0.22581 |
| 9196 | S11_6902745 | 11 | 6902745 | 15 | C | T | C | 0.91935 | T | 0.08065 | 5 | 0.16129 |
| 9197 | S11_6902766 | 11 | 6902766 | 21 | G | A | A | 0.83871 | G | 0.16129 | 10 | 0.32258 |
| 9198 | S11_7236612 | 11 | 7236612 | 333846 | C | T | C | 0.58065 | T | 0.41935 | 18 | 0.58065 |
| 9199 | S11_7236613 | 11 | 7236613 | 1 | G | T | G | 0.58065 | T | 0.41935 | 18 | 0.58065 |
| 9200 | S11_7592994 | 11 | 7592994 | 356381 | G | A | G | 0.93548 | A | 0.06452 | 4 | 0.12903 |
| 9201 | S11_7593059 | 11 | 7593059 | 65 | G | A | G | 0.90323 | A | 0.09677 | 6 | 0.19355 |
| 9202 | S11_7637813 | 11 | 7637813 | 44754 | C | T | C | 0.80645 | T | 0.19355 | 12 | 0.3871 |
| 9203 | S11_7637816 | 11 | 7637816 | 3 | G | C | C | 0.91935 | G | 0.08065 | 5 | 0.16129 |
| 9204 | S11_7637849 | 11 | 7637849 | 33 | G | A | A | 0.91935 | G | 0.08065 | 5 | 0.16129 |
| 9205 | S11_7637926 | 11 | 7637926 | 77 | C | T | T | 0.72581 | C | 0.27419 | 15 | 0.48387 |
| 9206 | S11_7637932 | 11 | 7637932 | 6 | C | T | C | 0.90323 | T | 0.09677 | 6 | 0.19355 |
| 9207 | S11_7637953 | 11 | 7637953 | 21 | G | A | G | 0.95161 | A | 0.04839 | 3 | 0.09677 |
| 9208 | S11_7751513 | 11 | 7751513 | 113560 | C | A | C | 0.69355 | A | 0.30645 | 17 | 0.54839 |
| 9209 | S11_7751536 | 11 | 7751536 | 23 | G | A | A | 0.85484 | G | 0.14516 | 7 | 0.22581 |
| 9210 | S11_7751689 | 11 | 7751689 | 153 | G | C | G | 0.95161 | C | 0.04839 | 3 | 0.09677 |
| 9211 | S11_7751693 | 11 | 7751693 | 4 | T | A | T | 0.8871 | A | 0.1129 | 7 | 0.22581 |
| 9212 | S11_7816578 | 11 | 7816578 | 64885 | G | A | G | 0.75806 | A | 0.24194 | 13 | 0.41935 |
| 9213 | S11_7816585 | 11 | 7816585 | 7 | G | A | G | 0.91935 | A | 0.08065 | 5 | 0.16129 |
| 9214 | S11_8223229 | 11 | 8223229 | 406644 | A | G | A | 0.83871 | G | 0.16129 | 8 | 0.25806 |
| 9215 | S11_8223331 | 11 | 8223331 | 102 | C | T | C | 0.75806 | T | 0.24194 | 13 | 0.41935 |
| 9216 | S11_8333643 | 11 | 8333643 | 110312 | T | C | C | 0.74194 | T | 0.25806 | 8 | 0.25806 |
| 9217 | S11_8581020 | 11 | 8581020 | 247377 | T | C | T | 0.67742 | C | 0.32258 | 14 | 0.45161 |
| 9218 | S11_8581225 | 11 | 8581225 | 205 | C | T | C | 0.93548 | T | 0.06452 | 4 | 0.12903 |
| 9219 | S11_8581226 | 11 | 8581226 | 1 | A | G | A | 0.82258 | G | 0.17742 | 11 | 0.35484 |
| 9220 | S11_8638625 | 11 | 8638625 | 57399 | G | A | G | 0.93548 | A | 0.06452 | 2 | 0.06452 |
| 9221 | S11_8838583 | 11 | 8838583 | 199958 | G | A | G | 0.90323 | A | 0.09677 | 6 | 0.19355 |
| 9222 | S11_9032241 | 11 | 9032241 | 193658 | A | G | A | 0.8871 | G | 0.1129 | 7 | 0.22581 |
| 9223 | S11_9032292 | 11 | 9032292 | 51 | A | G | A | 0.8871 | G | 0.1129 | 7 | 0.22581 |
| 9224 | S11_9032344 | 11 | 9032344 | 52 | C | A | A | 0.83871 | C | 0.16129 | 10 | 0.32258 |
| 9225 | S11_9032361 | 11 | 9032361 | 17 | T | C | T | 0.82258 | C | 0.17742 | 11 | 0.35484 |
| 9226 | S11_9563594 | 11 | 9563594 | 531233 | G | A | G | 0.93548 | A | 0.06452 | 4 | 0.12903 |
| 9227 | S11_9563705 | 11 | 9563705 | 111 | C | G | G | 0.66129 | C | 0.33871 | 17 | 0.54839 |
| 9228 | S11_9563739 | 11 | 9563739 | 34 | C | T | C | 0.93548 | T | 0.06452 | 4 | 0.12903 |
| 9229 | S11_9703923 | 11 | 9703923 | 140184 | A | G | A | 0.8871 | G | 0.1129 | 7 | 0.22581 |
| 9230 | S11_9703928 | 11 | 9703928 | 5 | C | T | C | 0.8871 | T | 0.1129 | 7 | 0.22581 |
| 9231 | S11_9703955 | 11 | 9703955 | 27 | G | A | G | 0.8871 | A | 0.1129 | 7 | 0.22581 |
| 9232 | S11_9760819 | 11 | 9760819 | 56864 | T | C | C | 0.79032 | T | 0.20968 | 11 | 0.35484 |

| Site number | SNP name | Chromosome | Physical position | Physical distance from the previous SNP | Reference allele | Alternative allele | Major allele | Major allele frequency | Minor allele | Minor allele frequency | Number heterozygous | Heterozygosity |
|---|---|---|---|---|---|---|---|---|---|---|---|---|
| 9233 | S11_9760865 | 11 | 9760865 | 46 | A | G | G | 0.67742 | A | 0.32258 | 16 | 0.51613 |
| 9234 | S11_9760997 | 11 | 9760997 | 132 | T | C | C | 0.74194 | T | 0.25806 | 14 | 0.45161 |
| 9235 | S11_10008281 | 11 | 10008281 | 247284 | G | A | G | 0.82258 | A | 0.17742 | 11 | 0.35484 |
| 9236 | S11_10115282 | 11 | 10115282 | 107001 | G | A | G | 0.93548 | A | 0.06452 | 4 | 0.12903 |
| 9237 | S11_10146311 | 11 | 10146311 | 31029 | G | A | A | 0.54839 | G | 0.45161 | 16 | 0.51613 |
| 9238 | S11_10264898 | 11 | 10264898 | 118587 | T | C | T | 0.93548 | C | 0.06452 | 4 | 0.12903 |
| 9239 | S11_10264903 | 11 | 10264903 | 5 | T | C | T | 0.85484 | C | 0.14516 | 9 | 0.29032 |
| 9240 | S11_10264936 | 11 | 10264936 | 33 | C | T | C | 0.87097 | T | 0.12903 | 8 | 0.25806 |
| 9241 | S11_10264996 | 11 | 10264996 | 60 | T | C | C | 0.85484 | T | 0.14516 | 9 | 0.29032 |
| 9242 | S11_10265059 | 11 | 10265059 | 63 | A | T | T | 0.54839 | A | 0.45161 | 18 | 0.58065 |
| 9243 | S11_10265097 | 11 | 10265097 | 38 | C | T | T | 0.54839 | C | 0.45161 | 18 | 0.58065 |
| 9244 | S11_10265104 | 11 | 10265104 | 7 | G | A | G | 0.58065 | A | 0.41935 | 14 | 0.45161 |
| 9245 | S11_10933680 | 11 | 10933680 | 668576 | T | C | T | 0.69355 | C | 0.30645 | 13 | 0.41935 |
| 9246 | S11_10933797 | 11 | 10933797 | 117 | T | G | G | 0.67742 | T | 0.32258 | 12 | 0.3871 |
| 9247 | S11_10933816 | 11 | 10933816 | 19 | C | T | T | 0.67742 | C | 0.32258 | 12 | 0.3871 |
| 9248 | S11_10933856 | 11 | 10933856 | 40 | C | T | T | 0.67742 | C | 0.32258 | 12 | 0.3871 |
| 9249 | S11_11005137 | 11 | 11005137 | 71281 | G | A | G | 0.51613 | A | 0.48387 | 10 | 0.32258 |
| 9250 | S11_11005168 | 11 | 11005168 | 31 | C | T | C | 0.93548 | T | 0.06452 | 4 | 0.12903 |
| 9251 | S11_11005213 | 11 | 11005213 | 45 | C | T | C | 0.51613 | T | 0.48387 | 10 | 0.32258 |
| 9252 | S11_11005227 | 11 | 11005227 | 14 | G | A | G | 0.93548 | A | 0.06452 | 4 | 0.12903 |
| 9253 | S11_11005273 | 11 | 11005273 | 46 | G | A | G | 0.75806 | A | 0.24194 | 11 | 0.35484 |
| 9254 | S11_11005342 | 11 | 11005342 | 69 | G | C | C | 0.79032 | G | 0.20968 | 11 | 0.35484 |
| 9255 | S11_11005353 | 11 | 11005353 | 11 | A | G | G | 0.79032 | A | 0.20968 | 11 | 0.35484 |
| 9256 | S11_11005373 | 11 | 11005373 | 20 | A | G | G | 0.85484 | A | 0.14516 | 9 | 0.29032 |
| 9257 | S11_11125537 | 11 | 11125537 | 120164 | C | G | C | 0.93548 | G | 0.06452 | 4 | 0.12903 |
| 9258 | S11_11125651 | 11 | 11125651 | 114 | C | T | C | 0.93548 | T | 0.06452 | 4 | 0.12903 |
| 9259 | S11_11180472 | 11 | 11180472 | 54821 | T | C | T | 0.91935 | C | 0.08065 | 5 | 0.16129 |
| 9260 | S11_11180589 | 11 | 11180589 | 117 | G | T | G | 0.59677 | T | 0.40323 | 19 | 0.6129 |
| 9261 | S11_11461238 | 11 | 11461238 | 280649 | T | C | T | 0.95161 | C | 0.04839 | 3 | 0.09677 |
| 9262 | S11_11831264 | 11 | 11831264 | 370026 | G | A | G | 0.74194 | A | 0.25806 | 10 | 0.32258 |
| 9263 | S11_12064510 | 11 | 12064510 | 233246 | C | T | C | 0.93548 | T | 0.06452 | 4 | 0.12903 |
| 9264 | S11_12326048 | 11 | 12326048 | 261538 | A | G | A | 0.85484 | G | 0.14516 | 9 | 0.29032 |
| 9265 | S11_12434171 | 11 | 12434171 | 108123 | G | A | A | 0.64516 | G | 0.35484 | 14 | 0.45161 |
| 9266 | S11_12574778 | 11 | 12574778 | 140607 | C | T | T | 0.58065 | C | 0.41935 | 16 | 0.51613 |
| 9267 | S11_12574791 | 11 | 12574791 | 13 | C | T | C | 0.91935 | T | 0.08065 | 5 | 0.16129 |
| 9268 | S11_12574820 | 11 | 12574820 | 29 | T | C | T | 0.62903 | C | 0.37097 | 15 | 0.48387 |
| 9269 | S11_12574960 | 11 | 12574960 | 140 | C | T | C | 0.87097 | T | 0.12903 | 8 | 0.25806 |
| 9270 | S11_12574981 | 11 | 12574981 | 21 | G | A | A | 0.74194 | G | 0.25806 | 14 | 0.45161 |
| 9271 | S11_12879500 | 11 | 12879500 | 304519 | C | T | C | 0.77419 | T | 0.22581 | 10 | 0.32258 |
| 9272 | S11_12879501 | 11 | 12879501 | 1 | C | G | C | 0.6129 | G | 0.3871 | 16 | 0.51613 |
| 9273 | S11_12879563 | 11 | 12879563 | 62 | G | A | G | 0.93548 | A | 0.06452 | 4 | 0.12903 |
| 9274 | S11_12979663 | 11 | 12979663 | 100100 | C | T | C | 0.91935 | T | 0.08065 | 5 | 0.16129 |
| 9275 | S11_12979742 | 11 | 12979742 | 79 | A | G | A | 0.91935 | G | 0.08065 | 5 | 0.16129 |
| 9276 | S11_12979752 | 11 | 12979752 | 10 | T | C | T | 0.91935 | C | 0.08065 | 5 | 0.16129 |
| 9277 | S11_12979803 | 11 | 12979803 | 51 | C | A | C | 0.91935 | A | 0.08065 | 5 | 0.16129 |
| 9278 | S11_12979807 | 11 | 12979807 | 4 | G | A | G | 0.91935 | A | 0.08065 | 5 | 0.16129 |
| 9279 | S11_12979837 | 11 | 12979837 | 30 | A | G | G | 0.53226 | A | 0.46774 | 13 | 0.41935 |
| 9280 | S11_13213920 | 11 | 13213920 | 234083 | C | T | C | 0.62903 | T | 0.37097 | 13 | 0.41935 |
| 9281 | S11_13214101 | 11 | 13214101 | 181 | T | C | C | 0.5 | T | 0.5 | 19 | 0.6129 |
| 9282 | S11_13214155 | 11 | 13214155 | 54 | T | G | T | 0.90323 | G | 0.09677 | 4 | 0.12903 |
| 9283 | S11_13214211 | 11 | 13214211 | 56 | T | C | T | 0.51613 | C | 0.48387 | 18 | 0.58065 |
| 9284 | S11_13214237 | 11 | 13214237 | 26 | G | A | G | 0.91935 | A | 0.08065 | 5 | 0.16129 |
| 9285 | S11_13214287 | 11 | 13214287 | 50 | C | G | C | 0.51613 | G | 0.48387 | 18 | 0.58065 |
| 9286 | S11_13214320 | 11 | 13214320 | 33 | C | T | C | 0.59677 | T | 0.40323 | 19 | 0.6129 |

| Site number | SNP name | Chromosome | Physical position | Physical distance from the previous SNP | Reference allele | Alternative allele | Major allele | Major allele frequency | Minor allele | Minor allele frequency | Number heterozygous | Heterozygosity |
|---|---|---|---|---|---|---|---|---|---|---|---|---|
| 9287 | S11_13239083 | 11 | 13239083 | 24763 | C | T | C | 0.95161 | T | 0.04839 | 3 | 0.09677 |
| 9288 | S11_13485504 | 11 | 13485504 | 246421 | A | T | A | 0.82258 | T | 0.17742 | 7 | 0.22581 |
| 9289 | S11_13485538 | 11 | 13485538 | 34 | G | T | G | 0.91935 | T | 0.08065 | 5 | 0.16129 |
| 9290 | S11_14622886 | 11 | 14622886 | 1137348 | T | C | T | 0.72581 | C | 0.27419 | 13 | 0.41935 |
| 9291 | S11_15004147 | 11 | 15004147 | 381261 | G | A | G | 0.95161 | A | 0.04839 | 3 | 0.09677 |
| 9292 | S11_15157005 | 11 | 15157005 | 152858 | G | T | G | 0.95161 | T | 0.04839 | 3 | 0.09677 |
| 9293 | S11_15157006 | 11 | 15157006 | 1 | C | T | C | 0.95161 | T | 0.04839 | 3 | 0.09677 |
| 9294 | S11_15276510 | 11 | 15276510 | 119504 | G | A | G | 0.64516 | A | 0.35484 | 12 | 0.3871 |
| 9295 | S11_15276671 | 11 | 15276671 | 161 | A | G | A | 0.64516 | G | 0.35484 | 12 | 0.3871 |
| 9296 | S11_15347637 | 11 | 15347637 | 70966 | G | T | G | 0.90323 | T | 0.09677 | 4 | 0.12903 |
| 9297 | S11_15349664 | 11 | 15349664 | 2027 | A | C | A | 0.93548 | C | 0.06452 | 4 | 0.12903 |
| 9298 | S11_15349780 | 11 | 15349780 | 116 | G | A | G | 0.93548 | A | 0.06452 | 4 | 0.12903 |
| 9299 | S11_15349846 | 11 | 15349846 | 66 | A | G | A | 0.93548 | G | 0.06452 | 4 | 0.12903 |
| 9300 | S11_15557279 | 11 | 15557279 | 207433 | C | G | C | 0.72581 | G | 0.27419 | 13 | 0.41935 |
| 9301 | S11_15557298 | 11 | 15557298 | 19 | A | G | A | 0.93548 | G | 0.06452 | 2 | 0.06452 |
| 9302 | S11_15596165 | 11 | 15596165 | 38867 | C | T | C | 0.90323 | T | 0.09677 | 6 | 0.19355 |
| 9303 | S11_15596171 | 11 | 15596171 | 6 | C | T | C | 0.93548 | T | 0.06452 | 4 | 0.12903 |
| 9304 | S11_15596179 | 11 | 15596179 | 8 | C | T | C | 0.93548 | T | 0.06452 | 4 | 0.12903 |
| 9305 | S11_17626030 | 11 | 17626030 | 2029851 | G | A | G | 0.85484 | A | 0.14516 | 7 | 0.22581 |
| 9306 | S11_17626092 | 11 | 17626092 | 62 | A | G | A | 0.85484 | G | 0.14516 | 7 | 0.22581 |
| 9307 | S11_17626170 | 11 | 17626170 | 78 | C | A | C | 0.95161 | A | 0.04839 | 3 | 0.09677 |
| 9308 | S11_17626176 | 11 | 17626176 | 6 | C | T | T | 0.54839 | C | 0.45161 | 14 | 0.45161 |
| 9309 | S11_17626184 | 11 | 17626184 | 8 | T | C | T | 0.85484 | C | 0.14516 | 7 | 0.22581 |
| 9310 | S11_17626207 | 11 | 17626207 | 23 | A | C | A | 0.85484 | C | 0.14516 | 7 | 0.22581 |
| 9311 | S11_17734263 | 11 | 17734263 | 108056 | G | C | G | 0.95161 | C | 0.04839 | 3 | 0.09677 |
| 9312 | S11_17734270 | 11 | 17734270 | 7 | C | T | C | 0.93548 | T | 0.06452 | 4 | 0.12903 |
| 9313 | S11_18010102 | 11 | 18010102 | 275832 | G | A | G | 0.59677 | A | 0.40323 | 15 | 0.48387 |
| 9314 | S11_18010107 | 11 | 18010107 | 5 | C | T | C | 0.95161 | T | 0.04839 | 3 | 0.09677 |
| 9315 | S11_18010117 | 11 | 18010117 | 10 | T | C | T | 0.8871 | C | 0.1129 | 7 | 0.22581 |
| 9316 | S11_18785463 | 11 | 18785463 | 775346 | A | G | A | 0.83871 | G | 0.16129 | 10 | 0.32258 |
| 9317 | S11_18785471 | 11 | 18785471 | 8 | G | A | G | 0.90323 | A | 0.09677 | 6 | 0.19355 |
| 9318 | S11_18785490 | 11 | 18785490 | 19 | C | T | C | 0.62903 | T | 0.37097 | 17 | 0.54839 |
| 9319 | S11_18785541 | 11 | 18785541 | 51 | C | T | C | 0.90323 | T | 0.09677 | 6 | 0.19355 |
| 9320 | S11_18929060 | 11 | 18929060 | 143519 | G | A | A | 0.87097 | G | 0.12903 | 6 | 0.19355 |
| 9321 | S11_18929094 | 11 | 18929094 | 34 | T | A | A | 0.74194 | T | 0.25806 | 12 | 0.3871 |
| 9322 | S11_18929132 | 11 | 18929132 | 38 | T | G | T | 0.93548 | G | 0.06452 | 4 | 0.12903 |
| 9323 | S11_18936878 | 11 | 18936878 | 7746 | G | A | G | 0.69355 | A | 0.30645 | 11 | 0.35484 |
| 9324 | S11_18967977 | 11 | 18967977 | 31099 | A | G | G | 0.90323 | A | 0.09677 | 6 | 0.19355 |
| 9325 | S11_19366812 | 11 | 19366812 | 398835 | C | T | C | 0.62903 | T | 0.37097 | 11 | 0.35484 |
| 9326 | S11_19366842 | 11 | 19366842 | 30 | T | C | T | 0.62903 | C | 0.37097 | 11 | 0.35484 |
| 9327 | S11_19366895 | 11 | 19366895 | 53 | G | A | A | 0.53226 | G | 0.46774 | 11 | 0.35484 |
| 9328 | S11_19366912 | 11 | 19366912 | 17 | C | T | C | 0.91935 | T | 0.08065 | 5 | 0.16129 |
| 9329 | S11_19367001 | 11 | 19367001 | 89 | C | T | C | 0.93548 | T | 0.06452 | 4 | 0.12903 |
| 9330 | S11_19367009 | 11 | 19367009 | 8 | G | C | G | 0.93548 | C | 0.06452 | 4 | 0.12903 |
| 9331 | S11_19544953 | 11 | 19544953 | 177944 | G | A | G | 0.90323 | A | 0.09677 | 4 | 0.12903 |
| 9332 | S11_19545011 | 11 | 19545011 | 58 | C | T | C | 0.90323 | T | 0.09677 | 4 | 0.12903 |
| 9333 | S11_19999307 | 11 | 19999307 | 454296 | T | C | T | 0.72581 | C | 0.27419 | 15 | 0.48387 |
| 9334 | S11_19999362 | 11 | 19999362 | 55 | T | C | T | 0.72581 | C | 0.27419 | 15 | 0.48387 |
| 9335 | S11_19999464 | 11 | 19999464 | 102 | A | T | A | 0.95161 | T | 0.04839 | 3 | 0.09677 |
| 9336 | S11_20009438 | 11 | 20009438 | 9974 | T | C | C | 0.51613 | T | 0.48387 | 12 | 0.3871 |
| 9337 | S11_20611757 | 11 | 20611757 | 602319 | T | C | C | 0.75806 | T | 0.24194 | 13 | 0.41935 |
| 9338 | S11_20611786 | 11 | 20611786 | 29 | G | T | G | 0.8871 | T | 0.1129 | 7 | 0.22581 |
| 9339 | S11_20689249 | 11 | 20689249 | 77463 | A | G | G | 0.95161 | A | 0.04839 | 3 | 0.09677 |
| 9340 | S11_20889121 | 11 | 20889121 | 199872 | T | C | T | 0.83871 | C | 0.16129 | 10 | 0.32258 |

| Site number | SNP name | Chromosome | Physical position | Physical distance from the previous SNP | Reference allele | Alternative allele | Major allele | Major allele frequency | Minor allele | Minor allele frequency | Number heterozygous | Heterozygosity |
|---|---|---|---|---|---|---|---|---|---|---|---|---|
| 9341 | S11_20978294 | 11 | 20978294 | 89173 | T | G | G | 0.72581 | T | 0.27419 | 13 | 0.41935 |
| 9342 | S11_21719683 | 11 | 21719683 | 741389 | A | G | A | 0.80645 | G | 0.19355 | 8 | 0.25806 |
| 9343 | S11_21747374 | 11 | 21747374 | 27691 | C | T | T | 0.77419 | C | 0.22581 | 12 | 0.3871 |
| 9344 | S11_21747395 | 11 | 21747395 | 21 | C | T | C | 0.75806 | T | 0.24194 | 9 | 0.29032 |
| 9345 | S11_21747457 | 11 | 21747457 | 62 | C | T | C | 0.80645 | T | 0.19355 | 6 | 0.19355 |
| 9346 | S11_21747458 | 11 | 21747458 | 1 | C | A | C | 0.74194 | A | 0.25806 | 8 | 0.25806 |
| 9347 | S11_21747459 | 11 | 21747459 | 1 | G | A | G | 0.91935 | A | 0.08065 | 5 | 0.16129 |
| 9348 | S11_21747559 | 11 | 21747559 | 100 | G | A | G | 0.83871 | A | 0.16129 | 10 | 0.32258 |
| 9349 | S11_21747572 | 11 | 21747572 | 13 | C | T | C | 0.82258 | T | 0.17742 | 11 | 0.35484 |
| 9350 | S11_21747574 | 11 | 21747574 | 2 | G | A | G | 0.82258 | A | 0.17742 | 11 | 0.35484 |
| 9351 | S11_22307875 | 11 | 22307875 | 560301 | G | A | G | 0.93548 | A | 0.06452 | 4 | 0.12903 |
| 9352 | S11_22307973 | 11 | 22307973 | 98 | G | A | A | 0.56452 | G | 0.43548 | 15 | 0.48387 |
| 9353 | S11_22307993 | 11 | 22307993 | 20 | G | A | A | 0.56452 | G | 0.43548 | 15 | 0.48387 |
| 9354 | S11_22307994 | 11 | 22307994 | 1 | G | A | G | 0.91935 | A | 0.08065 | 5 | 0.16129 |
| 9355 | S11_22435904 | 11 | 22435904 | 127910 | C | A | C | 0.90323 | A | 0.09677 | 6 | 0.19355 |
| 9356 | S11_22509793 | 11 | 22509793 | 73889 | G | T | G | 0.91935 | T | 0.08065 | 3 | 0.09677 |
| 9357 | S11_22567452 | 11 | 22567452 | 57659 | T | C | T | 0.80645 | C | 0.19355 | 6 | 0.19355 |
| 9358 | S11_23300033 | 11 | 23300033 | 732581 | G | T | T | 0.91935 | G | 0.08065 | 5 | 0.16129 |
| 9359 | S11_23594751 | 11 | 23594751 | 294718 | A | G | A | 0.95161 | G | 0.04839 | 3 | 0.09677 |
| 9360 | S11_23594801 | 11 | 23594801 | 50 | C | T | C | 0.85484 | T | 0.14516 | 9 | 0.29032 |
| 9361 | S11_24754613 | 11 | 24754613 | 1159812 | C | T | C | 0.93548 | T | 0.06452 | 4 | 0.12903 |
| 9362 | S11_24754614 | 11 | 24754614 | 1 | G | A | G | 0.93548 | A | 0.06452 | 4 | 0.12903 |
| 9363 | S11_25010186 | 11 | 25010186 | 255572 | C | T | C | 0.93548 | T | 0.06452 | 4 | 0.12903 |
| 9364 | S11_25010194 | 11 | 25010194 | 8 | C | G | G | 0.90323 | C | 0.09677 | 6 | 0.19355 |
| 9365 | S11_25010300 | 11 | 25010300 | 106 | T | A | A | 0.91935 | T | 0.08065 | 5 | 0.16129 |
| 9366 | S11_25327380 | 11 | 25327380 | 317080 | G | A | G | 0.95161 | A | 0.04839 | 3 | 0.09677 |
| 9367 | S11_25523095 | 11 | 25523095 | 195715 | T | C | C | 0.95161 | T | 0.04839 | 3 | 0.09677 |
| 9368 | S11_25588125 | 11 | 25588125 | 65030 | C | T | T | 0.72581 | C | 0.27419 | 13 | 0.41935 |
| 9369 | S11_25588325 | 11 | 25588325 | 200 | C | G | C | 0.93548 | G | 0.06452 | 4 | 0.12903 |
| 9370 | S11_26148543 | 11 | 26148543 | 560218 | G | T | G | 0.75806 | T | 0.24194 | 13 | 0.41935 |
| 9371 | S11_26148645 | 11 | 26148645 | 102 | G | A | G | 0.56452 | A | 0.43548 | 21 | 0.67742 |
| 9372 | S11_26148747 | 11 | 26148747 | 102 | A | C | A | 0.93548 | C | 0.06452 | 4 | 0.12903 |
| 9373 | S11_26743869 | 11 | 26743869 | 595122 | C | T | C | 0.54839 | T | 0.45161 | 18 | 0.58065 |
| 9374 | S11_27017040 | 11 | 27017040 | 273171 | C | T | C | 0.75806 | T | 0.24194 | 11 | 0.35484 |
| 9375 | S11_27017252 | 11 | 27017252 | 212 | A | G | A | 0.62903 | G | 0.37097 | 11 | 0.35484 |
| 9376 | S11_27017284 | 11 | 27017284 | 32 | C | G | G | 0.82258 | C | 0.17742 | 7 | 0.22581 |
| 9377 | S11_27676337 | 11 | 27676337 | 659053 | C | T | C | 0.54839 | T | 0.45161 | 12 | 0.3871 |
| 9378 | S11_27726072 | 11 | 27726072 | 49735 | G | A | G | 0.58065 | A | 0.41935 | 16 | 0.51613 |
| 9379 | S11_27889007 | 11 | 27889007 | 162935 | G | A | G | 0.95161 | A | 0.04839 | 3 | 0.09677 |
| 9380 | S11_27889029 | 11 | 27889029 | 22 | A | G | A | 0.90323 | G | 0.09677 | 6 | 0.19355 |
| 9381 | S11_27889125 | 11 | 27889125 | 96 | G | A | G | 0.95161 | A | 0.04839 | 3 | 0.09677 |
| 9382 | S11_28188307 | 11 | 28188307 | 299182 | G | C | G | 0.77419 | C | 0.22581 | 10 | 0.32258 |
| 9383 | S11_28188416 | 11 | 28188416 | 109 | C | T | C | 0.72581 | T | 0.27419 | 13 | 0.41935 |
| 9384 | S11_28211072 | 11 | 28211072 | 22656 | G | A | A | 0.54839 | G | 0.45161 | 12 | 0.3871 |
| 9385 | S11_28398534 | 11 | 28398534 | 187462 | A | G | A | 0.91935 | G | 0.08065 | 3 | 0.09677 |
| 9386 | S11_28466524 | 11 | 28466524 | 67990 | G | T | G | 0.87097 | T | 0.12903 | 4 | 0.12903 |
| 9387 | S11_28466583 | 11 | 28466583 | 59 | A | G | A | 0.51613 | G | 0.48387 | 14 | 0.45161 |
| 9388 | S11_28466614 | 11 | 28466614 | 31 | A | G | A | 0.80645 | G | 0.19355 | 10 | 0.32258 |
| 9389 | S11_29167644 | 11 | 29167644 | 701030 | T | C | T | 0.82258 | C | 0.17742 | 11 | 0.35484 |
| 9390 | S11_29380980 | 11 | 29380980 | 213336 | C | T | C | 0.90323 | T | 0.09677 | 4 | 0.12903 |
| 9391 | S11_29816841 | 11 | 29816841 | 435861 | A | C | A | 0.8871 | C | 0.1129 | 5 | 0.16129 |
| 9392 | S11_29876015 | 11 | 29876015 | 59174 | C | T | C | 0.93548 | T | 0.06452 | 4 | 0.12903 |
| 9393 | S11_30217820 | 11 | 30217820 | 341805 | C | T | C | 0.6129 | T | 0.3871 | 14 | 0.45161 |
| 9394 | S11_30217823 | 11 | 30217823 | 3 | G | A | G | 0.82258 | A | 0.17742 | 11 | 0.35484 |

| Site number | SNP name | Chromosome | Physical position | Physical distance from the previous SNP | Reference allele | Alternative allele | Major allele | Major allele frequency | Minor allele | Minor allele frequency | Number heterozygous | Heterozygosity |
|---|---|---|---|---|---|---|---|---|---|---|---|---|
| 9395 | S11_30217935 | 11 | 30217935 | 112 | C | T | C | 0.82258 | T | 0.17742 | 11 | 0.35484 |
| 9396 | S11_34136425 | 11 | 34136425 | 3918490 | A | G | A | 0.6129 | G | 0.3871 | 14 | 0.45161 |
| 9397 | S11_34369582 | 11 | 34369582 | 233157 | G | T | G | 0.87097 | T | 0.12903 | 6 | 0.19355 |
| 9398 | S11_34369610 | 11 | 34369610 | 28 | G | A | G | 0.85484 | A | 0.14516 | 5 | 0.16129 |
| 9399 | S11_36055284 | 11 | 36055284 | 1685674 | G | A | G | 0.85484 | A | 0.14516 | 7 | 0.22581 |
| 9400 | S11_36055339 | 11 | 36055339 | 55 | T | C | T | 0.95161 | C | 0.04839 | 3 | 0.09677 |
| 9401 | S11_36156536 | 11 | 36156536 | 101197 | A | G | A | 0.58065 | G | 0.41935 | 12 | 0.3871 |
| 9402 | S11_36291695 | 11 | 36291695 | 135159 | T | C | C | 0.5 | T | 0.5 | 13 | 0.41935 |
| 9403 | S11_36291836 | 11 | 36291836 | 141 | C | T | C | 0.93548 | T | 0.06452 | 4 | 0.12903 |
| 9404 | S11_36546781 | 11 | 36546781 | 254945 | G | A | G | 0.87097 | A | 0.12903 | 6 | 0.19355 |
| 9405 | S11_36606838 | 11 | 36606838 | 60057 | G | A | G | 0.95161 | A | 0.04839 | 3 | 0.09677 |
| 9406 | S11_36606957 | 11 | 36606957 | 119 | C | G | C | 0.67742 | G | 0.32258 | 12 | 0.3871 |
| 9407 | S11_36771457 | 11 | 36771457 | 164500 | T | C | T | 0.87097 | C | 0.12903 | 8 | 0.25806 |
| 9408 | S11_36771523 | 11 | 36771523 | 66 | T | G | T | 0.6129 | G | 0.3871 | 18 | 0.58065 |
| 9409 | S11_36771574 | 11 | 36771574 | 51 | C | T | C | 0.87097 | T | 0.12903 | 8 | 0.25806 |
| 9410 | S11_38140545 | 11 | 38140545 | 1368971 | G | C | G | 0.85484 | C | 0.14516 | 5 | 0.16129 |
| 9411 | S11_38140590 | 11 | 38140590 | 45 | G | T | G | 0.91935 | T | 0.08065 | 3 | 0.09677 |
| 9412 | S11_38477040 | 11 | 38477040 | 336450 | G | A | A | 0.91935 | G | 0.08065 | 3 | 0.09677 |
| 9413 | S11_39283728 | 11 | 39283728 | 806688 | G | T | G | 0.90323 | T | 0.09677 | 6 | 0.19355 |
| 9414 | S11_39283741 | 11 | 39283741 | 13 | A | T | A | 0.59677 | T | 0.40323 | 11 | 0.35484 |
| 9415 | S11_39718189 | 11 | 39718189 | 434448 | C | T | C | 0.91935 | T | 0.08065 | 5 | 0.16129 |
| 9416 | S11_39718266 | 11 | 39718266 | 77 | C | A | C | 0.95161 | A | 0.04839 | 3 | 0.09677 |
| 9417 | S11_40694481 | 11 | 40694481 | 976215 | C | T | C | 0.95161 | T | 0.04839 | 3 | 0.09677 |
| 9418 | S11_41118402 | 11 | 41118402 | 423921 | C | T | C | 0.82258 | T | 0.17742 | 9 | 0.29032 |
| 9419 | S11_41118416 | 11 | 41118416 | 14 | C | T | C | 0.74194 | T | 0.25806 | 8 | 0.25806 |
| 9420 | S11_41234890 | 11 | 41234890 | 116474 | G | A | G | 0.93548 | A | 0.06452 | 4 | 0.12903 |
| 9421 | S11_41323411 | 11 | 41323411 | 88521 | T | C | T | 0.93548 | C | 0.06452 | 4 | 0.12903 |
| 9422 | S11_41323550 | 11 | 41323550 | 139 | A | T | A | 0.95161 | T | 0.04839 | 1 | 0.03226 |
| 9423 | S11_41323642 | 11 | 41323642 | 92 | C | T | C | 0.91935 | T | 0.08065 | 3 | 0.09677 |
| 9424 | S11_42001294 | 11 | 42001294 | 677652 | G | A | G | 0.95161 | A | 0.04839 | 3 | 0.09677 |
| 9425 | S11_42009498 | 11 | 42009498 | 8204 | G | T | G | 0.67742 | T | 0.32258 | 14 | 0.45161 |
| 9426 | S11_42009565 | 11 | 42009565 | 67 | T | C | T | 0.95161 | C | 0.04839 | 3 | 0.09677 |
| 9427 | S11_42554673 | 11 | 42554673 | 545108 | T | C | T | 0.77419 | C | 0.22581 | 8 | 0.25806 |
| 9428 | S11_42554804 | 11 | 42554804 | 131 | G | C | G | 0.8871 | C | 0.1129 | 7 | 0.22581 |
| 9429 | S11_42583293 | 11 | 42583293 | 28489 | T | C | T | 0.95161 | C | 0.04839 | 3 | 0.09677 |
| 9430 | S11_42583350 | 11 | 42583350 | 57 | A | T | A | 0.64516 | T | 0.35484 | 12 | 0.3871 |
| 9431 | S11_42583409 | 11 | 42583409 | 59 | G | C | G | 0.91935 | C | 0.08065 | 3 | 0.09677 |
| 9432 | S11_42857011 | 11 | 42857011 | 273602 | A | G | A | 0.77419 | G | 0.22581 | 10 | 0.32258 |
| 9433 | S11_42857137 | 11 | 42857137 | 126 | C | T | T | 0.70968 | C | 0.29032 | 12 | 0.3871 |
| 9434 | S11_43015092 | 11 | 43015092 | 157955 | C | T | T | 0.58065 | C | 0.41935 | 14 | 0.45161 |
| 9435 | S11_43659179 | 11 | 43659179 | 644087 | G | A | G | 0.95161 | A | 0.04839 | 3 | 0.09677 |
| 9436 | S11_43659340 | 11 | 43659340 | 161 | G | A | A | 0.82258 | G | 0.17742 | 11 | 0.35484 |
| 9437 | S11_43659350 | 11 | 43659350 | 10 | C | T | C | 0.8871 | T | 0.1129 | 7 | 0.22581 |
| 9438 | S11_44001975 | 11 | 44001975 | 342625 | G | C | G | 0.6129 | C | 0.3871 | 14 | 0.45161 |
| 9439 | S11_44002104 | 11 | 44002104 | 129 | A | G | A | 0.6129 | G | 0.3871 | 14 | 0.45161 |
| 9440 | S11_44223773 | 11 | 44223773 | 221669 | A | C | A | 0.64516 | C | 0.35484 | 12 | 0.3871 |
| 9441 | S11_44223897 | 11 | 44223897 | 124 | C | T | C | 0.83871 | T | 0.16129 | 8 | 0.25806 |
| 9442 | S11_44225484 | 11 | 44225484 | 1587 | T | C | C | 0.5 | T | 0.5 | 13 | 0.41935 |
| 9443 | S11_44361290 | 11 | 44361290 | 135806 | T | C | C | 0.74194 | T | 0.25806 | 12 | 0.3871 |
| 9444 | S11_44466194 | 11 | 44466194 | 104904 | G | A | G | 0.69355 | A | 0.30645 | 9 | 0.29032 |
| 9445 | S11_44487274 | 11 | 44487274 | 21080 | A | G | A | 0.80645 | G | 0.19355 | 8 | 0.25806 |
| 9446 | S11_44487306 | 11 | 44487306 | 32 | A | G | G | 0.93548 | A | 0.06452 | 2 | 0.06452 |
| 9447 | S11_44487461 | 11 | 44487461 | 155 | T | A | T | 0.67742 | A | 0.32258 | 12 | 0.3871 |
| 9448 | S11_44487476 | 11 | 44487476 | 15 | G | A | G | 0.66129 | A | 0.33871 | 13 | 0.41935 |

| Site number | SNP name | Chromosome | Physical position | Physical distance from the previous SNP | Reference allele | Alternative allele | Major allele | Major allele frequency | Minor allele | Minor allele frequency | Number heterozygous | Heterozygosity |
|---|---|---|---|---|---|---|---|---|---|---|---|---|
| 9449 | S11_44650743 | 11 | 44650743 | 163267 | C | T | C | 0.70968 | T | 0.29032 | 10 | 0.32258 |
| 9450 | S11_44650754 | 11 | 44650754 | 11 | G | A | G | 0.79032 | A | 0.20968 | 13 | 0.41935 |
| 9451 | S11_44650851 | 11 | 44650851 | 97 | C | G | G | 0.54839 | C | 0.45161 | 12 | 0.3871 |
| 9452 | S11_44862896 | 11 | 44862896 | 212045 | T | G | G | 0.95161 | T | 0.04839 | 3 | 0.09677 |
| 9453 | S11_45025583 | 11 | 45025583 | 162687 | T | C | C | 0.70968 | T | 0.29032 | 12 | 0.3871 |
| 9454 | S11_45025602 | 11 | 45025602 | 19 | T | C | T | 0.74194 | C | 0.25806 | 12 | 0.3871 |
| 9455 | S11_45025651 | 11 | 45025651 | 49 | A | G | A | 0.56452 | G | 0.43548 | 15 | 0.48387 |
| 9456 | S11_45135571 | 11 | 45135571 | 109920 | C | T | C | 0.95161 | T | 0.04839 | 1 | 0.03226 |
| 9457 | S11_45188004 | 11 | 45188004 | 52433 | T | C | T | 0.91935 | C | 0.08065 | 3 | 0.09677 |
| 9458 | S11_45295414 | 11 | 45295414 | 107410 | G | T | G | 0.80645 | T | 0.19355 | 8 | 0.25806 |
| 9459 | S11_45295572 | 11 | 45295572 | 158 | C | T | C | 0.8871 | T | 0.1129 | 7 | 0.22581 |
| 9460 | S11_45677174 | 11 | 45677174 | 381602 | C | T | C | 0.77419 | T | 0.22581 | 8 | 0.25806 |
| 9461 | S11_45677293 | 11 | 45677293 | 119 | T | C | T | 0.93548 | C | 0.06452 | 4 | 0.12903 |
| 9462 | S11_45677356 | 11 | 45677356 | 63 | C | G | C | 0.93548 | G | 0.06452 | 4 | 0.12903 |
| 9463 | S11_45688115 | 11 | 45688115 | 10759 | A | C | A | 0.74194 | C | 0.25806 | 12 | 0.3871 |
| 9464 | S11_45688143 | 11 | 45688143 | 28 | T | C | T | 0.74194 | C | 0.25806 | 12 | 0.3871 |
| 9465 | S11_45688163 | 11 | 45688163 | 20 | A | G | A | 0.74194 | G | 0.25806 | 12 | 0.3871 |
| 9466 | S11_45688180 | 11 | 45688180 | 17 | A | G | A | 0.74194 | G | 0.25806 | 12 | 0.3871 |
| 9467 | S11_45688266 | 11 | 45688266 | 86 | T | C | C | 0.74194 | T | 0.25806 | 12 | 0.3871 |
| 9468 | S11_45688276 | 11 | 45688276 | 10 | A | G | A | 0.74194 | G | 0.25806 | 12 | 0.3871 |
| 9469 | S11_45688307 | 11 | 45688307 | 31 | A | T | T | 0.74194 | A | 0.25806 | 12 | 0.3871 |
| 9470 | S11_45688315 | 11 | 45688315 | 8 | G | T | G | 0.74194 | T | 0.25806 | 12 | 0.3871 |
| 9471 | S11_45688355 | 11 | 45688355 | 40 | C | T | C | 0.74194 | T | 0.25806 | 12 | 0.3871 |
| 9472 | S11_45756141 | 11 | 45756141 | 67786 | T | C | T | 0.90323 | C | 0.09677 | 6 | 0.19355 |
| 9473 | S11_45756207 | 11 | 45756207 | 66 | A | G | A | 0.93548 | G | 0.06452 | 4 | 0.12903 |
| 9474 | S11_45756238 | 11 | 45756238 | 31 | T | A | T | 0.62903 | A | 0.37097 | 15 | 0.48387 |
| 9475 | S11_45756248 | 11 | 45756248 | 10 | G | A | G | 0.90323 | A | 0.09677 | 6 | 0.19355 |
| 9476 | S11_45756280 | 11 | 45756280 | 32 | C | T | C | 0.72581 | T | 0.27419 | 13 | 0.41935 |
| 9477 | S11_45756324 | 11 | 45756324 | 44 | G | C | G | 0.74194 | C | 0.25806 | 12 | 0.3871 |
| 9478 | S11_45756333 | 11 | 45756333 | 9 | G | A | G | 0.90323 | A | 0.09677 | 6 | 0.19355 |
| 9479 | S11_45866643 | 11 | 45866643 | 110310 | G | A | G | 0.80645 | A | 0.19355 | 8 | 0.25806 |
| 9480 | S11_45972257 | 11 | 45972257 | 105614 | C | T | T | 0.56452 | C | 0.43548 | 11 | 0.35484 |
| 9481 | S11_45972316 | 11 | 45972316 | 59 | C | A | A | 0.56452 | C | 0.43548 | 11 | 0.35484 |
| 9482 | S11_45972352 | 11 | 45972352 | 36 | G | T | G | 0.54839 | T | 0.45161 | 12 | 0.3871 |
| 9483 | S11_45995265 | 11 | 45995265 | 22913 | A | G | A | 0.59677 | G | 0.40323 | 19 | 0.6129 |
| 9484 | S11_45995272 | 11 | 45995272 | 7 | G | C | G | 0.93548 | C | 0.06452 | 4 | 0.12903 |
| 9485 | S11_46333979 | 11 | 46333979 | 338707 | A | C | A | 0.90323 | C | 0.09677 | 6 | 0.19355 |
| 9486 | S11_46333982 | 11 | 46333982 | 3 | T | C | C | 0.85484 | T | 0.14516 | 9 | 0.29032 |
| 9487 | S11_46334067 | 11 | 46334067 | 85 | G | A | G | 0.8871 | A | 0.1129 | 7 | 0.22581 |
| 9488 | S11_46334129 | 11 | 46334129 | 62 | G | A | G | 0.87097 | A | 0.12903 | 8 | 0.25806 |
| 9489 | S11_46334200 | 11 | 46334200 | 71 | T | A | T | 0.8871 | A | 0.1129 | 7 | 0.22581 |
| 9490 | S11_46399412 | 11 | 46399412 | 65212 | A | G | A | 0.67742 | G | 0.32258 | 12 | 0.3871 |
| 9491 | S11_46682888 | 11 | 46682888 | 283476 | C | T | T | 0.79032 | C | 0.20968 | 9 | 0.29032 |
| 9492 | S11_46858324 | 11 | 46858324 | 175436 | T | A | T | 0.72581 | A | 0.27419 | 13 | 0.41935 |
| 9493 | S11_46858431 | 11 | 46858431 | 107 | A | G | G | 0.95161 | A | 0.04839 | 3 | 0.09677 |
| 9494 | S11_47191886 | 11 | 47191886 | 333455 | G | C | G | 0.93548 | C | 0.06452 | 4 | 0.12903 |
| 9495 | S11_47379851 | 11 | 47379851 | 187965 | G | A | G | 0.93548 | A | 0.06452 | 4 | 0.12903 |
| 9496 | S11_47379931 | 11 | 47379931 | 80 | G | A | G | 0.91935 | A | 0.08065 | 5 | 0.16129 |
| 9497 | S11_47933500 | 11 | 47933500 | 553569 | C | T | C | 0.83871 | T | 0.16129 | 10 | 0.32258 |
| 9498 | S11_48196855 | 11 | 48196855 | 263355 | A | G | A | 0.87097 | G | 0.12903 | 8 | 0.25806 |
| 9499 | S11_48248891 | 11 | 48248891 | 52036 | T | C | C | 0.77419 | T | 0.22581 | 10 | 0.32258 |
| 9500 | S11_48260419 | 11 | 48260419 | 11528 | C | T | C | 0.8871 | T | 0.1129 | 7 | 0.22581 |
| 9501 | S11_48260462 | 11 | 48260462 | 43 | A | G | A | 0.75806 | G | 0.24194 | 13 | 0.41935 |
| 9502 | S11_48361898 | 11 | 48361898 | 101436 | C | T | C | 0.83871 | T | 0.16129 | 6 | 0.19355 |

| Site number | SNP name | Chromosome | Physical position | Physical distance from the previous SNP | Reference allele | Alternative allele | Major allele | Major allele frequency | Minor allele | Minor allele frequency | Number heterozygous | Heterozygosity |
|---|---|---|---|---|---|---|---|---|---|---|---|---|
| 9503 | S11_48421827 | 11 | 48421827 | 59929 | A | G | G | 0.93548 | A | 0.06452 | 2 | 0.06452 |
| 9504 | S11_48485762 | 11 | 48485762 | 63935 | A | G | G | 0.85484 | A | 0.14516 | 7 | 0.22581 |
| 9505 | S11_48485798 | 11 | 48485798 | 36 | G | T | G | 0.79032 | T | 0.20968 | 7 | 0.22581 |
| 9506 | S11_48490890 | 11 | 48490890 | 5092 | C | A | C | 0.62903 | A | 0.37097 | 9 | 0.29032 |
| 9507 | S11_48491074 | 11 | 48491074 | 184 | G | A | G | 0.8871 | A | 0.1129 | 7 | 0.22581 |
| 9508 | S11_48491129 | 11 | 48491129 | 55 | A | G | A | 0.8871 | G | 0.1129 | 7 | 0.22581 |
| 9509 | S11_48623687 | 11 | 48623687 | 132558 | C | T | C | 0.82258 | T | 0.17742 | 7 | 0.22581 |
| 9510 | S11_48708091 | 11 | 48708091 | 84404 | G | A | G | 0.95161 | A | 0.04839 | 3 | 0.09677 |
| 9511 | S11_48855472 | 11 | 48855472 | 147381 | C | T | C | 0.91935 | T | 0.08065 | 3 | 0.09677 |
| 9512 | S11_48960062 | 11 | 48960062 | 104590 | C | A | C | 0.85484 | A | 0.14516 | 9 | 0.29032 |
| 9513 | S11_49606951 | 11 | 49606951 | 646889 | G | A | G | 0.93548 | A | 0.06452 | 4 | 0.12903 |
| 9514 | S11_49936690 | 11 | 49936690 | 329739 | G | A | A | 0.51613 | G | 0.48387 | 16 | 0.51613 |
| 9515 | S11_49936726 | 11 | 49936726 | 36 | T | C | C | 0.51613 | T | 0.48387 | 16 | 0.51613 |
| 9516 | S11_49936771 | 11 | 49936771 | 45 | T | C | T | 0.93548 | C | 0.06452 | 4 | 0.12903 |
| 9517 | S11_49952287 | 11 | 49952287 | 15516 | T | C | T | 0.87097 | C | 0.12903 | 6 | 0.19355 |
| 9518 | S11_50068113 | 11 | 50068113 | 115826 | C | T | C | 0.85484 | T | 0.14516 | 9 | 0.29032 |
| 9519 | S11_50068195 | 11 | 50068195 | 82 | C | T | C | 0.83871 | T | 0.16129 | 10 | 0.32258 |
| 9520 | S11_50068250 | 11 | 50068250 | 55 | G | A | G | 0.79032 | A | 0.20968 | 13 | 0.41935 |
| 9521 | S11_50068257 | 11 | 50068257 | 7 | G | A | G | 0.93548 | A | 0.06452 | 2 | 0.06452 |
| 9522 | S11_50068289 | 11 | 50068289 | 32 | G | C | G | 0.95161 | C | 0.04839 | 3 | 0.09677 |
| 9523 | S11_50068318 | 11 | 50068318 | 29 | G | A | G | 0.91935 | A | 0.08065 | 5 | 0.16129 |
| 9524 | S11_50160965 | 11 | 50160965 | 92647 | A | G | G | 0.62903 | A | 0.37097 | 17 | 0.54839 |
| 9525 | S11_50354691 | 11 | 50354691 | 193726 | G | A | G | 0.83871 | A | 0.16129 | 8 | 0.25806 |
| 9526 | S11_50354709 | 11 | 50354709 | 18 | C | A | C | 0.8871 | A | 0.1129 | 7 | 0.22581 |
| 9527 | S11_50354729 | 11 | 50354729 | 20 | A | G | A | 0.82258 | G | 0.17742 | 11 | 0.35484 |
| 9528 | S11_50354748 | 11 | 50354748 | 19 | G | A | G | 0.58065 | A | 0.41935 | 12 | 0.3871 |
| 9529 | S11_50354753 | 11 | 50354753 | 5 | C | A | C | 0.90323 | A | 0.09677 | 6 | 0.19355 |
| 9530 | S11_50420657 | 11 | 50420657 | 65904 | G | T | G | 0.53226 | T | 0.46774 | 19 | 0.6129 |
| 9531 | S11_51080906 | 11 | 51080906 | 660249 | A | T | A | 0.6129 | T | 0.3871 | 16 | 0.51613 |
| 9532 | S11_51081018 | 11 | 51081018 | 112 | G | T | G | 0.87097 | T | 0.12903 | 4 | 0.12903 |
| 9533 | S11_51081074 | 11 | 51081074 | 56 | T | G | G | 0.51613 | T | 0.48387 | 16 | 0.51613 |
| 9534 | S11_51081096 | 11 | 51081096 | 22 | A | G | A | 0.93548 | G | 0.06452 | 4 | 0.12903 |
| 9535 | S11_51081100 | 11 | 51081100 | 4 | C | T | C | 0.95161 | T | 0.04839 | 3 | 0.09677 |
| 9536 | S11_51436500 | 11 | 51436500 | 355400 | C | G | G | 0.93548 | C | 0.06452 | 4 | 0.12903 |
| 9537 | S11_51436570 | 11 | 51436570 | 70 | G | A | G | 0.87097 | A | 0.12903 | 8 | 0.25806 |
| 9538 | S11_51682287 | 11 | 51682287 | 245717 | A | G | G | 0.75806 | A | 0.24194 | 13 | 0.41935 |
| 9539 | S11_51682325 | 11 | 51682325 | 38 | C | T | T | 0.59677 | C | 0.40323 | 17 | 0.54839 |
| 9540 | S11_51682355 | 11 | 51682355 | 30 | C | T | T | 0.59677 | C | 0.40323 | 17 | 0.54839 |
| 9541 | S11_51858440 | 11 | 51858440 | 176085 | T | C | C | 0.83871 | T | 0.16129 | 8 | 0.25806 |
| 9542 | S11_51858553 | 11 | 51858553 | 113 | T | C | C | 0.83871 | T | 0.16129 | 8 | 0.25806 |
| 9543 | S11_51858593 | 11 | 51858593 | 40 | A | G | G | 0.80645 | A | 0.19355 | 8 | 0.25806 |
| 9544 | S11_52574130 | 11 | 52574130 | 715537 | T | C | C | 0.58065 | T | 0.41935 | 14 | 0.45161 |
| 9545 | S11_52574151 | 11 | 52574151 | 21 | A | G | A | 0.80645 | G | 0.19355 | 8 | 0.25806 |
| 9546 | S11_52574363 | 11 | 52574363 | 212 | G | A | G | 0.67742 | A | 0.32258 | 12 | 0.3871 |
| 9547 | S11_53170425 | 11 | 53170425 | 596062 | T | A | T | 0.59677 | A | 0.40323 | 15 | 0.48387 |
| 9548 | S11_53170426 | 11 | 53170426 | 1 | G | C | G | 0.59677 | C | 0.40323 | 15 | 0.48387 |
| 9549 | S11_54154747 | 11 | 54154747 | 984321 | C | T | C | 0.90323 | T | 0.09677 | 6 | 0.19355 |
| 9550 | S11_54154834 | 11 | 54154834 | 87 | T | C | T | 0.72581 | C | 0.27419 | 13 | 0.41935 |
| 9551 | S11_54154877 | 11 | 54154877 | 43 | A | C | A | 0.85484 | C | 0.14516 | 9 | 0.29032 |
| 9552 | S11_54154908 | 11 | 54154908 | 31 | C | A | C | 0.59677 | A | 0.40323 | 13 | 0.41935 |
| 9553 | S11_54651081 | 11 | 54651081 | 496173 | T | C | T | 0.85484 | C | 0.14516 | 9 | 0.29032 |
| 9554 | S11_54838999 | 11 | 54838999 | 187918 | T | A | T | 0.75806 | A | 0.24194 | 11 | 0.35484 |
| 9555 | S11_54839059 | 11 | 54839059 | 60 | G | A | G | 0.93548 | A | 0.06452 | 4 | 0.12903 |
| 9556 | S11_54839114 | 11 | 54839114 | 55 | T | C | T | 0.70968 | C | 0.29032 | 12 | 0.3871 |

| Site number | SNP name | Chromosome | Physical position | Physical distance from the previous SNP | Reference allele | Alternative allele | Major allele | Major allele frequency | Minor allele | Minor allele frequency | Number heterozygous | Heterozygosity |
|---|---|---|---|---|---|---|---|---|---|---|---|---|
| 9557 | S11_55456572 | 11 | 55456572 | 617458 | G | A | G | 0.95161 | A | 0.04839 | 3 | 0.09677 |
| 9558 | S11_55769187 | 11 | 55769187 | 312615 | C | A | C | 0.72581 | A | 0.27419 | 11 | 0.35484 |
| 9559 | S11_55769292 | 11 | 55769292 | 105 | T | C | T | 0.82258 | C | 0.17742 | 9 | 0.29032 |
| 9560 | S11_55891022 | 11 | 55891022 | 121730 | G | A | G | 0.85484 | A | 0.14516 | 7 | 0.22581 |
| 9561 | S11_55891067 | 11 | 55891067 | 45 | C | A | C | 0.85484 | A | 0.14516 | 7 | 0.22581 |
| 9562 | S11_55891185 | 11 | 55891185 | 118 | A | G | G | 0.93548 | A | 0.06452 | 4 | 0.12903 |
| 9563 | S11_55907428 | 11 | 55907428 | 16243 | G | A | G | 0.74194 | A | 0.25806 | 12 | 0.3871 |
| 9564 | S11_55907571 | 11 | 55907571 | 143 | C | T | C | 0.85484 | T | 0.14516 | 9 | 0.29032 |
| 9565 | S11_55907583 | 11 | 55907583 | 12 | G | T | G | 0.90323 | T | 0.09677 | 6 | 0.19355 |
| 9566 | S11_55907645 | 11 | 55907645 | 62 | G | A | G | 0.79032 | A | 0.20968 | 11 | 0.35484 |
| 9567 | S11_56127765 | 11 | 56127765 | 220120 | A | G | A | 0.87097 | G | 0.12903 | 8 | 0.25806 |
| 9568 | S11_56159888 | 11 | 56159888 | 32123 | A | G | A | 0.8871 | G | 0.1129 | 5 | 0.16129 |
| 9569 | S11_56159953 | 11 | 56159953 | 65 | A | G | A | 0.8871 | G | 0.1129 | 5 | 0.16129 |
| 9570 | S11_56160032 | 11 | 56160032 | 79 | T | C | T | 0.8871 | C | 0.1129 | 5 | 0.16129 |
| 9571 | S11_56235164 | 11 | 56235164 | 75132 | C | T | C | 0.82258 | T | 0.17742 | 9 | 0.29032 |
| 9572 | S11_56235264 | 11 | 56235264 | 100 | G | T | G | 0.51613 | T | 0.48387 | 18 | 0.58065 |
| 9573 | S11_56235366 | 11 | 56235366 | 102 | G | A | G | 0.51613 | A | 0.48387 | 18 | 0.58065 |
| 9574 | S11_56235374 | 11 | 56235374 | 8 | A | G | G | 0.82258 | A | 0.17742 | 9 | 0.29032 |
| 9575 | S11_56627595 | 11 | 56627595 | 392221 | T | C | T | 0.95161 | C | 0.04839 | 3 | 0.09677 |
| 9576 | S11_56627657 | 11 | 56627657 | 62 | C | T | C | 0.95161 | T | 0.04839 | 3 | 0.09677 |
| 9577 | S11_56966022 | 11 | 56966022 | 338365 | G | A | A | 0.56452 | G | 0.43548 | 17 | 0.54839 |
| 9578 | S11_58372628 | 11 | 58372628 | 1406606 | G | A | G | 0.87097 | A | 0.12903 | 6 | 0.19355 |
| 9579 | S11_58372717 | 11 | 58372717 | 89 | G | A | G | 0.91935 | A | 0.08065 | 5 | 0.16129 |
| 9580 | S11_58372758 | 11 | 58372758 | 41 | G | A | G | 0.85484 | A | 0.14516 | 7 | 0.22581 |
| 9581 | S11_59058282 | 11 | 59058282 | 685524 | T | C | T | 0.87097 | C | 0.12903 | 6 | 0.19355 |
| 9582 | S11_59531089 | 11 | 59531089 | 472807 | G | C | C | 0.79032 | G | 0.20968 | 9 | 0.29032 |
| 9583 | S11_59774767 | 11 | 59774767 | 243678 | T | C | T | 0.77419 | C | 0.22581 | 12 | 0.3871 |
| 9584 | S11_59774768 | 11 | 59774768 | 1 | C | T | C | 0.77419 | T | 0.22581 | 12 | 0.3871 |
| 9585 | S11_59774791 | 11 | 59774791 | 23 | T | C | C | 0.90323 | T | 0.09677 | 4 | 0.12903 |
| 9586 | S11_60000154 | 11 | 60000154 | 225363 | G | C | G | 0.74194 | C | 0.25806 | 14 | 0.45161 |
| 9587 | S11_60000231 | 11 | 60000231 | 77 | C | T | C | 0.74194 | T | 0.25806 | 14 | 0.45161 |
| 9588 | S11_60311478 | 11 | 60311478 | 311247 | G | A | A | 0.93548 | G | 0.06452 | 4 | 0.12903 |
| 9589 | S11_60422829 | 11 | 60422829 | 111351 | G | A | G | 0.93548 | A | 0.06452 | 4 | 0.12903 |
| 9590 | S11_60422879 | 11 | 60422879 | 50 | G | C | G | 0.93548 | C | 0.06452 | 4 | 0.12903 |
| 9591 | S11_60423007 | 11 | 60423007 | 128 | A | G | A | 0.91935 | G | 0.08065 | 5 | 0.16129 |
| 9592 | S11_60424570 | 11 | 60424570 | 1563 | A | G | G | 0.70968 | A | 0.29032 | 16 | 0.51613 |
| 9593 | S11_60424702 | 11 | 60424702 | 132 | G | A | G | 0.93548 | A | 0.06452 | 4 | 0.12903 |
| 9594 | S11_60800444 | 11 | 60800444 | 375742 | A | G | A | 0.51613 | G | 0.48387 | 18 | 0.58065 |
| 9595 | S11_61052335 | 11 | 61052335 | 251891 | C | T | C | 0.91935 | T | 0.08065 | 5 | 0.16129 |
| 9596 | S11_61052406 | 11 | 61052406 | 71 | G | C | C | 0.93548 | G | 0.06452 | 4 | 0.12903 |
| 9597 | S11_61641054 | 11 | 61641054 | 588648 | C | T | C | 0.93548 | T | 0.06452 | 4 | 0.12903 |
| 9598 | S11_61641253 | 11 | 61641253 | 199 | T | G | T | 0.91935 | G | 0.08065 | 5 | 0.16129 |
| 9599 | S11_62590289 | 11 | 62590289 | 949036 | A | T | A | 0.93548 | T | 0.06452 | 4 | 0.12903 |
| 9600 | S11_62653589 | 11 | 62653589 | 63300 | A | G | A | 0.70968 | G | 0.29032 | 10 | 0.32258 |
| 9601 | S11_62815750 | 11 | 62815750 | 162161 | G | A | G | 0.95161 | A | 0.04839 | 3 | 0.09677 |
| 9602 | S11_62844626 | 11 | 62844626 | 28876 | C | T | C | 0.95161 | T | 0.04839 | 3 | 0.09677 |
| 9603 | S11_62844735 | 11 | 62844735 | 109 | G | A | G | 0.95161 | A | 0.04839 | 3 | 0.09677 |
| 9604 | S11_63031384 | 11 | 63031384 | 186649 | C | T | C | 0.93548 | T | 0.06452 | 2 | 0.06452 |
| 9605 | S11_63344662 | 11 | 63344662 | 313278 | T | A | T | 0.87097 | A | 0.12903 | 8 | 0.25806 |
| 9606 | S11_63536787 | 11 | 63536787 | 192125 | A | G | G | 0.77419 | A | 0.22581 | 12 | 0.3871 |
| 9607 | S11_64289313 | 11 | 64289313 | 752526 | G | T | T | 0.66129 | G | 0.33871 | 17 | 0.54839 |
| 9608 | S11_64390335 | 11 | 64390335 | 101022 | G | A | G | 0.66129 | A | 0.33871 | 19 | 0.6129 |
| 9609 | S11_64390449 | 11 | 64390449 | 114 | G | A | G | 0.72581 | A | 0.27419 | 15 | 0.48387 |
| 9610 | S11_64530816 | 11 | 64530816 | 140367 | A | G | A | 0.74194 | G | 0.25806 | 12 | 0.3871 |

| Site number | SNP name | Chromosome | Physical position | Physical distance from the previous SNP | Reference allele | Alternative allele | Major allele | Major allele frequency | Minor allele | Minor allele frequency | Number heterozygous | Heterozygosity |
|---|---|---|---|---|---|---|---|---|---|---|---|---|
| 9611 | S11_64743220 | 11 | 64743220 | 212404 | A | G | A | 0.90323 | G | 0.09677 | 6 | 0.19355 |
| 9612 | S11_64841272 | 11 | 64841272 | 98052 | C | T | C | 0.66129 | T | 0.33871 | 17 | 0.54839 |
| 9613 | S11_64853490 | 11 | 64853490 | 12218 | T | C | C | 0.51613 | T | 0.48387 | 18 | 0.58065 |
| 9614 | S11_65125165 | 11 | 65125165 | 271675 | A | G | A | 0.95161 | G | 0.04839 | 3 | 0.09677 |
| 9615 | S11_65125180 | 11 | 65125180 | 15 | T | C | T | 0.69355 | C | 0.30645 | 11 | 0.35484 |
| 9616 | S11_65384386 | 11 | 65384386 | 259206 | G | A | G | 0.87097 | A | 0.12903 | 8 | 0.25806 |
| 9617 | S11_65517141 | 11 | 65517141 | 132755 | C | A | C | 0.93548 | A | 0.06452 | 4 | 0.12903 |
| 9618 | S11_65517205 | 11 | 65517205 | 64 | G | A | G | 0.91935 | A | 0.08065 | 3 | 0.09677 |
| 9619 | S11_65546961 | 11 | 65546961 | 29756 | G | A | G | 0.72581 | A | 0.27419 | 13 | 0.41935 |
| 9620 | S11_65546978 | 11 | 65546978 | 17 | A | G | A | 0.91935 | G | 0.08065 | 3 | 0.09677 |
| 9621 | S11_66001365 | 11 | 66001365 | 454387 | G | A | G | 0.59677 | A | 0.40323 | 17 | 0.54839 |
| 9622 | S11_66126567 | 11 | 66126567 | 125202 | T | C | T | 0.74194 | C | 0.25806 | 16 | 0.51613 |
| 9623 | S11_66126712 | 11 | 66126712 | 145 | C | T | C | 0.67742 | T | 0.32258 | 16 | 0.51613 |
| 9624 | S11_66126737 | 11 | 66126737 | 25 | C | T | C | 0.67742 | T | 0.32258 | 16 | 0.51613 |
| 9625 | S11_66130664 | 11 | 66130664 | 3927 | T | C | T | 0.62903 | C | 0.37097 | 17 | 0.54839 |
| 9626 | S11_66130749 | 11 | 66130749 | 85 | G | A | A | 0.93548 | G | 0.06452 | 4 | 0.12903 |
| 9627 | S11_66130784 | 11 | 66130784 | 35 | C | T | C | 0.74194 | T | 0.25806 | 16 | 0.51613 |
| 9628 | S11_66698054 | 11 | 66698054 | 567270 | G | A | G | 0.8871 | A | 0.1129 | 5 | 0.16129 |
| 9629 | S11_66698063 | 11 | 66698063 | 9 | C | G | C | 0.93548 | G | 0.06452 | 4 | 0.12903 |
| 9630 | S11_66698232 | 11 | 66698232 | 169 | A | G | A | 0.83871 | G | 0.16129 | 8 | 0.25806 |
| 9631 | S11_67266053 | 11 | 67266053 | 567821 | G | A | G | 0.83871 | A | 0.16129 | 8 | 0.25806 |
| 9632 | S11_67266201 | 11 | 67266201 | 148 | T | C | T | 0.83871 | C | 0.16129 | 8 | 0.25806 |
| 9633 | S11_67266205 | 11 | 67266205 | 4 | G | A | G | 0.83871 | A | 0.16129 | 8 | 0.25806 |
| 9634 | S11_68420532 | 11 | 68420532 | 1154327 | T | C | T | 0.53226 | C | 0.46774 | 19 | 0.6129 |
| 9635 | S11_68463027 | 11 | 68463027 | 42495 | T | G | T | 0.90323 | G | 0.09677 | 6 | 0.19355 |
| 9636 | S11_68496906 | 11 | 68496906 | 33879 | T | C | C | 0.91935 | T | 0.08065 | 5 | 0.16129 |
| 9637 | S11_68497004 | 11 | 68497004 | 98 | G | A | G | 0.90323 | A | 0.09677 | 6 | 0.19355 |
| 9638 | S11_68547128 | 11 | 68547128 | 50124 | A | G | A | 0.87097 | G | 0.12903 | 6 | 0.19355 |
| 9639 | S11_68623347 | 11 | 68623347 | 76219 | C | T | T | 0.77419 | C | 0.22581 | 12 | 0.3871 |
| 9640 | S11_68623472 | 11 | 68623472 | 125 | A | G | A | 0.53226 | G | 0.46774 | 13 | 0.41935 |
| 9641 | S11_68623508 | 11 | 68623508 | 36 | C | T | C | 0.90323 | T | 0.09677 | 6 | 0.19355 |
| 9642 | S11_68906499 | 11 | 68906499 | 282991 | C | T | C | 0.64516 | T | 0.35484 | 14 | 0.45161 |
| 9643 | S11_68955198 | 11 | 68955198 | 48699 | C | T | C | 0.85484 | T | 0.14516 | 9 | 0.29032 |
| 9644 | S11_68955218 | 11 | 68955218 | 20 | G | A | G | 0.82258 | A | 0.17742 | 11 | 0.35484 |
| 9645 | S11_68955243 | 11 | 68955243 | 25 | G | T | G | 0.74194 | T | 0.25806 | 16 | 0.51613 |
| 9646 | S11_68955340 | 11 | 68955340 | 97 | C | T | C | 0.8871 | T | 0.1129 | 7 | 0.22581 |
| 9647 | S11_68973989 | 11 | 68973989 | 18649 | G | A | G | 0.8871 | A | 0.1129 | 7 | 0.22581 |
| 9648 | S11_69414976 | 11 | 69414976 | 440987 | G | C | G | 0.93548 | C | 0.06452 | 4 | 0.12903 |
| 9649 | S11_69414987 | 11 | 69414987 | 11 | G | C | C | 0.82258 | G | 0.17742 | 11 | 0.35484 |
| 9650 | S11_69465956 | 11 | 69465956 | 50969 | G | A | G | 0.87097 | A | 0.12903 | 8 | 0.25806 |
| 9651 | S11_69465967 | 11 | 69465967 | 11 | C | T | T | 0.59677 | C | 0.40323 | 19 | 0.6129 |
| 9652 | S11_69466009 | 11 | 69466009 | 42 | A | G | A | 0.85484 | G | 0.14516 | 7 | 0.22581 |
| 9653 | S11_69466018 | 11 | 69466018 | 9 | C | T | C | 0.87097 | T | 0.12903 | 8 | 0.25806 |
| 9654 | S11_69466041 | 11 | 69466041 | 23 | C | A | C | 0.58065 | A | 0.41935 | 14 | 0.45161 |
| 9655 | S11_69600512 | 11 | 69600512 | 134471 | C | A | A | 0.77419 | C | 0.22581 | 14 | 0.45161 |
| 9656 | S11_69748368 | 11 | 69748368 | 147856 | C | T | T | 0.67742 | C | 0.32258 | 14 | 0.45161 |
| 9657 | S11_69748428 | 11 | 69748428 | 60 | T | C | T | 0.69355 | C | 0.30645 | 13 | 0.41935 |
| 9658 | S11_69748538 | 11 | 69748538 | 110 | T | C | C | 0.66129 | T | 0.33871 | 15 | 0.48387 |
| 9659 | S11_69748579 | 11 | 69748579 | 41 | G | A | G | 0.95161 | A | 0.04839 | 3 | 0.09677 |
| 9660 | S11_69868807 | 11 | 69868807 | 120228 | A | G | A | 0.66129 | G | 0.33871 | 17 | 0.54839 |
| 9661 | S11_70180183 | 11 | 70180183 | 311376 | A | G | A | 0.91935 | G | 0.08065 | 5 | 0.16129 |
| 9662 | S11_70180275 | 11 | 70180275 | 92 | A | G | G | 0.53226 | A | 0.46774 | 11 | 0.35484 |
| 9663 | S11_70891697 | 11 | 70891697 | 711422 | C | A | C | 0.90323 | A | 0.09677 | 6 | 0.19355 |
| 9664 | S11_70935508 | 11 | 70935508 | 43811 | T | C | T | 0.93548 | C | 0.06452 | 4 | 0.12903 |

| Site number | SNP name | Chromosome | Physical position | Physical distance from the previous SNP | Reference allele | Alternative allele | Major allele | Major allele frequency | Minor allele | Minor allele frequency | Number heterozygous | Heterozygosity |
|---|---|---|---|---|---|---|---|---|---|---|---|---|
| 9665 | S11_71621333 | 11 | 71621333 | 685825 | T | C | C | 0.69355 | T | 0.30645 | 17 | 0.54839 |
| 9666 | S11_72244386 | 11 | 72244386 | 623053 | G | A | G | 0.75806 | A | 0.24194 | 11 | 0.35484 |
| 9667 | S11_72244409 | 11 | 72244409 | 23 | T | C | T | 0.75806 | C | 0.24194 | 11 | 0.35484 |
| 9668 | S11_72605707 | 11 | 72605707 | 361298 | A | G | A | 0.91935 | G | 0.08065 | 5 | 0.16129 |
| 9669 | S11_72605772 | 11 | 72605772 | 65 | G | A | G | 0.74194 | A | 0.25806 | 14 | 0.45161 |
| 9670 | S11_72605871 | 11 | 72605871 | 99 | A | G | A | 0.95161 | G | 0.04839 | 3 | 0.09677 |
| 9671 | S11_72605909 | 11 | 72605909 | 38 | T | C | C | 0.91935 | T | 0.08065 | 5 | 0.16129 |
| 9672 | S11_73152316 | 11 | 73152316 | 546407 | A | G | A | 0.91935 | G | 0.08065 | 5 | 0.16129 |
| 9673 | S11_73251074 | 11 | 73251074 | 98758 | G | C | G | 0.95161 | C | 0.04839 | 3 | 0.09677 |
| 9674 | S11_73251084 | 11 | 73251084 | 10 | G | C | G | 0.95161 | C | 0.04839 | 3 | 0.09677 |
| 9675 | S11_73431192 | 11 | 73431192 | 180108 | T | C | T | 0.93548 | C | 0.06452 | 2 | 0.06452 |
| 9676 | S11_73431195 | 11 | 73431195 | 3 | G | A | G | 0.93548 | A | 0.06452 | 4 | 0.12903 |
| 9677 | S11_73546109 | 11 | 73546109 | 114914 | C | T | C | 0.93548 | T | 0.06452 | 2 | 0.06452 |
| 9678 | S11_73592776 | 11 | 73592776 | 46667 | C | T | C | 0.91935 | T | 0.08065 | 5 | 0.16129 |
| 9679 | S11_73592983 | 11 | 73592983 | 207 | T | A | T | 0.67742 | A | 0.32258 | 14 | 0.45161 |
| 9680 | S11_73717035 | 11 | 73717035 | 124052 | C | T | C | 0.93548 | T | 0.06452 | 4 | 0.12903 |
| 9681 | S11_73851827 | 11 | 73851827 | 134792 | G | A | G | 0.8871 | A | 0.1129 | 7 | 0.22581 |
| 9682 | S11_73851876 | 11 | 73851876 | 49 | C | T | C | 0.91935 | T | 0.08065 | 5 | 0.16129 |
| 9683 | S11_74214914 | 11 | 74214914 | 363038 | T | C | T | 0.90323 | C | 0.09677 | 6 | 0.19355 |
| 9684 | S11_74214938 | 11 | 74214938 | 24 | G | A | G | 0.90323 | A | 0.09677 | 6 | 0.19355 |
| 9685 | S11_74214964 | 11 | 74214964 | 26 | G | T | T | 0.56452 | G | 0.43548 | 11 | 0.35484 |
| 9686 | S11_75171674 | 11 | 75171674 | 956710 | C | T | C | 0.95161 | T | 0.04839 | 3 | 0.09677 |
| 9687 | S11_75171700 | 11 | 75171700 | 26 | G | A | G | 0.53226 | A | 0.46774 | 13 | 0.41935 |
| 9688 | S11_75171709 | 11 | 75171709 | 9 | G | A | G | 0.53226 | A | 0.46774 | 13 | 0.41935 |
| 9689 | S11_75171733 | 11 | 75171733 | 24 | G | A | A | 0.6129 | G | 0.3871 | 10 | 0.32258 |
| 9690 | S11_75555290 | 11 | 75555290 | 383557 | A | G | A | 0.91935 | G | 0.08065 | 5 | 0.16129 |
| 9691 | S11_75653785 | 11 | 75653785 | 98495 | G | A | A | 0.56452 | G | 0.43548 | 15 | 0.48387 |
| 9692 | S11_75653888 | 11 | 75653888 | 103 | C | T | C | 0.93548 | T | 0.06452 | 4 | 0.12903 |
| 9693 | S11_75653889 | 11 | 75653889 | 1 | G | A | G | 0.64516 | A | 0.35484 | 18 | 0.58065 |
| 9694 | S11_75653937 | 11 | 75653937 | 48 | G | T | G | 0.8871 | T | 0.1129 | 5 | 0.16129 |
| 9695 | S11_75957777 | 11 | 75957777 | 303840 | A | T | T | 0.91935 | A | 0.08065 | 5 | 0.16129 |
| 9696 | S11_75985957 | 11 | 75985957 | 28180 | C | T | T | 0.82258 | C | 0.17742 | 9 | 0.29032 |
| 9697 | S11_75986158 | 11 | 75986158 | 201 | G | T | T | 0.82258 | G | 0.17742 | 9 | 0.29032 |
| 9698 | S11_76187506 | 11 | 76187506 | 201348 | A | G | G | 0.79032 | A | 0.20968 | 11 | 0.35484 |
| 9699 | S11_76365848 | 11 | 76365848 | 178342 | G | C | G | 0.72581 | C | 0.27419 | 15 | 0.48387 |
| 9700 | S11_77638325 | 11 | 77638325 | 1272477 | A | G | A | 0.72581 | G | 0.27419 | 9 | 0.29032 |
| 9701 | S11_77638400 | 11 | 77638400 | 75 | C | T | C | 0.72581 | T | 0.27419 | 9 | 0.29032 |
| 9702 | S11_77638404 | 11 | 77638404 | 4 | T | G | T | 0.72581 | G | 0.27419 | 9 | 0.29032 |
| 9703 | S11_77799418 | 11 | 77799418 | 161014 | T | C | T | 0.70968 | C | 0.29032 | 14 | 0.45161 |
| 9704 | S11_77799588 | 11 | 77799588 | 170 | T | C | T | 0.62903 | C | 0.37097 | 15 | 0.48387 |
| 9705 | S11_78164975 | 11 | 78164975 | 365387 | C | T | C | 0.54839 | T | 0.45161 | 14 | 0.45161 |
| 9706 | S11_78165117 | 11 | 78165117 | 142 | C | T | C | 0.77419 | T | 0.22581 | 8 | 0.25806 |
| 9707 | S11_78165130 | 11 | 78165130 | 13 | G | A | G | 0.95161 | A | 0.04839 | 3 | 0.09677 |
| 9708 | S11_78167839 | 11 | 78167839 | 2709 | G | T | G | 0.75806 | T | 0.24194 | 11 | 0.35484 |
| 9709 | S11_78167997 | 11 | 78167997 | 158 | A | C | A | 0.6129 | C | 0.3871 | 16 | 0.51613 |
| 9710 | S11_78665770 | 11 | 78665770 | 497773 | G | A | G | 0.75806 | A | 0.24194 | 15 | 0.48387 |
| 9711 | S11_78752255 | 11 | 78752255 | 86485 | G | C | C | 0.90323 | G | 0.09677 | 6 | 0.19355 |
| 9712 | S11_78850588 | 11 | 78850588 | 98333 | T | C | T | 0.82258 | C | 0.17742 | 9 | 0.29032 |
| 9713 | S11_80339888 | 11 | 80339888 | 1489300 | C | A | C | 0.83871 | A | 0.16129 | 8 | 0.25806 |
| 9714 | S11_80339960 | 11 | 80339960 | 72 | T | C | C | 0.95161 | T | 0.04839 | 3 | 0.09677 |
| 9715 | S11_80469407 | 11 | 80469407 | 129447 | A | G | A | 0.83871 | G | 0.16129 | 8 | 0.25806 |
| 9716 | S11_80692863 | 11 | 80692863 | 223456 | G | A | G | 0.74194 | A | 0.25806 | 10 | 0.32258 |
| 9717 | S11_80692887 | 11 | 80692887 | 24 | A | C | C | 0.90323 | A | 0.09677 | 6 | 0.19355 |
| 9718 | S11_80692955 | 11 | 80692955 | 68 | C | G | C | 0.6129 | G | 0.3871 | 14 | 0.45161 |

| Site number | SNP name | Chromosome | Physical position | Physical distance from the previous SNP | Reference allele | Alternative allele | Major allele | Major allele frequency | Minor allele | Minor allele frequency | Number heterozygous | Heterozygosity |
|---|---|---|---|---|---|---|---|---|---|---|---|---|
| 9719 | S11_80751208 | 11 | 80751208 | 58253 | C | T | C | 0.74194 | T | 0.25806 | 14 | 0.45161 |
| 9720 | S11_80767653 | 11 | 80767653 | 16445 | G | C | G | 0.85484 | C | 0.14516 | 7 | 0.22581 |
| 9721 | S11_80767655 | 11 | 80767655 | 2 | G | C | G | 0.67742 | C | 0.32258 | 12 | 0.3871 |
| 9722 | S11_80767680 | 11 | 80767680 | 25 | C | T | C | 0.64516 | T | 0.35484 | 14 | 0.45161 |
| 9723 | S11_81311241 | 11 | 81311241 | 543561 | G | T | G | 0.8871 | T | 0.1129 | 7 | 0.22581 |
| 9724 | S11_81876582 | 11 | 81876582 | 565341 | G | A | G | 0.8871 | A | 0.1129 | 7 | 0.22581 |
| 9725 | S11_81876671 | 11 | 81876671 | 89 | G | A | G | 0.77419 | A | 0.22581 | 12 | 0.3871 |
| 9726 | S11_82049451 | 11 | 82049451 | 172780 | C | T | T | 0.53226 | C | 0.46774 | 19 | 0.6129 |
| 9727 | S11_82069324 | 11 | 82069324 | 19873 | A | G | A | 0.72581 | G | 0.27419 | 13 | 0.41935 |
| 9728 | S11_82069346 | 11 | 82069346 | 22 | A | G | A | 0.70968 | G | 0.29032 | 16 | 0.51613 |
| 9729 | S11_82501329 | 11 | 82501329 | 431983 | C | T | C | 0.90323 | T | 0.09677 | 6 | 0.19355 |
| 9730 | S11_82501333 | 11 | 82501333 | 4 | T | A | T | 0.93548 | A | 0.06452 | 4 | 0.12903 |
| 9731 | S11_82501352 | 11 | 82501352 | 19 | C | T | C | 0.93548 | T | 0.06452 | 4 | 0.12903 |
| 9732 | S11_82501401 | 11 | 82501401 | 49 | T | C | T | 0.93548 | C | 0.06452 | 4 | 0.12903 |
| 9733 | S11_82501453 | 11 | 82501453 | 52 | G | A | G | 0.91935 | A | 0.08065 | 5 | 0.16129 |
| 9734 | S11_82501464 | 11 | 82501464 | 11 | C | T | C | 0.8871 | T | 0.1129 | 7 | 0.22581 |
| 9735 | S11_82501470 | 11 | 82501470 | 6 | G | A | G | 0.8871 | A | 0.1129 | 7 | 0.22581 |
| 9736 | S11_82501483 | 11 | 82501483 | 13 | C | T | C | 0.93548 | T | 0.06452 | 4 | 0.12903 |
| 9737 | S11_83236739 | 11 | 83236739 | 735256 | T | C | T | 0.93548 | C | 0.06452 | 4 | 0.12903 |
| 9738 | S11_83335971 | 11 | 83335971 | 99232 | C | T | C | 0.82258 | T | 0.17742 | 11 | 0.35484 |
| 9739 | S11_83336053 | 11 | 83336053 | 82 | A | G | A | 0.82258 | G | 0.17742 | 11 | 0.35484 |
| 9740 | S11_83336143 | 11 | 83336143 | 90 | A | G | A | 0.82258 | G | 0.17742 | 11 | 0.35484 |
| 9741 | S11_83588317 | 11 | 83588317 | 252174 | A | G | G | 0.53226 | A | 0.46774 | 17 | 0.54839 |
| 9742 | S11_83588439 | 11 | 83588439 | 122 | C | T | C | 0.95161 | T | 0.04839 | 3 | 0.09677 |
| 9743 | S11_83588440 | 11 | 83588440 | 1 | A | G | A | 0.59677 | G | 0.40323 | 17 | 0.54839 |
| 9744 | S11_83588451 | 11 | 83588451 | 11 | C | G | C | 0.59677 | G | 0.40323 | 19 | 0.6129 |
| 9745 | S11_83588487 | 11 | 83588487 | 36 | A | G | A | 0.87097 | G | 0.12903 | 8 | 0.25806 |
| 9746 | S11_83588506 | 11 | 83588506 | 19 | C | T | C | 0.87097 | T | 0.12903 | 8 | 0.25806 |
| 9747 | S11_84073213 | 11 | 84073213 | 484707 | C | T | C | 0.79032 | T | 0.20968 | 11 | 0.35484 |
| 9748 | S11_84832109 | 11 | 84832109 | 758896 | C | A | A | 0.82258 | C | 0.17742 | 9 | 0.29032 |
| 9749 | S11_85036059 | 11 | 85036059 | 203950 | C | T | C | 0.69355 | T | 0.30645 | 11 | 0.35484 |
| 9750 | S11_85146310 | 11 | 85146310 | 110251 | G | A | G | 0.6129 | A | 0.3871 | 14 | 0.45161 |
| 9751 | S11_85146314 | 11 | 85146314 | 4 | G | A | A | 0.53226 | G | 0.46774 | 17 | 0.54839 |
| 9752 | S11_85470961 | 11 | 85470961 | 324647 | G | A | G | 0.70968 | A | 0.29032 | 12 | 0.3871 |
| 9753 | S11_85526911 | 11 | 85526911 | 55950 | G | A | G | 0.95161 | A | 0.04839 | 3 | 0.09677 |
| 9754 | S11_85526996 | 11 | 85526996 | 85 | A | G | G | 0.82258 | A | 0.17742 | 11 | 0.35484 |
| 9755 | S11_85527010 | 11 | 85527010 | 14 | G | A | G | 0.82258 | A | 0.17742 | 9 | 0.29032 |
| 9756 | S11_85527113 | 11 | 85527113 | 103 | C | T | T | 0.82258 | C | 0.17742 | 11 | 0.35484 |
| 9757 | S11_85769428 | 11 | 85769428 | 242315 | A | G | G | 0.80645 | A | 0.19355 | 8 | 0.25806 |
| 9758 | S11_85769505 | 11 | 85769505 | 77 | A | C | C | 0.51613 | A | 0.48387 | 16 | 0.51613 |
| 9759 | S11_85865391 | 11 | 85865391 | 95886 | G | A | G | 0.90323 | A | 0.09677 | 4 | 0.12903 |
| 9760 | S11_85881236 | 11 | 85881236 | 15845 | G | A | G | 0.59677 | A | 0.40323 | 17 | 0.54839 |
| 9761 | S11_85881238 | 11 | 85881238 | 2 | A | G | G | 0.67742 | A | 0.32258 | 16 | 0.51613 |
| 9762 | S11_86292390 | 11 | 86292390 | 411152 | A | G | A | 0.93548 | G | 0.06452 | 4 | 0.12903 |
| 9763 | S11_86292415 | 11 | 86292415 | 25 | G | A | A | 0.82258 | G | 0.17742 | 9 | 0.29032 |
| 9764 | S11_86292496 | 11 | 86292496 | 81 | C | A | C | 0.91935 | A | 0.08065 | 3 | 0.09677 |
| 9765 | S11_86292579 | 11 | 86292579 | 83 | C | A | A | 0.82258 | C | 0.17742 | 9 | 0.29032 |
| 9766 | S11_86382435 | 11 | 86382435 | 89856 | A | C | A | 0.93548 | C | 0.06452 | 2 | 0.06452 |
| 9767 | S11_86382512 | 11 | 86382512 | 77 | C | T | C | 0.79032 | T | 0.20968 | 9 | 0.29032 |
| 9768 | S11_86901831 | 11 | 86901831 | 519319 | G | A | G | 0.72581 | A | 0.27419 | 15 | 0.48387 |
| 9769 | S11_87473855 | 11 | 87473855 | 572024 | C | T | C | 0.82258 | T | 0.17742 | 11 | 0.35484 |
| 9770 | S11_87473877 | 11 | 87473877 | 22 | A | G | A | 0.83871 | G | 0.16129 | 10 | 0.32258 |
| 9771 | S11_87473889 | 11 | 87473889 | 12 | T | C | T | 0.93548 | C | 0.06452 | 4 | 0.12903 |
| 9772 | S11_87481489 | 11 | 87481489 | 7600 | G | A | G | 0.91935 | A | 0.08065 | 5 | 0.16129 |

| Site number | SNP name | Chromosome | Physical position | Physical distance from the previous SNP | Reference allele | Alternative allele | Major allele | Major allele frequency | Minor allele | Minor allele frequency | Number heterozygous | Heterozygosity |
|---|---|---|---|---|---|---|---|---|---|---|---|---|
| 9773 | S11_87481533 | 11 | 87481533 | 44 | G | A | G | 0.79032 | A | 0.20968 | 11 | 0.35484 |
| 9774 | S11_87733391 | 11 | 87733391 | 251858 | A | G | A | 0.91935 | G | 0.08065 | 5 | 0.16129 |
| 9775 | S11_87733442 | 11 | 87733442 | 51 | T | C | T | 0.91935 | C | 0.08065 | 5 | 0.16129 |
| 9776 | S11_87733464 | 11 | 87733464 | 22 | T | C | T | 0.91935 | C | 0.08065 | 5 | 0.16129 |
| 9777 | S11_87733533 | 11 | 87733533 | 69 | T | C | T | 0.91935 | C | 0.08065 | 5 | 0.16129 |
| 9778 | S11_87897317 | 11 | 87897317 | 163784 | T | A | T | 0.95161 | A | 0.04839 | 3 | 0.09677 |
| 9779 | S11_87970652 | 11 | 87970652 | 73335 | C | T | T | 0.90323 | C | 0.09677 | 6 | 0.19355 |
| 9780 | S11_88004750 | 11 | 88004750 | 34098 | T | C | T | 0.90323 | C | 0.09677 | 4 | 0.12903 |
| 9781 | S11_88304079 | 11 | 88304079 | 299329 | G | C | G | 0.82258 | C | 0.17742 | 11 | 0.35484 |
| 9782 | S11_88411275 | 11 | 88411275 | 107196 | C | T | C | 0.64516 | T | 0.35484 | 16 | 0.51613 |
| 9783 | S11_88411326 | 11 | 88411326 | 51 | C | T | C | 0.56452 | T | 0.43548 | 19 | 0.6129 |
| 9784 | S11_88411404 | 11 | 88411404 | 78 | C | T | C | 0.70968 | T | 0.29032 | 14 | 0.45161 |
| 9785 | S11_88451658 | 11 | 88451658 | 40254 | T | C | C | 0.5 | T | 0.5 | 17 | 0.54839 |
| 9786 | S11_88459275 | 11 | 88459275 | 7617 | G | C | G | 0.90323 | C | 0.09677 | 6 | 0.19355 |
| 9787 | S11_88465978 | 11 | 88465978 | 6703 | G | A | G | 0.93548 | A | 0.06452 | 4 | 0.12903 |
| 9788 | S11_88590107 | 11 | 88590107 | 124129 | C | T | C | 0.87097 | T | 0.12903 | 6 | 0.19355 |
| 9789 | S11_88697444 | 11 | 88697444 | 107337 | T | G | T | 0.90323 | G | 0.09677 | 6 | 0.19355 |
| 9790 | S11_88697446 | 11 | 88697446 | 2 | C | T | C | 0.93548 | T | 0.06452 | 4 | 0.12903 |
| 9791 | S11_88920438 | 11 | 88920438 | 222992 | T | C | C | 0.91935 | T | 0.08065 | 5 | 0.16129 |
| 9792 | S11_88920639 | 11 | 88920639 | 201 | T | C | T | 0.80645 | C | 0.19355 | 10 | 0.32258 |
| 9793 | S11_88920648 | 11 | 88920648 | 9 | T | C | T | 0.66129 | C | 0.33871 | 17 | 0.54839 |
| 9794 | S11_89060612 | 11 | 89060612 | 139964 | C | T | C | 0.93548 | T | 0.06452 | 4 | 0.12903 |
| 9795 | S11_89278086 | 11 | 89278086 | 217474 | G | A | G | 0.95161 | A | 0.04839 | 3 | 0.09677 |
| 9796 | S11_89345775 | 11 | 89345775 | 67689 | G | A | A | 0.87097 | G | 0.12903 | 8 | 0.25806 |
| 9797 | S11_89784381 | 11 | 89784381 | 438606 | T | C | T | 0.62903 | C | 0.37097 | 17 | 0.54839 |
| 9798 | S11_89784478 | 11 | 89784478 | 97 | C | A | C | 0.62903 | A | 0.37097 | 17 | 0.54839 |
| 9799 | S11_89784521 | 11 | 89784521 | 43 | G | T | G | 0.93548 | T | 0.06452 | 4 | 0.12903 |
| 9800 | S11_90080907 | 11 | 90080907 | 296386 | T | C | T | 0.90323 | C | 0.09677 | 6 | 0.19355 |
| 9801 | S11_90080996 | 11 | 90080996 | 89 | C | G | C | 0.91935 | G | 0.08065 | 5 | 0.16129 |
| 9802 | S11_90188482 | 11 | 90188482 | 107486 | T | C | C | 0.66129 | T | 0.33871 | 19 | 0.6129 |
| 9803 | S11_90188525 | 11 | 90188525 | 43 | T | C | T | 0.8871 | C | 0.1129 | 7 | 0.22581 |
| 9804 | S11_90370009 | 11 | 90370009 | 181484 | G | A | G | 0.72581 | A | 0.27419 | 13 | 0.41935 |
| 9805 | S11_91273164 | 11 | 91273164 | 903155 | C | T | C | 0.75806 | T | 0.24194 | 13 | 0.41935 |
| 9806 | S11_91273194 | 11 | 91273194 | 30 | C | A | A | 0.56452 | C | 0.43548 | 15 | 0.48387 |
| 9807 | S11_91273308 | 11 | 91273308 | 114 | G | C | G | 0.69355 | C | 0.30645 | 13 | 0.41935 |
| 9808 | S11_91330050 | 11 | 91330050 | 56742 | T | C | T | 0.72581 | C | 0.27419 | 9 | 0.29032 |
| 9809 | S11_91617401 | 11 | 91617401 | 287351 | G | A | G | 0.80645 | A | 0.19355 | 8 | 0.25806 |
| 9810 | S11_92322044 | 11 | 92322044 | 704643 | C | T | C | 0.93548 | T | 0.06452 | 4 | 0.12903 |
| 9811 | S11_92619895 | 11 | 92619895 | 297851 | T | C | T | 0.72581 | C | 0.27419 | 9 | 0.29032 |
| 9812 | S11_92619906 | 11 | 92619906 | 11 | G | A | G | 0.93548 | A | 0.06452 | 4 | 0.12903 |
| 9813 | S11_92619960 | 11 | 92619960 | 54 | A | G | A | 0.72581 | G | 0.27419 | 9 | 0.29032 |
| 9814 | S11_92620017 | 11 | 92620017 | 57 | T | C | T | 0.72581 | C | 0.27419 | 9 | 0.29032 |
| 9815 | S11_92620062 | 11 | 92620062 | 45 | A | G | A | 0.72581 | G | 0.27419 | 9 | 0.29032 |
| 9816 | S11_92927574 | 11 | 92927574 | 307512 | G | A | G | 0.90323 | A | 0.09677 | 6 | 0.19355 |
| 9817 | S11_93103590 | 11 | 93103590 | 176016 | A | G | G | 0.62903 | A | 0.37097 | 17 | 0.54839 |
| 9818 | S11_93103699 | 11 | 93103699 | 109 | T | C | T | 0.91935 | C | 0.08065 | 5 | 0.16129 |
| 9819 | S11_94730915 | 11 | 94730915 | 1627216 | G | A | G | 0.87097 | A | 0.12903 | 4 | 0.12903 |
| 9820 | S11_94731056 | 11 | 94731056 | 141 | A | G | G | 0.90323 | A | 0.09677 | 4 | 0.12903 |
| 9821 | S11_95206675 | 11 | 95206675 | 475619 | C | T | C | 0.93548 | T | 0.06452 | 4 | 0.12903 |
| 9822 | S11_95300633 | 11 | 95300633 | 93958 | T | G | T | 0.53226 | G | 0.46774 | 15 | 0.48387 |
| 9823 | S11_95300708 | 11 | 95300708 | 75 | A | C | A | 0.70968 | C | 0.29032 | 14 | 0.45161 |
| 9824 | S11_95829790 | 11 | 95829790 | 529082 | C | T | C | 0.8871 | T | 0.1129 | 5 | 0.16129 |
| 9825 | S11_95829795 | 11 | 95829795 | 5 | C | A | C | 0.8871 | A | 0.1129 | 5 | 0.16129 |
| 9826 | S11_95829829 | 11 | 95829829 | 34 | C | T | C | 0.8871 | T | 0.1129 | 5 | 0.16129 |

| Site number | SNP name | Chromosome | Physical position | Physical distance from the previous SNP | Reference allele | Alternative allele | Major allele | Major allele frequency | Minor allele | Minor allele frequency | Number heterozygous | Heterozygosity |
|---|---|---|---|---|---|---|---|---|---|---|---|---|
| 9827 | S11_95829840 | 11 | 95829840 | 11 | C | T | C | 0.8871 | T | 0.1129 | 5 | 0.16129 |
| 9828 | S11_95829883 | 11 | 95829883 | 43 | C | T | C | 0.8871 | T | 0.1129 | 5 | 0.16129 |
| 9829 | S11_95829982 | 11 | 95829982 | 99 | C | T | C | 0.69355 | T | 0.30645 | 13 | 0.41935 |
| 9830 | S11_96678098 | 11 | 96678098 | 848116 | C | T | T | 0.59677 | C | 0.40323 | 13 | 0.41935 |
| 9831 | S11_97130374 | 11 | 97130374 | 452276 | C | T | C | 0.93548 | T | 0.06452 | 4 | 0.12903 |
| 9832 | S11_97649781 | 11 | 97649781 | 519407 | G | A | G | 0.79032 | A | 0.20968 | 9 | 0.29032 |
| 9833 | S11_97652709 | 11 | 97652709 | 2928 | C | T | C | 0.83871 | T | 0.16129 | 8 | 0.25806 |
| 9834 | S11_98272783 | 11 | 98272783 | 620074 | T | C | T | 0.82258 | C | 0.17742 | 9 | 0.29032 |
| 9835 | S11_98541919 | 11 | 98541919 | 269136 | T | C | T | 0.8871 | C | 0.1129 | 7 | 0.22581 |
| 9836 | S11_98611569 | 11 | 98611569 | 69650 | G | A | G | 0.95161 | A | 0.04839 | 3 | 0.09677 |
| 9837 | S11_98684713 | 11 | 98684713 | 73144 | G | A | G | 0.72581 | A | 0.27419 | 15 | 0.48387 |
| 9838 | S11_98684740 | 11 | 98684740 | 27 | A | G | A | 0.59677 | G | 0.40323 | 19 | 0.6129 |
| 9839 | S11_98684765 | 11 | 98684765 | 25 | G | A | G | 0.85484 | A | 0.14516 | 9 | 0.29032 |
| 9840 | S11_98859508 | 11 | 98859508 | 174743 | G | C | G | 0.74194 | C | 0.25806 | 12 | 0.3871 |
| 9841 | S11_98917269 | 11 | 98917269 | 57761 | G | T | G | 0.90323 | T | 0.09677 | 6 | 0.19355 |
| 9842 | S11_98917461 | 11 | 98917461 | 192 | C | T | C | 0.95161 | T | 0.04839 | 3 | 0.09677 |
| 9843 | S11_99001212 | 11 | 99001212 | 83751 | C | T | C | 0.83871 | T | 0.16129 | 8 | 0.25806 |
| 9844 | S11_99295465 | 11 | 99295465 | 294253 | G | A | G | 0.82258 | A | 0.17742 | 7 | 0.22581 |
| 9845 | S11_99415417 | 11 | 99415417 | 119952 | C | T | C | 0.75806 | T | 0.24194 | 11 | 0.35484 |
| 9846 | S11_99415457 | 11 | 99415457 | 40 | C | T | T | 0.56452 | C | 0.43548 | 15 | 0.48387 |
| 9847 | S11_99415476 | 11 | 99415476 | 19 | T | C | T | 0.87097 | C | 0.12903 | 6 | 0.19355 |
| 9848 | S11_99415511 | 11 | 99415511 | 35 | G | A | G | 0.6129 | A | 0.3871 | 16 | 0.51613 |
| 9849 | S11_99415521 | 11 | 99415521 | 10 | C | G | C | 0.8871 | G | 0.1129 | 5 | 0.16129 |
| 9850 | S11_99415604 | 11 | 99415604 | 83 | C | T | C | 0.59677 | T | 0.40323 | 13 | 0.41935 |
| 9851 | S11_99416990 | 11 | 99416990 | 1386 | C | T | T | 0.83871 | C | 0.16129 | 8 | 0.25806 |
| 9852 | S11_99417019 | 11 | 99417019 | 29 | C | T | T | 0.83871 | C | 0.16129 | 8 | 0.25806 |
| 9853 | S11_99417023 | 11 | 99417023 | 4 | C | G | G | 0.79032 | C | 0.20968 | 11 | 0.35484 |
| 9854 | S11_99882837 | 11 | 99882837 | 465814 | A | C | A | 0.8871 | C | 0.1129 | 5 | 0.16129 |
| 9855 | S11_99882860 | 11 | 99882860 | 23 | C | T | T | 0.83871 | C | 0.16129 | 8 | 0.25806 |
| 9856 | S11_99882921 | 11 | 99882921 | 61 | G | A | G | 0.8871 | A | 0.1129 | 7 | 0.22581 |
| 9857 | S11_99882979 | 11 | 99882979 | 58 | G | A | A | 0.83871 | G | 0.16129 | 8 | 0.25806 |
| 9858 | S11_99904053 | 11 | 99904053 | 21074 | C | T | C | 0.87097 | T | 0.12903 | 8 | 0.25806 |
| 9859 | S11_100039231 | 11 | 100039231 | 135178 | G | A | G | 0.6129 | A | 0.3871 | 18 | 0.58065 |
| 9860 | S11_100039314 | 11 | 100039314 | 83 | A | T | T | 0.82258 | A | 0.17742 | 9 | 0.29032 |
| 9861 | S11_100077096 | 11 | 100077096 | 37782 | C | T | C | 0.66129 | T | 0.33871 | 13 | 0.41935 |
| 9862 | S11_100101463 | 11 | 100101463 | 24367 | G | A | G | 0.91935 | A | 0.08065 | 5 | 0.16129 |
| 9863 | S11_100213337 | 11 | 100213337 | 111874 | T | C | T | 0.77419 | C | 0.22581 | 14 | 0.45161 |
| 9864 | S11_100213347 | 11 | 100213347 | 10 | G | A | G | 0.77419 | A | 0.22581 | 14 | 0.45161 |
| 9865 | S11_100311526 | 11 | 100311526 | 98179 | G | A | G | 0.66129 | A | 0.33871 | 15 | 0.48387 |
| 9866 | S11_100311631 | 11 | 100311631 | 105 | C | T | C | 0.8871 | T | 0.1129 | 7 | 0.22581 |
| 9867 | S11_100621957 | 11 | 100621957 | 310326 | A | G | G | 0.93548 | A | 0.06452 | 4 | 0.12903 |
| 9868 | S11_100820911 | 11 | 100820911 | 198954 | G | A | G | 0.93548 | A | 0.06452 | 4 | 0.12903 |
| 9869 | S11_100820926 | 11 | 100820926 | 15 | G | A | G | 0.62903 | A | 0.37097 | 13 | 0.41935 |
| 9870 | S11_100846383 | 11 | 100846383 | 25457 | C | G | C | 0.87097 | G | 0.12903 | 6 | 0.19355 |
| 9871 | S11_100942269 | 11 | 100942269 | 95886 | A | G | A | 0.83871 | G | 0.16129 | 6 | 0.19355 |
| 9872 | S11_100942354 | 11 | 100942354 | 85 | A | G | A | 0.83871 | G | 0.16129 | 6 | 0.19355 |
| 9873 | S11_101126395 | 11 | 101126395 | 184041 | C | T | C | 0.91935 | T | 0.08065 | 5 | 0.16129 |
| 9874 | S11_101126423 | 11 | 101126423 | 28 | C | T | C | 0.56452 | T | 0.43548 | 19 | 0.6129 |
| 9875 | S11_101126459 | 11 | 101126459 | 36 | C | T | C | 0.93548 | T | 0.06452 | 4 | 0.12903 |
| 9876 | S11_101126618 | 11 | 101126618 | 159 | A | C | A | 0.56452 | C | 0.43548 | 19 | 0.6129 |
| 9877 | S11_101323437 | 11 | 101323437 | 196819 | C | T | C | 0.93548 | T | 0.06452 | 4 | 0.12903 |
| 9878 | S11_101323580 | 11 | 101323580 | 143 | C | T | C | 0.80645 | T | 0.19355 | 8 | 0.25806 |
| 9879 | S11_101518204 | 11 | 101518204 | 194624 | C | A | C | 0.75806 | A | 0.24194 | 13 | 0.41935 |
| 9880 | S11_101544330 | 11 | 101544330 | 26126 | G | A | G | 0.93548 | A | 0.06452 | 4 | 0.12903 |

| Site number | SNP name | Chromosome | Physical position | Physical distance from the previous SNP | Reference allele | Alternative allele | Major allele | Major allele frequency | Minor allele | Minor allele frequency | Number heterozygous | Heterozygosity |
|---|---|---|---|---|---|---|---|---|---|---|---|---|
| 9881 | S11_101544350 | 11 | 101544350 | 20 | G | A | G | 0.85484 | A | 0.14516 | 5 | 0.16129 |
| 9882 | S11_101544394 | 11 | 101544394 | 44 | T | C | T | 0.91935 | C | 0.08065 | 5 | 0.16129 |
| 9883 | S11_101555280 | 11 | 101555280 | 10886 | A | G | A | 0.90323 | G | 0.09677 | 6 | 0.19355 |
| 9884 | S11_101701856 | 11 | 101701856 | 146576 | A | C | A | 0.93548 | C | 0.06452 | 4 | 0.12903 |
| 9885 | S11_101701881 | 11 | 101701881 | 25 | A | C | A | 0.93548 | C | 0.06452 | 4 | 0.12903 |
| 9886 | S11_101865698 | 11 | 101865698 | 163817 | G | C | G | 0.95161 | C | 0.04839 | 3 | 0.09677 |
| 9887 | S11_101939143 | 11 | 101939143 | 73445 | G | C | G | 0.95161 | C | 0.04839 | 3 | 0.09677 |
| 9888 | S11_101939256 | 11 | 101939256 | 113 | C | T | C | 0.90323 | T | 0.09677 | 6 | 0.19355 |
| 9889 | S11_102019050 | 11 | 102019050 | 79794 | C | T | C | 0.87097 | T | 0.12903 | 6 | 0.19355 |
| 9890 | S11_102019057 | 11 | 102019057 | 7 | G | A | G | 0.87097 | A | 0.12903 | 6 | 0.19355 |
| 9891 | S11_102019130 | 11 | 102019130 | 73 | C | T | C | 0.58065 | T | 0.41935 | 18 | 0.58065 |
| 9892 | S11_102321835 | 11 | 102321835 | 302705 | G | A | G | 0.95161 | A | 0.04839 | 3 | 0.09677 |
| 9893 | S11_102473603 | 11 | 102473603 | 151768 | T | C | C | 0.54839 | T | 0.45161 | 10 | 0.32258 |
| 9894 | S11_102473698 | 11 | 102473698 | 95 | G | A | G | 0.83871 | A | 0.16129 | 8 | 0.25806 |
| 9895 | S11_102473720 | 11 | 102473720 | 22 | C | T | C | 0.72581 | T | 0.27419 | 13 | 0.41935 |
| 9896 | S11_102473777 | 11 | 102473777 | 57 | C | T | C | 0.72581 | T | 0.27419 | 13 | 0.41935 |
| 9897 | S11_102635914 | 11 | 102635914 | 162137 | G | A | G | 0.87097 | A | 0.12903 | 6 | 0.19355 |
| 9898 | S11_102635948 | 11 | 102635948 | 34 | C | T | T | 0.79032 | C | 0.20968 | 11 | 0.35484 |
| 9899 | S11_102882670 | 11 | 102882670 | 246722 | C | G | G | 0.67742 | C | 0.32258 | 12 | 0.3871 |
| 9900 | S11_103116296 | 11 | 103116296 | 233626 | C | T | C | 0.8871 | T | 0.1129 | 7 | 0.22581 |
| 9901 | S11_103116319 | 11 | 103116319 | 23 | G | C | C | 0.75806 | G | 0.24194 | 15 | 0.48387 |
| 9902 | S11_103116436 | 11 | 103116436 | 117 | A | G | G | 0.75806 | A | 0.24194 | 15 | 0.48387 |
| 9903 | S11_103116483 | 11 | 103116483 | 47 | C | T | C | 0.82258 | T | 0.17742 | 9 | 0.29032 |
| 9904 | S11_103116511 | 11 | 103116511 | 28 | C | T | C | 0.82258 | T | 0.17742 | 9 | 0.29032 |
| 9905 | S11_103477647 | 11 | 103477647 | 361136 | T | C | T | 0.56452 | C | 0.43548 | 11 | 0.35484 |
| 9906 | S11_103477716 | 11 | 103477716 | 69 | A | G | G | 0.58065 | A | 0.41935 | 12 | 0.3871 |
| 9907 | S11_103477765 | 11 | 103477765 | 49 | C | T | C | 0.80645 | T | 0.19355 | 6 | 0.19355 |
| 9908 | S11_103633557 | 11 | 103633557 | 155792 | G | A | G | 0.93548 | A | 0.06452 | 2 | 0.06452 |
| 9909 | S11_103633757 | 11 | 103633757 | 200 | C | T | C | 0.72581 | T | 0.27419 | 9 | 0.29032 |
| 9910 | S11_104006132 | 11 | 104006132 | 372375 | C | T | C | 0.93548 | T | 0.06452 | 2 | 0.06452 |
| 9911 | S11_104147177 | 11 | 104147177 | 141045 | A | G | G | 0.95161 | A | 0.04839 | 1 | 0.03226 |
| 9912 | S11_105033341 | 11 | 105033341 | 886164 | T | C | T | 0.91935 | C | 0.08065 | 5 | 0.16129 |
| 9913 | S11_105033373 | 11 | 105033373 | 32 | A | G | A | 0.83871 | G | 0.16129 | 10 | 0.32258 |
| 9914 | S11_105033412 | 11 | 105033412 | 39 | A | G | A | 0.91935 | G | 0.08065 | 5 | 0.16129 |
| 9915 | S11_105818806 | 11 | 105818806 | 785394 | A | G | A | 0.56452 | G | 0.43548 | 19 | 0.6129 |
| 9916 | S11_105980632 | 11 | 105980632 | 161826 | T | C | T | 0.90323 | C | 0.09677 | 6 | 0.19355 |
| 9917 | S11_105980688 | 11 | 105980688 | 56 | C | T | C | 0.90323 | T | 0.09677 | 6 | 0.19355 |
| 9918 | S11_106131606 | 11 | 106131606 | 150918 | T | C | C | 0.74194 | T | 0.25806 | 12 | 0.3871 |
| 9919 | S11_106131659 | 11 | 106131659 | 53 | T | C | C | 0.69355 | T | 0.30645 | 15 | 0.48387 |
| 9920 | S11_106131717 | 11 | 106131717 | 58 | T | C | C | 0.69355 | T | 0.30645 | 15 | 0.48387 |
| 9921 | S11_106131757 | 11 | 106131757 | 40 | C | T | T | 0.69355 | C | 0.30645 | 15 | 0.48387 |
| 9922 | S12_568871 | 12 | 568871 | 0 | G | A | G | 0.85484 | A | 0.14516 | 7 | 0.22581 |
| 9923 | S12_568889 | 12 | 568889 | 18 | A | T | A | 0.85484 | T | 0.14516 | 7 | 0.22581 |
| 9924 | S12_568895 | 12 | 568895 | 6 | C | T | C | 0.85484 | T | 0.14516 | 7 | 0.22581 |
| 9925 | S12_913583 | 12 | 913583 | 344688 | G | A | G | 0.87097 | A | 0.12903 | 6 | 0.19355 |
| 9926 | S12_913684 | 12 | 913684 | 101 | G | C | G | 0.90323 | C | 0.09677 | 6 | 0.19355 |
| 9927 | S12_973777 | 12 | 973777 | 60093 | G | A | G | 0.93548 | A | 0.06452 | 4 | 0.12903 |
| 9928 | S12_976061 | 12 | 976061 | 2284 | A | C | A | 0.93548 | C | 0.06452 | 4 | 0.12903 |
| 9929 | S12_976106 | 12 | 976106 | 45 | C | T | C | 0.87097 | T | 0.12903 | 6 | 0.19355 |
| 9930 | S12_1199452 | 12 | 1199452 | 223346 | C | A | C | 0.87097 | A | 0.12903 | 8 | 0.25806 |
| 9931 | S12_1227058 | 12 | 1227058 | 27606 | T | A | A | 0.74194 | T | 0.25806 | 12 | 0.3871 |
| 9932 | S12_1240878 | 12 | 1240878 | 13820 | A | T | A | 0.80645 | T | 0.19355 | 8 | 0.25806 |
| 9933 | S12_1398937 | 12 | 1398937 | 158059 | C | T | C | 0.59677 | T | 0.40323 | 13 | 0.41935 |
| 9934 | S12_1606984 | 12 | 1606984 | 208047 | C | T | C | 0.75806 | T | 0.24194 | 9 | 0.29032 |

| Site number | SNP name | Chromosome | Physical position | Physical distance from the previous SNP | Reference allele | Alternative allele | Major allele | Major allele frequency | Minor allele | Minor allele frequency | Number heterozygous | Heterozygosity |
|---|---|---|---|---|---|---|---|---|---|---|---|---|
| 9935 | S12_1607034 | 12 | 1607034 | 50 | G | A | G | 0.8871 | A | 0.1129 | 7 | 0.22581 |
| 9936 | S12_1953122 | 12 | 1953122 | 346088 | G | A | G | 0.91935 | A | 0.08065 | 5 | 0.16129 |
| 9937 | S12_1953177 | 12 | 1953177 | 55 | C | T | C | 0.87097 | T | 0.12903 | 6 | 0.19355 |
| 9938 | S12_1953258 | 12 | 1953258 | 81 | C | T | C | 0.66129 | T | 0.33871 | 13 | 0.41935 |
| 9939 | S12_2367526 | 12 | 2367526 | 414268 | G | A | G | 0.90323 | A | 0.09677 | 6 | 0.19355 |
| 9940 | S12_2643733 | 12 | 2643733 | 276207 | G | A | G | 0.8871 | A | 0.1129 | 5 | 0.16129 |
| 9941 | S12_2643760 | 12 | 2643760 | 27 | G | A | G | 0.82258 | A | 0.17742 | 9 | 0.29032 |
| 9942 | S12_2643820 | 12 | 2643820 | 60 | T | C | T | 0.74194 | C | 0.25806 | 8 | 0.25806 |
| 9943 | S12_2643823 | 12 | 2643823 | 3 | T | C | T | 0.80645 | C | 0.19355 | 8 | 0.25806 |
| 9944 | S12_2643863 | 12 | 2643863 | 40 | A | G | A | 0.74194 | G | 0.25806 | 8 | 0.25806 |
| 9945 | S12_2909669 | 12 | 2909669 | 265806 | T | C | T | 0.64516 | C | 0.35484 | 16 | 0.51613 |
| 9946 | S12_2909677 | 12 | 2909677 | 8 | G | A | G | 0.70968 | A | 0.29032 | 14 | 0.45161 |
| 9947 | S12_2909819 | 12 | 2909819 | 142 | G | T | G | 0.67742 | T | 0.32258 | 14 | 0.45161 |
| 9948 | S12_3264351 | 12 | 3264351 | 354532 | C | T | T | 0.72581 | C | 0.27419 | 11 | 0.35484 |
| 9949 | S12_3264356 | 12 | 3264356 | 5 | T | A | A | 0.93548 | T | 0.06452 | 4 | 0.12903 |
| 9950 | S12_3264380 | 12 | 3264380 | 24 | A | G | A | 0.79032 | G | 0.20968 | 9 | 0.29032 |
| 9951 | S12_3264437 | 12 | 3264437 | 57 | T | C | T | 0.79032 | C | 0.20968 | 9 | 0.29032 |
| 9952 | S12_3264452 | 12 | 3264452 | 15 | G | C | G | 0.79032 | C | 0.20968 | 9 | 0.29032 |
| 9953 | S12_3264463 | 12 | 3264463 | 11 | T | C | C | 0.93548 | T | 0.06452 | 4 | 0.12903 |
| 9954 | S12_3729526 | 12 | 3729526 | 465063 | C | T | C | 0.95161 | T | 0.04839 | 3 | 0.09677 |
| 9955 | S12_3729635 | 12 | 3729635 | 109 | T | C | T | 0.72581 | C | 0.27419 | 13 | 0.41935 |
| 9956 | S12_3993507 | 12 | 3993507 | 263872 | G | A | G | 0.93548 | A | 0.06452 | 4 | 0.12903 |
| 9957 | S12_4195061 | 12 | 4195061 | 201554 | A | G | A | 0.82258 | G | 0.17742 | 9 | 0.29032 |
| 9958 | S12_4369622 | 12 | 4369622 | 174561 | C | T | C | 0.72581 | T | 0.27419 | 13 | 0.41935 |
| 9959 | S12_4369828 | 12 | 4369828 | 206 | C | G | G | 0.90323 | C | 0.09677 | 6 | 0.19355 |
| 9960 | S12_4464225 | 12 | 4464225 | 94397 | A | G | A | 0.56452 | G | 0.43548 | 15 | 0.48387 |
| 9961 | S12_4464356 | 12 | 4464356 | 131 | C | T | C | 0.93548 | T | 0.06452 | 4 | 0.12903 |
| 9962 | S12_4465134 | 12 | 4465134 | 778 | G | C | G | 0.85484 | C | 0.14516 | 7 | 0.22581 |
| 9963 | S12_4507589 | 12 | 4507589 | 42455 | C | T | C | 0.74194 | T | 0.25806 | 10 | 0.32258 |
| 9964 | S12_4737612 | 12 | 4737612 | 230023 | A | G | A | 0.95161 | G | 0.04839 | 3 | 0.09677 |
| 9965 | S12_5026999 | 12 | 5026999 | 289387 | T | C | T | 0.90323 | C | 0.09677 | 6 | 0.19355 |
| 9966 | S12_5031624 | 12 | 5031624 | 4625 | C | T | C | 0.93548 | T | 0.06452 | 4 | 0.12903 |
| 9967 | S12_5173087 | 12 | 5173087 | 141463 | G | A | G | 0.93548 | A | 0.06452 | 4 | 0.12903 |
| 9968 | S12_5173150 | 12 | 5173150 | 63 | A | G | G | 0.53226 | A | 0.46774 | 15 | 0.48387 |
| 9969 | S12_5350635 | 12 | 5350635 | 177485 | T | C | T | 0.93548 | C | 0.06452 | 4 | 0.12903 |
| 9970 | S12_5350826 | 12 | 5350826 | 191 | A | T | A | 0.93548 | T | 0.06452 | 4 | 0.12903 |
| 9971 | S12_5350838 | 12 | 5350838 | 12 | C | T | C | 0.77419 | T | 0.22581 | 10 | 0.32258 |
| 9972 | S12_5367368 | 12 | 5367368 | 16530 | T | A | T | 0.53226 | A | 0.46774 | 17 | 0.54839 |
| 9973 | S12_5794128 | 12 | 5794128 | 426760 | G | A | G | 0.62903 | A | 0.37097 | 17 | 0.54839 |
| 9974 | S12_6121858 | 12 | 6121858 | 327730 | C | G | C | 0.95161 | G | 0.04839 | 3 | 0.09677 |
| 9975 | S12_6802053 | 12 | 6802053 | 680195 | T | C | T | 0.80645 | C | 0.19355 | 10 | 0.32258 |
| 9976 | S12_6802055 | 12 | 6802055 | 2 | G | A | G | 0.83871 | A | 0.16129 | 10 | 0.32258 |
| 9977 | S12_6809072 | 12 | 6809072 | 7017 | G | T | G | 0.75806 | T | 0.24194 | 13 | 0.41935 |
| 9978 | S12_6910394 | 12 | 6910394 | 101322 | T | A | A | 0.74194 | T | 0.25806 | 12 | 0.3871 |
| 9979 | S12_6910473 | 12 | 6910473 | 79 | T | A | T | 0.93548 | A | 0.06452 | 4 | 0.12903 |
| 9980 | S12_6928815 | 12 | 6928815 | 18342 | A | G | G | 0.87097 | A | 0.12903 | 8 | 0.25806 |
| 9981 | S12_6928941 | 12 | 6928941 | 126 | C | G | C | 0.87097 | G | 0.12903 | 6 | 0.19355 |
| 9982 | S12_7308394 | 12 | 7308394 | 379453 | G | A | A | 0.54839 | G | 0.45161 | 16 | 0.51613 |
| 9983 | S12_7324058 | 12 | 7324058 | 15664 | C | T | C | 0.91935 | T | 0.08065 | 5 | 0.16129 |
| 9984 | S12_7324083 | 12 | 7324083 | 25 | C | T | C | 0.91935 | T | 0.08065 | 5 | 0.16129 |
| 9985 | S12_7324163 | 12 | 7324163 | 80 | G | T | G | 0.83871 | T | 0.16129 | 8 | 0.25806 |
| 9986 | S12_7324164 | 12 | 7324164 | 1 | A | G | A | 0.83871 | G | 0.16129 | 8 | 0.25806 |
| 9987 | S12_7550872 | 12 | 7550872 | 226708 | C | T | C | 0.8871 | T | 0.1129 | 5 | 0.16129 |
| 9988 | S12_7679391 | 12 | 7679391 | 128519 | G | A | G | 0.64516 | A | 0.35484 | 18 | 0.58065 |

| Site number | SNP name | Chromosome | Physical position | Physical distance from the previous SNP | Reference allele | Alternative allele | Major allele | Major allele frequency | Minor allele | Minor allele frequency | Number heterozygous | Heterozygosity |
|---|---|---|---|---|---|---|---|---|---|---|---|---|
| 9989 | S12_7679518 | 12 | 7679518 | 127 | C | A | C | 0.90323 | A | 0.09677 | 4 | 0.12903 |
| 9990 | S12_7679525 | 12 | 7679525 | 7 | T | G | T | 0.90323 | G | 0.09677 | 6 | 0.19355 |
| 9991 | S12_7826858 | 12 | 7826858 | 147333 | T | C | C | 0.80645 | T | 0.19355 | 8 | 0.25806 |
| 9992 | S12_7927186 | 12 | 7927186 | 100328 | C | T | T | 0.75806 | C | 0.24194 | 13 | 0.41935 |
| 9993 | S12_7927187 | 12 | 7927187 | 1 | G | A | G | 0.90323 | A | 0.09677 | 6 | 0.19355 |
| 9994 | S12_7927217 | 12 | 7927217 | 30 | T | C | C | 0.75806 | T | 0.24194 | 13 | 0.41935 |
| 9995 | S12_7927235 | 12 | 7927235 | 18 | C | A | A | 0.75806 | C | 0.24194 | 13 | 0.41935 |
| 9996 | S12_7927247 | 12 | 7927247 | 12 | C | G | G | 0.75806 | C | 0.24194 | 13 | 0.41935 |
| 9997 | S12_7927291 | 12 | 7927291 | 44 | G | A | A | 0.75806 | G | 0.24194 | 13 | 0.41935 |
| 9998 | S12_7927330 | 12 | 7927330 | 39 | T | C | C | 0.75806 | T | 0.24194 | 13 | 0.41935 |
| 9999 | S12_7927333 | 12 | 7927333 | 3 | C | T | C | 0.56452 | T | 0.43548 | 19 | 0.6129 |
| 10000 | S12_7927334 | 12 | 7927334 | 1 | T | G | G | 0.56452 | T | 0.43548 | 21 | 0.67742 |
| 10001 | S12_7927335 | 12 | 7927335 | 1 | T | C | C | 0.56452 | T | 0.43548 | 21 | 0.67742 |
| 10002 | S12_7960686 | 12 | 7960686 | 33351 | A | G | A | 0.95161 | G | 0.04839 | 3 | 0.09677 |
| 10003 | S12_7960734 | 12 | 7960734 | 48 | A | G | A | 0.95161 | G | 0.04839 | 3 | 0.09677 |
| 10004 | S12_7960759 | 12 | 7960759 | 25 | A | G | G | 0.75806 | A | 0.24194 | 11 | 0.35484 |
| 10005 | S12_8014607 | 12 | 8014607 | 53848 | G | T | G | 0.87097 | T | 0.12903 | 6 | 0.19355 |
| 10006 | S12_8014838 | 12 | 8014838 | 231 | C | T | C | 0.93548 | T | 0.06452 | 4 | 0.12903 |
| 10007 | S12_8658650 | 12 | 8658650 | 643812 | G | C | G | 0.93548 | C | 0.06452 | 4 | 0.12903 |
| 10008 | S12_8658668 | 12 | 8658668 | 18 | C | T | C | 0.93548 | T | 0.06452 | 4 | 0.12903 |
| 10009 | S12_8658679 | 12 | 8658679 | 11 | C | G | C | 0.93548 | G | 0.06452 | 4 | 0.12903 |
| 10010 | S12_8658715 | 12 | 8658715 | 36 | G | C | C | 0.6129 | G | 0.3871 | 14 | 0.45161 |
| 10011 | S12_8969914 | 12 | 8969914 | 311199 | G | A | A | 0.53226 | G | 0.46774 | 19 | 0.6129 |
| 10012 | S12_9039439 | 12 | 9039439 | 69525 | G | T | G | 0.90323 | T | 0.09677 | 4 | 0.12903 |
| 10013 | S12_9585247 | 12 | 9585247 | 545808 | A | C | C | 0.95161 | A | 0.04839 | 3 | 0.09677 |
| 10014 | S12_9625539 | 12 | 9625539 | 40292 | C | T | T | 0.66129 | C | 0.33871 | 17 | 0.54839 |
| 10015 | S12_9625553 | 12 | 9625553 | 14 | T | G | T | 0.8871 | G | 0.1129 | 5 | 0.16129 |
| 10016 | S12_9625630 | 12 | 9625630 | 77 | A | T | T | 0.82258 | A | 0.17742 | 11 | 0.35484 |
| 10017 | S12_9992971 | 12 | 9992971 | 367341 | A | G | A | 0.74194 | G | 0.25806 | 14 | 0.45161 |
| 10018 | S12_10021203 | 12 | 10021203 | 28232 | T | C | T | 0.62903 | C | 0.37097 | 15 | 0.48387 |
| 10019 | S12_10021281 | 12 | 10021281 | 78 | G | C | G | 0.62903 | C | 0.37097 | 15 | 0.48387 |
| 10020 | S12_10025861 | 12 | 10025861 | 4580 | A | G | G | 0.70968 | A | 0.29032 | 14 | 0.45161 |
| 10021 | S12_10231853 | 12 | 10231853 | 205992 | G | A | G | 0.56452 | A | 0.43548 | 11 | 0.35484 |
| 10022 | S12_10232044 | 12 | 10232044 | 191 | A | G | G | 0.53226 | A | 0.46774 | 13 | 0.41935 |
| 10023 | S12_10342230 | 12 | 10342230 | 110186 | C | T | T | 0.51613 | C | 0.48387 | 18 | 0.58065 |
| 10024 | S12_10342306 | 12 | 10342306 | 76 | G | A | G | 0.8871 | A | 0.1129 | 7 | 0.22581 |
| 10025 | S12_10394916 | 12 | 10394916 | 52610 | G | A | G | 0.77419 | A | 0.22581 | 12 | 0.3871 |
| 10026 | S12_10395029 | 12 | 10395029 | 113 | G | A | G | 0.77419 | A | 0.22581 | 12 | 0.3871 |
| 10027 | S12_10395075 | 12 | 10395075 | 46 | C | T | T | 0.69355 | C | 0.30645 | 15 | 0.48387 |
| 10028 | S12_10501926 | 12 | 10501926 | 106851 | C | T | C | 0.93548 | T | 0.06452 | 4 | 0.12903 |
| 10029 | S12_10501960 | 12 | 10501960 | 34 | T | C | C | 0.83871 | T | 0.16129 | 10 | 0.32258 |
| 10030 | S12_10502079 | 12 | 10502079 | 119 | G | A | G | 0.80645 | A | 0.19355 | 10 | 0.32258 |
| 10031 | S12_10668457 | 12 | 10668457 | 166378 | A | G | G | 0.91935 | A | 0.08065 | 3 | 0.09677 |
| 10032 | S12_10692922 | 12 | 10692922 | 24465 | T | C | C | 0.85484 | T | 0.14516 | 5 | 0.16129 |
| 10033 | S12_10692934 | 12 | 10692934 | 12 | C | T | T | 0.54839 | C | 0.45161 | 10 | 0.32258 |
| 10034 | S12_10692991 | 12 | 10692991 | 57 | C | G | G | 0.85484 | C | 0.14516 | 5 | 0.16129 |
| 10035 | S12_10781134 | 12 | 10781134 | 88143 | A | G | A | 0.90323 | G | 0.09677 | 4 | 0.12903 |
| 10036 | S12_10872564 | 12 | 10872564 | 91430 | A | G | G | 0.67742 | A | 0.32258 | 14 | 0.45161 |
| 10037 | S12_11187997 | 12 | 11187997 | 315433 | T | C | T | 0.8871 | C | 0.1129 | 7 | 0.22581 |
| 10038 | S12_11188006 | 12 | 11188006 | 9 | G | A | G | 0.91935 | A | 0.08065 | 5 | 0.16129 |
| 10039 | S12_11348240 | 12 | 11348240 | 160234 | T | A | T | 0.93548 | A | 0.06452 | 4 | 0.12903 |
| 10040 | S12_11348352 | 12 | 11348352 | 112 | A | G | A | 0.69355 | G | 0.30645 | 13 | 0.41935 |
| 10041 | S12_11530147 | 12 | 11530147 | 181795 | T | C | C | 0.72581 | T | 0.27419 | 13 | 0.41935 |
| 10042 | S12_11743860 | 12 | 11743860 | 213713 | T | C | C | 0.5 | T | 0.5 | 19 | 0.6129 |

| Site number | SNP name | Chromosome | Physical position | Physical distance from the previous SNP | Reference allele | Alternative allele | Major allele | Major allele frequency | Minor allele | Minor allele frequency | Number heterozygous | Heterozygosity |
|---|---|---|---|---|---|---|---|---|---|---|---|---|
| 10043 | S12_11743958 | 12 | 11743958 | 98 | C | T | C | 0.91935 | T | 0.08065 | 5 | 0.16129 |
| 10044 | S12_12310882 | 12 | 12310882 | 566924 | T | C | C | 0.5 | T | 0.5 | 17 | 0.54839 |
| 10045 | S12_12368598 | 12 | 12368598 | 57716 | C | G | C | 0.95161 | G | 0.04839 | 3 | 0.09677 |
| 10046 | S12_12453557 | 12 | 12453557 | 84959 | T | C | T | 0.91935 | C | 0.08065 | 5 | 0.16129 |
| 10047 | S12_12732183 | 12 | 12732183 | 278626 | C | T | T | 0.90323 | C | 0.09677 | 4 | 0.12903 |
| 10048 | S12_12732226 | 12 | 12732226 | 43 | G | A | G | 0.93548 | A | 0.06452 | 4 | 0.12903 |
| 10049 | S12_12756138 | 12 | 12756138 | 23912 | G | T | G | 0.80645 | T | 0.19355 | 10 | 0.32258 |
| 10050 | S12_13011752 | 12 | 13011752 | 255614 | A | C | A | 0.91935 | C | 0.08065 | 3 | 0.09677 |
| 10051 | S12_13257495 | 12 | 13257495 | 245743 | T | C | T | 0.69355 | C | 0.30645 | 17 | 0.54839 |
| 10052 | S12_14797623 | 12 | 14797623 | 1540128 | C | T | C | 0.67742 | T | 0.32258 | 12 | 0.3871 |
| 10053 | S12_14797639 | 12 | 14797639 | 16 | A | G | A | 0.75806 | G | 0.24194 | 9 | 0.29032 |
| 10054 | S12_14797711 | 12 | 14797711 | 72 | G | A | A | 0.5 | G | 0.5 | 17 | 0.54839 |
| 10055 | S12_14797796 | 12 | 14797796 | 85 | G | C | G | 0.75806 | C | 0.24194 | 9 | 0.29032 |
| 10056 | S12_16303341 | 12 | 16303341 | 1505545 | A | T | A | 0.83871 | T | 0.16129 | 10 | 0.32258 |
| 10057 | S12_16303374 | 12 | 16303374 | 33 | G | A | G | 0.56452 | A | 0.43548 | 13 | 0.41935 |
| 10058 | S12_16303441 | 12 | 16303441 | 67 | G | A | G | 0.95161 | A | 0.04839 | 3 | 0.09677 |
| 10059 | S12_16303496 | 12 | 16303496 | 55 | C | T | C | 0.83871 | T | 0.16129 | 10 | 0.32258 |
| 10060 | S12_16303562 | 12 | 16303562 | 66 | C | A | C | 0.69355 | A | 0.30645 | 13 | 0.41935 |
| 10061 | S12_16557238 | 12 | 16557238 | 253676 | G | T | G | 0.93548 | T | 0.06452 | 4 | 0.12903 |
| 10062 | S12_16713308 | 12 | 16713308 | 156070 | T | C | T | 0.90323 | C | 0.09677 | 6 | 0.19355 |
| 10063 | S12_16713309 | 12 | 16713309 | 1 | G | A | G | 0.91935 | A | 0.08065 | 5 | 0.16129 |
| 10064 | S12_16713317 | 12 | 16713317 | 8 | T | A | T | 0.87097 | A | 0.12903 | 8 | 0.25806 |
| 10065 | S12_16713347 | 12 | 16713347 | 30 | G | A | G | 0.85484 | A | 0.14516 | 9 | 0.29032 |
| 10066 | S12_16713438 | 12 | 16713438 | 91 | A | G | A | 0.91935 | G | 0.08065 | 5 | 0.16129 |
| 10067 | S12_16814344 | 12 | 16814344 | 100906 | C | T | C | 0.82258 | T | 0.17742 | 9 | 0.29032 |
| 10068 | S12_16814418 | 12 | 16814418 | 74 | A | G | A | 0.70968 | G | 0.29032 | 14 | 0.45161 |
| 10069 | S12_16814438 | 12 | 16814438 | 20 | C | T | C | 0.95161 | T | 0.04839 | 3 | 0.09677 |
| 10070 | S12_17109445 | 12 | 17109445 | 295007 | G | C | C | 0.83871 | G | 0.16129 | 8 | 0.25806 |
| 10071 | S12_17109671 | 12 | 17109671 | 226 | C | T | C | 0.80645 | T | 0.19355 | 8 | 0.25806 |
| 10072 | S12_17130993 | 12 | 17130993 | 21322 | T | C | T | 0.91935 | C | 0.08065 | 5 | 0.16129 |
| 10073 | S12_17172313 | 12 | 17172313 | 41320 | G | A | G | 0.6129 | A | 0.3871 | 14 | 0.45161 |
| 10074 | S12_17172326 | 12 | 17172326 | 13 | T | C | T | 0.83871 | C | 0.16129 | 6 | 0.19355 |
| 10075 | S12_17172413 | 12 | 17172413 | 87 | T | C | T | 0.95161 | C | 0.04839 | 3 | 0.09677 |
| 10076 | S12_17411088 | 12 | 17411088 | 238675 | C | T | T | 0.56452 | C | 0.43548 | 21 | 0.67742 |
| 10077 | S12_18299711 | 12 | 18299711 | 888623 | C | G | C | 0.74194 | G | 0.25806 | 16 | 0.51613 |
| 10078 | S12_19496534 | 12 | 19496534 | 1196823 | G | T | T | 0.91935 | G | 0.08065 | 5 | 0.16129 |
| 10079 | S12_19496537 | 12 | 19496537 | 3 | G | C | G | 0.6129 | C | 0.3871 | 14 | 0.45161 |
| 10080 | S12_19496581 | 12 | 19496581 | 44 | G | A | G | 0.91935 | A | 0.08065 | 5 | 0.16129 |
| 10081 | S12_19496588 | 12 | 19496588 | 7 | A | G | A | 0.85484 | G | 0.14516 | 7 | 0.22581 |
| 10082 | S12_19496589 | 12 | 19496589 | 1 | G | A | G | 0.85484 | A | 0.14516 | 7 | 0.22581 |
| 10083 | S12_19496732 | 12 | 19496732 | 143 | C | T | C | 0.85484 | T | 0.14516 | 7 | 0.22581 |
| 10084 | S12_19636739 | 12 | 19636739 | 140007 | A | G | A | 0.95161 | G | 0.04839 | 3 | 0.09677 |
| 10085 | S12_20280537 | 12 | 20280537 | 643798 | T | C | T | 0.80645 | C | 0.19355 | 10 | 0.32258 |
| 10086 | S12_20280538 | 12 | 20280538 | 1 | T | C | T | 0.64516 | C | 0.35484 | 12 | 0.3871 |
| 10087 | S12_20280636 | 12 | 20280636 | 98 | C | T | C | 0.72581 | T | 0.27419 | 11 | 0.35484 |
| 10088 | S12_20424795 | 12 | 20424795 | 144159 | G | C | C | 0.53226 | G | 0.46774 | 15 | 0.48387 |
| 10089 | S12_20632701 | 12 | 20632701 | 207906 | C | G | C | 0.70968 | G | 0.29032 | 14 | 0.45161 |
| 10090 | S12_20632745 | 12 | 20632745 | 44 | G | A | G | 0.93548 | A | 0.06452 | 4 | 0.12903 |
| 10091 | S12_20753362 | 12 | 20753362 | 120617 | T | G | T | 0.66129 | G | 0.33871 | 15 | 0.48387 |
| 10092 | S12_20753438 | 12 | 20753438 | 76 | C | T | C | 0.87097 | T | 0.12903 | 8 | 0.25806 |
| 10093 | S12_20938087 | 12 | 20938087 | 184649 | G | A | G | 0.95161 | A | 0.04839 | 3 | 0.09677 |
| 10094 | S12_20938134 | 12 | 20938134 | 47 | A | G | A | 0.95161 | G | 0.04839 | 3 | 0.09677 |
| 10095 | S12_21091468 | 12 | 21091468 | 153334 | C | T | C | 0.93548 | T | 0.06452 | 4 | 0.12903 |
| 10096 | S12_21091530 | 12 | 21091530 | 62 | G | A | G | 0.83871 | A | 0.16129 | 10 | 0.32258 |

| Site number | SNP name | Chromosome | Physical position | Physical distance from the previous SNP | Reference allele | Alternative allele | Major allele | Major allele frequency | Minor allele | Minor allele frequency | Number heterozygous | Heterozygosity |
|---|---|---|---|---|---|---|---|---|---|---|---|---|
| 10097 | S12_21267270 | 12 | 21267270 | 175740 | T | C | T | 0.74194 | C | 0.25806 | 12 | 0.3871 |
| 10098 | S12_21267271 | 12 | 21267271 | 1 | G | A | G | 0.74194 | A | 0.25806 | 12 | 0.3871 |
| 10099 | S12_21267302 | 12 | 21267302 | 31 | T | A | T | 0.74194 | A | 0.25806 | 12 | 0.3871 |
| 10100 | S12_21815116 | 12 | 21815116 | 547814 | C | G | C | 0.67742 | G | 0.32258 | 16 | 0.51613 |
| 10101 | S12_21815237 | 12 | 21815237 | 121 | C | G | C | 0.67742 | G | 0.32258 | 16 | 0.51613 |
| 10102 | S12_22541372 | 12 | 22541372 | 726135 | G | C | G | 0.64516 | C | 0.35484 | 16 | 0.51613 |
| 10103 | S12_22541389 | 12 | 22541389 | 17 | G | C | G | 0.69355 | C | 0.30645 | 13 | 0.41935 |
| 10104 | S12_22541411 | 12 | 22541411 | 22 | C | A | C | 0.64516 | A | 0.35484 | 16 | 0.51613 |
| 10105 | S12_22541440 | 12 | 22541440 | 29 | A | T | A | 0.69355 | T | 0.30645 | 13 | 0.41935 |
| 10106 | S12_22541493 | 12 | 22541493 | 53 | G | C | C | 0.54839 | G | 0.45161 | 18 | 0.58065 |
| 10107 | S12_22541520 | 12 | 22541520 | 27 | G | A | G | 0.64516 | A | 0.35484 | 16 | 0.51613 |
| 10108 | S12_22541532 | 12 | 22541532 | 12 | C | G | G | 0.90323 | C | 0.09677 | 6 | 0.19355 |
| 10109 | S12_22541548 | 12 | 22541548 | 16 | A | C | A | 0.64516 | C | 0.35484 | 16 | 0.51613 |
| 10110 | S12_22542415 | 12 | 22542415 | 867 | A | G | A | 0.64516 | G | 0.35484 | 16 | 0.51613 |
| 10111 | S12_24109950 | 12 | 24109950 | 1567535 | C | G | C | 0.91935 | G | 0.08065 | 5 | 0.16129 |
| 10112 | S12_24403586 | 12 | 24403586 | 293636 | G | T | G | 0.64516 | T | 0.35484 | 18 | 0.58065 |
| 10113 | S12_24514061 | 12 | 24514061 | 110475 | G | T | G | 0.95161 | T | 0.04839 | 3 | 0.09677 |
| 10114 | S12_24514140 | 12 | 24514140 | 79 | C | T | C | 0.95161 | T | 0.04839 | 3 | 0.09677 |
| 10115 | S12_24514283 | 12 | 24514283 | 143 | C | T | C | 0.8871 | T | 0.1129 | 7 | 0.22581 |
| 10116 | S12_25443811 | 12 | 25443811 | 929528 | C | G | G | 0.87097 | C | 0.12903 | 8 | 0.25806 |
| 10117 | S12_25443868 | 12 | 25443868 | 57 | G | A | G | 0.93548 | A | 0.06452 | 4 | 0.12903 |
| 10118 | S12_25443873 | 12 | 25443873 | 5 | G | A | A | 0.87097 | G | 0.12903 | 8 | 0.25806 |
| 10119 | S12_25771005 | 12 | 25771005 | 327132 | G | A | G | 0.66129 | A | 0.33871 | 15 | 0.48387 |
| 10120 | S12_25771014 | 12 | 25771014 | 9 | C | T | C | 0.82258 | T | 0.17742 | 11 | 0.35484 |
| 10121 | S12_25771034 | 12 | 25771034 | 20 | T | C | C | 0.51613 | T | 0.48387 | 20 | 0.64516 |
| 10122 | S12_25771045 | 12 | 25771045 | 11 | T | C | C | 0.51613 | T | 0.48387 | 20 | 0.64516 |
| 10123 | S12_25771049 | 12 | 25771049 | 4 | G | A | A | 0.53226 | G | 0.46774 | 21 | 0.67742 |
| 10124 | S12_25771072 | 12 | 25771072 | 23 | G | A | A | 0.53226 | G | 0.46774 | 19 | 0.6129 |
| 10125 | S12_25771105 | 12 | 25771105 | 33 | T | C | C | 0.53226 | T | 0.46774 | 21 | 0.67742 |
| 10126 | S12_25771106 | 12 | 25771106 | 1 | G | A | G | 0.82258 | A | 0.17742 | 11 | 0.35484 |
| 10127 | S12_25771113 | 12 | 25771113 | 7 | T | G | G | 0.53226 | T | 0.46774 | 21 | 0.67742 |
| 10128 | S12_25771165 | 12 | 25771165 | 52 | C | A | C | 0.82258 | A | 0.17742 | 11 | 0.35484 |
| 10129 | S12_25771175 | 12 | 25771175 | 10 | T | C | C | 0.51613 | T | 0.48387 | 20 | 0.64516 |
| 10130 | S12_25771176 | 12 | 25771176 | 1 | G | C | C | 0.51613 | G | 0.48387 | 20 | 0.64516 |
| 10131 | S12_25771185 | 12 | 25771185 | 9 | C | T | T | 0.51613 | C | 0.48387 | 20 | 0.64516 |
| 10132 | S12_26324812 | 12 | 26324812 | 553627 | G | A | G | 0.64516 | A | 0.35484 | 14 | 0.45161 |
| 10133 | S12_26584586 | 12 | 26584586 | 259774 | C | T | T | 0.59677 | C | 0.40323 | 13 | 0.41935 |
| 10134 | S12_26966638 | 12 | 26966638 | 382052 | C | T | C | 0.85484 | T | 0.14516 | 7 | 0.22581 |
| 10135 | S12_27319788 | 12 | 27319788 | 353150 | G | C | G | 0.83871 | C | 0.16129 | 8 | 0.25806 |
| 10136 | S12_27319863 | 12 | 27319863 | 75 | C | T | C | 0.85484 | T | 0.14516 | 9 | 0.29032 |
| 10137 | S12_27319979 | 12 | 27319979 | 116 | T | C | C | 0.67742 | T | 0.32258 | 12 | 0.3871 |
| 10138 | S12_27464817 | 12 | 27464817 | 144838 | C | T | C | 0.75806 | T | 0.24194 | 11 | 0.35484 |
| 10139 | S12_27464842 | 12 | 27464842 | 25 | A | G | G | 0.56452 | A | 0.43548 | 19 | 0.6129 |
| 10140 | S12_27464917 | 12 | 27464917 | 75 | C | T | C | 0.90323 | T | 0.09677 | 6 | 0.19355 |
| 10141 | S12_27464989 | 12 | 27464989 | 72 | G | C | G | 0.74194 | C | 0.25806 | 12 | 0.3871 |
| 10142 | S12_29591928 | 12 | 29591928 | 2126939 | T | C | C | 0.58065 | T | 0.41935 | 12 | 0.3871 |
| 10143 | S12_29591960 | 12 | 29591960 | 32 | T | C | T | 0.87097 | C | 0.12903 | 4 | 0.12903 |
| 10144 | S12_29592003 | 12 | 29592003 | 43 | G | A | G | 0.74194 | A | 0.25806 | 10 | 0.32258 |
| 10145 | S12_29985716 | 12 | 29985716 | 393713 | A | G | G | 0.67742 | A | 0.32258 | 18 | 0.58065 |
| 10146 | S12_29985825 | 12 | 29985825 | 109 | C | T | C | 0.93548 | T | 0.06452 | 4 | 0.12903 |
| 10147 | S12_30733649 | 12 | 30733649 | 747824 | C | T | C | 0.91935 | T | 0.08065 | 5 | 0.16129 |
| 10148 | S12_30733650 | 12 | 30733650 | 1 | G | A | G | 0.72581 | A | 0.27419 | 11 | 0.35484 |
| 10149 | S12_30845835 | 12 | 30845835 | 112185 | T | C | T | 0.93548 | C | 0.06452 | 4 | 0.12903 |
| 10150 | S12_30845848 | 12 | 30845848 | 13 | G | A | G | 0.6129 | A | 0.3871 | 14 | 0.45161 |

| Site number | SNP name | Chromosome | Physical position | Physical distance from the previous SNP | Reference allele | Alternative allele | Major allele | Major allele frequency | Minor allele | Minor allele frequency | Number heterozygous | Heterozygosity |
|---|---|---|---|---|---|---|---|---|---|---|---|---|
| 10151 | S12_30845849 | 12 | 30845849 | 1 | C | G | C | 0.90323 | G | 0.09677 | 6 | 0.19355 |
| 10152 | S12_30845882 | 12 | 30845882 | 33 | T | C | C | 0.66129 | T | 0.33871 | 15 | 0.48387 |
| 10153 | S12_30845954 | 12 | 30845954 | 72 | A | G | A | 0.85484 | G | 0.14516 | 9 | 0.29032 |
| 10154 | S12_30928247 | 12 | 30928247 | 82293 | T | C | T | 0.93548 | C | 0.06452 | 4 | 0.12903 |
| 10155 | S12_31180131 | 12 | 31180131 | 251884 | C | G | C | 0.87097 | G | 0.12903 | 8 | 0.25806 |
| 10156 | S12_31950995 | 12 | 31950995 | 770864 | T | C | C | 0.58065 | T | 0.41935 | 18 | 0.58065 |
| 10157 | S12_31951055 | 12 | 31951055 | 60 | A | G | A | 0.93548 | G | 0.06452 | 4 | 0.12903 |
| 10158 | S12_32568292 | 12 | 32568292 | 617237 | G | A | G | 0.87097 | A | 0.12903 | 6 | 0.19355 |
| 10159 | S12_32568316 | 12 | 32568316 | 24 | A | G | G | 0.91935 | A | 0.08065 | 5 | 0.16129 |
| 10160 | S12_32769841 | 12 | 32769841 | 201525 | A | G | G | 0.69355 | A | 0.30645 | 15 | 0.48387 |
| 10161 | S12_32769858 | 12 | 32769858 | 17 | C | T | C | 0.83871 | T | 0.16129 | 10 | 0.32258 |
| 10162 | S12_33270698 | 12 | 33270698 | 500840 | A | G | A | 0.91935 | G | 0.08065 | 5 | 0.16129 |
| 10163 | S12_33270712 | 12 | 33270712 | 14 | T | C | T | 0.83871 | C | 0.16129 | 10 | 0.32258 |
| 10164 | S12_33638245 | 12 | 33638245 | 367533 | G | A | G | 0.95161 | A | 0.04839 | 3 | 0.09677 |
| 10165 | S12_34451034 | 12 | 34451034 | 812789 | C | A | C | 0.8871 | A | 0.1129 | 7 | 0.22581 |
| 10166 | S12_34451206 | 12 | 34451206 | 172 | T | C | T | 0.90323 | C | 0.09677 | 6 | 0.19355 |
| 10167 | S12_34534756 | 12 | 34534756 | 83550 | C | T | C | 0.58065 | T | 0.41935 | 14 | 0.45161 |
| 10168 | S12_34534774 | 12 | 34534774 | 18 | T | A | T | 0.58065 | A | 0.41935 | 14 | 0.45161 |
| 10169 | S12_34534784 | 12 | 34534784 | 10 | T | C | T | 0.58065 | C | 0.41935 | 14 | 0.45161 |
| 10170 | S12_34534796 | 12 | 34534796 | 12 | G | A | G | 0.90323 | A | 0.09677 | 6 | 0.19355 |
| 10171 | S12_34534898 | 12 | 34534898 | 102 | C | A | C | 0.58065 | A | 0.41935 | 14 | 0.45161 |
| 10172 | S12_34534910 | 12 | 34534910 | 12 | T | A | T | 0.85484 | A | 0.14516 | 9 | 0.29032 |
| 10173 | S12_35793467 | 12 | 35793467 | 1258557 | C | T | C | 0.82258 | T | 0.17742 | 11 | 0.35484 |
| 10174 | S12_35793509 | 12 | 35793509 | 42 | A | G | G | 0.66129 | A | 0.33871 | 15 | 0.48387 |
| 10175 | S12_35847192 | 12 | 35847192 | 53683 | G | A | G | 0.72581 | A | 0.27419 | 13 | 0.41935 |
| 10176 | S12_35847207 | 12 | 35847207 | 15 | C | T | C | 0.8871 | T | 0.1129 | 7 | 0.22581 |
| 10177 | S12_35847208 | 12 | 35847208 | 1 | G | A | G | 0.87097 | A | 0.12903 | 8 | 0.25806 |
| 10178 | S12_35994135 | 12 | 35994135 | 146927 | G | A | G | 0.93548 | A | 0.06452 | 4 | 0.12903 |
| 10179 | S12_35994182 | 12 | 35994182 | 47 | A | G | A | 0.79032 | G | 0.20968 | 9 | 0.29032 |
| 10180 | S12_35994204 | 12 | 35994204 | 22 | G | T | G | 0.72581 | T | 0.27419 | 9 | 0.29032 |
| 10181 | S12_35994234 | 12 | 35994234 | 30 | C | T | C | 0.5 | T | 0.5 | 15 | 0.48387 |
| 10182 | S12_35994240 | 12 | 35994240 | 6 | A | G | G | 0.77419 | A | 0.22581 | 12 | 0.3871 |
| 10183 | S12_35994314 | 12 | 35994314 | 74 | G | A | A | 0.5 | G | 0.5 | 15 | 0.48387 |
| 10184 | S12_36050617 | 12 | 36050617 | 56303 | A | G | G | 0.74194 | A | 0.25806 | 10 | 0.32258 |
| 10185 | S12_36050758 | 12 | 36050758 | 141 | T | C | T | 0.59677 | C | 0.40323 | 15 | 0.48387 |
| 10186 | S12_36050803 | 12 | 36050803 | 45 | T | C | T | 0.72581 | C | 0.27419 | 13 | 0.41935 |
| 10187 | S12_36478883 | 12 | 36478883 | 428080 | G | A | G | 0.95161 | A | 0.04839 | 3 | 0.09677 |
| 10188 | S12_36492397 | 12 | 36492397 | 13514 | A | G | A | 0.91935 | G | 0.08065 | 5 | 0.16129 |
| 10189 | S12_36619116 | 12 | 36619116 | 126719 | A | G | G | 0.90323 | A | 0.09677 | 4 | 0.12903 |
| 10190 | S12_36640794 | 12 | 36640794 | 21678 | T | A | T | 0.6129 | A | 0.3871 | 16 | 0.51613 |
| 10191 | S12_37166415 | 12 | 37166415 | 525621 | T | C | T | 0.85484 | C | 0.14516 | 9 | 0.29032 |
| 10192 | S12_37970323 | 12 | 37970323 | 803908 | G | C | G | 0.91935 | C | 0.08065 | 5 | 0.16129 |
| 10193 | S12_37970456 | 12 | 37970456 | 133 | A | G | G | 0.8871 | A | 0.1129 | 7 | 0.22581 |
| 10194 | S12_38431298 | 12 | 38431298 | 460842 | C | T | C | 0.95161 | T | 0.04839 | 3 | 0.09677 |
| 10195 | S12_38431461 | 12 | 38431461 | 163 | T | C | T | 0.91935 | C | 0.08065 | 3 | 0.09677 |
| 10196 | S12_38431462 | 12 | 38431462 | 1 | A | T | A | 0.91935 | T | 0.08065 | 3 | 0.09677 |
| 10197 | S12_39385081 | 12 | 39385081 | 953619 | A | G | A | 0.95161 | G | 0.04839 | 1 | 0.03226 |
| 10198 | S12_39421224 | 12 | 39421224 | 36143 | G | A | G | 0.83871 | A | 0.16129 | 8 | 0.25806 |
| 10199 | S12_39421276 | 12 | 39421276 | 52 | T | C | C | 0.8871 | T | 0.1129 | 5 | 0.16129 |
| 10200 | S12_39696923 | 12 | 39696923 | 275647 | C | T | C | 0.95161 | T | 0.04839 | 3 | 0.09677 |
| 10201 | S12_39697039 | 12 | 39697039 | 116 | G | C | G | 0.87097 | C | 0.12903 | 8 | 0.25806 |
| 10202 | S12_39969355 | 12 | 39969355 | 272316 | G | C | G | 0.53226 | C | 0.46774 | 15 | 0.48387 |
| 10203 | S12_39969363 | 12 | 39969363 | 8 | A | G | A | 0.56452 | G | 0.43548 | 17 | 0.54839 |
| 10204 | S12_40046822 | 12 | 40046822 | 77459 | C | A | C | 0.93548 | A | 0.06452 | 4 | 0.12903 |

| Site number | SNP name | Chromosome | Physical position | Physical distance from the previous SNP | Reference allele | Alternative allele | Major allele | Major allele frequency | Minor allele | Minor allele frequency | Number heterozygous | Heterozygosity |
|---|---|---|---|---|---|---|---|---|---|---|---|---|
| 10205 | S12_40046888 | 12 | 40046888 | 66 | C | T | C | 0.72581 | T | 0.27419 | 17 | 0.54839 |
| 10206 | S12_40441637 | 12 | 40441637 | 394749 | G | T | G | 0.79032 | T | 0.20968 | 11 | 0.35484 |
| 10207 | S12_40733684 | 12 | 40733684 | 292047 | G | C | C | 0.54839 | G | 0.45161 | 20 | 0.64516 |
| 10208 | S12_42493314 | 12 | 42493314 | 1759630 | G | A | G | 0.67742 | A | 0.32258 | 18 | 0.58065 |
| 10209 | S12_42493369 | 12 | 42493369 | 55 | A | C | A | 0.93548 | C | 0.06452 | 4 | 0.12903 |
| 10210 | S12_42787505 | 12 | 42787505 | 294136 | C | T | C | 0.77419 | T | 0.22581 | 14 | 0.45161 |
| 10211 | S12_42787587 | 12 | 42787587 | 82 | T | A | T | 0.91935 | A | 0.08065 | 5 | 0.16129 |
| 10212 | S12_42849456 | 12 | 42849456 | 61869 | A | C | A | 0.83871 | C | 0.16129 | 8 | 0.25806 |
| 10213 | S12_42849502 | 12 | 42849502 | 46 | C | G | G | 0.74194 | C | 0.25806 | 10 | 0.32258 |
| 10214 | S12_45148763 | 12 | 45148763 | 2299261 | G | T | G | 0.79032 | T | 0.20968 | 7 | 0.22581 |
| 10215 | S12_45148938 | 12 | 45148938 | 175 | G | A | G | 0.93548 | A | 0.06452 | 4 | 0.12903 |
| 10216 | S12_46111129 | 12 | 46111129 | 962191 | C | T | C | 0.66129 | T | 0.33871 | 11 | 0.35484 |
| 10217 | S12_46775176 | 12 | 46775176 | 664047 | C | T | C | 0.91935 | T | 0.08065 | 5 | 0.16129 |
| 10218 | S12_46775228 | 12 | 46775228 | 52 | T | C | C | 0.59677 | T | 0.40323 | 19 | 0.6129 |
| 10219 | S12_46775269 | 12 | 46775269 | 41 | C | A | C | 0.90323 | A | 0.09677 | 6 | 0.19355 |
| 10220 | S12_46996803 | 12 | 46996803 | 221534 | A | G | A | 0.90323 | G | 0.09677 | 4 | 0.12903 |
| 10221 | S12_47963532 | 12 | 47963532 | 966729 | T | A | T | 0.91935 | A | 0.08065 | 5 | 0.16129 |
| 10222 | S12_49074614 | 12 | 49074614 | 1111082 | G | C | G | 0.72581 | C | 0.27419 | 11 | 0.35484 |
| 10223 | S12_49919472 | 12 | 49919472 | 844858 | A | G | A | 0.69355 | G | 0.30645 | 13 | 0.41935 |
| 10224 | S12_50062727 | 12 | 50062727 | 143255 | T | C | T | 0.95161 | C | 0.04839 | 3 | 0.09677 |
| 10225 | S12_50062763 | 12 | 50062763 | 36 | A | G | A | 0.8871 | G | 0.1129 | 5 | 0.16129 |
| 10226 | S12_50416540 | 12 | 50416540 | 353777 | C | T | C | 0.93548 | T | 0.06452 | 4 | 0.12903 |
| 10227 | S12_50416652 | 12 | 50416652 | 112 | G | A | G | 0.93548 | A | 0.06452 | 4 | 0.12903 |
| 10228 | S12_50416705 | 12 | 50416705 | 53 | G | C | G | 0.74194 | C | 0.25806 | 8 | 0.25806 |
| 10229 | S12_50656376 | 12 | 50656376 | 239671 | G | A | G | 0.93548 | A | 0.06452 | 4 | 0.12903 |
| 10230 | S12_51375113 | 12 | 51375113 | 718737 | T | C | T | 0.69355 | C | 0.30645 | 9 | 0.29032 |
| 10231 | S12_51375273 | 12 | 51375273 | 160 | C | A | C | 0.82258 | A | 0.17742 | 7 | 0.22581 |
| 10232 | S12_51375274 | 12 | 51375274 | 1 | G | A | G | 0.8871 | A | 0.1129 | 7 | 0.22581 |
| 10233 | S12_51453099 | 12 | 51453099 | 77825 | C | A | C | 0.67742 | A | 0.32258 | 16 | 0.51613 |
| 10234 | S12_51608035 | 12 | 51608035 | 154936 | T | C | T | 0.90323 | C | 0.09677 | 6 | 0.19355 |
| 10235 | S12_51608218 | 12 | 51608218 | 183 | C | T | C | 0.62903 | T | 0.37097 | 15 | 0.48387 |
| 10236 | S12_51792792 | 12 | 51792792 | 184574 | C | T | C | 0.79032 | T | 0.20968 | 9 | 0.29032 |
| 10237 | S12_51792881 | 12 | 51792881 | 89 | A | G | A | 0.69355 | G | 0.30645 | 13 | 0.41935 |
| 10238 | S12_51915855 | 12 | 51915855 | 122974 | G | A | G | 0.93548 | A | 0.06452 | 4 | 0.12903 |
| 10239 | S12_51916047 | 12 | 51916047 | 192 | C | T | C | 0.91935 | T | 0.08065 | 5 | 0.16129 |
| 10240 | S12_51928811 | 12 | 51928811 | 12764 | A | T | T | 0.90323 | A | 0.09677 | 6 | 0.19355 |
| 10241 | S12_51952488 | 12 | 51952488 | 23677 | G | C | C | 0.72581 | G | 0.27419 | 11 | 0.35484 |
| 10242 | S12_51952551 | 12 | 51952551 | 63 | A | G | A | 0.77419 | G | 0.22581 | 10 | 0.32258 |
| 10243 | S12_52125719 | 12 | 52125719 | 173168 | C | G | C | 0.8871 | G | 0.1129 | 7 | 0.22581 |
| 10244 | S12_52228237 | 12 | 52228237 | 102518 | C | T | C | 0.83871 | T | 0.16129 | 10 | 0.32258 |
| 10245 | S12_52247707 | 12 | 52247707 | 19470 | T | C | T | 0.74194 | C | 0.25806 | 12 | 0.3871 |
| 10246 | S12_52344616 | 12 | 52344616 | 96909 | G | T | G | 0.83871 | T | 0.16129 | 8 | 0.25806 |
| 10247 | S12_52344825 | 12 | 52344825 | 209 | G | A | G | 0.91935 | A | 0.08065 | 5 | 0.16129 |
| 10248 | S12_52507020 | 12 | 52507020 | 162195 | A | G | A | 0.95161 | G | 0.04839 | 3 | 0.09677 |
| 10249 | S12_52727178 | 12 | 52727178 | 220158 | G | A | G | 0.74194 | A | 0.25806 | 10 | 0.32258 |
| 10250 | S12_52819407 | 12 | 52819407 | 92229 | A | G | A | 0.91935 | G | 0.08065 | 5 | 0.16129 |
| 10251 | S12_52819521 | 12 | 52819521 | 114 | G | A | G | 0.91935 | A | 0.08065 | 5 | 0.16129 |
| 10252 | S12_52998189 | 12 | 52998189 | 178668 | C | T | C | 0.82258 | T | 0.17742 | 9 | 0.29032 |
| 10253 | S12_52998260 | 12 | 52998260 | 71 | A | G | G | 0.83871 | A | 0.16129 | 10 | 0.32258 |
| 10254 | S12_52998273 | 12 | 52998273 | 13 | C | T | C | 0.95161 | T | 0.04839 | 3 | 0.09677 |
| 10255 | S12_52998311 | 12 | 52998311 | 38 | C | G | G | 0.83871 | C | 0.16129 | 10 | 0.32258 |
| 10256 | S12_52998334 | 12 | 52998334 | 23 | C | T | C | 0.8871 | T | 0.1129 | 7 | 0.22581 |
| 10257 | S12_53016138 | 12 | 53016138 | 17804 | A | G | G | 0.69355 | A | 0.30645 | 15 | 0.48387 |
| 10258 | S12_53016173 | 12 | 53016173 | 35 | C | T | T | 0.59677 | C | 0.40323 | 19 | 0.6129 |

| Site number | SNP name | Chromosome | Physical position | Physical distance from the previous SNP | Reference allele | Alternative allele | Major allele | Major allele frequency | Minor allele | Minor allele frequency | Number heterozygous | Heterozygosity |
|---|---|---|---|---|---|---|---|---|---|---|---|---|
| 10259 | S12_53209016 | 12 | 53209016 | 192843 | A | G | A | 0.8871 | G | 0.1129 | 7 | 0.22581 |
| 10260 | S12_53209081 | 12 | 53209081 | 65 | G | A | G | 0.95161 | A | 0.04839 | 3 | 0.09677 |
| 10261 | S12_53296484 | 12 | 53296484 | 87403 | C | T | C | 0.91935 | T | 0.08065 | 5 | 0.16129 |
| 10262 | S12_53296499 | 12 | 53296499 | 15 | C | T | C | 0.93548 | T | 0.06452 | 4 | 0.12903 |
| 10263 | S12_53296512 | 12 | 53296512 | 13 | C | T | C | 0.91935 | T | 0.08065 | 5 | 0.16129 |
| 10264 | S12_53784330 | 12 | 53784330 | 487818 | A | G | A | 0.95161 | G | 0.04839 | 3 | 0.09677 |
| 10265 | S12_53784474 | 12 | 53784474 | 144 | C | T | C | 0.95161 | T | 0.04839 | 3 | 0.09677 |
| 10266 | S12_53784522 | 12 | 53784522 | 48 | G | A | G | 0.91935 | A | 0.08065 | 3 | 0.09677 |
| 10267 | S12_53863070 | 12 | 53863070 | 78548 | C | A | C | 0.83871 | A | 0.16129 | 6 | 0.19355 |
| 10268 | S12_54060205 | 12 | 54060205 | 197135 | C | T | C | 0.90323 | T | 0.09677 | 6 | 0.19355 |
| 10269 | S12_54154993 | 12 | 54154993 | 94788 | A | G | A | 0.70968 | G | 0.29032 | 14 | 0.45161 |
| 10270 | S12_54155019 | 12 | 54155019 | 26 | G | A | G | 0.70968 | A | 0.29032 | 14 | 0.45161 |
| 10271 | S12_54155069 | 12 | 54155069 | 50 | T | C | T | 0.67742 | C | 0.32258 | 14 | 0.45161 |
| 10272 | S12_54155106 | 12 | 54155106 | 37 | T | C | T | 0.70968 | C | 0.29032 | 14 | 0.45161 |
| 10273 | S12_54277127 | 12 | 54277127 | 122021 | A | G | A | 0.58065 | G | 0.41935 | 18 | 0.58065 |
| 10274 | S12_54306195 | 12 | 54306195 | 29068 | C | T | C | 0.87097 | T | 0.12903 | 6 | 0.19355 |
| 10275 | S12_54352566 | 12 | 54352566 | 46371 | T | C | T | 0.95161 | C | 0.04839 | 1 | 0.03226 |
| 10276 | S12_54352602 | 12 | 54352602 | 36 | G | A | G | 0.75806 | A | 0.24194 | 11 | 0.35484 |
| 10277 | S12_54352654 | 12 | 54352654 | 52 | C | A | C | 0.80645 | A | 0.19355 | 12 | 0.3871 |
| 10278 | S12_54352756 | 12 | 54352756 | 102 | G | C | G | 0.85484 | C | 0.14516 | 9 | 0.29032 |
| 10279 | S12_54643381 | 12 | 54643381 | 290625 | C | T | T | 0.62903 | C | 0.37097 | 17 | 0.54839 |
| 10280 | S12_54947315 | 12 | 54947315 | 303934 | G | A | G | 0.62903 | A | 0.37097 | 17 | 0.54839 |
| 10281 | S12_54947430 | 12 | 54947430 | 115 | G | A | G | 0.85484 | A | 0.14516 | 9 | 0.29032 |
| 10282 | S12_54996592 | 12 | 54996592 | 49162 | G | A | G | 0.67742 | A | 0.32258 | 14 | 0.45161 |
| 10283 | S12_55278771 | 12 | 55278771 | 282179 | G | A | G | 0.75806 | A | 0.24194 | 13 | 0.41935 |
| 10284 | S12_55418409 | 12 | 55418409 | 139638 | C | T | C | 0.80645 | T | 0.19355 | 12 | 0.3871 |
| 10285 | S12_55433450 | 12 | 55433450 | 15041 | G | A | G | 0.95161 | A | 0.04839 | 3 | 0.09677 |
| 10286 | S12_55442513 | 12 | 55442513 | 9063 | C | T | C | 0.75806 | T | 0.24194 | 9 | 0.29032 |
| 10287 | S12_55695184 | 12 | 55695184 | 252671 | T | C | T | 0.74194 | C | 0.25806 | 12 | 0.3871 |
| 10288 | S12_55695405 | 12 | 55695405 | 221 | T | G | T | 0.90323 | G | 0.09677 | 6 | 0.19355 |
| 10289 | S12_55777718 | 12 | 55777718 | 82313 | C | T | C | 0.53226 | T | 0.46774 | 17 | 0.54839 |
| 10290 | S12_55777757 | 12 | 55777757 | 39 | A | C | C | 0.59677 | A | 0.40323 | 15 | 0.48387 |
| 10291 | S12_55777796 | 12 | 55777796 | 39 | C | T | T | 0.59677 | C | 0.40323 | 15 | 0.48387 |
| 10292 | S12_55799961 | 12 | 55799961 | 22165 | T | C | T | 0.91935 | C | 0.08065 | 5 | 0.16129 |
| 10293 | S12_55799977 | 12 | 55799977 | 16 | A | T | A | 0.66129 | T | 0.33871 | 11 | 0.35484 |
| 10294 | S12_55800090 | 12 | 55800090 | 113 | A | G | A | 0.72581 | G | 0.27419 | 9 | 0.29032 |
| 10295 | S12_55802352 | 12 | 55802352 | 2262 | C | G | C | 0.74194 | G | 0.25806 | 10 | 0.32258 |
| 10296 | S12_55802485 | 12 | 55802485 | 133 | G | A | G | 0.59677 | A | 0.40323 | 13 | 0.41935 |
| 10297 | S12_55853803 | 12 | 55853803 | 51318 | G | T | G | 0.8871 | T | 0.1129 | 7 | 0.22581 |
| 10298 | S12_55853826 | 12 | 55853826 | 23 | C | G | C | 0.82258 | G | 0.17742 | 7 | 0.22581 |
| 10299 | S12_55853929 | 12 | 55853929 | 103 | G | C | G | 0.79032 | C | 0.20968 | 9 | 0.29032 |
| 10300 | S12_55853939 | 12 | 55853939 | 10 | G | C | G | 0.67742 | C | 0.32258 | 12 | 0.3871 |
| 10301 | S12_55919429 | 12 | 55919429 | 65490 | C | T | C | 0.87097 | T | 0.12903 | 6 | 0.19355 |
| 10302 | S12_55919453 | 12 | 55919453 | 24 | C | T | C | 0.75806 | T | 0.24194 | 13 | 0.41935 |
| 10303 | S12_55919564 | 12 | 55919564 | 111 | C | T | C | 0.93548 | T | 0.06452 | 4 | 0.12903 |
| 10304 | S12_56092571 | 12 | 56092571 | 173007 | T | A | T | 0.8871 | A | 0.1129 | 5 | 0.16129 |
| 10305 | S12_56450592 | 12 | 56450592 | 358021 | C | T | C | 0.79032 | T | 0.20968 | 13 | 0.41935 |
| 10306 | S12_56450798 | 12 | 56450798 | 206 | C | A | A | 0.93548 | C | 0.06452 | 4 | 0.12903 |
| 10307 | S12_56512426 | 12 | 56512426 | 61628 | A | G | G | 0.91935 | A | 0.08065 | 5 | 0.16129 |
| 10308 | S12_56548456 | 12 | 56548456 | 36030 | G | T | G | 0.79032 | T | 0.20968 | 13 | 0.41935 |
| 10309 | S12_56729685 | 12 | 56729685 | 181229 | G | A | G | 0.83871 | A | 0.16129 | 6 | 0.19355 |
| 10310 | S12_56820101 | 12 | 56820101 | 90416 | T | C | T | 0.70968 | C | 0.29032 | 10 | 0.32258 |
| 10311 | S12_56820110 | 12 | 56820110 | 9 | A | G | A | 0.75806 | G | 0.24194 | 9 | 0.29032 |
| 10312 | S12_57373234 | 12 | 57373234 | 553124 | A | T | A | 0.8871 | T | 0.1129 | 7 | 0.22581 |

| Site number | SNP name | Chromosome | Physical position | Physical distance from the previous SNP | Reference allele | Alternative allele | Major allele | Major allele frequency | Minor allele | Minor allele frequency | Number heterozygous | Heterozygosity |
|---|---|---|---|---|---|---|---|---|---|---|---|---|
| 10313 | S12_57529065 | 12 | 57529065 | 155831 | C | T | C | 0.5 | T | 0.5 | 17 | 0.54839 |
| 10314 | S12_57656689 | 12 | 57656689 | 127624 | A | C | C | 0.69355 | A | 0.30645 | 9 | 0.29032 |
| 10315 | S12_57797164 | 12 | 57797164 | 140475 | T | C | T | 0.80645 | C | 0.19355 | 8 | 0.25806 |
| 10316 | S12_57797198 | 12 | 57797198 | 34 | G | A | G | 0.8871 | A | 0.1129 | 5 | 0.16129 |
| 10317 | S12_57797227 | 12 | 57797227 | 29 | C | T | C | 0.8871 | T | 0.1129 | 5 | 0.16129 |
| 10318 | S12_57889364 | 12 | 57889364 | 92137 | G | A | A | 0.53226 | G | 0.46774 | 13 | 0.41935 |
| 10319 | S12_57889472 | 12 | 57889472 | 108 | A | G | G | 0.53226 | A | 0.46774 | 13 | 0.41935 |
| 10320 | S12_57889474 | 12 | 57889474 | 2 | A | G | G | 0.51613 | A | 0.48387 | 12 | 0.3871 |
| 10321 | S12_57889482 | 12 | 57889482 | 8 | T | C | C | 0.51613 | T | 0.48387 | 12 | 0.3871 |
| 10322 | S12_57979680 | 12 | 57979680 | 90198 | T | C | T | 0.74194 | C | 0.25806 | 12 | 0.3871 |
| 10323 | S12_58193131 | 12 | 58193131 | 213451 | T | G | T | 0.95161 | G | 0.04839 | 3 | 0.09677 |
| 10324 | S12_58898368 | 12 | 58898368 | 705237 | C | T | T | 0.85484 | C | 0.14516 | 5 | 0.16129 |
| 10325 | S12_58902003 | 12 | 58902003 | 3635 | T | C | T | 0.83871 | C | 0.16129 | 6 | 0.19355 |
| 10326 | S12_59190028 | 12 | 59190028 | 288025 | C | T | C | 0.74194 | T | 0.25806 | 10 | 0.32258 |
| 10327 | S12_59258881 | 12 | 59258881 | 68853 | C | T | T | 0.53226 | C | 0.46774 | 15 | 0.48387 |
| 10328 | S12_59663287 | 12 | 59663287 | 404406 | T | C | T | 0.93548 | C | 0.06452 | 4 | 0.12903 |
| 10329 | S12_59663297 | 12 | 59663297 | 10 | T | C | T | 0.87097 | C | 0.12903 | 6 | 0.19355 |
| 10330 | S12_59663315 | 12 | 59663315 | 18 | T | C | T | 0.67742 | C | 0.32258 | 12 | 0.3871 |
| 10331 | S12_59663317 | 12 | 59663317 | 2 | C | T | C | 0.87097 | T | 0.12903 | 6 | 0.19355 |
| 10332 | S12_59663324 | 12 | 59663324 | 7 | G | A | G | 0.87097 | A | 0.12903 | 6 | 0.19355 |
| 10333 | S12_59663327 | 12 | 59663327 | 3 | G | C | G | 0.93548 | C | 0.06452 | 4 | 0.12903 |
| 10334 | S12_59663417 | 12 | 59663417 | 90 | C | T | C | 0.82258 | T | 0.17742 | 7 | 0.22581 |
| 10335 | S12_59663456 | 12 | 59663456 | 39 | A | G | A | 0.93548 | G | 0.06452 | 4 | 0.12903 |
| 10336 | S12_59676715 | 12 | 59676715 | 13259 | T | C | T | 0.8871 | C | 0.1129 | 5 | 0.16129 |
| 10337 | S12_59676741 | 12 | 59676741 | 26 | C | T | C | 0.93548 | T | 0.06452 | 4 | 0.12903 |
| 10338 | S12_60167442 | 12 | 60167442 | 490701 | C | A | C | 0.95161 | A | 0.04839 | 1 | 0.03226 |
| 10339 | S12_60911934 | 12 | 60911934 | 744492 | T | C | T | 0.6129 | C | 0.3871 | 16 | 0.51613 |
| 10340 | S12_60915974 | 12 | 60915974 | 4040 | C | T | C | 0.77419 | T | 0.22581 | 14 | 0.45161 |
| 10341 | S12_61287146 | 12 | 61287146 | 371172 | T | C | T | 0.93548 | C | 0.06452 | 2 | 0.06452 |
| 10342 | S12_61298135 | 12 | 61298135 | 10989 | G | A | G | 0.59677 | A | 0.40323 | 11 | 0.35484 |
| 10343 | S12_61298175 | 12 | 61298175 | 40 | A | G | A | 0.93548 | G | 0.06452 | 2 | 0.06452 |
| 10344 | S12_61298360 | 12 | 61298360 | 185 | A | G | A | 0.66129 | G | 0.33871 | 11 | 0.35484 |
| 10345 | S12_61385471 | 12 | 61385471 | 87111 | A | G | A | 0.91935 | G | 0.08065 | 5 | 0.16129 |
| 10346 | S12_61385497 | 12 | 61385497 | 26 | G | A | G | 0.77419 | A | 0.22581 | 10 | 0.32258 |
| 10347 | S12_61385565 | 12 | 61385565 | 68 | C | T | C | 0.87097 | T | 0.12903 | 8 | 0.25806 |
| 10348 | S12_61602786 | 12 | 61602786 | 217221 | G | A | G | 0.93548 | A | 0.06452 | 4 | 0.12903 |
| 10349 | S12_61602800 | 12 | 61602800 | 14 | C | T | C | 0.82258 | T | 0.17742 | 11 | 0.35484 |
| 10350 | S12_61689384 | 12 | 61689384 | 86584 | A | G | A | 0.56452 | G | 0.43548 | 15 | 0.48387 |
| 10351 | S12_61689471 | 12 | 61689471 | 87 | G | C | G | 0.77419 | C | 0.22581 | 8 | 0.25806 |
| 10352 | S12_61689478 | 12 | 61689478 | 7 | G | A | G | 0.56452 | A | 0.43548 | 15 | 0.48387 |
| 10353 | S12_61689623 | 12 | 61689623 | 145 | G | T | T | 0.91935 | G | 0.08065 | 5 | 0.16129 |
| 10354 | S12_62067706 | 12 | 62067706 | 378083 | C | T | T | 0.85484 | C | 0.14516 | 5 | 0.16129 |
| 10355 | S12_62067792 | 12 | 62067792 | 86 | C | G | C | 0.85484 | G | 0.14516 | 7 | 0.22581 |
| 10356 | S12_62394763 | 12 | 62394763 | 326971 | A | G | A | 0.87097 | G | 0.12903 | 6 | 0.19355 |
| 10357 | S12_62394883 | 12 | 62394883 | 120 | A | G | A | 0.87097 | G | 0.12903 | 6 | 0.19355 |
| 10358 | S12_62394937 | 12 | 62394937 | 54 | C | T | C | 0.93548 | T | 0.06452 | 2 | 0.06452 |
| 10359 | S12_62636847 | 12 | 62636847 | 241910 | G | T | T | 0.85484 | G | 0.14516 | 7 | 0.22581 |
| 10360 | S12_62636872 | 12 | 62636872 | 25 | A | G | A | 0.69355 | G | 0.30645 | 11 | 0.35484 |
| 10361 | S12_62637010 | 12 | 62637010 | 138 | C | T | C | 0.69355 | T | 0.30645 | 11 | 0.35484 |
| 10362 | S12_62637053 | 12 | 62637053 | 43 | A | G | G | 0.54839 | A | 0.45161 | 16 | 0.51613 |
| 10363 | S12_63065887 | 12 | 63065887 | 428834 | G | A | G | 0.85484 | A | 0.14516 | 5 | 0.16129 |
| 10364 | S12_63065931 | 12 | 63065931 | 44 | C | A | C | 0.93548 | A | 0.06452 | 4 | 0.12903 |
| 10365 | S12_63219204 | 12 | 63219204 | 153273 | G | A | G | 0.90323 | A | 0.09677 | 6 | 0.19355 |
| 10366 | S12_63219542 | 12 | 63219542 | 338 | G | C | G | 0.85484 | C | 0.14516 | 9 | 0.29032 |

| Site number | SNP name | Chromosome | Physical position | Physical distance from the previous SNP | Reference allele | Alternative allele | Major allele | Major allele frequency | Minor allele | Minor allele frequency | Number heterozygous | Heterozygosity |
|---|---|---|---|---|---|---|---|---|---|---|---|---|
| 10367 | S12_63252071 | 12 | 63252071 | 32529 | T | C | T | 0.58065 | C | 0.41935 | 10 | 0.32258 |
| 10368 | S12_63252099 | 12 | 63252099 | 28 | A | G | A | 0.87097 | G | 0.12903 | 8 | 0.25806 |
| 10369 | S12_63279252 | 12 | 63279252 | 27153 | G | A | G | 0.95161 | A | 0.04839 | 1 | 0.03226 |
| 10370 | S12_63628337 | 12 | 63628337 | 349085 | T | C | T | 0.79032 | C | 0.20968 | 9 | 0.29032 |
| 10371 | S12_63628381 | 12 | 63628381 | 44 | G | A | G | 0.95161 | A | 0.04839 | 3 | 0.09677 |
| 10372 | S12_63628388 | 12 | 63628388 | 7 | A | G | A | 0.79032 | G | 0.20968 | 9 | 0.29032 |
| 10373 | S12_63736817 | 12 | 63736817 | 108429 | C | T | C | 0.80645 | T | 0.19355 | 10 | 0.32258 |
| 10374 | S12_63736841 | 12 | 63736841 | 24 | G | A | G | 0.54839 | A | 0.45161 | 20 | 0.64516 |
| 10375 | S12_64348871 | 12 | 64348871 | 612030 | T | C | T | 0.8871 | C | 0.1129 | 5 | 0.16129 |
| 10376 | S12_64348899 | 12 | 64348899 | 28 | A | G | A | 0.8871 | G | 0.1129 | 5 | 0.16129 |
| 10377 | S12_64348960 | 12 | 64348960 | 61 | G | A | G | 0.80645 | A | 0.19355 | 12 | 0.3871 |
| 10378 | S12_64348969 | 12 | 64348969 | 9 | G | A | G | 0.8871 | A | 0.1129 | 5 | 0.16129 |
| 10379 | S12_64403784 | 12 | 64403784 | 54815 | G | T | G | 0.82258 | T | 0.17742 | 9 | 0.29032 |
| 10380 | S12_64483040 | 12 | 64483040 | 79256 | A | G | A | 0.67742 | G | 0.32258 | 14 | 0.45161 |
| 10381 | S12_64483091 | 12 | 64483091 | 51 | A | G | A | 0.53226 | G | 0.46774 | 19 | 0.6129 |
| 10382 | S12_65050809 | 12 | 65050809 | 567718 | G | A | G | 0.69355 | A | 0.30645 | 17 | 0.54839 |
| 10383 | S12_65050855 | 12 | 65050855 | 46 | G | A | G | 0.69355 | A | 0.30645 | 17 | 0.54839 |
| 10384 | S12_65050928 | 12 | 65050928 | 73 | G | A | G | 0.93548 | A | 0.06452 | 2 | 0.06452 |
| 10385 | S12_65428165 | 12 | 65428165 | 377237 | T | G | T | 0.59677 | G | 0.40323 | 13 | 0.41935 |
| 10386 | S12_65428326 | 12 | 65428326 | 161 | G | A | G | 0.70968 | A | 0.29032 | 14 | 0.45161 |
| 10387 | S12_65604484 | 12 | 65604484 | 176158 | T | C | C | 0.5 | T | 0.5 | 11 | 0.35484 |
| 10388 | S12_65604539 | 12 | 65604539 | 55 | G | A | A | 0.5 | G | 0.5 | 11 | 0.35484 |
| 10389 | S12_65961556 | 12 | 65961556 | 357017 | C | A | A | 0.59677 | C | 0.40323 | 13 | 0.41935 |
| 10390 | S12_65961557 | 12 | 65961557 | 1 | A | G | G | 0.93548 | A | 0.06452 | 4 | 0.12903 |
| 10391 | S12_65961612 | 12 | 65961612 | 55 | T | C | C | 0.93548 | T | 0.06452 | 4 | 0.12903 |
| 10392 | S12_65961614 | 12 | 65961614 | 2 | C | T | C | 0.66129 | T | 0.33871 | 13 | 0.41935 |
| 10393 | S12_65961680 | 12 | 65961680 | 66 | C | T | C | 0.93548 | T | 0.06452 | 4 | 0.12903 |
| 10394 | S12_66054269 | 12 | 66054269 | 92589 | C | T | C | 0.93548 | T | 0.06452 | 4 | 0.12903 |
| 10395 | S12_66054297 | 12 | 66054297 | 28 | C | T | C | 0.95161 | T | 0.04839 | 3 | 0.09677 |
| 10396 | S12_66075163 | 12 | 66075163 | 20866 | C | T | C | 0.95161 | T | 0.04839 | 3 | 0.09677 |
| 10397 | S12_66685057 | 12 | 66685057 | 609894 | C | T | C | 0.75806 | T | 0.24194 | 13 | 0.41935 |
| 10398 | S12_67887622 | 12 | 67887622 | 1202565 | G | T | G | 0.90323 | T | 0.09677 | 6 | 0.19355 |
| 10399 | S12_67962855 | 12 | 67962855 | 75233 | T | C | C | 0.51613 | T | 0.48387 | 14 | 0.45161 |
| 10400 | S12_67979935 | 12 | 67979935 | 17080 | C | T | C | 0.95161 | T | 0.04839 | 3 | 0.09677 |
| 10401 | S12_67980095 | 12 | 67980095 | 160 | T | G | G | 0.77419 | T | 0.22581 | 12 | 0.3871 |
| 10402 | S12_68108502 | 12 | 68108502 | 128407 | G | A | G | 0.72581 | A | 0.27419 | 11 | 0.35484 |
| 10403 | S12_68108529 | 12 | 68108529 | 27 | G | C | G | 0.75806 | C | 0.24194 | 9 | 0.29032 |
| 10404 | S12_68147494 | 12 | 68147494 | 38965 | G | A | G | 0.69355 | A | 0.30645 | 11 | 0.35484 |
| 10405 | S12_68397731 | 12 | 68397731 | 250237 | G | A | G | 0.95161 | A | 0.04839 | 3 | 0.09677 |
| 10406 | S12_68397800 | 12 | 68397800 | 69 | T | G | T | 0.80645 | G | 0.19355 | 10 | 0.32258 |
| 10407 | S12_68634583 | 12 | 68634583 | 236783 | G | A | A | 0.74194 | G | 0.25806 | 10 | 0.32258 |
| 10408 | S12_68634599 | 12 | 68634599 | 16 | T | C | C | 0.74194 | T | 0.25806 | 10 | 0.32258 |
| 10409 | S12_68684047 | 12 | 68684047 | 49448 | C | G | C | 0.90323 | G | 0.09677 | 6 | 0.19355 |
| 10410 | S12_68684181 | 12 | 68684181 | 134 | G | T | G | 0.80645 | T | 0.19355 | 8 | 0.25806 |
| 10411 | S12_68684234 | 12 | 68684234 | 53 | T | G | T | 0.90323 | G | 0.09677 | 6 | 0.19355 |
| 10412 | S12_68743913 | 12 | 68743913 | 59679 | G | T | G | 0.8871 | T | 0.1129 | 7 | 0.22581 |
| 10413 | S12_69435062 | 12 | 69435062 | 691149 | A | C | A | 0.85484 | C | 0.14516 | 7 | 0.22581 |
| 10414 | S12_69435231 | 12 | 69435231 | 169 | A | C | A | 0.91935 | C | 0.08065 | 5 | 0.16129 |
| 10415 | S12_69509285 | 12 | 69509285 | 74054 | G | A | G | 0.95161 | A | 0.04839 | 3 | 0.09677 |
| 10416 | S12_69509289 | 12 | 69509289 | 4 | A | G | A | 0.95161 | G | 0.04839 | 1 | 0.03226 |
| 10417 | S12_69509337 | 12 | 69509337 | 48 | C | T | C | 0.95161 | T | 0.04839 | 1 | 0.03226 |
| 10418 | S12_69614878 | 12 | 69614878 | 105541 | C | T | C | 0.54839 | T | 0.45161 | 12 | 0.3871 |
| 10419 | S12_69614879 | 12 | 69614879 | 1 | G | A | G | 0.70968 | A | 0.29032 | 10 | 0.32258 |
| 10420 | S12_69614898 | 12 | 69614898 | 19 | C | T | C | 0.87097 | T | 0.12903 | 8 | 0.25806 |

| Site number | SNP name | Chromosome | Physical position | Physical distance from the previous SNP | Reference allele | Alternative allele | Major allele | Major allele frequency | Minor allele | Minor allele frequency | Number heterozygous | Heterozygosity |
|---|---|---|---|---|---|---|---|---|---|---|---|---|
| 10421 | S12_69615022 | 12 | 69615022 | 124 | C | T | C | 0.87097 | T | 0.12903 | 8 | 0.25806 |
| 10422 | S12_69615023 | 12 | 69615023 | 1 | A | G | A | 0.72581 | G | 0.27419 | 9 | 0.29032 |
| 10423 | S12_69615049 | 12 | 69615049 | 26 | C | T | C | 0.85484 | T | 0.14516 | 9 | 0.29032 |
| 10424 | S12_69633048 | 12 | 69633048 | 17999 | T | A | T | 0.93548 | A | 0.06452 | 4 | 0.12903 |
| 10425 | S12_69862537 | 12 | 69862537 | 229489 | A | G | A | 0.59677 | G | 0.40323 | 21 | 0.67742 |
| 10426 | S12_69862566 | 12 | 69862566 | 29 | C | G | C | 0.80645 | G | 0.19355 | 8 | 0.25806 |
| 10427 | S12_69898383 | 12 | 69898383 | 35817 | C | T | C | 0.75806 | T | 0.24194 | 9 | 0.29032 |
| 10428 | S12_69898505 | 12 | 69898505 | 122 | C | T | C | 0.75806 | T | 0.24194 | 9 | 0.29032 |
| 10429 | S12_70311990 | 12 | 70311990 | 413485 | C | G | C | 0.83871 | G | 0.16129 | 10 | 0.32258 |
| 10430 | S12_70312000 | 12 | 70312000 | 10 | C | T | C | 0.72581 | T | 0.27419 | 13 | 0.41935 |
| 10431 | S12_70312025 | 12 | 70312025 | 25 | C | T | C | 0.72581 | T | 0.27419 | 15 | 0.48387 |
| 10432 | S12_70312059 | 12 | 70312059 | 34 | T | G | T | 0.83871 | G | 0.16129 | 8 | 0.25806 |
| 10433 | S12_70365016 | 12 | 70365016 | 52957 | G | A | G | 0.87097 | A | 0.12903 | 4 | 0.12903 |
| 10434 | S12_70562742 | 12 | 70562742 | 197726 | T | C | T | 0.58065 | C | 0.41935 | 16 | 0.51613 |
| 10435 | S12_70562843 | 12 | 70562843 | 101 | G | A | G | 0.58065 | A | 0.41935 | 16 | 0.51613 |
| 10436 | S12_70669668 | 12 | 70669668 | 106825 | T | C | T | 0.90323 | C | 0.09677 | 4 | 0.12903 |
| 10437 | S12_70669669 | 12 | 70669669 | 1 | G | A | G | 0.93548 | A | 0.06452 | 2 | 0.06452 |
| 10438 | S12_70819698 | 12 | 70819698 | 150029 | G | A | G | 0.91935 | A | 0.08065 | 3 | 0.09677 |
| 10439 | S12_70819701 | 12 | 70819701 | 3 | T | C | T | 0.90323 | C | 0.09677 | 6 | 0.19355 |
| 10440 | S12_70884298 | 12 | 70884298 | 64597 | G | A | G | 0.91935 | A | 0.08065 | 5 | 0.16129 |
| 10441 | S12_70884305 | 12 | 70884305 | 7 | G | C | G | 0.91935 | C | 0.08065 | 5 | 0.16129 |
| 10442 | S12_70884454 | 12 | 70884454 | 149 | T | C | T | 0.87097 | C | 0.12903 | 8 | 0.25806 |
| 10443 | S12_70884537 | 12 | 70884537 | 83 | C | T | C | 0.91935 | T | 0.08065 | 5 | 0.16129 |
| 10444 | S12_70884538 | 12 | 70884538 | 1 | G | A | G | 0.90323 | A | 0.09677 | 6 | 0.19355 |
| 10445 | S12_70951232 | 12 | 70951232 | 66694 | C | T | T | 0.59677 | C | 0.40323 | 15 | 0.48387 |
| 10446 | S12_70951233 | 12 | 70951233 | 1 | G | A | G | 0.8871 | A | 0.1129 | 5 | 0.16129 |
| 10447 | S12_70951237 | 12 | 70951237 | 4 | T | C | T | 0.72581 | C | 0.27419 | 13 | 0.41935 |
| 10448 | S12_71195037 | 12 | 71195037 | 243800 | C | T | C | 0.77419 | T | 0.22581 | 8 | 0.25806 |
| 10449 | S12_71298072 | 12 | 71298072 | 103035 | C | T | C | 0.79032 | T | 0.20968 | 9 | 0.29032 |
| 10450 | S12_71298109 | 12 | 71298109 | 37 | A | G | A | 0.74194 | G | 0.25806 | 14 | 0.45161 |
| 10451 | S12_72045308 | 12 | 72045308 | 747199 | A | G | A | 0.77419 | G | 0.22581 | 10 | 0.32258 |
| 10452 | S12_72045378 | 12 | 72045378 | 70 | A | G | A | 0.8871 | G | 0.1129 | 7 | 0.22581 |
| 10453 | S12_72045520 | 12 | 72045520 | 142 | C | T | C | 0.93548 | T | 0.06452 | 4 | 0.12903 |
| 10454 | S12_72367633 | 12 | 72367633 | 322113 | A | G | G | 0.59677 | A | 0.40323 | 17 | 0.54839 |
| 10455 | S12_72367754 | 12 | 72367754 | 121 | G | C | C | 0.58065 | G | 0.41935 | 16 | 0.51613 |
| 10456 | S12_72367820 | 12 | 72367820 | 66 | C | T | T | 0.59677 | C | 0.40323 | 17 | 0.54839 |
| 10457 | S12_72414355 | 12 | 72414355 | 46535 | T | A | A | 0.80645 | T | 0.19355 | 6 | 0.19355 |
| 10458 | S12_72414358 | 12 | 72414358 | 3 | A | G | A | 0.95161 | G | 0.04839 | 3 | 0.09677 |
| 10459 | S12_72414593 | 12 | 72414593 | 235 | G | A | G | 0.80645 | A | 0.19355 | 6 | 0.19355 |
| 10460 | S12_72662773 | 12 | 72662773 | 248180 | C | T | C | 0.82258 | T | 0.17742 | 9 | 0.29032 |
| 10461 | S12_72782199 | 12 | 72782199 | 119426 | A | G | A | 0.8871 | G | 0.1129 | 7 | 0.22581 |
| 10462 | S12_72876904 | 12 | 72876904 | 94705 | A | G | A | 0.93548 | G | 0.06452 | 4 | 0.12903 |
| 10463 | S12_73015803 | 12 | 73015803 | 138899 | A | G | A | 0.67742 | G | 0.32258 | 12 | 0.3871 |
| 10464 | S12_73015838 | 12 | 73015838 | 35 | G | A | G | 0.90323 | A | 0.09677 | 4 | 0.12903 |
| 10465 | S12_73015997 | 12 | 73015997 | 159 | G | A | G | 0.79032 | A | 0.20968 | 9 | 0.29032 |
| 10466 | S12_73016013 | 12 | 73016013 | 16 | G | C | G | 0.79032 | C | 0.20968 | 9 | 0.29032 |
| 10467 | S12_73164715 | 12 | 73164715 | 148702 | T | C | T | 0.70968 | C | 0.29032 | 16 | 0.51613 |
| 10468 | S12_73164780 | 12 | 73164780 | 65 | A | G | A | 0.66129 | G | 0.33871 | 17 | 0.54839 |
| 10469 | S12_73223063 | 12 | 73223063 | 58283 | C | T | T | 0.82258 | C | 0.17742 | 9 | 0.29032 |
| 10470 | S12_73223081 | 12 | 73223081 | 18 | G | A | G | 0.91935 | A | 0.08065 | 5 | 0.16129 |
| 10471 | S12_73223100 | 12 | 73223100 | 19 | A | G | A | 0.64516 | G | 0.35484 | 20 | 0.64516 |
| 10472 | S12_73223102 | 12 | 73223102 | 2 | G | A | G | 0.91935 | A | 0.08065 | 5 | 0.16129 |
| 10473 | S12_73223123 | 12 | 73223123 | 21 | G | A | G | 0.93548 | A | 0.06452 | 4 | 0.12903 |
| 10474 | S12_73245749 | 12 | 73245749 | 22626 | T | C | C | 0.6129 | T | 0.3871 | 16 | 0.51613 |

| Site number | SNP name | Chromosome | Physical position | Physical distance from the previous SNP | Reference allele | Alternative allele | Major allele | Major allele frequency | Minor allele | Minor allele frequency | Number heterozygous | Heterozygosity |
|---|---|---|---|---|---|---|---|---|---|---|---|---|
| 10475 | S12_73245949 | 12 | 73245949 | 200 | C | T | C | 0.87097 | T | 0.12903 | 8 | 0.25806 |
| 10476 | S12_73383996 | 12 | 73383996 | 138047 | G | T | G | 0.90323 | T | 0.09677 | 6 | 0.19355 |
| 10477 | S12_73510554 | 12 | 73510554 | 126558 | G | C | G | 0.87097 | C | 0.12903 | 8 | 0.25806 |
| 10478 | S12_73836959 | 12 | 73836959 | 326405 | T | C | T | 0.93548 | C | 0.06452 | 4 | 0.12903 |
| 10479 | S12_73837144 | 12 | 73837144 | 185 | G | A | G | 0.93548 | A | 0.06452 | 4 | 0.12903 |
| 10480 | S12_73837183 | 12 | 73837183 | 39 | C | T | C | 0.93548 | T | 0.06452 | 4 | 0.12903 |
| 10481 | S12_73837184 | 12 | 73837184 | 1 | A | G | A | 0.93548 | G | 0.06452 | 4 | 0.12903 |
| 10482 | S12_73847939 | 12 | 73847939 | 10755 | T | C | T | 0.93548 | C | 0.06452 | 4 | 0.12903 |
| 10483 | S12_73848049 | 12 | 73848049 | 110 | A | G | G | 0.51613 | A | 0.48387 | 16 | 0.51613 |
| 10484 | S12_73863662 | 12 | 73863662 | 15613 | A | G | A | 0.95161 | G | 0.04839 | 3 | 0.09677 |
| 10485 | S12_73863774 | 12 | 73863774 | 112 | A | C | A | 0.93548 | C | 0.06452 | 4 | 0.12903 |
| 10486 | S12_73863866 | 12 | 73863866 | 92 | G | A | A | 0.5 | G | 0.5 | 13 | 0.41935 |
| 10487 | S12_73903212 | 12 | 73903212 | 39346 | G | A | G | 0.8871 | A | 0.1129 | 5 | 0.16129 |
| 10488 | S12_74174872 | 12 | 74174872 | 271660 | C | T | C | 0.90323 | T | 0.09677 | 6 | 0.19355 |
| 10489 | S12_74174946 | 12 | 74174946 | 74 | G | A | G | 0.90323 | A | 0.09677 | 6 | 0.19355 |
| 10490 | S12_74174977 | 12 | 74174977 | 31 | G | T | G | 0.91935 | T | 0.08065 | 5 | 0.16129 |
| 10491 | S12_74174990 | 12 | 74174990 | 13 | A | G | A | 0.90323 | G | 0.09677 | 6 | 0.19355 |
| 10492 | S12_74469145 | 12 | 74469145 | 294155 | C | T | C | 0.66129 | T | 0.33871 | 13 | 0.41935 |
| 10493 | S12_74469276 | 12 | 74469276 | 131 | G | A | G | 0.90323 | A | 0.09677 | 6 | 0.19355 |
| 10494 | S12_75307534 | 12 | 75307534 | 838258 | T | G | T | 0.82258 | G | 0.17742 | 7 | 0.22581 |
| 10495 | S12_75307555 | 12 | 75307555 | 21 | G | A | G | 0.70968 | A | 0.29032 | 16 | 0.51613 |
| 10496 | S12_75307575 | 12 | 75307575 | 20 | G | A | A | 0.70968 | G | 0.29032 | 14 | 0.45161 |
| 10497 | S12_75307648 | 12 | 75307648 | 73 | A | T | A | 0.70968 | T | 0.29032 | 16 | 0.51613 |
| 10498 | S12_75307750 | 12 | 75307750 | 102 | A | T | A | 0.79032 | T | 0.20968 | 9 | 0.29032 |
| 10499 | S12_75420184 | 12 | 75420184 | 112434 | G | A | A | 0.58065 | G | 0.41935 | 10 | 0.32258 |
| 10500 | S12_75858849 | 12 | 75858849 | 438665 | T | C | T | 0.95161 | C | 0.04839 | 3 | 0.09677 |
| 10501 | S12_75858894 | 12 | 75858894 | 45 | G | A | G | 0.93548 | A | 0.06452 | 4 | 0.12903 |
| 10502 | S12_75858913 | 12 | 75858913 | 19 | T | C | T | 0.8871 | C | 0.1129 | 7 | 0.22581 |
| 10503 | S12_75859041 | 12 | 75859041 | 128 | G | A | G | 0.93548 | A | 0.06452 | 4 | 0.12903 |
| 10504 | S12_76114660 | 12 | 76114660 | 255619 | T | C | T | 0.56452 | C | 0.43548 | 15 | 0.48387 |
| 10505 | S12_76114661 | 12 | 76114661 | 1 | G | A | G | 0.75806 | A | 0.24194 | 11 | 0.35484 |
| 10506 | S12_76114775 | 12 | 76114775 | 114 | C | T | C | 0.95161 | T | 0.04839 | 3 | 0.09677 |
| 10507 | S12_76114794 | 12 | 76114794 | 19 | C | T | C | 0.90323 | T | 0.09677 | 4 | 0.12903 |
| 10508 | S12_76114830 | 12 | 76114830 | 36 | C | T | C | 0.8871 | T | 0.1129 | 7 | 0.22581 |
| 10509 | S12_76246811 | 12 | 76246811 | 131981 | G | A | G | 0.66129 | A | 0.33871 | 19 | 0.6129 |
| 10510 | S12_76246995 | 12 | 76246995 | 184 | T | C | T | 0.69355 | C | 0.30645 | 17 | 0.54839 |
| 10511 | S12_77126083 | 12 | 77126083 | 879088 | G | A | G | 0.90323 | A | 0.09677 | 6 | 0.19355 |
| 10512 | S12_77126091 | 12 | 77126091 | 8 | C | T | C | 0.53226 | T | 0.46774 | 19 | 0.6129 |
| 10513 | S12_77126114 | 12 | 77126114 | 23 | A | C | A | 0.90323 | C | 0.09677 | 6 | 0.19355 |
| 10514 | S12_77417201 | 12 | 77417201 | 291087 | C | T | C | 0.64516 | T | 0.35484 | 14 | 0.45161 |
| 10515 | S12_77417202 | 12 | 77417202 | 1 | G | A | G | 0.79032 | A | 0.20968 | 9 | 0.29032 |
| 10516 | S12_77417294 | 12 | 77417294 | 92 | C | G | C | 0.79032 | G | 0.20968 | 9 | 0.29032 |
| 10517 | S12_77499305 | 12 | 77499305 | 82011 | T | C | T | 0.62903 | C | 0.37097 | 17 | 0.54839 |
| 10518 | S12_78028773 | 12 | 78028773 | 529468 | T | C | T | 0.90323 | C | 0.09677 | 6 | 0.19355 |
| 10519 | S12_78028881 | 12 | 78028881 | 108 | A | T | A | 0.83871 | T | 0.16129 | 8 | 0.25806 |
| 10520 | S12_78028906 | 12 | 78028906 | 25 | C | T | C | 0.91935 | T | 0.08065 | 3 | 0.09677 |
| 10521 | S12_78191498 | 12 | 78191498 | 162592 | G | A | G | 0.93548 | A | 0.06452 | 4 | 0.12903 |
| 10522 | S12_78522312 | 12 | 78522312 | 330814 | G | A | G | 0.79032 | A | 0.20968 | 9 | 0.29032 |
| 10523 | S12_78522351 | 12 | 78522351 | 39 | T | C | T | 0.8871 | C | 0.1129 | 5 | 0.16129 |
| 10524 | S12_78834899 | 12 | 78834899 | 312548 | T | A | T | 0.70968 | A | 0.29032 | 10 | 0.32258 |
| 10525 | S12_78834973 | 12 | 78834973 | 74 | T | C | C | 0.90323 | T | 0.09677 | 4 | 0.12903 |
| 10526 | S12_78834992 | 12 | 78834992 | 19 | T | G | T | 0.90323 | G | 0.09677 | 4 | 0.12903 |
| 10527 | S12_79837062 | 12 | 79837062 | 1002070 | G | A | G | 0.90323 | A | 0.09677 | 6 | 0.19355 |
| 10528 | S12_79837127 | 12 | 79837127 | 65 | G | A | G | 0.53226 | A | 0.46774 | 13 | 0.41935 |

| Site number | SNP name | Chromosome | Physical position | Physical distance from the previous SNP | Reference allele | Alternative allele | Major allele | Major allele frequency | Minor allele | Minor allele frequency | Number heterozygous | Heterozygosity |
|---|---|---|---|---|---|---|---|---|---|---|---|---|
| 10529 | S12_80858407 | 12 | 80858407 | 1021280 | C | T | C | 0.6129 | T | 0.3871 | 12 | 0.3871 |
| 10530 | S12_80858432 | 12 | 80858432 | 25 | C | T | C | 0.90323 | T | 0.09677 | 6 | 0.19355 |
| 10531 | S12_80858451 | 12 | 80858451 | 19 | G | A | G | 0.95161 | A | 0.04839 | 3 | 0.09677 |
| 10532 | S12_80858508 | 12 | 80858508 | 57 | G | A | G | 0.8871 | A | 0.1129 | 5 | 0.16129 |
| 10533 | S12_80858543 | 12 | 80858543 | 35 | G | A | G | 0.95161 | A | 0.04839 | 3 | 0.09677 |
| 10534 | S12_80858568 | 12 | 80858568 | 25 | C | T | C | 0.74194 | T | 0.25806 | 12 | 0.3871 |
| 10535 | S12_81083652 | 12 | 81083652 | 225084 | A | T | A | 0.59677 | T | 0.40323 | 13 | 0.41935 |
| 10536 | S12_81083849 | 12 | 81083849 | 197 | C | A | C | 0.75806 | A | 0.24194 | 13 | 0.41935 |
| 10537 | S12_81201699 | 12 | 81201699 | 117850 | C | T | C | 0.90323 | T | 0.09677 | 4 | 0.12903 |
| 10538 | S12_82160249 | 12 | 82160249 | 958550 | G | T | T | 0.66129 | G | 0.33871 | 13 | 0.41935 |
| 10539 | S12_82160310 | 12 | 82160310 | 61 | A | G | A | 0.80645 | G | 0.19355 | 8 | 0.25806 |
| 10540 | S12_83339131 | 12 | 83339131 | 1178821 | A | G | A | 0.91935 | G | 0.08065 | 5 | 0.16129 |
| 10541 | S12_83339182 | 12 | 83339182 | 51 | A | G | A | 0.80645 | G | 0.19355 | 12 | 0.3871 |
| 10542 | S12_83339206 | 12 | 83339206 | 24 | T | G | T | 0.95161 | G | 0.04839 | 3 | 0.09677 |
| 10543 | S12_83987450 | 12 | 83987450 | 648244 | C | T | C | 0.91935 | T | 0.08065 | 5 | 0.16129 |
| 10544 | S12_83987562 | 12 | 83987562 | 112 | C | T | C | 0.91935 | T | 0.08065 | 5 | 0.16129 |
| 10545 | S12_84136127 | 12 | 84136127 | 148565 | A | C | A | 0.8871 | C | 0.1129 | 5 | 0.16129 |
| 10546 | S12_84141719 | 12 | 84141719 | 5592 | A | G | A | 0.8871 | G | 0.1129 | 5 | 0.16129 |
| 10547 | S12_84203891 | 12 | 84203891 | 62172 | A | G | A | 0.95161 | G | 0.04839 | 1 | 0.03226 |
| 10548 | S12_84203903 | 12 | 84203903 | 12 | T | C | T | 0.85484 | C | 0.14516 | 9 | 0.29032 |
| 10549 | S12_84204058 | 12 | 84204058 | 155 | A | C | A | 0.85484 | C | 0.14516 | 9 | 0.29032 |
| 10550 | S12_84236999 | 12 | 84236999 | 32941 | T | G | T | 0.95161 | G | 0.04839 | 3 | 0.09677 |
| 10551 | S12_84457384 | 12 | 84457384 | 220385 | G | A | G | 0.93548 | A | 0.06452 | 2 | 0.06452 |
| 10552 | S12_84457434 | 12 | 84457434 | 50 | C | T | C | 0.77419 | T | 0.22581 | 10 | 0.32258 |
| 10553 | S12_84457452 | 12 | 84457452 | 18 | G | A | G | 0.83871 | A | 0.16129 | 8 | 0.25806 |
| 10554 | S12_84457524 | 12 | 84457524 | 72 | G | C | G | 0.93548 | C | 0.06452 | 2 | 0.06452 |
| 10555 | S12_84457531 | 12 | 84457531 | 7 | C | T | C | 0.90323 | T | 0.09677 | 6 | 0.19355 |
| 10556 | S12_84457535 | 12 | 84457535 | 4 | C | A | C | 0.83871 | A | 0.16129 | 8 | 0.25806 |
| 10557 | S12_85448872 | 12 | 85448872 | 991337 | C | T | T | 0.82258 | C | 0.17742 | 11 | 0.35484 |
| 10558 | S12_85448909 | 12 | 85448909 | 37 | C | T | T | 0.87097 | C | 0.12903 | 8 | 0.25806 |
| 10559 | S12_85448923 | 12 | 85448923 | 14 | T | C | C | 0.82258 | T | 0.17742 | 11 | 0.35484 |
| 10560 | S12_85514173 | 12 | 85514173 | 65250 | A | T | T | 0.93548 | A | 0.06452 | 4 | 0.12903 |
| 10561 | S12_86200868 | 12 | 86200868 | 686695 | G | A | A | 0.91935 | G | 0.08065 | 5 | 0.16129 |
| 10562 | S12_86996863 | 12 | 86996863 | 795995 | A | T | A | 0.85484 | T | 0.14516 | 9 | 0.29032 |
| 10563 | S12_86996873 | 12 | 86996873 | 10 | G | A | A | 0.85484 | G | 0.14516 | 9 | 0.29032 |
| 10564 | S12_86996949 | 12 | 86996949 | 76 | C | G | C | 0.70968 | G | 0.29032 | 18 | 0.58065 |
| 10565 | S12_86996990 | 12 | 86996990 | 41 | G | C | G | 0.87097 | C | 0.12903 | 8 | 0.25806 |
| 10566 | S12_86997016 | 12 | 86997016 | 26 | C | G | G | 0.54839 | C | 0.45161 | 14 | 0.45161 |
| 10567 | S13_43681 | 13 | 43681 | 0 | C | T | C | 0.91935 | T | 0.08065 | 3 | 0.09677 |
| 10568 | S13_140336 | 13 | 140336 | 96655 | A | G | A | 0.77419 | G | 0.22581 | 14 | 0.45161 |
| 10569 | S13_140472 | 13 | 140472 | 136 | G | A | G | 0.95161 | A | 0.04839 | 3 | 0.09677 |
| 10570 | S13_189744 | 13 | 189744 | 49272 | T | C | T | 0.82258 | C | 0.17742 | 11 | 0.35484 |
| 10571 | S13_189762 | 13 | 189762 | 18 | G | C | G | 0.72581 | C | 0.27419 | 17 | 0.54839 |
| 10572 | S13_258444 | 13 | 258444 | 68682 | A | G | A | 0.79032 | G | 0.20968 | 11 | 0.35484 |
| 10573 | S13_344426 | 13 | 344426 | 85982 | T | A | T | 0.91935 | A | 0.08065 | 3 | 0.09677 |
| 10574 | S13_344427 | 13 | 344427 | 1 | T | C | T | 0.91935 | C | 0.08065 | 3 | 0.09677 |
| 10575 | S13_1519725 | 13 | 1519725 | 1175298 | A | G | A | 0.80645 | G | 0.19355 | 12 | 0.3871 |
| 10576 | S13_1521562 | 13 | 1521562 | 1837 | T | C | T | 0.80645 | C | 0.19355 | 8 | 0.25806 |
| 10577 | S13_1521787 | 13 | 1521787 | 225 | T | C | T | 0.93548 | C | 0.06452 | 4 | 0.12903 |
| 10578 | S13_1564110 | 13 | 1564110 | 42323 | G | A | G | 0.64516 | A | 0.35484 | 12 | 0.3871 |
| 10579 | S13_1583676 | 13 | 1583676 | 19566 | T | C | T | 0.6129 | C | 0.3871 | 14 | 0.45161 |
| 10580 | S13_1583791 | 13 | 1583791 | 115 | C | G | C | 0.67742 | G | 0.32258 | 14 | 0.45161 |
| 10581 | S13_1583890 | 13 | 1583890 | 99 | A | G | G | 0.85484 | A | 0.14516 | 5 | 0.16129 |
| 10582 | S13_2006774 | 13 | 2006774 | 422884 | G | A | A | 0.5 | G | 0.5 | 13 | 0.41935 |

| Site number | SNP name | Chromosome | Physical position | Physical distance from the previous SNP | Reference allele | Alternative allele | Major allele | Major allele frequency | Minor allele | Minor allele frequency | Number heterozygous | Heterozygosity |
|---|---|---|---|---|---|---|---|---|---|---|---|---|
| 10583 | S13_2006780 | 13 | 2006780 | 6 | T | G | T | 0.93548 | G | 0.06452 | 4 | 0.12903 |
| 10584 | S13_2006914 | 13 | 2006914 | 134 | T | C | T | 0.95161 | C | 0.04839 | 3 | 0.09677 |
| 10585 | S13_2022588 | 13 | 2022588 | 15674 | C | T | C | 0.79032 | T | 0.20968 | 11 | 0.35484 |
| 10586 | S13_2090506 | 13 | 2090506 | 67918 | G | A | G | 0.75806 | A | 0.24194 | 11 | 0.35484 |
| 10587 | S13_2090532 | 13 | 2090532 | 26 | G | C | G | 0.74194 | C | 0.25806 | 12 | 0.3871 |
| 10588 | S13_2144147 | 13 | 2144147 | 53615 | A | C | A | 0.93548 | C | 0.06452 | 4 | 0.12903 |
| 10589 | S13_2144330 | 13 | 2144330 | 183 | G | A | G | 0.93548 | A | 0.06452 | 4 | 0.12903 |
| 10590 | S13_2777010 | 13 | 2777010 | 632680 | C | T | C | 0.95161 | T | 0.04839 | 3 | 0.09677 |
| 10591 | S13_2777028 | 13 | 2777028 | 18 | A | G | G | 0.54839 | A | 0.45161 | 14 | 0.45161 |
| 10592 | S13_2777085 | 13 | 2777085 | 57 | A | G | G | 0.54839 | A | 0.45161 | 14 | 0.45161 |
| 10593 | S13_2777147 | 13 | 2777147 | 62 | T | A | T | 0.72581 | A | 0.27419 | 9 | 0.29032 |
| 10594 | S13_2819961 | 13 | 2819961 | 42814 | C | T | T | 0.80645 | C | 0.19355 | 6 | 0.19355 |
| 10595 | S13_3134938 | 13 | 3134938 | 314977 | C | T | C | 0.93548 | T | 0.06452 | 4 | 0.12903 |
| 10596 | S13_3134944 | 13 | 3134944 | 6 | G | A | G | 0.95161 | A | 0.04839 | 3 | 0.09677 |
| 10597 | S13_3134949 | 13 | 3134949 | 5 | A | T | T | 0.93548 | A | 0.06452 | 4 | 0.12903 |
| 10598 | S13_3134967 | 13 | 3134967 | 18 | T | C | T | 0.95161 | C | 0.04839 | 3 | 0.09677 |
| 10599 | S13_3134970 | 13 | 3134970 | 3 | C | T | T | 0.93548 | C | 0.06452 | 4 | 0.12903 |
| 10600 | S13_3135077 | 13 | 3135077 | 107 | C | T | C | 0.93548 | T | 0.06452 | 4 | 0.12903 |
| 10601 | S13_3276765 | 13 | 3276765 | 141688 | C | A | C | 0.95161 | A | 0.04839 | 3 | 0.09677 |
| 10602 | S13_3276913 | 13 | 3276913 | 148 | C | A | C | 0.95161 | A | 0.04839 | 3 | 0.09677 |
| 10603 | S13_3354771 | 13 | 3354771 | 77858 | G | A | G | 0.70968 | A | 0.29032 | 16 | 0.51613 |
| 10604 | S13_3530284 | 13 | 3530284 | 175513 | G | A | G | 0.54839 | A | 0.45161 | 16 | 0.51613 |
| 10605 | S13_3601336 | 13 | 3601336 | 71052 | C | T | C | 0.75806 | T | 0.24194 | 11 | 0.35484 |
| 10606 | S13_3601420 | 13 | 3601420 | 84 | C | G | C | 0.74194 | G | 0.25806 | 12 | 0.3871 |
| 10607 | S13_3651225 | 13 | 3651225 | 49805 | C | T | C | 0.79032 | T | 0.20968 | 11 | 0.35484 |
| 10608 | S13_3710293 | 13 | 3710293 | 59068 | C | T | C | 0.95161 | T | 0.04839 | 3 | 0.09677 |
| 10609 | S13_3710442 | 13 | 3710442 | 149 | T | C | T | 0.6129 | C | 0.3871 | 12 | 0.3871 |
| 10610 | S13_3825553 | 13 | 3825553 | 115111 | G | T | G | 0.77419 | T | 0.22581 | 12 | 0.3871 |
| 10611 | S13_3825704 | 13 | 3825704 | 151 | G | T | G | 0.53226 | T | 0.46774 | 17 | 0.54839 |
| 10612 | S13_3825761 | 13 | 3825761 | 57 | A | G | A | 0.53226 | G | 0.46774 | 17 | 0.54839 |
| 10613 | S13_4489924 | 13 | 4489924 | 664163 | C | A | C | 0.90323 | A | 0.09677 | 6 | 0.19355 |
| 10614 | S13_4489985 | 13 | 4489985 | 61 | A | G | G | 0.64516 | A | 0.35484 | 14 | 0.45161 |
| 10615 | S13_4490000 | 13 | 4490000 | 15 | G | A | G | 0.90323 | A | 0.09677 | 6 | 0.19355 |
| 10616 | S13_4490064 | 13 | 4490064 | 64 | G | A | G | 0.90323 | A | 0.09677 | 6 | 0.19355 |
| 10617 | S13_4783089 | 13 | 4783089 | 293025 | A | G | A | 0.90323 | G | 0.09677 | 4 | 0.12903 |
| 10618 | S13_4783283 | 13 | 4783283 | 194 | G | A | G | 0.87097 | A | 0.12903 | 6 | 0.19355 |
| 10619 | S13_5567040 | 13 | 5567040 | 783757 | C | T | T | 0.90323 | C | 0.09677 | 6 | 0.19355 |
| 10620 | S13_5567112 | 13 | 5567112 | 72 | A | T | A | 0.87097 | T | 0.12903 | 6 | 0.19355 |
| 10621 | S13_6443841 | 13 | 6443841 | 876729 | G | A | G | 0.90323 | A | 0.09677 | 6 | 0.19355 |
| 10622 | S13_6443909 | 13 | 6443909 | 68 | C | A | C | 0.90323 | A | 0.09677 | 6 | 0.19355 |
| 10623 | S13_6443956 | 13 | 6443956 | 47 | A | G | A | 0.6129 | G | 0.3871 | 16 | 0.51613 |
| 10624 | S13_6566077 | 13 | 6566077 | 122121 | T | C | T | 0.87097 | C | 0.12903 | 8 | 0.25806 |
| 10625 | S13_6566101 | 13 | 6566101 | 24 | A | C | A | 0.90323 | C | 0.09677 | 6 | 0.19355 |
| 10626 | S13_7241775 | 13 | 7241775 | 675674 | C | T | T | 0.72581 | C | 0.27419 | 15 | 0.48387 |
| 10627 | S13_9143878 | 13 | 9143878 | 1902103 | C | T | C | 0.90323 | T | 0.09677 | 6 | 0.19355 |
| 10628 | S13_9143963 | 13 | 9143963 | 85 | A | G | A | 0.87097 | G | 0.12903 | 8 | 0.25806 |
| 10629 | S13_9143976 | 13 | 9143976 | 13 | C | T | C | 0.87097 | T | 0.12903 | 8 | 0.25806 |
| 10630 | S13_9144074 | 13 | 9144074 | 98 | G | A | G | 0.87097 | A | 0.12903 | 8 | 0.25806 |
| 10631 | S13_9378446 | 13 | 9378446 | 234372 | A | G | A | 0.75806 | G | 0.24194 | 9 | 0.29032 |
| 10632 | S13_9378516 | 13 | 9378516 | 70 | A | G | A | 0.74194 | G | 0.25806 | 10 | 0.32258 |
| 10633 | S13_9378526 | 13 | 9378526 | 10 | C | T | C | 0.74194 | T | 0.25806 | 10 | 0.32258 |
| 10634 | S13_9378625 | 13 | 9378625 | 99 | T | C | T | 0.75806 | C | 0.24194 | 9 | 0.29032 |
| 10635 | S13_9378634 | 13 | 9378634 | 9 | T | C | T | 0.75806 | C | 0.24194 | 9 | 0.29032 |
| 10636 | S13_9715970 | 13 | 9715970 | 337336 | A | G | A | 0.93548 | G | 0.06452 | 4 | 0.12903 |

| Site number | SNP name | Chromosome | Physical position | Physical distance from the previous SNP | Reference allele | Alternative allele | Major allele | Major allele frequency | Minor allele | Minor allele frequency | Number heterozygous | Heterozygosity |
|---|---|---|---|---|---|---|---|---|---|---|---|---|
| 10637 | S13_9715976 | 13 | 9715976 | 6 | T | G | T | 0.93548 | G | 0.06452 | 4 | 0.12903 |
| 10638 | S13_9716099 | 13 | 9716099 | 123 | A | G | A | 0.91935 | G | 0.08065 | 5 | 0.16129 |
| 10639 | S13_9716119 | 13 | 9716119 | 20 | A | G | A | 0.95161 | G | 0.04839 | 1 | 0.03226 |
| 10640 | S13_9815446 | 13 | 9815446 | 99327 | T | C | C | 0.91935 | T | 0.08065 | 5 | 0.16129 |
| 10641 | S13_10048591 | 13 | 10048591 | 233145 | G | A | G | 0.93548 | A | 0.06452 | 4 | 0.12903 |
| 10642 | S13_10048645 | 13 | 10048645 | 54 | T | C | T | 0.77419 | C | 0.22581 | 12 | 0.3871 |
| 10643 | S13_10048744 | 13 | 10048744 | 99 | T | C | T | 0.62903 | C | 0.37097 | 15 | 0.48387 |
| 10644 | S13_10059397 | 13 | 10059397 | 10653 | C | A | C | 0.95161 | A | 0.04839 | 3 | 0.09677 |
| 10645 | S13_10188275 | 13 | 10188275 | 128878 | C | G | C | 0.75806 | G | 0.24194 | 13 | 0.41935 |
| 10646 | S13_10188300 | 13 | 10188300 | 25 | G | A | G | 0.72581 | A | 0.27419 | 11 | 0.35484 |
| 10647 | S13_10188405 | 13 | 10188405 | 105 | A | C | A | 0.62903 | C | 0.37097 | 19 | 0.6129 |
| 10648 | S13_10192290 | 13 | 10192290 | 3885 | T | G | T | 0.93548 | G | 0.06452 | 4 | 0.12903 |
| 10649 | S13_10192303 | 13 | 10192303 | 13 | C | T | C | 0.93548 | T | 0.06452 | 4 | 0.12903 |
| 10650 | S13_10192355 | 13 | 10192355 | 52 | G | C | G | 0.56452 | C | 0.43548 | 17 | 0.54839 |
| 10651 | S13_10414467 | 13 | 10414467 | 222112 | A | T | A | 0.91935 | T | 0.08065 | 5 | 0.16129 |
| 10652 | S13_10783762 | 13 | 10783762 | 369295 | C | T | T | 0.51613 | C | 0.48387 | 16 | 0.51613 |
| 10653 | S13_10783786 | 13 | 10783786 | 24 | G | A | G | 0.85484 | A | 0.14516 | 5 | 0.16129 |
| 10654 | S13_10783793 | 13 | 10783793 | 7 | G | A | A | 0.51613 | G | 0.48387 | 16 | 0.51613 |
| 10655 | S13_10783801 | 13 | 10783801 | 8 | A | G | G | 0.51613 | A | 0.48387 | 16 | 0.51613 |
| 10656 | S13_10783809 | 13 | 10783809 | 8 | T | G | G | 0.51613 | T | 0.48387 | 16 | 0.51613 |
| 10657 | S13_10783861 | 13 | 10783861 | 52 | C | T | C | 0.80645 | T | 0.19355 | 8 | 0.25806 |
| 10658 | S13_10783891 | 13 | 10783891 | 30 | T | C | T | 0.80645 | C | 0.19355 | 8 | 0.25806 |
| 10659 | S13_10851976 | 13 | 10851976 | 68085 | G | A | G | 0.79032 | A | 0.20968 | 13 | 0.41935 |
| 10660 | S13_10851991 | 13 | 10851991 | 15 | C | T | C | 0.83871 | T | 0.16129 | 6 | 0.19355 |
| 10661 | S13_10852041 | 13 | 10852041 | 50 | C | T | T | 0.53226 | C | 0.46774 | 19 | 0.6129 |
| 10662 | S13_10852042 | 13 | 10852042 | 1 | G | A | A | 0.53226 | G | 0.46774 | 19 | 0.6129 |
| 10663 | S13_10930460 | 13 | 10930460 | 78418 | G | T | G | 0.70968 | T | 0.29032 | 10 | 0.32258 |
| 10664 | S13_11094678 | 13 | 11094678 | 164218 | C | T | C | 0.87097 | T | 0.12903 | 8 | 0.25806 |
| 10665 | S13_11214915 | 13 | 11214915 | 120237 | G | A | G | 0.62903 | A | 0.37097 | 15 | 0.48387 |
| 10666 | S13_11214925 | 13 | 11214925 | 10 | C | T | C | 0.93548 | T | 0.06452 | 4 | 0.12903 |
| 10667 | S13_11214926 | 13 | 11214926 | 1 | A | G | G | 0.91935 | A | 0.08065 | 5 | 0.16129 |
| 10668 | S13_11302348 | 13 | 11302348 | 87422 | G | T | G | 0.64516 | T | 0.35484 | 12 | 0.3871 |
| 10669 | S13_11302431 | 13 | 11302431 | 83 | G | A | G | 0.95161 | A | 0.04839 | 1 | 0.03226 |
| 10670 | S13_11302498 | 13 | 11302498 | 67 | C | T | C | 0.56452 | T | 0.43548 | 17 | 0.54839 |
| 10671 | S13_11569882 | 13 | 11569882 | 267384 | G | A | G | 0.91935 | A | 0.08065 | 3 | 0.09677 |
| 10672 | S13_11730792 | 13 | 11730792 | 160910 | A | G | A | 0.90323 | G | 0.09677 | 6 | 0.19355 |
| 10673 | S13_11818128 | 13 | 11818128 | 87336 | G | A | G | 0.75806 | A | 0.24194 | 11 | 0.35484 |
| 10674 | S13_11818164 | 13 | 11818164 | 36 | A | G | A | 0.74194 | G | 0.25806 | 10 | 0.32258 |
| 10675 | S13_11818165 | 13 | 11818165 | 1 | T | G | T | 0.74194 | G | 0.25806 | 10 | 0.32258 |
| 10676 | S13_11818233 | 13 | 11818233 | 68 | T | C | T | 0.74194 | C | 0.25806 | 10 | 0.32258 |
| 10677 | S13_12080296 | 13 | 12080296 | 262063 | C | T | C | 0.91935 | T | 0.08065 | 5 | 0.16129 |
| 10678 | S13_12111739 | 13 | 12111739 | 31443 | T | C | T | 0.91935 | C | 0.08065 | 5 | 0.16129 |
| 10679 | S13_12145332 | 13 | 12145332 | 33593 | C | T | C | 0.95161 | T | 0.04839 | 1 | 0.03226 |
| 10680 | S13_12145398 | 13 | 12145398 | 66 | T | C | T | 0.53226 | C | 0.46774 | 13 | 0.41935 |
| 10681 | S13_12370786 | 13 | 12370786 | 225388 | C | G | C | 0.93548 | G | 0.06452 | 2 | 0.06452 |
| 10682 | S13_12370862 | 13 | 12370862 | 76 | C | T | C | 0.74194 | T | 0.25806 | 10 | 0.32258 |
| 10683 | S13_12371176 | 13 | 12371176 | 314 | G | A | G | 0.91935 | A | 0.08065 | 5 | 0.16129 |
| 10684 | S13_12824764 | 13 | 12824764 | 453588 | A | G | A | 0.77419 | G | 0.22581 | 8 | 0.25806 |
| 10685 | S13_12848598 | 13 | 12848598 | 23834 | T | C | T | 0.56452 | C | 0.43548 | 11 | 0.35484 |
| 10686 | S13_12848647 | 13 | 12848647 | 49 | G | A | G | 0.56452 | A | 0.43548 | 11 | 0.35484 |
| 10687 | S13_12848702 | 13 | 12848702 | 55 | A | G | A | 0.56452 | G | 0.43548 | 11 | 0.35484 |
| 10688 | S13_12884458 | 13 | 12884458 | 35756 | C | T | C | 0.85484 | T | 0.14516 | 7 | 0.22581 |
| 10689 | S13_12889578 | 13 | 12889578 | 5120 | G | A | A | 0.58065 | G | 0.41935 | 14 | 0.45161 |
| 10690 | S13_12889741 | 13 | 12889741 | 163 | C | G | C | 0.8871 | G | 0.1129 | 7 | 0.22581 |

| Site number | SNP name | Chromosome | Physical position | Physical distance from the previous SNP | Reference allele | Alternative allele | Major allele | Major allele frequency | Minor allele | Minor allele frequency | Number heterozygous | Heterozygosity |
|---|---|---|---|---|---|---|---|---|---|---|---|---|
| 10691 | S13_13009833 | 13 | 13009833 | 120092 | C | T | C | 0.90323 | T | 0.09677 | 4 | 0.12903 |
| 10692 | S13_13700217 | 13 | 13700217 | 690384 | C | T | C | 0.90323 | T | 0.09677 | 6 | 0.19355 |
| 10693 | S13_13701077 | 13 | 13701077 | 860 | T | C | T | 0.87097 | C | 0.12903 | 6 | 0.19355 |
| 10694 | S13_14456514 | 13 | 14456514 | 755437 | T | C | T | 0.8871 | C | 0.1129 | 7 | 0.22581 |
| 10695 | S13_14662813 | 13 | 14662813 | 206299 | A | G | A | 0.75806 | G | 0.24194 | 11 | 0.35484 |
| 10696 | S13_14662819 | 13 | 14662819 | 6 | T | C | T | 0.79032 | C | 0.20968 | 11 | 0.35484 |
| 10697 | S13_14662861 | 13 | 14662861 | 42 | A | G | A | 0.91935 | G | 0.08065 | 5 | 0.16129 |
| 10698 | S13_14662928 | 13 | 14662928 | 67 | A | G | G | 0.51613 | A | 0.48387 | 18 | 0.58065 |
| 10699 | S13_14793773 | 13 | 14793773 | 130845 | C | G | G | 0.53226 | C | 0.46774 | 15 | 0.48387 |
| 10700 | S13_15151012 | 13 | 15151012 | 357239 | T | A | T | 0.85484 | A | 0.14516 | 5 | 0.16129 |
| 10701 | S13_15151101 | 13 | 15151101 | 89 | G | A | G | 0.85484 | A | 0.14516 | 5 | 0.16129 |
| 10702 | S13_15430320 | 13 | 15430320 | 279219 | G | A | G | 0.64516 | A | 0.35484 | 14 | 0.45161 |
| 10703 | S13_15430360 | 13 | 15430360 | 40 | A | C | C | 0.87097 | A | 0.12903 | 8 | 0.25806 |
| 10704 | S13_15430370 | 13 | 15430370 | 10 | C | T | C | 0.87097 | T | 0.12903 | 8 | 0.25806 |
| 10705 | S13_15430422 | 13 | 15430422 | 52 | T | A | T | 0.83871 | A | 0.16129 | 10 | 0.32258 |
| 10706 | S13_15781157 | 13 | 15781157 | 350735 | C | T | T | 0.80645 | C | 0.19355 | 8 | 0.25806 |
| 10707 | S13_15781254 | 13 | 15781254 | 97 | C | T | C | 0.85484 | T | 0.14516 | 7 | 0.22581 |
| 10708 | S13_15886748 | 13 | 15886748 | 105494 | C | T | C | 0.93548 | T | 0.06452 | 2 | 0.06452 |
| 10709 | S13_16379546 | 13 | 16379546 | 492798 | A | G | G | 0.8871 | A | 0.1129 | 7 | 0.22581 |
| 10710 | S13_16379548 | 13 | 16379548 | 2 | C | T | C | 0.56452 | T | 0.43548 | 17 | 0.54839 |
| 10711 | S13_16379550 | 13 | 16379550 | 2 | A | G | A | 0.90323 | G | 0.09677 | 6 | 0.19355 |
| 10712 | S13_16438809 | 13 | 16438809 | 59259 | A | T | A | 0.95161 | T | 0.04839 | 3 | 0.09677 |
| 10713 | S13_16666300 | 13 | 16666300 | 227491 | A | C | A | 0.53226 | C | 0.46774 | 15 | 0.48387 |
| 10714 | S13_16666508 | 13 | 16666508 | 208 | G | C | G | 0.93548 | C | 0.06452 | 4 | 0.12903 |
| 10715 | S13_16761500 | 13 | 16761500 | 94992 | C | T | C | 0.72581 | T | 0.27419 | 11 | 0.35484 |
| 10716 | S13_16761507 | 13 | 16761507 | 7 | A | G | A | 0.72581 | G | 0.27419 | 11 | 0.35484 |
| 10717 | S13_16761589 | 13 | 16761589 | 82 | T | C | T | 0.70968 | C | 0.29032 | 12 | 0.3871 |
| 10718 | S13_16761595 | 13 | 16761595 | 6 | A | C | A | 0.70968 | C | 0.29032 | 12 | 0.3871 |
| 10719 | S13_16761599 | 13 | 16761599 | 4 | C | G | C | 0.72581 | G | 0.27419 | 11 | 0.35484 |
| 10720 | S13_17495196 | 13 | 17495196 | 733597 | T | C | T | 0.8871 | C | 0.1129 | 7 | 0.22581 |
| 10721 | S13_17495372 | 13 | 17495372 | 176 | G | A | G | 0.91935 | A | 0.08065 | 5 | 0.16129 |
| 10722 | S13_17751870 | 13 | 17751870 | 256498 | T | A | T | 0.6129 | A | 0.3871 | 12 | 0.3871 |
| 10723 | S13_17828018 | 13 | 17828018 | 76148 | A | T | A | 0.79032 | T | 0.20968 | 7 | 0.22581 |
| 10724 | S13_17828044 | 13 | 17828044 | 26 | A | C | A | 0.95161 | C | 0.04839 | 3 | 0.09677 |
| 10725 | S13_18888926 | 13 | 18888926 | 1060882 | C | T | C | 0.83871 | T | 0.16129 | 8 | 0.25806 |
| 10726 | S13_18889093 | 13 | 18889093 | 167 | T | G | T | 0.83871 | G | 0.16129 | 8 | 0.25806 |
| 10727 | S13_18969851 | 13 | 18969851 | 80758 | C | G | C | 0.85484 | G | 0.14516 | 9 | 0.29032 |
| 10728 | S13_19124106 | 13 | 19124106 | 154255 | T | C | T | 0.69355 | C | 0.30645 | 9 | 0.29032 |
| 10729 | S13_19124132 | 13 | 19124132 | 26 | T | C | T | 0.91935 | C | 0.08065 | 3 | 0.09677 |
| 10730 | S13_19260522 | 13 | 19260522 | 136390 | G | T | G | 0.72581 | T | 0.27419 | 15 | 0.48387 |
| 10731 | S13_19260545 | 13 | 19260545 | 23 | G | A | G | 0.72581 | A | 0.27419 | 15 | 0.48387 |
| 10732 | S13_19260660 | 13 | 19260660 | 115 | A | G | A | 0.85484 | G | 0.14516 | 7 | 0.22581 |
| 10733 | S13_19260708 | 13 | 19260708 | 48 | C | T | C | 0.85484 | T | 0.14516 | 7 | 0.22581 |
| 10734 | S13_19523801 | 13 | 19523801 | 263093 | A | G | G | 0.51613 | A | 0.48387 | 12 | 0.3871 |
| 10735 | S13_19523845 | 13 | 19523845 | 44 | G | C | G | 0.53226 | C | 0.46774 | 11 | 0.35484 |
| 10736 | S13_19652787 | 13 | 19652787 | 128942 | C | A | C | 0.83871 | A | 0.16129 | 10 | 0.32258 |
| 10737 | S13_19652830 | 13 | 19652830 | 43 | G | A | G | 0.95161 | A | 0.04839 | 3 | 0.09677 |
| 10738 | S13_19833022 | 13 | 19833022 | 180192 | C | T | C | 0.82258 | T | 0.17742 | 11 | 0.35484 |
| 10739 | S13_19833128 | 13 | 19833128 | 106 | G | T | G | 0.82258 | T | 0.17742 | 11 | 0.35484 |
| 10740 | S13_19833177 | 13 | 19833177 | 49 | A | G | G | 0.93548 | A | 0.06452 | 4 | 0.12903 |
| 10741 | S13_19833217 | 13 | 19833217 | 40 | A | G | G | 0.93548 | A | 0.06452 | 4 | 0.12903 |
| 10742 | S13_19956946 | 13 | 19956946 | 123729 | G | A | G | 0.87097 | A | 0.12903 | 6 | 0.19355 |
| 10743 | S13_19956999 | 13 | 19956999 | 53 | A | G | G | 0.83871 | A | 0.16129 | 6 | 0.19355 |
| 10744 | S13_20809838 | 13 | 20809838 | 852839 | T | C | T | 0.91935 | C | 0.08065 | 5 | 0.16129 |

| Site number | SNP name | Chromosome | Physical position | Physical distance from the previous SNP | Reference allele | Alternative allele | Major allele | Major allele frequency | Minor allele | Minor allele frequency | Number heterozygous | Heterozygosity |
|---|---|---|---|---|---|---|---|---|---|---|---|---|
| 10745 | S13_20809921 | 13 | 20809921 | 83 | G | C | G | 0.91935 | C | 0.08065 | 5 | 0.16129 |
| 10746 | S13_20962114 | 13 | 20962114 | 152193 | A | G | A | 0.69355 | G | 0.30645 | 11 | 0.35484 |
| 10747 | S13_20962209 | 13 | 20962209 | 95 | C | A | C | 0.77419 | A | 0.22581 | 12 | 0.3871 |
| 10748 | S13_20962291 | 13 | 20962291 | 82 | T | C | T | 0.8871 | C | 0.1129 | 7 | 0.22581 |
| 10749 | S13_21100896 | 13 | 21100896 | 138605 | G | T | G | 0.95161 | T | 0.04839 | 3 | 0.09677 |
| 10750 | S13_21100906 | 13 | 21100906 | 10 | T | G | T | 0.83871 | G | 0.16129 | 8 | 0.25806 |
| 10751 | S13_21100967 | 13 | 21100967 | 61 | G | A | G | 0.95161 | A | 0.04839 | 3 | 0.09677 |
| 10752 | S13_21329698 | 13 | 21329698 | 228731 | A | G | A | 0.53226 | G | 0.46774 | 15 | 0.48387 |
| 10753 | S13_21329815 | 13 | 21329815 | 117 | T | G | T | 0.90323 | G | 0.09677 | 4 | 0.12903 |
| 10754 | S13_21378920 | 13 | 21378920 | 49105 | G | A | G | 0.87097 | A | 0.12903 | 6 | 0.19355 |
| 10755 | S13_21379082 | 13 | 21379082 | 162 | A | C | C | 0.56452 | A | 0.43548 | 15 | 0.48387 |
| 10756 | S13_21753048 | 13 | 21753048 | 373966 | T | C | T | 0.91935 | C | 0.08065 | 5 | 0.16129 |
| 10757 | S13_21845782 | 13 | 21845782 | 92734 | T | C | T | 0.93548 | C | 0.06452 | 4 | 0.12903 |
| 10758 | S13_22991514 | 13 | 22991514 | 1145732 | C | A | A | 0.70968 | C | 0.29032 | 12 | 0.3871 |
| 10759 | S13_23035373 | 13 | 23035373 | 43859 | C | A | C | 0.87097 | A | 0.12903 | 8 | 0.25806 |
| 10760 | S13_23035525 | 13 | 23035525 | 152 | G | A | A | 0.51613 | G | 0.48387 | 14 | 0.45161 |
| 10761 | S13_23270794 | 13 | 23270794 | 235269 | G | A | G | 0.93548 | A | 0.06452 | 2 | 0.06452 |
| 10762 | S13_23270848 | 13 | 23270848 | 54 | G | A | G | 0.87097 | A | 0.12903 | 6 | 0.19355 |
| 10763 | S13_23630944 | 13 | 23630944 | 360096 | T | A | T | 0.8871 | A | 0.1129 | 7 | 0.22581 |
| 10764 | S13_23631070 | 13 | 23631070 | 126 | G | A | G | 0.8871 | A | 0.1129 | 7 | 0.22581 |
| 10765 | S13_23736832 | 13 | 23736832 | 105762 | G | A | G | 0.90323 | A | 0.09677 | 6 | 0.19355 |
| 10766 | S13_23736911 | 13 | 23736911 | 79 | T | G | G | 0.79032 | T | 0.20968 | 9 | 0.29032 |
| 10767 | S13_24278517 | 13 | 24278517 | 541606 | C | T | C | 0.70968 | T | 0.29032 | 14 | 0.45161 |
| 10768 | S13_24278557 | 13 | 24278557 | 40 | G | A | G | 0.8871 | A | 0.1129 | 5 | 0.16129 |
| 10769 | S13_24559180 | 13 | 24559180 | 280623 | G | T | G | 0.95161 | T | 0.04839 | 3 | 0.09677 |
| 10770 | S13_24568800 | 13 | 24568800 | 9620 | G | A | G | 0.90323 | A | 0.09677 | 6 | 0.19355 |
| 10771 | S13_24568915 | 13 | 24568915 | 115 | A | G | A | 0.66129 | G | 0.33871 | 15 | 0.48387 |
| 10772 | S13_24653547 | 13 | 24653547 | 84632 | C | T | T | 0.67742 | C | 0.32258 | 16 | 0.51613 |
| 10773 | S13_24653620 | 13 | 24653620 | 73 | G | A | G | 0.93548 | A | 0.06452 | 4 | 0.12903 |
| 10774 | S13_25057408 | 13 | 25057408 | 403788 | T | C | T | 0.91935 | C | 0.08065 | 5 | 0.16129 |
| 10775 | S13_25057468 | 13 | 25057468 | 60 | G | A | G | 0.53226 | A | 0.46774 | 11 | 0.35484 |
| 10776 | S13_25057476 | 13 | 25057476 | 8 | T | C | T | 0.53226 | C | 0.46774 | 11 | 0.35484 |
| 10777 | S13_25174980 | 13 | 25174980 | 117504 | C | T | T | 0.56452 | C | 0.43548 | 19 | 0.6129 |
| 10778 | S13_25174981 | 13 | 25174981 | 1 | G | A | G | 0.90323 | A | 0.09677 | 4 | 0.12903 |
| 10779 | S13_25175012 | 13 | 25175012 | 31 | C | T | T | 0.70968 | C | 0.29032 | 14 | 0.45161 |
| 10780 | S13_25175136 | 13 | 25175136 | 124 | T | C | T | 0.90323 | C | 0.09677 | 6 | 0.19355 |
| 10781 | S13_25303869 | 13 | 25303869 | 128733 | A | G | A | 0.74194 | G | 0.25806 | 12 | 0.3871 |
| 10782 | S13_25304033 | 13 | 25304033 | 164 | C | T | C | 0.91935 | T | 0.08065 | 5 | 0.16129 |
| 10783 | S13_25304051 | 13 | 25304051 | 18 | T | C | C | 0.64516 | T | 0.35484 | 16 | 0.51613 |
| 10784 | S13_25304066 | 13 | 25304066 | 15 | C | T | C | 0.91935 | T | 0.08065 | 5 | 0.16129 |
| 10785 | S13_25705394 | 13 | 25705394 | 401328 | G | C | C | 0.85484 | G | 0.14516 | 7 | 0.22581 |
| 10786 | S13_25737016 | 13 | 25737016 | 31622 | A | G | G | 0.72581 | A | 0.27419 | 11 | 0.35484 |
| 10787 | S13_25737037 | 13 | 25737037 | 21 | T | A | A | 0.72581 | T | 0.27419 | 11 | 0.35484 |
| 10788 | S13_25737090 | 13 | 25737090 | 53 | T | A | A | 0.67742 | T | 0.32258 | 12 | 0.3871 |
| 10789 | S13_25737200 | 13 | 25737200 | 110 | C | A | A | 0.72581 | C | 0.27419 | 11 | 0.35484 |
| 10790 | S13_25745306 | 13 | 25745306 | 8106 | A | C | A | 0.90323 | C | 0.09677 | 4 | 0.12903 |
| 10791 | S13_25745384 | 13 | 25745384 | 78 | C | T | C | 0.85484 | T | 0.14516 | 9 | 0.29032 |
| 10792 | S13_25745424 | 13 | 25745424 | 40 | T | C | T | 0.85484 | C | 0.14516 | 9 | 0.29032 |
| 10793 | S13_26013982 | 13 | 26013982 | 268558 | G | A | G | 0.93548 | A | 0.06452 | 2 | 0.06452 |
| 10794 | S13_26059396 | 13 | 26059396 | 45414 | A | T | A | 0.69355 | T | 0.30645 | 9 | 0.29032 |
| 10795 | S13_26059403 | 13 | 26059403 | 7 | A | G | A | 0.69355 | G | 0.30645 | 9 | 0.29032 |
| 10796 | S13_26059431 | 13 | 26059431 | 28 | T | G | T | 0.69355 | G | 0.30645 | 9 | 0.29032 |
| 10797 | S13_26059476 | 13 | 26059476 | 45 | A | G | A | 0.69355 | G | 0.30645 | 9 | 0.29032 |
| 10798 | S13_26059614 | 13 | 26059614 | 138 | G | A | G | 0.69355 | A | 0.30645 | 9 | 0.29032 |

| Site number | SNP name | Chromosome | Physical position | Physical distance from the previous SNP | Reference allele | Alternative allele | Major allele | Major allele frequency | Minor allele | Minor allele frequency | Number heterozygous | Heterozygosity |
|---|---|---|---|---|---|---|---|---|---|---|---|---|
| 10799 | S13_26065360 | 13 | 26065360 | 5746 | C | T | C | 0.87097 | T | 0.12903 | 8 | 0.25806 |
| 10800 | S13_26068149 | 13 | 26068149 | 2789 | C | G | C | 0.59677 | G | 0.40323 | 13 | 0.41935 |
| 10801 | S13_26398861 | 13 | 26398861 | 330712 | C | T | C | 0.95161 | T | 0.04839 | 3 | 0.09677 |
| 10802 | S13_26446791 | 13 | 26446791 | 47930 | A | G | A | 0.90323 | G | 0.09677 | 6 | 0.19355 |
| 10803 | S13_26695639 | 13 | 26695639 | 248848 | G | A | G | 0.91935 | A | 0.08065 | 5 | 0.16129 |
| 10804 | S13_27113441 | 13 | 27113441 | 417802 | C | T | C | 0.93548 | T | 0.06452 | 4 | 0.12903 |
| 10805 | S13_27113511 | 13 | 27113511 | 70 | C | T | C | 0.93548 | T | 0.06452 | 4 | 0.12903 |
| 10806 | S13_27113523 | 13 | 27113523 | 12 | C | G | G | 0.83871 | C | 0.16129 | 8 | 0.25806 |
| 10807 | S13_27113611 | 13 | 27113611 | 88 | T | C | T | 0.74194 | C | 0.25806 | 10 | 0.32258 |
| 10808 | S13_27366268 | 13 | 27366268 | 252657 | G | A | G | 0.75806 | A | 0.24194 | 11 | 0.35484 |
| 10809 | S13_27390914 | 13 | 27390914 | 24646 | T | C | T | 0.87097 | C | 0.12903 | 6 | 0.19355 |
| 10810 | S13_27571332 | 13 | 27571332 | 180418 | G | T | G | 0.93548 | T | 0.06452 | 4 | 0.12903 |
| 10811 | S13_27974410 | 13 | 27974410 | 403078 | T | C | T | 0.93548 | C | 0.06452 | 4 | 0.12903 |
| 10812 | S13_28493702 | 13 | 28493702 | 519292 | T | G | T | 0.85484 | G | 0.14516 | 7 | 0.22581 |
| 10813 | S13_28524799 | 13 | 28524799 | 31097 | T | C | T | 0.72581 | C | 0.27419 | 13 | 0.41935 |
| 10814 | S13_28852223 | 13 | 28852223 | 327424 | C | T | T | 0.69355 | C | 0.30645 | 11 | 0.35484 |
| 10815 | S13_29023879 | 13 | 29023879 | 171656 | T | G | T | 0.80645 | G | 0.19355 | 10 | 0.32258 |
| 10816 | S13_29024038 | 13 | 29024038 | 159 | G | A | G | 0.80645 | A | 0.19355 | 8 | 0.25806 |
| 10817 | S13_29049373 | 13 | 29049373 | 25335 | C | T | T | 0.83871 | C | 0.16129 | 6 | 0.19355 |
| 10818 | S13_29422992 | 13 | 29422992 | 373619 | C | T | C | 0.85484 | T | 0.14516 | 9 | 0.29032 |
| 10819 | S13_29601454 | 13 | 29601454 | 178462 | T | C | T | 0.93548 | C | 0.06452 | 4 | 0.12903 |
| 10820 | S13_29616275 | 13 | 29616275 | 14821 | T | C | T | 0.91935 | C | 0.08065 | 3 | 0.09677 |
| 10821 | S13_29616284 | 13 | 29616284 | 9 | G | A | G | 0.95161 | A | 0.04839 | 3 | 0.09677 |
| 10822 | S13_29616413 | 13 | 29616413 | 129 | T | C | T | 0.93548 | C | 0.06452 | 4 | 0.12903 |
| 10823 | S13_29908101 | 13 | 29908101 | 291688 | A | G | A | 0.5 | G | 0.5 | 19 | 0.6129 |
| 10824 | S13_30609624 | 13 | 30609624 | 701523 | C | T | C | 0.91935 | T | 0.08065 | 5 | 0.16129 |
| 10825 | S13_30609709 | 13 | 30609709 | 85 | A | G | G | 0.74194 | A | 0.25806 | 14 | 0.45161 |
| 10826 | S13_30696713 | 13 | 30696713 | 87004 | C | T | C | 0.95161 | T | 0.04839 | 3 | 0.09677 |
| 10827 | S13_30696805 | 13 | 30696805 | 92 | C | T | C | 0.93548 | T | 0.06452 | 4 | 0.12903 |
| 10828 | S13_30710013 | 13 | 30710013 | 13208 | T | C | T | 0.95161 | C | 0.04839 | 3 | 0.09677 |
| 10829 | S13_30966684 | 13 | 30966684 | 256671 | C | T | C | 0.82258 | T | 0.17742 | 11 | 0.35484 |
| 10830 | S13_30966732 | 13 | 30966732 | 48 | T | C | C | 0.5 | T | 0.5 | 19 | 0.6129 |
| 10831 | S13_30966844 | 13 | 30966844 | 112 | A | C | A | 0.5 | C | 0.5 | 19 | 0.6129 |
| 10832 | S13_31072743 | 13 | 31072743 | 105899 | C | A | C | 0.90323 | A | 0.09677 | 4 | 0.12903 |
| 10833 | S13_31134716 | 13 | 31134716 | 61973 | G | A | G | 0.79032 | A | 0.20968 | 11 | 0.35484 |
| 10834 | S13_31134737 | 13 | 31134737 | 21 | T | C | T | 0.87097 | C | 0.12903 | 8 | 0.25806 |
| 10835 | S13_31493719 | 13 | 31493719 | 358982 | G | A | G | 0.72581 | A | 0.27419 | 15 | 0.48387 |
| 10836 | S13_31727918 | 13 | 31727918 | 234199 | G | A | G | 0.91935 | A | 0.08065 | 5 | 0.16129 |
| 10837 | S13_31838538 | 13 | 31838538 | 110620 | G | A | G | 0.67742 | A | 0.32258 | 14 | 0.45161 |
| 10838 | S13_32116735 | 13 | 32116735 | 278197 | T | G | T | 0.91935 | G | 0.08065 | 5 | 0.16129 |
| 10839 | S13_32116761 | 13 | 32116761 | 26 | G | A | G | 0.70968 | A | 0.29032 | 10 | 0.32258 |
| 10840 | S13_32189326 | 13 | 32189326 | 72565 | G | A | G | 0.90323 | A | 0.09677 | 6 | 0.19355 |
| 10841 | S13_32336746 | 13 | 32336746 | 147420 | T | C | T | 0.77419 | C | 0.22581 | 8 | 0.25806 |
| 10842 | S13_32336827 | 13 | 32336827 | 81 | A | G | G | 0.90323 | A | 0.09677 | 6 | 0.19355 |
| 10843 | S13_32336910 | 13 | 32336910 | 83 | G | A | G | 0.8871 | A | 0.1129 | 5 | 0.16129 |
| 10844 | S13_32361605 | 13 | 32361605 | 24695 | G | A | G | 0.93548 | A | 0.06452 | 4 | 0.12903 |
| 10845 | S13_32361796 | 13 | 32361796 | 191 | A | C | A | 0.53226 | C | 0.46774 | 13 | 0.41935 |
| 10846 | S13_32369261 | 13 | 32369261 | 7465 | A | G | G | 0.91935 | A | 0.08065 | 5 | 0.16129 |
| 10847 | S13_32369289 | 13 | 32369289 | 28 | A | G | G | 0.91935 | A | 0.08065 | 5 | 0.16129 |
| 10848 | S13_32369400 | 13 | 32369400 | 111 | T | C | T | 0.8871 | C | 0.1129 | 5 | 0.16129 |
| 10849 | S13_32369433 | 13 | 32369433 | 33 | T | C | C | 0.91935 | T | 0.08065 | 5 | 0.16129 |
| 10850 | S13_32807865 | 13 | 32807865 | 438432 | G | A | G | 0.72581 | A | 0.27419 | 13 | 0.41935 |
| 10851 | S13_33264297 | 13 | 33264297 | 456432 | T | C | T | 0.80645 | C | 0.19355 | 8 | 0.25806 |
| 10852 | S13_33264330 | 13 | 33264330 | 33 | C | T | C | 0.80645 | T | 0.19355 | 8 | 0.25806 |

| Site number | SNP name | Chromosome | Physical position | Physical distance from the previous SNP | Reference allele | Alternative allele | Major allele | Major allele frequency | Minor allele | Minor allele frequency | Number heterozygous | Heterozygosity |
|---|---|---|---|---|---|---|---|---|---|---|---|---|
| 10853 | S13_33383352 | 13 | 33383352 | 119022 | G | T | T | 0.58065 | G | 0.41935 | 16 | 0.51613 |
| 10854 | S13_33471906 | 13 | 33471906 | 88554 | G | T | T | 0.69355 | G | 0.30645 | 9 | 0.29032 |
| 10855 | S13_33471987 | 13 | 33471987 | 81 | G | T | T | 0.69355 | G | 0.30645 | 9 | 0.29032 |
| 10856 | S13_33472110 | 13 | 33472110 | 123 | T | C | T | 0.58065 | C | 0.41935 | 14 | 0.45161 |
| 10857 | S13_34010271 | 13 | 34010271 | 538161 | T | G | G | 0.72581 | T | 0.27419 | 11 | 0.35484 |
| 10858 | S13_34010277 | 13 | 34010277 | 6 | T | C | T | 0.80645 | C | 0.19355 | 8 | 0.25806 |
| 10859 | S13_34010279 | 13 | 34010279 | 2 | T | G | G | 0.72581 | T | 0.27419 | 11 | 0.35484 |
| 10860 | S13_34010310 | 13 | 34010310 | 31 | A | G | G | 0.72581 | A | 0.27419 | 11 | 0.35484 |
| 10861 | S13_34085913 | 13 | 34085913 | 75603 | C | T | C | 0.69355 | T | 0.30645 | 9 | 0.29032 |
| 10862 | S13_34085929 | 13 | 34085929 | 16 | T | C | T | 0.83871 | C | 0.16129 | 8 | 0.25806 |
| 10863 | S13_34303335 | 13 | 34303335 | 217406 | C | T | C | 0.75806 | T | 0.24194 | 15 | 0.48387 |
| 10864 | S13_34303397 | 13 | 34303397 | 62 | T | A | T | 0.91935 | A | 0.08065 | 5 | 0.16129 |
| 10865 | S13_34650092 | 13 | 34650092 | 346695 | T | C | C | 0.6129 | T | 0.3871 | 16 | 0.51613 |
| 10866 | S13_34831869 | 13 | 34831869 | 181777 | C | T | C | 0.95161 | T | 0.04839 | 3 | 0.09677 |
| 10867 | S13_34848561 | 13 | 34848561 | 16692 | T | C | T | 0.66129 | C | 0.33871 | 15 | 0.48387 |
| 10868 | S13_34848627 | 13 | 34848627 | 66 | G | A | G | 0.72581 | A | 0.27419 | 15 | 0.48387 |
| 10869 | S13_34964231 | 13 | 34964231 | 115604 | C | T | C | 0.82258 | T | 0.17742 | 9 | 0.29032 |
| 10870 | S13_35413138 | 13 | 35413138 | 448907 | G | A | G | 0.54839 | A | 0.45161 | 18 | 0.58065 |
| 10871 | S13_35701832 | 13 | 35701832 | 288694 | A | G | A | 0.54839 | G | 0.45161 | 18 | 0.58065 |
| 10872 | S13_35701869 | 13 | 35701869 | 37 | A | C | A | 0.54839 | C | 0.45161 | 12 | 0.3871 |
| 10873 | S13_35701954 | 13 | 35701954 | 85 | G | T | G | 0.54839 | T | 0.45161 | 14 | 0.45161 |
| 10874 | S13_35702036 | 13 | 35702036 | 82 | G | A | G | 0.56452 | A | 0.43548 | 13 | 0.41935 |
| 10875 | S13_36405910 | 13 | 36405910 | 703874 | C | T | T | 0.8871 | C | 0.1129 | 7 | 0.22581 |
| 10876 | S13_36405939 | 13 | 36405939 | 29 | A | T | A | 0.83871 | T | 0.16129 | 10 | 0.32258 |
| 10877 | S13_37001937 | 13 | 37001937 | 595998 | A | G | A | 0.79032 | G | 0.20968 | 9 | 0.29032 |
| 10878 | S13_37001950 | 13 | 37001950 | 13 | C | T | C | 0.8871 | T | 0.1129 | 5 | 0.16129 |
| 10879 | S13_37001987 | 13 | 37001987 | 37 | G | A | G | 0.64516 | A | 0.35484 | 12 | 0.3871 |
| 10880 | S13_37001994 | 13 | 37001994 | 7 | A | G | A | 0.64516 | G | 0.35484 | 12 | 0.3871 |
| 10881 | S13_37002011 | 13 | 37002011 | 17 | T | G | T | 0.64516 | G | 0.35484 | 12 | 0.3871 |
| 10882 | S13_37070796 | 13 | 37070796 | 68785 | G | A | G | 0.83871 | A | 0.16129 | 8 | 0.25806 |
| 10883 | S13_37070835 | 13 | 37070835 | 39 | A | G | G | 0.58065 | A | 0.41935 | 16 | 0.51613 |
| 10884 | S13_37070872 | 13 | 37070872 | 37 | C | T | T | 0.58065 | C | 0.41935 | 16 | 0.51613 |
| 10885 | S13_37070893 | 13 | 37070893 | 21 | C | T | C | 0.64516 | T | 0.35484 | 14 | 0.45161 |
| 10886 | S13_37200993 | 13 | 37200993 | 130100 | T | C | C | 0.87097 | T | 0.12903 | 8 | 0.25806 |
| 10887 | S13_37218895 | 13 | 37218895 | 17902 | T | G | G | 0.93548 | T | 0.06452 | 4 | 0.12903 |
| 10888 | S13_37468451 | 13 | 37468451 | 249556 | A | G | A | 0.87097 | G | 0.12903 | 6 | 0.19355 |
| 10889 | S13_37468484 | 13 | 37468484 | 33 | C | A | C | 0.90323 | A | 0.09677 | 6 | 0.19355 |
| 10890 | S13_37910920 | 13 | 37910920 | 442436 | T | C | T | 0.8871 | C | 0.1129 | 7 | 0.22581 |
| 10891 | S13_37932588 | 13 | 37932588 | 21668 | C | T | C | 0.90323 | T | 0.09677 | 6 | 0.19355 |
| 10892 | S13_37932620 | 13 | 37932620 | 32 | C | T | C | 0.93548 | T | 0.06452 | 4 | 0.12903 |
| 10893 | S13_38023293 | 13 | 38023293 | 90673 | T | C | T | 0.64516 | C | 0.35484 | 10 | 0.32258 |
| 10894 | S13_38023294 | 13 | 38023294 | 1 | G | A | G | 0.90323 | A | 0.09677 | 6 | 0.19355 |
| 10895 | S13_38054575 | 13 | 38054575 | 31281 | T | G | T | 0.83871 | G | 0.16129 | 6 | 0.19355 |
| 10896 | S13_38092324 | 13 | 38092324 | 37749 | G | A | G | 0.87097 | A | 0.12903 | 4 | 0.12903 |
| 10897 | S13_38115542 | 13 | 38115542 | 23218 | C | T | C | 0.95161 | T | 0.04839 | 3 | 0.09677 |
| 10898 | S13_38470745 | 13 | 38470745 | 355203 | A | G | A | 0.53226 | G | 0.46774 | 17 | 0.54839 |
| 10899 | S13_39052987 | 13 | 39052987 | 582242 | G | T | T | 0.77419 | G | 0.22581 | 10 | 0.32258 |
| 10900 | S13_39225005 | 13 | 39225005 | 172018 | C | T | C | 0.79032 | T | 0.20968 | 11 | 0.35484 |
| 10901 | S13_39345677 | 13 | 39345677 | 120672 | T | A | A | 0.5 | T | 0.5 | 17 | 0.54839 |
| 10902 | S13_39345686 | 13 | 39345686 | 9 | G | A | G | 0.85484 | A | 0.14516 | 5 | 0.16129 |
| 10903 | S13_39345755 | 13 | 39345755 | 69 | T | C | T | 0.87097 | C | 0.12903 | 8 | 0.25806 |
| 10904 | S13_39383542 | 13 | 39383542 | 37787 | T | A | T | 0.87097 | A | 0.12903 | 6 | 0.19355 |
| 10905 | S13_39383648 | 13 | 39383648 | 106 | T | C | T | 0.87097 | C | 0.12903 | 6 | 0.19355 |
| 10906 | S13_39520642 | 13 | 39520642 | 136994 | C | T | C | 0.83871 | T | 0.16129 | 8 | 0.25806 |

| Site number | SNP name | Chromosome | Physical position | Physical distance from the previous SNP | Reference allele | Alternative allele | Major allele | Major allele frequency | Minor allele | Minor allele frequency | Number heterozygous | Heterozygosity |
|---|---|---|---|---|---|---|---|---|---|---|---|---|
| 10907 | S13_39597304 | 13 | 39597304 | 76662 | G | A | G | 0.90323 | A | 0.09677 | 6 | 0.19355 |
| 10908 | S13_39597467 | 13 | 39597467 | 163 | T | A | T | 0.93548 | A | 0.06452 | 4 | 0.12903 |
| 10909 | S13_39689735 | 13 | 39689735 | 92268 | G | T | G | 0.80645 | T | 0.19355 | 10 | 0.32258 |
| 10910 | S13_39740426 | 13 | 39740426 | 50691 | G | A | G | 0.95161 | A | 0.04839 | 1 | 0.03226 |
| 10911 | S13_39740432 | 13 | 39740432 | 6 | A | G | G | 0.70968 | A | 0.29032 | 12 | 0.3871 |
| 10912 | S13_39920530 | 13 | 39920530 | 180098 | C | T | C | 0.91935 | T | 0.08065 | 5 | 0.16129 |
| 10913 | S13_39923844 | 13 | 39923844 | 3314 | T | A | T | 0.83871 | A | 0.16129 | 8 | 0.25806 |
| 10914 | S13_39923885 | 13 | 39923885 | 41 | C | T | C | 0.8871 | T | 0.1129 | 7 | 0.22581 |
| 10915 | S13_40025140 | 13 | 40025140 | 101255 | G | A | A | 0.51613 | G | 0.48387 | 18 | 0.58065 |
| 10916 | S13_40138281 | 13 | 40138281 | 113141 | A | G | G | 0.64516 | A | 0.35484 | 16 | 0.51613 |
| 10917 | S13_40936804 | 13 | 40936804 | 798523 | T | C | T | 0.77419 | C | 0.22581 | 10 | 0.32258 |
| 10918 | S13_40937000 | 13 | 40937000 | 196 | T | C | T | 0.77419 | C | 0.22581 | 10 | 0.32258 |
| 10919 | S13_40988966 | 13 | 40988966 | 51966 | T | C | C | 0.51613 | T | 0.48387 | 14 | 0.45161 |
| 10920 | S13_40989080 | 13 | 40989080 | 114 | C | T | C | 0.59677 | T | 0.40323 | 13 | 0.41935 |
| 10921 | S13_40989182 | 13 | 40989182 | 102 | G | A | G | 0.79032 | A | 0.20968 | 9 | 0.29032 |
| 10922 | S13_40990738 | 13 | 40990738 | 1556 | T | C | T | 0.91935 | C | 0.08065 | 5 | 0.16129 |
| 10923 | S13_40990752 | 13 | 40990752 | 14 | C | G | C | 0.91935 | G | 0.08065 | 5 | 0.16129 |
| 10924 | S13_40990805 | 13 | 40990805 | 53 | C | T | C | 0.53226 | T | 0.46774 | 13 | 0.41935 |
| 10925 | S13_40990894 | 13 | 40990894 | 89 | T | C | T | 0.91935 | C | 0.08065 | 5 | 0.16129 |
| 10926 | S13_40990913 | 13 | 40990913 | 19 | T | G | G | 0.77419 | T | 0.22581 | 12 | 0.3871 |
| 10927 | S13_40990937 | 13 | 40990937 | 24 | G | T | G | 0.54839 | T | 0.45161 | 14 | 0.45161 |
| 10928 | S13_40990938 | 13 | 40990938 | 1 | G | A | G | 0.54839 | A | 0.45161 | 14 | 0.45161 |
| 10929 | S13_41111209 | 13 | 41111209 | 120271 | A | T | A | 0.58065 | T | 0.41935 | 18 | 0.58065 |
| 10930 | S13_41280300 | 13 | 41280300 | 169091 | G | A | G | 0.77419 | A | 0.22581 | 8 | 0.25806 |
| 10931 | S13_41280337 | 13 | 41280337 | 37 | G | A | G | 0.87097 | A | 0.12903 | 6 | 0.19355 |
| 10932 | S13_41280352 | 13 | 41280352 | 15 | C | T | T | 0.6129 | C | 0.3871 | 14 | 0.45161 |
| 10933 | S13_41303932 | 13 | 41303932 | 23580 | C | T | C | 0.66129 | T | 0.33871 | 13 | 0.41935 |
| 10934 | S13_41547156 | 13 | 41547156 | 243224 | T | A | T | 0.91935 | A | 0.08065 | 5 | 0.16129 |
| 10935 | S13_41547263 | 13 | 41547263 | 107 | C | T | C | 0.8871 | T | 0.1129 | 7 | 0.22581 |
| 10936 | S13_41623807 | 13 | 41623807 | 76544 | C | T | C | 0.90323 | T | 0.09677 | 6 | 0.19355 |
| 10937 | S13_41700510 | 13 | 41700510 | 76703 | A | G | A | 0.70968 | G | 0.29032 | 14 | 0.45161 |
| 10938 | S13_41700521 | 13 | 41700521 | 11 | A | G | A | 0.91935 | G | 0.08065 | 5 | 0.16129 |
| 10939 | S13_41727686 | 13 | 41727686 | 27165 | A | G | A | 0.95161 | G | 0.04839 | 3 | 0.09677 |
| 10940 | S13_41727707 | 13 | 41727707 | 21 | G | A | G | 0.93548 | A | 0.06452 | 4 | 0.12903 |
| 10941 | S13_41727852 | 13 | 41727852 | 145 | A | G | A | 0.95161 | G | 0.04839 | 3 | 0.09677 |
| 10942 | S13_41727854 | 13 | 41727854 | 2 | C | T | C | 0.93548 | T | 0.06452 | 4 | 0.12903 |
| 10943 | S13_41727890 | 13 | 41727890 | 36 | T | C | C | 0.69355 | T | 0.30645 | 15 | 0.48387 |
| 10944 | S13_41727903 | 13 | 41727903 | 13 | T | C | T | 0.95161 | C | 0.04839 | 3 | 0.09677 |
| 10945 | S13_41804492 | 13 | 41804492 | 76589 | G | A | G | 0.93548 | A | 0.06452 | 4 | 0.12903 |
| 10946 | S13_41804513 | 13 | 41804513 | 21 | C | T | C | 0.70968 | T | 0.29032 | 12 | 0.3871 |
| 10947 | S13_42022622 | 13 | 42022622 | 218109 | G | A | G | 0.79032 | A | 0.20968 | 11 | 0.35484 |
| 10948 | S13_42110838 | 13 | 42110838 | 88216 | G | T | T | 0.69355 | G | 0.30645 | 11 | 0.35484 |
| 10949 | S13_43462316 | 13 | 43462316 | 1351478 | A | G | A | 0.93548 | G | 0.06452 | 4 | 0.12903 |
| 10950 | S13_43462504 | 13 | 43462504 | 188 | G | A | G | 0.83871 | A | 0.16129 | 6 | 0.19355 |
| 10951 | S13_43821961 | 13 | 43821961 | 359457 | T | C | T | 0.79032 | C | 0.20968 | 11 | 0.35484 |
| 10952 | S13_44071093 | 13 | 44071093 | 249132 | G | A | G | 0.91935 | A | 0.08065 | 5 | 0.16129 |
| 10953 | S13_44071123 | 13 | 44071123 | 30 | G | A | G | 0.77419 | A | 0.22581 | 8 | 0.25806 |
| 10954 | S13_44502063 | 13 | 44502063 | 430940 | A | G | A | 0.70968 | G | 0.29032 | 16 | 0.51613 |
| 10955 | S13_44619641 | 13 | 44619641 | 117578 | C | T | C | 0.93548 | T | 0.06452 | 4 | 0.12903 |
| 10956 | S13_44619711 | 13 | 44619711 | 70 | T | C | T | 0.53226 | C | 0.46774 | 13 | 0.41935 |
| 10957 | S13_44942992 | 13 | 44942992 | 323281 | T | C | T | 0.90323 | C | 0.09677 | 6 | 0.19355 |
| 10958 | S13_44961165 | 13 | 44961165 | 18173 | C | T | C | 0.62903 | T | 0.37097 | 9 | 0.29032 |
| 10959 | S13_44961226 | 13 | 44961226 | 61 | C | T | C | 0.85484 | T | 0.14516 | 7 | 0.22581 |
| 10960 | S13_45231366 | 13 | 45231366 | 270140 | C | T | C | 0.67742 | T | 0.32258 | 14 | 0.45161 |

| Site number | SNP name | Chromosome | Physical position | Physical distance from the previous SNP | Reference allele | Alternative allele | Major allele | Major allele frequency | Minor allele | Minor allele frequency | Number heterozygous | Heterozygosity |
|---|---|---|---|---|---|---|---|---|---|---|---|---|
| 10961 | S13_45541603 | 13 | 45541603 | 310237 | G | A | G | 0.93548 | A | 0.06452 | 2 | 0.06452 |
| 10962 | S13_45541676 | 13 | 45541676 | 73 | C | G | C | 0.72581 | G | 0.27419 | 11 | 0.35484 |
| 10963 | S13_45644327 | 13 | 45644327 | 102651 | C | T | C | 0.93548 | T | 0.06452 | 2 | 0.06452 |
| 10964 | S13_45644354 | 13 | 45644354 | 27 | G | A | G | 0.91935 | A | 0.08065 | 5 | 0.16129 |
| 10965 | S13_45644510 | 13 | 45644510 | 156 | C | T | C | 0.72581 | T | 0.27419 | 15 | 0.48387 |
| 10966 | S13_45650929 | 13 | 45650929 | 6419 | C | T | C | 0.93548 | T | 0.06452 | 2 | 0.06452 |
| 10967 | S13_45671909 | 13 | 45671909 | 20980 | G | T | G | 0.8871 | T | 0.1129 | 7 | 0.22581 |
| 10968 | S13_45672007 | 13 | 45672007 | 98 | G | A | G | 0.70968 | A | 0.29032 | 14 | 0.45161 |
| 10969 | S13_45737811 | 13 | 45737811 | 65804 | C | A | C | 0.95161 | A | 0.04839 | 3 | 0.09677 |
| 10970 | S13_45937847 | 13 | 45937847 | 200036 | C | G | G | 0.53226 | C | 0.46774 | 15 | 0.48387 |
| 10971 | S13_45938891 | 13 | 45938891 | 1044 | C | T | C | 0.91935 | T | 0.08065 | 5 | 0.16129 |
| 10972 | S13_45938936 | 13 | 45938936 | 45 | C | T | T | 0.53226 | C | 0.46774 | 15 | 0.48387 |
| 10973 | S13_46077887 | 13 | 46077887 | 138951 | A | G | G | 0.93548 | A | 0.06452 | 4 | 0.12903 |
| 10974 | S13_46077920 | 13 | 46077920 | 33 | G | A | G | 0.91935 | A | 0.08065 | 5 | 0.16129 |
| 10975 | S13_46388049 | 13 | 46388049 | 310129 | G | A | A | 0.79032 | G | 0.20968 | 9 | 0.29032 |
| 10976 | S13_46388065 | 13 | 46388065 | 16 | T | C | T | 0.91935 | C | 0.08065 | 5 | 0.16129 |
| 10977 | S13_46388166 | 13 | 46388166 | 101 | A | C | C | 0.87097 | A | 0.12903 | 8 | 0.25806 |
| 10978 | S13_46659502 | 13 | 46659502 | 271336 | C | T | C | 0.90323 | T | 0.09677 | 4 | 0.12903 |
| 10979 | S13_46874668 | 13 | 46874668 | 215166 | T | C | T | 0.82258 | C | 0.17742 | 9 | 0.29032 |
| 10980 | S13_46874887 | 13 | 46874887 | 219 | G | A | G | 0.80645 | A | 0.19355 | 10 | 0.32258 |
| 10981 | S13_46878216 | 13 | 46878216 | 3329 | T | C | T | 0.79032 | C | 0.20968 | 9 | 0.29032 |
| 10982 | S13_46878248 | 13 | 46878248 | 32 | G | A | G | 0.87097 | A | 0.12903 | 6 | 0.19355 |
| 10983 | S13_46878284 | 13 | 46878284 | 36 | A | G | A | 0.79032 | G | 0.20968 | 9 | 0.29032 |
| 10984 | S13_46968544 | 13 | 46968544 | 90260 | T | C | C | 0.70968 | T | 0.29032 | 14 | 0.45161 |
| 10985 | S13_47372651 | 13 | 47372651 | 404107 | T | C | T | 0.72581 | C | 0.27419 | 15 | 0.48387 |
| 10986 | S13_47372655 | 13 | 47372655 | 4 | C | T | C | 0.74194 | T | 0.25806 | 12 | 0.3871 |
| 10987 | S13_47372675 | 13 | 47372675 | 20 | T | G | T | 0.80645 | G | 0.19355 | 10 | 0.32258 |
| 10988 | S13_47440449 | 13 | 47440449 | 67774 | A | G | G | 0.70968 | A | 0.29032 | 16 | 0.51613 |
| 10989 | S13_47440529 | 13 | 47440529 | 80 | A | G | A | 0.83871 | G | 0.16129 | 10 | 0.32258 |
| 10990 | S13_47480192 | 13 | 47480192 | 39663 | C | G | C | 0.77419 | G | 0.22581 | 12 | 0.3871 |
| 10991 | S13_48406266 | 13 | 48406266 | 926074 | G | A | G | 0.93548 | A | 0.06452 | 4 | 0.12903 |
| 10992 | S13_48406339 | 13 | 48406339 | 73 | T | C | T | 0.95161 | C | 0.04839 | 3 | 0.09677 |
| 10993 | S13_49911568 | 13 | 49911568 | 1505229 | T | C | C | 0.5 | T | 0.5 | 15 | 0.48387 |
| 10994 | S13_50283095 | 13 | 50283095 | 371527 | G | C | G | 0.70968 | C | 0.29032 | 10 | 0.32258 |
| 10995 | S13_50389692 | 13 | 50389692 | 106597 | A | G | G | 0.67742 | A | 0.32258 | 16 | 0.51613 |
| 10996 | S13_50389881 | 13 | 50389881 | 189 | G | A | A | 0.67742 | G | 0.32258 | 16 | 0.51613 |
| 10997 | S13_50452260 | 13 | 50452260 | 62379 | C | A | C | 0.93548 | A | 0.06452 | 4 | 0.12903 |
| 10998 | S13_50476835 | 13 | 50476835 | 24575 | A | G | A | 0.82258 | G | 0.17742 | 9 | 0.29032 |
| 10999 | S13_50476900 | 13 | 50476900 | 65 | G | C | G | 0.82258 | C | 0.17742 | 9 | 0.29032 |
| 11000 | S13_50476909 | 13 | 50476909 | 9 | C | A | C | 0.82258 | A | 0.17742 | 9 | 0.29032 |
| 11001 | S13_51964261 | 13 | 51964261 | 1487352 | A | G | A | 0.91935 | G | 0.08065 | 5 | 0.16129 |
| 11002 | S13_51964289 | 13 | 51964289 | 28 | C | T | C | 0.91935 | T | 0.08065 | 5 | 0.16129 |
| 11003 | S13_51964377 | 13 | 51964377 | 88 | C | T | C | 0.95161 | T | 0.04839 | 3 | 0.09677 |
| 11004 | S13_52075732 | 13 | 52075732 | 111355 | T | C | C | 0.95161 | T | 0.04839 | 3 | 0.09677 |
| 11005 | S13_52075744 | 13 | 52075744 | 12 | G | A | G | 0.95161 | A | 0.04839 | 3 | 0.09677 |
| 11006 | S13_52075949 | 13 | 52075949 | 205 | C | T | C | 0.90323 | T | 0.09677 | 6 | 0.19355 |
| 11007 | S13_52180979 | 13 | 52180979 | 105030 | C | T | T | 0.54839 | C | 0.45161 | 14 | 0.45161 |
| 11008 | S13_52180984 | 13 | 52180984 | 5 | C | T | C | 0.8871 | T | 0.1129 | 7 | 0.22581 |
| 11009 | S13_52412099 | 13 | 52412099 | 231115 | G | C | G | 0.67742 | C | 0.32258 | 14 | 0.45161 |
| 11010 | S13_53066813 | 13 | 53066813 | 654714 | T | C | T | 0.72581 | C | 0.27419 | 13 | 0.41935 |
| 11011 | S13_53581557 | 13 | 53581557 | 514744 | C | T | C | 0.91935 | T | 0.08065 | 5 | 0.16129 |
| 11012 | S13_53983929 | 13 | 53983929 | 402372 | G | A | A | 0.64516 | G | 0.35484 | 16 | 0.51613 |
| 11013 | S13_54401459 | 13 | 54401459 | 417530 | G | T | G | 0.8871 | T | 0.1129 | 7 | 0.22581 |
| 11014 | S13_54401522 | 13 | 54401522 | 63 | C | A | A | 0.77419 | C | 0.22581 | 12 | 0.3871 |

| Site number | SNP name | Chromosome | Physical position | Physical distance from the previous SNP | Reference allele | Alternative allele | Major allele | Major allele frequency | Minor allele | Minor allele frequency | Number heterozygous | Heterozygosity |
|---|---|---|---|---|---|---|---|---|---|---|---|---|
| 11015 | S13_54401587 | 13 | 54401587 | 65 | T | C | T | 0.85484 | C | 0.14516 | 7 | 0.22581 |
| 11016 | S13_54401640 | 13 | 54401640 | 53 | C | T | C | 0.72581 | T | 0.27419 | 11 | 0.35484 |
| 11017 | S13_54409223 | 13 | 54409223 | 7583 | A | G | A | 0.62903 | G | 0.37097 | 13 | 0.41935 |
| 11018 | S13_54865241 | 13 | 54865241 | 456018 | A | G | A | 0.91935 | G | 0.08065 | 5 | 0.16129 |
| 11019 | S13_54867495 | 13 | 54867495 | 2254 | A | G | G | 0.66129 | A | 0.33871 | 19 | 0.6129 |
| 11020 | S13_54867535 | 13 | 54867535 | 40 | C | T | C | 0.8871 | T | 0.1129 | 7 | 0.22581 |
| 11021 | S13_54995768 | 13 | 54995768 | 128233 | C | T | C | 0.87097 | T | 0.12903 | 6 | 0.19355 |
| 11022 | S13_55028817 | 13 | 55028817 | 33049 | G | A | G | 0.85484 | A | 0.14516 | 7 | 0.22581 |
| 11023 | S13_55258010 | 13 | 55258010 | 229193 | C | T | C | 0.93548 | T | 0.06452 | 4 | 0.12903 |
| 11024 | S13_55258064 | 13 | 55258064 | 54 | T | C | C | 0.75806 | T | 0.24194 | 13 | 0.41935 |
| 11025 | S13_55258177 | 13 | 55258177 | 113 | G | T | G | 0.91935 | T | 0.08065 | 5 | 0.16129 |
| 11026 | S13_55258192 | 13 | 55258192 | 15 | A | G | A | 0.70968 | G | 0.29032 | 14 | 0.45161 |
| 11027 | S13_55332648 | 13 | 55332648 | 74456 | G | A | A | 0.90323 | G | 0.09677 | 6 | 0.19355 |
| 11028 | S13_55347719 | 13 | 55347719 | 15071 | C | T | C | 0.67742 | T | 0.32258 | 14 | 0.45161 |
| 11029 | S13_55347844 | 13 | 55347844 | 125 | C | T | T | 0.91935 | C | 0.08065 | 5 | 0.16129 |
| 11030 | S13_55347895 | 13 | 55347895 | 51 | T | C | C | 0.64516 | T | 0.35484 | 20 | 0.64516 |
| 11031 | S13_55422646 | 13 | 55422646 | 74751 | A | G | A | 0.93548 | G | 0.06452 | 2 | 0.06452 |
| 11032 | S13_55422766 | 13 | 55422766 | 120 | T | C | C | 0.90323 | T | 0.09677 | 6 | 0.19355 |
| 11033 | S13_55422772 | 13 | 55422772 | 6 | T | A | T | 0.69355 | A | 0.30645 | 13 | 0.41935 |
| 11034 | S13_55516383 | 13 | 55516383 | 93611 | G | A | G | 0.74194 | A | 0.25806 | 10 | 0.32258 |
| 11035 | S13_55587472 | 13 | 55587472 | 71089 | G | A | G | 0.82258 | A | 0.17742 | 7 | 0.22581 |
| 11036 | S13_55587648 | 13 | 55587648 | 176 | A | T | A | 0.85484 | T | 0.14516 | 9 | 0.29032 |
| 11037 | S13_55947903 | 13 | 55947903 | 360255 | A | G | A | 0.83871 | G | 0.16129 | 8 | 0.25806 |
| 11038 | S13_55948039 | 13 | 55948039 | 136 | G | A | G | 0.87097 | A | 0.12903 | 6 | 0.19355 |
| 11039 | S13_55948044 | 13 | 55948044 | 5 | G | A | G | 0.90323 | A | 0.09677 | 6 | 0.19355 |
| 11040 | S13_55948092 | 13 | 55948092 | 48 | C | T | C | 0.87097 | T | 0.12903 | 6 | 0.19355 |
| 11041 | S13_56087824 | 13 | 56087824 | 139732 | G | A | A | 0.54839 | G | 0.45161 | 16 | 0.51613 |
| 11042 | S13_56088011 | 13 | 56088011 | 187 | T | C | T | 0.93548 | C | 0.06452 | 4 | 0.12903 |
| 11043 | S13_56219370 | 13 | 56219370 | 131359 | T | A | T | 0.93548 | A | 0.06452 | 4 | 0.12903 |
| 11044 | S13_56270069 | 13 | 56270069 | 50699 | G | T | G | 0.87097 | T | 0.12903 | 8 | 0.25806 |
| 11045 | S13_56338679 | 13 | 56338679 | 68610 | T | C | T | 0.72581 | C | 0.27419 | 13 | 0.41935 |
| 11046 | S13_56461275 | 13 | 56461275 | 122596 | C | T | C | 0.87097 | T | 0.12903 | 8 | 0.25806 |
| 11047 | S13_56461337 | 13 | 56461337 | 62 | T | C | T | 0.87097 | C | 0.12903 | 8 | 0.25806 |
| 11048 | S13_56681824 | 13 | 56681824 | 220487 | C | T | C | 0.80645 | T | 0.19355 | 8 | 0.25806 |
| 11049 | S13_56768327 | 13 | 56768327 | 86503 | A | G | G | 0.53226 | A | 0.46774 | 13 | 0.41935 |
| 11050 | S13_56914333 | 13 | 56914333 | 146006 | A | G | G | 0.8871 | A | 0.1129 | 5 | 0.16129 |
| 11051 | S13_57156980 | 13 | 57156980 | 242647 | C | T | T | 0.85484 | C | 0.14516 | 7 | 0.22581 |
| 11052 | S13_57336304 | 13 | 57336304 | 179324 | T | C | T | 0.90323 | C | 0.09677 | 6 | 0.19355 |
| 11053 | S13_57336395 | 13 | 57336395 | 91 | A | G | A | 0.90323 | G | 0.09677 | 6 | 0.19355 |
| 11054 | S13_57512099 | 13 | 57512099 | 175704 | T | C | C | 0.72581 | T | 0.27419 | 13 | 0.41935 |
| 11055 | S13_57871759 | 13 | 57871759 | 359660 | G | C | G | 0.58065 | C | 0.41935 | 14 | 0.45161 |
| 11056 | S13_57871768 | 13 | 57871768 | 9 | C | A | A | 0.58065 | C | 0.41935 | 14 | 0.45161 |
| 11057 | S13_57871769 | 13 | 57871769 | 1 | G | T | G | 0.85484 | T | 0.14516 | 5 | 0.16129 |
| 11058 | S13_58015917 | 13 | 58015917 | 144148 | C | T | C | 0.51613 | T | 0.48387 | 16 | 0.51613 |
| 11059 | S13_58016051 | 13 | 58016051 | 134 | G | A | G | 0.90323 | A | 0.09677 | 4 | 0.12903 |
| 11060 | S13_58180800 | 13 | 58180800 | 164749 | G | A | G | 0.90323 | A | 0.09677 | 4 | 0.12903 |
| 11061 | S13_58293497 | 13 | 58293497 | 112697 | C | T | T | 0.58065 | C | 0.41935 | 16 | 0.51613 |
| 11062 | S13_58688737 | 13 | 58688737 | 395240 | G | A | G | 0.93548 | A | 0.06452 | 4 | 0.12903 |
| 11063 | S13_58688821 | 13 | 58688821 | 84 | G | C | G | 0.8871 | C | 0.1129 | 5 | 0.16129 |
| 11064 | S13_58692462 | 13 | 58692462 | 3641 | A | G | G | 0.58065 | A | 0.41935 | 18 | 0.58065 |
| 11065 | S13_58692580 | 13 | 58692580 | 118 | G | C | G | 0.95161 | C | 0.04839 | 3 | 0.09677 |
| 11066 | S13_58959460 | 13 | 58959460 | 266880 | T | C | T | 0.87097 | C | 0.12903 | 6 | 0.19355 |
| 11067 | S13_58959575 | 13 | 58959575 | 115 | T | C | C | 0.72581 | T | 0.27419 | 13 | 0.41935 |
| 11068 | S13_59633509 | 13 | 59633509 | 673934 | G | C | G | 0.82258 | C | 0.17742 | 9 | 0.29032 |

| Site number | SNP name | Chromosome | Physical position | Physical distance from the previous SNP | Reference allele | Alternative allele | Major allele | Major allele frequency | Minor allele | Minor allele frequency | Number heterozygous | Heterozygosity |
|---|---|---|---|---|---|---|---|---|---|---|---|---|
| 11069 | S13_59687891 | 13 | 59687891 | 54382 | T | C | C | 0.70968 | T | 0.29032 | 14 | 0.45161 |
| 11070 | S13_60403910 | 13 | 60403910 | 716019 | C | T | C | 0.93548 | T | 0.06452 | 4 | 0.12903 |
| 11071 | S13_60904103 | 13 | 60904103 | 500193 | C | T | C | 0.90323 | T | 0.09677 | 6 | 0.19355 |
| 11072 | S13_61026117 | 13 | 61026117 | 122014 | T | A | A | 0.70968 | T | 0.29032 | 10 | 0.32258 |
| 11073 | S13_61026145 | 13 | 61026145 | 28 | G | A | A | 0.8871 | G | 0.1129 | 7 | 0.22581 |
| 11074 | S13_61255369 | 13 | 61255369 | 229224 | G | A | G | 0.93548 | A | 0.06452 | 4 | 0.12903 |
| 11075 | S13_61288480 | 13 | 61288480 | 33111 | C | T | C | 0.62903 | T | 0.37097 | 13 | 0.41935 |
| 11076 | S13_61288606 | 13 | 61288606 | 126 | T | C | T | 0.91935 | C | 0.08065 | 5 | 0.16129 |
| 11077 | S13_61341664 | 13 | 61341664 | 53058 | G | A | G | 0.74194 | A | 0.25806 | 12 | 0.3871 |
| 11078 | S13_61384459 | 13 | 61384459 | 42795 | C | T | C | 0.90323 | T | 0.09677 | 6 | 0.19355 |
| 11079 | S13_61384505 | 13 | 61384505 | 46 | G | A | G | 0.77419 | A | 0.22581 | 10 | 0.32258 |
| 11080 | S13_61384526 | 13 | 61384526 | 21 | G | A | G | 0.90323 | A | 0.09677 | 6 | 0.19355 |
| 11081 | S13_61384614 | 13 | 61384614 | 88 | A | G | A | 0.90323 | G | 0.09677 | 6 | 0.19355 |
| 11082 | S13_61552539 | 13 | 61552539 | 167925 | C | T | T | 0.70968 | C | 0.29032 | 14 | 0.45161 |
| 11083 | S13_61552546 | 13 | 61552546 | 7 | T | A | T | 0.85484 | A | 0.14516 | 9 | 0.29032 |
| 11084 | S13_61552765 | 13 | 61552765 | 219 | T | C | T | 0.90323 | C | 0.09677 | 6 | 0.19355 |
| 11085 | S13_61685664 | 13 | 61685664 | 132899 | C | T | C | 0.59677 | T | 0.40323 | 17 | 0.54839 |
| 11086 | S13_61685665 | 13 | 61685665 | 1 | A | G | A | 0.59677 | G | 0.40323 | 17 | 0.54839 |
| 11087 | S13_61685679 | 13 | 61685679 | 14 | G | A | G | 0.6129 | A | 0.3871 | 18 | 0.58065 |
| 11088 | S13_61685714 | 13 | 61685714 | 35 | A | G | A | 0.59677 | G | 0.40323 | 17 | 0.54839 |
| 11089 | S13_61685715 | 13 | 61685715 | 1 | C | T | C | 0.59677 | T | 0.40323 | 17 | 0.54839 |
| 11090 | S13_61685724 | 13 | 61685724 | 9 | C | A | C | 0.90323 | A | 0.09677 | 6 | 0.19355 |
| 11091 | S13_61685733 | 13 | 61685733 | 9 | A | T | A | 0.59677 | T | 0.40323 | 17 | 0.54839 |
| 11092 | S13_61685748 | 13 | 61685748 | 15 | G | A | G | 0.59677 | A | 0.40323 | 17 | 0.54839 |
| 11093 | S13_61685764 | 13 | 61685764 | 16 | G | A | G | 0.59677 | A | 0.40323 | 17 | 0.54839 |
| 11094 | S13_61685860 | 13 | 61685860 | 96 | A | T | A | 0.82258 | T | 0.17742 | 11 | 0.35484 |
| 11095 | S13_62062585 | 13 | 62062585 | 376725 | G | A | G | 0.75806 | A | 0.24194 | 15 | 0.48387 |
| 11096 | S13_62062604 | 13 | 62062604 | 19 | G | A | G | 0.74194 | A | 0.25806 | 16 | 0.51613 |
| 11097 | S13_62204560 | 13 | 62204560 | 141956 | T | A | T | 0.93548 | A | 0.06452 | 4 | 0.12903 |
| 11098 | S13_62204619 | 13 | 62204619 | 59 | T | C | T | 0.93548 | C | 0.06452 | 4 | 0.12903 |
| 11099 | S13_62204634 | 13 | 62204634 | 15 | G | A | G | 0.93548 | A | 0.06452 | 4 | 0.12903 |
| 11100 | S13_62204712 | 13 | 62204712 | 78 | C | T | C | 0.83871 | T | 0.16129 | 10 | 0.32258 |
| 11101 | S13_62273329 | 13 | 62273329 | 68617 | T | C | C | 0.51613 | T | 0.48387 | 16 | 0.51613 |
| 11102 | S13_62750107 | 13 | 62750107 | 476778 | T | G | G | 0.5 | T | 0.5 | 11 | 0.35484 |
| 11103 | S13_63141594 | 13 | 63141594 | 391487 | C | T | C | 0.66129 | T | 0.33871 | 9 | 0.29032 |
| 11104 | S13_63295239 | 13 | 63295239 | 153645 | T | C | T | 0.6129 | C | 0.3871 | 18 | 0.58065 |
| 11105 | S13_64076633 | 13 | 64076633 | 781394 | T | C | C | 0.91935 | T | 0.08065 | 5 | 0.16129 |
| 11106 | S13_64076806 | 13 | 64076806 | 173 | C | T | C | 0.80645 | T | 0.19355 | 8 | 0.25806 |
| 11107 | S13_64380608 | 13 | 64380608 | 303802 | C | A | C | 0.80645 | A | 0.19355 | 10 | 0.32258 |
| 11108 | S13_64380624 | 13 | 64380624 | 16 | A | G | A | 0.83871 | G | 0.16129 | 10 | 0.32258 |
| 11109 | S13_64384124 | 13 | 64384124 | 3500 | C | T | C | 0.83871 | T | 0.16129 | 8 | 0.25806 |
| 11110 | S13_64718905 | 13 | 64718905 | 334781 | A | G | A | 0.8871 | G | 0.1129 | 7 | 0.22581 |
| 11111 | S13_64718913 | 13 | 64718913 | 8 | A | G | A | 0.56452 | G | 0.43548 | 15 | 0.48387 |
| 11112 | S13_64740497 | 13 | 64740497 | 21584 | C | T | C | 0.72581 | T | 0.27419 | 15 | 0.48387 |
| 11113 | S13_64740556 | 13 | 64740556 | 59 | G | A | G | 0.87097 | A | 0.12903 | 4 | 0.12903 |
| 11114 | S13_64974425 | 13 | 64974425 | 233869 | A | G | A | 0.5 | G | 0.5 | 15 | 0.48387 |
| 11115 | S13_64974441 | 13 | 64974441 | 16 | C | T | C | 0.93548 | T | 0.06452 | 4 | 0.12903 |
| 11116 | S13_65140640 | 13 | 65140640 | 166199 | A | G | G | 0.85484 | A | 0.14516 | 7 | 0.22581 |
| 11117 | S13_65140678 | 13 | 65140678 | 38 | G | C | C | 0.85484 | G | 0.14516 | 7 | 0.22581 |
| 11118 | S13_65662633 | 13 | 65662633 | 521955 | C | T | C | 0.90323 | T | 0.09677 | 6 | 0.19355 |
| 11119 | S13_65960289 | 13 | 65960289 | 297656 | G | T | G | 0.93548 | T | 0.06452 | 4 | 0.12903 |
| 11120 | S13_65960316 | 13 | 65960316 | 27 | T | C | T | 0.93548 | C | 0.06452 | 4 | 0.12903 |
| 11121 | S13_65960418 | 13 | 65960418 | 102 | T | C | T | 0.93548 | C | 0.06452 | 4 | 0.12903 |
| 11122 | S13_65960475 | 13 | 65960475 | 57 | C | T | C | 0.6129 | T | 0.3871 | 10 | 0.32258 |

| Site number | SNP name | Chromosome | Physical position | Physical distance from the previous SNP | Reference allele | Alternative allele | Major allele | Major allele frequency | Minor allele | Minor allele frequency | Number heterozygous | Heterozygosity |
|---|---|---|---|---|---|---|---|---|---|---|---|---|
| 11123 | S13_66715683 | 13 | 66715683 | 755208 | C | T | C | 0.91935 | T | 0.08065 | 3 | 0.09677 |
| 11124 | S13_66715717 | 13 | 66715717 | 34 | C | T | C | 0.91935 | T | 0.08065 | 3 | 0.09677 |
| 11125 | S13_66715771 | 13 | 66715771 | 54 | T | C | T | 0.91935 | C | 0.08065 | 3 | 0.09677 |
| 11126 | S13_66715777 | 13 | 66715777 | 6 | C | T | C | 0.91935 | T | 0.08065 | 3 | 0.09677 |
| 11127 | S13_66715788 | 13 | 66715788 | 11 | A | G | A | 0.91935 | G | 0.08065 | 3 | 0.09677 |
| 11128 | S13_66715812 | 13 | 66715812 | 24 | A | G | A | 0.91935 | G | 0.08065 | 3 | 0.09677 |
| 11129 | S13_66715820 | 13 | 66715820 | 8 | C | T | C | 0.93548 | T | 0.06452 | 4 | 0.12903 |
| 11130 | S13_66715826 | 13 | 66715826 | 6 | T | C | T | 0.79032 | C | 0.20968 | 9 | 0.29032 |
| 11131 | S13_66715830 | 13 | 66715830 | 4 | T | A | A | 0.67742 | T | 0.32258 | 12 | 0.3871 |
| 11132 | S13_66715848 | 13 | 66715848 | 18 | C | A | C | 0.91935 | A | 0.08065 | 3 | 0.09677 |
| 11133 | S13_66715879 | 13 | 66715879 | 31 | C | G | C | 0.91935 | G | 0.08065 | 3 | 0.09677 |
| 11134 | S13_66715887 | 13 | 66715887 | 8 | C | A | C | 0.82258 | A | 0.17742 | 9 | 0.29032 |
| 11135 | S13_66715888 | 13 | 66715888 | 1 | A | G | A | 0.91935 | G | 0.08065 | 3 | 0.09677 |
| 11136 | S13_66715904 | 13 | 66715904 | 16 | C | T | C | 0.82258 | T | 0.17742 | 7 | 0.22581 |
| 11137 | S13_66715918 | 13 | 66715918 | 14 | T | A | T | 0.51613 | A | 0.48387 | 12 | 0.3871 |
| 11138 | S13_66850288 | 13 | 66850288 | 134370 | T | C | T | 0.67742 | C | 0.32258 | 12 | 0.3871 |
| 11139 | S13_66850319 | 13 | 66850319 | 31 | G | A | G | 0.74194 | A | 0.25806 | 14 | 0.45161 |
| 11140 | S13_66850335 | 13 | 66850335 | 16 | G | A | G | 0.82258 | A | 0.17742 | 11 | 0.35484 |
| 11141 | S13_66850349 | 13 | 66850349 | 14 | C | T | C | 0.8871 | T | 0.1129 | 5 | 0.16129 |
| 11142 | S13_66850428 | 13 | 66850428 | 79 | C | T | C | 0.74194 | T | 0.25806 | 14 | 0.45161 |
| 11143 | S13_66854383 | 13 | 66854383 | 3955 | T | C | T | 0.85484 | C | 0.14516 | 7 | 0.22581 |
| 11144 | S13_66918175 | 13 | 66918175 | 63792 | T | C | T | 0.79032 | C | 0.20968 | 7 | 0.22581 |
| 11145 | S13_66918260 | 13 | 66918260 | 85 | C | G | G | 0.8871 | C | 0.1129 | 5 | 0.16129 |
| 11146 | S13_66918287 | 13 | 66918287 | 27 | T | G | T | 0.77419 | G | 0.22581 | 8 | 0.25806 |
| 11147 | S13_66924673 | 13 | 66924673 | 6386 | G | T | G | 0.95161 | T | 0.04839 | 1 | 0.03226 |
| 11148 | S13_66924721 | 13 | 66924721 | 48 | C | A | C | 0.91935 | A | 0.08065 | 5 | 0.16129 |
| 11149 | S13_66924786 | 13 | 66924786 | 65 | G | A | G | 0.8871 | A | 0.1129 | 7 | 0.22581 |
| 11150 | S13_67254027 | 13 | 67254027 | 329241 | A | C | C | 0.79032 | A | 0.20968 | 9 | 0.29032 |
| 11151 | S13_67424643 | 13 | 67424643 | 170616 | C | T | C | 0.56452 | T | 0.43548 | 15 | 0.48387 |
| 11152 | S13_67797152 | 13 | 67797152 | 372509 | G | A | G | 0.69355 | A | 0.30645 | 13 | 0.41935 |
| 11153 | S13_67883550 | 13 | 67883550 | 86398 | G | A | G | 0.87097 | A | 0.12903 | 6 | 0.19355 |
| 11154 | S13_67883559 | 13 | 67883559 | 9 | C | T | C | 0.8871 | T | 0.1129 | 5 | 0.16129 |
| 11155 | S13_67958822 | 13 | 67958822 | 75263 | G | A | G | 0.8871 | A | 0.1129 | 5 | 0.16129 |
| 11156 | S13_68015319 | 13 | 68015319 | 56497 | G | C | G | 0.91935 | C | 0.08065 | 5 | 0.16129 |
| 11157 | S13_68015351 | 13 | 68015351 | 32 | C | T | C | 0.91935 | T | 0.08065 | 5 | 0.16129 |
| 11158 | S13_68015475 | 13 | 68015475 | 124 | A | G | A | 0.66129 | G | 0.33871 | 15 | 0.48387 |
| 11159 | S13_68015503 | 13 | 68015503 | 28 | G | A | G | 0.79032 | A | 0.20968 | 11 | 0.35484 |
| 11160 | S13_68015505 | 13 | 68015505 | 2 | G | A | G | 0.79032 | A | 0.20968 | 11 | 0.35484 |
| 11161 | S13_68015506 | 13 | 68015506 | 1 | T | C | C | 0.83871 | T | 0.16129 | 6 | 0.19355 |
| 11162 | S13_68946937 | 13 | 68946937 | 931431 | T | C | T | 0.8871 | C | 0.1129 | 7 | 0.22581 |
| 11163 | S13_69128138 | 13 | 69128138 | 181201 | C | T | C | 0.83871 | T | 0.16129 | 10 | 0.32258 |
| 11164 | S13_69128195 | 13 | 69128195 | 57 | C | T | C | 0.90323 | T | 0.09677 | 6 | 0.19355 |
| 11165 | S13_69128196 | 13 | 69128196 | 1 | G | A | G | 0.83871 | A | 0.16129 | 10 | 0.32258 |
| 11166 | S13_69128346 | 13 | 69128346 | 150 | C | A | C | 0.62903 | A | 0.37097 | 15 | 0.48387 |
| 11167 | S13_69128355 | 13 | 69128355 | 9 | A | G | A | 0.85484 | G | 0.14516 | 9 | 0.29032 |
| 11168 | S13_69487584 | 13 | 69487584 | 359229 | G | A | G | 0.90323 | A | 0.09677 | 6 | 0.19355 |
| 11169 | S13_69504890 | 13 | 69504890 | 17306 | G | T | G | 0.91935 | T | 0.08065 | 5 | 0.16129 |
| 11170 | S13_69652202 | 13 | 69652202 | 147312 | C | G | G | 0.53226 | C | 0.46774 | 15 | 0.48387 |
| 11171 | S13_69652247 | 13 | 69652247 | 45 | T | C | T | 0.58065 | C | 0.41935 | 12 | 0.3871 |
| 11172 | S13_69652298 | 13 | 69652298 | 51 | A | C | A | 0.58065 | C | 0.41935 | 12 | 0.3871 |
| 11173 | S13_69823487 | 13 | 69823487 | 171189 | A | G | A | 0.82258 | G | 0.17742 | 9 | 0.29032 |
| 11174 | S13_70084186 | 13 | 70084186 | 260699 | G | C | G | 0.77419 | C | 0.22581 | 14 | 0.45161 |
| 11175 | S13_70084315 | 13 | 70084315 | 129 | G | A | G | 0.8871 | A | 0.1129 | 7 | 0.22581 |
| 11176 | S13_70315702 | 13 | 70315702 | 231387 | C | T | C | 0.93548 | T | 0.06452 | 4 | 0.12903 |

| Site number | SNP name | Chromosome | Physical position | Physical distance from the previous SNP | Reference allele | Alternative allele | Major allele | Major allele frequency | Minor allele | Minor allele frequency | Number heterozygous | Heterozygosity |
|---|---|---|---|---|---|---|---|---|---|---|---|---|
| 11177 | S13_70315735 | 13 | 70315735 | 33 | G | A | A | 0.87097 | G | 0.12903 | 6 | 0.19355 |
| 11178 | S13_70545220 | 13 | 70545220 | 229485 | A | C | A | 0.8871 | C | 0.1129 | 7 | 0.22581 |
| 11179 | S13_70545278 | 13 | 70545278 | 58 | T | C | C | 0.91935 | T | 0.08065 | 5 | 0.16129 |
| 11180 | S13_70545332 | 13 | 70545332 | 54 | A | G | A | 0.54839 | G | 0.45161 | 16 | 0.51613 |
| 11181 | S13_70593803 | 13 | 70593803 | 48471 | T | G | T | 0.79032 | G | 0.20968 | 13 | 0.41935 |
| 11182 | S13_70593940 | 13 | 70593940 | 137 | C | T | C | 0.91935 | T | 0.08065 | 5 | 0.16129 |
| 11183 | S13_70607835 | 13 | 70607835 | 13895 | A | G | A | 0.83871 | G | 0.16129 | 10 | 0.32258 |
| 11184 | S13_70724700 | 13 | 70724700 | 116865 | G | A | G | 0.87097 | A | 0.12903 | 8 | 0.25806 |
| 11185 | S13_70724701 | 13 | 70724701 | 1 | A | C | A | 0.95161 | C | 0.04839 | 3 | 0.09677 |
| 11186 | S13_70805865 | 13 | 70805865 | 81164 | A | C | A | 0.93548 | C | 0.06452 | 4 | 0.12903 |
| 11187 | S13_70805942 | 13 | 70805942 | 77 | T | C | T | 0.93548 | C | 0.06452 | 4 | 0.12903 |
| 11188 | S13_70968944 | 13 | 70968944 | 163002 | A | G | G | 0.53226 | A | 0.46774 | 17 | 0.54839 |
| 11189 | S13_70969036 | 13 | 70969036 | 92 | A | G | A | 0.91935 | G | 0.08065 | 5 | 0.16129 |
| 11190 | S13_70972949 | 13 | 70972949 | 3913 | G | A | G | 0.75806 | A | 0.24194 | 11 | 0.35484 |
| 11191 | S13_70973088 | 13 | 70973088 | 139 | A | G | G | 0.67742 | A | 0.32258 | 10 | 0.32258 |
| 11192 | S13_70973089 | 13 | 70973089 | 1 | T | C | T | 0.91935 | C | 0.08065 | 5 | 0.16129 |
| 11193 | S13_70974139 | 13 | 70974139 | 1050 | G | A | G | 0.75806 | A | 0.24194 | 11 | 0.35484 |
| 11194 | S13_71099328 | 13 | 71099328 | 125189 | C | T | C | 0.93548 | T | 0.06452 | 4 | 0.12903 |
| 11195 | S13_71099539 | 13 | 71099539 | 211 | T | C | T | 0.8871 | C | 0.1129 | 5 | 0.16129 |
| 11196 | S13_71151302 | 13 | 71151302 | 51763 | C | A | C | 0.75806 | A | 0.24194 | 11 | 0.35484 |
| 11197 | S13_71151398 | 13 | 71151398 | 96 | C | T | C | 0.85484 | T | 0.14516 | 7 | 0.22581 |
| 11198 | S13_71244331 | 13 | 71244331 | 92933 | T | C | C | 0.58065 | T | 0.41935 | 10 | 0.32258 |
| 11199 | S13_71622262 | 13 | 71622262 | 377931 | A | C | A | 0.95161 | C | 0.04839 | 3 | 0.09677 |
| 11200 | S13_71622271 | 13 | 71622271 | 9 | G | A | G | 0.8871 | A | 0.1129 | 7 | 0.22581 |
| 11201 | S13_71622352 | 13 | 71622352 | 81 | C | T | C | 0.93548 | T | 0.06452 | 4 | 0.12903 |
| 11202 | S13_71812947 | 13 | 71812947 | 190595 | G | A | G | 0.72581 | A | 0.27419 | 11 | 0.35484 |
| 11203 | S13_71904207 | 13 | 71904207 | 91260 | C | T | C | 0.74194 | T | 0.25806 | 14 | 0.45161 |
| 11204 | S13_71921895 | 13 | 71921895 | 17688 | C | G | C | 0.69355 | G | 0.30645 | 15 | 0.48387 |
| 11205 | S13_71971432 | 13 | 71971432 | 49537 | C | G | C | 0.59677 | G | 0.40323 | 19 | 0.6129 |
| 11206 | S13_71971462 | 13 | 71971462 | 30 | C | T | C | 0.59677 | T | 0.40323 | 19 | 0.6129 |
| 11207 | S13_71971618 | 13 | 71971618 | 156 | A | T | A | 0.59677 | T | 0.40323 | 19 | 0.6129 |
| 11208 | S13_71971636 | 13 | 71971636 | 18 | T | G | T | 0.59677 | G | 0.40323 | 19 | 0.6129 |
| 11209 | S13_72538577 | 13 | 72538577 | 566941 | A | G | A | 0.85484 | G | 0.14516 | 9 | 0.29032 |
| 11210 | S13_72609343 | 13 | 72609343 | 70766 | G | A | G | 0.90323 | A | 0.09677 | 6 | 0.19355 |
| 11211 | S13_72609366 | 13 | 72609366 | 23 | C | T | C | 0.90323 | T | 0.09677 | 6 | 0.19355 |
| 11212 | S13_72609378 | 13 | 72609378 | 12 | C | G | C | 0.90323 | G | 0.09677 | 6 | 0.19355 |
| 11213 | S13_72609386 | 13 | 72609386 | 8 | C | A | C | 0.90323 | A | 0.09677 | 6 | 0.19355 |
| 11214 | S13_72609387 | 13 | 72609387 | 1 | A | G | G | 0.59677 | A | 0.40323 | 15 | 0.48387 |
| 11215 | S13_72609390 | 13 | 72609390 | 3 | T | G | G | 0.59677 | T | 0.40323 | 15 | 0.48387 |
| 11216 | S13_72609392 | 13 | 72609392 | 2 | T | A | A | 0.59677 | T | 0.40323 | 15 | 0.48387 |
| 11217 | S13_72609398 | 13 | 72609398 | 6 | T | C | C | 0.59677 | T | 0.40323 | 15 | 0.48387 |
| 11218 | S13_72609406 | 13 | 72609406 | 8 | C | T | C | 0.90323 | T | 0.09677 | 6 | 0.19355 |
| 11219 | S13_72609445 | 13 | 72609445 | 39 | G | A | G | 0.90323 | A | 0.09677 | 6 | 0.19355 |
| 11220 | S13_72674481 | 13 | 72674481 | 65036 | G | A | A | 0.6129 | G | 0.3871 | 18 | 0.58065 |
| 11221 | S13_72674531 | 13 | 72674531 | 50 | G | A | G | 0.80645 | A | 0.19355 | 12 | 0.3871 |
| 11222 | S13_72674572 | 13 | 72674572 | 41 | A | G | G | 0.62903 | A | 0.37097 | 15 | 0.48387 |
| 11223 | S13_72677520 | 13 | 72677520 | 2948 | G | A | G | 0.93548 | A | 0.06452 | 2 | 0.06452 |
| 11224 | S13_72677618 | 13 | 72677618 | 98 | G | T | G | 0.83871 | T | 0.16129 | 10 | 0.32258 |
| 11225 | S13_72680806 | 13 | 72680806 | 3188 | G | A | G | 0.79032 | A | 0.20968 | 11 | 0.35484 |
| 11226 | S13_72680828 | 13 | 72680828 | 22 | G | A | G | 0.87097 | A | 0.12903 | 6 | 0.19355 |
| 11227 | S13_72680972 | 13 | 72680972 | 144 | T | C | T | 0.93548 | C | 0.06452 | 4 | 0.12903 |
| 11228 | S13_72697133 | 13 | 72697133 | 16161 | C | T | C | 0.72581 | T | 0.27419 | 9 | 0.29032 |
| 11229 | S13_72739025 | 13 | 72739025 | 41892 | T | C | T | 0.51613 | C | 0.48387 | 16 | 0.51613 |
| 11230 | S13_72739039 | 13 | 72739039 | 14 | C | T | C | 0.95161 | T | 0.04839 | 3 | 0.09677 |

| Site number | SNP name | Chromosome | Physical position | Physical distance from the previous SNP | Reference allele | Alternative allele | Major allele | Major allele frequency | Minor allele | Minor allele frequency | Number heterozygous | Heterozygosity |
|---|---|---|---|---|---|---|---|---|---|---|---|---|
| 11231 | S13_72806473 | 13 | 72806473 | 67434 | T | C | C | 0.87097 | T | 0.12903 | 8 | 0.25806 |
| 11232 | S13_72891310 | 13 | 72891310 | 84837 | G | A | A | 0.72581 | G | 0.27419 | 13 | 0.41935 |
| 11233 | S13_72891347 | 13 | 72891347 | 37 | C | G | C | 0.72581 | G | 0.27419 | 13 | 0.41935 |
| 11234 | S13_72891484 | 13 | 72891484 | 137 | G | T | G | 0.72581 | T | 0.27419 | 13 | 0.41935 |
| 11235 | S13_73035477 | 13 | 73035477 | 143993 | G | A | G | 0.80645 | A | 0.19355 | 12 | 0.3871 |
| 11236 | S13_73035516 | 13 | 73035516 | 39 | G | A | G | 0.87097 | A | 0.12903 | 6 | 0.19355 |
| 11237 | S13_73253383 | 13 | 73253383 | 217867 | G | A | G | 0.91935 | A | 0.08065 | 5 | 0.16129 |
| 11238 | S13_73253478 | 13 | 73253478 | 95 | C | T | C | 0.91935 | T | 0.08065 | 5 | 0.16129 |
| 11239 | S13_73253479 | 13 | 73253479 | 1 | G | A | G | 0.93548 | A | 0.06452 | 4 | 0.12903 |
| 11240 | S13_73705877 | 13 | 73705877 | 452398 | T | C | C | 0.72581 | T | 0.27419 | 11 | 0.35484 |
| 11241 | S13_74252996 | 13 | 74252996 | 547119 | C | T | C | 0.93548 | T | 0.06452 | 2 | 0.06452 |
| 11242 | S13_74382056 | 13 | 74382056 | 129060 | T | C | T | 0.8871 | C | 0.1129 | 5 | 0.16129 |
| 11243 | S13_74416307 | 13 | 74416307 | 34251 | T | C | T | 0.80645 | C | 0.19355 | 8 | 0.25806 |
| 11244 | S13_74416396 | 13 | 74416396 | 89 | C | T | C | 0.80645 | T | 0.19355 | 8 | 0.25806 |
| 11245 | S13_74416497 | 13 | 74416497 | 101 | C | A | C | 0.80645 | A | 0.19355 | 8 | 0.25806 |
| 11246 | S13_74416498 | 13 | 74416498 | 1 | G | A | G | 0.93548 | A | 0.06452 | 4 | 0.12903 |
| 11247 | S13_74416513 | 13 | 74416513 | 15 | C | T | C | 0.80645 | T | 0.19355 | 8 | 0.25806 |
| 11248 | S13_74416877 | 13 | 74416877 | 364 | C | T | C | 0.80645 | T | 0.19355 | 8 | 0.25806 |
| 11249 | S13_74416890 | 13 | 74416890 | 13 | A | C | A | 0.87097 | C | 0.12903 | 6 | 0.19355 |
| 11250 | S13_74416891 | 13 | 74416891 | 1 | A | T | A | 0.87097 | T | 0.12903 | 6 | 0.19355 |
| 11251 | S13_74910940 | 13 | 74910940 | 494049 | T | G | T | 0.8871 | G | 0.1129 | 7 | 0.22581 |
| 11252 | S13_74910996 | 13 | 74910996 | 56 | G | A | G | 0.8871 | A | 0.1129 | 7 | 0.22581 |
| 11253 | S13_74911038 | 13 | 74911038 | 42 | T | C | T | 0.8871 | C | 0.1129 | 7 | 0.22581 |
| 11254 | S13_74911039 | 13 | 74911039 | 1 | G | A | G | 0.8871 | A | 0.1129 | 7 | 0.22581 |
| 11255 | S13_74911144 | 13 | 74911144 | 105 | G | A | G | 0.8871 | A | 0.1129 | 7 | 0.22581 |
| 11256 | S13_75017780 | 13 | 75017780 | 106636 | T | C | T | 0.53226 | C | 0.46774 | 13 | 0.41935 |
| 11257 | S13_75459027 | 13 | 75459027 | 441247 | C | T | C | 0.93548 | T | 0.06452 | 2 | 0.06452 |
| 11258 | S13_75513779 | 13 | 75513779 | 54752 | C | A | C | 0.90323 | A | 0.09677 | 6 | 0.19355 |
| 11259 | S13_75583108 | 13 | 75583108 | 69329 | T | C | T | 0.8871 | C | 0.1129 | 5 | 0.16129 |
| 11260 | S13_75583115 | 13 | 75583115 | 7 | A | C | A | 0.80645 | C | 0.19355 | 12 | 0.3871 |
| 11261 | S13_75855407 | 13 | 75855407 | 272292 | C | A | C | 0.87097 | A | 0.12903 | 8 | 0.25806 |
| 11262 | S13_75855421 | 13 | 75855421 | 14 | G | A | G | 0.85484 | A | 0.14516 | 7 | 0.22581 |
| 11263 | S13_75878882 | 13 | 75878882 | 23461 | C | T | C | 0.93548 | T | 0.06452 | 4 | 0.12903 |
| 11264 | S13_75878883 | 13 | 75878883 | 1 | G | A | G | 0.85484 | A | 0.14516 | 7 | 0.22581 |
| 11265 | S13_75878885 | 13 | 75878885 | 2 | C | T | C | 0.79032 | T | 0.20968 | 13 | 0.41935 |
| 11266 | S13_75878898 | 13 | 75878898 | 13 | G | C | G | 0.77419 | C | 0.22581 | 14 | 0.45161 |
| 11267 | S13_75976332 | 13 | 75976332 | 97434 | C | T | T | 0.8871 | C | 0.1129 | 7 | 0.22581 |
| 11268 | S13_76485881 | 13 | 76485881 | 509549 | C | T | C | 0.67742 | T | 0.32258 | 12 | 0.3871 |
| 11269 | S13_76810802 | 13 | 76810802 | 324921 | T | C | T | 0.56452 | C | 0.43548 | 15 | 0.48387 |
| 11270 | S13_76830669 | 13 | 76830669 | 19867 | A | T | T | 0.53226 | A | 0.46774 | 15 | 0.48387 |
| 11271 | S13_76830720 | 13 | 76830720 | 51 | G | A | G | 0.85484 | A | 0.14516 | 9 | 0.29032 |
| 11272 | S13_76976240 | 13 | 76976240 | 145520 | C | T | C | 0.90323 | T | 0.09677 | 6 | 0.19355 |
| 11273 | S13_76976313 | 13 | 76976313 | 73 | A | G | G | 0.62903 | A | 0.37097 | 15 | 0.48387 |
| 11274 | S13_77041956 | 13 | 77041956 | 65643 | T | C | T | 0.93548 | C | 0.06452 | 4 | 0.12903 |
| 11275 | S13_77041992 | 13 | 77041992 | 36 | A | G | A | 0.82258 | G | 0.17742 | 7 | 0.22581 |
| 11276 | S13_77042040 | 13 | 77042040 | 48 | G | A | G | 0.90323 | A | 0.09677 | 6 | 0.19355 |
| 11277 | S13_77256814 | 13 | 77256814 | 214774 | G | A | G | 0.66129 | A | 0.33871 | 13 | 0.41935 |
| 11278 | S13_77256823 | 13 | 77256823 | 9 | A | G | A | 0.66129 | G | 0.33871 | 13 | 0.41935 |
| 11279 | S13_77256841 | 13 | 77256841 | 18 | C | T | C | 0.66129 | T | 0.33871 | 13 | 0.41935 |
| 11280 | S13_77676986 | 13 | 77676986 | 420145 | G | C | G | 0.83871 | C | 0.16129 | 10 | 0.32258 |
| 11281 | S13_77888487 | 13 | 77888487 | 211501 | C | T | C | 0.83871 | T | 0.16129 | 10 | 0.32258 |
| 11282 | S13_77888653 | 13 | 77888653 | 166 | C | T | C | 0.80645 | T | 0.19355 | 12 | 0.3871 |
| 11283 | S13_78211283 | 13 | 78211283 | 322630 | G | A | G | 0.56452 | A | 0.43548 | 17 | 0.54839 |
| 11284 | S13_78211287 | 13 | 78211287 | 4 | A | G | G | 0.80645 | A | 0.19355 | 8 | 0.25806 |

| Site number | SNP name | Chromosome | Physical position | Physical distance from the previous SNP | Reference allele | Alternative allele | Major allele | Major allele frequency | Minor allele | Minor allele frequency | Number heterozygous | Heterozygosity |
|---|---|---|---|---|---|---|---|---|---|---|---|---|
| 11285 | S13_78211298 | 13 | 78211298 | 11 | G | A | G | 0.79032 | A | 0.20968 | 11 | 0.35484 |
| 11286 | S13_78211360 | 13 | 78211360 | 62 | A | G | A | 0.93548 | G | 0.06452 | 4 | 0.12903 |
| 11287 | S13_78310219 | 13 | 78310219 | 98859 | C | T | C | 0.79032 | T | 0.20968 | 9 | 0.29032 |
| 11288 | S13_78310248 | 13 | 78310248 | 29 | G | A | G | 0.77419 | A | 0.22581 | 10 | 0.32258 |
| 11289 | S13_78455749 | 13 | 78455749 | 145501 | A | T | A | 0.90323 | T | 0.09677 | 6 | 0.19355 |
| 11290 | S13_78455770 | 13 | 78455770 | 21 | C | T | T | 0.51613 | C | 0.48387 | 14 | 0.45161 |
| 11291 | S13_78455780 | 13 | 78455780 | 10 | G | T | T | 0.51613 | G | 0.48387 | 14 | 0.45161 |
| 11292 | S13_78455838 | 13 | 78455838 | 58 | C | T | C | 0.93548 | T | 0.06452 | 4 | 0.12903 |
| 11293 | S13_78455848 | 13 | 78455848 | 10 | T | C | C | 0.74194 | T | 0.25806 | 12 | 0.3871 |
| 11294 | S13_78455905 | 13 | 78455905 | 57 | T | C | C | 0.74194 | T | 0.25806 | 12 | 0.3871 |
| 11295 | S13_78541489 | 13 | 78541489 | 85584 | G | A | G | 0.82258 | A | 0.17742 | 7 | 0.22581 |
| 11296 | S13_78541502 | 13 | 78541502 | 13 | C | A | C | 0.90323 | A | 0.09677 | 6 | 0.19355 |
| 11297 | S13_78541671 | 13 | 78541671 | 169 | C | T | T | 0.66129 | C | 0.33871 | 13 | 0.41935 |
| 11298 | S13_78637192 | 13 | 78637192 | 95521 | G | A | G | 0.74194 | A | 0.25806 | 12 | 0.3871 |
| 11299 | S13_79027960 | 13 | 79027960 | 390768 | T | A | T | 0.90323 | A | 0.09677 | 4 | 0.12903 |
| 11300 | S13_79290036 | 13 | 79290036 | 262076 | C | T | C | 0.93548 | T | 0.06452 | 2 | 0.06452 |
| 11301 | S13_79443964 | 13 | 79443964 | 153928 | A | C | A | 0.77419 | C | 0.22581 | 12 | 0.3871 |
| 11302 | S13_79837217 | 13 | 79837217 | 393253 | G | A | G | 0.87097 | A | 0.12903 | 8 | 0.25806 |
| 11303 | S13_79857418 | 13 | 79857418 | 20201 | G | A | G | 0.87097 | A | 0.12903 | 6 | 0.19355 |
| 11304 | S13_79857526 | 13 | 79857526 | 108 | C | T | C | 0.62903 | T | 0.37097 | 15 | 0.48387 |
| 11305 | S13_79889666 | 13 | 79889666 | 32140 | G | A | G | 0.85484 | A | 0.14516 | 7 | 0.22581 |
| 11306 | S13_79926654 | 13 | 79926654 | 36988 | T | C | C | 0.80645 | T | 0.19355 | 10 | 0.32258 |
| 11307 | S13_79926806 | 13 | 79926806 | 152 | T | C | T | 0.87097 | C | 0.12903 | 6 | 0.19355 |
| 11308 | S13_80181026 | 13 | 80181026 | 254220 | T | C | C | 0.93548 | T | 0.06452 | 4 | 0.12903 |
| 11309 | S13_80187976 | 13 | 80187976 | 6950 | C | T | C | 0.79032 | T | 0.20968 | 7 | 0.22581 |
| 11310 | S13_80187998 | 13 | 80187998 | 22 | C | A | C | 0.79032 | A | 0.20968 | 13 | 0.41935 |
| 11311 | S13_80188016 | 13 | 80188016 | 18 | T | C | T | 0.91935 | C | 0.08065 | 5 | 0.16129 |
| 11312 | S13_80188064 | 13 | 80188064 | 48 | T | C | T | 0.90323 | C | 0.09677 | 6 | 0.19355 |
| 11313 | S13_80188083 | 13 | 80188083 | 19 | G | A | G | 0.6129 | A | 0.3871 | 12 | 0.3871 |
| 11314 | S13_80188198 | 13 | 80188198 | 115 | A | G | A | 0.8871 | G | 0.1129 | 7 | 0.22581 |
| 11315 | S13_80281645 | 13 | 80281645 | 93447 | T | C | C | 0.72581 | T | 0.27419 | 9 | 0.29032 |
| 11316 | S13_80367229 | 13 | 80367229 | 85584 | G | C | C | 0.80645 | G | 0.19355 | 12 | 0.3871 |
| 11317 | S13_80367235 | 13 | 80367235 | 6 | G | A | G | 0.82258 | A | 0.17742 | 9 | 0.29032 |
| 11318 | S13_80367328 | 13 | 80367328 | 93 | G | A | G | 0.69355 | A | 0.30645 | 15 | 0.48387 |
| 11319 | S13_80367329 | 13 | 80367329 | 1 | T | C | T | 0.69355 | C | 0.30645 | 15 | 0.48387 |
| 11320 | S13_80493675 | 13 | 80493675 | 126346 | C | T | C | 0.93548 | T | 0.06452 | 4 | 0.12903 |
| 11321 | S13_80732666 | 13 | 80732666 | 238991 | C | T | C | 0.95161 | T | 0.04839 | 3 | 0.09677 |
| 11322 | S13_80865627 | 13 | 80865627 | 132961 | C | T | T | 0.87097 | C | 0.12903 | 6 | 0.19355 |
| 11323 | S13_80865704 | 13 | 80865704 | 77 | T | C | C | 0.87097 | T | 0.12903 | 6 | 0.19355 |
| 11324 | S13_80865727 | 13 | 80865727 | 23 | C | T | C | 0.8871 | T | 0.1129 | 5 | 0.16129 |
| 11325 | S13_80865749 | 13 | 80865749 | 22 | T | A | A | 0.87097 | T | 0.12903 | 6 | 0.19355 |
| 11326 | S13_80865771 | 13 | 80865771 | 22 | T | C | T | 0.74194 | C | 0.25806 | 10 | 0.32258 |
| 11327 | S13_80865831 | 13 | 80865831 | 60 | G | A | G | 0.8871 | A | 0.1129 | 5 | 0.16129 |
| 11328 | S13_81025525 | 13 | 81025525 | 159694 | C | G | C | 0.95161 | G | 0.04839 | 3 | 0.09677 |
| 11329 | S13_81025537 | 13 | 81025537 | 12 | G | A | G | 0.74194 | A | 0.25806 | 12 | 0.3871 |
| 11330 | S13_81105901 | 13 | 81105901 | 80364 | A | C | C | 0.53226 | A | 0.46774 | 17 | 0.54839 |
| 11331 | S13_81105969 | 13 | 81105969 | 68 | A | C | A | 0.90323 | C | 0.09677 | 6 | 0.19355 |
| 11332 | S13_81105970 | 13 | 81105970 | 1 | A | G | A | 0.90323 | G | 0.09677 | 6 | 0.19355 |
| 11333 | S13_81106064 | 13 | 81106064 | 94 | C | T | C | 0.91935 | T | 0.08065 | 5 | 0.16129 |
| 11334 | S13_81186288 | 13 | 81186288 | 80224 | G | A | G | 0.87097 | A | 0.12903 | 6 | 0.19355 |
| 11335 | S13_81262430 | 13 | 81262430 | 76142 | T | C | C | 0.85484 | T | 0.14516 | 9 | 0.29032 |
| 11336 | S13_81280717 | 13 | 81280717 | 18287 | G | A | G | 0.95161 | A | 0.04839 | 3 | 0.09677 |
| 11337 | S13_82119740 | 13 | 82119740 | 839023 | G | A | G | 0.70968 | A | 0.29032 | 14 | 0.45161 |
| 11338 | S13_82320380 | 13 | 82320380 | 200640 | G | A | G | 0.95161 | A | 0.04839 | 3 | 0.09677 |

| Site number | SNP name | Chromosome | Physical position | Physical distance from the previous SNP | Reference allele | Alternative allele | Major allele | Major allele frequency | Minor allele | Minor allele frequency | Number heterozygous | Heterozygosity |
|---|---|---|---|---|---|---|---|---|---|---|---|---|
| 11339 | S13_82320384 | 13 | 82320384 | 4 | G | A | G | 0.83871 | A | 0.16129 | 8 | 0.25806 |
| 11340 | S13_82399129 | 13 | 82399129 | 78745 | C | T | C | 0.75806 | T | 0.24194 | 11 | 0.35484 |
| 11341 | S13_82399198 | 13 | 82399198 | 69 | C | T | C | 0.83871 | T | 0.16129 | 10 | 0.32258 |
| 11342 | S13_82399226 | 13 | 82399226 | 28 | T | C | C | 0.51613 | T | 0.48387 | 16 | 0.51613 |
| 11343 | S13_82448538 | 13 | 82448538 | 49312 | T | C | T | 0.90323 | C | 0.09677 | 6 | 0.19355 |
| 11344 | S13_82448617 | 13 | 82448617 | 79 | C | T | T | 0.75806 | C | 0.24194 | 13 | 0.41935 |
| 11345 | S13_82448621 | 13 | 82448621 | 4 | G | A | G | 0.90323 | A | 0.09677 | 6 | 0.19355 |
| 11346 | S13_82579198 | 13 | 82579198 | 130577 | A | G | A | 0.8871 | G | 0.1129 | 7 | 0.22581 |
| 11347 | S13_82624262 | 13 | 82624262 | 45064 | G | A | G | 0.95161 | A | 0.04839 | 3 | 0.09677 |
| 11348 | S13_82624330 | 13 | 82624330 | 68 | T | C | T | 0.93548 | C | 0.06452 | 4 | 0.12903 |
| 11349 | S13_82624347 | 13 | 82624347 | 17 | C | T | T | 0.54839 | C | 0.45161 | 16 | 0.51613 |
| 11350 | S13_82624363 | 13 | 82624363 | 16 | C | T | C | 0.5 | T | 0.5 | 15 | 0.48387 |
| 11351 | S14_346139 | 14 | 346139 | 0 | T | A | A | 0.72581 | T | 0.27419 | 15 | 0.48387 |
| 11352 | S14_393214 | 14 | 393214 | 47075 | G | C | G | 0.69355 | C | 0.30645 | 11 | 0.35484 |
| 11353 | S14_393294 | 14 | 393294 | 80 | A | G | A | 0.69355 | G | 0.30645 | 11 | 0.35484 |
| 11354 | S14_393328 | 14 | 393328 | 34 | G | A | G | 0.95161 | A | 0.04839 | 3 | 0.09677 |
| 11355 | S14_469712 | 14 | 469712 | 76384 | G | A | A | 0.51613 | G | 0.48387 | 16 | 0.51613 |
| 11356 | S14_542674 | 14 | 542674 | 72962 | C | T | C | 0.95161 | T | 0.04839 | 3 | 0.09677 |
| 11357 | S14_542700 | 14 | 542700 | 26 | T | C | T | 0.95161 | C | 0.04839 | 3 | 0.09677 |
| 11358 | S14_542752 | 14 | 542752 | 52 | A | G | A | 0.8871 | G | 0.1129 | 5 | 0.16129 |
| 11359 | S14_542788 | 14 | 542788 | 36 | G | A | G | 0.87097 | A | 0.12903 | 6 | 0.19355 |
| 11360 | S14_542799 | 14 | 542799 | 11 | T | C | T | 0.95161 | C | 0.04839 | 3 | 0.09677 |
| 11361 | S14_542841 | 14 | 542841 | 42 | C | T | C | 0.93548 | T | 0.06452 | 4 | 0.12903 |
| 11362 | S14_786699 | 14 | 786699 | 243858 | G | A | A | 0.70968 | G | 0.29032 | 12 | 0.3871 |
| 11363 | S14_786703 | 14 | 786703 | 4 | G | A | G | 0.87097 | A | 0.12903 | 6 | 0.19355 |
| 11364 | S14_786709 | 14 | 786709 | 6 | C | T | C | 0.69355 | T | 0.30645 | 11 | 0.35484 |
| 11365 | S14_832921 | 14 | 832921 | 46212 | C | T | C | 0.91935 | T | 0.08065 | 3 | 0.09677 |
| 11366 | S14_1157464 | 14 | 1157464 | 324543 | T | C | C | 0.82258 | T | 0.17742 | 7 | 0.22581 |
| 11367 | S14_1157472 | 14 | 1157472 | 8 | C | T | C | 0.93548 | T | 0.06452 | 4 | 0.12903 |
| 11368 | S14_2031776 | 14 | 2031776 | 874304 | G | A | A | 0.67742 | G | 0.32258 | 12 | 0.3871 |
| 11369 | S14_2031808 | 14 | 2031808 | 32 | C | G | C | 0.8871 | G | 0.1129 | 5 | 0.16129 |
| 11370 | S14_2031831 | 14 | 2031831 | 23 | C | T | T | 0.83871 | C | 0.16129 | 6 | 0.19355 |
| 11371 | S14_2099953 | 14 | 2099953 | 68122 | C | T | C | 0.95161 | T | 0.04839 | 3 | 0.09677 |
| 11372 | S14_2581992 | 14 | 2581992 | 482039 | C | T | C | 0.87097 | T | 0.12903 | 8 | 0.25806 |
| 11373 | S14_2582042 | 14 | 2582042 | 50 | A | G | A | 0.90323 | G | 0.09677 | 6 | 0.19355 |
| 11374 | S14_2582097 | 14 | 2582097 | 55 | T | C | C | 0.93548 | T | 0.06452 | 4 | 0.12903 |
| 11375 | S14_2599865 | 14 | 2599865 | 17768 | A | C | C | 0.64516 | A | 0.35484 | 14 | 0.45161 |
| 11376 | S14_2599876 | 14 | 2599876 | 11 | T | C | C | 0.95161 | T | 0.04839 | 3 | 0.09677 |
| 11377 | S14_2599950 | 14 | 2599950 | 74 | T | G | G | 0.64516 | T | 0.35484 | 14 | 0.45161 |
| 11378 | S14_2599973 | 14 | 2599973 | 23 | C | T | T | 0.64516 | C | 0.35484 | 14 | 0.45161 |
| 11379 | S14_2600066 | 14 | 2600066 | 93 | A | T | T | 0.64516 | A | 0.35484 | 14 | 0.45161 |
| 11380 | S14_3237972 | 14 | 3237972 | 637906 | C | T | C | 0.91935 | T | 0.08065 | 5 | 0.16129 |
| 11381 | S14_3238032 | 14 | 3238032 | 60 | G | A | G | 0.93548 | A | 0.06452 | 4 | 0.12903 |
| 11382 | S14_3238061 | 14 | 3238061 | 29 | T | C | T | 0.91935 | C | 0.08065 | 5 | 0.16129 |
| 11383 | S14_3238064 | 14 | 3238064 | 3 | A | C | A | 0.91935 | C | 0.08065 | 5 | 0.16129 |
| 11384 | S14_3238084 | 14 | 3238084 | 20 | G | C | G | 0.93548 | C | 0.06452 | 4 | 0.12903 |
| 11385 | S14_3238085 | 14 | 3238085 | 1 | C | T | C | 0.87097 | T | 0.12903 | 8 | 0.25806 |
| 11386 | S14_3238098 | 14 | 3238098 | 13 | A | G | A | 0.93548 | G | 0.06452 | 2 | 0.06452 |
| 11387 | S14_3238102 | 14 | 3238102 | 4 | T | G | T | 0.91935 | G | 0.08065 | 5 | 0.16129 |
| 11388 | S14_3789768 | 14 | 3789768 | 551666 | C | T | C | 0.66129 | T | 0.33871 | 17 | 0.54839 |
| 11389 | S14_3789801 | 14 | 3789801 | 33 | T | C | C | 0.58065 | T | 0.41935 | 16 | 0.51613 |
| 11390 | S14_3791714 | 14 | 3791714 | 1913 | C | T | C | 0.66129 | T | 0.33871 | 17 | 0.54839 |
| 11391 | S14_3791715 | 14 | 3791715 | 1 | A | G | G | 0.56452 | A | 0.43548 | 17 | 0.54839 |
| 11392 | S14_3791737 | 14 | 3791737 | 22 | T | C | T | 0.75806 | C | 0.24194 | 13 | 0.41935 |

| Site number | SNP name | Chromosome | Physical position | Physical distance from the previous SNP | Reference allele | Alternative allele | Major allele | Major allele frequency | Minor allele | Minor allele frequency | Number heterozygous | Heterozygosity |
|---|---|---|---|---|---|---|---|---|---|---|---|---|
| 11393 | S14_3791859 | 14 | 3791859 | 122 | A | G | G | 0.59677 | A | 0.40323 | 15 | 0.48387 |
| 11394 | S14_3791860 | 14 | 3791860 | 1 | C | T | C | 0.64516 | T | 0.35484 | 16 | 0.51613 |
| 11395 | S14_5031617 | 14 | 5031617 | 1239757 | T | C | T | 0.66129 | C | 0.33871 | 11 | 0.35484 |
| 11396 | S14_5309568 | 14 | 5309568 | 277951 | G | A | G | 0.77419 | A | 0.22581 | 14 | 0.45161 |
| 11397 | S14_5455276 | 14 | 5455276 | 145708 | A | G | A | 0.93548 | G | 0.06452 | 4 | 0.12903 |
| 11398 | S14_5455420 | 14 | 5455420 | 144 | A | G | A | 0.80645 | G | 0.19355 | 12 | 0.3871 |
| 11399 | S14_5741450 | 14 | 5741450 | 286030 | T | C | C | 0.70968 | T | 0.29032 | 14 | 0.45161 |
| 11400 | S14_5749059 | 14 | 5749059 | 7609 | T | C | C | 0.77419 | T | 0.22581 | 14 | 0.45161 |
| 11401 | S14_5749076 | 14 | 5749076 | 17 | A | G | A | 0.70968 | G | 0.29032 | 12 | 0.3871 |
| 11402 | S14_5749214 | 14 | 5749214 | 138 | T | G | T | 0.75806 | G | 0.24194 | 11 | 0.35484 |
| 11403 | S14_5988547 | 14 | 5988547 | 239333 | C | T | C | 0.83871 | T | 0.16129 | 10 | 0.32258 |
| 11404 | S14_6051020 | 14 | 6051020 | 62473 | A | C | A | 0.95161 | C | 0.04839 | 3 | 0.09677 |
| 11405 | S14_6806302 | 14 | 6806302 | 755282 | C | T | T | 0.80645 | C | 0.19355 | 12 | 0.3871 |
| 11406 | S14_6806342 | 14 | 6806342 | 40 | A | G | A | 0.51613 | G | 0.48387 | 18 | 0.58065 |
| 11407 | S14_6806396 | 14 | 6806396 | 54 | G | A | G | 0.82258 | A | 0.17742 | 9 | 0.29032 |
| 11408 | S14_6806429 | 14 | 6806429 | 33 | C | T | C | 0.51613 | T | 0.48387 | 18 | 0.58065 |
| 11409 | S14_6806471 | 14 | 6806471 | 42 | C | T | C | 0.51613 | T | 0.48387 | 18 | 0.58065 |
| 11410 | S14_7164588 | 14 | 7164588 | 358117 | C | T | C | 0.83871 | T | 0.16129 | 10 | 0.32258 |
| 11411 | S14_7164597 | 14 | 7164597 | 9 | A | G | A | 0.83871 | G | 0.16129 | 10 | 0.32258 |
| 11412 | S14_7164718 | 14 | 7164718 | 121 | G | A | G | 0.80645 | A | 0.19355 | 12 | 0.3871 |
| 11413 | S14_7164726 | 14 | 7164726 | 8 | G | A | G | 0.77419 | A | 0.22581 | 12 | 0.3871 |
| 11414 | S14_7582012 | 14 | 7582012 | 417286 | T | A | T | 0.56452 | A | 0.43548 | 15 | 0.48387 |
| 11415 | S14_7582129 | 14 | 7582129 | 117 | C | T | C | 0.75806 | T | 0.24194 | 13 | 0.41935 |
| 11416 | S14_7582217 | 14 | 7582217 | 88 | C | T | C | 0.90323 | T | 0.09677 | 6 | 0.19355 |
| 11417 | S14_7798136 | 14 | 7798136 | 215919 | T | A | A | 0.85484 | T | 0.14516 | 7 | 0.22581 |
| 11418 | S14_7798239 | 14 | 7798239 | 103 | T | C | T | 0.91935 | C | 0.08065 | 3 | 0.09677 |
| 11419 | S14_7798271 | 14 | 7798271 | 32 | G | A | G | 0.90323 | A | 0.09677 | 6 | 0.19355 |
| 11420 | S14_7896819 | 14 | 7896819 | 98548 | G | A | G | 0.8871 | A | 0.1129 | 5 | 0.16129 |
| 11421 | S14_7897021 | 14 | 7897021 | 202 | C | A | C | 0.90323 | A | 0.09677 | 6 | 0.19355 |
| 11422 | S14_7927054 | 14 | 7927054 | 30033 | C | G | C | 0.75806 | G | 0.24194 | 13 | 0.41935 |
| 11423 | S14_7927065 | 14 | 7927065 | 11 | G | A | G | 0.6129 | A | 0.3871 | 12 | 0.3871 |
| 11424 | S14_7927121 | 14 | 7927121 | 56 | G | T | G | 0.87097 | T | 0.12903 | 6 | 0.19355 |
| 11425 | S14_7927128 | 14 | 7927128 | 7 | C | A | A | 0.51613 | C | 0.48387 | 20 | 0.64516 |
| 11426 | S14_7927140 | 14 | 7927140 | 12 | C | T | C | 0.66129 | T | 0.33871 | 17 | 0.54839 |
| 11427 | S14_7927155 | 14 | 7927155 | 15 | A | G | A | 0.74194 | G | 0.25806 | 10 | 0.32258 |
| 11428 | S14_7927156 | 14 | 7927156 | 1 | T | G | T | 0.74194 | G | 0.25806 | 10 | 0.32258 |
| 11429 | S14_7937295 | 14 | 7937295 | 10139 | G | A | G | 0.85484 | A | 0.14516 | 9 | 0.29032 |
| 11430 | S14_7937305 | 14 | 7937305 | 10 | T | G | T | 0.6129 | G | 0.3871 | 16 | 0.51613 |
| 11431 | S14_7937321 | 14 | 7937321 | 16 | C | A | C | 0.85484 | A | 0.14516 | 9 | 0.29032 |
| 11432 | S14_7937327 | 14 | 7937327 | 6 | C | T | C | 0.6129 | T | 0.3871 | 16 | 0.51613 |
| 11433 | S14_7937389 | 14 | 7937389 | 62 | G | A | G | 0.80645 | A | 0.19355 | 10 | 0.32258 |
| 11434 | S14_8171814 | 14 | 8171814 | 234425 | G | A | G | 0.56452 | A | 0.43548 | 15 | 0.48387 |
| 11435 | S14_8172012 | 14 | 8172012 | 198 | G | C | G | 0.56452 | C | 0.43548 | 15 | 0.48387 |
| 11436 | S14_8172036 | 14 | 8172036 | 24 | C | T | C | 0.95161 | T | 0.04839 | 3 | 0.09677 |
| 11437 | S14_8515848 | 14 | 8515848 | 343812 | A | C | A | 0.90323 | C | 0.09677 | 6 | 0.19355 |
| 11438 | S14_8516087 | 14 | 8516087 | 239 | A | C | A | 0.75806 | C | 0.24194 | 9 | 0.29032 |
| 11439 | S14_9094175 | 14 | 9094175 | 578088 | C | T | C | 0.80645 | T | 0.19355 | 8 | 0.25806 |
| 11440 | S14_9094256 | 14 | 9094256 | 81 | A | T | A | 0.91935 | T | 0.08065 | 5 | 0.16129 |
| 11441 | S14_9322142 | 14 | 9322142 | 227886 | G | A | G | 0.79032 | A | 0.20968 | 9 | 0.29032 |
| 11442 | S14_9322351 | 14 | 9322351 | 209 | T | G | T | 0.8871 | G | 0.1129 | 5 | 0.16129 |
| 11443 | S14_9782371 | 14 | 9782371 | 460020 | A | T | T | 0.83871 | A | 0.16129 | 10 | 0.32258 |
| 11444 | S14_9831851 | 14 | 9831851 | 49480 | G | T | G | 0.91935 | T | 0.08065 | 5 | 0.16129 |
| 11445 | S14_9836930 | 14 | 9836930 | 5079 | A | G | G | 0.82258 | A | 0.17742 | 9 | 0.29032 |
| 11446 | S14_9836970 | 14 | 9836970 | 40 | C | T | T | 0.79032 | C | 0.20968 | 11 | 0.35484 |

| Site number | SNP name | Chromosome | Physical position | Physical distance from the previous SNP | Reference allele | Alternative allele | Major allele | Major allele frequency | Minor allele | Minor allele frequency | Number heterozygous | Heterozygosity |
|---|---|---|---|---|---|---|---|---|---|---|---|---|
| 11447 | S14_9836973 | 14 | 9836973 | 3 | T | G | T | 0.91935 | G | 0.08065 | 5 | 0.16129 |
| 11448 | S14_9837029 | 14 | 9837029 | 56 | C | T | T | 0.64516 | C | 0.35484 | 16 | 0.51613 |
| 11449 | S14_9837063 | 14 | 9837063 | 34 | A | G | G | 0.82258 | A | 0.17742 | 9 | 0.29032 |
| 11450 | S14_9865723 | 14 | 9865723 | 28660 | G | A | G | 0.91935 | A | 0.08065 | 3 | 0.09677 |
| 11451 | S14_9865908 | 14 | 9865908 | 185 | C | T | C | 0.66129 | T | 0.33871 | 11 | 0.35484 |
| 11452 | S14_9865929 | 14 | 9865929 | 21 | G | T | G | 0.53226 | T | 0.46774 | 15 | 0.48387 |
| 11453 | S14_10447476 | 14 | 10447476 | 581547 | C | T | C | 0.93548 | T | 0.06452 | 4 | 0.12903 |
| 11454 | S14_10675186 | 14 | 10675186 | 227710 | C | T | C | 0.69355 | T | 0.30645 | 13 | 0.41935 |
| 11455 | S14_10675231 | 14 | 10675231 | 45 | A | G | A | 0.90323 | G | 0.09677 | 6 | 0.19355 |
| 11456 | S14_10675277 | 14 | 10675277 | 46 | C | T | C | 0.74194 | T | 0.25806 | 12 | 0.3871 |
| 11457 | S14_10675285 | 14 | 10675285 | 8 | A | G | A | 0.74194 | G | 0.25806 | 12 | 0.3871 |
| 11458 | S14_10675376 | 14 | 10675376 | 91 | T | C | T | 0.8871 | C | 0.1129 | 5 | 0.16129 |
| 11459 | S14_10784384 | 14 | 10784384 | 109008 | A | G | A | 0.93548 | G | 0.06452 | 4 | 0.12903 |
| 11460 | S14_10784389 | 14 | 10784389 | 5 | A | G | A | 0.93548 | G | 0.06452 | 4 | 0.12903 |
| 11461 | S14_10784398 | 14 | 10784398 | 9 | A | G | A | 0.74194 | G | 0.25806 | 12 | 0.3871 |
| 11462 | S14_10784407 | 14 | 10784407 | 9 | C | T | C | 0.87097 | T | 0.12903 | 6 | 0.19355 |
| 11463 | S14_10784551 | 14 | 10784551 | 144 | C | T | C | 0.93548 | T | 0.06452 | 4 | 0.12903 |
| 11464 | S14_10948421 | 14 | 10948421 | 163870 | G | T | G | 0.53226 | T | 0.46774 | 15 | 0.48387 |
| 11465 | S14_10948563 | 14 | 10948563 | 142 | G | A | G | 0.69355 | A | 0.30645 | 11 | 0.35484 |
| 11466 | S14_10948590 | 14 | 10948590 | 27 | G | C | G | 0.80645 | C | 0.19355 | 8 | 0.25806 |
| 11467 | S14_11438834 | 14 | 11438834 | 490244 | T | C | C | 0.75806 | T | 0.24194 | 13 | 0.41935 |
| 11468 | S14_11438860 | 14 | 11438860 | 26 | C | G | C | 0.91935 | G | 0.08065 | 5 | 0.16129 |
| 11469 | S14_11575339 | 14 | 11575339 | 136479 | C | T | C | 0.79032 | T | 0.20968 | 9 | 0.29032 |
| 11470 | S14_11575360 | 14 | 11575360 | 21 | T | C | T | 0.79032 | C | 0.20968 | 9 | 0.29032 |
| 11471 | S14_11575536 | 14 | 11575536 | 176 | A | G | A | 0.79032 | G | 0.20968 | 9 | 0.29032 |
| 11472 | S14_12094470 | 14 | 12094470 | 518934 | C | T | C | 0.8871 | T | 0.1129 | 7 | 0.22581 |
| 11473 | S14_12209332 | 14 | 12209332 | 114862 | T | A | T | 0.95161 | A | 0.04839 | 3 | 0.09677 |
| 11474 | S14_12209364 | 14 | 12209364 | 32 | A | C | A | 0.72581 | C | 0.27419 | 13 | 0.41935 |
| 11475 | S14_12209500 | 14 | 12209500 | 136 | G | A | G | 0.90323 | A | 0.09677 | 4 | 0.12903 |
| 11476 | S14_12368701 | 14 | 12368701 | 159201 | T | C | T | 0.83871 | C | 0.16129 | 8 | 0.25806 |
| 11477 | S14_12514909 | 14 | 12514909 | 146208 | T | C | C | 0.77419 | T | 0.22581 | 12 | 0.3871 |
| 11478 | S14_12514981 | 14 | 12514981 | 72 | C | T | C | 0.95161 | T | 0.04839 | 3 | 0.09677 |
| 11479 | S14_12515039 | 14 | 12515039 | 58 | G | C | C | 0.77419 | G | 0.22581 | 12 | 0.3871 |
| 11480 | S14_12622254 | 14 | 12622254 | 107215 | A | G | A | 0.95161 | G | 0.04839 | 3 | 0.09677 |
| 11481 | S14_12622336 | 14 | 12622336 | 82 | C | T | C | 0.95161 | T | 0.04839 | 3 | 0.09677 |
| 11482 | S14_12733258 | 14 | 12733258 | 110922 | G | A | G | 0.91935 | A | 0.08065 | 5 | 0.16129 |
| 11483 | S14_13132916 | 14 | 13132916 | 399658 | G | A | G | 0.8871 | A | 0.1129 | 7 | 0.22581 |
| 11484 | S14_13132927 | 14 | 13132927 | 11 | T | C | T | 0.85484 | C | 0.14516 | 9 | 0.29032 |
| 11485 | S14_13132973 | 14 | 13132973 | 46 | C | T | C | 0.85484 | T | 0.14516 | 9 | 0.29032 |
| 11486 | S14_13133041 | 14 | 13133041 | 68 | G | A | G | 0.64516 | A | 0.35484 | 14 | 0.45161 |
| 11487 | S14_13362751 | 14 | 13362751 | 229710 | C | G | G | 0.64516 | C | 0.35484 | 10 | 0.32258 |
| 11488 | S14_13390488 | 14 | 13390488 | 27737 | A | G | G | 0.82258 | A | 0.17742 | 7 | 0.22581 |
| 11489 | S14_13414224 | 14 | 13414224 | 23736 | C | T | C | 0.8871 | T | 0.1129 | 5 | 0.16129 |
| 11490 | S14_13414234 | 14 | 13414234 | 10 | T | G | T | 0.8871 | G | 0.1129 | 5 | 0.16129 |
| 11491 | S14_13414252 | 14 | 13414252 | 18 | A | G | A | 0.8871 | G | 0.1129 | 5 | 0.16129 |
| 11492 | S14_13414268 | 14 | 13414268 | 16 | T | C | T | 0.91935 | C | 0.08065 | 5 | 0.16129 |
| 11493 | S14_13414292 | 14 | 13414292 | 24 | G | A | G | 0.70968 | A | 0.29032 | 14 | 0.45161 |
| 11494 | S14_13414377 | 14 | 13414377 | 85 | T | C | C | 0.54839 | T | 0.45161 | 16 | 0.51613 |
| 11495 | S14_13414437 | 14 | 13414437 | 60 | C | T | C | 0.70968 | T | 0.29032 | 14 | 0.45161 |
| 11496 | S14_13414439 | 14 | 13414439 | 2 | G | A | A | 0.54839 | G | 0.45161 | 16 | 0.51613 |
| 11497 | S14_13431772 | 14 | 13431772 | 17333 | G | A | G | 0.93548 | A | 0.06452 | 4 | 0.12903 |
| 11498 | S14_13498158 | 14 | 13498158 | 66386 | C | T | C | 0.93548 | T | 0.06452 | 4 | 0.12903 |
| 11499 | S14_13498225 | 14 | 13498225 | 67 | C | G | G | 0.75806 | C | 0.24194 | 11 | 0.35484 |
| 11500 | S14_13783448 | 14 | 13783448 | 285223 | G | A | G | 0.8871 | A | 0.1129 | 7 | 0.22581 |

| Site number | SNP name | Chromosome | Physical position | Physical distance from the previous SNP | Reference allele | Alternative allele | Major allele | Major allele frequency | Minor allele | Minor allele frequency | Number heterozygous | Heterozygosity |
|---|---|---|---|---|---|---|---|---|---|---|---|---|
| 11501 | S14_13783504 | 14 | 13783504 | 56 | C | T | C | 0.91935 | T | 0.08065 | 5 | 0.16129 |
| 11502 | S14_14125281 | 14 | 14125281 | 341777 | C | T | C | 0.64516 | T | 0.35484 | 18 | 0.58065 |
| 11503 | S14_14227976 | 14 | 14227976 | 102695 | C | T | C | 0.59677 | T | 0.40323 | 13 | 0.41935 |
| 11504 | S14_14227980 | 14 | 14227980 | 4 | T | C | T | 0.58065 | C | 0.41935 | 14 | 0.45161 |
| 11505 | S14_14228076 | 14 | 14228076 | 96 | C | T | C | 0.59677 | T | 0.40323 | 13 | 0.41935 |
| 11506 | S14_14228085 | 14 | 14228085 | 9 | C | T | C | 0.59677 | T | 0.40323 | 13 | 0.41935 |
| 11507 | S14_14394229 | 14 | 14394229 | 166144 | T | C | C | 0.90323 | T | 0.09677 | 4 | 0.12903 |
| 11508 | S14_14394320 | 14 | 14394320 | 91 | A | G | A | 0.93548 | G | 0.06452 | 4 | 0.12903 |
| 11509 | S14_14394410 | 14 | 14394410 | 90 | G | A | G | 0.75806 | A | 0.24194 | 13 | 0.41935 |
| 11510 | S14_14394440 | 14 | 14394440 | 30 | T | A | T | 0.75806 | A | 0.24194 | 13 | 0.41935 |
| 11511 | S14_14430503 | 14 | 14430503 | 36063 | A | G | A | 0.91935 | G | 0.08065 | 5 | 0.16129 |
| 11512 | S14_14430660 | 14 | 14430660 | 157 | A | C | A | 0.90323 | C | 0.09677 | 6 | 0.19355 |
| 11513 | S14_14963534 | 14 | 14963534 | 532874 | G | A | G | 0.54839 | A | 0.45161 | 14 | 0.45161 |
| 11514 | S14_15360004 | 14 | 15360004 | 396470 | T | C | T | 0.93548 | C | 0.06452 | 2 | 0.06452 |
| 11515 | S14_15438340 | 14 | 15438340 | 78336 | T | C | C | 0.70968 | T | 0.29032 | 10 | 0.32258 |
| 11516 | S14_15438341 | 14 | 15438341 | 1 | G | A | G | 0.93548 | A | 0.06452 | 4 | 0.12903 |
| 11517 | S14_15480980 | 14 | 15480980 | 42639 | A | C | A | 0.51613 | C | 0.48387 | 12 | 0.3871 |
| 11518 | S14_15480992 | 14 | 15480992 | 12 | C | T | C | 0.72581 | T | 0.27419 | 11 | 0.35484 |
| 11519 | S14_15485975 | 14 | 15485975 | 4983 | G | A | G | 0.93548 | A | 0.06452 | 4 | 0.12903 |
| 11520 | S14_15506300 | 14 | 15506300 | 20325 | G | A | G | 0.85484 | A | 0.14516 | 9 | 0.29032 |
| 11521 | S14_15652458 | 14 | 15652458 | 146158 | C | T | C | 0.8871 | T | 0.1129 | 7 | 0.22581 |
| 11522 | S14_15688689 | 14 | 15688689 | 36231 | A | G | G | 0.80645 | A | 0.19355 | 10 | 0.32258 |
| 11523 | S14_15688761 | 14 | 15688761 | 72 | T | C | C | 0.80645 | T | 0.19355 | 10 | 0.32258 |
| 11524 | S14_15688807 | 14 | 15688807 | 46 | T | C | T | 0.74194 | C | 0.25806 | 10 | 0.32258 |
| 11525 | S14_15735160 | 14 | 15735160 | 46353 | C | G | C | 0.85484 | G | 0.14516 | 5 | 0.16129 |
| 11526 | S14_15901369 | 14 | 15901369 | 166209 | G | A | G | 0.93548 | A | 0.06452 | 4 | 0.12903 |
| 11527 | S14_15901409 | 14 | 15901409 | 40 | C | A | C | 0.90323 | A | 0.09677 | 4 | 0.12903 |
| 11528 | S14_15901534 | 14 | 15901534 | 125 | C | T | T | 0.93548 | C | 0.06452 | 4 | 0.12903 |
| 11529 | S14_16191597 | 14 | 16191597 | 290063 | G | A | G | 0.83871 | A | 0.16129 | 8 | 0.25806 |
| 11530 | S14_16191755 | 14 | 16191755 | 158 | T | C | C | 0.93548 | T | 0.06452 | 2 | 0.06452 |
| 11531 | S14_16191756 | 14 | 16191756 | 1 | G | A | G | 0.91935 | A | 0.08065 | 5 | 0.16129 |
| 11532 | S14_16191838 | 14 | 16191838 | 82 | C | T | T | 0.58065 | C | 0.41935 | 14 | 0.45161 |
| 11533 | S14_16347609 | 14 | 16347609 | 155771 | C | T | C | 0.95161 | T | 0.04839 | 3 | 0.09677 |
| 11534 | S14_16381885 | 14 | 16381885 | 34276 | C | A | C | 0.82258 | A | 0.17742 | 7 | 0.22581 |
| 11535 | S14_16381896 | 14 | 16381896 | 11 | T | C | T | 0.8871 | C | 0.1129 | 7 | 0.22581 |
| 11536 | S14_16593876 | 14 | 16593876 | 211980 | T | C | C | 0.67742 | T | 0.32258 | 14 | 0.45161 |
| 11537 | S14_16991080 | 14 | 16991080 | 397204 | C | T | C | 0.93548 | T | 0.06452 | 4 | 0.12903 |
| 11538 | S14_17052091 | 14 | 17052091 | 61011 | C | T | C | 0.93548 | T | 0.06452 | 4 | 0.12903 |
| 11539 | S14_17052110 | 14 | 17052110 | 19 | G | A | G | 0.93548 | A | 0.06452 | 4 | 0.12903 |
| 11540 | S14_17929905 | 14 | 17929905 | 877795 | C | T | T | 0.62903 | C | 0.37097 | 17 | 0.54839 |
| 11541 | S14_18069343 | 14 | 18069343 | 139438 | C | T | T | 0.51613 | C | 0.48387 | 14 | 0.45161 |
| 11542 | S14_18069434 | 14 | 18069434 | 91 | A | G | G | 0.83871 | A | 0.16129 | 6 | 0.19355 |
| 11543 | S14_18164618 | 14 | 18164618 | 95184 | A | G | A | 0.58065 | G | 0.41935 | 18 | 0.58065 |
| 11544 | S14_18252599 | 14 | 18252599 | 87981 | C | T | C | 0.91935 | T | 0.08065 | 5 | 0.16129 |
| 11545 | S14_18252606 | 14 | 18252606 | 7 | A | G | A | 0.75806 | G | 0.24194 | 11 | 0.35484 |
| 11546 | S14_18252790 | 14 | 18252790 | 184 | C | G | C | 0.95161 | G | 0.04839 | 3 | 0.09677 |
| 11547 | S14_19058580 | 14 | 19058580 | 805790 | T | C | T | 0.85484 | C | 0.14516 | 7 | 0.22581 |
| 11548 | S14_19159796 | 14 | 19159796 | 101216 | A | G | G | 0.67742 | A | 0.32258 | 10 | 0.32258 |
| 11549 | S14_19159968 | 14 | 19159968 | 172 | G | C | G | 0.59677 | C | 0.40323 | 13 | 0.41935 |
| 11550 | S14_19536710 | 14 | 19536710 | 376742 | G | T | G | 0.83871 | T | 0.16129 | 8 | 0.25806 |
| 11551 | S14_19536780 | 14 | 19536780 | 70 | A | T | A | 0.83871 | T | 0.16129 | 8 | 0.25806 |
| 11552 | S14_19536804 | 14 | 19536804 | 24 | G | A | G | 0.83871 | A | 0.16129 | 8 | 0.25806 |
| 11553 | S14_19613530 | 14 | 19613530 | 76726 | C | T | C | 0.95161 | T | 0.04839 | 3 | 0.09677 |
| 11554 | S14_19876687 | 14 | 19876687 | 263157 | G | A | G | 0.64516 | A | 0.35484 | 14 | 0.45161 |

| Site number | SNP name | Chromosome | Physical position | Physical distance from the previous SNP | Reference allele | Alternative allele | Major allele | Major allele frequency | Minor allele | Minor allele frequency | Number heterozygous | Heterozygosity |
|---|---|---|---|---|---|---|---|---|---|---|---|---|
| 11555 | S14_20039674 | 14 | 20039674 | 162987 | C | T | C | 0.93548 | T | 0.06452 | 4 | 0.12903 |
| 11556 | S14_20039834 | 14 | 20039834 | 160 | T | A | T | 0.93548 | A | 0.06452 | 4 | 0.12903 |
| 11557 | S14_20039849 | 14 | 20039849 | 15 | G | A | G | 0.93548 | A | 0.06452 | 4 | 0.12903 |
| 11558 | S14_20343035 | 14 | 20343035 | 303186 | T | C | T | 0.80645 | C | 0.19355 | 10 | 0.32258 |
| 11559 | S14_20343039 | 14 | 20343039 | 4 | C | A | C | 0.80645 | A | 0.19355 | 10 | 0.32258 |
| 11560 | S14_20343050 | 14 | 20343050 | 11 | T | C | T | 0.80645 | C | 0.19355 | 10 | 0.32258 |
| 11561 | S14_20343146 | 14 | 20343146 | 96 | A | G | A | 0.80645 | G | 0.19355 | 10 | 0.32258 |
| 11562 | S14_20343163 | 14 | 20343163 | 17 | G | A | G | 0.80645 | A | 0.19355 | 10 | 0.32258 |
| 11563 | S14_20346335 | 14 | 20346335 | 3172 | C | T | C | 0.77419 | T | 0.22581 | 12 | 0.3871 |
| 11564 | S14_20346337 | 14 | 20346337 | 2 | C | T | C | 0.77419 | T | 0.22581 | 12 | 0.3871 |
| 11565 | S14_20441615 | 14 | 20441615 | 95278 | C | T | T | 0.58065 | C | 0.41935 | 18 | 0.58065 |
| 11566 | S14_20441666 | 14 | 20441666 | 51 | T | A | A | 0.58065 | T | 0.41935 | 18 | 0.58065 |
| 11567 | S14_20441759 | 14 | 20441759 | 93 | G | T | T | 0.58065 | G | 0.41935 | 18 | 0.58065 |
| 11568 | S14_20441822 | 14 | 20441822 | 63 | A | G | A | 0.8871 | G | 0.1129 | 7 | 0.22581 |
| 11569 | S14_20669124 | 14 | 20669124 | 227302 | C | T | C | 0.77419 | T | 0.22581 | 12 | 0.3871 |
| 11570 | S14_20861286 | 14 | 20861286 | 192162 | C | A | C | 0.95161 | A | 0.04839 | 3 | 0.09677 |
| 11571 | S14_20888135 | 14 | 20888135 | 26849 | G | A | A | 0.80645 | G | 0.19355 | 10 | 0.32258 |
| 11572 | S14_20888189 | 14 | 20888189 | 54 | T | C | C | 0.83871 | T | 0.16129 | 8 | 0.25806 |
| 11573 | S14_20888295 | 14 | 20888295 | 106 | C | G | G | 0.74194 | C | 0.25806 | 12 | 0.3871 |
| 11574 | S14_20888330 | 14 | 20888330 | 35 | C | T | C | 0.64516 | T | 0.35484 | 14 | 0.45161 |
| 11575 | S14_20888356 | 14 | 20888356 | 26 | G | A | G | 0.77419 | A | 0.22581 | 10 | 0.32258 |
| 11576 | S14_21078487 | 14 | 21078487 | 190131 | T | C | C | 0.74194 | T | 0.25806 | 8 | 0.25806 |
| 11577 | S14_21078488 | 14 | 21078488 | 1 | G | C | C | 0.74194 | G | 0.25806 | 8 | 0.25806 |
| 11578 | S14_21574087 | 14 | 21574087 | 495599 | G | A | G | 0.82258 | A | 0.17742 | 9 | 0.29032 |
| 11579 | S14_21708046 | 14 | 21708046 | 133959 | G | A | G | 0.83871 | A | 0.16129 | 8 | 0.25806 |
| 11580 | S14_21708152 | 14 | 21708152 | 106 | A | G | A | 0.62903 | G | 0.37097 | 17 | 0.54839 |
| 11581 | S14_22321716 | 14 | 22321716 | 613564 | C | T | C | 0.80645 | T | 0.19355 | 10 | 0.32258 |
| 11582 | S14_22321805 | 14 | 22321805 | 89 | A | G | A | 0.87097 | G | 0.12903 | 8 | 0.25806 |
| 11583 | S14_22321857 | 14 | 22321857 | 52 | A | G | G | 0.79032 | A | 0.20968 | 11 | 0.35484 |
| 11584 | S14_22329185 | 14 | 22329185 | 7328 | C | T | C | 0.5 | T | 0.5 | 13 | 0.41935 |
| 11585 | S14_22329205 | 14 | 22329205 | 20 | C | T | C | 0.93548 | T | 0.06452 | 4 | 0.12903 |
| 11586 | S14_22329232 | 14 | 22329232 | 27 | T | G | G | 0.56452 | T | 0.43548 | 15 | 0.48387 |
| 11587 | S14_22329247 | 14 | 22329247 | 15 | G | A | G | 0.8871 | A | 0.1129 | 7 | 0.22581 |
| 11588 | S14_22329319 | 14 | 22329319 | 72 | G | A | G | 0.54839 | A | 0.45161 | 12 | 0.3871 |
| 11589 | S14_22329338 | 14 | 22329338 | 19 | A | T | A | 0.8871 | T | 0.1129 | 7 | 0.22581 |
| 11590 | S14_22911392 | 14 | 22911392 | 582054 | C | T | C | 0.95161 | T | 0.04839 | 3 | 0.09677 |
| 11591 | S14_23230013 | 14 | 23230013 | 318621 | G | T | G | 0.80645 | T | 0.19355 | 10 | 0.32258 |
| 11592 | S14_23230035 | 14 | 23230035 | 22 | C | T | C | 0.95161 | T | 0.04839 | 3 | 0.09677 |
| 11593 | S14_23763175 | 14 | 23763175 | 533140 | G | A | G | 0.90323 | A | 0.09677 | 6 | 0.19355 |
| 11594 | S14_23763245 | 14 | 23763245 | 70 | G | A | G | 0.51613 | A | 0.48387 | 14 | 0.45161 |
| 11595 | S14_23872670 | 14 | 23872670 | 109425 | C | T | C | 0.90323 | T | 0.09677 | 6 | 0.19355 |
| 11596 | S14_23872736 | 14 | 23872736 | 66 | G | A | G | 0.95161 | A | 0.04839 | 3 | 0.09677 |
| 11597 | S14_23872857 | 14 | 23872857 | 121 | T | C | T | 0.8871 | C | 0.1129 | 7 | 0.22581 |
| 11598 | S14_24120135 | 14 | 24120135 | 247278 | G | T | G | 0.79032 | T | 0.20968 | 9 | 0.29032 |
| 11599 | S14_24120275 | 14 | 24120275 | 140 | C | T | C | 0.90323 | T | 0.09677 | 6 | 0.19355 |
| 11600 | S14_24470970 | 14 | 24470970 | 350695 | G | T | G | 0.54839 | T | 0.45161 | 12 | 0.3871 |
| 11601 | S14_24600775 | 14 | 24600775 | 129805 | G | A | A | 0.66129 | G | 0.33871 | 11 | 0.35484 |
| 11602 | S14_24781511 | 14 | 24781511 | 180736 | T | C | T | 0.74194 | C | 0.25806 | 12 | 0.3871 |
| 11603 | S14_25248060 | 14 | 25248060 | 466549 | C | T | C | 0.83871 | T | 0.16129 | 10 | 0.32258 |
| 11604 | S14_25248109 | 14 | 25248109 | 49 | C | T | C | 0.8871 | T | 0.1129 | 7 | 0.22581 |
| 11605 | S14_25248135 | 14 | 25248135 | 26 | G | A | A | 0.51613 | G | 0.48387 | 18 | 0.58065 |
| 11606 | S14_25248168 | 14 | 25248168 | 33 | A | G | G | 0.51613 | A | 0.48387 | 18 | 0.58065 |
| 11607 | S14_26450856 | 14 | 26450856 | 1202688 | G | A | A | 0.90323 | G | 0.09677 | 6 | 0.19355 |
| 11608 | S14_26960767 | 14 | 26960767 | 509911 | A | G | A | 0.82258 | G | 0.17742 | 7 | 0.22581 |

| Site number | SNP name | Chromosome | Physical position | Physical distance from the previous SNP | Reference allele | Alternative allele | Major allele | Major allele frequency | Minor allele | Minor allele frequency | Number heterozygous | Heterozygosity |
|---|---|---|---|---|---|---|---|---|---|---|---|---|
| 11609 | S14_26960835 | 14 | 26960835 | 68 | C | T | C | 0.82258 | T | 0.17742 | 7 | 0.22581 |
| 11610 | S14_27554204 | 14 | 27554204 | 593369 | G | A | G | 0.91935 | A | 0.08065 | 5 | 0.16129 |
| 11611 | S14_27554224 | 14 | 27554224 | 20 | G | C | G | 0.64516 | C | 0.35484 | 16 | 0.51613 |
| 11612 | S14_27554251 | 14 | 27554251 | 27 | C | T | C | 0.67742 | T | 0.32258 | 16 | 0.51613 |
| 11613 | S14_27554254 | 14 | 27554254 | 3 | A | G | A | 0.67742 | G | 0.32258 | 16 | 0.51613 |
| 11614 | S14_27554300 | 14 | 27554300 | 46 | C | T | C | 0.64516 | T | 0.35484 | 16 | 0.51613 |
| 11615 | S14_28470105 | 14 | 28470105 | 915805 | A | G | A | 0.85484 | G | 0.14516 | 7 | 0.22581 |
| 11616 | S14_28470184 | 14 | 28470184 | 79 | A | T | A | 0.77419 | T | 0.22581 | 10 | 0.32258 |
| 11617 | S14_28470217 | 14 | 28470217 | 33 | T | C | C | 0.5 | T | 0.5 | 15 | 0.48387 |
| 11618 | S14_28470222 | 14 | 28470222 | 5 | G | T | G | 0.93548 | T | 0.06452 | 4 | 0.12903 |
| 11619 | S14_28470234 | 14 | 28470234 | 12 | C | G | C | 0.64516 | G | 0.35484 | 16 | 0.51613 |
| 11620 | S14_28470256 | 14 | 28470256 | 22 | A | G | A | 0.64516 | G | 0.35484 | 16 | 0.51613 |
| 11621 | S14_28470285 | 14 | 28470285 | 29 | A | G | A | 0.80645 | G | 0.19355 | 12 | 0.3871 |
| 11622 | S14_28499032 | 14 | 28499032 | 28747 | C | G | C | 0.93548 | G | 0.06452 | 4 | 0.12903 |
| 11623 | S14_28894953 | 14 | 28894953 | 395921 | G | A | G | 0.69355 | A | 0.30645 | 17 | 0.54839 |
| 11624 | S14_28895035 | 14 | 28895035 | 82 | C | T | C | 0.91935 | T | 0.08065 | 5 | 0.16129 |
| 11625 | S14_29820736 | 14 | 29820736 | 925701 | G | A | G | 0.64516 | A | 0.35484 | 16 | 0.51613 |
| 11626 | S14_29820785 | 14 | 29820785 | 49 | C | T | C | 0.90323 | T | 0.09677 | 6 | 0.19355 |
| 11627 | S14_29820788 | 14 | 29820788 | 3 | A | G | A | 0.95161 | G | 0.04839 | 1 | 0.03226 |
| 11628 | S14_30458286 | 14 | 30458286 | 637498 | G | A | G | 0.90323 | A | 0.09677 | 6 | 0.19355 |
| 11629 | S14_30458400 | 14 | 30458400 | 114 | T | C | T | 0.64516 | C | 0.35484 | 18 | 0.58065 |
| 11630 | S14_31560568 | 14 | 31560568 | 1102168 | G | T | T | 0.54839 | G | 0.45161 | 14 | 0.45161 |
| 11631 | S14_31560621 | 14 | 31560621 | 53 | G | A | G | 0.69355 | A | 0.30645 | 13 | 0.41935 |
| 11632 | S14_32346771 | 14 | 32346771 | 786150 | G | A | G | 0.83871 | A | 0.16129 | 6 | 0.19355 |
| 11633 | S14_32346782 | 14 | 32346782 | 11 | T | C | T | 0.58065 | C | 0.41935 | 14 | 0.45161 |
| 11634 | S14_32411014 | 14 | 32411014 | 64232 | G | A | G | 0.93548 | A | 0.06452 | 4 | 0.12903 |
| 11635 | S14_32411123 | 14 | 32411123 | 109 | C | A | C | 0.95161 | A | 0.04839 | 3 | 0.09677 |
| 11636 | S14_32536028 | 14 | 32536028 | 124905 | G | T | G | 0.93548 | T | 0.06452 | 4 | 0.12903 |
| 11637 | S14_32536057 | 14 | 32536057 | 29 | G | A | G | 0.91935 | A | 0.08065 | 5 | 0.16129 |
| 11638 | S14_32536087 | 14 | 32536087 | 30 | T | C | T | 0.95161 | C | 0.04839 | 3 | 0.09677 |
| 11639 | S14_32675937 | 14 | 32675937 | 139850 | T | C | T | 0.69355 | C | 0.30645 | 15 | 0.48387 |
| 11640 | S14_32676012 | 14 | 32676012 | 75 | C | T | T | 0.75806 | C | 0.24194 | 9 | 0.29032 |
| 11641 | S14_32676032 | 14 | 32676032 | 20 | A | G | A | 0.54839 | G | 0.45161 | 10 | 0.32258 |
| 11642 | S14_32676139 | 14 | 32676139 | 107 | A | G | A | 0.69355 | G | 0.30645 | 15 | 0.48387 |
| 11643 | S14_32676172 | 14 | 32676172 | 33 | C | T | T | 0.54839 | C | 0.45161 | 10 | 0.32258 |
| 11644 | S14_33287093 | 14 | 33287093 | 610921 | T | A | A | 0.62903 | T | 0.37097 | 9 | 0.29032 |
| 11645 | S14_33853860 | 14 | 33853860 | 566767 | A | G | G | 0.85484 | A | 0.14516 | 9 | 0.29032 |
| 11646 | S14_33917401 | 14 | 33917401 | 63541 | G | A | G | 0.91935 | A | 0.08065 | 3 | 0.09677 |
| 11647 | S14_33917519 | 14 | 33917519 | 118 | T | C | T | 0.83871 | C | 0.16129 | 8 | 0.25806 |
| 11648 | S14_34004541 | 14 | 34004541 | 87022 | G | C | G | 0.56452 | C | 0.43548 | 13 | 0.41935 |
| 11649 | S14_34004570 | 14 | 34004570 | 29 | T | G | T | 0.90323 | G | 0.09677 | 4 | 0.12903 |
| 11650 | S14_34004646 | 14 | 34004646 | 76 | G | A | G | 0.90323 | A | 0.09677 | 4 | 0.12903 |
| 11651 | S14_34059844 | 14 | 34059844 | 55198 | T | C | T | 0.87097 | C | 0.12903 | 6 | 0.19355 |
| 11652 | S14_34059867 | 14 | 34059867 | 23 | G | A | G | 0.80645 | A | 0.19355 | 6 | 0.19355 |
| 11653 | S14_34107027 | 14 | 34107027 | 47160 | C | T | C | 0.93548 | T | 0.06452 | 4 | 0.12903 |
| 11654 | S14_34107041 | 14 | 34107041 | 14 | A | G | G | 0.93548 | A | 0.06452 | 4 | 0.12903 |
| 11655 | S14_34251906 | 14 | 34251906 | 144865 | G | A | G | 0.6129 | A | 0.3871 | 16 | 0.51613 |
| 11656 | S14_34251931 | 14 | 34251931 | 25 | G | A | G | 0.6129 | A | 0.3871 | 16 | 0.51613 |
| 11657 | S14_34784899 | 14 | 34784899 | 532968 | G | C | G | 0.59677 | C | 0.40323 | 11 | 0.35484 |
| 11658 | S14_34784914 | 14 | 34784914 | 15 | A | G | A | 0.77419 | G | 0.22581 | 8 | 0.25806 |
| 11659 | S14_34829383 | 14 | 34829383 | 44469 | T | C | C | 0.87097 | T | 0.12903 | 6 | 0.19355 |
| 11660 | S14_35159997 | 14 | 35159997 | 330614 | A | G | A | 0.80645 | G | 0.19355 | 12 | 0.3871 |
| 11661 | S14_35427302 | 14 | 35427302 | 267305 | C | A | C | 0.91935 | A | 0.08065 | 5 | 0.16129 |
| 11662 | S14_35517684 | 14 | 35517684 | 90382 | A | G | A | 0.72581 | G | 0.27419 | 13 | 0.41935 |

| Site number | SNP name | Chromosome | Physical position | Physical distance from the previous SNP | Reference allele | Alternative allele | Major allele | Major allele frequency | Minor allele | Minor allele frequency | Number heterozygous | Heterozygosity |
|---|---|---|---|---|---|---|---|---|---|---|---|---|
| 11663 | S14_35517780 | 14 | 35517780 | 96 | T | A | T | 0.72581 | A | 0.27419 | 13 | 0.41935 |
| 11664 | S14_35614091 | 14 | 35614091 | 96311 | C | T | C | 0.93548 | T | 0.06452 | 4 | 0.12903 |
| 11665 | S14_35800457 | 14 | 35800457 | 186366 | G | A | A | 0.62903 | G | 0.37097 | 15 | 0.48387 |
| 11666 | S14_36927259 | 14 | 36927259 | 1126802 | G | A | G | 0.95161 | A | 0.04839 | 3 | 0.09677 |
| 11667 | S14_37298725 | 14 | 37298725 | 371466 | A | G | A | 0.91935 | G | 0.08065 | 5 | 0.16129 |
| 11668 | S14_37446570 | 14 | 37446570 | 147845 | G | T | G | 0.93548 | T | 0.06452 | 2 | 0.06452 |
| 11669 | S14_37446718 | 14 | 37446718 | 148 | C | T | C | 0.72581 | T | 0.27419 | 9 | 0.29032 |
| 11670 | S14_37691512 | 14 | 37691512 | 244794 | C | T | C | 0.58065 | T | 0.41935 | 18 | 0.58065 |
| 11671 | S14_37691606 | 14 | 37691606 | 94 | A | G | A | 0.82258 | G | 0.17742 | 11 | 0.35484 |
| 11672 | S14_37811378 | 14 | 37811378 | 119772 | G | C | G | 0.85484 | C | 0.14516 | 9 | 0.29032 |
| 11673 | S14_37827178 | 14 | 37827178 | 15800 | C | T | C | 0.91935 | T | 0.08065 | 5 | 0.16129 |
| 11674 | S14_37827179 | 14 | 37827179 | 1 | G | A | G | 0.75806 | A | 0.24194 | 9 | 0.29032 |
| 11675 | S14_37827235 | 14 | 37827235 | 56 | C | T | C | 0.91935 | T | 0.08065 | 5 | 0.16129 |
| 11676 | S14_37847022 | 14 | 37847022 | 19787 | G | C | G | 0.93548 | C | 0.06452 | 2 | 0.06452 |
| 11677 | S14_38332212 | 14 | 38332212 | 485190 | T | C | C | 0.74194 | T | 0.25806 | 12 | 0.3871 |
| 11678 | S14_38466416 | 14 | 38466416 | 134204 | T | A | T | 0.8871 | A | 0.1129 | 5 | 0.16129 |
| 11679 | S14_38466458 | 14 | 38466458 | 42 | T | C | T | 0.72581 | C | 0.27419 | 15 | 0.48387 |
| 11680 | S14_38466460 | 14 | 38466460 | 2 | T | C | T | 0.72581 | C | 0.27419 | 15 | 0.48387 |
| 11681 | S14_38466539 | 14 | 38466539 | 79 | A | G | G | 0.8871 | A | 0.1129 | 5 | 0.16129 |
| 11682 | S14_38466587 | 14 | 38466587 | 48 | T | C | C | 0.79032 | T | 0.20968 | 13 | 0.41935 |
| 11683 | S14_38527497 | 14 | 38527497 | 60910 | T | C | T | 0.69355 | C | 0.30645 | 13 | 0.41935 |
| 11684 | S14_38636042 | 14 | 38636042 | 108545 | T | C | T | 0.69355 | C | 0.30645 | 13 | 0.41935 |
| 11685 | S14_38884154 | 14 | 38884154 | 248112 | A | G | G | 0.82258 | A | 0.17742 | 11 | 0.35484 |
| 11686 | S14_38884389 | 14 | 38884389 | 235 | C | T | C | 0.91935 | T | 0.08065 | 5 | 0.16129 |
| 11687 | S14_39037789 | 14 | 39037789 | 153400 | G | A | G | 0.79032 | A | 0.20968 | 9 | 0.29032 |
| 11688 | S14_39192513 | 14 | 39192513 | 154724 | G | T | T | 0.59677 | G | 0.40323 | 15 | 0.48387 |
| 11689 | S14_39211455 | 14 | 39211455 | 18942 | G | A | G | 0.54839 | A | 0.45161 | 12 | 0.3871 |
| 11690 | S14_39211578 | 14 | 39211578 | 123 | C | T | C | 0.95161 | T | 0.04839 | 3 | 0.09677 |
| 11691 | S14_39734684 | 14 | 39734684 | 523106 | G | A | G | 0.75806 | A | 0.24194 | 11 | 0.35484 |
| 11692 | S14_39734751 | 14 | 39734751 | 67 | C | T | C | 0.85484 | T | 0.14516 | 7 | 0.22581 |
| 11693 | S14_39734795 | 14 | 39734795 | 44 | G | A | G | 0.83871 | A | 0.16129 | 8 | 0.25806 |
| 11694 | S14_40337955 | 14 | 40337955 | 603160 | A | C | A | 0.85484 | C | 0.14516 | 9 | 0.29032 |
| 11695 | S14_40338046 | 14 | 40338046 | 91 | G | A | G | 0.87097 | A | 0.12903 | 8 | 0.25806 |
| 11696 | S14_40762327 | 14 | 40762327 | 424281 | G | A | G | 0.93548 | A | 0.06452 | 4 | 0.12903 |
| 11697 | S14_41019967 | 14 | 41019967 | 257640 | T | C | T | 0.8871 | C | 0.1129 | 7 | 0.22581 |
| 11698 | S14_41020018 | 14 | 41020018 | 51 | A | G | G | 0.59677 | A | 0.40323 | 19 | 0.6129 |
| 11699 | S14_42091226 | 14 | 42091226 | 1071208 | G | C | C | 0.93548 | G | 0.06452 | 4 | 0.12903 |
| 11700 | S14_42091239 | 14 | 42091239 | 13 | G | A | G | 0.85484 | A | 0.14516 | 7 | 0.22581 |
| 11701 | S14_42502551 | 14 | 42502551 | 411312 | A | G | A | 0.95161 | G | 0.04839 | 3 | 0.09677 |
| 11702 | S14_43946529 | 14 | 43946529 | 1443978 | T | C | C | 0.5 | T | 0.5 | 17 | 0.54839 |
| 11703 | S14_44300761 | 14 | 44300761 | 354232 | A | G | G | 0.80645 | A | 0.19355 | 10 | 0.32258 |
| 11704 | S14_44536713 | 14 | 44536713 | 235952 | G | A | G | 0.69355 | A | 0.30645 | 17 | 0.54839 |
| 11705 | S14_44585549 | 14 | 44585549 | 48836 | G | C | G | 0.80645 | C | 0.19355 | 12 | 0.3871 |
| 11706 | S14_44918269 | 14 | 44918269 | 332720 | A | G | A | 0.93548 | G | 0.06452 | 4 | 0.12903 |
| 11707 | S14_44953868 | 14 | 44953868 | 35599 | G | A | G | 0.70968 | A | 0.29032 | 18 | 0.58065 |
| 11708 | S14_45234449 | 14 | 45234449 | 280581 | A | G | A | 0.8871 | G | 0.1129 | 7 | 0.22581 |
| 11709 | S14_45234467 | 14 | 45234467 | 18 | T | C | T | 0.85484 | C | 0.14516 | 7 | 0.22581 |
| 11710 | S14_45234503 | 14 | 45234503 | 36 | G | A | G | 0.91935 | A | 0.08065 | 5 | 0.16129 |
| 11711 | S14_45234545 | 14 | 45234545 | 42 | C | T | C | 0.90323 | T | 0.09677 | 6 | 0.19355 |
| 11712 | S14_45234648 | 14 | 45234648 | 103 | A | C | A | 0.62903 | C | 0.37097 | 19 | 0.6129 |
| 11713 | S14_45514914 | 14 | 45514914 | 280266 | A | G | A | 0.74194 | G | 0.25806 | 16 | 0.51613 |
| 11714 | S14_45515087 | 14 | 45515087 | 173 | G | A | G | 0.58065 | A | 0.41935 | 18 | 0.58065 |
| 11715 | S14_45515092 | 14 | 45515092 | 5 | A | G | A | 0.58065 | G | 0.41935 | 18 | 0.58065 |
| 11716 | S14_45515166 | 14 | 45515166 | 74 | T | C | C | 0.67742 | T | 0.32258 | 12 | 0.3871 |

| Site number | SNP name | Chromosome | Physical position | Physical distance from the previous SNP | Reference allele | Alternative allele | Major allele | Major allele frequency | Minor allele | Minor allele frequency | Number heterozygous | Heterozygosity |
|---|---|---|---|---|---|---|---|---|---|---|---|---|
| 11717 | S14_45567548 | 14 | 45567548 | 52382 | T | C | T | 0.74194 | C | 0.25806 | 12 | 0.3871 |
| 11718 | S14_45567613 | 14 | 45567613 | 65 | A | G | A | 0.87097 | G | 0.12903 | 8 | 0.25806 |
| 11719 | S14_45567651 | 14 | 45567651 | 38 | C | T | C | 0.77419 | T | 0.22581 | 12 | 0.3871 |
| 11720 | S14_45567673 | 14 | 45567673 | 22 | A | G | A | 0.93548 | G | 0.06452 | 4 | 0.12903 |
| 11721 | S14_45663150 | 14 | 45663150 | 95477 | T | C | T | 0.82258 | C | 0.17742 | 9 | 0.29032 |
| 11722 | S14_45663185 | 14 | 45663185 | 35 | G | C | G | 0.82258 | C | 0.17742 | 9 | 0.29032 |
| 11723 | S14_46916314 | 14 | 46916314 | 1253129 | A | C | A | 0.77419 | C | 0.22581 | 14 | 0.45161 |
| 11724 | S14_46916315 | 14 | 46916315 | 1 | G | A | G | 0.77419 | A | 0.22581 | 14 | 0.45161 |
| 11725 | S14_46916380 | 14 | 46916380 | 65 | A | G | G | 0.80645 | A | 0.19355 | 10 | 0.32258 |
| 11726 | S14_48206139 | 14 | 48206139 | 1289759 | C | T | C | 0.87097 | T | 0.12903 | 6 | 0.19355 |
| 11727 | S14_48435774 | 14 | 48435774 | 229635 | G | C | C | 0.79032 | G | 0.20968 | 9 | 0.29032 |
| 11728 | S14_48435791 | 14 | 48435791 | 17 | C | T | C | 0.90323 | T | 0.09677 | 6 | 0.19355 |
| 11729 | S14_48470140 | 14 | 48470140 | 34349 | G | T | G | 0.91935 | T | 0.08065 | 5 | 0.16129 |
| 11730 | S14_48470215 | 14 | 48470215 | 75 | G | A | G | 0.93548 | A | 0.06452 | 2 | 0.06452 |
| 11731 | S14_48470251 | 14 | 48470251 | 36 | G | A | G | 0.91935 | A | 0.08065 | 3 | 0.09677 |
| 11732 | S14_48470312 | 14 | 48470312 | 61 | A | G | A | 0.90323 | G | 0.09677 | 6 | 0.19355 |
| 11733 | S14_48479707 | 14 | 48479707 | 9395 | G | A | G | 0.90323 | A | 0.09677 | 6 | 0.19355 |
| 11734 | S14_48495630 | 14 | 48495630 | 15923 | C | T | T | 0.58065 | C | 0.41935 | 14 | 0.45161 |
| 11735 | S14_48495762 | 14 | 48495762 | 132 | C | T | C | 0.8871 | T | 0.1129 | 7 | 0.22581 |
| 11736 | S14_48495836 | 14 | 48495836 | 74 | T | C | C | 0.93548 | T | 0.06452 | 4 | 0.12903 |
| 11737 | S14_48495846 | 14 | 48495846 | 10 | G | A | A | 0.58065 | G | 0.41935 | 14 | 0.45161 |
| 11738 | S14_48523850 | 14 | 48523850 | 28004 | G | A | G | 0.70968 | A | 0.29032 | 8 | 0.25806 |
| 11739 | S14_48523936 | 14 | 48523936 | 86 | C | T | C | 0.59677 | T | 0.40323 | 13 | 0.41935 |
| 11740 | S14_48589257 | 14 | 48589257 | 65321 | C | T | C | 0.69355 | T | 0.30645 | 11 | 0.35484 |
| 11741 | S14_48589377 | 14 | 48589377 | 120 | T | G | T | 0.95161 | G | 0.04839 | 1 | 0.03226 |
| 11742 | S14_48851255 | 14 | 48851255 | 261878 | G | T | G | 0.85484 | T | 0.14516 | 7 | 0.22581 |
| 11743 | S14_48851329 | 14 | 48851329 | 74 | T | A | T | 0.95161 | A | 0.04839 | 1 | 0.03226 |
| 11744 | S14_48980737 | 14 | 48980737 | 129408 | G | A | G | 0.90323 | A | 0.09677 | 4 | 0.12903 |
| 11745 | S14_49121271 | 14 | 49121271 | 140534 | A | G | A | 0.66129 | G | 0.33871 | 13 | 0.41935 |
| 11746 | S14_49121327 | 14 | 49121327 | 56 | C | T | C | 0.79032 | T | 0.20968 | 11 | 0.35484 |
| 11747 | S14_49121335 | 14 | 49121335 | 8 | C | T | C | 0.82258 | T | 0.17742 | 7 | 0.22581 |
| 11748 | S14_49121398 | 14 | 49121398 | 63 | C | T | C | 0.82258 | T | 0.17742 | 7 | 0.22581 |
| 11749 | S14_49121433 | 14 | 49121433 | 35 | A | G | A | 0.87097 | G | 0.12903 | 8 | 0.25806 |
| 11750 | S14_49121434 | 14 | 49121434 | 1 | G | A | G | 0.87097 | A | 0.12903 | 8 | 0.25806 |
| 11751 | S14_49121467 | 14 | 49121467 | 33 | C | T | C | 0.79032 | T | 0.20968 | 11 | 0.35484 |
| 11752 | S14_49121469 | 14 | 49121469 | 2 | G | A | G | 0.93548 | A | 0.06452 | 4 | 0.12903 |
| 11753 | S14_49121510 | 14 | 49121510 | 41 | T | C | T | 0.82258 | C | 0.17742 | 7 | 0.22581 |
| 11754 | S14_50100666 | 14 | 50100666 | 979156 | A | G | G | 0.58065 | A | 0.41935 | 20 | 0.64516 |
| 11755 | S14_51142036 | 14 | 51142036 | 1041370 | C | G | C | 0.79032 | G | 0.20968 | 9 | 0.29032 |
| 11756 | S14_51718864 | 14 | 51718864 | 576828 | C | T | C | 0.93548 | T | 0.06452 | 4 | 0.12903 |
| 11757 | S14_52001860 | 14 | 52001860 | 282996 | G | C | C | 0.90323 | G | 0.09677 | 6 | 0.19355 |
| 11758 | S14_52001920 | 14 | 52001920 | 60 | A | G | G | 0.87097 | A | 0.12903 | 6 | 0.19355 |
| 11759 | S14_52215259 | 14 | 52215259 | 213339 | G | A | G | 0.93548 | A | 0.06452 | 4 | 0.12903 |
| 11760 | S14_52220245 | 14 | 52220245 | 4986 | C | A | C | 0.74194 | A | 0.25806 | 12 | 0.3871 |
| 11761 | S14_52220253 | 14 | 52220253 | 8 | C | T | C | 0.87097 | T | 0.12903 | 6 | 0.19355 |
| 11762 | S14_52458333 | 14 | 52458333 | 238080 | G | A | G | 0.80645 | A | 0.19355 | 8 | 0.25806 |
| 11763 | S14_52503624 | 14 | 52503624 | 45291 | C | T | C | 0.80645 | T | 0.19355 | 10 | 0.32258 |
| 11764 | S14_52503659 | 14 | 52503659 | 35 | C | T | C | 0.91935 | T | 0.08065 | 5 | 0.16129 |
| 11765 | S14_52663241 | 14 | 52663241 | 159582 | A | G | A | 0.77419 | G | 0.22581 | 14 | 0.45161 |
| 11766 | S14_52858142 | 14 | 52858142 | 194901 | C | T | C | 0.93548 | T | 0.06452 | 4 | 0.12903 |
| 11767 | S14_52858143 | 14 | 52858143 | 1 | G | A | G | 0.95161 | A | 0.04839 | 1 | 0.03226 |
| 11768 | S14_52858162 | 14 | 52858162 | 19 | T | C | T | 0.54839 | C | 0.45161 | 14 | 0.45161 |
| 11769 | S14_52941517 | 14 | 52941517 | 83355 | T | C | C | 0.51613 | T | 0.48387 | 10 | 0.32258 |
| 11770 | S14_52941532 | 14 | 52941532 | 15 | G | C | G | 0.93548 | C | 0.06452 | 4 | 0.12903 |

| Site number | SNP name | Chromosome | Physical position | Physical distance from the previous SNP | Reference allele | Alternative allele | Major allele | Major allele frequency | Minor allele | Minor allele frequency | Number heterozygous | Heterozygosity |
|---|---|---|---|---|---|---|---|---|---|---|---|---|
| 11771 | S14_53056507 | 14 | 53056507 | 114975 | T | C | C | 0.70968 | T | 0.29032 | 12 | 0.3871 |
| 11772 | S14_53082945 | 14 | 53082945 | 26438 | T | C | T | 0.58065 | C | 0.41935 | 14 | 0.45161 |
| 11773 | S14_53147284 | 14 | 53147284 | 64339 | G | A | G | 0.87097 | A | 0.12903 | 8 | 0.25806 |
| 11774 | S14_53341186 | 14 | 53341186 | 193902 | G | A | G | 0.83871 | A | 0.16129 | 10 | 0.32258 |
| 11775 | S14_53551817 | 14 | 53551817 | 210631 | C | T | C | 0.87097 | T | 0.12903 | 6 | 0.19355 |
| 11776 | S14_53881757 | 14 | 53881757 | 329940 | C | A | C | 0.74194 | A | 0.25806 | 12 | 0.3871 |
| 11777 | S14_53892127 | 14 | 53892127 | 10370 | A | G | G | 0.90323 | A | 0.09677 | 6 | 0.19355 |
| 11778 | S14_53892214 | 14 | 53892214 | 87 | G | T | G | 0.95161 | T | 0.04839 | 1 | 0.03226 |
| 11779 | S14_53954372 | 14 | 53954372 | 62158 | T | C | T | 0.83871 | C | 0.16129 | 10 | 0.32258 |
| 11780 | S14_53954441 | 14 | 53954441 | 69 | C | A | C | 0.91935 | A | 0.08065 | 5 | 0.16129 |
| 11781 | S14_53954498 | 14 | 53954498 | 57 | C | T | C | 0.8871 | T | 0.1129 | 7 | 0.22581 |
| 11782 | S14_54603821 | 14 | 54603821 | 649323 | A | G | A | 0.82258 | G | 0.17742 | 9 | 0.29032 |
| 11783 | S14_54850533 | 14 | 54850533 | 246712 | A | G | G | 0.64516 | A | 0.35484 | 12 | 0.3871 |
| 11784 | S14_54850660 | 14 | 54850660 | 127 | G | A | G | 0.93548 | A | 0.06452 | 4 | 0.12903 |
| 11785 | S14_54851913 | 14 | 54851913 | 1253 | T | C | C | 0.53226 | T | 0.46774 | 15 | 0.48387 |
| 11786 | S14_54851978 | 14 | 54851978 | 65 | G | A | G | 0.75806 | A | 0.24194 | 13 | 0.41935 |
| 11787 | S14_54852004 | 14 | 54852004 | 26 | C | T | T | 0.77419 | C | 0.22581 | 12 | 0.3871 |
| 11788 | S14_54852005 | 14 | 54852005 | 1 | C | T | C | 0.80645 | T | 0.19355 | 10 | 0.32258 |
| 11789 | S14_54852009 | 14 | 54852009 | 4 | C | T | T | 0.51613 | C | 0.48387 | 14 | 0.45161 |
| 11790 | S14_54852010 | 14 | 54852010 | 1 | G | A | G | 0.75806 | A | 0.24194 | 11 | 0.35484 |
| 11791 | S14_54936432 | 14 | 54936432 | 84422 | G | A | G | 0.8871 | A | 0.1129 | 5 | 0.16129 |
| 11792 | S14_54936445 | 14 | 54936445 | 13 | C | G | C | 0.95161 | G | 0.04839 | 3 | 0.09677 |
| 11793 | S14_55042639 | 14 | 55042639 | 106194 | C | T | C | 0.91935 | T | 0.08065 | 5 | 0.16129 |
| 11794 | S14_55042664 | 14 | 55042664 | 25 | T | C | T | 0.91935 | C | 0.08065 | 3 | 0.09677 |
| 11795 | S14_55042668 | 14 | 55042668 | 4 | C | T | C | 0.75806 | T | 0.24194 | 11 | 0.35484 |
| 11796 | S14_55042702 | 14 | 55042702 | 34 | G | A | G | 0.80645 | A | 0.19355 | 10 | 0.32258 |
| 11797 | S14_55042719 | 14 | 55042719 | 17 | T | C | C | 0.69355 | T | 0.30645 | 13 | 0.41935 |
| 11798 | S14_55042772 | 14 | 55042772 | 53 | G | A | G | 0.80645 | A | 0.19355 | 10 | 0.32258 |
| 11799 | S14_55216262 | 14 | 55216262 | 173490 | T | C | T | 0.80645 | C | 0.19355 | 10 | 0.32258 |
| 11800 | S14_55216359 | 14 | 55216359 | 97 | G | A | G | 0.93548 | A | 0.06452 | 4 | 0.12903 |
| 11801 | S14_55235313 | 14 | 55235313 | 18954 | G | A | G | 0.75806 | A | 0.24194 | 9 | 0.29032 |
| 11802 | S14_55262426 | 14 | 55262426 | 27113 | C | T | C | 0.8871 | T | 0.1129 | 7 | 0.22581 |
| 11803 | S14_55262445 | 14 | 55262445 | 19 | T | A | T | 0.95161 | A | 0.04839 | 3 | 0.09677 |
| 11804 | S14_55598565 | 14 | 55598565 | 336120 | C | T | C | 0.82258 | T | 0.17742 | 7 | 0.22581 |
| 11805 | S14_55618306 | 14 | 55618306 | 19741 | G | A | G | 0.87097 | A | 0.12903 | 6 | 0.19355 |
| 11806 | S14_55883158 | 14 | 55883158 | 264852 | C | T | C | 0.83871 | T | 0.16129 | 8 | 0.25806 |
| 11807 | S14_55883275 | 14 | 55883275 | 117 | T | C | C | 0.59677 | T | 0.40323 | 15 | 0.48387 |
| 11808 | S14_55883306 | 14 | 55883306 | 31 | G | C | G | 0.83871 | C | 0.16129 | 8 | 0.25806 |
| 11809 | S14_56578099 | 14 | 56578099 | 694793 | A | G | A | 0.90323 | G | 0.09677 | 6 | 0.19355 |
| 11810 | S14_56683429 | 14 | 56683429 | 105330 | G | A | G | 0.95161 | A | 0.04839 | 3 | 0.09677 |
| 11811 | S14_56722148 | 14 | 56722148 | 38719 | C | T | T | 0.66129 | C | 0.33871 | 15 | 0.48387 |
| 11812 | S14_56722157 | 14 | 56722157 | 9 | G | A | G | 0.74194 | A | 0.25806 | 12 | 0.3871 |
| 11813 | S14_56970828 | 14 | 56970828 | 248671 | G | A | G | 0.95161 | A | 0.04839 | 3 | 0.09677 |
| 11814 | S14_57504001 | 14 | 57504001 | 533173 | A | G | A | 0.83871 | G | 0.16129 | 8 | 0.25806 |
| 11815 | S14_57611128 | 14 | 57611128 | 107127 | C | T | C | 0.8871 | T | 0.1129 | 5 | 0.16129 |
| 11816 | S14_57611263 | 14 | 57611263 | 135 | G | A | A | 0.83871 | G | 0.16129 | 8 | 0.25806 |
| 11817 | S14_57707796 | 14 | 57707796 | 96533 | C | T | C | 0.90323 | T | 0.09677 | 4 | 0.12903 |
| 11818 | S14_57710187 | 14 | 57710187 | 2391 | C | T | C | 0.91935 | T | 0.08065 | 3 | 0.09677 |
| 11819 | S14_57710234 | 14 | 57710234 | 47 | G | A | G | 0.93548 | A | 0.06452 | 4 | 0.12903 |
| 11820 | S14_57784968 | 14 | 57784968 | 74734 | G | A | G | 0.93548 | A | 0.06452 | 4 | 0.12903 |
| 11821 | S14_57786106 | 14 | 57786106 | 1138 | G | C | G | 0.90323 | C | 0.09677 | 6 | 0.19355 |
| 11822 | S14_57786163 | 14 | 57786163 | 57 | A | G | A | 0.93548 | G | 0.06452 | 4 | 0.12903 |
| 11823 | S14_57786167 | 14 | 57786167 | 4 | A | G | A | 0.93548 | G | 0.06452 | 4 | 0.12903 |
| 11824 | S14_57786173 | 14 | 57786173 | 6 | A | G | A | 0.93548 | G | 0.06452 | 4 | 0.12903 |

| Site number | SNP name | Chromosome | Physical position | Physical distance from the previous SNP | Reference allele | Alternative allele | Major allele | Major allele frequency | Minor allele | Minor allele frequency | Number heterozygous | Heterozygosity |
|---|---|---|---|---|---|---|---|---|---|---|---|---|
| 11825 | S14_57786201 | 14 | 57786201 | 28 | G | A | G | 0.80645 | A | 0.19355 | 6 | 0.19355 |
| 11826 | S14_58130154 | 14 | 58130154 | 343953 | G | A | G | 0.95161 | A | 0.04839 | 3 | 0.09677 |
| 11827 | S14_58419391 | 14 | 58419391 | 289237 | G | A | G | 0.6129 | A | 0.3871 | 14 | 0.45161 |
| 11828 | S14_58419408 | 14 | 58419408 | 17 | T | C | C | 0.59677 | T | 0.40323 | 11 | 0.35484 |
| 11829 | S14_58440793 | 14 | 58440793 | 21385 | G | A | G | 0.80645 | A | 0.19355 | 10 | 0.32258 |
| 11830 | S14_58440819 | 14 | 58440819 | 26 | C | T | C | 0.90323 | T | 0.09677 | 6 | 0.19355 |
| 11831 | S14_58440820 | 14 | 58440820 | 1 | G | A | G | 0.80645 | A | 0.19355 | 10 | 0.32258 |
| 11832 | S14_58440983 | 14 | 58440983 | 163 | C | T | C | 0.87097 | T | 0.12903 | 6 | 0.19355 |
| 11833 | S14_58492319 | 14 | 58492319 | 51336 | A | C | A | 0.79032 | C | 0.20968 | 13 | 0.41935 |
| 11834 | S14_58492418 | 14 | 58492418 | 99 | T | C | T | 0.91935 | C | 0.08065 | 5 | 0.16129 |
| 11835 | S14_58492427 | 14 | 58492427 | 9 | A | G | A | 0.53226 | G | 0.46774 | 17 | 0.54839 |
| 11836 | S14_58943218 | 14 | 58943218 | 450791 | C | T | C | 0.90323 | T | 0.09677 | 4 | 0.12903 |
| 11837 | S14_59045231 | 14 | 59045231 | 102013 | T | G | T | 0.87097 | G | 0.12903 | 8 | 0.25806 |
| 11838 | S14_59197036 | 14 | 59197036 | 151805 | G | A | G | 0.74194 | A | 0.25806 | 12 | 0.3871 |
| 11839 | S14_59197462 | 14 | 59197462 | 426 | G | A | A | 0.74194 | G | 0.25806 | 10 | 0.32258 |
| 11840 | S14_59257845 | 14 | 59257845 | 60383 | A | G | A | 0.93548 | G | 0.06452 | 2 | 0.06452 |
| 11841 | S14_59257969 | 14 | 59257969 | 124 | G | C | C | 0.79032 | G | 0.20968 | 13 | 0.41935 |
| 11842 | S14_59828465 | 14 | 59828465 | 570496 | T | G | G | 0.83871 | T | 0.16129 | 8 | 0.25806 |
| 11843 | S14_59878658 | 14 | 59878658 | 50193 | G | A | G | 0.93548 | A | 0.06452 | 4 | 0.12903 |
| 11844 | S14_59969431 | 14 | 59969431 | 90773 | T | C | C | 0.53226 | T | 0.46774 | 15 | 0.48387 |
| 11845 | S14_60285966 | 14 | 60285966 | 316535 | T | A | T | 0.8871 | A | 0.1129 | 7 | 0.22581 |
| 11846 | S14_60355848 | 14 | 60355848 | 69882 | C | T | C | 0.74194 | T | 0.25806 | 10 | 0.32258 |
| 11847 | S14_60694785 | 14 | 60694785 | 338937 | C | T | C | 0.95161 | T | 0.04839 | 3 | 0.09677 |
| 11848 | S14_60850711 | 14 | 60850711 | 155926 | G | T | T | 0.58065 | G | 0.41935 | 10 | 0.32258 |
| 11849 | S14_60850821 | 14 | 60850821 | 110 | T | C | T | 0.77419 | C | 0.22581 | 12 | 0.3871 |
| 11850 | S14_60850921 | 14 | 60850921 | 100 | C | T | C | 0.80645 | T | 0.19355 | 10 | 0.32258 |
| 11851 | S14_60906189 | 14 | 60906189 | 55268 | G | A | G | 0.90323 | A | 0.09677 | 6 | 0.19355 |
| 11852 | S14_60906348 | 14 | 60906348 | 159 | G | A | G | 0.90323 | A | 0.09677 | 6 | 0.19355 |
| 11853 | S14_61023001 | 14 | 61023001 | 116653 | T | G | T | 0.83871 | G | 0.16129 | 8 | 0.25806 |
| 11854 | S14_61131660 | 14 | 61131660 | 108659 | T | C | T | 0.91935 | C | 0.08065 | 5 | 0.16129 |
| 11855 | S14_61131725 | 14 | 61131725 | 65 | G | A | A | 0.90323 | G | 0.09677 | 6 | 0.19355 |
| 11856 | S14_61131753 | 14 | 61131753 | 28 | C | T | T | 0.90323 | C | 0.09677 | 6 | 0.19355 |
| 11857 | S14_61131754 | 14 | 61131754 | 1 | T | G | G | 0.93548 | T | 0.06452 | 4 | 0.12903 |
| 11858 | S14_61201296 | 14 | 61201296 | 69542 | C | G | C | 0.80645 | G | 0.19355 | 10 | 0.32258 |
| 11859 | S14_61201298 | 14 | 61201298 | 2 | G | C | G | 0.58065 | C | 0.41935 | 12 | 0.3871 |
| 11860 | S14_61201395 | 14 | 61201395 | 97 | G | A | A | 0.87097 | G | 0.12903 | 8 | 0.25806 |
| 11861 | S14_61201439 | 14 | 61201439 | 44 | C | T | C | 0.91935 | T | 0.08065 | 5 | 0.16129 |
| 11862 | S14_61233382 | 14 | 61233382 | 31943 | T | C | T | 0.79032 | C | 0.20968 | 9 | 0.29032 |
| 11863 | S14_61233404 | 14 | 61233404 | 22 | A | G | A | 0.8871 | G | 0.1129 | 5 | 0.16129 |
| 11864 | S14_61233469 | 14 | 61233469 | 65 | A | T | A | 0.91935 | T | 0.08065 | 5 | 0.16129 |
| 11865 | S14_61233481 | 14 | 61233481 | 12 | C | T | C | 0.79032 | T | 0.20968 | 9 | 0.29032 |
| 11866 | S14_61233515 | 14 | 61233515 | 34 | C | T | C | 0.95161 | T | 0.04839 | 3 | 0.09677 |
| 11867 | S14_61505337 | 14 | 61505337 | 271822 | A | G | G | 0.53226 | A | 0.46774 | 21 | 0.67742 |
| 11868 | S14_61505339 | 14 | 61505339 | 2 | G | T | T | 0.53226 | G | 0.46774 | 21 | 0.67742 |
| 11869 | S14_61870491 | 14 | 61870491 | 365152 | G | C | G | 0.66129 | C | 0.33871 | 17 | 0.54839 |
| 11870 | S14_61870526 | 14 | 61870526 | 35 | T | C | T | 0.54839 | C | 0.45161 | 14 | 0.45161 |
| 11871 | S14_61870595 | 14 | 61870595 | 69 | T | A | T | 0.93548 | A | 0.06452 | 2 | 0.06452 |
| 11872 | S14_61870611 | 14 | 61870611 | 16 | A | T | A | 0.8871 | T | 0.1129 | 7 | 0.22581 |
| 11873 | S14_62066116 | 14 | 62066116 | 195505 | C | A | C | 0.53226 | A | 0.46774 | 11 | 0.35484 |
| 11874 | S14_62066135 | 14 | 62066135 | 19 | G | C | G | 0.53226 | C | 0.46774 | 11 | 0.35484 |
| 11875 | S14_62066226 | 14 | 62066226 | 91 | A | T | A | 0.53226 | T | 0.46774 | 11 | 0.35484 |
| 11876 | S14_62066299 | 14 | 62066299 | 73 | A | G | A | 0.53226 | G | 0.46774 | 11 | 0.35484 |
| 11877 | S14_62066346 | 14 | 62066346 | 47 | C | T | C | 0.53226 | T | 0.46774 | 11 | 0.35484 |
| 11878 | S14_62448827 | 14 | 62448827 | 382481 | G | A | G | 0.95161 | A | 0.04839 | 3 | 0.09677 |

| Site number | SNP name | Chromosome | Physical position | Physical distance from the previous SNP | Reference allele | Alternative allele | Major allele | Major allele frequency | Minor allele | Minor allele frequency | Number heterozygous | Heterozygosity |
|---|---|---|---|---|---|---|---|---|---|---|---|---|
| 11879 | S14_62752509 | 14 | 62752509 | 303682 | T | A | A | 0.69355 | T | 0.30645 | 11 | 0.35484 |
| 11880 | S14_62752513 | 14 | 62752513 | 4 | C | T | C | 0.91935 | T | 0.08065 | 5 | 0.16129 |
| 11881 | S14_62931460 | 14 | 62931460 | 178947 | A | G | A | 0.77419 | G | 0.22581 | 12 | 0.3871 |
| 11882 | S14_62931555 | 14 | 62931555 | 95 | T | C | T | 0.77419 | C | 0.22581 | 12 | 0.3871 |
| 11883 | S14_63034603 | 14 | 63034603 | 103048 | T | C | T | 0.79032 | C | 0.20968 | 11 | 0.35484 |
| 11884 | S14_63034606 | 14 | 63034606 | 3 | G | A | G | 0.79032 | A | 0.20968 | 11 | 0.35484 |
| 11885 | S14_63289782 | 14 | 63289782 | 255176 | C | T | C | 0.91935 | T | 0.08065 | 5 | 0.16129 |
| 11886 | S14_63289809 | 14 | 63289809 | 27 | A | G | A | 0.67742 | G | 0.32258 | 12 | 0.3871 |
| 11887 | S14_63289824 | 14 | 63289824 | 15 | T | C | T | 0.67742 | C | 0.32258 | 12 | 0.3871 |
| 11888 | S14_63289829 | 14 | 63289829 | 5 | A | G | A | 0.67742 | G | 0.32258 | 12 | 0.3871 |
| 11889 | S14_63616571 | 14 | 63616571 | 326742 | G | C | G | 0.62903 | C | 0.37097 | 15 | 0.48387 |
| 11890 | S14_64002241 | 14 | 64002241 | 385670 | C | T | C | 0.85484 | T | 0.14516 | 7 | 0.22581 |
| 11891 | S14_64143977 | 14 | 64143977 | 141736 | A | G | G | 0.53226 | A | 0.46774 | 15 | 0.48387 |
| 11892 | S14_64144147 | 14 | 64144147 | 170 | C | G | C | 0.91935 | G | 0.08065 | 5 | 0.16129 |
| 11893 | S14_64541271 | 14 | 64541271 | 397124 | C | A | A | 0.56452 | C | 0.43548 | 17 | 0.54839 |
| 11894 | S14_64597711 | 14 | 64597711 | 56440 | A | G | A | 0.77419 | G | 0.22581 | 12 | 0.3871 |
| 11895 | S14_64597834 | 14 | 64597834 | 123 | T | C | C | 0.90323 | T | 0.09677 | 6 | 0.19355 |
| 11896 | S14_64597874 | 14 | 64597874 | 40 | C | T | C | 0.8871 | T | 0.1129 | 7 | 0.22581 |
| 11897 | S14_64885098 | 14 | 64885098 | 287224 | G | A | G | 0.80645 | A | 0.19355 | 8 | 0.25806 |
| 11898 | S14_65178712 | 14 | 65178712 | 293614 | C | T | C | 0.51613 | T | 0.48387 | 12 | 0.3871 |
| 11899 | S14_65181558 | 14 | 65181558 | 2846 | A | T | A | 0.90323 | T | 0.09677 | 4 | 0.12903 |
| 11900 | S14_65291930 | 14 | 65291930 | 110372 | A | C | A | 0.51613 | C | 0.48387 | 16 | 0.51613 |
| 11901 | S14_65292066 | 14 | 65292066 | 136 | A | G | G | 0.8871 | A | 0.1129 | 7 | 0.22581 |
| 11902 | S14_65292141 | 14 | 65292141 | 75 | C | T | T | 0.79032 | C | 0.20968 | 13 | 0.41935 |
| 11903 | S14_65348921 | 14 | 65348921 | 56780 | C | T | C | 0.72581 | T | 0.27419 | 13 | 0.41935 |
| 11904 | S14_65348942 | 14 | 65348942 | 21 | G | T | G | 0.72581 | T | 0.27419 | 13 | 0.41935 |
| 11905 | S14_65348970 | 14 | 65348970 | 28 | G | A | G | 0.72581 | A | 0.27419 | 13 | 0.41935 |
| 11906 | S14_65349037 | 14 | 65349037 | 67 | A | G | A | 0.75806 | G | 0.24194 | 11 | 0.35484 |
| 11907 | S14_65349096 | 14 | 65349096 | 59 | G | A | A | 0.67742 | G | 0.32258 | 16 | 0.51613 |
| 11908 | S14_65388362 | 14 | 65388362 | 39266 | A | G | G | 0.58065 | A | 0.41935 | 18 | 0.58065 |
| 11909 | S14_65388502 | 14 | 65388502 | 140 | A | G | G | 0.58065 | A | 0.41935 | 18 | 0.58065 |
| 11910 | S14_65388532 | 14 | 65388532 | 30 | G | A | A | 0.58065 | G | 0.41935 | 18 | 0.58065 |
| 11911 | S14_65573315 | 14 | 65573315 | 184783 | G | A | G | 0.93548 | A | 0.06452 | 4 | 0.12903 |
| 11912 | S14_65602383 | 14 | 65602383 | 29068 | T | G | T | 0.91935 | G | 0.08065 | 5 | 0.16129 |
| 11913 | S14_65623474 | 14 | 65623474 | 21091 | G | A | G | 0.79032 | A | 0.20968 | 9 | 0.29032 |
| 11914 | S14_65659815 | 14 | 65659815 | 36341 | T | G | T | 0.95161 | G | 0.04839 | 3 | 0.09677 |
| 11915 | S14_65660628 | 14 | 65660628 | 813 | G | T | T | 0.72581 | G | 0.27419 | 13 | 0.41935 |
| 11916 | S14_65762053 | 14 | 65762053 | 101425 | T | C | C | 0.91935 | T | 0.08065 | 5 | 0.16129 |
| 11917 | S14_65962882 | 14 | 65962882 | 200829 | C | T | C | 0.74194 | T | 0.25806 | 12 | 0.3871 |
| 11918 | S14_65962921 | 14 | 65962921 | 39 | C | G | C | 0.74194 | G | 0.25806 | 12 | 0.3871 |
| 11919 | S14_65973569 | 14 | 65973569 | 10648 | C | A | C | 0.90323 | A | 0.09677 | 4 | 0.12903 |
| 11920 | S14_65973594 | 14 | 65973594 | 25 | C | G | C | 0.90323 | G | 0.09677 | 4 | 0.12903 |
| 11921 | S14_66084362 | 14 | 66084362 | 110768 | A | G | G | 0.87097 | A | 0.12903 | 8 | 0.25806 |
| 11922 | S14_66084439 | 14 | 66084439 | 77 | C | T | C | 0.80645 | T | 0.19355 | 12 | 0.3871 |
| 11923 | S14_66179049 | 14 | 66179049 | 94610 | T | C | T | 0.74194 | C | 0.25806 | 14 | 0.45161 |
| 11924 | S14_66179132 | 14 | 66179132 | 83 | G | T | G | 0.75806 | T | 0.24194 | 13 | 0.41935 |
| 11925 | S14_66179235 | 14 | 66179235 | 103 | C | T | C | 0.74194 | T | 0.25806 | 14 | 0.45161 |
| 11926 | S14_66179248 | 14 | 66179248 | 13 | C | T | C | 0.74194 | T | 0.25806 | 14 | 0.45161 |
| 11927 | S14_66532037 | 14 | 66532037 | 352789 | G | A | G | 0.90323 | A | 0.09677 | 6 | 0.19355 |
| 11928 | S14_66772831 | 14 | 66772831 | 240794 | C | T | C | 0.93548 | T | 0.06452 | 4 | 0.12903 |
| 11929 | S14_66772987 | 14 | 66772987 | 156 | T | G | T | 0.91935 | G | 0.08065 | 5 | 0.16129 |
| 11930 | S14_66807469 | 14 | 66807469 | 34482 | T | C | T | 0.6129 | C | 0.3871 | 12 | 0.3871 |
| 11931 | S14_67545276 | 14 | 67545276 | 737807 | A | C | A | 0.77419 | C | 0.22581 | 10 | 0.32258 |
| 11932 | S14_67545286 | 14 | 67545286 | 10 | A | G | A | 0.93548 | G | 0.06452 | 4 | 0.12903 |

| Site number | SNP name | Chromosome | Physical position | Physical distance from the previous SNP | Reference allele | Alternative allele | Major allele | Major allele frequency | Minor allele | Minor allele frequency | Number heterozygous | Heterozygosity |
|---|---|---|---|---|---|---|---|---|---|---|---|---|
| 11933 | S14_67891061 | 14 | 67891061 | 345775 | T | G | G | 0.95161 | T | 0.04839 | 3 | 0.09677 |
| 11934 | S14_68005781 | 14 | 68005781 | 114720 | T | C | T | 0.93548 | C | 0.06452 | 4 | 0.12903 |
| 11935 | S14_68911002 | 14 | 68911002 | 905221 | C | G | C | 0.95161 | G | 0.04839 | 3 | 0.09677 |
| 11936 | S14_68911165 | 14 | 68911165 | 163 | C | T | C | 0.95161 | T | 0.04839 | 3 | 0.09677 |
| 11937 | S14_69109452 | 14 | 69109452 | 198287 | A | G | A | 0.93548 | G | 0.06452 | 4 | 0.12903 |
| 11938 | S14_69109467 | 14 | 69109467 | 15 | C | T | C | 0.74194 | T | 0.25806 | 12 | 0.3871 |
| 11939 | S14_69109570 | 14 | 69109570 | 103 | T | C | T | 0.58065 | C | 0.41935 | 16 | 0.51613 |
| 11940 | S14_69325222 | 14 | 69325222 | 215652 | G | C | G | 0.75806 | C | 0.24194 | 15 | 0.48387 |
| 11941 | S14_69325238 | 14 | 69325238 | 16 | T | G | T | 0.69355 | G | 0.30645 | 13 | 0.41935 |
| 11942 | S14_69715011 | 14 | 69715011 | 389773 | G | A | G | 0.90323 | A | 0.09677 | 6 | 0.19355 |
| 11943 | S14_69787559 | 14 | 69787559 | 72548 | A | G | G | 0.58065 | A | 0.41935 | 14 | 0.45161 |
| 11944 | S14_69787562 | 14 | 69787562 | 3 | T | A | T | 0.58065 | A | 0.41935 | 14 | 0.45161 |
| 11945 | S14_69787589 | 14 | 69787589 | 27 | G | A | G | 0.58065 | A | 0.41935 | 14 | 0.45161 |
| 11946 | S14_69787758 | 14 | 69787758 | 169 | G | T | G | 0.93548 | T | 0.06452 | 2 | 0.06452 |
| 11947 | S14_69787783 | 14 | 69787783 | 25 | G | C | G | 0.80645 | C | 0.19355 | 10 | 0.32258 |
| 11948 | S14_70676835 | 14 | 70676835 | 889052 | G | A | G | 0.95161 | A | 0.04839 | 3 | 0.09677 |
| 11949 | S14_70959368 | 14 | 70959368 | 282533 | G | A | G | 0.74194 | A | 0.25806 | 12 | 0.3871 |
| 11950 | S14_70988496 | 14 | 70988496 | 29128 | G | A | G | 0.64516 | A | 0.35484 | 16 | 0.51613 |
| 11951 | S14_71123184 | 14 | 71123184 | 134688 | G | A | G | 0.83871 | A | 0.16129 | 10 | 0.32258 |
| 11952 | S14_71123373 | 14 | 71123373 | 189 | G | T | G | 0.72581 | T | 0.27419 | 17 | 0.54839 |
| 11953 | S14_71415754 | 14 | 71415754 | 292381 | C | T | C | 0.6129 | T | 0.3871 | 14 | 0.45161 |
| 11954 | S14_71415785 | 14 | 71415785 | 31 | T | C | T | 0.62903 | C | 0.37097 | 13 | 0.41935 |
| 11955 | S14_71570009 | 14 | 71570009 | 154224 | A | G | A | 0.93548 | G | 0.06452 | 4 | 0.12903 |
| 11956 | S14_71570163 | 14 | 71570163 | 154 | A | C | A | 0.91935 | C | 0.08065 | 5 | 0.16129 |
| 11957 | S14_71809304 | 14 | 71809304 | 239141 | G | A | G | 0.64516 | A | 0.35484 | 16 | 0.51613 |
| 11958 | S14_71860868 | 14 | 71860868 | 51564 | G | A | G | 0.93548 | A | 0.06452 | 2 | 0.06452 |
| 11959 | S14_72087528 | 14 | 72087528 | 226660 | T | C | C | 0.77419 | T | 0.22581 | 10 | 0.32258 |
| 11960 | S14_72151770 | 14 | 72151770 | 64242 | C | T | C | 0.95161 | T | 0.04839 | 3 | 0.09677 |
| 11961 | S14_72285675 | 14 | 72285675 | 133905 | T | G | T | 0.95161 | G | 0.04839 | 3 | 0.09677 |
| 11962 | S14_72285735 | 14 | 72285735 | 60 | T | G | T | 0.72581 | G | 0.27419 | 13 | 0.41935 |
| 11963 | S14_72285805 | 14 | 72285805 | 70 | T | C | T | 0.54839 | C | 0.45161 | 18 | 0.58065 |
| 11964 | S14_72356545 | 14 | 72356545 | 70740 | G | A | G | 0.87097 | A | 0.12903 | 8 | 0.25806 |
| 11965 | S14_72356639 | 14 | 72356639 | 94 | G | A | G | 0.90323 | A | 0.09677 | 6 | 0.19355 |
| 11966 | S14_72356767 | 14 | 72356767 | 128 | A | G | A | 0.93548 | G | 0.06452 | 4 | 0.12903 |
| 11967 | S14_72933512 | 14 | 72933512 | 576745 | C | T | C | 0.95161 | T | 0.04839 | 3 | 0.09677 |
| 11968 | S14_72964957 | 14 | 72964957 | 31445 | T | C | T | 0.83871 | C | 0.16129 | 8 | 0.25806 |
| 11969 | S14_72964964 | 14 | 72964964 | 7 | C | A | C | 0.85484 | A | 0.14516 | 7 | 0.22581 |
| 11970 | S14_73099241 | 14 | 73099241 | 134277 | T | C | T | 0.51613 | C | 0.48387 | 16 | 0.51613 |
| 11971 | S14_73099332 | 14 | 73099332 | 91 | A | G | A | 0.51613 | G | 0.48387 | 16 | 0.51613 |
| 11972 | S14_73099342 | 14 | 73099342 | 10 | A | C | A | 0.51613 | C | 0.48387 | 16 | 0.51613 |
| 11973 | S14_73099361 | 14 | 73099361 | 19 | G | A | G | 0.95161 | A | 0.04839 | 3 | 0.09677 |
| 11974 | S14_73099362 | 14 | 73099362 | 1 | A | G | A | 0.53226 | G | 0.46774 | 17 | 0.54839 |
| 11975 | S14_73099378 | 14 | 73099378 | 16 | G | A | G | 0.82258 | A | 0.17742 | 9 | 0.29032 |
| 11976 | S14_73099409 | 14 | 73099409 | 31 | G | A | G | 0.82258 | A | 0.17742 | 9 | 0.29032 |
| 11977 | S14_73106569 | 14 | 73106569 | 7160 | C | G | C | 0.8871 | G | 0.1129 | 5 | 0.16129 |
| 11978 | S14_73106631 | 14 | 73106631 | 62 | C | T | C | 0.8871 | T | 0.1129 | 5 | 0.16129 |
| 11979 | S14_73106656 | 14 | 73106656 | 25 | C | T | C | 0.8871 | T | 0.1129 | 5 | 0.16129 |
| 11980 | S14_73106671 | 14 | 73106671 | 15 | C | T | C | 0.8871 | T | 0.1129 | 5 | 0.16129 |
| 11981 | S14_73106737 | 14 | 73106737 | 66 | T | G | T | 0.8871 | G | 0.1129 | 5 | 0.16129 |
| 11982 | S14_73106742 | 14 | 73106742 | 5 | T | G | T | 0.8871 | G | 0.1129 | 5 | 0.16129 |
| 11983 | S14_73106791 | 14 | 73106791 | 49 | C | T | C | 0.8871 | T | 0.1129 | 5 | 0.16129 |
| 11984 | S14_73117838 | 14 | 73117838 | 11047 | A | G | A | 0.59677 | G | 0.40323 | 15 | 0.48387 |
| 11985 | S14_73117841 | 14 | 73117841 | 3 | G | A | A | 0.5 | G | 0.5 | 17 | 0.54839 |
| 11986 | S14_73122608 | 14 | 73122608 | 4767 | A | G | A | 0.5 | G | 0.5 | 19 | 0.6129 |

| Site number | SNP name | Chromosome | Physical position | Physical distance from the previous SNP | Reference allele | Alternative allele | Major allele | Major allele frequency | Minor allele | Minor allele frequency | Number heterozygous | Heterozygosity |
|---|---|---|---|---|---|---|---|---|---|---|---|---|
| 11987 | S14_73122619 | 14 | 73122619 | 11 | G | A | G | 0.95161 | A | 0.04839 | 3 | 0.09677 |
| 11988 | S14_73620639 | 14 | 73620639 | 498020 | T | C | C | 0.70968 | T | 0.29032 | 12 | 0.3871 |
| 11989 | S14_73620696 | 14 | 73620696 | 57 | C | A | C | 0.90323 | A | 0.09677 | 6 | 0.19355 |
| 11990 | S14_73620708 | 14 | 73620708 | 12 | T | C | T | 0.90323 | C | 0.09677 | 6 | 0.19355 |
| 11991 | S14_73995481 | 14 | 73995481 | 374773 | G | A | G | 0.82258 | A | 0.17742 | 9 | 0.29032 |
| 11992 | S14_74125273 | 14 | 74125273 | 129792 | G | A | G | 0.77419 | A | 0.22581 | 10 | 0.32258 |
| 11993 | S14_74125277 | 14 | 74125277 | 4 | C | T | C | 0.75806 | T | 0.24194 | 11 | 0.35484 |
| 11994 | S14_74125380 | 14 | 74125380 | 103 | G | A | G | 0.87097 | A | 0.12903 | 8 | 0.25806 |
| 11995 | S14_74263973 | 14 | 74263973 | 138593 | T | C | C | 0.69355 | T | 0.30645 | 11 | 0.35484 |
| 11996 | S14_74263974 | 14 | 74263974 | 1 | G | A | G | 0.82258 | A | 0.17742 | 7 | 0.22581 |
| 11997 | S14_74264099 | 14 | 74264099 | 125 | A | G | A | 0.82258 | G | 0.17742 | 7 | 0.22581 |
| 11998 | S14_74617282 | 14 | 74617282 | 353183 | T | G | T | 0.72581 | G | 0.27419 | 11 | 0.35484 |
| 11999 | S14_74617339 | 14 | 74617339 | 57 | G | A | G | 0.85484 | A | 0.14516 | 7 | 0.22581 |
| 12000 | S14_74674647 | 14 | 74674647 | 57308 | C | T | C | 0.93548 | T | 0.06452 | 4 | 0.12903 |
| 12001 | S14_74674663 | 14 | 74674663 | 16 | C | A | C | 0.95161 | A | 0.04839 | 3 | 0.09677 |
| 12002 | S14_74674681 | 14 | 74674681 | 18 | A | G | A | 0.93548 | G | 0.06452 | 2 | 0.06452 |
| 12003 | S14_74674686 | 14 | 74674686 | 5 | A | G | A | 0.93548 | G | 0.06452 | 4 | 0.12903 |
| 12004 | S14_74694541 | 14 | 74694541 | 19855 | C | A | C | 0.80645 | A | 0.19355 | 10 | 0.32258 |
| 12005 | S14_74694583 | 14 | 74694583 | 42 | C | T | C | 0.95161 | T | 0.04839 | 3 | 0.09677 |
| 12006 | S14_74736796 | 14 | 74736796 | 42213 | T | C | T | 0.90323 | C | 0.09677 | 4 | 0.12903 |
| 12007 | S14_74894044 | 14 | 74894044 | 157248 | G | A | G | 0.91935 | A | 0.08065 | 5 | 0.16129 |
| 12008 | S14_75092776 | 14 | 75092776 | 198732 | G | A | G | 0.83871 | A | 0.16129 | 8 | 0.25806 |
| 12009 | S14_75229405 | 14 | 75229405 | 136629 | T | C | T | 0.90323 | C | 0.09677 | 6 | 0.19355 |
| 12010 | S14_75229497 | 14 | 75229497 | 92 | G | A | G | 0.93548 | A | 0.06452 | 4 | 0.12903 |
| 12011 | S14_75455834 | 14 | 75455834 | 226337 | G | T | T | 0.82258 | G | 0.17742 | 9 | 0.29032 |
| 12012 | S14_75681460 | 14 | 75681460 | 225626 | C | T | C | 0.91935 | T | 0.08065 | 3 | 0.09677 |
| 12013 | S14_75681465 | 14 | 75681465 | 5 | C | T | C | 0.82258 | T | 0.17742 | 11 | 0.35484 |
| 12014 | S14_75681466 | 14 | 75681466 | 1 | G | A | G | 0.95161 | A | 0.04839 | 3 | 0.09677 |
| 12015 | S14_75681535 | 14 | 75681535 | 69 | T | G | T | 0.91935 | G | 0.08065 | 5 | 0.16129 |
| 12016 | S14_75704840 | 14 | 75704840 | 23305 | G | C | G | 0.91935 | C | 0.08065 | 5 | 0.16129 |
| 12017 | S14_75704925 | 14 | 75704925 | 85 | T | C | T | 0.91935 | C | 0.08065 | 5 | 0.16129 |
| 12018 | S14_75791483 | 14 | 75791483 | 86558 | G | A | G | 0.83871 | A | 0.16129 | 6 | 0.19355 |
| 12019 | S14_75791543 | 14 | 75791543 | 60 | C | T | C | 0.8871 | T | 0.1129 | 7 | 0.22581 |
| 12020 | S14_76145355 | 14 | 76145355 | 353812 | C | T | T | 0.83871 | C | 0.16129 | 10 | 0.32258 |
| 12021 | S14_76310069 | 14 | 76310069 | 164714 | G | A | G | 0.75806 | A | 0.24194 | 13 | 0.41935 |
| 12022 | S14_76310097 | 14 | 76310097 | 28 | T | C | T | 0.56452 | C | 0.43548 | 17 | 0.54839 |
| 12023 | S14_76641521 | 14 | 76641521 | 331424 | G | A | G | 0.91935 | A | 0.08065 | 5 | 0.16129 |
| 12024 | S14_76641538 | 14 | 76641538 | 17 | A | T | A | 0.91935 | T | 0.08065 | 5 | 0.16129 |
| 12025 | S14_76641545 | 14 | 76641545 | 7 | A | G | A | 0.80645 | G | 0.19355 | 10 | 0.32258 |
| 12026 | S14_76641558 | 14 | 76641558 | 13 | G | A | G | 0.54839 | A | 0.45161 | 14 | 0.45161 |
| 12027 | S14_76641581 | 14 | 76641581 | 23 | T | C | T | 0.54839 | C | 0.45161 | 14 | 0.45161 |
| 12028 | S14_76954046 | 14 | 76954046 | 312465 | G | A | G | 0.91935 | A | 0.08065 | 5 | 0.16129 |
| 12029 | S14_76960386 | 14 | 76960386 | 6340 | C | T | T | 0.53226 | C | 0.46774 | 11 | 0.35484 |
| 12030 | S14_76960398 | 14 | 76960398 | 12 | T | C | C | 0.90323 | T | 0.09677 | 6 | 0.19355 |
| 12031 | S14_76960575 | 14 | 76960575 | 177 | A | G | A | 0.91935 | G | 0.08065 | 5 | 0.16129 |
| 12032 | S14_77104252 | 14 | 77104252 | 143677 | T | C | T | 0.58065 | C | 0.41935 | 16 | 0.51613 |
| 12033 | S14_77104433 | 14 | 77104433 | 181 | G | T | G | 0.80645 | T | 0.19355 | 10 | 0.32258 |
| 12034 | S14_77182043 | 14 | 77182043 | 77610 | A | G | A | 0.5 | G | 0.5 | 17 | 0.54839 |
| 12035 | S14_77182239 | 14 | 77182239 | 196 | G | C | G | 0.87097 | C | 0.12903 | 8 | 0.25806 |
| 12036 | S14_77360921 | 14 | 77360921 | 178682 | C | G | C | 0.95161 | G | 0.04839 | 3 | 0.09677 |
| 12037 | S14_77360935 | 14 | 77360935 | 14 | C | T | C | 0.95161 | T | 0.04839 | 3 | 0.09677 |
| 12038 | S14_77361077 | 14 | 77361077 | 142 | G | C | G | 0.91935 | C | 0.08065 | 5 | 0.16129 |
| 12039 | S14_77511156 | 14 | 77511156 | 150079 | T | G | G | 0.83871 | T | 0.16129 | 6 | 0.19355 |
| 12040 | S14_77511180 | 14 | 77511180 | 24 | C | A | A | 0.8871 | C | 0.1129 | 5 | 0.16129 |

| Site number | SNP name | Chromosome | Physical position | Physical distance from the previous SNP | Reference allele | Alternative allele | Major allele | Major allele frequency | Minor allele | Minor allele frequency | Number heterozygous | Heterozygosity |
|---|---|---|---|---|---|---|---|---|---|---|---|---|
| 12041 | S14_77511268 | 14 | 77511268 | 88 | C | G | G | 0.8871 | C | 0.1129 | 5 | 0.16129 |
| 12042 | S14_78188205 | 14 | 78188205 | 676937 | T | A | T | 0.93548 | A | 0.06452 | 4 | 0.12903 |
| 12043 | S14_78188252 | 14 | 78188252 | 47 | G | A | G | 0.91935 | A | 0.08065 | 5 | 0.16129 |
| 12044 | S14_78459100 | 14 | 78459100 | 270848 | G | A | A | 0.6129 | G | 0.3871 | 14 | 0.45161 |
| 12045 | S14_78575255 | 14 | 78575255 | 116155 | G | A | G | 0.85484 | A | 0.14516 | 7 | 0.22581 |
| 12046 | S14_78575312 | 14 | 78575312 | 57 | C | G | G | 0.66129 | C | 0.33871 | 15 | 0.48387 |
| 12047 | S14_78824139 | 14 | 78824139 | 248827 | C | T | C | 0.66129 | T | 0.33871 | 15 | 0.48387 |
| 12048 | S14_78824169 | 14 | 78824169 | 30 | G | A | G | 0.79032 | A | 0.20968 | 13 | 0.41935 |
| 12049 | S14_78824339 | 14 | 78824339 | 170 | A | G | G | 0.90323 | A | 0.09677 | 4 | 0.12903 |
| 12050 | S14_78908520 | 14 | 78908520 | 84181 | T | C | T | 0.91935 | C | 0.08065 | 5 | 0.16129 |
| 12051 | S14_79029993 | 14 | 79029993 | 121473 | G | A | G | 0.80645 | A | 0.19355 | 12 | 0.3871 |
| 12052 | S14_79112435 | 14 | 79112435 | 82442 | G | A | G | 0.90323 | A | 0.09677 | 6 | 0.19355 |
| 12053 | S14_79112439 | 14 | 79112439 | 4 | T | G | T | 0.91935 | G | 0.08065 | 5 | 0.16129 |
| 12054 | S14_79373161 | 14 | 79373161 | 260722 | A | C | A | 0.90323 | C | 0.09677 | 6 | 0.19355 |
| 12055 | S14_79731445 | 14 | 79731445 | 358284 | A | G | A | 0.87097 | G | 0.12903 | 6 | 0.19355 |
| 12056 | S14_79731511 | 14 | 79731511 | 66 | G | A | G | 0.87097 | A | 0.12903 | 8 | 0.25806 |
| 12057 | S14_79731521 | 14 | 79731521 | 10 | A | G | A | 0.87097 | G | 0.12903 | 8 | 0.25806 |
| 12058 | S14_79833318 | 14 | 79833318 | 101797 | C | T | C | 0.82258 | T | 0.17742 | 9 | 0.29032 |
| 12059 | S14_79833384 | 14 | 79833384 | 66 | G | T | G | 0.93548 | T | 0.06452 | 2 | 0.06452 |
| 12060 | S14_80057791 | 14 | 80057791 | 224407 | C | T | C | 0.90323 | T | 0.09677 | 6 | 0.19355 |
| 12061 | S14_80057792 | 14 | 80057792 | 1 | A | G | G | 0.87097 | A | 0.12903 | 8 | 0.25806 |
| 12062 | S14_80620573 | 14 | 80620573 | 562781 | G | A | G | 0.87097 | A | 0.12903 | 6 | 0.19355 |
| 12063 | S14_80620652 | 14 | 80620652 | 79 | A | G | G | 0.75806 | A | 0.24194 | 15 | 0.48387 |
| 12064 | S14_80620689 | 14 | 80620689 | 37 | G | A | G | 0.93548 | A | 0.06452 | 4 | 0.12903 |
| 12065 | S14_80761814 | 14 | 80761814 | 141125 | G | A | G | 0.85484 | A | 0.14516 | 7 | 0.22581 |
| 12066 | S14_80761889 | 14 | 80761889 | 75 | G | A | G | 0.82258 | A | 0.17742 | 11 | 0.35484 |
| 12067 | S14_80761952 | 14 | 80761952 | 63 | T | C | C | 0.93548 | T | 0.06452 | 4 | 0.12903 |
| 12068 | S14_81125712 | 14 | 81125712 | 363760 | G | T | G | 0.87097 | T | 0.12903 | 8 | 0.25806 |
| 12069 | S14_81369424 | 14 | 81369424 | 243712 | A | G | A | 0.82258 | G | 0.17742 | 11 | 0.35484 |
| 12070 | S14_82131375 | 14 | 82131375 | 761951 | A | G | A | 0.79032 | G | 0.20968 | 9 | 0.29032 |
| 12071 | S14_82230205 | 14 | 82230205 | 98830 | T | C | C | 0.79032 | T | 0.20968 | 13 | 0.41935 |
| 12072 | S14_82452592 | 14 | 82452592 | 222387 | C | T | C | 0.82258 | T | 0.17742 | 11 | 0.35484 |
| 12073 | S14_82452615 | 14 | 82452615 | 23 | C | T | C | 0.82258 | T | 0.17742 | 11 | 0.35484 |
| 12074 | S14_82452682 | 14 | 82452682 | 67 | A | G | A | 0.80645 | G | 0.19355 | 10 | 0.32258 |
| 12075 | S14_82452711 | 14 | 82452711 | 29 | T | C | T | 0.75806 | C | 0.24194 | 13 | 0.41935 |
| 12076 | S14_83790307 | 14 | 83790307 | 1337596 | T | C | T | 0.95161 | C | 0.04839 | 3 | 0.09677 |
| 12077 | S14_83790341 | 14 | 83790341 | 34 | C | T | C | 0.85484 | T | 0.14516 | 7 | 0.22581 |
| 12078 | S14_83790423 | 14 | 83790423 | 82 | G | C | G | 0.85484 | C | 0.14516 | 7 | 0.22581 |
| 12079 | S14_83790431 | 14 | 83790431 | 8 | T | C | T | 0.85484 | C | 0.14516 | 7 | 0.22581 |
| 12080 | S14_83790439 | 14 | 83790439 | 8 | A | G | G | 0.85484 | A | 0.14516 | 7 | 0.22581 |
| 12081 | S14_83790446 | 14 | 83790446 | 7 | C | G | C | 0.83871 | G | 0.16129 | 6 | 0.19355 |
| 12082 | S14_84581680 | 14 | 84581680 | 791234 | C | A | C | 0.90323 | A | 0.09677 | 4 | 0.12903 |
| 12083 | S14_84581804 | 14 | 84581804 | 124 | C | G | C | 0.64516 | G | 0.35484 | 14 | 0.45161 |
| 12084 | S14_84660032 | 14 | 84660032 | 78228 | C | T | C | 0.95161 | T | 0.04839 | 3 | 0.09677 |
| 12085 | S14_84660071 | 14 | 84660071 | 39 | C | G | C | 0.95161 | G | 0.04839 | 3 | 0.09677 |
| 12086 | S14_84660075 | 14 | 84660075 | 4 | A | C | A | 0.95161 | C | 0.04839 | 3 | 0.09677 |
| 12087 | S14_84660165 | 14 | 84660165 | 90 | G | A | G | 0.95161 | A | 0.04839 | 3 | 0.09677 |
| 12088 | S14_84660196 | 14 | 84660196 | 31 | A | G | A | 0.93548 | G | 0.06452 | 4 | 0.12903 |
| 12089 | S14_84739294 | 14 | 84739294 | 79098 | A | C | A | 0.58065 | C | 0.41935 | 18 | 0.58065 |
| 12090 | S14_85480991 | 14 | 85480991 | 741697 | C | T | T | 0.72581 | C | 0.27419 | 11 | 0.35484 |
| 12091 | S14_85481063 | 14 | 85481063 | 72 | C | T | C | 0.91935 | T | 0.08065 | 5 | 0.16129 |
| 12092 | S14_85481118 | 14 | 85481118 | 55 | G | A | G | 0.82258 | A | 0.17742 | 9 | 0.29032 |
| 12093 | S14_85481119 | 14 | 85481119 | 1 | C | A | C | 0.85484 | A | 0.14516 | 5 | 0.16129 |
| 12094 | S14_85498158 | 14 | 85498158 | 17039 | G | A | G | 0.95161 | A | 0.04839 | 3 | 0.09677 |

| Site number | SNP name | Chromosome | Physical position | Physical distance from the previous SNP | Reference allele | Alternative allele | Major allele | Major allele frequency | Minor allele | Minor allele frequency | Number heterozygous | Heterozygosity |
|---|---|---|---|---|---|---|---|---|---|---|---|---|
| 12095 | S14_87059647 | 14 | 87059647 | 1561489 | C | T | C | 0.6129 | T | 0.3871 | 16 | 0.51613 |
| 12096 | S14_88202308 | 14 | 88202308 | 1142661 | G | A | G | 0.70968 | A | 0.29032 | 16 | 0.51613 |
| 12097 | S14_88202477 | 14 | 88202477 | 169 | G | A | G | 0.74194 | A | 0.25806 | 10 | 0.32258 |
| 12098 | S14_88202509 | 14 | 88202509 | 32 | A | G | A | 0.95161 | G | 0.04839 | 1 | 0.03226 |
| 12099 | S14_88366119 | 14 | 88366119 | 163610 | C | T | C | 0.8871 | T | 0.1129 | 7 | 0.22581 |
| 12100 | S14_88366179 | 14 | 88366179 | 60 | G | T | G | 0.72581 | T | 0.27419 | 13 | 0.41935 |
| 12101 | S14_88366194 | 14 | 88366194 | 15 | A | G | A | 0.72581 | G | 0.27419 | 13 | 0.41935 |
| 12102 | S14_88366288 | 14 | 88366288 | 94 | A | G | A | 0.72581 | G | 0.27419 | 13 | 0.41935 |
| 12103 | S14_88366294 | 14 | 88366294 | 6 | T | C | T | 0.72581 | C | 0.27419 | 13 | 0.41935 |
| 12104 | S14_89961945 | 14 | 89961945 | 1595651 | A | T | A | 0.77419 | T | 0.22581 | 10 | 0.32258 |
| 12105 | S14_89961956 | 14 | 89961956 | 11 | G | A | G | 0.82258 | A | 0.17742 | 9 | 0.29032 |
| 12106 | S14_89961964 | 14 | 89961964 | 8 | C | T | C | 0.77419 | T | 0.22581 | 10 | 0.32258 |
| 12107 | S14_89961965 | 14 | 89961965 | 1 | A | G | A | 0.6129 | G | 0.3871 | 10 | 0.32258 |
| 12108 | S14_89962038 | 14 | 89962038 | 73 | C | T | C | 0.93548 | T | 0.06452 | 4 | 0.12903 |
| 12109 | S14_89962125 | 14 | 89962125 | 87 | T | C | C | 0.93548 | T | 0.06452 | 2 | 0.06452 |
| 12110 | S14_89962127 | 14 | 89962127 | 2 | A | G | G | 0.70968 | A | 0.29032 | 8 | 0.25806 |
| 12111 | S14_90178671 | 14 | 90178671 | 216544 | C | T | C | 0.90323 | T | 0.09677 | 6 | 0.19355 |
| 12112 | S14_90924820 | 14 | 90924820 | 746149 | A | G | A | 0.75806 | G | 0.24194 | 9 | 0.29032 |
| 12113 | S14_90924821 | 14 | 90924821 | 1 | A | C | A | 0.75806 | C | 0.24194 | 9 | 0.29032 |
| 12114 | S14_90924853 | 14 | 90924853 | 32 | A | G | A | 0.74194 | G | 0.25806 | 10 | 0.32258 |
| 12115 | S14_90924871 | 14 | 90924871 | 18 | C | T | C | 0.75806 | T | 0.24194 | 9 | 0.29032 |
| 12116 | S14_91207155 | 14 | 91207155 | 282284 | G | A | G | 0.83871 | A | 0.16129 | 8 | 0.25806 |
| 12117 | S14_91207187 | 14 | 91207187 | 32 | C | T | C | 0.85484 | T | 0.14516 | 7 | 0.22581 |
| 12118 | S14_91207346 | 14 | 91207346 | 159 | A | G | A | 0.83871 | G | 0.16129 | 8 | 0.25806 |
| 12119 | S14_91886697 | 14 | 91886697 | 679351 | A | G | A | 0.95161 | G | 0.04839 | 3 | 0.09677 |
| 12120 | S14_91886757 | 14 | 91886757 | 60 | T | C | T | 0.79032 | C | 0.20968 | 11 | 0.35484 |
| 12121 | S14_91886778 | 14 | 91886778 | 21 | C | T | C | 0.59677 | T | 0.40323 | 19 | 0.6129 |
| 12122 | S14_91886804 | 14 | 91886804 | 26 | C | T | T | 0.72581 | C | 0.27419 | 15 | 0.48387 |
| 12123 | S14_91886820 | 14 | 91886820 | 16 | G | A | G | 0.56452 | A | 0.43548 | 15 | 0.48387 |
| 12124 | S14_91886823 | 14 | 91886823 | 3 | T | C | T | 0.75806 | C | 0.24194 | 13 | 0.41935 |
| 12125 | S14_91886915 | 14 | 91886915 | 92 | A | G | G | 0.93548 | A | 0.06452 | 4 | 0.12903 |
| 12126 | S14_91886923 | 14 | 91886923 | 8 | T | C | T | 0.91935 | C | 0.08065 | 5 | 0.16129 |
| 12127 | S14_91886929 | 14 | 91886929 | 6 | A | T | T | 0.51613 | A | 0.48387 | 18 | 0.58065 |
| 12128 | S14_91960694 | 14 | 91960694 | 73765 | T | C | T | 0.79032 | C | 0.20968 | 11 | 0.35484 |
| 12129 | S14_92041501 | 14 | 92041501 | 80807 | T | C | T | 0.53226 | C | 0.46774 | 17 | 0.54839 |
| 12130 | S14_92041629 | 14 | 92041629 | 128 | A | G | A | 0.95161 | G | 0.04839 | 3 | 0.09677 |
| 12131 | S14_92041630 | 14 | 92041630 | 1 | T | C | T | 0.95161 | C | 0.04839 | 3 | 0.09677 |
| 12132 | S14_92041645 | 14 | 92041645 | 15 | C | A | C | 0.95161 | A | 0.04839 | 3 | 0.09677 |
| 12133 | S14_92041657 | 14 | 92041657 | 12 | T | C | T | 0.95161 | C | 0.04839 | 3 | 0.09677 |
| 12134 | S14_92041661 | 14 | 92041661 | 4 | G | A | G | 0.83871 | A | 0.16129 | 10 | 0.32258 |
| 12135 | S14_92377165 | 14 | 92377165 | 335504 | C | T | C | 0.8871 | T | 0.1129 | 7 | 0.22581 |
| 12136 | S14_92377273 | 14 | 92377273 | 108 | G | A | G | 0.80645 | A | 0.19355 | 8 | 0.25806 |
| 12137 | S14_92377360 | 14 | 92377360 | 87 | C | T | C | 0.8871 | T | 0.1129 | 7 | 0.22581 |
| 12138 | S14_92489685 | 14 | 92489685 | 112325 | C | A | C | 0.93548 | A | 0.06452 | 4 | 0.12903 |
| 12139 | S14_93356122 | 14 | 93356122 | 866437 | A | G | A | 0.69355 | G | 0.30645 | 13 | 0.41935 |
| 12140 | S14_93356138 | 14 | 93356138 | 16 | T | C | T | 0.75806 | C | 0.24194 | 15 | 0.48387 |
| 12141 | S14_93356153 | 14 | 93356153 | 15 | C | G | G | 0.95161 | C | 0.04839 | 3 | 0.09677 |
| 12142 | S14_93377465 | 14 | 93377465 | 21312 | T | C | T | 0.90323 | C | 0.09677 | 4 | 0.12903 |
| 12143 | S14_93747166 | 14 | 93747166 | 369701 | G | T | G | 0.95161 | T | 0.04839 | 3 | 0.09677 |
| 12144 | S14_93941182 | 14 | 93941182 | 194016 | T | C | C | 0.67742 | T | 0.32258 | 14 | 0.45161 |
| 12145 | S14_93941183 | 14 | 93941183 | 1 | G | A | G | 0.90323 | A | 0.09677 | 6 | 0.19355 |
| 12146 | S14_93941277 | 14 | 93941277 | 94 | C | G | C | 0.85484 | G | 0.14516 | 9 | 0.29032 |
| 12147 | S14_93941340 | 14 | 93941340 | 63 | G | T | G | 0.90323 | T | 0.09677 | 6 | 0.19355 |
| 12148 | S14_94269460 | 14 | 94269460 | 328120 | G | A | G | 0.69355 | A | 0.30645 | 13 | 0.41935 |

| Site number | SNP name | Chromosome | Physical position | Physical distance from the previous SNP | Reference allele | Alternative allele | Major allele | Major allele frequency | Minor allele | Minor allele frequency | Number heterozygous | Heterozygosity |
|---|---|---|---|---|---|---|---|---|---|---|---|---|
| 12149 | S15_51518 | 15 | 51518 | 0 | T | G | T | 0.62903 | G | 0.37097 | 13 | 0.41935 |
| 12150 | S15_51521 | 15 | 51521 | 3 | G | A | G | 0.62903 | A | 0.37097 | 13 | 0.41935 |
| 12151 | S15_77517 | 15 | 77517 | 25996 | C | G | C | 0.90323 | G | 0.09677 | 4 | 0.12903 |
| 12152 | S15_77530 | 15 | 77530 | 13 | C | A | A | 0.87097 | C | 0.12903 | 8 | 0.25806 |
| 12153 | S15_190481 | 15 | 190481 | 112951 | A | C | A | 0.5 | C | 0.5 | 15 | 0.48387 |
| 12154 | S15_190488 | 15 | 190488 | 7 | A | G | G | 0.79032 | A | 0.20968 | 11 | 0.35484 |
| 12155 | S15_190573 | 15 | 190573 | 85 | C | T | C | 0.87097 | T | 0.12903 | 6 | 0.19355 |
| 12156 | S15_314760 | 15 | 314760 | 124187 | A | G | G | 0.91935 | A | 0.08065 | 5 | 0.16129 |
| 12157 | S15_314954 | 15 | 314954 | 194 | C | T | C | 0.80645 | T | 0.19355 | 8 | 0.25806 |
| 12158 | S15_445942 | 15 | 445942 | 130988 | A | T | A | 0.95161 | T | 0.04839 | 1 | 0.03226 |
| 12159 | S15_446085 | 15 | 446085 | 143 | G | A | G | 0.95161 | A | 0.04839 | 1 | 0.03226 |
| 12160 | S15_448580 | 15 | 448580 | 2495 | G | A | G | 0.8871 | A | 0.1129 | 7 | 0.22581 |
| 12161 | S15_448615 | 15 | 448615 | 35 | G | A | G | 0.95161 | A | 0.04839 | 3 | 0.09677 |
| 12162 | S15_1275932 | 15 | 1275932 | 827317 | A | G | G | 0.93548 | A | 0.06452 | 2 | 0.06452 |
| 12163 | S15_1275994 | 15 | 1275994 | 62 | G | T | T | 0.6129 | G | 0.3871 | 16 | 0.51613 |
| 12164 | S15_1388178 | 15 | 1388178 | 112184 | C | T | C | 0.8871 | T | 0.1129 | 7 | 0.22581 |
| 12165 | S15_1399059 | 15 | 1399059 | 10881 | T | C | C | 0.70968 | T | 0.29032 | 14 | 0.45161 |
| 12166 | S15_1506298 | 15 | 1506298 | 107239 | A | G | G | 0.56452 | A | 0.43548 | 17 | 0.54839 |
| 12167 | S15_2025549 | 15 | 2025549 | 519251 | C | A | C | 0.74194 | A | 0.25806 | 14 | 0.45161 |
| 12168 | S15_2025636 | 15 | 2025636 | 87 | C | T | C | 0.93548 | T | 0.06452 | 2 | 0.06452 |
| 12169 | S15_2025728 | 15 | 2025728 | 92 | T | C | T | 0.95161 | C | 0.04839 | 1 | 0.03226 |
| 12170 | S15_2042071 | 15 | 2042071 | 16343 | C | T | T | 0.80645 | C | 0.19355 | 12 | 0.3871 |
| 12171 | S15_2042088 | 15 | 2042088 | 17 | T | C | C | 0.56452 | T | 0.43548 | 15 | 0.48387 |
| 12172 | S15_2042089 | 15 | 2042089 | 1 | A | G | G | 0.56452 | A | 0.43548 | 15 | 0.48387 |
| 12173 | S15_2042112 | 15 | 2042112 | 23 | A | C | C | 0.56452 | A | 0.43548 | 15 | 0.48387 |
| 12174 | S15_2042131 | 15 | 2042131 | 19 | G | C | G | 0.67742 | C | 0.32258 | 12 | 0.3871 |
| 12175 | S15_2042171 | 15 | 2042171 | 40 | T | C | C | 0.56452 | T | 0.43548 | 15 | 0.48387 |
| 12176 | S15_2042194 | 15 | 2042194 | 23 | A | T | A | 0.67742 | T | 0.32258 | 12 | 0.3871 |
| 12177 | S15_2043223 | 15 | 2043223 | 1029 | G | A | G | 0.90323 | A | 0.09677 | 4 | 0.12903 |
| 12178 | S15_2043256 | 15 | 2043256 | 33 | G | A | G | 0.87097 | A | 0.12903 | 6 | 0.19355 |
| 12179 | S15_2043345 | 15 | 2043345 | 89 | C | T | T | 0.56452 | C | 0.43548 | 15 | 0.48387 |
| 12180 | S15_2043371 | 15 | 2043371 | 26 | C | T | C | 0.75806 | T | 0.24194 | 9 | 0.29032 |
| 12181 | S15_2043400 | 15 | 2043400 | 29 | G | A | G | 0.75806 | A | 0.24194 | 9 | 0.29032 |
| 12182 | S15_2043403 | 15 | 2043403 | 3 | G | T | T | 0.56452 | G | 0.43548 | 15 | 0.48387 |
| 12183 | S15_2123840 | 15 | 2123840 | 80437 | A | G | A | 0.90323 | G | 0.09677 | 4 | 0.12903 |
| 12184 | S15_2123908 | 15 | 2123908 | 68 | C | T | C | 0.91935 | T | 0.08065 | 5 | 0.16129 |
| 12185 | S15_2123943 | 15 | 2123943 | 35 | G | T | G | 0.90323 | T | 0.09677 | 4 | 0.12903 |
| 12186 | S15_2150222 | 15 | 2150222 | 26279 | G | T | G | 0.72581 | T | 0.27419 | 11 | 0.35484 |
| 12187 | S15_2150229 | 15 | 2150229 | 7 | A | C | A | 0.91935 | C | 0.08065 | 5 | 0.16129 |
| 12188 | S15_2150322 | 15 | 2150322 | 93 | G | A | G | 0.8871 | A | 0.1129 | 7 | 0.22581 |
| 12189 | S15_2160314 | 15 | 2160314 | 9992 | A | G | G | 0.8871 | A | 0.1129 | 5 | 0.16129 |
| 12190 | S15_2219606 | 15 | 2219606 | 59292 | T | C | T | 0.85484 | C | 0.14516 | 5 | 0.16129 |
| 12191 | S15_2219634 | 15 | 2219634 | 28 | C | G | C | 0.95161 | G | 0.04839 | 3 | 0.09677 |
| 12192 | S15_2219707 | 15 | 2219707 | 73 | C | T | C | 0.91935 | T | 0.08065 | 3 | 0.09677 |
| 12193 | S15_2219735 | 15 | 2219735 | 28 | G | A | G | 0.83871 | A | 0.16129 | 8 | 0.25806 |
| 12194 | S15_2471241 | 15 | 2471241 | 251506 | C | T | C | 0.66129 | T | 0.33871 | 17 | 0.54839 |
| 12195 | S15_2471341 | 15 | 2471341 | 100 | T | C | T | 0.66129 | C | 0.33871 | 17 | 0.54839 |
| 12196 | S15_2471425 | 15 | 2471425 | 84 | C | T | C | 0.93548 | T | 0.06452 | 4 | 0.12903 |
| 12197 | S15_2805409 | 15 | 2805409 | 333984 | C | T | C | 0.75806 | T | 0.24194 | 9 | 0.29032 |
| 12198 | S15_2805502 | 15 | 2805502 | 93 | G | A | G | 0.82258 | A | 0.17742 | 9 | 0.29032 |
| 12199 | S15_3191808 | 15 | 3191808 | 386306 | A | T | A | 0.91935 | T | 0.08065 | 5 | 0.16129 |
| 12200 | S15_3191815 | 15 | 3191815 | 7 | G | C | G | 0.95161 | C | 0.04839 | 3 | 0.09677 |
| 12201 | S15_3191838 | 15 | 3191838 | 23 | T | C | T | 0.90323 | C | 0.09677 | 6 | 0.19355 |
| 12202 | S15_3191896 | 15 | 3191896 | 58 | G | A | G | 0.95161 | A | 0.04839 | 3 | 0.09677 |

| Site number | SNP name | Chromosome | Physical position | Physical distance from the previous SNP | Reference allele | Alternative allele | Major allele | Major allele frequency | Minor allele | Minor allele frequency | Number heterozygous | Heterozygosity |
|---|---|---|---|---|---|---|---|---|---|---|---|---|
| 12203 | S15_3191929 | 15 | 3191929 | 33 | A | G | A | 0.85484 | G | 0.14516 | 9 | 0.29032 |
| 12204 | S15_3191955 | 15 | 3191955 | 26 | C | T | C | 0.91935 | T | 0.08065 | 5 | 0.16129 |
| 12205 | S15_3191975 | 15 | 3191975 | 20 | G | C | G | 0.62903 | C | 0.37097 | 19 | 0.6129 |
| 12206 | S15_3754790 | 15 | 3754790 | 562815 | T | C | T | 0.79032 | C | 0.20968 | 11 | 0.35484 |
| 12207 | S15_3754839 | 15 | 3754839 | 49 | A | G | A | 0.56452 | G | 0.43548 | 19 | 0.6129 |
| 12208 | S15_3754840 | 15 | 3754840 | 1 | T | A | T | 0.56452 | A | 0.43548 | 19 | 0.6129 |
| 12209 | S15_3754922 | 15 | 3754922 | 82 | A | G | A | 0.93548 | G | 0.06452 | 4 | 0.12903 |
| 12210 | S15_3850677 | 15 | 3850677 | 95755 | T | C | T | 0.83871 | C | 0.16129 | 10 | 0.32258 |
| 12211 | S15_3966190 | 15 | 3966190 | 115513 | T | C | T | 0.93548 | C | 0.06452 | 4 | 0.12903 |
| 12212 | S15_3966285 | 15 | 3966285 | 95 | T | C | T | 0.75806 | C | 0.24194 | 15 | 0.48387 |
| 12213 | S15_3966291 | 15 | 3966291 | 6 | C | T | C | 0.91935 | T | 0.08065 | 5 | 0.16129 |
| 12214 | S15_3966353 | 15 | 3966353 | 62 | C | T | C | 0.75806 | T | 0.24194 | 15 | 0.48387 |
| 12215 | S15_3966371 | 15 | 3966371 | 18 | G | C | C | 0.72581 | G | 0.27419 | 17 | 0.54839 |
| 12216 | S15_3966388 | 15 | 3966388 | 17 | T | C | T | 0.75806 | C | 0.24194 | 15 | 0.48387 |
| 12217 | S15_4011428 | 15 | 4011428 | 45040 | T | C | T | 0.87097 | C | 0.12903 | 8 | 0.25806 |
| 12218 | S15_4011610 | 15 | 4011610 | 182 | A | G | A | 0.72581 | G | 0.27419 | 17 | 0.54839 |
| 12219 | S15_4011632 | 15 | 4011632 | 22 | A | G | A | 0.87097 | G | 0.12903 | 8 | 0.25806 |
| 12220 | S15_4179137 | 15 | 4179137 | 167505 | C | T | C | 0.79032 | T | 0.20968 | 11 | 0.35484 |
| 12221 | S15_4179254 | 15 | 4179254 | 117 | T | C | C | 0.93548 | T | 0.06452 | 4 | 0.12903 |
| 12222 | S15_4334048 | 15 | 4334048 | 154794 | A | C | A | 0.91935 | C | 0.08065 | 5 | 0.16129 |
| 12223 | S15_4334061 | 15 | 4334061 | 13 | C | G | C | 0.69355 | G | 0.30645 | 17 | 0.54839 |
| 12224 | S15_4777789 | 15 | 4777789 | 443728 | T | C | T | 0.93548 | C | 0.06452 | 4 | 0.12903 |
| 12225 | S15_4888289 | 15 | 4888289 | 110500 | A | G | A | 0.72581 | G | 0.27419 | 13 | 0.41935 |
| 12226 | S15_4888294 | 15 | 4888294 | 5 | A | G | G | 0.62903 | A | 0.37097 | 11 | 0.35484 |
| 12227 | S15_4888412 | 15 | 4888412 | 118 | T | C | T | 0.72581 | C | 0.27419 | 13 | 0.41935 |
| 12228 | S15_4888489 | 15 | 4888489 | 77 | T | C | T | 0.75806 | C | 0.24194 | 15 | 0.48387 |
| 12229 | S15_4911116 | 15 | 4911116 | 22627 | G | A | G | 0.8871 | A | 0.1129 | 7 | 0.22581 |
| 12230 | S15_4911122 | 15 | 4911122 | 6 | T | C | T | 0.56452 | C | 0.43548 | 19 | 0.6129 |
| 12231 | S15_4911258 | 15 | 4911258 | 136 | T | G | T | 0.83871 | G | 0.16129 | 8 | 0.25806 |
| 12232 | S15_4932823 | 15 | 4932823 | 21565 | C | T | T | 0.82258 | C | 0.17742 | 11 | 0.35484 |
| 12233 | S15_4932841 | 15 | 4932841 | 18 | T | C | T | 0.95161 | C | 0.04839 | 3 | 0.09677 |
| 12234 | S15_4932960 | 15 | 4932960 | 119 | A | G | G | 0.82258 | A | 0.17742 | 11 | 0.35484 |
| 12235 | S15_5010292 | 15 | 5010292 | 77332 | A | C | A | 0.93548 | C | 0.06452 | 2 | 0.06452 |
| 12236 | S15_5010359 | 15 | 5010359 | 67 | T | A | A | 0.62903 | T | 0.37097 | 15 | 0.48387 |
| 12237 | S15_5010407 | 15 | 5010407 | 48 | C | T | C | 0.79032 | T | 0.20968 | 11 | 0.35484 |
| 12238 | S15_5049644 | 15 | 5049644 | 39237 | C | T | C | 0.95161 | T | 0.04839 | 3 | 0.09677 |
| 12239 | S15_5049844 | 15 | 5049844 | 200 | G | A | G | 0.95161 | A | 0.04839 | 3 | 0.09677 |
| 12240 | S15_5049860 | 15 | 5049860 | 16 | C | T | C | 0.95161 | T | 0.04839 | 3 | 0.09677 |
| 12241 | S15_5049863 | 15 | 5049863 | 3 | A | G | A | 0.95161 | G | 0.04839 | 3 | 0.09677 |
| 12242 | S15_5160166 | 15 | 5160166 | 110303 | A | C | A | 0.72581 | C | 0.27419 | 13 | 0.41935 |
| 12243 | S15_5160227 | 15 | 5160227 | 61 | A | G | G | 0.70968 | A | 0.29032 | 10 | 0.32258 |
| 12244 | S15_5160244 | 15 | 5160244 | 17 | T | G | T | 0.85484 | G | 0.14516 | 9 | 0.29032 |
| 12245 | S15_5552970 | 15 | 5552970 | 392726 | C | T | C | 0.83871 | T | 0.16129 | 10 | 0.32258 |
| 12246 | S15_5553144 | 15 | 5553144 | 174 | C | G | C | 0.90323 | G | 0.09677 | 6 | 0.19355 |
| 12247 | S15_5562720 | 15 | 5562720 | 9576 | G | A | G | 0.95161 | A | 0.04839 | 3 | 0.09677 |
| 12248 | S15_5562933 | 15 | 5562933 | 213 | G | A | G | 0.8871 | A | 0.1129 | 7 | 0.22581 |
| 12249 | S15_5562945 | 15 | 5562945 | 12 | G | T | G | 0.80645 | T | 0.19355 | 12 | 0.3871 |
| 12250 | S15_5575265 | 15 | 5575265 | 12320 | A | G | A | 0.70968 | G | 0.29032 | 10 | 0.32258 |
| 12251 | S15_5824731 | 15 | 5824731 | 249466 | G | A | G | 0.91935 | A | 0.08065 | 3 | 0.09677 |
| 12252 | S15_6302470 | 15 | 6302470 | 477739 | C | T | T | 0.79032 | C | 0.20968 | 9 | 0.29032 |
| 12253 | S15_6598223 | 15 | 6598223 | 295753 | C | T | C | 0.80645 | T | 0.19355 | 8 | 0.25806 |
| 12254 | S15_6598264 | 15 | 6598264 | 41 | C | T | C | 0.80645 | T | 0.19355 | 8 | 0.25806 |
| 12255 | S15_6736739 | 15 | 6736739 | 138475 | T | C | T | 0.75806 | C | 0.24194 | 13 | 0.41935 |
| 12256 | S15_6769772 | 15 | 6769772 | 33033 | G | A | G | 0.80645 | A | 0.19355 | 10 | 0.32258 |

| Site number | SNP name | Chromosome | Physical position | Physical distance from the previous SNP | Reference allele | Alternative allele | Major allele | Major allele frequency | Minor allele | Minor allele frequency | Number heterozygous | Heterozygosity |
|---|---|---|---|---|---|---|---|---|---|---|---|---|
| 12257 | S15_7164881 | 15 | 7164881 | 395109 | T | A | T | 0.8871 | A | 0.1129 | 7 | 0.22581 |
| 12258 | S15_7193712 | 15 | 7193712 | 28831 | G | A | A | 0.8871 | G | 0.1129 | 7 | 0.22581 |
| 12259 | S15_7301131 | 15 | 7301131 | 107419 | T | C | C | 0.87097 | T | 0.12903 | 8 | 0.25806 |
| 12260 | S15_7611171 | 15 | 7611171 | 310040 | A | C | A | 0.79032 | C | 0.20968 | 7 | 0.22581 |
| 12261 | S15_7781179 | 15 | 7781179 | 170008 | C | T | T | 0.74194 | C | 0.25806 | 12 | 0.3871 |
| 12262 | S15_8199389 | 15 | 8199389 | 418210 | C | T | C | 0.91935 | T | 0.08065 | 5 | 0.16129 |
| 12263 | S15_8868267 | 15 | 8868267 | 668878 | A | T | A | 0.80645 | T | 0.19355 | 12 | 0.3871 |
| 12264 | S15_8868405 | 15 | 8868405 | 138 | C | T | C | 0.80645 | T | 0.19355 | 10 | 0.32258 |
| 12265 | S15_9029574 | 15 | 9029574 | 161169 | C | T | C | 0.69355 | T | 0.30645 | 11 | 0.35484 |
| 12266 | S15_9029673 | 15 | 9029673 | 99 | C | T | C | 0.90323 | T | 0.09677 | 6 | 0.19355 |
| 12267 | S15_9299596 | 15 | 9299596 | 269923 | C | T | C | 0.80645 | T | 0.19355 | 8 | 0.25806 |
| 12268 | S15_9554028 | 15 | 9554028 | 254432 | A | G | A | 0.64516 | G | 0.35484 | 10 | 0.32258 |
| 12269 | S15_9554100 | 15 | 9554100 | 72 | C | T | C | 0.62903 | T | 0.37097 | 11 | 0.35484 |
| 12270 | S15_9618115 | 15 | 9618115 | 64015 | C | T | C | 0.95161 | T | 0.04839 | 3 | 0.09677 |
| 12271 | S15_9618141 | 15 | 9618141 | 26 | G | C | G | 0.72581 | C | 0.27419 | 11 | 0.35484 |
| 12272 | S15_9618154 | 15 | 9618154 | 13 | C | T | C | 0.91935 | T | 0.08065 | 5 | 0.16129 |
| 12273 | S15_9618173 | 15 | 9618173 | 19 | C | A | C | 0.91935 | A | 0.08065 | 5 | 0.16129 |
| 12274 | S15_10066308 | 15 | 10066308 | 448135 | T | C | T | 0.72581 | C | 0.27419 | 13 | 0.41935 |
| 12275 | S15_10066309 | 15 | 10066309 | 1 | A | G | A | 0.72581 | G | 0.27419 | 13 | 0.41935 |
| 12276 | S15_10066314 | 15 | 10066314 | 5 | T | C | T | 0.72581 | C | 0.27419 | 13 | 0.41935 |
| 12277 | S15_10066318 | 15 | 10066318 | 4 | T | C | C | 0.64516 | T | 0.35484 | 12 | 0.3871 |
| 12278 | S15_10066333 | 15 | 10066333 | 15 | A | T | A | 0.93548 | T | 0.06452 | 4 | 0.12903 |
| 12279 | S15_10066422 | 15 | 10066422 | 89 | A | G | A | 0.72581 | G | 0.27419 | 13 | 0.41935 |
| 12280 | S15_10066427 | 15 | 10066427 | 5 | C | G | C | 0.72581 | G | 0.27419 | 13 | 0.41935 |
| 12281 | S15_10066482 | 15 | 10066482 | 55 | C | T | C | 0.72581 | T | 0.27419 | 13 | 0.41935 |
| 12282 | S15_10793348 | 15 | 10793348 | 726866 | G | A | A | 0.69355 | G | 0.30645 | 13 | 0.41935 |
| 12283 | S15_10877110 | 15 | 10877110 | 83762 | G | C | G | 0.85484 | C | 0.14516 | 7 | 0.22581 |
| 12284 | S15_10877243 | 15 | 10877243 | 133 | T | C | T | 0.93548 | C | 0.06452 | 4 | 0.12903 |
| 12285 | S15_11327567 | 15 | 11327567 | 450324 | A | C | A | 0.62903 | C | 0.37097 | 11 | 0.35484 |
| 12286 | S15_11327618 | 15 | 11327618 | 51 | G | A | G | 0.93548 | A | 0.06452 | 4 | 0.12903 |
| 12287 | S15_11327754 | 15 | 11327754 | 136 | G | A | G | 0.62903 | A | 0.37097 | 11 | 0.35484 |
| 12288 | S15_11471877 | 15 | 11471877 | 144123 | A | G | A | 0.8871 | G | 0.1129 | 7 | 0.22581 |
| 12289 | S15_11471907 | 15 | 11471907 | 30 | T | C | T | 0.8871 | C | 0.1129 | 7 | 0.22581 |
| 12290 | S15_11471957 | 15 | 11471957 | 50 | T | C | T | 0.91935 | C | 0.08065 | 5 | 0.16129 |
| 12291 | S15_11471989 | 15 | 11471989 | 32 | C | T | C | 0.93548 | T | 0.06452 | 4 | 0.12903 |
| 12292 | S15_11472000 | 15 | 11472000 | 11 | G | A | G | 0.91935 | A | 0.08065 | 5 | 0.16129 |
| 12293 | S15_11481530 | 15 | 11481530 | 9530 | G | C | G | 0.90323 | C | 0.09677 | 6 | 0.19355 |
| 12294 | S15_11537063 | 15 | 11537063 | 55533 | G | A | G | 0.69355 | A | 0.30645 | 9 | 0.29032 |
| 12295 | S15_11950671 | 15 | 11950671 | 413608 | T | G | T | 0.93548 | G | 0.06452 | 4 | 0.12903 |
| 12296 | S15_11950709 | 15 | 11950709 | 38 | A | T | A | 0.90323 | T | 0.09677 | 4 | 0.12903 |
| 12297 | S15_11989856 | 15 | 11989856 | 39147 | T | C | T | 0.54839 | C | 0.45161 | 14 | 0.45161 |
| 12298 | S15_11989857 | 15 | 11989857 | 1 | G | A | G | 0.95161 | A | 0.04839 | 3 | 0.09677 |
| 12299 | S15_11989921 | 15 | 11989921 | 64 | C | T | C | 0.95161 | T | 0.04839 | 3 | 0.09677 |
| 12300 | S15_11989928 | 15 | 11989928 | 7 | C | A | C | 0.93548 | A | 0.06452 | 4 | 0.12903 |
| 12301 | S15_11989996 | 15 | 11989996 | 68 | A | T | A | 0.93548 | T | 0.06452 | 4 | 0.12903 |
| 12302 | S15_12741112 | 15 | 12741112 | 751116 | A | T | A | 0.93548 | T | 0.06452 | 4 | 0.12903 |
| 12303 | S15_12741226 | 15 | 12741226 | 114 | A | G | A | 0.93548 | G | 0.06452 | 4 | 0.12903 |
| 12304 | S15_12741303 | 15 | 12741303 | 77 | G | T | G | 0.95161 | T | 0.04839 | 3 | 0.09677 |
| 12305 | S15_12974035 | 15 | 12974035 | 232732 | G | A | G | 0.62903 | A | 0.37097 | 13 | 0.41935 |
| 12306 | S15_12993537 | 15 | 12993537 | 19502 | G | C | G | 0.82258 | C | 0.17742 | 11 | 0.35484 |
| 12307 | S15_13226504 | 15 | 13226504 | 232967 | C | T | C | 0.6129 | T | 0.3871 | 12 | 0.3871 |
| 12308 | S15_13226509 | 15 | 13226509 | 5 | G | C | G | 0.66129 | C | 0.33871 | 9 | 0.29032 |
| 12309 | S15_13226621 | 15 | 13226621 | 112 | G | A | G | 0.62903 | A | 0.37097 | 11 | 0.35484 |
| 12310 | S15_13226641 | 15 | 13226641 | 20 | G | C | C | 0.90323 | G | 0.09677 | 4 | 0.12903 |

| Site number | SNP name | Chromosome | Physical position | Physical distance from the previous SNP | Reference allele | Alternative allele | Major allele | Major allele frequency | Minor allele | Minor allele frequency | Number heterozygous | Heterozygosity |
|---|---|---|---|---|---|---|---|---|---|---|---|---|
| 12311 | S15_13300297 | 15 | 13300297 | 73656 | A | G | A | 0.6129 | G | 0.3871 | 14 | 0.45161 |
| 12312 | S15_13300302 | 15 | 13300302 | 5 | T | G | T | 0.59677 | G | 0.40323 | 15 | 0.48387 |
| 12313 | S15_13300303 | 15 | 13300303 | 1 | T | C | T | 0.59677 | C | 0.40323 | 13 | 0.41935 |
| 12314 | S15_13679552 | 15 | 13679552 | 379249 | G | A | G | 0.85484 | A | 0.14516 | 5 | 0.16129 |
| 12315 | S15_13679610 | 15 | 13679610 | 58 | C | T | C | 0.85484 | T | 0.14516 | 5 | 0.16129 |
| 12316 | S15_13679615 | 15 | 13679615 | 5 | G | A | G | 0.85484 | A | 0.14516 | 5 | 0.16129 |
| 12317 | S15_13679625 | 15 | 13679625 | 10 | C | T | C | 0.85484 | T | 0.14516 | 5 | 0.16129 |
| 12318 | S15_13806827 | 15 | 13806827 | 127202 | G | C | G | 0.82258 | C | 0.17742 | 9 | 0.29032 |
| 12319 | S15_13806865 | 15 | 13806865 | 38 | A | G | A | 0.5 | G | 0.5 | 17 | 0.54839 |
| 12320 | S15_13806917 | 15 | 13806917 | 52 | G | A | G | 0.93548 | A | 0.06452 | 4 | 0.12903 |
| 12321 | S15_13842200 | 15 | 13842200 | 35283 | A | G | A | 0.56452 | G | 0.43548 | 15 | 0.48387 |
| 12322 | S15_13842246 | 15 | 13842246 | 46 | G | T | G | 0.56452 | T | 0.43548 | 15 | 0.48387 |
| 12323 | S15_13842309 | 15 | 13842309 | 63 | A | G | A | 0.56452 | G | 0.43548 | 15 | 0.48387 |
| 12324 | S15_13842343 | 15 | 13842343 | 34 | C | G | C | 0.56452 | G | 0.43548 | 15 | 0.48387 |
| 12325 | S15_14101960 | 15 | 14101960 | 259617 | G | A | G | 0.8871 | A | 0.1129 | 7 | 0.22581 |
| 12326 | S15_14102018 | 15 | 14102018 | 58 | T | C | T | 0.72581 | C | 0.27419 | 15 | 0.48387 |
| 12327 | S15_14346740 | 15 | 14346740 | 244722 | C | T | C | 0.95161 | T | 0.04839 | 3 | 0.09677 |
| 12328 | S15_14346741 | 15 | 14346741 | 1 | G | A | G | 0.8871 | A | 0.1129 | 5 | 0.16129 |
| 12329 | S15_14346787 | 15 | 14346787 | 46 | A | G | A | 0.95161 | G | 0.04839 | 3 | 0.09677 |
| 12330 | S15_14475573 | 15 | 14475573 | 128786 | G | A | G | 0.85484 | A | 0.14516 | 9 | 0.29032 |
| 12331 | S15_14475816 | 15 | 14475816 | 243 | A | G | G | 0.56452 | A | 0.43548 | 15 | 0.48387 |
| 12332 | S15_14545735 | 15 | 14545735 | 69919 | C | T | T | 0.90323 | C | 0.09677 | 6 | 0.19355 |
| 12333 | S15_15685225 | 15 | 15685225 | 1139490 | C | A | C | 0.91935 | A | 0.08065 | 5 | 0.16129 |
| 12334 | S15_15685266 | 15 | 15685266 | 41 | A | G | A | 0.93548 | G | 0.06452 | 4 | 0.12903 |
| 12335 | S15_15685314 | 15 | 15685314 | 48 | G | A | G | 0.91935 | A | 0.08065 | 5 | 0.16129 |
| 12336 | S15_15685316 | 15 | 15685316 | 2 | G | A | G | 0.91935 | A | 0.08065 | 5 | 0.16129 |
| 12337 | S15_15685339 | 15 | 15685339 | 23 | C | A | C | 0.93548 | A | 0.06452 | 4 | 0.12903 |
| 12338 | S15_15685374 | 15 | 15685374 | 35 | G | A | G | 0.58065 | A | 0.41935 | 14 | 0.45161 |
| 12339 | S15_15685433 | 15 | 15685433 | 59 | C | T | C | 0.93548 | T | 0.06452 | 4 | 0.12903 |
| 12340 | S15_15685435 | 15 | 15685435 | 2 | T | C | T | 0.91935 | C | 0.08065 | 5 | 0.16129 |
| 12341 | S15_15854325 | 15 | 15854325 | 168890 | C | T | C | 0.91935 | T | 0.08065 | 5 | 0.16129 |
| 12342 | S15_15854504 | 15 | 15854504 | 179 | T | C | T | 0.91935 | C | 0.08065 | 5 | 0.16129 |
| 12343 | S15_16044484 | 15 | 16044484 | 189980 | A | G | A | 0.83871 | G | 0.16129 | 8 | 0.25806 |
| 12344 | S15_16224351 | 15 | 16224351 | 179867 | C | T | C | 0.74194 | T | 0.25806 | 8 | 0.25806 |
| 12345 | S15_16224477 | 15 | 16224477 | 126 | C | T | C | 0.90323 | T | 0.09677 | 6 | 0.19355 |
| 12346 | S15_16336468 | 15 | 16336468 | 111991 | A | G | A | 0.74194 | G | 0.25806 | 8 | 0.25806 |
| 12347 | S15_16336498 | 15 | 16336498 | 30 | T | C | C | 0.91935 | T | 0.08065 | 5 | 0.16129 |
| 12348 | S15_16336539 | 15 | 16336539 | 41 | G | T | G | 0.91935 | T | 0.08065 | 3 | 0.09677 |
| 12349 | S15_16463225 | 15 | 16463225 | 126686 | A | C | C | 0.51613 | A | 0.48387 | 14 | 0.45161 |
| 12350 | S15_16463252 | 15 | 16463252 | 27 | G | A | G | 0.62903 | A | 0.37097 | 13 | 0.41935 |
| 12351 | S15_16463365 | 15 | 16463365 | 113 | G | A | G | 0.87097 | A | 0.12903 | 6 | 0.19355 |
| 12352 | S15_16467532 | 15 | 16467532 | 4167 | C | T | C | 0.93548 | T | 0.06452 | 4 | 0.12903 |
| 12353 | S15_16726013 | 15 | 16726013 | 258481 | C | T | C | 0.75806 | T | 0.24194 | 15 | 0.48387 |
| 12354 | S15_17030778 | 15 | 17030778 | 304765 | G | A | G | 0.56452 | A | 0.43548 | 11 | 0.35484 |
| 12355 | S15_17030818 | 15 | 17030818 | 40 | T | C | T | 0.56452 | C | 0.43548 | 11 | 0.35484 |
| 12356 | S15_17061950 | 15 | 17061950 | 31132 | T | C | T | 0.79032 | C | 0.20968 | 9 | 0.29032 |
| 12357 | S15_17130349 | 15 | 17130349 | 68399 | A | C | A | 0.93548 | C | 0.06452 | 4 | 0.12903 |
| 12358 | S15_17130373 | 15 | 17130373 | 24 | A | G | A | 0.62903 | G | 0.37097 | 19 | 0.6129 |
| 12359 | S15_17130381 | 15 | 17130381 | 8 | G | A | G | 0.93548 | A | 0.06452 | 4 | 0.12903 |
| 12360 | S15_17251994 | 15 | 17251994 | 121613 | G | A | A | 0.5 | G | 0.5 | 19 | 0.6129 |
| 12361 | S15_17252086 | 15 | 17252086 | 92 | A | C | A | 0.5 | C | 0.5 | 19 | 0.6129 |
| 12362 | S15_17252197 | 15 | 17252197 | 111 | G | A | A | 0.5 | G | 0.5 | 19 | 0.6129 |
| 12363 | S15_17298325 | 15 | 17298325 | 46128 | G | A | G | 0.75806 | A | 0.24194 | 13 | 0.41935 |
| 12364 | S15_17470427 | 15 | 17470427 | 172102 | A | C | C | 0.80645 | A | 0.19355 | 8 | 0.25806 |

| Site number | SNP name | Chromosome | Physical position | Physical distance from the previous SNP | Reference allele | Alternative allele | Major allele | Major allele frequency | Minor allele | Minor allele frequency | Number heterozygous | Heterozygosity |
|---|---|---|---|---|---|---|---|---|---|---|---|---|
| 12365 | S15_17470484 | 15 | 17470484 | 57 | C | G | C | 0.95161 | G | 0.04839 | 1 | 0.03226 |
| 12366 | S15_17499466 | 15 | 17499466 | 28982 | T | C | T | 0.8871 | C | 0.1129 | 7 | 0.22581 |
| 12367 | S15_17499517 | 15 | 17499517 | 51 | C | T | C | 0.8871 | T | 0.1129 | 7 | 0.22581 |
| 12368 | S15_17662683 | 15 | 17662683 | 163166 | T | C | T | 0.80645 | C | 0.19355 | 10 | 0.32258 |
| 12369 | S15_17875615 | 15 | 17875615 | 212932 | G | T | G | 0.51613 | T | 0.48387 | 14 | 0.45161 |
| 12370 | S15_17879846 | 15 | 17879846 | 4231 | C | T | C | 0.66129 | T | 0.33871 | 11 | 0.35484 |
| 12371 | S15_18574640 | 15 | 18574640 | 694794 | A | G | A | 0.70968 | G | 0.29032 | 12 | 0.3871 |
| 12372 | S15_18800086 | 15 | 18800086 | 225446 | G | A | G | 0.66129 | A | 0.33871 | 13 | 0.41935 |
| 12373 | S15_18827286 | 15 | 18827286 | 27200 | C | G | C | 0.64516 | G | 0.35484 | 14 | 0.45161 |
| 12374 | S15_18827309 | 15 | 18827309 | 23 | C | T | C | 0.85484 | T | 0.14516 | 5 | 0.16129 |
| 12375 | S15_19825030 | 15 | 19825030 | 997721 | T | C | T | 0.91935 | C | 0.08065 | 5 | 0.16129 |
| 12376 | S15_19825109 | 15 | 19825109 | 79 | T | A | T | 0.93548 | A | 0.06452 | 4 | 0.12903 |
| 12377 | S15_20113676 | 15 | 20113676 | 288567 | T | C | T | 0.75806 | C | 0.24194 | 13 | 0.41935 |
| 12378 | S15_20162446 | 15 | 20162446 | 48770 | G | T | T | 0.95161 | G | 0.04839 | 3 | 0.09677 |
| 12379 | S15_20162578 | 15 | 20162578 | 132 | A | G | G | 0.95161 | A | 0.04839 | 3 | 0.09677 |
| 12380 | S15_20162600 | 15 | 20162600 | 22 | T | C | T | 0.95161 | C | 0.04839 | 3 | 0.09677 |
| 12381 | S15_20553434 | 15 | 20553434 | 390834 | T | C | T | 0.8871 | C | 0.1129 | 5 | 0.16129 |
| 12382 | S15_20553475 | 15 | 20553475 | 41 | C | G | C | 0.90323 | G | 0.09677 | 6 | 0.19355 |
| 12383 | S15_20583766 | 15 | 20583766 | 30291 | G | A | G | 0.62903 | A | 0.37097 | 11 | 0.35484 |
| 12384 | S15_21132163 | 15 | 21132163 | 548397 | G | C | G | 0.95161 | C | 0.04839 | 3 | 0.09677 |
| 12385 | S15_21360015 | 15 | 21360015 | 227852 | G | A | A | 0.67742 | G | 0.32258 | 14 | 0.45161 |
| 12386 | S15_21360042 | 15 | 21360042 | 27 | A | G | G | 0.66129 | A | 0.33871 | 15 | 0.48387 |
| 12387 | S15_21629527 | 15 | 21629527 | 269485 | G | A | G | 0.75806 | A | 0.24194 | 11 | 0.35484 |
| 12388 | S15_21629554 | 15 | 21629554 | 27 | C | A | C | 0.72581 | A | 0.27419 | 11 | 0.35484 |
| 12389 | S15_21629692 | 15 | 21629692 | 138 | C | T | C | 0.85484 | T | 0.14516 | 5 | 0.16129 |
| 12390 | S15_21673584 | 15 | 21673584 | 43892 | T | C | C | 0.8871 | T | 0.1129 | 5 | 0.16129 |
| 12391 | S15_21673717 | 15 | 21673717 | 133 | G | T | G | 0.75806 | T | 0.24194 | 13 | 0.41935 |
| 12392 | S15_21677594 | 15 | 21677594 | 3877 | C | T | C | 0.93548 | T | 0.06452 | 4 | 0.12903 |
| 12393 | S15_21677680 | 15 | 21677680 | 86 | A | G | A | 0.91935 | G | 0.08065 | 5 | 0.16129 |
| 12394 | S15_22348091 | 15 | 22348091 | 670411 | T | C | T | 0.77419 | C | 0.22581 | 8 | 0.25806 |
| 12395 | S15_22348108 | 15 | 22348108 | 17 | A | C | A | 0.91935 | C | 0.08065 | 3 | 0.09677 |
| 12396 | S15_22348212 | 15 | 22348212 | 104 | T | C | T | 0.91935 | C | 0.08065 | 3 | 0.09677 |
| 12397 | S15_22348233 | 15 | 22348233 | 21 | G | A | A | 0.95161 | G | 0.04839 | 3 | 0.09677 |
| 12398 | S15_23080552 | 15 | 23080552 | 732319 | C | T | C | 0.93548 | T | 0.06452 | 2 | 0.06452 |
| 12399 | S15_23080600 | 15 | 23080600 | 48 | T | G | T | 0.85484 | G | 0.14516 | 9 | 0.29032 |
| 12400 | S15_23080601 | 15 | 23080601 | 1 | T | C | T | 0.93548 | C | 0.06452 | 2 | 0.06452 |
| 12401 | S15_23080644 | 15 | 23080644 | 43 | T | A | T | 0.83871 | A | 0.16129 | 8 | 0.25806 |
| 12402 | S15_23464133 | 15 | 23464133 | 383489 | C | T | C | 0.56452 | T | 0.43548 | 13 | 0.41935 |
| 12403 | S15_23464252 | 15 | 23464252 | 119 | C | T | C | 0.74194 | T | 0.25806 | 16 | 0.51613 |
| 12404 | S15_23464266 | 15 | 23464266 | 14 | C | T | T | 0.53226 | C | 0.46774 | 15 | 0.48387 |
| 12405 | S15_23509510 | 15 | 23509510 | 45244 | G | A | G | 0.77419 | A | 0.22581 | 10 | 0.32258 |
| 12406 | S15_23509634 | 15 | 23509634 | 124 | A | G | A | 0.77419 | G | 0.22581 | 12 | 0.3871 |
| 12407 | S15_23853704 | 15 | 23853704 | 344070 | T | C | T | 0.59677 | C | 0.40323 | 11 | 0.35484 |
| 12408 | S15_23853847 | 15 | 23853847 | 143 | C | T | C | 0.70968 | T | 0.29032 | 14 | 0.45161 |
| 12409 | S15_23853892 | 15 | 23853892 | 45 | G | A | A | 0.58065 | G | 0.41935 | 12 | 0.3871 |
| 12410 | S15_23900194 | 15 | 23900194 | 46302 | T | C | C | 0.53226 | T | 0.46774 | 15 | 0.48387 |
| 12411 | S15_23900246 | 15 | 23900246 | 52 | T | A | A | 0.53226 | T | 0.46774 | 15 | 0.48387 |
| 12412 | S15_23900280 | 15 | 23900280 | 34 | G | A | G | 0.74194 | A | 0.25806 | 14 | 0.45161 |
| 12413 | S15_23900323 | 15 | 23900323 | 43 | A | G | A | 0.74194 | G | 0.25806 | 14 | 0.45161 |
| 12414 | S15_23916237 | 15 | 23916237 | 15914 | A | G | A | 0.91935 | G | 0.08065 | 5 | 0.16129 |
| 12415 | S15_23940232 | 15 | 23940232 | 23995 | G | A | G | 0.95161 | A | 0.04839 | 3 | 0.09677 |
| 12416 | S15_23940265 | 15 | 23940265 | 33 | G | A | G | 0.87097 | A | 0.12903 | 8 | 0.25806 |
| 12417 | S15_23940268 | 15 | 23940268 | 3 | A | C | A | 0.59677 | C | 0.40323 | 15 | 0.48387 |
| 12418 | S15_24550070 | 15 | 24550070 | 609802 | G | T | G | 0.91935 | T | 0.08065 | 5 | 0.16129 |

| Site number | SNP name | Chromosome | Physical position | Physical distance from the previous SNP | Reference allele | Alternative allele | Major allele | Major allele frequency | Minor allele | Minor allele frequency | Number heterozygous | Heterozygosity |
|---|---|---|---|---|---|---|---|---|---|---|---|---|
| 12419 | S15_24764178 | 15 | 24764178 | 214108 | C | T | C | 0.70968 | T | 0.29032 | 12 | 0.3871 |
| 12420 | S15_25223618 | 15 | 25223618 | 459440 | C | T | C | 0.58065 | T | 0.41935 | 12 | 0.3871 |
| 12421 | S15_25223782 | 15 | 25223782 | 164 | T | C | T | 0.77419 | C | 0.22581 | 8 | 0.25806 |
| 12422 | S15_25586397 | 15 | 25586397 | 362615 | C | G | C | 0.77419 | G | 0.22581 | 12 | 0.3871 |
| 12423 | S15_25656221 | 15 | 25656221 | 69824 | G | A | G | 0.90323 | A | 0.09677 | 6 | 0.19355 |
| 12424 | S15_25656365 | 15 | 25656365 | 144 | C | T | C | 0.8871 | T | 0.1129 | 7 | 0.22581 |
| 12425 | S15_25704378 | 15 | 25704378 | 48013 | T | A | T | 0.93548 | A | 0.06452 | 4 | 0.12903 |
| 12426 | S15_26760196 | 15 | 26760196 | 1055818 | T | G | G | 0.82258 | T | 0.17742 | 11 | 0.35484 |
| 12427 | S15_26760214 | 15 | 26760214 | 18 | T | A | T | 0.80645 | A | 0.19355 | 12 | 0.3871 |
| 12428 | S15_26760253 | 15 | 26760253 | 39 | G | A | G | 0.80645 | A | 0.19355 | 12 | 0.3871 |
| 12429 | S15_26818645 | 15 | 26818645 | 58392 | G | A | G | 0.93548 | A | 0.06452 | 2 | 0.06452 |
| 12430 | S15_26818723 | 15 | 26818723 | 78 | G | A | G | 0.77419 | A | 0.22581 | 12 | 0.3871 |
| 12431 | S15_27407508 | 15 | 27407508 | 588785 | C | T | C | 0.74194 | T | 0.25806 | 16 | 0.51613 |
| 12432 | S15_28238108 | 15 | 28238108 | 830600 | G | A | A | 0.53226 | G | 0.46774 | 15 | 0.48387 |
| 12433 | S15_28238176 | 15 | 28238176 | 68 | C | G | C | 0.91935 | G | 0.08065 | 5 | 0.16129 |
| 12434 | S15_28869247 | 15 | 28869247 | 631071 | G | A | G | 0.95161 | A | 0.04839 | 3 | 0.09677 |
| 12435 | S15_28869284 | 15 | 28869284 | 37 | C | A | C | 0.80645 | A | 0.19355 | 8 | 0.25806 |
| 12436 | S15_28869337 | 15 | 28869337 | 53 | A | G | A | 0.95161 | G | 0.04839 | 3 | 0.09677 |
| 12437 | S15_29612771 | 15 | 29612771 | 743434 | G | A | G | 0.90323 | A | 0.09677 | 6 | 0.19355 |
| 12438 | S15_29612815 | 15 | 29612815 | 44 | G | A | G | 0.91935 | A | 0.08065 | 5 | 0.16129 |
| 12439 | S15_30096618 | 15 | 30096618 | 483803 | G | A | G | 0.91935 | A | 0.08065 | 5 | 0.16129 |
| 12440 | S15_30541664 | 15 | 30541664 | 445046 | G | A | G | 0.90323 | A | 0.09677 | 6 | 0.19355 |
| 12441 | S15_30541753 | 15 | 30541753 | 89 | A | G | G | 0.85484 | A | 0.14516 | 7 | 0.22581 |
| 12442 | S15_30541769 | 15 | 30541769 | 16 | C | T | C | 0.77419 | T | 0.22581 | 12 | 0.3871 |
| 12443 | S15_30717961 | 15 | 30717961 | 176192 | C | T | C | 0.91935 | T | 0.08065 | 5 | 0.16129 |
| 12444 | S15_30717962 | 15 | 30717962 | 1 | G | A | G | 0.8871 | A | 0.1129 | 7 | 0.22581 |
| 12445 | S15_30900749 | 15 | 30900749 | 182787 | T | G | T | 0.75806 | G | 0.24194 | 15 | 0.48387 |
| 12446 | S15_30900805 | 15 | 30900805 | 56 | G | A | G | 0.75806 | A | 0.24194 | 15 | 0.48387 |
| 12447 | S15_30900816 | 15 | 30900816 | 11 | C | T | C | 0.85484 | T | 0.14516 | 9 | 0.29032 |
| 12448 | S15_30900870 | 15 | 30900870 | 54 | A | G | A | 0.75806 | G | 0.24194 | 15 | 0.48387 |
| 12449 | S15_30939985 | 15 | 30939985 | 39115 | C | T | C | 0.85484 | T | 0.14516 | 7 | 0.22581 |
| 12450 | S15_30975709 | 15 | 30975709 | 35724 | G | A | G | 0.51613 | A | 0.48387 | 16 | 0.51613 |
| 12451 | S15_30975712 | 15 | 30975712 | 3 | T | C | T | 0.51613 | C | 0.48387 | 16 | 0.51613 |
| 12452 | S15_30975723 | 15 | 30975723 | 11 | T | C | T | 0.51613 | C | 0.48387 | 16 | 0.51613 |
| 12453 | S15_30975839 | 15 | 30975839 | 116 | G | A | G | 0.64516 | A | 0.35484 | 14 | 0.45161 |
| 12454 | S15_31142298 | 15 | 31142298 | 166459 | T | C | T | 0.93548 | C | 0.06452 | 4 | 0.12903 |
| 12455 | S15_31142429 | 15 | 31142429 | 131 | A | G | A | 0.79032 | G | 0.20968 | 9 | 0.29032 |
| 12456 | S15_32107419 | 15 | 32107419 | 964990 | C | T | C | 0.8871 | T | 0.1129 | 7 | 0.22581 |
| 12457 | S15_32107450 | 15 | 32107450 | 31 | A | C | A | 0.8871 | C | 0.1129 | 7 | 0.22581 |
| 12458 | S15_32221701 | 15 | 32221701 | 114251 | G | A | G | 0.83871 | A | 0.16129 | 10 | 0.32258 |
| 12459 | S15_32366304 | 15 | 32366304 | 144603 | T | C | T | 0.8871 | C | 0.1129 | 7 | 0.22581 |
| 12460 | S15_33060511 | 15 | 33060511 | 694207 | A | T | A | 0.91935 | T | 0.08065 | 5 | 0.16129 |
| 12461 | S15_33060540 | 15 | 33060540 | 29 | T | C | T | 0.91935 | C | 0.08065 | 5 | 0.16129 |
| 12462 | S15_33060678 | 15 | 33060678 | 138 | A | G | A | 0.83871 | G | 0.16129 | 8 | 0.25806 |
| 12463 | S15_33904805 | 15 | 33904805 | 844127 | G | A | G | 0.95161 | A | 0.04839 | 1 | 0.03226 |
| 12464 | S15_34238068 | 15 | 34238068 | 333263 | T | G | T | 0.95161 | G | 0.04839 | 3 | 0.09677 |
| 12465 | S15_34238204 | 15 | 34238204 | 136 | C | A | A | 0.90323 | C | 0.09677 | 6 | 0.19355 |
| 12466 | S15_34375596 | 15 | 34375596 | 137392 | C | T | C | 0.54839 | T | 0.45161 | 18 | 0.58065 |
| 12467 | S15_34375600 | 15 | 34375600 | 4 | C | T | T | 0.74194 | C | 0.25806 | 12 | 0.3871 |
| 12468 | S15_34375614 | 15 | 34375614 | 14 | C | T | C | 0.69355 | T | 0.30645 | 13 | 0.41935 |
| 12469 | S15_34375646 | 15 | 34375646 | 32 | C | T | C | 0.74194 | T | 0.25806 | 12 | 0.3871 |
| 12470 | S15_34375746 | 15 | 34375746 | 100 | T | C | T | 0.93548 | C | 0.06452 | 4 | 0.12903 |
| 12471 | S15_34375763 | 15 | 34375763 | 17 | A | G | A | 0.54839 | G | 0.45161 | 18 | 0.58065 |
| 12472 | S15_34718664 | 15 | 34718664 | 342901 | C | T | C | 0.82258 | T | 0.17742 | 7 | 0.22581 |

| Site number | SNP name | Chromosome | Physical position | Physical distance from the previous SNP | Reference allele | Alternative allele | Major allele | Major allele frequency | Minor allele | Minor allele frequency | Number heterozygous | Heterozygosity |
|---|---|---|---|---|---|---|---|---|---|---|---|---|
| 12473 | S15_35308449 | 15 | 35308449 | 589785 | G | A | G | 0.95161 | A | 0.04839 | 3 | 0.09677 |
| 12474 | S15_35308496 | 15 | 35308496 | 47 | A | G | G | 0.58065 | A | 0.41935 | 14 | 0.45161 |
| 12475 | S15_35308512 | 15 | 35308512 | 16 | A | C | C | 0.51613 | A | 0.48387 | 16 | 0.51613 |
| 12476 | S15_35708162 | 15 | 35708162 | 399650 | G | A | A | 0.82258 | G | 0.17742 | 9 | 0.29032 |
| 12477 | S15_35950021 | 15 | 35950021 | 241859 | G | A | G | 0.95161 | A | 0.04839 | 3 | 0.09677 |
| 12478 | S15_35950126 | 15 | 35950126 | 105 | C | T | C | 0.87097 | T | 0.12903 | 8 | 0.25806 |
| 12479 | S15_35950135 | 15 | 35950135 | 9 | T | C | T | 0.95161 | C | 0.04839 | 3 | 0.09677 |
| 12480 | S15_36284082 | 15 | 36284082 | 333947 | A | G | A | 0.80645 | G | 0.19355 | 12 | 0.3871 |
| 12481 | S15_36296787 | 15 | 36296787 | 12705 | G | A | G | 0.93548 | A | 0.06452 | 4 | 0.12903 |
| 12482 | S15_36296789 | 15 | 36296789 | 2 | T | G | T | 0.80645 | G | 0.19355 | 12 | 0.3871 |
| 12483 | S15_36296904 | 15 | 36296904 | 115 | C | G | G | 0.80645 | C | 0.19355 | 12 | 0.3871 |
| 12484 | S15_36296931 | 15 | 36296931 | 27 | A | T | A | 0.93548 | T | 0.06452 | 4 | 0.12903 |
| 12485 | S15_36299446 | 15 | 36299446 | 2515 | T | C | C | 0.79032 | T | 0.20968 | 13 | 0.41935 |
| 12486 | S15_36299462 | 15 | 36299462 | 16 | C | T | C | 0.93548 | T | 0.06452 | 4 | 0.12903 |
| 12487 | S15_36299556 | 15 | 36299556 | 94 | G | A | G | 0.91935 | A | 0.08065 | 5 | 0.16129 |
| 12488 | S15_36299612 | 15 | 36299612 | 56 | C | A | A | 0.79032 | C | 0.20968 | 13 | 0.41935 |
| 12489 | S15_36828592 | 15 | 36828592 | 528980 | G | A | G | 0.91935 | A | 0.08065 | 3 | 0.09677 |
| 12490 | S15_36828624 | 15 | 36828624 | 32 | C | T | C | 0.90323 | T | 0.09677 | 6 | 0.19355 |
| 12491 | S15_37107030 | 15 | 37107030 | 278406 | G | T | G | 0.95161 | T | 0.04839 | 3 | 0.09677 |
| 12492 | S15_37107081 | 15 | 37107081 | 51 | C | T | C | 0.93548 | T | 0.06452 | 2 | 0.06452 |
| 12493 | S15_37107091 | 15 | 37107091 | 10 | G | A | G | 0.93548 | A | 0.06452 | 4 | 0.12903 |
| 12494 | S15_37380891 | 15 | 37380891 | 273800 | C | T | T | 0.77419 | C | 0.22581 | 10 | 0.32258 |
| 12495 | S15_38134632 | 15 | 38134632 | 753741 | A | G | A | 0.91935 | G | 0.08065 | 5 | 0.16129 |
| 12496 | S15_38134676 | 15 | 38134676 | 44 | A | G | A | 0.91935 | G | 0.08065 | 5 | 0.16129 |
| 12497 | S15_38134690 | 15 | 38134690 | 14 | T | C | T | 0.91935 | C | 0.08065 | 5 | 0.16129 |
| 12498 | S15_38147706 | 15 | 38147706 | 13016 | A | G | A | 0.95161 | G | 0.04839 | 3 | 0.09677 |
| 12499 | S15_38147713 | 15 | 38147713 | 7 | A | G | G | 0.8871 | A | 0.1129 | 7 | 0.22581 |
| 12500 | S15_38147715 | 15 | 38147715 | 2 | A | G | A | 0.95161 | G | 0.04839 | 3 | 0.09677 |
| 12501 | S15_38168440 | 15 | 38168440 | 20725 | G | A | G | 0.93548 | A | 0.06452 | 2 | 0.06452 |
| 12502 | S15_39024582 | 15 | 39024582 | 856142 | C | T | C | 0.95161 | T | 0.04839 | 3 | 0.09677 |
| 12503 | S15_39088025 | 15 | 39088025 | 63443 | G | T | G | 0.95161 | T | 0.04839 | 3 | 0.09677 |
| 12504 | S15_39187966 | 15 | 39187966 | 99941 | A | G | A | 0.56452 | G | 0.43548 | 15 | 0.48387 |
| 12505 | S15_39188003 | 15 | 39188003 | 37 | C | T | C | 0.91935 | T | 0.08065 | 5 | 0.16129 |
| 12506 | S15_39188012 | 15 | 39188012 | 9 | C | G | C | 0.91935 | G | 0.08065 | 5 | 0.16129 |
| 12507 | S15_39400125 | 15 | 39400125 | 212113 | T | G | G | 0.67742 | T | 0.32258 | 14 | 0.45161 |
| 12508 | S15_39400144 | 15 | 39400144 | 19 | G | C | C | 0.67742 | G | 0.32258 | 14 | 0.45161 |
| 12509 | S15_39400179 | 15 | 39400179 | 35 | T | C | T | 0.83871 | C | 0.16129 | 10 | 0.32258 |
| 12510 | S15_39400193 | 15 | 39400193 | 14 | A | G | A | 0.79032 | G | 0.20968 | 9 | 0.29032 |
| 12511 | S15_39400198 | 15 | 39400198 | 5 | C | T | C | 0.82258 | T | 0.17742 | 7 | 0.22581 |
| 12512 | S15_39400206 | 15 | 39400206 | 8 | A | G | A | 0.85484 | G | 0.14516 | 5 | 0.16129 |
| 12513 | S15_39400265 | 15 | 39400265 | 59 | A | T | A | 0.83871 | T | 0.16129 | 10 | 0.32258 |
| 12514 | S15_39400295 | 15 | 39400295 | 30 | C | T | C | 0.82258 | T | 0.17742 | 7 | 0.22581 |
| 12515 | S15_39469371 | 15 | 39469371 | 69076 | A | G | A | 0.79032 | G | 0.20968 | 9 | 0.29032 |
| 12516 | S15_40638166 | 15 | 40638166 | 1168795 | G | A | G | 0.6129 | A | 0.3871 | 14 | 0.45161 |
| 12517 | S15_40638255 | 15 | 40638255 | 89 | C | T | C | 0.93548 | T | 0.06452 | 2 | 0.06452 |
| 12518 | S15_40821432 | 15 | 40821432 | 183177 | A | C | A | 0.85484 | C | 0.14516 | 7 | 0.22581 |
| 12519 | S15_40821496 | 15 | 40821496 | 64 | T | A | T | 0.95161 | A | 0.04839 | 3 | 0.09677 |
| 12520 | S15_40821503 | 15 | 40821503 | 7 | G | A | G | 0.85484 | A | 0.14516 | 7 | 0.22581 |
| 12521 | S15_40821621 | 15 | 40821621 | 118 | A | G | A | 0.79032 | G | 0.20968 | 9 | 0.29032 |
| 12522 | S15_40821631 | 15 | 40821631 | 10 | G | A | G | 0.77419 | A | 0.22581 | 10 | 0.32258 |
| 12523 | S15_40951076 | 15 | 40951076 | 129445 | G | A | G | 0.8871 | A | 0.1129 | 5 | 0.16129 |
| 12524 | S15_41676541 | 15 | 41676541 | 725465 | A | G | A | 0.87097 | G | 0.12903 | 8 | 0.25806 |
| 12525 | S15_42043445 | 15 | 42043445 | 366904 | A | G | A | 0.93548 | G | 0.06452 | 4 | 0.12903 |
| 12526 | S15_42043568 | 15 | 42043568 | 123 | C | G | C | 0.93548 | G | 0.06452 | 2 | 0.06452 |

| Site number | SNP name | Chromosome | Physical position | Physical distance from the previous SNP | Reference allele | Alternative allele | Major allele | Major allele frequency | Minor allele | Minor allele frequency | Number heterozygous | Heterozygosity |
|---|---|---|---|---|---|---|---|---|---|---|---|---|
| 12527 | S15_42043641 | 15 | 42043641 | 73 | G | C | G | 0.75806 | C | 0.24194 | 11 | 0.35484 |
| 12528 | S15_42153913 | 15 | 42153913 | 110272 | G | A | G | 0.90323 | A | 0.09677 | 6 | 0.19355 |
| 12529 | S15_42481478 | 15 | 42481478 | 327565 | G | A | G | 0.90323 | A | 0.09677 | 6 | 0.19355 |
| 12530 | S15_42481624 | 15 | 42481624 | 146 | A | G | G | 0.75806 | A | 0.24194 | 13 | 0.41935 |
| 12531 | S15_42546175 | 15 | 42546175 | 64551 | A | G | G | 0.69355 | A | 0.30645 | 15 | 0.48387 |
| 12532 | S15_42666073 | 15 | 42666073 | 119898 | A | T | A | 0.66129 | T | 0.33871 | 13 | 0.41935 |
| 12533 | S15_42666094 | 15 | 42666094 | 21 | A | C | A | 0.82258 | C | 0.17742 | 7 | 0.22581 |
| 12534 | S15_42666145 | 15 | 42666145 | 51 | C | T | C | 0.93548 | T | 0.06452 | 4 | 0.12903 |
| 12535 | S15_42801910 | 15 | 42801910 | 135765 | C | T | C | 0.83871 | T | 0.16129 | 8 | 0.25806 |
| 12536 | S15_42802019 | 15 | 42802019 | 109 | A | G | G | 0.51613 | A | 0.48387 | 18 | 0.58065 |
| 12537 | S15_42802054 | 15 | 42802054 | 35 | C | T | C | 0.93548 | T | 0.06452 | 4 | 0.12903 |
| 12538 | S15_42802100 | 15 | 42802100 | 46 | A | G | A | 0.80645 | G | 0.19355 | 10 | 0.32258 |
| 12539 | S15_42919172 | 15 | 42919172 | 117072 | C | T | C | 0.64516 | T | 0.35484 | 14 | 0.45161 |
| 12540 | S15_43039246 | 15 | 43039246 | 120074 | A | G | A | 0.91935 | G | 0.08065 | 5 | 0.16129 |
| 12541 | S15_43386907 | 15 | 43386907 | 347661 | G | T | G | 0.83871 | T | 0.16129 | 8 | 0.25806 |
| 12542 | S15_43387035 | 15 | 43387035 | 128 | G | A | G | 0.83871 | A | 0.16129 | 8 | 0.25806 |
| 12543 | S15_43387052 | 15 | 43387052 | 17 | G | C | G | 0.87097 | C | 0.12903 | 6 | 0.19355 |
| 12544 | S15_43389154 | 15 | 43389154 | 2102 | A | G | A | 0.91935 | G | 0.08065 | 5 | 0.16129 |
| 12545 | S15_43580532 | 15 | 43580532 | 191378 | C | T | C | 0.66129 | T | 0.33871 | 9 | 0.29032 |
| 12546 | S15_43580556 | 15 | 43580556 | 24 | C | T | T | 0.72581 | C | 0.27419 | 13 | 0.41935 |
| 12547 | S15_43580563 | 15 | 43580563 | 7 | A | G | G | 0.83871 | A | 0.16129 | 8 | 0.25806 |
| 12548 | S15_43580589 | 15 | 43580589 | 26 | C | T | T | 0.72581 | C | 0.27419 | 13 | 0.41935 |
| 12549 | S15_43789946 | 15 | 43789946 | 209357 | T | C | T | 0.72581 | C | 0.27419 | 15 | 0.48387 |
| 12550 | S15_43789980 | 15 | 43789980 | 34 | T | G | T | 0.80645 | G | 0.19355 | 8 | 0.25806 |
| 12551 | S15_43790062 | 15 | 43790062 | 82 | T | C | C | 0.67742 | T | 0.32258 | 10 | 0.32258 |
| 12552 | S15_43868637 | 15 | 43868637 | 78575 | A | C | A | 0.95161 | C | 0.04839 | 3 | 0.09677 |
| 12553 | S15_43868686 | 15 | 43868686 | 49 | G | C | G | 0.66129 | C | 0.33871 | 15 | 0.48387 |
| 12554 | S15_43868702 | 15 | 43868702 | 16 | C | T | C | 0.93548 | T | 0.06452 | 4 | 0.12903 |
| 12555 | S15_44154469 | 15 | 44154469 | 285767 | T | C | C | 0.62903 | T | 0.37097 | 11 | 0.35484 |
| 12556 | S15_44154584 | 15 | 44154584 | 115 | G | T | G | 0.85484 | T | 0.14516 | 7 | 0.22581 |
| 12557 | S15_44205091 | 15 | 44205091 | 50507 | C | T | C | 0.95161 | T | 0.04839 | 3 | 0.09677 |
| 12558 | S15_44205092 | 15 | 44205092 | 1 | G | A | G | 0.83871 | A | 0.16129 | 10 | 0.32258 |
| 12559 | S15_44205107 | 15 | 44205107 | 15 | G | C | G | 0.85484 | C | 0.14516 | 7 | 0.22581 |
| 12560 | S15_44321810 | 15 | 44321810 | 116703 | T | C | C | 0.91935 | T | 0.08065 | 5 | 0.16129 |
| 12561 | S15_44330356 | 15 | 44330356 | 8546 | A | G | A | 0.95161 | G | 0.04839 | 3 | 0.09677 |
| 12562 | S15_44533275 | 15 | 44533275 | 202919 | C | T | C | 0.91935 | T | 0.08065 | 5 | 0.16129 |
| 12563 | S15_44533307 | 15 | 44533307 | 32 | C | G | C | 0.87097 | G | 0.12903 | 8 | 0.25806 |
| 12564 | S15_44757966 | 15 | 44757966 | 224659 | G | A | G | 0.59677 | A | 0.40323 | 13 | 0.41935 |
| 12565 | S15_44758016 | 15 | 44758016 | 50 | C | T | C | 0.90323 | T | 0.09677 | 4 | 0.12903 |
| 12566 | S15_44758020 | 15 | 44758020 | 4 | A | G | G | 0.95161 | A | 0.04839 | 3 | 0.09677 |
| 12567 | S15_44758143 | 15 | 44758143 | 123 | G | A | G | 0.59677 | A | 0.40323 | 13 | 0.41935 |
| 12568 | S15_44923277 | 15 | 44923277 | 165134 | A | G | A | 0.79032 | G | 0.20968 | 7 | 0.22581 |
| 12569 | S15_45344191 | 15 | 45344191 | 420914 | G | A | G | 0.90323 | A | 0.09677 | 4 | 0.12903 |
| 12570 | S15_45344252 | 15 | 45344252 | 61 | G | A | G | 0.90323 | A | 0.09677 | 6 | 0.19355 |
| 12571 | S15_45344307 | 15 | 45344307 | 55 | A | G | G | 0.70968 | A | 0.29032 | 10 | 0.32258 |
| 12572 | S15_45350494 | 15 | 45350494 | 6187 | A | C | A | 0.93548 | C | 0.06452 | 4 | 0.12903 |
| 12573 | S15_45779180 | 15 | 45779180 | 428686 | A | T | A | 0.91935 | T | 0.08065 | 5 | 0.16129 |
| 12574 | S15_45779268 | 15 | 45779268 | 88 | G | A | A | 0.72581 | G | 0.27419 | 13 | 0.41935 |
| 12575 | S15_45779286 | 15 | 45779286 | 18 | T | C | C | 0.74194 | T | 0.25806 | 12 | 0.3871 |
| 12576 | S15_45779351 | 15 | 45779351 | 65 | T | C | C | 0.74194 | T | 0.25806 | 12 | 0.3871 |
| 12577 | S15_45813520 | 15 | 45813520 | 34169 | A | G | A | 0.79032 | G | 0.20968 | 13 | 0.41935 |
| 12578 | S15_46118670 | 15 | 46118670 | 305150 | C | G | C | 0.67742 | G | 0.32258 | 14 | 0.45161 |
| 12579 | S15_46739109 | 15 | 46739109 | 620439 | T | C | T | 0.74194 | C | 0.25806 | 14 | 0.45161 |
| 12580 | S15_46832253 | 15 | 46832253 | 93144 | G | A | G | 0.90323 | A | 0.09677 | 4 | 0.12903 |

| Site number | SNP name | Chromosome | Physical position | Physical distance from the previous SNP | Reference allele | Alternative allele | Major allele | Major allele frequency | Minor allele | Minor allele frequency | Number heterozygous | Heterozygosity |
|---|---|---|---|---|---|---|---|---|---|---|---|---|
| 12581 | S15_46914625 | 15 | 46914625 | 82372 | A | G | G | 0.79032 | A | 0.20968 | 7 | 0.22581 |
| 12582 | S15_46914777 | 15 | 46914777 | 152 | G | A | G | 0.56452 | A | 0.43548 | 13 | 0.41935 |
| 12583 | S15_46988230 | 15 | 46988230 | 73453 | G | A | G | 0.8871 | A | 0.1129 | 7 | 0.22581 |
| 12584 | S15_47203325 | 15 | 47203325 | 215095 | G | A | G | 0.95161 | A | 0.04839 | 3 | 0.09677 |
| 12585 | S15_47203332 | 15 | 47203332 | 7 | G | A | G | 0.82258 | A | 0.17742 | 9 | 0.29032 |
| 12586 | S15_47203382 | 15 | 47203382 | 50 | G | A | G | 0.91935 | A | 0.08065 | 5 | 0.16129 |
| 12587 | S15_47203432 | 15 | 47203432 | 50 | C | T | C | 0.91935 | T | 0.08065 | 5 | 0.16129 |
| 12588 | S15_47485683 | 15 | 47485683 | 282251 | C | T | C | 0.59677 | T | 0.40323 | 13 | 0.41935 |
| 12589 | S15_47485759 | 15 | 47485759 | 76 | A | G | A | 0.62903 | G | 0.37097 | 13 | 0.41935 |
| 12590 | S15_47628020 | 15 | 47628020 | 142261 | T | C | T | 0.87097 | C | 0.12903 | 8 | 0.25806 |
| 12591 | S15_47831275 | 15 | 47831275 | 203255 | G | A | G | 0.77419 | A | 0.22581 | 14 | 0.45161 |
| 12592 | S15_47954988 | 15 | 47954988 | 123713 | G | A | G | 0.95161 | A | 0.04839 | 3 | 0.09677 |
| 12593 | S15_47955000 | 15 | 47955000 | 12 | G | T | G | 0.93548 | T | 0.06452 | 4 | 0.12903 |
| 12594 | S15_48088121 | 15 | 48088121 | 133121 | C | T | C | 0.90323 | T | 0.09677 | 6 | 0.19355 |
| 12595 | S15_48097149 | 15 | 48097149 | 9028 | G | A | G | 0.82258 | A | 0.17742 | 9 | 0.29032 |
| 12596 | S15_48299933 | 15 | 48299933 | 202784 | G | A | G | 0.91935 | A | 0.08065 | 5 | 0.16129 |
| 12597 | S15_48299958 | 15 | 48299958 | 25 | C | T | C | 0.58065 | T | 0.41935 | 16 | 0.51613 |
| 12598 | S15_48300016 | 15 | 48300016 | 58 | G | A | G | 0.93548 | A | 0.06452 | 4 | 0.12903 |
| 12599 | S15_48420319 | 15 | 48420319 | 120303 | G | A | G | 0.79032 | A | 0.20968 | 7 | 0.22581 |
| 12600 | S15_48493699 | 15 | 48493699 | 73380 | C | G | C | 0.5 | G | 0.5 | 17 | 0.54839 |
| 12601 | S15_48493718 | 15 | 48493718 | 19 | T | C | C | 0.83871 | T | 0.16129 | 10 | 0.32258 |
| 12602 | S15_48493789 | 15 | 48493789 | 71 | A | T | A | 0.5 | T | 0.5 | 17 | 0.54839 |
| 12603 | S15_48593890 | 15 | 48593890 | 100101 | A | G | G | 0.77419 | A | 0.22581 | 12 | 0.3871 |
| 12604 | S15_48780719 | 15 | 48780719 | 186829 | G | A | G | 0.87097 | A | 0.12903 | 8 | 0.25806 |
| 12605 | S15_48780755 | 15 | 48780755 | 36 | G | A | G | 0.91935 | A | 0.08065 | 5 | 0.16129 |
| 12606 | S15_48780799 | 15 | 48780799 | 44 | A | G | A | 0.87097 | G | 0.12903 | 8 | 0.25806 |
| 12607 | S15_48780884 | 15 | 48780884 | 85 | A | C | A | 0.87097 | C | 0.12903 | 8 | 0.25806 |
| 12608 | S15_48851729 | 15 | 48851729 | 70845 | A | T | A | 0.90323 | T | 0.09677 | 6 | 0.19355 |
| 12609 | S15_48851841 | 15 | 48851841 | 112 | G | A | G | 0.95161 | A | 0.04839 | 3 | 0.09677 |
| 12610 | S15_49016180 | 15 | 49016180 | 164339 | G | A | G | 0.91935 | A | 0.08065 | 5 | 0.16129 |
| 12611 | S15_49084407 | 15 | 49084407 | 68227 | C | T | C | 0.91935 | T | 0.08065 | 5 | 0.16129 |
| 12612 | S15_49084492 | 15 | 49084492 | 85 | A | C | A | 0.87097 | C | 0.12903 | 6 | 0.19355 |
| 12613 | S15_49084551 | 15 | 49084551 | 59 | G | A | G | 0.91935 | A | 0.08065 | 5 | 0.16129 |
| 12614 | S15_49099649 | 15 | 49099649 | 15098 | C | T | C | 0.95161 | T | 0.04839 | 3 | 0.09677 |
| 12615 | S15_49099671 | 15 | 49099671 | 22 | A | G | A | 0.95161 | G | 0.04839 | 3 | 0.09677 |
| 12616 | S15_49111756 | 15 | 49111756 | 12085 | T | C | T | 0.87097 | C | 0.12903 | 6 | 0.19355 |
| 12617 | S15_49111833 | 15 | 49111833 | 77 | A | G | A | 0.95161 | G | 0.04839 | 3 | 0.09677 |
| 12618 | S15_49122738 | 15 | 49122738 | 10905 | G | A | G | 0.93548 | A | 0.06452 | 4 | 0.12903 |
| 12619 | S15_49122847 | 15 | 49122847 | 109 | C | A | C | 0.87097 | A | 0.12903 | 6 | 0.19355 |
| 12620 | S15_49122909 | 15 | 49122909 | 62 | A | C | A | 0.56452 | C | 0.43548 | 15 | 0.48387 |
| 12621 | S15_49170628 | 15 | 49170628 | 47719 | A | T | T | 0.56452 | A | 0.43548 | 15 | 0.48387 |
| 12622 | S15_49269408 | 15 | 49269408 | 98780 | A | G | A | 0.95161 | G | 0.04839 | 1 | 0.03226 |
| 12623 | S15_49505912 | 15 | 49505912 | 236504 | C | T | C | 0.95161 | T | 0.04839 | 3 | 0.09677 |
| 12624 | S15_49727508 | 15 | 49727508 | 221596 | C | T | C | 0.75806 | T | 0.24194 | 11 | 0.35484 |
| 12625 | S15_49909272 | 15 | 49909272 | 181764 | C | T | C | 0.83871 | T | 0.16129 | 6 | 0.19355 |
| 12626 | S15_50007617 | 15 | 50007617 | 98345 | G | A | G | 0.62903 | A | 0.37097 | 15 | 0.48387 |
| 12627 | S15_50007688 | 15 | 50007688 | 71 | C | T | C | 0.79032 | T | 0.20968 | 9 | 0.29032 |
| 12628 | S15_50007759 | 15 | 50007759 | 71 | G | A | G | 0.91935 | A | 0.08065 | 5 | 0.16129 |
| 12629 | S15_50383513 | 15 | 50383513 | 375754 | C | A | A | 0.85484 | C | 0.14516 | 7 | 0.22581 |
| 12630 | S15_50444451 | 15 | 50444451 | 60938 | C | T | C | 0.75806 | T | 0.24194 | 13 | 0.41935 |
| 12631 | S15_50475364 | 15 | 50475364 | 30913 | G | A | G | 0.85484 | A | 0.14516 | 9 | 0.29032 |
| 12632 | S15_50606243 | 15 | 50606243 | 130879 | T | C | T | 0.91935 | C | 0.08065 | 5 | 0.16129 |
| 12633 | S15_50754714 | 15 | 50754714 | 148471 | G | A | G | 0.77419 | A | 0.22581 | 12 | 0.3871 |
| 12634 | S15_50754767 | 15 | 50754767 | 53 | G | T | G | 0.83871 | T | 0.16129 | 10 | 0.32258 |

| Site number | SNP name | Chromosome | Physical position | Physical distance from the previous SNP | Reference allele | Alternative allele | Major allele | Major allele frequency | Minor allele | Minor allele frequency | Number heterozygous | Heterozygosity |
|---|---|---|---|---|---|---|---|---|---|---|---|---|
| 12635 | S15_50878110 | 15 | 50878110 | 123343 | C | T | C | 0.91935 | T | 0.08065 | 5 | 0.16129 |
| 12636 | S15_51061385 | 15 | 51061385 | 183275 | G | C | G | 0.8871 | C | 0.1129 | 7 | 0.22581 |
| 12637 | S15_51061464 | 15 | 51061464 | 79 | T | G | T | 0.8871 | G | 0.1129 | 7 | 0.22581 |
| 12638 | S15_51237290 | 15 | 51237290 | 175826 | G | A | G | 0.72581 | A | 0.27419 | 11 | 0.35484 |
| 12639 | S15_51328674 | 15 | 51328674 | 91384 | C | A | C | 0.87097 | A | 0.12903 | 8 | 0.25806 |
| 12640 | S15_51328727 | 15 | 51328727 | 53 | C | A | C | 0.87097 | A | 0.12903 | 8 | 0.25806 |
| 12641 | S15_51464811 | 15 | 51464811 | 136084 | C | T | C | 0.82258 | T | 0.17742 | 11 | 0.35484 |
| 12642 | S15_51464837 | 15 | 51464837 | 26 | T | G | T | 0.82258 | G | 0.17742 | 11 | 0.35484 |
| 12643 | S15_51705609 | 15 | 51705609 | 240772 | C | T | C | 0.95161 | T | 0.04839 | 3 | 0.09677 |
| 12644 | S15_51730661 | 15 | 51730661 | 25052 | T | C | T | 0.93548 | C | 0.06452 | 4 | 0.12903 |
| 12645 | S15_51916760 | 15 | 51916760 | 186099 | T | A | T | 0.93548 | A | 0.06452 | 4 | 0.12903 |
| 12646 | S15_51916826 | 15 | 51916826 | 66 | C | G | C | 0.93548 | G | 0.06452 | 4 | 0.12903 |
| 12647 | S15_52776402 | 15 | 52776402 | 859576 | C | T | C | 0.66129 | T | 0.33871 | 17 | 0.54839 |
| 12648 | S15_52776618 | 15 | 52776618 | 216 | G | A | G | 0.87097 | A | 0.12903 | 8 | 0.25806 |
| 12649 | S15_52870335 | 15 | 52870335 | 93717 | A | T | T | 0.54839 | A | 0.45161 | 16 | 0.51613 |
| 12650 | S15_53526562 | 15 | 53526562 | 656227 | A | G | A | 0.91935 | G | 0.08065 | 5 | 0.16129 |
| 12651 | S15_53568888 | 15 | 53568888 | 42326 | C | T | C | 0.51613 | T | 0.48387 | 14 | 0.45161 |
| 12652 | S15_53568901 | 15 | 53568901 | 13 | T | C | T | 0.95161 | C | 0.04839 | 3 | 0.09677 |
| 12653 | S15_53568949 | 15 | 53568949 | 48 | C | T | T | 0.93548 | C | 0.06452 | 4 | 0.12903 |
| 12654 | S15_53890253 | 15 | 53890253 | 321304 | C | T | C | 0.91935 | T | 0.08065 | 3 | 0.09677 |
| 12655 | S15_54223096 | 15 | 54223096 | 332843 | G | A | G | 0.93548 | A | 0.06452 | 4 | 0.12903 |
| 12656 | S15_54428232 | 15 | 54428232 | 205136 | A | G | A | 0.93548 | G | 0.06452 | 4 | 0.12903 |
| 12657 | S15_54792526 | 15 | 54792526 | 364294 | T | C | T | 0.82258 | C | 0.17742 | 11 | 0.35484 |
| 12658 | S15_54792542 | 15 | 54792542 | 16 | C | T | T | 0.93548 | C | 0.06452 | 4 | 0.12903 |
| 12659 | S15_54792642 | 15 | 54792642 | 100 | A | G | A | 0.87097 | G | 0.12903 | 6 | 0.19355 |
| 12660 | S15_54963804 | 15 | 54963804 | 171162 | G | T | G | 0.93548 | T | 0.06452 | 4 | 0.12903 |
| 12661 | S15_54963900 | 15 | 54963900 | 96 | A | T | A | 0.75806 | T | 0.24194 | 11 | 0.35484 |
| 12662 | S15_55023921 | 15 | 55023921 | 60021 | A | G | A | 0.83871 | G | 0.16129 | 10 | 0.32258 |
| 12663 | S15_55024010 | 15 | 55024010 | 89 | C | T | C | 0.83871 | T | 0.16129 | 10 | 0.32258 |
| 12664 | S15_55030896 | 15 | 55030896 | 6886 | G | A | G | 0.72581 | A | 0.27419 | 13 | 0.41935 |
| 12665 | S15_55030965 | 15 | 55030965 | 69 | A | G | A | 0.72581 | G | 0.27419 | 13 | 0.41935 |
| 12666 | S15_55308443 | 15 | 55308443 | 277478 | G | A | G | 0.93548 | A | 0.06452 | 4 | 0.12903 |
| 12667 | S15_55359479 | 15 | 55359479 | 51036 | G | A | G | 0.85484 | A | 0.14516 | 5 | 0.16129 |
| 12668 | S15_55629131 | 15 | 55629131 | 269652 | A | G | G | 0.74194 | A | 0.25806 | 10 | 0.32258 |
| 12669 | S15_55629202 | 15 | 55629202 | 71 | G | T | G | 0.90323 | T | 0.09677 | 6 | 0.19355 |
| 12670 | S15_55629232 | 15 | 55629232 | 30 | C | T | C | 0.87097 | T | 0.12903 | 6 | 0.19355 |
| 12671 | S15_55629249 | 15 | 55629249 | 17 | T | C | T | 0.93548 | C | 0.06452 | 4 | 0.12903 |
| 12672 | S15_56035846 | 15 | 56035846 | 406597 | A | C | C | 0.79032 | A | 0.20968 | 11 | 0.35484 |
| 12673 | S15_56035853 | 15 | 56035853 | 7 | T | C | T | 0.8871 | C | 0.1129 | 7 | 0.22581 |
| 12674 | S15_56035988 | 15 | 56035988 | 135 | G | A | G | 0.8871 | A | 0.1129 | 7 | 0.22581 |
| 12675 | S15_56609229 | 15 | 56609229 | 573241 | C | T | C | 0.79032 | T | 0.20968 | 11 | 0.35484 |
| 12676 | S15_56609238 | 15 | 56609238 | 9 | G | A | G | 0.53226 | A | 0.46774 | 19 | 0.6129 |
| 12677 | S15_56887305 | 15 | 56887305 | 278067 | A | G | A | 0.59677 | G | 0.40323 | 19 | 0.6129 |
| 12678 | S15_56887358 | 15 | 56887358 | 53 | T | C | C | 0.53226 | T | 0.46774 | 17 | 0.54839 |
| 12679 | S15_56887370 | 15 | 56887370 | 12 | T | C | C | 0.83871 | T | 0.16129 | 8 | 0.25806 |
| 12680 | S15_57109495 | 15 | 57109495 | 222125 | G | A | G | 0.79032 | A | 0.20968 | 11 | 0.35484 |
| 12681 | S15_57109532 | 15 | 57109532 | 37 | T | C | C | 0.95161 | T | 0.04839 | 1 | 0.03226 |
| 12682 | S15_57109533 | 15 | 57109533 | 1 | G | A | G | 0.79032 | A | 0.20968 | 11 | 0.35484 |
| 12683 | S15_57109546 | 15 | 57109546 | 13 | T | C | C | 0.95161 | T | 0.04839 | 1 | 0.03226 |
| 12684 | S15_57109613 | 15 | 57109613 | 67 | T | C | T | 0.93548 | C | 0.06452 | 4 | 0.12903 |
| 12685 | S15_57109685 | 15 | 57109685 | 72 | A | G | A | 0.79032 | G | 0.20968 | 11 | 0.35484 |
| 12686 | S15_57509883 | 15 | 57509883 | 400198 | G | A | G | 0.93548 | A | 0.06452 | 4 | 0.12903 |
| 12687 | S15_57654359 | 15 | 57654359 | 144476 | C | T | C | 0.8871 | T | 0.1129 | 7 | 0.22581 |
| 12688 | S15_57686270 | 15 | 57686270 | 31911 | T | C | T | 0.6129 | C | 0.3871 | 14 | 0.45161 |

| Site number | SNP name | Chromosome | Physical position | Physical distance from the previous SNP | Reference allele | Alternative allele | Major allele | Major allele frequency | Minor allele | Minor allele frequency | Number heterozygous | Heterozygosity |
|---|---|---|---|---|---|---|---|---|---|---|---|---|
| 12689 | S15_57686282 | 15 | 57686282 | 12 | G | A | A | 0.51613 | G | 0.48387 | 16 | 0.51613 |
| 12690 | S15_57686405 | 15 | 57686405 | 123 | G | T | T | 0.59677 | G | 0.40323 | 15 | 0.48387 |
| 12691 | S15_57686425 | 15 | 57686425 | 20 | G | A | G | 0.93548 | A | 0.06452 | 4 | 0.12903 |
| 12692 | S15_57716066 | 15 | 57716066 | 29641 | G | A | A | 0.66129 | G | 0.33871 | 11 | 0.35484 |
| 12693 | S15_57868950 | 15 | 57868950 | 152884 | T | A | T | 0.83871 | A | 0.16129 | 10 | 0.32258 |
| 12694 | S15_57869017 | 15 | 57869017 | 67 | C | T | C | 0.83871 | T | 0.16129 | 10 | 0.32258 |
| 12695 | S15_57869018 | 15 | 57869018 | 1 | A | G | A | 0.70968 | G | 0.29032 | 12 | 0.3871 |
| 12696 | S15_57869026 | 15 | 57869026 | 8 | C | T | C | 0.83871 | T | 0.16129 | 10 | 0.32258 |
| 12697 | S15_58083482 | 15 | 58083482 | 214456 | T | C | T | 0.82258 | C | 0.17742 | 11 | 0.35484 |
| 12698 | S15_58083487 | 15 | 58083487 | 5 | C | T | C | 0.8871 | T | 0.1129 | 7 | 0.22581 |
| 12699 | S15_58133101 | 15 | 58133101 | 49614 | T | G | T | 0.87097 | G | 0.12903 | 8 | 0.25806 |
| 12700 | S15_58133138 | 15 | 58133138 | 37 | G | A | G | 0.87097 | A | 0.12903 | 8 | 0.25806 |
| 12701 | S15_58133310 | 15 | 58133310 | 172 | C | T | T | 0.87097 | C | 0.12903 | 8 | 0.25806 |
| 12702 | S15_58135999 | 15 | 58135999 | 2689 | G | A | G | 0.64516 | A | 0.35484 | 16 | 0.51613 |
| 12703 | S15_58439888 | 15 | 58439888 | 303889 | A | G | A | 0.62903 | G | 0.37097 | 15 | 0.48387 |
| 12704 | S15_58439944 | 15 | 58439944 | 56 | C | T | C | 0.77419 | T | 0.22581 | 14 | 0.45161 |
| 12705 | S15_58779726 | 15 | 58779726 | 339782 | A | G | A | 0.80645 | G | 0.19355 | 10 | 0.32258 |
| 12706 | S15_58803363 | 15 | 58803363 | 23637 | C | T | C | 0.62903 | T | 0.37097 | 17 | 0.54839 |
| 12707 | S15_58803400 | 15 | 58803400 | 37 | G | A | G | 0.69355 | A | 0.30645 | 17 | 0.54839 |
| 12708 | S15_58803416 | 15 | 58803416 | 16 | C | T | C | 0.87097 | T | 0.12903 | 6 | 0.19355 |
| 12709 | S15_58803432 | 15 | 58803432 | 16 | G | A | G | 0.51613 | A | 0.48387 | 16 | 0.51613 |
| 12710 | S15_58861541 | 15 | 58861541 | 58109 | G | A | G | 0.74194 | A | 0.25806 | 14 | 0.45161 |
| 12711 | S15_58874618 | 15 | 58874618 | 13077 | C | T | C | 0.83871 | T | 0.16129 | 10 | 0.32258 |
| 12712 | S15_58874637 | 15 | 58874637 | 19 | G | A | G | 0.91935 | A | 0.08065 | 5 | 0.16129 |
| 12713 | S15_59440007 | 15 | 59440007 | 565370 | A | C | C | 0.93548 | A | 0.06452 | 4 | 0.12903 |
| 12714 | S15_59440124 | 15 | 59440124 | 117 | A | G | G | 0.93548 | A | 0.06452 | 4 | 0.12903 |
| 12715 | S15_59440234 | 15 | 59440234 | 110 | C | T | T | 0.93548 | C | 0.06452 | 4 | 0.12903 |
| 12716 | S15_59660608 | 15 | 59660608 | 220374 | G | A | G | 0.93548 | A | 0.06452 | 2 | 0.06452 |
| 12717 | S15_59991691 | 15 | 59991691 | 331083 | G | A | G | 0.90323 | A | 0.09677 | 4 | 0.12903 |
| 12718 | S15_59991859 | 15 | 59991859 | 168 | C | T | C | 0.8871 | T | 0.1129 | 7 | 0.22581 |
| 12719 | S15_60536243 | 15 | 60536243 | 544384 | C | T | C | 0.85484 | T | 0.14516 | 7 | 0.22581 |
| 12720 | S15_60536298 | 15 | 60536298 | 55 | G | A | G | 0.93548 | A | 0.06452 | 4 | 0.12903 |
| 12721 | S15_60643032 | 15 | 60643032 | 106734 | C | G | C | 0.67742 | G | 0.32258 | 14 | 0.45161 |
| 12722 | S15_61027782 | 15 | 61027782 | 384750 | A | T | A | 0.95161 | T | 0.04839 | 3 | 0.09677 |
| 12723 | S15_61027882 | 15 | 61027882 | 100 | T | G | T | 0.93548 | G | 0.06452 | 4 | 0.12903 |
| 12724 | S15_61027922 | 15 | 61027922 | 40 | G | A | G | 0.95161 | A | 0.04839 | 1 | 0.03226 |
| 12725 | S15_61441565 | 15 | 61441565 | 413643 | C | T | T | 0.6129 | C | 0.3871 | 16 | 0.51613 |
| 12726 | S15_61441572 | 15 | 61441572 | 7 | C | T | C | 0.93548 | T | 0.06452 | 4 | 0.12903 |
| 12727 | S15_61626161 | 15 | 61626161 | 184589 | A | G | A | 0.93548 | G | 0.06452 | 4 | 0.12903 |
| 12728 | S15_61989922 | 15 | 61989922 | 363761 | T | C | T | 0.95161 | C | 0.04839 | 3 | 0.09677 |
| 12729 | S15_62035267 | 15 | 62035267 | 45345 | C | T | C | 0.69355 | T | 0.30645 | 17 | 0.54839 |
| 12730 | S15_62035420 | 15 | 62035420 | 153 | G | T | G | 0.90323 | T | 0.09677 | 6 | 0.19355 |
| 12731 | S15_62035461 | 15 | 62035461 | 41 | T | C | T | 0.93548 | C | 0.06452 | 4 | 0.12903 |
| 12732 | S15_62245133 | 15 | 62245133 | 209672 | A | T | A | 0.83871 | T | 0.16129 | 10 | 0.32258 |
| 12733 | S15_62319180 | 15 | 62319180 | 74047 | T | C | C | 0.79032 | T | 0.20968 | 11 | 0.35484 |
| 12734 | S15_62319206 | 15 | 62319206 | 26 | G | A | A | 0.79032 | G | 0.20968 | 11 | 0.35484 |
| 12735 | S15_62319220 | 15 | 62319220 | 14 | G | A | G | 0.83871 | A | 0.16129 | 10 | 0.32258 |
| 12736 | S15_62760000 | 15 | 62760000 | 440780 | T | C | C | 0.70968 | T | 0.29032 | 16 | 0.51613 |
| 12737 | S15_63561845 | 15 | 63561845 | 801845 | T | G | G | 0.72581 | T | 0.27419 | 13 | 0.41935 |
| 12738 | S15_63628729 | 15 | 63628729 | 66884 | A | G | G | 0.8871 | A | 0.1129 | 7 | 0.22581 |
| 12739 | S15_64021793 | 15 | 64021793 | 393064 | A | C | A | 0.64516 | C | 0.35484 | 12 | 0.3871 |
| 12740 | S15_64021810 | 15 | 64021810 | 17 | G | A | G | 0.64516 | A | 0.35484 | 12 | 0.3871 |
| 12741 | S15_64021832 | 15 | 64021832 | 22 | C | T | C | 0.64516 | T | 0.35484 | 12 | 0.3871 |
| 12742 | S15_64021934 | 15 | 64021934 | 102 | G | A | G | 0.93548 | A | 0.06452 | 2 | 0.06452 |

| Site number | SNP name | Chromosome | Physical position | Physical distance from the previous SNP | Reference allele | Alternative allele | Major allele | Major allele frequency | Minor allele | Minor allele frequency | Number heterozygous | Heterozygosity |
|---|---|---|---|---|---|---|---|---|---|---|---|---|
| 12743 | S15_64021946 | 15 | 64021946 | 12 | A | T | A | 0.56452 | T | 0.43548 | 13 | 0.41935 |
| 12744 | S15_64021947 | 15 | 64021947 | 1 | G | A | G | 0.91935 | A | 0.08065 | 3 | 0.09677 |
| 12745 | S15_64037184 | 15 | 64037184 | 15237 | G | A | G | 0.91935 | A | 0.08065 | 5 | 0.16129 |
| 12746 | S15_64037191 | 15 | 64037191 | 7 | G | A | G | 0.91935 | A | 0.08065 | 5 | 0.16129 |
| 12747 | S15_64037199 | 15 | 64037199 | 8 | C | T | C | 0.93548 | T | 0.06452 | 4 | 0.12903 |
| 12748 | S15_64037235 | 15 | 64037235 | 36 | T | C | T | 0.93548 | C | 0.06452 | 4 | 0.12903 |
| 12749 | S15_64421787 | 15 | 64421787 | 384552 | G | C | G | 0.95161 | C | 0.04839 | 3 | 0.09677 |
| 12750 | S15_64421953 | 15 | 64421953 | 166 | C | T | C | 0.75806 | T | 0.24194 | 11 | 0.35484 |
| 12751 | S15_64687322 | 15 | 64687322 | 265369 | C | T | C | 0.87097 | T | 0.12903 | 8 | 0.25806 |
| 12752 | S15_67122054 | 15 | 67122054 | 2434732 | T | C | T | 0.70968 | C | 0.29032 | 12 | 0.3871 |
| 12753 | S15_67122082 | 15 | 67122082 | 28 | T | C | T | 0.87097 | C | 0.12903 | 8 | 0.25806 |
| 12754 | S15_67420281 | 15 | 67420281 | 298199 | G | A | G | 0.83871 | A | 0.16129 | 8 | 0.25806 |
| 12755 | S15_67420418 | 15 | 67420418 | 137 | T | C | C | 0.69355 | T | 0.30645 | 11 | 0.35484 |
| 12756 | S15_67420434 | 15 | 67420434 | 16 | C | T | T | 0.64516 | C | 0.35484 | 12 | 0.3871 |
| 12757 | S15_67420497 | 15 | 67420497 | 63 | A | G | A | 0.82258 | G | 0.17742 | 9 | 0.29032 |
| 12758 | S15_67470488 | 15 | 67470488 | 49991 | C | T | T | 0.51613 | C | 0.48387 | 18 | 0.58065 |
| 12759 | S15_67605482 | 15 | 67605482 | 134994 | A | G | G | 0.67742 | A | 0.32258 | 16 | 0.51613 |
| 12760 | S15_68033736 | 15 | 68033736 | 428254 | C | A | C | 0.90323 | A | 0.09677 | 6 | 0.19355 |
| 12761 | S15_68115769 | 15 | 68115769 | 82033 | G | A | G | 0.95161 | A | 0.04839 | 3 | 0.09677 |
| 12762 | S15_68173268 | 15 | 68173268 | 57499 | C | T | C | 0.59677 | T | 0.40323 | 15 | 0.48387 |
| 12763 | S15_68315886 | 15 | 68315886 | 142618 | C | G | C | 0.91935 | G | 0.08065 | 3 | 0.09677 |
| 12764 | S15_68393497 | 15 | 68393497 | 77611 | G | A | G | 0.87097 | A | 0.12903 | 8 | 0.25806 |
| 12765 | S15_68393543 | 15 | 68393543 | 46 | C | T | C | 0.62903 | T | 0.37097 | 15 | 0.48387 |
| 12766 | S15_69833245 | 15 | 69833245 | 1439702 | G | A | G | 0.8871 | A | 0.1129 | 7 | 0.22581 |
| 12767 | S15_70041544 | 15 | 70041544 | 208299 | G | A | G | 0.69355 | A | 0.30645 | 13 | 0.41935 |
| 12768 | S15_73572260 | 15 | 73572260 | 3530716 | A | G | G | 0.54839 | A | 0.45161 | 14 | 0.45161 |
| 12769 | S15_73572307 | 15 | 73572307 | 47 | T | G | G | 0.67742 | T | 0.32258 | 12 | 0.3871 |
| 12770 | S15_73572374 | 15 | 73572374 | 67 | C | T | T | 0.67742 | C | 0.32258 | 12 | 0.3871 |
| 12771 | S15_73600858 | 15 | 73600858 | 28484 | T | C | T | 0.80645 | C | 0.19355 | 10 | 0.32258 |
| 12772 | S15_73600865 | 15 | 73600865 | 7 | A | T | T | 0.67742 | A | 0.32258 | 16 | 0.51613 |
| 12773 | S15_73600910 | 15 | 73600910 | 45 | A | C | C | 0.95161 | A | 0.04839 | 3 | 0.09677 |
| 12774 | S15_73801229 | 15 | 73801229 | 200319 | G | C | G | 0.90323 | C | 0.09677 | 6 | 0.19355 |
| 12775 | S15_73801237 | 15 | 73801237 | 8 | T | C | C | 0.83871 | T | 0.16129 | 10 | 0.32258 |
| 12776 | S15_74420460 | 15 | 74420460 | 619223 | C | T | C | 0.75806 | T | 0.24194 | 13 | 0.41935 |
| 12777 | S15_74420513 | 15 | 74420513 | 53 | C | T | C | 0.80645 | T | 0.19355 | 10 | 0.32258 |
| 12778 | S15_75011882 | 15 | 75011882 | 591369 | C | T | C | 0.79032 | T | 0.20968 | 13 | 0.41935 |
| 12779 | S15_75011945 | 15 | 75011945 | 63 | G | A | G | 0.90323 | A | 0.09677 | 6 | 0.19355 |
| 12780 | S15_75011956 | 15 | 75011956 | 11 | C | T | C | 0.93548 | T | 0.06452 | 4 | 0.12903 |
| 12781 | S15_75779665 | 15 | 75779665 | 767709 | A | G | A | 0.8871 | G | 0.1129 | 5 | 0.16129 |
| 12782 | S15_75779706 | 15 | 75779706 | 41 | C | G | C | 0.90323 | G | 0.09677 | 4 | 0.12903 |
| 12783 | S15_75841836 | 15 | 75841836 | 62130 | A | T | A | 0.75806 | T | 0.24194 | 11 | 0.35484 |
| 12784 | S15_76896910 | 15 | 76896910 | 1055074 | G | A | A | 0.95161 | G | 0.04839 | 3 | 0.09677 |
| 12785 | S15_76897003 | 15 | 76897003 | 93 | T | C | C | 0.77419 | T | 0.22581 | 14 | 0.45161 |
| 12786 | S15_76897004 | 15 | 76897004 | 1 | G | A | G | 0.93548 | A | 0.06452 | 4 | 0.12903 |
| 12787 | S15_76897007 | 15 | 76897007 | 3 | C | T | C | 0.77419 | T | 0.22581 | 12 | 0.3871 |
| 12788 | S15_76897012 | 15 | 76897012 | 5 | A | G | A | 0.85484 | G | 0.14516 | 9 | 0.29032 |
| 12789 | S15_76897054 | 15 | 76897054 | 42 | T | G | T | 0.85484 | G | 0.14516 | 9 | 0.29032 |
| 12790 | S15_76897074 | 15 | 76897074 | 20 | T | C | T | 0.80645 | C | 0.19355 | 12 | 0.3871 |
| 12791 | S15_77048967 | 15 | 77048967 | 151893 | C | T | T | 0.66129 | C | 0.33871 | 11 | 0.35484 |
| 12792 | S15_77049056 | 15 | 77049056 | 89 | C | T | T | 0.66129 | C | 0.33871 | 11 | 0.35484 |
| 12793 | S15_77049092 | 15 | 77049092 | 36 | G | A | A | 0.91935 | G | 0.08065 | 5 | 0.16129 |
| 12794 | S15_77091898 | 15 | 77091898 | 42806 | G | A | G | 0.79032 | A | 0.20968 | 7 | 0.22581 |
| 12795 | S15_77091989 | 15 | 77091989 | 91 | A | C | A | 0.95161 | C | 0.04839 | 3 | 0.09677 |
| 12796 | S15_77092003 | 15 | 77092003 | 14 | C | T | C | 0.95161 | T | 0.04839 | 3 | 0.09677 |

| Site number | SNP name | Chromosome | Physical position | Physical distance from the previous SNP | Reference allele | Alternative allele | Major allele | Major allele frequency | Minor allele | Minor allele frequency | Number heterozygous | Heterozygosity |
|---|---|---|---|---|---|---|---|---|---|---|---|---|
| 12797 | S15_77092004 | 15 | 77092004 | 1 | A | T | A | 0.8871 | T | 0.1129 | 5 | 0.16129 |
| 12798 | S15_77848303 | 15 | 77848303 | 756299 | C | T | C | 0.91935 | T | 0.08065 | 5 | 0.16129 |
| 12799 | S15_77848529 | 15 | 77848529 | 226 | C | G | C | 0.8871 | G | 0.1129 | 7 | 0.22581 |
| 12800 | S15_78189261 | 15 | 78189261 | 340732 | C | T | C | 0.70968 | T | 0.29032 | 12 | 0.3871 |
| 12801 | S15_78189380 | 15 | 78189380 | 119 | C | G | C | 0.87097 | G | 0.12903 | 8 | 0.25806 |
| 12802 | S15_79399833 | 15 | 79399833 | 1210453 | T | C | T | 0.83871 | C | 0.16129 | 10 | 0.32258 |
| 12803 | S15_79399897 | 15 | 79399897 | 64 | A | G | G | 0.8871 | A | 0.1129 | 5 | 0.16129 |
| 12804 | S15_80171599 | 15 | 80171599 | 771702 | C | T | C | 0.77419 | T | 0.22581 | 12 | 0.3871 |
| 12805 | S15_80171612 | 15 | 80171612 | 13 | C | T | C | 0.69355 | T | 0.30645 | 9 | 0.29032 |
| 12806 | S15_80171626 | 15 | 80171626 | 14 | C | G | C | 0.70968 | G | 0.29032 | 8 | 0.25806 |
| 12807 | S15_80171652 | 15 | 80171652 | 26 | C | T | C | 0.83871 | T | 0.16129 | 8 | 0.25806 |
| 12808 | S15_80171731 | 15 | 80171731 | 79 | C | T | T | 0.74194 | C | 0.25806 | 12 | 0.3871 |
| 12809 | S15_80972496 | 15 | 80972496 | 800765 | G | T | G | 0.80645 | T | 0.19355 | 10 | 0.32258 |
| 12810 | S15_80972588 | 15 | 80972588 | 92 | G | A | G | 0.70968 | A | 0.29032 | 16 | 0.51613 |
| 12811 | S15_81264171 | 15 | 81264171 | 291583 | C | T | C | 0.93548 | T | 0.06452 | 4 | 0.12903 |
| 12812 | S15_81521975 | 15 | 81521975 | 257804 | A | T | A | 0.80645 | T | 0.19355 | 8 | 0.25806 |
| 12813 | S15_81522154 | 15 | 81522154 | 179 | T | C | T | 0.80645 | C | 0.19355 | 8 | 0.25806 |
| 12814 | S16_39221 | 16 | 39221 | 0 | G | A | G | 0.72581 | A | 0.27419 | 11 | 0.35484 |
| 12815 | S16_39233 | 16 | 39233 | 12 | T | C | T | 0.64516 | C | 0.35484 | 16 | 0.51613 |
| 12816 | S16_53649 | 16 | 53649 | 14416 | C | T | C | 0.87097 | T | 0.12903 | 8 | 0.25806 |
| 12817 | S16_790996 | 16 | 790996 | 737347 | C | T | C | 0.95161 | T | 0.04839 | 3 | 0.09677 |
| 12818 | S16_791050 | 16 | 791050 | 54 | G | A | G | 0.95161 | A | 0.04839 | 3 | 0.09677 |
| 12819 | S16_1416757 | 16 | 1416757 | 625707 | G | T | G | 0.69355 | T | 0.30645 | 13 | 0.41935 |
| 12820 | S16_1696927 | 16 | 1696927 | 280170 | T | A | T | 0.75806 | A | 0.24194 | 13 | 0.41935 |
| 12821 | S16_1914055 | 16 | 1914055 | 217128 | G | T | G | 0.93548 | T | 0.06452 | 2 | 0.06452 |
| 12822 | S16_1914065 | 16 | 1914065 | 10 | A | G | A | 0.83871 | G | 0.16129 | 6 | 0.19355 |
| 12823 | S16_2002668 | 16 | 2002668 | 88603 | G | A | G | 0.6129 | A | 0.3871 | 14 | 0.45161 |
| 12824 | S16_2059495 | 16 | 2059495 | 56827 | C | A | A | 0.87097 | C | 0.12903 | 4 | 0.12903 |
| 12825 | S16_2059606 | 16 | 2059606 | 111 | T | C | T | 0.79032 | C | 0.20968 | 13 | 0.41935 |
| 12826 | S16_2059659 | 16 | 2059659 | 53 | C | T | T | 0.59677 | C | 0.40323 | 9 | 0.29032 |
| 12827 | S16_2059687 | 16 | 2059687 | 28 | A | G | A | 0.90323 | G | 0.09677 | 6 | 0.19355 |
| 12828 | S16_2616082 | 16 | 2616082 | 556395 | C | T | C | 0.90323 | T | 0.09677 | 6 | 0.19355 |
| 12829 | S16_2616102 | 16 | 2616102 | 20 | A | T | A | 0.80645 | T | 0.19355 | 8 | 0.25806 |
| 12830 | S16_2616129 | 16 | 2616129 | 27 | T | C | T | 0.70968 | C | 0.29032 | 14 | 0.45161 |
| 12831 | S16_2616224 | 16 | 2616224 | 95 | C | T | C | 0.70968 | T | 0.29032 | 14 | 0.45161 |
| 12832 | S16_2616229 | 16 | 2616229 | 5 | G | A | G | 0.80645 | A | 0.19355 | 12 | 0.3871 |
| 12833 | S16_2746611 | 16 | 2746611 | 130382 | G | A | G | 0.93548 | A | 0.06452 | 4 | 0.12903 |
| 12834 | S16_2762118 | 16 | 2762118 | 15507 | G | C | G | 0.93548 | C | 0.06452 | 4 | 0.12903 |
| 12835 | S16_2762179 | 16 | 2762179 | 61 | C | T | C | 0.90323 | T | 0.09677 | 6 | 0.19355 |
| 12836 | S16_2913173 | 16 | 2913173 | 150994 | G | C | G | 0.87097 | C | 0.12903 | 6 | 0.19355 |
| 12837 | S16_2913197 | 16 | 2913197 | 24 | T | G | T | 0.90323 | G | 0.09677 | 6 | 0.19355 |
| 12838 | S16_2913198 | 16 | 2913198 | 1 | T | C | T | 0.90323 | C | 0.09677 | 6 | 0.19355 |
| 12839 | S16_3265138 | 16 | 3265138 | 351940 | G | T | T | 0.70968 | G | 0.29032 | 12 | 0.3871 |
| 12840 | S16_3265201 | 16 | 3265201 | 63 | G | A | G | 0.93548 | A | 0.06452 | 4 | 0.12903 |
| 12841 | S16_3265218 | 16 | 3265218 | 17 | C | T | T | 0.70968 | C | 0.29032 | 12 | 0.3871 |
| 12842 | S16_3358234 | 16 | 3358234 | 93016 | T | A | T | 0.87097 | A | 0.12903 | 8 | 0.25806 |
| 12843 | S16_4357352 | 16 | 4357352 | 999118 | G | A | G | 0.90323 | A | 0.09677 | 6 | 0.19355 |
| 12844 | S16_4357423 | 16 | 4357423 | 71 | T | C | T | 0.77419 | C | 0.22581 | 12 | 0.3871 |
| 12845 | S16_4357432 | 16 | 4357432 | 9 | G | A | G | 0.72581 | A | 0.27419 | 17 | 0.54839 |
| 12846 | S16_4357464 | 16 | 4357464 | 32 | T | C | T | 0.6129 | C | 0.3871 | 14 | 0.45161 |
| 12847 | S16_4357530 | 16 | 4357530 | 66 | A | G | G | 0.72581 | A | 0.27419 | 15 | 0.48387 |
| 12848 | S16_4357545 | 16 | 4357545 | 15 | T | G | G | 0.72581 | T | 0.27419 | 15 | 0.48387 |
| 12849 | S16_4364064 | 16 | 4364064 | 6519 | G | T | G | 0.8871 | T | 0.1129 | 5 | 0.16129 |
| 12850 | S16_4364291 | 16 | 4364291 | 227 | C | G | C | 0.70968 | G | 0.29032 | 14 | 0.45161 |

| Site number | SNP name | Chromosome | Physical position | Physical distance from the previous SNP | Reference allele | Alternative allele | Major allele | Major allele frequency | Minor allele | Minor allele frequency | Number heterozygous | Heterozygosity |
|---|---|---|---|---|---|---|---|---|---|---|---|---|
| 12851 | S16_4364317 | 16 | 4364317 | 26 | T | G | T | 0.54839 | G | 0.45161 | 20 | 0.64516 |
| 12852 | S16_4691221 | 16 | 4691221 | 326904 | C | T | C | 0.90323 | T | 0.09677 | 4 | 0.12903 |
| 12853 | S16_4691322 | 16 | 4691322 | 101 | G | A | G | 0.82258 | A | 0.17742 | 7 | 0.22581 |
| 12854 | S16_5194022 | 16 | 5194022 | 502700 | G | A | G | 0.83871 | A | 0.16129 | 10 | 0.32258 |
| 12855 | S16_5194032 | 16 | 5194032 | 10 | C | T | C | 0.83871 | T | 0.16129 | 10 | 0.32258 |
| 12856 | S16_5194033 | 16 | 5194033 | 1 | A | G | A | 0.83871 | G | 0.16129 | 10 | 0.32258 |
| 12857 | S16_5194039 | 16 | 5194039 | 6 | G | A | G | 0.83871 | A | 0.16129 | 10 | 0.32258 |
| 12858 | S16_5194057 | 16 | 5194057 | 18 | C | T | C | 0.93548 | T | 0.06452 | 4 | 0.12903 |
| 12859 | S16_5194067 | 16 | 5194067 | 10 | T | G | T | 0.83871 | G | 0.16129 | 10 | 0.32258 |
| 12860 | S16_5194077 | 16 | 5194077 | 10 | G | T | G | 0.83871 | T | 0.16129 | 10 | 0.32258 |
| 12861 | S16_5194158 | 16 | 5194158 | 81 | T | A | A | 0.83871 | T | 0.16129 | 10 | 0.32258 |
| 12862 | S16_5194229 | 16 | 5194229 | 71 | A | C | A | 0.87097 | C | 0.12903 | 8 | 0.25806 |
| 12863 | S16_5194247 | 16 | 5194247 | 18 | G | A | G | 0.83871 | A | 0.16129 | 10 | 0.32258 |
| 12864 | S16_5273166 | 16 | 5273166 | 78919 | A | G | A | 0.91935 | G | 0.08065 | 5 | 0.16129 |
| 12865 | S16_5273286 | 16 | 5273286 | 120 | C | T | C | 0.74194 | T | 0.25806 | 16 | 0.51613 |
| 12866 | S16_5273290 | 16 | 5273290 | 4 | A | G | A | 0.74194 | G | 0.25806 | 16 | 0.51613 |
| 12867 | S16_5273366 | 16 | 5273366 | 76 | C | A | C | 0.93548 | A | 0.06452 | 4 | 0.12903 |
| 12868 | S16_5298817 | 16 | 5298817 | 25451 | C | G | C | 0.93548 | G | 0.06452 | 4 | 0.12903 |
| 12869 | S16_5298827 | 16 | 5298827 | 10 | G | A | G | 0.93548 | A | 0.06452 | 4 | 0.12903 |
| 12870 | S16_5477713 | 16 | 5477713 | 178886 | T | A | T | 0.8871 | A | 0.1129 | 7 | 0.22581 |
| 12871 | S16_5477862 | 16 | 5477862 | 149 | T | C | T | 0.8871 | C | 0.1129 | 7 | 0.22581 |
| 12872 | S16_5555820 | 16 | 5555820 | 77958 | G | A | G | 0.77419 | A | 0.22581 | 10 | 0.32258 |
| 12873 | S16_5555840 | 16 | 5555840 | 20 | C | T | C | 0.79032 | T | 0.20968 | 13 | 0.41935 |
| 12874 | S16_5555841 | 16 | 5555841 | 1 | G | A | G | 0.87097 | A | 0.12903 | 8 | 0.25806 |
| 12875 | S16_5889219 | 16 | 5889219 | 333378 | C | A | C | 0.90323 | A | 0.09677 | 6 | 0.19355 |
| 12876 | S16_5889460 | 16 | 5889460 | 241 | A | G | A | 0.54839 | G | 0.45161 | 12 | 0.3871 |
| 12877 | S16_6305275 | 16 | 6305275 | 415815 | A | G | A | 0.85484 | G | 0.14516 | 5 | 0.16129 |
| 12878 | S16_6305325 | 16 | 6305325 | 50 | G | A | G | 0.8871 | A | 0.1129 | 7 | 0.22581 |
| 12879 | S16_6305347 | 16 | 6305347 | 22 | C | T | C | 0.8871 | T | 0.1129 | 7 | 0.22581 |
| 12880 | S16_6305365 | 16 | 6305365 | 18 | T | C | T | 0.95161 | C | 0.04839 | 3 | 0.09677 |
| 12881 | S16_6305468 | 16 | 6305468 | 103 | C | T | C | 0.83871 | T | 0.16129 | 10 | 0.32258 |
| 12882 | S16_6632177 | 16 | 6632177 | 326709 | C | G | C | 0.91935 | G | 0.08065 | 5 | 0.16129 |
| 12883 | S16_6632186 | 16 | 6632186 | 9 | T | G | G | 0.59677 | T | 0.40323 | 13 | 0.41935 |
| 12884 | S16_6632228 | 16 | 6632228 | 42 | G | T | T | 0.51613 | G | 0.48387 | 18 | 0.58065 |
| 12885 | S16_6632242 | 16 | 6632242 | 14 | G | C | C | 0.59677 | G | 0.40323 | 13 | 0.41935 |
| 12886 | S16_6632250 | 16 | 6632250 | 8 | T | G | G | 0.59677 | T | 0.40323 | 13 | 0.41935 |
| 12887 | S16_6632340 | 16 | 6632340 | 90 | G | C | G | 0.91935 | C | 0.08065 | 5 | 0.16129 |
| 12888 | S16_6711882 | 16 | 6711882 | 79542 | T | C | T | 0.67742 | C | 0.32258 | 14 | 0.45161 |
| 12889 | S16_6711974 | 16 | 6711974 | 92 | C | G | C | 0.74194 | G | 0.25806 | 10 | 0.32258 |
| 12890 | S16_6711983 | 16 | 6711983 | 9 | C | T | T | 0.59677 | C | 0.40323 | 19 | 0.6129 |
| 12891 | S16_6712042 | 16 | 6712042 | 59 | A | G | A | 0.74194 | G | 0.25806 | 10 | 0.32258 |
| 12892 | S16_7120823 | 16 | 7120823 | 408781 | A | C | A | 0.70968 | C | 0.29032 | 8 | 0.25806 |
| 12893 | S16_7120902 | 16 | 7120902 | 79 | A | T | A | 0.83871 | T | 0.16129 | 6 | 0.19355 |
| 12894 | S16_7120958 | 16 | 7120958 | 56 | A | G | A | 0.53226 | G | 0.46774 | 13 | 0.41935 |
| 12895 | S16_7121005 | 16 | 7121005 | 47 | C | A | C | 0.91935 | A | 0.08065 | 5 | 0.16129 |
| 12896 | S16_7847012 | 16 | 7847012 | 726007 | C | T | C | 0.87097 | T | 0.12903 | 8 | 0.25806 |
| 12897 | S16_7847071 | 16 | 7847071 | 59 | A | G | G | 0.53226 | A | 0.46774 | 13 | 0.41935 |
| 12898 | S16_8504737 | 16 | 8504737 | 657666 | C | T | C | 0.95161 | T | 0.04839 | 3 | 0.09677 |
| 12899 | S16_8504784 | 16 | 8504784 | 47 | G | A | G | 0.95161 | A | 0.04839 | 3 | 0.09677 |
| 12900 | S16_8504885 | 16 | 8504885 | 101 | G | A | G | 0.95161 | A | 0.04839 | 3 | 0.09677 |
| 12901 | S16_8990295 | 16 | 8990295 | 485410 | C | A | C | 0.80645 | A | 0.19355 | 6 | 0.19355 |
| 12902 | S16_8990311 | 16 | 8990311 | 16 | G | T | G | 0.90323 | T | 0.09677 | 6 | 0.19355 |
| 12903 | S16_8990405 | 16 | 8990405 | 94 | G | T | G | 0.85484 | T | 0.14516 | 7 | 0.22581 |
| 12904 | S16_8990478 | 16 | 8990478 | 73 | C | A | C | 0.51613 | A | 0.48387 | 18 | 0.58065 |

| Site number | SNP name | Chromosome | Physical position | Physical distance from the previous SNP | Reference allele | Alternative allele | Major allele | Major allele frequency | Minor allele | Minor allele frequency | Number heterozygous | Heterozygosity |
|---|---|---|---|---|---|---|---|---|---|---|---|---|
| 12905 | S16_8990481 | 16 | 8990481 | 3 | G | A | G | 0.74194 | A | 0.25806 | 12 | 0.3871 |
| 12906 | S16_9180328 | 16 | 9180328 | 189847 | C | A | C | 0.79032 | A | 0.20968 | 13 | 0.41935 |
| 12907 | S16_9180333 | 16 | 9180333 | 5 | A | C | A | 0.93548 | C | 0.06452 | 4 | 0.12903 |
| 12908 | S16_9180390 | 16 | 9180390 | 57 | C | A | C | 0.74194 | A | 0.25806 | 10 | 0.32258 |
| 12909 | S16_9180405 | 16 | 9180405 | 15 | G | T | G | 0.90323 | T | 0.09677 | 6 | 0.19355 |
| 12910 | S16_9180472 | 16 | 9180472 | 67 | T | C | T | 0.80645 | C | 0.19355 | 12 | 0.3871 |
| 12911 | S16_9180504 | 16 | 9180504 | 32 | G | T | G | 0.93548 | T | 0.06452 | 4 | 0.12903 |
| 12912 | S16_9642517 | 16 | 9642517 | 462013 | C | T | C | 0.87097 | T | 0.12903 | 4 | 0.12903 |
| 12913 | S16_11244176 | 16 | 11244176 | 1601659 | C | T | C | 0.93548 | T | 0.06452 | 4 | 0.12903 |
| 12914 | S16_11244215 | 16 | 11244215 | 39 | G | T | G | 0.77419 | T | 0.22581 | 12 | 0.3871 |
| 12915 | S16_11355587 | 16 | 11355587 | 111372 | T | C | C | 0.91935 | T | 0.08065 | 5 | 0.16129 |
| 12916 | S16_11514900 | 16 | 11514900 | 159313 | C | T | C | 0.91935 | T | 0.08065 | 5 | 0.16129 |
| 12917 | S16_11719897 | 16 | 11719897 | 204997 | T | C | T | 0.83871 | C | 0.16129 | 8 | 0.25806 |
| 12918 | S16_11719921 | 16 | 11719921 | 24 | G | A | G | 0.83871 | A | 0.16129 | 8 | 0.25806 |
| 12919 | S16_14119236 | 16 | 14119236 | 2399315 | A | T | A | 0.74194 | T | 0.25806 | 10 | 0.32258 |
| 12920 | S16_14119244 | 16 | 14119244 | 8 | G | A | G | 0.82258 | A | 0.17742 | 7 | 0.22581 |
| 12921 | S16_14512514 | 16 | 14512514 | 393270 | T | C | T | 0.85484 | C | 0.14516 | 9 | 0.29032 |
| 12922 | S16_14512604 | 16 | 14512604 | 90 | C | G | C | 0.85484 | G | 0.14516 | 9 | 0.29032 |
| 12923 | S16_14512607 | 16 | 14512607 | 3 | C | T | C | 0.72581 | T | 0.27419 | 13 | 0.41935 |
| 12924 | S16_14512720 | 16 | 14512720 | 113 | T | C | T | 0.85484 | C | 0.14516 | 9 | 0.29032 |
| 12925 | S16_16347424 | 16 | 16347424 | 1834704 | C | T | C | 0.69355 | T | 0.30645 | 17 | 0.54839 |
| 12926 | S16_16840093 | 16 | 16840093 | 492669 | T | C | C | 0.83871 | T | 0.16129 | 8 | 0.25806 |
| 12927 | S16_16840116 | 16 | 16840116 | 23 | T | C | T | 0.75806 | C | 0.24194 | 9 | 0.29032 |
| 12928 | S16_16840133 | 16 | 16840133 | 17 | C | T | C | 0.77419 | T | 0.22581 | 8 | 0.25806 |
| 12929 | S16_16840150 | 16 | 16840150 | 17 | G | A | G | 0.75806 | A | 0.24194 | 9 | 0.29032 |
| 12930 | S16_16840215 | 16 | 16840215 | 65 | C | G | C | 0.77419 | G | 0.22581 | 8 | 0.25806 |
| 12931 | S16_16840274 | 16 | 16840274 | 59 | C | G | C | 0.77419 | G | 0.22581 | 8 | 0.25806 |
| 12932 | S16_16840293 | 16 | 16840293 | 19 | G | T | G | 0.77419 | T | 0.22581 | 8 | 0.25806 |
| 12933 | S16_17446501 | 16 | 17446501 | 606208 | G | A | G | 0.79032 | A | 0.20968 | 11 | 0.35484 |
| 12934 | S16_17446525 | 16 | 17446525 | 24 | C | T | C | 0.82258 | T | 0.17742 | 11 | 0.35484 |
| 12935 | S16_17446554 | 16 | 17446554 | 29 | G | A | G | 0.85484 | A | 0.14516 | 7 | 0.22581 |
| 12936 | S16_17453265 | 16 | 17453265 | 6711 | G | A | G | 0.83871 | A | 0.16129 | 8 | 0.25806 |
| 12937 | S16_17453286 | 16 | 17453286 | 21 | C | T | T | 0.66129 | C | 0.33871 | 13 | 0.41935 |
| 12938 | S16_17453339 | 16 | 17453339 | 53 | A | G | A | 0.54839 | G | 0.45161 | 16 | 0.51613 |
| 12939 | S16_17453374 | 16 | 17453374 | 35 | T | C | T | 0.54839 | C | 0.45161 | 16 | 0.51613 |
| 12940 | S16_17647969 | 16 | 17647969 | 194595 | C | T | C | 0.95161 | T | 0.04839 | 3 | 0.09677 |
| 12941 | S16_17647970 | 16 | 17647970 | 1 | G | A | G | 0.90323 | A | 0.09677 | 6 | 0.19355 |
| 12942 | S16_17647990 | 16 | 17647990 | 20 | C | T | C | 0.90323 | T | 0.09677 | 6 | 0.19355 |
| 12943 | S16_17648091 | 16 | 17648091 | 101 | G | A | G | 0.85484 | A | 0.14516 | 7 | 0.22581 |
| 12944 | S16_17648160 | 16 | 17648160 | 69 | G | A | G | 0.83871 | A | 0.16129 | 6 | 0.19355 |
| 12945 | S16_17648163 | 16 | 17648163 | 3 | A | G | A | 0.79032 | G | 0.20968 | 9 | 0.29032 |
| 12946 | S16_17648189 | 16 | 17648189 | 26 | T | C | T | 0.79032 | C | 0.20968 | 13 | 0.41935 |
| 12947 | S16_17651824 | 16 | 17651824 | 3635 | G | C | G | 0.80645 | C | 0.19355 | 12 | 0.3871 |
| 12948 | S16_17706718 | 16 | 17706718 | 54894 | C | G | G | 0.87097 | C | 0.12903 | 6 | 0.19355 |
| 12949 | S16_17706786 | 16 | 17706786 | 68 | T | C | T | 0.91935 | C | 0.08065 | 5 | 0.16129 |
| 12950 | S16_17935165 | 16 | 17935165 | 228379 | C | T | C | 0.93548 | T | 0.06452 | 4 | 0.12903 |
| 12951 | S16_17935196 | 16 | 17935196 | 31 | T | C | T | 0.77419 | C | 0.22581 | 12 | 0.3871 |
| 12952 | S16_18560265 | 16 | 18560265 | 625069 | T | C | C | 0.77419 | T | 0.22581 | 10 | 0.32258 |
| 12953 | S16_18670413 | 16 | 18670413 | 110148 | C | T | T | 0.91935 | C | 0.08065 | 5 | 0.16129 |
| 12954 | S16_18703159 | 16 | 18703159 | 32746 | A | G | A | 0.77419 | G | 0.22581 | 10 | 0.32258 |
| 12955 | S16_18703212 | 16 | 18703212 | 53 | T | C | T | 0.75806 | C | 0.24194 | 11 | 0.35484 |
| 12956 | S16_18703283 | 16 | 18703283 | 71 | C | A | C | 0.69355 | A | 0.30645 | 17 | 0.54839 |
| 12957 | S16_18831244 | 16 | 18831244 | 127961 | T | A | T | 0.8871 | A | 0.1129 | 7 | 0.22581 |
| 12958 | S16_18940904 | 16 | 18940904 | 109660 | C | T | C | 0.8871 | T | 0.1129 | 7 | 0.22581 |

| Site number | SNP name | Chromosome | Physical position | Physical distance from the previous SNP | Reference allele | Alternative allele | Major allele | Major allele frequency | Minor allele | Minor allele frequency | Number heterozygous | Heterozygosity |
|---|---|---|---|---|---|---|---|---|---|---|---|---|
| 12959 | S16_18941107 | 16 | 18941107 | 203 | C | T | C | 0.91935 | T | 0.08065 | 5 | 0.16129 |
| 12960 | S16_19107331 | 16 | 19107331 | 166224 | T | A | T | 0.95161 | A | 0.04839 | 3 | 0.09677 |
| 12961 | S16_19107407 | 16 | 19107407 | 76 | C | T | C | 0.83871 | T | 0.16129 | 6 | 0.19355 |
| 12962 | S16_19156959 | 16 | 19156959 | 49552 | G | A | G | 0.90323 | A | 0.09677 | 6 | 0.19355 |
| 12963 | S16_19156980 | 16 | 19156980 | 21 | C | T | C | 0.95161 | T | 0.04839 | 3 | 0.09677 |
| 12964 | S16_19156997 | 16 | 19156997 | 17 | G | A | G | 0.77419 | A | 0.22581 | 10 | 0.32258 |
| 12965 | S16_19156998 | 16 | 19156998 | 1 | C | A | C | 0.77419 | A | 0.22581 | 10 | 0.32258 |
| 12966 | S16_19157061 | 16 | 19157061 | 63 | C | T | T | 0.59677 | C | 0.40323 | 9 | 0.29032 |
| 12967 | S16_19157131 | 16 | 19157131 | 70 | C | T | C | 0.90323 | T | 0.09677 | 6 | 0.19355 |
| 12968 | S16_19157163 | 16 | 19157163 | 32 | C | A | C | 0.66129 | A | 0.33871 | 9 | 0.29032 |
| 12969 | S16_19232330 | 16 | 19232330 | 75167 | A | G | G | 0.79032 | A | 0.20968 | 9 | 0.29032 |
| 12970 | S16_20457455 | 16 | 20457455 | 1225125 | G | A | G | 0.75806 | A | 0.24194 | 11 | 0.35484 |
| 12971 | S16_20629307 | 16 | 20629307 | 171852 | G | A | G | 0.83871 | A | 0.16129 | 10 | 0.32258 |
| 12972 | S16_20629329 | 16 | 20629329 | 22 | G | T | G | 0.74194 | T | 0.25806 | 14 | 0.45161 |
| 12973 | S16_20629353 | 16 | 20629353 | 24 | G | A | G | 0.79032 | A | 0.20968 | 7 | 0.22581 |
| 12974 | S16_20679229 | 16 | 20679229 | 49876 | G | A | G | 0.64516 | A | 0.35484 | 16 | 0.51613 |
| 12975 | S16_20737508 | 16 | 20737508 | 58279 | G | A | G | 0.70968 | A | 0.29032 | 14 | 0.45161 |
| 12976 | S16_20737713 | 16 | 20737713 | 205 | T | A | T | 0.85484 | A | 0.14516 | 5 | 0.16129 |
| 12977 | S16_20891994 | 16 | 20891994 | 154281 | A | T | A | 0.79032 | T | 0.20968 | 7 | 0.22581 |
| 12978 | S16_20900230 | 16 | 20900230 | 8236 | A | G | A | 0.91935 | G | 0.08065 | 5 | 0.16129 |
| 12979 | S16_20900260 | 16 | 20900260 | 30 | G | A | A | 0.69355 | G | 0.30645 | 11 | 0.35484 |
| 12980 | S16_20900319 | 16 | 20900319 | 59 | G | A | G | 0.93548 | A | 0.06452 | 4 | 0.12903 |
| 12981 | S16_20900333 | 16 | 20900333 | 14 | G | C | C | 0.69355 | G | 0.30645 | 11 | 0.35484 |
| 12982 | S16_20983839 | 16 | 20983839 | 83506 | T | G | T | 0.83871 | G | 0.16129 | 6 | 0.19355 |
| 12983 | S16_20983984 | 16 | 20983984 | 145 | C | T | C | 0.95161 | T | 0.04839 | 3 | 0.09677 |
| 12984 | S16_21202273 | 16 | 21202273 | 218289 | C | G | C | 0.91935 | G | 0.08065 | 5 | 0.16129 |
| 12985 | S16_21202391 | 16 | 21202391 | 118 | C | T | C | 0.93548 | T | 0.06452 | 2 | 0.06452 |
| 12986 | S16_21284906 | 16 | 21284906 | 82515 | G | A | G | 0.95161 | A | 0.04839 | 3 | 0.09677 |
| 12987 | S16_21294511 | 16 | 21294511 | 9605 | A | G | A | 0.90323 | G | 0.09677 | 4 | 0.12903 |
| 12988 | S16_21932537 | 16 | 21932537 | 638026 | T | C | C | 0.91935 | T | 0.08065 | 5 | 0.16129 |
| 12989 | S16_21932598 | 16 | 21932598 | 61 | T | C | T | 0.90323 | C | 0.09677 | 6 | 0.19355 |
| 12990 | S16_22211867 | 16 | 22211867 | 279269 | A | G | A | 0.93548 | G | 0.06452 | 4 | 0.12903 |
| 12991 | S16_22591652 | 16 | 22591652 | 379785 | C | G | C | 0.74194 | G | 0.25806 | 12 | 0.3871 |
| 12992 | S16_22591730 | 16 | 22591730 | 78 | G | A | G | 0.74194 | A | 0.25806 | 12 | 0.3871 |
| 12993 | S16_22591803 | 16 | 22591803 | 73 | G | A | A | 0.91935 | G | 0.08065 | 3 | 0.09677 |
| 12994 | S16_22591812 | 16 | 22591812 | 9 | T | C | T | 0.95161 | C | 0.04839 | 3 | 0.09677 |
| 12995 | S16_22775142 | 16 | 22775142 | 183330 | G | A | G | 0.8871 | A | 0.1129 | 5 | 0.16129 |
| 12996 | S16_22775158 | 16 | 22775158 | 16 | C | T | C | 0.56452 | T | 0.43548 | 11 | 0.35484 |
| 12997 | S16_22775166 | 16 | 22775166 | 8 | G | A | G | 0.56452 | A | 0.43548 | 11 | 0.35484 |
| 12998 | S16_22775218 | 16 | 22775218 | 52 | A | G | A | 0.62903 | G | 0.37097 | 13 | 0.41935 |
| 12999 | S16_22775232 | 16 | 22775232 | 14 | C | A | C | 0.87097 | A | 0.12903 | 6 | 0.19355 |
| 13000 | S16_22806786 | 16 | 22806786 | 31554 | C | G | C | 0.58065 | G | 0.41935 | 16 | 0.51613 |
| 13001 | S16_22837223 | 16 | 22837223 | 30437 | G | A | G | 0.6129 | A | 0.3871 | 12 | 0.3871 |
| 13002 | S16_22837243 | 16 | 22837243 | 20 | G | A | G | 0.69355 | A | 0.30645 | 11 | 0.35484 |
| 13003 | S16_22837358 | 16 | 22837358 | 115 | A | G | A | 0.95161 | G | 0.04839 | 3 | 0.09677 |
| 13004 | S16_23240675 | 16 | 23240675 | 403317 | G | A | G | 0.8871 | A | 0.1129 | 5 | 0.16129 |
| 13005 | S16_23240692 | 16 | 23240692 | 17 | T | C | T | 0.85484 | C | 0.14516 | 7 | 0.22581 |
| 13006 | S16_23240717 | 16 | 23240717 | 25 | G | T | G | 0.82258 | T | 0.17742 | 9 | 0.29032 |
| 13007 | S16_23261834 | 16 | 23261834 | 21117 | C | T | C | 0.90323 | T | 0.09677 | 4 | 0.12903 |
| 13008 | S16_23521831 | 16 | 23521831 | 259997 | G | A | G | 0.54839 | A | 0.45161 | 18 | 0.58065 |
| 13009 | S16_23860324 | 16 | 23860324 | 338493 | T | C | T | 0.93548 | C | 0.06452 | 4 | 0.12903 |
| 13010 | S16_23860478 | 16 | 23860478 | 154 | C | T | C | 0.72581 | T | 0.27419 | 9 | 0.29032 |
| 13011 | S16_24181224 | 16 | 24181224 | 320746 | T | C | C | 0.77419 | T | 0.22581 | 10 | 0.32258 |
| 13012 | S16_24445806 | 16 | 24445806 | 264582 | C | G | G | 0.59677 | C | 0.40323 | 11 | 0.35484 |

| Site number | SNP name | Chromosome | Physical position | Physical distance from the previous SNP | Reference allele | Alternative allele | Major allele | Major allele frequency | Minor allele | Minor allele frequency | Number heterozygous | Heterozygosity |
|---|---|---|---|---|---|---|---|---|---|---|---|---|
| 13013 | S16_24445980 | 16 | 24445980 | 174 | G | T | G | 0.59677 | T | 0.40323 | 13 | 0.41935 |
| 13014 | S16_24446021 | 16 | 24446021 | 41 | T | G | T | 0.62903 | G | 0.37097 | 15 | 0.48387 |
| 13015 | S16_24459809 | 16 | 24459809 | 13788 | G | A | G | 0.85484 | A | 0.14516 | 9 | 0.29032 |
| 13016 | S16_24459816 | 16 | 24459816 | 7 | G | A | G | 0.85484 | A | 0.14516 | 9 | 0.29032 |
| 13017 | S16_24459835 | 16 | 24459835 | 19 | G | A | G | 0.91935 | A | 0.08065 | 5 | 0.16129 |
| 13018 | S16_24459885 | 16 | 24459885 | 50 | A | G | A | 0.51613 | G | 0.48387 | 14 | 0.45161 |
| 13019 | S16_24550518 | 16 | 24550518 | 90633 | G | C | G | 0.82258 | C | 0.17742 | 9 | 0.29032 |
| 13020 | S16_24550591 | 16 | 24550591 | 73 | G | A | G | 0.80645 | A | 0.19355 | 10 | 0.32258 |
| 13021 | S16_24579180 | 16 | 24579180 | 28589 | G | A | A | 0.5 | G | 0.5 | 17 | 0.54839 |
| 13022 | S16_24752808 | 16 | 24752808 | 173628 | C | G | C | 0.90323 | G | 0.09677 | 6 | 0.19355 |
| 13023 | S16_24752821 | 16 | 24752821 | 13 | A | G | G | 0.85484 | A | 0.14516 | 7 | 0.22581 |
| 13024 | S16_24851590 | 16 | 24851590 | 98769 | C | T | C | 0.93548 | T | 0.06452 | 4 | 0.12903 |
| 13025 | S16_24851622 | 16 | 24851622 | 32 | G | A | A | 0.58065 | G | 0.41935 | 14 | 0.45161 |
| 13026 | S16_24945326 | 16 | 24945326 | 93704 | G | A | G | 0.82258 | A | 0.17742 | 9 | 0.29032 |
| 13027 | S16_24945393 | 16 | 24945393 | 67 | A | C | A | 0.93548 | C | 0.06452 | 4 | 0.12903 |
| 13028 | S16_25382158 | 16 | 25382158 | 436765 | G | T | T | 0.70968 | G | 0.29032 | 14 | 0.45161 |
| 13029 | S16_25608567 | 16 | 25608567 | 226409 | C | T | C | 0.87097 | T | 0.12903 | 8 | 0.25806 |
| 13030 | S16_25608681 | 16 | 25608681 | 114 | G | A | G | 0.87097 | A | 0.12903 | 8 | 0.25806 |
| 13031 | S16_25890447 | 16 | 25890447 | 281766 | A | G | A | 0.90323 | G | 0.09677 | 6 | 0.19355 |
| 13032 | S16_26232915 | 16 | 26232915 | 342468 | C | G | C | 0.93548 | G | 0.06452 | 4 | 0.12903 |
| 13033 | S16_26319213 | 16 | 26319213 | 86298 | A | G | A | 0.85484 | G | 0.14516 | 7 | 0.22581 |
| 13034 | S16_26319266 | 16 | 26319266 | 53 | G | A | G | 0.91935 | A | 0.08065 | 5 | 0.16129 |
| 13035 | S16_26319287 | 16 | 26319287 | 21 | C | T | T | 0.75806 | C | 0.24194 | 13 | 0.41935 |
| 13036 | S16_26333519 | 16 | 26333519 | 14232 | G | T | T | 0.59677 | G | 0.40323 | 17 | 0.54839 |
| 13037 | S16_26444988 | 16 | 26444988 | 111469 | G | A | G | 0.95161 | A | 0.04839 | 3 | 0.09677 |
| 13038 | S16_26873987 | 16 | 26873987 | 428999 | C | T | T | 0.95161 | C | 0.04839 | 3 | 0.09677 |
| 13039 | S16_26919295 | 16 | 26919295 | 45308 | C | A | C | 0.64516 | A | 0.35484 | 14 | 0.45161 |
| 13040 | S16_26919479 | 16 | 26919479 | 184 | A | G | A | 0.66129 | G | 0.33871 | 13 | 0.41935 |
| 13041 | S16_26919512 | 16 | 26919512 | 33 | G | A | G | 0.64516 | A | 0.35484 | 14 | 0.45161 |
| 13042 | S16_27632933 | 16 | 27632933 | 713421 | A | G | G | 0.53226 | A | 0.46774 | 15 | 0.48387 |
| 13043 | S16_27953095 | 16 | 27953095 | 320162 | G | A | A | 0.6129 | G | 0.3871 | 10 | 0.32258 |
| 13044 | S16_27953214 | 16 | 27953214 | 119 | C | A | C | 0.6129 | A | 0.3871 | 10 | 0.32258 |
| 13045 | S16_27953228 | 16 | 27953228 | 14 | G | A | G | 0.6129 | A | 0.3871 | 10 | 0.32258 |
| 13046 | S16_27953231 | 16 | 27953231 | 3 | T | C | C | 0.53226 | T | 0.46774 | 13 | 0.41935 |
| 13047 | S16_27953261 | 16 | 27953261 | 30 | A | C | A | 0.6129 | C | 0.3871 | 10 | 0.32258 |
| 13048 | S16_27953262 | 16 | 27953262 | 1 | T | C | T | 0.6129 | C | 0.3871 | 10 | 0.32258 |
| 13049 | S16_27953313 | 16 | 27953313 | 51 | T | C | T | 0.85484 | C | 0.14516 | 9 | 0.29032 |
| 13050 | S16_27985218 | 16 | 27985218 | 31905 | C | T | C | 0.87097 | T | 0.12903 | 8 | 0.25806 |
| 13051 | S16_28433405 | 16 | 28433405 | 448187 | T | C | T | 0.69355 | C | 0.30645 | 13 | 0.41935 |
| 13052 | S16_28433475 | 16 | 28433475 | 70 | A | G | A | 0.69355 | G | 0.30645 | 13 | 0.41935 |
| 13053 | S16_28857695 | 16 | 28857695 | 424220 | A | G | G | 0.53226 | A | 0.46774 | 13 | 0.41935 |
| 13054 | S16_29126709 | 16 | 29126709 | 269014 | C | T | C | 0.75806 | T | 0.24194 | 9 | 0.29032 |
| 13055 | S16_29126758 | 16 | 29126758 | 49 | A | T | A | 0.93548 | T | 0.06452 | 4 | 0.12903 |
| 13056 | S16_29525173 | 16 | 29525173 | 398415 | A | G | A | 0.70968 | G | 0.29032 | 12 | 0.3871 |
| 13057 | S16_29525276 | 16 | 29525276 | 103 | T | C | C | 0.51613 | T | 0.48387 | 20 | 0.64516 |
| 13058 | S16_29660725 | 16 | 29660725 | 135449 | C | A | C | 0.67742 | A | 0.32258 | 12 | 0.3871 |
| 13059 | S16_29660726 | 16 | 29660726 | 1 | A | G | A | 0.67742 | G | 0.32258 | 12 | 0.3871 |
| 13060 | S16_29660860 | 16 | 29660860 | 134 | T | C | C | 0.75806 | T | 0.24194 | 11 | 0.35484 |
| 13061 | S16_29660876 | 16 | 29660876 | 16 | T | C | T | 0.59677 | C | 0.40323 | 15 | 0.48387 |
| 13062 | S16_29717343 | 16 | 29717343 | 56467 | A | G | G | 0.95161 | A | 0.04839 | 3 | 0.09677 |
| 13063 | S16_29717361 | 16 | 29717361 | 18 | T | C | T | 0.93548 | C | 0.06452 | 4 | 0.12903 |
| 13064 | S16_29717538 | 16 | 29717538 | 177 | A | G | A | 0.82258 | G | 0.17742 | 7 | 0.22581 |
| 13065 | S16_29854892 | 16 | 29854892 | 137354 | G | A | G | 0.95161 | A | 0.04839 | 3 | 0.09677 |
| 13066 | S16_29855092 | 16 | 29855092 | 200 | T | C | C | 0.6129 | T | 0.3871 | 14 | 0.45161 |

| Site number | SNP name | Chromosome | Physical position | Physical distance from the previous SNP | Reference allele | Alternative allele | Major allele | Major allele frequency | Minor allele | Minor allele frequency | Number heterozygous | Heterozygosity |
|---|---|---|---|---|---|---|---|---|---|---|---|---|
| 13067 | S16_29855094 | 16 | 29855094 | 2 | C | T | C | 0.90323 | T | 0.09677 | 6 | 0.19355 |
| 13068 | S16_29906655 | 16 | 29906655 | 51561 | G | A | A | 0.77419 | G | 0.22581 | 10 | 0.32258 |
| 13069 | S16_29906665 | 16 | 29906665 | 10 | A | G | A | 0.91935 | G | 0.08065 | 5 | 0.16129 |
| 13070 | S16_30471345 | 16 | 30471345 | 564680 | A | T | A | 0.69355 | T | 0.30645 | 15 | 0.48387 |
| 13071 | S16_30471381 | 16 | 30471381 | 36 | G | A | G | 0.74194 | A | 0.25806 | 14 | 0.45161 |
| 13072 | S16_30471425 | 16 | 30471425 | 44 | C | T | C | 0.77419 | T | 0.22581 | 12 | 0.3871 |
| 13073 | S16_31040944 | 16 | 31040944 | 569519 | C | T | C | 0.91935 | T | 0.08065 | 5 | 0.16129 |
| 13074 | S16_31380228 | 16 | 31380228 | 339284 | C | T | C | 0.87097 | T | 0.12903 | 6 | 0.19355 |
| 13075 | S16_31380335 | 16 | 31380335 | 107 | C | T | C | 0.8871 | T | 0.1129 | 7 | 0.22581 |
| 13076 | S16_31380426 | 16 | 31380426 | 91 | A | C | A | 0.77419 | C | 0.22581 | 10 | 0.32258 |
| 13077 | S16_31380436 | 16 | 31380436 | 10 | C | G | C | 0.91935 | G | 0.08065 | 5 | 0.16129 |
| 13078 | S16_31505473 | 16 | 31505473 | 125037 | C | T | C | 0.79032 | T | 0.20968 | 11 | 0.35484 |
| 13079 | S16_31728613 | 16 | 31728613 | 223140 | G | A | G | 0.93548 | A | 0.06452 | 4 | 0.12903 |
| 13080 | S16_31728645 | 16 | 31728645 | 32 | T | C | T | 0.85484 | C | 0.14516 | 9 | 0.29032 |
| 13081 | S16_31748965 | 16 | 31748965 | 20320 | A | G | A | 0.64516 | G | 0.35484 | 16 | 0.51613 |
| 13082 | S16_31749030 | 16 | 31749030 | 65 | A | T | A | 0.79032 | T | 0.20968 | 9 | 0.29032 |
| 13083 | S16_31749041 | 16 | 31749041 | 11 | T | C | T | 0.79032 | C | 0.20968 | 9 | 0.29032 |
| 13084 | S16_32173706 | 16 | 32173706 | 424665 | T | G | T | 0.80645 | G | 0.19355 | 6 | 0.19355 |
| 13085 | S16_32185079 | 16 | 32185079 | 11373 | G | A | G | 0.8871 | A | 0.1129 | 7 | 0.22581 |
| 13086 | S16_32185089 | 16 | 32185089 | 10 | C | G | C | 0.90323 | G | 0.09677 | 4 | 0.12903 |
| 13087 | S16_32185201 | 16 | 32185201 | 112 | C | T | C | 0.87097 | T | 0.12903 | 8 | 0.25806 |
| 13088 | S16_32346454 | 16 | 32346454 | 161253 | G | T | G | 0.56452 | T | 0.43548 | 13 | 0.41935 |
| 13089 | S16_32346585 | 16 | 32346585 | 131 | T | C | C | 0.56452 | T | 0.43548 | 13 | 0.41935 |
| 13090 | S16_33301852 | 16 | 33301852 | 955267 | T | C | T | 0.87097 | C | 0.12903 | 8 | 0.25806 |
| 13091 | S16_33533049 | 16 | 33533049 | 231197 | T | C | C | 0.74194 | T | 0.25806 | 16 | 0.51613 |
| 13092 | S16_33672109 | 16 | 33672109 | 139060 | C | T | T | 0.62903 | C | 0.37097 | 15 | 0.48387 |
| 13093 | S16_33672130 | 16 | 33672130 | 21 | G | A | A | 0.64516 | G | 0.35484 | 14 | 0.45161 |
| 13094 | S16_34500984 | 16 | 34500984 | 828854 | T | C | T | 0.67742 | C | 0.32258 | 10 | 0.32258 |
| 13095 | S16_34678922 | 16 | 34678922 | 177938 | T | C | T | 0.8871 | C | 0.1129 | 5 | 0.16129 |
| 13096 | S16_34678950 | 16 | 34678950 | 28 | T | G | T | 0.8871 | G | 0.1129 | 5 | 0.16129 |
| 13097 | S16_34679013 | 16 | 34679013 | 63 | G | T | G | 0.66129 | T | 0.33871 | 13 | 0.41935 |
| 13098 | S16_35199898 | 16 | 35199898 | 520885 | A | G | G | 0.54839 | A | 0.45161 | 14 | 0.45161 |
| 13099 | S16_36489145 | 16 | 36489145 | 1289247 | G | T | G | 0.85484 | T | 0.14516 | 9 | 0.29032 |
| 13100 | S16_36489282 | 16 | 36489282 | 137 | T | C | T | 0.82258 | C | 0.17742 | 11 | 0.35484 |
| 13101 | S16_36489285 | 16 | 36489285 | 3 | T | C | T | 0.82258 | C | 0.17742 | 11 | 0.35484 |
| 13102 | S16_36489287 | 16 | 36489287 | 2 | T | C | T | 0.82258 | C | 0.17742 | 11 | 0.35484 |
| 13103 | S16_36489288 | 16 | 36489288 | 1 | T | C | T | 0.82258 | C | 0.17742 | 11 | 0.35484 |
| 13104 | S16_37234212 | 16 | 37234212 | 744924 | C | T | C | 0.79032 | T | 0.20968 | 9 | 0.29032 |
| 13105 | S16_37237883 | 16 | 37237883 | 3671 | T | C | T | 0.69355 | C | 0.30645 | 11 | 0.35484 |
| 13106 | S16_38029707 | 16 | 38029707 | 791824 | C | T | C | 0.91935 | T | 0.08065 | 5 | 0.16129 |
| 13107 | S16_38150568 | 16 | 38150568 | 120861 | C | T | C | 0.70968 | T | 0.29032 | 12 | 0.3871 |
| 13108 | S16_38687050 | 16 | 38687050 | 536482 | G | A | A | 0.72581 | G | 0.27419 | 15 | 0.48387 |
| 13109 | S16_38687158 | 16 | 38687158 | 108 | A | C | C | 0.72581 | A | 0.27419 | 15 | 0.48387 |
| 13110 | S16_38721002 | 16 | 38721002 | 33844 | T | C | T | 0.56452 | C | 0.43548 | 11 | 0.35484 |
| 13111 | S16_38721008 | 16 | 38721008 | 6 | C | T | C | 0.91935 | T | 0.08065 | 5 | 0.16129 |
| 13112 | S16_39704597 | 16 | 39704597 | 983589 | T | C | T | 0.85484 | C | 0.14516 | 7 | 0.22581 |
| 13113 | S16_39926789 | 16 | 39926789 | 222192 | A | C | A | 0.95161 | C | 0.04839 | 3 | 0.09677 |
| 13114 | S16_40165723 | 16 | 40165723 | 238934 | C | G | G | 0.87097 | C | 0.12903 | 6 | 0.19355 |
| 13115 | S16_40165768 | 16 | 40165768 | 45 | G | A | G | 0.93548 | A | 0.06452 | 4 | 0.12903 |
| 13116 | S16_40165850 | 16 | 40165850 | 82 | G | A | G | 0.90323 | A | 0.09677 | 4 | 0.12903 |
| 13117 | S16_40671920 | 16 | 40671920 | 506070 | T | C | T | 0.90323 | C | 0.09677 | 4 | 0.12903 |
| 13118 | S16_40729295 | 16 | 40729295 | 57375 | G | A | G | 0.85484 | A | 0.14516 | 9 | 0.29032 |
| 13119 | S16_40833043 | 16 | 40833043 | 103748 | A | T | A | 0.53226 | T | 0.46774 | 11 | 0.35484 |
| 13120 | S16_41221502 | 16 | 41221502 | 388459 | G | A | G | 0.93548 | A | 0.06452 | 4 | 0.12903 |

| Site number | SNP name | Chromosome | Physical position | Physical distance from the previous SNP | Reference allele | Alternative allele | Major allele | Major allele frequency | Minor allele | Minor allele frequency | Number heterozygous | Heterozygosity |
|---|---|---|---|---|---|---|---|---|---|---|---|---|
| 13121 | S16_41221914 | 16 | 41221914 | 412 | G | C | G | 0.93548 | C | 0.06452 | 4 | 0.12903 |
| 13122 | S16_41263195 | 16 | 41263195 | 41281 | T | C | C | 0.62903 | T | 0.37097 | 15 | 0.48387 |
| 13123 | S16_41263340 | 16 | 41263340 | 145 | C | T | T | 0.62903 | C | 0.37097 | 15 | 0.48387 |
| 13124 | S16_41360813 | 16 | 41360813 | 97473 | C | T | C | 0.85484 | T | 0.14516 | 7 | 0.22581 |
| 13125 | S16_41360870 | 16 | 41360870 | 57 | A | C | C | 0.83871 | A | 0.16129 | 8 | 0.25806 |
| 13126 | S16_41360970 | 16 | 41360970 | 100 | C | G | C | 0.91935 | G | 0.08065 | 5 | 0.16129 |
| 13127 | S16_41486122 | 16 | 41486122 | 125152 | T | C | C | 0.87097 | T | 0.12903 | 8 | 0.25806 |
| 13128 | S16_41486151 | 16 | 41486151 | 29 | C | T | C | 0.90323 | T | 0.09677 | 6 | 0.19355 |
| 13129 | S16_41617395 | 16 | 41617395 | 131244 | T | A | T | 0.90323 | A | 0.09677 | 6 | 0.19355 |
| 13130 | S16_41617396 | 16 | 41617396 | 1 | C | A | C | 0.90323 | A | 0.09677 | 6 | 0.19355 |
| 13131 | S16_41939811 | 16 | 41939811 | 322415 | A | G | G | 0.53226 | A | 0.46774 | 17 | 0.54839 |
| 13132 | S16_42090537 | 16 | 42090537 | 150726 | A | G | A | 0.8871 | G | 0.1129 | 5 | 0.16129 |
| 13133 | S16_42129765 | 16 | 42129765 | 39228 | A | T | T | 0.59677 | A | 0.40323 | 17 | 0.54839 |
| 13134 | S16_42228108 | 16 | 42228108 | 98343 | T | C | T | 0.83871 | C | 0.16129 | 8 | 0.25806 |
| 13135 | S16_42418338 | 16 | 42418338 | 190230 | C | T | C | 0.93548 | T | 0.06452 | 4 | 0.12903 |
| 13136 | S16_42474581 | 16 | 42474581 | 56243 | G | A | G | 0.85484 | A | 0.14516 | 5 | 0.16129 |
| 13137 | S16_42517698 | 16 | 42517698 | 43117 | C | T | C | 0.77419 | T | 0.22581 | 8 | 0.25806 |
| 13138 | S16_42695054 | 16 | 42695054 | 177356 | T | C | C | 0.70968 | T | 0.29032 | 14 | 0.45161 |
| 13139 | S16_42695065 | 16 | 42695065 | 11 | A | C | C | 0.70968 | A | 0.29032 | 14 | 0.45161 |
| 13140 | S16_42949743 | 16 | 42949743 | 254678 | A | G | A | 0.83871 | G | 0.16129 | 8 | 0.25806 |
| 13141 | S16_42949752 | 16 | 42949752 | 9 | G | C | G | 0.83871 | C | 0.16129 | 8 | 0.25806 |
| 13142 | S16_42949767 | 16 | 42949767 | 15 | T | G | T | 0.82258 | G | 0.17742 | 9 | 0.29032 |
| 13143 | S16_42949771 | 16 | 42949771 | 4 | G | A | G | 0.82258 | A | 0.17742 | 9 | 0.29032 |
| 13144 | S16_43349535 | 16 | 43349535 | 399764 | T | C | T | 0.8871 | C | 0.1129 | 5 | 0.16129 |
| 13145 | S16_43517995 | 16 | 43517995 | 168460 | C | T | C | 0.75806 | T | 0.24194 | 11 | 0.35484 |
| 13146 | S16_43613230 | 16 | 43613230 | 95235 | G | A | G | 0.91935 | A | 0.08065 | 5 | 0.16129 |
| 13147 | S16_43793406 | 16 | 43793406 | 180176 | A | G | A | 0.93548 | G | 0.06452 | 4 | 0.12903 |
| 13148 | S16_43793451 | 16 | 43793451 | 45 | A | G | A | 0.91935 | G | 0.08065 | 5 | 0.16129 |
| 13149 | S16_43793492 | 16 | 43793492 | 41 | G | A | G | 0.83871 | A | 0.16129 | 10 | 0.32258 |
| 13150 | S16_43793511 | 16 | 43793511 | 19 | G | A | G | 0.59677 | A | 0.40323 | 21 | 0.67742 |
| 13151 | S16_43863873 | 16 | 43863873 | 70362 | C | T | C | 0.69355 | T | 0.30645 | 13 | 0.41935 |
| 13152 | S16_43870693 | 16 | 43870693 | 6820 | C | T | C | 0.58065 | T | 0.41935 | 14 | 0.45161 |
| 13153 | S16_43883806 | 16 | 43883806 | 13113 | T | C | T | 0.93548 | C | 0.06452 | 4 | 0.12903 |
| 13154 | S16_43973069 | 16 | 43973069 | 89263 | C | A | C | 0.91935 | A | 0.08065 | 5 | 0.16129 |
| 13155 | S16_44052167 | 16 | 44052167 | 79098 | G | A | G | 0.82258 | A | 0.17742 | 11 | 0.35484 |
| 13156 | S16_44378138 | 16 | 44378138 | 325971 | C | T | T | 0.93548 | C | 0.06452 | 2 | 0.06452 |
| 13157 | S16_44509203 | 16 | 44509203 | 131065 | C | T | C | 0.72581 | T | 0.27419 | 15 | 0.48387 |
| 13158 | S16_44594718 | 16 | 44594718 | 85515 | C | T | C | 0.93548 | T | 0.06452 | 2 | 0.06452 |
| 13159 | S16_44832127 | 16 | 44832127 | 237409 | T | G | T | 0.91935 | G | 0.08065 | 5 | 0.16129 |
| 13160 | S16_45644192 | 16 | 45644192 | 812065 | C | T | C | 0.8871 | T | 0.1129 | 7 | 0.22581 |
| 13161 | S16_45909333 | 16 | 45909333 | 265141 | A | G | A | 0.69355 | G | 0.30645 | 13 | 0.41935 |
| 13162 | S16_46032153 | 16 | 46032153 | 122820 | A | G | A | 0.5 | G | 0.5 | 15 | 0.48387 |
| 13163 | S16_46032222 | 16 | 46032222 | 69 | C | G | C | 0.6129 | G | 0.3871 | 12 | 0.3871 |
| 13164 | S16_46032257 | 16 | 46032257 | 35 | A | T | A | 0.6129 | T | 0.3871 | 12 | 0.3871 |
| 13165 | S16_46032265 | 16 | 46032265 | 8 | C | T | C | 0.6129 | T | 0.3871 | 12 | 0.3871 |
| 13166 | S16_46032289 | 16 | 46032289 | 24 | G | T | G | 0.6129 | T | 0.3871 | 12 | 0.3871 |
| 13167 | S16_46032351 | 16 | 46032351 | 62 | A | G | A | 0.74194 | G | 0.25806 | 12 | 0.3871 |
| 13168 | S16_46165282 | 16 | 46165282 | 132931 | G | A | A | 0.51613 | G | 0.48387 | 20 | 0.64516 |
| 13169 | S16_46295847 | 16 | 46295847 | 130565 | G | A | G | 0.56452 | A | 0.43548 | 19 | 0.6129 |
| 13170 | S16_46339449 | 16 | 46339449 | 43602 | G | A | G | 0.69355 | A | 0.30645 | 13 | 0.41935 |
| 13171 | S16_46339522 | 16 | 46339522 | 73 | A | G | A | 0.69355 | G | 0.30645 | 13 | 0.41935 |
| 13172 | S16_46339569 | 16 | 46339569 | 47 | C | T | C | 0.6129 | T | 0.3871 | 16 | 0.51613 |
| 13173 | S16_46460912 | 16 | 46460912 | 121343 | A | G | G | 0.93548 | A | 0.06452 | 4 | 0.12903 |
| 13174 | S16_46460994 | 16 | 46460994 | 82 | T | C | T | 0.69355 | C | 0.30645 | 15 | 0.48387 |

| Site number | SNP name | Chromosome | Physical position | Physical distance from the previous SNP | Reference allele | Alternative allele | Major allele | Major allele frequency | Minor allele | Minor allele frequency | Number heterozygous | Heterozygosity |
|---|---|---|---|---|---|---|---|---|---|---|---|---|
| 13175 | S16_46461071 | 16 | 46461071 | 77 | G | T | G | 0.72581 | T | 0.27419 | 13 | 0.41935 |
| 13176 | S16_46621143 | 16 | 46621143 | 160072 | G | A | A | 0.5 | G | 0.5 | 15 | 0.48387 |
| 13177 | S16_46993342 | 16 | 46993342 | 372199 | G | A | G | 0.91935 | A | 0.08065 | 5 | 0.16129 |
| 13178 | S16_47154730 | 16 | 47154730 | 161388 | C | T | C | 0.93548 | T | 0.06452 | 4 | 0.12903 |
| 13179 | S16_47154732 | 16 | 47154732 | 2 | G | A | G | 0.70968 | A | 0.29032 | 12 | 0.3871 |
| 13180 | S16_47154766 | 16 | 47154766 | 34 | A | G | A | 0.70968 | G | 0.29032 | 12 | 0.3871 |
| 13181 | S16_47225780 | 16 | 47225780 | 71014 | G | A | G | 0.80645 | A | 0.19355 | 6 | 0.19355 |
| 13182 | S16_47287705 | 16 | 47287705 | 61925 | G | A | G | 0.93548 | A | 0.06452 | 4 | 0.12903 |
| 13183 | S16_47287710 | 16 | 47287710 | 5 | A | C | C | 0.90323 | A | 0.09677 | 6 | 0.19355 |
| 13184 | S16_47287752 | 16 | 47287752 | 42 | A | G | A | 0.87097 | G | 0.12903 | 4 | 0.12903 |
| 13185 | S16_47386795 | 16 | 47386795 | 99043 | C | G | G | 0.75806 | C | 0.24194 | 9 | 0.29032 |
| 13186 | S16_47425870 | 16 | 47425870 | 39075 | G | A | G | 0.75806 | A | 0.24194 | 9 | 0.29032 |
| 13187 | S16_47575399 | 16 | 47575399 | 149529 | A | G | A | 0.87097 | G | 0.12903 | 8 | 0.25806 |
| 13188 | S16_47575499 | 16 | 47575499 | 100 | G | A | G | 0.91935 | A | 0.08065 | 5 | 0.16129 |
| 13189 | S16_47716625 | 16 | 47716625 | 141126 | C | T | C | 0.70968 | T | 0.29032 | 12 | 0.3871 |
| 13190 | S16_49254393 | 16 | 49254393 | 1537768 | A | G | A | 0.91935 | G | 0.08065 | 5 | 0.16129 |
| 13191 | S16_50392645 | 16 | 50392645 | 1138252 | T | C | T | 0.93548 | C | 0.06452 | 4 | 0.12903 |
| 13192 | S16_50654528 | 16 | 50654528 | 261883 | T | C | T | 0.91935 | C | 0.08065 | 5 | 0.16129 |
| 13193 | S16_50818377 | 16 | 50818377 | 163849 | G | A | G | 0.80645 | A | 0.19355 | 10 | 0.32258 |
| 13194 | S16_50961160 | 16 | 50961160 | 142783 | C | G | C | 0.87097 | G | 0.12903 | 6 | 0.19355 |
| 13195 | S16_50961256 | 16 | 50961256 | 96 | A | G | A | 0.87097 | G | 0.12903 | 6 | 0.19355 |
| 13196 | S16_50961267 | 16 | 50961267 | 11 | T | G | G | 0.80645 | T | 0.19355 | 12 | 0.3871 |
| 13197 | S16_51074071 | 16 | 51074071 | 112804 | C | G | C | 0.79032 | G | 0.20968 | 11 | 0.35484 |
| 13198 | S16_51074119 | 16 | 51074119 | 48 | T | C | C | 0.70968 | T | 0.29032 | 12 | 0.3871 |
| 13199 | S16_51137860 | 16 | 51137860 | 63741 | C | T | C | 0.62903 | T | 0.37097 | 15 | 0.48387 |
| 13200 | S16_51680768 | 16 | 51680768 | 542908 | C | T | C | 0.82258 | T | 0.17742 | 11 | 0.35484 |
| 13201 | S16_52215173 | 16 | 52215173 | 534405 | T | G | G | 0.83871 | T | 0.16129 | 8 | 0.25806 |
| 13202 | S16_52215291 | 16 | 52215291 | 118 | G | A | G | 0.95161 | A | 0.04839 | 3 | 0.09677 |
| 13203 | S16_52275519 | 16 | 52275519 | 60228 | C | G | G | 0.82258 | C | 0.17742 | 9 | 0.29032 |
| 13204 | S16_52275717 | 16 | 52275717 | 198 | G | A | G | 0.87097 | A | 0.12903 | 6 | 0.19355 |
| 13205 | S16_52283531 | 16 | 52283531 | 7814 | G | A | G | 0.59677 | A | 0.40323 | 19 | 0.6129 |
| 13206 | S16_52449925 | 16 | 52449925 | 166394 | G | A | G | 0.85484 | A | 0.14516 | 9 | 0.29032 |
| 13207 | S16_52449967 | 16 | 52449967 | 42 | G | A | G | 0.8871 | A | 0.1129 | 7 | 0.22581 |
| 13208 | S16_52482954 | 16 | 52482954 | 32987 | G | A | G | 0.85484 | A | 0.14516 | 9 | 0.29032 |
| 13209 | S16_53012135 | 16 | 53012135 | 529181 | T | C | C | 0.66129 | T | 0.33871 | 13 | 0.41935 |
| 13210 | S16_53147309 | 16 | 53147309 | 135174 | G | A | G | 0.74194 | A | 0.25806 | 12 | 0.3871 |
| 13211 | S16_53620575 | 16 | 53620575 | 473266 | C | T | T | 0.54839 | C | 0.45161 | 16 | 0.51613 |
| 13212 | S16_54437107 | 16 | 54437107 | 816532 | A | G | G | 0.62903 | A | 0.37097 | 17 | 0.54839 |
| 13213 | S16_55077144 | 16 | 55077144 | 640037 | T | C | C | 0.72581 | T | 0.27419 | 13 | 0.41935 |
| 13214 | S16_55077187 | 16 | 55077187 | 43 | C | T | C | 0.79032 | T | 0.20968 | 13 | 0.41935 |
| 13215 | S16_55313942 | 16 | 55313942 | 236755 | T | C | C | 0.85484 | T | 0.14516 | 7 | 0.22581 |
| 13216 | S16_55318248 | 16 | 55318248 | 4306 | A | G | G | 0.70968 | A | 0.29032 | 16 | 0.51613 |
| 13217 | S16_55453695 | 16 | 55453695 | 135447 | G | A | G | 0.79032 | A | 0.20968 | 11 | 0.35484 |
| 13218 | S16_55453785 | 16 | 55453785 | 90 | C | T | C | 0.85484 | T | 0.14516 | 7 | 0.22581 |
| 13219 | S16_55453921 | 16 | 55453921 | 136 | G | A | G | 0.83871 | A | 0.16129 | 8 | 0.25806 |
| 13220 | S16_55598237 | 16 | 55598237 | 144316 | T | C | T | 0.62903 | C | 0.37097 | 15 | 0.48387 |
| 13221 | S16_55682010 | 16 | 55682010 | 83773 | G | A | G | 0.85484 | A | 0.14516 | 7 | 0.22581 |
| 13222 | S16_55682014 | 16 | 55682014 | 4 | C | A | C | 0.93548 | A | 0.06452 | 4 | 0.12903 |
| 13223 | S16_55682148 | 16 | 55682148 | 134 | T | C | T | 0.54839 | C | 0.45161 | 16 | 0.51613 |
| 13224 | S16_56400443 | 16 | 56400443 | 718295 | C | T | C | 0.83871 | T | 0.16129 | 8 | 0.25806 |
| 13225 | S16_56429700 | 16 | 56429700 | 29257 | T | C | T | 0.67742 | C | 0.32258 | 12 | 0.3871 |
| 13226 | S16_56659575 | 16 | 56659575 | 229875 | C | T | C | 0.91935 | T | 0.08065 | 5 | 0.16129 |
| 13227 | S16_57168821 | 16 | 57168821 | 509246 | G | A | G | 0.93548 | A | 0.06452 | 4 | 0.12903 |
| 13228 | S16_57168822 | 16 | 57168822 | 1 | C | A | C | 0.62903 | A | 0.37097 | 13 | 0.41935 |

| Site number | SNP name | Chromosome | Physical position | Physical distance from the previous SNP | Reference allele | Alternative allele | Major allele | Major allele frequency | Minor allele | Minor allele frequency | Number heterozygous | Heterozygosity |
|---|---|---|---|---|---|---|---|---|---|---|---|---|
| 13229 | S16_57168823 | 16 | 57168823 | 1 | G | A | G | 0.93548 | A | 0.06452 | 4 | 0.12903 |
| 13230 | S16_57168843 | 16 | 57168843 | 20 | T | C | T | 0.59677 | C | 0.40323 | 15 | 0.48387 |
| 13231 | S16_57168860 | 16 | 57168860 | 17 | C | T | C | 0.93548 | T | 0.06452 | 4 | 0.12903 |
| 13232 | S16_57168882 | 16 | 57168882 | 22 | C | T | C | 0.58065 | T | 0.41935 | 20 | 0.64516 |
| 13233 | S16_57168900 | 16 | 57168900 | 18 | A | T | A | 0.8871 | T | 0.1129 | 7 | 0.22581 |
| 13234 | S16_57168920 | 16 | 57168920 | 20 | T | C | C | 0.51613 | T | 0.48387 | 18 | 0.58065 |
| 13235 | S16_57168922 | 16 | 57168922 | 2 | G | A | G | 0.69355 | A | 0.30645 | 11 | 0.35484 |
| 13236 | S16_57391822 | 16 | 57391822 | 222900 | C | T | C | 0.77419 | T | 0.22581 | 8 | 0.25806 |
| 13237 | S16_57391849 | 16 | 57391849 | 27 | A | G | G | 0.62903 | A | 0.37097 | 15 | 0.48387 |
| 13238 | S16_57391869 | 16 | 57391869 | 20 | T | C | C | 0.62903 | T | 0.37097 | 15 | 0.48387 |
| 13239 | S16_57391923 | 16 | 57391923 | 54 | C | T | T | 0.62903 | C | 0.37097 | 15 | 0.48387 |
| 13240 | S16_57683183 | 16 | 57683183 | 291260 | C | T | C | 0.8871 | T | 0.1129 | 7 | 0.22581 |
| 13241 | S16_57683269 | 16 | 57683269 | 86 | A | G | A | 0.90323 | G | 0.09677 | 6 | 0.19355 |
| 13242 | S16_57985599 | 16 | 57985599 | 302330 | G | A | G | 0.69355 | A | 0.30645 | 17 | 0.54839 |
| 13243 | S16_57985722 | 16 | 57985722 | 123 | A | C | A | 0.69355 | C | 0.30645 | 17 | 0.54839 |
| 13244 | S16_58003233 | 16 | 58003233 | 17511 | T | C | T | 0.70968 | C | 0.29032 | 16 | 0.51613 |
| 13245 | S16_58270426 | 16 | 58270426 | 267193 | G | A | G | 0.90323 | A | 0.09677 | 6 | 0.19355 |
| 13246 | S16_58325336 | 16 | 58325336 | 54910 | G | T | G | 0.5 | T | 0.5 | 15 | 0.48387 |
| 13247 | S16_58325337 | 16 | 58325337 | 1 | C | T | C | 0.5 | T | 0.5 | 15 | 0.48387 |
| 13248 | S16_58508811 | 16 | 58508811 | 183474 | C | T | C | 0.64516 | T | 0.35484 | 14 | 0.45161 |
| 13249 | S16_58508891 | 16 | 58508891 | 80 | G | A | G | 0.82258 | A | 0.17742 | 11 | 0.35484 |
| 13250 | S16_58523730 | 16 | 58523730 | 14839 | C | T | C | 0.85484 | T | 0.14516 | 9 | 0.29032 |
| 13251 | S16_58523846 | 16 | 58523846 | 116 | A | G | G | 0.6129 | A | 0.3871 | 16 | 0.51613 |
| 13252 | S16_58633499 | 16 | 58633499 | 109653 | A | G | A | 0.82258 | G | 0.17742 | 9 | 0.29032 |
| 13253 | S16_58679823 | 16 | 58679823 | 46324 | C | T | C | 0.82258 | T | 0.17742 | 9 | 0.29032 |
| 13254 | S16_58679966 | 16 | 58679966 | 143 | A | G | A | 0.66129 | G | 0.33871 | 19 | 0.6129 |
| 13255 | S16_59104412 | 16 | 59104412 | 424446 | T | C | C | 0.72581 | T | 0.27419 | 13 | 0.41935 |
| 13256 | S16_59104442 | 16 | 59104442 | 30 | C | T | T | 0.53226 | C | 0.46774 | 17 | 0.54839 |
| 13257 | S16_59136225 | 16 | 59136225 | 31783 | G | C | G | 0.85484 | C | 0.14516 | 9 | 0.29032 |
| 13258 | S16_59388292 | 16 | 59388292 | 252067 | C | T | C | 0.95161 | T | 0.04839 | 3 | 0.09677 |
| 13259 | S16_59388338 | 16 | 59388338 | 46 | C | A | C | 0.95161 | A | 0.04839 | 3 | 0.09677 |
| 13260 | S16_59530784 | 16 | 59530784 | 142446 | G | A | G | 0.93548 | A | 0.06452 | 2 | 0.06452 |
| 13261 | S16_59652310 | 16 | 59652310 | 121526 | G | A | A | 0.51613 | G | 0.48387 | 12 | 0.3871 |
| 13262 | S16_59652450 | 16 | 59652450 | 140 | A | C | A | 0.83871 | C | 0.16129 | 10 | 0.32258 |
| 13263 | S16_59697828 | 16 | 59697828 | 45378 | T | C | T | 0.90323 | C | 0.09677 | 4 | 0.12903 |
| 13264 | S16_59697844 | 16 | 59697844 | 16 | G | C | G | 0.93548 | C | 0.06452 | 4 | 0.12903 |
| 13265 | S16_59697878 | 16 | 59697878 | 34 | A | G | A | 0.90323 | G | 0.09677 | 4 | 0.12903 |
| 13266 | S16_59697961 | 16 | 59697961 | 83 | C | T | C | 0.93548 | T | 0.06452 | 4 | 0.12903 |
| 13267 | S16_59719524 | 16 | 59719524 | 21563 | G | A | G | 0.95161 | A | 0.04839 | 3 | 0.09677 |
| 13268 | S16_60241190 | 16 | 60241190 | 521666 | C | T | C | 0.93548 | T | 0.06452 | 4 | 0.12903 |
| 13269 | S16_60295495 | 16 | 60295495 | 54305 | A | G | A | 0.93548 | G | 0.06452 | 4 | 0.12903 |
| 13270 | S16_60295557 | 16 | 60295557 | 62 | C | G | C | 0.95161 | G | 0.04839 | 3 | 0.09677 |
| 13271 | S16_60295693 | 16 | 60295693 | 136 | C | T | C | 0.93548 | T | 0.06452 | 4 | 0.12903 |
| 13272 | S16_60295697 | 16 | 60295697 | 4 | G | A | G | 0.67742 | A | 0.32258 | 18 | 0.58065 |
| 13273 | S16_60299441 | 16 | 60299441 | 3744 | A | G | A | 0.83871 | G | 0.16129 | 6 | 0.19355 |
| 13274 | S16_61500055 | 16 | 61500055 | 1200614 | C | T | C | 0.93548 | T | 0.06452 | 4 | 0.12903 |
| 13275 | S16_61508280 | 16 | 61508280 | 8225 | G | C | G | 0.56452 | C | 0.43548 | 17 | 0.54839 |
| 13276 | S16_61508281 | 16 | 61508281 | 1 | G | A | G | 0.56452 | A | 0.43548 | 17 | 0.54839 |
| 13277 | S16_61508398 | 16 | 61508398 | 117 | G | A | A | 0.72581 | G | 0.27419 | 13 | 0.41935 |
| 13278 | S16_61508405 | 16 | 61508405 | 7 | A | G | G | 0.85484 | A | 0.14516 | 9 | 0.29032 |
| 13279 | S16_61508428 | 16 | 61508428 | 23 | G | A | G | 0.90323 | A | 0.09677 | 6 | 0.19355 |
| 13280 | S16_61508448 | 16 | 61508448 | 20 | A | G | A | 0.56452 | G | 0.43548 | 17 | 0.54839 |
| 13281 | S16_61572171 | 16 | 61572171 | 63723 | T | C | T | 0.54839 | C | 0.45161 | 16 | 0.51613 |
| 13282 | S16_61572191 | 16 | 61572191 | 20 | A | G | A | 0.54839 | G | 0.45161 | 16 | 0.51613 |

| Site number | SNP name | Chromosome | Physical position | Physical distance from the previous SNP | Reference allele | Alternative allele | Major allele | Major allele frequency | Minor allele | Minor allele frequency | Number heterozygous | Heterozygosity |
|---|---|---|---|---|---|---|---|---|---|---|---|---|
| 13283 | S16_61572209 | 16 | 61572209 | 18 | C | T | C | 0.93548 | T | 0.06452 | 2 | 0.06452 |
| 13284 | S16_61572328 | 16 | 61572328 | 119 | T | C | C | 0.80645 | T | 0.19355 | 12 | 0.3871 |
| 13285 | S16_61716980 | 16 | 61716980 | 144652 | A | G | A | 0.82258 | G | 0.17742 | 11 | 0.35484 |
| 13286 | S16_61717024 | 16 | 61717024 | 44 | C | T | C | 0.82258 | T | 0.17742 | 11 | 0.35484 |
| 13287 | S16_61717030 | 16 | 61717030 | 6 | T | G | T | 0.82258 | G | 0.17742 | 11 | 0.35484 |
| 13288 | S16_61717082 | 16 | 61717082 | 52 | A | G | A | 0.82258 | G | 0.17742 | 11 | 0.35484 |
| 13289 | S16_61717122 | 16 | 61717122 | 40 | A | C | A | 0.82258 | C | 0.17742 | 11 | 0.35484 |
| 13290 | S16_61818912 | 16 | 61818912 | 101790 | G | A | G | 0.93548 | A | 0.06452 | 4 | 0.12903 |
| 13291 | S16_61818956 | 16 | 61818956 | 44 | A | G | A | 0.56452 | G | 0.43548 | 13 | 0.41935 |
| 13292 | S16_61819046 | 16 | 61819046 | 90 | G | T | G | 0.93548 | T | 0.06452 | 4 | 0.12903 |
| 13293 | S16_61819090 | 16 | 61819090 | 44 | G | A | G | 0.87097 | A | 0.12903 | 8 | 0.25806 |
| 13294 | S16_61838469 | 16 | 61838469 | 19379 | A | C | A | 0.77419 | C | 0.22581 | 14 | 0.45161 |
| 13295 | S16_61838590 | 16 | 61838590 | 121 | G | T | G | 0.93548 | T | 0.06452 | 4 | 0.12903 |
| 13296 | S16_61838596 | 16 | 61838596 | 6 | T | C | C | 0.70968 | T | 0.29032 | 16 | 0.51613 |
| 13297 | S16_61838661 | 16 | 61838661 | 65 | A | C | A | 0.82258 | C | 0.17742 | 9 | 0.29032 |
| 13298 | S16_62066685 | 16 | 62066685 | 228024 | C | G | C | 0.91935 | G | 0.08065 | 5 | 0.16129 |
| 13299 | S16_62066763 | 16 | 62066763 | 78 | C | T | T | 0.8871 | C | 0.1129 | 7 | 0.22581 |
| 13300 | S16_62066849 | 16 | 62066849 | 86 | C | G | C | 0.93548 | G | 0.06452 | 4 | 0.12903 |
| 13301 | S16_62212941 | 16 | 62212941 | 146092 | A | G | G | 0.74194 | A | 0.25806 | 10 | 0.32258 |
| 13302 | S16_62420005 | 16 | 62420005 | 207064 | G | A | G | 0.83871 | A | 0.16129 | 6 | 0.19355 |
| 13303 | S16_62508197 | 16 | 62508197 | 88192 | A | G | A | 0.90323 | G | 0.09677 | 6 | 0.19355 |
| 13304 | S16_62633102 | 16 | 62633102 | 124905 | A | G | G | 0.90323 | A | 0.09677 | 4 | 0.12903 |
| 13305 | S16_62633210 | 16 | 62633210 | 108 | A | G | G | 0.93548 | A | 0.06452 | 4 | 0.12903 |
| 13306 | S16_62633282 | 16 | 62633282 | 72 | C | A | A | 0.90323 | C | 0.09677 | 4 | 0.12903 |
| 13307 | S16_62633285 | 16 | 62633285 | 3 | C | T | C | 0.87097 | T | 0.12903 | 6 | 0.19355 |
| 13308 | S16_62633286 | 16 | 62633286 | 1 | A | G | G | 0.90323 | A | 0.09677 | 4 | 0.12903 |
| 13309 | S16_62696707 | 16 | 62696707 | 63421 | C | T | T | 0.62903 | C | 0.37097 | 19 | 0.6129 |
| 13310 | S16_62696810 | 16 | 62696810 | 103 | G | A | G | 0.75806 | A | 0.24194 | 13 | 0.41935 |
| 13311 | S16_63410765 | 16 | 63410765 | 713955 | G | A | G | 0.90323 | A | 0.09677 | 6 | 0.19355 |
| 13312 | S16_63410861 | 16 | 63410861 | 96 | G | C | C | 0.58065 | G | 0.41935 | 12 | 0.3871 |
| 13313 | S16_64044279 | 16 | 64044279 | 633418 | T | A | T | 0.85484 | A | 0.14516 | 9 | 0.29032 |
| 13314 | S16_64180471 | 16 | 64180471 | 136192 | G | A | G | 0.77419 | A | 0.22581 | 12 | 0.3871 |
| 13315 | S16_64180553 | 16 | 64180553 | 82 | C | T | C | 0.56452 | T | 0.43548 | 21 | 0.67742 |
| 13316 | S16_64180664 | 16 | 64180664 | 111 | G | A | G | 0.8871 | A | 0.1129 | 7 | 0.22581 |
| 13317 | S16_64309808 | 16 | 64309808 | 129144 | C | T | C | 0.85484 | T | 0.14516 | 7 | 0.22581 |
| 13318 | S16_64309836 | 16 | 64309836 | 28 | C | T | C | 0.66129 | T | 0.33871 | 13 | 0.41935 |
| 13319 | S16_64310003 | 16 | 64310003 | 167 | A | C | A | 0.83871 | C | 0.16129 | 10 | 0.32258 |
| 13320 | S16_64322871 | 16 | 64322871 | 12868 | T | C | T | 0.83871 | C | 0.16129 | 10 | 0.32258 |
| 13321 | S16_64322907 | 16 | 64322907 | 36 | T | G | T | 0.8871 | G | 0.1129 | 7 | 0.22581 |
| 13322 | S16_64322932 | 16 | 64322932 | 25 | C | T | C | 0.85484 | T | 0.14516 | 9 | 0.29032 |
| 13323 | S16_64783136 | 16 | 64783136 | 460204 | C | G | C | 0.93548 | G | 0.06452 | 4 | 0.12903 |
| 13324 | S16_64836729 | 16 | 64836729 | 53593 | G | T | G | 0.54839 | T | 0.45161 | 12 | 0.3871 |
| 13325 | S16_64836734 | 16 | 64836734 | 5 | G | A | G | 0.54839 | A | 0.45161 | 12 | 0.3871 |
| 13326 | S16_64836737 | 16 | 64836737 | 3 | C | G | G | 0.58065 | C | 0.41935 | 14 | 0.45161 |
| 13327 | S16_65023863 | 16 | 65023863 | 187126 | G | A | G | 0.66129 | A | 0.33871 | 15 | 0.48387 |
| 13328 | S16_65081025 | 16 | 65081025 | 57162 | C | T | C | 0.93548 | T | 0.06452 | 4 | 0.12903 |
| 13329 | S16_65081047 | 16 | 65081047 | 22 | C | T | C | 0.74194 | T | 0.25806 | 12 | 0.3871 |
| 13330 | S16_65081117 | 16 | 65081117 | 70 | C | T | C | 0.93548 | T | 0.06452 | 4 | 0.12903 |
| 13331 | S16_65557086 | 16 | 65557086 | 475969 | T | A | T | 0.91935 | A | 0.08065 | 5 | 0.16129 |
| 13332 | S16_65557213 | 16 | 65557213 | 127 | C | T | C | 0.90323 | T | 0.09677 | 6 | 0.19355 |
| 13333 | S16_65557334 | 16 | 65557334 | 121 | C | T | C | 0.66129 | T | 0.33871 | 13 | 0.41935 |
| 13334 | S16_65988686 | 16 | 65988686 | 431352 | G | T | G | 0.67742 | T | 0.32258 | 18 | 0.58065 |
| 13335 | S16_65988724 | 16 | 65988724 | 38 | C | T | T | 0.53226 | C | 0.46774 | 19 | 0.6129 |
| 13336 | S16_65988847 | 16 | 65988847 | 123 | G | A | A | 0.91935 | G | 0.08065 | 3 | 0.09677 |

| Site number | SNP name | Chromosome | Physical position | Physical distance from the previous SNP | Reference allele | Alternative allele | Major allele | Major allele frequency | Minor allele | Minor allele frequency | Number heterozygous | Heterozygosity |
|---|---|---|---|---|---|---|---|---|---|---|---|---|
| 13337 | S16_66344163 | 16 | 66344163 | 355316 | T | C | T | 0.64516 | C | 0.35484 | 16 | 0.51613 |
| 13338 | S16_66344213 | 16 | 66344213 | 50 | G | A | G | 0.85484 | A | 0.14516 | 7 | 0.22581 |
| 13339 | S16_66683402 | 16 | 66683402 | 339189 | A | G | A | 0.59677 | G | 0.40323 | 15 | 0.48387 |
| 13340 | S16_66683504 | 16 | 66683504 | 102 | C | A | C | 0.95161 | A | 0.04839 | 3 | 0.09677 |
| 13341 | S16_66683563 | 16 | 66683563 | 59 | G | A | G | 0.85484 | A | 0.14516 | 7 | 0.22581 |
| 13342 | S16_67045645 | 16 | 67045645 | 362082 | C | T | C | 0.91935 | T | 0.08065 | 5 | 0.16129 |
| 13343 | S16_67045716 | 16 | 67045716 | 71 | T | A | T | 0.70968 | A | 0.29032 | 12 | 0.3871 |
| 13344 | S16_67045870 | 16 | 67045870 | 154 | A | G | A | 0.93548 | G | 0.06452 | 2 | 0.06452 |
| 13345 | S16_67197239 | 16 | 67197239 | 151369 | C | T | C | 0.72581 | T | 0.27419 | 13 | 0.41935 |
| 13346 | S16_67197300 | 16 | 67197300 | 61 | T | C | T | 0.53226 | C | 0.46774 | 19 | 0.6129 |
| 13347 | S16_67659500 | 16 | 67659500 | 462200 | C | T | C | 0.90323 | T | 0.09677 | 6 | 0.19355 |
| 13348 | S16_67659557 | 16 | 67659557 | 57 | A | G | A | 0.90323 | G | 0.09677 | 4 | 0.12903 |
| 13349 | S16_67694734 | 16 | 67694734 | 35177 | C | T | C | 0.91935 | T | 0.08065 | 5 | 0.16129 |
| 13350 | S16_67694776 | 16 | 67694776 | 42 | G | A | G | 0.77419 | A | 0.22581 | 10 | 0.32258 |
| 13351 | S16_67694883 | 16 | 67694883 | 107 | T | G | G | 0.85484 | T | 0.14516 | 9 | 0.29032 |
| 13352 | S16_67694891 | 16 | 67694891 | 8 | C | T | T | 0.6129 | C | 0.3871 | 12 | 0.3871 |
| 13353 | S16_67852428 | 16 | 67852428 | 157537 | T | A | T | 0.85484 | A | 0.14516 | 9 | 0.29032 |
| 13354 | S16_67852490 | 16 | 67852490 | 62 | A | T | A | 0.85484 | T | 0.14516 | 9 | 0.29032 |
| 13355 | S16_67852536 | 16 | 67852536 | 46 | C | T | C | 0.82258 | T | 0.17742 | 9 | 0.29032 |
| 13356 | S16_67971947 | 16 | 67971947 | 119411 | C | T | C | 0.67742 | T | 0.32258 | 10 | 0.32258 |
| 13357 | S16_68143791 | 16 | 68143791 | 171844 | G | A | G | 0.77419 | A | 0.22581 | 12 | 0.3871 |
| 13358 | S16_68510788 | 16 | 68510788 | 366997 | T | C | T | 0.83871 | C | 0.16129 | 10 | 0.32258 |
| 13359 | S16_68510824 | 16 | 68510824 | 36 | C | T | C | 0.58065 | T | 0.41935 | 14 | 0.45161 |
| 13360 | S16_68740863 | 16 | 68740863 | 230039 | A | G | A | 0.69355 | G | 0.30645 | 13 | 0.41935 |
| 13361 | S16_68740928 | 16 | 68740928 | 65 | T | C | C | 0.85484 | T | 0.14516 | 5 | 0.16129 |
| 13362 | S16_68879710 | 16 | 68879710 | 138782 | T | G | T | 0.95161 | G | 0.04839 | 3 | 0.09677 |
| 13363 | S16_69073403 | 16 | 69073403 | 193693 | C | T | C | 0.54839 | T | 0.45161 | 14 | 0.45161 |
| 13364 | S16_69073426 | 16 | 69073426 | 23 | C | G | C | 0.56452 | G | 0.43548 | 13 | 0.41935 |
| 13365 | S16_69073450 | 16 | 69073450 | 24 | C | T | C | 0.56452 | T | 0.43548 | 13 | 0.41935 |
| 13366 | S16_69073571 | 16 | 69073571 | 121 | C | T | C | 0.90323 | T | 0.09677 | 6 | 0.19355 |
| 13367 | S16_69265195 | 16 | 69265195 | 191624 | T | C | T | 0.51613 | C | 0.48387 | 18 | 0.58065 |
| 13368 | S16_69265368 | 16 | 69265368 | 173 | T | C | T | 0.87097 | C | 0.12903 | 6 | 0.19355 |
| 13369 | S16_69609637 | 16 | 69609637 | 344269 | T | G | T | 0.93548 | G | 0.06452 | 4 | 0.12903 |
| 13370 | S16_70110976 | 16 | 70110976 | 501339 | A | C | A | 0.95161 | C | 0.04839 | 3 | 0.09677 |
| 13371 | S16_70111019 | 16 | 70111019 | 43 | T | A | A | 0.82258 | T | 0.17742 | 9 | 0.29032 |
| 13372 | S16_70111197 | 16 | 70111197 | 178 | G | A | G | 0.90323 | A | 0.09677 | 4 | 0.12903 |
| 13373 | S16_70218180 | 16 | 70218180 | 106983 | T | C | T | 0.95161 | C | 0.04839 | 3 | 0.09677 |
| 13374 | S16_70218186 | 16 | 70218186 | 6 | A | G | A | 0.95161 | G | 0.04839 | 3 | 0.09677 |
| 13375 | S16_70218322 | 16 | 70218322 | 136 | C | A | C | 0.66129 | A | 0.33871 | 13 | 0.41935 |
| 13376 | S16_70218351 | 16 | 70218351 | 29 | T | C | T | 0.83871 | C | 0.16129 | 10 | 0.32258 |
| 13377 | S16_71040097 | 16 | 71040097 | 821746 | G | A | G | 0.93548 | A | 0.06452 | 4 | 0.12903 |
| 13378 | S16_71040127 | 16 | 71040127 | 30 | A | G | A | 0.75806 | G | 0.24194 | 11 | 0.35484 |
| 13379 | S16_71040174 | 16 | 71040174 | 47 | T | C | C | 0.51613 | T | 0.48387 | 12 | 0.3871 |
| 13380 | S16_71040211 | 16 | 71040211 | 37 | G | A | G | 0.75806 | A | 0.24194 | 11 | 0.35484 |
| 13381 | S16_71040231 | 16 | 71040231 | 20 | A | C | A | 0.74194 | C | 0.25806 | 10 | 0.32258 |
| 13382 | S16_71082074 | 16 | 71082074 | 41843 | G | A | G | 0.70968 | A | 0.29032 | 16 | 0.51613 |
| 13383 | S16_71082097 | 16 | 71082097 | 23 | C | A | C | 0.95161 | A | 0.04839 | 3 | 0.09677 |
| 13384 | S16_71082119 | 16 | 71082119 | 22 | G | T | G | 0.77419 | T | 0.22581 | 12 | 0.3871 |
| 13385 | S16_71083156 | 16 | 71083156 | 1037 | G | A | G | 0.72581 | A | 0.27419 | 15 | 0.48387 |
| 13386 | S16_71083159 | 16 | 71083159 | 3 | G | A | G | 0.72581 | A | 0.27419 | 15 | 0.48387 |
| 13387 | S16_71083267 | 16 | 71083267 | 108 | T | C | T | 0.72581 | C | 0.27419 | 15 | 0.48387 |
| 13388 | S16_71083309 | 16 | 71083309 | 42 | T | C | C | 0.51613 | T | 0.48387 | 18 | 0.58065 |
| 13389 | S16_71083358 | 16 | 71083358 | 49 | A | G | A | 0.58065 | G | 0.41935 | 16 | 0.51613 |
| 13390 | S16_71083611 | 16 | 71083611 | 253 | C | T | C | 0.8871 | T | 0.1129 | 7 | 0.22581 |

| Site number | SNP name | Chromosome | Physical position | Physical distance from the previous SNP | Reference allele | Alternative allele | Major allele | Major allele frequency | Minor allele | Minor allele frequency | Number heterozygous | Heterozygosity |
|---|---|---|---|---|---|---|---|---|---|---|---|---|
| 13391 | S16_71272203 | 16 | 71272203 | 188592 | G | C | G | 0.72581 | C | 0.27419 | 13 | 0.41935 |
| 13392 | S16_71272321 | 16 | 71272321 | 118 | C | T | C | 0.83871 | T | 0.16129 | 10 | 0.32258 |
| 13393 | S16_71272344 | 16 | 71272344 | 23 | A | G | G | 0.75806 | A | 0.24194 | 13 | 0.41935 |
| 13394 | S16_71272426 | 16 | 71272426 | 82 | C | T | C | 0.95161 | T | 0.04839 | 3 | 0.09677 |
| 13395 | S16_72090201 | 16 | 72090201 | 817775 | T | C | T | 0.77419 | C | 0.22581 | 12 | 0.3871 |
| 13396 | S16_72163101 | 16 | 72163101 | 72900 | C | T | C | 0.90323 | T | 0.09677 | 6 | 0.19355 |
| 13397 | S16_72177060 | 16 | 72177060 | 13959 | T | C | T | 0.82258 | C | 0.17742 | 11 | 0.35484 |
| 13398 | S16_72177204 | 16 | 72177204 | 144 | G | C | G | 0.95161 | C | 0.04839 | 3 | 0.09677 |
| 13399 | S16_72262132 | 16 | 72262132 | 84928 | C | A | C | 0.79032 | A | 0.20968 | 11 | 0.35484 |
| 13400 | S16_72278231 | 16 | 72278231 | 16099 | A | T | A | 0.87097 | T | 0.12903 | 6 | 0.19355 |
| 13401 | S16_72411032 | 16 | 72411032 | 132801 | C | T | C | 0.5 | T | 0.5 | 21 | 0.67742 |
| 13402 | S16_72411048 | 16 | 72411048 | 16 | T | A | T | 0.62903 | A | 0.37097 | 13 | 0.41935 |
| 13403 | S16_72411065 | 16 | 72411065 | 17 | C | T | C | 0.67742 | T | 0.32258 | 14 | 0.45161 |
| 13404 | S16_72411129 | 16 | 72411129 | 64 | C | T | C | 0.83871 | T | 0.16129 | 10 | 0.32258 |
| 13405 | S16_72472385 | 16 | 72472385 | 61256 | G | A | G | 0.93548 | A | 0.06452 | 4 | 0.12903 |
| 13406 | S16_72472456 | 16 | 72472456 | 71 | A | C | A | 0.69355 | C | 0.30645 | 17 | 0.54839 |
| 13407 | S16_72723595 | 16 | 72723595 | 251139 | G | T | G | 0.82258 | T | 0.17742 | 11 | 0.35484 |
| 13408 | S16_72723605 | 16 | 72723605 | 10 | A | G | A | 0.54839 | G | 0.45161 | 16 | 0.51613 |
| 13409 | S16_72861714 | 16 | 72861714 | 138109 | G | T | T | 0.74194 | G | 0.25806 | 14 | 0.45161 |
| 13410 | S16_72861741 | 16 | 72861741 | 27 | C | A | A | 0.74194 | C | 0.25806 | 14 | 0.45161 |
| 13411 | S16_72861775 | 16 | 72861775 | 34 | A | G | G | 0.74194 | A | 0.25806 | 14 | 0.45161 |
| 13412 | S16_72861869 | 16 | 72861869 | 94 | T | C | C | 0.74194 | T | 0.25806 | 14 | 0.45161 |
| 13413 | S16_73111853 | 16 | 73111853 | 249984 | G | A | G | 0.93548 | A | 0.06452 | 4 | 0.12903 |
| 13414 | S16_73629959 | 16 | 73629959 | 518106 | T | C | T | 0.62903 | C | 0.37097 | 15 | 0.48387 |
| 13415 | S16_73629960 | 16 | 73629960 | 1 | G | A | G | 0.62903 | A | 0.37097 | 15 | 0.48387 |
| 13416 | S16_73629993 | 16 | 73629993 | 33 | C | T | C | 0.85484 | T | 0.14516 | 9 | 0.29032 |
| 13417 | S16_73884942 | 16 | 73884942 | 254949 | G | C | C | 0.69355 | G | 0.30645 | 13 | 0.41935 |
| 13418 | S16_73897684 | 16 | 73897684 | 12742 | C | T | T | 0.91935 | C | 0.08065 | 5 | 0.16129 |
| 13419 | S16_73897691 | 16 | 73897691 | 7 | T | C | T | 0.72581 | C | 0.27419 | 13 | 0.41935 |
| 13420 | S16_73993251 | 16 | 73993251 | 95560 | C | T | C | 0.72581 | T | 0.27419 | 11 | 0.35484 |
| 13421 | S16_73993283 | 16 | 73993283 | 32 | G | A | G | 0.93548 | A | 0.06452 | 4 | 0.12903 |
| 13422 | S16_73993292 | 16 | 73993292 | 9 | A | C | A | 0.91935 | C | 0.08065 | 5 | 0.16129 |
| 13423 | S16_73993420 | 16 | 73993420 | 128 | C | T | C | 0.90323 | T | 0.09677 | 4 | 0.12903 |
| 13424 | S16_74430209 | 16 | 74430209 | 436789 | T | G | T | 0.93548 | G | 0.06452 | 4 | 0.12903 |
| 13425 | S16_74729031 | 16 | 74729031 | 298822 | A | G | A | 0.80645 | G | 0.19355 | 8 | 0.25806 |
| 13426 | S16_74936096 | 16 | 74936096 | 207065 | A | G | A | 0.79032 | G | 0.20968 | 9 | 0.29032 |
| 13427 | S16_74936192 | 16 | 74936192 | 96 | G | A | G | 0.8871 | A | 0.1129 | 7 | 0.22581 |
| 13428 | S16_74936210 | 16 | 74936210 | 18 | G | A | G | 0.82258 | A | 0.17742 | 9 | 0.29032 |
| 13429 | S16_74936233 | 16 | 74936233 | 23 | G | A | G | 0.58065 | A | 0.41935 | 20 | 0.64516 |
| 13430 | S16_75148247 | 16 | 75148247 | 212014 | G | A | G | 0.95161 | A | 0.04839 | 3 | 0.09677 |
| 13431 | S16_75283201 | 16 | 75283201 | 134954 | T | C | T | 0.77419 | C | 0.22581 | 12 | 0.3871 |
| 13432 | S16_75515879 | 16 | 75515879 | 232678 | A | G | A | 0.53226 | G | 0.46774 | 19 | 0.6129 |
| 13433 | S16_75520850 | 16 | 75520850 | 4971 | G | A | A | 0.6129 | G | 0.3871 | 16 | 0.51613 |
| 13434 | S16_75521005 | 16 | 75521005 | 155 | T | C | C | 0.6129 | T | 0.3871 | 16 | 0.51613 |
| 13435 | S16_75532725 | 16 | 75532725 | 11720 | G | A | G | 0.69355 | A | 0.30645 | 17 | 0.54839 |
| 13436 | S16_75532784 | 16 | 75532784 | 59 | A | G | A | 0.67742 | G | 0.32258 | 16 | 0.51613 |
| 13437 | S16_75532817 | 16 | 75532817 | 33 | G | C | G | 0.79032 | C | 0.20968 | 13 | 0.41935 |
| 13438 | S16_75550850 | 16 | 75550850 | 18033 | C | T | C | 0.74194 | T | 0.25806 | 14 | 0.45161 |
| 13439 | S16_75656474 | 16 | 75656474 | 105624 | G | C | C | 0.87097 | G | 0.12903 | 8 | 0.25806 |
| 13440 | S16_75936023 | 16 | 75936023 | 279549 | C | T | C | 0.8871 | T | 0.1129 | 7 | 0.22581 |
| 13441 | S16_75936134 | 16 | 75936134 | 111 | C | T | C | 0.95161 | T | 0.04839 | 3 | 0.09677 |
| 13442 | S16_75936135 | 16 | 75936135 | 1 | G | A | A | 0.62903 | G | 0.37097 | 13 | 0.41935 |
| 13443 | S16_76082302 | 16 | 76082302 | 146167 | C | T | C | 0.70968 | T | 0.29032 | 14 | 0.45161 |
| 13444 | S16_76293711 | 16 | 76293711 | 211409 | A | G | A | 0.95161 | G | 0.04839 | 3 | 0.09677 |

| Site number | SNP name | Chromosome | Physical position | Physical distance from the previous SNP | Reference allele | Alternative allele | Major allele | Major allele frequency | Minor allele | Minor allele frequency | Number heterozygous | Heterozygosity |
|---|---|---|---|---|---|---|---|---|---|---|---|---|
| 13445 | S16_76293765 | 16 | 76293765 | 54 | T | C | C | 0.59677 | T | 0.40323 | 15 | 0.48387 |
| 13446 | S16_76293941 | 16 | 76293941 | 176 | G | A | A | 0.59677 | G | 0.40323 | 15 | 0.48387 |
| 13447 | S16_76430293 | 16 | 76430293 | 136352 | G | C | G | 0.95161 | C | 0.04839 | 3 | 0.09677 |
| 13448 | S16_76430525 | 16 | 76430525 | 232 | G | A | G | 0.95161 | A | 0.04839 | 3 | 0.09677 |
| 13449 | S16_76979440 | 16 | 76979440 | 548915 | G | C | G | 0.85484 | C | 0.14516 | 9 | 0.29032 |
| 13450 | S16_76979484 | 16 | 76979484 | 44 | G | A | G | 0.93548 | A | 0.06452 | 4 | 0.12903 |
| 13451 | S16_76979567 | 16 | 76979567 | 83 | G | A | G | 0.85484 | A | 0.14516 | 9 | 0.29032 |
| 13452 | S16_77080007 | 16 | 77080007 | 100440 | T | G | G | 0.77419 | T | 0.22581 | 14 | 0.45161 |
| 13453 | S16_77080027 | 16 | 77080027 | 20 | G | A | G | 0.69355 | A | 0.30645 | 13 | 0.41935 |
| 13454 | S16_77080040 | 16 | 77080040 | 13 | T | G | G | 0.77419 | T | 0.22581 | 14 | 0.45161 |
| 13455 | S16_77080098 | 16 | 77080098 | 58 | G | C | G | 0.91935 | C | 0.08065 | 5 | 0.16129 |
| 13456 | S16_77080148 | 16 | 77080148 | 50 | A | C | A | 0.90323 | C | 0.09677 | 6 | 0.19355 |
| 13457 | S16_77220009 | 16 | 77220009 | 139861 | A | G | G | 0.58065 | A | 0.41935 | 14 | 0.45161 |
| 13458 | S16_77220129 | 16 | 77220129 | 120 | G | A | G | 0.8871 | A | 0.1129 | 7 | 0.22581 |
| 13459 | S16_77342953 | 16 | 77342953 | 122824 | G | A | G | 0.95161 | A | 0.04839 | 3 | 0.09677 |
| 13460 | S16_77343075 | 16 | 77343075 | 122 | T | C | T | 0.87097 | C | 0.12903 | 6 | 0.19355 |
| 13461 | S16_77343080 | 16 | 77343080 | 5 | T | C | T | 0.95161 | C | 0.04839 | 3 | 0.09677 |
| 13462 | S16_77343099 | 16 | 77343099 | 19 | T | C | C | 0.8871 | T | 0.1129 | 7 | 0.22581 |
| 13463 | S16_77343100 | 16 | 77343100 | 1 | G | A | G | 0.87097 | A | 0.12903 | 8 | 0.25806 |
| 13464 | S16_77638523 | 16 | 77638523 | 295423 | T | C | T | 0.79032 | C | 0.20968 | 11 | 0.35484 |
| 13465 | S16_77638582 | 16 | 77638582 | 59 | A | G | G | 0.67742 | A | 0.32258 | 18 | 0.58065 |
| 13466 | S16_77638599 | 16 | 77638599 | 17 | C | T | C | 0.90323 | T | 0.09677 | 6 | 0.19355 |
| 13467 | S16_77660500 | 16 | 77660500 | 21901 | G | A | A | 0.67742 | G | 0.32258 | 14 | 0.45161 |
| 13468 | S16_77660531 | 16 | 77660531 | 31 | G | A | A | 0.67742 | G | 0.32258 | 14 | 0.45161 |
| 13469 | S16_77660573 | 16 | 77660573 | 42 | T | C | C | 0.67742 | T | 0.32258 | 14 | 0.45161 |
| 13470 | S16_77660598 | 16 | 77660598 | 25 | G | A | G | 0.83871 | A | 0.16129 | 6 | 0.19355 |
| 13471 | S16_77660703 | 16 | 77660703 | 105 | C | T | T | 0.67742 | C | 0.32258 | 14 | 0.45161 |
| 13472 | S16_77954601 | 16 | 77954601 | 293898 | G | C | G | 0.8871 | C | 0.1129 | 7 | 0.22581 |
| 13473 | S16_78139629 | 16 | 78139629 | 185028 | A | G | A | 0.95161 | G | 0.04839 | 3 | 0.09677 |
| 13474 | S16_78394629 | 16 | 78394629 | 255000 | G | A | G | 0.8871 | A | 0.1129 | 5 | 0.16129 |
| 13475 | S16_78692128 | 16 | 78692128 | 297499 | C | T | T | 0.51613 | C | 0.48387 | 18 | 0.58065 |
| 13476 | S16_78692288 | 16 | 78692288 | 160 | T | C | T | 0.95161 | C | 0.04839 | 3 | 0.09677 |
| 13477 | S16_78692328 | 16 | 78692328 | 40 | C | T | T | 0.58065 | C | 0.41935 | 16 | 0.51613 |
| 13478 | S16_78785679 | 16 | 78785679 | 93351 | C | A | A | 0.59677 | C | 0.40323 | 15 | 0.48387 |
| 13479 | S16_78785858 | 16 | 78785858 | 179 | T | C | T | 0.80645 | C | 0.19355 | 10 | 0.32258 |
| 13480 | S17_54640 | 17 | 54640 | 0 | T | G | T | 0.62903 | G | 0.37097 | 15 | 0.48387 |
| 13481 | S17_54723 | 17 | 54723 | 83 | T | C | T | 0.6129 | C | 0.3871 | 16 | 0.51613 |
| 13482 | S17_54778 | 17 | 54778 | 55 | A | G | A | 0.64516 | G | 0.35484 | 14 | 0.45161 |
| 13483 | S17_518456 | 17 | 518456 | 463678 | G | A | G | 0.75806 | A | 0.24194 | 15 | 0.48387 |
| 13484 | S17_518460 | 17 | 518460 | 4 | T | C | T | 0.91935 | C | 0.08065 | 5 | 0.16129 |
| 13485 | S17_518472 | 17 | 518472 | 12 | G | T | G | 0.91935 | T | 0.08065 | 5 | 0.16129 |
| 13486 | S17_518553 | 17 | 518553 | 81 | T | C | T | 0.91935 | C | 0.08065 | 5 | 0.16129 |
| 13487 | S17_590381 | 17 | 590381 | 71828 | A | C | A | 0.95161 | C | 0.04839 | 3 | 0.09677 |
| 13488 | S17_605515 | 17 | 605515 | 15134 | C | A | C | 0.90323 | A | 0.09677 | 6 | 0.19355 |
| 13489 | S17_662568 | 17 | 662568 | 57053 | C | T | C | 0.90323 | T | 0.09677 | 4 | 0.12903 |
| 13490 | S17_828333 | 17 | 828333 | 165765 | T | G | T | 0.58065 | G | 0.41935 | 16 | 0.51613 |
| 13491 | S17_842963 | 17 | 842963 | 14630 | G | T | T | 0.83871 | G | 0.16129 | 6 | 0.19355 |
| 13492 | S17_843139 | 17 | 843139 | 176 | A | G | A | 0.91935 | G | 0.08065 | 3 | 0.09677 |
| 13493 | S17_843195 | 17 | 843195 | 56 | G | A | G | 0.91935 | A | 0.08065 | 3 | 0.09677 |
| 13494 | S17_1309721 | 17 | 1309721 | 466526 | G | A | G | 0.95161 | A | 0.04839 | 3 | 0.09677 |
| 13495 | S17_1309819 | 17 | 1309819 | 98 | G | A | G | 0.90323 | A | 0.09677 | 6 | 0.19355 |
| 13496 | S17_1543221 | 17 | 1543221 | 233402 | G | A | G | 0.90323 | A | 0.09677 | 4 | 0.12903 |
| 13497 | S17_1543324 | 17 | 1543324 | 103 | G | A | G | 0.90323 | A | 0.09677 | 6 | 0.19355 |
| 13498 | S17_1543328 | 17 | 1543328 | 4 | A | G | A | 0.93548 | G | 0.06452 | 4 | 0.12903 |

| Site number | SNP name | Chromosome | Physical position | Physical distance from the previous SNP | Reference allele | Alternative allele | Major allele | Major allele frequency | Minor allele | Minor allele frequency | Number heterozygous | Heterozygosity |
|---|---|---|---|---|---|---|---|---|---|---|---|---|
| 13499 | S17_1543404 | 17 | 1543404 | 76 | C | T | C | 0.93548 | T | 0.06452 | 4 | 0.12903 |
| 13500 | S17_1543409 | 17 | 1543409 | 5 | G | A | G | 0.83871 | A | 0.16129 | 8 | 0.25806 |
| 13501 | S17_1622985 | 17 | 1622985 | 79576 | C | T | C | 0.87097 | T | 0.12903 | 4 | 0.12903 |
| 13502 | S17_1819488 | 17 | 1819488 | 196503 | C | T | C | 0.75806 | T | 0.24194 | 11 | 0.35484 |
| 13503 | S17_1819682 | 17 | 1819682 | 194 | C | T | C | 0.75806 | T | 0.24194 | 11 | 0.35484 |
| 13504 | S17_1819709 | 17 | 1819709 | 27 | G | C | C | 0.79032 | G | 0.20968 | 7 | 0.22581 |
| 13505 | S17_2034302 | 17 | 2034302 | 214593 | G | A | G | 0.82258 | A | 0.17742 | 7 | 0.22581 |
| 13506 | S17_2034385 | 17 | 2034385 | 83 | C | T | C | 0.93548 | T | 0.06452 | 4 | 0.12903 |
| 13507 | S17_2034480 | 17 | 2034480 | 95 | C | T | C | 0.6129 | T | 0.3871 | 12 | 0.3871 |
| 13508 | S17_2083023 | 17 | 2083023 | 48543 | T | C | T | 0.95161 | C | 0.04839 | 3 | 0.09677 |
| 13509 | S17_2083142 | 17 | 2083142 | 119 | T | C | T | 0.85484 | C | 0.14516 | 9 | 0.29032 |
| 13510 | S17_2149667 | 17 | 2149667 | 66525 | G | C | G | 0.93548 | C | 0.06452 | 2 | 0.06452 |
| 13511 | S17_2149752 | 17 | 2149752 | 85 | C | T | C | 0.8871 | T | 0.1129 | 7 | 0.22581 |
| 13512 | S17_2169796 | 17 | 2169796 | 20044 | C | T | C | 0.85484 | T | 0.14516 | 7 | 0.22581 |
| 13513 | S17_2169997 | 17 | 2169997 | 201 | A | G | A | 0.72581 | G | 0.27419 | 11 | 0.35484 |
| 13514 | S17_2273700 | 17 | 2273700 | 103703 | G | A | G | 0.79032 | A | 0.20968 | 9 | 0.29032 |
| 13515 | S17_2273874 | 17 | 2273874 | 174 | T | C | C | 0.56452 | T | 0.43548 | 15 | 0.48387 |
| 13516 | S17_2327587 | 17 | 2327587 | 53713 | A | G | A | 0.83871 | G | 0.16129 | 10 | 0.32258 |
| 13517 | S17_2703894 | 17 | 2703894 | 376307 | C | T | T | 0.80645 | C | 0.19355 | 8 | 0.25806 |
| 13518 | S17_2704025 | 17 | 2704025 | 131 | C | G | C | 0.95161 | G | 0.04839 | 3 | 0.09677 |
| 13519 | S17_2957344 | 17 | 2957344 | 253319 | G | A | G | 0.56452 | A | 0.43548 | 15 | 0.48387 |
| 13520 | S17_3054495 | 17 | 3054495 | 97151 | C | T | C | 0.93548 | T | 0.06452 | 4 | 0.12903 |
| 13521 | S17_3054516 | 17 | 3054516 | 21 | C | T | T | 0.6129 | C | 0.3871 | 12 | 0.3871 |
| 13522 | S17_3587920 | 17 | 3587920 | 533404 | C | G | C | 0.83871 | G | 0.16129 | 10 | 0.32258 |
| 13523 | S17_3832772 | 17 | 3832772 | 244852 | T | C | C | 0.82258 | T | 0.17742 | 9 | 0.29032 |
| 13524 | S17_3832913 | 17 | 3832913 | 141 | T | C | C | 0.82258 | T | 0.17742 | 9 | 0.29032 |
| 13525 | S17_3976100 | 17 | 3976100 | 143187 | A | G | A | 0.93548 | G | 0.06452 | 2 | 0.06452 |
| 13526 | S17_4388716 | 17 | 4388716 | 412616 | C | A | C | 0.91935 | A | 0.08065 | 5 | 0.16129 |
| 13527 | S17_4388823 | 17 | 4388823 | 107 | G | C | G | 0.8871 | C | 0.1129 | 7 | 0.22581 |
| 13528 | S17_4388877 | 17 | 4388877 | 54 | G | T | G | 0.85484 | T | 0.14516 | 7 | 0.22581 |
| 13529 | S17_4406073 | 17 | 4406073 | 17196 | T | C | T | 0.8871 | C | 0.1129 | 7 | 0.22581 |
| 13530 | S17_4406258 | 17 | 4406258 | 185 | T | C | C | 0.8871 | T | 0.1129 | 7 | 0.22581 |
| 13531 | S17_4406259 | 17 | 4406259 | 1 | G | A | G | 0.79032 | A | 0.20968 | 11 | 0.35484 |
| 13532 | S17_4429686 | 17 | 4429686 | 23427 | C | T | T | 0.54839 | C | 0.45161 | 10 | 0.32258 |
| 13533 | S17_4844292 | 17 | 4844292 | 414606 | G | A | G | 0.93548 | A | 0.06452 | 4 | 0.12903 |
| 13534 | S17_5375806 | 17 | 5375806 | 531514 | C | A | C | 0.91935 | A | 0.08065 | 3 | 0.09677 |
| 13535 | S17_5672598 | 17 | 5672598 | 296792 | G | C | G | 0.91935 | C | 0.08065 | 5 | 0.16129 |
| 13536 | S17_5672696 | 17 | 5672696 | 98 | G | T | G | 0.95161 | T | 0.04839 | 1 | 0.03226 |
| 13537 | S17_5889496 | 17 | 5889496 | 216800 | C | T | C | 0.93548 | T | 0.06452 | 4 | 0.12903 |
| 13538 | S17_5889602 | 17 | 5889602 | 106 | T | C | T | 0.90323 | C | 0.09677 | 4 | 0.12903 |
| 13539 | S17_5889627 | 17 | 5889627 | 25 | A | G | A | 0.80645 | G | 0.19355 | 12 | 0.3871 |
| 13540 | S17_5889654 | 17 | 5889654 | 27 | C | T | C | 0.80645 | T | 0.19355 | 12 | 0.3871 |
| 13541 | S17_6001439 | 17 | 6001439 | 111785 | C | T | C | 0.95161 | T | 0.04839 | 3 | 0.09677 |
| 13542 | S17_6042349 | 17 | 6042349 | 40910 | T | A | T | 0.80645 | A | 0.19355 | 10 | 0.32258 |
| 13543 | S17_6386632 | 17 | 6386632 | 344283 | G | C | G | 0.82258 | C | 0.17742 | 9 | 0.29032 |
| 13544 | S17_6508954 | 17 | 6508954 | 122322 | T | G | T | 0.70968 | G | 0.29032 | 12 | 0.3871 |
| 13545 | S17_6676448 | 17 | 6676448 | 167494 | C | T | T | 0.66129 | C | 0.33871 | 17 | 0.54839 |
| 13546 | S17_6676623 | 17 | 6676623 | 175 | A | C | C | 0.66129 | A | 0.33871 | 17 | 0.54839 |
| 13547 | S17_6744392 | 17 | 6744392 | 67769 | C | T | C | 0.91935 | T | 0.08065 | 5 | 0.16129 |
| 13548 | S17_6801809 | 17 | 6801809 | 57417 | A | T | A | 0.54839 | T | 0.45161 | 12 | 0.3871 |
| 13549 | S17_6801841 | 17 | 6801841 | 32 | G | A | G | 0.53226 | A | 0.46774 | 13 | 0.41935 |
| 13550 | S17_7249796 | 17 | 7249796 | 447955 | G | A | G | 0.77419 | A | 0.22581 | 10 | 0.32258 |
| 13551 | S17_7249858 | 17 | 7249858 | 62 | C | G | C | 0.72581 | G | 0.27419 | 15 | 0.48387 |
| 13552 | S17_7249937 | 17 | 7249937 | 79 | A | G | G | 0.64516 | A | 0.35484 | 14 | 0.45161 |

| Site number | SNP name | Chromosome | Physical position | Physical distance from the previous SNP | Reference allele | Alternative allele | Major allele | Major allele frequency | Minor allele | Minor allele frequency | Number heterozygous | Heterozygosity |
|---|---|---|---|---|---|---|---|---|---|---|---|---|
| 13553 | S17_7259418 | 17 | 7259418 | 9481 | G | A | G | 0.8871 | A | 0.1129 | 5 | 0.16129 |
| 13554 | S17_7259427 | 17 | 7259427 | 9 | T | C | C | 0.79032 | T | 0.20968 | 7 | 0.22581 |
| 13555 | S17_7259428 | 17 | 7259428 | 1 | G | A | G | 0.87097 | A | 0.12903 | 8 | 0.25806 |
| 13556 | S17_7259492 | 17 | 7259492 | 64 | C | T | C | 0.74194 | T | 0.25806 | 10 | 0.32258 |
| 13557 | S17_8129770 | 17 | 8129770 | 870278 | T | C | T | 0.69355 | C | 0.30645 | 15 | 0.48387 |
| 13558 | S17_8129819 | 17 | 8129819 | 49 | G | C | G | 0.93548 | C | 0.06452 | 4 | 0.12903 |
| 13559 | S17_8129941 | 17 | 8129941 | 122 | G | A | G | 0.93548 | A | 0.06452 | 4 | 0.12903 |
| 13560 | S17_8129970 | 17 | 8129970 | 29 | A | C | A | 0.54839 | C | 0.45161 | 18 | 0.58065 |
| 13561 | S17_8495103 | 17 | 8495103 | 365133 | C | A | C | 0.93548 | A | 0.06452 | 4 | 0.12903 |
| 13562 | S17_8495141 | 17 | 8495141 | 38 | T | C | T | 0.93548 | C | 0.06452 | 4 | 0.12903 |
| 13563 | S17_8779310 | 17 | 8779310 | 284169 | C | T | C | 0.93548 | T | 0.06452 | 4 | 0.12903 |
| 13564 | S17_9279644 | 17 | 9279644 | 500334 | G | C | G | 0.91935 | C | 0.08065 | 5 | 0.16129 |
| 13565 | S17_9448271 | 17 | 9448271 | 168627 | G | T | G | 0.91935 | T | 0.08065 | 3 | 0.09677 |
| 13566 | S17_9565316 | 17 | 9565316 | 117045 | C | T | C | 0.79032 | T | 0.20968 | 13 | 0.41935 |
| 13567 | S17_9565362 | 17 | 9565362 | 46 | C | T | C | 0.79032 | T | 0.20968 | 13 | 0.41935 |
| 13568 | S17_9565423 | 17 | 9565423 | 61 | A | G | G | 0.6129 | A | 0.3871 | 16 | 0.51613 |
| 13569 | S17_9565450 | 17 | 9565450 | 27 | A | G | A | 0.79032 | G | 0.20968 | 13 | 0.41935 |
| 13570 | S17_9639479 | 17 | 9639479 | 74029 | G | A | G | 0.95161 | A | 0.04839 | 3 | 0.09677 |
| 13571 | S17_10209496 | 17 | 10209496 | 570017 | T | C | T | 0.79032 | C | 0.20968 | 11 | 0.35484 |
| 13572 | S17_10209610 | 17 | 10209610 | 114 | G | T | G | 0.93548 | T | 0.06452 | 4 | 0.12903 |
| 13573 | S17_10209678 | 17 | 10209678 | 68 | G | A | G | 0.8871 | A | 0.1129 | 5 | 0.16129 |
| 13574 | S17_10307593 | 17 | 10307593 | 97915 | A | G | A | 0.8871 | G | 0.1129 | 7 | 0.22581 |
| 13575 | S17_10329485 | 17 | 10329485 | 21892 | G | A | G | 0.82258 | A | 0.17742 | 11 | 0.35484 |
| 13576 | S17_10329601 | 17 | 10329601 | 116 | C | T | C | 0.74194 | T | 0.25806 | 14 | 0.45161 |
| 13577 | S17_10329653 | 17 | 10329653 | 52 | A | C | A | 0.87097 | C | 0.12903 | 8 | 0.25806 |
| 13578 | S17_10711480 | 17 | 10711480 | 381827 | G | A | G | 0.64516 | A | 0.35484 | 14 | 0.45161 |
| 13579 | S17_10803487 | 17 | 10803487 | 92007 | T | G | T | 0.69355 | G | 0.30645 | 11 | 0.35484 |
| 13580 | S17_10849521 | 17 | 10849521 | 46034 | C | T | C | 0.87097 | T | 0.12903 | 4 | 0.12903 |
| 13581 | S17_10849599 | 17 | 10849599 | 78 | G | A | G | 0.93548 | A | 0.06452 | 4 | 0.12903 |
| 13582 | S17_11222789 | 17 | 11222789 | 373190 | C | G | C | 0.82258 | G | 0.17742 | 9 | 0.29032 |
| 13583 | S17_11222794 | 17 | 11222794 | 5 | G | C | G | 0.91935 | C | 0.08065 | 5 | 0.16129 |
| 13584 | S17_11222864 | 17 | 11222864 | 70 | G | C | G | 0.51613 | C | 0.48387 | 18 | 0.58065 |
| 13585 | S17_11222883 | 17 | 11222883 | 19 | C | T | C | 0.51613 | T | 0.48387 | 18 | 0.58065 |
| 13586 | S17_11222936 | 17 | 11222936 | 53 | A | G | A | 0.51613 | G | 0.48387 | 18 | 0.58065 |
| 13587 | S17_11222955 | 17 | 11222955 | 19 | G | A | G | 0.91935 | A | 0.08065 | 5 | 0.16129 |
| 13588 | S17_11222957 | 17 | 11222957 | 2 | A | C | A | 0.75806 | C | 0.24194 | 11 | 0.35484 |
| 13589 | S17_11222987 | 17 | 11222987 | 30 | C | T | C | 0.51613 | T | 0.48387 | 18 | 0.58065 |
| 13590 | S17_11542224 | 17 | 11542224 | 319237 | A | G | G | 0.58065 | A | 0.41935 | 16 | 0.51613 |
| 13591 | S17_11686632 | 17 | 11686632 | 144408 | A | T | A | 0.95161 | T | 0.04839 | 3 | 0.09677 |
| 13592 | S17_11809911 | 17 | 11809911 | 123279 | A | G | A | 0.85484 | G | 0.14516 | 9 | 0.29032 |
| 13593 | S17_11809912 | 17 | 11809912 | 1 | T | C | T | 0.87097 | C | 0.12903 | 6 | 0.19355 |
| 13594 | S17_12260189 | 17 | 12260189 | 450277 | T | G | T | 0.70968 | G | 0.29032 | 14 | 0.45161 |
| 13595 | S17_12370277 | 17 | 12370277 | 110088 | C | T | C | 0.83871 | T | 0.16129 | 8 | 0.25806 |
| 13596 | S17_12598279 | 17 | 12598279 | 228002 | A | C | A | 0.58065 | C | 0.41935 | 10 | 0.32258 |
| 13597 | S17_12981295 | 17 | 12981295 | 383016 | C | G | C | 0.80645 | G | 0.19355 | 8 | 0.25806 |
| 13598 | S17_12981488 | 17 | 12981488 | 193 | T | C | T | 0.91935 | C | 0.08065 | 3 | 0.09677 |
| 13599 | S17_13175368 | 17 | 13175368 | 193880 | C | T | T | 0.51613 | C | 0.48387 | 16 | 0.51613 |
| 13600 | S17_13195562 | 17 | 13195562 | 20194 | C | T | C | 0.90323 | T | 0.09677 | 6 | 0.19355 |
| 13601 | S17_13195598 | 17 | 13195598 | 36 | G | A | A | 0.56452 | G | 0.43548 | 19 | 0.6129 |
| 13602 | S17_13216351 | 17 | 13216351 | 20753 | T | G | T | 0.72581 | G | 0.27419 | 15 | 0.48387 |
| 13603 | S17_13216385 | 17 | 13216385 | 34 | C | G | C | 0.74194 | G | 0.25806 | 14 | 0.45161 |
| 13604 | S17_13234351 | 17 | 13234351 | 17966 | G | A | G | 0.91935 | A | 0.08065 | 5 | 0.16129 |
| 13605 | S17_13234462 | 17 | 13234462 | 111 | A | G | A | 0.90323 | G | 0.09677 | 6 | 0.19355 |
| 13606 | S17_13251824 | 17 | 13251824 | 17362 | C | T | C | 0.90323 | T | 0.09677 | 6 | 0.19355 |

| Site number | SNP name | Chromosome | Physical position | Physical distance from the previous SNP | Reference allele | Alternative allele | Major allele | Major allele frequency | Minor allele | Minor allele frequency | Number heterozygous | Heterozygosity |
|---|---|---|---|---|---|---|---|---|---|---|---|---|
| 13607 | S17_13251949 | 17 | 13251949 | 125 | T | C | T | 0.8871 | C | 0.1129 | 7 | 0.22581 |
| 13608 | S17_13471890 | 17 | 13471890 | 219941 | C | T | C | 0.95161 | T | 0.04839 | 3 | 0.09677 |
| 13609 | S17_13471906 | 17 | 13471906 | 16 | C | G | C | 0.82258 | G | 0.17742 | 11 | 0.35484 |
| 13610 | S17_13640374 | 17 | 13640374 | 168468 | G | A | G | 0.93548 | A | 0.06452 | 4 | 0.12903 |
| 13611 | S17_13640483 | 17 | 13640483 | 109 | C | T | C | 0.62903 | T | 0.37097 | 15 | 0.48387 |
| 13612 | S17_13722207 | 17 | 13722207 | 81724 | T | C | C | 0.79032 | T | 0.20968 | 11 | 0.35484 |
| 13613 | S17_13910112 | 17 | 13910112 | 187905 | A | G | A | 0.90323 | G | 0.09677 | 4 | 0.12903 |
| 13614 | S17_14081133 | 17 | 14081133 | 171021 | C | T | C | 0.93548 | T | 0.06452 | 4 | 0.12903 |
| 13615 | S17_14085547 | 17 | 14085547 | 4414 | C | T | C | 0.79032 | T | 0.20968 | 13 | 0.41935 |
| 13616 | S17_14085703 | 17 | 14085703 | 156 | A | G | A | 0.93548 | G | 0.06452 | 4 | 0.12903 |
| 13617 | S17_14534979 | 17 | 14534979 | 449276 | C | T | C | 0.5 | T | 0.5 | 19 | 0.6129 |
| 13618 | S17_14534985 | 17 | 14534985 | 6 | G | C | G | 0.85484 | C | 0.14516 | 7 | 0.22581 |
| 13619 | S17_14535098 | 17 | 14535098 | 113 | C | G | C | 0.91935 | G | 0.08065 | 3 | 0.09677 |
| 13620 | S17_14542002 | 17 | 14542002 | 6904 | G | T | G | 0.69355 | T | 0.30645 | 17 | 0.54839 |
| 13621 | S17_14542020 | 17 | 14542020 | 18 | C | T | C | 0.6129 | T | 0.3871 | 16 | 0.51613 |
| 13622 | S17_14542129 | 17 | 14542129 | 109 | G | A | G | 0.93548 | A | 0.06452 | 4 | 0.12903 |
| 13623 | S17_14542133 | 17 | 14542133 | 4 | A | G | G | 0.54839 | A | 0.45161 | 16 | 0.51613 |
| 13624 | S17_14542195 | 17 | 14542195 | 62 | T | C | T | 0.87097 | C | 0.12903 | 6 | 0.19355 |
| 13625 | S17_14681288 | 17 | 14681288 | 139093 | T | C | C | 0.62903 | T | 0.37097 | 17 | 0.54839 |
| 13626 | S17_14681374 | 17 | 14681374 | 86 | C | T | C | 0.80645 | T | 0.19355 | 10 | 0.32258 |
| 13627 | S17_14749158 | 17 | 14749158 | 67784 | C | T | C | 0.85484 | T | 0.14516 | 9 | 0.29032 |
| 13628 | S17_14749190 | 17 | 14749190 | 32 | T | C | T | 0.80645 | C | 0.19355 | 6 | 0.19355 |
| 13629 | S17_14923498 | 17 | 14923498 | 174308 | G | A | G | 0.59677 | A | 0.40323 | 13 | 0.41935 |
| 13630 | S17_15036761 | 17 | 15036761 | 113263 | G | A | G | 0.74194 | A | 0.25806 | 10 | 0.32258 |
| 13631 | S17_15036800 | 17 | 15036800 | 39 | C | T | C | 0.8871 | T | 0.1129 | 5 | 0.16129 |
| 13632 | S17_15036867 | 17 | 15036867 | 67 | C | T | C | 0.74194 | T | 0.25806 | 10 | 0.32258 |
| 13633 | S17_15036976 | 17 | 15036976 | 109 | A | G | A | 0.74194 | G | 0.25806 | 10 | 0.32258 |
| 13634 | S17_15051583 | 17 | 15051583 | 14607 | A | G | A | 0.95161 | G | 0.04839 | 3 | 0.09677 |
| 13635 | S17_15051599 | 17 | 15051599 | 16 | G | A | G | 0.90323 | A | 0.09677 | 4 | 0.12903 |
| 13636 | S17_15091883 | 17 | 15091883 | 40284 | G | A | G | 0.90323 | A | 0.09677 | 6 | 0.19355 |
| 13637 | S17_15091985 | 17 | 15091985 | 102 | T | C | C | 0.82258 | T | 0.17742 | 7 | 0.22581 |
| 13638 | S17_15091986 | 17 | 15091986 | 1 | G | A | A | 0.82258 | G | 0.17742 | 7 | 0.22581 |
| 13639 | S17_15275808 | 17 | 15275808 | 183822 | A | C | A | 0.93548 | C | 0.06452 | 4 | 0.12903 |
| 13640 | S17_16003716 | 17 | 16003716 | 727908 | C | T | C | 0.87097 | T | 0.12903 | 4 | 0.12903 |
| 13641 | S17_16003763 | 17 | 16003763 | 47 | G | A | G | 0.93548 | A | 0.06452 | 4 | 0.12903 |
| 13642 | S17_16252975 | 17 | 16252975 | 249212 | G | T | G | 0.59677 | T | 0.40323 | 13 | 0.41935 |
| 13643 | S17_16482013 | 17 | 16482013 | 229038 | G | A | A | 0.90323 | G | 0.09677 | 6 | 0.19355 |
| 13644 | S17_16529599 | 17 | 16529599 | 47586 | G | T | G | 0.91935 | T | 0.08065 | 5 | 0.16129 |
| 13645 | S17_16647491 | 17 | 16647491 | 117892 | G | A | G | 0.95161 | A | 0.04839 | 3 | 0.09677 |
| 13646 | S17_16919226 | 17 | 16919226 | 271735 | G | T | G | 0.54839 | T | 0.45161 | 14 | 0.45161 |
| 13647 | S17_17795129 | 17 | 17795129 | 875903 | A | G | G | 0.87097 | A | 0.12903 | 8 | 0.25806 |
| 13648 | S17_17795211 | 17 | 17795211 | 82 | G | A | G | 0.91935 | A | 0.08065 | 5 | 0.16129 |
| 13649 | S17_18202374 | 17 | 18202374 | 407163 | G | A | G | 0.83871 | A | 0.16129 | 6 | 0.19355 |
| 13650 | S17_18202473 | 17 | 18202473 | 99 | T | C | T | 0.54839 | C | 0.45161 | 14 | 0.45161 |
| 13651 | S17_18631327 | 17 | 18631327 | 428854 | T | C | C | 0.58065 | T | 0.41935 | 12 | 0.3871 |
| 13652 | S17_18798100 | 17 | 18798100 | 166773 | G | A | G | 0.77419 | A | 0.22581 | 12 | 0.3871 |
| 13653 | S17_18818490 | 17 | 18818490 | 20390 | C | T | C | 0.93548 | T | 0.06452 | 4 | 0.12903 |
| 13654 | S17_19215501 | 17 | 19215501 | 397011 | G | T | G | 0.91935 | T | 0.08065 | 5 | 0.16129 |
| 13655 | S17_19277720 | 17 | 19277720 | 62219 | G | A | A | 0.74194 | G | 0.25806 | 16 | 0.51613 |
| 13656 | S17_19308123 | 17 | 19308123 | 30403 | C | T | C | 0.87097 | T | 0.12903 | 6 | 0.19355 |
| 13657 | S17_19308134 | 17 | 19308134 | 11 | C | A | C | 0.58065 | A | 0.41935 | 16 | 0.51613 |
| 13658 | S17_19308135 | 17 | 19308135 | 1 | G | A | G | 0.87097 | A | 0.12903 | 6 | 0.19355 |
| 13659 | S17_19380015 | 17 | 19380015 | 71880 | C | T | C | 0.72581 | T | 0.27419 | 11 | 0.35484 |
| 13660 | S17_19380028 | 17 | 19380028 | 13 | A | G | A | 0.72581 | G | 0.27419 | 11 | 0.35484 |

| Site number | SNP name | Chromosome | Physical position | Physical distance from the previous SNP | Reference allele | Alternative allele | Major allele | Major allele frequency | Minor allele | Minor allele frequency | Number heterozygous | Heterozygosity |
|---|---|---|---|---|---|---|---|---|---|---|---|---|
| 13661 | S17_19380055 | 17 | 19380055 | 27 | G | A | A | 0.58065 | G | 0.41935 | 14 | 0.45161 |
| 13662 | S17_19441616 | 17 | 19441616 | 61561 | C | T | C | 0.64516 | T | 0.35484 | 12 | 0.3871 |
| 13663 | S17_19645827 | 17 | 19645827 | 204211 | A | G | G | 0.91935 | A | 0.08065 | 3 | 0.09677 |
| 13664 | S17_19674490 | 17 | 19674490 | 28663 | G | A | G | 0.82258 | A | 0.17742 | 9 | 0.29032 |
| 13665 | S17_19674503 | 17 | 19674503 | 13 | A | G | A | 0.91935 | G | 0.08065 | 3 | 0.09677 |
| 13666 | S17_19674524 | 17 | 19674524 | 21 | G | T | G | 0.82258 | T | 0.17742 | 9 | 0.29032 |
| 13667 | S17_19849298 | 17 | 19849298 | 174774 | G | A | G | 0.91935 | A | 0.08065 | 3 | 0.09677 |
| 13668 | S17_19857935 | 17 | 19857935 | 8637 | G | C | G | 0.87097 | C | 0.12903 | 6 | 0.19355 |
| 13669 | S17_19858070 | 17 | 19858070 | 135 | G | A | G | 0.83871 | A | 0.16129 | 6 | 0.19355 |
| 13670 | S17_19858071 | 17 | 19858071 | 1 | G | A | G | 0.83871 | A | 0.16129 | 6 | 0.19355 |
| 13671 | S17_19858155 | 17 | 19858155 | 84 | T | C | T | 0.77419 | C | 0.22581 | 12 | 0.3871 |
| 13672 | S17_20045930 | 17 | 20045930 | 187775 | A | G | G | 0.70968 | A | 0.29032 | 12 | 0.3871 |
| 13673 | S17_20048027 | 17 | 20048027 | 2097 | T | C | T | 0.83871 | C | 0.16129 | 10 | 0.32258 |
| 13674 | S17_20064771 | 17 | 20064771 | 16744 | G | T | G | 0.93548 | T | 0.06452 | 4 | 0.12903 |
| 13675 | S17_20064830 | 17 | 20064830 | 59 | C | T | C | 0.66129 | T | 0.33871 | 13 | 0.41935 |
| 13676 | S17_20064856 | 17 | 20064856 | 26 | A | G | A | 0.85484 | G | 0.14516 | 7 | 0.22581 |
| 13677 | S17_20069388 | 17 | 20069388 | 4532 | G | A | G | 0.95161 | A | 0.04839 | 3 | 0.09677 |
| 13678 | S17_20069420 | 17 | 20069420 | 32 | C | A | C | 0.8871 | A | 0.1129 | 7 | 0.22581 |
| 13679 | S17_20069497 | 17 | 20069497 | 77 | G | A | G | 0.8871 | A | 0.1129 | 7 | 0.22581 |
| 13680 | S17_20195882 | 17 | 20195882 | 126385 | A | G | A | 0.93548 | G | 0.06452 | 4 | 0.12903 |
| 13681 | S17_20195895 | 17 | 20195895 | 13 | G | A | G | 0.85484 | A | 0.14516 | 9 | 0.29032 |
| 13682 | S17_20195965 | 17 | 20195965 | 70 | T | A | A | 0.66129 | T | 0.33871 | 13 | 0.41935 |
| 13683 | S17_20198612 | 17 | 20198612 | 2647 | G | T | G | 0.85484 | T | 0.14516 | 9 | 0.29032 |
| 13684 | S17_20357043 | 17 | 20357043 | 158431 | T | C | T | 0.74194 | C | 0.25806 | 14 | 0.45161 |
| 13685 | S17_20596297 | 17 | 20596297 | 239254 | C | G | C | 0.91935 | G | 0.08065 | 5 | 0.16129 |
| 13686 | S17_20724048 | 17 | 20724048 | 127751 | A | G | A | 0.70968 | G | 0.29032 | 14 | 0.45161 |
| 13687 | S17_20724275 | 17 | 20724275 | 227 | T | C | C | 0.62903 | T | 0.37097 | 17 | 0.54839 |
| 13688 | S17_20993266 | 17 | 20993266 | 268991 | A | T | A | 0.93548 | T | 0.06452 | 4 | 0.12903 |
| 13689 | S17_21148460 | 17 | 21148460 | 155194 | G | A | G | 0.95161 | A | 0.04839 | 3 | 0.09677 |
| 13690 | S17_21148489 | 17 | 21148489 | 29 | C | T | C | 0.74194 | T | 0.25806 | 14 | 0.45161 |
| 13691 | S17_21285565 | 17 | 21285565 | 137076 | G | C | G | 0.53226 | C | 0.46774 | 17 | 0.54839 |
| 13692 | S17_21285754 | 17 | 21285754 | 189 | A | G | A | 0.87097 | G | 0.12903 | 8 | 0.25806 |
| 13693 | S17_21305133 | 17 | 21305133 | 19379 | C | A | C | 0.70968 | A | 0.29032 | 12 | 0.3871 |
| 13694 | S17_21307424 | 17 | 21307424 | 2291 | T | C | T | 0.72581 | C | 0.27419 | 11 | 0.35484 |
| 13695 | S17_21987806 | 17 | 21987806 | 680382 | C | T | C | 0.93548 | T | 0.06452 | 4 | 0.12903 |
| 13696 | S17_21987829 | 17 | 21987829 | 23 | C | A | C | 0.93548 | A | 0.06452 | 4 | 0.12903 |
| 13697 | S17_21987836 | 17 | 21987836 | 7 | G | A | G | 0.93548 | A | 0.06452 | 4 | 0.12903 |
| 13698 | S17_22051436 | 17 | 22051436 | 63600 | T | C | T | 0.51613 | C | 0.48387 | 20 | 0.64516 |
| 13699 | S17_22051451 | 17 | 22051451 | 15 | G | T | T | 0.83871 | G | 0.16129 | 10 | 0.32258 |
| 13700 | S17_22189868 | 17 | 22189868 | 138417 | T | A | T | 0.80645 | A | 0.19355 | 12 | 0.3871 |
| 13701 | S17_22271827 | 17 | 22271827 | 81959 | T | G | T | 0.85484 | G | 0.14516 | 9 | 0.29032 |
| 13702 | S17_22351335 | 17 | 22351335 | 79508 | G | A | G | 0.75806 | A | 0.24194 | 9 | 0.29032 |
| 13703 | S17_22351338 | 17 | 22351338 | 3 | G | A | A | 0.54839 | G | 0.45161 | 16 | 0.51613 |
| 13704 | S17_22351347 | 17 | 22351347 | 9 | A | G | G | 0.79032 | A | 0.20968 | 9 | 0.29032 |
| 13705 | S17_22351395 | 17 | 22351395 | 48 | C | T | C | 0.75806 | T | 0.24194 | 9 | 0.29032 |
| 13706 | S17_22434761 | 17 | 22434761 | 83366 | G | T | G | 0.93548 | T | 0.06452 | 4 | 0.12903 |
| 13707 | S17_22434765 | 17 | 22434765 | 4 | A | G | A | 0.93548 | G | 0.06452 | 4 | 0.12903 |
| 13708 | S17_22459992 | 17 | 22459992 | 25227 | C | T | C | 0.82258 | T | 0.17742 | 7 | 0.22581 |
| 13709 | S17_22505044 | 17 | 22505044 | 45052 | A | G | A | 0.93548 | G | 0.06452 | 4 | 0.12903 |
| 13710 | S17_22506886 | 17 | 22506886 | 1842 | A | G | A | 0.93548 | G | 0.06452 | 4 | 0.12903 |
| 13711 | S17_22557026 | 17 | 22557026 | 50140 | G | A | G | 0.82258 | A | 0.17742 | 11 | 0.35484 |
| 13712 | S17_22611549 | 17 | 22611549 | 54523 | C | T | C | 0.54839 | T | 0.45161 | 14 | 0.45161 |
| 13713 | S17_22617743 | 17 | 22617743 | 6194 | A | G | G | 0.66129 | A | 0.33871 | 13 | 0.41935 |
| 13714 | S17_22617815 | 17 | 22617815 | 72 | C | T | C | 0.8871 | T | 0.1129 | 5 | 0.16129 |

| Site number | SNP name | Chromosome | Physical position | Physical distance from the previous SNP | Reference allele | Alternative allele | Major allele | Major allele frequency | Minor allele | Minor allele frequency | Number heterozygous | Heterozygosity |
|---|---|---|---|---|---|---|---|---|---|---|---|---|
| 13715 | S17_22625585 | 17 | 22625585 | 7770 | A | G | A | 0.87097 | G | 0.12903 | 6 | 0.19355 |
| 13716 | S17_22778864 | 17 | 22778864 | 153279 | G | A | G | 0.80645 | A | 0.19355 | 8 | 0.25806 |
| 13717 | S17_22855610 | 17 | 22855610 | 76746 | G | A | G | 0.56452 | A | 0.43548 | 13 | 0.41935 |
| 13718 | S17_22855720 | 17 | 22855720 | 110 | T | C | T | 0.56452 | C | 0.43548 | 13 | 0.41935 |
| 13719 | S17_23236726 | 17 | 23236726 | 381006 | T | C | T | 0.79032 | C | 0.20968 | 9 | 0.29032 |
| 13720 | S17_23713536 | 17 | 23713536 | 476810 | C | T | C | 0.90323 | T | 0.09677 | 6 | 0.19355 |
| 13721 | S17_23737455 | 17 | 23737455 | 23919 | C | A | C | 0.91935 | A | 0.08065 | 5 | 0.16129 |
| 13722 | S17_23737515 | 17 | 23737515 | 60 | G | T | G | 0.91935 | T | 0.08065 | 5 | 0.16129 |
| 13723 | S17_23767850 | 17 | 23767850 | 30335 | G | A | G | 0.85484 | A | 0.14516 | 7 | 0.22581 |
| 13724 | S17_23768047 | 17 | 23768047 | 197 | G | A | G | 0.93548 | A | 0.06452 | 2 | 0.06452 |
| 13725 | S17_23939801 | 17 | 23939801 | 171754 | G | A | G | 0.79032 | A | 0.20968 | 11 | 0.35484 |
| 13726 | S17_23939819 | 17 | 23939819 | 18 | C | T | C | 0.66129 | T | 0.33871 | 15 | 0.48387 |
| 13727 | S17_23939868 | 17 | 23939868 | 49 | T | C | T | 0.91935 | C | 0.08065 | 3 | 0.09677 |
| 13728 | S17_23939915 | 17 | 23939915 | 47 | A | C | A | 0.77419 | C | 0.22581 | 12 | 0.3871 |
| 13729 | S17_23939952 | 17 | 23939952 | 37 | C | T | C | 0.95161 | T | 0.04839 | 1 | 0.03226 |
| 13730 | S17_23939989 | 17 | 23939989 | 37 | C | T | C | 0.8871 | T | 0.1129 | 5 | 0.16129 |
| 13731 | S17_24055775 | 17 | 24055775 | 115786 | G | A | G | 0.74194 | A | 0.25806 | 10 | 0.32258 |
| 13732 | S17_24055845 | 17 | 24055845 | 70 | C | T | C | 0.51613 | T | 0.48387 | 12 | 0.3871 |
| 13733 | S17_24055850 | 17 | 24055850 | 5 | G | A | G | 0.90323 | A | 0.09677 | 4 | 0.12903 |
| 13734 | S17_24055864 | 17 | 24055864 | 14 | C | A | C | 0.91935 | A | 0.08065 | 3 | 0.09677 |
| 13735 | S17_24097447 | 17 | 24097447 | 41583 | A | G | G | 0.62903 | A | 0.37097 | 11 | 0.35484 |
| 13736 | S17_24097497 | 17 | 24097497 | 50 | C | A | A | 0.5 | C | 0.5 | 11 | 0.35484 |
| 13737 | S17_24097506 | 17 | 24097506 | 9 | G | A | G | 0.90323 | A | 0.09677 | 4 | 0.12903 |
| 13738 | S17_24097551 | 17 | 24097551 | 45 | C | T | T | 0.59677 | C | 0.40323 | 11 | 0.35484 |
| 13739 | S17_24097663 | 17 | 24097663 | 112 | G | A | A | 0.59677 | G | 0.40323 | 11 | 0.35484 |
| 13740 | S17_24535558 | 17 | 24535558 | 437895 | C | A | C | 0.8871 | A | 0.1129 | 5 | 0.16129 |
| 13741 | S17_24535618 | 17 | 24535618 | 60 | G | A | G | 0.56452 | A | 0.43548 | 13 | 0.41935 |
| 13742 | S17_24535708 | 17 | 24535708 | 90 | G | A | G | 0.83871 | A | 0.16129 | 10 | 0.32258 |
| 13743 | S17_24735234 | 17 | 24735234 | 199526 | A | T | T | 0.59677 | A | 0.40323 | 17 | 0.54839 |
| 13744 | S17_24735385 | 17 | 24735385 | 151 | C | T | C | 0.93548 | T | 0.06452 | 4 | 0.12903 |
| 13745 | S17_24892086 | 17 | 24892086 | 156701 | A | G | A | 0.75806 | G | 0.24194 | 13 | 0.41935 |
| 13746 | S17_24892203 | 17 | 24892203 | 117 | C | T | C | 0.56452 | T | 0.43548 | 11 | 0.35484 |
| 13747 | S17_25004991 | 17 | 25004991 | 112788 | C | T | C | 0.93548 | T | 0.06452 | 2 | 0.06452 |
| 13748 | S17_25005034 | 17 | 25005034 | 43 | C | T | C | 0.91935 | T | 0.08065 | 5 | 0.16129 |
| 13749 | S17_25005051 | 17 | 25005051 | 17 | T | C | T | 0.87097 | C | 0.12903 | 8 | 0.25806 |
| 13750 | S17_25219062 | 17 | 25219062 | 214011 | T | G | T | 0.93548 | G | 0.06452 | 4 | 0.12903 |
| 13751 | S17_25219083 | 17 | 25219083 | 21 | T | A | A | 0.70968 | T | 0.29032 | 14 | 0.45161 |
| 13752 | S17_25219134 | 17 | 25219134 | 51 | T | C | C | 0.70968 | T | 0.29032 | 14 | 0.45161 |
| 13753 | S17_25323969 | 17 | 25323969 | 104835 | C | T | C | 0.91935 | T | 0.08065 | 5 | 0.16129 |
| 13754 | S17_25324019 | 17 | 25324019 | 50 | G | A | G | 0.91935 | A | 0.08065 | 5 | 0.16129 |
| 13755 | S17_25324045 | 17 | 25324045 | 26 | C | T | C | 0.5 | T | 0.5 | 17 | 0.54839 |
| 13756 | S17_25549101 | 17 | 25549101 | 225056 | T | C | C | 0.82258 | T | 0.17742 | 11 | 0.35484 |
| 13757 | S17_25549123 | 17 | 25549123 | 22 | G | A | A | 0.82258 | G | 0.17742 | 11 | 0.35484 |
| 13758 | S17_25549174 | 17 | 25549174 | 51 | T | C | C | 0.82258 | T | 0.17742 | 11 | 0.35484 |
| 13759 | S17_25549188 | 17 | 25549188 | 14 | G | C | G | 0.90323 | C | 0.09677 | 6 | 0.19355 |
| 13760 | S17_25875094 | 17 | 25875094 | 325906 | A | C | A | 0.93548 | C | 0.06452 | 4 | 0.12903 |
| 13761 | S17_25875143 | 17 | 25875143 | 49 | C | T | T | 0.59677 | C | 0.40323 | 21 | 0.67742 |
| 13762 | S17_26069869 | 17 | 26069869 | 194726 | G | A | G | 0.53226 | A | 0.46774 | 15 | 0.48387 |
| 13763 | S17_26069896 | 17 | 26069896 | 27 | T | C | T | 0.8871 | C | 0.1129 | 7 | 0.22581 |
| 13764 | S17_26611937 | 17 | 26611937 | 542041 | G | A | G | 0.58065 | A | 0.41935 | 14 | 0.45161 |
| 13765 | S17_26611985 | 17 | 26611985 | 48 | G | A | G | 0.74194 | A | 0.25806 | 12 | 0.3871 |
| 13766 | S17_26980399 | 17 | 26980399 | 368414 | T | C | T | 0.90323 | C | 0.09677 | 6 | 0.19355 |
| 13767 | S17_27915476 | 17 | 27915476 | 935077 | C | T | C | 0.95161 | T | 0.04839 | 3 | 0.09677 |
| 13768 | S17_27915604 | 17 | 27915604 | 128 | T | C | T | 0.64516 | C | 0.35484 | 18 | 0.58065 |

| Site number | SNP name | Chromosome | Physical position | Physical distance from the previous SNP | Reference allele | Alternative allele | Major allele | Major allele frequency | Minor allele | Minor allele frequency | Number heterozygous | Heterozygosity |
|---|---|---|---|---|---|---|---|---|---|---|---|---|
| 13769 | S17_28064739 | 17 | 28064739 | 149135 | T | C | T | 0.66129 | C | 0.33871 | 17 | 0.54839 |
| 13770 | S17_28768181 | 17 | 28768181 | 703442 | T | C | T | 0.58065 | C | 0.41935 | 16 | 0.51613 |
| 13771 | S17_28768207 | 17 | 28768207 | 26 | C | T | C | 0.58065 | T | 0.41935 | 16 | 0.51613 |
| 13772 | S17_28768339 | 17 | 28768339 | 132 | T | C | T | 0.95161 | C | 0.04839 | 3 | 0.09677 |
| 13773 | S17_29154040 | 17 | 29154040 | 385701 | A | G | G | 0.54839 | A | 0.45161 | 16 | 0.51613 |
| 13774 | S17_29154047 | 17 | 29154047 | 7 | C | T | T | 0.56452 | C | 0.43548 | 17 | 0.54839 |
| 13775 | S17_29154116 | 17 | 29154116 | 69 | G | A | A | 0.56452 | G | 0.43548 | 17 | 0.54839 |
| 13776 | S17_29154122 | 17 | 29154122 | 6 | C | T | C | 0.93548 | T | 0.06452 | 4 | 0.12903 |
| 13777 | S17_30281622 | 17 | 30281622 | 1127500 | G | A | G | 0.82258 | A | 0.17742 | 7 | 0.22581 |
| 13778 | S17_30281674 | 17 | 30281674 | 52 | A | G | A | 0.6129 | G | 0.3871 | 14 | 0.45161 |
| 13779 | S17_30281718 | 17 | 30281718 | 44 | A | C | C | 0.62903 | A | 0.37097 | 15 | 0.48387 |
| 13780 | S17_30281740 | 17 | 30281740 | 22 | T | C | C | 0.6129 | T | 0.3871 | 16 | 0.51613 |
| 13781 | S17_30626486 | 17 | 30626486 | 344746 | G | A | G | 0.82258 | A | 0.17742 | 11 | 0.35484 |
| 13782 | S17_31057043 | 17 | 31057043 | 430557 | G | A | G | 0.87097 | A | 0.12903 | 6 | 0.19355 |
| 13783 | S17_31057052 | 17 | 31057052 | 9 | T | G | T | 0.66129 | G | 0.33871 | 15 | 0.48387 |
| 13784 | S17_31057177 | 17 | 31057177 | 125 | C | T | C | 0.95161 | T | 0.04839 | 3 | 0.09677 |
| 13785 | S17_31057206 | 17 | 31057206 | 29 | A | G | A | 0.66129 | G | 0.33871 | 15 | 0.48387 |
| 13786 | S17_31057230 | 17 | 31057230 | 24 | G | T | G | 0.87097 | T | 0.12903 | 6 | 0.19355 |
| 13787 | S17_31351825 | 17 | 31351825 | 294595 | C | T | C | 0.80645 | T | 0.19355 | 8 | 0.25806 |
| 13788 | S17_31351878 | 17 | 31351878 | 53 | T | A | T | 0.80645 | A | 0.19355 | 8 | 0.25806 |
| 13789 | S17_32565965 | 17 | 32565965 | 1214087 | C | T | C | 0.53226 | T | 0.46774 | 13 | 0.41935 |
| 13790 | S17_32566088 | 17 | 32566088 | 123 | C | T | C | 0.79032 | T | 0.20968 | 13 | 0.41935 |
| 13791 | S17_32566157 | 17 | 32566157 | 69 | G | A | G | 0.70968 | A | 0.29032 | 16 | 0.51613 |
| 13792 | S17_32908705 | 17 | 32908705 | 342548 | A | G | A | 0.6129 | G | 0.3871 | 16 | 0.51613 |
| 13793 | S17_32908712 | 17 | 32908712 | 7 | C | T | C | 0.77419 | T | 0.22581 | 12 | 0.3871 |
| 13794 | S17_32908818 | 17 | 32908818 | 106 | G | C | G | 0.80645 | C | 0.19355 | 6 | 0.19355 |
| 13795 | S17_32908876 | 17 | 32908876 | 58 | C | T | C | 0.93548 | T | 0.06452 | 4 | 0.12903 |
| 13796 | S17_32908900 | 17 | 32908900 | 24 | T | C | T | 0.64516 | C | 0.35484 | 16 | 0.51613 |
| 13797 | S17_33466510 | 17 | 33466510 | 557610 | G | A | G | 0.93548 | A | 0.06452 | 4 | 0.12903 |
| 13798 | S17_33466716 | 17 | 33466716 | 206 | G | A | G | 0.93548 | A | 0.06452 | 2 | 0.06452 |
| 13799 | S17_33714852 | 17 | 33714852 | 248136 | G | C | G | 0.8871 | C | 0.1129 | 7 | 0.22581 |
| 13800 | S17_33999919 | 17 | 33999919 | 285067 | G | A | A | 0.77419 | G | 0.22581 | 10 | 0.32258 |
| 13801 | S17_33999941 | 17 | 33999941 | 22 | T | C | C | 0.83871 | T | 0.16129 | 8 | 0.25806 |
| 13802 | S17_34074389 | 17 | 34074389 | 74448 | A | G | A | 0.90323 | G | 0.09677 | 6 | 0.19355 |
| 13803 | S17_34074409 | 17 | 34074409 | 20 | G | T | G | 0.72581 | T | 0.27419 | 9 | 0.29032 |
| 13804 | S17_34074500 | 17 | 34074500 | 91 | G | A | G | 0.90323 | A | 0.09677 | 6 | 0.19355 |
| 13805 | S17_34074592 | 17 | 34074592 | 92 | T | C | T | 0.90323 | C | 0.09677 | 6 | 0.19355 |
| 13806 | S17_34074622 | 17 | 34074622 | 30 | A | G | A | 0.8871 | G | 0.1129 | 7 | 0.22581 |
| 13807 | S17_35025773 | 17 | 35025773 | 951151 | C | T | C | 0.82258 | T | 0.17742 | 7 | 0.22581 |
| 13808 | S17_35025835 | 17 | 35025835 | 62 | G | A | G | 0.82258 | A | 0.17742 | 7 | 0.22581 |
| 13809 | S17_35025890 | 17 | 35025890 | 55 | G | A | G | 0.82258 | A | 0.17742 | 7 | 0.22581 |
| 13810 | S17_36057857 | 17 | 36057857 | 1031967 | G | A | G | 0.79032 | A | 0.20968 | 9 | 0.29032 |
| 13811 | S17_36152072 | 17 | 36152072 | 94215 | G | T | G | 0.79032 | T | 0.20968 | 9 | 0.29032 |
| 13812 | S17_37285858 | 17 | 37285858 | 1133786 | C | G | C | 0.80645 | G | 0.19355 | 8 | 0.25806 |
| 13813 | S17_37430241 | 17 | 37430241 | 144383 | A | C | C | 0.6129 | A | 0.3871 | 12 | 0.3871 |
| 13814 | S17_37430314 | 17 | 37430314 | 73 | A | G | G | 0.91935 | A | 0.08065 | 5 | 0.16129 |
| 13815 | S17_38092903 | 17 | 38092903 | 662589 | T | C | T | 0.80645 | C | 0.19355 | 8 | 0.25806 |
| 13816 | S17_38092956 | 17 | 38092956 | 53 | T | G | T | 0.80645 | G | 0.19355 | 8 | 0.25806 |
| 13817 | S17_38093090 | 17 | 38093090 | 134 | G | A | G | 0.80645 | A | 0.19355 | 8 | 0.25806 |
| 13818 | S17_38093135 | 17 | 38093135 | 45 | A | G | A | 0.80645 | G | 0.19355 | 8 | 0.25806 |
| 13819 | S17_38219334 | 17 | 38219334 | 126199 | C | T | C | 0.90323 | T | 0.09677 | 6 | 0.19355 |
| 13820 | S17_38219465 | 17 | 38219465 | 131 | T | C | T | 0.90323 | C | 0.09677 | 6 | 0.19355 |
| 13821 | S17_38219478 | 17 | 38219478 | 13 | A | G | A | 0.90323 | G | 0.09677 | 6 | 0.19355 |
| 13822 | S17_38219485 | 17 | 38219485 | 7 | T | C | T | 0.90323 | C | 0.09677 | 6 | 0.19355 |

| Site number | SNP name | Chromosome | Physical position | Physical distance from the previous SNP | Reference allele | Alternative allele | Major allele | Major allele frequency | Minor allele | Minor allele frequency | Number heterozygous | Heterozygosity |
|---|---|---|---|---|---|---|---|---|---|---|---|---|
| 13823 | S17_38219487 | 17 | 38219487 | 2 | C | T | C | 0.90323 | T | 0.09677 | 6 | 0.19355 |
| 13824 | S17_38219492 | 17 | 38219492 | 5 | A | C | A | 0.90323 | C | 0.09677 | 6 | 0.19355 |
| 13825 | S17_38219493 | 17 | 38219493 | 1 | G | A | G | 0.90323 | A | 0.09677 | 6 | 0.19355 |
| 13826 | S17_38219494 | 17 | 38219494 | 1 | C | T | C | 0.90323 | T | 0.09677 | 6 | 0.19355 |
| 13827 | S17_38219556 | 17 | 38219556 | 62 | G | A | G | 0.70968 | A | 0.29032 | 12 | 0.3871 |
| 13828 | S17_38219558 | 17 | 38219558 | 2 | A | T | A | 0.90323 | T | 0.09677 | 6 | 0.19355 |
| 13829 | S17_38219570 | 17 | 38219570 | 12 | A | G | A | 0.90323 | G | 0.09677 | 6 | 0.19355 |
| 13830 | S17_38219577 | 17 | 38219577 | 7 | G | A | G | 0.90323 | A | 0.09677 | 6 | 0.19355 |
| 13831 | S17_38684457 | 17 | 38684457 | 464880 | G | A | G | 0.79032 | A | 0.20968 | 9 | 0.29032 |
| 13832 | S17_38969143 | 17 | 38969143 | 284686 | C | T | C | 0.90323 | T | 0.09677 | 4 | 0.12903 |
| 13833 | S17_41403014 | 17 | 41403014 | 2433871 | A | G | A | 0.83871 | G | 0.16129 | 8 | 0.25806 |
| 13834 | S17_41403047 | 17 | 41403047 | 33 | A | G | A | 0.87097 | G | 0.12903 | 8 | 0.25806 |
| 13835 | S17_41403231 | 17 | 41403231 | 184 | A | G | A | 0.82258 | G | 0.17742 | 7 | 0.22581 |
| 13836 | S17_41539162 | 17 | 41539162 | 135931 | T | C | T | 0.66129 | C | 0.33871 | 11 | 0.35484 |
| 13837 | S17_42239558 | 17 | 42239558 | 700396 | G | A | A | 0.77419 | G | 0.22581 | 12 | 0.3871 |
| 13838 | S17_42239607 | 17 | 42239607 | 49 | C | T | C | 0.8871 | T | 0.1129 | 7 | 0.22581 |
| 13839 | S17_42239638 | 17 | 42239638 | 31 | G | A | G | 0.8871 | A | 0.1129 | 7 | 0.22581 |
| 13840 | S17_42239643 | 17 | 42239643 | 5 | A | G | G | 0.80645 | A | 0.19355 | 10 | 0.32258 |
| 13841 | S17_42239657 | 17 | 42239657 | 14 | A | G | A | 0.85484 | G | 0.14516 | 9 | 0.29032 |
| 13842 | S17_42249041 | 17 | 42249041 | 9384 | C | T | C | 0.6129 | T | 0.3871 | 18 | 0.58065 |
| 13843 | S17_42307736 | 17 | 42307736 | 58695 | T | C | C | 0.53226 | T | 0.46774 | 17 | 0.54839 |
| 13844 | S17_42365052 | 17 | 42365052 | 57316 | G | T | G | 0.91935 | T | 0.08065 | 5 | 0.16129 |
| 13845 | S17_44721575 | 17 | 44721575 | 2356523 | T | G | T | 0.67742 | G | 0.32258 | 16 | 0.51613 |
| 13846 | S17_45049177 | 17 | 45049177 | 327602 | C | G | C | 0.90323 | G | 0.09677 | 6 | 0.19355 |
| 13847 | S17_46058493 | 17 | 46058493 | 1009316 | T | G | T | 0.87097 | G | 0.12903 | 6 | 0.19355 |
| 13848 | S17_46058562 | 17 | 46058562 | 69 | C | A | C | 0.95161 | A | 0.04839 | 3 | 0.09677 |
| 13849 | S17_46133821 | 17 | 46133821 | 75259 | C | A | C | 0.75806 | A | 0.24194 | 11 | 0.35484 |
| 13850 | S17_46133873 | 17 | 46133873 | 52 | G | A | G | 0.87097 | A | 0.12903 | 6 | 0.19355 |
| 13851 | S17_46660902 | 17 | 46660902 | 527029 | T | C | C | 0.72581 | T | 0.27419 | 13 | 0.41935 |
| 13852 | S17_46660967 | 17 | 46660967 | 65 | A | G | G | 0.72581 | A | 0.27419 | 13 | 0.41935 |
| 13853 | S17_46660999 | 17 | 46660999 | 32 | T | C | C | 0.72581 | T | 0.27419 | 13 | 0.41935 |
| 13854 | S17_46661021 | 17 | 46661021 | 22 | A | G | G | 0.72581 | A | 0.27419 | 13 | 0.41935 |
| 13855 | S17_46661036 | 17 | 46661036 | 15 | C | T | T | 0.72581 | C | 0.27419 | 13 | 0.41935 |
| 13856 | S17_46661046 | 17 | 46661046 | 10 | C | T | C | 0.5 | T | 0.5 | 13 | 0.41935 |
| 13857 | S17_48883186 | 17 | 48883186 | 2222140 | A | G | A | 0.66129 | G | 0.33871 | 11 | 0.35484 |
| 13858 | S17_48883198 | 17 | 48883198 | 12 | C | T | C | 0.8871 | T | 0.1129 | 5 | 0.16129 |
| 13859 | S17_48883235 | 17 | 48883235 | 37 | G | A | G | 0.95161 | A | 0.04839 | 3 | 0.09677 |
| 13860 | S17_48883258 | 17 | 48883258 | 23 | G | A | G | 0.87097 | A | 0.12903 | 6 | 0.19355 |
| 13861 | S17_49468399 | 17 | 49468399 | 585141 | C | T | C | 0.66129 | T | 0.33871 | 15 | 0.48387 |
| 13862 | S17_49468430 | 17 | 49468430 | 31 | G | T | G | 0.8871 | T | 0.1129 | 7 | 0.22581 |
| 13863 | S17_49659815 | 17 | 49659815 | 191385 | A | G | A | 0.59677 | G | 0.40323 | 13 | 0.41935 |
| 13864 | S17_49659891 | 17 | 49659891 | 76 | A | G | A | 0.72581 | G | 0.27419 | 11 | 0.35484 |
| 13865 | S17_49659893 | 17 | 49659893 | 2 | C | T | C | 0.72581 | T | 0.27419 | 11 | 0.35484 |
| 13866 | S17_50630928 | 17 | 50630928 | 971035 | C | T | C | 0.93548 | T | 0.06452 | 4 | 0.12903 |
| 13867 | S17_52303571 | 17 | 52303571 | 1672643 | T | C | C | 0.87097 | T | 0.12903 | 8 | 0.25806 |
| 13868 | S17_52303651 | 17 | 52303651 | 80 | C | T | C | 0.91935 | T | 0.08065 | 5 | 0.16129 |
| 13869 | S17_52303699 | 17 | 52303699 | 48 | C | T | T | 0.51613 | C | 0.48387 | 12 | 0.3871 |
| 13870 | S17_52444529 | 17 | 52444529 | 140830 | A | G | A | 0.66129 | G | 0.33871 | 15 | 0.48387 |
| 13871 | S17_53070647 | 17 | 53070647 | 626118 | A | C | A | 0.95161 | C | 0.04839 | 3 | 0.09677 |
| 13872 | S17_53070715 | 17 | 53070715 | 68 | T | C | T | 0.93548 | C | 0.06452 | 4 | 0.12903 |
| 13873 | S17_53079513 | 17 | 53079513 | 8798 | G | A | G | 0.59677 | A | 0.40323 | 17 | 0.54839 |
| 13874 | S17_53079528 | 17 | 53079528 | 15 | T | C | C | 0.74194 | T | 0.25806 | 10 | 0.32258 |
| 13875 | S17_53079558 | 17 | 53079558 | 30 | G | A | A | 0.74194 | G | 0.25806 | 10 | 0.32258 |
| 13876 | S17_53079640 | 17 | 53079640 | 82 | C | T | T | 0.74194 | C | 0.25806 | 10 | 0.32258 |

| Site number | SNP name | Chromosome | Physical position | Physical distance from the previous SNP | Reference allele | Alternative allele | Major allele | Major allele frequency | Minor allele | Minor allele frequency | Number heterozygous | Heterozygosity |
|---|---|---|---|---|---|---|---|---|---|---|---|---|
| 13877 | S17_53230000 | 17 | 53230000 | 150360 | T | G | T | 0.83871 | G | 0.16129 | 8 | 0.25806 |
| 13878 | S17_53230034 | 17 | 53230034 | 34 | A | G | A | 0.91935 | G | 0.08065 | 5 | 0.16129 |
| 13879 | S17_53341445 | 17 | 53341445 | 111411 | G | A | G | 0.72581 | A | 0.27419 | 15 | 0.48387 |
| 13880 | S17_53341590 | 17 | 53341590 | 145 | T | C | T | 0.72581 | C | 0.27419 | 15 | 0.48387 |
| 13881 | S17_53524575 | 17 | 53524575 | 182985 | C | A | A | 0.64516 | C | 0.35484 | 14 | 0.45161 |
| 13882 | S17_53585425 | 17 | 53585425 | 60850 | G | A | G | 0.93548 | A | 0.06452 | 4 | 0.12903 |
| 13883 | S17_53585526 | 17 | 53585526 | 101 | C | T | C | 0.8871 | T | 0.1129 | 7 | 0.22581 |
| 13884 | S17_53638561 | 17 | 53638561 | 53035 | C | T | C | 0.93548 | T | 0.06452 | 4 | 0.12903 |
| 13885 | S17_53638581 | 17 | 53638581 | 20 | A | G | G | 0.8871 | A | 0.1129 | 7 | 0.22581 |
| 13886 | S17_53638627 | 17 | 53638627 | 46 | C | T | C | 0.91935 | T | 0.08065 | 5 | 0.16129 |
| 13887 | S17_53755611 | 17 | 53755611 | 116984 | G | A | G | 0.70968 | A | 0.29032 | 16 | 0.51613 |
| 13888 | S17_53755685 | 17 | 53755685 | 74 | A | G | A | 0.91935 | G | 0.08065 | 5 | 0.16129 |
| 13889 | S17_53755705 | 17 | 53755705 | 20 | G | A | G | 0.93548 | A | 0.06452 | 4 | 0.12903 |
| 13890 | S17_53755725 | 17 | 53755725 | 20 | G | A | G | 0.95161 | A | 0.04839 | 3 | 0.09677 |
| 13891 | S17_53755776 | 17 | 53755776 | 51 | G | T | G | 0.58065 | T | 0.41935 | 18 | 0.58065 |
| 13892 | S17_53755781 | 17 | 53755781 | 5 | G | T | G | 0.95161 | T | 0.04839 | 3 | 0.09677 |
| 13893 | S17_53841787 | 17 | 53841787 | 86006 | C | G | G | 0.62903 | C | 0.37097 | 13 | 0.41935 |
| 13894 | S17_53877777 | 17 | 53877777 | 35990 | C | T | C | 0.95161 | T | 0.04839 | 3 | 0.09677 |
| 13895 | S17_54422494 | 17 | 54422494 | 544717 | G | A | G | 0.85484 | A | 0.14516 | 9 | 0.29032 |
| 13896 | S17_54422499 | 17 | 54422499 | 5 | G | A | G | 0.91935 | A | 0.08065 | 5 | 0.16129 |
| 13897 | S17_54422686 | 17 | 54422686 | 187 | C | T | C | 0.93548 | T | 0.06452 | 4 | 0.12903 |
| 13898 | S17_54706468 | 17 | 54706468 | 283782 | G | A | G | 0.95161 | A | 0.04839 | 3 | 0.09677 |
| 13899 | S17_54706503 | 17 | 54706503 | 35 | C | T | T | 0.59677 | C | 0.40323 | 17 | 0.54839 |
| 13900 | S17_54706556 | 17 | 54706556 | 53 | G | A | G | 0.93548 | A | 0.06452 | 4 | 0.12903 |
| 13901 | S17_54706615 | 17 | 54706615 | 59 | T | C | T | 0.75806 | C | 0.24194 | 15 | 0.48387 |
| 13902 | S17_54811469 | 17 | 54811469 | 104854 | T | C | T | 0.87097 | C | 0.12903 | 6 | 0.19355 |
| 13903 | S17_54811594 | 17 | 54811594 | 125 | A | G | A | 0.87097 | G | 0.12903 | 6 | 0.19355 |
| 13904 | S17_54811616 | 17 | 54811616 | 22 | A | C | A | 0.79032 | C | 0.20968 | 11 | 0.35484 |
| 13905 | S17_54900105 | 17 | 54900105 | 88489 | G | A | A | 0.77419 | G | 0.22581 | 12 | 0.3871 |
| 13906 | S17_54900158 | 17 | 54900158 | 53 | G | A | G | 0.90323 | A | 0.09677 | 6 | 0.19355 |
| 13907 | S17_55141847 | 17 | 55141847 | 241689 | C | T | C | 0.93548 | T | 0.06452 | 4 | 0.12903 |
| 13908 | S17_55141852 | 17 | 55141852 | 5 | C | T | T | 0.93548 | C | 0.06452 | 4 | 0.12903 |
| 13909 | S17_55141952 | 17 | 55141952 | 100 | A | C | A | 0.90323 | C | 0.09677 | 6 | 0.19355 |
| 13910 | S17_55141953 | 17 | 55141953 | 1 | C | T | C | 0.90323 | T | 0.09677 | 6 | 0.19355 |
| 13911 | S17_55376812 | 17 | 55376812 | 234859 | A | G | A | 0.54839 | G | 0.45161 | 16 | 0.51613 |
| 13912 | S17_55376815 | 17 | 55376815 | 3 | T | C | T | 0.54839 | C | 0.45161 | 16 | 0.51613 |
| 13913 | S17_55376827 | 17 | 55376827 | 12 | C | T | C | 0.91935 | T | 0.08065 | 5 | 0.16129 |
| 13914 | S17_55376841 | 17 | 55376841 | 14 | G | A | G | 0.74194 | A | 0.25806 | 14 | 0.45161 |
| 13915 | S17_55551536 | 17 | 55551536 | 174695 | T | A | A | 0.91935 | T | 0.08065 | 3 | 0.09677 |
| 13916 | S17_55551548 | 17 | 55551548 | 12 | C | T | C | 0.87097 | T | 0.12903 | 8 | 0.25806 |
| 13917 | S17_55551722 | 17 | 55551722 | 174 | C | T | C | 0.82258 | T | 0.17742 | 9 | 0.29032 |
| 13918 | S17_56039134 | 17 | 56039134 | 487412 | A | G | A | 0.93548 | G | 0.06452 | 4 | 0.12903 |
| 13919 | S17_56039257 | 17 | 56039257 | 123 | G | A | G | 0.95161 | A | 0.04839 | 3 | 0.09677 |
| 13920 | S17_56039333 | 17 | 56039333 | 76 | G | A | G | 0.93548 | A | 0.06452 | 4 | 0.12903 |
| 13921 | S17_56039351 | 17 | 56039351 | 18 | A | G | A | 0.93548 | G | 0.06452 | 4 | 0.12903 |
| 13922 | S17_56156953 | 17 | 56156953 | 117602 | T | G | T | 0.82258 | G | 0.17742 | 11 | 0.35484 |
| 13923 | S17_56157013 | 17 | 56157013 | 60 | C | T | C | 0.82258 | T | 0.17742 | 11 | 0.35484 |
| 13924 | S17_56157094 | 17 | 56157094 | 81 | T | C | C | 0.69355 | T | 0.30645 | 17 | 0.54839 |
| 13925 | S17_56157096 | 17 | 56157096 | 2 | C | T | T | 0.6129 | C | 0.3871 | 18 | 0.58065 |
| 13926 | S17_56219955 | 17 | 56219955 | 62859 | G | A | G | 0.72581 | A | 0.27419 | 11 | 0.35484 |
| 13927 | S17_56357162 | 17 | 56357162 | 137207 | C | T | C | 0.87097 | T | 0.12903 | 8 | 0.25806 |
| 13928 | S17_56357163 | 17 | 56357163 | 1 | G | A | G | 0.62903 | A | 0.37097 | 17 | 0.54839 |
| 13929 | S17_56357195 | 17 | 56357195 | 32 | T | C | T | 0.62903 | C | 0.37097 | 17 | 0.54839 |
| 13930 | S17_56469005 | 17 | 56469005 | 111810 | C | T | C | 0.79032 | T | 0.20968 | 11 | 0.35484 |

| Site number | SNP name | Chromosome | Physical position | Physical distance from the previous SNP | Reference allele | Alternative allele | Major allele | Major allele frequency | Minor allele | Minor allele frequency | Number heterozygous | Heterozygosity |
|---|---|---|---|---|---|---|---|---|---|---|---|---|
| 13931 | S17_56469040 | 17 | 56469040 | 35 | G | A | G | 0.80645 | A | 0.19355 | 10 | 0.32258 |
| 13932 | S17_56511811 | 17 | 56511811 | 42771 | C | G | C | 0.93548 | G | 0.06452 | 4 | 0.12903 |
| 13933 | S17_56784262 | 17 | 56784262 | 272451 | A | G | A | 0.70968 | G | 0.29032 | 10 | 0.32258 |
| 13934 | S17_57276422 | 17 | 57276422 | 492160 | C | T | C | 0.6129 | T | 0.3871 | 14 | 0.45161 |
| 13935 | S17_57276438 | 17 | 57276438 | 16 | T | C | T | 0.91935 | C | 0.08065 | 3 | 0.09677 |
| 13936 | S17_57573714 | 17 | 57573714 | 297276 | C | T | C | 0.8871 | T | 0.1129 | 5 | 0.16129 |
| 13937 | S17_57573797 | 17 | 57573797 | 83 | G | A | G | 0.91935 | A | 0.08065 | 5 | 0.16129 |
| 13938 | S17_57583128 | 17 | 57583128 | 9331 | C | T | T | 0.66129 | C | 0.33871 | 13 | 0.41935 |
| 13939 | S17_57583362 | 17 | 57583362 | 234 | T | C | T | 0.95161 | C | 0.04839 | 3 | 0.09677 |
| 13940 | S17_57686734 | 17 | 57686734 | 103372 | C | T | C | 0.85484 | T | 0.14516 | 7 | 0.22581 |
| 13941 | S17_57930831 | 17 | 57930831 | 244097 | A | G | G | 0.85484 | A | 0.14516 | 9 | 0.29032 |
| 13942 | S17_58262901 | 17 | 58262901 | 332070 | C | T | T | 0.93548 | C | 0.06452 | 4 | 0.12903 |
| 13943 | S17_58262914 | 17 | 58262914 | 13 | A | C | A | 0.93548 | C | 0.06452 | 4 | 0.12903 |
| 13944 | S17_58262975 | 17 | 58262975 | 61 | T | C | C | 0.93548 | T | 0.06452 | 4 | 0.12903 |
| 13945 | S17_58263047 | 17 | 58263047 | 72 | A | G | G | 0.93548 | A | 0.06452 | 4 | 0.12903 |
| 13946 | S17_59177568 | 17 | 59177568 | 914521 | G | C | G | 0.91935 | C | 0.08065 | 5 | 0.16129 |
| 13947 | S17_59177630 | 17 | 59177630 | 62 | T | G | T | 0.93548 | G | 0.06452 | 2 | 0.06452 |
| 13948 | S17_59177732 | 17 | 59177732 | 102 | C | T | T | 0.6129 | C | 0.3871 | 12 | 0.3871 |
| 13949 | S17_59374687 | 17 | 59374687 | 196955 | A | G | A | 0.74194 | G | 0.25806 | 10 | 0.32258 |
| 13950 | S17_59640846 | 17 | 59640846 | 266159 | T | C | T | 0.82258 | C | 0.17742 | 9 | 0.29032 |
| 13951 | S17_59640875 | 17 | 59640875 | 29 | A | T | A | 0.53226 | T | 0.46774 | 13 | 0.41935 |
| 13952 | S17_59641052 | 17 | 59641052 | 177 | G | C | G | 0.82258 | C | 0.17742 | 7 | 0.22581 |
| 13953 | S17_59749581 | 17 | 59749581 | 108529 | G | A | A | 0.58065 | G | 0.41935 | 12 | 0.3871 |
| 13954 | S17_59749719 | 17 | 59749719 | 138 | G | A | G | 0.74194 | A | 0.25806 | 12 | 0.3871 |
| 13955 | S17_59939109 | 17 | 59939109 | 189390 | A | T | A | 0.5 | T | 0.5 | 15 | 0.48387 |
| 13956 | S17_59939240 | 17 | 59939240 | 131 | C | A | C | 0.64516 | A | 0.35484 | 14 | 0.45161 |
| 13957 | S17_59939324 | 17 | 59939324 | 84 | A | C | A | 0.85484 | C | 0.14516 | 7 | 0.22581 |
| 13958 | S17_60280155 | 17 | 60280155 | 340831 | T | C | T | 0.93548 | C | 0.06452 | 4 | 0.12903 |
| 13959 | S17_60280235 | 17 | 60280235 | 80 | G | A | G | 0.74194 | A | 0.25806 | 14 | 0.45161 |
| 13960 | S17_60280265 | 17 | 60280265 | 30 | G | C | G | 0.93548 | C | 0.06452 | 4 | 0.12903 |
| 13961 | S17_60280269 | 17 | 60280269 | 4 | A | G | A | 0.93548 | G | 0.06452 | 4 | 0.12903 |
| 13962 | S17_60280276 | 17 | 60280276 | 7 | T | C | T | 0.93548 | C | 0.06452 | 4 | 0.12903 |
| 13963 | S17_60280287 | 17 | 60280287 | 11 | G | A | G | 0.93548 | A | 0.06452 | 4 | 0.12903 |
| 13964 | S17_60280291 | 17 | 60280291 | 4 | C | G | C | 0.93548 | G | 0.06452 | 4 | 0.12903 |
| 13965 | S17_60280298 | 17 | 60280298 | 7 | G | T | G | 0.93548 | T | 0.06452 | 4 | 0.12903 |
| 13966 | S17_60280335 | 17 | 60280335 | 37 | C | T | C | 0.93548 | T | 0.06452 | 4 | 0.12903 |
| 13967 | S17_60280369 | 17 | 60280369 | 34 | T | C | T | 0.74194 | C | 0.25806 | 14 | 0.45161 |
| 13968 | S17_60280379 | 17 | 60280379 | 10 | C | G | G | 0.56452 | C | 0.43548 | 15 | 0.48387 |
| 13969 | S17_60309023 | 17 | 60309023 | 28644 | A | G | A | 0.67742 | G | 0.32258 | 14 | 0.45161 |
| 13970 | S17_60309076 | 17 | 60309076 | 53 | T | C | T | 0.67742 | C | 0.32258 | 14 | 0.45161 |
| 13971 | S17_60525755 | 17 | 60525755 | 216679 | A | G | A | 0.80645 | G | 0.19355 | 8 | 0.25806 |
| 13972 | S17_60525764 | 17 | 60525764 | 9 | A | C | C | 0.8871 | A | 0.1129 | 5 | 0.16129 |
| 13973 | S17_60525802 | 17 | 60525802 | 38 | G | C | G | 0.93548 | C | 0.06452 | 4 | 0.12903 |
| 13974 | S17_60549851 | 17 | 60549851 | 24049 | G | A | G | 0.95161 | A | 0.04839 | 3 | 0.09677 |
| 13975 | S17_60549859 | 17 | 60549859 | 8 | T | C | T | 0.66129 | C | 0.33871 | 13 | 0.41935 |
| 13976 | S17_60574792 | 17 | 60574792 | 24933 | G | T | T | 0.51613 | G | 0.48387 | 10 | 0.32258 |
| 13977 | S17_60574901 | 17 | 60574901 | 109 | A | G | A | 0.91935 | G | 0.08065 | 5 | 0.16129 |
| 13978 | S17_60574986 | 17 | 60574986 | 85 | C | T | C | 0.87097 | T | 0.12903 | 6 | 0.19355 |
| 13979 | S17_60585091 | 17 | 60585091 | 10105 | T | C | T | 0.83871 | C | 0.16129 | 10 | 0.32258 |
| 13980 | S17_60585193 | 17 | 60585193 | 102 | T | C | C | 0.85484 | T | 0.14516 | 9 | 0.29032 |
| 13981 | S17_60585215 | 17 | 60585215 | 22 | C | T | T | 0.53226 | C | 0.46774 | 17 | 0.54839 |
| 13982 | S17_60585244 | 17 | 60585244 | 29 | G | T | T | 0.53226 | G | 0.46774 | 17 | 0.54839 |
| 13983 | S17_60585262 | 17 | 60585262 | 18 | C | T | C | 0.8871 | T | 0.1129 | 7 | 0.22581 |
| 13984 | S17_60679927 | 17 | 60679927 | 94665 | C | T | C | 0.93548 | T | 0.06452 | 4 | 0.12903 |

| Site number | SNP name | Chromosome | Physical position | Physical distance from the previous SNP | Reference allele | Alternative allele | Major allele | Major allele frequency | Minor allele | Minor allele frequency | Number heterozygous | Heterozygosity |
|---|---|---|---|---|---|---|---|---|---|---|---|---|
| 13985 | S17_60679982 | 17 | 60679982 | 55 | C | A | C | 0.77419 | A | 0.22581 | 12 | 0.3871 |
| 13986 | S17_60680005 | 17 | 60680005 | 23 | G | C | C | 0.66129 | G | 0.33871 | 15 | 0.48387 |
| 13987 | S17_60680078 | 17 | 60680078 | 73 | G | A | A | 0.62903 | G | 0.37097 | 15 | 0.48387 |
| 13988 | S17_60680130 | 17 | 60680130 | 52 | C | T | T | 0.72581 | C | 0.27419 | 11 | 0.35484 |
| 13989 | S17_60680143 | 17 | 60680143 | 13 | A | G | A | 0.91935 | G | 0.08065 | 5 | 0.16129 |
| 13990 | S17_60811646 | 17 | 60811646 | 131503 | G | A | G | 0.95161 | A | 0.04839 | 3 | 0.09677 |
| 13991 | S17_60832870 | 17 | 60832870 | 21224 | T | C | T | 0.77419 | C | 0.22581 | 12 | 0.3871 |
| 13992 | S17_60832872 | 17 | 60832872 | 2 | C | T | C | 0.77419 | T | 0.22581 | 12 | 0.3871 |
| 13993 | S17_60832879 | 17 | 60832879 | 7 | G | A | A | 0.87097 | G | 0.12903 | 8 | 0.25806 |
| 13994 | S17_60832971 | 17 | 60832971 | 92 | T | C | T | 0.77419 | C | 0.22581 | 12 | 0.3871 |
| 13995 | S17_60833017 | 17 | 60833017 | 46 | G | A | G | 0.77419 | A | 0.22581 | 12 | 0.3871 |
| 13996 | S17_60833057 | 17 | 60833057 | 40 | C | T | C | 0.77419 | T | 0.22581 | 12 | 0.3871 |
| 13997 | S17_60857287 | 17 | 60857287 | 24230 | A | G | G | 0.6129 | A | 0.3871 | 18 | 0.58065 |
| 13998 | S17_60857357 | 17 | 60857357 | 70 | A | G | G | 0.64516 | A | 0.35484 | 18 | 0.58065 |
| 13999 | S17_60857432 | 17 | 60857432 | 75 | A | G | A | 0.91935 | G | 0.08065 | 5 | 0.16129 |
| 14000 | S17_60895111 | 17 | 60895111 | 37679 | C | T | T | 0.90323 | C | 0.09677 | 6 | 0.19355 |
| 14001 | S17_60895120 | 17 | 60895120 | 9 | C | T | C | 0.85484 | T | 0.14516 | 7 | 0.22581 |
| 14002 | S17_61041420 | 17 | 61041420 | 146300 | G | A | G | 0.91935 | A | 0.08065 | 3 | 0.09677 |
| 14003 | S17_61041457 | 17 | 61041457 | 37 | G | T | G | 0.91935 | T | 0.08065 | 3 | 0.09677 |
| 14004 | S17_61041475 | 17 | 61041475 | 18 | C | T | T | 0.51613 | C | 0.48387 | 18 | 0.58065 |
| 14005 | S17_61041558 | 17 | 61041558 | 83 | A | T | A | 0.93548 | T | 0.06452 | 4 | 0.12903 |
| 14006 | S17_61255162 | 17 | 61255162 | 213604 | C | G | C | 0.95161 | G | 0.04839 | 3 | 0.09677 |
| 14007 | S17_61255243 | 17 | 61255243 | 81 | G | A | G | 0.87097 | A | 0.12903 | 8 | 0.25806 |
| 14008 | S17_61491563 | 17 | 61491563 | 236320 | G | A | G | 0.93548 | A | 0.06452 | 4 | 0.12903 |
| 14009 | S17_61491583 | 17 | 61491583 | 20 | G | A | A | 0.80645 | G | 0.19355 | 10 | 0.32258 |
| 14010 | S17_61491590 | 17 | 61491590 | 7 | G | A | A | 0.54839 | G | 0.45161 | 18 | 0.58065 |
| 14011 | S17_61491596 | 17 | 61491596 | 6 | A | G | G | 0.82258 | A | 0.17742 | 9 | 0.29032 |
| 14012 | S17_61491705 | 17 | 61491705 | 109 | T | C | C | 0.80645 | T | 0.19355 | 10 | 0.32258 |
| 14013 | S17_61938946 | 17 | 61938946 | 447241 | C | T | C | 0.79032 | T | 0.20968 | 9 | 0.29032 |
| 14014 | S17_61939046 | 17 | 61939046 | 100 | G | A | G | 0.79032 | A | 0.20968 | 9 | 0.29032 |
| 14015 | S17_61939051 | 17 | 61939051 | 5 | G | A | G | 0.79032 | A | 0.20968 | 9 | 0.29032 |
| 14016 | S17_61939076 | 17 | 61939076 | 25 | G | A | G | 0.79032 | A | 0.20968 | 9 | 0.29032 |
| 14017 | S17_62297617 | 17 | 62297617 | 358541 | C | T | C | 0.93548 | T | 0.06452 | 4 | 0.12903 |
| 14018 | S17_62437451 | 17 | 62437451 | 139834 | C | T | C | 0.85484 | T | 0.14516 | 7 | 0.22581 |
| 14019 | S17_62437526 | 17 | 62437526 | 75 | T | G | T | 0.6129 | G | 0.3871 | 14 | 0.45161 |
| 14020 | S17_62437542 | 17 | 62437542 | 16 | C | T | C | 0.91935 | T | 0.08065 | 5 | 0.16129 |
| 14021 | S17_62586437 | 17 | 62586437 | 148895 | T | C | T | 0.95161 | C | 0.04839 | 3 | 0.09677 |
| 14022 | S17_62646085 | 17 | 62646085 | 59648 | G | T | T | 0.66129 | G | 0.33871 | 11 | 0.35484 |
| 14023 | S17_62646097 | 17 | 62646097 | 12 | C | A | C | 0.83871 | A | 0.16129 | 8 | 0.25806 |
| 14024 | S17_62792419 | 17 | 62792419 | 146322 | T | C | C | 0.59677 | T | 0.40323 | 15 | 0.48387 |
| 14025 | S17_63079329 | 17 | 63079329 | 286910 | A | G | A | 0.8871 | G | 0.1129 | 5 | 0.16129 |
| 14026 | S17_63096182 | 17 | 63096182 | 16853 | A | T | A | 0.82258 | T | 0.17742 | 11 | 0.35484 |
| 14027 | S17_63096237 | 17 | 63096237 | 55 | A | T | A | 0.93548 | T | 0.06452 | 4 | 0.12903 |
| 14028 | S17_63206654 | 17 | 63206654 | 110417 | C | A | C | 0.67742 | A | 0.32258 | 10 | 0.32258 |
| 14029 | S17_63206770 | 17 | 63206770 | 116 | G | A | G | 0.93548 | A | 0.06452 | 4 | 0.12903 |
| 14030 | S17_63206785 | 17 | 63206785 | 15 | G | C | G | 0.67742 | C | 0.32258 | 10 | 0.32258 |
| 14031 | S17_64133029 | 17 | 64133029 | 926244 | C | T | C | 0.85484 | T | 0.14516 | 7 | 0.22581 |
| 14032 | S17_64133095 | 17 | 64133095 | 66 | T | C | T | 0.91935 | C | 0.08065 | 3 | 0.09677 |
| 14033 | S17_64133113 | 17 | 64133113 | 18 | A | G | G | 0.80645 | A | 0.19355 | 6 | 0.19355 |
| 14034 | S17_64133145 | 17 | 64133145 | 32 | C | T | C | 0.91935 | T | 0.08065 | 3 | 0.09677 |
| 14035 | S17_64133182 | 17 | 64133182 | 37 | C | G | G | 0.80645 | C | 0.19355 | 6 | 0.19355 |
| 14036 | S17_64133190 | 17 | 64133190 | 8 | A | G | A | 0.85484 | G | 0.14516 | 7 | 0.22581 |
| 14037 | S17_64133212 | 17 | 64133212 | 22 | G | A | G | 0.8871 | A | 0.1129 | 5 | 0.16129 |
| 14038 | S17_64203809 | 17 | 64203809 | 70597 | G | A | G | 0.87097 | A | 0.12903 | 6 | 0.19355 |

| Site number | SNP name | Chromosome | Physical position | Physical distance from the previous SNP | Reference allele | Alternative allele | Major allele | Major allele frequency | Minor allele | Minor allele frequency | Number heterozygous | Heterozygosity |
|---|---|---|---|---|---|---|---|---|---|---|---|---|
| 14039 | S17_64203899 | 17 | 64203899 | 90 | G | A | G | 0.87097 | A | 0.12903 | 6 | 0.19355 |
| 14040 | S17_64203975 | 17 | 64203975 | 76 | A | G | G | 0.66129 | A | 0.33871 | 15 | 0.48387 |
| 14041 | S17_64265640 | 17 | 64265640 | 61665 | T | C | C | 0.79032 | T | 0.20968 | 11 | 0.35484 |
| 14042 | S17_64265660 | 17 | 64265660 | 20 | G | A | G | 0.8871 | A | 0.1129 | 7 | 0.22581 |
| 14043 | S17_64448002 | 17 | 64448002 | 182342 | T | C | T | 0.80645 | C | 0.19355 | 10 | 0.32258 |
| 14044 | S17_64448007 | 17 | 64448007 | 5 | T | C | T | 0.58065 | C | 0.41935 | 14 | 0.45161 |
| 14045 | S17_64605259 | 17 | 64605259 | 157252 | A | C | A | 0.93548 | C | 0.06452 | 2 | 0.06452 |
| 14046 | S17_64605340 | 17 | 64605340 | 81 | C | T | C | 0.90323 | T | 0.09677 | 6 | 0.19355 |
| 14047 | S17_64605357 | 17 | 64605357 | 17 | C | T | C | 0.93548 | T | 0.06452 | 4 | 0.12903 |
| 14048 | S17_64635839 | 17 | 64635839 | 30482 | G | A | G | 0.90323 | A | 0.09677 | 4 | 0.12903 |
| 14049 | S17_64635933 | 17 | 64635933 | 94 | C | T | C | 0.67742 | T | 0.32258 | 16 | 0.51613 |
| 14050 | S17_64635941 | 17 | 64635941 | 8 | G | A | G | 0.90323 | A | 0.09677 | 4 | 0.12903 |
| 14051 | S17_64635973 | 17 | 64635973 | 32 | G | A | G | 0.90323 | A | 0.09677 | 4 | 0.12903 |
| 14052 | S17_64737115 | 17 | 64737115 | 101142 | C | G | G | 0.87097 | C | 0.12903 | 8 | 0.25806 |
| 14053 | S17_64737119 | 17 | 64737119 | 4 | T | C | C | 0.67742 | T | 0.32258 | 14 | 0.45161 |
| 14054 | S17_64737228 | 17 | 64737228 | 109 | C | G | C | 0.87097 | G | 0.12903 | 8 | 0.25806 |
| 14055 | S17_64760882 | 17 | 64760882 | 23654 | C | G | C | 0.95161 | G | 0.04839 | 3 | 0.09677 |
| 14056 | S17_64979896 | 17 | 64979896 | 219014 | A | C | C | 0.87097 | A | 0.12903 | 6 | 0.19355 |
| 14057 | S17_64979949 | 17 | 64979949 | 53 | G | T | T | 0.87097 | G | 0.12903 | 6 | 0.19355 |
| 14058 | S17_64979950 | 17 | 64979950 | 1 | A | G | G | 0.87097 | A | 0.12903 | 6 | 0.19355 |
| 14059 | S17_64979998 | 17 | 64979998 | 48 | T | C | C | 0.87097 | T | 0.12903 | 6 | 0.19355 |
| 14060 | S17_64980118 | 17 | 64980118 | 120 | C | T | C | 0.54839 | T | 0.45161 | 16 | 0.51613 |
| 14061 | S17_64997013 | 17 | 64997013 | 16895 | G | A | G | 0.54839 | A | 0.45161 | 16 | 0.51613 |
| 14062 | S17_65080683 | 17 | 65080683 | 83670 | C | T | T | 0.87097 | C | 0.12903 | 6 | 0.19355 |
| 14063 | S17_65080740 | 17 | 65080740 | 57 | T | C | T | 0.93548 | C | 0.06452 | 4 | 0.12903 |
| 14064 | S17_65225266 | 17 | 65225266 | 144526 | A | G | G | 0.70968 | A | 0.29032 | 14 | 0.45161 |
| 14065 | S17_65225269 | 17 | 65225269 | 3 | A | G | A | 0.85484 | G | 0.14516 | 7 | 0.22581 |
| 14066 | S17_65225274 | 17 | 65225274 | 5 | A | G | A | 0.85484 | G | 0.14516 | 7 | 0.22581 |
| 14067 | S17_65225316 | 17 | 65225316 | 42 | G | C | C | 0.58065 | G | 0.41935 | 12 | 0.3871 |
| 14068 | S17_65225401 | 17 | 65225401 | 85 | T | C | T | 0.53226 | C | 0.46774 | 19 | 0.6129 |
| 14069 | S17_65225440 | 17 | 65225440 | 39 | A | G | G | 0.58065 | A | 0.41935 | 12 | 0.3871 |
| 14070 | S17_65596860 | 17 | 65596860 | 371420 | C | T | C | 0.8871 | T | 0.1129 | 5 | 0.16129 |
| 14071 | S17_65615903 | 17 | 65615903 | 19043 | C | T | C | 0.83871 | T | 0.16129 | 8 | 0.25806 |
| 14072 | S17_65742989 | 17 | 65742989 | 127086 | T | C | T | 0.93548 | C | 0.06452 | 2 | 0.06452 |
| 14073 | S17_65994117 | 17 | 65994117 | 251128 | G | A | A | 0.80645 | G | 0.19355 | 10 | 0.32258 |
| 14074 | S17_65994163 | 17 | 65994163 | 46 | C | A | C | 0.91935 | A | 0.08065 | 3 | 0.09677 |
| 14075 | S17_66415867 | 17 | 66415867 | 421704 | A | G | A | 0.67742 | G | 0.32258 | 12 | 0.3871 |
| 14076 | S17_66415903 | 17 | 66415903 | 36 | A | C | A | 0.67742 | C | 0.32258 | 12 | 0.3871 |
| 14077 | S17_66415917 | 17 | 66415917 | 14 | T | C | C | 0.53226 | T | 0.46774 | 17 | 0.54839 |
| 14078 | S17_66415938 | 17 | 66415938 | 21 | G | C | G | 0.93548 | C | 0.06452 | 4 | 0.12903 |
| 14079 | S17_66415975 | 17 | 66415975 | 37 | A | C | A | 0.67742 | C | 0.32258 | 12 | 0.3871 |
| 14080 | S17_66415981 | 17 | 66415981 | 6 | G | A | A | 0.64516 | G | 0.35484 | 14 | 0.45161 |
| 14081 | S17_66416003 | 17 | 66416003 | 22 | T | C | T | 0.66129 | C | 0.33871 | 11 | 0.35484 |
| 14082 | S17_66450353 | 17 | 66450353 | 34350 | T | G | T | 0.91935 | G | 0.08065 | 5 | 0.16129 |
| 14083 | S17_66751789 | 17 | 66751789 | 301436 | C | T | C | 0.5 | T | 0.5 | 21 | 0.67742 |
| 14084 | S17_66751810 | 17 | 66751810 | 21 | G | T | G | 0.5 | T | 0.5 | 21 | 0.67742 |
| 14085 | S17_66770486 | 17 | 66770486 | 18676 | C | G | C | 0.53226 | G | 0.46774 | 15 | 0.48387 |
| 14086 | S17_66783913 | 17 | 66783913 | 13427 | C | T | C | 0.75806 | T | 0.24194 | 13 | 0.41935 |
| 14087 | S17_66784019 | 17 | 66784019 | 106 | G | A | G | 0.75806 | A | 0.24194 | 13 | 0.41935 |
| 14088 | S17_66784023 | 17 | 66784023 | 4 | C | T | C | 0.93548 | T | 0.06452 | 4 | 0.12903 |
| 14089 | S17_66975053 | 17 | 66975053 | 191030 | T | C | T | 0.8871 | C | 0.1129 | 5 | 0.16129 |
| 14090 | S17_66975066 | 17 | 66975066 | 13 | G | T | G | 0.8871 | T | 0.1129 | 5 | 0.16129 |
| 14091 | S17_67101191 | 17 | 67101191 | 126125 | A | G | A | 0.75806 | G | 0.24194 | 9 | 0.29032 |
| 14092 | S17_67101207 | 17 | 67101207 | 16 | A | G | G | 0.6129 | A | 0.3871 | 12 | 0.3871 |

| Site number | SNP name | Chromosome | Physical position | Physical distance from the previous SNP | Reference allele | Alternative allele | Major allele | Major allele frequency | Minor allele | Minor allele frequency | Number heterozygous | Heterozygosity |
|---|---|---|---|---|---|---|---|---|---|---|---|---|
| 14093 | S17_67340268 | 17 | 67340268 | 239061 | T | A | T | 0.83871 | A | 0.16129 | 6 | 0.19355 |
| 14094 | S17_67340327 | 17 | 67340327 | 59 | T | C | T | 0.83871 | C | 0.16129 | 6 | 0.19355 |
| 14095 | S17_67340401 | 17 | 67340401 | 74 | G | T | G | 0.83871 | T | 0.16129 | 6 | 0.19355 |
| 14096 | S17_67833016 | 17 | 67833016 | 492615 | C | T | C | 0.93548 | T | 0.06452 | 2 | 0.06452 |
| 14097 | S17_67833017 | 17 | 67833017 | 1 | A | G | A | 0.93548 | G | 0.06452 | 2 | 0.06452 |
| 14098 | S17_67833050 | 17 | 67833050 | 33 | C | T | C | 0.93548 | T | 0.06452 | 2 | 0.06452 |
| 14099 | S17_67833079 | 17 | 67833079 | 29 | G | C | G | 0.82258 | C | 0.17742 | 11 | 0.35484 |
| 14100 | S17_67833087 | 17 | 67833087 | 8 | C | T | C | 0.87097 | T | 0.12903 | 6 | 0.19355 |
| 14101 | S17_67955937 | 17 | 67955937 | 122850 | C | T | C | 0.93548 | T | 0.06452 | 4 | 0.12903 |
| 14102 | S17_67956013 | 17 | 67956013 | 76 | T | C | T | 0.93548 | C | 0.06452 | 2 | 0.06452 |
| 14103 | S17_68104545 | 17 | 68104545 | 148532 | T | G | G | 0.85484 | T | 0.14516 | 9 | 0.29032 |
| 14104 | S17_68104646 | 17 | 68104646 | 101 | T | C | T | 0.93548 | C | 0.06452 | 4 | 0.12903 |
| 14105 | S17_68815425 | 17 | 68815425 | 710779 | A | G | A | 0.85484 | G | 0.14516 | 9 | 0.29032 |
| 14106 | S17_68815653 | 17 | 68815653 | 228 | G | A | G | 0.95161 | A | 0.04839 | 3 | 0.09677 |
| 14107 | S17_69212690 | 17 | 69212690 | 397037 | G | A | G | 0.75806 | A | 0.24194 | 9 | 0.29032 |
| 14108 | S17_69212727 | 17 | 69212727 | 37 | A | C | A | 0.82258 | C | 0.17742 | 9 | 0.29032 |
| 14109 | S17_69212731 | 17 | 69212731 | 4 | T | C | T | 0.83871 | C | 0.16129 | 8 | 0.25806 |
| 14110 | S17_69212732 | 17 | 69212732 | 1 | C | T | T | 0.85484 | C | 0.14516 | 9 | 0.29032 |
| 14111 | S17_69212752 | 17 | 69212752 | 20 | G | C | G | 0.74194 | C | 0.25806 | 10 | 0.32258 |
| 14112 | S17_69212769 | 17 | 69212769 | 17 | T | C | T | 0.91935 | C | 0.08065 | 5 | 0.16129 |
| 14113 | S17_69212896 | 17 | 69212896 | 127 | G | A | G | 0.53226 | A | 0.46774 | 13 | 0.41935 |
| 14114 | S17_69821290 | 17 | 69821290 | 608394 | A | G | G | 0.69355 | A | 0.30645 | 13 | 0.41935 |
| 14115 | S17_69821478 | 17 | 69821478 | 188 | C | T | T | 0.69355 | C | 0.30645 | 13 | 0.41935 |
| 14116 | S17_69821479 | 17 | 69821479 | 1 | A | G | G | 0.70968 | A | 0.29032 | 14 | 0.45161 |
| 14117 | S17_69872978 | 17 | 69872978 | 51499 | C | G | G | 0.87097 | C | 0.12903 | 6 | 0.19355 |
| 14118 | S17_69873019 | 17 | 69873019 | 41 | G | A | G | 0.74194 | A | 0.25806 | 10 | 0.32258 |
| 14119 | S17_70856336 | 17 | 70856336 | 983317 | C | T | C | 0.69355 | T | 0.30645 | 17 | 0.54839 |
| 14120 | S17_70856363 | 17 | 70856363 | 27 | C | T | C | 0.74194 | T | 0.25806 | 14 | 0.45161 |
| 14121 | S17_70856384 | 17 | 70856384 | 21 | A | C | A | 0.93548 | C | 0.06452 | 4 | 0.12903 |
| 14122 | S17_70856456 | 17 | 70856456 | 72 | G | T | G | 0.82258 | T | 0.17742 | 11 | 0.35484 |
| 14123 | S17_70856501 | 17 | 70856501 | 45 | G | A | G | 0.67742 | A | 0.32258 | 18 | 0.58065 |
| 14124 | S17_71046365 | 17 | 71046365 | 189864 | G | A | G | 0.95161 | A | 0.04839 | 3 | 0.09677 |
| 14125 | S17_71046448 | 17 | 71046448 | 83 | C | T | C | 0.95161 | T | 0.04839 | 3 | 0.09677 |
| 14126 | S17_71046457 | 17 | 71046457 | 9 | C | T | C | 0.95161 | T | 0.04839 | 3 | 0.09677 |
| 14127 | S17_71046574 | 17 | 71046574 | 117 | A | T | A | 0.91935 | T | 0.08065 | 5 | 0.16129 |
| 14128 | S18_28142 | 18 | 28142 | 0 | T | C | T | 0.90323 | C | 0.09677 | 6 | 0.19355 |
| 14129 | S18_82670 | 18 | 82670 | 54528 | G | A | G | 0.93548 | A | 0.06452 | 4 | 0.12903 |
| 14130 | S18_82696 | 18 | 82696 | 26 | G | A | A | 0.58065 | G | 0.41935 | 20 | 0.64516 |
| 14131 | S18_257200 | 18 | 257200 | 174504 | T | C | T | 0.79032 | C | 0.20968 | 9 | 0.29032 |
| 14132 | S18_257248 | 18 | 257248 | 48 | A | G | A | 0.79032 | G | 0.20968 | 9 | 0.29032 |
| 14133 | S18_257313 | 18 | 257313 | 65 | A | G | A | 0.79032 | G | 0.20968 | 9 | 0.29032 |
| 14134 | S18_257314 | 18 | 257314 | 1 | C | T | T | 0.90323 | C | 0.09677 | 6 | 0.19355 |
| 14135 | S18_257393 | 18 | 257393 | 79 | G | A | G | 0.79032 | A | 0.20968 | 9 | 0.29032 |
| 14136 | S18_291154 | 18 | 291154 | 33761 | T | C | T | 0.82258 | C | 0.17742 | 9 | 0.29032 |
| 14137 | S18_291193 | 18 | 291193 | 39 | G | T | G | 0.91935 | T | 0.08065 | 5 | 0.16129 |
| 14138 | S18_291222 | 18 | 291222 | 29 | T | C | C | 0.87097 | T | 0.12903 | 6 | 0.19355 |
| 14139 | S18_661076 | 18 | 661076 | 369854 | C | T | C | 0.93548 | T | 0.06452 | 4 | 0.12903 |
| 14140 | S18_661094 | 18 | 661094 | 18 | C | T | C | 0.75806 | T | 0.24194 | 11 | 0.35484 |
| 14141 | S18_788273 | 18 | 788273 | 127179 | T | C | T | 0.91935 | C | 0.08065 | 5 | 0.16129 |
| 14142 | S18_940000 | 18 | 940000 | 151727 | G | A | G | 0.85484 | A | 0.14516 | 9 | 0.29032 |
| 14143 | S18_1519205 | 18 | 1519205 | 579205 | C | T | C | 0.82258 | T | 0.17742 | 11 | 0.35484 |
| 14144 | S18_1519206 | 18 | 1519206 | 1 | A | G | A | 0.82258 | G | 0.17742 | 11 | 0.35484 |
| 14145 | S18_1519227 | 18 | 1519227 | 21 | A | G | A | 0.82258 | G | 0.17742 | 11 | 0.35484 |
| 14146 | S18_1519229 | 18 | 1519229 | 2 | A | G | A | 0.82258 | G | 0.17742 | 11 | 0.35484 |

| Site number | SNP name | Chromosome | Physical position | Physical distance from the previous SNP | Reference allele | Alternative allele | Major allele | Major allele frequency | Minor allele | Minor allele frequency | Number heterozygous | Heterozygosity |
|---|---|---|---|---|---|---|---|---|---|---|---|---|
| 14147 | S18_1519240 | 18 | 1519240 | 11 | A | C | A | 0.82258 | C | 0.17742 | 11 | 0.35484 |
| 14148 | S18_1519256 | 18 | 1519256 | 16 | G | A | G | 0.83871 | A | 0.16129 | 10 | 0.32258 |
| 14149 | S18_1519356 | 18 | 1519356 | 100 | A | G | A | 0.82258 | G | 0.17742 | 11 | 0.35484 |
| 14150 | S18_1519404 | 18 | 1519404 | 48 | T | A | T | 0.82258 | A | 0.17742 | 11 | 0.35484 |
| 14151 | S18_1519410 | 18 | 1519410 | 6 | T | G | T | 0.82258 | G | 0.17742 | 11 | 0.35484 |
| 14152 | S18_1531922 | 18 | 1531922 | 12512 | A | G | A | 0.72581 | G | 0.27419 | 17 | 0.54839 |
| 14153 | S18_1771682 | 18 | 1771682 | 239760 | G | C | G | 0.85484 | C | 0.14516 | 9 | 0.29032 |
| 14154 | S18_1854825 | 18 | 1854825 | 83143 | C | G | C | 0.82258 | G | 0.17742 | 11 | 0.35484 |
| 14155 | S18_1865462 | 18 | 1865462 | 10637 | C | T | C | 0.74194 | T | 0.25806 | 16 | 0.51613 |
| 14156 | S18_1865470 | 18 | 1865470 | 8 | T | C | T | 0.72581 | C | 0.27419 | 17 | 0.54839 |
| 14157 | S18_1865557 | 18 | 1865557 | 87 | G | T | T | 0.91935 | G | 0.08065 | 5 | 0.16129 |
| 14158 | S18_2987597 | 18 | 2987597 | 1122040 | C | T | C | 0.91935 | T | 0.08065 | 5 | 0.16129 |
| 14159 | S18_2987720 | 18 | 2987720 | 123 | G | T | G | 0.91935 | T | 0.08065 | 5 | 0.16129 |
| 14160 | S18_3131467 | 18 | 3131467 | 143747 | T | C | T | 0.91935 | C | 0.08065 | 5 | 0.16129 |
| 14161 | S18_3306527 | 18 | 3306527 | 175060 | T | C | C | 0.72581 | T | 0.27419 | 13 | 0.41935 |
| 14162 | S18_3306664 | 18 | 3306664 | 137 | C | G | G | 0.54839 | C | 0.45161 | 18 | 0.58065 |
| 14163 | S18_3391273 | 18 | 3391273 | 84609 | A | G | A | 0.90323 | G | 0.09677 | 6 | 0.19355 |
| 14164 | S18_3423489 | 18 | 3423489 | 32216 | A | T | A | 0.51613 | T | 0.48387 | 18 | 0.58065 |
| 14165 | S18_3423567 | 18 | 3423567 | 78 | G | A | G | 0.90323 | A | 0.09677 | 6 | 0.19355 |
| 14166 | S18_3423647 | 18 | 3423647 | 80 | C | G | C | 0.53226 | G | 0.46774 | 17 | 0.54839 |
| 14167 | S18_3423684 | 18 | 3423684 | 37 | C | T | C | 0.80645 | T | 0.19355 | 10 | 0.32258 |
| 14168 | S18_3647196 | 18 | 3647196 | 223512 | G | C | G | 0.91935 | C | 0.08065 | 5 | 0.16129 |
| 14169 | S18_3710085 | 18 | 3710085 | 62889 | C | T | C | 0.66129 | T | 0.33871 | 19 | 0.6129 |
| 14170 | S18_3780391 | 18 | 3780391 | 70306 | T | A | A | 0.77419 | T | 0.22581 | 8 | 0.25806 |
| 14171 | S18_3782063 | 18 | 3782063 | 1672 | T | G | G | 0.51613 | T | 0.48387 | 20 | 0.64516 |
| 14172 | S18_4073049 | 18 | 4073049 | 290986 | A | G | A | 0.95161 | G | 0.04839 | 3 | 0.09677 |
| 14173 | S18_4090821 | 18 | 4090821 | 17772 | C | T | T | 0.58065 | C | 0.41935 | 12 | 0.3871 |
| 14174 | S18_4090824 | 18 | 4090824 | 3 | A | C | C | 0.54839 | A | 0.45161 | 12 | 0.3871 |
| 14175 | S18_4090864 | 18 | 4090864 | 40 | G | A | A | 0.54839 | G | 0.45161 | 12 | 0.3871 |
| 14176 | S18_4487543 | 18 | 4487543 | 396679 | G | C | G | 0.79032 | C | 0.20968 | 13 | 0.41935 |
| 14177 | S18_4836436 | 18 | 4836436 | 348893 | G | A | A | 0.85484 | G | 0.14516 | 9 | 0.29032 |
| 14178 | S18_4836594 | 18 | 4836594 | 158 | A | C | C | 0.85484 | A | 0.14516 | 9 | 0.29032 |
| 14179 | S18_4893291 | 18 | 4893291 | 56697 | T | A | T | 0.95161 | A | 0.04839 | 3 | 0.09677 |
| 14180 | S18_5287158 | 18 | 5287158 | 393867 | C | T | C | 0.90323 | T | 0.09677 | 6 | 0.19355 |
| 14181 | S18_5287179 | 18 | 5287179 | 21 | C | T | C | 0.90323 | T | 0.09677 | 6 | 0.19355 |
| 14182 | S18_5693567 | 18 | 5693567 | 406388 | T | C | C | 0.66129 | T | 0.33871 | 15 | 0.48387 |
| 14183 | S18_5924187 | 18 | 5924187 | 230620 | G | A | A | 0.75806 | G | 0.24194 | 13 | 0.41935 |
| 14184 | S18_5924338 | 18 | 5924338 | 151 | C | T | C | 0.90323 | T | 0.09677 | 4 | 0.12903 |
| 14185 | S18_5924424 | 18 | 5924424 | 86 | G | A | G | 0.75806 | A | 0.24194 | 15 | 0.48387 |
| 14186 | S18_6001633 | 18 | 6001633 | 77209 | T | C | C | 0.83871 | T | 0.16129 | 10 | 0.32258 |
| 14187 | S18_6001652 | 18 | 6001652 | 19 | C | T | C | 0.59677 | T | 0.40323 | 15 | 0.48387 |
| 14188 | S18_6070684 | 18 | 6070684 | 69032 | T | C | C | 0.87097 | T | 0.12903 | 4 | 0.12903 |
| 14189 | S18_6070708 | 18 | 6070708 | 24 | G | C | C | 0.85484 | G | 0.14516 | 5 | 0.16129 |
| 14190 | S18_6070714 | 18 | 6070714 | 6 | C | T | C | 0.95161 | T | 0.04839 | 3 | 0.09677 |
| 14191 | S18_6070749 | 18 | 6070749 | 35 | A | G | A | 0.53226 | G | 0.46774 | 15 | 0.48387 |
| 14192 | S18_6286201 | 18 | 6286201 | 215452 | G | C | G | 0.79032 | C | 0.20968 | 13 | 0.41935 |
| 14193 | S18_6286301 | 18 | 6286301 | 100 | G | A | G | 0.8871 | A | 0.1129 | 7 | 0.22581 |
| 14194 | S18_6322644 | 18 | 6322644 | 36343 | G | A | G | 0.54839 | A | 0.45161 | 16 | 0.51613 |
| 14195 | S18_6322743 | 18 | 6322743 | 99 | C | T | C | 0.79032 | T | 0.20968 | 13 | 0.41935 |
| 14196 | S18_6776981 | 18 | 6776981 | 454238 | T | C | T | 0.83871 | C | 0.16129 | 8 | 0.25806 |
| 14197 | S18_6776988 | 18 | 6776988 | 7 | A | G | A | 0.95161 | G | 0.04839 | 3 | 0.09677 |
| 14198 | S18_6777154 | 18 | 6777154 | 166 | G | A | G | 0.91935 | A | 0.08065 | 5 | 0.16129 |
| 14199 | S18_6929483 | 18 | 6929483 | 152329 | A | G | G | 0.51613 | A | 0.48387 | 14 | 0.45161 |
| 14200 | S18_6929511 | 18 | 6929511 | 28 | G | A | G | 0.93548 | A | 0.06452 | 4 | 0.12903 |

| Site number | SNP name | Chromosome | Physical position | Physical distance from the previous SNP | Reference allele | Alternative allele | Major allele | Major allele frequency | Minor allele | Minor allele frequency | Number heterozygous | Heterozygosity |
|---|---|---|---|---|---|---|---|---|---|---|---|---|
| 14201 | S18_7127131 | 18 | 7127131 | 197620 | A | G | A | 0.79032 | G | 0.20968 | 9 | 0.29032 |
| 14202 | S18_7127276 | 18 | 7127276 | 145 | G | A | G | 0.59677 | A | 0.40323 | 11 | 0.35484 |
| 14203 | S18_7366023 | 18 | 7366023 | 238747 | A | G | A | 0.90323 | G | 0.09677 | 6 | 0.19355 |
| 14204 | S18_7366041 | 18 | 7366041 | 18 | A | G | A | 0.90323 | G | 0.09677 | 6 | 0.19355 |
| 14205 | S18_7366108 | 18 | 7366108 | 67 | G | C | G | 0.93548 | C | 0.06452 | 4 | 0.12903 |
| 14206 | S18_7564050 | 18 | 7564050 | 197942 | C | T | C | 0.82258 | T | 0.17742 | 7 | 0.22581 |
| 14207 | S18_8093301 | 18 | 8093301 | 529251 | G | A | G | 0.95161 | A | 0.04839 | 3 | 0.09677 |
| 14208 | S18_8093352 | 18 | 8093352 | 51 | G | T | G | 0.75806 | T | 0.24194 | 9 | 0.29032 |
| 14209 | S18_8248250 | 18 | 8248250 | 154898 | A | C | C | 0.77419 | A | 0.22581 | 14 | 0.45161 |
| 14210 | S18_8248251 | 18 | 8248251 | 1 | G | T | G | 0.95161 | T | 0.04839 | 1 | 0.03226 |
| 14211 | S18_8248304 | 18 | 8248304 | 53 | C | T | C | 0.90323 | T | 0.09677 | 6 | 0.19355 |
| 14212 | S18_8248350 | 18 | 8248350 | 46 | T | C | T | 0.95161 | C | 0.04839 | 3 | 0.09677 |
| 14213 | S18_8249062 | 18 | 8249062 | 712 | A | G | G | 0.53226 | A | 0.46774 | 19 | 0.6129 |
| 14214 | S18_8298947 | 18 | 8298947 | 49885 | G | A | A | 0.59677 | G | 0.40323 | 11 | 0.35484 |
| 14215 | S18_8298977 | 18 | 8298977 | 30 | T | C | T | 0.72581 | C | 0.27419 | 11 | 0.35484 |
| 14216 | S18_8313951 | 18 | 8313951 | 14974 | C | T | T | 0.62903 | C | 0.37097 | 15 | 0.48387 |
| 14217 | S18_8313994 | 18 | 8313994 | 43 | A | C | C | 0.56452 | A | 0.43548 | 13 | 0.41935 |
| 14218 | S18_8314005 | 18 | 8314005 | 11 | T | C | C | 0.56452 | T | 0.43548 | 13 | 0.41935 |
| 14219 | S18_8314027 | 18 | 8314027 | 22 | C | T | C | 0.82258 | T | 0.17742 | 7 | 0.22581 |
| 14220 | S18_8314085 | 18 | 8314085 | 58 | A | T | A | 0.64516 | T | 0.35484 | 18 | 0.58065 |
| 14221 | S18_8488870 | 18 | 8488870 | 174785 | T | G | T | 0.93548 | G | 0.06452 | 4 | 0.12903 |
| 14222 | S18_8488951 | 18 | 8488951 | 81 | G | A | G | 0.93548 | A | 0.06452 | 4 | 0.12903 |
| 14223 | S18_8774224 | 18 | 8774224 | 285273 | C | A | C | 0.91935 | A | 0.08065 | 5 | 0.16129 |
| 14224 | S18_8774359 | 18 | 8774359 | 135 | G | A | G | 0.90323 | A | 0.09677 | 4 | 0.12903 |
| 14225 | S18_8828808 | 18 | 8828808 | 54449 | A | C | C | 0.74194 | A | 0.25806 | 16 | 0.51613 |
| 14226 | S18_8828921 | 18 | 8828921 | 113 | G | T | T | 0.74194 | G | 0.25806 | 16 | 0.51613 |
| 14227 | S18_9225178 | 18 | 9225178 | 396257 | G | C | G | 0.91935 | C | 0.08065 | 5 | 0.16129 |
| 14228 | S18_9225192 | 18 | 9225192 | 14 | A | T | A | 0.91935 | T | 0.08065 | 5 | 0.16129 |
| 14229 | S18_9225314 | 18 | 9225314 | 122 | C | T | C | 0.91935 | T | 0.08065 | 5 | 0.16129 |
| 14230 | S18_9229855 | 18 | 9229855 | 4541 | G | A | A | 0.8871 | G | 0.1129 | 5 | 0.16129 |
| 14231 | S18_9229946 | 18 | 9229946 | 91 | G | A | G | 0.87097 | A | 0.12903 | 6 | 0.19355 |
| 14232 | S18_9240337 | 18 | 9240337 | 10391 | A | C | C | 0.62903 | A | 0.37097 | 13 | 0.41935 |
| 14233 | S18_9240362 | 18 | 9240362 | 25 | G | C | C | 0.62903 | G | 0.37097 | 13 | 0.41935 |
| 14234 | S18_10374593 | 18 | 10374593 | 1134231 | C | A | C | 0.82258 | A | 0.17742 | 7 | 0.22581 |
| 14235 | S18_10565089 | 18 | 10565089 | 190496 | T | C | T | 0.87097 | C | 0.12903 | 8 | 0.25806 |
| 14236 | S18_10646193 | 18 | 10646193 | 81104 | T | C | T | 0.62903 | C | 0.37097 | 19 | 0.6129 |
| 14237 | S18_10646239 | 18 | 10646239 | 46 | C | T | C | 0.8871 | T | 0.1129 | 7 | 0.22581 |
| 14238 | S18_10646384 | 18 | 10646384 | 145 | G | A | G | 0.83871 | A | 0.16129 | 10 | 0.32258 |
| 14239 | S18_10672798 | 18 | 10672798 | 26414 | G | C | G | 0.93548 | C | 0.06452 | 4 | 0.12903 |
| 14240 | S18_10672800 | 18 | 10672800 | 2 | G | A | G | 0.85484 | A | 0.14516 | 9 | 0.29032 |
| 14241 | S18_11033682 | 18 | 11033682 | 360882 | T | C | C | 0.80645 | T | 0.19355 | 8 | 0.25806 |
| 14242 | S18_11473724 | 18 | 11473724 | 440042 | G | A | G | 0.90323 | A | 0.09677 | 6 | 0.19355 |
| 14243 | S18_11473821 | 18 | 11473821 | 97 | C | T | C | 0.85484 | T | 0.14516 | 9 | 0.29032 |
| 14244 | S18_11486505 | 18 | 11486505 | 12684 | A | G | A | 0.67742 | G | 0.32258 | 18 | 0.58065 |
| 14245 | S18_11486539 | 18 | 11486539 | 34 | C | A | C | 0.67742 | A | 0.32258 | 18 | 0.58065 |
| 14246 | S18_11550155 | 18 | 11550155 | 63616 | G | A | G | 0.70968 | A | 0.29032 | 14 | 0.45161 |
| 14247 | S18_11555888 | 18 | 11555888 | 5733 | G | A | G | 0.93548 | A | 0.06452 | 4 | 0.12903 |
| 14248 | S18_11555990 | 18 | 11555990 | 102 | C | G | C | 0.87097 | G | 0.12903 | 8 | 0.25806 |
| 14249 | S18_11822509 | 18 | 11822509 | 266519 | G | A | G | 0.93548 | A | 0.06452 | 4 | 0.12903 |
| 14250 | S18_12019361 | 18 | 12019361 | 196852 | C | T | C | 0.83871 | T | 0.16129 | 10 | 0.32258 |
| 14251 | S18_12019418 | 18 | 12019418 | 57 | G | A | A | 0.56452 | G | 0.43548 | 17 | 0.54839 |
| 14252 | S18_12584876 | 18 | 12584876 | 565458 | C | A | A | 0.59677 | C | 0.40323 | 13 | 0.41935 |
| 14253 | S18_13175762 | 18 | 13175762 | 590886 | T | G | T | 0.79032 | G | 0.20968 | 9 | 0.29032 |
| 14254 | S18_13175863 | 18 | 13175863 | 101 | G | C | G | 0.83871 | C | 0.16129 | 8 | 0.25806 |

| Site number | SNP name | Chromosome | Physical position | Physical distance from the previous SNP | Reference allele | Alternative allele | Major allele | Major allele frequency | Minor allele | Minor allele frequency | Number heterozygous | Heterozygosity |
|---|---|---|---|---|---|---|---|---|---|---|---|---|
| 14255 | S18_13175884 | 18 | 13175884 | 21 | C | G | C | 0.79032 | G | 0.20968 | 9 | 0.29032 |
| 14256 | S18_13175922 | 18 | 13175922 | 38 | C | T | C | 0.83871 | T | 0.16129 | 8 | 0.25806 |
| 14257 | S18_13200544 | 18 | 13200544 | 24622 | C | T | C | 0.77419 | T | 0.22581 | 12 | 0.3871 |
| 14258 | S18_13200588 | 18 | 13200588 | 44 | C | T | C | 0.93548 | T | 0.06452 | 4 | 0.12903 |
| 14259 | S18_13200639 | 18 | 13200639 | 51 | G | A | G | 0.75806 | A | 0.24194 | 9 | 0.29032 |
| 14260 | S18_13237191 | 18 | 13237191 | 36552 | T | C | T | 0.87097 | C | 0.12903 | 4 | 0.12903 |
| 14261 | S18_13652375 | 18 | 13652375 | 415184 | C | G | C | 0.93548 | G | 0.06452 | 4 | 0.12903 |
| 14262 | S18_13652443 | 18 | 13652443 | 68 | C | T | C | 0.72581 | T | 0.27419 | 13 | 0.41935 |
| 14263 | S18_13652490 | 18 | 13652490 | 47 | C | A | C | 0.87097 | A | 0.12903 | 8 | 0.25806 |
| 14264 | S18_13856543 | 18 | 13856543 | 204053 | T | C | T | 0.69355 | C | 0.30645 | 15 | 0.48387 |
| 14265 | S18_13867614 | 18 | 13867614 | 11071 | T | C | C | 0.83871 | T | 0.16129 | 8 | 0.25806 |
| 14266 | S18_14077386 | 18 | 14077386 | 209772 | G | A | G | 0.91935 | A | 0.08065 | 5 | 0.16129 |
| 14267 | S18_14077489 | 18 | 14077489 | 103 | C | T | C | 0.91935 | T | 0.08065 | 5 | 0.16129 |
| 14268 | S18_14160691 | 18 | 14160691 | 83202 | C | T | C | 0.8871 | T | 0.1129 | 7 | 0.22581 |
| 14269 | S18_14177422 | 18 | 14177422 | 16731 | G | A | G | 0.8871 | A | 0.1129 | 7 | 0.22581 |
| 14270 | S18_14205457 | 18 | 14205457 | 28035 | C | T | C | 0.90323 | T | 0.09677 | 6 | 0.19355 |
| 14271 | S18_14205507 | 18 | 14205507 | 50 | T | C | C | 0.79032 | T | 0.20968 | 11 | 0.35484 |
| 14272 | S18_15853332 | 18 | 15853332 | 1647825 | C | T | C | 0.83871 | T | 0.16129 | 10 | 0.32258 |
| 14273 | S18_15862809 | 18 | 15862809 | 9477 | G | C | C | 0.83871 | G | 0.16129 | 10 | 0.32258 |
| 14274 | S18_15905220 | 18 | 15905220 | 42411 | T | C | T | 0.8871 | C | 0.1129 | 7 | 0.22581 |
| 14275 | S18_15905221 | 18 | 15905221 | 1 | G | A | G | 0.77419 | A | 0.22581 | 12 | 0.3871 |
| 14276 | S18_16017328 | 18 | 16017328 | 112107 | G | T | G | 0.90323 | T | 0.09677 | 6 | 0.19355 |
| 14277 | S18_16527235 | 18 | 16527235 | 509907 | T | C | T | 0.90323 | C | 0.09677 | 4 | 0.12903 |
| 14278 | S18_16527267 | 18 | 16527267 | 32 | T | C | C | 0.79032 | T | 0.20968 | 11 | 0.35484 |
| 14279 | S18_16592371 | 18 | 16592371 | 65104 | T | G | G | 0.82258 | T | 0.17742 | 11 | 0.35484 |
| 14280 | S18_16592396 | 18 | 16592396 | 25 | A | G | A | 0.91935 | G | 0.08065 | 5 | 0.16129 |
| 14281 | S18_16710919 | 18 | 16710919 | 118523 | T | C | C | 0.93548 | T | 0.06452 | 4 | 0.12903 |
| 14282 | S18_16710972 | 18 | 16710972 | 53 | G | A | G | 0.8871 | A | 0.1129 | 7 | 0.22581 |
| 14283 | S18_16935244 | 18 | 16935244 | 224272 | A | G | G | 0.91935 | A | 0.08065 | 5 | 0.16129 |
| 14284 | S18_16973984 | 18 | 16973984 | 38740 | C | T | C | 0.93548 | T | 0.06452 | 4 | 0.12903 |
| 14285 | S18_17016156 | 18 | 17016156 | 42172 | T | C | T | 0.77419 | C | 0.22581 | 12 | 0.3871 |
| 14286 | S18_17210661 | 18 | 17210661 | 194505 | C | T | C | 0.80645 | T | 0.19355 | 8 | 0.25806 |
| 14287 | S18_17210686 | 18 | 17210686 | 25 | A | G | A | 0.95161 | G | 0.04839 | 3 | 0.09677 |
| 14288 | S18_17210814 | 18 | 17210814 | 128 | C | T | C | 0.90323 | T | 0.09677 | 4 | 0.12903 |
| 14289 | S18_17210821 | 18 | 17210821 | 7 | T | C | T | 0.74194 | C | 0.25806 | 12 | 0.3871 |
| 14290 | S18_17812653 | 18 | 17812653 | 601832 | T | C | T | 0.69355 | C | 0.30645 | 13 | 0.41935 |
| 14291 | S18_17812654 | 18 | 17812654 | 1 | G | A | G | 0.69355 | A | 0.30645 | 13 | 0.41935 |
| 14292 | S18_17957128 | 18 | 17957128 | 144474 | A | G | G | 0.69355 | A | 0.30645 | 9 | 0.29032 |
| 14293 | S18_17957146 | 18 | 17957146 | 18 | A | G | G | 0.69355 | A | 0.30645 | 9 | 0.29032 |
| 14294 | S18_18052540 | 18 | 18052540 | 95394 | G | A | G | 0.58065 | A | 0.41935 | 16 | 0.51613 |
| 14295 | S18_18257202 | 18 | 18257202 | 204662 | C | T | C | 0.95161 | T | 0.04839 | 3 | 0.09677 |
| 14296 | S18_18257259 | 18 | 18257259 | 57 | G | A | G | 0.90323 | A | 0.09677 | 6 | 0.19355 |
| 14297 | S18_18257286 | 18 | 18257286 | 27 | A | G | A | 0.90323 | G | 0.09677 | 6 | 0.19355 |
| 14298 | S18_18363417 | 18 | 18363417 | 106131 | A | T | A | 0.91935 | T | 0.08065 | 5 | 0.16129 |
| 14299 | S18_18471427 | 18 | 18471427 | 108010 | C | T | C | 0.77419 | T | 0.22581 | 12 | 0.3871 |
| 14300 | S18_18500497 | 18 | 18500497 | 29070 | C | A | C | 0.82258 | A | 0.17742 | 9 | 0.29032 |
| 14301 | S18_18500567 | 18 | 18500567 | 70 | G | C | G | 0.79032 | C | 0.20968 | 11 | 0.35484 |
| 14302 | S18_18500673 | 18 | 18500673 | 106 | C | G | G | 0.6129 | C | 0.3871 | 12 | 0.3871 |
| 14303 | S18_18759403 | 18 | 18759403 | 258730 | T | C | T | 0.74194 | C | 0.25806 | 12 | 0.3871 |
| 14304 | S18_18857380 | 18 | 18857380 | 97977 | C | A | C | 0.91935 | A | 0.08065 | 3 | 0.09677 |
| 14305 | S18_18947761 | 18 | 18947761 | 90381 | G | A | G | 0.80645 | A | 0.19355 | 8 | 0.25806 |
| 14306 | S18_19699035 | 18 | 19699035 | 751274 | C | T | C | 0.6129 | T | 0.3871 | 16 | 0.51613 |
| 14307 | S18_20148464 | 18 | 20148464 | 449429 | C | T | C | 0.82258 | T | 0.17742 | 11 | 0.35484 |
| 14308 | S18_20148515 | 18 | 20148515 | 51 | C | T | C | 0.82258 | T | 0.17742 | 11 | 0.35484 |

| Site number | SNP name | Chromosome | Physical position | Physical distance from the previous SNP | Reference allele | Alternative allele | Major allele | Major allele frequency | Minor allele | Minor allele frequency | Number heterozygous | Heterozygosity |
|---|---|---|---|---|---|---|---|---|---|---|---|---|
| 14309 | S18_20148519 | 18 | 20148519 | 4 | C | T | T | 0.82258 | C | 0.17742 | 9 | 0.29032 |
| 14310 | S18_20148625 | 18 | 20148625 | 106 | C | G | G | 0.90323 | C | 0.09677 | 6 | 0.19355 |
| 14311 | S18_20309521 | 18 | 20309521 | 160896 | C | T | C | 0.70968 | T | 0.29032 | 12 | 0.3871 |
| 14312 | S18_20309584 | 18 | 20309584 | 63 | C | T | C | 0.87097 | T | 0.12903 | 4 | 0.12903 |
| 14313 | S18_20309618 | 18 | 20309618 | 34 | A | C | A | 0.95161 | C | 0.04839 | 3 | 0.09677 |
| 14314 | S18_20538765 | 18 | 20538765 | 229147 | A | C | C | 0.51613 | A | 0.48387 | 16 | 0.51613 |
| 14315 | S18_20538786 | 18 | 20538786 | 21 | T | C | C | 0.85484 | T | 0.14516 | 9 | 0.29032 |
| 14316 | S18_20538793 | 18 | 20538793 | 7 | G | A | G | 0.93548 | A | 0.06452 | 4 | 0.12903 |
| 14317 | S18_22038956 | 18 | 22038956 | 1500163 | C | T | C | 0.66129 | T | 0.33871 | 19 | 0.6129 |
| 14318 | S18_22546771 | 18 | 22546771 | 507815 | A | G | A | 0.91935 | G | 0.08065 | 5 | 0.16129 |
| 14319 | S18_22603335 | 18 | 22603335 | 56564 | G | A | G | 0.90323 | A | 0.09677 | 6 | 0.19355 |
| 14320 | S18_22603383 | 18 | 22603383 | 48 | G | T | G | 0.90323 | T | 0.09677 | 6 | 0.19355 |
| 14321 | S18_22603512 | 18 | 22603512 | 129 | A | G | A | 0.90323 | G | 0.09677 | 6 | 0.19355 |
| 14322 | S18_22807481 | 18 | 22807481 | 203969 | A | T | T | 0.74194 | A | 0.25806 | 14 | 0.45161 |
| 14323 | S18_22807566 | 18 | 22807566 | 85 | G | A | G | 0.95161 | A | 0.04839 | 3 | 0.09677 |
| 14324 | S18_22807620 | 18 | 22807620 | 54 | G | A | G | 0.64516 | A | 0.35484 | 14 | 0.45161 |
| 14325 | S18_23534449 | 18 | 23534449 | 726829 | T | C | T | 0.93548 | C | 0.06452 | 4 | 0.12903 |
| 14326 | S18_23716787 | 18 | 23716787 | 182338 | C | T | C | 0.59677 | T | 0.40323 | 11 | 0.35484 |
| 14327 | S18_23816338 | 18 | 23816338 | 99551 | T | A | T | 0.70968 | A | 0.29032 | 14 | 0.45161 |
| 14328 | S18_23816452 | 18 | 23816452 | 114 | T | C | C | 0.70968 | T | 0.29032 | 12 | 0.3871 |
| 14329 | S18_23868722 | 18 | 23868722 | 52270 | C | T | C | 0.95161 | T | 0.04839 | 3 | 0.09677 |
| 14330 | S18_23884537 | 18 | 23884537 | 15815 | G | T | G | 0.80645 | T | 0.19355 | 8 | 0.25806 |
| 14331 | S18_23884545 | 18 | 23884545 | 8 | C | T | C | 0.8871 | T | 0.1129 | 7 | 0.22581 |
| 14332 | S18_23884605 | 18 | 23884605 | 60 | A | C | A | 0.69355 | C | 0.30645 | 15 | 0.48387 |
| 14333 | S18_23884669 | 18 | 23884669 | 64 | A | G | A | 0.70968 | G | 0.29032 | 14 | 0.45161 |
| 14334 | S18_23884681 | 18 | 23884681 | 12 | T | C | T | 0.69355 | C | 0.30645 | 15 | 0.48387 |
| 14335 | S18_23884750 | 18 | 23884750 | 69 | A | G | A | 0.70968 | G | 0.29032 | 14 | 0.45161 |
| 14336 | S18_23884787 | 18 | 23884787 | 37 | C | T | C | 0.91935 | T | 0.08065 | 5 | 0.16129 |
| 14337 | S18_23940354 | 18 | 23940354 | 55567 | A | T | A | 0.95161 | T | 0.04839 | 3 | 0.09677 |
| 14338 | S18_23940405 | 18 | 23940405 | 51 | G | T | G | 0.77419 | T | 0.22581 | 10 | 0.32258 |
| 14339 | S18_23946410 | 18 | 23946410 | 6005 | G | A | G | 0.54839 | A | 0.45161 | 20 | 0.64516 |
| 14340 | S18_23946528 | 18 | 23946528 | 118 | C | T | C | 0.83871 | T | 0.16129 | 8 | 0.25806 |
| 14341 | S18_24025812 | 18 | 24025812 | 79284 | C | T | C | 0.83871 | T | 0.16129 | 10 | 0.32258 |
| 14342 | S18_24025865 | 18 | 24025865 | 53 | T | A | T | 0.83871 | A | 0.16129 | 10 | 0.32258 |
| 14343 | S18_24025906 | 18 | 24025906 | 41 | G | A | G | 0.62903 | A | 0.37097 | 19 | 0.6129 |
| 14344 | S18_24025963 | 18 | 24025963 | 57 | T | C | T | 0.59677 | C | 0.40323 | 21 | 0.67742 |
| 14345 | S18_24048709 | 18 | 24048709 | 22746 | A | G | A | 0.87097 | G | 0.12903 | 8 | 0.25806 |
| 14346 | S18_24048741 | 18 | 24048741 | 32 | A | G | A | 0.75806 | G | 0.24194 | 9 | 0.29032 |
| 14347 | S18_24048822 | 18 | 24048822 | 81 | C | G | C | 0.87097 | G | 0.12903 | 8 | 0.25806 |
| 14348 | S18_24345948 | 18 | 24345948 | 297126 | C | T | T | 0.51613 | C | 0.48387 | 12 | 0.3871 |
| 14349 | S18_24346058 | 18 | 24346058 | 110 | A | G | G | 0.87097 | A | 0.12903 | 8 | 0.25806 |
| 14350 | S18_24346069 | 18 | 24346069 | 11 | G | T | T | 0.72581 | G | 0.27419 | 15 | 0.48387 |
| 14351 | S18_24346096 | 18 | 24346096 | 27 | T | C | C | 0.87097 | T | 0.12903 | 8 | 0.25806 |
| 14352 | S18_24346097 | 18 | 24346097 | 1 | G | A | A | 0.72581 | G | 0.27419 | 15 | 0.48387 |
| 14353 | S18_24346122 | 18 | 24346122 | 25 | T | C | C | 0.72581 | T | 0.27419 | 15 | 0.48387 |
| 14354 | S18_24453623 | 18 | 24453623 | 107501 | T | C | C | 0.70968 | T | 0.29032 | 10 | 0.32258 |
| 14355 | S18_24453624 | 18 | 24453624 | 1 | G | A | G | 0.90323 | A | 0.09677 | 4 | 0.12903 |
| 14356 | S18_24453676 | 18 | 24453676 | 52 | T | A | T | 0.69355 | A | 0.30645 | 11 | 0.35484 |
| 14357 | S18_24687205 | 18 | 24687205 | 233529 | A | G | A | 0.91935 | G | 0.08065 | 5 | 0.16129 |
| 14358 | S18_24688329 | 18 | 24688329 | 1124 | G | A | G | 0.83871 | A | 0.16129 | 10 | 0.32258 |
| 14359 | S18_24688365 | 18 | 24688365 | 36 | C | T | C | 0.51613 | T | 0.48387 | 14 | 0.45161 |
| 14360 | S18_24726494 | 18 | 24726494 | 38129 | C | T | C | 0.83871 | T | 0.16129 | 10 | 0.32258 |
| 14361 | S18_24726515 | 18 | 24726515 | 21 | C | T | C | 0.51613 | T | 0.48387 | 12 | 0.3871 |
| 14362 | S18_24726516 | 18 | 24726516 | 1 | T | C | T | 0.51613 | C | 0.48387 | 12 | 0.3871 |

| Site number | SNP name | Chromosome | Physical position | Physical distance from the previous SNP | Reference allele | Alternative allele | Major allele | Major allele frequency | Minor allele | Minor allele frequency | Number heterozygous | Heterozygosity |
|---|---|---|---|---|---|---|---|---|---|---|---|---|
| 14363 | S18_24777458 | 18 | 24777458 | 50942 | A | G | A | 0.90323 | G | 0.09677 | 6 | 0.19355 |
| 14364 | S18_25041926 | 18 | 25041926 | 264468 | G | A | G | 0.90323 | A | 0.09677 | 4 | 0.12903 |
| 14365 | S18_25047626 | 18 | 25047626 | 5700 | C | T | T | 0.64516 | C | 0.35484 | 12 | 0.3871 |
| 14366 | S18_25047627 | 18 | 25047627 | 1 | A | G | G | 0.64516 | A | 0.35484 | 12 | 0.3871 |
| 14367 | S18_25203178 | 18 | 25203178 | 155551 | C | T | T | 0.56452 | C | 0.43548 | 17 | 0.54839 |
| 14368 | S18_25428381 | 18 | 25428381 | 225203 | C | G | C | 0.95161 | G | 0.04839 | 3 | 0.09677 |
| 14369 | S18_25965035 | 18 | 25965035 | 536654 | C | T | C | 0.93548 | T | 0.06452 | 4 | 0.12903 |
| 14370 | S18_25965040 | 18 | 25965040 | 5 | A | G | G | 0.90323 | A | 0.09677 | 6 | 0.19355 |
| 14371 | S18_26394977 | 18 | 26394977 | 429937 | A | G | A | 0.82258 | G | 0.17742 | 9 | 0.29032 |
| 14372 | S18_26419282 | 18 | 26419282 | 24305 | G | T | G | 0.67742 | T | 0.32258 | 18 | 0.58065 |
| 14373 | S18_26419291 | 18 | 26419291 | 9 | G | T | G | 0.67742 | T | 0.32258 | 18 | 0.58065 |
| 14374 | S18_26898597 | 18 | 26898597 | 479306 | C | G | C | 0.77419 | G | 0.22581 | 14 | 0.45161 |
| 14375 | S18_26953139 | 18 | 26953139 | 54542 | G | C | G | 0.58065 | C | 0.41935 | 20 | 0.64516 |
| 14376 | S18_26953284 | 18 | 26953284 | 145 | G | C | G | 0.58065 | C | 0.41935 | 20 | 0.64516 |
| 14377 | S18_27356692 | 18 | 27356692 | 403408 | A | G | A | 0.85484 | G | 0.14516 | 5 | 0.16129 |
| 14378 | S18_27554803 | 18 | 27554803 | 198111 | C | T | C | 0.87097 | T | 0.12903 | 4 | 0.12903 |
| 14379 | S18_27554904 | 18 | 27554904 | 101 | C | T | C | 0.74194 | T | 0.25806 | 10 | 0.32258 |
| 14380 | S18_27597615 | 18 | 27597615 | 42711 | T | C | T | 0.64516 | C | 0.35484 | 12 | 0.3871 |
| 14381 | S18_27597669 | 18 | 27597669 | 54 | C | T | C | 0.79032 | T | 0.20968 | 9 | 0.29032 |
| 14382 | S18_27739983 | 18 | 27739983 | 142314 | A | G | A | 0.90323 | G | 0.09677 | 6 | 0.19355 |
| 14383 | S18_27801455 | 18 | 27801455 | 61472 | A | G | G | 0.79032 | A | 0.20968 | 7 | 0.22581 |
| 14384 | S18_27801457 | 18 | 27801457 | 2 | G | A | A | 0.79032 | G | 0.20968 | 7 | 0.22581 |
| 14385 | S18_27883362 | 18 | 27883362 | 81905 | A | C | C | 0.62903 | A | 0.37097 | 15 | 0.48387 |
| 14386 | S18_27883470 | 18 | 27883470 | 108 | A | G | G | 0.64516 | A | 0.35484 | 14 | 0.45161 |
| 14387 | S18_28532655 | 18 | 28532655 | 649185 | A | G | A | 0.5 | G | 0.5 | 11 | 0.35484 |
| 14388 | S18_28532685 | 18 | 28532685 | 30 | A | G | A | 0.64516 | G | 0.35484 | 14 | 0.45161 |
| 14389 | S18_28532700 | 18 | 28532700 | 15 | G | A | G | 0.93548 | A | 0.06452 | 4 | 0.12903 |
| 14390 | S18_28532752 | 18 | 28532752 | 52 | G | A | G | 0.93548 | A | 0.06452 | 4 | 0.12903 |
| 14391 | S18_28532822 | 18 | 28532822 | 70 | A | G | A | 0.70968 | G | 0.29032 | 12 | 0.3871 |
| 14392 | S18_29109632 | 18 | 29109632 | 576810 | G | A | G | 0.95161 | A | 0.04839 | 3 | 0.09677 |
| 14393 | S18_29161626 | 18 | 29161626 | 51994 | G | A | A | 0.74194 | G | 0.25806 | 12 | 0.3871 |
| 14394 | S18_29161709 | 18 | 29161709 | 83 | C | T | C | 0.93548 | T | 0.06452 | 4 | 0.12903 |
| 14395 | S18_29304121 | 18 | 29304121 | 142412 | T | A | T | 0.93548 | A | 0.06452 | 4 | 0.12903 |
| 14396 | S18_29313432 | 18 | 29313432 | 9311 | A | G | G | 0.53226 | A | 0.46774 | 15 | 0.48387 |
| 14397 | S18_29795763 | 18 | 29795763 | 482331 | C | T | C | 0.8871 | T | 0.1129 | 7 | 0.22581 |
| 14398 | S18_29795935 | 18 | 29795935 | 172 | G | A | G | 0.8871 | A | 0.1129 | 7 | 0.22581 |
| 14399 | S18_30635530 | 18 | 30635530 | 839595 | T | C | T | 0.90323 | C | 0.09677 | 6 | 0.19355 |
| 14400 | S18_30635532 | 18 | 30635532 | 2 | C | T | C | 0.70968 | T | 0.29032 | 14 | 0.45161 |
| 14401 | S18_30635651 | 18 | 30635651 | 119 | C | T | C | 0.90323 | T | 0.09677 | 6 | 0.19355 |
| 14402 | S18_30635652 | 18 | 30635652 | 1 | G | A | G | 0.83871 | A | 0.16129 | 8 | 0.25806 |
| 14403 | S18_30635678 | 18 | 30635678 | 26 | A | G | G | 0.64516 | A | 0.35484 | 18 | 0.58065 |
| 14404 | S18_30635731 | 18 | 30635731 | 53 | C | T | C | 0.93548 | T | 0.06452 | 4 | 0.12903 |
| 14405 | S18_30635732 | 18 | 30635732 | 1 | T | G | G | 0.91935 | T | 0.08065 | 5 | 0.16129 |
| 14406 | S18_30637891 | 18 | 30637891 | 2159 | C | T | T | 0.83871 | C | 0.16129 | 10 | 0.32258 |
| 14407 | S18_30816155 | 18 | 30816155 | 178264 | C | G | C | 0.91935 | G | 0.08065 | 5 | 0.16129 |
| 14408 | S18_30816338 | 18 | 30816338 | 183 | G | A | G | 0.87097 | A | 0.12903 | 8 | 0.25806 |
| 14409 | S18_31436695 | 18 | 31436695 | 620357 | A | G | A | 0.90323 | G | 0.09677 | 6 | 0.19355 |
| 14410 | S18_32033017 | 18 | 32033017 | 596322 | T | G | G | 0.64516 | T | 0.35484 | 16 | 0.51613 |
| 14411 | S18_32193776 | 18 | 32193776 | 160759 | G | C | G | 0.85484 | C | 0.14516 | 7 | 0.22581 |
| 14412 | S18_33220097 | 18 | 33220097 | 1026321 | G | A | G | 0.90323 | A | 0.09677 | 6 | 0.19355 |
| 14413 | S18_33395257 | 18 | 33395257 | 175160 | A | C | A | 0.95161 | C | 0.04839 | 3 | 0.09677 |
| 14414 | S18_33395262 | 18 | 33395262 | 5 | G | T | G | 0.95161 | T | 0.04839 | 3 | 0.09677 |
| 14415 | S18_33395395 | 18 | 33395395 | 133 | G | A | G | 0.95161 | A | 0.04839 | 3 | 0.09677 |
| 14416 | S18_33843888 | 18 | 33843888 | 448493 | C | G | G | 0.54839 | C | 0.45161 | 14 | 0.45161 |

| Site number | SNP name | Chromosome | Physical position | Physical distance from the previous SNP | Reference allele | Alternative allele | Major allele | Major allele frequency | Minor allele | Minor allele frequency | Number heterozygous | Heterozygosity |
|---|---|---|---|---|---|---|---|---|---|---|---|---|
| 14417 | S18_34114749 | 18 | 34114749 | 270861 | T | C | T | 0.90323 | C | 0.09677 | 6 | 0.19355 |
| 14418 | S18_34335642 | 18 | 34335642 | 220893 | G | A | G | 0.77419 | A | 0.22581 | 12 | 0.3871 |
| 14419 | S18_34335759 | 18 | 34335759 | 117 | T | C | T | 0.77419 | C | 0.22581 | 12 | 0.3871 |
| 14420 | S18_35447243 | 18 | 35447243 | 1111484 | C | A | A | 0.53226 | C | 0.46774 | 15 | 0.48387 |
| 14421 | S18_35596894 | 18 | 35596894 | 149651 | A | G | G | 0.95161 | A | 0.04839 | 3 | 0.09677 |
| 14422 | S18_35654646 | 18 | 35654646 | 57752 | T | C | C | 0.51613 | T | 0.48387 | 14 | 0.45161 |
| 14423 | S18_35714481 | 18 | 35714481 | 59835 | G | A | G | 0.90323 | A | 0.09677 | 6 | 0.19355 |
| 14424 | S18_36049714 | 18 | 36049714 | 335233 | G | A | A | 0.82258 | G | 0.17742 | 9 | 0.29032 |
| 14425 | S18_36049768 | 18 | 36049768 | 54 | A | G | G | 0.70968 | A | 0.29032 | 16 | 0.51613 |
| 14426 | S18_36591170 | 18 | 36591170 | 541402 | T | C | T | 0.90323 | C | 0.09677 | 6 | 0.19355 |
| 14427 | S18_36757717 | 18 | 36757717 | 166547 | G | A | A | 0.64516 | G | 0.35484 | 12 | 0.3871 |
| 14428 | S18_37339820 | 18 | 37339820 | 582103 | T | C | T | 0.91935 | C | 0.08065 | 5 | 0.16129 |
| 14429 | S18_37712400 | 18 | 37712400 | 372580 | A | G | A | 0.53226 | G | 0.46774 | 17 | 0.54839 |
| 14430 | S18_37712484 | 18 | 37712484 | 84 | C | G | C | 0.59677 | G | 0.40323 | 13 | 0.41935 |
| 14431 | S18_38077733 | 18 | 38077733 | 365249 | A | C | A | 0.93548 | C | 0.06452 | 4 | 0.12903 |
| 14432 | S18_38077793 | 18 | 38077793 | 60 | G | A | A | 0.5 | G | 0.5 | 15 | 0.48387 |
| 14433 | S18_38077898 | 18 | 38077898 | 105 | C | T | C | 0.95161 | T | 0.04839 | 3 | 0.09677 |
| 14434 | S18_38552642 | 18 | 38552642 | 474744 | T | C | T | 0.54839 | C | 0.45161 | 12 | 0.3871 |
| 14435 | S18_38552736 | 18 | 38552736 | 94 | G | A | G | 0.82258 | A | 0.17742 | 7 | 0.22581 |
| 14436 | S18_38689670 | 18 | 38689670 | 136934 | C | T | T | 0.93548 | C | 0.06452 | 4 | 0.12903 |
| 14437 | S18_38689774 | 18 | 38689774 | 104 | C | T | T | 0.91935 | C | 0.08065 | 5 | 0.16129 |
| 14438 | S18_38692193 | 18 | 38692193 | 2419 | G | A | G | 0.90323 | A | 0.09677 | 6 | 0.19355 |
| 14439 | S18_39020251 | 18 | 39020251 | 328058 | T | C | C | 0.90323 | T | 0.09677 | 4 | 0.12903 |
| 14440 | S18_39020292 | 18 | 39020292 | 41 | A | G | A | 0.91935 | G | 0.08065 | 5 | 0.16129 |
| 14441 | S18_40276621 | 18 | 40276621 | 1256329 | G | C | G | 0.90323 | C | 0.09677 | 6 | 0.19355 |
| 14442 | S18_40276701 | 18 | 40276701 | 80 | C | A | C | 0.90323 | A | 0.09677 | 6 | 0.19355 |
| 14443 | S18_40276791 | 18 | 40276791 | 90 | T | C | T | 0.93548 | C | 0.06452 | 4 | 0.12903 |
| 14444 | S18_40412944 | 18 | 40412944 | 136153 | G | A | G | 0.82258 | A | 0.17742 | 9 | 0.29032 |
| 14445 | S18_40412981 | 18 | 40412981 | 37 | G | A | G | 0.93548 | A | 0.06452 | 4 | 0.12903 |
| 14446 | S18_40752396 | 18 | 40752396 | 339415 | G | A | G | 0.83871 | A | 0.16129 | 10 | 0.32258 |
| 14447 | S18_40777918 | 18 | 40777918 | 25522 | T | C | T | 0.93548 | C | 0.06452 | 4 | 0.12903 |
| 14448 | S18_41636961 | 18 | 41636961 | 859043 | C | T | C | 0.75806 | T | 0.24194 | 9 | 0.29032 |
| 14449 | S18_41637003 | 18 | 41637003 | 42 | G | A | G | 0.93548 | A | 0.06452 | 4 | 0.12903 |
| 14450 | S18_41912372 | 18 | 41912372 | 275369 | A | G | A | 0.87097 | G | 0.12903 | 8 | 0.25806 |
| 14451 | S18_41912466 | 18 | 41912466 | 94 | G | A | G | 0.93548 | A | 0.06452 | 4 | 0.12903 |
| 14452 | S18_42089518 | 18 | 42089518 | 177052 | T | C | C | 0.8871 | T | 0.1129 | 7 | 0.22581 |
| 14453 | S18_42089570 | 18 | 42089570 | 52 | G | A | G | 0.51613 | A | 0.48387 | 16 | 0.51613 |
| 14454 | S18_42089603 | 18 | 42089603 | 33 | T | C | T | 0.70968 | C | 0.29032 | 12 | 0.3871 |
| 14455 | S18_42089624 | 18 | 42089624 | 21 | A | G | A | 0.83871 | G | 0.16129 | 6 | 0.19355 |
| 14456 | S18_42338891 | 18 | 42338891 | 249267 | G | C | G | 0.79032 | C | 0.20968 | 11 | 0.35484 |
| 14457 | S18_42338944 | 18 | 42338944 | 53 | A | G | A | 0.95161 | G | 0.04839 | 3 | 0.09677 |
| 14458 | S18_42497260 | 18 | 42497260 | 158316 | G | A | G | 0.90323 | A | 0.09677 | 6 | 0.19355 |
| 14459 | S18_42497275 | 18 | 42497275 | 15 | C | T | C | 0.90323 | T | 0.09677 | 6 | 0.19355 |
| 14460 | S18_42497368 | 18 | 42497368 | 93 | G | A | G | 0.93548 | A | 0.06452 | 4 | 0.12903 |
| 14461 | S18_42497381 | 18 | 42497381 | 13 | T | C | T | 0.95161 | C | 0.04839 | 3 | 0.09677 |
| 14462 | S18_42734614 | 18 | 42734614 | 237233 | C | T | C | 0.79032 | T | 0.20968 | 9 | 0.29032 |
| 14463 | S18_42734661 | 18 | 42734661 | 47 | T | C | T | 0.90323 | C | 0.09677 | 6 | 0.19355 |
| 14464 | S18_42734697 | 18 | 42734697 | 36 | T | C | T | 0.90323 | C | 0.09677 | 6 | 0.19355 |
| 14465 | S18_42734721 | 18 | 42734721 | 24 | T | A | T | 0.91935 | A | 0.08065 | 5 | 0.16129 |
| 14466 | S18_43238439 | 18 | 43238439 | 503718 | T | C | T | 0.93548 | C | 0.06452 | 4 | 0.12903 |
| 14467 | S18_43238519 | 18 | 43238519 | 80 | A | G | A | 0.87097 | G | 0.12903 | 8 | 0.25806 |
| 14468 | S18_43238608 | 18 | 43238608 | 89 | A | C | A | 0.87097 | C | 0.12903 | 8 | 0.25806 |
| 14469 | S18_43283205 | 18 | 43283205 | 44597 | A | G | A | 0.93548 | G | 0.06452 | 2 | 0.06452 |
| 14470 | S18_43283322 | 18 | 43283322 | 117 | A | G | A | 0.93548 | G | 0.06452 | 2 | 0.06452 |

| Site number | SNP name | Chromosome | Physical position | Physical distance from the previous SNP | Reference allele | Alternative allele | Major allele | Major allele frequency | Minor allele | Minor allele frequency | Number heterozygous | Heterozygosity |
|---|---|---|---|---|---|---|---|---|---|---|---|---|
| 14471 | S18_43385184 | 18 | 43385184 | 101862 | A | G | A | 0.91935 | G | 0.08065 | 5 | 0.16129 |
| 14472 | S18_43385191 | 18 | 43385191 | 7 | T | C | T | 0.93548 | C | 0.06452 | 4 | 0.12903 |
| 14473 | S18_43385236 | 18 | 43385236 | 45 | C | T | C | 0.8871 | T | 0.1129 | 7 | 0.22581 |
| 14474 | S18_43385288 | 18 | 43385288 | 52 | T | C | T | 0.91935 | C | 0.08065 | 3 | 0.09677 |
| 14475 | S18_43385301 | 18 | 43385301 | 13 | T | A | A | 0.90323 | T | 0.09677 | 6 | 0.19355 |
| 14476 | S18_43461292 | 18 | 43461292 | 75991 | G | A | A | 0.54839 | G | 0.45161 | 18 | 0.58065 |
| 14477 | S18_43960088 | 18 | 43960088 | 498796 | C | A | C | 0.83871 | A | 0.16129 | 6 | 0.19355 |
| 14478 | S18_43960091 | 18 | 43960091 | 3 | C | G | C | 0.82258 | G | 0.17742 | 7 | 0.22581 |
| 14479 | S18_44010004 | 18 | 44010004 | 49913 | A | G | A | 0.91935 | G | 0.08065 | 3 | 0.09677 |
| 14480 | S18_44193243 | 18 | 44193243 | 183239 | T | C | C | 0.58065 | T | 0.41935 | 16 | 0.51613 |
| 14481 | S18_44193276 | 18 | 44193276 | 33 | T | G | T | 0.51613 | G | 0.48387 | 16 | 0.51613 |
| 14482 | S18_44193369 | 18 | 44193369 | 93 | T | C | T | 0.51613 | C | 0.48387 | 16 | 0.51613 |
| 14483 | S18_44320194 | 18 | 44320194 | 126825 | A | G | G | 0.66129 | A | 0.33871 | 17 | 0.54839 |
| 14484 | S18_44320376 | 18 | 44320376 | 182 | A | G | A | 0.79032 | G | 0.20968 | 9 | 0.29032 |
| 14485 | S18_44407354 | 18 | 44407354 | 86978 | G | A | G | 0.67742 | A | 0.32258 | 14 | 0.45161 |
| 14486 | S18_44441002 | 18 | 44441002 | 33648 | T | C | T | 0.79032 | C | 0.20968 | 9 | 0.29032 |
| 14487 | S18_44441027 | 18 | 44441027 | 25 | A | G | A | 0.91935 | G | 0.08065 | 5 | 0.16129 |
| 14488 | S18_44441029 | 18 | 44441029 | 2 | C | T | C | 0.90323 | T | 0.09677 | 6 | 0.19355 |
| 14489 | S18_44441097 | 18 | 44441097 | 68 | A | G | A | 0.95161 | G | 0.04839 | 3 | 0.09677 |
| 14490 | S18_44441200 | 18 | 44441200 | 103 | C | T | T | 0.91935 | C | 0.08065 | 5 | 0.16129 |
| 14491 | S18_44556524 | 18 | 44556524 | 115324 | C | T | C | 0.90323 | T | 0.09677 | 6 | 0.19355 |
| 14492 | S18_44969732 | 18 | 44969732 | 413208 | C | T | T | 0.93548 | C | 0.06452 | 4 | 0.12903 |
| 14493 | S18_44969919 | 18 | 44969919 | 187 | T | G | T | 0.93548 | G | 0.06452 | 4 | 0.12903 |
| 14494 | S18_45163447 | 18 | 45163447 | 193528 | C | T | C | 0.77419 | T | 0.22581 | 10 | 0.32258 |
| 14495 | S18_45163471 | 18 | 45163471 | 24 | A | G | A | 0.77419 | G | 0.22581 | 12 | 0.3871 |
| 14496 | S18_45163562 | 18 | 45163562 | 91 | C | T | C | 0.93548 | T | 0.06452 | 4 | 0.12903 |
| 14497 | S18_45245112 | 18 | 45245112 | 81550 | T | C | C | 0.5 | T | 0.5 | 15 | 0.48387 |
| 14498 | S18_45245244 | 18 | 45245244 | 132 | T | C | C | 0.95161 | T | 0.04839 | 1 | 0.03226 |
| 14499 | S18_45245292 | 18 | 45245292 | 48 | C | T | T | 0.53226 | C | 0.46774 | 15 | 0.48387 |
| 14500 | S18_45245307 | 18 | 45245307 | 15 | T | C | C | 0.53226 | T | 0.46774 | 15 | 0.48387 |
| 14501 | S18_45349479 | 18 | 45349479 | 104172 | G | A | G | 0.90323 | A | 0.09677 | 6 | 0.19355 |
| 14502 | S18_45841325 | 18 | 45841325 | 491846 | G | A | A | 0.56452 | G | 0.43548 | 15 | 0.48387 |
| 14503 | S18_46624356 | 18 | 46624356 | 783031 | G | A | G | 0.93548 | A | 0.06452 | 4 | 0.12903 |
| 14504 | S18_46728974 | 18 | 46728974 | 104618 | G | A | G | 0.80645 | A | 0.19355 | 8 | 0.25806 |
| 14505 | S18_46729152 | 18 | 46729152 | 178 | A | T | A | 0.80645 | T | 0.19355 | 8 | 0.25806 |
| 14506 | S18_46778291 | 18 | 46778291 | 49139 | T | C | T | 0.91935 | C | 0.08065 | 5 | 0.16129 |
| 14507 | S18_46778374 | 18 | 46778374 | 83 | G | A | G | 0.77419 | A | 0.22581 | 14 | 0.45161 |
| 14508 | S18_46778502 | 18 | 46778502 | 128 | C | T | C | 0.77419 | T | 0.22581 | 14 | 0.45161 |
| 14509 | S18_46841683 | 18 | 46841683 | 63181 | G | A | G | 0.83871 | A | 0.16129 | 10 | 0.32258 |
| 14510 | S18_46841726 | 18 | 46841726 | 43 | C | T | C | 0.8871 | T | 0.1129 | 7 | 0.22581 |
| 14511 | S18_46841770 | 18 | 46841770 | 44 | G | C | G | 0.90323 | C | 0.09677 | 6 | 0.19355 |
| 14512 | S18_46841839 | 18 | 46841839 | 69 | C | T | C | 0.8871 | T | 0.1129 | 7 | 0.22581 |
| 14513 | S18_46893590 | 18 | 46893590 | 51751 | G | A | G | 0.6129 | A | 0.3871 | 16 | 0.51613 |
| 14514 | S18_46936940 | 18 | 46936940 | 43350 | A | G | A | 0.83871 | G | 0.16129 | 8 | 0.25806 |
| 14515 | S18_46936965 | 18 | 46936965 | 25 | G | A | G | 0.67742 | A | 0.32258 | 18 | 0.58065 |
| 14516 | S18_46936967 | 18 | 46936967 | 2 | T | C | T | 0.93548 | C | 0.06452 | 4 | 0.12903 |
| 14517 | S18_46936993 | 18 | 46936993 | 26 | C | T | C | 0.91935 | T | 0.08065 | 5 | 0.16129 |
| 14518 | S18_46980191 | 18 | 46980191 | 43198 | C | T | C | 0.95161 | T | 0.04839 | 3 | 0.09677 |
| 14519 | S18_47178226 | 18 | 47178226 | 198035 | G | C | G | 0.93548 | C | 0.06452 | 4 | 0.12903 |
| 14520 | S18_47367976 | 18 | 47367976 | 189750 | G | A | G | 0.83871 | A | 0.16129 | 8 | 0.25806 |
| 14521 | S18_47368062 | 18 | 47368062 | 86 | G | A | G | 0.83871 | A | 0.16129 | 10 | 0.32258 |
| 14522 | S18_47754985 | 18 | 47754985 | 386923 | G | A | A | 0.5 | G | 0.5 | 17 | 0.54839 |
| 14523 | S18_48049590 | 18 | 48049590 | 294605 | C | T | C | 0.56452 | T | 0.43548 | 17 | 0.54839 |
| 14524 | S18_48466675 | 18 | 48466675 | 417085 | A | C | A | 0.95161 | C | 0.04839 | 3 | 0.09677 |

| Site number | SNP name | Chromosome | Physical position | Physical distance from the previous SNP | Reference allele | Alternative allele | Major allele | Major allele frequency | Minor allele | Minor allele frequency | Number heterozygous | Heterozygosity |
|---|---|---|---|---|---|---|---|---|---|---|---|---|
| 14525 | S18_48473860 | 18 | 48473860 | 7185 | G | C | G | 0.95161 | C | 0.04839 | 3 | 0.09677 |
| 14526 | S18_48474037 | 18 | 48474037 | 177 | G | A | G | 0.75806 | A | 0.24194 | 11 | 0.35484 |
| 14527 | S18_48684719 | 18 | 48684719 | 210682 | T | A | T | 0.93548 | A | 0.06452 | 4 | 0.12903 |
| 14528 | S18_48794616 | 18 | 48794616 | 109897 | A | G | G | 0.93548 | A | 0.06452 | 4 | 0.12903 |
| 14529 | S18_48794752 | 18 | 48794752 | 136 | G | A | G | 0.82258 | A | 0.17742 | 11 | 0.35484 |
| 14530 | S18_48794794 | 18 | 48794794 | 42 | A | G | A | 0.64516 | G | 0.35484 | 16 | 0.51613 |
| 14531 | S18_48794805 | 18 | 48794805 | 11 | G | A | G | 0.82258 | A | 0.17742 | 11 | 0.35484 |
| 14532 | S18_48881608 | 18 | 48881608 | 86803 | A | G | A | 0.93548 | G | 0.06452 | 4 | 0.12903 |
| 14533 | S18_48950106 | 18 | 48950106 | 68498 | C | T | C | 0.91935 | T | 0.08065 | 5 | 0.16129 |
| 14534 | S18_48950199 | 18 | 48950199 | 93 | T | G | T | 0.87097 | G | 0.12903 | 8 | 0.25806 |
| 14535 | S18_49619605 | 18 | 49619605 | 669406 | A | C | C | 0.85484 | A | 0.14516 | 9 | 0.29032 |
| 14536 | S18_49619614 | 18 | 49619614 | 9 | C | T | C | 0.90323 | T | 0.09677 | 6 | 0.19355 |
| 14537 | S18_49619660 | 18 | 49619660 | 46 | G | A | G | 0.74194 | A | 0.25806 | 16 | 0.51613 |
| 14538 | S18_49787294 | 18 | 49787294 | 167634 | T | A | A | 0.5 | T | 0.5 | 17 | 0.54839 |
| 14539 | S18_49856549 | 18 | 49856549 | 69255 | T | C | C | 0.66129 | T | 0.33871 | 13 | 0.41935 |
| 14540 | S18_49856728 | 18 | 49856728 | 179 | A | G | G | 0.66129 | A | 0.33871 | 13 | 0.41935 |
| 14541 | S18_49856739 | 18 | 49856739 | 11 | G | A | G | 0.91935 | A | 0.08065 | 5 | 0.16129 |
| 14542 | S18_49955609 | 18 | 49955609 | 98870 | C | A | C | 0.91935 | A | 0.08065 | 5 | 0.16129 |
| 14543 | S18_50098647 | 18 | 50098647 | 143038 | A | G | G | 0.91935 | A | 0.08065 | 5 | 0.16129 |
| 14544 | S18_50458829 | 18 | 50458829 | 360182 | G | A | A | 0.93548 | G | 0.06452 | 4 | 0.12903 |
| 14545 | S18_50458975 | 18 | 50458975 | 146 | C | T | C | 0.95161 | T | 0.04839 | 3 | 0.09677 |
| 14546 | S18_50622258 | 18 | 50622258 | 163283 | G | A | A | 0.69355 | G | 0.30645 | 15 | 0.48387 |
| 14547 | S18_50622362 | 18 | 50622362 | 104 | G | A | A | 0.74194 | G | 0.25806 | 12 | 0.3871 |
| 14548 | S18_50622363 | 18 | 50622363 | 1 | T | C | T | 0.8871 | C | 0.1129 | 7 | 0.22581 |
| 14549 | S18_50903426 | 18 | 50903426 | 281063 | G | C | G | 0.91935 | C | 0.08065 | 5 | 0.16129 |
| 14550 | S18_50981395 | 18 | 50981395 | 77969 | T | C | C | 0.85484 | T | 0.14516 | 7 | 0.22581 |
| 14551 | S18_50981584 | 18 | 50981584 | 189 | C | A | C | 0.91935 | A | 0.08065 | 5 | 0.16129 |
| 14552 | S18_51190494 | 18 | 51190494 | 208910 | G | A | G | 0.77419 | A | 0.22581 | 14 | 0.45161 |
| 14553 | S18_51260059 | 18 | 51260059 | 69565 | T | C | T | 0.80645 | C | 0.19355 | 10 | 0.32258 |
| 14554 | S18_51260088 | 18 | 51260088 | 29 | G | C | C | 0.82258 | G | 0.17742 | 11 | 0.35484 |
| 14555 | S18_51260097 | 18 | 51260097 | 9 | A | G | G | 0.82258 | A | 0.17742 | 11 | 0.35484 |
| 14556 | S18_51260145 | 18 | 51260145 | 48 | C | T | C | 0.79032 | T | 0.20968 | 11 | 0.35484 |
| 14557 | S18_51444391 | 18 | 51444391 | 184246 | C | T | C | 0.91935 | T | 0.08065 | 5 | 0.16129 |
| 14558 | S18_51534642 | 18 | 51534642 | 90251 | C | T | C | 0.90323 | T | 0.09677 | 6 | 0.19355 |
| 14559 | S18_51534673 | 18 | 51534673 | 31 | T | C | T | 0.56452 | C | 0.43548 | 15 | 0.48387 |
| 14560 | S18_51534709 | 18 | 51534709 | 36 | T | C | T | 0.93548 | C | 0.06452 | 4 | 0.12903 |
| 14561 | S18_51534784 | 18 | 51534784 | 75 | C | T | C | 0.93548 | T | 0.06452 | 4 | 0.12903 |
| 14562 | S18_51534846 | 18 | 51534846 | 62 | C | T | T | 0.56452 | C | 0.43548 | 17 | 0.54839 |
| 14563 | S18_51537414 | 18 | 51537414 | 2568 | G | A | G | 0.72581 | A | 0.27419 | 17 | 0.54839 |
| 14564 | S18_51537472 | 18 | 51537472 | 58 | T | C | T | 0.72581 | C | 0.27419 | 17 | 0.54839 |
| 14565 | S18_51552358 | 18 | 51552358 | 14886 | A | G | A | 0.53226 | G | 0.46774 | 21 | 0.67742 |
| 14566 | S18_51552389 | 18 | 51552389 | 31 | A | G | A | 0.83871 | G | 0.16129 | 10 | 0.32258 |
| 14567 | S18_51552500 | 18 | 51552500 | 111 | G | A | G | 0.53226 | A | 0.46774 | 21 | 0.67742 |
| 14568 | S18_51552600 | 18 | 51552600 | 100 | G | A | A | 0.8871 | G | 0.1129 | 7 | 0.22581 |
| 14569 | S18_51579301 | 18 | 51579301 | 26701 | A | G | A | 0.53226 | G | 0.46774 | 21 | 0.67742 |
| 14570 | S18_51579407 | 18 | 51579407 | 106 | T | C | T | 0.74194 | C | 0.25806 | 12 | 0.3871 |
| 14571 | S18_51579424 | 18 | 51579424 | 17 | A | T | A | 0.83871 | T | 0.16129 | 8 | 0.25806 |
| 14572 | S18_51583597 | 18 | 51583597 | 4173 | T | C | T | 0.87097 | C | 0.12903 | 8 | 0.25806 |
| 14573 | S18_51583629 | 18 | 51583629 | 32 | C | T | C | 0.8871 | T | 0.1129 | 7 | 0.22581 |
| 14574 | S18_51583641 | 18 | 51583641 | 12 | T | C | T | 0.85484 | C | 0.14516 | 9 | 0.29032 |
| 14575 | S18_51583678 | 18 | 51583678 | 37 | G | A | G | 0.87097 | A | 0.12903 | 8 | 0.25806 |
| 14576 | S18_51606907 | 18 | 51606907 | 23229 | C | T | C | 0.75806 | T | 0.24194 | 15 | 0.48387 |
| 14577 | S18_51607066 | 18 | 51607066 | 159 | C | T | C | 0.93548 | T | 0.06452 | 4 | 0.12903 |
| 14578 | S18_51651728 | 18 | 51651728 | 44662 | T | G | T | 0.95161 | G | 0.04839 | 3 | 0.09677 |

| Site number | SNP name | Chromosome | Physical position | Physical distance from the previous SNP | Reference allele | Alternative allele | Major allele | Major allele frequency | Minor allele | Minor allele frequency | Number heterozygous | Heterozygosity |
|---|---|---|---|---|---|---|---|---|---|---|---|---|
| 14579 | S18_51653505 | 18 | 51653505 | 1777 | T | C | T | 0.80645 | C | 0.19355 | 12 | 0.3871 |
| 14580 | S18_51653637 | 18 | 51653637 | 132 | C | T | C | 0.8871 | T | 0.1129 | 7 | 0.22581 |
| 14581 | S18_51660942 | 18 | 51660942 | 7305 | C | G | C | 0.83871 | G | 0.16129 | 10 | 0.32258 |
| 14582 | S18_51660948 | 18 | 51660948 | 6 | G | A | G | 0.90323 | A | 0.09677 | 6 | 0.19355 |
| 14583 | S18_51660977 | 18 | 51660977 | 29 | T | C | T | 0.93548 | C | 0.06452 | 4 | 0.12903 |
| 14584 | S18_51660995 | 18 | 51660995 | 18 | G | C | G | 0.59677 | C | 0.40323 | 21 | 0.67742 |
| 14585 | S18_52017246 | 18 | 52017246 | 356251 | A | C | C | 0.77419 | A | 0.22581 | 12 | 0.3871 |
| 14586 | S18_52301100 | 18 | 52301100 | 283854 | G | C | G | 0.87097 | C | 0.12903 | 8 | 0.25806 |
| 14587 | S18_52849290 | 18 | 52849290 | 548190 | C | T | C | 0.80645 | T | 0.19355 | 10 | 0.32258 |
| 14588 | S18_52868243 | 18 | 52868243 | 18953 | T | C | C | 0.54839 | T | 0.45161 | 16 | 0.51613 |
| 14589 | S18_52868459 | 18 | 52868459 | 216 | G | A | G | 0.8871 | A | 0.1129 | 5 | 0.16129 |
| 14590 | S18_52955057 | 18 | 52955057 | 86598 | A | G | A | 0.75806 | G | 0.24194 | 11 | 0.35484 |
| 14591 | S18_52955062 | 18 | 52955062 | 5 | C | T | C | 0.93548 | T | 0.06452 | 4 | 0.12903 |
| 14592 | S18_52955082 | 18 | 52955082 | 20 | A | G | A | 0.75806 | G | 0.24194 | 11 | 0.35484 |
| 14593 | S18_52955099 | 18 | 52955099 | 17 | T | C | C | 0.83871 | T | 0.16129 | 8 | 0.25806 |
| 14594 | S18_52987017 | 18 | 52987017 | 31918 | G | A | G | 0.83871 | A | 0.16129 | 8 | 0.25806 |
| 14595 | S18_52987108 | 18 | 52987108 | 91 | C | G | C | 0.91935 | G | 0.08065 | 5 | 0.16129 |
| 14596 | S18_52987128 | 18 | 52987128 | 20 | T | C | T | 0.70968 | C | 0.29032 | 14 | 0.45161 |
| 14597 | S18_53230635 | 18 | 53230635 | 243507 | T | G | T | 0.8871 | G | 0.1129 | 7 | 0.22581 |
| 14598 | S18_53394377 | 18 | 53394377 | 163742 | G | A | G | 0.95161 | A | 0.04839 | 3 | 0.09677 |
| 14599 | S18_53507146 | 18 | 53507146 | 112769 | A | T | A | 0.8871 | T | 0.1129 | 5 | 0.16129 |
| 14600 | S18_53507318 | 18 | 53507318 | 172 | A | G | G | 0.70968 | A | 0.29032 | 16 | 0.51613 |
| 14601 | S18_53632862 | 18 | 53632862 | 125544 | G | A | G | 0.95161 | A | 0.04839 | 3 | 0.09677 |
| 14602 | S18_53882236 | 18 | 53882236 | 249374 | T | G | T | 0.93548 | G | 0.06452 | 4 | 0.12903 |
| 14603 | S18_53898830 | 18 | 53898830 | 16594 | T | C | T | 0.72581 | C | 0.27419 | 11 | 0.35484 |
| 14604 | S18_53898833 | 18 | 53898833 | 3 | C | T | C | 0.74194 | T | 0.25806 | 10 | 0.32258 |
| 14605 | S18_53898875 | 18 | 53898875 | 42 | C | T | C | 0.82258 | T | 0.17742 | 9 | 0.29032 |
| 14606 | S18_53898933 | 18 | 53898933 | 58 | T | C | T | 0.72581 | C | 0.27419 | 11 | 0.35484 |
| 14607 | S18_53898980 | 18 | 53898980 | 47 | C | A | C | 0.79032 | A | 0.20968 | 11 | 0.35484 |
| 14608 | S18_53899017 | 18 | 53899017 | 37 | G | A | G | 0.79032 | A | 0.20968 | 11 | 0.35484 |
| 14609 | S18_53899029 | 18 | 53899029 | 12 | A | C | A | 0.62903 | C | 0.37097 | 15 | 0.48387 |
| 14610 | S18_53899067 | 18 | 53899067 | 38 | A | G | A | 0.90323 | G | 0.09677 | 6 | 0.19355 |
| 14611 | S18_53959101 | 18 | 53959101 | 60034 | A | C | A | 0.90323 | C | 0.09677 | 6 | 0.19355 |
| 14612 | S18_53959136 | 18 | 53959136 | 35 | G | T | G | 0.91935 | T | 0.08065 | 5 | 0.16129 |
| 14613 | S18_53959172 | 18 | 53959172 | 36 | G | C | G | 0.85484 | C | 0.14516 | 9 | 0.29032 |
| 14614 | S18_53959209 | 18 | 53959209 | 37 | C | G | C | 0.75806 | G | 0.24194 | 9 | 0.29032 |
| 14615 | S18_54095195 | 18 | 54095195 | 135986 | T | C | C | 0.80645 | T | 0.19355 | 10 | 0.32258 |
| 14616 | S18_54095210 | 18 | 54095210 | 15 | G | A | G | 0.54839 | A | 0.45161 | 18 | 0.58065 |
| 14617 | S18_54120129 | 18 | 54120129 | 24919 | T | C | T | 0.59677 | C | 0.40323 | 17 | 0.54839 |
| 14618 | S18_54138493 | 18 | 54138493 | 18364 | C | A | C | 0.93548 | A | 0.06452 | 4 | 0.12903 |
| 14619 | S18_54138494 | 18 | 54138494 | 1 | C | G | C | 0.82258 | G | 0.17742 | 7 | 0.22581 |
| 14620 | S18_54180144 | 18 | 54180144 | 41650 | G | A | G | 0.91935 | A | 0.08065 | 3 | 0.09677 |
| 14621 | S18_54305091 | 18 | 54305091 | 124947 | A | G | G | 0.79032 | A | 0.20968 | 11 | 0.35484 |
| 14622 | S18_54570349 | 18 | 54570349 | 265258 | G | A | G | 0.90323 | A | 0.09677 | 6 | 0.19355 |
| 14623 | S18_54688444 | 18 | 54688444 | 118095 | G | A | G | 0.69355 | A | 0.30645 | 11 | 0.35484 |
| 14624 | S18_54688520 | 18 | 54688520 | 76 | G | A | G | 0.77419 | A | 0.22581 | 10 | 0.32258 |
| 14625 | S18_54697753 | 18 | 54697753 | 9233 | A | G | A | 0.67742 | G | 0.32258 | 14 | 0.45161 |
| 14626 | S18_54701081 | 18 | 54701081 | 3328 | A | C | C | 0.56452 | A | 0.43548 | 15 | 0.48387 |
| 14627 | S18_54701105 | 18 | 54701105 | 24 | C | G | G | 0.75806 | C | 0.24194 | 13 | 0.41935 |
| 14628 | S18_54701182 | 18 | 54701182 | 77 | G | A | A | 0.75806 | G | 0.24194 | 13 | 0.41935 |
| 14629 | S18_54701234 | 18 | 54701234 | 52 | A | G | G | 0.75806 | A | 0.24194 | 13 | 0.41935 |
| 14630 | S18_54701259 | 18 | 54701259 | 25 | T | C | T | 0.80645 | C | 0.19355 | 8 | 0.25806 |
| 14631 | S18_55504282 | 18 | 55504282 | 803023 | T | C | T | 0.95161 | C | 0.04839 | 3 | 0.09677 |
| 14632 | S18_55619517 | 18 | 55619517 | 115235 | C | T | C | 0.87097 | T | 0.12903 | 6 | 0.19355 |

| Site number | SNP name | Chromosome | Physical position | Physical distance from the previous SNP | Reference allele | Alternative allele | Major allele | Major allele frequency | Minor allele | Minor allele frequency | Number heterozygous | Heterozygosity |
|---|---|---|---|---|---|---|---|---|---|---|---|---|
| 14633 | S18_55806478 | 18 | 55806478 | 186961 | G | T | T | 0.72581 | G | 0.27419 | 11 | 0.35484 |
| 14634 | S18_55806489 | 18 | 55806489 | 11 | G | A | G | 0.8871 | A | 0.1129 | 7 | 0.22581 |
| 14635 | S18_55806526 | 18 | 55806526 | 37 | A | G | G | 0.72581 | A | 0.27419 | 11 | 0.35484 |
| 14636 | S18_55806610 | 18 | 55806610 | 84 | A | G | A | 0.66129 | G | 0.33871 | 13 | 0.41935 |
| 14637 | S18_55806628 | 18 | 55806628 | 18 | C | T | T | 0.72581 | C | 0.27419 | 11 | 0.35484 |
| 14638 | S18_55814348 | 18 | 55814348 | 7720 | G | T | G | 0.75806 | T | 0.24194 | 13 | 0.41935 |
| 14639 | S18_55814414 | 18 | 55814414 | 66 | C | T | T | 0.72581 | C | 0.27419 | 11 | 0.35484 |
| 14640 | S18_55876940 | 18 | 55876940 | 62526 | T | C | T | 0.66129 | C | 0.33871 | 11 | 0.35484 |
| 14641 | S18_55876943 | 18 | 55876943 | 3 | A | G | A | 0.66129 | G | 0.33871 | 11 | 0.35484 |
| 14642 | S18_55888088 | 18 | 55888088 | 11145 | A | G | A | 0.90323 | G | 0.09677 | 6 | 0.19355 |
| 14643 | S18_55985266 | 18 | 55985266 | 97178 | A | G | A | 0.87097 | G | 0.12903 | 4 | 0.12903 |
| 14644 | S18_55985348 | 18 | 55985348 | 82 | G | A | G | 0.67742 | A | 0.32258 | 14 | 0.45161 |
| 14645 | S18_55985504 | 18 | 55985504 | 156 | C | T | C | 0.82258 | T | 0.17742 | 11 | 0.35484 |
| 14646 | S18_56411235 | 18 | 56411235 | 425731 | C | T | C | 0.95161 | T | 0.04839 | 3 | 0.09677 |
| 14647 | S18_57267485 | 18 | 57267485 | 856250 | C | T | C | 0.74194 | T | 0.25806 | 14 | 0.45161 |
| 14648 | S18_57396452 | 18 | 57396452 | 128967 | G | C | G | 0.8871 | C | 0.1129 | 7 | 0.22581 |
| 14649 | S18_57684994 | 18 | 57684994 | 288542 | C | G | C | 0.8871 | G | 0.1129 | 7 | 0.22581 |
| 14650 | S18_57684995 | 18 | 57684995 | 1 | C | G | C | 0.67742 | G | 0.32258 | 12 | 0.3871 |
| 14651 | S18_57685044 | 18 | 57685044 | 49 | G | C | G | 0.82258 | C | 0.17742 | 11 | 0.35484 |
| 14652 | S18_57763906 | 18 | 57763906 | 78862 | G | A | G | 0.95161 | A | 0.04839 | 3 | 0.09677 |
| 14653 | S18_58130768 | 18 | 58130768 | 366862 | G | A | G | 0.91935 | A | 0.08065 | 5 | 0.16129 |
| 14654 | S18_58290647 | 18 | 58290647 | 159879 | T | A | T | 0.95161 | A | 0.04839 | 3 | 0.09677 |
| 14655 | S18_58290727 | 18 | 58290727 | 80 | C | T | C | 0.93548 | T | 0.06452 | 4 | 0.12903 |
| 14656 | S18_58290851 | 18 | 58290851 | 124 | C | T | T | 0.56452 | C | 0.43548 | 21 | 0.67742 |
| 14657 | S18_58568967 | 18 | 58568967 | 278116 | G | A | G | 0.95161 | A | 0.04839 | 3 | 0.09677 |
| 14658 | S18_58569011 | 18 | 58569011 | 44 | C | A | C | 0.95161 | A | 0.04839 | 3 | 0.09677 |
| 14659 | S18_58569061 | 18 | 58569061 | 50 | G | T | G | 0.66129 | T | 0.33871 | 13 | 0.41935 |
| 14660 | S18_59439087 | 18 | 59439087 | 870026 | G | A | G | 0.90323 | A | 0.09677 | 6 | 0.19355 |
| 14661 | S18_59499135 | 18 | 59499135 | 60048 | C | T | C | 0.95161 | T | 0.04839 | 3 | 0.09677 |
| 14662 | S18_59693943 | 18 | 59693943 | 194808 | A | G | A | 0.51613 | G | 0.48387 | 10 | 0.32258 |
| 14663 | S18_59693966 | 18 | 59693966 | 23 | G | A | G | 0.53226 | A | 0.46774 | 13 | 0.41935 |
| 14664 | S18_59693983 | 18 | 59693983 | 17 | A | C | A | 0.66129 | C | 0.33871 | 11 | 0.35484 |
| 14665 | S18_59694165 | 18 | 59694165 | 182 | T | C | T | 0.85484 | C | 0.14516 | 7 | 0.22581 |
| 14666 | S18_59694172 | 18 | 59694172 | 7 | G | C | G | 0.91935 | C | 0.08065 | 5 | 0.16129 |
| 14667 | S18_59759239 | 18 | 59759239 | 65067 | C | T | C | 0.91935 | T | 0.08065 | 5 | 0.16129 |
| 14668 | S18_59848729 | 18 | 59848729 | 89490 | T | A | T | 0.75806 | A | 0.24194 | 15 | 0.48387 |
| 14669 | S18_59852295 | 18 | 59852295 | 3566 | A | G | A | 0.93548 | G | 0.06452 | 4 | 0.12903 |
| 14670 | S18_59852428 | 18 | 59852428 | 133 | T | G | T | 0.79032 | G | 0.20968 | 13 | 0.41935 |
| 14671 | S18_59852440 | 18 | 59852440 | 12 | G | A | G | 0.95161 | A | 0.04839 | 3 | 0.09677 |
| 14672 | S18_59852543 | 18 | 59852543 | 103 | T | C | C | 0.93548 | T | 0.06452 | 4 | 0.12903 |
| 14673 | S18_60020398 | 18 | 60020398 | 167855 | G | A | G | 0.77419 | A | 0.22581 | 14 | 0.45161 |
| 14674 | S18_60020403 | 18 | 60020403 | 5 | G | A | G | 0.77419 | A | 0.22581 | 14 | 0.45161 |
| 14675 | S18_60020423 | 18 | 60020423 | 20 | A | T | A | 0.79032 | T | 0.20968 | 13 | 0.41935 |
| 14676 | S18_60020427 | 18 | 60020427 | 4 | T | G | G | 0.85484 | T | 0.14516 | 9 | 0.29032 |
| 14677 | S18_60020488 | 18 | 60020488 | 61 | T | C | T | 0.80645 | C | 0.19355 | 12 | 0.3871 |
| 14678 | S18_60020496 | 18 | 60020496 | 8 | G | C | G | 0.74194 | C | 0.25806 | 16 | 0.51613 |
| 14679 | S18_60149633 | 18 | 60149633 | 129137 | A | G | A | 0.93548 | G | 0.06452 | 4 | 0.12903 |
| 14680 | S18_60149814 | 18 | 60149814 | 181 | G | T | G | 0.90323 | T | 0.09677 | 6 | 0.19355 |
| 14681 | S18_60157268 | 18 | 60157268 | 7454 | A | G | A | 0.64516 | G | 0.35484 | 18 | 0.58065 |
| 14682 | S18_60157303 | 18 | 60157303 | 35 | T | C | T | 0.6129 | C | 0.3871 | 16 | 0.51613 |
| 14683 | S18_60198082 | 18 | 60198082 | 40779 | A | C | A | 0.66129 | C | 0.33871 | 15 | 0.48387 |
| 14684 | S18_60268020 | 18 | 60268020 | 69938 | T | C | T | 0.72581 | C | 0.27419 | 13 | 0.41935 |
| 14685 | S18_60268114 | 18 | 60268114 | 94 | C | A | C | 0.56452 | A | 0.43548 | 17 | 0.54839 |
| 14686 | S18_60434259 | 18 | 60434259 | 166145 | A | T | A | 0.93548 | T | 0.06452 | 2 | 0.06452 |

| Site number | SNP name | Chromosome | Physical position | Physical distance from the previous SNP | Reference allele | Alternative allele | Major allele | Major allele frequency | Minor allele | Minor allele frequency | Number heterozygous | Heterozygosity |
|---|---|---|---|---|---|---|---|---|---|---|---|---|
| 14687 | S18_60434455 | 18 | 60434455 | 196 | A | G | A | 0.93548 | G | 0.06452 | 2 | 0.06452 |
| 14688 | S18_60505260 | 18 | 60505260 | 70805 | A | G | A | 0.87097 | G | 0.12903 | 8 | 0.25806 |
| 14689 | S18_60505293 | 18 | 60505293 | 33 | A | T | A | 0.87097 | T | 0.12903 | 8 | 0.25806 |
| 14690 | S18_60505441 | 18 | 60505441 | 148 | G | C | C | 0.64516 | G | 0.35484 | 20 | 0.64516 |
| 14691 | S18_60540695 | 18 | 60540695 | 35254 | G | A | G | 0.91935 | A | 0.08065 | 3 | 0.09677 |
| 14692 | S18_60583615 | 18 | 60583615 | 42920 | C | A | C | 0.91935 | A | 0.08065 | 5 | 0.16129 |
| 14693 | S18_60583660 | 18 | 60583660 | 45 | A | C | A | 0.91935 | C | 0.08065 | 5 | 0.16129 |
| 14694 | S18_60583756 | 18 | 60583756 | 96 | C | T | C | 0.8871 | T | 0.1129 | 7 | 0.22581 |
| 14695 | S18_60583757 | 18 | 60583757 | 1 | G | A | G | 0.80645 | A | 0.19355 | 12 | 0.3871 |
| 14696 | S18_60583791 | 18 | 60583791 | 34 | G | A | G | 0.95161 | A | 0.04839 | 3 | 0.09677 |
| 14697 | S18_60600193 | 18 | 60600193 | 16402 | C | T | C | 0.79032 | T | 0.20968 | 7 | 0.22581 |
| 14698 | S18_60702789 | 18 | 60702789 | 102596 | C | T | C | 0.8871 | T | 0.1129 | 7 | 0.22581 |
| 14699 | S18_60702808 | 18 | 60702808 | 19 | C | G | C | 0.91935 | G | 0.08065 | 5 | 0.16129 |
| 14700 | S18_60769494 | 18 | 60769494 | 66686 | G | C | G | 0.93548 | C | 0.06452 | 4 | 0.12903 |
| 14701 | S18_60769499 | 18 | 60769499 | 5 | C | T | C | 0.75806 | T | 0.24194 | 15 | 0.48387 |
| 14702 | S18_60769515 | 18 | 60769515 | 16 | C | T | C | 0.75806 | T | 0.24194 | 15 | 0.48387 |
| 14703 | S18_60769642 | 18 | 60769642 | 127 | A | G | A | 0.75806 | G | 0.24194 | 15 | 0.48387 |
| 14704 | S18_60789468 | 18 | 60789468 | 19826 | T | C | T | 0.95161 | C | 0.04839 | 3 | 0.09677 |
| 14705 | S18_60789481 | 18 | 60789481 | 13 | C | T | C | 0.75806 | T | 0.24194 | 15 | 0.48387 |
| 14706 | S18_60789623 | 18 | 60789623 | 142 | G | T | G | 0.93548 | T | 0.06452 | 4 | 0.12903 |
| 14707 | S18_60816424 | 18 | 60816424 | 26801 | G | A | G | 0.8871 | A | 0.1129 | 5 | 0.16129 |
| 14708 | S18_60816429 | 18 | 60816429 | 5 | A | G | G | 0.64516 | A | 0.35484 | 14 | 0.45161 |
| 14709 | S18_60816535 | 18 | 60816535 | 106 | A | C | A | 0.8871 | C | 0.1129 | 5 | 0.16129 |
| 14710 | S18_60816549 | 18 | 60816549 | 14 | C | T | C | 0.87097 | T | 0.12903 | 6 | 0.19355 |
| 14711 | S18_60995747 | 18 | 60995747 | 179198 | T | G | T | 0.8871 | G | 0.1129 | 7 | 0.22581 |
| 14712 | S18_60995777 | 18 | 60995777 | 30 | G | A | G | 0.72581 | A | 0.27419 | 17 | 0.54839 |
| 14713 | S18_61100573 | 18 | 61100573 | 104796 | C | T | C | 0.90323 | T | 0.09677 | 6 | 0.19355 |
| 14714 | S18_61100612 | 18 | 61100612 | 39 | A | T | A | 0.90323 | T | 0.09677 | 6 | 0.19355 |
| 14715 | S18_61459891 | 18 | 61459891 | 359279 | A | T | A | 0.93548 | T | 0.06452 | 4 | 0.12903 |
| 14716 | S18_61808818 | 18 | 61808818 | 348927 | A | G | A | 0.77419 | G | 0.22581 | 12 | 0.3871 |
| 14717 | S18_61808994 | 18 | 61808994 | 176 | C | A | C | 0.54839 | A | 0.45161 | 16 | 0.51613 |
| 14718 | S18_61879568 | 18 | 61879568 | 70574 | A | G | G | 0.8871 | A | 0.1129 | 7 | 0.22581 |
| 14719 | S18_61879573 | 18 | 61879573 | 5 | A | T | A | 0.87097 | T | 0.12903 | 6 | 0.19355 |
| 14720 | S18_62282409 | 18 | 62282409 | 402836 | G | A | G | 0.87097 | A | 0.12903 | 8 | 0.25806 |
| 14721 | S18_62282415 | 18 | 62282415 | 6 | T | G | G | 0.74194 | T | 0.25806 | 16 | 0.51613 |
| 14722 | S18_62282438 | 18 | 62282438 | 23 | T | G | G | 0.72581 | T | 0.27419 | 17 | 0.54839 |
| 14723 | S18_62370141 | 18 | 62370141 | 87703 | C | T | C | 0.91935 | T | 0.08065 | 5 | 0.16129 |
| 14724 | S18_62370183 | 18 | 62370183 | 42 | A | G | G | 0.59677 | A | 0.40323 | 15 | 0.48387 |
| 14725 | S18_62370232 | 18 | 62370232 | 49 | G | C | G | 0.93548 | C | 0.06452 | 4 | 0.12903 |
| 14726 | S18_62391582 | 18 | 62391582 | 21350 | G | A | G | 0.82258 | A | 0.17742 | 9 | 0.29032 |
| 14727 | S18_62391597 | 18 | 62391597 | 15 | T | C | T | 0.77419 | C | 0.22581 | 14 | 0.45161 |
| 14728 | S18_62391654 | 18 | 62391654 | 57 | A | C | A | 0.77419 | C | 0.22581 | 14 | 0.45161 |
| 14729 | S18_62391684 | 18 | 62391684 | 30 | A | G | G | 0.95161 | A | 0.04839 | 3 | 0.09677 |
| 14730 | S18_62769452 | 18 | 62769452 | 377768 | G | A | G | 0.95161 | A | 0.04839 | 3 | 0.09677 |
| 14731 | S18_62769572 | 18 | 62769572 | 120 | C | T | T | 0.93548 | C | 0.06452 | 4 | 0.12903 |
| 14732 | S18_62769588 | 18 | 62769588 | 16 | T | C | T | 0.51613 | C | 0.48387 | 16 | 0.51613 |
| 14733 | S18_62786262 | 18 | 62786262 | 16674 | A | G | A | 0.8871 | G | 0.1129 | 7 | 0.22581 |
| 14734 | S18_62786278 | 18 | 62786278 | 16 | A | G | G | 0.82258 | A | 0.17742 | 11 | 0.35484 |
| 14735 | S18_62990896 | 18 | 62990896 | 204618 | G | C | C | 0.54839 | G | 0.45161 | 16 | 0.51613 |
| 14736 | S18_62990983 | 18 | 62990983 | 87 | T | C | T | 0.58065 | C | 0.41935 | 16 | 0.51613 |
| 14737 | S18_62991035 | 18 | 62991035 | 52 | T | C | T | 0.93548 | C | 0.06452 | 4 | 0.12903 |
| 14738 | S18_62991042 | 18 | 62991042 | 7 | A | G | A | 0.59677 | G | 0.40323 | 17 | 0.54839 |
| 14739 | S18_63006165 | 18 | 63006165 | 15123 | A | G | G | 0.75806 | A | 0.24194 | 13 | 0.41935 |
| 14740 | S18_63006344 | 18 | 63006344 | 179 | G | A | G | 0.74194 | A | 0.25806 | 10 | 0.32258 |

| Site number | SNP name | Chromosome | Physical position | Physical distance from the previous SNP | Reference allele | Alternative allele | Major allele | Major allele frequency | Minor allele | Minor allele frequency | Number heterozygous | Heterozygosity |
|---|---|---|---|---|---|---|---|---|---|---|---|---|
| 14741 | S18_63041622 | 18 | 63041622 | 35278 | A | G | G | 0.75806 | A | 0.24194 | 9 | 0.29032 |
| 14742 | S18_63041644 | 18 | 63041644 | 22 | T | G | T | 0.58065 | G | 0.41935 | 12 | 0.3871 |
| 14743 | S18_63041809 | 18 | 63041809 | 165 | G | A | A | 0.83871 | G | 0.16129 | 10 | 0.32258 |
| 14744 | S18_63351021 | 18 | 63351021 | 309212 | G | A | G | 0.87097 | A | 0.12903 | 6 | 0.19355 |
| 14745 | S18_63351022 | 18 | 63351022 | 1 | A | G | A | 0.87097 | G | 0.12903 | 6 | 0.19355 |
| 14746 | S18_63351220 | 18 | 63351220 | 198 | A | C | A | 0.87097 | C | 0.12903 | 6 | 0.19355 |
| 14747 | S18_63748117 | 18 | 63748117 | 396897 | A | T | A | 0.79032 | T | 0.20968 | 9 | 0.29032 |
| 14748 | S18_64361495 | 18 | 64361495 | 613378 | G | T | G | 0.74194 | T | 0.25806 | 16 | 0.51613 |
| 14749 | S18_64361521 | 18 | 64361521 | 26 | C | T | C | 0.75806 | T | 0.24194 | 15 | 0.48387 |
| 14750 | S18_64361525 | 18 | 64361525 | 4 | T | A | T | 0.72581 | A | 0.27419 | 17 | 0.54839 |
| 14751 | S18_64361571 | 18 | 64361571 | 46 | G | C | G | 0.82258 | C | 0.17742 | 11 | 0.35484 |
| 14752 | S18_64361584 | 18 | 64361584 | 13 | C | G | C | 0.80645 | G | 0.19355 | 12 | 0.3871 |
| 14753 | S18_64361705 | 18 | 64361705 | 121 | C | T | C | 0.74194 | T | 0.25806 | 16 | 0.51613 |
| 14754 | S18_64361706 | 18 | 64361706 | 1 | T | C | T | 0.74194 | C | 0.25806 | 16 | 0.51613 |
| 14755 | S18_64361725 | 18 | 64361725 | 19 | T | C | T | 0.75806 | C | 0.24194 | 15 | 0.48387 |
| 14756 | S18_64374741 | 18 | 64374741 | 13016 | T | G | T | 0.85484 | G | 0.14516 | 9 | 0.29032 |
| 14757 | S18_64374747 | 18 | 64374747 | 6 | T | C | T | 0.82258 | C | 0.17742 | 11 | 0.35484 |
| 14758 | S18_64374751 | 18 | 64374751 | 4 | T | G | T | 0.82258 | G | 0.17742 | 11 | 0.35484 |
| 14759 | S18_64374757 | 18 | 64374757 | 6 | C | T | C | 0.83871 | T | 0.16129 | 10 | 0.32258 |
| 14760 | S18_64374767 | 18 | 64374767 | 10 | C | T | C | 0.83871 | T | 0.16129 | 10 | 0.32258 |
| 14761 | S18_64374779 | 18 | 64374779 | 12 | C | T | T | 0.72581 | C | 0.27419 | 17 | 0.54839 |
| 14762 | S18_64374782 | 18 | 64374782 | 3 | C | G | C | 0.85484 | G | 0.14516 | 9 | 0.29032 |
| 14763 | S18_64374797 | 18 | 64374797 | 15 | A | G | A | 0.85484 | G | 0.14516 | 9 | 0.29032 |
| 14764 | S18_64374809 | 18 | 64374809 | 12 | G | C | G | 0.90323 | C | 0.09677 | 6 | 0.19355 |
| 14765 | S18_64374832 | 18 | 64374832 | 23 | C | T | C | 0.75806 | T | 0.24194 | 15 | 0.48387 |
| 14766 | S18_64374842 | 18 | 64374842 | 10 | C | G | C | 0.83871 | G | 0.16129 | 10 | 0.32258 |
| 14767 | S18_64374843 | 18 | 64374843 | 1 | C | T | C | 0.72581 | T | 0.27419 | 17 | 0.54839 |
| 14768 | S18_64374854 | 18 | 64374854 | 11 | T | G | T | 0.77419 | G | 0.22581 | 14 | 0.45161 |
| 14769 | S18_64374857 | 18 | 64374857 | 3 | G | C | G | 0.91935 | C | 0.08065 | 5 | 0.16129 |
| 14770 | S18_64374859 | 18 | 64374859 | 2 | G | A | G | 0.82258 | A | 0.17742 | 11 | 0.35484 |
| 14771 | S18_64374860 | 18 | 64374860 | 1 | C | T | C | 0.75806 | T | 0.24194 | 15 | 0.48387 |
| 14772 | S18_64374861 | 18 | 64374861 | 1 | G | A | G | 0.83871 | A | 0.16129 | 10 | 0.32258 |
| 14773 | S18_64374869 | 18 | 64374869 | 8 | G | T | G | 0.93548 | T | 0.06452 | 4 | 0.12903 |
| 14774 | S18_64374870 | 18 | 64374870 | 1 | G | A | G | 0.91935 | A | 0.08065 | 5 | 0.16129 |
| 14775 | S18_64374873 | 18 | 64374873 | 3 | G | T | G | 0.90323 | T | 0.09677 | 6 | 0.19355 |
| 14776 | S18_64374878 | 18 | 64374878 | 5 | G | A | G | 0.79032 | A | 0.20968 | 13 | 0.41935 |
| 14777 | S18_64374880 | 18 | 64374880 | 2 | C | T | C | 0.82258 | T | 0.17742 | 11 | 0.35484 |
| 14778 | S18_64374892 | 18 | 64374892 | 12 | G | A | G | 0.83871 | A | 0.16129 | 10 | 0.32258 |
| 14779 | S18_64374895 | 18 | 64374895 | 3 | G | C | G | 0.83871 | C | 0.16129 | 10 | 0.32258 |
| 14780 | S18_64374901 | 18 | 64374901 | 6 | G | T | G | 0.82258 | T | 0.17742 | 11 | 0.35484 |
| 14781 | S18_64374905 | 18 | 64374905 | 4 | C | G | C | 0.82258 | G | 0.17742 | 11 | 0.35484 |
| 14782 | S18_64374930 | 18 | 64374930 | 25 | A | G | A | 0.91935 | G | 0.08065 | 5 | 0.16129 |
| 14783 | S18_64374935 | 18 | 64374935 | 5 | C | T | C | 0.83871 | T | 0.16129 | 10 | 0.32258 |
| 14784 | S18_64374945 | 18 | 64374945 | 10 | T | C | T | 0.82258 | C | 0.17742 | 11 | 0.35484 |
| 14785 | S18_64374952 | 18 | 64374952 | 7 | G | A | G | 0.79032 | A | 0.20968 | 13 | 0.41935 |
| 14786 | S18_64374957 | 18 | 64374957 | 5 | C | A | C | 0.79032 | A | 0.20968 | 13 | 0.41935 |
| 14787 | S18_65055993 | 18 | 65055993 | 681036 | G | A | G | 0.87097 | A | 0.12903 | 8 | 0.25806 |
| 14788 | S18_65128372 | 18 | 65128372 | 72379 | C | T | C | 0.56452 | T | 0.43548 | 19 | 0.6129 |
| 14789 | S18_65215250 | 18 | 65215250 | 86878 | A | T | A | 0.75806 | T | 0.24194 | 13 | 0.41935 |
| 14790 | S18_65215279 | 18 | 65215279 | 29 | C | T | T | 0.64516 | C | 0.35484 | 18 | 0.58065 |
| 14791 | S18_65215289 | 18 | 65215289 | 10 | G | T | T | 0.64516 | G | 0.35484 | 18 | 0.58065 |
| 14792 | S18_65215396 | 18 | 65215396 | 107 | A | G | G | 0.8871 | A | 0.1129 | 7 | 0.22581 |
| 14793 | S18_65215462 | 18 | 65215462 | 66 | G | A | G | 0.75806 | A | 0.24194 | 13 | 0.41935 |
| 14794 | S18_65215482 | 18 | 65215482 | 20 | C | T | C | 0.75806 | T | 0.24194 | 13 | 0.41935 |

| Site number | SNP name | Chromosome | Physical position | Physical distance from the previous SNP | Reference allele | Alternative allele | Major allele | Major allele frequency | Minor allele | Minor allele frequency | Number heterozygous | Heterozygosity |
|---|---|---|---|---|---|---|---|---|---|---|---|---|
| 14795 | S18_65294352 | 18 | 65294352 | 78870 | C | T | C | 0.82258 | T | 0.17742 | 11 | 0.35484 |
| 14796 | S18_65725406 | 18 | 65725406 | 431054 | C | T | C | 0.8871 | T | 0.1129 | 5 | 0.16129 |
| 14797 | S18_65725420 | 18 | 65725420 | 14 | C | A | C | 0.83871 | A | 0.16129 | 10 | 0.32258 |
| 14798 | S18_66163010 | 18 | 66163010 | 437590 | A | G | A | 0.91935 | G | 0.08065 | 5 | 0.16129 |
| 14799 | S18_66163095 | 18 | 66163095 | 85 | C | T | C | 0.93548 | T | 0.06452 | 4 | 0.12903 |
| 14800 | S18_66163100 | 18 | 66163100 | 5 | G | A | G | 0.91935 | A | 0.08065 | 3 | 0.09677 |
| 14801 | S18_66206351 | 18 | 66206351 | 43251 | A | C | A | 0.87097 | C | 0.12903 | 6 | 0.19355 |
| 14802 | S18_66206464 | 18 | 66206464 | 113 | C | T | C | 0.93548 | T | 0.06452 | 4 | 0.12903 |
| 14803 | S18_66206588 | 18 | 66206588 | 124 | C | T | T | 0.66129 | C | 0.33871 | 15 | 0.48387 |
| 14804 | S18_66967195 | 18 | 66967195 | 760607 | C | A | C | 0.53226 | A | 0.46774 | 17 | 0.54839 |
| 14805 | S18_66967387 | 18 | 66967387 | 192 | T | G | T | 0.95161 | G | 0.04839 | 3 | 0.09677 |
| 14806 | S19_319176 | 19 | 319176 | 0 | A | G | A | 0.93548 | G | 0.06452 | 4 | 0.12903 |
| 14807 | S19_539701 | 19 | 539701 | 220525 | C | T | C | 0.75806 | T | 0.24194 | 11 | 0.35484 |
| 14808 | S19_2544644 | 19 | 2544644 | 2004943 | A | G | A | 0.93548 | G | 0.06452 | 4 | 0.12903 |
| 14809 | S19_2544795 | 19 | 2544795 | 151 | G | A | G | 0.93548 | A | 0.06452 | 4 | 0.12903 |
| 14810 | S19_2544850 | 19 | 2544850 | 55 | C | A | C | 0.93548 | A | 0.06452 | 4 | 0.12903 |
| 14811 | S19_3289355 | 19 | 3289355 | 744505 | G | A | G | 0.93548 | A | 0.06452 | 4 | 0.12903 |
| 14812 | S19_3289365 | 19 | 3289365 | 10 | T | C | T | 0.75806 | C | 0.24194 | 13 | 0.41935 |
| 14813 | S19_3289418 | 19 | 3289418 | 53 | T | C | C | 0.95161 | T | 0.04839 | 3 | 0.09677 |
| 14814 | S19_3289509 | 19 | 3289509 | 91 | T | C | T | 0.72581 | C | 0.27419 | 11 | 0.35484 |
| 14815 | S19_4261887 | 19 | 4261887 | 972378 | T | A | T | 0.87097 | A | 0.12903 | 8 | 0.25806 |
| 14816 | S19_4261925 | 19 | 4261925 | 38 | G | A | G | 0.85484 | A | 0.14516 | 7 | 0.22581 |
| 14817 | S19_4301471 | 19 | 4301471 | 39546 | C | T | C | 0.91935 | T | 0.08065 | 5 | 0.16129 |
| 14818 | S19_4301541 | 19 | 4301541 | 70 | G | A | A | 0.77419 | G | 0.22581 | 14 | 0.45161 |
| 14819 | S19_4301610 | 19 | 4301610 | 69 | C | T | T | 0.77419 | C | 0.22581 | 14 | 0.45161 |
| 14820 | S19_4422601 | 19 | 4422601 | 120991 | C | T | C | 0.95161 | T | 0.04839 | 3 | 0.09677 |
| 14821 | S19_4455181 | 19 | 4455181 | 32580 | G | A | G | 0.90323 | A | 0.09677 | 4 | 0.12903 |
| 14822 | S19_4455184 | 19 | 4455184 | 3 | C | G | C | 0.90323 | G | 0.09677 | 4 | 0.12903 |
| 14823 | S19_4455235 | 19 | 4455235 | 51 | T | C | T | 0.90323 | C | 0.09677 | 4 | 0.12903 |
| 14824 | S19_4455260 | 19 | 4455260 | 25 | C | T | C | 0.8871 | T | 0.1129 | 7 | 0.22581 |
| 14825 | S19_4455336 | 19 | 4455336 | 76 | A | T | A | 0.93548 | T | 0.06452 | 4 | 0.12903 |
| 14826 | S19_4455372 | 19 | 4455372 | 36 | C | T | C | 0.90323 | T | 0.09677 | 6 | 0.19355 |
| 14827 | S19_4455385 | 19 | 4455385 | 13 | C | T | C | 0.83871 | T | 0.16129 | 8 | 0.25806 |
| 14828 | S19_4658240 | 19 | 4658240 | 202855 | G | A | G | 0.95161 | A | 0.04839 | 1 | 0.03226 |
| 14829 | S19_4658313 | 19 | 4658313 | 73 | A | G | G | 0.59677 | A | 0.40323 | 11 | 0.35484 |
| 14830 | S19_4882469 | 19 | 4882469 | 224156 | A | C | A | 0.95161 | C | 0.04839 | 3 | 0.09677 |
| 14831 | S19_4922625 | 19 | 4922625 | 40156 | T | C | C | 0.95161 | T | 0.04839 | 3 | 0.09677 |
| 14832 | S19_5035345 | 19 | 5035345 | 112720 | G | T | G | 0.91935 | T | 0.08065 | 5 | 0.16129 |
| 14833 | S19_5176798 | 19 | 5176798 | 141453 | C | T | C | 0.90323 | T | 0.09677 | 6 | 0.19355 |
| 14834 | S19_5176932 | 19 | 5176932 | 134 | G | A | G | 0.91935 | A | 0.08065 | 5 | 0.16129 |
| 14835 | S19_5341512 | 19 | 5341512 | 164580 | C | T | C | 0.87097 | T | 0.12903 | 8 | 0.25806 |
| 14836 | S19_5341631 | 19 | 5341631 | 119 | G | T | G | 0.8871 | T | 0.1129 | 5 | 0.16129 |
| 14837 | S19_5341692 | 19 | 5341692 | 61 | A | G | A | 0.8871 | G | 0.1129 | 5 | 0.16129 |
| 14838 | S19_5987555 | 19 | 5987555 | 645863 | T | C | T | 0.56452 | C | 0.43548 | 13 | 0.41935 |
| 14839 | S19_5987576 | 19 | 5987576 | 21 | T | C | T | 0.8871 | C | 0.1129 | 7 | 0.22581 |
| 14840 | S19_5987579 | 19 | 5987579 | 3 | G | A | A | 0.87097 | G | 0.12903 | 8 | 0.25806 |
| 14841 | S19_6091121 | 19 | 6091121 | 103542 | C | T | C | 0.90323 | T | 0.09677 | 6 | 0.19355 |
| 14842 | S19_6187001 | 19 | 6187001 | 95880 | T | C | C | 0.66129 | T | 0.33871 | 13 | 0.41935 |
| 14843 | S19_6187021 | 19 | 6187021 | 20 | C | G | C | 0.87097 | G | 0.12903 | 8 | 0.25806 |
| 14844 | S19_6187100 | 19 | 6187100 | 79 | G | A | G | 0.53226 | A | 0.46774 | 15 | 0.48387 |
| 14845 | S19_6351976 | 19 | 6351976 | 164876 | G | A | G | 0.75806 | A | 0.24194 | 11 | 0.35484 |
| 14846 | S19_6352022 | 19 | 6352022 | 46 | C | T | C | 0.59677 | T | 0.40323 | 17 | 0.54839 |
| 14847 | S19_6352023 | 19 | 6352023 | 1 | A | G | A | 0.59677 | G | 0.40323 | 17 | 0.54839 |
| 14848 | S19_6352101 | 19 | 6352101 | 78 | T | C | T | 0.80645 | C | 0.19355 | 10 | 0.32258 |

| Site number | SNP name | Chromosome | Physical position | Physical distance from the previous SNP | Reference allele | Alternative allele | Major allele | Major allele frequency | Minor allele | Minor allele frequency | Number heterozygous | Heterozygosity |
|---|---|---|---|---|---|---|---|---|---|---|---|---|
| 14849 | S19_6352119 | 19 | 6352119 | 18 | G | A | A | 0.74194 | G | 0.25806 | 12 | 0.3871 |
| 14850 | S19_6352145 | 19 | 6352145 | 26 | G | A | G | 0.90323 | A | 0.09677 | 6 | 0.19355 |
| 14851 | S19_6419475 | 19 | 6419475 | 67330 | A | G | G | 0.72581 | A | 0.27419 | 15 | 0.48387 |
| 14852 | S19_6421521 | 19 | 6421521 | 2046 | A | G | A | 0.8871 | G | 0.1129 | 5 | 0.16129 |
| 14853 | S19_6489513 | 19 | 6489513 | 67992 | A | G | A | 0.80645 | G | 0.19355 | 10 | 0.32258 |
| 14854 | S19_6489648 | 19 | 6489648 | 135 | G | A | G | 0.80645 | A | 0.19355 | 12 | 0.3871 |
| 14855 | S19_6604024 | 19 | 6604024 | 114376 | C | T | C | 0.95161 | T | 0.04839 | 3 | 0.09677 |
| 14856 | S19_6604116 | 19 | 6604116 | 92 | C | T | C | 0.93548 | T | 0.06452 | 4 | 0.12903 |
| 14857 | S19_6604254 | 19 | 6604254 | 138 | G | A | G | 0.6129 | A | 0.3871 | 18 | 0.58065 |
| 14858 | S19_6730564 | 19 | 6730564 | 126310 | C | T | T | 0.69355 | C | 0.30645 | 11 | 0.35484 |
| 14859 | S19_6961677 | 19 | 6961677 | 231113 | C | T | C | 0.74194 | T | 0.25806 | 14 | 0.45161 |
| 14860 | S19_7240586 | 19 | 7240586 | 278909 | G | A | G | 0.70968 | A | 0.29032 | 16 | 0.51613 |
| 14861 | S19_7240590 | 19 | 7240590 | 4 | G | T | G | 0.6129 | T | 0.3871 | 16 | 0.51613 |
| 14862 | S19_7240716 | 19 | 7240716 | 126 | G | A | G | 0.90323 | A | 0.09677 | 6 | 0.19355 |
| 14863 | S19_7483162 | 19 | 7483162 | 242446 | A | C | A | 0.90323 | C | 0.09677 | 6 | 0.19355 |
| 14864 | S19_7483194 | 19 | 7483194 | 32 | A | C | C | 0.62903 | A | 0.37097 | 17 | 0.54839 |
| 14865 | S19_7483291 | 19 | 7483291 | 97 | G | A | G | 0.79032 | A | 0.20968 | 9 | 0.29032 |
| 14866 | S19_7812256 | 19 | 7812256 | 328965 | G | A | G | 0.82258 | A | 0.17742 | 11 | 0.35484 |
| 14867 | S19_7812266 | 19 | 7812266 | 10 | C | T | C | 0.8871 | T | 0.1129 | 7 | 0.22581 |
| 14868 | S19_7812297 | 19 | 7812297 | 31 | T | C | T | 0.93548 | C | 0.06452 | 4 | 0.12903 |
| 14869 | S19_7812322 | 19 | 7812322 | 25 | T | C | T | 0.70968 | C | 0.29032 | 10 | 0.32258 |
| 14870 | S19_7812343 | 19 | 7812343 | 21 | C | G | G | 0.77419 | C | 0.22581 | 10 | 0.32258 |
| 14871 | S19_7812344 | 19 | 7812344 | 1 | T | C | C | 0.77419 | T | 0.22581 | 10 | 0.32258 |
| 14872 | S19_7812348 | 19 | 7812348 | 4 | C | A | A | 0.77419 | C | 0.22581 | 10 | 0.32258 |
| 14873 | S19_7812388 | 19 | 7812388 | 40 | T | C | C | 0.77419 | T | 0.22581 | 10 | 0.32258 |
| 14874 | S19_7911259 | 19 | 7911259 | 98871 | T | C | T | 0.8871 | C | 0.1129 | 7 | 0.22581 |
| 14875 | S19_7911307 | 19 | 7911307 | 48 | C | A | C | 0.90323 | A | 0.09677 | 6 | 0.19355 |
| 14876 | S19_8160896 | 19 | 8160896 | 249589 | C | T | C | 0.93548 | T | 0.06452 | 4 | 0.12903 |
| 14877 | S19_8189109 | 19 | 8189109 | 28213 | C | T | C | 0.91935 | T | 0.08065 | 5 | 0.16129 |
| 14878 | S19_8189224 | 19 | 8189224 | 115 | A | C | A | 0.90323 | C | 0.09677 | 4 | 0.12903 |
| 14879 | S19_8380543 | 19 | 8380543 | 191319 | A | G | G | 0.54839 | A | 0.45161 | 20 | 0.64516 |
| 14880 | S19_8385516 | 19 | 8385516 | 4973 | C | T | C | 0.90323 | T | 0.09677 | 6 | 0.19355 |
| 14881 | S19_9129537 | 19 | 9129537 | 744021 | G | C | C | 0.83871 | G | 0.16129 | 10 | 0.32258 |
| 14882 | S19_9129594 | 19 | 9129594 | 57 | T | G | T | 0.83871 | G | 0.16129 | 8 | 0.25806 |
| 14883 | S19_9129614 | 19 | 9129614 | 20 | C | T | C | 0.77419 | T | 0.22581 | 10 | 0.32258 |
| 14884 | S19_9129671 | 19 | 9129671 | 57 | G | A | G | 0.91935 | A | 0.08065 | 5 | 0.16129 |
| 14885 | S19_9129722 | 19 | 9129722 | 51 | C | T | T | 0.74194 | C | 0.25806 | 10 | 0.32258 |
| 14886 | S19_9129761 | 19 | 9129761 | 39 | G | A | G | 0.75806 | A | 0.24194 | 11 | 0.35484 |
| 14887 | S19_9129784 | 19 | 9129784 | 23 | C | T | C | 0.82258 | T | 0.17742 | 11 | 0.35484 |
| 14888 | S19_9479404 | 19 | 9479404 | 349620 | G | A | A | 0.91935 | G | 0.08065 | 5 | 0.16129 |
| 14889 | S19_9479541 | 19 | 9479541 | 137 | A | G | A | 0.90323 | G | 0.09677 | 6 | 0.19355 |
| 14890 | S19_9633634 | 19 | 9633634 | 154093 | C | T | T | 0.87097 | C | 0.12903 | 4 | 0.12903 |
| 14891 | S19_9633640 | 19 | 9633640 | 6 | G | A | G | 0.91935 | A | 0.08065 | 5 | 0.16129 |
| 14892 | S19_10042172 | 19 | 10042172 | 408532 | G | A | G | 0.58065 | A | 0.41935 | 20 | 0.64516 |
| 14893 | S19_10236266 | 19 | 10236266 | 194094 | G | A | G | 0.77419 | A | 0.22581 | 8 | 0.25806 |
| 14894 | S19_10236333 | 19 | 10236333 | 67 | C | T | C | 0.91935 | T | 0.08065 | 5 | 0.16129 |
| 14895 | S19_10236432 | 19 | 10236432 | 99 | G | A | A | 0.91935 | G | 0.08065 | 5 | 0.16129 |
| 14896 | S19_10236445 | 19 | 10236445 | 13 | A | G | G | 0.93548 | A | 0.06452 | 4 | 0.12903 |
| 14897 | S19_10466188 | 19 | 10466188 | 229743 | C | T | C | 0.93548 | T | 0.06452 | 4 | 0.12903 |
| 14898 | S19_10656235 | 19 | 10656235 | 190047 | T | C | C | 0.59677 | T | 0.40323 | 15 | 0.48387 |
| 14899 | S19_11293091 | 19 | 11293091 | 636856 | C | T | C | 0.90323 | T | 0.09677 | 6 | 0.19355 |
| 14900 | S19_11293289 | 19 | 11293289 | 198 | A | G | G | 0.91935 | A | 0.08065 | 5 | 0.16129 |
| 14901 | S19_11293304 | 19 | 11293304 | 15 | T | C | T | 0.59677 | C | 0.40323 | 21 | 0.67742 |
| 14902 | S19_11293306 | 19 | 11293306 | 2 | T | C | T | 0.59677 | C | 0.40323 | 21 | 0.67742 |

| Site number | SNP name | Chromosome | Physical position | Physical distance from the previous SNP | Reference allele | Alternative allele | Major allele | Major allele frequency | Minor allele | Minor allele frequency | Number heterozygous | Heterozygosity |
|---|---|---|---|---|---|---|---|---|---|---|---|---|
| 14903 | S19_11302269 | 19 | 11302269 | 8963 | G | A | G | 0.62903 | A | 0.37097 | 17 | 0.54839 |
| 14904 | S19_11302316 | 19 | 11302316 | 47 | A | G | A | 0.87097 | G | 0.12903 | 8 | 0.25806 |
| 14905 | S19_11302379 | 19 | 11302379 | 63 | C | T | C | 0.85484 | T | 0.14516 | 7 | 0.22581 |
| 14906 | S19_11302455 | 19 | 11302455 | 76 | C | A | C | 0.62903 | A | 0.37097 | 17 | 0.54839 |
| 14907 | S19_11302486 | 19 | 11302486 | 31 | A | C | A | 0.62903 | C | 0.37097 | 17 | 0.54839 |
| 14908 | S19_11302493 | 19 | 11302493 | 7 | G | A | G | 0.85484 | A | 0.14516 | 7 | 0.22581 |
| 14909 | S19_11638743 | 19 | 11638743 | 336250 | G | A | G | 0.95161 | A | 0.04839 | 3 | 0.09677 |
| 14910 | S19_11840913 | 19 | 11840913 | 202170 | G | C | G | 0.90323 | C | 0.09677 | 6 | 0.19355 |
| 14911 | S19_11840914 | 19 | 11840914 | 1 | C | T | C | 0.90323 | T | 0.09677 | 6 | 0.19355 |
| 14912 | S19_11840963 | 19 | 11840963 | 49 | T | C | C | 0.64516 | T | 0.35484 | 20 | 0.64516 |
| 14913 | S19_12004369 | 19 | 12004369 | 163406 | G | A | G | 0.54839 | A | 0.45161 | 14 | 0.45161 |
| 14914 | S19_12004384 | 19 | 12004384 | 15 | A | G | A | 0.54839 | G | 0.45161 | 14 | 0.45161 |
| 14915 | S19_12004401 | 19 | 12004401 | 17 | A | C | A | 0.95161 | C | 0.04839 | 3 | 0.09677 |
| 14916 | S19_12004482 | 19 | 12004482 | 81 | T | G | G | 0.5 | T | 0.5 | 13 | 0.41935 |
| 14917 | S19_12004505 | 19 | 12004505 | 23 | T | G | T | 0.72581 | G | 0.27419 | 9 | 0.29032 |
| 14918 | S19_12183013 | 19 | 12183013 | 178508 | T | A | T | 0.66129 | A | 0.33871 | 17 | 0.54839 |
| 14919 | S19_12183084 | 19 | 12183084 | 71 | A | G | G | 0.95161 | A | 0.04839 | 3 | 0.09677 |
| 14920 | S19_12183219 | 19 | 12183219 | 135 | T | C | T | 0.79032 | C | 0.20968 | 13 | 0.41935 |
| 14921 | S19_12233812 | 19 | 12233812 | 50593 | T | C | T | 0.90323 | C | 0.09677 | 6 | 0.19355 |
| 14922 | S19_12290154 | 19 | 12290154 | 56342 | C | T | C | 0.8871 | T | 0.1129 | 7 | 0.22581 |
| 14923 | S19_12320579 | 19 | 12320579 | 30425 | G | A | G | 0.8871 | A | 0.1129 | 7 | 0.22581 |
| 14924 | S19_12320688 | 19 | 12320688 | 109 | C | T | C | 0.62903 | T | 0.37097 | 11 | 0.35484 |
| 14925 | S19_12513923 | 19 | 12513923 | 193235 | C | T | C | 0.95161 | T | 0.04839 | 3 | 0.09677 |
| 14926 | S19_12513974 | 19 | 12513974 | 51 | G | A | G | 0.95161 | A | 0.04839 | 3 | 0.09677 |
| 14927 | S19_12513992 | 19 | 12513992 | 18 | C | T | C | 0.83871 | T | 0.16129 | 10 | 0.32258 |
| 14928 | S19_12514072 | 19 | 12514072 | 80 | C | T | C | 0.83871 | T | 0.16129 | 10 | 0.32258 |
| 14929 | S19_13043307 | 19 | 13043307 | 529235 | G | T | G | 0.82258 | T | 0.17742 | 9 | 0.29032 |
| 14930 | S19_13132082 | 19 | 13132082 | 88775 | A | G | A | 0.5 | G | 0.5 | 19 | 0.6129 |
| 14931 | S19_13132140 | 19 | 13132140 | 58 | C | T | C | 0.93548 | T | 0.06452 | 4 | 0.12903 |
| 14932 | S19_13342162 | 19 | 13342162 | 210022 | T | C | C | 0.58065 | T | 0.41935 | 14 | 0.45161 |
| 14933 | S19_13342171 | 19 | 13342171 | 9 | G | A | G | 0.64516 | A | 0.35484 | 10 | 0.32258 |
| 14934 | S19_13347682 | 19 | 13347682 | 5511 | A | G | A | 0.95161 | G | 0.04839 | 3 | 0.09677 |
| 14935 | S19_13599919 | 19 | 13599919 | 252237 | G | C | C | 0.5 | G | 0.5 | 11 | 0.35484 |
| 14936 | S19_13599933 | 19 | 13599933 | 14 | T | C | C | 0.5 | T | 0.5 | 11 | 0.35484 |
| 14937 | S19_13600054 | 19 | 13600054 | 121 | T | C | C | 0.74194 | T | 0.25806 | 10 | 0.32258 |
| 14938 | S19_13625087 | 19 | 13625087 | 25033 | A | T | A | 0.91935 | T | 0.08065 | 5 | 0.16129 |
| 14939 | S19_13748128 | 19 | 13748128 | 123041 | T | C | T | 0.8871 | C | 0.1129 | 7 | 0.22581 |
| 14940 | S19_13928235 | 19 | 13928235 | 180107 | C | T | C | 0.67742 | T | 0.32258 | 14 | 0.45161 |
| 14941 | S19_14227679 | 19 | 14227679 | 299444 | T | C | T | 0.87097 | C | 0.12903 | 4 | 0.12903 |
| 14942 | S19_14227681 | 19 | 14227681 | 2 | G | C | G | 0.82258 | C | 0.17742 | 7 | 0.22581 |
| 14943 | S19_14227689 | 19 | 14227689 | 8 | G | A | A | 0.79032 | G | 0.20968 | 11 | 0.35484 |
| 14944 | S19_14227856 | 19 | 14227856 | 167 | G | A | G | 0.56452 | A | 0.43548 | 13 | 0.41935 |
| 14945 | S19_14227864 | 19 | 14227864 | 8 | A | G | G | 0.6129 | A | 0.3871 | 14 | 0.45161 |
| 14946 | S19_14227912 | 19 | 14227912 | 48 | T | G | T | 0.69355 | G | 0.30645 | 11 | 0.35484 |
| 14947 | S19_14749107 | 19 | 14749107 | 521195 | A | G | A | 0.83871 | G | 0.16129 | 8 | 0.25806 |
| 14948 | S19_14749113 | 19 | 14749113 | 6 | G | A | G | 0.83871 | A | 0.16129 | 8 | 0.25806 |
| 14949 | S19_14749226 | 19 | 14749226 | 113 | C | T | C | 0.80645 | T | 0.19355 | 10 | 0.32258 |
| 14950 | S19_15038090 | 19 | 15038090 | 288864 | A | G | A | 0.85484 | G | 0.14516 | 9 | 0.29032 |
| 14951 | S19_15038288 | 19 | 15038288 | 198 | C | T | C | 0.85484 | T | 0.14516 | 9 | 0.29032 |
| 14952 | S19_15087513 | 19 | 15087513 | 49225 | T | A | T | 0.91935 | A | 0.08065 | 5 | 0.16129 |
| 14953 | S19_15087574 | 19 | 15087574 | 61 | C | T | C | 0.90323 | T | 0.09677 | 4 | 0.12903 |
| 14954 | S19_15087710 | 19 | 15087710 | 136 | A | G | A | 0.93548 | G | 0.06452 | 4 | 0.12903 |
| 14955 | S19_15335442 | 19 | 15335442 | 247732 | G | T | G | 0.83871 | T | 0.16129 | 8 | 0.25806 |
| 14956 | S19_15335507 | 19 | 15335507 | 65 | T | G | T | 0.83871 | G | 0.16129 | 8 | 0.25806 |

| Site number | SNP name | Chromosome | Physical position | Physical distance from the previous SNP | Reference allele | Alternative allele | Major allele | Major allele frequency | Minor allele | Minor allele frequency | Number heterozygous | Heterozygosity |
|---|---|---|---|---|---|---|---|---|---|---|---|---|
| 14957 | S19_15667954 | 19 | 15667954 | 332447 | C | G | C | 0.93548 | G | 0.06452 | 4 | 0.12903 |
| 14958 | S19_15668028 | 19 | 15668028 | 74 | T | C | T | 0.83871 | C | 0.16129 | 10 | 0.32258 |
| 14959 | S19_15668085 | 19 | 15668085 | 57 | C | T | C | 0.90323 | T | 0.09677 | 6 | 0.19355 |
| 14960 | S19_15668119 | 19 | 15668119 | 34 | C | T | C | 0.91935 | T | 0.08065 | 3 | 0.09677 |
| 14961 | S19_15668138 | 19 | 15668138 | 19 | T | C | T | 0.82258 | C | 0.17742 | 7 | 0.22581 |
| 14962 | S19_15706469 | 19 | 15706469 | 38331 | C | T | C | 0.85484 | T | 0.14516 | 7 | 0.22581 |
| 14963 | S19_15706482 | 19 | 15706482 | 13 | G | A | G | 0.85484 | A | 0.14516 | 7 | 0.22581 |
| 14964 | S19_15706544 | 19 | 15706544 | 62 | C | T | C | 0.85484 | T | 0.14516 | 7 | 0.22581 |
| 14965 | S19_15706557 | 19 | 15706557 | 13 | G | T | G | 0.85484 | T | 0.14516 | 7 | 0.22581 |
| 14966 | S19_15706578 | 19 | 15706578 | 21 | C | T | C | 0.90323 | T | 0.09677 | 6 | 0.19355 |
| 14967 | S19_15706675 | 19 | 15706675 | 97 | G | C | G | 0.85484 | C | 0.14516 | 7 | 0.22581 |
| 14968 | S19_15743618 | 19 | 15743618 | 36943 | T | G | T | 0.72581 | G | 0.27419 | 13 | 0.41935 |
| 14969 | S19_15743631 | 19 | 15743631 | 13 | G | T | G | 0.83871 | T | 0.16129 | 8 | 0.25806 |
| 14970 | S19_15743642 | 19 | 15743642 | 11 | A | G | A | 0.85484 | G | 0.14516 | 7 | 0.22581 |
| 14971 | S19_15743656 | 19 | 15743656 | 14 | C | T | C | 0.85484 | T | 0.14516 | 7 | 0.22581 |
| 14972 | S19_15743790 | 19 | 15743790 | 134 | A | G | A | 0.85484 | G | 0.14516 | 7 | 0.22581 |
| 14973 | S19_15743794 | 19 | 15743794 | 4 | A | G | A | 0.85484 | G | 0.14516 | 7 | 0.22581 |
| 14974 | S19_15854866 | 19 | 15854866 | 111072 | C | T | C | 0.54839 | T | 0.45161 | 16 | 0.51613 |
| 14975 | S19_15996637 | 19 | 15996637 | 141771 | A | G | A | 0.79032 | G | 0.20968 | 9 | 0.29032 |
| 14976 | S19_15996776 | 19 | 15996776 | 139 | G | A | G | 0.83871 | A | 0.16129 | 10 | 0.32258 |
| 14977 | S19_15996810 | 19 | 15996810 | 34 | T | C | T | 0.62903 | C | 0.37097 | 11 | 0.35484 |
| 14978 | S19_16078199 | 19 | 16078199 | 81389 | G | T | G | 0.95161 | T | 0.04839 | 3 | 0.09677 |
| 14979 | S19_16078208 | 19 | 16078208 | 9 | G | C | C | 0.59677 | G | 0.40323 | 11 | 0.35484 |
| 14980 | S19_16078238 | 19 | 16078238 | 30 | C | G | G | 0.79032 | C | 0.20968 | 9 | 0.29032 |
| 14981 | S19_16078287 | 19 | 16078287 | 49 | C | T | T | 0.82258 | C | 0.17742 | 11 | 0.35484 |
| 14982 | S19_16098831 | 19 | 16098831 | 20544 | C | A | C | 0.91935 | A | 0.08065 | 5 | 0.16129 |
| 14983 | S19_16098863 | 19 | 16098863 | 32 | G | A | G | 0.64516 | A | 0.35484 | 10 | 0.32258 |
| 14984 | S19_16098876 | 19 | 16098876 | 13 | T | C | T | 0.93548 | C | 0.06452 | 4 | 0.12903 |
| 14985 | S19_16149295 | 19 | 16149295 | 50419 | C | T | C | 0.91935 | T | 0.08065 | 5 | 0.16129 |
| 14986 | S19_16149296 | 19 | 16149296 | 1 | C | G | G | 0.56452 | C | 0.43548 | 17 | 0.54839 |
| 14987 | S19_16149302 | 19 | 16149302 | 6 | C | T | C | 0.95161 | T | 0.04839 | 3 | 0.09677 |
| 14988 | S19_16191762 | 19 | 16191762 | 42460 | G | A | G | 0.53226 | A | 0.46774 | 15 | 0.48387 |
| 14989 | S19_16191808 | 19 | 16191808 | 46 | T | C | C | 0.70968 | T | 0.29032 | 10 | 0.32258 |
| 14990 | S19_16191812 | 19 | 16191812 | 4 | G | A | G | 0.77419 | A | 0.22581 | 8 | 0.25806 |
| 14991 | S19_16191817 | 19 | 16191817 | 5 | T | C | C | 0.70968 | T | 0.29032 | 10 | 0.32258 |
| 14992 | S19_16191844 | 19 | 16191844 | 27 | C | A | C | 0.75806 | A | 0.24194 | 11 | 0.35484 |
| 14993 | S19_16191853 | 19 | 16191853 | 9 | T | G | T | 0.53226 | G | 0.46774 | 15 | 0.48387 |
| 14994 | S19_16191867 | 19 | 16191867 | 14 | T | C | T | 0.53226 | C | 0.46774 | 15 | 0.48387 |
| 14995 | S19_16191874 | 19 | 16191874 | 7 | G | C | G | 0.53226 | C | 0.46774 | 15 | 0.48387 |
| 14996 | S19_16191889 | 19 | 16191889 | 15 | T | C | C | 0.93548 | T | 0.06452 | 4 | 0.12903 |
| 14997 | S19_16191899 | 19 | 16191899 | 10 | G | A | G | 0.53226 | A | 0.46774 | 15 | 0.48387 |
| 14998 | S19_16191933 | 19 | 16191933 | 34 | A | G | A | 0.90323 | G | 0.09677 | 4 | 0.12903 |
| 14999 | S19_16191956 | 19 | 16191956 | 23 | T | C | T | 0.77419 | C | 0.22581 | 8 | 0.25806 |
| 15000 | S19_16225710 | 19 | 16225710 | 33754 | G | T | G | 0.95161 | T | 0.04839 | 3 | 0.09677 |
| 15001 | S19_16336111 | 19 | 16336111 | 110401 | A | G | G | 0.62903 | A | 0.37097 | 15 | 0.48387 |
| 15002 | S19_16336223 | 19 | 16336223 | 112 | C | T | C | 0.95161 | T | 0.04839 | 3 | 0.09677 |
| 15003 | S19_16455503 | 19 | 16455503 | 119280 | G | T | G | 0.93548 | T | 0.06452 | 4 | 0.12903 |
| 15004 | S19_16455557 | 19 | 16455557 | 54 | T | C | C | 0.87097 | T | 0.12903 | 4 | 0.12903 |
| 15005 | S19_16455601 | 19 | 16455601 | 44 | C | T | C | 0.8871 | T | 0.1129 | 7 | 0.22581 |
| 15006 | S19_16455629 | 19 | 16455629 | 28 | A | G | A | 0.8871 | G | 0.1129 | 7 | 0.22581 |
| 15007 | S19_16455635 | 19 | 16455635 | 6 | C | T | C | 0.93548 | T | 0.06452 | 4 | 0.12903 |
| 15008 | S19_16455639 | 19 | 16455639 | 4 | G | A | G | 0.75806 | A | 0.24194 | 15 | 0.48387 |
| 15009 | S19_16455642 | 19 | 16455642 | 3 | T | A | T | 0.8871 | A | 0.1129 | 7 | 0.22581 |
| 15010 | S19_16497182 | 19 | 16497182 | 41540 | A | G | A | 0.91935 | G | 0.08065 | 5 | 0.16129 |

| Site number | SNP name | Chromosome | Physical position | Physical distance from the previous SNP | Reference allele | Alternative allele | Major allele | Major allele frequency | Minor allele | Minor allele frequency | Number heterozygous | Heterozygosity |
|---|---|---|---|---|---|---|---|---|---|---|---|---|
| 15011 | S19_16497250 | 19 | 16497250 | 68 | C | G | C | 0.8871 | G | 0.1129 | 5 | 0.16129 |
| 15012 | S19_16587036 | 19 | 16587036 | 89786 | T | G | T | 0.58065 | G | 0.41935 | 18 | 0.58065 |
| 15013 | S19_16587115 | 19 | 16587115 | 79 | A | G | A | 0.87097 | G | 0.12903 | 6 | 0.19355 |
| 15014 | S19_16759213 | 19 | 16759213 | 172098 | T | C | T | 0.6129 | C | 0.3871 | 12 | 0.3871 |
| 15015 | S19_16875311 | 19 | 16875311 | 116098 | G | A | G | 0.91935 | A | 0.08065 | 5 | 0.16129 |
| 15016 | S19_16875324 | 19 | 16875324 | 13 | C | T | C | 0.90323 | T | 0.09677 | 6 | 0.19355 |
| 15017 | S19_16875389 | 19 | 16875389 | 65 | A | G | G | 0.8871 | A | 0.1129 | 7 | 0.22581 |
| 15018 | S19_16954442 | 19 | 16954442 | 79053 | T | C | T | 0.93548 | C | 0.06452 | 4 | 0.12903 |
| 15019 | S19_16954564 | 19 | 16954564 | 122 | G | C | G | 0.82258 | C | 0.17742 | 7 | 0.22581 |
| 15020 | S19_16954597 | 19 | 16954597 | 33 | A | G | A | 0.74194 | G | 0.25806 | 14 | 0.45161 |
| 15021 | S19_16954612 | 19 | 16954612 | 15 | G | A | G | 0.91935 | A | 0.08065 | 5 | 0.16129 |
| 15022 | S19_17156418 | 19 | 17156418 | 201806 | G | A | G | 0.93548 | A | 0.06452 | 4 | 0.12903 |
| 15023 | S19_17156553 | 19 | 17156553 | 135 | A | G | A | 0.93548 | G | 0.06452 | 4 | 0.12903 |
| 15024 | S19_17156585 | 19 | 17156585 | 32 | A | G | A | 0.85484 | G | 0.14516 | 7 | 0.22581 |
| 15025 | S19_17256311 | 19 | 17256311 | 99726 | C | T | C | 0.8871 | T | 0.1129 | 5 | 0.16129 |
| 15026 | S19_17310840 | 19 | 17310840 | 54529 | A | G | A | 0.70968 | G | 0.29032 | 14 | 0.45161 |
| 15027 | S19_17686588 | 19 | 17686588 | 375748 | A | C | A | 0.90323 | C | 0.09677 | 4 | 0.12903 |
| 15028 | S19_17686605 | 19 | 17686605 | 17 | T | C | T | 0.79032 | C | 0.20968 | 11 | 0.35484 |
| 15029 | S19_18047152 | 19 | 18047152 | 360547 | G | A | G | 0.80645 | A | 0.19355 | 10 | 0.32258 |
| 15030 | S19_18047268 | 19 | 18047268 | 116 | C | T | C | 0.70968 | T | 0.29032 | 18 | 0.58065 |
| 15031 | S19_19106053 | 19 | 19106053 | 1058785 | A | G | A | 0.95161 | G | 0.04839 | 3 | 0.09677 |
| 15032 | S19_19106056 | 19 | 19106056 | 3 | T | C | T | 0.54839 | C | 0.45161 | 22 | 0.70968 |
| 15033 | S19_19106065 | 19 | 19106065 | 9 | C | T | C | 0.93548 | T | 0.06452 | 4 | 0.12903 |
| 15034 | S19_19143439 | 19 | 19143439 | 37374 | G | C | G | 0.91935 | C | 0.08065 | 5 | 0.16129 |
| 15035 | S19_19143481 | 19 | 19143481 | 42 | T | C | T | 0.83871 | C | 0.16129 | 10 | 0.32258 |
| 15036 | S19_19240391 | 19 | 19240391 | 96910 | C | T | C | 0.69355 | T | 0.30645 | 11 | 0.35484 |
| 15037 | S19_19295623 | 19 | 19295623 | 55232 | C | T | C | 0.8871 | T | 0.1129 | 7 | 0.22581 |
| 15038 | S19_19295719 | 19 | 19295719 | 96 | C | T | C | 0.8871 | T | 0.1129 | 7 | 0.22581 |
| 15039 | S19_19295759 | 19 | 19295759 | 40 | G | A | G | 0.70968 | A | 0.29032 | 12 | 0.3871 |
| 15040 | S19_19295771 | 19 | 19295771 | 12 | G | A | G | 0.8871 | A | 0.1129 | 7 | 0.22581 |
| 15041 | S19_19352098 | 19 | 19352098 | 56327 | A | G | G | 0.8871 | A | 0.1129 | 5 | 0.16129 |
| 15042 | S19_19352195 | 19 | 19352195 | 97 | T | C | T | 0.83871 | C | 0.16129 | 8 | 0.25806 |
| 15043 | S19_19374204 | 19 | 19374204 | 22009 | C | T | C | 0.91935 | T | 0.08065 | 5 | 0.16129 |
| 15044 | S19_19374240 | 19 | 19374240 | 36 | T | C | T | 0.74194 | C | 0.25806 | 12 | 0.3871 |
| 15045 | S19_19374280 | 19 | 19374280 | 40 | C | A | A | 0.72581 | C | 0.27419 | 15 | 0.48387 |
| 15046 | S19_19374344 | 19 | 19374344 | 64 | G | A | A | 0.66129 | G | 0.33871 | 15 | 0.48387 |
| 15047 | S19_19427159 | 19 | 19427159 | 52815 | A | G | G | 0.8871 | A | 0.1129 | 7 | 0.22581 |
| 15048 | S19_19427172 | 19 | 19427172 | 13 | G | T | G | 0.95161 | T | 0.04839 | 3 | 0.09677 |
| 15049 | S19_19427194 | 19 | 19427194 | 22 | A | G | A | 0.8871 | G | 0.1129 | 5 | 0.16129 |
| 15050 | S19_19427208 | 19 | 19427208 | 14 | C | T | C | 0.91935 | T | 0.08065 | 5 | 0.16129 |
| 15051 | S19_19427209 | 19 | 19427209 | 1 | G | A | G | 0.95161 | A | 0.04839 | 3 | 0.09677 |
| 15052 | S19_19729968 | 19 | 19729968 | 302759 | G | A | G | 0.80645 | A | 0.19355 | 10 | 0.32258 |
| 15053 | S19_19730093 | 19 | 19730093 | 125 | G | C | C | 0.70968 | G | 0.29032 | 10 | 0.32258 |
| 15054 | S19_19730153 | 19 | 19730153 | 60 | C | T | T | 0.75806 | C | 0.24194 | 9 | 0.29032 |
| 15055 | S19_19975220 | 19 | 19975220 | 245067 | A | C | A | 0.77419 | C | 0.22581 | 12 | 0.3871 |
| 15056 | S19_19975310 | 19 | 19975310 | 90 | G | A | G | 0.95161 | A | 0.04839 | 1 | 0.03226 |
| 15057 | S19_21138041 | 19 | 21138041 | 1162731 | A | G | A | 0.72581 | G | 0.27419 | 9 | 0.29032 |
| 15058 | S19_21686735 | 19 | 21686735 | 548694 | C | T | C | 0.93548 | T | 0.06452 | 2 | 0.06452 |
| 15059 | S19_21686950 | 19 | 21686950 | 215 | G | A | G | 0.83871 | A | 0.16129 | 10 | 0.32258 |
| 15060 | S19_21789742 | 19 | 21789742 | 102792 | C | T | C | 0.90323 | T | 0.09677 | 6 | 0.19355 |
| 15061 | S19_21789763 | 19 | 21789763 | 21 | A | C | C | 0.56452 | A | 0.43548 | 15 | 0.48387 |
| 15062 | S19_21789778 | 19 | 21789778 | 15 | C | T | C | 0.90323 | T | 0.09677 | 6 | 0.19355 |
| 15063 | S19_21789799 | 19 | 21789799 | 21 | G | T | G | 0.85484 | T | 0.14516 | 7 | 0.22581 |
| 15064 | S19_21820338 | 19 | 21820338 | 30539 | A | G | G | 0.72581 | A | 0.27419 | 9 | 0.29032 |

| Site number | SNP name | Chromosome | Physical position | Physical distance from the previous SNP | Reference allele | Alternative allele | Major allele | Major allele frequency | Minor allele | Minor allele frequency | Number heterozygous | Heterozygosity |
|---|---|---|---|---|---|---|---|---|---|---|---|---|
| 15065 | S19_21966066 | 19 | 21966066 | 145728 | G | A | A | 0.75806 | G | 0.24194 | 11 | 0.35484 |
| 15066 | S19_21966287 | 19 | 21966287 | 221 | A | G | G | 0.75806 | A | 0.24194 | 11 | 0.35484 |
| 15067 | S19_21966296 | 19 | 21966296 | 9 | C | T | C | 0.75806 | T | 0.24194 | 11 | 0.35484 |
| 15068 | S19_22496024 | 19 | 22496024 | 529728 | C | T | C | 0.83871 | T | 0.16129 | 6 | 0.19355 |
| 15069 | S19_22558860 | 19 | 22558860 | 62836 | C | A | C | 0.95161 | A | 0.04839 | 3 | 0.09677 |
| 15070 | S19_22707729 | 19 | 22707729 | 148869 | G | A | G | 0.59677 | A | 0.40323 | 11 | 0.35484 |
| 15071 | S19_22793845 | 19 | 22793845 | 86116 | C | T | C | 0.93548 | T | 0.06452 | 4 | 0.12903 |
| 15072 | S19_22793902 | 19 | 22793902 | 57 | C | T | C | 0.87097 | T | 0.12903 | 6 | 0.19355 |
| 15073 | S19_22803318 | 19 | 22803318 | 9416 | A | G | G | 0.80645 | A | 0.19355 | 8 | 0.25806 |
| 15074 | S19_22866183 | 19 | 22866183 | 62865 | A | G | G | 0.83871 | A | 0.16129 | 10 | 0.32258 |
| 15075 | S19_23496237 | 19 | 23496237 | 630054 | A | G | G | 0.93548 | A | 0.06452 | 2 | 0.06452 |
| 15076 | S19_23588207 | 19 | 23588207 | 91970 | A | G | G | 0.62903 | A | 0.37097 | 11 | 0.35484 |
| 15077 | S19_23913463 | 19 | 23913463 | 325256 | C | A | C | 0.64516 | A | 0.35484 | 14 | 0.45161 |
| 15078 | S19_23913480 | 19 | 23913480 | 17 | C | T | C | 0.80645 | T | 0.19355 | 8 | 0.25806 |
| 15079 | S19_23913489 | 19 | 23913489 | 9 | T | C | C | 0.51613 | T | 0.48387 | 22 | 0.70968 |
| 15080 | S19_23913491 | 19 | 23913491 | 2 | G | C | C | 0.51613 | G | 0.48387 | 22 | 0.70968 |
| 15081 | S19_23913493 | 19 | 23913493 | 2 | T | C | C | 0.51613 | T | 0.48387 | 22 | 0.70968 |
| 15082 | S19_23913529 | 19 | 23913529 | 36 | A | G | G | 0.51613 | A | 0.48387 | 14 | 0.45161 |
| 15083 | S19_24095519 | 19 | 24095519 | 181990 | G | A | G | 0.93548 | A | 0.06452 | 4 | 0.12903 |
| 15084 | S19_24213512 | 19 | 24213512 | 117993 | C | A | C | 0.82258 | A | 0.17742 | 11 | 0.35484 |
| 15085 | S19_24213625 | 19 | 24213625 | 113 | A | T | A | 0.72581 | T | 0.27419 | 11 | 0.35484 |
| 15086 | S19_24217584 | 19 | 24217584 | 3959 | A | G | A | 0.95161 | G | 0.04839 | 3 | 0.09677 |
| 15087 | S19_24217694 | 19 | 24217694 | 110 | C | A | C | 0.90323 | A | 0.09677 | 6 | 0.19355 |
| 15088 | S19_24217695 | 19 | 24217695 | 1 | C | T | C | 0.8871 | T | 0.1129 | 7 | 0.22581 |
| 15089 | S19_24217717 | 19 | 24217717 | 22 | C | A | C | 0.90323 | A | 0.09677 | 6 | 0.19355 |
| 15090 | S19_24217821 | 19 | 24217821 | 104 | A | C | A | 0.90323 | C | 0.09677 | 6 | 0.19355 |
| 15091 | S19_24461009 | 19 | 24461009 | 243188 | G | A | G | 0.82258 | A | 0.17742 | 11 | 0.35484 |
| 15092 | S19_24461017 | 19 | 24461017 | 8 | C | T | C | 0.91935 | T | 0.08065 | 5 | 0.16129 |
| 15093 | S19_24509992 | 19 | 24509992 | 48975 | T | G | G | 0.8871 | T | 0.1129 | 5 | 0.16129 |
| 15094 | S19_24509993 | 19 | 24509993 | 1 | T | C | T | 0.91935 | C | 0.08065 | 5 | 0.16129 |
| 15095 | S19_24702642 | 19 | 24702642 | 192649 | G | A | G | 0.67742 | A | 0.32258 | 10 | 0.32258 |
| 15096 | S19_24702659 | 19 | 24702659 | 17 | G | A | G | 0.72581 | A | 0.27419 | 11 | 0.35484 |
| 15097 | S19_24702702 | 19 | 24702702 | 43 | G | A | G | 0.69355 | A | 0.30645 | 11 | 0.35484 |
| 15098 | S19_24898960 | 19 | 24898960 | 196258 | C | T | C | 0.85484 | T | 0.14516 | 5 | 0.16129 |
| 15099 | S19_25125054 | 19 | 25125054 | 226094 | G | A | G | 0.69355 | A | 0.30645 | 11 | 0.35484 |
| 15100 | S19_25125165 | 19 | 25125165 | 111 | C | T | T | 0.67742 | C | 0.32258 | 12 | 0.3871 |
| 15101 | S19_25228631 | 19 | 25228631 | 103466 | G | A | A | 0.56452 | G | 0.43548 | 15 | 0.48387 |
| 15102 | S19_25228728 | 19 | 25228728 | 97 | G | T | G | 0.79032 | T | 0.20968 | 11 | 0.35484 |
| 15103 | S19_25228758 | 19 | 25228758 | 30 | C | T | C | 0.69355 | T | 0.30645 | 9 | 0.29032 |
| 15104 | S19_25228774 | 19 | 25228774 | 16 | C | T | C | 0.79032 | T | 0.20968 | 11 | 0.35484 |
| 15105 | S19_25254941 | 19 | 25254941 | 26167 | G | C | G | 0.91935 | C | 0.08065 | 5 | 0.16129 |
| 15106 | S19_25255024 | 19 | 25255024 | 83 | T | G | T | 0.85484 | G | 0.14516 | 7 | 0.22581 |
| 15107 | S19_25255081 | 19 | 25255081 | 57 | A | G | A | 0.85484 | G | 0.14516 | 7 | 0.22581 |
| 15108 | S19_25262498 | 19 | 25262498 | 7417 | C | T | T | 0.6129 | C | 0.3871 | 16 | 0.51613 |
| 15109 | S19_25262503 | 19 | 25262503 | 5 | T | C | C | 0.82258 | T | 0.17742 | 9 | 0.29032 |
| 15110 | S19_25262508 | 19 | 25262508 | 5 | T | C | C | 0.62903 | T | 0.37097 | 17 | 0.54839 |
| 15111 | S19_25262520 | 19 | 25262520 | 12 | T | C | T | 0.79032 | C | 0.20968 | 11 | 0.35484 |
| 15112 | S19_25357504 | 19 | 25357504 | 94984 | T | C | T | 0.53226 | C | 0.46774 | 19 | 0.6129 |
| 15113 | S19_25357554 | 19 | 25357554 | 50 | T | C | T | 0.53226 | C | 0.46774 | 19 | 0.6129 |
| 15114 | S19_25357567 | 19 | 25357567 | 13 | C | A | C | 0.53226 | A | 0.46774 | 19 | 0.6129 |
| 15115 | S19_25489282 | 19 | 25489282 | 131715 | C | T | C | 0.95161 | T | 0.04839 | 3 | 0.09677 |
| 15116 | S19_25489407 | 19 | 25489407 | 125 | C | T | C | 0.85484 | T | 0.14516 | 9 | 0.29032 |
| 15117 | S19_25513928 | 19 | 25513928 | 24521 | T | A | T | 0.90323 | A | 0.09677 | 6 | 0.19355 |
| 15118 | S19_25513969 | 19 | 25513969 | 41 | A | C | A | 0.90323 | C | 0.09677 | 6 | 0.19355 |

| Site number | SNP name | Chromosome | Physical position | Physical distance from the previous SNP | Reference allele | Alternative allele | Major allele | Major allele frequency | Minor allele | Minor allele frequency | Number heterozygous | Heterozygosity |
|---|---|---|---|---|---|---|---|---|---|---|---|---|
| 15119 | S19_25553161 | 19 | 25553161 | 39192 | A | T | A | 0.91935 | T | 0.08065 | 5 | 0.16129 |
| 15120 | S19_25734429 | 19 | 25734429 | 181268 | C | T | C | 0.8871 | T | 0.1129 | 5 | 0.16129 |
| 15121 | S19_25734438 | 19 | 25734438 | 9 | G | T | G | 0.95161 | T | 0.04839 | 3 | 0.09677 |
| 15122 | S19_25734595 | 19 | 25734595 | 157 | G | A | G | 0.95161 | A | 0.04839 | 3 | 0.09677 |
| 15123 | S19_25734619 | 19 | 25734619 | 24 | T | C | T | 0.62903 | C | 0.37097 | 13 | 0.41935 |
| 15124 | S19_25734671 | 19 | 25734671 | 52 | T | C | T | 0.74194 | C | 0.25806 | 10 | 0.32258 |
| 15125 | S19_25970080 | 19 | 25970080 | 235409 | C | T | C | 0.87097 | T | 0.12903 | 6 | 0.19355 |
| 15126 | S19_26017705 | 19 | 26017705 | 47625 | G | A | G | 0.93548 | A | 0.06452 | 4 | 0.12903 |
| 15127 | S19_26017747 | 19 | 26017747 | 42 | T | C | T | 0.91935 | C | 0.08065 | 3 | 0.09677 |
| 15128 | S19_26017788 | 19 | 26017788 | 41 | G | T | G | 0.91935 | T | 0.08065 | 5 | 0.16129 |
| 15129 | S19_26017821 | 19 | 26017821 | 33 | G | A | G | 0.83871 | A | 0.16129 | 8 | 0.25806 |
| 15130 | S19_27467325 | 19 | 27467325 | 1449504 | G | A | G | 0.85484 | A | 0.14516 | 9 | 0.29032 |
| 15131 | S19_27609983 | 19 | 27609983 | 142658 | G | A | G | 0.87097 | A | 0.12903 | 8 | 0.25806 |
| 15132 | S19_27764392 | 19 | 27764392 | 154409 | G | A | G | 0.8871 | A | 0.1129 | 7 | 0.22581 |
| 15133 | S19_27764567 | 19 | 27764567 | 175 | A | G | A | 0.95161 | G | 0.04839 | 3 | 0.09677 |
| 15134 | S19_27969692 | 19 | 27969692 | 205125 | A | G | A | 0.93548 | G | 0.06452 | 4 | 0.12903 |
| 15135 | S19_28056137 | 19 | 28056137 | 86445 | C | T | C | 0.90323 | T | 0.09677 | 6 | 0.19355 |
| 15136 | S19_28056349 | 19 | 28056349 | 212 | C | T | C | 0.87097 | T | 0.12903 | 8 | 0.25806 |
| 15137 | S19_28529403 | 19 | 28529403 | 473054 | A | G | A | 0.90323 | G | 0.09677 | 6 | 0.19355 |
| 15138 | S19_28529548 | 19 | 28529548 | 145 | C | T | C | 0.74194 | T | 0.25806 | 12 | 0.3871 |
| 15139 | S19_28529576 | 19 | 28529576 | 28 | G | A | G | 0.85484 | A | 0.14516 | 7 | 0.22581 |
| 15140 | S19_28642285 | 19 | 28642285 | 112709 | C | A | C | 0.80645 | A | 0.19355 | 12 | 0.3871 |
| 15141 | S19_28642485 | 19 | 28642485 | 200 | G | A | G | 0.79032 | A | 0.20968 | 13 | 0.41935 |
| 15142 | S19_28744018 | 19 | 28744018 | 101533 | T | G | T | 0.74194 | G | 0.25806 | 14 | 0.45161 |
| 15143 | S19_28744057 | 19 | 28744057 | 39 | A | G | G | 0.64516 | A | 0.35484 | 14 | 0.45161 |
| 15144 | S19_28744113 | 19 | 28744113 | 56 | G | A | G | 0.85484 | A | 0.14516 | 5 | 0.16129 |
| 15145 | S19_28844784 | 19 | 28844784 | 100671 | G | C | G | 0.54839 | C | 0.45161 | 14 | 0.45161 |
| 15146 | S19_28848926 | 19 | 28848926 | 4142 | C | T | C | 0.74194 | T | 0.25806 | 10 | 0.32258 |
| 15147 | S19_28849087 | 19 | 28849087 | 161 | T | C | T | 0.8871 | C | 0.1129 | 7 | 0.22581 |
| 15148 | S19_29012120 | 19 | 29012120 | 163033 | A | G | G | 0.58065 | A | 0.41935 | 18 | 0.58065 |
| 15149 | S19_29012171 | 19 | 29012171 | 51 | A | G | G | 0.67742 | A | 0.32258 | 16 | 0.51613 |
| 15150 | S19_29134328 | 19 | 29134328 | 122157 | C | T | C | 0.72581 | T | 0.27419 | 15 | 0.48387 |
| 15151 | S19_29134465 | 19 | 29134465 | 137 | C | T | T | 0.69355 | C | 0.30645 | 15 | 0.48387 |
| 15152 | S19_29359450 | 19 | 29359450 | 224985 | C | T | C | 0.87097 | T | 0.12903 | 8 | 0.25806 |
| 15153 | S19_29880941 | 19 | 29880941 | 521491 | G | A | G | 0.87097 | A | 0.12903 | 8 | 0.25806 |
| 15154 | S19_29881042 | 19 | 29881042 | 101 | G | A | G | 0.90323 | A | 0.09677 | 6 | 0.19355 |
| 15155 | S19_29936479 | 19 | 29936479 | 55437 | T | C | T | 0.91935 | C | 0.08065 | 5 | 0.16129 |
| 15156 | S19_29991804 | 19 | 29991804 | 55325 | T | C | T | 0.67742 | C | 0.32258 | 12 | 0.3871 |
| 15157 | S19_29991831 | 19 | 29991831 | 27 | G | A | G | 0.8871 | A | 0.1129 | 7 | 0.22581 |
| 15158 | S19_29991925 | 19 | 29991925 | 94 | G | A | G | 0.93548 | A | 0.06452 | 4 | 0.12903 |
| 15159 | S19_29991942 | 19 | 29991942 | 17 | C | T | C | 0.93548 | T | 0.06452 | 4 | 0.12903 |
| 15160 | S19_29991943 | 19 | 29991943 | 1 | G | A | G | 0.83871 | A | 0.16129 | 8 | 0.25806 |
| 15161 | S19_29991959 | 19 | 29991959 | 16 | C | G | C | 0.69355 | G | 0.30645 | 11 | 0.35484 |
| 15162 | S19_29995641 | 19 | 29995641 | 3682 | C | T | C | 0.83871 | T | 0.16129 | 10 | 0.32258 |
| 15163 | S19_30045233 | 19 | 30045233 | 49592 | C | A | C | 0.85484 | A | 0.14516 | 9 | 0.29032 |
| 15164 | S19_30045268 | 19 | 30045268 | 35 | C | T | C | 0.83871 | T | 0.16129 | 10 | 0.32258 |
| 15165 | S19_30045331 | 19 | 30045331 | 63 | G | A | G | 0.91935 | A | 0.08065 | 5 | 0.16129 |
| 15166 | S19_30045434 | 19 | 30045434 | 103 | T | A | T | 0.85484 | A | 0.14516 | 9 | 0.29032 |
| 15167 | S19_30302088 | 19 | 30302088 | 256654 | T | C | C | 0.83871 | T | 0.16129 | 10 | 0.32258 |
| 15168 | S19_30302226 | 19 | 30302226 | 138 | G | A | G | 0.69355 | A | 0.30645 | 15 | 0.48387 |
| 15169 | S19_30631952 | 19 | 30631952 | 329726 | C | T | C | 0.77419 | T | 0.22581 | 12 | 0.3871 |
| 15170 | S19_30631983 | 19 | 30631983 | 31 | G | A | G | 0.6129 | A | 0.3871 | 16 | 0.51613 |
| 15171 | S19_30864705 | 19 | 30864705 | 232722 | G | C | C | 0.51613 | G | 0.48387 | 14 | 0.45161 |
| 15172 | S19_30864740 | 19 | 30864740 | 35 | A | T | A | 0.8871 | T | 0.1129 | 7 | 0.22581 |

| Site number | SNP name | Chromosome | Physical position | Physical distance from the previous SNP | Reference allele | Alternative allele | Major allele | Major allele frequency | Minor allele | Minor allele frequency | Number heterozygous | Heterozygosity |
|---|---|---|---|---|---|---|---|---|---|---|---|---|
| 15173 | S19_31258868 | 19 | 31258868 | 394128 | C | T | C | 0.85484 | T | 0.14516 | 9 | 0.29032 |
| 15174 | S19_31258919 | 19 | 31258919 | 51 | G | A | G | 0.67742 | A | 0.32258 | 14 | 0.45161 |
| 15175 | S19_31487843 | 19 | 31487843 | 228924 | G | A | G | 0.82258 | A | 0.17742 | 7 | 0.22581 |
| 15176 | S19_31597089 | 19 | 31597089 | 109246 | G | A | G | 0.95161 | A | 0.04839 | 3 | 0.09677 |
| 15177 | S19_31684664 | 19 | 31684664 | 87575 | T | C | T | 0.91935 | C | 0.08065 | 5 | 0.16129 |
| 15178 | S19_31684846 | 19 | 31684846 | 182 | A | G | A | 0.8871 | G | 0.1129 | 5 | 0.16129 |
| 15179 | S19_31934401 | 19 | 31934401 | 249555 | G | A | G | 0.95161 | A | 0.04839 | 3 | 0.09677 |
| 15180 | S19_32050697 | 19 | 32050697 | 116296 | T | C | T | 0.93548 | C | 0.06452 | 4 | 0.12903 |
| 15181 | S19_32050726 | 19 | 32050726 | 29 | T | A | T | 0.93548 | A | 0.06452 | 4 | 0.12903 |
| 15182 | S19_32050803 | 19 | 32050803 | 77 | C | T | C | 0.93548 | T | 0.06452 | 4 | 0.12903 |
| 15183 | S19_32050824 | 19 | 32050824 | 21 | T | A | T | 0.93548 | A | 0.06452 | 4 | 0.12903 |
| 15184 | S19_32132554 | 19 | 32132554 | 81730 | C | T | C | 0.79032 | T | 0.20968 | 9 | 0.29032 |
| 15185 | S19_32132556 | 19 | 32132556 | 2 | G | T | G | 0.80645 | T | 0.19355 | 10 | 0.32258 |
| 15186 | S19_32275677 | 19 | 32275677 | 143121 | C | T | C | 0.91935 | T | 0.08065 | 5 | 0.16129 |
| 15187 | S19_32275725 | 19 | 32275725 | 48 | G | A | G | 0.82258 | A | 0.17742 | 7 | 0.22581 |
| 15188 | S19_32275741 | 19 | 32275741 | 16 | C | T | C | 0.93548 | T | 0.06452 | 4 | 0.12903 |
| 15189 | S19_32462488 | 19 | 32462488 | 186747 | G | A | G | 0.82258 | A | 0.17742 | 7 | 0.22581 |
| 15190 | S19_32462535 | 19 | 32462535 | 47 | G | A | G | 0.82258 | A | 0.17742 | 7 | 0.22581 |
| 15191 | S19_32462562 | 19 | 32462562 | 27 | T | C | T | 0.90323 | C | 0.09677 | 4 | 0.12903 |
| 15192 | S19_32868009 | 19 | 32868009 | 405447 | G | A | G | 0.69355 | A | 0.30645 | 15 | 0.48387 |
| 15193 | S19_32868027 | 19 | 32868027 | 18 | G | A | G | 0.90323 | A | 0.09677 | 6 | 0.19355 |
| 15194 | S19_32868035 | 19 | 32868035 | 8 | C | T | C | 0.90323 | T | 0.09677 | 4 | 0.12903 |
| 15195 | S19_32868206 | 19 | 32868206 | 171 | G | T | G | 0.90323 | T | 0.09677 | 6 | 0.19355 |
| 15196 | S19_33104959 | 19 | 33104959 | 236753 | C | T | C | 0.95161 | T | 0.04839 | 3 | 0.09677 |
| 15197 | S19_33388109 | 19 | 33388109 | 283150 | T | G | T | 0.85484 | G | 0.14516 | 5 | 0.16129 |
| 15198 | S19_33388274 | 19 | 33388274 | 165 | A | C | A | 0.51613 | C | 0.48387 | 16 | 0.51613 |
| 15199 | S19_33452760 | 19 | 33452760 | 64486 | C | G | C | 0.93548 | G | 0.06452 | 4 | 0.12903 |
| 15200 | S19_33452871 | 19 | 33452871 | 111 | A | G | A | 0.64516 | G | 0.35484 | 10 | 0.32258 |
| 15201 | S19_34105682 | 19 | 34105682 | 652811 | C | T | C | 0.75806 | T | 0.24194 | 9 | 0.29032 |
| 15202 | S19_35074396 | 19 | 35074396 | 968714 | C | T | C | 0.79032 | T | 0.20968 | 11 | 0.35484 |
| 15203 | S19_35135421 | 19 | 35135421 | 61025 | C | T | C | 0.95161 | T | 0.04839 | 3 | 0.09677 |
| 15204 | S19_35135571 | 19 | 35135571 | 150 | G | A | G | 0.80645 | A | 0.19355 | 8 | 0.25806 |
| 15205 | S19_35531901 | 19 | 35531901 | 396330 | G | A | G | 0.72581 | A | 0.27419 | 11 | 0.35484 |
| 15206 | S19_35531979 | 19 | 35531979 | 78 | C | G | C | 0.91935 | G | 0.08065 | 5 | 0.16129 |
| 15207 | S19_35632515 | 19 | 35632515 | 100536 | C | T | C | 0.95161 | T | 0.04839 | 3 | 0.09677 |
| 15208 | S19_35856064 | 19 | 35856064 | 223549 | A | G | A | 0.85484 | G | 0.14516 | 7 | 0.22581 |
| 15209 | S19_36101595 | 19 | 36101595 | 245531 | G | A | G | 0.91935 | A | 0.08065 | 5 | 0.16129 |
| 15210 | S19_36101737 | 19 | 36101737 | 142 | C | T | C | 0.87097 | T | 0.12903 | 6 | 0.19355 |
| 15211 | S19_36775086 | 19 | 36775086 | 673349 | T | G | T | 0.93548 | G | 0.06452 | 4 | 0.12903 |
| 15212 | S19_36775234 | 19 | 36775234 | 148 | G | A | G | 0.8871 | A | 0.1129 | 5 | 0.16129 |
| 15213 | S19_37007013 | 19 | 37007013 | 231779 | T | C | T | 0.91935 | C | 0.08065 | 5 | 0.16129 |
| 15214 | S19_37014982 | 19 | 37014982 | 7969 | C | T | C | 0.91935 | T | 0.08065 | 5 | 0.16129 |
| 15215 | S19_37169230 | 19 | 37169230 | 154248 | C | T | T | 0.62903 | C | 0.37097 | 15 | 0.48387 |
| 15216 | S19_37217149 | 19 | 37217149 | 47919 | G | A | A | 0.85484 | G | 0.14516 | 9 | 0.29032 |
| 15217 | S19_37274474 | 19 | 37274474 | 57325 | C | T | C | 0.91935 | T | 0.08065 | 5 | 0.16129 |
| 15218 | S19_37298798 | 19 | 37298798 | 24324 | C | T | C | 0.93548 | T | 0.06452 | 4 | 0.12903 |
| 15219 | S19_37938098 | 19 | 37938098 | 639300 | A | G | A | 0.6129 | G | 0.3871 | 20 | 0.64516 |
| 15220 | S19_38159655 | 19 | 38159655 | 221557 | G | A | G | 0.79032 | A | 0.20968 | 13 | 0.41935 |
| 15221 | S19_38159809 | 19 | 38159809 | 154 | G | A | G | 0.85484 | A | 0.14516 | 7 | 0.22581 |
| 15222 | S19_38204696 | 19 | 38204696 | 44887 | C | T | C | 0.77419 | T | 0.22581 | 12 | 0.3871 |
| 15223 | S19_38416693 | 19 | 38416693 | 211997 | G | A | G | 0.91935 | A | 0.08065 | 3 | 0.09677 |
| 15224 | S19_38796831 | 19 | 38796831 | 380138 | C | T | C | 0.93548 | T | 0.06452 | 4 | 0.12903 |
| 15225 | S19_39466468 | 19 | 39466468 | 669637 | A | G | A | 0.77419 | G | 0.22581 | 12 | 0.3871 |
| 15226 | S19_39755326 | 19 | 39755326 | 288858 | G | A | G | 0.83871 | A | 0.16129 | 10 | 0.32258 |

| Site number | SNP name | Chromosome | Physical position | Physical distance from the previous SNP | Reference allele | Alternative allele | Major allele | Major allele frequency | Minor allele | Minor allele frequency | Number heterozygous | Heterozygosity |
|---|---|---|---|---|---|---|---|---|---|---|---|---|
| 15227 | S19_39755328 | 19 | 39755328 | 2 | G | C | G | 0.93548 | C | 0.06452 | 4 | 0.12903 |
| 15228 | S19_39755448 | 19 | 39755448 | 120 | C | T | C | 0.93548 | T | 0.06452 | 4 | 0.12903 |
| 15229 | S19_39755525 | 19 | 39755525 | 77 | A | G | A | 0.79032 | G | 0.20968 | 13 | 0.41935 |
| 15230 | S19_40103626 | 19 | 40103626 | 348101 | A | G | A | 0.8871 | G | 0.1129 | 7 | 0.22581 |
| 15231 | S19_40188226 | 19 | 40188226 | 84600 | A | G | G | 0.91935 | A | 0.08065 | 5 | 0.16129 |
| 15232 | S19_40755992 | 19 | 40755992 | 567766 | T | C | T | 0.80645 | C | 0.19355 | 12 | 0.3871 |
| 15233 | S19_41114569 | 19 | 41114569 | 358577 | C | A | C | 0.70968 | A | 0.29032 | 14 | 0.45161 |
| 15234 | S19_41114582 | 19 | 41114582 | 13 | C | T | C | 0.75806 | T | 0.24194 | 11 | 0.35484 |
| 15235 | S19_41114621 | 19 | 41114621 | 39 | T | C | T | 0.80645 | C | 0.19355 | 12 | 0.3871 |
| 15236 | S19_41114653 | 19 | 41114653 | 32 | G | A | G | 0.91935 | A | 0.08065 | 5 | 0.16129 |
| 15237 | S19_41114702 | 19 | 41114702 | 49 | G | T | G | 0.93548 | T | 0.06452 | 4 | 0.12903 |
| 15238 | S19_41114738 | 19 | 41114738 | 36 | C | T | C | 0.79032 | T | 0.20968 | 13 | 0.41935 |
| 15239 | S19_41114780 | 19 | 41114780 | 42 | C | T | T | 0.66129 | C | 0.33871 | 19 | 0.6129 |
| 15240 | S19_41114788 | 19 | 41114788 | 8 | A | T | A | 0.82258 | T | 0.17742 | 11 | 0.35484 |
| 15241 | S19_41159500 | 19 | 41159500 | 44712 | A | G | A | 0.93548 | G | 0.06452 | 2 | 0.06452 |
| 15242 | S19_41228615 | 19 | 41228615 | 69115 | C | T | C | 0.74194 | T | 0.25806 | 10 | 0.32258 |
| 15243 | S19_41228670 | 19 | 41228670 | 55 | T | C | T | 0.74194 | C | 0.25806 | 14 | 0.45161 |
| 15244 | S19_41705598 | 19 | 41705598 | 476928 | C | A | C | 0.83871 | A | 0.16129 | 10 | 0.32258 |
| 15245 | S19_41757435 | 19 | 41757435 | 51837 | T | C | C | 0.80645 | T | 0.19355 | 12 | 0.3871 |
| 15246 | S19_41785292 | 19 | 41785292 | 27857 | G | A | G | 0.62903 | A | 0.37097 | 17 | 0.54839 |
| 15247 | S19_42248528 | 19 | 42248528 | 463236 | G | A | G | 0.93548 | A | 0.06452 | 4 | 0.12903 |
| 15248 | S19_42348010 | 19 | 42348010 | 99482 | A | G | A | 0.8871 | G | 0.1129 | 7 | 0.22581 |
| 15249 | S19_42348037 | 19 | 42348037 | 27 | G | A | G | 0.93548 | A | 0.06452 | 4 | 0.12903 |
| 15250 | S19_42348242 | 19 | 42348242 | 205 | T | C | T | 0.6129 | C | 0.3871 | 12 | 0.3871 |
| 15251 | S19_42440380 | 19 | 42440380 | 92138 | G | A | G | 0.95161 | A | 0.04839 | 3 | 0.09677 |
| 15252 | S19_42440409 | 19 | 42440409 | 29 | G | A | G | 0.70968 | A | 0.29032 | 16 | 0.51613 |
| 15253 | S19_42750349 | 19 | 42750349 | 309940 | C | T | C | 0.93548 | T | 0.06452 | 4 | 0.12903 |
| 15254 | S19_42750382 | 19 | 42750382 | 33 | G | A | G | 0.93548 | A | 0.06452 | 4 | 0.12903 |
| 15255 | S19_42932988 | 19 | 42932988 | 182606 | T | A | T | 0.79032 | A | 0.20968 | 13 | 0.41935 |
| 15256 | S19_42933028 | 19 | 42933028 | 40 | A | G | A | 0.82258 | G | 0.17742 | 11 | 0.35484 |
| 15257 | S19_42934797 | 19 | 42934797 | 1769 | T | C | T | 0.75806 | C | 0.24194 | 15 | 0.48387 |
| 15258 | S19_42947536 | 19 | 42947536 | 12739 | A | T | T | 0.56452 | A | 0.43548 | 15 | 0.48387 |
| 15259 | S19_43014683 | 19 | 43014683 | 67147 | T | G | T | 0.74194 | G | 0.25806 | 14 | 0.45161 |
| 15260 | S19_43014753 | 19 | 43014753 | 70 | G | C | G | 0.93548 | C | 0.06452 | 4 | 0.12903 |
| 15261 | S19_43027624 | 19 | 43027624 | 12871 | G | A | G | 0.54839 | A | 0.45161 | 20 | 0.64516 |
| 15262 | S19_43027629 | 19 | 43027629 | 5 | A | T | A | 0.59677 | T | 0.40323 | 19 | 0.6129 |
| 15263 | S19_43027685 | 19 | 43027685 | 56 | C | T | C | 0.91935 | T | 0.08065 | 3 | 0.09677 |
| 15264 | S19_43179127 | 19 | 43179127 | 151442 | G | T | G | 0.93548 | T | 0.06452 | 4 | 0.12903 |
| 15265 | S19_43179179 | 19 | 43179179 | 52 | T | C | T | 0.82258 | C | 0.17742 | 9 | 0.29032 |
| 15266 | S19_43179267 | 19 | 43179267 | 88 | G | A | G | 0.64516 | A | 0.35484 | 16 | 0.51613 |
| 15267 | S19_43335441 | 19 | 43335441 | 156174 | C | T | C | 0.79032 | T | 0.20968 | 9 | 0.29032 |
| 15268 | S19_43335507 | 19 | 43335507 | 66 | T | A | T | 0.74194 | A | 0.25806 | 10 | 0.32258 |
| 15269 | S19_43349128 | 19 | 43349128 | 13621 | G | A | G | 0.82258 | A | 0.17742 | 7 | 0.22581 |
| 15270 | S19_43349222 | 19 | 43349222 | 94 | A | C | A | 0.91935 | C | 0.08065 | 5 | 0.16129 |
| 15271 | S19_43349257 | 19 | 43349257 | 35 | T | C | T | 0.80645 | C | 0.19355 | 8 | 0.25806 |
| 15272 | S19_43415690 | 19 | 43415690 | 66433 | C | T | C | 0.83871 | T | 0.16129 | 10 | 0.32258 |
| 15273 | S19_43930647 | 19 | 43930647 | 514957 | C | T | C | 0.90323 | T | 0.09677 | 4 | 0.12903 |
| 15274 | S19_43930842 | 19 | 43930842 | 195 | T | C | C | 0.87097 | T | 0.12903 | 8 | 0.25806 |
| 15275 | S19_44163205 | 19 | 44163205 | 232363 | C | T | C | 0.8871 | T | 0.1129 | 7 | 0.22581 |
| 15276 | S19_44682098 | 19 | 44682098 | 518893 | T | A | T | 0.91935 | A | 0.08065 | 5 | 0.16129 |
| 15277 | S19_44682151 | 19 | 44682151 | 53 | C | T | C | 0.87097 | T | 0.12903 | 8 | 0.25806 |
| 15278 | S19_44682154 | 19 | 44682154 | 3 | C | T | C | 0.87097 | T | 0.12903 | 8 | 0.25806 |
| 15279 | S19_44958101 | 19 | 44958101 | 275947 | G | C | G | 0.95161 | C | 0.04839 | 3 | 0.09677 |
| 15280 | S19_45211686 | 19 | 45211686 | 253585 | C | T | C | 0.90323 | T | 0.09677 | 6 | 0.19355 |

| Site number | SNP name | Chromosome | Physical position | Physical distance from the previous SNP | Reference allele | Alternative allele | Major allele | Major allele frequency | Minor allele | Minor allele frequency | Number heterozygous | Heterozygosity |
|---|---|---|---|---|---|---|---|---|---|---|---|---|
| 15281 | S19_45493432 | 19 | 45493432 | 281746 | C | T | C | 0.93548 | T | 0.06452 | 4 | 0.12903 |
| 15282 | S19_45493439 | 19 | 45493439 | 7 | C | T | C | 0.8871 | T | 0.1129 | 7 | 0.22581 |
| 15283 | S19_45493449 | 19 | 45493449 | 10 | G | A | G | 0.90323 | A | 0.09677 | 6 | 0.19355 |
| 15284 | S19_45493489 | 19 | 45493489 | 40 | C | T | C | 0.90323 | T | 0.09677 | 6 | 0.19355 |
| 15285 | S19_45493635 | 19 | 45493635 | 146 | C | T | C | 0.93548 | T | 0.06452 | 4 | 0.12903 |
| 15286 | S19_46715100 | 19 | 46715100 | 1221465 | A | G | G | 0.53226 | A | 0.46774 | 13 | 0.41935 |
| 15287 | S19_46715176 | 19 | 46715176 | 76 | A | G | A | 0.82258 | G | 0.17742 | 9 | 0.29032 |
| 15288 | S19_46790623 | 19 | 46790623 | 75447 | T | C | T | 0.93548 | C | 0.06452 | 4 | 0.12903 |
| 15289 | S19_46790695 | 19 | 46790695 | 72 | G | A | G | 0.90323 | A | 0.09677 | 6 | 0.19355 |
| 15290 | S19_46790729 | 19 | 46790729 | 34 | G | C | C | 0.67742 | G | 0.32258 | 16 | 0.51613 |
| 15291 | S19_47355683 | 19 | 47355683 | 564954 | G | A | G | 0.85484 | A | 0.14516 | 9 | 0.29032 |
| 15292 | S19_47355715 | 19 | 47355715 | 32 | C | T | C | 0.91935 | T | 0.08065 | 5 | 0.16129 |
| 15293 | S19_47355737 | 19 | 47355737 | 22 | G | C | G | 0.8871 | C | 0.1129 | 7 | 0.22581 |
| 15294 | S19_47927096 | 19 | 47927096 | 571359 | T | C | T | 0.80645 | C | 0.19355 | 10 | 0.32258 |
| 15295 | S19_48018072 | 19 | 48018072 | 90976 | G | A | G | 0.72581 | A | 0.27419 | 15 | 0.48387 |
| 15296 | S19_48018191 | 19 | 48018191 | 119 | A | C | C | 0.82258 | A | 0.17742 | 11 | 0.35484 |
| 15297 | S19_48087969 | 19 | 48087969 | 69778 | G | A | G | 0.67742 | A | 0.32258 | 14 | 0.45161 |
| 15298 | S19_48087991 | 19 | 48087991 | 22 | C | T | C | 0.93548 | T | 0.06452 | 4 | 0.12903 |
| 15299 | S19_48088075 | 19 | 48088075 | 84 | A | C | A | 0.93548 | C | 0.06452 | 4 | 0.12903 |
| 15300 | S19_48098886 | 19 | 48098886 | 10811 | G | A | G | 0.85484 | A | 0.14516 | 9 | 0.29032 |
| 15301 | S19_48098893 | 19 | 48098893 | 7 | A | G | A | 0.67742 | G | 0.32258 | 14 | 0.45161 |
| 15302 | S19_48175909 | 19 | 48175909 | 77016 | G | A | G | 0.90323 | A | 0.09677 | 6 | 0.19355 |
| 15303 | S19_48175926 | 19 | 48175926 | 17 | C | T | T | 0.62903 | C | 0.37097 | 11 | 0.35484 |
| 15304 | S19_48175943 | 19 | 48175943 | 17 | T | C | T | 0.62903 | C | 0.37097 | 13 | 0.41935 |
| 15305 | S19_48176085 | 19 | 48176085 | 142 | G | A | G | 0.62903 | A | 0.37097 | 13 | 0.41935 |
| 15306 | S19_48176089 | 19 | 48176089 | 4 | G | A | G | 0.82258 | A | 0.17742 | 9 | 0.29032 |
| 15307 | S19_48176130 | 19 | 48176130 | 41 | C | T | C | 0.79032 | T | 0.20968 | 11 | 0.35484 |
| 15308 | S19_48608228 | 19 | 48608228 | 432098 | G | C | G | 0.8871 | C | 0.1129 | 7 | 0.22581 |
| 15309 | S19_48608450 | 19 | 48608450 | 222 | T | C | T | 0.87097 | C | 0.12903 | 8 | 0.25806 |
| 15310 | S19_48663335 | 19 | 48663335 | 54885 | G | A | A | 0.51613 | G | 0.48387 | 10 | 0.32258 |
| 15311 | S19_48663394 | 19 | 48663394 | 59 | A | G | G | 0.51613 | A | 0.48387 | 10 | 0.32258 |
| 15312 | S19_49046760 | 19 | 49046760 | 383366 | C | T | C | 0.70968 | T | 0.29032 | 10 | 0.32258 |
| 15313 | S19_49046780 | 19 | 49046780 | 20 | C | T | C | 0.70968 | T | 0.29032 | 10 | 0.32258 |
| 15314 | S19_49080667 | 19 | 49080667 | 33887 | G | A | G | 0.80645 | A | 0.19355 | 10 | 0.32258 |
| 15315 | S19_49177655 | 19 | 49177655 | 96988 | G | A | A | 0.54839 | G | 0.45161 | 14 | 0.45161 |
| 15316 | S19_49177863 | 19 | 49177863 | 208 | T | C | T | 0.85484 | C | 0.14516 | 9 | 0.29032 |
| 15317 | S19_49314816 | 19 | 49314816 | 136953 | G | A | A | 0.91935 | G | 0.08065 | 5 | 0.16129 |
| 15318 | S19_49486801 | 19 | 49486801 | 171985 | T | C | C | 0.91935 | T | 0.08065 | 5 | 0.16129 |
| 15319 | S19_49486955 | 19 | 49486955 | 154 | G | A | G | 0.91935 | A | 0.08065 | 5 | 0.16129 |
| 15320 | S19_49762023 | 19 | 49762023 | 275068 | G | A | G | 0.79032 | A | 0.20968 | 9 | 0.29032 |
| 15321 | S19_49762185 | 19 | 49762185 | 162 | A | C | A | 0.69355 | C | 0.30645 | 11 | 0.35484 |
| 15322 | S19_50058239 | 19 | 50058239 | 296054 | A | G | A | 0.80645 | G | 0.19355 | 12 | 0.3871 |
| 15323 | S19_50058334 | 19 | 50058334 | 95 | T | C | T | 0.85484 | C | 0.14516 | 9 | 0.29032 |
| 15324 | S19_51094235 | 19 | 51094235 | 1035901 | A | G | G | 0.72581 | A | 0.27419 | 13 | 0.41935 |
| 15325 | S19_51678438 | 19 | 51678438 | 584203 | G | A | G | 0.6129 | A | 0.3871 | 14 | 0.45161 |
| 15326 | S19_52079073 | 19 | 52079073 | 400635 | C | T | T | 0.77419 | C | 0.22581 | 12 | 0.3871 |
| 15327 | S19_52079078 | 19 | 52079078 | 5 | C | T | T | 0.77419 | C | 0.22581 | 12 | 0.3871 |
| 15328 | S19_52079083 | 19 | 52079083 | 5 | G | A | A | 0.77419 | G | 0.22581 | 12 | 0.3871 |
| 15329 | S19_52079085 | 19 | 52079085 | 2 | A | G | G | 0.77419 | A | 0.22581 | 12 | 0.3871 |
| 15330 | S19_52079115 | 19 | 52079115 | 30 | T | C | T | 0.87097 | C | 0.12903 | 8 | 0.25806 |
| 15331 | S19_52079127 | 19 | 52079127 | 12 | A | T | T | 0.72581 | A | 0.27419 | 11 | 0.35484 |
| 15332 | S19_52079133 | 19 | 52079133 | 6 | T | C | C | 0.77419 | T | 0.22581 | 12 | 0.3871 |
| 15333 | S19_52079170 | 19 | 52079170 | 37 | C | T | T | 0.77419 | C | 0.22581 | 12 | 0.3871 |
| 15334 | S19_52079184 | 19 | 52079184 | 14 | T | C | C | 0.77419 | T | 0.22581 | 12 | 0.3871 |

| Site number | SNP name | Chromosome | Physical position | Physical distance from the previous SNP | Reference allele | Alternative allele | Major allele | Major allele frequency | Minor allele | Minor allele frequency | Number heterozygous | Heterozygosity |
|---|---|---|---|---|---|---|---|---|---|---|---|---|
| 15335 | S19_52467096 | 19 | 52467096 | 387912 | G | A | G | 0.82258 | A | 0.17742 | 7 | 0.22581 |
| 15336 | S19_52467328 | 19 | 52467328 | 232 | C | T | C | 0.85484 | T | 0.14516 | 9 | 0.29032 |
| 15337 | S19_52467335 | 19 | 52467335 | 7 | A | G | G | 0.72581 | A | 0.27419 | 15 | 0.48387 |
| 15338 | S19_52467353 | 19 | 52467353 | 18 | G | A | A | 0.72581 | G | 0.27419 | 15 | 0.48387 |
| 15339 | S19_52467442 | 19 | 52467442 | 89 | G | A | G | 0.85484 | A | 0.14516 | 9 | 0.29032 |
| 15340 | S19_52623530 | 19 | 52623530 | 156088 | A | G | A | 0.77419 | G | 0.22581 | 8 | 0.25806 |
| 15341 | S19_52812315 | 19 | 52812315 | 188785 | G | A | A | 0.67742 | G | 0.32258 | 12 | 0.3871 |
| 15342 | S19_53018876 | 19 | 53018876 | 206561 | C | T | C | 0.8871 | T | 0.1129 | 7 | 0.22581 |
| 15343 | S19_53182632 | 19 | 53182632 | 163756 | C | T | C | 0.83871 | T | 0.16129 | 10 | 0.32258 |
| 15344 | S19_53182706 | 19 | 53182706 | 74 | A | G | A | 0.90323 | G | 0.09677 | 6 | 0.19355 |
| 15345 | S19_53182735 | 19 | 53182735 | 29 | T | C | T | 0.77419 | C | 0.22581 | 14 | 0.45161 |
| 15346 | S19_53319800 | 19 | 53319800 | 137065 | C | T | C | 0.74194 | T | 0.25806 | 10 | 0.32258 |
| 15347 | S19_53319805 | 19 | 53319805 | 5 | C | T | C | 0.91935 | T | 0.08065 | 5 | 0.16129 |
| 15348 | S19_53445347 | 19 | 53445347 | 125542 | G | A | A | 0.82258 | G | 0.17742 | 11 | 0.35484 |
| 15349 | S19_53445350 | 19 | 53445350 | 3 | C | G | C | 0.59677 | G | 0.40323 | 15 | 0.48387 |
| 15350 | S19_53445440 | 19 | 53445440 | 90 | T | C | C | 0.85484 | T | 0.14516 | 9 | 0.29032 |
| 15351 | S19_53544467 | 19 | 53544467 | 99027 | G | A | A | 0.90323 | G | 0.09677 | 6 | 0.19355 |
| 15352 | S19_53575592 | 19 | 53575592 | 31125 | C | T | C | 0.95161 | T | 0.04839 | 3 | 0.09677 |
| 15353 | S19_53575603 | 19 | 53575603 | 11 | A | G | A | 0.90323 | G | 0.09677 | 4 | 0.12903 |
| 15354 | S19_53575724 | 19 | 53575724 | 121 | T | C | T | 0.80645 | C | 0.19355 | 12 | 0.3871 |
| 15355 | S19_53701050 | 19 | 53701050 | 125326 | A | G | G | 0.53226 | A | 0.46774 | 19 | 0.6129 |
| 15356 | S19_54469375 | 19 | 54469375 | 768325 | C | T | C | 0.72581 | T | 0.27419 | 13 | 0.41935 |
| 15357 | S19_54469560 | 19 | 54469560 | 185 | G | C | G | 0.72581 | C | 0.27419 | 13 | 0.41935 |
| 15358 | S19_54519668 | 19 | 54519668 | 50108 | C | G | C | 0.93548 | G | 0.06452 | 4 | 0.12903 |
| 15359 | S19_54789114 | 19 | 54789114 | 269446 | T | C | T | 0.69355 | C | 0.30645 | 13 | 0.41935 |
| 15360 | S19_54789143 | 19 | 54789143 | 29 | G | T | G | 0.54839 | T | 0.45161 | 18 | 0.58065 |
| 15361 | S19_55539399 | 19 | 55539399 | 750256 | A | G | G | 0.8871 | A | 0.1129 | 7 | 0.22581 |
| 15362 | S19_55843484 | 19 | 55843484 | 304085 | T | C | T | 0.72581 | C | 0.27419 | 11 | 0.35484 |
| 15363 | S19_56149340 | 19 | 56149340 | 305856 | C | T | C | 0.95161 | T | 0.04839 | 3 | 0.09677 |
| 15364 | S19_56149498 | 19 | 56149498 | 158 | G | A | G | 0.75806 | A | 0.24194 | 9 | 0.29032 |
| 15365 | S19_56149554 | 19 | 56149554 | 56 | T | C | T | 0.93548 | C | 0.06452 | 4 | 0.12903 |
| 15366 | S19_56407991 | 19 | 56407991 | 258437 | T | C | T | 0.8871 | C | 0.1129 | 5 | 0.16129 |
| 15367 | S19_56408019 | 19 | 56408019 | 28 | T | C | T | 0.83871 | C | 0.16129 | 10 | 0.32258 |
| 15368 | S19_56408024 | 19 | 56408024 | 5 | C | T | C | 0.75806 | T | 0.24194 | 11 | 0.35484 |
| 15369 | S19_56408025 | 19 | 56408025 | 1 | G | C | G | 0.91935 | C | 0.08065 | 5 | 0.16129 |
| 15370 | S19_56408026 | 19 | 56408026 | 1 | T | C | T | 0.91935 | C | 0.08065 | 5 | 0.16129 |
| 15371 | S19_56408037 | 19 | 56408037 | 11 | G | A | G | 0.91935 | A | 0.08065 | 5 | 0.16129 |
| 15372 | S19_56408044 | 19 | 56408044 | 7 | T | C | T | 0.91935 | C | 0.08065 | 5 | 0.16129 |
| 15373 | S19_56408055 | 19 | 56408055 | 11 | C | A | C | 0.91935 | A | 0.08065 | 5 | 0.16129 |
| 15374 | S19_56408094 | 19 | 56408094 | 39 | A | G | A | 0.91935 | G | 0.08065 | 5 | 0.16129 |
| 15375 | S19_56541051 | 19 | 56541051 | 132957 | T | C | C | 0.67742 | T | 0.32258 | 10 | 0.32258 |
| 15376 | S19_56541099 | 19 | 56541099 | 48 | C | A | A | 0.67742 | C | 0.32258 | 10 | 0.32258 |
| 15377 | S19_56542452 | 19 | 56542452 | 1353 | G | C | C | 0.69355 | G | 0.30645 | 9 | 0.29032 |
| 15378 | S19_56672707 | 19 | 56672707 | 130255 | T | A | A | 0.74194 | T | 0.25806 | 14 | 0.45161 |
| 15379 | S19_56672764 | 19 | 56672764 | 57 | C | T | C | 0.90323 | T | 0.09677 | 6 | 0.19355 |
| 15380 | S19_56672770 | 19 | 56672770 | 6 | C | A | C | 0.87097 | A | 0.12903 | 8 | 0.25806 |
| 15381 | S19_56672856 | 19 | 56672856 | 86 | A | C | A | 0.90323 | C | 0.09677 | 6 | 0.19355 |
| 15382 | S19_56672859 | 19 | 56672859 | 3 | G | A | G | 0.80645 | A | 0.19355 | 10 | 0.32258 |
| 15383 | S19_56672871 | 19 | 56672871 | 12 | A | C | A | 0.80645 | C | 0.19355 | 10 | 0.32258 |
| 15384 | S19_56672883 | 19 | 56672883 | 12 | G | T | G | 0.77419 | T | 0.22581 | 12 | 0.3871 |
| 15385 | S19_56680541 | 19 | 56680541 | 7658 | G | A | G | 0.75806 | A | 0.24194 | 13 | 0.41935 |
| 15386 | S19_56680596 | 19 | 56680596 | 55 | G | C | G | 0.66129 | C | 0.33871 | 15 | 0.48387 |
| 15387 | S19_56680656 | 19 | 56680656 | 60 | T | G | G | 0.66129 | T | 0.33871 | 17 | 0.54839 |
| 15388 | S19_56783833 | 19 | 56783833 | 103177 | G | C | G | 0.8871 | C | 0.1129 | 7 | 0.22581 |

| Site number | SNP name | Chromosome | Physical position | Physical distance from the previous SNP | Reference allele | Alternative allele | Major allele | Major allele frequency | Minor allele | Minor allele frequency | Number heterozygous | Heterozygosity |
|---|---|---|---|---|---|---|---|---|---|---|---|---|
| 15389 | S19_56802503 | 19 | 56802503 | 18670 | C | T | C | 0.91935 | T | 0.08065 | 5 | 0.16129 |
| 15390 | S19_56802519 | 19 | 56802519 | 16 | A | T | A | 0.93548 | T | 0.06452 | 4 | 0.12903 |
| 15391 | S19_56802561 | 19 | 56802561 | 42 | A | G | A | 0.62903 | G | 0.37097 | 19 | 0.6129 |
| 15392 | S19_57206770 | 19 | 57206770 | 404209 | T | C | C | 0.93548 | T | 0.06452 | 4 | 0.12903 |
| 15393 | S19_57355733 | 19 | 57355733 | 148963 | A | G | A | 0.70968 | G | 0.29032 | 12 | 0.3871 |
| 15394 | S19_57355749 | 19 | 57355749 | 16 | A | G | G | 0.75806 | A | 0.24194 | 9 | 0.29032 |
| 15395 | S19_57355801 | 19 | 57355801 | 52 | C | T | C | 0.70968 | T | 0.29032 | 12 | 0.3871 |
| 15396 | S19_57390952 | 19 | 57390952 | 35151 | G | A | G | 0.91935 | A | 0.08065 | 5 | 0.16129 |
| 15397 | S19_57391074 | 19 | 57391074 | 122 | C | T | C | 0.91935 | T | 0.08065 | 5 | 0.16129 |
| 15398 | S19_57391081 | 19 | 57391081 | 7 | A | G | A | 0.53226 | G | 0.46774 | 17 | 0.54839 |
| 15399 | S19_57391160 | 19 | 57391160 | 79 | T | G | T | 0.91935 | G | 0.08065 | 5 | 0.16129 |
| 15400 | S19_57394621 | 19 | 57394621 | 3461 | C | G | C | 0.74194 | G | 0.25806 | 12 | 0.3871 |
| 15401 | S19_57394649 | 19 | 57394649 | 28 | T | G | T | 0.8871 | G | 0.1129 | 7 | 0.22581 |
| 15402 | S19_57394652 | 19 | 57394652 | 3 | G | A | G | 0.74194 | A | 0.25806 | 12 | 0.3871 |
| 15403 | S19_57394675 | 19 | 57394675 | 23 | T | C | T | 0.74194 | C | 0.25806 | 12 | 0.3871 |
| 15404 | S19_57394693 | 19 | 57394693 | 18 | A | G | A | 0.79032 | G | 0.20968 | 13 | 0.41935 |
| 15405 | S19_57394710 | 19 | 57394710 | 17 | T | C | T | 0.66129 | C | 0.33871 | 15 | 0.48387 |
| 15406 | S19_57453470 | 19 | 57453470 | 58760 | G | T | G | 0.72581 | T | 0.27419 | 15 | 0.48387 |
| 15407 | S19_57614720 | 19 | 57614720 | 161250 | G | A | G | 0.91935 | A | 0.08065 | 5 | 0.16129 |
| 15408 | S19_57614764 | 19 | 57614764 | 44 | C | G | G | 0.53226 | C | 0.46774 | 13 | 0.41935 |
| 15409 | S19_57614775 | 19 | 57614775 | 11 | T | C | C | 0.53226 | T | 0.46774 | 13 | 0.41935 |
| 15410 | S19_57614776 | 19 | 57614776 | 1 | G | A | A | 0.53226 | G | 0.46774 | 13 | 0.41935 |
| 15411 | S19_57614795 | 19 | 57614795 | 19 | C | T | T | 0.53226 | C | 0.46774 | 13 | 0.41935 |
| 15412 | S19_57614826 | 19 | 57614826 | 31 | G | A | G | 0.90323 | A | 0.09677 | 6 | 0.19355 |
| 15413 | S19_57614831 | 19 | 57614831 | 5 | C | A | C | 0.56452 | A | 0.43548 | 15 | 0.48387 |
| 15414 | S19_57867917 | 19 | 57867917 | 253086 | A | G | A | 0.93548 | G | 0.06452 | 4 | 0.12903 |
| 15415 | S19_57868020 | 19 | 57868020 | 103 | C | T | T | 0.83871 | C | 0.16129 | 10 | 0.32258 |
| 15416 | S19_57868125 | 19 | 57868125 | 105 | G | T | T | 0.72581 | G | 0.27419 | 13 | 0.41935 |
| 15417 | S19_57870060 | 19 | 57870060 | 1935 | A | T | A | 0.80645 | T | 0.19355 | 8 | 0.25806 |
| 15418 | S19_58132135 | 19 | 58132135 | 262075 | C | T | C | 0.91935 | T | 0.08065 | 5 | 0.16129 |
| 15419 | S19_58132256 | 19 | 58132256 | 121 | G | A | A | 0.85484 | G | 0.14516 | 9 | 0.29032 |
| 15420 | S19_58132311 | 19 | 58132311 | 55 | T | G | T | 0.95161 | G | 0.04839 | 3 | 0.09677 |
| 15421 | S19_58136965 | 19 | 58136965 | 4654 | G | A | G | 0.82258 | A | 0.17742 | 7 | 0.22581 |
| 15422 | S19_58138584 | 19 | 58138584 | 1619 | T | G | T | 0.6129 | G | 0.3871 | 12 | 0.3871 |
| 15423 | S19_58138690 | 19 | 58138690 | 106 | G | A | G | 0.90323 | A | 0.09677 | 6 | 0.19355 |
| 15424 | S19_58273244 | 19 | 58273244 | 134554 | C | T | C | 0.91935 | T | 0.08065 | 5 | 0.16129 |
| 15425 | S19_58449795 | 19 | 58449795 | 176551 | C | A | C | 0.75806 | A | 0.24194 | 11 | 0.35484 |
| 15426 | S19_58666280 | 19 | 58666280 | 216485 | C | T | C | 0.5 | T | 0.5 | 15 | 0.48387 |
| 15427 | S19_58666334 | 19 | 58666334 | 54 | G | A | A | 0.5 | G | 0.5 | 15 | 0.48387 |
| 15428 | S19_58710932 | 19 | 58710932 | 44598 | G | C | C | 0.53226 | G | 0.46774 | 13 | 0.41935 |
| 15429 | S19_58711093 | 19 | 58711093 | 161 | G | A | A | 0.51613 | G | 0.48387 | 14 | 0.45161 |
| 15430 | S19_58951694 | 19 | 58951694 | 240601 | A | G | A | 0.85484 | G | 0.14516 | 7 | 0.22581 |
| 15431 | S19_58951726 | 19 | 58951726 | 32 | C | A | C | 0.82258 | A | 0.17742 | 9 | 0.29032 |
| 15432 | S19_58951901 | 19 | 58951901 | 175 | G | A | G | 0.80645 | A | 0.19355 | 10 | 0.32258 |
| 15433 | S19_59257324 | 19 | 59257324 | 305423 | C | T | C | 0.93548 | T | 0.06452 | 4 | 0.12903 |
| 15434 | S19_59524530 | 19 | 59524530 | 267206 | A | C | A | 0.74194 | C | 0.25806 | 10 | 0.32258 |
| 15435 | S19_59524542 | 19 | 59524542 | 12 | G | A | G | 0.56452 | A | 0.43548 | 17 | 0.54839 |
| 15436 | S19_59552883 | 19 | 59552883 | 28341 | T | G | G | 0.82258 | T | 0.17742 | 7 | 0.22581 |
| 15437 | S19_59552960 | 19 | 59552960 | 77 | A | G | A | 0.90323 | G | 0.09677 | 6 | 0.19355 |
| 15438 | S19_59668977 | 19 | 59668977 | 116017 | C | A | C | 0.93548 | A | 0.06452 | 4 | 0.12903 |
| 15439 | S19_59676284 | 19 | 59676284 | 7307 | T | C | T | 0.6129 | C | 0.3871 | 18 | 0.58065 |
| 15440 | S19_59676410 | 19 | 59676410 | 126 | G | C | G | 0.82258 | C | 0.17742 | 11 | 0.35484 |
| 15441 | S19_59730986 | 19 | 59730986 | 54576 | C | G | C | 0.90323 | G | 0.09677 | 6 | 0.19355 |
| 15442 | S19_59731187 | 19 | 59731187 | 201 | G | A | G | 0.70968 | A | 0.29032 | 8 | 0.25806 |

| Site number | SNP name | Chromosome | Physical position | Physical distance from the previous SNP | Reference allele | Alternative allele | Major allele | Major allele frequency | Minor allele | Minor allele frequency | Number heterozygous | Heterozygosity |
|---|---|---|---|---|---|---|---|---|---|---|---|---|
| 15443 | S19_59793221 | 19 | 59793221 | 62034 | A | C | A | 0.93548 | C | 0.06452 | 4 | 0.12903 |
| 15444 | S19_59793294 | 19 | 59793294 | 73 | G | A | G | 0.90323 | A | 0.09677 | 6 | 0.19355 |
| 15445 | S19_59793371 | 19 | 59793371 | 77 | G | T | G | 0.93548 | T | 0.06452 | 4 | 0.12903 |
| 15446 | S19_59793425 | 19 | 59793425 | 54 | A | C | A | 0.59677 | C | 0.40323 | 11 | 0.35484 |
| 15447 | S19_59793434 | 19 | 59793434 | 9 | G | C | G | 0.83871 | C | 0.16129 | 8 | 0.25806 |
| 15448 | S19_60115858 | 19 | 60115858 | 322424 | G | A | G | 0.72581 | A | 0.27419 | 11 | 0.35484 |
| 15449 | S19_60172722 | 19 | 60172722 | 56864 | C | T | T | 0.51613 | C | 0.48387 | 16 | 0.51613 |
| 15450 | S19_60172725 | 19 | 60172725 | 3 | C | T | C | 0.95161 | T | 0.04839 | 3 | 0.09677 |
| 15451 | S19_60172814 | 19 | 60172814 | 89 | G | A | G | 0.80645 | A | 0.19355 | 6 | 0.19355 |
| 15452 | S19_60327848 | 19 | 60327848 | 155034 | G | A | G | 0.6129 | A | 0.3871 | 14 | 0.45161 |
| 15453 | S19_60504551 | 19 | 60504551 | 176703 | G | A | G | 0.6129 | A | 0.3871 | 12 | 0.3871 |
| 15454 | S19_60504641 | 19 | 60504641 | 90 | C | T | C | 0.87097 | T | 0.12903 | 6 | 0.19355 |
| 15455 | S19_60504644 | 19 | 60504644 | 3 | G | A | G | 0.8871 | A | 0.1129 | 5 | 0.16129 |
| 15456 | S19_60530958 | 19 | 60530958 | 26314 | C | T | C | 0.80645 | T | 0.19355 | 12 | 0.3871 |
| 15457 | S19_61157612 | 19 | 61157612 | 626654 | G | A | G | 0.87097 | A | 0.12903 | 8 | 0.25806 |
| 15458 | S19_61157696 | 19 | 61157696 | 84 | C | T | C | 0.8871 | T | 0.1129 | 7 | 0.22581 |
| 15459 | S19_61157702 | 19 | 61157702 | 6 | C | T | C | 0.80645 | T | 0.19355 | 10 | 0.32258 |
| 15460 | S19_61157707 | 19 | 61157707 | 5 | T | C | C | 0.5 | T | 0.5 | 17 | 0.54839 |
| 15461 | S19_61157717 | 19 | 61157717 | 10 | T | C | C | 0.67742 | T | 0.32258 | 10 | 0.32258 |
| 15462 | S19_61157730 | 19 | 61157730 | 13 | A | G | A | 0.70968 | G | 0.29032 | 12 | 0.3871 |
| 15463 | S19_61157753 | 19 | 61157753 | 23 | A | G | G | 0.75806 | A | 0.24194 | 13 | 0.41935 |
| 15464 | S19_61170068 | 19 | 61170068 | 12315 | C | A | C | 0.93548 | A | 0.06452 | 4 | 0.12903 |
| 15465 | S19_61326177 | 19 | 61326177 | 156109 | C | T | C | 0.83871 | T | 0.16129 | 8 | 0.25806 |
| 15466 | S19_61543597 | 19 | 61543597 | 217420 | A | G | G | 0.93548 | A | 0.06452 | 4 | 0.12903 |
| 15467 | S19_61543622 | 19 | 61543622 | 25 | C | T | C | 0.95161 | T | 0.04839 | 3 | 0.09677 |
| 15468 | S19_61543646 | 19 | 61543646 | 24 | C | T | C | 0.91935 | T | 0.08065 | 5 | 0.16129 |
| 15469 | S19_61543665 | 19 | 61543665 | 19 | C | A | C | 0.87097 | A | 0.12903 | 6 | 0.19355 |
| 15470 | S19_61543804 | 19 | 61543804 | 139 | G | A | G | 0.85484 | A | 0.14516 | 7 | 0.22581 |
| 15471 | S19_62105114 | 19 | 62105114 | 561310 | T | C | T | 0.77419 | C | 0.22581 | 8 | 0.25806 |
| 15472 | S19_62105191 | 19 | 62105191 | 77 | C | T | C | 0.80645 | T | 0.19355 | 6 | 0.19355 |
| 15473 | S19_62105203 | 19 | 62105203 | 12 | T | C | C | 0.75806 | T | 0.24194 | 13 | 0.41935 |
| 15474 | S19_62171999 | 19 | 62171999 | 66796 | G | A | G | 0.91935 | A | 0.08065 | 5 | 0.16129 |
| 15475 | S19_62390487 | 19 | 62390487 | 218488 | G | T | T | 0.83871 | G | 0.16129 | 8 | 0.25806 |
| 15476 | S19_62491974 | 19 | 62491974 | 101487 | A | G | A | 0.79032 | G | 0.20968 | 9 | 0.29032 |
| 15477 | S19_62492081 | 19 | 62492081 | 107 | G | A | G | 0.79032 | A | 0.20968 | 9 | 0.29032 |
| 15478 | S20_61650 | 20 | 61650 | 0 | C | T | C | 0.95161 | T | 0.04839 | 3 | 0.09677 |
| 15479 | S20_577073 | 20 | 577073 | 515423 | A | G | A | 0.54839 | G | 0.45161 | 14 | 0.45161 |
| 15480 | S20_577094 | 20 | 577094 | 21 | A | C | C | 0.53226 | A | 0.46774 | 13 | 0.41935 |
| 15481 | S20_577194 | 20 | 577194 | 100 | G | C | C | 0.53226 | G | 0.46774 | 15 | 0.48387 |
| 15482 | S20_577214 | 20 | 577214 | 20 | A | G | A | 0.93548 | G | 0.06452 | 4 | 0.12903 |
| 15483 | S20_577226 | 20 | 577226 | 12 | T | C | T | 0.51613 | C | 0.48387 | 14 | 0.45161 |
| 15484 | S20_577230 | 20 | 577230 | 4 | T | C | C | 0.91935 | T | 0.08065 | 5 | 0.16129 |
| 15485 | S20_770167 | 20 | 770167 | 192937 | G | A | G | 0.93548 | A | 0.06452 | 4 | 0.12903 |
| 15486 | S20_770259 | 20 | 770259 | 92 | A | T | A | 0.91935 | T | 0.08065 | 5 | 0.16129 |
| 15487 | S20_817672 | 20 | 817672 | 47413 | G | A | A | 0.83871 | G | 0.16129 | 6 | 0.19355 |
| 15488 | S20_884719 | 20 | 884719 | 67047 | C | G | C | 0.91935 | G | 0.08065 | 5 | 0.16129 |
| 15489 | S20_905326 | 20 | 905326 | 20607 | C | T | C | 0.80645 | T | 0.19355 | 12 | 0.3871 |
| 15490 | S20_905464 | 20 | 905464 | 138 | G | C | G | 0.87097 | C | 0.12903 | 4 | 0.12903 |
| 15491 | S20_905476 | 20 | 905476 | 12 | C | T | C | 0.90323 | T | 0.09677 | 6 | 0.19355 |
| 15492 | S20_905477 | 20 | 905477 | 1 | A | G | A | 0.90323 | G | 0.09677 | 6 | 0.19355 |
| 15493 | S20_911488 | 20 | 911488 | 6011 | T | A | T | 0.8871 | A | 0.1129 | 5 | 0.16129 |
| 15494 | S20_1118852 | 20 | 1118852 | 207364 | A | T | T | 0.58065 | A | 0.41935 | 12 | 0.3871 |
| 15495 | S20_1118875 | 20 | 1118875 | 23 | T | C | C | 0.58065 | T | 0.41935 | 12 | 0.3871 |
| 15496 | S20_1118928 | 20 | 1118928 | 53 | A | C | C | 0.58065 | A | 0.41935 | 12 | 0.3871 |

| Site number | SNP name | Chromosome | Physical position | Physical distance from the previous SNP | Reference allele | Alternative allele | Major allele | Major allele frequency | Minor allele | Minor allele frequency | Number heterozygous | Heterozygosity |
|---|---|---|---|---|---|---|---|---|---|---|---|---|
| 15497 | S20_1167247 | 20 | 1167247 | 48319 | C | T | C | 0.8871 | T | 0.1129 | 7 | 0.22581 |
| 15498 | S20_1177402 | 20 | 1177402 | 10155 | G | A | G | 0.91935 | A | 0.08065 | 5 | 0.16129 |
| 15499 | S20_1446816 | 20 | 1446816 | 269414 | T | C | T | 0.64516 | C | 0.35484 | 12 | 0.3871 |
| 15500 | S20_1447002 | 20 | 1447002 | 186 | A | G | A | 0.93548 | G | 0.06452 | 2 | 0.06452 |
| 15501 | S20_1570067 | 20 | 1570067 | 123065 | C | A | C | 0.53226 | A | 0.46774 | 15 | 0.48387 |
| 15502 | S20_1570111 | 20 | 1570111 | 44 | G | C | G | 0.95161 | C | 0.04839 | 3 | 0.09677 |
| 15503 | S20_1617058 | 20 | 1617058 | 46947 | T | G | G | 0.70968 | T | 0.29032 | 18 | 0.58065 |
| 15504 | S20_1918215 | 20 | 1918215 | 301157 | G | T | G | 0.80645 | T | 0.19355 | 10 | 0.32258 |
| 15505 | S20_1986534 | 20 | 1986534 | 68319 | C | T | C | 0.93548 | T | 0.06452 | 4 | 0.12903 |
| 15506 | S20_1986575 | 20 | 1986575 | 41 | G | A | G | 0.93548 | A | 0.06452 | 4 | 0.12903 |
| 15507 | S20_1986642 | 20 | 1986642 | 67 | C | T | C | 0.93548 | T | 0.06452 | 4 | 0.12903 |
| 15508 | S20_2116115 | 20 | 2116115 | 129473 | A | G | A | 0.83871 | G | 0.16129 | 6 | 0.19355 |
| 15509 | S20_2330843 | 20 | 2330843 | 214728 | T | G | G | 0.6129 | T | 0.3871 | 18 | 0.58065 |
| 15510 | S20_2330856 | 20 | 2330856 | 13 | A | C | A | 0.5 | C | 0.5 | 17 | 0.54839 |
| 15511 | S20_2330859 | 20 | 2330859 | 3 | A | G | G | 0.91935 | A | 0.08065 | 5 | 0.16129 |
| 15512 | S20_2330871 | 20 | 2330871 | 12 | C | A | C | 0.82258 | A | 0.17742 | 9 | 0.29032 |
| 15513 | S20_2330874 | 20 | 2330874 | 3 | T | C | C | 0.6129 | T | 0.3871 | 18 | 0.58065 |
| 15514 | S20_2330911 | 20 | 2330911 | 37 | A | G | G | 0.74194 | A | 0.25806 | 10 | 0.32258 |
| 15515 | S20_2454047 | 20 | 2454047 | 123136 | A | G | A | 0.82258 | G | 0.17742 | 9 | 0.29032 |
| 15516 | S20_2466954 | 20 | 2466954 | 12907 | T | C | T | 0.90323 | C | 0.09677 | 6 | 0.19355 |
| 15517 | S20_2582377 | 20 | 2582377 | 115423 | G | A | G | 0.93548 | A | 0.06452 | 4 | 0.12903 |
| 15518 | S20_2582380 | 20 | 2582380 | 3 | C | T | C | 0.64516 | T | 0.35484 | 12 | 0.3871 |
| 15519 | S20_2582381 | 20 | 2582381 | 1 | A | G | A | 0.64516 | G | 0.35484 | 12 | 0.3871 |
| 15520 | S20_2582399 | 20 | 2582399 | 18 | C | T | C | 0.64516 | T | 0.35484 | 12 | 0.3871 |
| 15521 | S20_2582418 | 20 | 2582418 | 19 | T | C | C | 0.54839 | T | 0.45161 | 14 | 0.45161 |
| 15522 | S20_2582436 | 20 | 2582436 | 18 | A | G | G | 0.90323 | A | 0.09677 | 6 | 0.19355 |
| 15523 | S20_2582441 | 20 | 2582441 | 5 | C | T | T | 0.90323 | C | 0.09677 | 6 | 0.19355 |
| 15524 | S20_2582442 | 20 | 2582442 | 1 | A | G | G | 0.90323 | A | 0.09677 | 6 | 0.19355 |
| 15525 | S20_2582451 | 20 | 2582451 | 9 | A | G | G | 0.90323 | A | 0.09677 | 6 | 0.19355 |
| 15526 | S20_2582452 | 20 | 2582452 | 1 | A | G | G | 0.90323 | A | 0.09677 | 6 | 0.19355 |
| 15527 | S20_2627065 | 20 | 2627065 | 44613 | A | G | G | 0.67742 | A | 0.32258 | 12 | 0.3871 |
| 15528 | S20_2627076 | 20 | 2627076 | 11 | T | G | G | 0.67742 | T | 0.32258 | 12 | 0.3871 |
| 15529 | S20_2627103 | 20 | 2627103 | 27 | G | A | A | 0.67742 | G | 0.32258 | 12 | 0.3871 |
| 15530 | S20_2627158 | 20 | 2627158 | 55 | G | T | T | 0.67742 | G | 0.32258 | 12 | 0.3871 |
| 15531 | S20_2627313 | 20 | 2627313 | 155 | T | A | T | 0.79032 | A | 0.20968 | 7 | 0.22581 |
| 15532 | S20_2735060 | 20 | 2735060 | 107747 | T | G | G | 0.82258 | T | 0.17742 | 9 | 0.29032 |
| 15533 | S20_2737823 | 20 | 2737823 | 2763 | G | C | G | 0.59677 | C | 0.40323 | 13 | 0.41935 |
| 15534 | S20_2762221 | 20 | 2762221 | 24398 | G | C | C | 0.91935 | G | 0.08065 | 3 | 0.09677 |
| 15535 | S20_2762278 | 20 | 2762278 | 57 | G | A | A | 0.91935 | G | 0.08065 | 3 | 0.09677 |
| 15536 | S20_2825073 | 20 | 2825073 | 62795 | T | G | T | 0.82258 | G | 0.17742 | 9 | 0.29032 |
| 15537 | S20_2825100 | 20 | 2825100 | 27 | C | T | C | 0.82258 | T | 0.17742 | 9 | 0.29032 |
| 15538 | S20_2825116 | 20 | 2825116 | 16 | A | G | A | 0.82258 | G | 0.17742 | 9 | 0.29032 |
| 15539 | S20_2825170 | 20 | 2825170 | 54 | C | T | C | 0.77419 | T | 0.22581 | 12 | 0.3871 |
| 15540 | S20_2825224 | 20 | 2825224 | 54 | A | G | A | 0.82258 | G | 0.17742 | 9 | 0.29032 |
| 15541 | S20_3503238 | 20 | 3503238 | 678014 | G | C | C | 0.74194 | G | 0.25806 | 10 | 0.32258 |
| 15542 | S20_3503445 | 20 | 3503445 | 207 | G | A | G | 0.82258 | A | 0.17742 | 11 | 0.35484 |
| 15543 | S20_3622629 | 20 | 3622629 | 119184 | T | C | T | 0.87097 | C | 0.12903 | 4 | 0.12903 |
| 15544 | S20_3622681 | 20 | 3622681 | 52 | C | T | C | 0.80645 | T | 0.19355 | 8 | 0.25806 |
| 15545 | S20_3622726 | 20 | 3622726 | 45 | A | G | G | 0.87097 | A | 0.12903 | 4 | 0.12903 |
| 15546 | S20_3622797 | 20 | 3622797 | 71 | T | C | T | 0.69355 | C | 0.30645 | 15 | 0.48387 |
| 15547 | S20_4044739 | 20 | 4044739 | 421942 | A | T | A | 0.82258 | T | 0.17742 | 9 | 0.29032 |
| 15548 | S20_4044804 | 20 | 4044804 | 65 | C | T | C | 0.85484 | T | 0.14516 | 5 | 0.16129 |
| 15549 | S20_4151833 | 20 | 4151833 | 107029 | C | T | C | 0.5 | T | 0.5 | 13 | 0.41935 |
| 15550 | S20_4303678 | 20 | 4303678 | 151845 | C | T | C | 0.80645 | T | 0.19355 | 10 | 0.32258 |

| Site number | SNP name | Chromosome | Physical position | Physical distance from the previous SNP | Reference allele | Alternative allele | Major allele | Major allele frequency | Minor allele | Minor allele frequency | Number heterozygous | Heterozygosity |
|---|---|---|---|---|---|---|---|---|---|---|---|---|
| 15551 | S20_4520811 | 20 | 4520811 | 217133 | A | T | A | 0.87097 | T | 0.12903 | 6 | 0.19355 |
| 15552 | S20_4520814 | 20 | 4520814 | 3 | A | G | A | 0.8871 | G | 0.1129 | 5 | 0.16129 |
| 15553 | S20_4520822 | 20 | 4520822 | 8 | T | C | T | 0.83871 | C | 0.16129 | 8 | 0.25806 |
| 15554 | S20_4520897 | 20 | 4520897 | 75 | A | G | A | 0.67742 | G | 0.32258 | 18 | 0.58065 |
| 15555 | S20_4987635 | 20 | 4987635 | 466738 | C | T | C | 0.69355 | T | 0.30645 | 15 | 0.48387 |
| 15556 | S20_4987681 | 20 | 4987681 | 46 | T | C | C | 0.6129 | T | 0.3871 | 14 | 0.45161 |
| 15557 | S20_5376678 | 20 | 5376678 | 388997 | T | C | T | 0.54839 | C | 0.45161 | 16 | 0.51613 |
| 15558 | S20_5448716 | 20 | 5448716 | 72038 | A | G | A | 0.90323 | G | 0.09677 | 6 | 0.19355 |
| 15559 | S20_5668278 | 20 | 5668278 | 219562 | A | G | G | 0.93548 | A | 0.06452 | 4 | 0.12903 |
| 15560 | S20_5668282 | 20 | 5668282 | 4 | T | C | T | 0.93548 | C | 0.06452 | 4 | 0.12903 |
| 15561 | S20_5758294 | 20 | 5758294 | 90012 | C | T | C | 0.70968 | T | 0.29032 | 12 | 0.3871 |
| 15562 | S20_5949014 | 20 | 5949014 | 190720 | A | G | A | 0.95161 | G | 0.04839 | 3 | 0.09677 |
| 15563 | S20_6032656 | 20 | 6032656 | 83642 | A | T | A | 0.93548 | T | 0.06452 | 2 | 0.06452 |
| 15564 | S20_6032670 | 20 | 6032670 | 14 | A | G | A | 0.95161 | G | 0.04839 | 1 | 0.03226 |
| 15565 | S20_6032735 | 20 | 6032735 | 65 | C | T | C | 0.91935 | T | 0.08065 | 5 | 0.16129 |
| 15566 | S20_6503934 | 20 | 6503934 | 471199 | T | C | C | 0.87097 | T | 0.12903 | 8 | 0.25806 |
| 15567 | S20_6503962 | 20 | 6503962 | 28 | G | A | G | 0.6129 | A | 0.3871 | 12 | 0.3871 |
| 15568 | S20_6656299 | 20 | 6656299 | 152337 | C | T | C | 0.62903 | T | 0.37097 | 15 | 0.48387 |
| 15569 | S20_6668915 | 20 | 6668915 | 12616 | T | C | T | 0.83871 | C | 0.16129 | 8 | 0.25806 |
| 15570 | S20_6668916 | 20 | 6668916 | 1 | G | A | G | 0.83871 | A | 0.16129 | 8 | 0.25806 |
| 15571 | S20_6668919 | 20 | 6668919 | 3 | T | C | T | 0.83871 | C | 0.16129 | 8 | 0.25806 |
| 15572 | S20_6668929 | 20 | 6668929 | 10 | C | T | C | 0.53226 | T | 0.46774 | 17 | 0.54839 |
| 15573 | S20_6668933 | 20 | 6668933 | 4 | C | T | T | 0.85484 | C | 0.14516 | 7 | 0.22581 |
| 15574 | S20_6668968 | 20 | 6668968 | 35 | T | C | T | 0.77419 | C | 0.22581 | 12 | 0.3871 |
| 15575 | S20_6669010 | 20 | 6669010 | 42 | T | C | T | 0.91935 | C | 0.08065 | 5 | 0.16129 |
| 15576 | S20_6857716 | 20 | 6857716 | 188706 | C | A | A | 0.51613 | C | 0.48387 | 14 | 0.45161 |
| 15577 | S20_7042050 | 20 | 7042050 | 184334 | C | A | C | 0.95161 | A | 0.04839 | 3 | 0.09677 |
| 15578 | S20_7063106 | 20 | 7063106 | 21056 | G | A | G | 0.82258 | A | 0.17742 | 9 | 0.29032 |
| 15579 | S20_7298916 | 20 | 7298916 | 235810 | C | A | A | 0.51613 | C | 0.48387 | 20 | 0.64516 |
| 15580 | S20_7298949 | 20 | 7298949 | 33 | A | C | A | 0.91935 | C | 0.08065 | 3 | 0.09677 |
| 15581 | S20_7631258 | 20 | 7631258 | 332309 | A | G | A | 0.91935 | G | 0.08065 | 5 | 0.16129 |
| 15582 | S20_7783554 | 20 | 7783554 | 152296 | T | C | T | 0.91935 | C | 0.08065 | 5 | 0.16129 |
| 15583 | S20_7961387 | 20 | 7961387 | 177833 | C | T | C | 0.80645 | T | 0.19355 | 10 | 0.32258 |
| 15584 | S20_7961435 | 20 | 7961435 | 48 | A | C | A | 0.80645 | C | 0.19355 | 10 | 0.32258 |
| 15585 | S20_7961471 | 20 | 7961471 | 36 | A | T | A | 0.77419 | T | 0.22581 | 12 | 0.3871 |
| 15586 | S20_8566277 | 20 | 8566277 | 604806 | C | T | C | 0.93548 | T | 0.06452 | 4 | 0.12903 |
| 15587 | S20_8566423 | 20 | 8566423 | 146 | C | T | C | 0.83871 | T | 0.16129 | 10 | 0.32258 |
| 15588 | S20_8566498 | 20 | 8566498 | 75 | G | A | G | 0.79032 | A | 0.20968 | 11 | 0.35484 |
| 15589 | S20_9027106 | 20 | 9027106 | 460608 | G | A | G | 0.56452 | A | 0.43548 | 15 | 0.48387 |
| 15590 | S20_9027295 | 20 | 9027295 | 189 | C | G | C | 0.56452 | G | 0.43548 | 15 | 0.48387 |
| 15591 | S20_9622137 | 20 | 9622137 | 594842 | A | G | A | 0.90323 | G | 0.09677 | 6 | 0.19355 |
| 15592 | S20_9622192 | 20 | 9622192 | 55 | A | G | A | 0.90323 | G | 0.09677 | 6 | 0.19355 |
| 15593 | S20_9622196 | 20 | 9622196 | 4 | T | C | T | 0.90323 | C | 0.09677 | 6 | 0.19355 |
| 15594 | S20_9622219 | 20 | 9622219 | 23 | A | G | A | 0.80645 | G | 0.19355 | 10 | 0.32258 |
| 15595 | S20_9622257 | 20 | 9622257 | 38 | T | C | T | 0.69355 | C | 0.30645 | 15 | 0.48387 |
| 15596 | S20_10073475 | 20 | 10073475 | 451218 | G | A | G | 0.90323 | A | 0.09677 | 6 | 0.19355 |
| 15597 | S20_10335073 | 20 | 10335073 | 261598 | T | C | C | 0.83871 | T | 0.16129 | 8 | 0.25806 |
| 15598 | S20_10335211 | 20 | 10335211 | 138 | G | C | G | 0.72581 | C | 0.27419 | 15 | 0.48387 |
| 15599 | S20_10335246 | 20 | 10335246 | 35 | G | A | G | 0.72581 | A | 0.27419 | 15 | 0.48387 |
| 15600 | S20_10633722 | 20 | 10633722 | 298476 | C | G | C | 0.85484 | G | 0.14516 | 7 | 0.22581 |
| 15601 | S20_10633733 | 20 | 10633733 | 11 | C | T | C | 0.77419 | T | 0.22581 | 12 | 0.3871 |
| 15602 | S20_10633858 | 20 | 10633858 | 125 | C | T | C | 0.77419 | T | 0.22581 | 12 | 0.3871 |
| 15603 | S20_11608319 | 20 | 11608319 | 974461 | G | A | G | 0.56452 | A | 0.43548 | 15 | 0.48387 |
| 15604 | S20_11608350 | 20 | 11608350 | 31 | T | C | T | 0.79032 | C | 0.20968 | 13 | 0.41935 |

| Site number | SNP name | Chromosome | Physical position | Physical distance from the previous SNP | Reference allele | Alternative allele | Major allele | Major allele frequency | Minor allele | Minor allele frequency | Number heterozygous | Heterozygosity |
|---|---|---|---|---|---|---|---|---|---|---|---|---|
| 15605 | S20_11608356 | 20 | 11608356 | 6 | G | A | G | 0.80645 | A | 0.19355 | 10 | 0.32258 |
| 15606 | S20_11608455 | 20 | 11608455 | 99 | A | G | A | 0.75806 | G | 0.24194 | 13 | 0.41935 |
| 15607 | S20_11608461 | 20 | 11608461 | 6 | G | A | G | 0.75806 | A | 0.24194 | 13 | 0.41935 |
| 15608 | S20_11608482 | 20 | 11608482 | 21 | G | A | G | 0.75806 | A | 0.24194 | 13 | 0.41935 |
| 15609 | S20_11681672 | 20 | 11681672 | 73190 | G | A | G | 0.82258 | A | 0.17742 | 9 | 0.29032 |
| 15610 | S20_12005302 | 20 | 12005302 | 323630 | T | C | T | 0.87097 | C | 0.12903 | 8 | 0.25806 |
| 15611 | S20_12005303 | 20 | 12005303 | 1 | G | A | G | 0.87097 | A | 0.12903 | 8 | 0.25806 |
| 15612 | S20_12374162 | 20 | 12374162 | 368859 | C | T | C | 0.95161 | T | 0.04839 | 3 | 0.09677 |
| 15613 | S20_12616935 | 20 | 12616935 | 242773 | G | A | G | 0.90323 | A | 0.09677 | 4 | 0.12903 |
| 15614 | S20_12617058 | 20 | 12617058 | 123 | T | C | T | 0.87097 | C | 0.12903 | 8 | 0.25806 |
| 15615 | S20_12668828 | 20 | 12668828 | 51770 | A | G | A | 0.93548 | G | 0.06452 | 4 | 0.12903 |
| 15616 | S20_12668966 | 20 | 12668966 | 138 | A | T | A | 0.93548 | T | 0.06452 | 4 | 0.12903 |
| 15617 | S20_12669013 | 20 | 12669013 | 47 | C | T | T | 0.77419 | C | 0.22581 | 10 | 0.32258 |
| 15618 | S20_12769537 | 20 | 12769537 | 100524 | C | T | C | 0.82258 | T | 0.17742 | 9 | 0.29032 |
| 15619 | S20_12769606 | 20 | 12769606 | 69 | T | G | T | 0.69355 | G | 0.30645 | 13 | 0.41935 |
| 15620 | S20_12769657 | 20 | 12769657 | 51 | T | A | T | 0.59677 | A | 0.40323 | 11 | 0.35484 |
| 15621 | S20_12805285 | 20 | 12805285 | 35628 | C | T | C | 0.95161 | T | 0.04839 | 3 | 0.09677 |
| 15622 | S20_12805310 | 20 | 12805310 | 25 | T | A | T | 0.85484 | A | 0.14516 | 9 | 0.29032 |
| 15623 | S20_12805473 | 20 | 12805473 | 163 | A | T | A | 0.75806 | T | 0.24194 | 9 | 0.29032 |
| 15624 | S20_13031999 | 20 | 13031999 | 226526 | G | A | G | 0.87097 | A | 0.12903 | 8 | 0.25806 |
| 15625 | S20_13082976 | 20 | 13082976 | 50977 | G | A | G | 0.59677 | A | 0.40323 | 17 | 0.54839 |
| 15626 | S20_13242738 | 20 | 13242738 | 159762 | A | C | A | 0.8871 | C | 0.1129 | 7 | 0.22581 |
| 15627 | S20_13242740 | 20 | 13242740 | 2 | T | C | T | 0.93548 | C | 0.06452 | 2 | 0.06452 |
| 15628 | S20_13242764 | 20 | 13242764 | 24 | G | A | G | 0.80645 | A | 0.19355 | 10 | 0.32258 |
| 15629 | S20_13671152 | 20 | 13671152 | 428388 | A | G | A | 0.79032 | G | 0.20968 | 9 | 0.29032 |
| 15630 | S20_13671256 | 20 | 13671256 | 104 | G | T | G | 0.8871 | T | 0.1129 | 5 | 0.16129 |
| 15631 | S20_14403211 | 20 | 14403211 | 731955 | C | T | C | 0.8871 | T | 0.1129 | 7 | 0.22581 |
| 15632 | S20_14655546 | 20 | 14655546 | 252335 | T | C | T | 0.87097 | C | 0.12903 | 8 | 0.25806 |
| 15633 | S20_14655651 | 20 | 14655651 | 105 | G | A | G | 0.8871 | A | 0.1129 | 7 | 0.22581 |
| 15634 | S20_14655679 | 20 | 14655679 | 28 | G | A | G | 0.85484 | A | 0.14516 | 9 | 0.29032 |
| 15635 | S20_16001068 | 20 | 16001068 | 1345389 | A | G | A | 0.93548 | G | 0.06452 | 2 | 0.06452 |
| 15636 | S20_16001174 | 20 | 16001174 | 106 | T | C | C | 0.74194 | T | 0.25806 | 12 | 0.3871 |
| 15637 | S20_16207145 | 20 | 16207145 | 205971 | A | G | A | 0.95161 | G | 0.04839 | 3 | 0.09677 |
| 15638 | S20_16885403 | 20 | 16885403 | 678258 | T | C | T | 0.83871 | C | 0.16129 | 8 | 0.25806 |
| 15639 | S20_16885489 | 20 | 16885489 | 86 | A | G | A | 0.90323 | G | 0.09677 | 4 | 0.12903 |
| 15640 | S20_16885538 | 20 | 16885538 | 49 | T | A | T | 0.93548 | A | 0.06452 | 4 | 0.12903 |
| 15641 | S20_17152053 | 20 | 17152053 | 266515 | T | C | T | 0.93548 | C | 0.06452 | 2 | 0.06452 |
| 15642 | S20_17152194 | 20 | 17152194 | 141 | A | G | A | 0.69355 | G | 0.30645 | 13 | 0.41935 |
| 15643 | S20_17206639 | 20 | 17206639 | 54445 | C | T | C | 0.93548 | T | 0.06452 | 4 | 0.12903 |
| 15644 | S20_17592236 | 20 | 17592236 | 385597 | A | C | A | 0.79032 | C | 0.20968 | 11 | 0.35484 |
| 15645 | S20_17785884 | 20 | 17785884 | 193648 | C | A | C | 0.82258 | A | 0.17742 | 7 | 0.22581 |
| 15646 | S20_17984541 | 20 | 17984541 | 198657 | G | A | A | 0.69355 | G | 0.30645 | 9 | 0.29032 |
| 15647 | S20_17984644 | 20 | 17984644 | 103 | C | G | G | 0.69355 | C | 0.30645 | 9 | 0.29032 |
| 15648 | S20_18563780 | 20 | 18563780 | 579136 | C | T | C | 0.6129 | T | 0.3871 | 14 | 0.45161 |
| 15649 | S20_19482385 | 20 | 19482385 | 918605 | A | G | A | 0.87097 | G | 0.12903 | 8 | 0.25806 |
| 15650 | S20_19482395 | 20 | 19482395 | 10 | G | A | G | 0.87097 | A | 0.12903 | 8 | 0.25806 |
| 15651 | S20_19792993 | 20 | 19792993 | 310598 | A | G | A | 0.95161 | G | 0.04839 | 3 | 0.09677 |
| 15652 | S20_19793003 | 20 | 19793003 | 10 | A | G | G | 0.54839 | A | 0.45161 | 10 | 0.32258 |
| 15653 | S20_20287095 | 20 | 20287095 | 494092 | C | T | C | 0.87097 | T | 0.12903 | 6 | 0.19355 |
| 15654 | S20_20287158 | 20 | 20287158 | 63 | C | T | C | 0.64516 | T | 0.35484 | 12 | 0.3871 |
| 15655 | S20_20345425 | 20 | 20345425 | 58267 | A | G | A | 0.58065 | G | 0.41935 | 14 | 0.45161 |
| 15656 | S20_20345595 | 20 | 20345595 | 170 | G | T | G | 0.91935 | T | 0.08065 | 3 | 0.09677 |
| 15657 | S20_20345622 | 20 | 20345622 | 27 | A | T | A | 0.64516 | T | 0.35484 | 16 | 0.51613 |
| 15658 | S20_20436952 | 20 | 20436952 | 91330 | C | G | C | 0.95161 | G | 0.04839 | 3 | 0.09677 |

| Site number | SNP name | Chromosome | Physical position | Physical distance from the previous SNP | Reference allele | Alternative allele | Major allele | Major allele frequency | Minor allele | Minor allele frequency | Number heterozygous | Heterozygosity |
|---|---|---|---|---|---|---|---|---|---|---|---|---|
| 15659 | S20_20437109 | 20 | 20437109 | 157 | G | A | G | 0.90323 | A | 0.09677 | 6 | 0.19355 |
| 15660 | S20_20466996 | 20 | 20466996 | 29887 | G | A | G | 0.8871 | A | 0.1129 | 5 | 0.16129 |
| 15661 | S20_20467156 | 20 | 20467156 | 160 | T | C | T | 0.8871 | C | 0.1129 | 5 | 0.16129 |
| 15662 | S20_20467198 | 20 | 20467198 | 42 | G | A | G | 0.83871 | A | 0.16129 | 6 | 0.19355 |
| 15663 | S20_21163354 | 20 | 21163354 | 696156 | G | A | A | 0.85484 | G | 0.14516 | 7 | 0.22581 |
| 15664 | S20_21163358 | 20 | 21163358 | 4 | G | A | G | 0.93548 | A | 0.06452 | 4 | 0.12903 |
| 15665 | S20_21163393 | 20 | 21163393 | 35 | G | A | G | 0.95161 | A | 0.04839 | 3 | 0.09677 |
| 15666 | S20_21163514 | 20 | 21163514 | 121 | C | T | C | 0.8871 | T | 0.1129 | 7 | 0.22581 |
| 15667 | S20_21163519 | 20 | 21163519 | 5 | C | A | C | 0.82258 | A | 0.17742 | 11 | 0.35484 |
| 15668 | S20_21771889 | 20 | 21771889 | 608370 | T | G | G | 0.58065 | T | 0.41935 | 18 | 0.58065 |
| 15669 | S20_21771918 | 20 | 21771918 | 29 | C | T | C | 0.83871 | T | 0.16129 | 8 | 0.25806 |
| 15670 | S20_21890442 | 20 | 21890442 | 118524 | T | C | T | 0.93548 | C | 0.06452 | 4 | 0.12903 |
| 15671 | S20_21890578 | 20 | 21890578 | 136 | A | G | A | 0.74194 | G | 0.25806 | 8 | 0.25806 |
| 15672 | S20_22481302 | 20 | 22481302 | 590724 | A | G | G | 0.95161 | A | 0.04839 | 3 | 0.09677 |
| 15673 | S20_22481305 | 20 | 22481305 | 3 | C | T | C | 0.59677 | T | 0.40323 | 19 | 0.6129 |
| 15674 | S20_22481317 | 20 | 22481317 | 12 | C | T | C | 0.87097 | T | 0.12903 | 6 | 0.19355 |
| 15675 | S20_22553505 | 20 | 22553505 | 72188 | C | T | C | 0.80645 | T | 0.19355 | 12 | 0.3871 |
| 15676 | S20_22865629 | 20 | 22865629 | 312124 | G | A | G | 0.95161 | A | 0.04839 | 3 | 0.09677 |
| 15677 | S20_22865636 | 20 | 22865636 | 7 | G | A | G | 0.87097 | A | 0.12903 | 6 | 0.19355 |
| 15678 | S20_23087769 | 20 | 23087769 | 222133 | C | T | C | 0.8871 | T | 0.1129 | 7 | 0.22581 |
| 15679 | S20_23302739 | 20 | 23302739 | 214970 | A | C | A | 0.8871 | C | 0.1129 | 7 | 0.22581 |
| 15680 | S20_23302914 | 20 | 23302914 | 175 | T | C | T | 0.8871 | C | 0.1129 | 7 | 0.22581 |
| 15681 | S20_23309529 | 20 | 23309529 | 6615 | G | A | G | 0.87097 | A | 0.12903 | 6 | 0.19355 |
| 15682 | S20_23309571 | 20 | 23309571 | 42 | A | G | A | 0.5 | G | 0.5 | 17 | 0.54839 |
| 15683 | S20_23309590 | 20 | 23309590 | 19 | A | G | A | 0.93548 | G | 0.06452 | 4 | 0.12903 |
| 15684 | S20_23309596 | 20 | 23309596 | 6 | A | G | A | 0.5 | G | 0.5 | 17 | 0.54839 |
| 15685 | S20_23309665 | 20 | 23309665 | 69 | C | A | A | 0.5 | C | 0.5 | 17 | 0.54839 |
| 15686 | S20_23526985 | 20 | 23526985 | 217320 | G | A | G | 0.90323 | A | 0.09677 | 6 | 0.19355 |
| 15687 | S20_23527122 | 20 | 23527122 | 137 | T | C | T | 0.90323 | C | 0.09677 | 6 | 0.19355 |
| 15688 | S20_23975479 | 20 | 23975479 | 448357 | A | G | A | 0.90323 | G | 0.09677 | 6 | 0.19355 |
| 15689 | S20_23975625 | 20 | 23975625 | 146 | C | T | C | 0.80645 | T | 0.19355 | 10 | 0.32258 |
| 15690 | S20_23975627 | 20 | 23975627 | 2 | T | C | T | 0.87097 | C | 0.12903 | 6 | 0.19355 |
| 15691 | S20_24183958 | 20 | 24183958 | 208331 | A | T | A | 0.74194 | T | 0.25806 | 10 | 0.32258 |
| 15692 | S20_24259653 | 20 | 24259653 | 75695 | G | C | C | 0.8871 | G | 0.1129 | 5 | 0.16129 |
| 15693 | S20_24259700 | 20 | 24259700 | 47 | G | A | A | 0.56452 | G | 0.43548 | 13 | 0.41935 |
| 15694 | S20_24561029 | 20 | 24561029 | 301329 | A | G | A | 0.87097 | G | 0.12903 | 6 | 0.19355 |
| 15695 | S20_24561050 | 20 | 24561050 | 21 | A | C | A | 0.90323 | C | 0.09677 | 6 | 0.19355 |
| 15696 | S20_24620711 | 20 | 24620711 | 59661 | A | C | A | 0.69355 | C | 0.30645 | 13 | 0.41935 |
| 15697 | S20_24620751 | 20 | 24620751 | 40 | G | C | G | 0.85484 | C | 0.14516 | 7 | 0.22581 |
| 15698 | S20_24620844 | 20 | 24620844 | 93 | C | A | C | 0.85484 | A | 0.14516 | 7 | 0.22581 |
| 15699 | S20_25051668 | 20 | 25051668 | 430824 | A | G | G | 0.83871 | A | 0.16129 | 10 | 0.32258 |
| 15700 | S20_25051698 | 20 | 25051698 | 30 | C | T | C | 0.90323 | T | 0.09677 | 6 | 0.19355 |
| 15701 | S20_25514734 | 20 | 25514734 | 463036 | C | T | C | 0.72581 | T | 0.27419 | 11 | 0.35484 |
| 15702 | S20_25514766 | 20 | 25514766 | 32 | C | T | C | 0.62903 | T | 0.37097 | 19 | 0.6129 |
| 15703 | S20_25514785 | 20 | 25514785 | 19 | C | T | C | 0.91935 | T | 0.08065 | 3 | 0.09677 |
| 15704 | S20_25544201 | 20 | 25544201 | 29416 | T | C | C | 0.72581 | T | 0.27419 | 13 | 0.41935 |
| 15705 | S20_25544344 | 20 | 25544344 | 143 | T | G | T | 0.95161 | G | 0.04839 | 3 | 0.09677 |
| 15706 | S20_25955328 | 20 | 25955328 | 410984 | G | A | G | 0.80645 | A | 0.19355 | 10 | 0.32258 |
| 15707 | S20_25955504 | 20 | 25955504 | 176 | T | C | T | 0.77419 | C | 0.22581 | 12 | 0.3871 |
| 15708 | S20_26132676 | 20 | 26132676 | 177172 | A | G | A | 0.91935 | G | 0.08065 | 5 | 0.16129 |
| 15709 | S20_26132706 | 20 | 26132706 | 30 | A | G | A | 0.8871 | G | 0.1129 | 7 | 0.22581 |
| 15710 | S20_26132707 | 20 | 26132707 | 1 | G | C | G | 0.8871 | C | 0.1129 | 7 | 0.22581 |
| 15711 | S20_26132713 | 20 | 26132713 | 6 | C | T | C | 0.8871 | T | 0.1129 | 7 | 0.22581 |
| 15712 | S20_26132726 | 20 | 26132726 | 13 | C | A | C | 0.8871 | A | 0.1129 | 7 | 0.22581 |

| Site number | SNP name | Chromosome | Physical position | Physical distance from the previous SNP | Reference allele | Alternative allele | Major allele | Major allele frequency | Minor allele | Minor allele frequency | Number heterozygous | Heterozygosity |
|---|---|---|---|---|---|---|---|---|---|---|---|---|
| 15713 | S20_26282425 | 20 | 26282425 | 149699 | C | T | C | 0.93548 | T | 0.06452 | 4 | 0.12903 |
| 15714 | S20_26282509 | 20 | 26282509 | 84 | T | A | T | 0.93548 | A | 0.06452 | 4 | 0.12903 |
| 15715 | S20_26366920 | 20 | 26366920 | 84411 | C | G | C | 0.93548 | G | 0.06452 | 4 | 0.12903 |
| 15716 | S20_28265671 | 20 | 28265671 | 1898751 | C | T | C | 0.77419 | T | 0.22581 | 14 | 0.45161 |
| 15717 | S20_28413669 | 20 | 28413669 | 147998 | T | C | T | 0.91935 | C | 0.08065 | 5 | 0.16129 |
| 15718 | S20_28498787 | 20 | 28498787 | 85118 | G | T | G | 0.93548 | T | 0.06452 | 4 | 0.12903 |
| 15719 | S20_28498929 | 20 | 28498929 | 142 | C | G | C | 0.6129 | G | 0.3871 | 14 | 0.45161 |
| 15720 | S20_28498979 | 20 | 28498979 | 50 | G | A | G | 0.6129 | A | 0.3871 | 14 | 0.45161 |
| 15721 | S20_28498986 | 20 | 28498986 | 7 | G | A | G | 0.69355 | A | 0.30645 | 11 | 0.35484 |
| 15722 | S20_28498990 | 20 | 28498990 | 4 | G | A | G | 0.91935 | A | 0.08065 | 5 | 0.16129 |
| 15723 | S20_28498994 | 20 | 28498994 | 4 | G | A | G | 0.91935 | A | 0.08065 | 5 | 0.16129 |
| 15724 | S20_29138297 | 20 | 29138297 | 639303 | C | T | C | 0.53226 | T | 0.46774 | 19 | 0.6129 |
| 15725 | S20_29138461 | 20 | 29138461 | 164 | C | T | C | 0.74194 | T | 0.25806 | 14 | 0.45161 |
| 15726 | S20_29138480 | 20 | 29138480 | 19 | C | A | C | 0.90323 | A | 0.09677 | 6 | 0.19355 |
| 15727 | S20_29138493 | 20 | 29138493 | 13 | C | T | C | 0.77419 | T | 0.22581 | 12 | 0.3871 |
| 15728 | S20_29642979 | 20 | 29642979 | 504486 | G | A | G | 0.72581 | A | 0.27419 | 13 | 0.41935 |
| 15729 | S20_29642985 | 20 | 29642985 | 6 | A | G | A | 0.8871 | G | 0.1129 | 7 | 0.22581 |
| 15730 | S20_29643043 | 20 | 29643043 | 58 | A | C | A | 0.8871 | C | 0.1129 | 7 | 0.22581 |
| 15731 | S20_29643076 | 20 | 29643076 | 33 | T | A | T | 0.72581 | A | 0.27419 | 15 | 0.48387 |
| 15732 | S20_30555862 | 20 | 30555862 | 912786 | G | T | G | 0.85484 | T | 0.14516 | 9 | 0.29032 |
| 15733 | S20_30555947 | 20 | 30555947 | 85 | T | C | T | 0.58065 | C | 0.41935 | 14 | 0.45161 |
| 15734 | S20_30556020 | 20 | 30556020 | 73 | G | A | A | 0.75806 | G | 0.24194 | 13 | 0.41935 |
| 15735 | S20_30793385 | 20 | 30793385 | 237365 | G | A | G | 0.8871 | A | 0.1129 | 5 | 0.16129 |
| 15736 | S20_30793446 | 20 | 30793446 | 61 | A | G | A | 0.83871 | G | 0.16129 | 8 | 0.25806 |
| 15737 | S20_31333444 | 20 | 31333444 | 539998 | G | C | C | 0.54839 | G | 0.45161 | 18 | 0.58065 |
| 15738 | S20_31343648 | 20 | 31343648 | 10204 | G | A | A | 0.62903 | G | 0.37097 | 15 | 0.48387 |
| 15739 | S20_31343698 | 20 | 31343698 | 50 | G | T | T | 0.6129 | G | 0.3871 | 16 | 0.51613 |
| 15740 | S20_31343804 | 20 | 31343804 | 106 | C | T | C | 0.93548 | T | 0.06452 | 4 | 0.12903 |
| 15741 | S20_31343831 | 20 | 31343831 | 27 | A | G | A | 0.93548 | G | 0.06452 | 4 | 0.12903 |
| 15742 | S20_31506957 | 20 | 31506957 | 163126 | C | T | C | 0.67742 | T | 0.32258 | 14 | 0.45161 |
| 15743 | S20_31602209 | 20 | 31602209 | 95252 | T | C | T | 0.85484 | C | 0.14516 | 9 | 0.29032 |
| 15744 | S20_31602251 | 20 | 31602251 | 42 | G | A | G | 0.95161 | A | 0.04839 | 3 | 0.09677 |
| 15745 | S20_31602344 | 20 | 31602344 | 93 | G | A | A | 0.6129 | G | 0.3871 | 10 | 0.32258 |
| 15746 | S20_31702525 | 20 | 31702525 | 100181 | G | C | G | 0.87097 | C | 0.12903 | 6 | 0.19355 |
| 15747 | S20_31702625 | 20 | 31702625 | 100 | C | T | C | 0.85484 | T | 0.14516 | 9 | 0.29032 |
| 15748 | S20_31711716 | 20 | 31711716 | 9091 | T | C | T | 0.64516 | C | 0.35484 | 14 | 0.45161 |
| 15749 | S20_31711952 | 20 | 31711952 | 236 | G | A | G | 0.95161 | A | 0.04839 | 3 | 0.09677 |
| 15750 | S20_32059399 | 20 | 32059399 | 347447 | C | T | C | 0.8871 | T | 0.1129 | 5 | 0.16129 |
| 15751 | S20_32059401 | 20 | 32059401 | 2 | C | T | C | 0.87097 | T | 0.12903 | 6 | 0.19355 |
| 15752 | S20_32382999 | 20 | 32382999 | 323598 | C | T | C | 0.91935 | T | 0.08065 | 5 | 0.16129 |
| 15753 | S20_32383095 | 20 | 32383095 | 96 | G | A | G | 0.93548 | A | 0.06452 | 4 | 0.12903 |
| 15754 | S20_32383128 | 20 | 32383128 | 33 | C | T | C | 0.93548 | T | 0.06452 | 4 | 0.12903 |
| 15755 | S20_32383202 | 20 | 32383202 | 74 | G | A | A | 0.64516 | G | 0.35484 | 14 | 0.45161 |
| 15756 | S20_33427714 | 20 | 33427714 | 1044512 | G | A | G | 0.93548 | A | 0.06452 | 4 | 0.12903 |
| 15757 | S20_33427716 | 20 | 33427716 | 2 | G | A | G | 0.93548 | A | 0.06452 | 4 | 0.12903 |
| 15758 | S20_33446302 | 20 | 33446302 | 18586 | A | G | A | 0.74194 | G | 0.25806 | 14 | 0.45161 |
| 15759 | S20_33446308 | 20 | 33446308 | 6 | A | G | G | 0.66129 | A | 0.33871 | 15 | 0.48387 |
| 15760 | S20_33446375 | 20 | 33446375 | 67 | G | A | G | 0.87097 | A | 0.12903 | 8 | 0.25806 |
| 15761 | S20_33785922 | 20 | 33785922 | 339547 | T | C | C | 0.67742 | T | 0.32258 | 14 | 0.45161 |
| 15762 | S20_34667858 | 20 | 34667858 | 881936 | G | T | G | 0.91935 | T | 0.08065 | 5 | 0.16129 |
| 15763 | S20_34668022 | 20 | 34668022 | 164 | C | G | C | 0.93548 | G | 0.06452 | 2 | 0.06452 |
| 15764 | S20_34896962 | 20 | 34896962 | 228940 | C | T | C | 0.53226 | T | 0.46774 | 13 | 0.41935 |
| 15765 | S20_34897026 | 20 | 34897026 | 64 | A | G | A | 0.6129 | G | 0.3871 | 16 | 0.51613 |
| 15766 | S20_34897082 | 20 | 34897082 | 56 | G | A | G | 0.85484 | A | 0.14516 | 9 | 0.29032 |

| Site number | SNP name | Chromosome | Physical position | Physical distance from the previous SNP | Reference allele | Alternative allele | Major allele | Major allele frequency | Minor allele | Minor allele frequency | Number heterozygous | Heterozygosity |
|---|---|---|---|---|---|---|---|---|---|---|---|---|
| 15767 | S20_34987939 | 20 | 34987939 | 90857 | G | T | G | 0.5 | T | 0.5 | 15 | 0.48387 |
| 15768 | S20_35065263 | 20 | 35065263 | 77324 | T | C | C | 0.6129 | T | 0.3871 | 18 | 0.58065 |
| 15769 | S20_35150726 | 20 | 35150726 | 85463 | A | T | A | 0.95161 | T | 0.04839 | 3 | 0.09677 |
| 15770 | S20_35150761 | 20 | 35150761 | 35 | G | A | G | 0.95161 | A | 0.04839 | 3 | 0.09677 |
| 15771 | S20_35150859 | 20 | 35150859 | 98 | T | C | T | 0.95161 | C | 0.04839 | 3 | 0.09677 |
| 15772 | S20_35150865 | 20 | 35150865 | 6 | C | T | C | 0.95161 | T | 0.04839 | 3 | 0.09677 |
| 15773 | S20_35150930 | 20 | 35150930 | 65 | A | G | A | 0.91935 | G | 0.08065 | 5 | 0.16129 |
| 15774 | S20_35150934 | 20 | 35150934 | 4 | G | T | G | 0.95161 | T | 0.04839 | 3 | 0.09677 |
| 15775 | S20_35373136 | 20 | 35373136 | 222202 | G | A | G | 0.85484 | A | 0.14516 | 5 | 0.16129 |
| 15776 | S20_35373197 | 20 | 35373197 | 61 | C | T | C | 0.90323 | T | 0.09677 | 6 | 0.19355 |
| 15777 | S20_35673927 | 20 | 35673927 | 300730 | A | G | A | 0.77419 | G | 0.22581 | 14 | 0.45161 |
| 15778 | S20_35673979 | 20 | 35673979 | 52 | C | A | C | 0.85484 | A | 0.14516 | 5 | 0.16129 |
| 15779 | S20_35673986 | 20 | 35673986 | 7 | G | C | G | 0.70968 | C | 0.29032 | 12 | 0.3871 |
| 15780 | S20_35674066 | 20 | 35674066 | 80 | C | G | C | 0.95161 | G | 0.04839 | 3 | 0.09677 |
| 15781 | S20_36056028 | 20 | 36056028 | 381962 | T | G | T | 0.90323 | G | 0.09677 | 6 | 0.19355 |
| 15782 | S20_36056145 | 20 | 36056145 | 117 | C | T | C | 0.53226 | T | 0.46774 | 15 | 0.48387 |
| 15783 | S20_36056191 | 20 | 36056191 | 46 | C | T | C | 0.82258 | T | 0.17742 | 7 | 0.22581 |
| 15784 | S20_36083542 | 20 | 36083542 | 27351 | T | C | C | 0.51613 | T | 0.48387 | 14 | 0.45161 |
| 15785 | S20_36403379 | 20 | 36403379 | 319837 | T | C | C | 0.6129 | T | 0.3871 | 16 | 0.51613 |
| 15786 | S20_36549219 | 20 | 36549219 | 145840 | G | A | G | 0.95161 | A | 0.04839 | 3 | 0.09677 |
| 15787 | S20_36590910 | 20 | 36590910 | 41691 | C | T | C | 0.93548 | T | 0.06452 | 4 | 0.12903 |
| 15788 | S20_36591037 | 20 | 36591037 | 127 | T | C | T | 0.85484 | C | 0.14516 | 7 | 0.22581 |
| 15789 | S20_36630818 | 20 | 36630818 | 39781 | C | T | C | 0.91935 | T | 0.08065 | 5 | 0.16129 |
| 15790 | S20_36630970 | 20 | 36630970 | 152 | G | A | G | 0.77419 | A | 0.22581 | 10 | 0.32258 |
| 15791 | S20_36729283 | 20 | 36729283 | 98313 | T | C | T | 0.87097 | C | 0.12903 | 8 | 0.25806 |
| 15792 | S20_36761528 | 20 | 36761528 | 32245 | T | C | T | 0.66129 | C | 0.33871 | 17 | 0.54839 |
| 15793 | S20_36795756 | 20 | 36795756 | 34228 | G | T | G | 0.93548 | T | 0.06452 | 4 | 0.12903 |
| 15794 | S20_37182605 | 20 | 37182605 | 386849 | G | C | G | 0.95161 | C | 0.04839 | 3 | 0.09677 |
| 15795 | S20_37633576 | 20 | 37633576 | 450971 | A | G | G | 0.72581 | A | 0.27419 | 13 | 0.41935 |
| 15796 | S20_37803145 | 20 | 37803145 | 169569 | A | G | A | 0.93548 | G | 0.06452 | 4 | 0.12903 |
| 15797 | S20_37803204 | 20 | 37803204 | 59 | A | C | A | 0.82258 | C | 0.17742 | 9 | 0.29032 |
| 15798 | S20_37803237 | 20 | 37803237 | 33 | T | C | T | 0.79032 | C | 0.20968 | 11 | 0.35484 |
| 15799 | S20_37834517 | 20 | 37834517 | 31280 | T | G | T | 0.83871 | G | 0.16129 | 10 | 0.32258 |
| 15800 | S20_37834638 | 20 | 37834638 | 121 | G | A | A | 0.72581 | G | 0.27419 | 13 | 0.41935 |
| 15801 | S20_37849069 | 20 | 37849069 | 14431 | T | C | T | 0.82258 | C | 0.17742 | 7 | 0.22581 |
| 15802 | S20_37849094 | 20 | 37849094 | 25 | C | T | T | 0.85484 | C | 0.14516 | 5 | 0.16129 |
| 15803 | S20_37849129 | 20 | 37849129 | 35 | C | T | C | 0.8871 | T | 0.1129 | 7 | 0.22581 |
| 15804 | S20_37849211 | 20 | 37849211 | 82 | A | G | G | 0.93548 | A | 0.06452 | 4 | 0.12903 |
| 15805 | S20_38949256 | 20 | 38949256 | 1100045 | A | G | A | 0.93548 | G | 0.06452 | 4 | 0.12903 |
| 15806 | S20_38949293 | 20 | 38949293 | 37 | C | T | C | 0.72581 | T | 0.27419 | 17 | 0.54839 |
| 15807 | S20_38949442 | 20 | 38949442 | 149 | C | G | C | 0.72581 | G | 0.27419 | 17 | 0.54839 |
| 15808 | S20_38949450 | 20 | 38949450 | 8 | C | G | C | 0.72581 | G | 0.27419 | 17 | 0.54839 |
| 15809 | S20_38949459 | 20 | 38949459 | 9 | T | G | T | 0.93548 | G | 0.06452 | 4 | 0.12903 |
| 15810 | S20_38949461 | 20 | 38949461 | 2 | T | A | T | 0.93548 | A | 0.06452 | 4 | 0.12903 |
| 15811 | S20_38949484 | 20 | 38949484 | 23 | C | T | C | 0.93548 | T | 0.06452 | 4 | 0.12903 |
| 15812 | S20_38968717 | 20 | 38968717 | 19233 | G | A | G | 0.77419 | A | 0.22581 | 12 | 0.3871 |
| 15813 | S20_38987300 | 20 | 38987300 | 18583 | G | C | G | 0.77419 | C | 0.22581 | 12 | 0.3871 |
| 15814 | S20_38995830 | 20 | 38995830 | 8530 | C | A | C | 0.54839 | A | 0.45161 | 20 | 0.64516 |
| 15815 | S20_39035443 | 20 | 39035443 | 39613 | G | A | G | 0.83871 | A | 0.16129 | 6 | 0.19355 |
| 15816 | S20_39035460 | 20 | 39035460 | 17 | C | G | C | 0.85484 | G | 0.14516 | 9 | 0.29032 |
| 15817 | S20_39117637 | 20 | 39117637 | 82177 | T | C | T | 0.8871 | C | 0.1129 | 7 | 0.22581 |
| 15818 | S20_39117750 | 20 | 39117750 | 113 | C | T | C | 0.8871 | T | 0.1129 | 7 | 0.22581 |
| 15819 | S20_39117754 | 20 | 39117754 | 4 | A | G | A | 0.83871 | G | 0.16129 | 8 | 0.25806 |
| 15820 | S20_39137087 | 20 | 39137087 | 19333 | G | C | G | 0.80645 | C | 0.19355 | 10 | 0.32258 |

| Site number | SNP name | Chromosome | Physical position | Physical distance from the previous SNP | Reference allele | Alternative allele | Major allele | Major allele frequency | Minor allele | Minor allele frequency | Number heterozygous | Heterozygosity |
|---|---|---|---|---|---|---|---|---|---|---|---|---|
| 15821 | S20_39819722 | 20 | 39819722 | 682635 | G | C | G | 0.54839 | C | 0.45161 | 16 | 0.51613 |
| 15822 | S20_39819734 | 20 | 39819734 | 12 | G | A | G | 0.54839 | A | 0.45161 | 16 | 0.51613 |
| 15823 | S20_39819814 | 20 | 39819814 | 80 | T | C | T | 0.80645 | C | 0.19355 | 10 | 0.32258 |
| 15824 | S20_39822411 | 20 | 39822411 | 2597 | G | A | A | 0.5 | G | 0.5 | 13 | 0.41935 |
| 15825 | S20_39836923 | 20 | 39836923 | 14512 | T | C | C | 0.74194 | T | 0.25806 | 16 | 0.51613 |
| 15826 | S20_39836954 | 20 | 39836954 | 31 | T | G | G | 0.56452 | T | 0.43548 | 21 | 0.67742 |
| 15827 | S20_39855955 | 20 | 39855955 | 19001 | C | G | C | 0.77419 | G | 0.22581 | 12 | 0.3871 |
| 15828 | S20_39909135 | 20 | 39909135 | 53180 | A | G | G | 0.90323 | A | 0.09677 | 4 | 0.12903 |
| 15829 | S20_39909157 | 20 | 39909157 | 22 | C | G | C | 0.83871 | G | 0.16129 | 10 | 0.32258 |
| 15830 | S20_39909206 | 20 | 39909206 | 49 | C | T | C | 0.87097 | T | 0.12903 | 8 | 0.25806 |
| 15831 | S20_40403590 | 20 | 40403590 | 494384 | A | G | A | 0.59677 | G | 0.40323 | 17 | 0.54839 |
| 15832 | S20_40403674 | 20 | 40403674 | 84 | G | A | G | 0.6129 | A | 0.3871 | 18 | 0.58065 |
| 15833 | S20_40403681 | 20 | 40403681 | 7 | C | A | C | 0.6129 | A | 0.3871 | 18 | 0.58065 |
| 15834 | S20_40403719 | 20 | 40403719 | 38 | G | C | G | 0.83871 | C | 0.16129 | 8 | 0.25806 |
| 15835 | S20_40403746 | 20 | 40403746 | 27 | A | G | A | 0.59677 | G | 0.40323 | 17 | 0.54839 |
| 15836 | S20_40403753 | 20 | 40403753 | 7 | G | A | G | 0.83871 | A | 0.16129 | 8 | 0.25806 |
| 15837 | S20_40403766 | 20 | 40403766 | 13 | A | G | A | 0.59677 | G | 0.40323 | 17 | 0.54839 |
| 15838 | S20_40403768 | 20 | 40403768 | 2 | C | G | C | 0.59677 | G | 0.40323 | 17 | 0.54839 |
| 15839 | S20_40403804 | 20 | 40403804 | 36 | G | A | G | 0.75806 | A | 0.24194 | 13 | 0.41935 |
| 15840 | S20_40985325 | 20 | 40985325 | 581521 | A | G | A | 0.69355 | G | 0.30645 | 13 | 0.41935 |
| 15841 | S20_41287869 | 20 | 41287869 | 302544 | C | T | C | 0.8871 | T | 0.1129 | 5 | 0.16129 |
| 15842 | S20_41287971 | 20 | 41287971 | 102 | T | G | G | 0.70968 | T | 0.29032 | 14 | 0.45161 |
| 15843 | S20_41376442 | 20 | 41376442 | 88471 | C | T | C | 0.87097 | T | 0.12903 | 8 | 0.25806 |
| 15844 | S20_41376556 | 20 | 41376556 | 114 | C | G | C | 0.91935 | G | 0.08065 | 5 | 0.16129 |
| 15845 | S20_41741028 | 20 | 41741028 | 364472 | A | G | A | 0.95161 | G | 0.04839 | 3 | 0.09677 |
| 15846 | S20_41828554 | 20 | 41828554 | 87526 | G | A | G | 0.83871 | A | 0.16129 | 8 | 0.25806 |
| 15847 | S20_41828670 | 20 | 41828670 | 116 | A | G | A | 0.83871 | G | 0.16129 | 8 | 0.25806 |
| 15848 | S20_41828736 | 20 | 41828736 | 66 | C | T | T | 0.83871 | C | 0.16129 | 8 | 0.25806 |
| 15849 | S20_41828743 | 20 | 41828743 | 7 | A | C | A | 0.80645 | C | 0.19355 | 10 | 0.32258 |
| 15850 | S20_41828767 | 20 | 41828767 | 24 | C | T | T | 0.83871 | C | 0.16129 | 8 | 0.25806 |
| 15851 | S20_42052392 | 20 | 42052392 | 223625 | G | C | G | 0.95161 | C | 0.04839 | 3 | 0.09677 |
| 15852 | S20_42052495 | 20 | 42052495 | 103 | T | C | T | 0.6129 | C | 0.3871 | 18 | 0.58065 |
| 15853 | S20_42052502 | 20 | 42052502 | 7 | G | A | G | 0.6129 | A | 0.3871 | 18 | 0.58065 |
| 15854 | S20_42052509 | 20 | 42052509 | 7 | A | C | A | 0.6129 | C | 0.3871 | 18 | 0.58065 |
| 15855 | S20_42162592 | 20 | 42162592 | 110083 | C | T | C | 0.91935 | T | 0.08065 | 5 | 0.16129 |
| 15856 | S20_42162662 | 20 | 42162662 | 70 | G | A | G | 0.91935 | A | 0.08065 | 5 | 0.16129 |
| 15857 | S20_42199378 | 20 | 42199378 | 36716 | T | C | T | 0.72581 | C | 0.27419 | 15 | 0.48387 |
| 15858 | S20_42199433 | 20 | 42199433 | 55 | A | G | G | 0.51613 | A | 0.48387 | 20 | 0.64516 |
| 15859 | S20_42199593 | 20 | 42199593 | 160 | T | A | T | 0.8871 | A | 0.1129 | 7 | 0.22581 |
| 15860 | S20_42827019 | 20 | 42827019 | 627426 | A | T | A | 0.95161 | T | 0.04839 | 3 | 0.09677 |
| 15861 | S20_42827216 | 20 | 42827216 | 197 | G | C | G | 0.91935 | C | 0.08065 | 5 | 0.16129 |
| 15862 | S20_42827224 | 20 | 42827224 | 8 | A | G | A | 0.95161 | G | 0.04839 | 3 | 0.09677 |
| 15863 | S20_43425089 | 20 | 43425089 | 597865 | T | C | T | 0.95161 | C | 0.04839 | 3 | 0.09677 |
| 15864 | S20_43932526 | 20 | 43932526 | 507437 | C | T | C | 0.74194 | T | 0.25806 | 12 | 0.3871 |
| 15865 | S20_43932528 | 20 | 43932528 | 2 | T | C | T | 0.70968 | C | 0.29032 | 14 | 0.45161 |
| 15866 | S20_43932642 | 20 | 43932642 | 114 | A | G | A | 0.70968 | G | 0.29032 | 14 | 0.45161 |
| 15867 | S20_43932692 | 20 | 43932692 | 50 | C | T | C | 0.70968 | T | 0.29032 | 14 | 0.45161 |
| 15868 | S20_44306170 | 20 | 44306170 | 373478 | G | A | G | 0.87097 | A | 0.12903 | 4 | 0.12903 |
| 15869 | S20_44499611 | 20 | 44499611 | 193441 | A | G | G | 0.83871 | A | 0.16129 | 6 | 0.19355 |
| 15870 | S20_44521880 | 20 | 44521880 | 22269 | T | G | G | 0.83871 | T | 0.16129 | 6 | 0.19355 |
| 15871 | S20_45802673 | 20 | 45802673 | 1280793 | T | C | T | 0.83871 | C | 0.16129 | 10 | 0.32258 |
| 15872 | S20_45802674 | 20 | 45802674 | 1 | G | C | G | 0.83871 | C | 0.16129 | 10 | 0.32258 |
| 15873 | S20_45802841 | 20 | 45802841 | 167 | G | T | G | 0.74194 | T | 0.25806 | 10 | 0.32258 |
| 15874 | S20_45871613 | 20 | 45871613 | 68772 | T | C | C | 0.66129 | T | 0.33871 | 13 | 0.41935 |

| Site number | SNP name | Chromosome | Physical position | Physical distance from the previous SNP | Reference allele | Alternative allele | Major allele | Major allele frequency | Minor allele | Minor allele frequency | Number heterozygous | Heterozygosity |
|---|---|---|---|---|---|---|---|---|---|---|---|---|
| 15875 | S20_46669930 | 20 | 46669930 | 798317 | A | G | G | 0.85484 | A | 0.14516 | 5 | 0.16129 |
| 15876 | S20_46669958 | 20 | 46669958 | 28 | A | G | A | 0.95161 | G | 0.04839 | 1 | 0.03226 |
| 15877 | S20_46669969 | 20 | 46669969 | 11 | G | C | G | 0.85484 | C | 0.14516 | 5 | 0.16129 |
| 15878 | S20_46670031 | 20 | 46670031 | 62 | C | T | C | 0.93548 | T | 0.06452 | 4 | 0.12903 |
| 15879 | S20_46670055 | 20 | 46670055 | 24 | G | A | G | 0.87097 | A | 0.12903 | 4 | 0.12903 |
| 15880 | S20_46670057 | 20 | 46670057 | 2 | G | A | G | 0.87097 | A | 0.12903 | 4 | 0.12903 |
| 15881 | S20_46670060 | 20 | 46670060 | 3 | T | C | T | 0.87097 | C | 0.12903 | 4 | 0.12903 |
| 15882 | S20_46670063 | 20 | 46670063 | 3 | T | C | T | 0.87097 | C | 0.12903 | 4 | 0.12903 |
| 15883 | S20_47431143 | 20 | 47431143 | 761080 | C | T | C | 0.8871 | T | 0.1129 | 5 | 0.16129 |
| 15884 | S20_47431161 | 20 | 47431161 | 18 | C | T | C | 0.80645 | T | 0.19355 | 8 | 0.25806 |
| 15885 | S20_47431273 | 20 | 47431273 | 112 | T | G | G | 0.8871 | T | 0.1129 | 5 | 0.16129 |
| 15886 | S20_47659646 | 20 | 47659646 | 228373 | A | G | A | 0.82258 | G | 0.17742 | 9 | 0.29032 |
| 15887 | S20_48511573 | 20 | 48511573 | 851927 | T | C | T | 0.95161 | C | 0.04839 | 3 | 0.09677 |
| 15888 | S20_48580220 | 20 | 48580220 | 68647 | C | T | T | 0.95161 | C | 0.04839 | 3 | 0.09677 |
| 15889 | S20_48580376 | 20 | 48580376 | 156 | A | G | G | 0.95161 | A | 0.04839 | 3 | 0.09677 |
| 15890 | S20_48580405 | 20 | 48580405 | 29 | C | A | C | 0.62903 | A | 0.37097 | 13 | 0.41935 |
| 15891 | S20_48580406 | 20 | 48580406 | 1 | G | A | G | 0.64516 | A | 0.35484 | 16 | 0.51613 |
| 15892 | S20_49270312 | 20 | 49270312 | 689906 | G | A | A | 0.87097 | G | 0.12903 | 8 | 0.25806 |
| 15893 | S20_49270391 | 20 | 49270391 | 79 | G | C | C | 0.87097 | G | 0.12903 | 8 | 0.25806 |
| 15894 | S20_49270447 | 20 | 49270447 | 56 | T | C | C | 0.90323 | T | 0.09677 | 6 | 0.19355 |
| 15895 | S20_50047065 | 20 | 50047065 | 776618 | A | G | G | 0.64516 | A | 0.35484 | 12 | 0.3871 |
| 15896 | S20_50971998 | 20 | 50971998 | 924933 | T | C | C | 0.82258 | T | 0.17742 | 9 | 0.29032 |
| 15897 | S20_50980751 | 20 | 50980751 | 8753 | C | A | C | 0.8871 | A | 0.1129 | 5 | 0.16129 |
| 15898 | S20_50980876 | 20 | 50980876 | 125 | C | T | C | 0.58065 | T | 0.41935 | 16 | 0.51613 |
| 15899 | S20_51726971 | 20 | 51726971 | 746095 | G | A | G | 0.66129 | A | 0.33871 | 13 | 0.41935 |
| 15900 | S20_51726973 | 20 | 51726973 | 2 | G | T | G | 0.91935 | T | 0.08065 | 5 | 0.16129 |
| 15901 | S20_51726997 | 20 | 51726997 | 24 | C | T | C | 0.59677 | T | 0.40323 | 13 | 0.41935 |
| 15902 | S20_51727005 | 20 | 51727005 | 8 | G | A | G | 0.59677 | A | 0.40323 | 13 | 0.41935 |
| 15903 | S20_51906323 | 20 | 51906323 | 179318 | G | A | G | 0.90323 | A | 0.09677 | 4 | 0.12903 |
| 15904 | S20_51906453 | 20 | 51906453 | 130 | C | T | C | 0.93548 | T | 0.06452 | 4 | 0.12903 |
| 15905 | S20_51906460 | 20 | 51906460 | 7 | G | A | G | 0.83871 | A | 0.16129 | 8 | 0.25806 |
| 15906 | S20_51906498 | 20 | 51906498 | 38 | C | T | C | 0.91935 | T | 0.08065 | 5 | 0.16129 |
| 15907 | S20_51906511 | 20 | 51906511 | 13 | T | C | T | 0.95161 | C | 0.04839 | 3 | 0.09677 |
| 15908 | S20_51906540 | 20 | 51906540 | 29 | T | C | C | 0.85484 | T | 0.14516 | 9 | 0.29032 |
| 15909 | S20_52080540 | 20 | 52080540 | 174000 | G | A | G | 0.85484 | A | 0.14516 | 7 | 0.22581 |
| 15910 | S20_52080702 | 20 | 52080702 | 162 | C | T | C | 0.91935 | T | 0.08065 | 5 | 0.16129 |
| 15911 | S20_52080732 | 20 | 52080732 | 30 | G | A | G | 0.72581 | A | 0.27419 | 13 | 0.41935 |
| 15912 | S20_52169187 | 20 | 52169187 | 88455 | C | A | C | 0.95161 | A | 0.04839 | 1 | 0.03226 |
| 15913 | S20_52169262 | 20 | 52169262 | 75 | A | C | A | 0.75806 | C | 0.24194 | 9 | 0.29032 |
| 15914 | S20_52169266 | 20 | 52169266 | 4 | C | G | C | 0.75806 | G | 0.24194 | 9 | 0.29032 |
| 15915 | S20_52208450 | 20 | 52208450 | 39184 | A | G | A | 0.90323 | G | 0.09677 | 4 | 0.12903 |
| 15916 | S20_52208475 | 20 | 52208475 | 25 | A | G | G | 0.66129 | A | 0.33871 | 13 | 0.41935 |
| 15917 | S20_52208482 | 20 | 52208482 | 7 | G | C | G | 0.90323 | C | 0.09677 | 4 | 0.12903 |
| 15918 | S20_53697273 | 20 | 53697273 | 1488791 | G | A | A | 0.8871 | G | 0.1129 | 7 | 0.22581 |
| 15919 | S20_53697287 | 20 | 53697287 | 14 | G | A | G | 0.58065 | A | 0.41935 | 12 | 0.3871 |
| 15920 | S20_53697346 | 20 | 53697346 | 59 | T | C | T | 0.91935 | C | 0.08065 | 3 | 0.09677 |
| 15921 | S20_54424102 | 20 | 54424102 | 726756 | G | A | A | 0.5 | G | 0.5 | 13 | 0.41935 |
| 15922 | S20_54424107 | 20 | 54424107 | 5 | A | G | G | 0.75806 | A | 0.24194 | 9 | 0.29032 |
| 15923 | S20_54424157 | 20 | 54424157 | 50 | G | T | T | 0.75806 | G | 0.24194 | 9 | 0.29032 |
| 15924 | S20_54424270 | 20 | 54424270 | 113 | G | T | T | 0.62903 | G | 0.37097 | 11 | 0.35484 |
| 15925 | S20_55544967 | 20 | 55544967 | 1120697 | A | G | A | 0.82258 | G | 0.17742 | 11 | 0.35484 |
| 15926 | S20_55545021 | 20 | 55545021 | 54 | T | C | T | 0.82258 | C | 0.17742 | 11 | 0.35484 |
| 15927 | S20_55545025 | 20 | 55545025 | 4 | G | A | G | 0.91935 | A | 0.08065 | 5 | 0.16129 |
| 15928 | S20_55815100 | 20 | 55815100 | 270075 | G | A | G | 0.79032 | A | 0.20968 | 11 | 0.35484 |

| Site number | SNP name | Chromosome | Physical position | Physical distance from the previous SNP | Reference allele | Alternative allele | Major allele | Major allele frequency | Minor allele | Minor allele frequency | Number heterozygous | Heterozygosity |
|---|---|---|---|---|---|---|---|---|---|---|---|---|
| 15929 | S20_55815103 | 20 | 55815103 | 3 | T | G | T | 0.93548 | G | 0.06452 | 4 | 0.12903 |
| 15930 | S20_55927212 | 20 | 55927212 | 112109 | A | G | A | 0.58065 | G | 0.41935 | 16 | 0.51613 |
| 15931 | S20_55927215 | 20 | 55927215 | 3 | T | C | T | 0.75806 | C | 0.24194 | 15 | 0.48387 |
| 15932 | S20_55927231 | 20 | 55927231 | 16 | G | A | G | 0.59677 | A | 0.40323 | 13 | 0.41935 |
| 15933 | S20_55927247 | 20 | 55927247 | 16 | C | T | C | 0.87097 | T | 0.12903 | 6 | 0.19355 |
| 15934 | S20_55927346 | 20 | 55927346 | 99 | C | A | C | 0.72581 | A | 0.27419 | 11 | 0.35484 |
| 15935 | S20_55948374 | 20 | 55948374 | 21028 | C | T | C | 0.69355 | T | 0.30645 | 13 | 0.41935 |
| 15936 | S20_56381097 | 20 | 56381097 | 432723 | T | C | C | 0.59677 | T | 0.40323 | 15 | 0.48387 |
| 15937 | S20_56417273 | 20 | 56417273 | 36176 | G | A | G | 0.95161 | A | 0.04839 | 3 | 0.09677 |
| 15938 | S20_56431659 | 20 | 56431659 | 14386 | G | T | G | 0.85484 | T | 0.14516 | 7 | 0.22581 |
| 15939 | S20_56431701 | 20 | 56431701 | 42 | C | T | C | 0.79032 | T | 0.20968 | 9 | 0.29032 |
| 15940 | S20_56541097 | 20 | 56541097 | 109396 | G | A | A | 0.82258 | G | 0.17742 | 9 | 0.29032 |
| 15941 | S20_56541133 | 20 | 56541133 | 36 | G | A | A | 0.82258 | G | 0.17742 | 9 | 0.29032 |
| 15942 | S20_56541134 | 20 | 56541134 | 1 | T | C | C | 0.82258 | T | 0.17742 | 9 | 0.29032 |
| 15943 | S20_56541157 | 20 | 56541157 | 23 | A | G | A | 0.74194 | G | 0.25806 | 14 | 0.45161 |
| 15944 | S20_56541206 | 20 | 56541206 | 49 | T | C | T | 0.82258 | C | 0.17742 | 9 | 0.29032 |
| 15945 | S20_56541214 | 20 | 56541214 | 8 | T | C | C | 0.69355 | T | 0.30645 | 17 | 0.54839 |
| 15946 | S20_56541219 | 20 | 56541219 | 5 | G | A | G | 0.69355 | A | 0.30645 | 17 | 0.54839 |
| 15947 | S20_56541288 | 20 | 56541288 | 69 | C | T | C | 0.69355 | T | 0.30645 | 17 | 0.54839 |
| 15948 | S20_56541311 | 20 | 56541311 | 23 | G | A | G | 0.90323 | A | 0.09677 | 6 | 0.19355 |
| 15949 | S20_56541320 | 20 | 56541320 | 9 | C | T | C | 0.90323 | T | 0.09677 | 6 | 0.19355 |
| 15950 | S20_56578885 | 20 | 56578885 | 37565 | A | C | A | 0.95161 | C | 0.04839 | 3 | 0.09677 |
| 15951 | S20_56579046 | 20 | 56579046 | 161 | G | A | A | 0.5 | G | 0.5 | 19 | 0.6129 |
| 15952 | S20_56579112 | 20 | 56579112 | 66 | C | T | C | 0.91935 | T | 0.08065 | 5 | 0.16129 |
| 15953 | S20_57144112 | 20 | 57144112 | 565000 | C | A | C | 0.83871 | A | 0.16129 | 8 | 0.25806 |
| 15954 | S20_57144235 | 20 | 57144235 | 123 | C | T | C | 0.83871 | T | 0.16129 | 8 | 0.25806 |
| 15955 | S20_57339590 | 20 | 57339590 | 195355 | T | C | T | 0.93548 | C | 0.06452 | 4 | 0.12903 |
| 15956 | S20_57358780 | 20 | 57358780 | 19190 | T | C | T | 0.91935 | C | 0.08065 | 3 | 0.09677 |
| 15957 | S20_57358849 | 20 | 57358849 | 69 | A | G | A | 0.91935 | G | 0.08065 | 3 | 0.09677 |
| 15958 | S20_57369746 | 20 | 57369746 | 10897 | G | A | G | 0.72581 | A | 0.27419 | 11 | 0.35484 |
| 15959 | S20_57369765 | 20 | 57369765 | 19 | C | G | C | 0.72581 | G | 0.27419 | 11 | 0.35484 |
| 15960 | S20_57369838 | 20 | 57369838 | 73 | G | C | G | 0.91935 | C | 0.08065 | 5 | 0.16129 |
| 15961 | S20_57369885 | 20 | 57369885 | 47 | C | G | C | 0.80645 | G | 0.19355 | 8 | 0.25806 |
| 15962 | S20_57767241 | 20 | 57767241 | 397356 | G | A | G | 0.90323 | A | 0.09677 | 6 | 0.19355 |
| 15963 | S20_58132209 | 20 | 58132209 | 364968 | A | G | G | 0.75806 | A | 0.24194 | 13 | 0.41935 |
| 15964 | S20_58234620 | 20 | 58234620 | 102411 | T | A | T | 0.87097 | A | 0.12903 | 8 | 0.25806 |
| 15965 | S20_58234666 | 20 | 58234666 | 46 | G | A | G | 0.79032 | A | 0.20968 | 11 | 0.35484 |
| 15966 | S20_58266130 | 20 | 58266130 | 31464 | A | G | A | 0.83871 | G | 0.16129 | 8 | 0.25806 |
| 15967 | S20_58368320 | 20 | 58368320 | 102190 | G | A | G | 0.93548 | A | 0.06452 | 2 | 0.06452 |
| 15968 | S20_58368422 | 20 | 58368422 | 102 | A | C | A | 0.93548 | C | 0.06452 | 2 | 0.06452 |
| 15969 | S20_58381112 | 20 | 58381112 | 12690 | G | A | G | 0.85484 | A | 0.14516 | 9 | 0.29032 |
| 15970 | S20_58381154 | 20 | 58381154 | 42 | G | A | G | 0.74194 | A | 0.25806 | 14 | 0.45161 |
| 15971 | S20_58844165 | 20 | 58844165 | 463011 | C | T | C | 0.93548 | T | 0.06452 | 4 | 0.12903 |
| 15972 | S20_58845331 | 20 | 58845331 | 1166 | T | C | T | 0.93548 | C | 0.06452 | 4 | 0.12903 |
| 15973 | S20_58845405 | 20 | 58845405 | 74 | G | A | G | 0.93548 | A | 0.06452 | 4 | 0.12903 |
| 15974 | S20_58968024 | 20 | 58968024 | 122619 | C | T | C | 0.91935 | T | 0.08065 | 5 | 0.16129 |
| 15975 | S20_58968038 | 20 | 58968038 | 14 | G | T | G | 0.91935 | T | 0.08065 | 5 | 0.16129 |
| 15976 | S20_58968085 | 20 | 58968085 | 47 | A | G | G | 0.62903 | A | 0.37097 | 13 | 0.41935 |
| 15977 | S20_58968103 | 20 | 58968103 | 18 | C | T | T | 0.62903 | C | 0.37097 | 13 | 0.41935 |
| 15978 | S20_58968161 | 20 | 58968161 | 58 | C | T | C | 0.91935 | T | 0.08065 | 3 | 0.09677 |
| 15979 | S20_59095993 | 20 | 59095993 | 127832 | C | T | C | 0.8871 | T | 0.1129 | 5 | 0.16129 |
| 15980 | S20_59162320 | 20 | 59162320 | 66327 | G | C | C | 0.53226 | G | 0.46774 | 17 | 0.54839 |
| 15981 | S20_59242731 | 20 | 59242731 | 80411 | A | G | A | 0.66129 | G | 0.33871 | 15 | 0.48387 |
| 15982 | S20_59850905 | 20 | 59850905 | 608174 | A | G | A | 0.59677 | G | 0.40323 | 15 | 0.48387 |

| Site number | SNP name | Chromosome | Physical position | Physical distance from the previous SNP | Reference allele | Alternative allele | Major allele | Major allele frequency | Minor allele | Minor allele frequency | Number heterozygous | Heterozygosity |
|---|---|---|---|---|---|---|---|---|---|---|---|---|
| 15983 | S20_59850914 | 20 | 59850914 | 9 | C | T | C | 0.67742 | T | 0.32258 | 12 | 0.3871 |
| 15984 | S20_59850931 | 20 | 59850931 | 17 | C | T | C | 0.6129 | T | 0.3871 | 14 | 0.45161 |
| 15985 | S20_59850932 | 20 | 59850932 | 1 | G | A | G | 0.67742 | A | 0.32258 | 12 | 0.3871 |
| 15986 | S20_59851023 | 20 | 59851023 | 91 | T | C | T | 0.90323 | C | 0.09677 | 6 | 0.19355 |
| 15987 | S20_59851041 | 20 | 59851041 | 18 | G | A | G | 0.82258 | A | 0.17742 | 7 | 0.22581 |
| 15988 | S20_59851050 | 20 | 59851050 | 9 | G | C | G | 0.83871 | C | 0.16129 | 8 | 0.25806 |
| 15989 | S20_59851073 | 20 | 59851073 | 23 | A | G | A | 0.77419 | G | 0.22581 | 12 | 0.3871 |
| 15990 | S20_60041387 | 20 | 60041387 | 190314 | G | A | G | 0.80645 | A | 0.19355 | 10 | 0.32258 |
| 15991 | S20_60041455 | 20 | 60041455 | 68 | T | G | T | 0.51613 | G | 0.48387 | 14 | 0.45161 |
| 15992 | S20_60041503 | 20 | 60041503 | 48 | A | T | A | 0.77419 | T | 0.22581 | 12 | 0.3871 |
| 15993 | S20_60041511 | 20 | 60041511 | 8 | C | A | C | 0.51613 | A | 0.48387 | 14 | 0.45161 |
| 15994 | S20_60107157 | 20 | 60107157 | 65646 | T | C | T | 0.95161 | C | 0.04839 | 3 | 0.09677 |
| 15995 | S20_60107185 | 20 | 60107185 | 28 | A | C | A | 0.64516 | C | 0.35484 | 16 | 0.51613 |
| 15996 | S20_60107190 | 20 | 60107190 | 5 | C | T | C | 0.70968 | T | 0.29032 | 12 | 0.3871 |
| 15997 | S20_60107320 | 20 | 60107320 | 130 | G | A | G | 0.77419 | A | 0.22581 | 12 | 0.3871 |
| 15998 | S20_60107329 | 20 | 60107329 | 9 | T | C | T | 0.77419 | C | 0.22581 | 12 | 0.3871 |
| 15999 | S20_60107333 | 20 | 60107333 | 4 | T | G | T | 0.77419 | G | 0.22581 | 12 | 0.3871 |
| 16000 | S20_60107335 | 20 | 60107335 | 2 | C | T | C | 0.95161 | T | 0.04839 | 3 | 0.09677 |
| 16001 | S20_60107336 | 20 | 60107336 | 1 | G | C | C | 0.5 | G | 0.5 | 17 | 0.54839 |
| 16002 | S20_60107339 | 20 | 60107339 | 3 | C | A | C | 0.77419 | A | 0.22581 | 12 | 0.3871 |
| 16003 | S20_60107366 | 20 | 60107366 | 27 | A | C | A | 0.74194 | C | 0.25806 | 14 | 0.45161 |
| 16004 | S20_60107369 | 20 | 60107369 | 3 | G | A | G | 0.77419 | A | 0.22581 | 12 | 0.3871 |
| 16005 | S20_60107371 | 20 | 60107371 | 2 | C | G | C | 0.77419 | G | 0.22581 | 12 | 0.3871 |
| 16006 | S20_60107377 | 20 | 60107377 | 6 | C | G | C | 0.77419 | G | 0.22581 | 12 | 0.3871 |
| 16007 | S20_61212698 | 20 | 61212698 | 1105321 | G | T | G | 0.90323 | T | 0.09677 | 6 | 0.19355 |
| 16008 | S20_61212846 | 20 | 61212846 | 148 | T | G | T | 0.69355 | G | 0.30645 | 11 | 0.35484 |
| 16009 | S20_61353473 | 20 | 61353473 | 140627 | C | T | C | 0.69355 | T | 0.30645 | 15 | 0.48387 |
| 16010 | S20_61353620 | 20 | 61353620 | 147 | G | A | G | 0.54839 | A | 0.45161 | 18 | 0.58065 |
| 16011 | S20_61527663 | 20 | 61527663 | 174043 | C | G | C | 0.8871 | G | 0.1129 | 7 | 0.22581 |
| 16012 | S20_61527721 | 20 | 61527721 | 58 | C | T | C | 0.8871 | T | 0.1129 | 7 | 0.22581 |
| 16013 | S20_61527740 | 20 | 61527740 | 19 | C | T | C | 0.90323 | T | 0.09677 | 6 | 0.19355 |
| 16014 | S20_61527809 | 20 | 61527809 | 69 | C | A | C | 0.8871 | A | 0.1129 | 7 | 0.22581 |
| 16015 | S20_61527880 | 20 | 61527880 | 71 | G | C | G | 0.8871 | C | 0.1129 | 7 | 0.22581 |
| 16016 | S20_61597475 | 20 | 61597475 | 69595 | G | A | G | 0.8871 | A | 0.1129 | 7 | 0.22581 |
| 16017 | S20_61661533 | 20 | 61661533 | 64058 | C | G | C | 0.62903 | G | 0.37097 | 17 | 0.54839 |
| 16018 | S20_62210855 | 20 | 62210855 | 549322 | T | C | C | 0.6129 | T | 0.3871 | 16 | 0.51613 |
| 16019 | S20_62210881 | 20 | 62210881 | 26 | G | T | T | 0.6129 | G | 0.3871 | 16 | 0.51613 |
| 16020 | S20_62211042 | 20 | 62211042 | 161 | T | G | T | 0.82258 | G | 0.17742 | 9 | 0.29032 |
| 16021 | S20_62376210 | 20 | 62376210 | 165168 | A | T | A | 0.95161 | T | 0.04839 | 3 | 0.09677 |
| 16022 | S20_62376225 | 20 | 62376225 | 15 | G | A | G | 0.90323 | A | 0.09677 | 4 | 0.12903 |
| 16023 | S20_62376338 | 20 | 62376338 | 113 | T | C | T | 0.67742 | C | 0.32258 | 16 | 0.51613 |
| 16024 | S20_62376383 | 20 | 62376383 | 45 | A | T | A | 0.95161 | T | 0.04839 | 3 | 0.09677 |
| 16025 | S20_62376396 | 20 | 62376396 | 13 | C | T | C | 0.58065 | T | 0.41935 | 18 | 0.58065 |
| 16026 | S20_62376419 | 20 | 62376419 | 23 | C | T | C | 0.82258 | T | 0.17742 | 7 | 0.22581 |
| 16027 | S20_62446877 | 20 | 62446877 | 70458 | T | G | T | 0.70968 | G | 0.29032 | 14 | 0.45161 |
| 16028 | S20_62558217 | 20 | 62558217 | 111340 | A | G | A | 0.93548 | G | 0.06452 | 4 | 0.12903 |
| 16029 | S20_62558279 | 20 | 62558279 | 62 | G | T | G | 0.91935 | T | 0.08065 | 5 | 0.16129 |
| 16030 | S20_62558311 | 20 | 62558311 | 32 | A | G | G | 0.62903 | A | 0.37097 | 17 | 0.54839 |
| 16031 | S20_62830164 | 20 | 62830164 | 271853 | G | A | G | 0.93548 | A | 0.06452 | 4 | 0.12903 |
| 16032 | S20_62830191 | 20 | 62830191 | 27 | A | G | A | 0.91935 | G | 0.08065 | 3 | 0.09677 |
| 16033 | S20_63070038 | 20 | 63070038 | 239847 | C | G | C | 0.91935 | G | 0.08065 | 3 | 0.09677 |
| 16034 | S20_63070039 | 20 | 63070039 | 1 | G | T | T | 0.53226 | G | 0.46774 | 13 | 0.41935 |
| 16035 | S20_63070054 | 20 | 63070054 | 15 | C | T | C | 0.83871 | T | 0.16129 | 8 | 0.25806 |
| 16036 | S20_63070104 | 20 | 63070104 | 50 | G | A | A | 0.56452 | G | 0.43548 | 15 | 0.48387 |

| Site number | SNP name | Chromosome | Physical position | Physical distance from the previous SNP | Reference allele | Alternative allele | Major allele | Major allele frequency | Minor allele | Minor allele frequency | Number heterozygous | Heterozygosity |
|---|---|---|---|---|---|---|---|---|---|---|---|---|
| 16037 | S20_63070107 | 20 | 63070107 | 3 | A | G | G | 0.58065 | A | 0.41935 | 14 | 0.45161 |
| 16038 | S20_63081153 | 20 | 63081153 | 11046 | C | T | T | 0.58065 | C | 0.41935 | 16 | 0.51613 |
| 16039 | S20_63081199 | 20 | 63081199 | 46 | T | G | G | 0.6129 | T | 0.3871 | 12 | 0.3871 |
| 16040 | S20_63081285 | 20 | 63081285 | 86 | C | T | T | 0.6129 | C | 0.3871 | 12 | 0.3871 |
| 16041 | S20_63081313 | 20 | 63081313 | 28 | C | T | T | 0.6129 | C | 0.3871 | 12 | 0.3871 |
| 16042 | S20_63081438 | 20 | 63081438 | 125 | A | T | A | 0.83871 | T | 0.16129 | 8 | 0.25806 |
| 16043 | S20_63081499 | 20 | 63081499 | 61 | A | G | A | 0.93548 | G | 0.06452 | 4 | 0.12903 |
| 16044 | S20_63081560 | 20 | 63081560 | 61 | T | G | G | 0.6129 | T | 0.3871 | 12 | 0.3871 |
| 16045 | S20_63081561 | 20 | 63081561 | 1 | C | T | T | 0.6129 | C | 0.3871 | 12 | 0.3871 |
| 16046 | S20_63081601 | 20 | 63081601 | 40 | A | G | A | 0.83871 | G | 0.16129 | 8 | 0.25806 |
| 16047 | S20_63398428 | 20 | 63398428 | 316827 | T | C | T | 0.93548 | C | 0.06452 | 2 | 0.06452 |
| 16048 | S20_63920901 | 20 | 63920901 | 522473 | C | T | C | 0.74194 | T | 0.25806 | 14 | 0.45161 |
| 16049 | S20_63920940 | 20 | 63920940 | 39 | A | G | A | 0.74194 | G | 0.25806 | 14 | 0.45161 |
| 16050 | S20_63921002 | 20 | 63921002 | 62 | G | A | G | 0.95161 | A | 0.04839 | 3 | 0.09677 |
| 16051 | S20_63921038 | 20 | 63921038 | 36 | T | C | T | 0.74194 | C | 0.25806 | 14 | 0.45161 |
| 16052 | S20_63921062 | 20 | 63921062 | 24 | C | A | C | 0.95161 | A | 0.04839 | 3 | 0.09677 |
| 16053 | S20_64293691 | 20 | 64293691 | 372629 | A | G | A | 0.70968 | G | 0.29032 | 12 | 0.3871 |
| 16054 | S20_64293728 | 20 | 64293728 | 37 | A | G | A | 0.70968 | G | 0.29032 | 12 | 0.3871 |
| 16055 | S20_64293732 | 20 | 64293732 | 4 | T | C | T | 0.93548 | C | 0.06452 | 4 | 0.12903 |
| 16056 | S20_64293790 | 20 | 64293790 | 58 | C | T | C | 0.70968 | T | 0.29032 | 12 | 0.3871 |
| 16057 | S20_64293834 | 20 | 64293834 | 44 | T | C | T | 0.74194 | C | 0.25806 | 12 | 0.3871 |
| 16058 | S20_64374943 | 20 | 64374943 | 81109 | A | T | A | 0.93548 | T | 0.06452 | 4 | 0.12903 |
| 16059 | S20_64374957 | 20 | 64374957 | 14 | G | A | A | 0.54839 | G | 0.45161 | 18 | 0.58065 |
| 16060 | S20_64375049 | 20 | 64375049 | 92 | T | A | T | 0.87097 | A | 0.12903 | 6 | 0.19355 |
| 16061 | S20_64379958 | 20 | 64379958 | 4909 | G | A | G | 0.87097 | A | 0.12903 | 8 | 0.25806 |
| 16062 | S20_64380028 | 20 | 64380028 | 70 | C | T | C | 0.93548 | T | 0.06452 | 4 | 0.12903 |
| 16063 | S20_64380029 | 20 | 64380029 | 1 | G | A | G | 0.93548 | A | 0.06452 | 4 | 0.12903 |
| 16064 | S20_64380038 | 20 | 64380038 | 9 | T | G | T | 0.77419 | G | 0.22581 | 10 | 0.32258 |
| 16065 | S20_64380338 | 20 | 64380338 | 300 | T | C | C | 0.79032 | T | 0.20968 | 11 | 0.35484 |
| 16066 | S20_64380430 | 20 | 64380430 | 92 | G | A | G | 0.74194 | A | 0.25806 | 12 | 0.3871 |
| 16067 | S20_64380440 | 20 | 64380440 | 10 | G | A | G | 0.90323 | A | 0.09677 | 6 | 0.19355 |
| 16068 | S20_64380455 | 20 | 64380455 | 15 | G | A | G | 0.93548 | A | 0.06452 | 4 | 0.12903 |
| 16069 | S20_64380472 | 20 | 64380472 | 17 | C | G | G | 0.90323 | C | 0.09677 | 4 | 0.12903 |
| 16070 | S20_64380484 | 20 | 64380484 | 12 | C | G | C | 0.90323 | G | 0.09677 | 6 | 0.19355 |
| 16071 | S20_64380492 | 20 | 64380492 | 8 | T | C | T | 0.90323 | C | 0.09677 | 6 | 0.19355 |
| 16072 | S20_64702237 | 20 | 64702237 | 321745 | G | A | G | 0.79032 | A | 0.20968 | 9 | 0.29032 |
| 16073 | S20_64702256 | 20 | 64702256 | 19 | C | T | T | 0.83871 | C | 0.16129 | 8 | 0.25806 |
| 16074 | S20_64702286 | 20 | 64702286 | 30 | G | A | A | 0.83871 | G | 0.16129 | 8 | 0.25806 |
| 16075 | S20_64976537 | 20 | 64976537 | 274251 | T | C | T | 0.83871 | C | 0.16129 | 10 | 0.32258 |
| 16076 | S20_64976571 | 20 | 64976571 | 34 | A | G | A | 0.72581 | G | 0.27419 | 15 | 0.48387 |
| 16077 | S20_64976652 | 20 | 64976652 | 81 | A | G | A | 0.70968 | G | 0.29032 | 14 | 0.45161 |
| 16078 | S20_64976653 | 20 | 64976653 | 1 | C | A | C | 0.90323 | A | 0.09677 | 6 | 0.19355 |
| 16079 | S20_64976674 | 20 | 64976674 | 21 | A | G | A | 0.70968 | G | 0.29032 | 14 | 0.45161 |
| 16080 | S20_64976678 | 20 | 64976678 | 4 | T | C | T | 0.70968 | C | 0.29032 | 14 | 0.45161 |
| 16081 | S20_65012893 | 20 | 65012893 | 36215 | T | G | T | 0.83871 | G | 0.16129 | 8 | 0.25806 |
| 16082 | S20_65012904 | 20 | 65012904 | 11 | T | C | T | 0.83871 | C | 0.16129 | 8 | 0.25806 |
| 16083 | S20_65013083 | 20 | 65013083 | 179 | A | C | C | 0.83871 | A | 0.16129 | 8 | 0.25806 |
| 16084 | S20_65052440 | 20 | 65052440 | 39357 | C | A | C | 0.72581 | A | 0.27419 | 9 | 0.29032 |
| 16085 | S20_65052468 | 20 | 65052468 | 28 | G | A | G | 0.85484 | A | 0.14516 | 7 | 0.22581 |
| 16086 | S20_65052507 | 20 | 65052507 | 39 | A | G | A | 0.80645 | G | 0.19355 | 6 | 0.19355 |
| 16087 | S20_65052557 | 20 | 65052557 | 50 | T | C | T | 0.79032 | C | 0.20968 | 11 | 0.35484 |
| 16088 | S20_65052593 | 20 | 65052593 | 36 | A | G | A | 0.80645 | G | 0.19355 | 6 | 0.19355 |
| 16089 | S20_65052615 | 20 | 65052615 | 22 | C | T | C | 0.80645 | T | 0.19355 | 6 | 0.19355 |
| 16090 | S20_65052646 | 20 | 65052646 | 31 | C | T | C | 0.80645 | T | 0.19355 | 6 | 0.19355 |

| Site number | SNP name | Chromosome | Physical position | Physical distance from the previous SNP | Reference allele | Alternative allele | Major allele | Major allele frequency | Minor allele | Minor allele frequency | Number heterozygous | Heterozygosity |
|---|---|---|---|---|---|---|---|---|---|---|---|---|
| 16091 | S20_65052650 | 20 | 65052650 | 4 | G | C | G | 0.80645 | C | 0.19355 | 6 | 0.19355 |
| 16092 | S20_65052657 | 20 | 65052657 | 7 | C | G | C | 0.80645 | G | 0.19355 | 6 | 0.19355 |
| 16093 | S20_65342475 | 20 | 65342475 | 289818 | G | T | G | 0.6129 | T | 0.3871 | 12 | 0.3871 |
| 16094 | S20_65342491 | 20 | 65342491 | 16 | G | T | G | 0.90323 | T | 0.09677 | 6 | 0.19355 |
| 16095 | S20_65342607 | 20 | 65342607 | 116 | C | A | C | 0.6129 | A | 0.3871 | 12 | 0.3871 |
| 16096 | S20_65342656 | 20 | 65342656 | 49 | C | T | C | 0.70968 | T | 0.29032 | 10 | 0.32258 |
| 16097 | S20_65800088 | 20 | 65800088 | 457432 | G | A | G | 0.8871 | A | 0.1129 | 5 | 0.16129 |
| 16098 | S20_65800089 | 20 | 65800089 | 1 | C | T | T | 0.69355 | C | 0.30645 | 13 | 0.41935 |
| 16099 | S20_65800098 | 20 | 65800098 | 9 | T | C | T | 0.82258 | C | 0.17742 | 9 | 0.29032 |
| 16100 | S20_65800172 | 20 | 65800172 | 74 | G | A | A | 0.75806 | G | 0.24194 | 9 | 0.29032 |
| 16101 | S20_65800271 | 20 | 65800271 | 99 | T | C | C | 0.95161 | T | 0.04839 | 3 | 0.09677 |
| 16102 | S20_65800273 | 20 | 65800273 | 2 | T | C | T | 0.80645 | C | 0.19355 | 8 | 0.25806 |
| 16103 | S20_65824979 | 20 | 65824979 | 24706 | A | G | A | 0.95161 | G | 0.04839 | 3 | 0.09677 |
| 16104 | S20_65939111 | 20 | 65939111 | 114132 | A | C | A | 0.83871 | C | 0.16129 | 8 | 0.25806 |
| 16105 | S20_65939275 | 20 | 65939275 | 164 | A | G | A | 0.72581 | G | 0.27419 | 9 | 0.29032 |
| 16106 | S20_65939312 | 20 | 65939312 | 37 | A | G | A | 0.8871 | G | 0.1129 | 5 | 0.16129 |
| 16107 | S20_65939317 | 20 | 65939317 | 5 | C | T | C | 0.91935 | T | 0.08065 | 5 | 0.16129 |
| 16108 | S20_65971181 | 20 | 65971181 | 31864 | C | A | C | 0.83871 | A | 0.16129 | 6 | 0.19355 |
| 16109 | S20_66023102 | 20 | 66023102 | 51921 | C | T | T | 0.53226 | C | 0.46774 | 19 | 0.6129 |
| 16110 | S20_66023233 | 20 | 66023233 | 131 | T | C | T | 0.83871 | C | 0.16129 | 8 | 0.25806 |
| 16111 | S20_66023306 | 20 | 66023306 | 73 | T | A | T | 0.95161 | A | 0.04839 | 1 | 0.03226 |
| 16112 | S20_66145553 | 20 | 66145553 | 122247 | A | G | G | 0.90323 | A | 0.09677 | 6 | 0.19355 |
| 16113 | S20_66723125 | 20 | 66723125 | 577572 | T | A | T | 0.54839 | A | 0.45161 | 16 | 0.51613 |
| 16114 | S20_66723152 | 20 | 66723152 | 27 | G | C | G | 0.54839 | C | 0.45161 | 16 | 0.51613 |
| 16115 | S20_66723162 | 20 | 66723162 | 10 | T | G | T | 0.54839 | G | 0.45161 | 16 | 0.51613 |
| 16116 | S20_66723176 | 20 | 66723176 | 14 | T | C | T | 0.54839 | C | 0.45161 | 16 | 0.51613 |
| 16117 | S20_66723195 | 20 | 66723195 | 19 | A | G | A | 0.54839 | G | 0.45161 | 16 | 0.51613 |
| 16118 | S20_66812443 | 20 | 66812443 | 89248 | A | C | A | 0.83871 | C | 0.16129 | 8 | 0.25806 |
| 16119 | S20_66812477 | 20 | 66812477 | 34 | G | A | G | 0.85484 | A | 0.14516 | 7 | 0.22581 |
| 16120 | S20_66812483 | 20 | 66812483 | 6 | T | C | C | 0.77419 | T | 0.22581 | 12 | 0.3871 |
| 16121 | S20_67063588 | 20 | 67063588 | 251105 | A | G | G | 0.53226 | A | 0.46774 | 15 | 0.48387 |
| 16122 | S20_67156984 | 20 | 67156984 | 93396 | C | T | C | 0.8871 | T | 0.1129 | 5 | 0.16129 |
| 16123 | S20_67157030 | 20 | 67157030 | 46 | T | G | T | 0.95161 | G | 0.04839 | 3 | 0.09677 |
| 16124 | S20_67356340 | 20 | 67356340 | 199310 | T | C | T | 0.95161 | C | 0.04839 | 3 | 0.09677 |
| 16125 | S20_67356344 | 20 | 67356344 | 4 | T | C | T | 0.91935 | C | 0.08065 | 5 | 0.16129 |
| 16126 | S20_67410182 | 20 | 67410182 | 53838 | G | A | G | 0.90323 | A | 0.09677 | 6 | 0.19355 |
| 16127 | S20_67410195 | 20 | 67410195 | 13 | C | T | C | 0.95161 | T | 0.04839 | 3 | 0.09677 |
| 16128 | S20_67410205 | 20 | 67410205 | 10 | G | A | G | 0.93548 | A | 0.06452 | 4 | 0.12903 |
| 16129 | S20_67410223 | 20 | 67410223 | 18 | C | T | C | 0.82258 | T | 0.17742 | 9 | 0.29032 |
| 16130 | S20_67410257 | 20 | 67410257 | 34 | A | T | A | 0.93548 | T | 0.06452 | 4 | 0.12903 |
| 16131 | S20_67410258 | 20 | 67410258 | 1 | T | C | T | 0.87097 | C | 0.12903 | 6 | 0.19355 |
| 16132 | S20_67410290 | 20 | 67410290 | 32 | G | A | G | 0.72581 | A | 0.27419 | 11 | 0.35484 |
| 16133 | S20_67763712 | 20 | 67763712 | 353422 | C | T | C | 0.74194 | T | 0.25806 | 10 | 0.32258 |
| 16134 | S20_67763842 | 20 | 67763842 | 130 | C | T | C | 0.85484 | T | 0.14516 | 5 | 0.16129 |
| 16135 | S20_68058084 | 20 | 68058084 | 294242 | G | A | G | 0.95161 | A | 0.04839 | 3 | 0.09677 |
| 16136 | S20_68143263 | 20 | 68143263 | 85179 | C | T | C | 0.87097 | T | 0.12903 | 4 | 0.12903 |
| 16137 | S20_68143319 | 20 | 68143319 | 56 | A | G | A | 0.93548 | G | 0.06452 | 4 | 0.12903 |
| 16138 | S20_68199039 | 20 | 68199039 | 55720 | A | G | A | 0.87097 | G | 0.12903 | 6 | 0.19355 |
| 16139 | S20_68199055 | 20 | 68199055 | 16 | T | C | C | 0.79032 | T | 0.20968 | 11 | 0.35484 |
| 16140 | S20_68199183 | 20 | 68199183 | 128 | C | T | C | 0.90323 | T | 0.09677 | 4 | 0.12903 |
| 16141 | S20_68290293 | 20 | 68290293 | 91110 | G | A | G | 0.91935 | A | 0.08065 | 5 | 0.16129 |
| 16142 | S20_68290315 | 20 | 68290315 | 22 | T | G | G | 0.70968 | T | 0.29032 | 12 | 0.3871 |
| 16143 | S20_68290445 | 20 | 68290445 | 130 | T | C | T | 0.74194 | C | 0.25806 | 12 | 0.3871 |
| 16144 | S20_68290478 | 20 | 68290478 | 33 | C | T | C | 0.79032 | T | 0.20968 | 11 | 0.35484 |

| Site number | SNP name | Chromosome | Physical position | Physical distance from the previous SNP | Reference allele | Alternative allele | Major allele | Major allele frequency | Minor allele | Minor allele frequency | Number heterozygous | Heterozygosity |
|---|---|---|---|---|---|---|---|---|---|---|---|---|
| 16145 | S20_68441919 | 20 | 68441919 | 151441 | T | C | C | 0.67742 | T | 0.32258 | 14 | 0.45161 |
| 16146 | S20_68537742 | 20 | 68537742 | 95823 | T | C | C | 0.72581 | T | 0.27419 | 13 | 0.41935 |
| 16147 | S20_68537818 | 20 | 68537818 | 76 | A | T | A | 0.91935 | T | 0.08065 | 5 | 0.16129 |
| 16148 | S20_68537828 | 20 | 68537828 | 10 | C | T | C | 0.72581 | T | 0.27419 | 11 | 0.35484 |
| 16149 | S20_68537887 | 20 | 68537887 | 59 | A | G | A | 0.82258 | G | 0.17742 | 11 | 0.35484 |
| 16150 | S20_68537992 | 20 | 68537992 | 105 | G | A | G | 0.80645 | A | 0.19355 | 8 | 0.25806 |
| 16151 | S20_68593862 | 20 | 68593862 | 55870 | A | T | A | 0.93548 | T | 0.06452 | 4 | 0.12903 |
| 16152 | S20_68594061 | 20 | 68594061 | 199 | T | C | T | 0.90323 | C | 0.09677 | 6 | 0.19355 |
| 16153 | S20_68878291 | 20 | 68878291 | 284230 | G | T | G | 0.90323 | T | 0.09677 | 4 | 0.12903 |
| 16154 | S20_68878340 | 20 | 68878340 | 49 | T | A | A | 0.93548 | T | 0.06452 | 2 | 0.06452 |
| 16155 | S20_68878503 | 20 | 68878503 | 163 | C | T | T | 0.69355 | C | 0.30645 | 13 | 0.41935 |
| 16156 | S20_68878525 | 20 | 68878525 | 22 | A | G | G | 0.83871 | A | 0.16129 | 6 | 0.19355 |
| 16157 | S20_68899940 | 20 | 68899940 | 21415 | T | C | C | 0.5 | T | 0.5 | 19 | 0.6129 |
| 16158 | S20_68899981 | 20 | 68899981 | 41 | C | T | C | 0.77419 | T | 0.22581 | 14 | 0.45161 |
| 16159 | S20_68900077 | 20 | 68900077 | 96 | C | T | C | 0.72581 | T | 0.27419 | 17 | 0.54839 |
| 16160 | S20_69010756 | 20 | 69010756 | 110679 | A | C | A | 0.83871 | C | 0.16129 | 10 | 0.32258 |
| 16161 | S20_69010778 | 20 | 69010778 | 22 | T | C | T | 0.83871 | C | 0.16129 | 10 | 0.32258 |
| 16162 | S20_69010817 | 20 | 69010817 | 39 | G | A | G | 0.8871 | A | 0.1129 | 5 | 0.16129 |
| 16163 | S20_69010861 | 20 | 69010861 | 44 | G | A | G | 0.83871 | A | 0.16129 | 10 | 0.32258 |
| 16164 | S20_69017099 | 20 | 69017099 | 6238 | A | G | A | 0.70968 | G | 0.29032 | 12 | 0.3871 |
| 16165 | S20_69017241 | 20 | 69017241 | 142 | T | C | T | 0.70968 | C | 0.29032 | 12 | 0.3871 |
| 16166 | S20_69017302 | 20 | 69017302 | 61 | T | C | T | 0.8871 | C | 0.1129 | 5 | 0.16129 |
| 16167 | S20_69329024 | 20 | 69329024 | 311722 | G | A | G | 0.74194 | A | 0.25806 | 12 | 0.3871 |
| 16168 | S20_69459135 | 20 | 69459135 | 130111 | G | A | G | 0.95161 | A | 0.04839 | 1 | 0.03226 |
| 16169 | S20_69459169 | 20 | 69459169 | 34 | A | C | A | 0.62903 | C | 0.37097 | 15 | 0.48387 |
| 16170 | S20_69459172 | 20 | 69459172 | 3 | A | G | A | 0.62903 | G | 0.37097 | 15 | 0.48387 |
| 16171 | S20_69518260 | 20 | 69518260 | 59088 | C | G | C | 0.91935 | G | 0.08065 | 5 | 0.16129 |
| 16172 | S20_69573611 | 20 | 69573611 | 55351 | G | A | A | 0.62903 | G | 0.37097 | 17 | 0.54839 |
| 16173 | S20_69573638 | 20 | 69573638 | 27 | T | C | T | 0.82258 | C | 0.17742 | 9 | 0.29032 |
| 16174 | S20_69573672 | 20 | 69573672 | 34 | G | A | G | 0.82258 | A | 0.17742 | 9 | 0.29032 |
| 16175 | S20_69679398 | 20 | 69679398 | 105726 | C | T | C | 0.8871 | T | 0.1129 | 5 | 0.16129 |
| 16176 | S20_69679450 | 20 | 69679450 | 52 | G | A | A | 0.90323 | G | 0.09677 | 6 | 0.19355 |
| 16177 | S20_69744771 | 20 | 69744771 | 65321 | A | C | A | 0.93548 | C | 0.06452 | 4 | 0.12903 |
| 16178 | S20_69891158 | 20 | 69891158 | 146387 | G | T | G | 0.79032 | T | 0.20968 | 11 | 0.35484 |
| 16179 | S20_69891162 | 20 | 69891162 | 4 | G | A | G | 0.93548 | A | 0.06452 | 4 | 0.12903 |
| 16180 | S20_69891232 | 20 | 69891232 | 70 | G | A | G | 0.93548 | A | 0.06452 | 4 | 0.12903 |
| 16181 | S20_69891788 | 20 | 69891788 | 556 | T | C | T | 0.53226 | C | 0.46774 | 19 | 0.6129 |
| 16182 | S20_70141480 | 20 | 70141480 | 249692 | C | T | T | 0.93548 | C | 0.06452 | 4 | 0.12903 |
| 16183 | S20_70141531 | 20 | 70141531 | 51 | C | T | C | 0.91935 | T | 0.08065 | 5 | 0.16129 |
| 16184 | S20_70141533 | 20 | 70141533 | 2 | G | A | G | 0.79032 | A | 0.20968 | 11 | 0.35484 |
| 16185 | S20_70141542 | 20 | 70141542 | 9 | C | T | C | 0.91935 | T | 0.08065 | 5 | 0.16129 |
| 16186 | S20_70141638 | 20 | 70141638 | 96 | C | A | C | 0.87097 | A | 0.12903 | 8 | 0.25806 |
| 16187 | S20_70141696 | 20 | 70141696 | 58 | C | T | C | 0.91935 | T | 0.08065 | 5 | 0.16129 |
| 16188 | S20_70398569 | 20 | 70398569 | 256873 | A | G | G | 0.64516 | A | 0.35484 | 14 | 0.45161 |
| 16189 | S20_70398570 | 20 | 70398570 | 1 | T | C | C | 0.66129 | T | 0.33871 | 13 | 0.41935 |
| 16190 | S20_70398646 | 20 | 70398646 | 76 | T | C | C | 0.66129 | T | 0.33871 | 13 | 0.41935 |
| 16191 | S20_70518115 | 20 | 70518115 | 119469 | A | G | A | 0.67742 | G | 0.32258 | 12 | 0.3871 |
| 16192 | S20_70701365 | 20 | 70701365 | 183250 | C | T | C | 0.8871 | T | 0.1129 | 7 | 0.22581 |
| 16193 | S20_70701370 | 20 | 70701370 | 5 | T | G | G | 0.5 | T | 0.5 | 13 | 0.41935 |
| 16194 | S20_70701450 | 20 | 70701450 | 80 | A | G | G | 0.58065 | A | 0.41935 | 14 | 0.45161 |
| 16195 | S20_70701561 | 20 | 70701561 | 111 | A | G | A | 0.80645 | G | 0.19355 | 6 | 0.19355 |
| 16196 | S20_70701577 | 20 | 70701577 | 16 | T | A | A | 0.67742 | T | 0.32258 | 12 | 0.3871 |
| 16197 | S20_70873281 | 20 | 70873281 | 171704 | G | A | G | 0.95161 | A | 0.04839 | 3 | 0.09677 |
| 16198 | S20_70873378 | 20 | 70873378 | 97 | C | A | C | 0.95161 | A | 0.04839 | 3 | 0.09677 |

| Site number | SNP name | Chromosome | Physical position | Physical distance from the previous SNP | Reference allele | Alternative allele | Major allele | Major allele frequency | Minor allele | Minor allele frequency | Number heterozygous | Heterozygosity |
|---|---|---|---|---|---|---|---|---|---|---|---|---|
| 16199 | S21_442032 | 21 | 442032 | 0 | T | G | T | 0.83871 | G | 0.16129 | 8 | 0.25806 |
| 16200 | S21_442160 | 21 | 442160 | 128 | A | G | A | 0.80645 | G | 0.19355 | 12 | 0.3871 |
| 16201 | S21_1200291 | 21 | 1200291 | 758131 | G | A | G | 0.59677 | A | 0.40323 | 13 | 0.41935 |
| 16202 | S21_1200313 | 21 | 1200313 | 22 | C | T | C | 0.90323 | T | 0.09677 | 6 | 0.19355 |
| 16203 | S21_1339157 | 21 | 1339157 | 138844 | G | A | G | 0.74194 | A | 0.25806 | 12 | 0.3871 |
| 16204 | S21_1573430 | 21 | 1573430 | 234273 | A | G | G | 0.83871 | A | 0.16129 | 6 | 0.19355 |
| 16205 | S21_1573588 | 21 | 1573588 | 158 | C | T | C | 0.8871 | T | 0.1129 | 5 | 0.16129 |
| 16206 | S21_1885552 | 21 | 1885552 | 311964 | G | A | G | 0.82258 | A | 0.17742 | 7 | 0.22581 |
| 16207 | S21_1885612 | 21 | 1885612 | 60 | A | G | A | 0.85484 | G | 0.14516 | 7 | 0.22581 |
| 16208 | S21_1885615 | 21 | 1885615 | 3 | A | C | A | 0.85484 | C | 0.14516 | 7 | 0.22581 |
| 16209 | S21_1885694 | 21 | 1885694 | 79 | G | A | G | 0.82258 | A | 0.17742 | 7 | 0.22581 |
| 16210 | S21_1885706 | 21 | 1885706 | 12 | C | A | C | 0.85484 | A | 0.14516 | 7 | 0.22581 |
| 16211 | S21_1885747 | 21 | 1885747 | 41 | A | G | G | 0.70968 | A | 0.29032 | 10 | 0.32258 |
| 16212 | S21_1885777 | 21 | 1885777 | 30 | G | C | C | 0.74194 | G | 0.25806 | 8 | 0.25806 |
| 16213 | S21_1885944 | 21 | 1885944 | 167 | A | T | T | 0.74194 | A | 0.25806 | 8 | 0.25806 |
| 16214 | S21_1886003 | 21 | 1886003 | 59 | G | A | A | 0.56452 | G | 0.43548 | 11 | 0.35484 |
| 16215 | S21_2811245 | 21 | 2811245 | 925242 | G | A | G | 0.91935 | A | 0.08065 | 5 | 0.16129 |
| 16216 | S21_2811322 | 21 | 2811322 | 77 | G | T | G | 0.91935 | T | 0.08065 | 5 | 0.16129 |
| 16217 | S21_2811343 | 21 | 2811343 | 21 | C | T | C | 0.69355 | T | 0.30645 | 9 | 0.29032 |
| 16218 | S21_2811370 | 21 | 2811370 | 27 | G | A | G | 0.79032 | A | 0.20968 | 9 | 0.29032 |
| 16219 | S21_3422884 | 21 | 3422884 | 611514 | C | T | C | 0.91935 | T | 0.08065 | 5 | 0.16129 |
| 16220 | S21_3549111 | 21 | 3549111 | 126227 | G | A | G | 0.95161 | A | 0.04839 | 3 | 0.09677 |
| 16221 | S21_3549132 | 21 | 3549132 | 21 | C | G | C | 0.6129 | G | 0.3871 | 14 | 0.45161 |
| 16222 | S21_3549177 | 21 | 3549177 | 45 | C | A | C | 0.79032 | A | 0.20968 | 11 | 0.35484 |
| 16223 | S21_3932181 | 21 | 3932181 | 383004 | C | T | T | 0.77419 | C | 0.22581 | 12 | 0.3871 |
| 16224 | S21_3932224 | 21 | 3932224 | 43 | G | T | G | 0.80645 | T | 0.19355 | 12 | 0.3871 |
| 16225 | S21_3932258 | 21 | 3932258 | 34 | G | A | G | 0.91935 | A | 0.08065 | 5 | 0.16129 |
| 16226 | S21_3932268 | 21 | 3932268 | 10 | C | A | C | 0.91935 | A | 0.08065 | 5 | 0.16129 |
| 16227 | S21_3932277 | 21 | 3932277 | 9 | T | C | T | 0.80645 | C | 0.19355 | 12 | 0.3871 |
| 16228 | S21_3982894 | 21 | 3982894 | 50617 | T | C | T | 0.93548 | C | 0.06452 | 4 | 0.12903 |
| 16229 | S21_4184235 | 21 | 4184235 | 201341 | T | A | T | 0.91935 | A | 0.08065 | 5 | 0.16129 |
| 16230 | S21_4184254 | 21 | 4184254 | 19 | C | A | A | 0.74194 | C | 0.25806 | 14 | 0.45161 |
| 16231 | S21_4184334 | 21 | 4184334 | 80 | A | G | A | 0.90323 | G | 0.09677 | 4 | 0.12903 |
| 16232 | S21_4510463 | 21 | 4510463 | 326129 | C | T | C | 0.79032 | T | 0.20968 | 13 | 0.41935 |
| 16233 | S21_4710048 | 21 | 4710048 | 199585 | T | C | T | 0.59677 | C | 0.40323 | 17 | 0.54839 |
| 16234 | S21_4710107 | 21 | 4710107 | 59 | G | T | G | 0.80645 | T | 0.19355 | 6 | 0.19355 |
| 16235 | S21_4710172 | 21 | 4710172 | 65 | G | A | G | 0.80645 | A | 0.19355 | 6 | 0.19355 |
| 16236 | S21_4710201 | 21 | 4710201 | 29 | G | A | G | 0.87097 | A | 0.12903 | 4 | 0.12903 |
| 16237 | S21_4768162 | 21 | 4768162 | 57961 | T | C | T | 0.62903 | C | 0.37097 | 15 | 0.48387 |
| 16238 | S21_4768212 | 21 | 4768212 | 50 | G | A | G | 0.87097 | A | 0.12903 | 8 | 0.25806 |
| 16239 | S21_4858306 | 21 | 4858306 | 90094 | G | A | G | 0.93548 | A | 0.06452 | 4 | 0.12903 |
| 16240 | S21_5815712 | 21 | 5815712 | 957406 | G | C | G | 0.8871 | C | 0.1129 | 7 | 0.22581 |
| 16241 | S21_5815818 | 21 | 5815818 | 106 | G | A | A | 0.72581 | G | 0.27419 | 13 | 0.41935 |
| 16242 | S21_5816860 | 21 | 5816860 | 1042 | T | C | C | 0.75806 | T | 0.24194 | 15 | 0.48387 |
| 16243 | S21_5817044 | 21 | 5817044 | 184 | C | T | C | 0.72581 | T | 0.27419 | 15 | 0.48387 |
| 16244 | S21_6225129 | 21 | 6225129 | 408085 | A | C | C | 0.53226 | A | 0.46774 | 11 | 0.35484 |
| 16245 | S21_6225231 | 21 | 6225231 | 102 | A | G | A | 0.8871 | G | 0.1129 | 7 | 0.22581 |
| 16246 | S21_6276168 | 21 | 6276168 | 50937 | A | G | A | 0.87097 | G | 0.12903 | 6 | 0.19355 |
| 16247 | S21_6395458 | 21 | 6395458 | 119290 | G | A | G | 0.87097 | A | 0.12903 | 6 | 0.19355 |
| 16248 | S21_6395467 | 21 | 6395467 | 9 | T | C | T | 0.82258 | C | 0.17742 | 9 | 0.29032 |
| 16249 | S21_6395562 | 21 | 6395562 | 95 | T | C | T | 0.83871 | C | 0.16129 | 8 | 0.25806 |
| 16250 | S21_6634792 | 21 | 6634792 | 239230 | G | T | G | 0.95161 | T | 0.04839 | 1 | 0.03226 |
| 16251 | S21_6634950 | 21 | 6634950 | 158 | G | A | G | 0.70968 | A | 0.29032 | 12 | 0.3871 |
| 16252 | S21_6877109 | 21 | 6877109 | 242159 | A | G | G | 0.77419 | A | 0.22581 | 14 | 0.45161 |

| Site number | SNP name | Chromosome | Physical position | Physical distance from the previous SNP | Reference allele | Alternative allele | Major allele | Major allele frequency | Minor allele | Minor allele frequency | Number heterozygous | Heterozygosity |
|---|---|---|---|---|---|---|---|---|---|---|---|---|
| 16253 | S21_6877220 | 21 | 6877220 | 111 | C | T | C | 0.95161 | T | 0.04839 | 3 | 0.09677 |
| 16254 | S21_6877228 | 21 | 6877228 | 8 | G | A | G | 0.93548 | A | 0.06452 | 4 | 0.12903 |
| 16255 | S21_6938619 | 21 | 6938619 | 61391 | A | G | A | 0.93548 | G | 0.06452 | 2 | 0.06452 |
| 16256 | S21_6938688 | 21 | 6938688 | 69 | C | A | C | 0.72581 | A | 0.27419 | 11 | 0.35484 |
| 16257 | S21_7053561 | 21 | 7053561 | 114873 | A | G | A | 0.83871 | G | 0.16129 | 8 | 0.25806 |
| 16258 | S21_7053638 | 21 | 7053638 | 77 | A | G | G | 0.95161 | A | 0.04839 | 3 | 0.09677 |
| 16259 | S21_7053704 | 21 | 7053704 | 66 | A | G | A | 0.83871 | G | 0.16129 | 8 | 0.25806 |
| 16260 | S21_7073101 | 21 | 7073101 | 19397 | C | T | C | 0.91935 | T | 0.08065 | 5 | 0.16129 |
| 16261 | S21_7073200 | 21 | 7073200 | 99 | C | T | C | 0.8871 | T | 0.1129 | 7 | 0.22581 |
| 16262 | S21_7200776 | 21 | 7200776 | 127576 | C | T | C | 0.95161 | T | 0.04839 | 3 | 0.09677 |
| 16263 | S21_7200778 | 21 | 7200778 | 2 | G | A | G | 0.93548 | A | 0.06452 | 4 | 0.12903 |
| 16264 | S21_7200871 | 21 | 7200871 | 93 | A | G | A | 0.83871 | G | 0.16129 | 10 | 0.32258 |
| 16265 | S21_7303103 | 21 | 7303103 | 102232 | G | C | G | 0.91935 | C | 0.08065 | 5 | 0.16129 |
| 16266 | S21_7303159 | 21 | 7303159 | 56 | T | C | C | 0.70968 | T | 0.29032 | 8 | 0.25806 |
| 16267 | S21_7303177 | 21 | 7303177 | 18 | G | A | G | 0.91935 | A | 0.08065 | 5 | 0.16129 |
| 16268 | S21_7303311 | 21 | 7303311 | 134 | A | G | A | 0.80645 | G | 0.19355 | 8 | 0.25806 |
| 16269 | S21_7805907 | 21 | 7805907 | 502596 | T | C | T | 0.74194 | C | 0.25806 | 14 | 0.45161 |
| 16270 | S21_7861028 | 21 | 7861028 | 55121 | C | T | C | 0.82258 | T | 0.17742 | 9 | 0.29032 |
| 16271 | S21_8018857 | 21 | 8018857 | 157829 | G | A | G | 0.90323 | A | 0.09677 | 6 | 0.19355 |
| 16272 | S21_8018959 | 21 | 8018959 | 102 | T | C | T | 0.93548 | C | 0.06452 | 4 | 0.12903 |
| 16273 | S21_8203616 | 21 | 8203616 | 184657 | T | C | C | 0.70968 | T | 0.29032 | 10 | 0.32258 |
| 16274 | S21_8203658 | 21 | 8203658 | 42 | G | C | C | 0.64516 | G | 0.35484 | 10 | 0.32258 |
| 16275 | S21_8203676 | 21 | 8203676 | 18 | G | T | T | 0.64516 | G | 0.35484 | 10 | 0.32258 |
| 16276 | S21_8253204 | 21 | 8253204 | 49528 | A | G | A | 0.80645 | G | 0.19355 | 8 | 0.25806 |
| 16277 | S21_8253232 | 21 | 8253232 | 28 | G | A | G | 0.93548 | A | 0.06452 | 4 | 0.12903 |
| 16278 | S21_8253293 | 21 | 8253293 | 61 | C | T | T | 0.64516 | C | 0.35484 | 10 | 0.32258 |
| 16279 | S21_8917666 | 21 | 8917666 | 664373 | A | G | A | 0.54839 | G | 0.45161 | 14 | 0.45161 |
| 16280 | S21_8917718 | 21 | 8917718 | 52 | C | T | C | 0.95161 | T | 0.04839 | 3 | 0.09677 |
| 16281 | S21_8917730 | 21 | 8917730 | 12 | A | G | A | 0.54839 | G | 0.45161 | 14 | 0.45161 |
| 16282 | S21_9016432 | 21 | 9016432 | 98702 | G | A | A | 0.54839 | G | 0.45161 | 16 | 0.51613 |
| 16283 | S21_9016495 | 21 | 9016495 | 63 | C | G | G | 0.79032 | C | 0.20968 | 9 | 0.29032 |
| 16284 | S21_9016564 | 21 | 9016564 | 69 | A | C | C | 0.54839 | A | 0.45161 | 16 | 0.51613 |
| 16285 | S21_9016582 | 21 | 9016582 | 18 | A | G | G | 0.53226 | A | 0.46774 | 17 | 0.54839 |
| 16286 | S21_9016684 | 21 | 9016684 | 102 | A | G | G | 0.54839 | A | 0.45161 | 16 | 0.51613 |
| 16287 | S21_9086200 | 21 | 9086200 | 69516 | C | T | C | 0.64516 | T | 0.35484 | 12 | 0.3871 |
| 16288 | S21_9086206 | 21 | 9086206 | 6 | G | A | G | 0.93548 | A | 0.06452 | 4 | 0.12903 |
| 16289 | S21_9303024 | 21 | 9303024 | 216818 | T | C | T | 0.90323 | C | 0.09677 | 6 | 0.19355 |
| 16290 | S21_9303098 | 21 | 9303098 | 74 | C | A | C | 0.93548 | A | 0.06452 | 4 | 0.12903 |
| 16291 | S21_9303179 | 21 | 9303179 | 81 | C | T | C | 0.90323 | T | 0.09677 | 6 | 0.19355 |
| 16292 | S21_9303185 | 21 | 9303185 | 6 | C | T | C | 0.75806 | T | 0.24194 | 13 | 0.41935 |
| 16293 | S21_9548751 | 21 | 9548751 | 245566 | T | G | T | 0.82258 | G | 0.17742 | 11 | 0.35484 |
| 16294 | S21_9689437 | 21 | 9689437 | 140686 | C | T | C | 0.93548 | T | 0.06452 | 2 | 0.06452 |
| 16295 | S21_9703029 | 21 | 9703029 | 13592 | T | C | T | 0.75806 | C | 0.24194 | 13 | 0.41935 |
| 16296 | S21_9983863 | 21 | 9983863 | 280834 | C | T | C | 0.93548 | T | 0.06452 | 4 | 0.12903 |
| 16297 | S21_10465286 | 21 | 10465286 | 481423 | G | A | G | 0.95161 | A | 0.04839 | 3 | 0.09677 |
| 16298 | S21_11287927 | 21 | 11287927 | 822641 | A | G | A | 0.62903 | G | 0.37097 | 9 | 0.29032 |
| 16299 | S21_11633762 | 21 | 11633762 | 345835 | T | C | C | 0.93548 | T | 0.06452 | 4 | 0.12903 |
| 16300 | S21_11633772 | 21 | 11633772 | 10 | A | G | G | 0.67742 | A | 0.32258 | 10 | 0.32258 |
| 16301 | S21_11799392 | 21 | 11799392 | 165620 | C | T | C | 0.79032 | T | 0.20968 | 7 | 0.22581 |
| 16302 | S21_11799424 | 21 | 11799424 | 32 | G | A | G | 0.75806 | A | 0.24194 | 9 | 0.29032 |
| 16303 | S21_11912845 | 21 | 11912845 | 113421 | G | A | G | 0.59677 | A | 0.40323 | 13 | 0.41935 |
| 16304 | S21_11967692 | 21 | 11967692 | 54847 | T | C | T | 0.93548 | C | 0.06452 | 4 | 0.12903 |
| 16305 | S21_11967774 | 21 | 11967774 | 82 | T | C | C | 0.66129 | T | 0.33871 | 17 | 0.54839 |
| 16306 | S21_11979334 | 21 | 11979334 | 11560 | T | C | T | 0.72581 | C | 0.27419 | 9 | 0.29032 |

| Site number | SNP name | Chromosome | Physical position | Physical distance from the previous SNP | Reference allele | Alternative allele | Major allele | Major allele frequency | Minor allele | Minor allele frequency | Number heterozygous | Heterozygosity |
|---|---|---|---|---|---|---|---|---|---|---|---|---|
| 16307 | S21_11979358 | 21 | 11979358 | 24 | T | C | T | 0.72581 | C | 0.27419 | 9 | 0.29032 |
| 16308 | S21_11979405 | 21 | 11979405 | 47 | T | C | C | 0.82258 | T | 0.17742 | 11 | 0.35484 |
| 16309 | S21_11979516 | 21 | 11979516 | 111 | G | A | G | 0.93548 | A | 0.06452 | 4 | 0.12903 |
| 16310 | S21_12179606 | 21 | 12179606 | 200090 | A | C | A | 0.90323 | C | 0.09677 | 6 | 0.19355 |
| 16311 | S21_12179663 | 21 | 12179663 | 57 | T | C | T | 0.95161 | C | 0.04839 | 3 | 0.09677 |
| 16312 | S21_12645126 | 21 | 12645126 | 465463 | C | G | C | 0.90323 | G | 0.09677 | 6 | 0.19355 |
| 16313 | S21_12645181 | 21 | 12645181 | 55 | C | T | C | 0.93548 | T | 0.06452 | 4 | 0.12903 |
| 16314 | S21_12696415 | 21 | 12696415 | 51234 | C | T | C | 0.93548 | T | 0.06452 | 4 | 0.12903 |
| 16315 | S21_13255664 | 21 | 13255664 | 559249 | G | C | C | 0.53226 | G | 0.46774 | 11 | 0.35484 |
| 16316 | S21_13347217 | 21 | 13347217 | 91553 | C | T | C | 0.8871 | T | 0.1129 | 7 | 0.22581 |
| 16317 | S21_13347297 | 21 | 13347297 | 80 | C | T | C | 0.95161 | T | 0.04839 | 3 | 0.09677 |
| 16318 | S21_13635340 | 21 | 13635340 | 288043 | A | G | G | 0.53226 | A | 0.46774 | 13 | 0.41935 |
| 16319 | S21_13840443 | 21 | 13840443 | 205103 | C | T | C | 0.95161 | T | 0.04839 | 3 | 0.09677 |
| 16320 | S21_13876477 | 21 | 13876477 | 36034 | A | G | G | 0.62903 | A | 0.37097 | 13 | 0.41935 |
| 16321 | S21_13876537 | 21 | 13876537 | 60 | T | C | C | 0.62903 | T | 0.37097 | 13 | 0.41935 |
| 16322 | S21_13876610 | 21 | 13876610 | 73 | G | T | G | 0.90323 | T | 0.09677 | 6 | 0.19355 |
| 16323 | S21_13931637 | 21 | 13931637 | 55027 | A | G | A | 0.80645 | G | 0.19355 | 10 | 0.32258 |
| 16324 | S21_13931649 | 21 | 13931649 | 12 | G | A | G | 0.87097 | A | 0.12903 | 6 | 0.19355 |
| 16325 | S21_13931651 | 21 | 13931651 | 2 | C | T | C | 0.80645 | T | 0.19355 | 10 | 0.32258 |
| 16326 | S21_13931667 | 21 | 13931667 | 16 | G | C | G | 0.90323 | C | 0.09677 | 4 | 0.12903 |
| 16327 | S21_14442364 | 21 | 14442364 | 510697 | T | G | G | 0.56452 | T | 0.43548 | 17 | 0.54839 |
| 16328 | S21_14442424 | 21 | 14442424 | 60 | G | A | A | 0.59677 | G | 0.40323 | 17 | 0.54839 |
| 16329 | S21_14442498 | 21 | 14442498 | 74 | C | T | T | 0.6129 | C | 0.3871 | 18 | 0.58065 |
| 16330 | S21_14790579 | 21 | 14790579 | 348081 | C | T | C | 0.95161 | T | 0.04839 | 3 | 0.09677 |
| 16331 | S21_14790583 | 21 | 14790583 | 4 | A | G | A | 0.95161 | G | 0.04839 | 3 | 0.09677 |
| 16332 | S21_14790612 | 21 | 14790612 | 29 | G | A | G | 0.74194 | A | 0.25806 | 16 | 0.51613 |
| 16333 | S21_14838149 | 21 | 14838149 | 47537 | G | C | G | 0.80645 | C | 0.19355 | 8 | 0.25806 |
| 16334 | S21_14838240 | 21 | 14838240 | 91 | G | A | G | 0.95161 | A | 0.04839 | 3 | 0.09677 |
| 16335 | S21_14838274 | 21 | 14838274 | 34 | C | T | C | 0.91935 | T | 0.08065 | 5 | 0.16129 |
| 16336 | S21_14879770 | 21 | 14879770 | 41496 | T | C | C | 0.53226 | T | 0.46774 | 11 | 0.35484 |
| 16337 | S21_14910188 | 21 | 14910188 | 30418 | A | G | A | 0.91935 | G | 0.08065 | 5 | 0.16129 |
| 16338 | S21_14910279 | 21 | 14910279 | 91 | G | A | A | 0.85484 | G | 0.14516 | 9 | 0.29032 |
| 16339 | S21_14910293 | 21 | 14910293 | 14 | G | A | G | 0.93548 | A | 0.06452 | 4 | 0.12903 |
| 16340 | S21_15109333 | 21 | 15109333 | 199040 | C | T | C | 0.83871 | T | 0.16129 | 8 | 0.25806 |
| 16341 | S21_15109394 | 21 | 15109394 | 61 | C | G | C | 0.75806 | G | 0.24194 | 15 | 0.48387 |
| 16342 | S21_15109429 | 21 | 15109429 | 35 | C | A | C | 0.83871 | A | 0.16129 | 8 | 0.25806 |
| 16343 | S21_15166412 | 21 | 15166412 | 56983 | C | A | A | 0.80645 | C | 0.19355 | 10 | 0.32258 |
| 16344 | S21_15166574 | 21 | 15166574 | 162 | A | G | A | 0.53226 | G | 0.46774 | 13 | 0.41935 |
| 16345 | S21_15457087 | 21 | 15457087 | 290513 | T | C | C | 0.74194 | T | 0.25806 | 8 | 0.25806 |
| 16346 | S21_15457174 | 21 | 15457174 | 87 | C | T | C | 0.82258 | T | 0.17742 | 11 | 0.35484 |
| 16347 | S21_15457178 | 21 | 15457178 | 4 | G | A | G | 0.82258 | A | 0.17742 | 11 | 0.35484 |
| 16348 | S21_15457179 | 21 | 15457179 | 1 | A | G | G | 0.74194 | A | 0.25806 | 8 | 0.25806 |
| 16349 | S21_15476052 | 21 | 15476052 | 18873 | G | A | A | 0.54839 | G | 0.45161 | 16 | 0.51613 |
| 16350 | S21_15476069 | 21 | 15476069 | 17 | T | C | T | 0.79032 | C | 0.20968 | 9 | 0.29032 |
| 16351 | S21_15979068 | 21 | 15979068 | 502999 | T | C | T | 0.95161 | C | 0.04839 | 3 | 0.09677 |
| 16352 | S21_15979083 | 21 | 15979083 | 15 | C | T | C | 0.95161 | T | 0.04839 | 3 | 0.09677 |
| 16353 | S21_15979122 | 21 | 15979122 | 39 | G | A | A | 0.85484 | G | 0.14516 | 9 | 0.29032 |
| 16354 | S21_15979185 | 21 | 15979185 | 63 | T | C | T | 0.95161 | C | 0.04839 | 3 | 0.09677 |
| 16355 | S21_15979198 | 21 | 15979198 | 13 | G | A | G | 0.91935 | A | 0.08065 | 5 | 0.16129 |
| 16356 | S21_15979217 | 21 | 15979217 | 19 | T | G | T | 0.95161 | G | 0.04839 | 3 | 0.09677 |
| 16357 | S21_16085379 | 21 | 16085379 | 106162 | G | A | G | 0.59677 | A | 0.40323 | 11 | 0.35484 |
| 16358 | S21_16085383 | 21 | 16085383 | 4 | G | T | G | 0.66129 | T | 0.33871 | 11 | 0.35484 |
| 16359 | S21_16085467 | 21 | 16085467 | 84 | C | T | C | 0.93548 | T | 0.06452 | 4 | 0.12903 |
| 16360 | S21_16085611 | 21 | 16085611 | 144 | T | C | T | 0.66129 | C | 0.33871 | 11 | 0.35484 |

| Site number | SNP name | Chromosome | Physical position | Physical distance from the previous SNP | Reference allele | Alternative allele | Major allele | Major allele frequency | Minor allele | Minor allele frequency | Number heterozygous | Heterozygosity |
|---|---|---|---|---|---|---|---|---|---|---|---|---|
| 16361 | S21_16202622 | 21 | 16202622 | 117011 | G | A | G | 0.54839 | A | 0.45161 | 10 | 0.32258 |
| 16362 | S21_16242431 | 21 | 16242431 | 39809 | C | T | T | 0.56452 | C | 0.43548 | 15 | 0.48387 |
| 16363 | S21_16242451 | 21 | 16242451 | 20 | G | A | G | 0.64516 | A | 0.35484 | 14 | 0.45161 |
| 16364 | S21_16242455 | 21 | 16242455 | 4 | G | A | A | 0.93548 | G | 0.06452 | 2 | 0.06452 |
| 16365 | S21_16250931 | 21 | 16250931 | 8476 | A | G | A | 0.80645 | G | 0.19355 | 10 | 0.32258 |
| 16366 | S21_16250981 | 21 | 16250981 | 50 | C | T | C | 0.91935 | T | 0.08065 | 5 | 0.16129 |
| 16367 | S21_16251019 | 21 | 16251019 | 38 | A | G | G | 0.83871 | A | 0.16129 | 6 | 0.19355 |
| 16368 | S21_16296334 | 21 | 16296334 | 45315 | A | T | T | 0.77419 | A | 0.22581 | 10 | 0.32258 |
| 16369 | S21_16296438 | 21 | 16296438 | 104 | C | T | C | 0.93548 | T | 0.06452 | 4 | 0.12903 |
| 16370 | S21_16296493 | 21 | 16296493 | 55 | T | C | T | 0.90323 | C | 0.09677 | 4 | 0.12903 |
| 16371 | S21_16296516 | 21 | 16296516 | 23 | G | A | G | 0.85484 | A | 0.14516 | 9 | 0.29032 |
| 16372 | S21_16296549 | 21 | 16296549 | 33 | A | G | A | 0.95161 | G | 0.04839 | 3 | 0.09677 |
| 16373 | S21_16296560 | 21 | 16296560 | 11 | G | C | C | 0.91935 | G | 0.08065 | 5 | 0.16129 |
| 16374 | S21_16315616 | 21 | 16315616 | 19056 | T | C | T | 0.8871 | C | 0.1129 | 7 | 0.22581 |
| 16375 | S21_16315670 | 21 | 16315670 | 54 | C | T | C | 0.72581 | T | 0.27419 | 9 | 0.29032 |
| 16376 | S21_16315693 | 21 | 16315693 | 23 | T | C | T | 0.85484 | C | 0.14516 | 5 | 0.16129 |
| 16377 | S21_16317013 | 21 | 16317013 | 1320 | T | G | T | 0.93548 | G | 0.06452 | 4 | 0.12903 |
| 16378 | S21_16339511 | 21 | 16339511 | 22498 | T | G | T | 0.79032 | G | 0.20968 | 11 | 0.35484 |
| 16379 | S21_16339591 | 21 | 16339591 | 80 | T | A | T | 0.69355 | A | 0.30645 | 11 | 0.35484 |
| 16380 | S21_16339645 | 21 | 16339645 | 54 | C | T | C | 0.59677 | T | 0.40323 | 11 | 0.35484 |
| 16381 | S21_16737787 | 21 | 16737787 | 398142 | C | T | C | 0.5 | T | 0.5 | 17 | 0.54839 |
| 16382 | S21_16737858 | 21 | 16737858 | 71 | C | T | C | 0.93548 | T | 0.06452 | 4 | 0.12903 |
| 16383 | S21_16737920 | 21 | 16737920 | 62 | G | A | G | 0.85484 | A | 0.14516 | 7 | 0.22581 |
| 16384 | S21_17378752 | 21 | 17378752 | 640832 | G | T | G | 0.82258 | T | 0.17742 | 7 | 0.22581 |
| 16385 | S21_17378776 | 21 | 17378776 | 24 | C | T | C | 0.8871 | T | 0.1129 | 5 | 0.16129 |
| 16386 | S21_17378836 | 21 | 17378836 | 60 | C | T | C | 0.90323 | T | 0.09677 | 6 | 0.19355 |
| 16387 | S21_17378924 | 21 | 17378924 | 88 | G | A | G | 0.79032 | A | 0.20968 | 11 | 0.35484 |
| 16388 | S21_17541386 | 21 | 17541386 | 162462 | C | T | T | 0.93548 | C | 0.06452 | 4 | 0.12903 |
| 16389 | S21_17895286 | 21 | 17895286 | 353900 | C | T | T | 0.83871 | C | 0.16129 | 10 | 0.32258 |
| 16390 | S21_17895324 | 21 | 17895324 | 38 | T | C | T | 0.91935 | C | 0.08065 | 5 | 0.16129 |
| 16391 | S21_17895325 | 21 | 17895325 | 1 | A | G | G | 0.83871 | A | 0.16129 | 10 | 0.32258 |
| 16392 | S21_17895337 | 21 | 17895337 | 12 | T | G | G | 0.83871 | T | 0.16129 | 10 | 0.32258 |
| 16393 | S21_17910834 | 21 | 17910834 | 15497 | C | T | C | 0.85484 | T | 0.14516 | 7 | 0.22581 |
| 16394 | S21_17910858 | 21 | 17910858 | 24 | G | A | A | 0.64516 | G | 0.35484 | 10 | 0.32258 |
| 16395 | S21_17910967 | 21 | 17910967 | 109 | G | A | G | 0.83871 | A | 0.16129 | 10 | 0.32258 |
| 16396 | S21_17910983 | 21 | 17910983 | 16 | A | G | A | 0.90323 | G | 0.09677 | 6 | 0.19355 |
| 16397 | S21_17911030 | 21 | 17911030 | 47 | C | T | C | 0.83871 | T | 0.16129 | 6 | 0.19355 |
| 16398 | S21_18164071 | 21 | 18164071 | 253041 | C | T | C | 0.80645 | T | 0.19355 | 8 | 0.25806 |
| 16399 | S21_18164082 | 21 | 18164082 | 11 | A | T | A | 0.80645 | T | 0.19355 | 8 | 0.25806 |
| 16400 | S21_18199476 | 21 | 18199476 | 35394 | T | G | T | 0.66129 | G | 0.33871 | 17 | 0.54839 |
| 16401 | S21_18199618 | 21 | 18199618 | 142 | A | G | G | 0.56452 | A | 0.43548 | 19 | 0.6129 |
| 16402 | S21_18211687 | 21 | 18211687 | 12069 | G | A | G | 0.87097 | A | 0.12903 | 6 | 0.19355 |
| 16403 | S21_18211729 | 21 | 18211729 | 42 | G | A | G | 0.75806 | A | 0.24194 | 11 | 0.35484 |
| 16404 | S21_18211806 | 21 | 18211806 | 77 | C | T | C | 0.85484 | T | 0.14516 | 7 | 0.22581 |
| 16405 | S21_18342067 | 21 | 18342067 | 130261 | C | T | T | 0.58065 | C | 0.41935 | 16 | 0.51613 |
| 16406 | S21_18353102 | 21 | 18353102 | 11035 | G | A | G | 0.6129 | A | 0.3871 | 14 | 0.45161 |
| 16407 | S21_18353105 | 21 | 18353105 | 3 | G | A | G | 0.95161 | A | 0.04839 | 3 | 0.09677 |
| 16408 | S21_18353216 | 21 | 18353216 | 111 | G | A | G | 0.95161 | A | 0.04839 | 3 | 0.09677 |
| 16409 | S21_18768438 | 21 | 18768438 | 415222 | C | T | C | 0.93548 | T | 0.06452 | 4 | 0.12903 |
| 16410 | S21_18768515 | 21 | 18768515 | 77 | G | A | G | 0.95161 | A | 0.04839 | 3 | 0.09677 |
| 16411 | S21_18902118 | 21 | 18902118 | 133603 | C | T | C | 0.64516 | T | 0.35484 | 16 | 0.51613 |
| 16412 | S21_18902234 | 21 | 18902234 | 116 | G | C | G | 0.91935 | C | 0.08065 | 5 | 0.16129 |
| 16413 | S21_18902249 | 21 | 18902249 | 15 | C | T | C | 0.8871 | T | 0.1129 | 7 | 0.22581 |
| 16414 | S21_18986162 | 21 | 18986162 | 83913 | C | A | C | 0.69355 | A | 0.30645 | 15 | 0.48387 |

| Site number | SNP name | Chromosome | Physical position | Physical distance from the previous SNP | Reference allele | Alternative allele | Major allele | Major allele frequency | Minor allele | Minor allele frequency | Number heterozygous | Heterozygosity |
|---|---|---|---|---|---|---|---|---|---|---|---|---|
| 16415 | S21_18986195 | 21 | 18986195 | 33 | G | A | G | 0.95161 | A | 0.04839 | 3 | 0.09677 |
| 16416 | S21_18986205 | 21 | 18986205 | 10 | T | C | T | 0.82258 | C | 0.17742 | 11 | 0.35484 |
| 16417 | S21_18986217 | 21 | 18986217 | 12 | G | A | G | 0.66129 | A | 0.33871 | 19 | 0.6129 |
| 16418 | S21_18986242 | 21 | 18986242 | 25 | G | A | G | 0.95161 | A | 0.04839 | 3 | 0.09677 |
| 16419 | S21_18986264 | 21 | 18986264 | 22 | A | C | A | 0.95161 | C | 0.04839 | 3 | 0.09677 |
| 16420 | S21_18986265 | 21 | 18986265 | 1 | G | A | G | 0.95161 | A | 0.04839 | 3 | 0.09677 |
| 16421 | S21_18986281 | 21 | 18986281 | 16 | T | C | T | 0.80645 | C | 0.19355 | 12 | 0.3871 |
| 16422 | S21_18986284 | 21 | 18986284 | 3 | T | C | T | 0.82258 | C | 0.17742 | 9 | 0.29032 |
| 16423 | S21_18986317 | 21 | 18986317 | 33 | G | A | G | 0.93548 | A | 0.06452 | 4 | 0.12903 |
| 16424 | S21_18986345 | 21 | 18986345 | 28 | T | A | T | 0.93548 | A | 0.06452 | 4 | 0.12903 |
| 16425 | S21_18986356 | 21 | 18986356 | 11 | G | T | G | 0.95161 | T | 0.04839 | 3 | 0.09677 |
| 16426 | S21_18986360 | 21 | 18986360 | 4 | T | C | T | 0.93548 | C | 0.06452 | 4 | 0.12903 |
| 16427 | S21_18986373 | 21 | 18986373 | 13 | A | G | A | 0.80645 | G | 0.19355 | 12 | 0.3871 |
| 16428 | S21_18986383 | 21 | 18986383 | 10 | A | C | A | 0.75806 | C | 0.24194 | 13 | 0.41935 |
| 16429 | S21_18986394 | 21 | 18986394 | 11 | C | G | C | 0.64516 | G | 0.35484 | 20 | 0.64516 |
| 16430 | S21_18986401 | 21 | 18986401 | 7 | G | T | G | 0.56452 | T | 0.43548 | 17 | 0.54839 |
| 16431 | S21_19492583 | 21 | 19492583 | 506182 | A | G | G | 0.54839 | A | 0.45161 | 14 | 0.45161 |
| 16432 | S21_19492589 | 21 | 19492589 | 6 | T | A | A | 0.74194 | T | 0.25806 | 14 | 0.45161 |
| 16433 | S21_19492593 | 21 | 19492593 | 4 | G | A | G | 0.79032 | A | 0.20968 | 11 | 0.35484 |
| 16434 | S21_19492679 | 21 | 19492679 | 86 | A | G | G | 0.91935 | A | 0.08065 | 5 | 0.16129 |
| 16435 | S21_19492706 | 21 | 19492706 | 27 | T | C | C | 0.91935 | T | 0.08065 | 5 | 0.16129 |
| 16436 | S21_19668358 | 21 | 19668358 | 175652 | G | T | G | 0.90323 | T | 0.09677 | 6 | 0.19355 |
| 16437 | S21_19668422 | 21 | 19668422 | 64 | C | G | C | 0.69355 | G | 0.30645 | 15 | 0.48387 |
| 16438 | S21_19668464 | 21 | 19668464 | 42 | C | T | C | 0.95161 | T | 0.04839 | 3 | 0.09677 |
| 16439 | S21_19749790 | 21 | 19749790 | 81326 | C | A | C | 0.91935 | A | 0.08065 | 5 | 0.16129 |
| 16440 | S21_19749965 | 21 | 19749965 | 175 | C | G | C | 0.56452 | G | 0.43548 | 11 | 0.35484 |
| 16441 | S21_19977422 | 21 | 19977422 | 227457 | C | T | C | 0.85484 | T | 0.14516 | 9 | 0.29032 |
| 16442 | S21_20015448 | 21 | 20015448 | 38026 | A | G | A | 0.87097 | G | 0.12903 | 6 | 0.19355 |
| 16443 | S21_20082400 | 21 | 20082400 | 66952 | G | A | G | 0.54839 | A | 0.45161 | 10 | 0.32258 |
| 16444 | S21_20924038 | 21 | 20924038 | 841638 | G | A | G | 0.80645 | A | 0.19355 | 10 | 0.32258 |
| 16445 | S21_21425721 | 21 | 21425721 | 501683 | G | A | G | 0.87097 | A | 0.12903 | 6 | 0.19355 |
| 16446 | S21_21438054 | 21 | 21438054 | 12333 | G | A | G | 0.8871 | A | 0.1129 | 7 | 0.22581 |
| 16447 | S21_21485432 | 21 | 21485432 | 47378 | T | C | C | 0.82258 | T | 0.17742 | 7 | 0.22581 |
| 16448 | S21_21485503 | 21 | 21485503 | 71 | T | C | T | 0.83871 | C | 0.16129 | 8 | 0.25806 |
| 16449 | S21_21485552 | 21 | 21485552 | 49 | T | G | T | 0.53226 | G | 0.46774 | 15 | 0.48387 |
| 16450 | S21_21852803 | 21 | 21852803 | 367251 | A | C | A | 0.6129 | C | 0.3871 | 16 | 0.51613 |
| 16451 | S21_21852811 | 21 | 21852811 | 8 | G | T | G | 0.6129 | T | 0.3871 | 16 | 0.51613 |
| 16452 | S21_21852883 | 21 | 21852883 | 72 | G | A | G | 0.6129 | A | 0.3871 | 16 | 0.51613 |
| 16453 | S21_21852895 | 21 | 21852895 | 12 | G | A | G | 0.90323 | A | 0.09677 | 6 | 0.19355 |
| 16454 | S21_21852907 | 21 | 21852907 | 12 | T | C | T | 0.93548 | C | 0.06452 | 2 | 0.06452 |
| 16455 | S21_21852932 | 21 | 21852932 | 25 | C | T | C | 0.90323 | T | 0.09677 | 6 | 0.19355 |
| 16456 | S21_21852948 | 21 | 21852948 | 16 | A | G | A | 0.93548 | G | 0.06452 | 2 | 0.06452 |
| 16457 | S21_21938419 | 21 | 21938419 | 85471 | C | T | C | 0.56452 | T | 0.43548 | 17 | 0.54839 |
| 16458 | S21_22409841 | 21 | 22409841 | 471422 | G | T | T | 0.72581 | G | 0.27419 | 13 | 0.41935 |
| 16459 | S21_22490908 | 21 | 22490908 | 81067 | C | G | G | 0.53226 | C | 0.46774 | 17 | 0.54839 |
| 16460 | S21_22574241 | 21 | 22574241 | 83333 | A | T | A | 0.56452 | T | 0.43548 | 15 | 0.48387 |
| 16461 | S21_22598358 | 21 | 22598358 | 24117 | A | T | A | 0.90323 | T | 0.09677 | 6 | 0.19355 |
| 16462 | S21_22598387 | 21 | 22598387 | 29 | G | T | G | 0.82258 | T | 0.17742 | 11 | 0.35484 |
| 16463 | S21_22598539 | 21 | 22598539 | 152 | T | C | T | 0.82258 | C | 0.17742 | 11 | 0.35484 |
| 16464 | S21_22598549 | 21 | 22598549 | 10 | C | T | C | 0.80645 | T | 0.19355 | 12 | 0.3871 |
| 16465 | S21_22598556 | 21 | 22598556 | 7 | T | G | T | 0.82258 | G | 0.17742 | 11 | 0.35484 |
| 16466 | S21_22598568 | 21 | 22598568 | 12 | A | G | A | 0.93548 | G | 0.06452 | 4 | 0.12903 |
| 16467 | S21_22598571 | 21 | 22598571 | 3 | C | T | C | 0.87097 | T | 0.12903 | 8 | 0.25806 |
| 16468 | S21_22795092 | 21 | 22795092 | 196521 | G | T | G | 0.77419 | T | 0.22581 | 14 | 0.45161 |

| Site number | SNP name | Chromosome | Physical position | Physical distance from the previous SNP | Reference allele | Alternative allele | Major allele | Major allele frequency | Minor allele | Minor allele frequency | Number heterozygous | Heterozygosity |
|---|---|---|---|---|---|---|---|---|---|---|---|---|
| 16469 | S21_22851723 | 21 | 22851723 | 56631 | C | A | C | 0.80645 | A | 0.19355 | 12 | 0.3871 |
| 16470 | S21_22851787 | 21 | 22851787 | 64 | A | G | A | 0.93548 | G | 0.06452 | 4 | 0.12903 |
| 16471 | S21_22851821 | 21 | 22851821 | 34 | G | A | G | 0.80645 | A | 0.19355 | 12 | 0.3871 |
| 16472 | S21_22851830 | 21 | 22851830 | 9 | G | C | G | 0.82258 | C | 0.17742 | 11 | 0.35484 |
| 16473 | S21_22851880 | 21 | 22851880 | 50 | G | A | G | 0.91935 | A | 0.08065 | 5 | 0.16129 |
| 16474 | S21_22854161 | 21 | 22854161 | 2281 | C | T | C | 0.87097 | T | 0.12903 | 8 | 0.25806 |
| 16475 | S21_22854240 | 21 | 22854240 | 79 | C | G | C | 0.80645 | G | 0.19355 | 12 | 0.3871 |
| 16476 | S21_22854283 | 21 | 22854283 | 43 | C | G | C | 0.95161 | G | 0.04839 | 3 | 0.09677 |
| 16477 | S21_23306530 | 21 | 23306530 | 452247 | C | T | C | 0.70968 | T | 0.29032 | 12 | 0.3871 |
| 16478 | S21_23306625 | 21 | 23306625 | 95 | C | T | C | 0.91935 | T | 0.08065 | 5 | 0.16129 |
| 16479 | S21_23375870 | 21 | 23375870 | 69245 | C | T | C | 0.85484 | T | 0.14516 | 7 | 0.22581 |
| 16480 | S21_23445975 | 21 | 23445975 | 70105 | C | A | C | 0.91935 | A | 0.08065 | 5 | 0.16129 |
| 16481 | S21_23586002 | 21 | 23586002 | 140027 | T | C | T | 0.91935 | C | 0.08065 | 5 | 0.16129 |
| 16482 | S21_23586173 | 21 | 23586173 | 171 | G | C | G | 0.90323 | C | 0.09677 | 6 | 0.19355 |
| 16483 | S21_23586192 | 21 | 23586192 | 19 | T | C | T | 0.95161 | C | 0.04839 | 3 | 0.09677 |
| 16484 | S21_23713472 | 21 | 23713472 | 127280 | T | C | T | 0.69355 | C | 0.30645 | 17 | 0.54839 |
| 16485 | S21_24281911 | 21 | 24281911 | 568439 | A | G | G | 0.53226 | A | 0.46774 | 17 | 0.54839 |
| 16486 | S21_24284551 | 21 | 24284551 | 2640 | G | A | G | 0.54839 | A | 0.45161 | 20 | 0.64516 |
| 16487 | S21_24284605 | 21 | 24284605 | 54 | A | G | G | 0.62903 | A | 0.37097 | 17 | 0.54839 |
| 16488 | S21_24527724 | 21 | 24527724 | 243119 | C | T | C | 0.75806 | T | 0.24194 | 11 | 0.35484 |
| 16489 | S21_24527863 | 21 | 24527863 | 139 | T | C | T | 0.8871 | C | 0.1129 | 7 | 0.22581 |
| 16490 | S21_24527901 | 21 | 24527901 | 38 | A | G | A | 0.93548 | G | 0.06452 | 4 | 0.12903 |
| 16491 | S21_24587111 | 21 | 24587111 | 59210 | T | C | T | 0.77419 | C | 0.22581 | 12 | 0.3871 |
| 16492 | S21_24587164 | 21 | 24587164 | 53 | G | A | G | 0.58065 | A | 0.41935 | 18 | 0.58065 |
| 16493 | S21_24587168 | 21 | 24587168 | 4 | A | G | A | 0.93548 | G | 0.06452 | 4 | 0.12903 |
| 16494 | S21_24587172 | 21 | 24587172 | 4 | C | A | C | 0.93548 | A | 0.06452 | 4 | 0.12903 |
| 16495 | S21_24587174 | 21 | 24587174 | 2 | A | C | A | 0.93548 | C | 0.06452 | 4 | 0.12903 |
| 16496 | S21_24587181 | 21 | 24587181 | 7 | C | T | C | 0.93548 | T | 0.06452 | 4 | 0.12903 |
| 16497 | S21_24587183 | 21 | 24587183 | 2 | C | G | C | 0.80645 | G | 0.19355 | 12 | 0.3871 |
| 16498 | S21_24587184 | 21 | 24587184 | 1 | G | A | G | 0.80645 | A | 0.19355 | 12 | 0.3871 |
| 16499 | S21_24587203 | 21 | 24587203 | 19 | G | A | G | 0.93548 | A | 0.06452 | 4 | 0.12903 |
| 16500 | S21_24587254 | 21 | 24587254 | 51 | C | T | C | 0.58065 | T | 0.41935 | 18 | 0.58065 |
| 16501 | S21_24587255 | 21 | 24587255 | 1 | G | A | G | 0.8871 | A | 0.1129 | 7 | 0.22581 |
| 16502 | S21_24615208 | 21 | 24615208 | 27953 | T | A | T | 0.80645 | A | 0.19355 | 10 | 0.32258 |
| 16503 | S21_24615242 | 21 | 24615242 | 34 | G | A | G | 0.8871 | A | 0.1129 | 7 | 0.22581 |
| 16504 | S21_24615276 | 21 | 24615276 | 34 | C | T | C | 0.74194 | T | 0.25806 | 16 | 0.51613 |
| 16505 | S21_24615286 | 21 | 24615286 | 10 | A | T | A | 0.75806 | T | 0.24194 | 15 | 0.48387 |
| 16506 | S21_24648730 | 21 | 24648730 | 33444 | C | G | C | 0.93548 | G | 0.06452 | 4 | 0.12903 |
| 16507 | S21_24648779 | 21 | 24648779 | 49 | A | C | A | 0.87097 | C | 0.12903 | 8 | 0.25806 |
| 16508 | S21_24648781 | 21 | 24648781 | 2 | A | C | A | 0.95161 | C | 0.04839 | 3 | 0.09677 |
| 16509 | S21_24648928 | 21 | 24648928 | 147 | C | T | C | 0.70968 | T | 0.29032 | 18 | 0.58065 |
| 16510 | S21_24648945 | 21 | 24648945 | 17 | G | A | G | 0.75806 | A | 0.24194 | 15 | 0.48387 |
| 16511 | S21_24714772 | 21 | 24714772 | 65827 | A | G | G | 0.8871 | A | 0.1129 | 7 | 0.22581 |
| 16512 | S21_24827299 | 21 | 24827299 | 112527 | C | A | C | 0.8871 | A | 0.1129 | 7 | 0.22581 |
| 16513 | S21_24827311 | 21 | 24827311 | 12 | G | C | G | 0.85484 | C | 0.14516 | 7 | 0.22581 |
| 16514 | S21_24827332 | 21 | 24827332 | 21 | A | G | G | 0.83871 | A | 0.16129 | 8 | 0.25806 |
| 16515 | S21_24827351 | 21 | 24827351 | 19 | G | T | G | 0.93548 | T | 0.06452 | 4 | 0.12903 |
| 16516 | S21_24827370 | 21 | 24827370 | 19 | C | T | C | 0.8871 | T | 0.1129 | 7 | 0.22581 |
| 16517 | S21_24827484 | 21 | 24827484 | 114 | T | A | A | 0.95161 | T | 0.04839 | 3 | 0.09677 |
| 16518 | S21_25262617 | 21 | 25262617 | 435133 | T | C | C | 0.74194 | T | 0.25806 | 14 | 0.45161 |
| 16519 | S21_25288175 | 21 | 25288175 | 25558 | A | G | A | 0.77419 | G | 0.22581 | 8 | 0.25806 |
| 16520 | S21_25288249 | 21 | 25288249 | 74 | C | T | C | 0.72581 | T | 0.27419 | 11 | 0.35484 |
| 16521 | S21_25304480 | 21 | 25304480 | 16231 | C | T | C | 0.91935 | T | 0.08065 | 5 | 0.16129 |
| 16522 | S21_25304566 | 21 | 25304566 | 86 | G | A | A | 0.53226 | G | 0.46774 | 13 | 0.41935 |

| Site number | SNP name | Chromosome | Physical position | Physical distance from the previous SNP | Reference allele | Alternative allele | Major allele | Major allele frequency | Minor allele | Minor allele frequency | Number heterozygous | Heterozygosity |
|---|---|---|---|---|---|---|---|---|---|---|---|---|
| 16523 | S21_25604706 | 21 | 25604706 | 300140 | A | T | A | 0.93548 | T | 0.06452 | 4 | 0.12903 |
| 16524 | S21_25787673 | 21 | 25787673 | 182967 | G | A | G | 0.8871 | A | 0.1129 | 5 | 0.16129 |
| 16525 | S21_25787823 | 21 | 25787823 | 150 | G | A | G | 0.87097 | A | 0.12903 | 6 | 0.19355 |
| 16526 | S21_25787851 | 21 | 25787851 | 28 | G | A | G | 0.87097 | A | 0.12903 | 8 | 0.25806 |
| 16527 | S21_26028285 | 21 | 26028285 | 240434 | G | C | G | 0.67742 | C | 0.32258 | 16 | 0.51613 |
| 16528 | S21_26105331 | 21 | 26105331 | 77046 | T | G | T | 0.74194 | G | 0.25806 | 12 | 0.3871 |
| 16529 | S21_26105394 | 21 | 26105394 | 63 | A | G | A | 0.62903 | G | 0.37097 | 17 | 0.54839 |
| 16530 | S21_26297068 | 21 | 26297068 | 191674 | C | T | C | 0.95161 | T | 0.04839 | 3 | 0.09677 |
| 16531 | S21_26517551 | 21 | 26517551 | 220483 | G | C | C | 0.93548 | G | 0.06452 | 4 | 0.12903 |
| 16532 | S21_26625165 | 21 | 26625165 | 107614 | C | T | T | 0.64516 | C | 0.35484 | 12 | 0.3871 |
| 16533 | S21_26625259 | 21 | 26625259 | 94 | T | C | T | 0.90323 | C | 0.09677 | 6 | 0.19355 |
| 16534 | S21_26625268 | 21 | 26625268 | 9 | T | C | T | 0.75806 | C | 0.24194 | 15 | 0.48387 |
| 16535 | S21_26711248 | 21 | 26711248 | 85980 | C | T | C | 0.8871 | T | 0.1129 | 5 | 0.16129 |
| 16536 | S21_26790867 | 21 | 26790867 | 79619 | G | T | T | 0.67742 | G | 0.32258 | 12 | 0.3871 |
| 16537 | S21_26790961 | 21 | 26790961 | 94 | A | G | G | 0.67742 | A | 0.32258 | 12 | 0.3871 |
| 16538 | S21_26790975 | 21 | 26790975 | 14 | G | A | G | 0.83871 | A | 0.16129 | 10 | 0.32258 |
| 16539 | S21_26886535 | 21 | 26886535 | 95560 | G | A | G | 0.90323 | A | 0.09677 | 6 | 0.19355 |
| 16540 | S21_27281513 | 21 | 27281513 | 394978 | T | A | T | 0.95161 | A | 0.04839 | 3 | 0.09677 |
| 16541 | S21_27281550 | 21 | 27281550 | 37 | G | A | G | 0.8871 | A | 0.1129 | 5 | 0.16129 |
| 16542 | S21_27286807 | 21 | 27286807 | 5257 | A | G | A | 0.8871 | G | 0.1129 | 5 | 0.16129 |
| 16543 | S21_27368815 | 21 | 27368815 | 82008 | C | T | C | 0.72581 | T | 0.27419 | 11 | 0.35484 |
| 16544 | S21_27368833 | 21 | 27368833 | 18 | C | T | C | 0.95161 | T | 0.04839 | 3 | 0.09677 |
| 16545 | S21_27368916 | 21 | 27368916 | 83 | T | C | C | 0.82258 | T | 0.17742 | 11 | 0.35484 |
| 16546 | S21_27591284 | 21 | 27591284 | 222368 | G | A | G | 0.85484 | A | 0.14516 | 5 | 0.16129 |
| 16547 | S21_27591461 | 21 | 27591461 | 177 | C | T | C | 0.93548 | T | 0.06452 | 4 | 0.12903 |
| 16548 | S21_27648485 | 21 | 27648485 | 57024 | G | C | G | 0.91935 | C | 0.08065 | 3 | 0.09677 |
| 16549 | S21_27653622 | 21 | 27653622 | 5137 | A | C | A | 0.93548 | C | 0.06452 | 4 | 0.12903 |
| 16550 | S21_27653705 | 21 | 27653705 | 83 | G | A | G | 0.91935 | A | 0.08065 | 5 | 0.16129 |
| 16551 | S21_27792442 | 21 | 27792442 | 138737 | C | T | C | 0.75806 | T | 0.24194 | 9 | 0.29032 |
| 16552 | S21_27792484 | 21 | 27792484 | 42 | A | C | C | 0.59677 | A | 0.40323 | 15 | 0.48387 |
| 16553 | S21_28051496 | 21 | 28051496 | 259012 | G | C | G | 0.95161 | C | 0.04839 | 3 | 0.09677 |
| 16554 | S21_28128027 | 21 | 28128027 | 76531 | G | A | G | 0.59677 | A | 0.40323 | 15 | 0.48387 |
| 16555 | S21_28187833 | 21 | 28187833 | 59806 | G | A | G | 0.83871 | A | 0.16129 | 8 | 0.25806 |
| 16556 | S21_28189082 | 21 | 28189082 | 1249 | G | A | G | 0.51613 | A | 0.48387 | 14 | 0.45161 |
| 16557 | S21_28826138 | 21 | 28826138 | 637056 | A | G | A | 0.95161 | G | 0.04839 | 3 | 0.09677 |
| 16558 | S21_28826224 | 21 | 28826224 | 86 | G | A | G | 0.87097 | A | 0.12903 | 6 | 0.19355 |
| 16559 | S21_29564735 | 21 | 29564735 | 738511 | A | G | G | 0.83871 | A | 0.16129 | 8 | 0.25806 |
| 16560 | S21_29912295 | 21 | 29912295 | 347560 | T | C | T | 0.80645 | C | 0.19355 | 12 | 0.3871 |
| 16561 | S21_29912436 | 21 | 29912436 | 141 | A | G | A | 0.91935 | G | 0.08065 | 3 | 0.09677 |
| 16562 | S21_30058474 | 21 | 30058474 | 146038 | C | T | T | 0.91935 | C | 0.08065 | 5 | 0.16129 |
| 16563 | S21_30482633 | 21 | 30482633 | 424159 | C | A | C | 0.72581 | A | 0.27419 | 15 | 0.48387 |
| 16564 | S21_30559007 | 21 | 30559007 | 76374 | G | A | A | 0.51613 | G | 0.48387 | 16 | 0.51613 |
| 16565 | S21_30559147 | 21 | 30559147 | 140 | G | A | G | 0.90323 | A | 0.09677 | 6 | 0.19355 |
| 16566 | S21_30712067 | 21 | 30712067 | 152920 | A | G | A | 0.83871 | G | 0.16129 | 8 | 0.25806 |
| 16567 | S21_30925037 | 21 | 30925037 | 212970 | A | C | C | 0.90323 | A | 0.09677 | 6 | 0.19355 |
| 16568 | S21_30962599 | 21 | 30962599 | 37562 | G | A | G | 0.95161 | A | 0.04839 | 3 | 0.09677 |
| 16569 | S21_30982265 | 21 | 30982265 | 19666 | G | A | G | 0.83871 | A | 0.16129 | 8 | 0.25806 |
| 16570 | S21_30982312 | 21 | 30982312 | 47 | A | C | A | 0.91935 | C | 0.08065 | 5 | 0.16129 |
| 16571 | S21_31206858 | 21 | 31206858 | 224546 | G | A | G | 0.91935 | A | 0.08065 | 3 | 0.09677 |
| 16572 | S21_31206896 | 21 | 31206896 | 38 | A | G | A | 0.8871 | G | 0.1129 | 7 | 0.22581 |
| 16573 | S21_31206939 | 21 | 31206939 | 43 | G | A | G | 0.91935 | A | 0.08065 | 5 | 0.16129 |
| 16574 | S21_31324228 | 21 | 31324228 | 117289 | C | T | T | 0.74194 | C | 0.25806 | 14 | 0.45161 |
| 16575 | S21_31662461 | 21 | 31662461 | 338233 | A | G | A | 0.79032 | G | 0.20968 | 7 | 0.22581 |
| 16576 | S21_31662562 | 21 | 31662562 | 101 | C | T | C | 0.79032 | T | 0.20968 | 7 | 0.22581 |

| Site number | SNP name | Chromosome | Physical position | Physical distance from the previous SNP | Reference allele | Alternative allele | Major allele | Major allele frequency | Minor allele | Minor allele frequency | Number heterozygous | Heterozygosity |
|---|---|---|---|---|---|---|---|---|---|---|---|---|
| 16577 | S21_31662565 | 21 | 31662565 | 3 | C | T | C | 0.79032 | T | 0.20968 | 7 | 0.22581 |
| 16578 | S21_32240737 | 21 | 32240737 | 578172 | C | T | C | 0.95161 | T | 0.04839 | 3 | 0.09677 |
| 16579 | S21_32240843 | 21 | 32240843 | 106 | A | G | G | 0.91935 | A | 0.08065 | 5 | 0.16129 |
| 16580 | S21_32507575 | 21 | 32507575 | 266732 | A | G | A | 0.87097 | G | 0.12903 | 8 | 0.25806 |
| 16581 | S21_32756098 | 21 | 32756098 | 248523 | T | G | T | 0.91935 | G | 0.08065 | 5 | 0.16129 |
| 16582 | S21_32845708 | 21 | 32845708 | 89610 | A | G | A | 0.51613 | G | 0.48387 | 18 | 0.58065 |
| 16583 | S21_33000964 | 21 | 33000964 | 155256 | A | G | A | 0.91935 | G | 0.08065 | 5 | 0.16129 |
| 16584 | S21_33000975 | 21 | 33000975 | 11 | G | A | G | 0.93548 | A | 0.06452 | 4 | 0.12903 |
| 16585 | S21_33157525 | 21 | 33157525 | 156550 | A | T | T | 0.91935 | A | 0.08065 | 3 | 0.09677 |
| 16586 | S21_33602475 | 21 | 33602475 | 444950 | G | A | G | 0.91935 | A | 0.08065 | 5 | 0.16129 |
| 16587 | S21_33602498 | 21 | 33602498 | 23 | C | T | T | 0.85484 | C | 0.14516 | 9 | 0.29032 |
| 16588 | S21_33755347 | 21 | 33755347 | 152849 | T | C | T | 0.90323 | C | 0.09677 | 6 | 0.19355 |
| 16589 | S21_33886834 | 21 | 33886834 | 131487 | C | T | C | 0.91935 | T | 0.08065 | 5 | 0.16129 |
| 16590 | S21_33887026 | 21 | 33887026 | 192 | G | A | G | 0.87097 | A | 0.12903 | 6 | 0.19355 |
| 16591 | S21_33964311 | 21 | 33964311 | 77285 | G | A | A | 0.67742 | G | 0.32258 | 18 | 0.58065 |
| 16592 | S21_34069332 | 21 | 34069332 | 105021 | C | T | C | 0.70968 | T | 0.29032 | 10 | 0.32258 |
| 16593 | S21_34216065 | 21 | 34216065 | 146733 | A | G | A | 0.85484 | G | 0.14516 | 7 | 0.22581 |
| 16594 | S21_34216074 | 21 | 34216074 | 9 | A | G | A | 0.82258 | G | 0.17742 | 7 | 0.22581 |
| 16595 | S21_34216215 | 21 | 34216215 | 141 | T | G | T | 0.82258 | G | 0.17742 | 7 | 0.22581 |
| 16596 | S21_34493081 | 21 | 34493081 | 276866 | G | A | G | 0.91935 | A | 0.08065 | 5 | 0.16129 |
| 16597 | S21_34828398 | 21 | 34828398 | 335317 | G | A | G | 0.69355 | A | 0.30645 | 15 | 0.48387 |
| 16598 | S21_34828525 | 21 | 34828525 | 127 | C | T | T | 0.93548 | C | 0.06452 | 4 | 0.12903 |
| 16599 | S21_34828541 | 21 | 34828541 | 16 | G | A | A | 0.64516 | G | 0.35484 | 18 | 0.58065 |
| 16600 | S21_35065115 | 21 | 35065115 | 236574 | G | C | G | 0.95161 | C | 0.04839 | 3 | 0.09677 |
| 16601 | S21_35845672 | 21 | 35845672 | 780557 | T | C | C | 0.72581 | T | 0.27419 | 11 | 0.35484 |
| 16602 | S21_35845826 | 21 | 35845826 | 154 | A | G | G | 0.72581 | A | 0.27419 | 11 | 0.35484 |
| 16603 | S21_35845867 | 21 | 35845867 | 41 | A | C | C | 0.72581 | A | 0.27419 | 11 | 0.35484 |
| 16604 | S21_35845873 | 21 | 35845873 | 6 | T | C | T | 0.8871 | C | 0.1129 | 5 | 0.16129 |
| 16605 | S21_36100883 | 21 | 36100883 | 255010 | A | C | A | 0.62903 | C | 0.37097 | 17 | 0.54839 |
| 16606 | S21_36100952 | 21 | 36100952 | 69 | C | T | C | 0.8871 | T | 0.1129 | 7 | 0.22581 |
| 16607 | S21_36100959 | 21 | 36100959 | 7 | T | A | T | 0.77419 | A | 0.22581 | 12 | 0.3871 |
| 16608 | S21_36101014 | 21 | 36101014 | 55 | C | A | C | 0.66129 | A | 0.33871 | 15 | 0.48387 |
| 16609 | S21_37378567 | 21 | 37378567 | 1277553 | T | C | C | 0.77419 | T | 0.22581 | 12 | 0.3871 |
| 16610 | S21_37378573 | 21 | 37378573 | 6 | G | C | G | 0.77419 | C | 0.22581 | 12 | 0.3871 |
| 16611 | S21_37378590 | 21 | 37378590 | 17 | A | G | A | 0.8871 | G | 0.1129 | 7 | 0.22581 |
| 16612 | S21_38350442 | 21 | 38350442 | 971852 | A | C | C | 0.53226 | A | 0.46774 | 11 | 0.35484 |
| 16613 | S21_38350451 | 21 | 38350451 | 9 | G | A | G | 0.75806 | A | 0.24194 | 13 | 0.41935 |
| 16614 | S21_38350609 | 21 | 38350609 | 158 | C | T | C | 0.74194 | T | 0.25806 | 14 | 0.45161 |
| 16615 | S21_38350619 | 21 | 38350619 | 10 | C | T | C | 0.74194 | T | 0.25806 | 14 | 0.45161 |
| 16616 | S21_38606794 | 21 | 38606794 | 256175 | C | T | C | 0.79032 | T | 0.20968 | 13 | 0.41935 |
| 16617 | S21_39075785 | 21 | 39075785 | 468991 | A | G | A | 0.74194 | G | 0.25806 | 14 | 0.45161 |
| 16618 | S21_39075850 | 21 | 39075850 | 65 | C | A | C | 0.8871 | A | 0.1129 | 7 | 0.22581 |
| 16619 | S21_39075910 | 21 | 39075910 | 60 | T | C | T | 0.93548 | C | 0.06452 | 4 | 0.12903 |
| 16620 | S21_39075924 | 21 | 39075924 | 14 | A | G | A | 0.93548 | G | 0.06452 | 4 | 0.12903 |
| 16621 | S21_39075953 | 21 | 39075953 | 29 | C | T | T | 0.67742 | C | 0.32258 | 14 | 0.45161 |
| 16622 | S21_39075962 | 21 | 39075962 | 9 | G | A | G | 0.93548 | A | 0.06452 | 4 | 0.12903 |
| 16623 | S21_39558368 | 21 | 39558368 | 482406 | C | T | C | 0.91935 | T | 0.08065 | 5 | 0.16129 |
| 16624 | S21_39558484 | 21 | 39558484 | 116 | A | G | G | 0.77419 | A | 0.22581 | 12 | 0.3871 |
| 16625 | S21_39810941 | 21 | 39810941 | 252457 | C | T | C | 0.59677 | T | 0.40323 | 13 | 0.41935 |
| 16626 | S21_39948640 | 21 | 39948640 | 137699 | T | C | T | 0.95161 | C | 0.04839 | 3 | 0.09677 |
| 16627 | S21_39948666 | 21 | 39948666 | 26 | A | G | A | 0.79032 | G | 0.20968 | 9 | 0.29032 |
| 16628 | S21_40286673 | 21 | 40286673 | 338007 | T | C | T | 0.6129 | C | 0.3871 | 18 | 0.58065 |
| 16629 | S21_40286739 | 21 | 40286739 | 66 | C | T | C | 0.91935 | T | 0.08065 | 5 | 0.16129 |
| 16630 | S21_40286873 | 21 | 40286873 | 134 | C | T | C | 0.90323 | T | 0.09677 | 6 | 0.19355 |

| Site number | SNP name | Chromosome | Physical position | Physical distance from the previous SNP | Reference allele | Alternative allele | Major allele | Major allele frequency | Minor allele | Minor allele frequency | Number heterozygous | Heterozygosity |
|---|---|---|---|---|---|---|---|---|---|---|---|---|
| 16631 | S21_40286877 | 21 | 40286877 | 4 | G | T | G | 0.6129 | T | 0.3871 | 18 | 0.58065 |
| 16632 | S21_40597194 | 21 | 40597194 | 310317 | G | A | G | 0.77419 | A | 0.22581 | 10 | 0.32258 |
| 16633 | S21_40597372 | 21 | 40597372 | 178 | C | T | C | 0.91935 | T | 0.08065 | 5 | 0.16129 |
| 16634 | S21_42208233 | 21 | 42208233 | 1610861 | C | T | C | 0.93548 | T | 0.06452 | 4 | 0.12903 |
| 16635 | S21_42501689 | 21 | 42501689 | 293456 | G | A | G | 0.82258 | A | 0.17742 | 7 | 0.22581 |
| 16636 | S21_43485618 | 21 | 43485618 | 983929 | G | A | G | 0.93548 | A | 0.06452 | 4 | 0.12903 |
| 16637 | S21_43930860 | 21 | 43930860 | 445242 | G | T | G | 0.87097 | T | 0.12903 | 6 | 0.19355 |
| 16638 | S21_43930888 | 21 | 43930888 | 28 | T | A | T | 0.66129 | A | 0.33871 | 13 | 0.41935 |
| 16639 | S21_43930934 | 21 | 43930934 | 46 | T | C | T | 0.66129 | C | 0.33871 | 13 | 0.41935 |
| 16640 | S21_44088433 | 21 | 44088433 | 157499 | C | T | C | 0.87097 | T | 0.12903 | 6 | 0.19355 |
| 16641 | S21_44088675 | 21 | 44088675 | 242 | A | T | A | 0.66129 | T | 0.33871 | 17 | 0.54839 |
| 16642 | S21_44229807 | 21 | 44229807 | 141132 | C | G | C | 0.80645 | G | 0.19355 | 8 | 0.25806 |
| 16643 | S21_44289536 | 21 | 44289536 | 59729 | C | T | C | 0.87097 | T | 0.12903 | 8 | 0.25806 |
| 16644 | S21_44289544 | 21 | 44289544 | 8 | T | C | T | 0.87097 | C | 0.12903 | 8 | 0.25806 |
| 16645 | S21_44289564 | 21 | 44289564 | 20 | G | A | G | 0.8871 | A | 0.1129 | 5 | 0.16129 |
| 16646 | S21_44715209 | 21 | 44715209 | 425645 | C | A | C | 0.87097 | A | 0.12903 | 8 | 0.25806 |
| 16647 | S21_45829115 | 21 | 45829115 | 1113906 | A | G | A | 0.95161 | G | 0.04839 | 3 | 0.09677 |
| 16648 | S21_46562991 | 21 | 46562991 | 733876 | A | G | A | 0.90323 | G | 0.09677 | 6 | 0.19355 |
| 16649 | S21_46713407 | 21 | 46713407 | 150416 | C | T | C | 0.95161 | T | 0.04839 | 3 | 0.09677 |
| 16650 | S21_46713458 | 21 | 46713458 | 51 | G | C | G | 0.91935 | C | 0.08065 | 5 | 0.16129 |
| 16651 | S21_46822933 | 21 | 46822933 | 109475 | T | G | T | 0.8871 | G | 0.1129 | 7 | 0.22581 |
| 16652 | S21_46823015 | 21 | 46823015 | 82 | G | A | G | 0.87097 | A | 0.12903 | 8 | 0.25806 |
| 16653 | S21_47070817 | 21 | 47070817 | 247802 | G | A | G | 0.58065 | A | 0.41935 | 12 | 0.3871 |
| 16654 | S21_47070889 | 21 | 47070889 | 72 | G | A | G | 0.75806 | A | 0.24194 | 9 | 0.29032 |
| 16655 | S21_47259422 | 21 | 47259422 | 188533 | G | A | G | 0.95161 | A | 0.04839 | 3 | 0.09677 |
| 16656 | S21_47259466 | 21 | 47259466 | 44 | C | T | T | 0.56452 | C | 0.43548 | 15 | 0.48387 |
| 16657 | S21_47259478 | 21 | 47259478 | 12 | C | T | C | 0.93548 | T | 0.06452 | 4 | 0.12903 |
| 16658 | S21_47259520 | 21 | 47259520 | 42 | G | A | A | 0.56452 | G | 0.43548 | 15 | 0.48387 |
| 16659 | S21_47449215 | 21 | 47449215 | 189695 | G | A | G | 0.83871 | A | 0.16129 | 8 | 0.25806 |
| 16660 | S21_47662542 | 21 | 47662542 | 213327 | A | C | C | 0.77419 | A | 0.22581 | 12 | 0.3871 |
| 16661 | S21_47863146 | 21 | 47863146 | 200604 | G | A | G | 0.93548 | A | 0.06452 | 4 | 0.12903 |
| 16662 | S21_47863221 | 21 | 47863221 | 75 | C | T | C | 0.93548 | T | 0.06452 | 4 | 0.12903 |
| 16663 | S21_48017870 | 21 | 48017870 | 154649 | T | G | G | 0.56452 | T | 0.43548 | 15 | 0.48387 |
| 16664 | S21_48307591 | 21 | 48307591 | 289721 | C | T | C | 0.75806 | T | 0.24194 | 9 | 0.29032 |
| 16665 | S21_48432115 | 21 | 48432115 | 124524 | C | T | C | 0.95161 | T | 0.04839 | 3 | 0.09677 |
| 16666 | S21_48432193 | 21 | 48432193 | 78 | G | A | G | 0.95161 | A | 0.04839 | 3 | 0.09677 |
| 16667 | S21_48432272 | 21 | 48432272 | 79 | G | A | G | 0.95161 | A | 0.04839 | 3 | 0.09677 |
| 16668 | S21_48432287 | 21 | 48432287 | 15 | G | A | G | 0.6129 | A | 0.3871 | 12 | 0.3871 |
| 16669 | S21_48665682 | 21 | 48665682 | 233395 | C | T | C | 0.90323 | T | 0.09677 | 6 | 0.19355 |
| 16670 | S21_48665689 | 21 | 48665689 | 7 | A | G | A | 0.95161 | G | 0.04839 | 3 | 0.09677 |
| 16671 | S21_48665711 | 21 | 48665711 | 22 | T | C | T | 0.91935 | C | 0.08065 | 5 | 0.16129 |
| 16672 | S21_48665852 | 21 | 48665852 | 141 | A | G | G | 0.51613 | A | 0.48387 | 14 | 0.45161 |
| 16673 | S21_49252934 | 21 | 49252934 | 587082 | C | T | C | 0.83871 | T | 0.16129 | 10 | 0.32258 |
| 16674 | S21_49870390 | 21 | 49870390 | 617456 | A | G | A | 0.72581 | G | 0.27419 | 13 | 0.41935 |
| 16675 | S21_49870410 | 21 | 49870410 | 20 | G | A | G | 0.95161 | A | 0.04839 | 3 | 0.09677 |
| 16676 | S21_50023194 | 21 | 50023194 | 152784 | C | T | C | 0.80645 | T | 0.19355 | 12 | 0.3871 |
| 16677 | S21_50258846 | 21 | 50258846 | 235652 | T | C | T | 0.79032 | C | 0.20968 | 9 | 0.29032 |
| 16678 | S21_50586068 | 21 | 50586068 | 327222 | G | C | G | 0.95161 | C | 0.04839 | 3 | 0.09677 |
| 16679 | S21_50586155 | 21 | 50586155 | 87 | G | A | G | 0.91935 | A | 0.08065 | 5 | 0.16129 |
| 16680 | S21_51933235 | 21 | 51933235 | 1347080 | T | C | T | 0.85484 | C | 0.14516 | 9 | 0.29032 |
| 16681 | S21_51933258 | 21 | 51933258 | 23 | T | C | T | 0.93548 | C | 0.06452 | 4 | 0.12903 |
| 16682 | S21_51933264 | 21 | 51933264 | 6 | G | A | A | 0.8871 | G | 0.1129 | 7 | 0.22581 |
| 16683 | S21_51933287 | 21 | 51933287 | 23 | G | A | G | 0.95161 | A | 0.04839 | 3 | 0.09677 |
| 16684 | S21_51933309 | 21 | 51933309 | 22 | C | T | T | 0.8871 | C | 0.1129 | 7 | 0.22581 |

| Site number | SNP name | Chromosome | Physical position | Physical distance from the previous SNP | Reference allele | Alternative allele | Major allele | Major allele frequency | Minor allele | Minor allele frequency | Number heterozygous | Heterozygosity |
|---|---|---|---|---|---|---|---|---|---|---|---|---|
| 16685 | S21_51933340 | 21 | 51933340 | 31 | C | T | C | 0.93548 | T | 0.06452 | 4 | 0.12903 |
| 16686 | S21_51994504 | 21 | 51994504 | 61164 | G | A | G | 0.95161 | A | 0.04839 | 3 | 0.09677 |
| 16687 | S21_51994727 | 21 | 51994727 | 223 | C | T | C | 0.91935 | T | 0.08065 | 3 | 0.09677 |
| 16688 | S21_52013938 | 21 | 52013938 | 19211 | A | G | A | 0.95161 | G | 0.04839 | 3 | 0.09677 |
| 16689 | S21_52013939 | 21 | 52013939 | 1 | T | C | T | 0.83871 | C | 0.16129 | 10 | 0.32258 |
| 16690 | S21_52013957 | 21 | 52013957 | 18 | A | G | A | 0.53226 | G | 0.46774 | 11 | 0.35484 |
| 16691 | S21_52013994 | 21 | 52013994 | 37 | C | T | C | 0.91935 | T | 0.08065 | 5 | 0.16129 |
| 16692 | S21_52014012 | 21 | 52014012 | 18 | T | G | T | 0.90323 | G | 0.09677 | 6 | 0.19355 |
| 16693 | S21_52302500 | 21 | 52302500 | 288488 | T | C | T | 0.77419 | C | 0.22581 | 10 | 0.32258 |
| 16694 | S21_52302522 | 21 | 52302522 | 22 | T | C | T | 0.6129 | C | 0.3871 | 16 | 0.51613 |
| 16695 | S21_52302527 | 21 | 52302527 | 5 | A | C | A | 0.70968 | C | 0.29032 | 14 | 0.45161 |
| 16696 | S21_52706602 | 21 | 52706602 | 404075 | A | G | A | 0.69355 | G | 0.30645 | 15 | 0.48387 |
| 16697 | S21_52706762 | 21 | 52706762 | 160 | T | G | T | 0.51613 | G | 0.48387 | 16 | 0.51613 |
| 16698 | S21_52706763 | 21 | 52706763 | 1 | T | C | T | 0.79032 | C | 0.20968 | 9 | 0.29032 |
| 16699 | S21_52706818 | 21 | 52706818 | 55 | T | G | T | 0.79032 | G | 0.20968 | 9 | 0.29032 |
| 16700 | S21_53235161 | 21 | 53235161 | 528343 | C | T | C | 0.56452 | T | 0.43548 | 13 | 0.41935 |
| 16701 | S21_53235169 | 21 | 53235169 | 8 | G | A | G | 0.72581 | A | 0.27419 | 15 | 0.48387 |
| 16702 | S21_53235284 | 21 | 53235284 | 115 | C | A | C | 0.93548 | A | 0.06452 | 4 | 0.12903 |
| 16703 | S21_53235302 | 21 | 53235302 | 18 | T | C | T | 0.56452 | C | 0.43548 | 13 | 0.41935 |
| 16704 | S21_53235308 | 21 | 53235308 | 6 | C | T | C | 0.72581 | T | 0.27419 | 15 | 0.48387 |
| 16705 | S21_53235401 | 21 | 53235401 | 93 | A | T | A | 0.83871 | T | 0.16129 | 8 | 0.25806 |
| 16706 | S21_53570984 | 21 | 53570984 | 335583 | G | A | G | 0.74194 | A | 0.25806 | 14 | 0.45161 |
| 16707 | S21_53571005 | 21 | 53571005 | 21 | T | C | T | 0.54839 | C | 0.45161 | 16 | 0.51613 |
| 16708 | S21_54353098 | 21 | 54353098 | 782093 | T | C | C | 0.93548 | T | 0.06452 | 4 | 0.12903 |
| 16709 | S21_54353107 | 21 | 54353107 | 9 | A | G | G | 0.83871 | A | 0.16129 | 10 | 0.32258 |
| 16710 | S21_54353154 | 21 | 54353154 | 47 | C | G | G | 0.80645 | C | 0.19355 | 12 | 0.3871 |
| 16711 | S21_54373613 | 21 | 54373613 | 20459 | A | C | A | 0.90323 | C | 0.09677 | 4 | 0.12903 |
| 16712 | S21_54373653 | 21 | 54373653 | 40 | C | T | C | 0.90323 | T | 0.09677 | 4 | 0.12903 |
| 16713 | S21_54597777 | 21 | 54597777 | 224124 | T | C | T | 0.8871 | C | 0.1129 | 5 | 0.16129 |
| 16714 | S21_54597976 | 21 | 54597976 | 199 | T | C | T | 0.85484 | C | 0.14516 | 7 | 0.22581 |
| 16715 | S21_55101422 | 21 | 55101422 | 503446 | C | T | C | 0.95161 | T | 0.04839 | 3 | 0.09677 |
| 16716 | S21_55161993 | 21 | 55161993 | 60571 | G | A | G | 0.90323 | A | 0.09677 | 6 | 0.19355 |
| 16717 | S21_55162057 | 21 | 55162057 | 64 | C | T | C | 0.90323 | T | 0.09677 | 6 | 0.19355 |
| 16718 | S21_55162062 | 21 | 55162062 | 5 | G | A | G | 0.8871 | A | 0.1129 | 7 | 0.22581 |
| 16719 | S21_55162122 | 21 | 55162122 | 60 | T | C | C | 0.8871 | T | 0.1129 | 7 | 0.22581 |
| 16720 | S21_55162207 | 21 | 55162207 | 85 | C | T | C | 0.93548 | T | 0.06452 | 2 | 0.06452 |
| 16721 | S21_55418853 | 21 | 55418853 | 256646 | T | C | C | 0.77419 | T | 0.22581 | 12 | 0.3871 |
| 16722 | S21_55418890 | 21 | 55418890 | 37 | G | C | G | 0.67742 | C | 0.32258 | 12 | 0.3871 |
| 16723 | S21_55418957 | 21 | 55418957 | 67 | A | G | A | 0.67742 | G | 0.32258 | 12 | 0.3871 |
| 16724 | S21_55469665 | 21 | 55469665 | 50708 | G | T | G | 0.62903 | T | 0.37097 | 13 | 0.41935 |
| 16725 | S21_55469835 | 21 | 55469835 | 170 | C | T | C | 0.80645 | T | 0.19355 | 6 | 0.19355 |
| 16726 | S21_55469841 | 21 | 55469841 | 6 | C | T | C | 0.93548 | T | 0.06452 | 4 | 0.12903 |
| 16727 | S21_55469847 | 21 | 55469847 | 6 | T | C | C | 0.85484 | T | 0.14516 | 7 | 0.22581 |
| 16728 | S21_55469906 | 21 | 55469906 | 59 | A | G | A | 0.83871 | G | 0.16129 | 6 | 0.19355 |
| 16729 | S21_55504429 | 21 | 55504429 | 34523 | G | A | G | 0.59677 | A | 0.40323 | 13 | 0.41935 |
| 16730 | S21_55504613 | 21 | 55504613 | 184 | A | G | A | 0.8871 | G | 0.1129 | 7 | 0.22581 |
| 16731 | S21_55504649 | 21 | 55504649 | 36 | T | A | T | 0.93548 | A | 0.06452 | 4 | 0.12903 |
| 16732 | S21_55783403 | 21 | 55783403 | 278754 | G | A | G | 0.54839 | A | 0.45161 | 14 | 0.45161 |
| 16733 | S21_55783443 | 21 | 55783443 | 40 | G | A | G | 0.66129 | A | 0.33871 | 15 | 0.48387 |
| 16734 | S21_55875243 | 21 | 55875243 | 91800 | G | A | G | 0.91935 | A | 0.08065 | 3 | 0.09677 |
| 16735 | S21_55875272 | 21 | 55875272 | 29 | T | C | T | 0.56452 | C | 0.43548 | 15 | 0.48387 |
| 16736 | S21_56239358 | 21 | 56239358 | 364086 | A | G | G | 0.70968 | A | 0.29032 | 14 | 0.45161 |
| 16737 | S21_56280478 | 21 | 56280478 | 41120 | A | G | A | 0.77419 | G | 0.22581 | 14 | 0.45161 |
| 16738 | S21_56280644 | 21 | 56280644 | 166 | C | A | C | 0.85484 | A | 0.14516 | 7 | 0.22581 |

| Site number | SNP name | Chromosome | Physical position | Physical distance from the previous SNP | Reference allele | Alternative allele | Major allele | Major allele frequency | Minor allele | Minor allele frequency | Number heterozygous | Heterozygosity |
|---|---|---|---|---|---|---|---|---|---|---|---|---|
| 16739 | S21_56367304 | 21 | 56367304 | 86660 | T | C | T | 0.69355 | C | 0.30645 | 9 | 0.29032 |
| 16740 | S21_56511603 | 21 | 56511603 | 144299 | G | T | G | 0.58065 | T | 0.41935 | 14 | 0.45161 |
| 16741 | S21_56511647 | 21 | 56511647 | 44 | C | T | C | 0.8871 | T | 0.1129 | 7 | 0.22581 |
| 16742 | S21_56511665 | 21 | 56511665 | 18 | T | C | C | 0.6129 | T | 0.3871 | 14 | 0.45161 |
| 16743 | S21_56660668 | 21 | 56660668 | 149003 | A | G | G | 0.6129 | A | 0.3871 | 10 | 0.32258 |
| 16744 | S21_56727744 | 21 | 56727744 | 67076 | G | T | G | 0.69355 | T | 0.30645 | 15 | 0.48387 |
| 16745 | S21_56727770 | 21 | 56727770 | 26 | G | A | G | 0.72581 | A | 0.27419 | 11 | 0.35484 |
| 16746 | S21_56729811 | 21 | 56729811 | 2041 | A | G | G | 0.70968 | A | 0.29032 | 10 | 0.32258 |
| 16747 | S21_56729903 | 21 | 56729903 | 92 | T | C | T | 0.91935 | C | 0.08065 | 5 | 0.16129 |
| 16748 | S21_56729936 | 21 | 56729936 | 33 | C | T | C | 0.69355 | T | 0.30645 | 15 | 0.48387 |
| 16749 | S21_56810487 | 21 | 56810487 | 80551 | T | C | C | 0.87097 | T | 0.12903 | 8 | 0.25806 |
| 16750 | S21_56810538 | 21 | 56810538 | 51 | A | G | G | 0.62903 | A | 0.37097 | 11 | 0.35484 |
| 16751 | S21_56810572 | 21 | 56810572 | 34 | T | C | T | 0.69355 | C | 0.30645 | 13 | 0.41935 |
| 16752 | S21_56810604 | 21 | 56810604 | 32 | C | T | C | 0.91935 | T | 0.08065 | 5 | 0.16129 |
| 16753 | S21_56810637 | 21 | 56810637 | 33 | C | T | C | 0.85484 | T | 0.14516 | 9 | 0.29032 |
| 16754 | S21_57324793 | 21 | 57324793 | 514156 | G | A | G | 0.95161 | A | 0.04839 | 3 | 0.09677 |
| 16755 | S21_57354892 | 21 | 57354892 | 30099 | G | A | G | 0.64516 | A | 0.35484 | 14 | 0.45161 |
| 16756 | S21_57354942 | 21 | 57354942 | 50 | G | A | G | 0.87097 | A | 0.12903 | 8 | 0.25806 |
| 16757 | S21_57635650 | 21 | 57635650 | 280708 | G | A | G | 0.74194 | A | 0.25806 | 12 | 0.3871 |
| 16758 | S21_57694111 | 21 | 57694111 | 58461 | C | T | C | 0.93548 | T | 0.06452 | 4 | 0.12903 |
| 16759 | S21_57978779 | 21 | 57978779 | 284668 | C | T | C | 0.91935 | T | 0.08065 | 5 | 0.16129 |
| 16760 | S21_57983050 | 21 | 57983050 | 4271 | A | G | A | 0.82258 | G | 0.17742 | 9 | 0.29032 |
| 16761 | S21_57983109 | 21 | 57983109 | 59 | G | A | G | 0.91935 | A | 0.08065 | 5 | 0.16129 |
| 16762 | S21_57983184 | 21 | 57983184 | 75 | C | T | C | 0.91935 | T | 0.08065 | 5 | 0.16129 |
| 16763 | S21_58049608 | 21 | 58049608 | 66424 | C | T | C | 0.91935 | T | 0.08065 | 3 | 0.09677 |
| 16764 | S21_58049617 | 21 | 58049617 | 9 | C | T | C | 0.91935 | T | 0.08065 | 3 | 0.09677 |
| 16765 | S21_58049639 | 21 | 58049639 | 22 | A | T | A | 0.87097 | T | 0.12903 | 6 | 0.19355 |
| 16766 | S21_58049704 | 21 | 58049704 | 65 | A | G | A | 0.82258 | G | 0.17742 | 7 | 0.22581 |
| 16767 | S21_58049736 | 21 | 58049736 | 32 | C | T | C | 0.91935 | T | 0.08065 | 3 | 0.09677 |
| 16768 | S21_58049750 | 21 | 58049750 | 14 | G | A | A | 0.59677 | G | 0.40323 | 13 | 0.41935 |
| 16769 | S21_58049773 | 21 | 58049773 | 23 | C | T | C | 0.82258 | T | 0.17742 | 7 | 0.22581 |
| 16770 | S21_58049782 | 21 | 58049782 | 9 | C | T | C | 0.91935 | T | 0.08065 | 3 | 0.09677 |
| 16771 | S21_58235954 | 21 | 58235954 | 186172 | C | T | T | 0.90323 | C | 0.09677 | 4 | 0.12903 |
| 16772 | S21_58235960 | 21 | 58235960 | 6 | A | G | A | 0.8871 | G | 0.1129 | 7 | 0.22581 |
| 16773 | S21_58236023 | 21 | 58236023 | 63 | C | T | C | 0.8871 | T | 0.1129 | 7 | 0.22581 |
| 16774 | S21_58236080 | 21 | 58236080 | 57 | T | A | T | 0.79032 | A | 0.20968 | 9 | 0.29032 |
| 16775 | S21_58257865 | 21 | 58257865 | 21785 | T | C | T | 0.74194 | C | 0.25806 | 14 | 0.45161 |
| 16776 | S21_58499312 | 21 | 58499312 | 241447 | G | C | G | 0.70968 | C | 0.29032 | 14 | 0.45161 |
| 16777 | S21_58499350 | 21 | 58499350 | 38 | A | C | A | 0.6129 | C | 0.3871 | 16 | 0.51613 |
| 16778 | S21_58499367 | 21 | 58499367 | 17 | C | A | C | 0.75806 | A | 0.24194 | 13 | 0.41935 |
| 16779 | S21_58499384 | 21 | 58499384 | 17 | C | T | C | 0.85484 | T | 0.14516 | 9 | 0.29032 |
| 16780 | S21_58499426 | 21 | 58499426 | 42 | C | T | C | 0.85484 | T | 0.14516 | 9 | 0.29032 |
| 16781 | S21_58499430 | 21 | 58499430 | 4 | G | C | G | 0.79032 | C | 0.20968 | 13 | 0.41935 |
| 16782 | S21_58499503 | 21 | 58499503 | 73 | T | C | C | 0.67742 | T | 0.32258 | 10 | 0.32258 |
| 16783 | S21_58534059 | 21 | 58534059 | 34556 | C | T | T | 0.53226 | C | 0.46774 | 17 | 0.54839 |
| 16784 | S21_58534060 | 21 | 58534060 | 1 | A | G | G | 0.77419 | A | 0.22581 | 14 | 0.45161 |
| 16785 | S21_58534232 | 21 | 58534232 | 172 | G | C | C | 0.77419 | G | 0.22581 | 14 | 0.45161 |
| 16786 | S21_59263901 | 21 | 59263901 | 729669 | T | C | T | 0.64516 | C | 0.35484 | 16 | 0.51613 |
| 16787 | S21_59263912 | 21 | 59263912 | 11 | G | A | G | 0.64516 | A | 0.35484 | 16 | 0.51613 |
| 16788 | S21_59263940 | 21 | 59263940 | 28 | A | G | A | 0.64516 | G | 0.35484 | 16 | 0.51613 |
| 16789 | S21_59264063 | 21 | 59264063 | 123 | T | C | T | 0.91935 | C | 0.08065 | 5 | 0.16129 |
| 16790 | S21_59371227 | 21 | 59371227 | 107164 | C | T | T | 0.54839 | C | 0.45161 | 18 | 0.58065 |
| 16791 | S21_59371286 | 21 | 59371286 | 59 | T | G | G | 0.54839 | T | 0.45161 | 18 | 0.58065 |
| 16792 | S21_59398215 | 21 | 59398215 | 26929 | C | T | C | 0.90323 | T | 0.09677 | 6 | 0.19355 |

| Site number | SNP name | Chromosome | Physical position | Physical distance from the previous SNP | Reference allele | Alternative allele | Major allele | Major allele frequency | Minor allele | Minor allele frequency | Number heterozygous | Heterozygosity |
|---|---|---|---|---|---|---|---|---|---|---|---|---|
| 16793 | S21_59403174 | 21 | 59403174 | 4959 | C | G | C | 0.80645 | G | 0.19355 | 8 | 0.25806 |
| 16794 | S21_59568791 | 21 | 59568791 | 165617 | C | T | C | 0.82258 | T | 0.17742 | 7 | 0.22581 |
| 16795 | S21_59613580 | 21 | 59613580 | 44789 | T | C | T | 0.90323 | C | 0.09677 | 4 | 0.12903 |
| 16796 | S21_59613648 | 21 | 59613648 | 68 | G | C | G | 0.93548 | C | 0.06452 | 4 | 0.12903 |
| 16797 | S21_59613682 | 21 | 59613682 | 34 | C | T | C | 0.93548 | T | 0.06452 | 4 | 0.12903 |
| 16798 | S21_59613695 | 21 | 59613695 | 13 | T | C | T | 0.87097 | C | 0.12903 | 8 | 0.25806 |
| 16799 | S21_59613785 | 21 | 59613785 | 90 | A | G | A | 0.83871 | G | 0.16129 | 10 | 0.32258 |
| 16800 | S21_59613788 | 21 | 59613788 | 3 | T | C | T | 0.95161 | C | 0.04839 | 3 | 0.09677 |
| 16801 | S21_59613808 | 21 | 59613808 | 20 | T | C | T | 0.83871 | C | 0.16129 | 10 | 0.32258 |
| 16802 | S21_59738572 | 21 | 59738572 | 124764 | G | A | G | 0.75806 | A | 0.24194 | 9 | 0.29032 |
| 16803 | S21_59738714 | 21 | 59738714 | 142 | A | C | A | 0.95161 | C | 0.04839 | 3 | 0.09677 |
| 16804 | S21_59945787 | 21 | 59945787 | 207073 | C | T | C | 0.93548 | T | 0.06452 | 4 | 0.12903 |
| 16805 | S21_60103603 | 21 | 60103603 | 157816 | G | C | G | 0.8871 | C | 0.1129 | 7 | 0.22581 |
| 16806 | S21_60115820 | 21 | 60115820 | 12217 | A | G | G | 0.53226 | A | 0.46774 | 13 | 0.41935 |
| 16807 | S21_60115841 | 21 | 60115841 | 21 | C | T | C | 0.79032 | T | 0.20968 | 11 | 0.35484 |
| 16808 | S21_60182242 | 21 | 60182242 | 66401 | C | T | C | 0.93548 | T | 0.06452 | 4 | 0.12903 |
| 16809 | S21_60222965 | 21 | 60222965 | 40723 | G | T | G | 0.56452 | T | 0.43548 | 11 | 0.35484 |
| 16810 | S21_60381138 | 21 | 60381138 | 158173 | G | A | G | 0.53226 | A | 0.46774 | 21 | 0.67742 |
| 16811 | S21_60381142 | 21 | 60381142 | 4 | G | A | G | 0.95161 | A | 0.04839 | 1 | 0.03226 |
| 16812 | S21_60402679 | 21 | 60402679 | 21537 | G | T | G | 0.67742 | T | 0.32258 | 12 | 0.3871 |
| 16813 | S21_60402710 | 21 | 60402710 | 31 | G | A | G | 0.95161 | A | 0.04839 | 3 | 0.09677 |
| 16814 | S21_60822198 | 21 | 60822198 | 419488 | C | G | C | 0.90323 | G | 0.09677 | 6 | 0.19355 |
| 16815 | S21_60822242 | 21 | 60822242 | 44 | G | A | G | 0.91935 | A | 0.08065 | 3 | 0.09677 |
| 16816 | S21_60822337 | 21 | 60822337 | 95 | G | A | G | 0.93548 | A | 0.06452 | 2 | 0.06452 |
| 16817 | S21_60822354 | 21 | 60822354 | 17 | T | C | T | 0.64516 | C | 0.35484 | 10 | 0.32258 |
| 16818 | S21_60989575 | 21 | 60989575 | 167221 | C | T | C | 0.83871 | T | 0.16129 | 8 | 0.25806 |
| 16819 | S21_61057993 | 21 | 61057993 | 68418 | C | G | C | 0.8871 | G | 0.1129 | 5 | 0.16129 |
| 16820 | S21_61058029 | 21 | 61058029 | 36 | G | A | G | 0.79032 | A | 0.20968 | 11 | 0.35484 |
| 16821 | S21_61297623 | 21 | 61297623 | 239594 | C | T | C | 0.64516 | T | 0.35484 | 12 | 0.3871 |
| 16822 | S21_61297647 | 21 | 61297647 | 24 | C | G | C | 0.64516 | G | 0.35484 | 12 | 0.3871 |
| 16823 | S21_61297704 | 21 | 61297704 | 57 | G | C | G | 0.82258 | C | 0.17742 | 9 | 0.29032 |
| 16824 | S21_61381366 | 21 | 61381366 | 83662 | G | T | G | 0.75806 | T | 0.24194 | 11 | 0.35484 |
| 16825 | S21_61392591 | 21 | 61392591 | 11225 | G | A | G | 0.56452 | A | 0.43548 | 17 | 0.54839 |
| 16826 | S21_61392720 | 21 | 61392720 | 129 | C | T | C | 0.72581 | T | 0.27419 | 13 | 0.41935 |
| 16827 | S21_61598081 | 21 | 61598081 | 205361 | T | C | C | 0.59677 | T | 0.40323 | 11 | 0.35484 |
| 16828 | S21_61709754 | 21 | 61709754 | 111673 | G | A | G | 0.64516 | A | 0.35484 | 14 | 0.45161 |
| 16829 | S21_61709841 | 21 | 61709841 | 87 | T | G | G | 0.64516 | T | 0.35484 | 10 | 0.32258 |
| 16830 | S21_61709944 | 21 | 61709944 | 103 | C | T | C | 0.83871 | T | 0.16129 | 8 | 0.25806 |
| 16831 | S21_61912992 | 21 | 61912992 | 203048 | G | T | G | 0.93548 | T | 0.06452 | 4 | 0.12903 |
| 16832 | S21_61913149 | 21 | 61913149 | 157 | A | C | C | 0.8871 | A | 0.1129 | 5 | 0.16129 |
| 16833 | S21_62289682 | 21 | 62289682 | 376533 | A | G | A | 0.93548 | G | 0.06452 | 4 | 0.12903 |
| 16834 | S21_62289717 | 21 | 62289717 | 35 | G | A | A | 0.59677 | G | 0.40323 | 15 | 0.48387 |
| 16835 | S21_62289853 | 21 | 62289853 | 136 | G | T | G | 0.67742 | T | 0.32258 | 14 | 0.45161 |
| 16836 | S21_62330045 | 21 | 62330045 | 40192 | C | T | C | 0.95161 | T | 0.04839 | 1 | 0.03226 |
| 16837 | S21_62336870 | 21 | 62336870 | 6825 | T | C | C | 0.66129 | T | 0.33871 | 15 | 0.48387 |
| 16838 | S21_62337007 | 21 | 62337007 | 137 | T | A | T | 0.80645 | A | 0.19355 | 10 | 0.32258 |
| 16839 | S21_62408360 | 21 | 62408360 | 71353 | G | T | G | 0.85484 | T | 0.14516 | 7 | 0.22581 |
| 16840 | S21_62683201 | 21 | 62683201 | 274841 | A | G | A | 0.77419 | G | 0.22581 | 12 | 0.3871 |
| 16841 | S21_62683381 | 21 | 62683381 | 180 | A | G | A | 0.91935 | G | 0.08065 | 3 | 0.09677 |
| 16842 | S21_62734970 | 21 | 62734970 | 51589 | C | T | C | 0.70968 | T | 0.29032 | 14 | 0.45161 |
| 16843 | S21_62789366 | 21 | 62789366 | 54396 | G | A | G | 0.85484 | A | 0.14516 | 7 | 0.22581 |
| 16844 | S21_62813241 | 21 | 62813241 | 23875 | G | A | A | 0.56452 | G | 0.43548 | 13 | 0.41935 |
| 16845 | S21_62813262 | 21 | 62813262 | 21 | C | T | C | 0.95161 | T | 0.04839 | 3 | 0.09677 |
| 16846 | S21_62813291 | 21 | 62813291 | 29 | T | C | C | 0.58065 | T | 0.41935 | 12 | 0.3871 |

| Site number | SNP name | Chromosome | Physical position | Physical distance from the previous SNP | Reference allele | Alternative allele | Major allele | Major allele frequency | Minor allele | Minor allele frequency | Number heterozygous | Heterozygosity |
|---|---|---|---|---|---|---|---|---|---|---|---|---|
| 16847 | S21_62813319 | 21 | 62813319 | 28 | G | A | G | 0.93548 | A | 0.06452 | 4 | 0.12903 |
| 16848 | S21_62884040 | 21 | 62884040 | 70721 | G | C | G | 0.87097 | C | 0.12903 | 6 | 0.19355 |
| 16849 | S21_63066201 | 21 | 63066201 | 182161 | A | G | A | 0.90323 | G | 0.09677 | 6 | 0.19355 |
| 16850 | S21_63194813 | 21 | 63194813 | 128612 | G | A | G | 0.82258 | A | 0.17742 | 7 | 0.22581 |
| 16851 | S21_63194826 | 21 | 63194826 | 13 | A | G | A | 0.91935 | G | 0.08065 | 5 | 0.16129 |
| 16852 | S21_63295861 | 21 | 63295861 | 101035 | A | G | A | 0.93548 | G | 0.06452 | 4 | 0.12903 |
| 16853 | S21_63671847 | 21 | 63671847 | 375986 | A | C | C | 0.91935 | A | 0.08065 | 3 | 0.09677 |
| 16854 | S21_63671988 | 21 | 63671988 | 141 | C | T | C | 0.64516 | T | 0.35484 | 10 | 0.32258 |
| 16855 | S21_63672022 | 21 | 63672022 | 34 | A | G | G | 0.91935 | A | 0.08065 | 3 | 0.09677 |
| 16856 | S21_63679021 | 21 | 63679021 | 6999 | T | C | C | 0.93548 | T | 0.06452 | 4 | 0.12903 |
| 16857 | S21_63679195 | 21 | 63679195 | 174 | C | T | T | 0.72581 | C | 0.27419 | 13 | 0.41935 |
| 16858 | S21_63706552 | 21 | 63706552 | 27357 | T | C | T | 0.95161 | C | 0.04839 | 3 | 0.09677 |
| 16859 | S21_64390833 | 21 | 64390833 | 684281 | C | T | C | 0.75806 | T | 0.24194 | 9 | 0.29032 |
| 16860 | S21_64390978 | 21 | 64390978 | 145 | T | C | T | 0.93548 | C | 0.06452 | 4 | 0.12903 |
| 16861 | S21_64391036 | 21 | 64391036 | 58 | T | A | T | 0.67742 | A | 0.32258 | 12 | 0.3871 |
| 16862 | S21_64427757 | 21 | 64427757 | 36721 | C | T | C | 0.91935 | T | 0.08065 | 5 | 0.16129 |
| 16863 | S21_64427760 | 21 | 64427760 | 3 | T | C | T | 0.91935 | C | 0.08065 | 5 | 0.16129 |
| 16864 | S21_64427764 | 21 | 64427764 | 4 | T | G | T | 0.93548 | G | 0.06452 | 4 | 0.12903 |
| 16865 | S21_64427985 | 21 | 64427985 | 221 | T | C | T | 0.8871 | C | 0.1129 | 7 | 0.22581 |
| 16866 | S21_64535795 | 21 | 64535795 | 107810 | G | C | G | 0.93548 | C | 0.06452 | 4 | 0.12903 |
| 16867 | S21_64636221 | 21 | 64636221 | 100426 | T | C | C | 0.85484 | T | 0.14516 | 9 | 0.29032 |
| 16868 | S21_64636279 | 21 | 64636279 | 58 | A | C | C | 0.85484 | A | 0.14516 | 9 | 0.29032 |
| 16869 | S21_64636346 | 21 | 64636346 | 67 | G | A | G | 0.93548 | A | 0.06452 | 4 | 0.12903 |
| 16870 | S21_64636360 | 21 | 64636360 | 14 | T | C | C | 0.85484 | T | 0.14516 | 9 | 0.29032 |
| 16871 | S21_65306151 | 21 | 65306151 | 669791 | G | T | G | 0.80645 | T | 0.19355 | 10 | 0.32258 |
| 16872 | S21_65306311 | 21 | 65306311 | 160 | A | G | G | 0.66129 | A | 0.33871 | 11 | 0.35484 |
| 16873 | S21_65636364 | 21 | 65636364 | 330053 | G | A | G | 0.91935 | A | 0.08065 | 5 | 0.16129 |
| 16874 | S21_65685087 | 21 | 65685087 | 48723 | A | G | G | 0.58065 | A | 0.41935 | 16 | 0.51613 |
| 16875 | S21_66255669 | 21 | 66255669 | 570582 | C | T | C | 0.62903 | T | 0.37097 | 11 | 0.35484 |
| 16876 | S21_66583976 | 21 | 66583976 | 328307 | G | A | G | 0.79032 | A | 0.20968 | 13 | 0.41935 |
| 16877 | S21_66847857 | 21 | 66847857 | 263881 | C | A | C | 0.91935 | A | 0.08065 | 5 | 0.16129 |
| 16878 | S21_66847962 | 21 | 66847962 | 105 | G | A | G | 0.90323 | A | 0.09677 | 6 | 0.19355 |
| 16879 | S21_67350885 | 21 | 67350885 | 502923 | T | C | T | 0.91935 | C | 0.08065 | 5 | 0.16129 |
| 16880 | S21_67351112 | 21 | 67351112 | 227 | T | C | T | 0.72581 | C | 0.27419 | 11 | 0.35484 |
| 16881 | S21_67550975 | 21 | 67550975 | 199863 | G | T | G | 0.95161 | T | 0.04839 | 3 | 0.09677 |
| 16882 | S21_67752325 | 21 | 67752325 | 201350 | T | C | T | 0.79032 | C | 0.20968 | 11 | 0.35484 |
| 16883 | S21_67752327 | 21 | 67752327 | 2 | C | T | C | 0.95161 | T | 0.04839 | 3 | 0.09677 |
| 16884 | S21_67752342 | 21 | 67752342 | 15 | G | T | G | 0.77419 | T | 0.22581 | 12 | 0.3871 |
| 16885 | S21_67986618 | 21 | 67986618 | 234276 | G | A | G | 0.95161 | A | 0.04839 | 3 | 0.09677 |
| 16886 | S21_67986690 | 21 | 67986690 | 72 | G | A | G | 0.95161 | A | 0.04839 | 3 | 0.09677 |
| 16887 | S21_67986714 | 21 | 67986714 | 24 | G | A | G | 0.87097 | A | 0.12903 | 6 | 0.19355 |
| 16888 | S21_68310964 | 21 | 68310964 | 324250 | A | T | A | 0.95161 | T | 0.04839 | 3 | 0.09677 |
| 16889 | S21_69272724 | 21 | 69272724 | 961760 | G | A | G | 0.95161 | A | 0.04839 | 3 | 0.09677 |
| 16890 | S21_69281004 | 21 | 69281004 | 8280 | C | T | C | 0.91935 | T | 0.08065 | 5 | 0.16129 |
| 16891 | S21_69281024 | 21 | 69281024 | 20 | C | T | C | 0.87097 | T | 0.12903 | 8 | 0.25806 |
| 16892 | S21_69281025 | 21 | 69281025 | 1 | G | A | G | 0.87097 | A | 0.12903 | 8 | 0.25806 |
| 16893 | S21_69281029 | 21 | 69281029 | 4 | A | C | A | 0.80645 | C | 0.19355 | 12 | 0.3871 |
| 16894 | S21_69281042 | 21 | 69281042 | 13 | C | G | C | 0.90323 | G | 0.09677 | 6 | 0.19355 |
| 16895 | S22_117539 | 22 | 117539 | 0 | C | T | C | 0.62903 | T | 0.37097 | 13 | 0.41935 |
| 16896 | S22_124714 | 22 | 124714 | 7175 | A | G | G | 0.91935 | A | 0.08065 | 5 | 0.16129 |
| 16897 | S22_456505 | 22 | 456505 | 331791 | G | T | G | 0.82258 | T | 0.17742 | 11 | 0.35484 |
| 16898 | S22_456533 | 22 | 456533 | 28 | T | C | T | 0.66129 | C | 0.33871 | 15 | 0.48387 |
| 16899 | S22_465150 | 22 | 465150 | 8617 | G | A | G | 0.75806 | A | 0.24194 | 13 | 0.41935 |
| 16900 | S22_465217 | 22 | 465217 | 67 | A | C | A | 0.91935 | C | 0.08065 | 5 | 0.16129 |

| Site number | SNP name | Chromosome | Physical position | Physical distance from the previous SNP | Reference allele | Alternative allele | Major allele | Major allele frequency | Minor allele | Minor allele frequency | Number heterozygous | Heterozygosity |
|---|---|---|---|---|---|---|---|---|---|---|---|---|
| 16901 | S22_720266 | 22 | 720266 | 255049 | G | C | G | 0.93548 | C | 0.06452 | 4 | 0.12903 |
| 16902 | S22_720278 | 22 | 720278 | 12 | T | G | T | 0.8871 | G | 0.1129 | 7 | 0.22581 |
| 16903 | S22_720364 | 22 | 720364 | 86 | G | C | G | 0.91935 | C | 0.08065 | 5 | 0.16129 |
| 16904 | S22_720466 | 22 | 720466 | 102 | C | G | G | 0.69355 | C | 0.30645 | 13 | 0.41935 |
| 16905 | S22_849239 | 22 | 849239 | 128773 | G | A | G | 0.77419 | A | 0.22581 | 12 | 0.3871 |
| 16906 | S22_849254 | 22 | 849254 | 15 | C | T | C | 0.90323 | T | 0.09677 | 6 | 0.19355 |
| 16907 | S22_849255 | 22 | 849255 | 1 | G | A | G | 0.53226 | A | 0.46774 | 13 | 0.41935 |
| 16908 | S22_911607 | 22 | 911607 | 62352 | T | G | T | 0.85484 | G | 0.14516 | 7 | 0.22581 |
| 16909 | S22_911624 | 22 | 911624 | 17 | A | G | A | 0.83871 | G | 0.16129 | 6 | 0.19355 |
| 16910 | S22_911692 | 22 | 911692 | 68 | C | T | C | 0.85484 | T | 0.14516 | 7 | 0.22581 |
| 16911 | S22_980788 | 22 | 980788 | 69096 | A | T | A | 0.53226 | T | 0.46774 | 17 | 0.54839 |
| 16912 | S22_980824 | 22 | 980824 | 36 | G | T | G | 0.85484 | T | 0.14516 | 7 | 0.22581 |
| 16913 | S22_980858 | 22 | 980858 | 34 | A | G | A | 0.75806 | G | 0.24194 | 13 | 0.41935 |
| 16914 | S22_1429876 | 22 | 1429876 | 449018 | C | A | C | 0.75806 | A | 0.24194 | 9 | 0.29032 |
| 16915 | S22_1647068 | 22 | 1647068 | 217192 | G | A | G | 0.70968 | A | 0.29032 | 16 | 0.51613 |
| 16916 | S22_1647076 | 22 | 1647076 | 8 | G | T | G | 0.87097 | T | 0.12903 | 8 | 0.25806 |
| 16917 | S22_1650451 | 22 | 1650451 | 3375 | G | T | G | 0.87097 | T | 0.12903 | 8 | 0.25806 |
| 16918 | S22_1650487 | 22 | 1650487 | 36 | C | T | C | 0.93548 | T | 0.06452 | 4 | 0.12903 |
| 16919 | S22_1650499 | 22 | 1650499 | 12 | A | C | A | 0.93548 | C | 0.06452 | 4 | 0.12903 |
| 16920 | S22_1693370 | 22 | 1693370 | 42871 | G | A | G | 0.95161 | A | 0.04839 | 3 | 0.09677 |
| 16921 | S22_1693407 | 22 | 1693407 | 37 | T | C | T | 0.83871 | C | 0.16129 | 8 | 0.25806 |
| 16922 | S22_1693483 | 22 | 1693483 | 76 | C | T | T | 0.87097 | C | 0.12903 | 6 | 0.19355 |
| 16923 | S22_1722987 | 22 | 1722987 | 29504 | T | C | C | 0.95161 | T | 0.04839 | 3 | 0.09677 |
| 16924 | S22_1737746 | 22 | 1737746 | 14759 | G | A | A | 0.64516 | G | 0.35484 | 10 | 0.32258 |
| 16925 | S22_1821414 | 22 | 1821414 | 83668 | G | A | G | 0.87097 | A | 0.12903 | 6 | 0.19355 |
| 16926 | S22_1821442 | 22 | 1821442 | 28 | C | T | C | 0.95161 | T | 0.04839 | 3 | 0.09677 |
| 16927 | S22_1821470 | 22 | 1821470 | 28 | G | A | G | 0.95161 | A | 0.04839 | 3 | 0.09677 |
| 16928 | S22_1821524 | 22 | 1821524 | 54 | G | A | G | 0.83871 | A | 0.16129 | 10 | 0.32258 |
| 16929 | S22_1821540 | 22 | 1821540 | 16 | C | T | C | 0.8871 | T | 0.1129 | 7 | 0.22581 |
| 16930 | S22_1876215 | 22 | 1876215 | 54675 | G | A | G | 0.93548 | A | 0.06452 | 4 | 0.12903 |
| 16931 | S22_2103251 | 22 | 2103251 | 227036 | A | G | A | 0.93548 | G | 0.06452 | 4 | 0.12903 |
| 16932 | S22_2407156 | 22 | 2407156 | 303905 | A | G | A | 0.80645 | G | 0.19355 | 10 | 0.32258 |
| 16933 | S22_2407189 | 22 | 2407189 | 33 | G | A | A | 0.6129 | G | 0.3871 | 12 | 0.3871 |
| 16934 | S22_2407239 | 22 | 2407239 | 50 | G | T | G | 0.95161 | T | 0.04839 | 3 | 0.09677 |
| 16935 | S22_2706256 | 22 | 2706256 | 299017 | G | C | G | 0.62903 | C | 0.37097 | 17 | 0.54839 |
| 16936 | S22_2706351 | 22 | 2706351 | 95 | C | T | C | 0.64516 | T | 0.35484 | 16 | 0.51613 |
| 16937 | S22_2864563 | 22 | 2864563 | 158212 | G | A | G | 0.82258 | A | 0.17742 | 7 | 0.22581 |
| 16938 | S22_2864611 | 22 | 2864611 | 48 | G | A | G | 0.83871 | A | 0.16129 | 10 | 0.32258 |
| 16939 | S22_2888443 | 22 | 2888443 | 23832 | G | A | G | 0.83871 | A | 0.16129 | 8 | 0.25806 |
| 16940 | S22_3215571 | 22 | 3215571 | 327128 | G | A | G | 0.51613 | A | 0.48387 | 16 | 0.51613 |
| 16941 | S22_3215594 | 22 | 3215594 | 23 | T | C | C | 0.53226 | T | 0.46774 | 17 | 0.54839 |
| 16942 | S22_3215603 | 22 | 3215603 | 9 | T | C | T | 0.64516 | C | 0.35484 | 14 | 0.45161 |
| 16943 | S22_3215672 | 22 | 3215672 | 69 | G | C | C | 0.53226 | G | 0.46774 | 17 | 0.54839 |
| 16944 | S22_3412802 | 22 | 3412802 | 197130 | A | C | A | 0.82258 | C | 0.17742 | 7 | 0.22581 |
| 16945 | S22_3866111 | 22 | 3866111 | 453309 | T | C | T | 0.95161 | C | 0.04839 | 3 | 0.09677 |
| 16946 | S22_3866120 | 22 | 3866120 | 9 | T | C | T | 0.83871 | C | 0.16129 | 8 | 0.25806 |
| 16947 | S22_3866302 | 22 | 3866302 | 182 | A | G | G | 0.64516 | A | 0.35484 | 14 | 0.45161 |
| 16948 | S22_4376109 | 22 | 4376109 | 509807 | A | G | A | 0.91935 | G | 0.08065 | 5 | 0.16129 |
| 16949 | S22_4376153 | 22 | 4376153 | 44 | G | A | G | 0.90323 | A | 0.09677 | 6 | 0.19355 |
| 16950 | S22_4376185 | 22 | 4376185 | 32 | G | A | G | 0.91935 | A | 0.08065 | 5 | 0.16129 |
| 16951 | S22_4376230 | 22 | 4376230 | 45 | C | T | C | 0.91935 | T | 0.08065 | 5 | 0.16129 |
| 16952 | S22_4886041 | 22 | 4886041 | 509811 | G | C | G | 0.82258 | C | 0.17742 | 11 | 0.35484 |
| 16953 | S22_5027874 | 22 | 5027874 | 141833 | G | A | A | 0.87097 | G | 0.12903 | 8 | 0.25806 |
| 16954 | S22_5027937 | 22 | 5027937 | 63 | C | G | C | 0.95161 | G | 0.04839 | 3 | 0.09677 |

| Site number | SNP name | Chromosome | Physical position | Physical distance from the previous SNP | Reference allele | Alternative allele | Major allele | Major allele frequency | Minor allele | Minor allele frequency | Number heterozygous | Heterozygosity |
|---|---|---|---|---|---|---|---|---|---|---|---|---|
| 16955 | S22_5564725 | 22 | 5564725 | 536788 | G | A | G | 0.95161 | A | 0.04839 | 3 | 0.09677 |
| 16956 | S22_5638564 | 22 | 5638564 | 73839 | A | C | A | 0.95161 | C | 0.04839 | 3 | 0.09677 |
| 16957 | S22_5638566 | 22 | 5638566 | 2 | C | T | C | 0.70968 | T | 0.29032 | 12 | 0.3871 |
| 16958 | S22_5638613 | 22 | 5638613 | 47 | C | T | C | 0.75806 | T | 0.24194 | 11 | 0.35484 |
| 16959 | S22_5781640 | 22 | 5781640 | 143027 | T | C | T | 0.72581 | C | 0.27419 | 13 | 0.41935 |
| 16960 | S22_5781658 | 22 | 5781658 | 18 | A | G | A | 0.51613 | G | 0.48387 | 14 | 0.45161 |
| 16961 | S22_5781709 | 22 | 5781709 | 51 | T | C | T | 0.54839 | C | 0.45161 | 14 | 0.45161 |
| 16962 | S22_5781861 | 22 | 5781861 | 152 | A | G | A | 0.80645 | G | 0.19355 | 8 | 0.25806 |
| 16963 | S22_5781862 | 22 | 5781862 | 1 | T | C | T | 0.64516 | C | 0.35484 | 16 | 0.51613 |
| 16964 | S22_6121800 | 22 | 6121800 | 339938 | G | C | G | 0.83871 | C | 0.16129 | 10 | 0.32258 |
| 16965 | S22_6121861 | 22 | 6121861 | 61 | C | T | C | 0.91935 | T | 0.08065 | 5 | 0.16129 |
| 16966 | S22_6121865 | 22 | 6121865 | 4 | T | C | T | 0.75806 | C | 0.24194 | 11 | 0.35484 |
| 16967 | S22_6121903 | 22 | 6121903 | 38 | C | T | C | 0.67742 | T | 0.32258 | 16 | 0.51613 |
| 16968 | S22_6121921 | 22 | 6121921 | 18 | G | A | G | 0.75806 | A | 0.24194 | 11 | 0.35484 |
| 16969 | S22_6245263 | 22 | 6245263 | 123342 | T | C | T | 0.91935 | C | 0.08065 | 5 | 0.16129 |
| 16970 | S22_6282133 | 22 | 6282133 | 36870 | T | C | T | 0.93548 | C | 0.06452 | 4 | 0.12903 |
| 16971 | S22_6282165 | 22 | 6282165 | 32 | T | G | T | 0.93548 | G | 0.06452 | 4 | 0.12903 |
| 16972 | S22_6282288 | 22 | 6282288 | 123 | G | A | G | 0.91935 | A | 0.08065 | 3 | 0.09677 |
| 16973 | S22_6282339 | 22 | 6282339 | 51 | T | G | T | 0.58065 | G | 0.41935 | 10 | 0.32258 |
| 16974 | S22_6287262 | 22 | 6287262 | 4923 | A | G | A | 0.90323 | G | 0.09677 | 6 | 0.19355 |
| 16975 | S22_6287279 | 22 | 6287279 | 17 | C | T | C | 0.95161 | T | 0.04839 | 3 | 0.09677 |
| 16976 | S22_6287290 | 22 | 6287290 | 11 | T | C | T | 0.72581 | C | 0.27419 | 11 | 0.35484 |
| 16977 | S22_6287348 | 22 | 6287348 | 58 | T | C | T | 0.67742 | C | 0.32258 | 14 | 0.45161 |
| 16978 | S22_6287385 | 22 | 6287385 | 37 | C | T | T | 0.90323 | C | 0.09677 | 6 | 0.19355 |
| 16979 | S22_6287396 | 22 | 6287396 | 11 | C | T | C | 0.82258 | T | 0.17742 | 7 | 0.22581 |
| 16980 | S22_6287419 | 22 | 6287419 | 23 | C | T | C | 0.59677 | T | 0.40323 | 11 | 0.35484 |
| 16981 | S22_6287431 | 22 | 6287431 | 12 | G | A | G | 0.82258 | A | 0.17742 | 7 | 0.22581 |
| 16982 | S22_6287438 | 22 | 6287438 | 7 | C | T | C | 0.82258 | T | 0.17742 | 9 | 0.29032 |
| 16983 | S22_6287439 | 22 | 6287439 | 1 | G | C | G | 0.95161 | C | 0.04839 | 3 | 0.09677 |
| 16984 | S22_6287456 | 22 | 6287456 | 17 | G | A | G | 0.82258 | A | 0.17742 | 7 | 0.22581 |
| 16985 | S22_6287486 | 22 | 6287486 | 30 | C | T | C | 0.82258 | T | 0.17742 | 7 | 0.22581 |
| 16986 | S22_6479455 | 22 | 6479455 | 191969 | C | T | C | 0.93548 | T | 0.06452 | 4 | 0.12903 |
| 16987 | S22_6479456 | 22 | 6479456 | 1 | A | G | A | 0.93548 | G | 0.06452 | 4 | 0.12903 |
| 16988 | S22_6479481 | 22 | 6479481 | 25 | C | T | C | 0.93548 | T | 0.06452 | 4 | 0.12903 |
| 16989 | S22_6479499 | 22 | 6479499 | 18 | G | A | G | 0.93548 | A | 0.06452 | 4 | 0.12903 |
| 16990 | S22_6479512 | 22 | 6479512 | 13 | T | C | T | 0.93548 | C | 0.06452 | 4 | 0.12903 |
| 16991 | S22_6572061 | 22 | 6572061 | 92549 | T | C | T | 0.77419 | C | 0.22581 | 14 | 0.45161 |
| 16992 | S22_6572075 | 22 | 6572075 | 14 | C | T | C | 0.77419 | T | 0.22581 | 14 | 0.45161 |
| 16993 | S22_6625808 | 22 | 6625808 | 53733 | T | A | A | 0.54839 | T | 0.45161 | 14 | 0.45161 |
| 16994 | S22_6625854 | 22 | 6625854 | 46 | G | A | G | 0.90323 | A | 0.09677 | 6 | 0.19355 |
| 16995 | S22_6625868 | 22 | 6625868 | 14 | G | C | G | 0.90323 | C | 0.09677 | 6 | 0.19355 |
| 16996 | S22_6625879 | 22 | 6625879 | 11 | G | A | G | 0.95161 | A | 0.04839 | 3 | 0.09677 |
| 16997 | S22_6625904 | 22 | 6625904 | 25 | G | A | A | 0.58065 | G | 0.41935 | 14 | 0.45161 |
| 16998 | S22_6669219 | 22 | 6669219 | 43315 | A | G | A | 0.75806 | G | 0.24194 | 11 | 0.35484 |
| 16999 | S22_6724628 | 22 | 6724628 | 55409 | G | A | G | 0.80645 | A | 0.19355 | 10 | 0.32258 |
| 17000 | S22_6724666 | 22 | 6724666 | 38 | A | G | G | 0.56452 | A | 0.43548 | 13 | 0.41935 |
| 17001 | S22_6724768 | 22 | 6724768 | 102 | C | G | C | 0.77419 | G | 0.22581 | 10 | 0.32258 |
| 17002 | S22_6746845 | 22 | 6746845 | 22077 | C | G | C | 0.93548 | G | 0.06452 | 4 | 0.12903 |
| 17003 | S22_6855180 | 22 | 6855180 | 108335 | C | T | C | 0.79032 | T | 0.20968 | 13 | 0.41935 |
| 17004 | S22_6857324 | 22 | 6857324 | 2144 | G | T | G | 0.62903 | T | 0.37097 | 9 | 0.29032 |
| 17005 | S22_7123901 | 22 | 7123901 | 266577 | G | C | G | 0.69355 | C | 0.30645 | 15 | 0.48387 |
| 17006 | S22_7130643 | 22 | 7130643 | 6742 | G | A | A | 0.80645 | G | 0.19355 | 10 | 0.32258 |
| 17007 | S22_7130730 | 22 | 7130730 | 87 | T | C | C | 0.80645 | T | 0.19355 | 10 | 0.32258 |
| 17008 | S22_7160356 | 22 | 7160356 | 29626 | C | G | C | 0.70968 | G | 0.29032 | 12 | 0.3871 |

| Site number | SNP name | Chromosome | Physical position | Physical distance from the previous SNP | Reference allele | Alternative allele | Major allele | Major allele frequency | Minor allele | Minor allele frequency | Number heterozygous | Heterozygosity |
|---|---|---|---|---|---|---|---|---|---|---|---|---|
| 17009 | S22_7174260 | 22 | 7174260 | 13904 | G | A | G | 0.91935 | A | 0.08065 | 3 | 0.09677 |
| 17010 | S22_7174328 | 22 | 7174328 | 68 | C | T | C | 0.91935 | T | 0.08065 | 3 | 0.09677 |
| 17011 | S22_7174405 | 22 | 7174405 | 77 | T | C | T | 0.91935 | C | 0.08065 | 3 | 0.09677 |
| 17012 | S22_7174417 | 22 | 7174417 | 12 | G | T | G | 0.91935 | T | 0.08065 | 3 | 0.09677 |
| 17013 | S22_7372977 | 22 | 7372977 | 198560 | T | G | T | 0.90323 | G | 0.09677 | 4 | 0.12903 |
| 17014 | S22_7373021 | 22 | 7373021 | 44 | T | C | C | 0.53226 | T | 0.46774 | 15 | 0.48387 |
| 17015 | S22_7373100 | 22 | 7373100 | 79 | G | A | A | 0.6129 | G | 0.3871 | 16 | 0.51613 |
| 17016 | S22_7373159 | 22 | 7373159 | 59 | G | A | G | 0.8871 | A | 0.1129 | 5 | 0.16129 |
| 17017 | S22_7402119 | 22 | 7402119 | 28960 | C | T | C | 0.8871 | T | 0.1129 | 5 | 0.16129 |
| 17018 | S22_7402212 | 22 | 7402212 | 93 | A | G | G | 0.8871 | A | 0.1129 | 5 | 0.16129 |
| 17019 | S22_7402222 | 22 | 7402222 | 10 | A | C | C | 0.8871 | A | 0.1129 | 5 | 0.16129 |
| 17020 | S22_7402250 | 22 | 7402250 | 28 | T | C | C | 0.8871 | T | 0.1129 | 5 | 0.16129 |
| 17021 | S22_7402251 | 22 | 7402251 | 1 | G | A | A | 0.8871 | G | 0.1129 | 5 | 0.16129 |
| 17022 | S22_7402263 | 22 | 7402263 | 12 | G | C | C | 0.8871 | G | 0.1129 | 5 | 0.16129 |
| 17023 | S22_7402302 | 22 | 7402302 | 39 | C | G | G | 0.8871 | C | 0.1129 | 5 | 0.16129 |
| 17024 | S22_7402316 | 22 | 7402316 | 14 | C | T | T | 0.8871 | C | 0.1129 | 5 | 0.16129 |
| 17025 | S22_7602705 | 22 | 7602705 | 200389 | G | A | G | 0.95161 | A | 0.04839 | 3 | 0.09677 |
| 17026 | S22_8369189 | 22 | 8369189 | 766484 | G | A | G | 0.8871 | A | 0.1129 | 7 | 0.22581 |
| 17027 | S22_8369231 | 22 | 8369231 | 42 | C | T | C | 0.91935 | T | 0.08065 | 5 | 0.16129 |
| 17028 | S22_8797624 | 22 | 8797624 | 428393 | T | C | C | 0.6129 | T | 0.3871 | 14 | 0.45161 |
| 17029 | S22_8797709 | 22 | 8797709 | 85 | C | T | C | 0.72581 | T | 0.27419 | 13 | 0.41935 |
| 17030 | S22_8797730 | 22 | 8797730 | 21 | A | G | A | 0.77419 | G | 0.22581 | 14 | 0.45161 |
| 17031 | S22_8797745 | 22 | 8797745 | 15 | T | C | T | 0.80645 | C | 0.19355 | 8 | 0.25806 |
| 17032 | S22_8810684 | 22 | 8810684 | 12939 | C | T | C | 0.93548 | T | 0.06452 | 4 | 0.12903 |
| 17033 | S22_8810685 | 22 | 8810685 | 1 | G | A | G | 0.8871 | A | 0.1129 | 7 | 0.22581 |
| 17034 | S22_8810775 | 22 | 8810775 | 90 | C | T | C | 0.93548 | T | 0.06452 | 4 | 0.12903 |
| 17035 | S22_8810804 | 22 | 8810804 | 29 | A | G | A | 0.93548 | G | 0.06452 | 4 | 0.12903 |
| 17036 | S22_9720332 | 22 | 9720332 | 909528 | G | A | G | 0.56452 | A | 0.43548 | 15 | 0.48387 |
| 17037 | S22_9720357 | 22 | 9720357 | 25 | C | G | G | 0.85484 | C | 0.14516 | 7 | 0.22581 |
| 17038 | S22_9773540 | 22 | 9773540 | 53183 | G | A | G | 0.82258 | A | 0.17742 | 11 | 0.35484 |
| 17039 | S22_9947373 | 22 | 9947373 | 173833 | G | A | A | 0.5 | G | 0.5 | 19 | 0.6129 |
| 17040 | S22_9947503 | 22 | 9947503 | 130 | T | C | C | 0.5 | T | 0.5 | 19 | 0.6129 |
| 17041 | S22_9947511 | 22 | 9947511 | 8 | A | G | A | 0.5 | G | 0.5 | 19 | 0.6129 |
| 17042 | S22_10238340 | 22 | 10238340 | 290829 | C | T | C | 0.93548 | T | 0.06452 | 4 | 0.12903 |
| 17043 | S22_10238353 | 22 | 10238353 | 13 | T | G | G | 0.75806 | T | 0.24194 | 13 | 0.41935 |
| 17044 | S22_10238446 | 22 | 10238446 | 93 | A | C | A | 0.87097 | C | 0.12903 | 8 | 0.25806 |
| 17045 | S22_10238516 | 22 | 10238516 | 70 | T | G | G | 0.56452 | T | 0.43548 | 13 | 0.41935 |
| 17046 | S22_10271708 | 22 | 10271708 | 33192 | T | C | C | 0.74194 | T | 0.25806 | 12 | 0.3871 |
| 17047 | S22_10271870 | 22 | 10271870 | 162 | C | T | C | 0.95161 | T | 0.04839 | 3 | 0.09677 |
| 17048 | S22_10416894 | 22 | 10416894 | 145024 | C | G | G | 0.72581 | C | 0.27419 | 9 | 0.29032 |
| 17049 | S22_10435749 | 22 | 10435749 | 18855 | G | C | G | 0.77419 | C | 0.22581 | 10 | 0.32258 |
| 17050 | S22_10435823 | 22 | 10435823 | 74 | T | C | T | 0.79032 | C | 0.20968 | 11 | 0.35484 |
| 17051 | S22_10461284 | 22 | 10461284 | 25461 | G | A | G | 0.75806 | A | 0.24194 | 11 | 0.35484 |
| 17052 | S22_10461308 | 22 | 10461308 | 24 | A | G | A | 0.75806 | G | 0.24194 | 11 | 0.35484 |
| 17053 | S22_10570473 | 22 | 10570473 | 109165 | C | T | C | 0.56452 | T | 0.43548 | 19 | 0.6129 |
| 17054 | S22_10620635 | 22 | 10620635 | 50162 | G | A | G | 0.80645 | A | 0.19355 | 10 | 0.32258 |
| 17055 | S22_10620735 | 22 | 10620735 | 100 | C | T | C | 0.91935 | T | 0.08065 | 5 | 0.16129 |
| 17056 | S22_10899196 | 22 | 10899196 | 278461 | T | A | A | 0.80645 | T | 0.19355 | 12 | 0.3871 |
| 17057 | S22_11043159 | 22 | 11043159 | 143963 | A | G | A | 0.82258 | G | 0.17742 | 11 | 0.35484 |
| 17058 | S22_11238839 | 22 | 11238839 | 195680 | C | T | C | 0.93548 | T | 0.06452 | 4 | 0.12903 |
| 17059 | S22_12338287 | 22 | 12338287 | 1099448 | G | A | G | 0.95161 | A | 0.04839 | 1 | 0.03226 |
| 17060 | S22_12346326 | 22 | 12346326 | 8039 | C | T | T | 0.85484 | C | 0.14516 | 9 | 0.29032 |
| 17061 | S22_12577097 | 22 | 12577097 | 230771 | G | A | G | 0.69355 | A | 0.30645 | 15 | 0.48387 |
| 17062 | S22_12606421 | 22 | 12606421 | 29324 | T | G | T | 0.8871 | G | 0.1129 | 7 | 0.22581 |

| Site number | SNP name | Chromosome | Physical position | Physical distance from the previous SNP | Reference allele | Alternative allele | Major allele | Major allele frequency | Minor allele | Minor allele frequency | Number heterozygous | Heterozygosity |
|---|---|---|---|---|---|---|---|---|---|---|---|---|
| 17063 | S22_12998363 | 22 | 12998363 | 391942 | C | T | C | 0.91935 | T | 0.08065 | 3 | 0.09677 |
| 17064 | S22_13077772 | 22 | 13077772 | 79409 | G | A | G | 0.54839 | A | 0.45161 | 20 | 0.64516 |
| 17065 | S22_13077923 | 22 | 13077923 | 151 | C | T | C | 0.54839 | T | 0.45161 | 20 | 0.64516 |
| 17066 | S22_13083017 | 22 | 13083017 | 5094 | G | A | G | 0.95161 | A | 0.04839 | 1 | 0.03226 |
| 17067 | S22_13083072 | 22 | 13083072 | 55 | C | T | C | 0.91935 | T | 0.08065 | 3 | 0.09677 |
| 17068 | S22_13083085 | 22 | 13083085 | 13 | C | T | C | 0.80645 | T | 0.19355 | 10 | 0.32258 |
| 17069 | S22_13083105 | 22 | 13083105 | 20 | G | A | G | 0.80645 | A | 0.19355 | 10 | 0.32258 |
| 17070 | S22_13118546 | 22 | 13118546 | 35441 | G | A | G | 0.87097 | A | 0.12903 | 8 | 0.25806 |
| 17071 | S22_13118584 | 22 | 13118584 | 38 | A | G | A | 0.87097 | G | 0.12903 | 6 | 0.19355 |
| 17072 | S22_14001765 | 22 | 14001765 | 883181 | G | A | G | 0.85484 | A | 0.14516 | 9 | 0.29032 |
| 17073 | S22_14330062 | 22 | 14330062 | 328297 | G | A | G | 0.85484 | A | 0.14516 | 5 | 0.16129 |
| 17074 | S22_14330119 | 22 | 14330119 | 57 | G | T | G | 0.85484 | T | 0.14516 | 5 | 0.16129 |
| 17075 | S22_14330195 | 22 | 14330195 | 76 | G | A | G | 0.85484 | A | 0.14516 | 5 | 0.16129 |
| 17076 | S22_14548617 | 22 | 14548617 | 218422 | T | C | T | 0.83871 | C | 0.16129 | 10 | 0.32258 |
| 17077 | S22_15083928 | 22 | 15083928 | 535311 | T | C | T | 0.54839 | C | 0.45161 | 14 | 0.45161 |
| 17078 | S22_15083951 | 22 | 15083951 | 23 | C | T | C | 0.95161 | T | 0.04839 | 3 | 0.09677 |
| 17079 | S22_15084148 | 22 | 15084148 | 197 | G | A | A | 0.67742 | G | 0.32258 | 14 | 0.45161 |
| 17080 | S22_15281792 | 22 | 15281792 | 197644 | G | A | G | 0.62903 | A | 0.37097 | 13 | 0.41935 |
| 17081 | S22_15990644 | 22 | 15990644 | 708852 | A | G | G | 0.8871 | A | 0.1129 | 7 | 0.22581 |
| 17082 | S22_16290460 | 22 | 16290460 | 299816 | T | C | C | 0.74194 | T | 0.25806 | 12 | 0.3871 |
| 17083 | S22_16498703 | 22 | 16498703 | 208243 | C | G | G | 0.67742 | C | 0.32258 | 10 | 0.32258 |
| 17084 | S22_16498812 | 22 | 16498812 | 109 | T | A | T | 0.58065 | A | 0.41935 | 14 | 0.45161 |
| 17085 | S22_16498834 | 22 | 16498834 | 22 | A | G | G | 0.77419 | A | 0.22581 | 8 | 0.25806 |
| 17086 | S22_16498842 | 22 | 16498842 | 8 | C | T | C | 0.91935 | T | 0.08065 | 3 | 0.09677 |
| 17087 | S22_16701202 | 22 | 16701202 | 202360 | T | C | T | 0.74194 | C | 0.25806 | 10 | 0.32258 |
| 17088 | S22_16708692 | 22 | 16708692 | 7490 | G | A | G | 0.74194 | A | 0.25806 | 10 | 0.32258 |
| 17089 | S22_16708742 | 22 | 16708742 | 50 | G | A | G | 0.74194 | A | 0.25806 | 10 | 0.32258 |
| 17090 | S22_17326268 | 22 | 17326268 | 617526 | A | G | A | 0.72581 | G | 0.27419 | 15 | 0.48387 |
| 17091 | S22_17647042 | 22 | 17647042 | 320774 | G | A | G | 0.82258 | A | 0.17742 | 9 | 0.29032 |
| 17092 | S22_17647044 | 22 | 17647044 | 2 | G | A | A | 0.87097 | G | 0.12903 | 8 | 0.25806 |
| 17093 | S22_17647108 | 22 | 17647108 | 64 | C | G | C | 0.93548 | G | 0.06452 | 4 | 0.12903 |
| 17094 | S22_17667246 | 22 | 17667246 | 20138 | T | G | G | 0.95161 | T | 0.04839 | 3 | 0.09677 |
| 17095 | S22_17667379 | 22 | 17667379 | 133 | G | T | G | 0.93548 | T | 0.06452 | 4 | 0.12903 |
| 17096 | S22_17672413 | 22 | 17672413 | 5034 | G | C | C | 0.90323 | G | 0.09677 | 4 | 0.12903 |
| 17097 | S22_18134174 | 22 | 18134174 | 461761 | C | G | G | 0.82258 | C | 0.17742 | 9 | 0.29032 |
| 17098 | S22_18265748 | 22 | 18265748 | 131574 | A | G | A | 0.62903 | G | 0.37097 | 13 | 0.41935 |
| 17099 | S22_18283860 | 22 | 18283860 | 18112 | C | T | C | 0.95161 | T | 0.04839 | 3 | 0.09677 |
| 17100 | S22_18283948 | 22 | 18283948 | 88 | C | T | C | 0.72581 | T | 0.27419 | 15 | 0.48387 |
| 17101 | S22_18283961 | 22 | 18283961 | 13 | T | C | C | 0.72581 | T | 0.27419 | 15 | 0.48387 |
| 17102 | S22_18283983 | 22 | 18283983 | 22 | C | T | C | 0.87097 | T | 0.12903 | 8 | 0.25806 |
| 17103 | S22_18477709 | 22 | 18477709 | 193726 | G | A | A | 0.79032 | G | 0.20968 | 9 | 0.29032 |
| 17104 | S22_18723682 | 22 | 18723682 | 245973 | T | C | T | 0.85484 | C | 0.14516 | 9 | 0.29032 |
| 17105 | S22_18723688 | 22 | 18723688 | 6 | G | C | G | 0.80645 | C | 0.19355 | 10 | 0.32258 |
| 17106 | S22_18723699 | 22 | 18723699 | 11 | A | G | A | 0.70968 | G | 0.29032 | 14 | 0.45161 |
| 17107 | S22_18762599 | 22 | 18762599 | 38900 | C | T | C | 0.8871 | T | 0.1129 | 7 | 0.22581 |
| 17108 | S22_19053216 | 22 | 19053216 | 290617 | T | G | T | 0.93548 | G | 0.06452 | 4 | 0.12903 |
| 17109 | S22_19053281 | 22 | 19053281 | 65 | C | A | C | 0.95161 | A | 0.04839 | 3 | 0.09677 |
| 17110 | S22_19098328 | 22 | 19098328 | 45047 | C | T | C | 0.77419 | T | 0.22581 | 12 | 0.3871 |
| 17111 | S22_19098378 | 22 | 19098378 | 50 | T | C | T | 0.77419 | C | 0.22581 | 12 | 0.3871 |
| 17112 | S22_19098498 | 22 | 19098498 | 120 | G | A | G | 0.87097 | A | 0.12903 | 8 | 0.25806 |
| 17113 | S22_19098523 | 22 | 19098523 | 25 | T | C | T | 0.77419 | C | 0.22581 | 12 | 0.3871 |
| 17114 | S22_19838569 | 22 | 19838569 | 740046 | A | G | A | 0.91935 | G | 0.08065 | 5 | 0.16129 |
| 17115 | S22_19838633 | 22 | 19838633 | 64 | G | A | G | 0.8871 | A | 0.1129 | 7 | 0.22581 |
| 17116 | S22_20492520 | 22 | 20492520 | 653887 | G | A | G | 0.74194 | A | 0.25806 | 14 | 0.45161 |

| Site number | SNP name | Chromosome | Physical position | Physical distance from the previous SNP | Reference allele | Alternative allele | Major allele | Major allele frequency | Minor allele | Minor allele frequency | Number heterozygous | Heterozygosity |
|---|---|---|---|---|---|---|---|---|---|---|---|---|
| 17117 | S22_20829956 | 22 | 20829956 | 337436 | A | C | A | 0.87097 | C | 0.12903 | 4 | 0.12903 |
| 17118 | S22_20830007 | 22 | 20830007 | 51 | G | A | G | 0.93548 | A | 0.06452 | 4 | 0.12903 |
| 17119 | S22_20830060 | 22 | 20830060 | 53 | C | G | C | 0.6129 | G | 0.3871 | 14 | 0.45161 |
| 17120 | S22_20880385 | 22 | 20880385 | 50325 | G | A | G | 0.82258 | A | 0.17742 | 11 | 0.35484 |
| 17121 | S22_20880412 | 22 | 20880412 | 27 | A | G | A | 0.82258 | G | 0.17742 | 11 | 0.35484 |
| 17122 | S22_20959553 | 22 | 20959553 | 79141 | T | C | T | 0.91935 | C | 0.08065 | 5 | 0.16129 |
| 17123 | S22_20959580 | 22 | 20959580 | 27 | G | A | G | 0.8871 | A | 0.1129 | 7 | 0.22581 |
| 17124 | S22_20959653 | 22 | 20959653 | 73 | G | A | G | 0.91935 | A | 0.08065 | 5 | 0.16129 |
| 17125 | S22_20959705 | 22 | 20959705 | 52 | A | T | A | 0.91935 | T | 0.08065 | 5 | 0.16129 |
| 17126 | S22_20959734 | 22 | 20959734 | 29 | T | C | T | 0.8871 | C | 0.1129 | 5 | 0.16129 |
| 17127 | S22_20959737 | 22 | 20959737 | 3 | G | A | G | 0.91935 | A | 0.08065 | 5 | 0.16129 |
| 17128 | S22_21128637 | 22 | 21128637 | 168900 | T | C | T | 0.79032 | C | 0.20968 | 9 | 0.29032 |
| 17129 | S22_21128681 | 22 | 21128681 | 44 | A | G | A | 0.79032 | G | 0.20968 | 9 | 0.29032 |
| 17130 | S22_21128719 | 22 | 21128719 | 38 | G | A | G | 0.79032 | A | 0.20968 | 9 | 0.29032 |
| 17131 | S22_21128791 | 22 | 21128791 | 72 | C | T | C | 0.79032 | T | 0.20968 | 9 | 0.29032 |
| 17132 | S22_21378140 | 22 | 21378140 | 249349 | G | A | A | 0.82258 | G | 0.17742 | 11 | 0.35484 |
| 17133 | S22_21378189 | 22 | 21378189 | 49 | C | T | T | 0.87097 | C | 0.12903 | 8 | 0.25806 |
| 17134 | S22_21397305 | 22 | 21397305 | 19116 | C | T | C | 0.8871 | T | 0.1129 | 5 | 0.16129 |
| 17135 | S22_21397315 | 22 | 21397315 | 10 | T | C | T | 0.66129 | C | 0.33871 | 17 | 0.54839 |
| 17136 | S22_21420250 | 22 | 21420250 | 22935 | C | A | C | 0.93548 | A | 0.06452 | 4 | 0.12903 |
| 17137 | S22_21420276 | 22 | 21420276 | 26 | T | G | T | 0.90323 | G | 0.09677 | 6 | 0.19355 |
| 17138 | S22_21420345 | 22 | 21420345 | 69 | T | C | T | 0.56452 | C | 0.43548 | 15 | 0.48387 |
| 17139 | S22_21646542 | 22 | 21646542 | 226197 | A | C | C | 0.51613 | A | 0.48387 | 18 | 0.58065 |
| 17140 | S22_21646654 | 22 | 21646654 | 112 | G | A | G | 0.64516 | A | 0.35484 | 18 | 0.58065 |
| 17141 | S22_21646664 | 22 | 21646664 | 10 | C | T | C | 0.90323 | T | 0.09677 | 6 | 0.19355 |
| 17142 | S22_21646674 | 22 | 21646674 | 10 | G | A | A | 0.51613 | G | 0.48387 | 18 | 0.58065 |
| 17143 | S22_22498902 | 22 | 22498902 | 852228 | A | G | G | 0.85484 | A | 0.14516 | 9 | 0.29032 |
| 17144 | S22_22921113 | 22 | 22921113 | 422211 | G | A | G | 0.80645 | A | 0.19355 | 10 | 0.32258 |
| 17145 | S22_22921127 | 22 | 22921127 | 14 | T | C | T | 0.64516 | C | 0.35484 | 12 | 0.3871 |
| 17146 | S22_22973613 | 22 | 22973613 | 52486 | G | T | G | 0.83871 | T | 0.16129 | 10 | 0.32258 |
| 17147 | S22_23011676 | 22 | 23011676 | 38063 | C | T | C | 0.8871 | T | 0.1129 | 5 | 0.16129 |
| 17148 | S22_23091614 | 22 | 23091614 | 79938 | C | A | C | 0.93548 | A | 0.06452 | 4 | 0.12903 |
| 17149 | S22_23091695 | 22 | 23091695 | 81 | C | A | C | 0.8871 | A | 0.1129 | 7 | 0.22581 |
| 17150 | S22_23541189 | 22 | 23541189 | 449494 | C | T | C | 0.90323 | T | 0.09677 | 6 | 0.19355 |
| 17151 | S22_23541241 | 22 | 23541241 | 52 | A | G | A | 0.64516 | G | 0.35484 | 10 | 0.32258 |
| 17152 | S22_23541245 | 22 | 23541245 | 4 | G | T | G | 0.64516 | T | 0.35484 | 10 | 0.32258 |
| 17153 | S22_23541272 | 22 | 23541272 | 27 | C | T | C | 0.64516 | T | 0.35484 | 10 | 0.32258 |
| 17154 | S22_23541281 | 22 | 23541281 | 9 | C | T | C | 0.64516 | T | 0.35484 | 10 | 0.32258 |
| 17155 | S22_23541318 | 22 | 23541318 | 37 | A | C | A | 0.91935 | C | 0.08065 | 3 | 0.09677 |
| 17156 | S22_23541390 | 22 | 23541390 | 72 | T | C | T | 0.90323 | C | 0.09677 | 6 | 0.19355 |
| 17157 | S22_23541406 | 22 | 23541406 | 16 | C | T | C | 0.90323 | T | 0.09677 | 6 | 0.19355 |
| 17158 | S22_23541421 | 22 | 23541421 | 15 | T | C | T | 0.62903 | C | 0.37097 | 11 | 0.35484 |
| 17159 | S22_23541422 | 22 | 23541422 | 1 | G | A | G | 0.91935 | A | 0.08065 | 5 | 0.16129 |
| 17160 | S22_23812308 | 22 | 23812308 | 270886 | C | T | C | 0.95161 | T | 0.04839 | 3 | 0.09677 |
| 17161 | S22_23812309 | 22 | 23812309 | 1 | G | A | G | 0.8871 | A | 0.1129 | 7 | 0.22581 |
| 17162 | S22_23859953 | 22 | 23859953 | 47644 | C | A | C | 0.77419 | A | 0.22581 | 12 | 0.3871 |
| 17163 | S22_23860173 | 22 | 23860173 | 220 | G | A | G | 0.95161 | A | 0.04839 | 3 | 0.09677 |
| 17164 | S22_23891500 | 22 | 23891500 | 31327 | C | T | C | 0.59677 | T | 0.40323 | 17 | 0.54839 |
| 17165 | S22_23891506 | 22 | 23891506 | 6 | T | C | T | 0.91935 | C | 0.08065 | 5 | 0.16129 |
| 17166 | S22_23891517 | 22 | 23891517 | 11 | G | A | G | 0.91935 | A | 0.08065 | 5 | 0.16129 |
| 17167 | S22_23891605 | 22 | 23891605 | 88 | T | C | C | 0.72581 | T | 0.27419 | 15 | 0.48387 |
| 17168 | S22_23891613 | 22 | 23891613 | 8 | T | C | T | 0.93548 | C | 0.06452 | 4 | 0.12903 |
| 17169 | S22_23891630 | 22 | 23891630 | 17 | C | A | C | 0.91935 | A | 0.08065 | 5 | 0.16129 |
| 17170 | S22_23891663 | 22 | 23891663 | 33 | C | T | C | 0.59677 | T | 0.40323 | 17 | 0.54839 |

| Site number | SNP name | Chromosome | Physical position | Physical distance from the previous SNP | Reference allele | Alternative allele | Major allele | Major allele frequency | Minor allele | Minor allele frequency | Number heterozygous | Heterozygosity |
|---|---|---|---|---|---|---|---|---|---|---|---|---|
| 17171 | S22_24108669 | 22 | 24108669 | 217006 | T | A | T | 0.56452 | A | 0.43548 | 17 | 0.54839 |
| 17172 | S22_24108695 | 22 | 24108695 | 26 | T | C | C | 0.54839 | T | 0.45161 | 20 | 0.64516 |
| 17173 | S22_24805164 | 22 | 24805164 | 696469 | T | C | T | 0.95161 | C | 0.04839 | 3 | 0.09677 |
| 17174 | S22_25093966 | 22 | 25093966 | 288802 | T | C | T | 0.77419 | C | 0.22581 | 12 | 0.3871 |
| 17175 | S22_25283978 | 22 | 25283978 | 190012 | C | T | C | 0.90323 | T | 0.09677 | 6 | 0.19355 |
| 17176 | S22_25284147 | 22 | 25284147 | 169 | G | A | G | 0.87097 | A | 0.12903 | 8 | 0.25806 |
| 17177 | S22_25799100 | 22 | 25799100 | 514953 | G | A | G | 0.70968 | A | 0.29032 | 14 | 0.45161 |
| 17178 | S22_25816233 | 22 | 25816233 | 17133 | A | G | A | 0.83871 | G | 0.16129 | 8 | 0.25806 |
| 17179 | S22_25816419 | 22 | 25816419 | 186 | C | T | T | 0.90323 | C | 0.09677 | 6 | 0.19355 |
| 17180 | S22_26038659 | 22 | 26038659 | 222240 | C | T | C | 0.8871 | T | 0.1129 | 7 | 0.22581 |
| 17181 | S22_26048401 | 22 | 26048401 | 9742 | G | C | G | 0.91935 | C | 0.08065 | 5 | 0.16129 |
| 17182 | S22_26048470 | 22 | 26048470 | 69 | G | A | G | 0.82258 | A | 0.17742 | 9 | 0.29032 |
| 17183 | S22_26133121 | 22 | 26133121 | 84651 | A | C | A | 0.95161 | C | 0.04839 | 3 | 0.09677 |
| 17184 | S22_26133153 | 22 | 26133153 | 32 | A | C | A | 0.95161 | C | 0.04839 | 3 | 0.09677 |
| 17185 | S22_26133230 | 22 | 26133230 | 77 | A | C | A | 0.95161 | C | 0.04839 | 3 | 0.09677 |
| 17186 | S22_26133253 | 22 | 26133253 | 23 | G | A | G | 0.95161 | A | 0.04839 | 3 | 0.09677 |
| 17187 | S22_26133339 | 22 | 26133339 | 86 | G | C | G | 0.91935 | C | 0.08065 | 5 | 0.16129 |
| 17188 | S22_26544963 | 22 | 26544963 | 411624 | C | T | C | 0.93548 | T | 0.06452 | 4 | 0.12903 |
| 17189 | S22_26545102 | 22 | 26545102 | 139 | C | T | C | 0.91935 | T | 0.08065 | 5 | 0.16129 |
| 17190 | S22_26545144 | 22 | 26545144 | 42 | A | T | A | 0.93548 | T | 0.06452 | 4 | 0.12903 |
| 17191 | S22_27236576 | 22 | 27236576 | 691432 | A | G | A | 0.87097 | G | 0.12903 | 8 | 0.25806 |
| 17192 | S22_27236629 | 22 | 27236629 | 53 | C | T | C | 0.93548 | T | 0.06452 | 4 | 0.12903 |
| 17193 | S22_27841514 | 22 | 27841514 | 604885 | C | T | C | 0.85484 | T | 0.14516 | 7 | 0.22581 |
| 17194 | S22_27841675 | 22 | 27841675 | 161 | C | T | C | 0.82258 | T | 0.17742 | 9 | 0.29032 |
| 17195 | S22_27841733 | 22 | 27841733 | 58 | T | A | T | 0.79032 | A | 0.20968 | 11 | 0.35484 |
| 17196 | S22_27841759 | 22 | 27841759 | 26 | G | C | G | 0.90323 | C | 0.09677 | 4 | 0.12903 |
| 17197 | S22_27857361 | 22 | 27857361 | 15602 | A | G | A | 0.91935 | G | 0.08065 | 5 | 0.16129 |
| 17198 | S22_27932727 | 22 | 27932727 | 75366 | A | T | A | 0.95161 | T | 0.04839 | 3 | 0.09677 |
| 17199 | S22_27932870 | 22 | 27932870 | 143 | G | A | G | 0.85484 | A | 0.14516 | 9 | 0.29032 |
| 17200 | S22_28267698 | 22 | 28267698 | 334828 | T | C | C | 0.87097 | T | 0.12903 | 6 | 0.19355 |
| 17201 | S22_28267708 | 22 | 28267708 | 10 | C | T | C | 0.79032 | T | 0.20968 | 11 | 0.35484 |
| 17202 | S22_28267744 | 22 | 28267744 | 36 | T | C | T | 0.93548 | C | 0.06452 | 4 | 0.12903 |
| 17203 | S22_28267942 | 22 | 28267942 | 198 | T | C | T | 0.6129 | C | 0.3871 | 16 | 0.51613 |
| 17204 | S22_28669524 | 22 | 28669524 | 401582 | C | T | C | 0.8871 | T | 0.1129 | 7 | 0.22581 |
| 17205 | S22_29438951 | 22 | 29438951 | 769427 | C | T | C | 0.8871 | T | 0.1129 | 7 | 0.22581 |
| 17206 | S22_29439078 | 22 | 29439078 | 127 | C | G | C | 0.8871 | G | 0.1129 | 7 | 0.22581 |
| 17207 | S22_29492412 | 22 | 29492412 | 53334 | A | G | A | 0.77419 | G | 0.22581 | 14 | 0.45161 |
| 17208 | S22_29936405 | 22 | 29936405 | 443993 | G | A | G | 0.69355 | A | 0.30645 | 13 | 0.41935 |
| 17209 | S22_29936485 | 22 | 29936485 | 80 | A | G | A | 0.66129 | G | 0.33871 | 15 | 0.48387 |
| 17210 | S22_29936598 | 22 | 29936598 | 113 | G | A | G | 0.66129 | A | 0.33871 | 15 | 0.48387 |
| 17211 | S22_30208435 | 22 | 30208435 | 271837 | G | A | A | 0.67742 | G | 0.32258 | 14 | 0.45161 |
| 17212 | S22_30275661 | 22 | 30275661 | 67226 | C | T | C | 0.93548 | T | 0.06452 | 4 | 0.12903 |
| 17213 | S22_30275770 | 22 | 30275770 | 109 | G | A | A | 0.75806 | G | 0.24194 | 15 | 0.48387 |
| 17214 | S22_30297159 | 22 | 30297159 | 21389 | T | C | T | 0.87097 | C | 0.12903 | 8 | 0.25806 |
| 17215 | S22_30576099 | 22 | 30576099 | 278940 | C | A | C | 0.95161 | A | 0.04839 | 3 | 0.09677 |
| 17216 | S22_30684159 | 22 | 30684159 | 108060 | C | T | C | 0.95161 | T | 0.04839 | 3 | 0.09677 |
| 17217 | S22_30684193 | 22 | 30684193 | 34 | G | A | A | 0.51613 | G | 0.48387 | 20 | 0.64516 |
| 17218 | S22_31008975 | 22 | 31008975 | 324782 | G | A | G | 0.70968 | A | 0.29032 | 10 | 0.32258 |
| 17219 | S22_31009016 | 22 | 31009016 | 41 | T | C | C | 0.91935 | T | 0.08065 | 3 | 0.09677 |
| 17220 | S22_31080589 | 22 | 31080589 | 71573 | G | T | G | 0.79032 | T | 0.20968 | 9 | 0.29032 |
| 17221 | S22_31729548 | 22 | 31729548 | 648959 | T | C | C | 0.59677 | T | 0.40323 | 15 | 0.48387 |
| 17222 | S22_31877382 | 22 | 31877382 | 147834 | A | G | A | 0.66129 | G | 0.33871 | 13 | 0.41935 |
| 17223 | S22_32068771 | 22 | 32068771 | 191389 | C | A | C | 0.93548 | A | 0.06452 | 4 | 0.12903 |
| 17224 | S22_32068772 | 22 | 32068772 | 1 | C | T | C | 0.67742 | T | 0.32258 | 16 | 0.51613 |

| Site number | SNP name | Chromosome | Physical position | Physical distance from the previous SNP | Reference allele | Alternative allele | Major allele | Major allele frequency | Minor allele | Minor allele frequency | Number heterozygous | Heterozygosity |
|---|---|---|---|---|---|---|---|---|---|---|---|---|
| 17225 | S22_32068820 | 22 | 32068820 | 48 | C | T | C | 0.91935 | T | 0.08065 | 3 | 0.09677 |
| 17226 | S22_32177679 | 22 | 32177679 | 108859 | G | A | G | 0.90323 | A | 0.09677 | 6 | 0.19355 |
| 17227 | S22_32177734 | 22 | 32177734 | 55 | C | G | C | 0.91935 | G | 0.08065 | 5 | 0.16129 |
| 17228 | S22_32181701 | 22 | 32181701 | 3967 | C | T | C | 0.95161 | T | 0.04839 | 3 | 0.09677 |
| 17229 | S22_32181835 | 22 | 32181835 | 134 | A | G | A | 0.87097 | G | 0.12903 | 6 | 0.19355 |
| 17230 | S22_32341988 | 22 | 32341988 | 160153 | G | T | G | 0.77419 | T | 0.22581 | 10 | 0.32258 |
| 17231 | S22_33223168 | 22 | 33223168 | 881180 | A | T | A | 0.77419 | T | 0.22581 | 10 | 0.32258 |
| 17232 | S22_33223265 | 22 | 33223265 | 97 | G | A | G | 0.90323 | A | 0.09677 | 6 | 0.19355 |
| 17233 | S22_33223300 | 22 | 33223300 | 35 | A | G | A | 0.90323 | G | 0.09677 | 6 | 0.19355 |
| 17234 | S22_33223329 | 22 | 33223329 | 29 | G | A | G | 0.95161 | A | 0.04839 | 3 | 0.09677 |
| 17235 | S22_34687032 | 22 | 34687032 | 1463703 | G | A | G | 0.62903 | A | 0.37097 | 15 | 0.48387 |
| 17236 | S22_34687060 | 22 | 34687060 | 28 | G | C | G | 0.91935 | C | 0.08065 | 5 | 0.16129 |
| 17237 | S22_35300850 | 22 | 35300850 | 613790 | G | A | G | 0.91935 | A | 0.08065 | 5 | 0.16129 |
| 17238 | S22_35300888 | 22 | 35300888 | 38 | G | A | G | 0.91935 | A | 0.08065 | 5 | 0.16129 |
| 17239 | S22_35687882 | 22 | 35687882 | 386994 | T | C | T | 0.90323 | C | 0.09677 | 6 | 0.19355 |
| 17240 | S22_35687923 | 22 | 35687923 | 41 | C | T | C | 0.95161 | T | 0.04839 | 3 | 0.09677 |
| 17241 | S22_35896771 | 22 | 35896771 | 208848 | C | G | G | 0.51613 | C | 0.48387 | 14 | 0.45161 |
| 17242 | S22_36381434 | 22 | 36381434 | 484663 | G | A | G | 0.62903 | A | 0.37097 | 13 | 0.41935 |
| 17243 | S22_36469442 | 22 | 36469442 | 88008 | A | C | A | 0.91935 | C | 0.08065 | 5 | 0.16129 |
| 17244 | S22_36469670 | 22 | 36469670 | 228 | A | G | G | 0.67742 | A | 0.32258 | 16 | 0.51613 |
| 17245 | S22_36651925 | 22 | 36651925 | 182255 | A | G | A | 0.6129 | G | 0.3871 | 16 | 0.51613 |
| 17246 | S22_36651982 | 22 | 36651982 | 57 | C | T | C | 0.91935 | T | 0.08065 | 5 | 0.16129 |
| 17247 | S22_36771069 | 22 | 36771069 | 119087 | A | G | A | 0.59677 | G | 0.40323 | 15 | 0.48387 |
| 17248 | S22_37066264 | 22 | 37066264 | 295195 | C | T | T | 0.56452 | C | 0.43548 | 11 | 0.35484 |
| 17249 | S22_37289436 | 22 | 37289436 | 223172 | A | G | A | 0.6129 | G | 0.3871 | 14 | 0.45161 |
| 17250 | S22_37496726 | 22 | 37496726 | 207290 | C | T | C | 0.8871 | T | 0.1129 | 5 | 0.16129 |
| 17251 | S22_37782761 | 22 | 37782761 | 286035 | C | T | C | 0.74194 | T | 0.25806 | 12 | 0.3871 |
| 17252 | S22_37951138 | 22 | 37951138 | 168377 | A | G | A | 0.53226 | G | 0.46774 | 17 | 0.54839 |
| 17253 | S22_38489611 | 22 | 38489611 | 538473 | C | T | C | 0.93548 | T | 0.06452 | 4 | 0.12903 |
| 17254 | S22_38704092 | 22 | 38704092 | 214481 | T | C | C | 0.87097 | T | 0.12903 | 8 | 0.25806 |
| 17255 | S22_38888099 | 22 | 38888099 | 184007 | C | T | C | 0.62903 | T | 0.37097 | 11 | 0.35484 |
| 17256 | S22_39217246 | 22 | 39217246 | 329147 | C | T | C | 0.93548 | T | 0.06452 | 4 | 0.12903 |
| 17257 | S22_39217438 | 22 | 39217438 | 192 | C | T | C | 0.66129 | T | 0.33871 | 17 | 0.54839 |
| 17258 | S22_39346280 | 22 | 39346280 | 128842 | G | A | G | 0.93548 | A | 0.06452 | 4 | 0.12903 |
| 17259 | S22_39404897 | 22 | 39404897 | 58617 | G | A | G | 0.83871 | A | 0.16129 | 8 | 0.25806 |
| 17260 | S22_39772107 | 22 | 39772107 | 367210 | T | C | T | 0.93548 | C | 0.06452 | 4 | 0.12903 |
| 17261 | S22_39772125 | 22 | 39772125 | 18 | G | A | G | 0.8871 | A | 0.1129 | 7 | 0.22581 |
| 17262 | S22_39772131 | 22 | 39772131 | 6 | G | A | G | 0.79032 | A | 0.20968 | 11 | 0.35484 |
| 17263 | S22_39772228 | 22 | 39772228 | 97 | G | T | G | 0.83871 | T | 0.16129 | 10 | 0.32258 |
| 17264 | S22_39978824 | 22 | 39978824 | 206596 | C | T | C | 0.91935 | T | 0.08065 | 5 | 0.16129 |
| 17265 | S22_39978953 | 22 | 39978953 | 129 | G | A | G | 0.93548 | A | 0.06452 | 4 | 0.12903 |
| 17266 | S22_40002145 | 22 | 40002145 | 23192 | G | A | G | 0.93548 | A | 0.06452 | 4 | 0.12903 |
| 17267 | S22_40002179 | 22 | 40002179 | 34 | T | C | T | 0.95161 | C | 0.04839 | 3 | 0.09677 |
| 17268 | S22_40661201 | 22 | 40661201 | 659022 | C | G | C | 0.90323 | G | 0.09677 | 4 | 0.12903 |
| 17269 | S22_40661271 | 22 | 40661271 | 70 | C | T | C | 0.90323 | T | 0.09677 | 4 | 0.12903 |
| 17270 | S22_40661308 | 22 | 40661308 | 37 | A | G | A | 0.83871 | G | 0.16129 | 6 | 0.19355 |
| 17271 | S22_40686386 | 22 | 40686386 | 25078 | G | A | G | 0.51613 | A | 0.48387 | 18 | 0.58065 |
| 17272 | S22_40854238 | 22 | 40854238 | 167852 | C | T | C | 0.69355 | T | 0.30645 | 13 | 0.41935 |
| 17273 | S22_40854304 | 22 | 40854304 | 66 | A | T | A | 0.56452 | T | 0.43548 | 17 | 0.54839 |
| 17274 | S22_41071604 | 22 | 41071604 | 217300 | C | T | C | 0.82258 | T | 0.17742 | 9 | 0.29032 |
| 17275 | S22_41116033 | 22 | 41116033 | 44429 | G | A | G | 0.77419 | A | 0.22581 | 12 | 0.3871 |
| 17276 | S22_41116060 | 22 | 41116060 | 27 | T | C | T | 0.77419 | C | 0.22581 | 12 | 0.3871 |
| 17277 | S22_41116118 | 22 | 41116118 | 58 | A | G | G | 0.77419 | A | 0.22581 | 12 | 0.3871 |
| 17278 | S22_41116189 | 22 | 41116189 | 71 | C | T | C | 0.85484 | T | 0.14516 | 7 | 0.22581 |

| Site number | SNP name | Chromosome | Physical position | Physical distance from the previous SNP | Reference allele | Alternative allele | Major allele | Major allele frequency | Minor allele | Minor allele frequency | Number heterozygous | Heterozygosity |
|---|---|---|---|---|---|---|---|---|---|---|---|---|
| 17279 | S22_41116199 | 22 | 41116199 | 10 | T | G | T | 0.85484 | G | 0.14516 | 7 | 0.22581 |
| 17280 | S22_41116208 | 22 | 41116208 | 9 | T | C | T | 0.85484 | C | 0.14516 | 7 | 0.22581 |
| 17281 | S22_41154063 | 22 | 41154063 | 37855 | T | C | T | 0.75806 | C | 0.24194 | 11 | 0.35484 |
| 17282 | S22_41272985 | 22 | 41272985 | 118922 | A | G | G | 0.56452 | A | 0.43548 | 15 | 0.48387 |
| 17283 | S22_41273081 | 22 | 41273081 | 96 | C | G | C | 0.90323 | G | 0.09677 | 4 | 0.12903 |
| 17284 | S22_41428521 | 22 | 41428521 | 155440 | C | T | C | 0.74194 | T | 0.25806 | 12 | 0.3871 |
| 17285 | S22_42364788 | 22 | 42364788 | 936267 | T | C | C | 0.8871 | T | 0.1129 | 5 | 0.16129 |
| 17286 | S22_42771698 | 22 | 42771698 | 406910 | G | A | G | 0.87097 | A | 0.12903 | 8 | 0.25806 |
| 17287 | S22_42771758 | 22 | 42771758 | 60 | C | T | C | 0.87097 | T | 0.12903 | 8 | 0.25806 |
| 17288 | S22_43003526 | 22 | 43003526 | 231768 | T | C | T | 0.66129 | C | 0.33871 | 13 | 0.41935 |
| 17289 | S22_43003674 | 22 | 43003674 | 148 | C | T | C | 0.75806 | T | 0.24194 | 13 | 0.41935 |
| 17290 | S22_43797937 | 22 | 43797937 | 794263 | C | T | C | 0.93548 | T | 0.06452 | 4 | 0.12903 |
| 17291 | S22_43985565 | 22 | 43985565 | 187628 | A | G | A | 0.77419 | G | 0.22581 | 14 | 0.45161 |
| 17292 | S22_44093851 | 22 | 44093851 | 108286 | G | A | G | 0.95161 | A | 0.04839 | 3 | 0.09677 |
| 17293 | S22_44310881 | 22 | 44310881 | 217030 | G | A | G | 0.95161 | A | 0.04839 | 3 | 0.09677 |
| 17294 | S22_44310902 | 22 | 44310902 | 21 | A | C | C | 0.83871 | A | 0.16129 | 10 | 0.32258 |
| 17295 | S22_44403106 | 22 | 44403106 | 92204 | C | T | C | 0.95161 | T | 0.04839 | 3 | 0.09677 |
| 17296 | S22_44403147 | 22 | 44403147 | 41 | C | T | C | 0.91935 | T | 0.08065 | 3 | 0.09677 |
| 17297 | S22_44403243 | 22 | 44403243 | 96 | G | C | G | 0.95161 | C | 0.04839 | 3 | 0.09677 |
| 17298 | S22_44473865 | 22 | 44473865 | 70622 | C | G | C | 0.93548 | G | 0.06452 | 4 | 0.12903 |
| 17299 | S22_44618691 | 22 | 44618691 | 144826 | G | A | G | 0.66129 | A | 0.33871 | 11 | 0.35484 |
| 17300 | S22_44707700 | 22 | 44707700 | 89009 | C | A | C | 0.8871 | A | 0.1129 | 7 | 0.22581 |
| 17301 | S22_44707807 | 22 | 44707807 | 107 | T | C | C | 0.54839 | T | 0.45161 | 14 | 0.45161 |
| 17302 | S22_45581167 | 22 | 45581167 | 873360 | C | T | T | 0.62903 | C | 0.37097 | 15 | 0.48387 |
| 17303 | S22_45581204 | 22 | 45581204 | 37 | A | C | A | 0.74194 | C | 0.25806 | 10 | 0.32258 |
| 17304 | S22_45581282 | 22 | 45581282 | 78 | T | C | T | 0.6129 | C | 0.3871 | 18 | 0.58065 |
| 17305 | S22_45581305 | 22 | 45581305 | 23 | G | C | G | 0.6129 | C | 0.3871 | 18 | 0.58065 |
| 17306 | S22_45951618 | 22 | 45951618 | 370313 | G | T | G | 0.72581 | T | 0.27419 | 11 | 0.35484 |
| 17307 | S22_46508029 | 22 | 46508029 | 556411 | G | A | G | 0.91935 | A | 0.08065 | 5 | 0.16129 |
| 17308 | S22_46830855 | 22 | 46830855 | 322826 | A | C | C | 0.74194 | A | 0.25806 | 16 | 0.51613 |
| 17309 | S22_46830869 | 22 | 46830869 | 14 | T | C | T | 0.85484 | C | 0.14516 | 5 | 0.16129 |
| 17310 | S22_46873909 | 22 | 46873909 | 43040 | A | C | A | 0.93548 | C | 0.06452 | 4 | 0.12903 |
| 17311 | S22_47730722 | 22 | 47730722 | 856813 | C | T | C | 0.72581 | T | 0.27419 | 9 | 0.29032 |
| 17312 | S22_47730795 | 22 | 47730795 | 73 | A | G | G | 0.87097 | A | 0.12903 | 8 | 0.25806 |
| 17313 | S22_47781397 | 22 | 47781397 | 50602 | G | A | G | 0.91935 | A | 0.08065 | 5 | 0.16129 |
| 17314 | S22_47880096 | 22 | 47880096 | 98699 | A | G | A | 0.72581 | G | 0.27419 | 15 | 0.48387 |
| 17315 | S22_48412177 | 22 | 48412177 | 532081 | T | G | T | 0.90323 | G | 0.09677 | 6 | 0.19355 |
| 17316 | S22_48412324 | 22 | 48412324 | 147 | C | A | C | 0.8871 | A | 0.1129 | 7 | 0.22581 |
| 17317 | S22_48424291 | 22 | 48424291 | 11967 | G | C | G | 0.79032 | C | 0.20968 | 9 | 0.29032 |
| 17318 | S22_48424407 | 22 | 48424407 | 116 | G | A | G | 0.77419 | A | 0.22581 | 8 | 0.25806 |
| 17319 | S22_48424430 | 22 | 48424430 | 23 | G | A | G | 0.77419 | A | 0.22581 | 8 | 0.25806 |
| 17320 | S22_48424456 | 22 | 48424456 | 26 | T | G | G | 0.77419 | T | 0.22581 | 8 | 0.25806 |
| 17321 | S22_48424461 | 22 | 48424461 | 5 | C | T | C | 0.77419 | T | 0.22581 | 8 | 0.25806 |
| 17322 | S22_49104427 | 22 | 49104427 | 679966 | G | A | G | 0.93548 | A | 0.06452 | 4 | 0.12903 |
| 17323 | S22_49104452 | 22 | 49104452 | 25 | A | G | A | 0.58065 | G | 0.41935 | 18 | 0.58065 |
| 17324 | S22_50554502 | 22 | 50554502 | 1450050 | C | T | T | 0.59677 | C | 0.40323 | 11 | 0.35484 |
| 17325 | S22_50554512 | 22 | 50554512 | 10 | T | G | T | 0.58065 | G | 0.41935 | 10 | 0.32258 |
| 17326 | S22_50554517 | 22 | 50554517 | 5 | G | C | G | 0.58065 | C | 0.41935 | 10 | 0.32258 |
| 17327 | S22_50864524 | 22 | 50864524 | 310007 | C | G | C | 0.77419 | G | 0.22581 | 12 | 0.3871 |
| 17328 | S22_51168761 | 22 | 51168761 | 304237 | G | A | A | 0.62903 | G | 0.37097 | 13 | 0.41935 |
| 17329 | S22_51168917 | 22 | 51168917 | 156 | A | G | A | 0.66129 | G | 0.33871 | 13 | 0.41935 |
| 17330 | S22_51431576 | 22 | 51431576 | 262659 | C | T | C | 0.83871 | T | 0.16129 | 8 | 0.25806 |
| 17331 | S22_51615783 | 22 | 51615783 | 184207 | A | C | A | 0.93548 | C | 0.06452 | 4 | 0.12903 |
| 17332 | S22_51615866 | 22 | 51615866 | 83 | C | G | C | 0.93548 | G | 0.06452 | 4 | 0.12903 |

| Site number | SNP name | Chromosome | Physical position | Physical distance from the previous SNP | Reference allele | Alternative allele | Major allele | Major allele frequency | Minor allele | Minor allele frequency | Number heterozygous | Heterozygosity |
|---|---|---|---|---|---|---|---|---|---|---|---|---|
| 17333 | S22_51895801 | 22 | 51895801 | 279935 | A | G | A | 0.93548 | G | 0.06452 | 4 | 0.12903 |
| 17334 | S22_52130587 | 22 | 52130587 | 234786 | C | T | C | 0.85484 | T | 0.14516 | 5 | 0.16129 |
| 17335 | S22_52130600 | 22 | 52130600 | 13 | G | C | G | 0.85484 | C | 0.14516 | 5 | 0.16129 |
| 17336 | S22_52130646 | 22 | 52130646 | 46 | C | T | C | 0.66129 | T | 0.33871 | 13 | 0.41935 |
| 17337 | S22_52130697 | 22 | 52130697 | 51 | G | A | G | 0.66129 | A | 0.33871 | 13 | 0.41935 |
| 17338 | S22_52130731 | 22 | 52130731 | 34 | G | A | A | 0.85484 | G | 0.14516 | 7 | 0.22581 |
| 17339 | S22_52185437 | 22 | 52185437 | 54706 | G | A | G | 0.91935 | A | 0.08065 | 5 | 0.16129 |
| 17340 | S22_52185446 | 22 | 52185446 | 9 | G | T | G | 0.83871 | T | 0.16129 | 8 | 0.25806 |
| 17341 | S22_52369894 | 22 | 52369894 | 184448 | C | T | C | 0.8871 | T | 0.1129 | 7 | 0.22581 |
| 17342 | S22_52419905 | 22 | 52419905 | 50011 | G | A | G | 0.58065 | A | 0.41935 | 16 | 0.51613 |
| 17343 | S22_52419940 | 22 | 52419940 | 35 | C | T | C | 0.79032 | T | 0.20968 | 11 | 0.35484 |
| 17344 | S22_52419981 | 22 | 52419981 | 41 | G | T | G | 0.80645 | T | 0.19355 | 8 | 0.25806 |
| 17345 | S22_52420083 | 22 | 52420083 | 102 | C | A | A | 0.85484 | C | 0.14516 | 7 | 0.22581 |
| 17346 | S22_52620830 | 22 | 52620830 | 200747 | C | T | C | 0.82258 | T | 0.17742 | 9 | 0.29032 |
| 17347 | S22_52620900 | 22 | 52620900 | 70 | G | A | G | 0.59677 | A | 0.40323 | 13 | 0.41935 |
| 17348 | S22_52620991 | 22 | 52620991 | 91 | G | A | G | 0.93548 | A | 0.06452 | 4 | 0.12903 |
| 17349 | S22_52621048 | 22 | 52621048 | 57 | T | C | T | 0.93548 | C | 0.06452 | 4 | 0.12903 |
| 17350 | S22_52621072 | 22 | 52621072 | 24 | C | T | C | 0.83871 | T | 0.16129 | 10 | 0.32258 |
| 17351 | S22_52955833 | 22 | 52955833 | 334761 | G | A | G | 0.72581 | A | 0.27419 | 13 | 0.41935 |
| 17352 | S22_52955841 | 22 | 52955841 | 8 | G | A | G | 0.87097 | A | 0.12903 | 6 | 0.19355 |
| 17353 | S22_53006323 | 22 | 53006323 | 50482 | G | A | G | 0.93548 | A | 0.06452 | 4 | 0.12903 |
| 17354 | S22_53006361 | 22 | 53006361 | 38 | C | G | G | 0.79032 | C | 0.20968 | 11 | 0.35484 |
| 17355 | S22_53006428 | 22 | 53006428 | 67 | C | T | C | 0.90323 | T | 0.09677 | 4 | 0.12903 |
| 17356 | S22_53392972 | 22 | 53392972 | 386544 | C | T | C | 0.93548 | T | 0.06452 | 2 | 0.06452 |
| 17357 | S22_53392974 | 22 | 53392974 | 2 | C | T | C | 0.70968 | T | 0.29032 | 8 | 0.25806 |
| 17358 | S22_53392979 | 22 | 53392979 | 5 | G | A | G | 0.91935 | A | 0.08065 | 5 | 0.16129 |
| 17359 | S22_53687638 | 22 | 53687638 | 294659 | C | T | C | 0.83871 | T | 0.16129 | 6 | 0.19355 |
| 17360 | S22_53687673 | 22 | 53687673 | 35 | A | G | G | 0.51613 | A | 0.48387 | 14 | 0.45161 |
| 17361 | S22_53719510 | 22 | 53719510 | 31837 | G | A | G | 0.66129 | A | 0.33871 | 17 | 0.54839 |
| 17362 | S22_53837106 | 22 | 53837106 | 117596 | A | T | A | 0.79032 | T | 0.20968 | 11 | 0.35484 |
| 17363 | S22_53837195 | 22 | 53837195 | 89 | G | C | G | 0.62903 | C | 0.37097 | 17 | 0.54839 |
| 17364 | S22_53837218 | 22 | 53837218 | 23 | G | A | G | 0.93548 | A | 0.06452 | 4 | 0.12903 |
| 17365 | S22_53837249 | 22 | 53837249 | 31 | A | G | G | 0.93548 | A | 0.06452 | 2 | 0.06452 |
| 17366 | S22_53837258 | 22 | 53837258 | 9 | C | T | C | 0.93548 | T | 0.06452 | 4 | 0.12903 |
| 17367 | S22_53837293 | 22 | 53837293 | 35 | G | A | G | 0.82258 | A | 0.17742 | 11 | 0.35484 |
| 17368 | S22_53867930 | 22 | 53867930 | 30637 | T | C | C | 0.70968 | T | 0.29032 | 12 | 0.3871 |
| 17369 | S22_53867943 | 22 | 53867943 | 13 | G | A | G | 0.93548 | A | 0.06452 | 2 | 0.06452 |
| 17370 | S22_53867994 | 22 | 53867994 | 51 | G | A | G | 0.87097 | A | 0.12903 | 8 | 0.25806 |
| 17371 | S22_53868036 | 22 | 53868036 | 42 | A | G | A | 0.80645 | G | 0.19355 | 10 | 0.32258 |
| 17372 | S22_53868067 | 22 | 53868067 | 31 | T | C | T | 0.93548 | C | 0.06452 | 4 | 0.12903 |
| 17373 | S22_53925922 | 22 | 53925922 | 57855 | C | T | C | 0.95161 | T | 0.04839 | 3 | 0.09677 |
| 17374 | S22_53925933 | 22 | 53925933 | 11 | C | T | C | 0.95161 | T | 0.04839 | 1 | 0.03226 |
| 17375 | S22_53925961 | 22 | 53925961 | 28 | G | C | G | 0.91935 | C | 0.08065 | 5 | 0.16129 |
| 17376 | S22_53925982 | 22 | 53925982 | 21 | C | A | C | 0.54839 | A | 0.45161 | 14 | 0.45161 |
| 17377 | S22_53926006 | 22 | 53926006 | 24 | G | A | A | 0.54839 | G | 0.45161 | 14 | 0.45161 |
| 17378 | S22_53926024 | 22 | 53926024 | 18 | A | G | A | 0.62903 | G | 0.37097 | 13 | 0.41935 |
| 17379 | S22_53926045 | 22 | 53926045 | 21 | T | C | C | 0.54839 | T | 0.45161 | 14 | 0.45161 |
| 17380 | S22_53926046 | 22 | 53926046 | 1 | G | A | A | 0.54839 | G | 0.45161 | 14 | 0.45161 |
| 17381 | S22_53926062 | 22 | 53926062 | 16 | A | G | G | 0.54839 | A | 0.45161 | 14 | 0.45161 |
| 17382 | S22_53926065 | 22 | 53926065 | 3 | G | A | A | 0.54839 | G | 0.45161 | 14 | 0.45161 |
| 17383 | S22_53989828 | 22 | 53989828 | 63763 | G | A | G | 0.66129 | A | 0.33871 | 11 | 0.35484 |
| 17384 | S22_54105111 | 22 | 54105111 | 115283 | G | A | G | 0.90323 | A | 0.09677 | 6 | 0.19355 |
| 17385 | S22_54114726 | 22 | 54114726 | 9615 | T | C | T | 0.8871 | C | 0.1129 | 7 | 0.22581 |
| 17386 | S22_54772795 | 22 | 54772795 | 658069 | A | G | G | 0.6129 | A | 0.3871 | 16 | 0.51613 |

| Site number | SNP name | Chromosome | Physical position | Physical distance from the previous SNP | Reference allele | Alternative allele | Major allele | Major allele frequency | Minor allele | Minor allele frequency | Number heterozygous | Heterozygosity |
|---|---|---|---|---|---|---|---|---|---|---|---|---|
| 17387 | S22_54899703 | 22 | 54899703 | 126908 | T | C | T | 0.90323 | C | 0.09677 | 6 | 0.19355 |
| 17388 | S22_54899730 | 22 | 54899730 | 27 | C | T | C | 0.90323 | T | 0.09677 | 6 | 0.19355 |
| 17389 | S22_54899736 | 22 | 54899736 | 6 | C | T | C | 0.54839 | T | 0.45161 | 20 | 0.64516 |
| 17390 | S22_54899746 | 22 | 54899746 | 10 | C | T | C | 0.82258 | T | 0.17742 | 9 | 0.29032 |
| 17391 | S22_54899791 | 22 | 54899791 | 45 | G | T | T | 0.67742 | G | 0.32258 | 16 | 0.51613 |
| 17392 | S22_55326164 | 22 | 55326164 | 426373 | C | T | C | 0.93548 | T | 0.06452 | 4 | 0.12903 |
| 17393 | S22_55516791 | 22 | 55516791 | 190627 | G | A | A | 0.5 | G | 0.5 | 17 | 0.54839 |
| 17394 | S22_56370081 | 22 | 56370081 | 853290 | G | A | G | 0.93548 | A | 0.06452 | 4 | 0.12903 |
| 17395 | S22_56650749 | 22 | 56650749 | 280668 | C | T | T | 0.83871 | C | 0.16129 | 6 | 0.19355 |
| 17396 | S22_56650759 | 22 | 56650759 | 10 | T | C | C | 0.90323 | T | 0.09677 | 6 | 0.19355 |
| 17397 | S22_56650901 | 22 | 56650901 | 142 | A | G | A | 0.91935 | G | 0.08065 | 5 | 0.16129 |
| 17398 | S22_56650926 | 22 | 56650926 | 25 | G | A | G | 0.85484 | A | 0.14516 | 7 | 0.22581 |
| 17399 | S22_57240682 | 22 | 57240682 | 589756 | C | T | C | 0.91935 | T | 0.08065 | 5 | 0.16129 |
| 17400 | S22_57240703 | 22 | 57240703 | 21 | G | A | G | 0.83871 | A | 0.16129 | 8 | 0.25806 |
| 17401 | S22_57464129 | 22 | 57464129 | 223426 | T | C | T | 0.70968 | C | 0.29032 | 10 | 0.32258 |
| 17402 | S22_57464139 | 22 | 57464139 | 10 | T | C | T | 0.70968 | C | 0.29032 | 10 | 0.32258 |
| 17403 | S22_57464149 | 22 | 57464149 | 10 | T | C | T | 0.70968 | C | 0.29032 | 10 | 0.32258 |
| 17404 | S22_57464165 | 22 | 57464165 | 16 | A | G | A | 0.70968 | G | 0.29032 | 10 | 0.32258 |
| 17405 | S22_57464216 | 22 | 57464216 | 51 | A | G | A | 0.64516 | G | 0.35484 | 12 | 0.3871 |
| 17406 | S22_57464234 | 22 | 57464234 | 18 | T | G | T | 0.70968 | G | 0.29032 | 10 | 0.32258 |
| 17407 | S22_57975420 | 22 | 57975420 | 511186 | G | A | G | 0.67742 | A | 0.32258 | 12 | 0.3871 |
| 17408 | S22_57975584 | 22 | 57975584 | 164 | C | T | T | 0.62903 | C | 0.37097 | 15 | 0.48387 |
| 17409 | S22_58687566 | 22 | 58687566 | 711982 | C | T | C | 0.67742 | T | 0.32258 | 14 | 0.45161 |
| 17410 | S22_58687695 | 22 | 58687695 | 129 | G | A | G | 0.79032 | A | 0.20968 | 11 | 0.35484 |
| 17411 | S22_58687728 | 22 | 58687728 | 33 | G | A | G | 0.77419 | A | 0.22581 | 10 | 0.32258 |
| 17412 | S22_59015505 | 22 | 59015505 | 327777 | G | A | G | 0.85484 | A | 0.14516 | 9 | 0.29032 |
| 17413 | S22_59777583 | 22 | 59777583 | 762078 | T | C | T | 0.64516 | C | 0.35484 | 10 | 0.32258 |
| 17414 | S22_59912436 | 22 | 59912436 | 134853 | G | A | G | 0.90323 | A | 0.09677 | 6 | 0.19355 |
| 17415 | S22_60172721 | 22 | 60172721 | 260285 | T | C | C | 0.83871 | T | 0.16129 | 8 | 0.25806 |
| 17416 | S22_60172723 | 22 | 60172723 | 2 | G | A | A | 0.59677 | G | 0.40323 | 17 | 0.54839 |
| 17417 | S22_60172809 | 22 | 60172809 | 86 | G | A | G | 0.90323 | A | 0.09677 | 6 | 0.19355 |
| 17418 | S22_60172822 | 22 | 60172822 | 13 | G | A | A | 0.5 | G | 0.5 | 19 | 0.6129 |
| 17419 | S23_98775 | 23 | 98775 | 0 | G | A | G | 0.67742 | A | 0.32258 | 18 | 0.58065 |
| 17420 | S23_98930 | 23 | 98930 | 155 | G | A | G | 0.93548 | A | 0.06452 | 4 | 0.12903 |
| 17421 | S23_98987 | 23 | 98987 | 57 | T | G | G | 0.80645 | T | 0.19355 | 8 | 0.25806 |
| 17422 | S23_99002 | 23 | 99002 | 15 | C | T | T | 0.80645 | C | 0.19355 | 10 | 0.32258 |
| 17423 | S23_272899 | 23 | 272899 | 173897 | G | A | G | 0.75806 | A | 0.24194 | 13 | 0.41935 |
| 17424 | S23_311275 | 23 | 311275 | 38376 | A | G | A | 0.85484 | G | 0.14516 | 9 | 0.29032 |
| 17425 | S23_349276 | 23 | 349276 | 38001 | T | C | T | 0.90323 | C | 0.09677 | 6 | 0.19355 |
| 17426 | S23_349303 | 23 | 349303 | 27 | G | A | G | 0.80645 | A | 0.19355 | 12 | 0.3871 |
| 17427 | S23_349315 | 23 | 349315 | 12 | A | G | G | 0.77419 | A | 0.22581 | 12 | 0.3871 |
| 17428 | S23_364672 | 23 | 364672 | 15357 | A | G | A | 0.91935 | G | 0.08065 | 5 | 0.16129 |
| 17429 | S23_2670996 | 23 | 2670996 | 2306324 | T | C | T | 0.77419 | C | 0.22581 | 8 | 0.25806 |
| 17430 | S23_2671089 | 23 | 2671089 | 93 | T | C | T | 0.62903 | C | 0.37097 | 11 | 0.35484 |
| 17431 | S23_2671152 | 23 | 2671152 | 63 | T | C | C | 0.6129 | T | 0.3871 | 12 | 0.3871 |
| 17432 | S23_2815842 | 23 | 2815842 | 144690 | C | T | C | 0.93548 | T | 0.06452 | 4 | 0.12903 |
| 17433 | S23_2877764 | 23 | 2877764 | 61922 | C | G | G | 0.6129 | C | 0.3871 | 14 | 0.45161 |
| 17434 | S23_2877801 | 23 | 2877801 | 37 | G | A | A | 0.59677 | G | 0.40323 | 15 | 0.48387 |
| 17435 | S23_2877838 | 23 | 2877838 | 37 | G | A | A | 0.58065 | G | 0.41935 | 16 | 0.51613 |
| 17436 | S23_2877969 | 23 | 2877969 | 131 | G | A | G | 0.91935 | A | 0.08065 | 5 | 0.16129 |
| 17437 | S23_2877982 | 23 | 2877982 | 13 | C | T | C | 0.91935 | T | 0.08065 | 5 | 0.16129 |
| 17438 | S23_2982696 | 23 | 2982696 | 104714 | A | C | A | 0.56452 | C | 0.43548 | 17 | 0.54839 |
| 17439 | S23_2982718 | 23 | 2982718 | 22 | A | G | A | 0.87097 | G | 0.12903 | 6 | 0.19355 |
| 17440 | S23_3172477 | 23 | 3172477 | 189759 | G | A | G | 0.87097 | A | 0.12903 | 8 | 0.25806 |

| Site number | SNP name | Chromosome | Physical position | Physical distance from the previous SNP | Reference allele | Alternative allele | Major allele | Major allele frequency | Minor allele | Minor allele frequency | Number heterozygous | Heterozygosity |
|---|---|---|---|---|---|---|---|---|---|---|---|---|
| 17441 | S23_3172481 | 23 | 3172481 | 4 | G | A | G | 0.87097 | A | 0.12903 | 8 | 0.25806 |
| 17442 | S23_3172635 | 23 | 3172635 | 154 | C | T | C | 0.90323 | T | 0.09677 | 6 | 0.19355 |
| 17443 | S23_3172689 | 23 | 3172689 | 54 | G | A | G | 0.91935 | A | 0.08065 | 5 | 0.16129 |
| 17444 | S23_3414045 | 23 | 3414045 | 241356 | T | C | T | 0.83871 | C | 0.16129 | 10 | 0.32258 |
| 17445 | S23_4312387 | 23 | 4312387 | 898342 | A | T | T | 0.58065 | A | 0.41935 | 14 | 0.45161 |
| 17446 | S23_4312472 | 23 | 4312472 | 85 | C | G | C | 0.91935 | G | 0.08065 | 3 | 0.09677 |
| 17447 | S23_4877525 | 23 | 4877525 | 565053 | A | G | G | 0.6129 | A | 0.3871 | 14 | 0.45161 |
| 17448 | S23_4877631 | 23 | 4877631 | 106 | T | C | C | 0.62903 | T | 0.37097 | 13 | 0.41935 |
| 17449 | S23_4877670 | 23 | 4877670 | 39 | C | T | C | 0.72581 | T | 0.27419 | 13 | 0.41935 |
| 17450 | S23_4877724 | 23 | 4877724 | 54 | C | T | C | 0.90323 | T | 0.09677 | 4 | 0.12903 |
| 17451 | S23_5393171 | 23 | 5393171 | 515447 | C | G | G | 0.80645 | C | 0.19355 | 10 | 0.32258 |
| 17452 | S23_5393244 | 23 | 5393244 | 73 | T | C | C | 0.79032 | T | 0.20968 | 11 | 0.35484 |
| 17453 | S23_5463356 | 23 | 5463356 | 70112 | C | G | C | 0.95161 | G | 0.04839 | 3 | 0.09677 |
| 17454 | S23_5703429 | 23 | 5703429 | 240073 | T | G | T | 0.87097 | G | 0.12903 | 8 | 0.25806 |
| 17455 | S23_5829968 | 23 | 5829968 | 126539 | T | C | T | 0.95161 | C | 0.04839 | 3 | 0.09677 |
| 17456 | S23_6174361 | 23 | 6174361 | 344393 | A | T | A | 0.87097 | T | 0.12903 | 6 | 0.19355 |
| 17457 | S23_6174391 | 23 | 6174391 | 30 | T | C | T | 0.75806 | C | 0.24194 | 13 | 0.41935 |
| 17458 | S23_6174424 | 23 | 6174424 | 33 | A | G | A | 0.74194 | G | 0.25806 | 14 | 0.45161 |
| 17459 | S23_6174524 | 23 | 6174524 | 100 | T | C | T | 0.75806 | C | 0.24194 | 13 | 0.41935 |
| 17460 | S23_6174542 | 23 | 6174542 | 18 | G | T | G | 0.75806 | T | 0.24194 | 13 | 0.41935 |
| 17461 | S23_6174595 | 23 | 6174595 | 53 | C | T | C | 0.75806 | T | 0.24194 | 13 | 0.41935 |
| 17462 | S23_6380353 | 23 | 6380353 | 205758 | G | A | G | 0.91935 | A | 0.08065 | 5 | 0.16129 |
| 17463 | S23_6720803 | 23 | 6720803 | 340450 | C | T | C | 0.80645 | T | 0.19355 | 10 | 0.32258 |
| 17464 | S23_6775898 | 23 | 6775898 | 55095 | C | T | C | 0.90323 | T | 0.09677 | 6 | 0.19355 |
| 17465 | S23_6775944 | 23 | 6775944 | 46 | G | A | G | 0.87097 | A | 0.12903 | 8 | 0.25806 |
| 17466 | S23_6775954 | 23 | 6775954 | 10 | A | G | A | 0.90323 | G | 0.09677 | 6 | 0.19355 |
| 17467 | S23_6775985 | 23 | 6775985 | 31 | A | G | A | 0.93548 | G | 0.06452 | 4 | 0.12903 |
| 17468 | S23_6790444 | 23 | 6790444 | 14459 | G | A | G | 0.85484 | A | 0.14516 | 9 | 0.29032 |
| 17469 | S23_6836700 | 23 | 6836700 | 46256 | G | A | G | 0.82258 | A | 0.17742 | 9 | 0.29032 |
| 17470 | S23_6836823 | 23 | 6836823 | 123 | T | G | T | 0.87097 | G | 0.12903 | 8 | 0.25806 |
| 17471 | S23_7653052 | 23 | 7653052 | 816229 | G | T | G | 0.79032 | T | 0.20968 | 11 | 0.35484 |
| 17472 | S23_7733733 | 23 | 7733733 | 80681 | G | A | G | 0.85484 | A | 0.14516 | 7 | 0.22581 |
| 17473 | S23_7796103 | 23 | 7796103 | 62370 | G | A | G | 0.66129 | A | 0.33871 | 15 | 0.48387 |
| 17474 | S23_7796221 | 23 | 7796221 | 118 | A | G | A | 0.87097 | G | 0.12903 | 8 | 0.25806 |
| 17475 | S23_7796276 | 23 | 7796276 | 55 | A | T | A | 0.95161 | T | 0.04839 | 3 | 0.09677 |
| 17476 | S23_8129054 | 23 | 8129054 | 332778 | C | T | C | 0.91935 | T | 0.08065 | 5 | 0.16129 |
| 17477 | S23_8333592 | 23 | 8333592 | 204538 | C | T | C | 0.79032 | T | 0.20968 | 11 | 0.35484 |
| 17478 | S23_8804497 | 23 | 8804497 | 470905 | A | G | A | 0.5 | G | 0.5 | 11 | 0.35484 |
| 17479 | S23_9155566 | 23 | 9155566 | 351069 | A | G | A | 0.58065 | G | 0.41935 | 16 | 0.51613 |
| 17480 | S23_9155760 | 23 | 9155760 | 194 | C | T | C | 0.91935 | T | 0.08065 | 5 | 0.16129 |
| 17481 | S23_9155761 | 23 | 9155761 | 1 | G | A | G | 0.8871 | A | 0.1129 | 7 | 0.22581 |
| 17482 | S23_9193720 | 23 | 9193720 | 37959 | T | C | T | 0.8871 | C | 0.1129 | 7 | 0.22581 |
| 17483 | S23_9193741 | 23 | 9193741 | 21 | G | A | G | 0.93548 | A | 0.06452 | 4 | 0.12903 |
| 17484 | S23_9241167 | 23 | 9241167 | 47426 | A | G | A | 0.85484 | G | 0.14516 | 9 | 0.29032 |
| 17485 | S23_9290495 | 23 | 9290495 | 49328 | T | A | A | 0.5 | T | 0.5 | 17 | 0.54839 |
| 17486 | S23_9290611 | 23 | 9290611 | 116 | T | C | C | 0.54839 | T | 0.45161 | 16 | 0.51613 |
| 17487 | S23_9290690 | 23 | 9290690 | 79 | G | A | G | 0.95161 | A | 0.04839 | 3 | 0.09677 |
| 17488 | S23_9642972 | 23 | 9642972 | 352282 | A | G | G | 0.66129 | A | 0.33871 | 15 | 0.48387 |
| 17489 | S23_9643007 | 23 | 9643007 | 35 | A | G | A | 0.83871 | G | 0.16129 | 8 | 0.25806 |
| 17490 | S23_9647528 | 23 | 9647528 | 4521 | A | G | A | 0.90323 | G | 0.09677 | 6 | 0.19355 |
| 17491 | S23_9956981 | 23 | 9956981 | 309453 | A | G | A | 0.83871 | G | 0.16129 | 8 | 0.25806 |
| 17492 | S23_9957030 | 23 | 9957030 | 49 | C | A | A | 0.53226 | C | 0.46774 | 17 | 0.54839 |
| 17493 | S23_10151218 | 23 | 10151218 | 194188 | C | T | C | 0.75806 | T | 0.24194 | 15 | 0.48387 |
| 17494 | S23_10151351 | 23 | 10151351 | 133 | C | G | C | 0.85484 | G | 0.14516 | 9 | 0.29032 |

| Site number | SNP name | Chromosome | Physical position | Physical distance from the previous SNP | Reference allele | Alternative allele | Major allele | Major allele frequency | Minor allele | Minor allele frequency | Number heterozygous | Heterozygosity |
|---|---|---|---|---|---|---|---|---|---|---|---|---|
| 17495 | S23_10151358 | 23 | 10151358 | 7 | C | G | G | 0.90323 | C | 0.09677 | 6 | 0.19355 |
| 17496 | S23_10208138 | 23 | 10208138 | 56780 | C | T | C | 0.95161 | T | 0.04839 | 3 | 0.09677 |
| 17497 | S23_10212239 | 23 | 10212239 | 4101 | C | A | C | 0.72581 | A | 0.27419 | 13 | 0.41935 |
| 17498 | S23_10637706 | 23 | 10637706 | 425467 | T | C | T | 0.70968 | C | 0.29032 | 14 | 0.45161 |
| 17499 | S23_10871844 | 23 | 10871844 | 234138 | C | T | C | 0.75806 | T | 0.24194 | 11 | 0.35484 |
| 17500 | S23_11209447 | 23 | 11209447 | 337603 | T | G | T | 0.82258 | G | 0.17742 | 7 | 0.22581 |
| 17501 | S23_11209485 | 23 | 11209485 | 38 | A | G | A | 0.62903 | G | 0.37097 | 9 | 0.29032 |
| 17502 | S23_11383207 | 23 | 11383207 | 173722 | G | A | G | 0.90323 | A | 0.09677 | 6 | 0.19355 |
| 17503 | S23_12140046 | 23 | 12140046 | 756839 | C | G | C | 0.66129 | G | 0.33871 | 15 | 0.48387 |
| 17504 | S23_12155436 | 23 | 12155436 | 15390 | T | C | C | 0.91935 | T | 0.08065 | 5 | 0.16129 |
| 17505 | S23_12155481 | 23 | 12155481 | 45 | A | G | A | 0.95161 | G | 0.04839 | 3 | 0.09677 |
| 17506 | S23_12356475 | 23 | 12356475 | 200994 | G | T | G | 0.79032 | T | 0.20968 | 9 | 0.29032 |
| 17507 | S23_12485818 | 23 | 12485818 | 129343 | G | A | G | 0.80645 | A | 0.19355 | 8 | 0.25806 |
| 17508 | S23_12485866 | 23 | 12485866 | 48 | C | G | G | 0.67742 | C | 0.32258 | 14 | 0.45161 |
| 17509 | S23_12485917 | 23 | 12485917 | 51 | A | G | G | 0.67742 | A | 0.32258 | 14 | 0.45161 |
| 17510 | S23_12524875 | 23 | 12524875 | 38958 | G | C | G | 0.82258 | C | 0.17742 | 9 | 0.29032 |
| 17511 | S23_12525103 | 23 | 12525103 | 228 | C | T | C | 0.79032 | T | 0.20968 | 9 | 0.29032 |
| 17512 | S23_12525125 | 23 | 12525125 | 22 | A | C | A | 0.79032 | C | 0.20968 | 9 | 0.29032 |
| 17513 | S23_12608113 | 23 | 12608113 | 82988 | C | T | C | 0.54839 | T | 0.45161 | 16 | 0.51613 |
| 17514 | S23_12763112 | 23 | 12763112 | 154999 | G | A | G | 0.75806 | A | 0.24194 | 9 | 0.29032 |
| 17515 | S23_12763118 | 23 | 12763118 | 6 | G | A | G | 0.83871 | A | 0.16129 | 6 | 0.19355 |
| 17516 | S23_12763216 | 23 | 12763216 | 98 | G | A | G | 0.95161 | A | 0.04839 | 3 | 0.09677 |
| 17517 | S23_12763320 | 23 | 12763320 | 104 | A | G | G | 0.62903 | A | 0.37097 | 17 | 0.54839 |
| 17518 | S23_13026430 | 23 | 13026430 | 263110 | A | G | A | 0.93548 | G | 0.06452 | 4 | 0.12903 |
| 17519 | S23_13592884 | 23 | 13592884 | 566454 | G | A | G | 0.87097 | A | 0.12903 | 8 | 0.25806 |
| 17520 | S23_13613717 | 23 | 13613717 | 20833 | G | A | G | 0.90323 | A | 0.09677 | 6 | 0.19355 |
| 17521 | S23_13613797 | 23 | 13613797 | 80 | T | A | T | 0.64516 | A | 0.35484 | 16 | 0.51613 |
| 17522 | S23_13613824 | 23 | 13613824 | 27 | A | G | A | 0.64516 | G | 0.35484 | 16 | 0.51613 |
| 17523 | S23_13616324 | 23 | 13616324 | 2500 | G | A | A | 0.62903 | G | 0.37097 | 15 | 0.48387 |
| 17524 | S23_13616427 | 23 | 13616427 | 103 | G | A | G | 0.58065 | A | 0.41935 | 12 | 0.3871 |
| 17525 | S23_13616437 | 23 | 13616437 | 10 | T | C | C | 0.64516 | T | 0.35484 | 16 | 0.51613 |
| 17526 | S23_13641396 | 23 | 13641396 | 24959 | C | T | C | 0.93548 | T | 0.06452 | 4 | 0.12903 |
| 17527 | S23_13641512 | 23 | 13641512 | 116 | C | T | C | 0.93548 | T | 0.06452 | 4 | 0.12903 |
| 17528 | S23_14069498 | 23 | 14069498 | 427986 | A | G | A | 0.95161 | G | 0.04839 | 3 | 0.09677 |
| 17529 | S23_14069535 | 23 | 14069535 | 37 | A | G | A | 0.87097 | G | 0.12903 | 6 | 0.19355 |
| 17530 | S23_14420977 | 23 | 14420977 | 351442 | G | T | G | 0.75806 | T | 0.24194 | 15 | 0.48387 |
| 17531 | S23_14568921 | 23 | 14568921 | 147944 | A | G | G | 0.66129 | A | 0.33871 | 17 | 0.54839 |
| 17532 | S23_14568966 | 23 | 14568966 | 45 | A | G | A | 0.93548 | G | 0.06452 | 4 | 0.12903 |
| 17533 | S23_14569091 | 23 | 14569091 | 125 | G | A | G | 0.80645 | A | 0.19355 | 10 | 0.32258 |
| 17534 | S23_14569129 | 23 | 14569129 | 38 | G | A | G | 0.91935 | A | 0.08065 | 5 | 0.16129 |
| 17535 | S23_15107305 | 23 | 15107305 | 538176 | G | A | G | 0.83871 | A | 0.16129 | 8 | 0.25806 |
| 17536 | S23_15291324 | 23 | 15291324 | 184019 | G | A | G | 0.85484 | A | 0.14516 | 7 | 0.22581 |
| 17537 | S23_15447486 | 23 | 15447486 | 156162 | C | T | C | 0.74194 | T | 0.25806 | 8 | 0.25806 |
| 17538 | S23_15447602 | 23 | 15447602 | 116 | C | T | C | 0.72581 | T | 0.27419 | 11 | 0.35484 |
| 17539 | S23_15540393 | 23 | 15540393 | 92791 | C | T | C | 0.90323 | T | 0.09677 | 6 | 0.19355 |
| 17540 | S23_15679306 | 23 | 15679306 | 138913 | A | C | C | 0.79032 | A | 0.20968 | 9 | 0.29032 |
| 17541 | S23_15679341 | 23 | 15679341 | 35 | C | T | C | 0.59677 | T | 0.40323 | 15 | 0.48387 |
| 17542 | S23_15679377 | 23 | 15679377 | 36 | C | A | C | 0.59677 | A | 0.40323 | 15 | 0.48387 |
| 17543 | S23_15679396 | 23 | 15679396 | 19 | T | C | T | 0.59677 | C | 0.40323 | 15 | 0.48387 |
| 17544 | S23_15887050 | 23 | 15887050 | 207654 | T | C | T | 0.87097 | C | 0.12903 | 8 | 0.25806 |
| 17545 | S23_15893022 | 23 | 15893022 | 5972 | G | A | G | 0.69355 | A | 0.30645 | 15 | 0.48387 |
| 17546 | S23_15899211 | 23 | 15899211 | 6189 | C | T | T | 0.77419 | C | 0.22581 | 14 | 0.45161 |
| 17547 | S23_15899247 | 23 | 15899247 | 36 | T | C | T | 0.67742 | C | 0.32258 | 18 | 0.58065 |
| 17548 | S23_15899289 | 23 | 15899289 | 42 | C | T | C | 0.90323 | T | 0.09677 | 6 | 0.19355 |

| Site number | SNP name | Chromosome | Physical position | Physical distance from the previous SNP | Reference allele | Alternative allele | Major allele | Major allele frequency | Minor allele | Minor allele frequency | Number heterozygous | Heterozygosity |
|---|---|---|---|---|---|---|---|---|---|---|---|---|
| 17549 | S23_15899320 | 23 | 15899320 | 31 | G | A | G | 0.67742 | A | 0.32258 | 18 | 0.58065 |
| 17550 | S23_15899420 | 23 | 15899420 | 100 | G | T | G | 0.87097 | T | 0.12903 | 8 | 0.25806 |
| 17551 | S23_15994621 | 23 | 15994621 | 95201 | A | C | A | 0.93548 | C | 0.06452 | 4 | 0.12903 |
| 17552 | S23_16226032 | 23 | 16226032 | 231411 | C | T | C | 0.8871 | T | 0.1129 | 5 | 0.16129 |
| 17553 | S23_16226050 | 23 | 16226050 | 18 | A | G | G | 0.66129 | A | 0.33871 | 15 | 0.48387 |
| 17554 | S23_16226197 | 23 | 16226197 | 147 | A | G | A | 0.8871 | G | 0.1129 | 5 | 0.16129 |
| 17555 | S23_16260500 | 23 | 16260500 | 34303 | G | A | G | 0.95161 | A | 0.04839 | 3 | 0.09677 |
| 17556 | S23_16260552 | 23 | 16260552 | 52 | C | T | T | 0.93548 | C | 0.06452 | 4 | 0.12903 |
| 17557 | S23_16343449 | 23 | 16343449 | 82897 | G | A | G | 0.85484 | A | 0.14516 | 9 | 0.29032 |
| 17558 | S23_16405450 | 23 | 16405450 | 62001 | G | C | G | 0.59677 | C | 0.40323 | 13 | 0.41935 |
| 17559 | S23_16405525 | 23 | 16405525 | 75 | A | G | A | 0.59677 | G | 0.40323 | 13 | 0.41935 |
| 17560 | S23_16442388 | 23 | 16442388 | 36863 | G | A | G | 0.93548 | A | 0.06452 | 4 | 0.12903 |
| 17561 | S23_16518501 | 23 | 16518501 | 76113 | A | G | A | 0.5 | G | 0.5 | 13 | 0.41935 |
| 17562 | S23_16518573 | 23 | 16518573 | 72 | G | A | G | 0.8871 | A | 0.1129 | 7 | 0.22581 |
| 17563 | S23_16665066 | 23 | 16665066 | 146493 | C | T | C | 0.62903 | T | 0.37097 | 19 | 0.6129 |
| 17564 | S23_16665081 | 23 | 16665081 | 15 | C | T | C | 0.62903 | T | 0.37097 | 19 | 0.6129 |
| 17565 | S23_16665136 | 23 | 16665136 | 55 | G | T | G | 0.62903 | T | 0.37097 | 19 | 0.6129 |
| 17566 | S23_16665181 | 23 | 16665181 | 45 | C | A | C | 0.91935 | A | 0.08065 | 5 | 0.16129 |
| 17567 | S23_16665312 | 23 | 16665312 | 131 | G | T | T | 0.62903 | G | 0.37097 | 17 | 0.54839 |
| 17568 | S23_16765710 | 23 | 16765710 | 100398 | G | T | G | 0.95161 | T | 0.04839 | 3 | 0.09677 |
| 17569 | S23_16836065 | 23 | 16836065 | 70355 | C | T | C | 0.80645 | T | 0.19355 | 10 | 0.32258 |
| 17570 | S23_16836079 | 23 | 16836079 | 14 | T | C | T | 0.80645 | C | 0.19355 | 10 | 0.32258 |
| 17571 | S23_16836105 | 23 | 16836105 | 26 | T | C | T | 0.72581 | C | 0.27419 | 13 | 0.41935 |
| 17572 | S23_16836165 | 23 | 16836165 | 60 | C | T | C | 0.80645 | T | 0.19355 | 10 | 0.32258 |
| 17573 | S23_16836184 | 23 | 16836184 | 19 | T | C | C | 0.75806 | T | 0.24194 | 9 | 0.29032 |
| 17574 | S23_16836250 | 23 | 16836250 | 66 | G | A | G | 0.80645 | A | 0.19355 | 10 | 0.32258 |
| 17575 | S23_17457171 | 23 | 17457171 | 620921 | T | G | T | 0.79032 | G | 0.20968 | 9 | 0.29032 |
| 17576 | S23_17569110 | 23 | 17569110 | 111939 | T | C | T | 0.80645 | C | 0.19355 | 12 | 0.3871 |
| 17577 | S23_17569179 | 23 | 17569179 | 69 | T | C | T | 0.80645 | C | 0.19355 | 12 | 0.3871 |
| 17578 | S23_17569202 | 23 | 17569202 | 23 | G | A | G | 0.79032 | A | 0.20968 | 13 | 0.41935 |
| 17579 | S23_17569257 | 23 | 17569257 | 55 | C | T | C | 0.77419 | T | 0.22581 | 12 | 0.3871 |
| 17580 | S23_17569313 | 23 | 17569313 | 56 | T | G | T | 0.91935 | G | 0.08065 | 5 | 0.16129 |
| 17581 | S23_18578266 | 23 | 18578266 | 1008953 | G | A | G | 0.77419 | A | 0.22581 | 12 | 0.3871 |
| 17582 | S23_18578365 | 23 | 18578365 | 99 | C | T | T | 0.85484 | C | 0.14516 | 7 | 0.22581 |
| 17583 | S23_18672102 | 23 | 18672102 | 93737 | G | A | G | 0.8871 | A | 0.1129 | 7 | 0.22581 |
| 17584 | S23_18672162 | 23 | 18672162 | 60 | C | G | C | 0.87097 | G | 0.12903 | 8 | 0.25806 |
| 17585 | S23_19587730 | 23 | 19587730 | 915568 | C | T | C | 0.93548 | T | 0.06452 | 4 | 0.12903 |
| 17586 | S23_19650452 | 23 | 19650452 | 62722 | C | T | T | 0.8871 | C | 0.1129 | 7 | 0.22581 |
| 17587 | S23_19650473 | 23 | 19650473 | 21 | C | G | C | 0.74194 | G | 0.25806 | 8 | 0.25806 |
| 17588 | S23_19650496 | 23 | 19650496 | 23 | C | T | C | 0.90323 | T | 0.09677 | 6 | 0.19355 |
| 17589 | S23_19650548 | 23 | 19650548 | 52 | A | G | A | 0.77419 | G | 0.22581 | 8 | 0.25806 |
| 17590 | S23_19992963 | 23 | 19992963 | 342415 | C | T | C | 0.64516 | T | 0.35484 | 16 | 0.51613 |
| 17591 | S23_19992966 | 23 | 19992966 | 3 | G | A | A | 0.56452 | G | 0.43548 | 19 | 0.6129 |
| 17592 | S23_19993009 | 23 | 19993009 | 43 | G | A | G | 0.93548 | A | 0.06452 | 4 | 0.12903 |
| 17593 | S23_19993110 | 23 | 19993110 | 101 | G | A | G | 0.87097 | A | 0.12903 | 6 | 0.19355 |
| 17594 | S23_19993121 | 23 | 19993121 | 11 | A | G | A | 0.56452 | G | 0.43548 | 19 | 0.6129 |
| 17595 | S23_19993179 | 23 | 19993179 | 58 | C | T | C | 0.56452 | T | 0.43548 | 19 | 0.6129 |
| 17596 | S23_19993181 | 23 | 19993181 | 2 | T | G | G | 0.69355 | T | 0.30645 | 15 | 0.48387 |
| 17597 | S23_20505066 | 23 | 20505066 | 511885 | G | C | G | 0.74194 | C | 0.25806 | 14 | 0.45161 |
| 17598 | S23_20505183 | 23 | 20505183 | 117 | C | T | C | 0.93548 | T | 0.06452 | 4 | 0.12903 |
| 17599 | S23_20505188 | 23 | 20505188 | 5 | C | T | C | 0.8871 | T | 0.1129 | 7 | 0.22581 |
| 17600 | S23_20505199 | 23 | 20505199 | 11 | T | G | T | 0.74194 | G | 0.25806 | 14 | 0.45161 |
| 17601 | S23_21029408 | 23 | 21029408 | 524209 | G | A | G | 0.91935 | A | 0.08065 | 5 | 0.16129 |
| 17602 | S23_21029440 | 23 | 21029440 | 32 | C | T | C | 0.91935 | T | 0.08065 | 5 | 0.16129 |

| Site number | SNP name | Chromosome | Physical position | Physical distance from the previous SNP | Reference allele | Alternative allele | Major allele | Major allele frequency | Minor allele | Minor allele frequency | Number heterozygous | Heterozygosity |
|---|---|---|---|---|---|---|---|---|---|---|---|---|
| 17603 | S23_21029599 | 23 | 21029599 | 159 | G | A | G | 0.90323 | A | 0.09677 | 4 | 0.12903 |
| 17604 | S23_21178094 | 23 | 21178094 | 148495 | A | G | A | 0.87097 | G | 0.12903 | 6 | 0.19355 |
| 17605 | S23_21178104 | 23 | 21178104 | 10 | G | C | G | 0.85484 | C | 0.14516 | 7 | 0.22581 |
| 17606 | S23_21178122 | 23 | 21178122 | 18 | T | G | T | 0.75806 | G | 0.24194 | 13 | 0.41935 |
| 17607 | S23_21178137 | 23 | 21178137 | 15 | G | A | G | 0.58065 | A | 0.41935 | 16 | 0.51613 |
| 17608 | S23_21178185 | 23 | 21178185 | 48 | A | G | A | 0.87097 | G | 0.12903 | 6 | 0.19355 |
| 17609 | S23_21178190 | 23 | 21178190 | 5 | T | C | T | 0.91935 | C | 0.08065 | 5 | 0.16129 |
| 17610 | S23_21178220 | 23 | 21178220 | 30 | A | C | A | 0.87097 | C | 0.12903 | 6 | 0.19355 |
| 17611 | S23_21207041 | 23 | 21207041 | 28821 | G | A | G | 0.93548 | A | 0.06452 | 4 | 0.12903 |
| 17612 | S23_21207131 | 23 | 21207131 | 90 | A | T | A | 0.91935 | T | 0.08065 | 5 | 0.16129 |
| 17613 | S23_21207168 | 23 | 21207168 | 37 | G | A | A | 0.51613 | G | 0.48387 | 16 | 0.51613 |
| 17614 | S23_21552788 | 23 | 21552788 | 345620 | G | A | G | 0.93548 | A | 0.06452 | 4 | 0.12903 |
| 17615 | S23_21767381 | 23 | 21767381 | 214593 | A | G | A | 0.74194 | G | 0.25806 | 14 | 0.45161 |
| 17616 | S23_21767433 | 23 | 21767433 | 52 | T | A | T | 0.87097 | A | 0.12903 | 6 | 0.19355 |
| 17617 | S23_21767460 | 23 | 21767460 | 27 | C | T | C | 0.74194 | T | 0.25806 | 14 | 0.45161 |
| 17618 | S23_21795934 | 23 | 21795934 | 28474 | C | T | C | 0.75806 | T | 0.24194 | 13 | 0.41935 |
| 17619 | S23_22016181 | 23 | 22016181 | 220247 | A | T | A | 0.93548 | T | 0.06452 | 4 | 0.12903 |
| 17620 | S23_22016186 | 23 | 22016186 | 5 | T | G | T | 0.93548 | G | 0.06452 | 4 | 0.12903 |
| 17621 | S23_22016192 | 23 | 22016192 | 6 | G | T | G | 0.93548 | T | 0.06452 | 4 | 0.12903 |
| 17622 | S23_22016211 | 23 | 22016211 | 19 | C | G | C | 0.83871 | G | 0.16129 | 8 | 0.25806 |
| 17623 | S23_22016262 | 23 | 22016262 | 51 | A | G | G | 0.74194 | A | 0.25806 | 12 | 0.3871 |
| 17624 | S23_22191326 | 23 | 22191326 | 175064 | G | A | G | 0.90323 | A | 0.09677 | 6 | 0.19355 |
| 17625 | S23_22191460 | 23 | 22191460 | 134 | G | A | G | 0.90323 | A | 0.09677 | 6 | 0.19355 |
| 17626 | S23_22361087 | 23 | 22361087 | 169627 | T | C | T | 0.77419 | C | 0.22581 | 12 | 0.3871 |
| 17627 | S23_22368766 | 23 | 22368766 | 7679 | A | C | A | 0.91935 | C | 0.08065 | 5 | 0.16129 |
| 17628 | S23_22642308 | 23 | 22642308 | 273542 | T | C | T | 0.93548 | C | 0.06452 | 4 | 0.12903 |
| 17629 | S23_22642340 | 23 | 22642340 | 32 | G | T | G | 0.74194 | T | 0.25806 | 14 | 0.45161 |
| 17630 | S23_23388365 | 23 | 23388365 | 746025 | C | T | C | 0.93548 | T | 0.06452 | 2 | 0.06452 |
| 17631 | S23_23388376 | 23 | 23388376 | 11 | T | C | T | 0.77419 | C | 0.22581 | 12 | 0.3871 |
| 17632 | S23_23388378 | 23 | 23388378 | 2 | A | G | A | 0.5 | G | 0.5 | 19 | 0.6129 |
| 17633 | S23_23388445 | 23 | 23388445 | 67 | A | G | A | 0.93548 | G | 0.06452 | 2 | 0.06452 |
| 17634 | S23_23977366 | 23 | 23977366 | 588921 | T | C | C | 0.64516 | T | 0.35484 | 16 | 0.51613 |
| 17635 | S23_23977411 | 23 | 23977411 | 45 | C | T | T | 0.77419 | C | 0.22581 | 14 | 0.45161 |
| 17636 | S23_23980381 | 23 | 23980381 | 2970 | G | A | G | 0.95161 | A | 0.04839 | 3 | 0.09677 |
| 17637 | S23_23980416 | 23 | 23980416 | 35 | C | G | C | 0.95161 | G | 0.04839 | 3 | 0.09677 |
| 17638 | S23_23980514 | 23 | 23980514 | 98 | A | G | A | 0.87097 | G | 0.12903 | 8 | 0.25806 |
| 17639 | S23_23980590 | 23 | 23980590 | 76 | A | G | A | 0.91935 | G | 0.08065 | 5 | 0.16129 |
| 17640 | S23_23982916 | 23 | 23982916 | 2326 | C | T | C | 0.91935 | T | 0.08065 | 5 | 0.16129 |
| 17641 | S23_23982923 | 23 | 23982923 | 7 | T | C | T | 0.93548 | C | 0.06452 | 4 | 0.12903 |
| 17642 | S23_24444351 | 23 | 24444351 | 461428 | G | C | G | 0.93548 | C | 0.06452 | 4 | 0.12903 |
| 17643 | S23_24444376 | 23 | 24444376 | 25 | G | A | G | 0.95161 | A | 0.04839 | 3 | 0.09677 |
| 17644 | S23_24444431 | 23 | 24444431 | 55 | G | C | G | 0.93548 | C | 0.06452 | 4 | 0.12903 |
| 17645 | S23_24444437 | 23 | 24444437 | 6 | C | T | C | 0.95161 | T | 0.04839 | 3 | 0.09677 |
| 17646 | S23_24444482 | 23 | 24444482 | 45 | A | G | A | 0.93548 | G | 0.06452 | 4 | 0.12903 |
| 17647 | S23_24690311 | 23 | 24690311 | 245829 | C | T | C | 0.93548 | T | 0.06452 | 4 | 0.12903 |
| 17648 | S23_24705279 | 23 | 24705279 | 14968 | A | C | A | 0.72581 | C | 0.27419 | 13 | 0.41935 |
| 17649 | S23_24705378 | 23 | 24705378 | 99 | G | A | G | 0.58065 | A | 0.41935 | 20 | 0.64516 |
| 17650 | S23_24705408 | 23 | 24705408 | 30 | C | T | C | 0.91935 | T | 0.08065 | 5 | 0.16129 |
| 17651 | S23_24826166 | 23 | 24826166 | 120758 | G | A | G | 0.91935 | A | 0.08065 | 5 | 0.16129 |
| 17652 | S23_24826216 | 23 | 24826216 | 50 | G | A | G | 0.95161 | A | 0.04839 | 3 | 0.09677 |
| 17653 | S23_24826271 | 23 | 24826271 | 55 | G | A | G | 0.62903 | A | 0.37097 | 19 | 0.6129 |
| 17654 | S23_24842486 | 23 | 24842486 | 16215 | A | G | G | 0.87097 | A | 0.12903 | 8 | 0.25806 |
| 17655 | S23_24976726 | 23 | 24976726 | 134240 | T | C | T | 0.64516 | C | 0.35484 | 16 | 0.51613 |
| 17656 | S23_25122603 | 23 | 25122603 | 145877 | T | A | T | 0.59677 | A | 0.40323 | 13 | 0.41935 |

| Site number | SNP name | Chromosome | Physical position | Physical distance from the previous SNP | Reference allele | Alternative allele | Major allele | Major allele frequency | Minor allele | Minor allele frequency | Number heterozygous | Heterozygosity |
|---|---|---|---|---|---|---|---|---|---|---|---|---|
| 17657 | S23_25122711 | 23 | 25122711 | 108 | C | T | C | 0.59677 | T | 0.40323 | 13 | 0.41935 |
| 17658 | S23_25833186 | 23 | 25833186 | 710475 | C | T | C | 0.80645 | T | 0.19355 | 10 | 0.32258 |
| 17659 | S23_25842822 | 23 | 25842822 | 9636 | T | C | C | 0.62903 | T | 0.37097 | 13 | 0.41935 |
| 17660 | S23_25842877 | 23 | 25842877 | 55 | A | G | A | 0.62903 | G | 0.37097 | 13 | 0.41935 |
| 17661 | S23_25842991 | 23 | 25842991 | 114 | G | A | A | 0.62903 | G | 0.37097 | 13 | 0.41935 |
| 17662 | S23_26251753 | 23 | 26251753 | 408762 | C | T | C | 0.87097 | T | 0.12903 | 6 | 0.19355 |
| 17663 | S23_27060682 | 23 | 27060682 | 808929 | T | A | T | 0.91935 | A | 0.08065 | 5 | 0.16129 |
| 17664 | S23_27060691 | 23 | 27060691 | 9 | T | C | T | 0.8871 | C | 0.1129 | 5 | 0.16129 |
| 17665 | S23_27214533 | 23 | 27214533 | 153842 | A | G | A | 0.67742 | G | 0.32258 | 14 | 0.45161 |
| 17666 | S23_27214557 | 23 | 27214557 | 24 | A | G | A | 0.6129 | G | 0.3871 | 14 | 0.45161 |
| 17667 | S23_27214569 | 23 | 27214569 | 12 | T | C | T | 0.56452 | C | 0.43548 | 17 | 0.54839 |
| 17668 | S23_27386756 | 23 | 27386756 | 172187 | G | A | G | 0.87097 | A | 0.12903 | 4 | 0.12903 |
| 17669 | S23_27386769 | 23 | 27386769 | 13 | C | T | C | 0.87097 | T | 0.12903 | 4 | 0.12903 |
| 17670 | S23_27386802 | 23 | 27386802 | 33 | T | G | T | 0.8871 | G | 0.1129 | 5 | 0.16129 |
| 17671 | S23_27386951 | 23 | 27386951 | 149 | C | T | C | 0.80645 | T | 0.19355 | 10 | 0.32258 |
| 17672 | S23_27785011 | 23 | 27785011 | 398060 | C | T | T | 0.59677 | C | 0.40323 | 15 | 0.48387 |
| 17673 | S23_29071971 | 23 | 29071971 | 1286960 | A | G | A | 0.93548 | G | 0.06452 | 4 | 0.12903 |
| 17674 | S23_29532938 | 23 | 29532938 | 460967 | T | C | C | 0.77419 | T | 0.22581 | 10 | 0.32258 |
| 17675 | S23_29533115 | 23 | 29533115 | 177 | C | T | C | 0.93548 | T | 0.06452 | 4 | 0.12903 |
| 17676 | S23_29762832 | 23 | 29762832 | 229717 | T | C | C | 0.8871 | T | 0.1129 | 5 | 0.16129 |
| 17677 | S23_29870464 | 23 | 29870464 | 107632 | T | C | T | 0.95161 | C | 0.04839 | 3 | 0.09677 |
| 17678 | S23_29873865 | 23 | 29873865 | 3401 | C | T | C | 0.95161 | T | 0.04839 | 3 | 0.09677 |
| 17679 | S23_29965022 | 23 | 29965022 | 91157 | A | C | A | 0.87097 | C | 0.12903 | 8 | 0.25806 |
| 17680 | S23_30007031 | 23 | 30007031 | 42009 | C | T | C | 0.87097 | T | 0.12903 | 6 | 0.19355 |
| 17681 | S23_30116989 | 23 | 30116989 | 109958 | T | C | T | 0.59677 | C | 0.40323 | 15 | 0.48387 |
| 17682 | S23_30117010 | 23 | 30117010 | 21 | T | G | T | 0.91935 | G | 0.08065 | 5 | 0.16129 |
| 17683 | S23_30117118 | 23 | 30117118 | 108 | C | A | C | 0.95161 | A | 0.04839 | 3 | 0.09677 |
| 17684 | S23_30117129 | 23 | 30117129 | 11 | G | A | G | 0.69355 | A | 0.30645 | 15 | 0.48387 |
| 17685 | S23_30645364 | 23 | 30645364 | 528235 | G | A | G | 0.93548 | A | 0.06452 | 4 | 0.12903 |
| 17686 | S23_30783826 | 23 | 30783826 | 138462 | T | A | T | 0.85484 | A | 0.14516 | 7 | 0.22581 |
| 17687 | S23_30783950 | 23 | 30783950 | 124 | G | C | G | 0.91935 | C | 0.08065 | 5 | 0.16129 |
| 17688 | S23_30926759 | 23 | 30926759 | 142809 | C | G | C | 0.90323 | G | 0.09677 | 6 | 0.19355 |
| 17689 | S23_30926851 | 23 | 30926851 | 92 | G | A | G | 0.64516 | A | 0.35484 | 16 | 0.51613 |
| 17690 | S23_31433161 | 23 | 31433161 | 506310 | C | T | C | 0.90323 | T | 0.09677 | 6 | 0.19355 |
| 17691 | S23_31453186 | 23 | 31453186 | 20025 | G | A | G | 0.51613 | A | 0.48387 | 16 | 0.51613 |
| 17692 | S23_31453346 | 23 | 31453346 | 160 | T | C | C | 0.51613 | T | 0.48387 | 14 | 0.45161 |
| 17693 | S23_31453350 | 23 | 31453350 | 4 | A | G | A | 0.75806 | G | 0.24194 | 11 | 0.35484 |
| 17694 | S23_31605867 | 23 | 31605867 | 152517 | T | C | T | 0.87097 | C | 0.12903 | 6 | 0.19355 |
| 17695 | S23_31611858 | 23 | 31611858 | 5991 | G | C | C | 0.59677 | G | 0.40323 | 15 | 0.48387 |
| 17696 | S23_31672206 | 23 | 31672206 | 60348 | T | C | T | 0.80645 | C | 0.19355 | 8 | 0.25806 |
| 17697 | S23_31806557 | 23 | 31806557 | 134351 | C | A | C | 0.90323 | A | 0.09677 | 6 | 0.19355 |
| 17698 | S23_31806571 | 23 | 31806571 | 14 | C | A | A | 0.58065 | C | 0.41935 | 12 | 0.3871 |
| 17699 | S23_32083400 | 23 | 32083400 | 276829 | C | T | C | 0.95161 | T | 0.04839 | 3 | 0.09677 |
| 17700 | S23_32083411 | 23 | 32083411 | 11 | T | C | C | 0.69355 | T | 0.30645 | 13 | 0.41935 |
| 17701 | S23_32083422 | 23 | 32083422 | 11 | G | A | A | 0.69355 | G | 0.30645 | 13 | 0.41935 |
| 17702 | S23_32083459 | 23 | 32083459 | 37 | C | T | T | 0.69355 | C | 0.30645 | 13 | 0.41935 |
| 17703 | S23_32083555 | 23 | 32083555 | 96 | C | T | T | 0.69355 | C | 0.30645 | 13 | 0.41935 |
| 17704 | S23_32374588 | 23 | 32374588 | 291033 | C | A | C | 0.87097 | A | 0.12903 | 6 | 0.19355 |
| 17705 | S23_32513607 | 23 | 32513607 | 139019 | T | C | C | 0.67742 | T | 0.32258 | 12 | 0.3871 |
| 17706 | S23_32513692 | 23 | 32513692 | 85 | T | G | G | 0.67742 | T | 0.32258 | 12 | 0.3871 |
| 17707 | S23_32513717 | 23 | 32513717 | 25 | G | A | G | 0.87097 | A | 0.12903 | 6 | 0.19355 |
| 17708 | S23_32513768 | 23 | 32513768 | 51 | C | A | C | 0.87097 | A | 0.12903 | 6 | 0.19355 |
| 17709 | S23_32901551 | 23 | 32901551 | 387783 | G | A | G | 0.77419 | A | 0.22581 | 12 | 0.3871 |
| 17710 | S23_32901568 | 23 | 32901568 | 17 | C | T | T | 0.66129 | C | 0.33871 | 19 | 0.6129 |

| Site number | SNP name | Chromosome | Physical position | Physical distance from the previous SNP | Reference allele | Alternative allele | Major allele | Major allele frequency | Minor allele | Minor allele frequency | Number heterozygous | Heterozygosity |
|---|---|---|---|---|---|---|---|---|---|---|---|---|
| 17711 | S23_33078176 | 23 | 33078176 | 176608 | G | T | G | 0.91935 | T | 0.08065 | 5 | 0.16129 |
| 17712 | S23_33078200 | 23 | 33078200 | 24 | A | G | A | 0.91935 | G | 0.08065 | 5 | 0.16129 |
| 17713 | S23_33464740 | 23 | 33464740 | 386540 | G | T | G | 0.93548 | T | 0.06452 | 4 | 0.12903 |
| 17714 | S23_33464880 | 23 | 33464880 | 140 | C | T | C | 0.67742 | T | 0.32258 | 16 | 0.51613 |
| 17715 | S23_33500548 | 23 | 33500548 | 35668 | C | T | C | 0.90323 | T | 0.09677 | 6 | 0.19355 |
| 17716 | S23_33500585 | 23 | 33500585 | 37 | G | T | G | 0.87097 | T | 0.12903 | 6 | 0.19355 |
| 17717 | S23_33520150 | 23 | 33520150 | 19565 | C | T | T | 0.95161 | C | 0.04839 | 3 | 0.09677 |
| 17718 | S23_33888451 | 23 | 33888451 | 368301 | T | C | T | 0.91935 | C | 0.08065 | 5 | 0.16129 |
| 17719 | S23_33888498 | 23 | 33888498 | 47 | C | T | C | 0.83871 | T | 0.16129 | 8 | 0.25806 |
| 17720 | S23_33902843 | 23 | 33902843 | 14345 | T | C | T | 0.77419 | C | 0.22581 | 10 | 0.32258 |
| 17721 | S23_33902883 | 23 | 33902883 | 40 | C | A | C | 0.64516 | A | 0.35484 | 16 | 0.51613 |
| 17722 | S23_33902939 | 23 | 33902939 | 56 | C | T | C | 0.64516 | T | 0.35484 | 16 | 0.51613 |
| 17723 | S23_33902996 | 23 | 33902996 | 57 | A | G | A | 0.64516 | G | 0.35484 | 16 | 0.51613 |
| 17724 | S23_33919597 | 23 | 33919597 | 16601 | C | T | C | 0.83871 | T | 0.16129 | 8 | 0.25806 |
| 17725 | S23_34010016 | 23 | 34010016 | 90419 | C | G | C | 0.8871 | G | 0.1129 | 7 | 0.22581 |
| 17726 | S23_34010017 | 23 | 34010017 | 1 | G | A | G | 0.83871 | A | 0.16129 | 10 | 0.32258 |
| 17727 | S23_34010062 | 23 | 34010062 | 45 | G | A | G | 0.8871 | A | 0.1129 | 7 | 0.22581 |
| 17728 | S23_34010096 | 23 | 34010096 | 34 | C | T | C | 0.58065 | T | 0.41935 | 18 | 0.58065 |
| 17729 | S23_34010121 | 23 | 34010121 | 25 | C | G | G | 0.62903 | C | 0.37097 | 15 | 0.48387 |
| 17730 | S23_34010139 | 23 | 34010139 | 18 | C | T | T | 0.53226 | C | 0.46774 | 19 | 0.6129 |
| 17731 | S23_34156070 | 23 | 34156070 | 145931 | G | A | G | 0.56452 | A | 0.43548 | 17 | 0.54839 |
| 17732 | S23_34156138 | 23 | 34156138 | 68 | G | A | G | 0.79032 | A | 0.20968 | 13 | 0.41935 |
| 17733 | S23_34156139 | 23 | 34156139 | 1 | C | T | C | 0.95161 | T | 0.04839 | 3 | 0.09677 |
| 17734 | S23_34365948 | 23 | 34365948 | 209809 | G | A | G | 0.8871 | A | 0.1129 | 7 | 0.22581 |
| 17735 | S23_34377919 | 23 | 34377919 | 11971 | G | A | G | 0.8871 | A | 0.1129 | 5 | 0.16129 |
| 17736 | S23_34378019 | 23 | 34378019 | 100 | C | T | C | 0.93548 | T | 0.06452 | 4 | 0.12903 |
| 17737 | S23_34694178 | 23 | 34694178 | 316159 | G | A | G | 0.83871 | A | 0.16129 | 6 | 0.19355 |
| 17738 | S23_35050615 | 23 | 35050615 | 356437 | T | C | T | 0.91935 | C | 0.08065 | 5 | 0.16129 |
| 17739 | S23_35050651 | 23 | 35050651 | 36 | G | A | G | 0.91935 | A | 0.08065 | 5 | 0.16129 |
| 17740 | S23_35072889 | 23 | 35072889 | 22238 | C | T | C | 0.83871 | T | 0.16129 | 8 | 0.25806 |
| 17741 | S23_35072900 | 23 | 35072900 | 11 | A | G | G | 0.75806 | A | 0.24194 | 13 | 0.41935 |
| 17742 | S23_35072953 | 23 | 35072953 | 53 | G | C | G | 0.93548 | C | 0.06452 | 4 | 0.12903 |
| 17743 | S23_35073019 | 23 | 35073019 | 66 | G | A | G | 0.91935 | A | 0.08065 | 5 | 0.16129 |
| 17744 | S23_35122590 | 23 | 35122590 | 49571 | G | A | G | 0.91935 | A | 0.08065 | 3 | 0.09677 |
| 17745 | S23_35292687 | 23 | 35292687 | 170097 | G | A | G | 0.95161 | A | 0.04839 | 3 | 0.09677 |
| 17746 | S23_35292769 | 23 | 35292769 | 82 | T | C | T | 0.62903 | C | 0.37097 | 11 | 0.35484 |
| 17747 | S23_35810850 | 23 | 35810850 | 518081 | G | A | A | 0.59677 | G | 0.40323 | 19 | 0.6129 |
| 17748 | S23_35838799 | 23 | 35838799 | 27949 | A | T | A | 0.54839 | T | 0.45161 | 18 | 0.58065 |
| 17749 | S23_35838854 | 23 | 35838854 | 55 | C | T | T | 0.58065 | C | 0.41935 | 16 | 0.51613 |
| 17750 | S23_35838857 | 23 | 35838857 | 3 | G | T | G | 0.54839 | T | 0.45161 | 18 | 0.58065 |
| 17751 | S23_35838947 | 23 | 35838947 | 90 | A | C | A | 0.53226 | C | 0.46774 | 19 | 0.6129 |
| 17752 | S23_35838953 | 23 | 35838953 | 6 | A | T | A | 0.54839 | T | 0.45161 | 18 | 0.58065 |
| 17753 | S23_35839010 | 23 | 35839010 | 57 | T | G | T | 0.91935 | G | 0.08065 | 5 | 0.16129 |
| 17754 | S23_35839029 | 23 | 35839029 | 19 | G | A | G | 0.87097 | A | 0.12903 | 8 | 0.25806 |
| 17755 | S23_35973816 | 23 | 35973816 | 134787 | T | G | G | 0.87097 | T | 0.12903 | 4 | 0.12903 |
| 17756 | S23_35992516 | 23 | 35992516 | 18700 | T | C | C | 0.59677 | T | 0.40323 | 17 | 0.54839 |
| 17757 | S23_35992559 | 23 | 35992559 | 43 | C | A | C | 0.93548 | A | 0.06452 | 4 | 0.12903 |
| 17758 | S23_36117572 | 23 | 36117572 | 125013 | C | T | C | 0.93548 | T | 0.06452 | 4 | 0.12903 |
| 17759 | S23_36117576 | 23 | 36117576 | 4 | C | T | C | 0.51613 | T | 0.48387 | 20 | 0.64516 |
| 17760 | S23_36117583 | 23 | 36117583 | 7 | A | G | A | 0.51613 | G | 0.48387 | 20 | 0.64516 |
| 17761 | S23_36117626 | 23 | 36117626 | 43 | A | C | A | 0.51613 | C | 0.48387 | 20 | 0.64516 |
| 17762 | S23_36117729 | 23 | 36117729 | 103 | T | C | C | 0.5 | T | 0.5 | 19 | 0.6129 |
| 17763 | S23_36282168 | 23 | 36282168 | 164439 | T | C | T | 0.93548 | C | 0.06452 | 4 | 0.12903 |
| 17764 | S23_36507647 | 23 | 36507647 | 225479 | G | A | G | 0.70968 | A | 0.29032 | 16 | 0.51613 |

| Site number | SNP name | Chromosome | Physical position | Physical distance from the previous SNP | Reference allele | Alternative allele | Major allele | Major allele frequency | Minor allele | Minor allele frequency | Number heterozygous | Heterozygosity |
|---|---|---|---|---|---|---|---|---|---|---|---|---|
| 17765 | S23_36726160 | 23 | 36726160 | 218513 | C | T | C | 0.93548 | T | 0.06452 | 4 | 0.12903 |
| 17766 | S23_36726186 | 23 | 36726186 | 26 | C | T | C | 0.80645 | T | 0.19355 | 8 | 0.25806 |
| 17767 | S23_36726220 | 23 | 36726220 | 34 | T | C | T | 0.74194 | C | 0.25806 | 10 | 0.32258 |
| 17768 | S23_36867204 | 23 | 36867204 | 140984 | G | A | G | 0.80645 | A | 0.19355 | 10 | 0.32258 |
| 17769 | S23_36867366 | 23 | 36867366 | 162 | C | T | C | 0.66129 | T | 0.33871 | 13 | 0.41935 |
| 17770 | S23_37363602 | 23 | 37363602 | 496236 | G | A | G | 0.91935 | A | 0.08065 | 5 | 0.16129 |
| 17771 | S23_37363636 | 23 | 37363636 | 34 | T | G | T | 0.91935 | G | 0.08065 | 5 | 0.16129 |
| 17772 | S23_37363658 | 23 | 37363658 | 22 | G | A | G | 0.90323 | A | 0.09677 | 6 | 0.19355 |
| 17773 | S23_37363715 | 23 | 37363715 | 57 | C | T | C | 0.87097 | T | 0.12903 | 6 | 0.19355 |
| 17774 | S23_37364069 | 23 | 37364069 | 354 | G | A | G | 0.87097 | A | 0.12903 | 6 | 0.19355 |
| 17775 | S23_37924050 | 23 | 37924050 | 559981 | G | A | G | 0.77419 | A | 0.22581 | 12 | 0.3871 |
| 17776 | S23_37924110 | 23 | 37924110 | 60 | A | T | A | 0.79032 | T | 0.20968 | 11 | 0.35484 |
| 17777 | S23_38137261 | 23 | 38137261 | 213151 | C | T | C | 0.72581 | T | 0.27419 | 15 | 0.48387 |
| 17778 | S23_38723910 | 23 | 38723910 | 586649 | G | A | G | 0.77419 | A | 0.22581 | 8 | 0.25806 |
| 17779 | S23_39126746 | 23 | 39126746 | 402836 | C | A | A | 0.66129 | C | 0.33871 | 13 | 0.41935 |
| 17780 | S23_39220962 | 23 | 39220962 | 94216 | A | G | A | 0.70968 | G | 0.29032 | 10 | 0.32258 |
| 17781 | S23_39220979 | 23 | 39220979 | 17 | A | C | A | 0.8871 | C | 0.1129 | 7 | 0.22581 |
| 17782 | S23_39233387 | 23 | 39233387 | 12408 | A | T | A | 0.93548 | T | 0.06452 | 4 | 0.12903 |
| 17783 | S23_39233391 | 23 | 39233391 | 4 | C | T | C | 0.91935 | T | 0.08065 | 3 | 0.09677 |
| 17784 | S23_39274880 | 23 | 39274880 | 41489 | A | G | G | 0.79032 | A | 0.20968 | 9 | 0.29032 |
| 17785 | S23_39336982 | 23 | 39336982 | 62102 | C | T | C | 0.90323 | T | 0.09677 | 6 | 0.19355 |
| 17786 | S23_39337007 | 23 | 39337007 | 25 | G | A | G | 0.91935 | A | 0.08065 | 3 | 0.09677 |
| 17787 | S23_39337090 | 23 | 39337090 | 83 | C | T | C | 0.91935 | T | 0.08065 | 3 | 0.09677 |
| 17788 | S23_39343285 | 23 | 39343285 | 6195 | T | G | T | 0.8871 | G | 0.1129 | 5 | 0.16129 |
| 17789 | S23_39343416 | 23 | 39343416 | 131 | C | T | C | 0.83871 | T | 0.16129 | 10 | 0.32258 |
| 17790 | S23_39411881 | 23 | 39411881 | 68465 | C | T | T | 0.90323 | C | 0.09677 | 6 | 0.19355 |
| 17791 | S23_39412036 | 23 | 39412036 | 155 | A | G | G | 0.87097 | A | 0.12903 | 8 | 0.25806 |
| 17792 | S23_39810528 | 23 | 39810528 | 398492 | C | T | C | 0.82258 | T | 0.17742 | 11 | 0.35484 |
| 17793 | S23_39810627 | 23 | 39810627 | 99 | C | T | C | 0.77419 | T | 0.22581 | 8 | 0.25806 |
| 17794 | S23_39940980 | 23 | 39940980 | 130353 | T | G | T | 0.91935 | G | 0.08065 | 3 | 0.09677 |
| 17795 | S23_40064142 | 23 | 40064142 | 123162 | A | G | A | 0.75806 | G | 0.24194 | 11 | 0.35484 |
| 17796 | S23_40295705 | 23 | 40295705 | 231563 | A | G | A | 0.91935 | G | 0.08065 | 3 | 0.09677 |
| 17797 | S23_40295841 | 23 | 40295841 | 136 | A | G | A | 0.54839 | G | 0.45161 | 18 | 0.58065 |
| 17798 | S23_40503198 | 23 | 40503198 | 207357 | T | C | T | 0.87097 | C | 0.12903 | 6 | 0.19355 |
| 17799 | S23_40503378 | 23 | 40503378 | 180 | G | C | G | 0.90323 | C | 0.09677 | 6 | 0.19355 |
| 17800 | S23_40605181 | 23 | 40605181 | 101803 | C | T | C | 0.93548 | T | 0.06452 | 4 | 0.12903 |
| 17801 | S23_40605334 | 23 | 40605334 | 153 | A | G | A | 0.93548 | G | 0.06452 | 4 | 0.12903 |
| 17802 | S23_40643859 | 23 | 40643859 | 38525 | G | A | G | 0.75806 | A | 0.24194 | 9 | 0.29032 |
| 17803 | S23_40643867 | 23 | 40643867 | 8 | C | T | C | 0.77419 | T | 0.22581 | 8 | 0.25806 |
| 17804 | S23_40643920 | 23 | 40643920 | 53 | C | G | C | 0.90323 | G | 0.09677 | 6 | 0.19355 |
| 17805 | S23_40815040 | 23 | 40815040 | 171120 | G | A | G | 0.67742 | A | 0.32258 | 16 | 0.51613 |
| 17806 | S23_40815061 | 23 | 40815061 | 21 | T | C | T | 0.87097 | C | 0.12903 | 6 | 0.19355 |
| 17807 | S23_40815116 | 23 | 40815116 | 55 | C | T | C | 0.93548 | T | 0.06452 | 4 | 0.12903 |
| 17808 | S23_40841606 | 23 | 40841606 | 26490 | G | C | C | 0.74194 | G | 0.25806 | 10 | 0.32258 |
| 17809 | S23_40841610 | 23 | 40841610 | 4 | T | C | C | 0.64516 | T | 0.35484 | 12 | 0.3871 |
| 17810 | S23_41119595 | 23 | 41119595 | 277985 | C | A | A | 0.59677 | C | 0.40323 | 15 | 0.48387 |
| 17811 | S23_41145791 | 23 | 41145791 | 26196 | C | G | C | 0.77419 | G | 0.22581 | 12 | 0.3871 |
| 17812 | S23_41466442 | 23 | 41466442 | 320651 | G | A | G | 0.91935 | A | 0.08065 | 3 | 0.09677 |
| 17813 | S23_41519080 | 23 | 41519080 | 52638 | C | T | C | 0.74194 | T | 0.25806 | 16 | 0.51613 |
| 17814 | S23_41519099 | 23 | 41519099 | 19 | C | T | C | 0.91935 | T | 0.08065 | 5 | 0.16129 |
| 17815 | S23_41525202 | 23 | 41525202 | 6103 | T | C | T | 0.82258 | C | 0.17742 | 9 | 0.29032 |
| 17816 | S23_41525253 | 23 | 41525253 | 51 | C | T | C | 0.62903 | T | 0.37097 | 13 | 0.41935 |
| 17817 | S23_41525326 | 23 | 41525326 | 73 | G | A | G | 0.82258 | A | 0.17742 | 9 | 0.29032 |
| 17818 | S23_41737362 | 23 | 41737362 | 212036 | C | A | C | 0.91935 | A | 0.08065 | 5 | 0.16129 |

| Site number | SNP name | Chromosome | Physical position | Physical distance from the previous SNP | Reference allele | Alternative allele | Major allele | Major allele frequency | Minor allele | Minor allele frequency | Number heterozygous | Heterozygosity |
|---|---|---|---|---|---|---|---|---|---|---|---|---|
| 17819 | S23_41737363 | 23 | 41737363 | 1 | A | G | G | 0.53226 | A | 0.46774 | 15 | 0.48387 |
| 17820 | S23_41737405 | 23 | 41737405 | 42 | T | C | C | 0.53226 | T | 0.46774 | 15 | 0.48387 |
| 17821 | S23_41737500 | 23 | 41737500 | 95 | T | C | C | 0.53226 | T | 0.46774 | 15 | 0.48387 |
| 17822 | S23_42013435 | 23 | 42013435 | 275935 | G | A | G | 0.87097 | A | 0.12903 | 8 | 0.25806 |
| 17823 | S23_42013664 | 23 | 42013664 | 229 | C | T | C | 0.87097 | T | 0.12903 | 8 | 0.25806 |
| 17824 | S23_42033871 | 23 | 42033871 | 20207 | G | A | G | 0.95161 | A | 0.04839 | 3 | 0.09677 |
| 17825 | S23_42033891 | 23 | 42033891 | 20 | A | G | A | 0.56452 | G | 0.43548 | 13 | 0.41935 |
| 17826 | S23_42033928 | 23 | 42033928 | 37 | C | T | C | 0.74194 | T | 0.25806 | 12 | 0.3871 |
| 17827 | S23_42176515 | 23 | 42176515 | 142587 | T | C | T | 0.74194 | C | 0.25806 | 10 | 0.32258 |
| 17828 | S23_42176528 | 23 | 42176528 | 13 | C | T | C | 0.74194 | T | 0.25806 | 10 | 0.32258 |
| 17829 | S23_42176602 | 23 | 42176602 | 74 | G | T | T | 0.67742 | G | 0.32258 | 14 | 0.45161 |
| 17830 | S23_42176639 | 23 | 42176639 | 37 | C | T | C | 0.70968 | T | 0.29032 | 12 | 0.3871 |
| 17831 | S23_42396674 | 23 | 42396674 | 220035 | G | A | G | 0.95161 | A | 0.04839 | 3 | 0.09677 |
| 17832 | S23_42396700 | 23 | 42396700 | 26 | G | T | G | 0.95161 | T | 0.04839 | 3 | 0.09677 |
| 17833 | S23_42733696 | 23 | 42733696 | 336996 | T | C | T | 0.56452 | C | 0.43548 | 17 | 0.54839 |
| 17834 | S23_42733771 | 23 | 42733771 | 75 | A | T | A | 0.54839 | T | 0.45161 | 18 | 0.58065 |
| 17835 | S23_42733844 | 23 | 42733844 | 73 | A | G | A | 0.91935 | G | 0.08065 | 3 | 0.09677 |
| 17836 | S23_42733918 | 23 | 42733918 | 74 | C | A | A | 0.93548 | C | 0.06452 | 2 | 0.06452 |
| 17837 | S23_43617519 | 23 | 43617519 | 883601 | C | T | C | 0.82258 | T | 0.17742 | 7 | 0.22581 |
| 17838 | S23_43623903 | 23 | 43623903 | 6384 | G | A | A | 0.82258 | G | 0.17742 | 9 | 0.29032 |
| 17839 | S23_43624073 | 23 | 43624073 | 170 | G | A | G | 0.90323 | A | 0.09677 | 6 | 0.19355 |
| 17840 | S23_44774083 | 23 | 44774083 | 1150010 | T | C | C | 0.83871 | T | 0.16129 | 8 | 0.25806 |
| 17841 | S23_44774249 | 23 | 44774249 | 166 | G | A | A | 0.67742 | G | 0.32258 | 14 | 0.45161 |
| 17842 | S23_44774256 | 23 | 44774256 | 7 | T | C | C | 0.66129 | T | 0.33871 | 11 | 0.35484 |
| 17843 | S23_44774262 | 23 | 44774262 | 6 | G | A | G | 0.80645 | A | 0.19355 | 12 | 0.3871 |
| 17844 | S23_45058209 | 23 | 45058209 | 283947 | C | A | A | 0.79032 | C | 0.20968 | 11 | 0.35484 |
| 17845 | S23_45058385 | 23 | 45058385 | 176 | A | G | G | 0.74194 | A | 0.25806 | 14 | 0.45161 |
| 17846 | S23_45058387 | 23 | 45058387 | 2 | A | G | A | 0.75806 | G | 0.24194 | 13 | 0.41935 |
| 17847 | S23_45058404 | 23 | 45058404 | 17 | T | C | T | 0.93548 | C | 0.06452 | 4 | 0.12903 |
| 17848 | S23_45202394 | 23 | 45202394 | 143990 | C | T | C | 0.5 | T | 0.5 | 11 | 0.35484 |
| 17849 | S23_45202414 | 23 | 45202414 | 20 | G | A | A | 0.5 | G | 0.5 | 11 | 0.35484 |
| 17850 | S23_45202415 | 23 | 45202415 | 1 | T | G | G | 0.5 | T | 0.5 | 11 | 0.35484 |
| 17851 | S23_45202448 | 23 | 45202448 | 33 | A | C | A | 0.5 | C | 0.5 | 11 | 0.35484 |
| 17852 | S23_45202529 | 23 | 45202529 | 81 | T | C | C | 0.5 | T | 0.5 | 11 | 0.35484 |
| 17853 | S23_45202572 | 23 | 45202572 | 43 | T | C | T | 0.72581 | C | 0.27419 | 11 | 0.35484 |
| 17854 | S23_45202636 | 23 | 45202636 | 64 | A | G | A | 0.58065 | G | 0.41935 | 14 | 0.45161 |
| 17855 | S23_45321591 | 23 | 45321591 | 118955 | T | C | C | 0.67742 | T | 0.32258 | 12 | 0.3871 |
| 17856 | S23_45735397 | 23 | 45735397 | 413806 | T | C | T | 0.66129 | C | 0.33871 | 13 | 0.41935 |
| 17857 | S23_45735474 | 23 | 45735474 | 77 | G | A | G | 0.8871 | A | 0.1129 | 5 | 0.16129 |
| 17858 | S23_45831169 | 23 | 45831169 | 95695 | C | T | C | 0.79032 | T | 0.20968 | 11 | 0.35484 |
| 17859 | S23_45831234 | 23 | 45831234 | 65 | T | C | T | 0.93548 | C | 0.06452 | 2 | 0.06452 |
| 17860 | S23_45831272 | 23 | 45831272 | 38 | C | T | C | 0.93548 | T | 0.06452 | 2 | 0.06452 |
| 17861 | S23_46620343 | 23 | 46620343 | 789071 | C | T | C | 0.70968 | T | 0.29032 | 12 | 0.3871 |
| 17862 | S23_47380033 | 23 | 47380033 | 759690 | A | G | A | 0.90323 | G | 0.09677 | 4 | 0.12903 |
| 17863 | S23_47380040 | 23 | 47380040 | 7 | C | T | T | 0.8871 | C | 0.1129 | 5 | 0.16129 |
| 17864 | S23_47380044 | 23 | 47380044 | 4 | G | C | C | 0.8871 | G | 0.1129 | 5 | 0.16129 |
| 17865 | S23_47380119 | 23 | 47380119 | 75 | G | T | G | 0.8871 | T | 0.1129 | 5 | 0.16129 |
| 17866 | S23_47380125 | 23 | 47380125 | 6 | G | A | G | 0.90323 | A | 0.09677 | 4 | 0.12903 |
| 17867 | S23_47380134 | 23 | 47380134 | 9 | C | A | A | 0.67742 | C | 0.32258 | 14 | 0.45161 |
| 17868 | S23_47380147 | 23 | 47380147 | 13 | A | G | G | 0.8871 | A | 0.1129 | 5 | 0.16129 |
| 17869 | S23_47380151 | 23 | 47380151 | 4 | T | C | C | 0.8871 | T | 0.1129 | 5 | 0.16129 |
| 17870 | S23_47380162 | 23 | 47380162 | 11 | T | C | C | 0.59677 | T | 0.40323 | 15 | 0.48387 |
| 17871 | S23_47380197 | 23 | 47380197 | 35 | G | T | T | 0.8871 | G | 0.1129 | 5 | 0.16129 |
| 17872 | S23_47380237 | 23 | 47380237 | 40 | C | T | T | 0.8871 | C | 0.1129 | 5 | 0.16129 |

| Site number | SNP name | Chromosome | Physical position | Physical distance from the previous SNP | Reference allele | Alternative allele | Major allele | Major allele frequency | Minor allele | Minor allele frequency | Number heterozygous | Heterozygosity |
|---|---|---|---|---|---|---|---|---|---|---|---|---|
| 17873 | S23_47380266 | 23 | 47380266 | 29 | G | T | T | 0.8871 | G | 0.1129 | 5 | 0.16129 |
| 17874 | S24_165245 | 24 | 165245 | 0 | C | T | C | 0.93548 | T | 0.06452 | 4 | 0.12903 |
| 17875 | S24_165264 | 24 | 165264 | 19 | T | C | T | 0.83871 | C | 0.16129 | 8 | 0.25806 |
| 17876 | S24_165298 | 24 | 165298 | 34 | T | C | T | 0.83871 | C | 0.16129 | 8 | 0.25806 |
| 17877 | S24_165338 | 24 | 165338 | 40 | C | G | C | 0.83871 | G | 0.16129 | 8 | 0.25806 |
| 17878 | S24_165362 | 24 | 165362 | 24 | C | T | C | 0.83871 | T | 0.16129 | 8 | 0.25806 |
| 17879 | S24_304504 | 24 | 304504 | 139142 | C | G | C | 0.77419 | G | 0.22581 | 12 | 0.3871 |
| 17880 | S24_304522 | 24 | 304522 | 18 | T | C | T | 0.56452 | C | 0.43548 | 17 | 0.54839 |
| 17881 | S24_371353 | 24 | 371353 | 66831 | T | C | T | 0.53226 | C | 0.46774 | 19 | 0.6129 |
| 17882 | S24_405696 | 24 | 405696 | 34343 | T | A | T | 0.91935 | A | 0.08065 | 5 | 0.16129 |
| 17883 | S24_405822 | 24 | 405822 | 126 | A | C | A | 0.90323 | C | 0.09677 | 6 | 0.19355 |
| 17884 | S24_979621 | 24 | 979621 | 573799 | T | G | T | 0.77419 | G | 0.22581 | 14 | 0.45161 |
| 17885 | S24_979723 | 24 | 979723 | 102 | A | G | A | 0.82258 | G | 0.17742 | 9 | 0.29032 |
| 17886 | S24_1140680 | 24 | 1140680 | 160957 | C | T | T | 0.8871 | C | 0.1129 | 7 | 0.22581 |
| 17887 | S24_1140701 | 24 | 1140701 | 21 | C | A | C | 0.93548 | A | 0.06452 | 4 | 0.12903 |
| 17888 | S24_1140910 | 24 | 1140910 | 209 | C | T | C | 0.67742 | T | 0.32258 | 16 | 0.51613 |
| 17889 | S24_1190839 | 24 | 1190839 | 49929 | C | G | C | 0.80645 | G | 0.19355 | 10 | 0.32258 |
| 17890 | S24_1384391 | 24 | 1384391 | 193552 | T | C | T | 0.87097 | C | 0.12903 | 8 | 0.25806 |
| 17891 | S24_1385447 | 24 | 1385447 | 1056 | G | A | G | 0.87097 | A | 0.12903 | 4 | 0.12903 |
| 17892 | S24_1465532 | 24 | 1465532 | 80085 | C | T | C | 0.90323 | T | 0.09677 | 6 | 0.19355 |
| 17893 | S24_2023004 | 24 | 2023004 | 557472 | C | T | C | 0.59677 | T | 0.40323 | 17 | 0.54839 |
| 17894 | S24_2023018 | 24 | 2023018 | 14 | A | G | A | 0.95161 | G | 0.04839 | 3 | 0.09677 |
| 17895 | S24_2023156 | 24 | 2023156 | 138 | C | T | C | 0.85484 | T | 0.14516 | 5 | 0.16129 |
| 17896 | S24_2023216 | 24 | 2023216 | 60 | G | A | G | 0.59677 | A | 0.40323 | 17 | 0.54839 |
| 17897 | S24_2226850 | 24 | 2226850 | 203634 | T | C | T | 0.85484 | C | 0.14516 | 9 | 0.29032 |
| 17898 | S24_2226913 | 24 | 2226913 | 63 | A | G | A | 0.5 | G | 0.5 | 17 | 0.54839 |
| 17899 | S24_2226976 | 24 | 2226976 | 63 | A | G | A | 0.5 | G | 0.5 | 17 | 0.54839 |
| 17900 | S24_2227022 | 24 | 2227022 | 46 | T | C | T | 0.85484 | C | 0.14516 | 9 | 0.29032 |
| 17901 | S24_2267880 | 24 | 2267880 | 40858 | G | A | G | 0.90323 | A | 0.09677 | 6 | 0.19355 |
| 17902 | S24_2267945 | 24 | 2267945 | 65 | G | A | G | 0.8871 | A | 0.1129 | 7 | 0.22581 |
| 17903 | S24_2480343 | 24 | 2480343 | 212398 | G | A | G | 0.93548 | A | 0.06452 | 4 | 0.12903 |
| 17904 | S24_2480502 | 24 | 2480502 | 159 | G | T | G | 0.83871 | T | 0.16129 | 8 | 0.25806 |
| 17905 | S24_2480513 | 24 | 2480513 | 11 | C | T | C | 0.82258 | T | 0.17742 | 9 | 0.29032 |
| 17906 | S24_2480522 | 24 | 2480522 | 9 | G | C | G | 0.80645 | C | 0.19355 | 10 | 0.32258 |
| 17907 | S24_2480548 | 24 | 2480548 | 26 | G | A | G | 0.80645 | A | 0.19355 | 10 | 0.32258 |
| 17908 | S24_2720210 | 24 | 2720210 | 239662 | G | A | G | 0.51613 | A | 0.48387 | 16 | 0.51613 |
| 17909 | S24_2720280 | 24 | 2720280 | 70 | G | A | G | 0.66129 | A | 0.33871 | 15 | 0.48387 |
| 17910 | S24_2720290 | 24 | 2720290 | 10 | G | A | G | 0.66129 | A | 0.33871 | 15 | 0.48387 |
| 17911 | S24_2812167 | 24 | 2812167 | 91877 | C | T | T | 0.6129 | C | 0.3871 | 16 | 0.51613 |
| 17912 | S24_2820407 | 24 | 2820407 | 8240 | A | G | A | 0.62903 | G | 0.37097 | 13 | 0.41935 |
| 17913 | S24_2820415 | 24 | 2820415 | 8 | C | A | C | 0.93548 | A | 0.06452 | 4 | 0.12903 |
| 17914 | S24_2820416 | 24 | 2820416 | 1 | G | A | G | 0.69355 | A | 0.30645 | 13 | 0.41935 |
| 17915 | S24_2820512 | 24 | 2820512 | 96 | G | T | G | 0.8871 | T | 0.1129 | 7 | 0.22581 |
| 17916 | S24_2878746 | 24 | 2878746 | 58234 | A | G | G | 0.93548 | A | 0.06452 | 4 | 0.12903 |
| 17917 | S24_2878786 | 24 | 2878786 | 40 | C | T | C | 0.85484 | T | 0.14516 | 9 | 0.29032 |
| 17918 | S24_2900780 | 24 | 2900780 | 21994 | C | A | A | 0.69355 | C | 0.30645 | 15 | 0.48387 |
| 17919 | S24_2900832 | 24 | 2900832 | 52 | G | A | G | 0.90323 | A | 0.09677 | 6 | 0.19355 |
| 17920 | S24_2900935 | 24 | 2900935 | 103 | G | A | G | 0.67742 | A | 0.32258 | 14 | 0.45161 |
| 17921 | S24_3340762 | 24 | 3340762 | 439827 | C | G | C | 0.83871 | G | 0.16129 | 8 | 0.25806 |
| 17922 | S24_3340828 | 24 | 3340828 | 66 | C | T | C | 0.77419 | T | 0.22581 | 10 | 0.32258 |
| 17923 | S24_3340861 | 24 | 3340861 | 33 | A | G | A | 0.91935 | G | 0.08065 | 5 | 0.16129 |
| 17924 | S24_3340910 | 24 | 3340910 | 49 | C | T | C | 0.72581 | T | 0.27419 | 17 | 0.54839 |
| 17925 | S24_3447483 | 24 | 3447483 | 106573 | G | A | G | 0.56452 | A | 0.43548 | 17 | 0.54839 |
| 17926 | S24_3815735 | 24 | 3815735 | 368252 | G | A | G | 0.93548 | A | 0.06452 | 4 | 0.12903 |

| Site number | SNP name | Chromosome | Physical position | Physical distance from the previous SNP | Reference allele | Alternative allele | Major allele | Major allele frequency | Minor allele | Minor allele frequency | Number heterozygous | Heterozygosity |
|---|---|---|---|---|---|---|---|---|---|---|---|---|
| 17927 | S24_3876667 | 24 | 3876667 | 60932 | C | T | C | 0.74194 | T | 0.25806 | 12 | 0.3871 |
| 17928 | S24_4027578 | 24 | 4027578 | 150911 | G | C | G | 0.87097 | C | 0.12903 | 6 | 0.19355 |
| 17929 | S24_4027679 | 24 | 4027679 | 101 | T | C | T | 0.90323 | C | 0.09677 | 6 | 0.19355 |
| 17930 | S24_4094321 | 24 | 4094321 | 66642 | G | A | G | 0.79032 | A | 0.20968 | 11 | 0.35484 |
| 17931 | S24_4094323 | 24 | 4094323 | 2 | G | A | G | 0.66129 | A | 0.33871 | 13 | 0.41935 |
| 17932 | S24_4225955 | 24 | 4225955 | 131632 | C | T | C | 0.8871 | T | 0.1129 | 7 | 0.22581 |
| 17933 | S24_4225975 | 24 | 4225975 | 20 | C | T | C | 0.91935 | T | 0.08065 | 3 | 0.09677 |
| 17934 | S24_4226056 | 24 | 4226056 | 81 | T | G | G | 0.67742 | T | 0.32258 | 14 | 0.45161 |
| 17935 | S24_4226104 | 24 | 4226104 | 48 | C | A | A | 0.75806 | C | 0.24194 | 13 | 0.41935 |
| 17936 | S24_4226124 | 24 | 4226124 | 20 | T | C | C | 0.83871 | T | 0.16129 | 8 | 0.25806 |
| 17937 | S24_4270657 | 24 | 4270657 | 44533 | C | T | C | 0.8871 | T | 0.1129 | 5 | 0.16129 |
| 17938 | S24_4270697 | 24 | 4270697 | 40 | C | T | C | 0.8871 | T | 0.1129 | 5 | 0.16129 |
| 17939 | S24_4270702 | 24 | 4270702 | 5 | T | C | C | 0.74194 | T | 0.25806 | 12 | 0.3871 |
| 17940 | S24_4270715 | 24 | 4270715 | 13 | C | T | C | 0.8871 | T | 0.1129 | 5 | 0.16129 |
| 17941 | S24_4270846 | 24 | 4270846 | 131 | T | C | T | 0.8871 | C | 0.1129 | 5 | 0.16129 |
| 17942 | S24_4307399 | 24 | 4307399 | 36553 | C | T | C | 0.91935 | T | 0.08065 | 5 | 0.16129 |
| 17943 | S24_4307429 | 24 | 4307429 | 30 | G | C | G | 0.8871 | C | 0.1129 | 5 | 0.16129 |
| 17944 | S24_4331581 | 24 | 4331581 | 24152 | A | G | A | 0.87097 | G | 0.12903 | 8 | 0.25806 |
| 17945 | S24_4331604 | 24 | 4331604 | 23 | C | T | C | 0.93548 | T | 0.06452 | 4 | 0.12903 |
| 17946 | S24_4331967 | 24 | 4331967 | 363 | C | T | C | 0.69355 | T | 0.30645 | 15 | 0.48387 |
| 17947 | S24_4348862 | 24 | 4348862 | 16895 | G | A | G | 0.91935 | A | 0.08065 | 3 | 0.09677 |
| 17948 | S24_4348913 | 24 | 4348913 | 51 | A | C | A | 0.5 | C | 0.5 | 17 | 0.54839 |
| 17949 | S24_4348991 | 24 | 4348991 | 78 | G | A | G | 0.93548 | A | 0.06452 | 2 | 0.06452 |
| 17950 | S24_4395400 | 24 | 4395400 | 46409 | C | T | C | 0.93548 | T | 0.06452 | 2 | 0.06452 |
| 17951 | S24_4544690 | 24 | 4544690 | 149290 | A | G | G | 0.91935 | A | 0.08065 | 5 | 0.16129 |
| 17952 | S24_4544764 | 24 | 4544764 | 74 | A | G | G | 0.91935 | A | 0.08065 | 5 | 0.16129 |
| 17953 | S24_4544772 | 24 | 4544772 | 8 | C | A | C | 0.6129 | A | 0.3871 | 16 | 0.51613 |
| 17954 | S24_4904069 | 24 | 4904069 | 359297 | T | C | T | 0.70968 | C | 0.29032 | 12 | 0.3871 |
| 17955 | S24_4993141 | 24 | 4993141 | 89072 | G | A | G | 0.90323 | A | 0.09677 | 6 | 0.19355 |
| 17956 | S24_4993232 | 24 | 4993232 | 91 | T | C | T | 0.77419 | C | 0.22581 | 8 | 0.25806 |
| 17957 | S24_5020134 | 24 | 5020134 | 26902 | G | A | G | 0.51613 | A | 0.48387 | 16 | 0.51613 |
| 17958 | S24_5189298 | 24 | 5189298 | 169164 | G | A | G | 0.93548 | A | 0.06452 | 2 | 0.06452 |
| 17959 | S24_5189468 | 24 | 5189468 | 170 | T | C | T | 0.87097 | C | 0.12903 | 6 | 0.19355 |
| 17960 | S24_5232778 | 24 | 5232778 | 43310 | A | T | T | 0.83871 | A | 0.16129 | 8 | 0.25806 |
| 17961 | S24_5265658 | 24 | 5265658 | 32880 | G | A | G | 0.87097 | A | 0.12903 | 8 | 0.25806 |
| 17962 | S24_5265703 | 24 | 5265703 | 45 | G | C | C | 0.91935 | G | 0.08065 | 5 | 0.16129 |
| 17963 | S24_5282066 | 24 | 5282066 | 16363 | C | T | C | 0.8871 | T | 0.1129 | 7 | 0.22581 |
| 17964 | S24_5282126 | 24 | 5282126 | 60 | G | T | G | 0.8871 | T | 0.1129 | 7 | 0.22581 |
| 17965 | S24_5282193 | 24 | 5282193 | 67 | T | C | T | 0.79032 | C | 0.20968 | 13 | 0.41935 |
| 17966 | S24_5282203 | 24 | 5282203 | 10 | C | T | C | 0.75806 | T | 0.24194 | 15 | 0.48387 |
| 17967 | S24_5382219 | 24 | 5382219 | 100016 | A | G | G | 0.53226 | A | 0.46774 | 19 | 0.6129 |
| 17968 | S24_5382231 | 24 | 5382231 | 12 | C | G | C | 0.95161 | G | 0.04839 | 3 | 0.09677 |
| 17969 | S24_5439465 | 24 | 5439465 | 57234 | T | G | T | 0.85484 | G | 0.14516 | 9 | 0.29032 |
| 17970 | S24_5439471 | 24 | 5439471 | 6 | A | G | A | 0.82258 | G | 0.17742 | 9 | 0.29032 |
| 17971 | S24_5439510 | 24 | 5439510 | 39 | A | G | A | 0.8871 | G | 0.1129 | 7 | 0.22581 |
| 17972 | S24_5478241 | 24 | 5478241 | 38731 | T | C | C | 0.74194 | T | 0.25806 | 12 | 0.3871 |
| 17973 | S24_5520364 | 24 | 5520364 | 42123 | T | A | T | 0.79032 | A | 0.20968 | 11 | 0.35484 |
| 17974 | S24_5520382 | 24 | 5520382 | 18 | T | C | T | 0.95161 | C | 0.04839 | 3 | 0.09677 |
| 17975 | S24_5816014 | 24 | 5816014 | 295632 | A | G | A | 0.95161 | G | 0.04839 | 3 | 0.09677 |
| 17976 | S24_5852701 | 24 | 5852701 | 36687 | T | C | T | 0.83871 | C | 0.16129 | 8 | 0.25806 |
| 17977 | S24_5852714 | 24 | 5852714 | 13 | T | G | T | 0.83871 | G | 0.16129 | 8 | 0.25806 |
| 17978 | S24_6668368 | 24 | 6668368 | 815654 | C | A | C | 0.93548 | A | 0.06452 | 4 | 0.12903 |
| 17979 | S24_6847341 | 24 | 6847341 | 178973 | C | T | C | 0.91935 | T | 0.08065 | 5 | 0.16129 |
| 17980 | S24_6847381 | 24 | 6847381 | 40 | T | C | C | 0.90323 | T | 0.09677 | 6 | 0.19355 |

| Site number | SNP name | Chromosome | Physical position | Physical distance from the previous SNP | Reference allele | Alternative allele | Major allele | Major allele frequency | Minor allele | Minor allele frequency | Number heterozygous | Heterozygosity |
|---|---|---|---|---|---|---|---|---|---|---|---|---|
| 17981 | S24_6847417 | 24 | 6847417 | 36 | A | G | A | 0.91935 | G | 0.08065 | 5 | 0.16129 |
| 17982 | S24_7465728 | 24 | 7465728 | 618311 | G | A | G | 0.66129 | A | 0.33871 | 13 | 0.41935 |
| 17983 | S24_7465743 | 24 | 7465743 | 15 | G | A | G | 0.66129 | A | 0.33871 | 13 | 0.41935 |
| 17984 | S24_7469402 | 24 | 7469402 | 3659 | A | G | G | 0.56452 | A | 0.43548 | 17 | 0.54839 |
| 17985 | S24_7469445 | 24 | 7469445 | 43 | G | A | A | 0.56452 | G | 0.43548 | 17 | 0.54839 |
| 17986 | S24_7469460 | 24 | 7469460 | 15 | A | G | G | 0.74194 | A | 0.25806 | 12 | 0.3871 |
| 17987 | S24_7469473 | 24 | 7469473 | 13 | C | T | T | 0.56452 | C | 0.43548 | 17 | 0.54839 |
| 17988 | S24_7469475 | 24 | 7469475 | 2 | G | A | G | 0.90323 | A | 0.09677 | 4 | 0.12903 |
| 17989 | S24_7469485 | 24 | 7469485 | 10 | C | T | T | 0.56452 | C | 0.43548 | 17 | 0.54839 |
| 17990 | S24_7469509 | 24 | 7469509 | 24 | G | A | A | 0.56452 | G | 0.43548 | 17 | 0.54839 |
| 17991 | S24_7469521 | 24 | 7469521 | 12 | G | A | A | 0.56452 | G | 0.43548 | 17 | 0.54839 |
| 17992 | S24_7469531 | 24 | 7469531 | 10 | G | A | A | 0.56452 | G | 0.43548 | 17 | 0.54839 |
| 17993 | S24_7469544 | 24 | 7469544 | 13 | G | A | A | 0.56452 | G | 0.43548 | 17 | 0.54839 |
| 17994 | S24_7469578 | 24 | 7469578 | 34 | C | A | C | 0.91935 | A | 0.08065 | 5 | 0.16129 |
| 17995 | S24_7519900 | 24 | 7519900 | 50322 | A | C | A | 0.74194 | C | 0.25806 | 12 | 0.3871 |
| 17996 | S24_7519961 | 24 | 7519961 | 61 | G | A | G | 0.91935 | A | 0.08065 | 5 | 0.16129 |
| 17997 | S24_7524353 | 24 | 7524353 | 4392 | C | T | C | 0.91935 | T | 0.08065 | 5 | 0.16129 |
| 17998 | S24_7524360 | 24 | 7524360 | 7 | C | T | C | 0.91935 | T | 0.08065 | 5 | 0.16129 |
| 17999 | S24_7524366 | 24 | 7524366 | 6 | T | C | T | 0.91935 | C | 0.08065 | 5 | 0.16129 |
| 18000 | S24_7524408 | 24 | 7524408 | 42 | G | A | G | 0.91935 | A | 0.08065 | 5 | 0.16129 |
| 18001 | S24_7718975 | 24 | 7718975 | 194567 | G | T | G | 0.90323 | T | 0.09677 | 4 | 0.12903 |
| 18002 | S24_7935348 | 24 | 7935348 | 216373 | G | C | G | 0.77419 | C | 0.22581 | 10 | 0.32258 |
| 18003 | S24_7935473 | 24 | 7935473 | 125 | C | A | C | 0.95161 | A | 0.04839 | 3 | 0.09677 |
| 18004 | S24_8911787 | 24 | 8911787 | 976314 | C | T | C | 0.95161 | T | 0.04839 | 3 | 0.09677 |
| 18005 | S24_9055482 | 24 | 9055482 | 143695 | G | A | G | 0.79032 | A | 0.20968 | 11 | 0.35484 |
| 18006 | S24_9055550 | 24 | 9055550 | 68 | C | T | C | 0.56452 | T | 0.43548 | 17 | 0.54839 |
| 18007 | S24_9055673 | 24 | 9055673 | 123 | C | T | C | 0.56452 | T | 0.43548 | 15 | 0.48387 |
| 18008 | S24_9068415 | 24 | 9068415 | 12742 | C | T | C | 0.91935 | T | 0.08065 | 5 | 0.16129 |
| 18009 | S24_9068441 | 24 | 9068441 | 26 | T | C | T | 0.91935 | C | 0.08065 | 5 | 0.16129 |
| 18010 | S24_9068503 | 24 | 9068503 | 62 | T | C | T | 0.80645 | C | 0.19355 | 6 | 0.19355 |
| 18011 | S24_9068511 | 24 | 9068511 | 8 | T | G | T | 0.91935 | G | 0.08065 | 5 | 0.16129 |
| 18012 | S24_9068524 | 24 | 9068524 | 13 | T | C | T | 0.8871 | C | 0.1129 | 5 | 0.16129 |
| 18013 | S24_9068542 | 24 | 9068542 | 18 | G | T | G | 0.8871 | T | 0.1129 | 5 | 0.16129 |
| 18014 | S24_10404569 | 24 | 10404569 | 1336027 | G | A | G | 0.80645 | A | 0.19355 | 10 | 0.32258 |
| 18015 | S24_10404601 | 24 | 10404601 | 32 | G | A | G | 0.90323 | A | 0.09677 | 6 | 0.19355 |
| 18016 | S24_10404653 | 24 | 10404653 | 52 | C | T | C | 0.93548 | T | 0.06452 | 4 | 0.12903 |
| 18017 | S24_10404665 | 24 | 10404665 | 12 | G | A | G | 0.95161 | A | 0.04839 | 3 | 0.09677 |
| 18018 | S24_10404707 | 24 | 10404707 | 42 | A | G | G | 0.90323 | A | 0.09677 | 4 | 0.12903 |
| 18019 | S24_10404771 | 24 | 10404771 | 64 | G | A | G | 0.95161 | A | 0.04839 | 3 | 0.09677 |
| 18020 | S24_10576958 | 24 | 10576958 | 172187 | C | T | C | 0.69355 | T | 0.30645 | 11 | 0.35484 |
| 18021 | S24_10576982 | 24 | 10576982 | 24 | C | T | C | 0.67742 | T | 0.32258 | 10 | 0.32258 |
| 18022 | S24_11116048 | 24 | 11116048 | 539066 | G | A | G | 0.93548 | A | 0.06452 | 4 | 0.12903 |
| 18023 | S24_11321331 | 24 | 11321331 | 205283 | C | T | T | 0.67742 | C | 0.32258 | 18 | 0.58065 |
| 18024 | S24_11389142 | 24 | 11389142 | 67811 | C | T | T | 0.66129 | C | 0.33871 | 13 | 0.41935 |
| 18025 | S24_11389260 | 24 | 11389260 | 118 | A | G | G | 0.66129 | A | 0.33871 | 13 | 0.41935 |
| 18026 | S24_11389315 | 24 | 11389315 | 55 | G | A | A | 0.66129 | G | 0.33871 | 13 | 0.41935 |
| 18027 | S24_11389339 | 24 | 11389339 | 24 | A | G | G | 0.66129 | A | 0.33871 | 13 | 0.41935 |
| 18028 | S24_11389342 | 24 | 11389342 | 3 | C | T | C | 0.8871 | T | 0.1129 | 7 | 0.22581 |
| 18029 | S24_11866866 | 24 | 11866866 | 477524 | C | T | C | 0.56452 | T | 0.43548 | 15 | 0.48387 |
| 18030 | S24_11866956 | 24 | 11866956 | 90 | G | A | A | 0.59677 | G | 0.40323 | 15 | 0.48387 |
| 18031 | S24_11866996 | 24 | 11866996 | 40 | G | A | G | 0.83871 | A | 0.16129 | 8 | 0.25806 |
| 18032 | S24_11867061 | 24 | 11867061 | 65 | A | G | G | 0.6129 | A | 0.3871 | 14 | 0.45161 |
| 18033 | S24_12476605 | 24 | 12476605 | 609544 | C | A | A | 0.95161 | C | 0.04839 | 3 | 0.09677 |
| 18034 | S24_12476631 | 24 | 12476631 | 26 | T | C | T | 0.85484 | C | 0.14516 | 9 | 0.29032 |

| Site number | SNP name | Chromosome | Physical position | Physical distance from the previous SNP | Reference allele | Alternative allele | Major allele | Major allele frequency | Minor allele | Minor allele frequency | Number heterozygous | Heterozygosity |
|---|---|---|---|---|---|---|---|---|---|---|---|---|
| 18035 | S24_12476776 | 24 | 12476776 | 145 | C | T | C | 0.91935 | T | 0.08065 | 5 | 0.16129 |
| 18036 | S24_12614282 | 24 | 12614282 | 137506 | C | T | C | 0.90323 | T | 0.09677 | 4 | 0.12903 |
| 18037 | S24_12614299 | 24 | 12614299 | 17 | A | T | T | 0.54839 | A | 0.45161 | 14 | 0.45161 |
| 18038 | S24_12614345 | 24 | 12614345 | 46 | G | T | T | 0.83871 | G | 0.16129 | 8 | 0.25806 |
| 18039 | S24_12614489 | 24 | 12614489 | 144 | A | G | A | 0.62903 | G | 0.37097 | 13 | 0.41935 |
| 18040 | S24_12666253 | 24 | 12666253 | 51764 | G | A | G | 0.82258 | A | 0.17742 | 11 | 0.35484 |
| 18041 | S24_12666358 | 24 | 12666358 | 105 | T | C | T | 0.83871 | C | 0.16129 | 10 | 0.32258 |
| 18042 | S24_13055482 | 24 | 13055482 | 389124 | G | A | G | 0.69355 | A | 0.30645 | 9 | 0.29032 |
| 18043 | S24_13055540 | 24 | 13055540 | 58 | T | C | T | 0.85484 | C | 0.14516 | 9 | 0.29032 |
| 18044 | S24_14061293 | 24 | 14061293 | 1005753 | G | A | G | 0.95161 | A | 0.04839 | 3 | 0.09677 |
| 18045 | S24_14061332 | 24 | 14061332 | 39 | C | T | C | 0.67742 | T | 0.32258 | 12 | 0.3871 |
| 18046 | S24_14061369 | 24 | 14061369 | 37 | T | C | T | 0.62903 | C | 0.37097 | 13 | 0.41935 |
| 18047 | S24_14061424 | 24 | 14061424 | 55 | A | G | G | 0.66129 | A | 0.33871 | 13 | 0.41935 |
| 18048 | S24_14061440 | 24 | 14061440 | 16 | A | G | G | 0.66129 | A | 0.33871 | 13 | 0.41935 |
| 18049 | S24_14522412 | 24 | 14522412 | 460972 | A | G | A | 0.56452 | G | 0.43548 | 15 | 0.48387 |
| 18050 | S24_14522426 | 24 | 14522426 | 14 | G | A | G | 0.93548 | A | 0.06452 | 4 | 0.12903 |
| 18051 | S24_14522532 | 24 | 14522532 | 106 | A | G | G | 0.85484 | A | 0.14516 | 7 | 0.22581 |
| 18052 | S24_14522535 | 24 | 14522535 | 3 | C | T | C | 0.82258 | T | 0.17742 | 7 | 0.22581 |
| 18053 | S24_14522536 | 24 | 14522536 | 1 | A | G | G | 0.75806 | A | 0.24194 | 11 | 0.35484 |
| 18054 | S24_14522603 | 24 | 14522603 | 67 | G | T | G | 0.53226 | T | 0.46774 | 13 | 0.41935 |
| 18055 | S24_15029206 | 24 | 15029206 | 506603 | A | G | A | 0.82258 | G | 0.17742 | 7 | 0.22581 |
| 18056 | S24_15786469 | 24 | 15786469 | 757263 | G | A | G | 0.67742 | A | 0.32258 | 10 | 0.32258 |
| 18057 | S24_15786564 | 24 | 15786564 | 95 | G | C | G | 0.67742 | C | 0.32258 | 10 | 0.32258 |
| 18058 | S24_19230075 | 24 | 19230075 | 3443511 | G | A | A | 0.91935 | G | 0.08065 | 5 | 0.16129 |
| 18059 | S24_19230232 | 24 | 19230232 | 157 | T | C | T | 0.95161 | C | 0.04839 | 3 | 0.09677 |
| 18060 | S24_19230247 | 24 | 19230247 | 15 | C | A | A | 0.91935 | C | 0.08065 | 5 | 0.16129 |
| 18061 | S24_19760882 | 24 | 19760882 | 530635 | A | G | A | 0.95161 | G | 0.04839 | 3 | 0.09677 |
| 18062 | S24_19760989 | 24 | 19760989 | 107 | C | T | C | 0.95161 | T | 0.04839 | 3 | 0.09677 |
| 18063 | S24_20129525 | 24 | 20129525 | 368536 | G | A | G | 0.79032 | A | 0.20968 | 13 | 0.41935 |
| 18064 | S24_20297289 | 24 | 20297289 | 167764 | C | T | T | 0.67742 | C | 0.32258 | 12 | 0.3871 |
| 18065 | S24_20297356 | 24 | 20297356 | 67 | C | A | C | 0.74194 | A | 0.25806 | 14 | 0.45161 |
| 18066 | S24_20602531 | 24 | 20602531 | 305175 | C | T | C | 0.66129 | T | 0.33871 | 9 | 0.29032 |
| 18067 | S24_20602559 | 24 | 20602559 | 28 | C | T | C | 0.87097 | T | 0.12903 | 6 | 0.19355 |
| 18068 | S24_20709820 | 24 | 20709820 | 107261 | C | T | C | 0.93548 | T | 0.06452 | 4 | 0.12903 |
| 18069 | S24_20734129 | 24 | 20734129 | 24309 | C | T | C | 0.79032 | T | 0.20968 | 9 | 0.29032 |
| 18070 | S24_20928911 | 24 | 20928911 | 194782 | A | T | T | 0.95161 | A | 0.04839 | 1 | 0.03226 |
| 18071 | S24_20942144 | 24 | 20942144 | 13233 | G | C | C | 0.85484 | G | 0.14516 | 7 | 0.22581 |
| 18072 | S24_20942153 | 24 | 20942153 | 9 | G | A | G | 0.79032 | A | 0.20968 | 9 | 0.29032 |
| 18073 | S24_20942213 | 24 | 20942213 | 60 | T | C | T | 0.83871 | C | 0.16129 | 8 | 0.25806 |
| 18074 | S24_21260990 | 24 | 21260990 | 318777 | G | A | G | 0.95161 | A | 0.04839 | 3 | 0.09677 |
| 18075 | S24_21261021 | 24 | 21261021 | 31 | G | A | G | 0.70968 | A | 0.29032 | 14 | 0.45161 |
| 18076 | S24_21844761 | 24 | 21844761 | 583740 | A | G | G | 0.51613 | A | 0.48387 | 12 | 0.3871 |
| 18077 | S24_21844902 | 24 | 21844902 | 141 | C | T | C | 0.90323 | T | 0.09677 | 6 | 0.19355 |
| 18078 | S24_21911837 | 24 | 21911837 | 66935 | T | A | T | 0.91935 | A | 0.08065 | 5 | 0.16129 |
| 18079 | S24_21911961 | 24 | 21911961 | 124 | A | G | A | 0.69355 | G | 0.30645 | 17 | 0.54839 |
| 18080 | S24_22020582 | 24 | 22020582 | 108621 | C | T | C | 0.90323 | T | 0.09677 | 6 | 0.19355 |
| 18081 | S24_22092741 | 24 | 22092741 | 72159 | G | C | G | 0.72581 | C | 0.27419 | 15 | 0.48387 |
| 18082 | S24_22092848 | 24 | 22092848 | 107 | C | A | C | 0.72581 | A | 0.27419 | 15 | 0.48387 |
| 18083 | S24_22092921 | 24 | 22092921 | 73 | C | T | C | 0.72581 | T | 0.27419 | 15 | 0.48387 |
| 18084 | S24_22214834 | 24 | 22214834 | 121913 | C | A | C | 0.90323 | A | 0.09677 | 4 | 0.12903 |
| 18085 | S24_22478755 | 24 | 22478755 | 263921 | C | T | C | 0.67742 | T | 0.32258 | 14 | 0.45161 |
| 18086 | S24_22478778 | 24 | 22478778 | 23 | A | G | A | 0.67742 | G | 0.32258 | 14 | 0.45161 |
| 18087 | S24_24213993 | 24 | 24213993 | 1735215 | C | T | C | 0.91935 | T | 0.08065 | 5 | 0.16129 |
| 18088 | S24_24214151 | 24 | 24214151 | 158 | C | T | C | 0.93548 | T | 0.06452 | 4 | 0.12903 |

| Site number | SNP name | Chromosome | Physical position | Physical distance from the previous SNP | Reference allele | Alternative allele | Major allele | Major allele frequency | Minor allele | Minor allele frequency | Number heterozygous | Heterozygosity |
|---|---|---|---|---|---|---|---|---|---|---|---|---|
| 18089 | S24_24439681 | 24 | 24439681 | 225530 | G | A | G | 0.90323 | A | 0.09677 | 6 | 0.19355 |
| 18090 | S24_24439702 | 24 | 24439702 | 21 | A | C | A | 0.91935 | C | 0.08065 | 5 | 0.16129 |
| 18091 | S24_24927012 | 24 | 24927012 | 487310 | T | A | A | 0.51613 | T | 0.48387 | 20 | 0.64516 |
| 18092 | S24_24927025 | 24 | 24927025 | 13 | G | A | G | 0.90323 | A | 0.09677 | 4 | 0.12903 |
| 18093 | S24_24927193 | 24 | 24927193 | 168 | G | A | G | 0.66129 | A | 0.33871 | 15 | 0.48387 |
| 18094 | S24_24927226 | 24 | 24927226 | 33 | G | A | G | 0.66129 | A | 0.33871 | 15 | 0.48387 |
| 18095 | S24_24969073 | 24 | 24969073 | 41847 | G | A | G | 0.87097 | A | 0.12903 | 6 | 0.19355 |
| 18096 | S24_24969147 | 24 | 24969147 | 74 | G | A | G | 0.85484 | A | 0.14516 | 7 | 0.22581 |
| 18097 | S24_25209968 | 24 | 25209968 | 240821 | T | C | T | 0.90323 | C | 0.09677 | 6 | 0.19355 |
| 18098 | S24_25210040 | 24 | 25210040 | 72 | C | T | C | 0.95161 | T | 0.04839 | 3 | 0.09677 |
| 18099 | S24_25210071 | 24 | 25210071 | 31 | A | T | A | 0.90323 | T | 0.09677 | 6 | 0.19355 |
| 18100 | S24_25210072 | 24 | 25210072 | 1 | T | C | C | 0.75806 | T | 0.24194 | 9 | 0.29032 |
| 18101 | S24_25210179 | 24 | 25210179 | 107 | A | G | G | 0.90323 | A | 0.09677 | 6 | 0.19355 |
| 18102 | S24_25934815 | 24 | 25934815 | 724636 | T | A | T | 0.93548 | A | 0.06452 | 4 | 0.12903 |
| 18103 | S24_25934932 | 24 | 25934932 | 117 | C | G | C | 0.83871 | G | 0.16129 | 10 | 0.32258 |
| 18104 | S24_25934978 | 24 | 25934978 | 46 | G | A | G | 0.93548 | A | 0.06452 | 4 | 0.12903 |
| 18105 | S24_25935032 | 24 | 25935032 | 54 | C | T | C | 0.83871 | T | 0.16129 | 10 | 0.32258 |
| 18106 | S24_25939822 | 24 | 25939822 | 4790 | G | A | A | 0.70968 | G | 0.29032 | 10 | 0.32258 |
| 18107 | S24_26594672 | 24 | 26594672 | 654850 | C | T | C | 0.80645 | T | 0.19355 | 10 | 0.32258 |
| 18108 | S24_26594835 | 24 | 26594835 | 163 | C | T | T | 0.56452 | C | 0.43548 | 17 | 0.54839 |
| 18109 | S24_27052495 | 24 | 27052495 | 457660 | A | G | A | 0.83871 | G | 0.16129 | 10 | 0.32258 |
| 18110 | S24_27052532 | 24 | 27052532 | 37 | C | T | C | 0.74194 | T | 0.25806 | 10 | 0.32258 |
| 18111 | S24_27914102 | 24 | 27914102 | 861570 | G | C | G | 0.62903 | C | 0.37097 | 13 | 0.41935 |
| 18112 | S24_28268358 | 24 | 28268358 | 354256 | G | A | G | 0.93548 | A | 0.06452 | 4 | 0.12903 |
| 18113 | S24_28268406 | 24 | 28268406 | 48 | G | A | G | 0.69355 | A | 0.30645 | 11 | 0.35484 |
| 18114 | S24_28268424 | 24 | 28268424 | 18 | G | A | G | 0.93548 | A | 0.06452 | 4 | 0.12903 |
| 18115 | S24_28351271 | 24 | 28351271 | 82847 | G | T | G | 0.91935 | T | 0.08065 | 5 | 0.16129 |
| 18116 | S24_28756103 | 24 | 28756103 | 404832 | C | T | C | 0.8871 | T | 0.1129 | 7 | 0.22581 |
| 18117 | S24_28756179 | 24 | 28756179 | 76 | A | G | G | 0.82258 | A | 0.17742 | 9 | 0.29032 |
| 18118 | S24_29030115 | 24 | 29030115 | 273936 | C | T | C | 0.83871 | T | 0.16129 | 8 | 0.25806 |
| 18119 | S24_29125838 | 24 | 29125838 | 95723 | A | G | A | 0.79032 | G | 0.20968 | 11 | 0.35484 |
| 18120 | S24_29283107 | 24 | 29283107 | 157269 | A | G | A | 0.80645 | G | 0.19355 | 8 | 0.25806 |
| 18121 | S24_29283109 | 24 | 29283109 | 2 | A | C | A | 0.80645 | C | 0.19355 | 8 | 0.25806 |
| 18122 | S24_29586057 | 24 | 29586057 | 302948 | G | T | T | 0.75806 | G | 0.24194 | 11 | 0.35484 |
| 18123 | S24_29586260 | 24 | 29586260 | 203 | C | T | C | 0.90323 | T | 0.09677 | 6 | 0.19355 |
| 18124 | S24_29586268 | 24 | 29586268 | 8 | G | A | G | 0.90323 | A | 0.09677 | 6 | 0.19355 |
| 18125 | S24_29586311 | 24 | 29586311 | 43 | T | C | T | 0.87097 | C | 0.12903 | 6 | 0.19355 |
| 18126 | S24_29767014 | 24 | 29767014 | 180703 | A | G | G | 0.64516 | A | 0.35484 | 20 | 0.64516 |
| 18127 | S24_29767053 | 24 | 29767053 | 39 | G | T | G | 0.62903 | T | 0.37097 | 19 | 0.6129 |
| 18128 | S24_29767198 | 24 | 29767198 | 145 | C | T | C | 0.62903 | T | 0.37097 | 19 | 0.6129 |
| 18129 | S24_30706796 | 24 | 30706796 | 939598 | C | A | A | 0.90323 | C | 0.09677 | 6 | 0.19355 |
| 18130 | S24_30757480 | 24 | 30757480 | 50684 | T | C | T | 0.82258 | C | 0.17742 | 9 | 0.29032 |
| 18131 | S24_30757515 | 24 | 30757515 | 35 | C | G | C | 0.95161 | G | 0.04839 | 3 | 0.09677 |
| 18132 | S24_30778587 | 24 | 30778587 | 21072 | G | A | G | 0.53226 | A | 0.46774 | 17 | 0.54839 |
| 18133 | S24_30807639 | 24 | 30807639 | 29052 | C | T | C | 0.80645 | T | 0.19355 | 8 | 0.25806 |
| 18134 | S24_31340737 | 24 | 31340737 | 533098 | C | T | T | 0.79032 | C | 0.20968 | 11 | 0.35484 |
| 18135 | S24_31340738 | 24 | 31340738 | 1 | A | G | G | 0.95161 | A | 0.04839 | 3 | 0.09677 |
| 18136 | S24_31694077 | 24 | 31694077 | 353339 | C | G | C | 0.90323 | G | 0.09677 | 6 | 0.19355 |
| 18137 | S24_31694226 | 24 | 31694226 | 149 | G | A | G | 0.79032 | A | 0.20968 | 13 | 0.41935 |
| 18138 | S24_31800244 | 24 | 31800244 | 106018 | G | A | G | 0.95161 | A | 0.04839 | 1 | 0.03226 |
| 18139 | S24_31800360 | 24 | 31800360 | 116 | C | T | C | 0.6129 | T | 0.3871 | 14 | 0.45161 |
| 18140 | S24_31997491 | 24 | 31997491 | 197131 | C | T | T | 0.95161 | C | 0.04839 | 3 | 0.09677 |
| 18141 | S24_32084491 | 24 | 32084491 | 87000 | C | T | C | 0.95161 | T | 0.04839 | 3 | 0.09677 |
| 18142 | S24_32084678 | 24 | 32084678 | 187 | G | A | G | 0.95161 | A | 0.04839 | 3 | 0.09677 |

| Site number | SNP name | Chromosome | Physical position | Physical distance from the previous SNP | Reference allele | Alternative allele | Major allele | Major allele frequency | Minor allele | Minor allele frequency | Number heterozygous | Heterozygosity |
|---|---|---|---|---|---|---|---|---|---|---|---|---|
| 18143 | S24_32260321 | 24 | 32260321 | 175643 | G | A | G | 0.95161 | A | 0.04839 | 3 | 0.09677 |
| 18144 | S24_32260359 | 24 | 32260359 | 38 | A | G | A | 0.95161 | G | 0.04839 | 3 | 0.09677 |
| 18145 | S24_32272060 | 24 | 32272060 | 11701 | G | A | G | 0.80645 | A | 0.19355 | 10 | 0.32258 |
| 18146 | S24_32276141 | 24 | 32276141 | 4081 | C | T | C | 0.79032 | T | 0.20968 | 11 | 0.35484 |
| 18147 | S24_32307383 | 24 | 32307383 | 31242 | C | T | C | 0.82258 | T | 0.17742 | 11 | 0.35484 |
| 18148 | S24_32330873 | 24 | 32330873 | 23490 | G | A | G | 0.95161 | A | 0.04839 | 3 | 0.09677 |
| 18149 | S24_32431219 | 24 | 32431219 | 100346 | A | G | G | 0.58065 | A | 0.41935 | 14 | 0.45161 |
| 18150 | S24_32431250 | 24 | 32431250 | 31 | C | T | C | 0.87097 | T | 0.12903 | 6 | 0.19355 |
| 18151 | S24_32734270 | 24 | 32734270 | 303020 | G | A | G | 0.6129 | A | 0.3871 | 20 | 0.64516 |
| 18152 | S24_32734280 | 24 | 32734280 | 10 | A | G | A | 0.79032 | G | 0.20968 | 11 | 0.35484 |
| 18153 | S24_32962634 | 24 | 32962634 | 228354 | T | C | T | 0.87097 | C | 0.12903 | 8 | 0.25806 |
| 18154 | S24_32962723 | 24 | 32962723 | 89 | C | T | C | 0.77419 | T | 0.22581 | 12 | 0.3871 |
| 18155 | S24_32962737 | 24 | 32962737 | 14 | T | C | C | 0.5 | T | 0.5 | 19 | 0.6129 |
| 18156 | S24_32962914 | 24 | 32962914 | 177 | A | T | A | 0.64516 | T | 0.35484 | 16 | 0.51613 |
| 18157 | S24_32962938 | 24 | 32962938 | 24 | G | A | G | 0.87097 | A | 0.12903 | 8 | 0.25806 |
| 18158 | S24_33013973 | 24 | 33013973 | 51035 | C | T | T | 0.77419 | C | 0.22581 | 14 | 0.45161 |
| 18159 | S24_33014158 | 24 | 33014158 | 185 | T | C | T | 0.53226 | C | 0.46774 | 17 | 0.54839 |
| 18160 | S24_33034356 | 24 | 33034356 | 20198 | T | C | T | 0.85484 | C | 0.14516 | 9 | 0.29032 |
| 18161 | S24_33034401 | 24 | 33034401 | 45 | C | T | C | 0.90323 | T | 0.09677 | 6 | 0.19355 |
| 18162 | S24_33034443 | 24 | 33034443 | 42 | C | T | C | 0.90323 | T | 0.09677 | 6 | 0.19355 |
| 18163 | S24_33034461 | 24 | 33034461 | 18 | C | T | C | 0.72581 | T | 0.27419 | 11 | 0.35484 |
| 18164 | S24_33034578 | 24 | 33034578 | 117 | A | G | A | 0.5 | G | 0.5 | 17 | 0.54839 |
| 18165 | S24_33210914 | 24 | 33210914 | 176336 | G | A | G | 0.82258 | A | 0.17742 | 9 | 0.29032 |
| 18166 | S24_33210925 | 24 | 33210925 | 11 | A | C | A | 0.93548 | C | 0.06452 | 2 | 0.06452 |
| 18167 | S24_33211053 | 24 | 33211053 | 128 | G | A | G | 0.77419 | A | 0.22581 | 10 | 0.32258 |
| 18168 | S24_33422232 | 24 | 33422232 | 211179 | A | T | A | 0.79032 | T | 0.20968 | 13 | 0.41935 |
| 18169 | S24_33603419 | 24 | 33603419 | 181187 | T | C | T | 0.72581 | C | 0.27419 | 13 | 0.41935 |
| 18170 | S24_33785122 | 24 | 33785122 | 181703 | C | T | C | 0.93548 | T | 0.06452 | 4 | 0.12903 |
| 18171 | S24_33819181 | 24 | 33819181 | 34059 | C | G | C | 0.54839 | G | 0.45161 | 14 | 0.45161 |
| 18172 | S24_34003417 | 24 | 34003417 | 184236 | G | A | G | 0.91935 | A | 0.08065 | 5 | 0.16129 |
| 18173 | S24_34357320 | 24 | 34357320 | 353903 | A | G | A | 0.62903 | G | 0.37097 | 11 | 0.35484 |
| 18174 | S24_34357398 | 24 | 34357398 | 78 | C | T | C | 0.90323 | T | 0.09677 | 6 | 0.19355 |
| 18175 | S24_34357471 | 24 | 34357471 | 73 | T | C | C | 0.80645 | T | 0.19355 | 8 | 0.25806 |
| 18176 | S24_34424903 | 24 | 34424903 | 67432 | T | C | T | 0.77419 | C | 0.22581 | 12 | 0.3871 |
| 18177 | S24_34477772 | 24 | 34477772 | 52869 | T | C | T | 0.51613 | C | 0.48387 | 18 | 0.58065 |
| 18178 | S24_34477923 | 24 | 34477923 | 151 | G | A | G | 0.8871 | A | 0.1129 | 5 | 0.16129 |
| 18179 | S24_35475787 | 24 | 35475787 | 997864 | C | A | A | 0.75806 | C | 0.24194 | 13 | 0.41935 |
| 18180 | S24_35521299 | 24 | 35521299 | 45512 | A | G | A | 0.90323 | G | 0.09677 | 6 | 0.19355 |
| 18181 | S24_35521326 | 24 | 35521326 | 27 | T | C | C | 0.54839 | T | 0.45161 | 14 | 0.45161 |
| 18182 | S24_35646151 | 24 | 35646151 | 124825 | T | C | T | 0.93548 | C | 0.06452 | 4 | 0.12903 |
| 18183 | S24_35653278 | 24 | 35653278 | 7127 | C | G | C | 0.6129 | G | 0.3871 | 14 | 0.45161 |
| 18184 | S24_35653428 | 24 | 35653428 | 150 | A | C | A | 0.77419 | C | 0.22581 | 10 | 0.32258 |
| 18185 | S24_35696792 | 24 | 35696792 | 43364 | G | T | G | 0.87097 | T | 0.12903 | 8 | 0.25806 |
| 18186 | S24_35834711 | 24 | 35834711 | 137919 | G | A | G | 0.62903 | A | 0.37097 | 15 | 0.48387 |
| 18187 | S24_35835422 | 24 | 35835422 | 711 | C | T | C | 0.74194 | T | 0.25806 | 12 | 0.3871 |
| 18188 | S24_36172241 | 24 | 36172241 | 336819 | C | T | C | 0.70968 | T | 0.29032 | 12 | 0.3871 |
| 18189 | S24_36945265 | 24 | 36945265 | 773024 | C | T | C | 0.95161 | T | 0.04839 | 3 | 0.09677 |
| 18190 | S24_36945267 | 24 | 36945267 | 2 | A | G | A | 0.95161 | G | 0.04839 | 3 | 0.09677 |
| 18191 | S24_36945309 | 24 | 36945309 | 42 | G | A | G | 0.91935 | A | 0.08065 | 5 | 0.16129 |
| 18192 | S24_36945475 | 24 | 36945475 | 166 | G | C | G | 0.87097 | C | 0.12903 | 4 | 0.12903 |
| 18193 | S24_37310739 | 24 | 37310739 | 365264 | G | C | G | 0.93548 | C | 0.06452 | 4 | 0.12903 |
| 18194 | S24_37310760 | 24 | 37310760 | 21 | T | C | C | 0.62903 | T | 0.37097 | 15 | 0.48387 |
| 18195 | S24_37535793 | 24 | 37535793 | 225033 | G | A | G | 0.93548 | A | 0.06452 | 2 | 0.06452 |
| 18196 | S24_37535926 | 24 | 37535926 | 133 | T | G | T | 0.93548 | G | 0.06452 | 4 | 0.12903 |

| Site number | SNP name | Chromosome | Physical position | Physical distance from the previous SNP | Reference allele | Alternative allele | Major allele | Major allele frequency | Minor allele | Minor allele frequency | Number heterozygous | Heterozygosity |
|---|---|---|---|---|---|---|---|---|---|---|---|---|
| 18197 | S24_37535986 | 24 | 37535986 | 60 | C | A | C | 0.83871 | A | 0.16129 | 10 | 0.32258 |
| 18198 | S24_37623596 | 24 | 37623596 | 87610 | C | G | C | 0.70968 | G | 0.29032 | 10 | 0.32258 |
| 18199 | S24_37623710 | 24 | 37623710 | 114 | C | G | C | 0.70968 | G | 0.29032 | 10 | 0.32258 |
| 18200 | S24_38282400 | 24 | 38282400 | 658690 | G | C | G | 0.93548 | C | 0.06452 | 4 | 0.12903 |
| 18201 | S24_38348065 | 24 | 38348065 | 65665 | A | C | A | 0.95161 | C | 0.04839 | 3 | 0.09677 |
| 18202 | S24_38348135 | 24 | 38348135 | 70 | G | A | G | 0.95161 | A | 0.04839 | 3 | 0.09677 |
| 18203 | S24_38348159 | 24 | 38348159 | 24 | C | T | C | 0.77419 | T | 0.22581 | 12 | 0.3871 |
| 18204 | S24_38348176 | 24 | 38348176 | 17 | C | T | C | 0.93548 | T | 0.06452 | 4 | 0.12903 |
| 18205 | S24_38348180 | 24 | 38348180 | 4 | C | T | C | 0.83871 | T | 0.16129 | 10 | 0.32258 |
| 18206 | S24_38348227 | 24 | 38348227 | 47 | C | T | C | 0.95161 | T | 0.04839 | 3 | 0.09677 |
| 18207 | S24_38348310 | 24 | 38348310 | 83 | G | A | G | 0.93548 | A | 0.06452 | 4 | 0.12903 |
| 18208 | S24_38362272 | 24 | 38362272 | 13962 | T | A | T | 0.69355 | A | 0.30645 | 15 | 0.48387 |
| 18209 | S24_38362309 | 24 | 38362309 | 37 | C | T | C | 0.95161 | T | 0.04839 | 3 | 0.09677 |
| 18210 | S24_38362350 | 24 | 38362350 | 41 | G | A | G | 0.95161 | A | 0.04839 | 3 | 0.09677 |
| 18211 | S24_38743017 | 24 | 38743017 | 380667 | A | T | A | 0.75806 | T | 0.24194 | 13 | 0.41935 |
| 18212 | S24_38743079 | 24 | 38743079 | 62 | C | T | C | 0.93548 | T | 0.06452 | 4 | 0.12903 |
| 18213 | S24_38743081 | 24 | 38743081 | 2 | C | T | C | 0.93548 | T | 0.06452 | 4 | 0.12903 |
| 18214 | S24_38808781 | 24 | 38808781 | 65700 | G | C | G | 0.83871 | C | 0.16129 | 8 | 0.25806 |
| 18215 | S24_38808814 | 24 | 38808814 | 33 | C | T | T | 0.69355 | C | 0.30645 | 13 | 0.41935 |
| 18216 | S24_38808855 | 24 | 38808855 | 41 | C | A | C | 0.77419 | A | 0.22581 | 10 | 0.32258 |
| 18217 | S24_38928479 | 24 | 38928479 | 119624 | A | T | A | 0.83871 | T | 0.16129 | 8 | 0.25806 |
| 18218 | S24_38928500 | 24 | 38928500 | 21 | A | C | A | 0.85484 | C | 0.14516 | 7 | 0.22581 |
| 18219 | S24_39470828 | 24 | 39470828 | 542328 | C | T | T | 0.95161 | C | 0.04839 | 3 | 0.09677 |
| 18220 | S24_39470871 | 24 | 39470871 | 43 | A | C | A | 0.83871 | C | 0.16129 | 6 | 0.19355 |
| 18221 | S24_39471013 | 24 | 39471013 | 142 | C | T | C | 0.95161 | T | 0.04839 | 3 | 0.09677 |
| 18222 | S24_39694940 | 24 | 39694940 | 223927 | T | C | C | 0.87097 | T | 0.12903 | 8 | 0.25806 |
| 18223 | S24_39735213 | 24 | 39735213 | 40273 | A | C | A | 0.85484 | C | 0.14516 | 7 | 0.22581 |
| 18224 | S24_40299965 | 24 | 40299965 | 564752 | C | T | C | 0.58065 | T | 0.41935 | 18 | 0.58065 |
| 18225 | S24_40404983 | 24 | 40404983 | 105018 | A | G | G | 0.87097 | A | 0.12903 | 6 | 0.19355 |
| 18226 | S24_40545040 | 24 | 40545040 | 140057 | C | G | C | 0.80645 | G | 0.19355 | 12 | 0.3871 |
| 18227 | S24_41094483 | 24 | 41094483 | 549443 | C | T | C | 0.95161 | T | 0.04839 | 3 | 0.09677 |
| 18228 | S24_41150130 | 24 | 41150130 | 55647 | A | G | A | 0.80645 | G | 0.19355 | 12 | 0.3871 |
| 18229 | S24_41150139 | 24 | 41150139 | 9 | G | A | G | 0.79032 | A | 0.20968 | 7 | 0.22581 |
| 18230 | S24_41150181 | 24 | 41150181 | 42 | A | G | A | 0.80645 | G | 0.19355 | 12 | 0.3871 |
| 18231 | S24_41150242 | 24 | 41150242 | 61 | C | A | C | 0.80645 | A | 0.19355 | 12 | 0.3871 |
| 18232 | S24_41258910 | 24 | 41258910 | 108668 | C | T | C | 0.77419 | T | 0.22581 | 14 | 0.45161 |
| 18233 | S24_41283838 | 24 | 41283838 | 24928 | A | G | G | 0.75806 | A | 0.24194 | 15 | 0.48387 |
| 18234 | S24_41283864 | 24 | 41283864 | 26 | C | G | G | 0.6129 | C | 0.3871 | 18 | 0.58065 |
| 18235 | S24_41283885 | 24 | 41283885 | 21 | A | G | A | 0.74194 | G | 0.25806 | 14 | 0.45161 |
| 18236 | S24_41627667 | 24 | 41627667 | 343782 | G | A | G | 0.95161 | A | 0.04839 | 3 | 0.09677 |
| 18237 | S24_41935703 | 24 | 41935703 | 308036 | T | C | T | 0.72581 | C | 0.27419 | 15 | 0.48387 |
| 18238 | S24_41935782 | 24 | 41935782 | 79 | G | A | G | 0.93548 | A | 0.06452 | 4 | 0.12903 |
| 18239 | S24_41957310 | 24 | 41957310 | 21528 | C | T | C | 0.91935 | T | 0.08065 | 5 | 0.16129 |
| 18240 | S24_41957481 | 24 | 41957481 | 171 | T | C | T | 0.93548 | C | 0.06452 | 4 | 0.12903 |
| 18241 | S24_41957523 | 24 | 41957523 | 42 | C | G | C | 0.95161 | G | 0.04839 | 3 | 0.09677 |
| 18242 | S24_41972685 | 24 | 41972685 | 15162 | A | G | A | 0.8871 | G | 0.1129 | 7 | 0.22581 |
| 18243 | S24_42145956 | 24 | 42145956 | 173271 | T | A | T | 0.90323 | A | 0.09677 | 6 | 0.19355 |
| 18244 | S24_42191657 | 24 | 42191657 | 45701 | A | G | A | 0.8871 | G | 0.1129 | 7 | 0.22581 |
| 18245 | S24_42191746 | 24 | 42191746 | 89 | C | T | C | 0.69355 | T | 0.30645 | 13 | 0.41935 |
| 18246 | S24_42204694 | 24 | 42204694 | 12948 | A | G | A | 0.59677 | G | 0.40323 | 13 | 0.41935 |
| 18247 | S24_42753410 | 24 | 42753410 | 548716 | G | A | A | 0.5 | G | 0.5 | 11 | 0.35484 |
| 18248 | S24_42753422 | 24 | 42753422 | 12 | G | A | G | 0.93548 | A | 0.06452 | 4 | 0.12903 |
| 18249 | S24_43078426 | 24 | 43078426 | 325004 | A | G | G | 0.80645 | A | 0.19355 | 8 | 0.25806 |
| 18250 | S24_43097292 | 24 | 43097292 | 18866 | G | A | G | 0.93548 | A | 0.06452 | 4 | 0.12903 |

| Site number | SNP name | Chromosome | Physical position | Physical distance from the previous SNP | Reference allele | Alternative allele | Major allele | Major allele frequency | Minor allele | Minor allele frequency | Number heterozygous | Heterozygosity |
|---|---|---|---|---|---|---|---|---|---|---|---|---|
| 18251 | S24_43097311 | 24 | 43097311 | 19 | C | G | G | 0.80645 | C | 0.19355 | 6 | 0.19355 |
| 18252 | S24_43097465 | 24 | 43097465 | 154 | C | T | C | 0.93548 | T | 0.06452 | 4 | 0.12903 |
| 18253 | S24_43104079 | 24 | 43104079 | 6614 | C | T | C | 0.66129 | T | 0.33871 | 17 | 0.54839 |
| 18254 | S24_43104080 | 24 | 43104080 | 1 | G | A | G | 0.93548 | A | 0.06452 | 4 | 0.12903 |
| 18255 | S24_43104178 | 24 | 43104178 | 98 | T | C | T | 0.66129 | C | 0.33871 | 17 | 0.54839 |
| 18256 | S24_43201452 | 24 | 43201452 | 97274 | G | A | G | 0.91935 | A | 0.08065 | 5 | 0.16129 |
| 18257 | S24_43316522 | 24 | 43316522 | 115070 | G | A | G | 0.95161 | A | 0.04839 | 3 | 0.09677 |
| 18258 | S24_43316655 | 24 | 43316655 | 133 | C | T | C | 0.95161 | T | 0.04839 | 3 | 0.09677 |
| 18259 | S24_43339451 | 24 | 43339451 | 22796 | T | C | C | 0.5 | T | 0.5 | 13 | 0.41935 |
| 18260 | S24_43339694 | 24 | 43339694 | 243 | T | C | T | 0.95161 | C | 0.04839 | 3 | 0.09677 |
| 18261 | S24_43441839 | 24 | 43441839 | 102145 | C | A | C | 0.62903 | A | 0.37097 | 13 | 0.41935 |
| 18262 | S24_43499798 | 24 | 43499798 | 57959 | G | A | G | 0.91935 | A | 0.08065 | 5 | 0.16129 |
| 18263 | S24_43499839 | 24 | 43499839 | 41 | C | T | T | 0.53226 | C | 0.46774 | 15 | 0.48387 |
| 18264 | S24_43499885 | 24 | 43499885 | 46 | A | G | G | 0.53226 | A | 0.46774 | 15 | 0.48387 |
| 18265 | S24_43690569 | 24 | 43690569 | 190684 | A | G | A | 0.93548 | G | 0.06452 | 4 | 0.12903 |
| 18266 | S24_44039049 | 24 | 44039049 | 348480 | T | C | T | 0.58065 | C | 0.41935 | 12 | 0.3871 |
| 18267 | S24_44125415 | 24 | 44125415 | 86366 | C | A | C | 0.87097 | A | 0.12903 | 8 | 0.25806 |
| 18268 | S24_44416239 | 24 | 44416239 | 290824 | C | T | C | 0.91935 | T | 0.08065 | 5 | 0.16129 |
| 18269 | S24_44416276 | 24 | 44416276 | 37 | G | A | A | 0.66129 | G | 0.33871 | 11 | 0.35484 |
| 18270 | S24_44504225 | 24 | 44504225 | 87949 | C | T | C | 0.70968 | T | 0.29032 | 10 | 0.32258 |
| 18271 | S24_45105483 | 24 | 45105483 | 601258 | T | C | T | 0.87097 | C | 0.12903 | 6 | 0.19355 |
| 18272 | S24_45560829 | 24 | 45560829 | 455346 | G | A | G | 0.82258 | A | 0.17742 | 9 | 0.29032 |
| 18273 | S24_45560855 | 24 | 45560855 | 26 | A | G | A | 0.93548 | G | 0.06452 | 4 | 0.12903 |
| 18274 | S24_45560931 | 24 | 45560931 | 76 | C | T | C | 0.56452 | T | 0.43548 | 19 | 0.6129 |
| 18275 | S24_45560988 | 24 | 45560988 | 57 | G | A | G | 0.72581 | A | 0.27419 | 13 | 0.41935 |
| 18276 | S24_45979128 | 24 | 45979128 | 418140 | G | A | G | 0.85484 | A | 0.14516 | 7 | 0.22581 |
| 18277 | S24_45979161 | 24 | 45979161 | 33 | G | A | G | 0.66129 | A | 0.33871 | 13 | 0.41935 |
| 18278 | S24_45979190 | 24 | 45979190 | 29 | T | C | T | 0.8871 | C | 0.1129 | 7 | 0.22581 |
| 18279 | S24_45979339 | 24 | 45979339 | 149 | C | T | T | 0.72581 | C | 0.27419 | 11 | 0.35484 |
| 18280 | S24_46098069 | 24 | 46098069 | 118730 | C | T | C | 0.75806 | T | 0.24194 | 11 | 0.35484 |
| 18281 | S24_46098081 | 24 | 46098081 | 12 | T | C | C | 0.53226 | T | 0.46774 | 19 | 0.6129 |
| 18282 | S24_46098300 | 24 | 46098300 | 219 | T | C | C | 0.56452 | T | 0.43548 | 17 | 0.54839 |
| 18283 | S24_46382923 | 24 | 46382923 | 284623 | G | A | G | 0.79032 | A | 0.20968 | 13 | 0.41935 |
| 18284 | S24_46425344 | 24 | 46425344 | 42421 | A | G | A | 0.90323 | G | 0.09677 | 4 | 0.12903 |
| 18285 | S24_46425364 | 24 | 46425364 | 20 | C | T | C | 0.87097 | T | 0.12903 | 6 | 0.19355 |
| 18286 | S24_46425368 | 24 | 46425368 | 4 | G | A | G | 0.90323 | A | 0.09677 | 4 | 0.12903 |
| 18287 | S24_46425437 | 24 | 46425437 | 69 | C | T | C | 0.90323 | T | 0.09677 | 4 | 0.12903 |
| 18288 | S24_46446370 | 24 | 46446370 | 20933 | C | A | C | 0.8871 | A | 0.1129 | 5 | 0.16129 |
| 18289 | S24_46446409 | 24 | 46446409 | 39 | G | A | G | 0.85484 | A | 0.14516 | 5 | 0.16129 |
| 18290 | S24_46446509 | 24 | 46446509 | 100 | T | C | T | 0.6129 | C | 0.3871 | 16 | 0.51613 |
| 18291 | S24_46953109 | 24 | 46953109 | 506600 | G | A | A | 0.70968 | G | 0.29032 | 10 | 0.32258 |
| 18292 | S24_46953730 | 24 | 46953730 | 621 | T | G | T | 0.95161 | G | 0.04839 | 3 | 0.09677 |
| 18293 | S24_47113710 | 24 | 47113710 | 159980 | G | A | G | 0.93548 | A | 0.06452 | 4 | 0.12903 |
| 18294 | S24_47256952 | 24 | 47256952 | 143242 | T | C | C | 0.77419 | T | 0.22581 | 10 | 0.32258 |
| 18295 | S24_47257005 | 24 | 47257005 | 53 | G | A | G | 0.91935 | A | 0.08065 | 5 | 0.16129 |
| 18296 | S24_48120637 | 24 | 48120637 | 863632 | A | G | A | 0.80645 | G | 0.19355 | 10 | 0.32258 |
| 18297 | S24_48120693 | 24 | 48120693 | 56 | G | A | A | 0.82258 | G | 0.17742 | 9 | 0.29032 |
| 18298 | S24_48120734 | 24 | 48120734 | 41 | T | C | T | 0.53226 | C | 0.46774 | 11 | 0.35484 |
| 18299 | S24_48189323 | 24 | 48189323 | 68589 | G | C | G | 0.74194 | C | 0.25806 | 12 | 0.3871 |
| 18300 | S24_48189330 | 24 | 48189330 | 7 | T | C | C | 0.58065 | T | 0.41935 | 12 | 0.3871 |
| 18301 | S24_48189430 | 24 | 48189430 | 100 | G | A | G | 0.8871 | A | 0.1129 | 7 | 0.22581 |
| 18302 | S24_48310375 | 24 | 48310375 | 120945 | G | A | G | 0.91935 | A | 0.08065 | 5 | 0.16129 |
| 18303 | S24_48310439 | 24 | 48310439 | 64 | T | C | T | 0.90323 | C | 0.09677 | 6 | 0.19355 |
| 18304 | S24_48310456 | 24 | 48310456 | 17 | C | T | C | 0.90323 | T | 0.09677 | 6 | 0.19355 |

| Site number | SNP name | Chromosome | Physical position | Physical distance from the previous SNP | Reference allele | Alternative allele | Major allele | Major allele frequency | Minor allele | Minor allele frequency | Number heterozygous | Heterozygosity |
|---|---|---|---|---|---|---|---|---|---|---|---|---|
| 18305 | S24_48310517 | 24 | 48310517 | 61 | C | A | C | 0.90323 | A | 0.09677 | 6 | 0.19355 |
| 18306 | S24_48310552 | 24 | 48310552 | 35 | C | T | T | 0.51613 | C | 0.48387 | 16 | 0.51613 |
| 18307 | S24_49271687 | 24 | 49271687 | 961135 | C | G | C | 0.79032 | G | 0.20968 | 13 | 0.41935 |
| 18308 | S24_49271739 | 24 | 49271739 | 52 | A | G | A | 0.51613 | G | 0.48387 | 16 | 0.51613 |
| 18309 | S24_49271841 | 24 | 49271841 | 102 | G | A | G | 0.90323 | A | 0.09677 | 6 | 0.19355 |
| 18310 | S24_49394243 | 24 | 49394243 | 122402 | A | G | A | 0.87097 | G | 0.12903 | 6 | 0.19355 |
| 18311 | S24_49489951 | 24 | 49489951 | 95708 | C | T | T | 0.69355 | C | 0.30645 | 13 | 0.41935 |
| 18312 | S24_49489988 | 24 | 49489988 | 37 | G | A | G | 0.70968 | A | 0.29032 | 14 | 0.45161 |
| 18313 | S24_49858714 | 24 | 49858714 | 368726 | T | C | T | 0.93548 | C | 0.06452 | 4 | 0.12903 |
| 18314 | S24_49858749 | 24 | 49858749 | 35 | C | A | C | 0.83871 | A | 0.16129 | 8 | 0.25806 |
| 18315 | S24_49984737 | 24 | 49984737 | 125988 | C | T | C | 0.90323 | T | 0.09677 | 6 | 0.19355 |
| 18316 | S24_49984826 | 24 | 49984826 | 89 | T | C | T | 0.95161 | C | 0.04839 | 3 | 0.09677 |
| 18317 | S24_50052383 | 24 | 50052383 | 67557 | T | C | T | 0.72581 | C | 0.27419 | 13 | 0.41935 |
| 18318 | S24_50502259 | 24 | 50502259 | 449876 | T | C | T | 0.91935 | C | 0.08065 | 5 | 0.16129 |
| 18319 | S24_50609198 | 24 | 50609198 | 106939 | C | G | C | 0.93548 | G | 0.06452 | 4 | 0.12903 |
| 18320 | S24_50678790 | 24 | 50678790 | 69592 | G | T | G | 0.62903 | T | 0.37097 | 9 | 0.29032 |
| 18321 | S24_50718126 | 24 | 50718126 | 39336 | C | T | C | 0.91935 | T | 0.08065 | 5 | 0.16129 |
| 18322 | S24_51029774 | 24 | 51029774 | 311648 | A | C | A | 0.80645 | C | 0.19355 | 12 | 0.3871 |
| 18323 | S24_51029814 | 24 | 51029814 | 40 | T | G | T | 0.80645 | G | 0.19355 | 12 | 0.3871 |
| 18324 | S24_51145061 | 24 | 51145061 | 115247 | A | G | A | 0.93548 | G | 0.06452 | 4 | 0.12903 |
| 18325 | S24_51145076 | 24 | 51145076 | 15 | T | C | T | 0.85484 | C | 0.14516 | 9 | 0.29032 |
| 18326 | S24_51145120 | 24 | 51145120 | 44 | G | A | G | 0.85484 | A | 0.14516 | 9 | 0.29032 |
| 18327 | S24_51304895 | 24 | 51304895 | 159775 | T | C | T | 0.91935 | C | 0.08065 | 3 | 0.09677 |
| 18328 | S24_51304919 | 24 | 51304919 | 24 | T | G | T | 0.91935 | G | 0.08065 | 3 | 0.09677 |
| 18329 | S24_51304974 | 24 | 51304974 | 55 | C | T | C | 0.91935 | T | 0.08065 | 3 | 0.09677 |
| 18330 | S24_51305003 | 24 | 51305003 | 29 | C | T | C | 0.91935 | T | 0.08065 | 3 | 0.09677 |
| 18331 | S24_51305058 | 24 | 51305058 | 55 | G | A | G | 0.91935 | A | 0.08065 | 3 | 0.09677 |
| 18332 | S24_51393313 | 24 | 51393313 | 88255 | G | A | G | 0.54839 | A | 0.45161 | 14 | 0.45161 |
| 18333 | S24_51627077 | 24 | 51627077 | 233764 | T | C | C | 0.75806 | T | 0.24194 | 13 | 0.41935 |
| 18334 | S24_51627087 | 24 | 51627087 | 10 | C | G | C | 0.87097 | G | 0.12903 | 6 | 0.19355 |
| 18335 | S24_51692403 | 24 | 51692403 | 65316 | A | C | A | 0.93548 | C | 0.06452 | 4 | 0.12903 |
| 18336 | S24_51692438 | 24 | 51692438 | 35 | A | G | A | 0.95161 | G | 0.04839 | 3 | 0.09677 |
| 18337 | S24_52440505 | 24 | 52440505 | 748067 | A | G | A | 0.6129 | G | 0.3871 | 18 | 0.58065 |
| 18338 | S24_52847136 | 24 | 52847136 | 406631 | C | G | C | 0.53226 | G | 0.46774 | 17 | 0.54839 |
| 18339 | S24_52847274 | 24 | 52847274 | 138 | T | C | T | 0.91935 | C | 0.08065 | 5 | 0.16129 |
| 18340 | S24_52847326 | 24 | 52847326 | 52 | C | T | C | 0.75806 | T | 0.24194 | 11 | 0.35484 |
| 18341 | S24_52847385 | 24 | 52847385 | 59 | T | G | T | 0.87097 | G | 0.12903 | 8 | 0.25806 |
| 18342 | S24_52966623 | 24 | 52966623 | 119238 | A | G | A | 0.70968 | G | 0.29032 | 14 | 0.45161 |
| 18343 | S24_52966636 | 24 | 52966636 | 13 | G | A | G | 0.90323 | A | 0.09677 | 6 | 0.19355 |
| 18344 | S24_53295184 | 24 | 53295184 | 328548 | T | C | T | 0.53226 | C | 0.46774 | 15 | 0.48387 |
| 18345 | S24_53295289 | 24 | 53295289 | 105 | C | G | C | 0.93548 | G | 0.06452 | 4 | 0.12903 |
| 18346 | S24_53478662 | 24 | 53478662 | 183373 | A | G | A | 0.70968 | G | 0.29032 | 14 | 0.45161 |
| 18347 | S24_53752957 | 24 | 53752957 | 274295 | A | C | C | 0.80645 | A | 0.19355 | 10 | 0.32258 |
| 18348 | S24_53752960 | 24 | 53752960 | 3 | T | C | T | 0.8871 | C | 0.1129 | 7 | 0.22581 |
| 18349 | S24_53753051 | 24 | 53753051 | 91 | G | A | A | 0.69355 | G | 0.30645 | 13 | 0.41935 |
| 18350 | S24_53777175 | 24 | 53777175 | 24124 | T | A | T | 0.91935 | A | 0.08065 | 3 | 0.09677 |
| 18351 | S24_53777218 | 24 | 53777218 | 43 | C | T | C | 0.87097 | T | 0.12903 | 8 | 0.25806 |
| 18352 | S24_53777251 | 24 | 53777251 | 33 | C | T | C | 0.83871 | T | 0.16129 | 10 | 0.32258 |
| 18353 | S24_53777342 | 24 | 53777342 | 91 | A | C | A | 0.83871 | C | 0.16129 | 10 | 0.32258 |
| 18354 | S24_53783722 | 24 | 53783722 | 6380 | A | G | A | 0.93548 | G | 0.06452 | 4 | 0.12903 |
| 18355 | S24_53783779 | 24 | 53783779 | 57 | C | A | C | 0.8871 | A | 0.1129 | 7 | 0.22581 |
| 18356 | S24_53807934 | 24 | 53807934 | 24155 | G | C | G | 0.93548 | C | 0.06452 | 2 | 0.06452 |
| 18357 | S24_53815259 | 24 | 53815259 | 7325 | G | T | G | 0.91935 | T | 0.08065 | 5 | 0.16129 |
| 18358 | S24_53815313 | 24 | 53815313 | 54 | G | A | G | 0.95161 | A | 0.04839 | 3 | 0.09677 |

| Site number | SNP name | Chromosome | Physical position | Physical distance from the previous SNP | Reference allele | Alternative allele | Major allele | Major allele frequency | Minor allele | Minor allele frequency | Number heterozygous | Heterozygosity |
|---|---|---|---|---|---|---|---|---|---|---|---|---|
| 18359 | S24_53892785 | 24 | 53892785 | 77472 | C | G | C | 0.83871 | G | 0.16129 | 10 | 0.32258 |
| 18360 | S24_53892790 | 24 | 53892790 | 5 | T | G | T | 0.93548 | G | 0.06452 | 4 | 0.12903 |
| 18361 | S24_54138542 | 24 | 54138542 | 245752 | G | A | G | 0.83871 | A | 0.16129 | 8 | 0.25806 |
| 18362 | S24_54138664 | 24 | 54138664 | 122 | A | G | A | 0.91935 | G | 0.08065 | 5 | 0.16129 |
| 18363 | S24_54231160 | 24 | 54231160 | 92496 | T | G | G | 0.54839 | T | 0.45161 | 16 | 0.51613 |
| 18364 | S24_54788380 | 24 | 54788380 | 557220 | G | A | G | 0.64516 | A | 0.35484 | 12 | 0.3871 |
| 18365 | S24_54788393 | 24 | 54788393 | 13 | T | C | T | 0.93548 | C | 0.06452 | 4 | 0.12903 |
| 18366 | S24_54788414 | 24 | 54788414 | 21 | C | T | C | 0.64516 | T | 0.35484 | 12 | 0.3871 |
| 18367 | S24_54788418 | 24 | 54788418 | 4 | C | T | C | 0.93548 | T | 0.06452 | 4 | 0.12903 |
| 18368 | S24_54788430 | 24 | 54788430 | 12 | G | A | G | 0.93548 | A | 0.06452 | 4 | 0.12903 |
| 18369 | S24_54843131 | 24 | 54843131 | 54701 | C | A | A | 0.75806 | C | 0.24194 | 11 | 0.35484 |
| 18370 | S24_55102315 | 24 | 55102315 | 259184 | G | A | G | 0.93548 | A | 0.06452 | 4 | 0.12903 |
| 18371 | S24_55403932 | 24 | 55403932 | 301617 | G | A | G | 0.85484 | A | 0.14516 | 9 | 0.29032 |
| 18372 | S24_55932773 | 24 | 55932773 | 528841 | T | C | T | 0.67742 | C | 0.32258 | 14 | 0.45161 |
| 18373 | S24_55932815 | 24 | 55932815 | 42 | C | T | C | 0.5 | T | 0.5 | 17 | 0.54839 |
| 18374 | S24_55932841 | 24 | 55932841 | 26 | G | A | G | 0.67742 | A | 0.32258 | 14 | 0.45161 |
| 18375 | S24_55932853 | 24 | 55932853 | 12 | C | T | C | 0.66129 | T | 0.33871 | 15 | 0.48387 |
| 18376 | S24_55932873 | 24 | 55932873 | 20 | G | A | G | 0.69355 | A | 0.30645 | 17 | 0.54839 |
| 18377 | S24_55932917 | 24 | 55932917 | 44 | A | G | A | 0.93548 | G | 0.06452 | 4 | 0.12903 |
| 18378 | S24_55932959 | 24 | 55932959 | 42 | T | C | T | 0.93548 | C | 0.06452 | 4 | 0.12903 |
| 18379 | S24_55986626 | 24 | 55986626 | 53667 | T | G | G | 0.87097 | T | 0.12903 | 6 | 0.19355 |
| 18380 | S24_55986693 | 24 | 55986693 | 67 | A | C | A | 0.90323 | C | 0.09677 | 6 | 0.19355 |
| 18381 | S24_56112568 | 24 | 56112568 | 125875 | C | T | C | 0.51613 | T | 0.48387 | 16 | 0.51613 |
| 18382 | S24_56112619 | 24 | 56112619 | 51 | C | T | C | 0.56452 | T | 0.43548 | 17 | 0.54839 |
| 18383 | S24_56125670 | 24 | 56125670 | 13051 | T | C | C | 0.66129 | T | 0.33871 | 15 | 0.48387 |
| 18384 | S24_56441620 | 24 | 56441620 | 315950 | T | C | C | 0.93548 | T | 0.06452 | 4 | 0.12903 |
| 18385 | S24_56441646 | 24 | 56441646 | 26 | C | A | A | 0.93548 | C | 0.06452 | 4 | 0.12903 |
| 18386 | S24_56441659 | 24 | 56441659 | 13 | T | C | T | 0.83871 | C | 0.16129 | 8 | 0.25806 |
| 18387 | S24_56441724 | 24 | 56441724 | 65 | A | G | A | 0.64516 | G | 0.35484 | 14 | 0.45161 |
| 18388 | S24_56441773 | 24 | 56441773 | 49 | A | G | A | 0.51613 | G | 0.48387 | 18 | 0.58065 |
| 18389 | S24_56566279 | 24 | 56566279 | 124506 | C | T | T | 0.70968 | C | 0.29032 | 12 | 0.3871 |
| 18390 | S24_56613720 | 24 | 56613720 | 47441 | C | G | C | 0.72581 | G | 0.27419 | 13 | 0.41935 |
| 18391 | S24_56613739 | 24 | 56613739 | 19 | C | G | C | 0.72581 | G | 0.27419 | 13 | 0.41935 |
| 18392 | S24_56613789 | 24 | 56613789 | 50 | C | G | C | 0.77419 | G | 0.22581 | 10 | 0.32258 |
| 18393 | S24_56613801 | 24 | 56613801 | 12 | G | A | G | 0.82258 | A | 0.17742 | 11 | 0.35484 |
| 18394 | S24_56613821 | 24 | 56613821 | 20 | C | A | C | 0.95161 | A | 0.04839 | 3 | 0.09677 |
| 18395 | S24_56658183 | 24 | 56658183 | 44362 | C | T | C | 0.93548 | T | 0.06452 | 4 | 0.12903 |
| 18396 | S24_56658185 | 24 | 56658185 | 2 | C | T | C | 0.72581 | T | 0.27419 | 13 | 0.41935 |
| 18397 | S24_56805044 | 24 | 56805044 | 146859 | C | G | G | 0.80645 | C | 0.19355 | 10 | 0.32258 |
| 18398 | S24_56854397 | 24 | 56854397 | 49353 | T | C | T | 0.93548 | C | 0.06452 | 4 | 0.12903 |
| 18399 | S24_56854443 | 24 | 56854443 | 46 | C | T | C | 0.64516 | T | 0.35484 | 18 | 0.58065 |
| 18400 | S24_56854511 | 24 | 56854511 | 68 | G | T | T | 0.79032 | G | 0.20968 | 13 | 0.41935 |
| 18401 | S24_56854557 | 24 | 56854557 | 46 | G | A | G | 0.95161 | A | 0.04839 | 3 | 0.09677 |
| 18402 | S24_56908378 | 24 | 56908378 | 53821 | C | T | C | 0.75806 | T | 0.24194 | 11 | 0.35484 |
| 18403 | S24_56908500 | 24 | 56908500 | 122 | C | T | C | 0.75806 | T | 0.24194 | 11 | 0.35484 |
| 18404 | S24_56955813 | 24 | 56955813 | 47313 | G | A | G | 0.80645 | A | 0.19355 | 10 | 0.32258 |
| 18405 | S24_56955877 | 24 | 56955877 | 64 | C | T | C | 0.69355 | T | 0.30645 | 13 | 0.41935 |
| 18406 | S24_57078192 | 24 | 57078192 | 122315 | C | G | G | 0.80645 | C | 0.19355 | 10 | 0.32258 |
| 18407 | S24_57078193 | 24 | 57078193 | 1 | T | C | C | 0.80645 | T | 0.19355 | 10 | 0.32258 |
| 18408 | S24_57078360 | 24 | 57078360 | 167 | G | A | G | 0.70968 | A | 0.29032 | 12 | 0.3871 |
| 18409 | S24_57397883 | 24 | 57397883 | 319523 | G | A | G | 0.62903 | A | 0.37097 | 11 | 0.35484 |
| 18410 | S24_57635813 | 24 | 57635813 | 237930 | G | A | G | 0.93548 | A | 0.06452 | 4 | 0.12903 |
| 18411 | S24_57635816 | 24 | 57635816 | 3 | G | T | G | 0.77419 | T | 0.22581 | 12 | 0.3871 |
| 18412 | S24_57635899 | 24 | 57635899 | 83 | C | A | C | 0.75806 | A | 0.24194 | 11 | 0.35484 |

| Site number | SNP name | Chromosome | Physical position | Physical distance from the previous SNP | Reference allele | Alternative allele | Major allele | Major allele frequency | Minor allele | Minor allele frequency | Number heterozygous | Heterozygosity |
|---|---|---|---|---|---|---|---|---|---|---|---|---|
| 18413 | S24_57635976 | 24 | 57635976 | 77 | G | C | G | 0.75806 | C | 0.24194 | 11 | 0.35484 |
| 18414 | S24_57779268 | 24 | 57779268 | 143292 | T | C | C | 0.72581 | T | 0.27419 | 15 | 0.48387 |
| 18415 | S24_57779349 | 24 | 57779349 | 81 | C | T | C | 0.8871 | T | 0.1129 | 7 | 0.22581 |
| 18416 | S24_57779395 | 24 | 57779395 | 46 | T | C | C | 0.77419 | T | 0.22581 | 14 | 0.45161 |
| 18417 | S24_57873537 | 24 | 57873537 | 94142 | A | T | A | 0.95161 | T | 0.04839 | 3 | 0.09677 |
| 18418 | S24_57992710 | 24 | 57992710 | 119173 | A | G | A | 0.87097 | G | 0.12903 | 6 | 0.19355 |
| 18419 | S24_57992716 | 24 | 57992716 | 6 | G | A | G | 0.83871 | A | 0.16129 | 10 | 0.32258 |
| 18420 | S24_58141999 | 24 | 58141999 | 149283 | C | T | C | 0.85484 | T | 0.14516 | 7 | 0.22581 |
| 18421 | S24_58142027 | 24 | 58142027 | 28 | C | T | C | 0.93548 | T | 0.06452 | 4 | 0.12903 |
| 18422 | S24_58142101 | 24 | 58142101 | 74 | C | T | C | 0.93548 | T | 0.06452 | 4 | 0.12903 |
| 18423 | S24_58342045 | 24 | 58342045 | 199944 | T | C | C | 0.90323 | T | 0.09677 | 4 | 0.12903 |
| 18424 | S24_58342136 | 24 | 58342136 | 91 | A | G | A | 0.80645 | G | 0.19355 | 10 | 0.32258 |
| 18425 | S24_58411738 | 24 | 58411738 | 69602 | A | G | A | 0.83871 | G | 0.16129 | 10 | 0.32258 |
| 18426 | S24_58498088 | 24 | 58498088 | 86350 | C | T | C | 0.62903 | T | 0.37097 | 15 | 0.48387 |
| 18427 | S24_58498091 | 24 | 58498091 | 3 | C | T | C | 0.62903 | T | 0.37097 | 15 | 0.48387 |
| 18428 | S24_58498142 | 24 | 58498142 | 51 | C | T | C | 0.8871 | T | 0.1129 | 7 | 0.22581 |
| 18429 | S24_58531751 | 24 | 58531751 | 33609 | G | A | G | 0.93548 | A | 0.06452 | 4 | 0.12903 |
| 18430 | S24_58531767 | 24 | 58531767 | 16 | G | C | C | 0.77419 | G | 0.22581 | 8 | 0.25806 |
| 18431 | S24_58531782 | 24 | 58531782 | 15 | G | A | G | 0.87097 | A | 0.12903 | 8 | 0.25806 |
| 18432 | S24_58544970 | 24 | 58544970 | 13188 | C | G | C | 0.79032 | G | 0.20968 | 13 | 0.41935 |
| 18433 | S24_58662812 | 24 | 58662812 | 117842 | T | C | T | 0.77419 | C | 0.22581 | 12 | 0.3871 |
| 18434 | S24_58662904 | 24 | 58662904 | 92 | T | A | A | 0.51613 | T | 0.48387 | 10 | 0.32258 |
| 18435 | S24_58662960 | 24 | 58662960 | 56 | G | A | G | 0.85484 | A | 0.14516 | 7 | 0.22581 |
| 18436 | S24_58772746 | 24 | 58772746 | 109786 | A | G | A | 0.77419 | G | 0.22581 | 10 | 0.32258 |
| 18437 | S24_58772788 | 24 | 58772788 | 42 | C | G | C | 0.90323 | G | 0.09677 | 6 | 0.19355 |
| 18438 | S24_58772931 | 24 | 58772931 | 143 | A | G | G | 0.58065 | A | 0.41935 | 16 | 0.51613 |
| 18439 | S24_58804043 | 24 | 58804043 | 31112 | A | G | G | 0.70968 | A | 0.29032 | 14 | 0.45161 |
| 18440 | S24_58804102 | 24 | 58804102 | 59 | C | T | C | 0.64516 | T | 0.35484 | 18 | 0.58065 |
| 18441 | S24_58804173 | 24 | 58804173 | 71 | T | C | C | 0.72581 | T | 0.27419 | 13 | 0.41935 |
| 18442 | S24_58804224 | 24 | 58804224 | 51 | C | G | G | 0.54839 | C | 0.45161 | 18 | 0.58065 |
| 18443 | S24_58804225 | 24 | 58804225 | 1 | C | G | C | 0.59677 | G | 0.40323 | 19 | 0.6129 |
| 18444 | S24_58804234 | 24 | 58804234 | 9 | A | G | G | 0.54839 | A | 0.45161 | 18 | 0.58065 |
| 18445 | S24_59025847 | 24 | 59025847 | 221613 | A | G | A | 0.85484 | G | 0.14516 | 7 | 0.22581 |
| 18446 | S24_59025912 | 24 | 59025912 | 65 | C | T | C | 0.77419 | T | 0.22581 | 12 | 0.3871 |
| 18447 | S24_59025989 | 24 | 59025989 | 77 | C | G | C | 0.85484 | G | 0.14516 | 7 | 0.22581 |
| 18448 | S24_59025998 | 24 | 59025998 | 9 | G | A | G | 0.85484 | A | 0.14516 | 7 | 0.22581 |
| 18449 | S24_59026009 | 24 | 59026009 | 11 | T | C | C | 0.85484 | T | 0.14516 | 9 | 0.29032 |
| 18450 | S24_59026040 | 24 | 59026040 | 31 | G | A | G | 0.93548 | A | 0.06452 | 4 | 0.12903 |
| 18451 | S24_59026052 | 24 | 59026052 | 12 | G | A | G | 0.93548 | A | 0.06452 | 4 | 0.12903 |
| 18452 | S24_59259922 | 24 | 59259922 | 233870 | T | C | C | 0.6129 | T | 0.3871 | 18 | 0.58065 |
| 18453 | S24_59259934 | 24 | 59259934 | 12 | C | T | C | 0.67742 | T | 0.32258 | 16 | 0.51613 |
| 18454 | S24_59260097 | 24 | 59260097 | 163 | A | T | A | 0.59677 | T | 0.40323 | 13 | 0.41935 |
| 18455 | S24_59260110 | 24 | 59260110 | 13 | C | T | T | 0.53226 | C | 0.46774 | 13 | 0.41935 |
| 18456 | S24_59562416 | 24 | 59562416 | 302306 | T | C | T | 0.85484 | C | 0.14516 | 7 | 0.22581 |
| 18457 | S24_59595588 | 24 | 59595588 | 33172 | A | G | G | 0.93548 | A | 0.06452 | 4 | 0.12903 |
| 18458 | S24_59595636 | 24 | 59595636 | 48 | C | G | G | 0.72581 | C | 0.27419 | 13 | 0.41935 |
| 18459 | S24_59595639 | 24 | 59595639 | 3 | T | A | A | 0.70968 | T | 0.29032 | 12 | 0.3871 |
| 18460 | S24_59626650 | 24 | 59626650 | 31011 | T | C | T | 0.95161 | C | 0.04839 | 3 | 0.09677 |
| 18461 | S24_59626683 | 24 | 59626683 | 33 | G | C | G | 0.95161 | C | 0.04839 | 3 | 0.09677 |
| 18462 | S24_59626742 | 24 | 59626742 | 59 | A | G | A | 0.5 | G | 0.5 | 17 | 0.54839 |
| 18463 | S24_59954376 | 24 | 59954376 | 327634 | G | A | A | 0.56452 | G | 0.43548 | 13 | 0.41935 |
| 18464 | S24_60283493 | 24 | 60283493 | 329117 | C | A | C | 0.90323 | A | 0.09677 | 6 | 0.19355 |
| 18465 | S24_60283499 | 24 | 60283499 | 6 | A | G | A | 0.67742 | G | 0.32258 | 14 | 0.45161 |
| 18466 | S24_60283526 | 24 | 60283526 | 27 | G | A | G | 0.85484 | A | 0.14516 | 7 | 0.22581 |

| Site number | SNP name | Chromosome | Physical position | Physical distance from the previous SNP | Reference allele | Alternative allele | Major allele | Major allele frequency | Minor allele | Minor allele frequency | Number heterozygous | Heterozygosity |
|---|---|---|---|---|---|---|---|---|---|---|---|---|
| 18467 | S24_60325256 | 24 | 60325256 | 41730 | A | G | A | 0.69355 | G | 0.30645 | 13 | 0.41935 |
| 18468 | S24_60644595 | 24 | 60644595 | 319339 | A | G | A | 0.77419 | G | 0.22581 | 12 | 0.3871 |
| 18469 | S24_60644667 | 24 | 60644667 | 72 | C | T | C | 0.8871 | T | 0.1129 | 7 | 0.22581 |
| 18470 | S24_60938387 | 24 | 60938387 | 293720 | A | C | A | 0.80645 | C | 0.19355 | 8 | 0.25806 |
| 18471 | S24_60938477 | 24 | 60938477 | 90 | C | T | C | 0.80645 | T | 0.19355 | 8 | 0.25806 |
| 18472 | S24_60938511 | 24 | 60938511 | 34 | T | C | T | 0.80645 | C | 0.19355 | 8 | 0.25806 |
| 18473 | S24_61071455 | 24 | 61071455 | 132944 | T | C | C | 0.56452 | T | 0.43548 | 15 | 0.48387 |
| 18474 | S24_61071483 | 24 | 61071483 | 28 | G | C | C | 0.51613 | G | 0.48387 | 16 | 0.51613 |
| 18475 | S24_61071504 | 24 | 61071504 | 21 | A | T | A | 0.93548 | T | 0.06452 | 4 | 0.12903 |
| 18476 | S24_61071514 | 24 | 61071514 | 10 | T | C | C | 0.59677 | T | 0.40323 | 15 | 0.48387 |
| 18477 | S24_61071534 | 24 | 61071534 | 20 | G | C | G | 0.91935 | C | 0.08065 | 5 | 0.16129 |
| 18478 | S24_61082920 | 24 | 61082920 | 11386 | C | G | C | 0.85484 | G | 0.14516 | 9 | 0.29032 |
| 18479 | S24_61111024 | 24 | 61111024 | 28104 | C | G | C | 0.85484 | G | 0.14516 | 9 | 0.29032 |
| 18480 | S24_61111240 | 24 | 61111240 | 216 | G | T | G | 0.8871 | T | 0.1129 | 5 | 0.16129 |
| 18481 | S24_61715355 | 24 | 61715355 | 604115 | C | T | T | 0.67742 | C | 0.32258 | 10 | 0.32258 |
| 18482 | S24_61715368 | 24 | 61715368 | 13 | C | G | C | 0.69355 | G | 0.30645 | 9 | 0.29032 |
| 18483 | S24_61715462 | 24 | 61715462 | 94 | C | T | C | 0.83871 | T | 0.16129 | 6 | 0.19355 |
| 18484 | S24_61715522 | 24 | 61715522 | 60 | C | A | C | 0.90323 | A | 0.09677 | 6 | 0.19355 |
| 18485 | S24_61786421 | 24 | 61786421 | 70899 | C | T | C | 0.5 | T | 0.5 | 19 | 0.6129 |
| 18486 | S24_61919597 | 24 | 61919597 | 133176 | C | T | T | 0.75806 | C | 0.24194 | 15 | 0.48387 |
| 18487 | S24_62060796 | 24 | 62060796 | 141199 | T | C | T | 0.8871 | C | 0.1129 | 7 | 0.22581 |
| 18488 | S24_62060798 | 24 | 62060798 | 2 | C | T | C | 0.8871 | T | 0.1129 | 7 | 0.22581 |
| 18489 | S24_62060800 | 24 | 62060800 | 2 | G | A | G | 0.90323 | A | 0.09677 | 6 | 0.19355 |
| 18490 | S24_62060825 | 24 | 62060825 | 25 | C | T | C | 0.8871 | T | 0.1129 | 7 | 0.22581 |
| 18491 | S24_62060827 | 24 | 62060827 | 2 | G | A | G | 0.8871 | A | 0.1129 | 7 | 0.22581 |
| 18492 | S24_62060922 | 24 | 62060922 | 95 | T | C | C | 0.64516 | T | 0.35484 | 18 | 0.58065 |
| 18493 | S24_62060924 | 24 | 62060924 | 2 | T | C | T | 0.51613 | C | 0.48387 | 18 | 0.58065 |
| 18494 | S24_62060957 | 24 | 62060957 | 33 | A | G | A | 0.87097 | G | 0.12903 | 8 | 0.25806 |
| 18495 | S24_62061007 | 24 | 62061007 | 50 | G | T | G | 0.87097 | T | 0.12903 | 8 | 0.25806 |
| 18496 | S24_62116625 | 24 | 62116625 | 55618 | G | C | G | 0.87097 | C | 0.12903 | 8 | 0.25806 |
| 18497 | S24_62116754 | 24 | 62116754 | 129 | A | G | A | 0.85484 | G | 0.14516 | 9 | 0.29032 |
| 18498 | S24_62116828 | 24 | 62116828 | 74 | G | A | G | 0.87097 | A | 0.12903 | 8 | 0.25806 |
| 18499 | S24_62161731 | 24 | 62161731 | 44903 | C | T | C | 0.53226 | T | 0.46774 | 17 | 0.54839 |
| 18500 | S24_62161815 | 24 | 62161815 | 84 | G | A | G | 0.53226 | A | 0.46774 | 17 | 0.54839 |
| 18501 | S24_62161905 | 24 | 62161905 | 90 | C | T | C | 0.6129 | T | 0.3871 | 20 | 0.64516 |
| 18502 | S24_62223157 | 24 | 62223157 | 61252 | C | T | C | 0.87097 | T | 0.12903 | 8 | 0.25806 |
| 18503 | S24_62223191 | 24 | 62223191 | 34 | C | T | C | 0.59677 | T | 0.40323 | 13 | 0.41935 |
| 18504 | S24_62223346 | 24 | 62223346 | 155 | C | T | C | 0.62903 | T | 0.37097 | 15 | 0.48387 |
| 18505 | S25_36353 | 25 | 36353 | 0 | A | C | C | 0.82258 | A | 0.17742 | 7 | 0.22581 |
| 18506 | S25_36446 | 25 | 36446 | 93 | G | A | G | 0.91935 | A | 0.08065 | 3 | 0.09677 |
| 18507 | S25_82419 | 25 | 82419 | 45973 | G | A | A | 0.59677 | G | 0.40323 | 17 | 0.54839 |
| 18508 | S25_82447 | 25 | 82447 | 28 | T | C | C | 0.59677 | T | 0.40323 | 17 | 0.54839 |
| 18509 | S25_2137562 | 25 | 2137562 | 2055115 | A | G | A | 0.90323 | G | 0.09677 | 4 | 0.12903 |
| 18510 | S25_2223383 | 25 | 2223383 | 85821 | C | T | T | 0.6129 | C | 0.3871 | 14 | 0.45161 |
| 18511 | S25_2475456 | 25 | 2475456 | 252073 | G | A | A | 0.59677 | G | 0.40323 | 19 | 0.6129 |
| 18512 | S25_2528303 | 25 | 2528303 | 52847 | G | A | A | 0.5 | G | 0.5 | 19 | 0.6129 |
| 18513 | S25_2528396 | 25 | 2528396 | 93 | A | C | C | 0.53226 | A | 0.46774 | 19 | 0.6129 |
| 18514 | S25_2764110 | 25 | 2764110 | 235714 | T | C | T | 0.8871 | C | 0.1129 | 5 | 0.16129 |
| 18515 | S25_2764169 | 25 | 2764169 | 59 | G | T | G | 0.90323 | T | 0.09677 | 4 | 0.12903 |
| 18516 | S25_2764209 | 25 | 2764209 | 40 | C | G | C | 0.74194 | G | 0.25806 | 12 | 0.3871 |
| 18517 | S25_2834117 | 25 | 2834117 | 69908 | G | A | G | 0.77419 | A | 0.22581 | 14 | 0.45161 |
| 18518 | S25_2850845 | 25 | 2850845 | 16728 | G | A | A | 0.80645 | G | 0.19355 | 8 | 0.25806 |
| 18519 | S25_3351744 | 25 | 3351744 | 500899 | C | T | C | 0.91935 | T | 0.08065 | 5 | 0.16129 |
| 18520 | S25_4598424 | 25 | 4598424 | 1246680 | G | A | G | 0.93548 | A | 0.06452 | 4 | 0.12903 |

| Site number | SNP name | Chromosome | Physical position | Physical distance from the previous SNP | Reference allele | Alternative allele | Major allele | Major allele frequency | Minor allele | Minor allele frequency | Number heterozygous | Heterozygosity |
|---|---|---|---|---|---|---|---|---|---|---|---|---|
| 18521 | S25_4681137 | 25 | 4681137 | 82713 | G | C | G | 0.90323 | C | 0.09677 | 6 | 0.19355 |
| 18522 | S25_4681228 | 25 | 4681228 | 91 | C | T | C | 0.90323 | T | 0.09677 | 6 | 0.19355 |
| 18523 | S25_5094422 | 25 | 5094422 | 413194 | A | G | A | 0.83871 | G | 0.16129 | 10 | 0.32258 |
| 18524 | S25_5094432 | 25 | 5094432 | 10 | A | T | A | 0.8871 | T | 0.1129 | 7 | 0.22581 |
| 18525 | S25_5244738 | 25 | 5244738 | 150306 | G | A | G | 0.58065 | A | 0.41935 | 16 | 0.51613 |
| 18526 | S25_5256388 | 25 | 5256388 | 11650 | T | C | T | 0.90323 | C | 0.09677 | 4 | 0.12903 |
| 18527 | S25_5256408 | 25 | 5256408 | 20 | G | A | G | 0.90323 | A | 0.09677 | 4 | 0.12903 |
| 18528 | S25_5256449 | 25 | 5256449 | 41 | C | T | C | 0.90323 | T | 0.09677 | 4 | 0.12903 |
| 18529 | S25_5256486 | 25 | 5256486 | 37 | G | T | G | 0.90323 | T | 0.09677 | 4 | 0.12903 |
| 18530 | S25_5256517 | 25 | 5256517 | 31 | G | C | G | 0.93548 | C | 0.06452 | 2 | 0.06452 |
| 18531 | S25_5387034 | 25 | 5387034 | 130517 | C | T | C | 0.90323 | T | 0.09677 | 6 | 0.19355 |
| 18532 | S25_5873184 | 25 | 5873184 | 486150 | G | A | G | 0.87097 | A | 0.12903 | 8 | 0.25806 |
| 18533 | S25_5873258 | 25 | 5873258 | 74 | A | G | A | 0.83871 | G | 0.16129 | 8 | 0.25806 |
| 18534 | S25_5873278 | 25 | 5873278 | 20 | A | G | G | 0.6129 | A | 0.3871 | 12 | 0.3871 |
| 18535 | S25_5873319 | 25 | 5873319 | 41 | A | G | G | 0.6129 | A | 0.3871 | 16 | 0.51613 |
| 18536 | S25_5873343 | 25 | 5873343 | 24 | A | G | G | 0.70968 | A | 0.29032 | 10 | 0.32258 |
| 18537 | S25_6290592 | 25 | 6290592 | 417249 | G | A | G | 0.56452 | A | 0.43548 | 11 | 0.35484 |
| 18538 | S25_6312228 | 25 | 6312228 | 21636 | T | C | T | 0.82258 | C | 0.17742 | 7 | 0.22581 |
| 18539 | S25_6312234 | 25 | 6312234 | 6 | G | A | G | 0.95161 | A | 0.04839 | 3 | 0.09677 |
| 18540 | S25_6312374 | 25 | 6312374 | 140 | C | T | C | 0.95161 | T | 0.04839 | 3 | 0.09677 |
| 18541 | S25_6364777 | 25 | 6364777 | 52403 | A | G | A | 0.91935 | G | 0.08065 | 5 | 0.16129 |
| 18542 | S25_6364871 | 25 | 6364871 | 94 | G | T | G | 0.95161 | T | 0.04839 | 3 | 0.09677 |
| 18543 | S25_6364934 | 25 | 6364934 | 63 | C | T | C | 0.95161 | T | 0.04839 | 3 | 0.09677 |
| 18544 | S25_6754990 | 25 | 6754990 | 390056 | T | C | C | 0.59677 | T | 0.40323 | 11 | 0.35484 |
| 18545 | S25_6944799 | 25 | 6944799 | 189809 | T | A | T | 0.90323 | A | 0.09677 | 6 | 0.19355 |
| 18546 | S25_6944929 | 25 | 6944929 | 130 | G | A | G | 0.8871 | A | 0.1129 | 5 | 0.16129 |
| 18547 | S25_7359404 | 25 | 7359404 | 414475 | G | A | G | 0.74194 | A | 0.25806 | 14 | 0.45161 |
| 18548 | S25_7359417 | 25 | 7359417 | 13 | C | T | C | 0.95161 | T | 0.04839 | 3 | 0.09677 |
| 18549 | S25_7359456 | 25 | 7359456 | 39 | G | T | G | 0.5 | T | 0.5 | 17 | 0.54839 |
| 18550 | S25_7359475 | 25 | 7359475 | 19 | C | T | C | 0.90323 | T | 0.09677 | 6 | 0.19355 |
| 18551 | S25_7393021 | 25 | 7393021 | 33546 | C | T | T | 0.85484 | C | 0.14516 | 9 | 0.29032 |
| 18552 | S25_7393124 | 25 | 7393124 | 103 | C | T | C | 0.80645 | T | 0.19355 | 12 | 0.3871 |
| 18553 | S25_7689655 | 25 | 7689655 | 296531 | C | A | C | 0.70968 | A | 0.29032 | 14 | 0.45161 |
| 18554 | S25_7689714 | 25 | 7689714 | 59 | C | G | C | 0.91935 | G | 0.08065 | 5 | 0.16129 |
| 18555 | S25_7689721 | 25 | 7689721 | 7 | A | G | A | 0.87097 | G | 0.12903 | 6 | 0.19355 |
| 18556 | S25_7689839 | 25 | 7689839 | 118 | G | A | G | 0.8871 | A | 0.1129 | 5 | 0.16129 |
| 18557 | S25_8003854 | 25 | 8003854 | 314015 | T | C | T | 0.70968 | C | 0.29032 | 14 | 0.45161 |
| 18558 | S25_8003922 | 25 | 8003922 | 68 | A | G | G | 0.87097 | A | 0.12903 | 6 | 0.19355 |
| 18559 | S25_8003946 | 25 | 8003946 | 24 | C | T | C | 0.69355 | T | 0.30645 | 15 | 0.48387 |
| 18560 | S25_8003958 | 25 | 8003958 | 12 | G | A | G | 0.70968 | A | 0.29032 | 14 | 0.45161 |
| 18561 | S25_8008797 | 25 | 8008797 | 4839 | C | T | C | 0.70968 | T | 0.29032 | 10 | 0.32258 |
| 18562 | S25_8008839 | 25 | 8008839 | 42 | C | A | C | 0.56452 | A | 0.43548 | 15 | 0.48387 |
| 18563 | S25_8008999 | 25 | 8008999 | 160 | T | C | T | 0.56452 | C | 0.43548 | 15 | 0.48387 |
| 18564 | S25_8067613 | 25 | 8067613 | 58614 | C | T | C | 0.93548 | T | 0.06452 | 2 | 0.06452 |
| 18565 | S25_8187226 | 25 | 8187226 | 119613 | G | A | G | 0.90323 | A | 0.09677 | 6 | 0.19355 |
| 18566 | S25_8187232 | 25 | 8187232 | 6 | A | G | A | 0.90323 | G | 0.09677 | 6 | 0.19355 |
| 18567 | S25_8187247 | 25 | 8187247 | 15 | T | C | T | 0.93548 | C | 0.06452 | 4 | 0.12903 |
| 18568 | S25_8187323 | 25 | 8187323 | 76 | T | C | C | 0.51613 | T | 0.48387 | 20 | 0.64516 |
| 18569 | S25_8254242 | 25 | 8254242 | 66919 | C | T | C | 0.80645 | T | 0.19355 | 10 | 0.32258 |
| 18570 | S25_8542555 | 25 | 8542555 | 288313 | T | C | T | 0.90323 | C | 0.09677 | 6 | 0.19355 |
| 18571 | S25_8542625 | 25 | 8542625 | 70 | A | G | A | 0.8871 | G | 0.1129 | 5 | 0.16129 |
| 18572 | S25_8542647 | 25 | 8542647 | 22 | G | T | G | 0.90323 | T | 0.09677 | 6 | 0.19355 |
| 18573 | S25_8615195 | 25 | 8615195 | 72548 | T | C | T | 0.91935 | C | 0.08065 | 5 | 0.16129 |
| 18574 | S25_8615324 | 25 | 8615324 | 129 | T | C | T | 0.91935 | C | 0.08065 | 5 | 0.16129 |

| Site number | SNP name | Chromosome | Physical position | Physical distance from the previous SNP | Reference allele | Alternative allele | Major allele | Major allele frequency | Minor allele | Minor allele frequency | Number heterozygous | Heterozygosity |
|---|---|---|---|---|---|---|---|---|---|---|---|---|
| 18575 | S25_8701600 | 25 | 8701600 | 86276 | C | T | C | 0.85484 | T | 0.14516 | 5 | 0.16129 |
| 18576 | S25_8845381 | 25 | 8845381 | 143781 | A | T | T | 0.83871 | A | 0.16129 | 10 | 0.32258 |
| 18577 | S25_8870442 | 25 | 8870442 | 25061 | G | A | G | 0.85484 | A | 0.14516 | 9 | 0.29032 |
| 18578 | S25_8870554 | 25 | 8870554 | 112 | G | A | G | 0.93548 | A | 0.06452 | 4 | 0.12903 |
| 18579 | S25_9188310 | 25 | 9188310 | 317756 | A | T | A | 0.77419 | T | 0.22581 | 12 | 0.3871 |
| 18580 | S25_9282820 | 25 | 9282820 | 94510 | G | A | G | 0.87097 | A | 0.12903 | 6 | 0.19355 |
| 18581 | S25_9282904 | 25 | 9282904 | 84 | A | G | G | 0.82258 | A | 0.17742 | 9 | 0.29032 |
| 18582 | S25_9353100 | 25 | 9353100 | 70196 | A | T | A | 0.91935 | T | 0.08065 | 5 | 0.16129 |
| 18583 | S25_9518891 | 25 | 9518891 | 165791 | C | G | C | 0.83871 | G | 0.16129 | 8 | 0.25806 |
| 18584 | S25_9646408 | 25 | 9646408 | 127517 | C | T | C | 0.75806 | T | 0.24194 | 9 | 0.29032 |
| 18585 | S25_9646561 | 25 | 9646561 | 153 | T | C | C | 0.54839 | T | 0.45161 | 16 | 0.51613 |
| 18586 | S25_10031401 | 25 | 10031401 | 384840 | G | T | G | 0.74194 | T | 0.25806 | 8 | 0.25806 |
| 18587 | S25_10096289 | 25 | 10096289 | 64888 | G | A | G | 0.87097 | A | 0.12903 | 8 | 0.25806 |
| 18588 | S25_10096361 | 25 | 10096361 | 72 | T | C | T | 0.8871 | C | 0.1129 | 5 | 0.16129 |
| 18589 | S25_10096389 | 25 | 10096389 | 28 | T | C | T | 0.80645 | C | 0.19355 | 8 | 0.25806 |
| 18590 | S25_10096406 | 25 | 10096406 | 17 | C | T | C | 0.8871 | T | 0.1129 | 7 | 0.22581 |
| 18591 | S25_10097449 | 25 | 10097449 | 1043 | G | A | G | 0.91935 | A | 0.08065 | 5 | 0.16129 |
| 18592 | S25_10217563 | 25 | 10217563 | 120114 | A | C | A | 0.95161 | C | 0.04839 | 3 | 0.09677 |
| 18593 | S25_10217584 | 25 | 10217584 | 21 | G | A | G | 0.83871 | A | 0.16129 | 8 | 0.25806 |
| 18594 | S25_10217643 | 25 | 10217643 | 59 | C | A | C | 0.95161 | A | 0.04839 | 3 | 0.09677 |
| 18595 | S25_10217771 | 25 | 10217771 | 128 | G | A | G | 0.72581 | A | 0.27419 | 11 | 0.35484 |
| 18596 | S25_10245223 | 25 | 10245223 | 27452 | A | G | G | 0.85484 | A | 0.14516 | 7 | 0.22581 |
| 18597 | S25_10911688 | 25 | 10911688 | 666465 | A | G | G | 0.74194 | A | 0.25806 | 10 | 0.32258 |
| 18598 | S25_10946309 | 25 | 10946309 | 34621 | G | A | G | 0.72581 | A | 0.27419 | 15 | 0.48387 |
| 18599 | S25_11051298 | 25 | 11051298 | 104989 | A | G | A | 0.90323 | G | 0.09677 | 6 | 0.19355 |
| 18600 | S25_11108217 | 25 | 11108217 | 56919 | T | G | G | 0.66129 | T | 0.33871 | 15 | 0.48387 |
| 18601 | S25_11108224 | 25 | 11108224 | 7 | C | T | T | 0.66129 | C | 0.33871 | 15 | 0.48387 |
| 18602 | S25_11108316 | 25 | 11108316 | 92 | A | G | G | 0.58065 | A | 0.41935 | 18 | 0.58065 |
| 18603 | S25_11108353 | 25 | 11108353 | 37 | A | G | G | 0.51613 | A | 0.48387 | 16 | 0.51613 |
| 18604 | S25_11115124 | 25 | 11115124 | 6771 | T | A | T | 0.53226 | A | 0.46774 | 17 | 0.54839 |
| 18605 | S25_11115142 | 25 | 11115142 | 18 | T | C | T | 0.8871 | C | 0.1129 | 7 | 0.22581 |
| 18606 | S25_11115173 | 25 | 11115173 | 31 | A | G | A | 0.85484 | G | 0.14516 | 7 | 0.22581 |
| 18607 | S25_11115181 | 25 | 11115181 | 8 | A | T | A | 0.93548 | T | 0.06452 | 4 | 0.12903 |
| 18608 | S25_11115276 | 25 | 11115276 | 95 | G | A | G | 0.6129 | A | 0.3871 | 14 | 0.45161 |
| 18609 | S25_11115285 | 25 | 11115285 | 9 | T | G | T | 0.82258 | G | 0.17742 | 7 | 0.22581 |
| 18610 | S25_11357980 | 25 | 11357980 | 242695 | T | G | T | 0.93548 | G | 0.06452 | 4 | 0.12903 |
| 18611 | S25_11358024 | 25 | 11358024 | 44 | C | T | C | 0.95161 | T | 0.04839 | 3 | 0.09677 |
| 18612 | S25_11358025 | 25 | 11358025 | 1 | A | G | G | 0.87097 | A | 0.12903 | 6 | 0.19355 |
| 18613 | S25_11358103 | 25 | 11358103 | 78 | C | T | C | 0.83871 | T | 0.16129 | 6 | 0.19355 |
| 18614 | S25_11358199 | 25 | 11358199 | 96 | A | C | C | 0.66129 | A | 0.33871 | 13 | 0.41935 |
| 18615 | S25_11366568 | 25 | 11366568 | 8369 | G | A | G | 0.90323 | A | 0.09677 | 6 | 0.19355 |
| 18616 | S25_11366570 | 25 | 11366570 | 2 | G | A | G | 0.59677 | A | 0.40323 | 13 | 0.41935 |
| 18617 | S25_11366576 | 25 | 11366576 | 6 | C | T | C | 0.90323 | T | 0.09677 | 4 | 0.12903 |
| 18618 | S25_11366624 | 25 | 11366624 | 48 | G | A | G | 0.90323 | A | 0.09677 | 4 | 0.12903 |
| 18619 | S25_11377196 | 25 | 11377196 | 10572 | C | T | T | 0.85484 | C | 0.14516 | 7 | 0.22581 |
| 18620 | S25_11377220 | 25 | 11377220 | 24 | C | G | C | 0.80645 | G | 0.19355 | 6 | 0.19355 |
| 18621 | S25_11377266 | 25 | 11377266 | 46 | G | C | C | 0.58065 | G | 0.41935 | 16 | 0.51613 |
| 18622 | S25_11377417 | 25 | 11377417 | 151 | G | A | A | 0.56452 | G | 0.43548 | 15 | 0.48387 |
| 18623 | S25_11491979 | 25 | 11491979 | 114562 | T | C | C | 0.54839 | T | 0.45161 | 16 | 0.51613 |
| 18624 | S25_11617807 | 25 | 11617807 | 125828 | T | A | T | 0.67742 | A | 0.32258 | 12 | 0.3871 |
| 18625 | S25_12055467 | 25 | 12055467 | 437660 | G | A | G | 0.90323 | A | 0.09677 | 6 | 0.19355 |
| 18626 | S25_12055588 | 25 | 12055588 | 121 | A | G | A | 0.90323 | G | 0.09677 | 6 | 0.19355 |
| 18627 | S25_12055603 | 25 | 12055603 | 15 | T | C | T | 0.90323 | C | 0.09677 | 6 | 0.19355 |
| 18628 | S25_12055614 | 25 | 12055614 | 11 | T | C | T | 0.90323 | C | 0.09677 | 6 | 0.19355 |

| Site number | SNP name | Chromosome | Physical position | Physical distance from the previous SNP | Reference allele | Alternative allele | Major allele | Major allele frequency | Minor allele | Minor allele frequency | Number heterozygous | Heterozygosity |
|---|---|---|---|---|---|---|---|---|---|---|---|---|
| 18629 | S25_12127866 | 25 | 12127866 | 72252 | C | T | C | 0.93548 | T | 0.06452 | 4 | 0.12903 |
| 18630 | S25_12381211 | 25 | 12381211 | 253345 | T | C | T | 0.67742 | C | 0.32258 | 18 | 0.58065 |
| 18631 | S25_12381215 | 25 | 12381215 | 4 | C | T | C | 0.8871 | T | 0.1129 | 7 | 0.22581 |
| 18632 | S25_12381223 | 25 | 12381223 | 8 | C | T | C | 0.95161 | T | 0.04839 | 3 | 0.09677 |
| 18633 | S25_12381401 | 25 | 12381401 | 178 | T | A | T | 0.62903 | A | 0.37097 | 19 | 0.6129 |
| 18634 | S25_12392231 | 25 | 12392231 | 10830 | A | G | G | 0.79032 | A | 0.20968 | 13 | 0.41935 |
| 18635 | S25_12392260 | 25 | 12392260 | 29 | T | G | G | 0.79032 | T | 0.20968 | 13 | 0.41935 |
| 18636 | S25_12392287 | 25 | 12392287 | 27 | G | A | A | 0.79032 | G | 0.20968 | 13 | 0.41935 |
| 18637 | S25_12487109 | 25 | 12487109 | 94822 | A | C | A | 0.8871 | C | 0.1129 | 7 | 0.22581 |
| 18638 | S25_12487173 | 25 | 12487173 | 64 | G | A | G | 0.90323 | A | 0.09677 | 6 | 0.19355 |
| 18639 | S25_12487222 | 25 | 12487222 | 49 | C | A | A | 0.82258 | C | 0.17742 | 9 | 0.29032 |
| 18640 | S25_12515029 | 25 | 12515029 | 27807 | T | C | T | 0.91935 | C | 0.08065 | 5 | 0.16129 |
| 18641 | S25_12515051 | 25 | 12515051 | 22 | A | G | G | 0.8871 | A | 0.1129 | 5 | 0.16129 |
| 18642 | S25_12515091 | 25 | 12515091 | 40 | C | T | C | 0.93548 | T | 0.06452 | 4 | 0.12903 |
| 18643 | S25_12609209 | 25 | 12609209 | 94118 | C | T | C | 0.90323 | T | 0.09677 | 6 | 0.19355 |
| 18644 | S25_12609329 | 25 | 12609329 | 120 | T | C | T | 0.67742 | C | 0.32258 | 10 | 0.32258 |
| 18645 | S25_13435393 | 25 | 13435393 | 826064 | C | T | C | 0.85484 | T | 0.14516 | 7 | 0.22581 |
| 18646 | S25_14049631 | 25 | 14049631 | 614238 | C | T | C | 0.91935 | T | 0.08065 | 5 | 0.16129 |
| 18647 | S25_14192562 | 25 | 14192562 | 142931 | G | A | G | 0.70968 | A | 0.29032 | 12 | 0.3871 |
| 18648 | S25_14192703 | 25 | 14192703 | 141 | C | A | C | 0.75806 | A | 0.24194 | 9 | 0.29032 |
| 18649 | S25_14195140 | 25 | 14195140 | 2437 | C | T | C | 0.75806 | T | 0.24194 | 9 | 0.29032 |
| 18650 | S25_14294740 | 25 | 14294740 | 99600 | C | T | C | 0.93548 | T | 0.06452 | 4 | 0.12903 |
| 18651 | S25_14294929 | 25 | 14294929 | 189 | G | A | G | 0.93548 | A | 0.06452 | 4 | 0.12903 |
| 18652 | S25_14294954 | 25 | 14294954 | 25 | G | A | G | 0.93548 | A | 0.06452 | 4 | 0.12903 |
| 18653 | S25_14481842 | 25 | 14481842 | 186888 | C | T | C | 0.91935 | T | 0.08065 | 5 | 0.16129 |
| 18654 | S25_14481868 | 25 | 14481868 | 26 | C | T | C | 0.72581 | T | 0.27419 | 11 | 0.35484 |
| 18655 | S25_14481983 | 25 | 14481983 | 115 | C | T | C | 0.67742 | T | 0.32258 | 14 | 0.45161 |
| 18656 | S25_14587224 | 25 | 14587224 | 105241 | T | C | T | 0.90323 | C | 0.09677 | 4 | 0.12903 |
| 18657 | S25_14668108 | 25 | 14668108 | 80884 | T | C | C | 0.58065 | T | 0.41935 | 20 | 0.64516 |
| 18658 | S25_14668238 | 25 | 14668238 | 130 | G | A | G | 0.87097 | A | 0.12903 | 6 | 0.19355 |
| 18659 | S25_14668268 | 25 | 14668268 | 30 | A | G | A | 0.87097 | G | 0.12903 | 6 | 0.19355 |
| 18660 | S25_14671232 | 25 | 14671232 | 2964 | A | C | A | 0.82258 | C | 0.17742 | 9 | 0.29032 |
| 18661 | S25_14671391 | 25 | 14671391 | 159 | A | T | A | 0.93548 | T | 0.06452 | 4 | 0.12903 |
| 18662 | S25_14728706 | 25 | 14728706 | 57315 | G | T | G | 0.95161 | T | 0.04839 | 3 | 0.09677 |
| 18663 | S25_14763931 | 25 | 14763931 | 35225 | G | T | G | 0.69355 | T | 0.30645 | 17 | 0.54839 |
| 18664 | S25_14834531 | 25 | 14834531 | 70600 | C | A | C | 0.85484 | A | 0.14516 | 7 | 0.22581 |
| 18665 | S25_15037045 | 25 | 15037045 | 202514 | C | T | C | 0.90323 | T | 0.09677 | 6 | 0.19355 |
| 18666 | S25_15037209 | 25 | 15037209 | 164 | T | C | T | 0.67742 | C | 0.32258 | 10 | 0.32258 |
| 18667 | S25_15217187 | 25 | 15217187 | 179978 | T | C | T | 0.8871 | C | 0.1129 | 5 | 0.16129 |
| 18668 | S25_15217205 | 25 | 15217205 | 18 | A | G | A | 0.8871 | G | 0.1129 | 5 | 0.16129 |
| 18669 | S25_15217348 | 25 | 15217348 | 143 | A | C | A | 0.77419 | C | 0.22581 | 8 | 0.25806 |
| 18670 | S25_15217349 | 25 | 15217349 | 1 | G | A | G | 0.8871 | A | 0.1129 | 5 | 0.16129 |
| 18671 | S25_15238468 | 25 | 15238468 | 21119 | C | T | C | 0.82258 | T | 0.17742 | 7 | 0.22581 |
| 18672 | S25_15238469 | 25 | 15238469 | 1 | A | G | A | 0.58065 | G | 0.41935 | 16 | 0.51613 |
| 18673 | S25_15238758 | 25 | 15238758 | 289 | T | C | T | 0.83871 | C | 0.16129 | 10 | 0.32258 |
| 18674 | S25_15238827 | 25 | 15238827 | 69 | C | T | T | 0.56452 | C | 0.43548 | 13 | 0.41935 |
| 18675 | S25_15238874 | 25 | 15238874 | 47 | T | A | T | 0.83871 | A | 0.16129 | 6 | 0.19355 |
| 18676 | S25_15473651 | 25 | 15473651 | 234777 | A | G | A | 0.64516 | G | 0.35484 | 14 | 0.45161 |
| 18677 | S25_15607365 | 25 | 15607365 | 133714 | T | C | C | 0.75806 | T | 0.24194 | 9 | 0.29032 |
| 18678 | S25_15607538 | 25 | 15607538 | 173 | G | A | G | 0.8871 | A | 0.1129 | 5 | 0.16129 |
| 18679 | S25_15725739 | 25 | 15725739 | 118201 | G | C | C | 0.64516 | G | 0.35484 | 14 | 0.45161 |
| 18680 | S25_15820148 | 25 | 15820148 | 94409 | A | C | A | 0.56452 | C | 0.43548 | 19 | 0.6129 |
| 18681 | S25_15824902 | 25 | 15824902 | 4754 | A | G | A | 0.64516 | G | 0.35484 | 12 | 0.3871 |
| 18682 | S25_15824931 | 25 | 15824931 | 29 | G | A | G | 0.64516 | A | 0.35484 | 12 | 0.3871 |

| Site number | SNP name | Chromosome | Physical position | Physical distance from the previous SNP | Reference allele | Alternative allele | Major allele | Major allele frequency | Minor allele | Minor allele frequency | Number heterozygous | Heterozygosity |
|---|---|---|---|---|---|---|---|---|---|---|---|---|
| 18683 | S25_15824963 | 25 | 15824963 | 32 | C | T | C | 0.75806 | T | 0.24194 | 15 | 0.48387 |
| 18684 | S25_15824978 | 25 | 15824978 | 15 | C | T | C | 0.64516 | T | 0.35484 | 12 | 0.3871 |
| 18685 | S25_15825002 | 25 | 15825002 | 24 | T | C | T | 0.64516 | C | 0.35484 | 12 | 0.3871 |
| 18686 | S25_15825018 | 25 | 15825018 | 16 | G | A | G | 0.64516 | A | 0.35484 | 12 | 0.3871 |
| 18687 | S25_15825023 | 25 | 15825023 | 5 | C | T | C | 0.70968 | T | 0.29032 | 14 | 0.45161 |
| 18688 | S25_15825044 | 25 | 15825044 | 21 | T | C | T | 0.64516 | C | 0.35484 | 12 | 0.3871 |
| 18689 | S25_15825055 | 25 | 15825055 | 11 | A | G | A | 0.95161 | G | 0.04839 | 3 | 0.09677 |
| 18690 | S25_15861779 | 25 | 15861779 | 36724 | G | A | A | 0.74194 | G | 0.25806 | 14 | 0.45161 |
| 18691 | S25_16212556 | 25 | 16212556 | 350777 | G | A | G | 0.87097 | A | 0.12903 | 8 | 0.25806 |
| 18692 | S25_16285678 | 25 | 16285678 | 73122 | A | G | G | 0.91935 | A | 0.08065 | 3 | 0.09677 |
| 18693 | S25_16285846 | 25 | 16285846 | 168 | A | G | G | 0.85484 | A | 0.14516 | 5 | 0.16129 |
| 18694 | S25_17510528 | 25 | 17510528 | 1224682 | G | A | A | 0.5 | G | 0.5 | 19 | 0.6129 |
| 18695 | S25_17510661 | 25 | 17510661 | 133 | A | G | A | 0.8871 | G | 0.1129 | 5 | 0.16129 |
| 18696 | S25_17967904 | 25 | 17967904 | 457243 | A | G | A | 0.90323 | G | 0.09677 | 4 | 0.12903 |
| 18697 | S25_17967938 | 25 | 17967938 | 34 | C | T | C | 0.93548 | T | 0.06452 | 4 | 0.12903 |
| 18698 | S25_17967941 | 25 | 17967941 | 3 | G | T | G | 0.85484 | T | 0.14516 | 5 | 0.16129 |
| 18699 | S25_17968108 | 25 | 17968108 | 167 | C | T | C | 0.90323 | T | 0.09677 | 4 | 0.12903 |
| 18700 | S25_17968132 | 25 | 17968132 | 24 | G | A | G | 0.74194 | A | 0.25806 | 12 | 0.3871 |
| 18701 | S25_17996684 | 25 | 17996684 | 28552 | A | C | A | 0.85484 | C | 0.14516 | 5 | 0.16129 |
| 18702 | S25_17996713 | 25 | 17996713 | 29 | G | A | G | 0.8871 | A | 0.1129 | 5 | 0.16129 |
| 18703 | S25_18470027 | 25 | 18470027 | 473314 | G | C | G | 0.90323 | C | 0.09677 | 6 | 0.19355 |
| 18704 | S25_18470035 | 25 | 18470035 | 8 | C | T | C | 0.93548 | T | 0.06452 | 4 | 0.12903 |
| 18705 | S25_18470044 | 25 | 18470044 | 9 | G | A | G | 0.93548 | A | 0.06452 | 4 | 0.12903 |
| 18706 | S25_18819556 | 25 | 18819556 | 349512 | C | T | C | 0.8871 | T | 0.1129 | 7 | 0.22581 |
| 18707 | S25_18819573 | 25 | 18819573 | 17 | G | A | G | 0.8871 | A | 0.1129 | 7 | 0.22581 |
| 18708 | S25_18819577 | 25 | 18819577 | 4 | T | C | T | 0.56452 | C | 0.43548 | 13 | 0.41935 |
| 18709 | S25_18987410 | 25 | 18987410 | 167833 | C | T | C | 0.95161 | T | 0.04839 | 3 | 0.09677 |
| 18710 | S25_19134196 | 25 | 19134196 | 146786 | C | T | C | 0.87097 | T | 0.12903 | 6 | 0.19355 |
| 18711 | S25_19134339 | 25 | 19134339 | 143 | T | A | T | 0.87097 | A | 0.12903 | 6 | 0.19355 |
| 18712 | S25_19769656 | 25 | 19769656 | 635317 | C | T | C | 0.90323 | T | 0.09677 | 4 | 0.12903 |
| 18713 | S25_19769701 | 25 | 19769701 | 45 | C | A | C | 0.66129 | A | 0.33871 | 15 | 0.48387 |
| 18714 | S25_20096159 | 25 | 20096159 | 326458 | C | T | C | 0.80645 | T | 0.19355 | 6 | 0.19355 |
| 18715 | S25_20540933 | 25 | 20540933 | 444774 | C | T | C | 0.91935 | T | 0.08065 | 3 | 0.09677 |
| 18716 | S25_20541012 | 25 | 20541012 | 79 | A | G | G | 0.79032 | A | 0.20968 | 9 | 0.29032 |
| 18717 | S25_20542932 | 25 | 20542932 | 1920 | G | T | G | 0.70968 | T | 0.29032 | 14 | 0.45161 |
| 18718 | S25_20542933 | 25 | 20542933 | 1 | G | A | G | 0.70968 | A | 0.29032 | 14 | 0.45161 |
| 18719 | S25_20542939 | 25 | 20542939 | 6 | T | C | T | 0.85484 | C | 0.14516 | 7 | 0.22581 |
| 18720 | S25_20542947 | 25 | 20542947 | 8 | A | G | A | 0.77419 | G | 0.22581 | 12 | 0.3871 |
| 18721 | S25_20543093 | 25 | 20543093 | 146 | C | T | C | 0.8871 | T | 0.1129 | 5 | 0.16129 |
| 18722 | S25_20543094 | 25 | 20543094 | 1 | A | G | A | 0.70968 | G | 0.29032 | 14 | 0.45161 |
| 18723 | S25_20647007 | 25 | 20647007 | 103913 | G | A | G | 0.91935 | A | 0.08065 | 3 | 0.09677 |
| 18724 | S25_20669707 | 25 | 20669707 | 22700 | A | C | A | 0.67742 | C | 0.32258 | 14 | 0.45161 |
| 18725 | S25_20745048 | 25 | 20745048 | 75341 | C | T | T | 0.64516 | C | 0.35484 | 16 | 0.51613 |
| 18726 | S25_20967611 | 25 | 20967611 | 222563 | C | T | C | 0.5 | T | 0.5 | 11 | 0.35484 |
| 18727 | S25_20967783 | 25 | 20967783 | 172 | G | T | G | 0.64516 | T | 0.35484 | 10 | 0.32258 |
| 18728 | S25_20967827 | 25 | 20967827 | 44 | A | G | A | 0.79032 | G | 0.20968 | 13 | 0.41935 |
| 18729 | S25_20967854 | 25 | 20967854 | 27 | G | A | G | 0.82258 | A | 0.17742 | 11 | 0.35484 |
| 18730 | S25_21128233 | 25 | 21128233 | 160379 | G | A | G | 0.82258 | A | 0.17742 | 11 | 0.35484 |
| 18731 | S25_21128268 | 25 | 21128268 | 35 | C | A | C | 0.91935 | A | 0.08065 | 3 | 0.09677 |
| 18732 | S25_21199892 | 25 | 21199892 | 71624 | T | C | T | 0.90323 | C | 0.09677 | 6 | 0.19355 |
| 18733 | S25_21215319 | 25 | 21215319 | 15427 | T | A | T | 0.82258 | A | 0.17742 | 9 | 0.29032 |
| 18734 | S25_21422884 | 25 | 21422884 | 207565 | G | A | A | 0.77419 | G | 0.22581 | 10 | 0.32258 |
| 18735 | S25_21422947 | 25 | 21422947 | 63 | C | T | T | 0.91935 | C | 0.08065 | 5 | 0.16129 |
| 18736 | S25_21423048 | 25 | 21423048 | 101 | G | A | A | 0.77419 | G | 0.22581 | 10 | 0.32258 |

| Site number | SNP name | Chromosome | Physical position | Physical distance from the previous SNP | Reference allele | Alternative allele | Major allele | Major allele frequency | Minor allele | Minor allele frequency | Number heterozygous | Heterozygosity |
|---|---|---|---|---|---|---|---|---|---|---|---|---|
| 18737 | S25_21423060 | 25 | 21423060 | 12 | G | A | A | 0.77419 | G | 0.22581 | 10 | 0.32258 |
| 18738 | S25_21433787 | 25 | 21433787 | 10727 | C | G | C | 0.93548 | G | 0.06452 | 4 | 0.12903 |
| 18739 | S25_21433813 | 25 | 21433813 | 26 | T | C | T | 0.93548 | C | 0.06452 | 4 | 0.12903 |
| 18740 | S25_21600667 | 25 | 21600667 | 166854 | C | T | C | 0.8871 | T | 0.1129 | 7 | 0.22581 |
| 18741 | S25_21894974 | 25 | 21894974 | 294307 | G | A | G | 0.95161 | A | 0.04839 | 1 | 0.03226 |
| 18742 | S25_22188540 | 25 | 22188540 | 293566 | G | A | G | 0.56452 | A | 0.43548 | 11 | 0.35484 |
| 18743 | S25_22188597 | 25 | 22188597 | 57 | A | G | A | 0.56452 | G | 0.43548 | 11 | 0.35484 |
| 18744 | S25_22188694 | 25 | 22188694 | 97 | T | C | T | 0.91935 | C | 0.08065 | 5 | 0.16129 |
| 18745 | S25_22197137 | 25 | 22197137 | 8443 | G | T | G | 0.80645 | T | 0.19355 | 10 | 0.32258 |
| 18746 | S25_22197315 | 25 | 22197315 | 178 | T | C | T | 0.95161 | C | 0.04839 | 1 | 0.03226 |
| 18747 | S25_22430754 | 25 | 22430754 | 233439 | C | T | C | 0.80645 | T | 0.19355 | 8 | 0.25806 |
| 18748 | S25_22430803 | 25 | 22430803 | 49 | T | A | T | 0.69355 | A | 0.30645 | 11 | 0.35484 |
| 18749 | S25_22562227 | 25 | 22562227 | 131424 | G | A | G | 0.67742 | A | 0.32258 | 12 | 0.3871 |
| 18750 | S25_23063230 | 25 | 23063230 | 501003 | A | G | G | 0.83871 | A | 0.16129 | 8 | 0.25806 |
| 18751 | S25_23063257 | 25 | 23063257 | 27 | C | T | C | 0.87097 | T | 0.12903 | 6 | 0.19355 |
| 18752 | S25_23289386 | 25 | 23289386 | 226129 | T | C | T | 0.93548 | C | 0.06452 | 4 | 0.12903 |
| 18753 | S25_23289481 | 25 | 23289481 | 95 | C | T | C | 0.93548 | T | 0.06452 | 4 | 0.12903 |
| 18754 | S25_23425057 | 25 | 23425057 | 135576 | A | G | A | 0.85484 | G | 0.14516 | 7 | 0.22581 |
| 18755 | S25_23425146 | 25 | 23425146 | 89 | A | G | A | 0.87097 | G | 0.12903 | 6 | 0.19355 |
| 18756 | S25_23514554 | 25 | 23514554 | 89408 | G | A | G | 0.8871 | A | 0.1129 | 7 | 0.22581 |
| 18757 | S25_23514625 | 25 | 23514625 | 71 | A | C | A | 0.80645 | C | 0.19355 | 8 | 0.25806 |
| 18758 | S25_23666700 | 25 | 23666700 | 152075 | G | A | G | 0.72581 | A | 0.27419 | 13 | 0.41935 |
| 18759 | S25_23759839 | 25 | 23759839 | 93139 | C | T | C | 0.51613 | T | 0.48387 | 14 | 0.45161 |
| 18760 | S25_23759935 | 25 | 23759935 | 96 | T | C | T | 0.53226 | C | 0.46774 | 13 | 0.41935 |
| 18761 | S25_23759949 | 25 | 23759949 | 14 | G | A | G | 0.53226 | A | 0.46774 | 13 | 0.41935 |
| 18762 | S25_23815268 | 25 | 23815268 | 55319 | C | T | C | 0.54839 | T | 0.45161 | 14 | 0.45161 |
| 18763 | S25_23815359 | 25 | 23815359 | 91 | A | G | A | 0.56452 | G | 0.43548 | 15 | 0.48387 |
| 18764 | S25_23815377 | 25 | 23815377 | 18 | A | G | G | 0.69355 | A | 0.30645 | 11 | 0.35484 |
| 18765 | S25_23912103 | 25 | 23912103 | 96726 | A | G | G | 0.75806 | A | 0.24194 | 11 | 0.35484 |
| 18766 | S25_23979251 | 25 | 23979251 | 67148 | C | T | C | 0.82258 | T | 0.17742 | 7 | 0.22581 |
| 18767 | S25_23979333 | 25 | 23979333 | 82 | A | G | A | 0.79032 | G | 0.20968 | 7 | 0.22581 |
| 18768 | S25_24182125 | 25 | 24182125 | 202792 | T | C | T | 0.90323 | C | 0.09677 | 6 | 0.19355 |
| 18769 | S25_24182140 | 25 | 24182140 | 15 | A | G | A | 0.85484 | G | 0.14516 | 5 | 0.16129 |
| 18770 | S25_24182166 | 25 | 24182166 | 26 | A | G | G | 0.59677 | A | 0.40323 | 13 | 0.41935 |
| 18771 | S25_24182171 | 25 | 24182171 | 5 | G | A | A | 0.79032 | G | 0.20968 | 11 | 0.35484 |
| 18772 | S25_24182190 | 25 | 24182190 | 19 | C | T | T | 0.79032 | C | 0.20968 | 11 | 0.35484 |
| 18773 | S25_24182191 | 25 | 24182191 | 1 | A | C | C | 0.79032 | A | 0.20968 | 11 | 0.35484 |
| 18774 | S25_24182264 | 25 | 24182264 | 73 | G | C | C | 0.79032 | G | 0.20968 | 11 | 0.35484 |
| 18775 | S25_24182293 | 25 | 24182293 | 29 | C | T | T | 0.79032 | C | 0.20968 | 11 | 0.35484 |
| 18776 | S25_24182308 | 25 | 24182308 | 15 | G | A | A | 0.59677 | G | 0.40323 | 13 | 0.41935 |
| 18777 | S25_24182332 | 25 | 24182332 | 24 | C | T | T | 0.64516 | C | 0.35484 | 10 | 0.32258 |
| 18778 | S25_24204094 | 25 | 24204094 | 21762 | C | T | C | 0.90323 | T | 0.09677 | 6 | 0.19355 |
| 18779 | S25_24624003 | 25 | 24624003 | 419909 | T | G | G | 0.87097 | T | 0.12903 | 8 | 0.25806 |
| 18780 | S25_24624033 | 25 | 24624033 | 30 | G | C | G | 0.79032 | C | 0.20968 | 11 | 0.35484 |
| 18781 | S25_24766680 | 25 | 24766680 | 142647 | C | T | C | 0.93548 | T | 0.06452 | 4 | 0.12903 |
| 18782 | S25_24825398 | 25 | 24825398 | 58718 | G | A | G | 0.93548 | A | 0.06452 | 4 | 0.12903 |
| 18783 | S25_24934848 | 25 | 24934848 | 109450 | A | G | A | 0.87097 | G | 0.12903 | 8 | 0.25806 |
| 18784 | S25_24934874 | 25 | 24934874 | 26 | C | T | C | 0.93548 | T | 0.06452 | 4 | 0.12903 |
| 18785 | S25_24934947 | 25 | 24934947 | 73 | C | T | T | 0.53226 | C | 0.46774 | 17 | 0.54839 |
| 18786 | S25_25026677 | 25 | 25026677 | 91730 | C | T | T | 0.80645 | C | 0.19355 | 10 | 0.32258 |
| 18787 | S25_25026736 | 25 | 25026736 | 59 | C | A | A | 0.54839 | C | 0.45161 | 12 | 0.3871 |
| 18788 | S25_25049838 | 25 | 25049838 | 23102 | G | C | C | 0.58065 | G | 0.41935 | 12 | 0.3871 |
| 18789 | S25_25099798 | 25 | 25099798 | 49960 | G | C | G | 0.51613 | C | 0.48387 | 16 | 0.51613 |
| 18790 | S25_25099987 | 25 | 25099987 | 189 | C | G | C | 0.95161 | G | 0.04839 | 1 | 0.03226 |

| Site number | SNP name | Chromosome | Physical position | Physical distance from the previous SNP | Reference allele | Alternative allele | Major allele | Major allele frequency | Minor allele | Minor allele frequency | Number heterozygous | Heterozygosity |
|---|---|---|---|---|---|---|---|---|---|---|---|---|
| 18791 | S25_25104236 | 25 | 25104236 | 4249 | T | C | T | 0.79032 | C | 0.20968 | 9 | 0.29032 |
| 18792 | S25_25104243 | 25 | 25104243 | 7 | C | A | A | 0.66129 | C | 0.33871 | 13 | 0.41935 |
| 18793 | S25_25104356 | 25 | 25104356 | 113 | A | G | A | 0.80645 | G | 0.19355 | 6 | 0.19355 |
| 18794 | S25_25108465 | 25 | 25108465 | 4109 | C | T | C | 0.77419 | T | 0.22581 | 8 | 0.25806 |
| 18795 | S25_25201273 | 25 | 25201273 | 92808 | G | A | G | 0.90323 | A | 0.09677 | 6 | 0.19355 |
| 18796 | S25_25201341 | 25 | 25201341 | 68 | G | T | G | 0.79032 | T | 0.20968 | 11 | 0.35484 |
| 18797 | S25_25239927 | 25 | 25239927 | 38586 | G | A | G | 0.67742 | A | 0.32258 | 14 | 0.45161 |
| 18798 | S25_25239938 | 25 | 25239938 | 11 | G | T | G | 0.67742 | T | 0.32258 | 14 | 0.45161 |
| 18799 | S25_25240062 | 25 | 25240062 | 124 | C | T | C | 0.91935 | T | 0.08065 | 5 | 0.16129 |
| 18800 | S25_25240157 | 25 | 25240157 | 95 | C | A | C | 0.56452 | A | 0.43548 | 21 | 0.67742 |
| 18801 | S25_25359666 | 25 | 25359666 | 119509 | G | A | G | 0.93548 | A | 0.06452 | 2 | 0.06452 |
| 18802 | S25_25359711 | 25 | 25359711 | 45 | G | C | G | 0.91935 | C | 0.08065 | 5 | 0.16129 |
| 18803 | S25_25757172 | 25 | 25757172 | 397461 | A | T | A | 0.87097 | T | 0.12903 | 8 | 0.25806 |
| 18804 | S25_26060139 | 25 | 26060139 | 302967 | C | A | C | 0.74194 | A | 0.25806 | 12 | 0.3871 |
| 18805 | S25_26538351 | 25 | 26538351 | 478212 | G | C | G | 0.90323 | C | 0.09677 | 6 | 0.19355 |
| 18806 | S25_26538378 | 25 | 26538378 | 27 | A | G | A | 0.91935 | G | 0.08065 | 3 | 0.09677 |
| 18807 | S25_26538457 | 25 | 26538457 | 79 | C | T | C | 0.87097 | T | 0.12903 | 8 | 0.25806 |
| 18808 | S25_26538474 | 25 | 26538474 | 17 | C | G | C | 0.69355 | G | 0.30645 | 11 | 0.35484 |
| 18809 | S25_26638376 | 25 | 26638376 | 99902 | G | A | G | 0.75806 | A | 0.24194 | 9 | 0.29032 |
| 18810 | S25_26654082 | 25 | 26654082 | 15706 | C | G | G | 0.8871 | C | 0.1129 | 7 | 0.22581 |
| 18811 | S25_26866848 | 25 | 26866848 | 212766 | C | T | C | 0.8871 | T | 0.1129 | 7 | 0.22581 |
| 18812 | S25_27180801 | 25 | 27180801 | 313953 | G | A | G | 0.93548 | A | 0.06452 | 4 | 0.12903 |
| 18813 | S25_27181002 | 25 | 27181002 | 201 | C | A | C | 0.56452 | A | 0.43548 | 15 | 0.48387 |
| 18814 | S25_27228056 | 25 | 27228056 | 47054 | A | G | A | 0.75806 | G | 0.24194 | 13 | 0.41935 |
| 18815 | S25_27228059 | 25 | 27228059 | 3 | C | T | C | 0.91935 | T | 0.08065 | 3 | 0.09677 |
| 18816 | S25_27234618 | 25 | 27234618 | 6559 | G | T | G | 0.58065 | T | 0.41935 | 12 | 0.3871 |
| 18817 | S25_27234624 | 25 | 27234624 | 6 | G | C | G | 0.91935 | C | 0.08065 | 5 | 0.16129 |
| 18818 | S25_27234630 | 25 | 27234630 | 6 | G | A | G | 0.58065 | A | 0.41935 | 12 | 0.3871 |
| 18819 | S25_27234723 | 25 | 27234723 | 93 | C | T | C | 0.95161 | T | 0.04839 | 3 | 0.09677 |
| 18820 | S25_27234730 | 25 | 27234730 | 7 | C | T | C | 0.79032 | T | 0.20968 | 9 | 0.29032 |
| 18821 | S25_27668157 | 25 | 27668157 | 433427 | T | C | T | 0.95161 | C | 0.04839 | 3 | 0.09677 |
| 18822 | S25_27668163 | 25 | 27668163 | 6 | T | C | C | 0.51613 | T | 0.48387 | 14 | 0.45161 |
| 18823 | S25_27910032 | 25 | 27910032 | 241869 | A | G | A | 0.53226 | G | 0.46774 | 13 | 0.41935 |
| 18824 | S25_27910098 | 25 | 27910098 | 66 | C | T | C | 0.85484 | T | 0.14516 | 7 | 0.22581 |
| 18825 | S25_28133785 | 25 | 28133785 | 223687 | C | T | C | 0.91935 | T | 0.08065 | 5 | 0.16129 |
| 18826 | S25_28133899 | 25 | 28133899 | 114 | G | A | A | 0.6129 | G | 0.3871 | 16 | 0.51613 |
| 18827 | S25_28288833 | 25 | 28288833 | 154934 | C | T | C | 0.59677 | T | 0.40323 | 15 | 0.48387 |
| 18828 | S25_28288834 | 25 | 28288834 | 1 | G | A | G | 0.93548 | A | 0.06452 | 4 | 0.12903 |
| 18829 | S25_28288850 | 25 | 28288850 | 16 | C | T | C | 0.87097 | T | 0.12903 | 8 | 0.25806 |
| 18830 | S25_28496150 | 25 | 28496150 | 207300 | G | A | G | 0.66129 | A | 0.33871 | 11 | 0.35484 |
| 18831 | S25_28548595 | 25 | 28548595 | 52445 | G | A | G | 0.95161 | A | 0.04839 | 3 | 0.09677 |
| 18832 | S25_28548633 | 25 | 28548633 | 38 | G | T | G | 0.8871 | T | 0.1129 | 7 | 0.22581 |
| 18833 | S25_28575070 | 25 | 28575070 | 26437 | T | C | T | 0.58065 | C | 0.41935 | 16 | 0.51613 |
| 18834 | S25_28575153 | 25 | 28575153 | 83 | T | C | C | 0.72581 | T | 0.27419 | 15 | 0.48387 |
| 18835 | S25_28575298 | 25 | 28575298 | 145 | A | G | A | 0.79032 | G | 0.20968 | 13 | 0.41935 |
| 18836 | S25_28575307 | 25 | 28575307 | 9 | T | C | C | 0.83871 | T | 0.16129 | 10 | 0.32258 |
| 18837 | S25_29133718 | 25 | 29133718 | 558411 | A | C | C | 0.93548 | A | 0.06452 | 4 | 0.12903 |
| 18838 | S25_29133724 | 25 | 29133724 | 6 | G | A | G | 0.95161 | A | 0.04839 | 3 | 0.09677 |
| 18839 | S25_29164481 | 25 | 29164481 | 30757 | G | A | G | 0.6129 | A | 0.3871 | 14 | 0.45161 |
| 18840 | S25_29164490 | 25 | 29164490 | 9 | A | G | A | 0.6129 | G | 0.3871 | 14 | 0.45161 |
| 18841 | S25_29164657 | 25 | 29164657 | 167 | G | A | G | 0.77419 | A | 0.22581 | 10 | 0.32258 |
| 18842 | S25_29301461 | 25 | 29301461 | 136804 | T | C | T | 0.90323 | C | 0.09677 | 6 | 0.19355 |
| 18843 | S25_29301481 | 25 | 29301481 | 20 | A | G | A | 0.95161 | G | 0.04839 | 3 | 0.09677 |
| 18844 | S25_29301487 | 25 | 29301487 | 6 | G | A | G | 0.95161 | A | 0.04839 | 3 | 0.09677 |

| Site number | SNP name | Chromosome | Physical position | Physical distance from the previous SNP | Reference allele | Alternative allele | Major allele | Major allele frequency | Minor allele | Minor allele frequency | Number heterozygous | Heterozygosity |
|---|---|---|---|---|---|---|---|---|---|---|---|---|
| 18845 | S25_29301515 | 25 | 29301515 | 28 | G | C | G | 0.8871 | C | 0.1129 | 7 | 0.22581 |
| 18846 | S25_29301560 | 25 | 29301560 | 45 | G | A | G | 0.95161 | A | 0.04839 | 3 | 0.09677 |
| 18847 | S25_29301587 | 25 | 29301587 | 27 | C | T | C | 0.95161 | T | 0.04839 | 3 | 0.09677 |
| 18848 | S25_29345376 | 25 | 29345376 | 43789 | G | C | C | 0.79032 | G | 0.20968 | 13 | 0.41935 |
| 18849 | S25_29345568 | 25 | 29345568 | 192 | T | C | T | 0.80645 | C | 0.19355 | 8 | 0.25806 |
| 18850 | S25_29489941 | 25 | 29489941 | 144373 | A | C | A | 0.8871 | C | 0.1129 | 5 | 0.16129 |
| 18851 | S25_29489984 | 25 | 29489984 | 43 | G | C | G | 0.72581 | C | 0.27419 | 11 | 0.35484 |
| 18852 | S25_29490019 | 25 | 29490019 | 35 | T | C | T | 0.6129 | C | 0.3871 | 14 | 0.45161 |
| 18853 | S25_29490126 | 25 | 29490126 | 107 | A | C | A | 0.87097 | C | 0.12903 | 8 | 0.25806 |
| 18854 | S25_29586047 | 25 | 29586047 | 95921 | C | T | C | 0.80645 | T | 0.19355 | 10 | 0.32258 |
| 18855 | S25_29678356 | 25 | 29678356 | 92309 | G | A | G | 0.95161 | A | 0.04839 | 3 | 0.09677 |
| 18856 | S25_29778409 | 25 | 29778409 | 100053 | A | T | A | 0.93548 | T | 0.06452 | 4 | 0.12903 |
| 18857 | S25_29844466 | 25 | 29844466 | 66057 | C | T | C | 0.79032 | T | 0.20968 | 9 | 0.29032 |
| 18858 | S25_29979039 | 25 | 29979039 | 134573 | C | G | C | 0.83871 | G | 0.16129 | 8 | 0.25806 |
| 18859 | S25_30090114 | 25 | 30090114 | 111075 | G | T | G | 0.95161 | T | 0.04839 | 3 | 0.09677 |
| 18860 | S25_30740699 | 25 | 30740699 | 650585 | G | A | G | 0.95161 | A | 0.04839 | 3 | 0.09677 |
| 18861 | S25_30783645 | 25 | 30783645 | 42946 | G | A | A | 0.72581 | G | 0.27419 | 13 | 0.41935 |
| 18862 | S25_30853426 | 25 | 30853426 | 69781 | T | G | T | 0.93548 | G | 0.06452 | 4 | 0.12903 |
| 18863 | S25_31223743 | 25 | 31223743 | 370317 | C | T | C | 0.95161 | T | 0.04839 | 3 | 0.09677 |
| 18864 | S25_31312390 | 25 | 31312390 | 88647 | T | C | C | 0.62903 | T | 0.37097 | 19 | 0.6129 |
| 18865 | S25_31313384 | 25 | 31313384 | 994 | G | A | G | 0.69355 | A | 0.30645 | 17 | 0.54839 |
| 18866 | S25_31313402 | 25 | 31313402 | 18 | G | A | G | 0.93548 | A | 0.06452 | 4 | 0.12903 |
| 18867 | S25_31313516 | 25 | 31313516 | 114 | G | A | A | 0.91935 | G | 0.08065 | 5 | 0.16129 |
| 18868 | S25_31313517 | 25 | 31313517 | 1 | T | C | C | 0.91935 | T | 0.08065 | 5 | 0.16129 |
| 18869 | S25_31828651 | 25 | 31828651 | 515134 | G | A | G | 0.75806 | A | 0.24194 | 15 | 0.48387 |
| 18870 | S25_31828736 | 25 | 31828736 | 85 | T | C | T | 0.77419 | C | 0.22581 | 14 | 0.45161 |
| 18871 | S25_31828776 | 25 | 31828776 | 40 | A | C | A | 0.75806 | C | 0.24194 | 15 | 0.48387 |
| 18872 | S25_31828836 | 25 | 31828836 | 60 | C | T | C | 0.77419 | T | 0.22581 | 14 | 0.45161 |
| 18873 | S25_31828859 | 25 | 31828859 | 23 | T | A | T | 0.77419 | A | 0.22581 | 14 | 0.45161 |
| 18874 | S25_32166910 | 25 | 32166910 | 338051 | G | A | G | 0.87097 | A | 0.12903 | 8 | 0.25806 |
| 18875 | S25_32167091 | 25 | 32167091 | 181 | G | A | G | 0.90323 | A | 0.09677 | 6 | 0.19355 |
| 18876 | S25_32167117 | 25 | 32167117 | 26 | G | A | G | 0.87097 | A | 0.12903 | 8 | 0.25806 |
| 18877 | S25_32227166 | 25 | 32227166 | 60049 | A | G | G | 0.91935 | A | 0.08065 | 5 | 0.16129 |
| 18878 | S25_32237579 | 25 | 32237579 | 10413 | G | A | G | 0.83871 | A | 0.16129 | 10 | 0.32258 |
| 18879 | S25_32237596 | 25 | 32237596 | 17 | G | A | G | 0.72581 | A | 0.27419 | 17 | 0.54839 |
| 18880 | S25_32237597 | 25 | 32237597 | 1 | G | A | G | 0.93548 | A | 0.06452 | 4 | 0.12903 |
| 18881 | S25_32303550 | 25 | 32303550 | 65953 | T | C | T | 0.82258 | C | 0.17742 | 7 | 0.22581 |
| 18882 | S25_32432276 | 25 | 32432276 | 128726 | A | C | C | 0.95161 | A | 0.04839 | 3 | 0.09677 |
| 18883 | S25_32471721 | 25 | 32471721 | 39445 | C | A | C | 0.54839 | A | 0.45161 | 20 | 0.64516 |
| 18884 | S25_32471867 | 25 | 32471867 | 146 | A | T | A | 0.91935 | T | 0.08065 | 3 | 0.09677 |
| 18885 | S25_32533612 | 25 | 32533612 | 61745 | G | A | G | 0.90323 | A | 0.09677 | 6 | 0.19355 |
| 18886 | S25_32628289 | 25 | 32628289 | 94677 | G | A | G | 0.8871 | A | 0.1129 | 7 | 0.22581 |
| 18887 | S25_32628326 | 25 | 32628326 | 37 | T | C | T | 0.87097 | C | 0.12903 | 6 | 0.19355 |
| 18888 | S25_32795526 | 25 | 32795526 | 167200 | C | A | C | 0.93548 | A | 0.06452 | 4 | 0.12903 |
| 18889 | S25_32795691 | 25 | 32795691 | 165 | G | A | G | 0.66129 | A | 0.33871 | 13 | 0.41935 |
| 18890 | S25_32874733 | 25 | 32874733 | 79042 | C | T | C | 0.91935 | T | 0.08065 | 5 | 0.16129 |
| 18891 | S25_32874873 | 25 | 32874873 | 140 | G | A | G | 0.90323 | A | 0.09677 | 4 | 0.12903 |
| 18892 | S25_33700827 | 25 | 33700827 | 825954 | T | C | C | 0.75806 | T | 0.24194 | 15 | 0.48387 |
| 18893 | S25_33979277 | 25 | 33979277 | 278450 | C | T | C | 0.95161 | T | 0.04839 | 3 | 0.09677 |
| 18894 | S25_34062051 | 25 | 34062051 | 82774 | T | A | T | 0.87097 | A | 0.12903 | 8 | 0.25806 |
| 18895 | S25_34062211 | 25 | 34062211 | 160 | C | A | C | 0.83871 | A | 0.16129 | 10 | 0.32258 |
| 18896 | S25_34062222 | 25 | 34062222 | 11 | C | G | C | 0.83871 | G | 0.16129 | 10 | 0.32258 |
| 18897 | S25_34076446 | 25 | 34076446 | 14224 | C | T | C | 0.72581 | T | 0.27419 | 15 | 0.48387 |
| 18898 | S25_34076470 | 25 | 34076470 | 24 | T | G | T | 0.72581 | G | 0.27419 | 15 | 0.48387 |

| Site number | SNP name | Chromosome | Physical position | Physical distance from the previous SNP | Reference allele | Alternative allele | Major allele | Major allele frequency | Minor allele | Minor allele frequency | Number heterozygous | Heterozygosity |
|---|---|---|---|---|---|---|---|---|---|---|---|---|
| 18899 | S25_34076480 | 25 | 34076480 | 10 | G | T | G | 0.72581 | T | 0.27419 | 15 | 0.48387 |
| 18900 | S25_34076523 | 25 | 34076523 | 43 | C | T | C | 0.93548 | T | 0.06452 | 4 | 0.12903 |
| 18901 | S25_34076538 | 25 | 34076538 | 15 | G | C | G | 0.70968 | C | 0.29032 | 14 | 0.45161 |
| 18902 | S25_34076540 | 25 | 34076540 | 2 | G | A | G | 0.83871 | A | 0.16129 | 10 | 0.32258 |
| 18903 | S25_34076557 | 25 | 34076557 | 17 | A | G | A | 0.72581 | G | 0.27419 | 15 | 0.48387 |
| 18904 | S25_34076610 | 25 | 34076610 | 53 | C | T | C | 0.93548 | T | 0.06452 | 4 | 0.12903 |
| 18905 | S25_34076630 | 25 | 34076630 | 20 | C | T | C | 0.72581 | T | 0.27419 | 15 | 0.48387 |
| 18906 | S25_34076636 | 25 | 34076636 | 6 | G | C | G | 0.70968 | C | 0.29032 | 14 | 0.45161 |
| 18907 | S25_34076673 | 25 | 34076673 | 37 | A | G | A | 0.66129 | G | 0.33871 | 17 | 0.54839 |
| 18908 | S25_34218303 | 25 | 34218303 | 141630 | G | A | G | 0.91935 | A | 0.08065 | 3 | 0.09677 |
| 18909 | S25_34486172 | 25 | 34486172 | 267869 | A | G | G | 0.58065 | A | 0.41935 | 12 | 0.3871 |
| 18910 | S25_35290777 | 25 | 35290777 | 804605 | T | C | T | 0.87097 | C | 0.12903 | 4 | 0.12903 |
| 18911 | S25_35290881 | 25 | 35290881 | 104 | C | T | C | 0.91935 | T | 0.08065 | 5 | 0.16129 |
| 18912 | S25_35555499 | 25 | 35555499 | 264618 | T | C | T | 0.83871 | C | 0.16129 | 8 | 0.25806 |
| 18913 | S25_35555529 | 25 | 35555529 | 30 | G | C | C | 0.8871 | G | 0.1129 | 5 | 0.16129 |
| 18914 | S25_35644672 | 25 | 35644672 | 89143 | G | C | G | 0.90323 | C | 0.09677 | 6 | 0.19355 |
| 18915 | S25_35732161 | 25 | 35732161 | 87489 | C | T | C | 0.58065 | T | 0.41935 | 14 | 0.45161 |
| 18916 | S25_35836119 | 25 | 35836119 | 103958 | C | G | C | 0.56452 | G | 0.43548 | 15 | 0.48387 |
| 18917 | S25_36348312 | 25 | 36348312 | 512193 | G | A | G | 0.77419 | A | 0.22581 | 14 | 0.45161 |
| 18918 | S25_36401516 | 25 | 36401516 | 53204 | C | T | C | 0.90323 | T | 0.09677 | 4 | 0.12903 |
| 18919 | S25_36562674 | 25 | 36562674 | 161158 | C | A | C | 0.59677 | A | 0.40323 | 17 | 0.54839 |
| 18920 | S25_36562691 | 25 | 36562691 | 17 | C | T | C | 0.85484 | T | 0.14516 | 9 | 0.29032 |
| 18921 | S25_37288537 | 25 | 37288537 | 725846 | T | C | C | 0.91935 | T | 0.08065 | 5 | 0.16129 |
| 18922 | S25_37756304 | 25 | 37756304 | 467767 | C | T | C | 0.70968 | T | 0.29032 | 10 | 0.32258 |
| 18923 | S25_37832123 | 25 | 37832123 | 75819 | A | G | A | 0.95161 | G | 0.04839 | 3 | 0.09677 |
| 18924 | S25_37963844 | 25 | 37963844 | 131721 | A | C | C | 0.85484 | A | 0.14516 | 5 | 0.16129 |
| 18925 | S25_37963845 | 25 | 37963845 | 1 | G | A | G | 0.85484 | A | 0.14516 | 7 | 0.22581 |
| 18926 | S25_38020807 | 25 | 38020807 | 56962 | T | C | C | 0.83871 | T | 0.16129 | 10 | 0.32258 |
| 18927 | S25_38588022 | 25 | 38588022 | 567215 | C | T | C | 0.62903 | T | 0.37097 | 19 | 0.6129 |
| 18928 | S25_38607893 | 25 | 38607893 | 19871 | C | T | C | 0.75806 | T | 0.24194 | 9 | 0.29032 |
| 18929 | S25_38638173 | 25 | 38638173 | 30280 | A | G | G | 0.51613 | A | 0.48387 | 18 | 0.58065 |
| 18930 | S25_38831348 | 25 | 38831348 | 193175 | T | C | C | 0.54839 | T | 0.45161 | 18 | 0.58065 |
| 18931 | S25_38831400 | 25 | 38831400 | 52 | A | G | A | 0.95161 | G | 0.04839 | 3 | 0.09677 |
| 18932 | S25_38831490 | 25 | 38831490 | 90 | G | A | G | 0.95161 | A | 0.04839 | 3 | 0.09677 |
| 18933 | S25_38844453 | 25 | 38844453 | 12963 | G | C | G | 0.95161 | C | 0.04839 | 3 | 0.09677 |
| 18934 | S25_39070764 | 25 | 39070764 | 226311 | C | T | C | 0.93548 | T | 0.06452 | 2 | 0.06452 |
| 18935 | S25_39729454 | 25 | 39729454 | 658690 | T | C | T | 0.77419 | C | 0.22581 | 8 | 0.25806 |
| 18936 | S25_39729571 | 25 | 39729571 | 117 | A | G | G | 0.62903 | A | 0.37097 | 15 | 0.48387 |
| 18937 | S25_39729576 | 25 | 39729576 | 5 | A | G | A | 0.62903 | G | 0.37097 | 11 | 0.35484 |
| 18938 | S25_39729662 | 25 | 39729662 | 86 | T | G | T | 0.6129 | G | 0.3871 | 12 | 0.3871 |
| 18939 | S25_40131103 | 25 | 40131103 | 401441 | C | T | C | 0.80645 | T | 0.19355 | 12 | 0.3871 |
| 18940 | S25_40439152 | 25 | 40439152 | 308049 | G | A | A | 0.66129 | G | 0.33871 | 17 | 0.54839 |
| 18941 | S25_40439164 | 25 | 40439164 | 12 | C | T | T | 0.67742 | C | 0.32258 | 16 | 0.51613 |
| 18942 | S25_40439258 | 25 | 40439258 | 94 | G | A | A | 0.5 | G | 0.5 | 15 | 0.48387 |
| 18943 | S25_40439290 | 25 | 40439290 | 32 | C | G | G | 0.67742 | C | 0.32258 | 16 | 0.51613 |
| 18944 | S25_40508917 | 25 | 40508917 | 69627 | T | C | T | 0.93548 | C | 0.06452 | 4 | 0.12903 |
| 18945 | S25_40509003 | 25 | 40509003 | 86 | A | G | G | 0.75806 | A | 0.24194 | 9 | 0.29032 |
| 18946 | S25_40509097 | 25 | 40509097 | 94 | C | G | C | 0.93548 | G | 0.06452 | 4 | 0.12903 |
| 18947 | S25_40509123 | 25 | 40509123 | 26 | G | A | G | 0.93548 | A | 0.06452 | 4 | 0.12903 |
| 18948 | S25_40568914 | 25 | 40568914 | 59791 | G | A | G | 0.95161 | A | 0.04839 | 3 | 0.09677 |
| 18949 | S25_40569042 | 25 | 40569042 | 128 | C | T | C | 0.91935 | T | 0.08065 | 5 | 0.16129 |
| 18950 | S25_40655238 | 25 | 40655238 | 86196 | G | A | G | 0.8871 | A | 0.1129 | 7 | 0.22581 |
| 18951 | S25_40655249 | 25 | 40655249 | 11 | G | T | G | 0.79032 | T | 0.20968 | 13 | 0.41935 |
| 18952 | S25_40655272 | 25 | 40655272 | 23 | T | C | T | 0.79032 | C | 0.20968 | 13 | 0.41935 |

| Site number | SNP name | Chromosome | Physical position | Physical distance from the previous SNP | Reference allele | Alternative allele | Major allele | Major allele frequency | Minor allele | Minor allele frequency | Number heterozygous | Heterozygosity |
|---|---|---|---|---|---|---|---|---|---|---|---|---|
| 18953 | S26_44890 | 26 | 44890 | 0 | G | T | G | 0.53226 | T | 0.46774 | 13 | 0.41935 |
| 18954 | S26_44907 | 26 | 44907 | 17 | T | C | T | 0.83871 | C | 0.16129 | 8 | 0.25806 |
| 18955 | S26_44941 | 26 | 44941 | 34 | A | G | A | 0.80645 | G | 0.19355 | 10 | 0.32258 |
| 18956 | S26_44987 | 26 | 44987 | 46 | G | A | A | 0.6129 | G | 0.3871 | 12 | 0.3871 |
| 18957 | S26_45002 | 26 | 45002 | 15 | A | T | A | 0.83871 | T | 0.16129 | 8 | 0.25806 |
| 18958 | S26_433327 | 26 | 433327 | 388325 | G | C | G | 0.82258 | C | 0.17742 | 7 | 0.22581 |
| 18959 | S26_848394 | 26 | 848394 | 415067 | A | G | A | 0.75806 | G | 0.24194 | 13 | 0.41935 |
| 18960 | S26_1338722 | 26 | 1338722 | 490328 | T | A | T | 0.90323 | A | 0.09677 | 4 | 0.12903 |
| 18961 | S26_1338733 | 26 | 1338733 | 11 | C | T | C | 0.87097 | T | 0.12903 | 8 | 0.25806 |
| 18962 | S26_1338736 | 26 | 1338736 | 3 | C | T | C | 0.90323 | T | 0.09677 | 4 | 0.12903 |
| 18963 | S26_1338764 | 26 | 1338764 | 28 | G | A | G | 0.90323 | A | 0.09677 | 4 | 0.12903 |
| 18964 | S26_1338802 | 26 | 1338802 | 38 | A | G | A | 0.90323 | G | 0.09677 | 4 | 0.12903 |
| 18965 | S26_1338806 | 26 | 1338806 | 4 | G | A | G | 0.90323 | A | 0.09677 | 6 | 0.19355 |
| 18966 | S26_1338813 | 26 | 1338813 | 7 | A | C | A | 0.90323 | C | 0.09677 | 4 | 0.12903 |
| 18967 | S26_1338912 | 26 | 1338912 | 99 | A | G | A | 0.69355 | G | 0.30645 | 17 | 0.54839 |
| 18968 | S26_1338920 | 26 | 1338920 | 8 | A | G | A | 0.91935 | G | 0.08065 | 5 | 0.16129 |
| 18969 | S26_1339658 | 26 | 1339658 | 738 | A | G | G | 0.82258 | A | 0.17742 | 11 | 0.35484 |
| 18970 | S26_1339680 | 26 | 1339680 | 22 | G | A | G | 0.91935 | A | 0.08065 | 3 | 0.09677 |
| 18971 | S26_1339739 | 26 | 1339739 | 59 | T | C | T | 0.6129 | C | 0.3871 | 16 | 0.51613 |
| 18972 | S26_1339777 | 26 | 1339777 | 38 | C | T | C | 0.90323 | T | 0.09677 | 4 | 0.12903 |
| 18973 | S26_1339837 | 26 | 1339837 | 60 | T | C | T | 0.72581 | C | 0.27419 | 13 | 0.41935 |
| 18974 | S26_1751328 | 26 | 1751328 | 411491 | C | T | C | 0.74194 | T | 0.25806 | 10 | 0.32258 |
| 18975 | S26_1751428 | 26 | 1751428 | 100 | G | A | G | 0.79032 | A | 0.20968 | 7 | 0.22581 |
| 18976 | S26_1751438 | 26 | 1751438 | 10 | G | A | G | 0.93548 | A | 0.06452 | 4 | 0.12903 |
| 18977 | S26_1996154 | 26 | 1996154 | 244716 | A | C | A | 0.90323 | C | 0.09677 | 6 | 0.19355 |
| 18978 | S26_1996183 | 26 | 1996183 | 29 | A | C | A | 0.67742 | C | 0.32258 | 14 | 0.45161 |
| 18979 | S26_1996189 | 26 | 1996189 | 6 | C | T | T | 0.91935 | C | 0.08065 | 5 | 0.16129 |
| 18980 | S26_1996220 | 26 | 1996220 | 31 | C | T | C | 0.72581 | T | 0.27419 | 11 | 0.35484 |
| 18981 | S26_1996245 | 26 | 1996245 | 25 | G | C | G | 0.90323 | C | 0.09677 | 6 | 0.19355 |
| 18982 | S26_1996260 | 26 | 1996260 | 15 | T | C | C | 0.64516 | T | 0.35484 | 14 | 0.45161 |
| 18983 | S26_2255977 | 26 | 2255977 | 259717 | C | T | C | 0.90323 | T | 0.09677 | 4 | 0.12903 |
| 18984 | S26_2487186 | 26 | 2487186 | 231209 | G | A | A | 0.75806 | G | 0.24194 | 11 | 0.35484 |
| 18985 | S26_2554935 | 26 | 2554935 | 67749 | C | T | C | 0.93548 | T | 0.06452 | 4 | 0.12903 |
| 18986 | S26_3028709 | 26 | 3028709 | 473774 | C | T | C | 0.74194 | T | 0.25806 | 8 | 0.25806 |
| 18987 | S26_3028853 | 26 | 3028853 | 144 | C | T | C | 0.56452 | T | 0.43548 | 13 | 0.41935 |
| 18988 | S26_3096082 | 26 | 3096082 | 67229 | C | T | C | 0.90323 | T | 0.09677 | 4 | 0.12903 |
| 18989 | S26_3096091 | 26 | 3096091 | 9 | G | A | G | 0.90323 | A | 0.09677 | 6 | 0.19355 |
| 18990 | S26_3096166 | 26 | 3096166 | 75 | T | C | T | 0.90323 | C | 0.09677 | 6 | 0.19355 |
| 18991 | S26_3347821 | 26 | 3347821 | 251655 | C | T | C | 0.90323 | T | 0.09677 | 6 | 0.19355 |
| 18992 | S26_3490098 | 26 | 3490098 | 142277 | C | T | T | 0.83871 | C | 0.16129 | 10 | 0.32258 |
| 18993 | S26_3941468 | 26 | 3941468 | 451370 | C | T | C | 0.93548 | T | 0.06452 | 4 | 0.12903 |
| 18994 | S26_4281923 | 26 | 4281923 | 340455 | C | T | C | 0.67742 | T | 0.32258 | 12 | 0.3871 |
| 18995 | S26_4282005 | 26 | 4282005 | 82 | G | C | G | 0.64516 | C | 0.35484 | 12 | 0.3871 |
| 18996 | S26_4282008 | 26 | 4282008 | 3 | T | C | T | 0.64516 | C | 0.35484 | 12 | 0.3871 |
| 18997 | S26_4282094 | 26 | 4282094 | 86 | A | G | A | 0.90323 | G | 0.09677 | 4 | 0.12903 |
| 18998 | S26_4652631 | 26 | 4652631 | 370537 | G | A | G | 0.62903 | A | 0.37097 | 13 | 0.41935 |
| 18999 | S26_4652834 | 26 | 4652834 | 203 | T | C | C | 0.67742 | T | 0.32258 | 10 | 0.32258 |
| 19000 | S26_4652837 | 26 | 4652837 | 3 | A | G | A | 0.56452 | G | 0.43548 | 13 | 0.41935 |
| 19001 | S26_4682934 | 26 | 4682934 | 30097 | T | C | C | 0.83871 | T | 0.16129 | 8 | 0.25806 |
| 19002 | S26_4682935 | 26 | 4682935 | 1 | G | A | G | 0.64516 | A | 0.35484 | 14 | 0.45161 |
| 19003 | S26_4879885 | 26 | 4879885 | 196950 | A | C | C | 0.72581 | A | 0.27419 | 13 | 0.41935 |
| 19004 | S26_4879886 | 26 | 4879886 | 1 | G | A | A | 0.72581 | G | 0.27419 | 13 | 0.41935 |
| 19005 | S26_4879894 | 26 | 4879894 | 8 | A | C | C | 0.74194 | A | 0.25806 | 12 | 0.3871 |
| 19006 | S26_5056851 | 26 | 5056851 | 176957 | G | A | A | 0.83871 | G | 0.16129 | 8 | 0.25806 |

| Site number | SNP name | Chromosome | Physical position | Physical distance from the previous SNP | Reference allele | Alternative allele | Major allele | Major allele frequency | Minor allele | Minor allele frequency | Number heterozygous | Heterozygosity |
|---|---|---|---|---|---|---|---|---|---|---|---|---|
| 19007 | S26_5138281 | 26 | 5138281 | 81430 | C | T | C | 0.91935 | T | 0.08065 | 5 | 0.16129 |
| 19008 | S26_5239252 | 26 | 5239252 | 100971 | G | A | G | 0.90323 | A | 0.09677 | 6 | 0.19355 |
| 19009 | S26_5239427 | 26 | 5239427 | 175 | C | A | C | 0.91935 | A | 0.08065 | 3 | 0.09677 |
| 19010 | S26_5847209 | 26 | 5847209 | 607782 | T | C | T | 0.83871 | C | 0.16129 | 6 | 0.19355 |
| 19011 | S26_5847336 | 26 | 5847336 | 127 | G | C | G | 0.64516 | C | 0.35484 | 12 | 0.3871 |
| 19012 | S26_5847369 | 26 | 5847369 | 33 | G | A | G | 0.85484 | A | 0.14516 | 7 | 0.22581 |
| 19013 | S26_6000164 | 26 | 6000164 | 152795 | T | C | T | 0.93548 | C | 0.06452 | 4 | 0.12903 |
| 19014 | S26_6868855 | 26 | 6868855 | 868691 | C | T | C | 0.90323 | T | 0.09677 | 6 | 0.19355 |
| 19015 | S26_6869065 | 26 | 6869065 | 210 | T | C | T | 0.80645 | C | 0.19355 | 10 | 0.32258 |
| 19016 | S26_7282950 | 26 | 7282950 | 413885 | T | C | T | 0.87097 | C | 0.12903 | 6 | 0.19355 |
| 19017 | S26_7282983 | 26 | 7282983 | 33 | G | A | G | 0.91935 | A | 0.08065 | 5 | 0.16129 |
| 19018 | S26_7283010 | 26 | 7283010 | 27 | T | C | T | 0.93548 | C | 0.06452 | 4 | 0.12903 |
| 19019 | S26_7383209 | 26 | 7383209 | 100199 | T | C | C | 0.91935 | T | 0.08065 | 3 | 0.09677 |
| 19020 | S26_7568458 | 26 | 7568458 | 185249 | A | G | A | 0.8871 | G | 0.1129 | 5 | 0.16129 |
| 19021 | S26_7708902 | 26 | 7708902 | 140444 | T | C | T | 0.8871 | C | 0.1129 | 5 | 0.16129 |
| 19022 | S26_7862526 | 26 | 7862526 | 153624 | T | C | T | 0.95161 | C | 0.04839 | 3 | 0.09677 |
| 19023 | S26_7862676 | 26 | 7862676 | 150 | C | A | C | 0.59677 | A | 0.40323 | 15 | 0.48387 |
| 19024 | S26_7918266 | 26 | 7918266 | 55590 | A | C | C | 0.79032 | A | 0.20968 | 11 | 0.35484 |
| 19025 | S26_8029171 | 26 | 8029171 | 110905 | G | C | C | 0.8871 | G | 0.1129 | 5 | 0.16129 |
| 19026 | S26_8029200 | 26 | 8029200 | 29 | G | A | G | 0.75806 | A | 0.24194 | 11 | 0.35484 |
| 19027 | S26_8029202 | 26 | 8029202 | 2 | A | G | A | 0.75806 | G | 0.24194 | 11 | 0.35484 |
| 19028 | S26_8175500 | 26 | 8175500 | 146298 | A | G | G | 0.8871 | A | 0.1129 | 7 | 0.22581 |
| 19029 | S26_8392126 | 26 | 8392126 | 216626 | C | T | C | 0.77419 | T | 0.22581 | 8 | 0.25806 |
| 19030 | S26_8524998 | 26 | 8524998 | 132872 | G | A | G | 0.8871 | A | 0.1129 | 5 | 0.16129 |
| 19031 | S26_8525031 | 26 | 8525031 | 33 | G | T | G | 0.87097 | T | 0.12903 | 8 | 0.25806 |
| 19032 | S26_8567558 | 26 | 8567558 | 42527 | G | T | G | 0.82258 | T | 0.17742 | 11 | 0.35484 |
| 19033 | S26_8567628 | 26 | 8567628 | 70 | G | A | G | 0.93548 | A | 0.06452 | 4 | 0.12903 |
| 19034 | S26_8567736 | 26 | 8567736 | 108 | G | A | G | 0.93548 | A | 0.06452 | 4 | 0.12903 |
| 19035 | S26_8567746 | 26 | 8567746 | 10 | A | G | A | 0.93548 | G | 0.06452 | 4 | 0.12903 |
| 19036 | S26_8567756 | 26 | 8567756 | 10 | C | A | C | 0.90323 | A | 0.09677 | 4 | 0.12903 |
| 19037 | S26_8715733 | 26 | 8715733 | 147977 | C | T | C | 0.79032 | T | 0.20968 | 11 | 0.35484 |
| 19038 | S26_8715794 | 26 | 8715794 | 61 | A | G | A | 0.77419 | G | 0.22581 | 12 | 0.3871 |
| 19039 | S26_10094771 | 26 | 10094771 | 1378977 | T | C | T | 0.91935 | C | 0.08065 | 3 | 0.09677 |
| 19040 | S26_10152496 | 26 | 10152496 | 57725 | C | T | C | 0.85484 | T | 0.14516 | 7 | 0.22581 |
| 19041 | S26_10152559 | 26 | 10152559 | 63 | C | T | C | 0.93548 | T | 0.06452 | 4 | 0.12903 |
| 19042 | S26_10152636 | 26 | 10152636 | 77 | G | C | G | 0.83871 | C | 0.16129 | 10 | 0.32258 |
| 19043 | S26_10270734 | 26 | 10270734 | 118098 | G | A | G | 0.90323 | A | 0.09677 | 6 | 0.19355 |
| 19044 | S26_10270855 | 26 | 10270855 | 121 | T | C | T | 0.56452 | C | 0.43548 | 19 | 0.6129 |
| 19045 | S26_10270900 | 26 | 10270900 | 45 | T | G | T | 0.90323 | G | 0.09677 | 6 | 0.19355 |
| 19046 | S26_10270924 | 26 | 10270924 | 24 | G | A | G | 0.91935 | A | 0.08065 | 3 | 0.09677 |
| 19047 | S26_10270954 | 26 | 10270954 | 30 | A | C | C | 0.77419 | A | 0.22581 | 10 | 0.32258 |
| 19048 | S26_10439113 | 26 | 10439113 | 168159 | C | T | T | 0.64516 | C | 0.35484 | 18 | 0.58065 |
| 19049 | S26_10572238 | 26 | 10572238 | 133125 | T | C | C | 0.70968 | T | 0.29032 | 10 | 0.32258 |
| 19050 | S26_10572271 | 26 | 10572271 | 33 | G | C | C | 0.70968 | G | 0.29032 | 10 | 0.32258 |
| 19051 | S26_10572436 | 26 | 10572436 | 165 | T | C | C | 0.70968 | T | 0.29032 | 10 | 0.32258 |
| 19052 | S26_10599641 | 26 | 10599641 | 27205 | C | A | C | 0.91935 | A | 0.08065 | 3 | 0.09677 |
| 19053 | S26_10706517 | 26 | 10706517 | 106876 | C | G | C | 0.87097 | G | 0.12903 | 6 | 0.19355 |
| 19054 | S26_10718976 | 26 | 10718976 | 12459 | G | C | C | 0.69355 | G | 0.30645 | 17 | 0.54839 |
| 19055 | S26_11225064 | 26 | 11225064 | 506088 | A | C | A | 0.90323 | C | 0.09677 | 4 | 0.12903 |
| 19056 | S26_11225185 | 26 | 11225185 | 121 | C | T | C | 0.90323 | T | 0.09677 | 4 | 0.12903 |
| 19057 | S26_11348448 | 26 | 11348448 | 123263 | T | C | C | 0.82258 | T | 0.17742 | 11 | 0.35484 |
| 19058 | S26_11653495 | 26 | 11653495 | 305047 | T | G | T | 0.8871 | G | 0.1129 | 7 | 0.22581 |
| 19059 | S26_11653563 | 26 | 11653563 | 68 | T | C | C | 0.77419 | T | 0.22581 | 12 | 0.3871 |
| 19060 | S26_11772938 | 26 | 11772938 | 119375 | T | C | T | 0.6129 | C | 0.3871 | 10 | 0.32258 |

| Site number | SNP name | Chromosome | Physical position | Physical distance from the previous SNP | Reference allele | Alternative allele | Major allele | Major allele frequency | Minor allele | Minor allele frequency | Number heterozygous | Heterozygosity |
|---|---|---|---|---|---|---|---|---|---|---|---|---|
| 19061 | S26_11773116 | 26 | 11773116 | 178 | T | C | T | 0.6129 | C | 0.3871 | 10 | 0.32258 |
| 19062 | S26_11773117 | 26 | 11773117 | 1 | G | A | G | 0.6129 | A | 0.3871 | 10 | 0.32258 |
| 19063 | S26_11800723 | 26 | 11800723 | 27606 | A | C | A | 0.91935 | C | 0.08065 | 5 | 0.16129 |
| 19064 | S26_11800848 | 26 | 11800848 | 125 | C | A | C | 0.87097 | A | 0.12903 | 4 | 0.12903 |
| 19065 | S26_12367344 | 26 | 12367344 | 566496 | A | G | A | 0.72581 | G | 0.27419 | 15 | 0.48387 |
| 19066 | S26_12447975 | 26 | 12447975 | 80631 | C | T | C | 0.90323 | T | 0.09677 | 4 | 0.12903 |
| 19067 | S26_12580532 | 26 | 12580532 | 132557 | T | C | T | 0.93548 | C | 0.06452 | 4 | 0.12903 |
| 19068 | S26_12580666 | 26 | 12580666 | 134 | G | A | G | 0.67742 | A | 0.32258 | 10 | 0.32258 |
| 19069 | S26_12865777 | 26 | 12865777 | 285111 | C | T | C | 0.91935 | T | 0.08065 | 3 | 0.09677 |
| 19070 | S26_12865895 | 26 | 12865895 | 118 | G | A | G | 0.8871 | A | 0.1129 | 7 | 0.22581 |
| 19071 | S26_13059719 | 26 | 13059719 | 193824 | A | G | A | 0.91935 | G | 0.08065 | 3 | 0.09677 |
| 19072 | S26_13281912 | 26 | 13281912 | 222193 | A | G | A | 0.91935 | G | 0.08065 | 3 | 0.09677 |
| 19073 | S26_13361186 | 26 | 13361186 | 79274 | C | G | C | 0.95161 | G | 0.04839 | 3 | 0.09677 |
| 19074 | S26_13361343 | 26 | 13361343 | 157 | T | C | T | 0.72581 | C | 0.27419 | 13 | 0.41935 |
| 19075 | S26_13414091 | 26 | 13414091 | 52748 | G | A | G | 0.87097 | A | 0.12903 | 8 | 0.25806 |
| 19076 | S26_13866113 | 26 | 13866113 | 452022 | A | G | A | 0.58065 | G | 0.41935 | 12 | 0.3871 |
| 19077 | S26_13866184 | 26 | 13866184 | 71 | C | G | C | 0.8871 | G | 0.1129 | 7 | 0.22581 |
| 19078 | S26_13866185 | 26 | 13866185 | 1 | T | G | T | 0.8871 | G | 0.1129 | 7 | 0.22581 |
| 19079 | S26_14010703 | 26 | 14010703 | 144518 | G | C | G | 0.93548 | C | 0.06452 | 2 | 0.06452 |
| 19080 | S26_14010704 | 26 | 14010704 | 1 | A | T | A | 0.93548 | T | 0.06452 | 2 | 0.06452 |
| 19081 | S26_14249817 | 26 | 14249817 | 239113 | G | A | G | 0.93548 | A | 0.06452 | 4 | 0.12903 |
| 19082 | S26_14288081 | 26 | 14288081 | 38264 | C | T | C | 0.95161 | T | 0.04839 | 3 | 0.09677 |
| 19083 | S26_14288117 | 26 | 14288117 | 36 | G | A | G | 0.67742 | A | 0.32258 | 16 | 0.51613 |
| 19084 | S26_14288195 | 26 | 14288195 | 78 | G | A | G | 0.87097 | A | 0.12903 | 8 | 0.25806 |
| 19085 | S26_14457611 | 26 | 14457611 | 169416 | A | C | A | 0.91935 | C | 0.08065 | 5 | 0.16129 |
| 19086 | S26_14457642 | 26 | 14457642 | 31 | T | C | T | 0.58065 | C | 0.41935 | 16 | 0.51613 |
| 19087 | S26_14457806 | 26 | 14457806 | 164 | G | A | G | 0.58065 | A | 0.41935 | 16 | 0.51613 |
| 19088 | S26_14825293 | 26 | 14825293 | 367487 | T | C | T | 0.83871 | C | 0.16129 | 8 | 0.25806 |
| 19089 | S26_14896569 | 26 | 14896569 | 71276 | G | A | A | 0.56452 | G | 0.43548 | 15 | 0.48387 |
| 19090 | S26_14904437 | 26 | 14904437 | 7868 | G | A | G | 0.87097 | A | 0.12903 | 8 | 0.25806 |
| 19091 | S26_15165865 | 26 | 15165865 | 261428 | G | A | G | 0.77419 | A | 0.22581 | 12 | 0.3871 |
| 19092 | S26_15613769 | 26 | 15613769 | 447904 | A | G | G | 0.87097 | A | 0.12903 | 8 | 0.25806 |
| 19093 | S26_15613814 | 26 | 15613814 | 45 | C | T | C | 0.93548 | T | 0.06452 | 4 | 0.12903 |
| 19094 | S26_15613971 | 26 | 15613971 | 157 | T | C | T | 0.80645 | C | 0.19355 | 10 | 0.32258 |
| 19095 | S26_15654621 | 26 | 15654621 | 40650 | G | A | G | 0.91935 | A | 0.08065 | 5 | 0.16129 |
| 19096 | S26_16573136 | 26 | 16573136 | 918515 | C | G | C | 0.93548 | G | 0.06452 | 4 | 0.12903 |
| 19097 | S26_16589518 | 26 | 16589518 | 16382 | G | A | G | 0.80645 | A | 0.19355 | 10 | 0.32258 |
| 19098 | S26_16777091 | 26 | 16777091 | 187573 | C | T | C | 0.85484 | T | 0.14516 | 7 | 0.22581 |
| 19099 | S26_16777161 | 26 | 16777161 | 70 | C | T | C | 0.90323 | T | 0.09677 | 6 | 0.19355 |
| 19100 | S26_16777211 | 26 | 16777211 | 50 | T | G | T | 0.51613 | G | 0.48387 | 12 | 0.3871 |
| 19101 | S26_16777212 | 26 | 16777212 | 1 | T | G | T | 0.51613 | G | 0.48387 | 12 | 0.3871 |
| 19102 | S26_16777223 | 26 | 16777223 | 11 | A | G | G | 0.6129 | A | 0.3871 | 18 | 0.58065 |
| 19103 | S26_16777224 | 26 | 16777224 | 1 | C | T | T | 0.6129 | C | 0.3871 | 18 | 0.58065 |
| 19104 | S26_16777227 | 26 | 16777227 | 3 | T | G | G | 0.6129 | T | 0.3871 | 18 | 0.58065 |
| 19105 | S26_16777244 | 26 | 16777244 | 17 | T | C | C | 0.56452 | T | 0.43548 | 19 | 0.6129 |
| 19106 | S26_16777246 | 26 | 16777246 | 2 | C | T | T | 0.6129 | C | 0.3871 | 18 | 0.58065 |
| 19107 | S26_16777250 | 26 | 16777250 | 4 | T | G | T | 0.51613 | G | 0.48387 | 12 | 0.3871 |
| 19108 | S26_16777257 | 26 | 16777257 | 7 | C | A | A | 0.6129 | C | 0.3871 | 18 | 0.58065 |
| 19109 | S26_17159181 | 26 | 17159181 | 381924 | C | T | C | 0.8871 | T | 0.1129 | 5 | 0.16129 |
| 19110 | S26_17171414 | 26 | 17171414 | 12233 | T | G | T | 0.8871 | G | 0.1129 | 5 | 0.16129 |
| 19111 | S26_17171539 | 26 | 17171539 | 125 | T | C | T | 0.8871 | C | 0.1129 | 5 | 0.16129 |
| 19112 | S26_17171544 | 26 | 17171544 | 5 | A | G | A | 0.6129 | G | 0.3871 | 12 | 0.3871 |
| 19113 | S26_17875782 | 26 | 17875782 | 704238 | G | A | G | 0.75806 | A | 0.24194 | 11 | 0.35484 |
| 19114 | S26_17887821 | 26 | 17887821 | 12039 | A | G | G | 0.80645 | A | 0.19355 | 10 | 0.32258 |

| Site number | SNP name | Chromosome | Physical position | Physical distance from the previous SNP | Reference allele | Alternative allele | Major allele | Major allele frequency | Minor allele | Minor allele frequency | Number heterozygous | Heterozygosity |
|---|---|---|---|---|---|---|---|---|---|---|---|---|
| 19115 | S26_17902589 | 26 | 17902589 | 14768 | G | A | A | 0.6129 | G | 0.3871 | 14 | 0.45161 |
| 19116 | S26_18091174 | 26 | 18091174 | 188585 | C | T | C | 0.82258 | T | 0.17742 | 11 | 0.35484 |
| 19117 | S26_18111764 | 26 | 18111764 | 20590 | A | G | A | 0.70968 | G | 0.29032 | 14 | 0.45161 |
| 19118 | S26_18139967 | 26 | 18139967 | 28203 | G | T | T | 0.6129 | G | 0.3871 | 14 | 0.45161 |
| 19119 | S26_18333060 | 26 | 18333060 | 193093 | C | G | C | 0.69355 | G | 0.30645 | 13 | 0.41935 |
| 19120 | S26_18393196 | 26 | 18393196 | 60136 | A | G | A | 0.90323 | G | 0.09677 | 6 | 0.19355 |
| 19121 | S26_18470065 | 26 | 18470065 | 76869 | A | G | A | 0.70968 | G | 0.29032 | 14 | 0.45161 |
| 19122 | S26_18470215 | 26 | 18470215 | 150 | G | A | G | 0.72581 | A | 0.27419 | 13 | 0.41935 |
| 19123 | S26_18777523 | 26 | 18777523 | 307308 | C | T | C | 0.58065 | T | 0.41935 | 16 | 0.51613 |
| 19124 | S26_19243091 | 26 | 19243091 | 465568 | G | A | G | 0.93548 | A | 0.06452 | 4 | 0.12903 |
| 19125 | S26_19251391 | 26 | 19251391 | 8300 | A | C | C | 0.77419 | A | 0.22581 | 14 | 0.45161 |
| 19126 | S26_19251475 | 26 | 19251475 | 84 | T | C | T | 0.72581 | C | 0.27419 | 15 | 0.48387 |
| 19127 | S26_19251604 | 26 | 19251604 | 129 | G | A | A | 0.5 | G | 0.5 | 21 | 0.67742 |
| 19128 | S26_19425371 | 26 | 19425371 | 173767 | G | C | G | 0.77419 | C | 0.22581 | 8 | 0.25806 |
| 19129 | S26_19425381 | 26 | 19425381 | 10 | C | T | T | 0.66129 | C | 0.33871 | 17 | 0.54839 |
| 19130 | S26_19425391 | 26 | 19425391 | 10 | G | A | G | 0.77419 | A | 0.22581 | 8 | 0.25806 |
| 19131 | S26_19425476 | 26 | 19425476 | 85 | G | A | A | 0.75806 | G | 0.24194 | 13 | 0.41935 |
| 19132 | S26_19425499 | 26 | 19425499 | 23 | C | T | C | 0.95161 | T | 0.04839 | 3 | 0.09677 |
| 19133 | S26_19664873 | 26 | 19664873 | 239374 | C | T | C | 0.93548 | T | 0.06452 | 4 | 0.12903 |
| 19134 | S26_19664997 | 26 | 19664997 | 124 | G | A | G | 0.90323 | A | 0.09677 | 6 | 0.19355 |
| 19135 | S26_19911166 | 26 | 19911166 | 246169 | C | T | C | 0.93548 | T | 0.06452 | 4 | 0.12903 |
| 19136 | S26_19911324 | 26 | 19911324 | 158 | G | A | A | 0.95161 | G | 0.04839 | 1 | 0.03226 |
| 19137 | S26_20318705 | 26 | 20318705 | 407381 | G | A | G | 0.83871 | A | 0.16129 | 10 | 0.32258 |
| 19138 | S26_20541049 | 26 | 20541049 | 222344 | T | C | T | 0.93548 | C | 0.06452 | 4 | 0.12903 |
| 19139 | S26_21021594 | 26 | 21021594 | 480545 | G | C | G | 0.70968 | C | 0.29032 | 12 | 0.3871 |
| 19140 | S26_21021689 | 26 | 21021689 | 95 | A | G | A | 0.70968 | G | 0.29032 | 12 | 0.3871 |
| 19141 | S26_21021769 | 26 | 21021769 | 80 | C | G | C | 0.70968 | G | 0.29032 | 12 | 0.3871 |
| 19142 | S26_21021786 | 26 | 21021786 | 17 | A | C | A | 0.70968 | C | 0.29032 | 12 | 0.3871 |
| 19143 | S26_21480784 | 26 | 21480784 | 458998 | T | C | T | 0.80645 | C | 0.19355 | 10 | 0.32258 |
| 19144 | S26_21504039 | 26 | 21504039 | 23255 | T | C | C | 0.51613 | T | 0.48387 | 16 | 0.51613 |
| 19145 | S26_21504152 | 26 | 21504152 | 113 | T | A | T | 0.87097 | A | 0.12903 | 6 | 0.19355 |
| 19146 | S26_21504179 | 26 | 21504179 | 27 | A | G | G | 0.53226 | A | 0.46774 | 15 | 0.48387 |
| 19147 | S26_21518506 | 26 | 21518506 | 14327 | T | C | C | 0.58065 | T | 0.41935 | 16 | 0.51613 |
| 19148 | S26_21518535 | 26 | 21518535 | 29 | G | T | G | 0.56452 | T | 0.43548 | 17 | 0.54839 |
| 19149 | S26_21518676 | 26 | 21518676 | 141 | C | G | C | 0.93548 | G | 0.06452 | 4 | 0.12903 |
| 19150 | S26_21591745 | 26 | 21591745 | 73069 | T | C | T | 0.93548 | C | 0.06452 | 4 | 0.12903 |
| 19151 | S26_22126092 | 26 | 22126092 | 534347 | T | C | T | 0.67742 | C | 0.32258 | 16 | 0.51613 |
| 19152 | S26_23091836 | 26 | 23091836 | 965744 | T | C | T | 0.6129 | C | 0.3871 | 18 | 0.58065 |
| 19153 | S26_23290522 | 26 | 23290522 | 198686 | C | T | C | 0.93548 | T | 0.06452 | 4 | 0.12903 |
| 19154 | S26_23290531 | 26 | 23290531 | 9 | G | C | C | 0.83871 | G | 0.16129 | 8 | 0.25806 |
| 19155 | S26_23387531 | 26 | 23387531 | 97000 | G | A | G | 0.70968 | A | 0.29032 | 14 | 0.45161 |
| 19156 | S26_23754704 | 26 | 23754704 | 367173 | G | A | G | 0.90323 | A | 0.09677 | 6 | 0.19355 |
| 19157 | S26_23776404 | 26 | 23776404 | 21700 | A | G | A | 0.91935 | G | 0.08065 | 5 | 0.16129 |
| 19158 | S26_23776436 | 26 | 23776436 | 32 | G | C | G | 0.91935 | C | 0.08065 | 5 | 0.16129 |
| 19159 | S26_23776482 | 26 | 23776482 | 46 | A | G | A | 0.51613 | G | 0.48387 | 16 | 0.51613 |
| 19160 | S26_23776499 | 26 | 23776499 | 17 | A | G | A | 0.91935 | G | 0.08065 | 5 | 0.16129 |
| 19161 | S26_23776532 | 26 | 23776532 | 33 | A | G | G | 0.58065 | A | 0.41935 | 16 | 0.51613 |
| 19162 | S26_23776539 | 26 | 23776539 | 7 | C | T | C | 0.8871 | T | 0.1129 | 5 | 0.16129 |
| 19163 | S26_23833900 | 26 | 23833900 | 57361 | G | A | G | 0.95161 | A | 0.04839 | 3 | 0.09677 |
| 19164 | S26_23833979 | 26 | 23833979 | 79 | C | G | C | 0.93548 | G | 0.06452 | 4 | 0.12903 |
| 19165 | S26_24532188 | 26 | 24532188 | 698209 | G | A | G | 0.8871 | A | 0.1129 | 7 | 0.22581 |
| 19166 | S26_24532262 | 26 | 24532262 | 74 | T | G | G | 0.82258 | T | 0.17742 | 7 | 0.22581 |
| 19167 | S26_24532292 | 26 | 24532292 | 30 | G | A | A | 0.93548 | G | 0.06452 | 2 | 0.06452 |
| 19168 | S26_24532354 | 26 | 24532354 | 62 | T | C | T | 0.54839 | C | 0.45161 | 12 | 0.3871 |

| Site number | SNP name | Chromosome | Physical position | Physical distance from the previous SNP | Reference allele | Alternative allele | Major allele | Major allele frequency | Minor allele | Minor allele frequency | Number heterozygous | Heterozygosity |
|---|---|---|---|---|---|---|---|---|---|---|---|---|
| 19169 | S26_24532378 | 26 | 24532378 | 24 | G | A | G | 0.75806 | A | 0.24194 | 13 | 0.41935 |
| 19170 | S26_24767044 | 26 | 24767044 | 234666 | A | G | G | 0.54839 | A | 0.45161 | 16 | 0.51613 |
| 19171 | S26_24767047 | 26 | 24767047 | 3 | G | C | G | 0.95161 | C | 0.04839 | 3 | 0.09677 |
| 19172 | S26_24767080 | 26 | 24767080 | 33 | C | T | T | 0.54839 | C | 0.45161 | 16 | 0.51613 |
| 19173 | S26_24841088 | 26 | 24841088 | 74008 | G | T | G | 0.93548 | T | 0.06452 | 4 | 0.12903 |
| 19174 | S26_25185922 | 26 | 25185922 | 344834 | C | A | C | 0.82258 | A | 0.17742 | 11 | 0.35484 |
| 19175 | S26_25185953 | 26 | 25185953 | 31 | A | G | A | 0.87097 | G | 0.12903 | 8 | 0.25806 |
| 19176 | S26_25186008 | 26 | 25186008 | 55 | A | G | A | 0.74194 | G | 0.25806 | 14 | 0.45161 |
| 19177 | S26_25455089 | 26 | 25455089 | 269081 | A | T | T | 0.70968 | A | 0.29032 | 18 | 0.58065 |
| 19178 | S26_25455146 | 26 | 25455146 | 57 | A | G | A | 0.56452 | G | 0.43548 | 21 | 0.67742 |
| 19179 | S26_25455169 | 26 | 25455169 | 23 | C | T | C | 0.93548 | T | 0.06452 | 4 | 0.12903 |
| 19180 | S26_26038362 | 26 | 26038362 | 583193 | G | A | G | 0.90323 | A | 0.09677 | 6 | 0.19355 |
| 19181 | S26_26038527 | 26 | 26038527 | 165 | C | T | C | 0.5 | T | 0.5 | 17 | 0.54839 |
| 19182 | S26_26038540 | 26 | 26038540 | 13 | T | C | C | 0.5 | T | 0.5 | 17 | 0.54839 |
| 19183 | S26_26038541 | 26 | 26038541 | 1 | G | C | C | 0.5 | G | 0.5 | 17 | 0.54839 |
| 19184 | S26_26038543 | 26 | 26038543 | 2 | G | A | A | 0.83871 | G | 0.16129 | 8 | 0.25806 |
| 19185 | S26_26202052 | 26 | 26202052 | 163509 | T | C | T | 0.91935 | C | 0.08065 | 5 | 0.16129 |
| 19186 | S26_26202205 | 26 | 26202205 | 153 | C | T | C | 0.80645 | T | 0.19355 | 10 | 0.32258 |
| 19187 | S26_26202206 | 26 | 26202206 | 1 | T | A | T | 0.80645 | A | 0.19355 | 10 | 0.32258 |
| 19188 | S26_26245174 | 26 | 26245174 | 42968 | G | A | G | 0.91935 | A | 0.08065 | 5 | 0.16129 |
| 19189 | S26_26429830 | 26 | 26429830 | 184656 | G | T | G | 0.91935 | T | 0.08065 | 5 | 0.16129 |
| 19190 | S26_26429839 | 26 | 26429839 | 9 | T | C | T | 0.67742 | C | 0.32258 | 16 | 0.51613 |
| 19191 | S26_26429849 | 26 | 26429849 | 10 | C | T | C | 0.67742 | T | 0.32258 | 16 | 0.51613 |
| 19192 | S26_26429878 | 26 | 26429878 | 29 | A | G | A | 0.91935 | G | 0.08065 | 3 | 0.09677 |
| 19193 | S26_26703193 | 26 | 26703193 | 273315 | A | G | G | 0.83871 | A | 0.16129 | 6 | 0.19355 |
| 19194 | S26_26703275 | 26 | 26703275 | 82 | G | A | G | 0.75806 | A | 0.24194 | 15 | 0.48387 |
| 19195 | S26_26703404 | 26 | 26703404 | 129 | T | C | T | 0.87097 | C | 0.12903 | 8 | 0.25806 |
| 19196 | S26_26712681 | 26 | 26712681 | 9277 | T | C | T | 0.87097 | C | 0.12903 | 8 | 0.25806 |
| 19197 | S26_27283088 | 26 | 27283088 | 570407 | T | C | T | 0.51613 | C | 0.48387 | 16 | 0.51613 |
| 19198 | S26_27368091 | 26 | 27368091 | 85003 | C | G | C | 0.75806 | G | 0.24194 | 9 | 0.29032 |
| 19199 | S26_27368264 | 26 | 27368264 | 173 | G | T | G | 0.75806 | T | 0.24194 | 9 | 0.29032 |
| 19200 | S26_27368265 | 26 | 27368265 | 1 | A | G | A | 0.90323 | G | 0.09677 | 6 | 0.19355 |
| 19201 | S26_27368291 | 26 | 27368291 | 26 | G | A | G | 0.75806 | A | 0.24194 | 9 | 0.29032 |
| 19202 | S26_27368309 | 26 | 27368309 | 18 | A | G | A | 0.69355 | G | 0.30645 | 11 | 0.35484 |
| 19203 | S26_27391146 | 26 | 27391146 | 22837 | A | G | A | 0.58065 | G | 0.41935 | 14 | 0.45161 |
| 19204 | S26_27852088 | 26 | 27852088 | 460942 | C | T | C | 0.77419 | T | 0.22581 | 14 | 0.45161 |
| 19205 | S26_27852198 | 26 | 27852198 | 110 | T | C | T | 0.77419 | C | 0.22581 | 14 | 0.45161 |
| 19206 | S26_28474383 | 26 | 28474383 | 622185 | G | A | G | 0.93548 | A | 0.06452 | 4 | 0.12903 |
| 19207 | S26_28474443 | 26 | 28474443 | 60 | A | G | A | 0.91935 | G | 0.08065 | 5 | 0.16129 |
| 19208 | S26_29146913 | 26 | 29146913 | 672470 | G | C | G | 0.91935 | C | 0.08065 | 5 | 0.16129 |
| 19209 | S26_29935345 | 26 | 29935345 | 788432 | C | T | C | 0.90323 | T | 0.09677 | 6 | 0.19355 |
| 19210 | S26_30037363 | 26 | 30037363 | 102018 | C | A | C | 0.82258 | A | 0.17742 | 7 | 0.22581 |
| 19211 | S26_30037397 | 26 | 30037397 | 34 | C | G | C | 0.95161 | G | 0.04839 | 3 | 0.09677 |
| 19212 | S26_30170006 | 26 | 30170006 | 132609 | T | C | T | 0.8871 | C | 0.1129 | 7 | 0.22581 |
| 19213 | S26_30907243 | 26 | 30907243 | 737237 | T | C | T | 0.79032 | C | 0.20968 | 7 | 0.22581 |
| 19214 | S26_30907360 | 26 | 30907360 | 117 | A | G | G | 0.77419 | A | 0.22581 | 8 | 0.25806 |
| 19215 | S26_31173724 | 26 | 31173724 | 266364 | C | A | C | 0.8871 | A | 0.1129 | 7 | 0.22581 |
| 19216 | S26_31173743 | 26 | 31173743 | 19 | G | A | G | 0.91935 | A | 0.08065 | 5 | 0.16129 |
| 19217 | S26_31173903 | 26 | 31173903 | 160 | G | A | A | 0.87097 | G | 0.12903 | 8 | 0.25806 |
| 19218 | S26_31173924 | 26 | 31173924 | 21 | G | A | G | 0.95161 | A | 0.04839 | 3 | 0.09677 |
| 19219 | S26_32149682 | 26 | 32149682 | 975758 | C | T | C | 0.66129 | T | 0.33871 | 15 | 0.48387 |
| 19220 | S26_32149728 | 26 | 32149728 | 46 | G | T | G | 0.66129 | T | 0.33871 | 15 | 0.48387 |
| 19221 | S26_32168246 | 26 | 32168246 | 18518 | A | T | A | 0.91935 | T | 0.08065 | 5 | 0.16129 |
| 19222 | S26_32168319 | 26 | 32168319 | 73 | C | G | G | 0.62903 | C | 0.37097 | 15 | 0.48387 |

| Site number | SNP name | Chromosome | Physical position | Physical distance from the previous SNP | Reference allele | Alternative allele | Major allele | Major allele frequency | Minor allele | Minor allele frequency | Number heterozygous | Heterozygosity |
|---|---|---|---|---|---|---|---|---|---|---|---|---|
| 19223 | S26_32517077 | 26 | 32517077 | 348758 | G | C | G | 0.90323 | C | 0.09677 | 6 | 0.19355 |
| 19224 | S26_32517272 | 26 | 32517272 | 195 | G | A | G | 0.90323 | A | 0.09677 | 6 | 0.19355 |
| 19225 | S26_32686061 | 26 | 32686061 | 168789 | G | C | G | 0.85484 | C | 0.14516 | 9 | 0.29032 |
| 19226 | S26_32938415 | 26 | 32938415 | 252354 | T | C | T | 0.70968 | C | 0.29032 | 12 | 0.3871 |
| 19227 | S26_33047847 | 26 | 33047847 | 109432 | C | T | C | 0.79032 | T | 0.20968 | 11 | 0.35484 |
| 19228 | S26_33201332 | 26 | 33201332 | 153485 | A | G | G | 0.54839 | A | 0.45161 | 16 | 0.51613 |
| 19229 | S26_33201471 | 26 | 33201471 | 139 | T | C | T | 0.67742 | C | 0.32258 | 16 | 0.51613 |
| 19230 | S26_33530457 | 26 | 33530457 | 328986 | T | A | T | 0.95161 | A | 0.04839 | 3 | 0.09677 |
| 19231 | S26_34365117 | 26 | 34365117 | 834660 | G | A | A | 0.58065 | G | 0.41935 | 10 | 0.32258 |
| 19232 | S26_34493348 | 26 | 34493348 | 128231 | C | T | C | 0.82258 | T | 0.17742 | 7 | 0.22581 |
| 19233 | S26_34493355 | 26 | 34493355 | 7 | G | C | G | 0.82258 | C | 0.17742 | 7 | 0.22581 |
| 19234 | S26_34493444 | 26 | 34493444 | 89 | T | A | T | 0.82258 | A | 0.17742 | 7 | 0.22581 |
| 19235 | S26_34527616 | 26 | 34527616 | 34172 | C | T | C | 0.82258 | T | 0.17742 | 9 | 0.29032 |
| 19236 | S26_34582351 | 26 | 34582351 | 54735 | T | C | C | 0.70968 | T | 0.29032 | 12 | 0.3871 |
| 19237 | S26_34612836 | 26 | 34612836 | 30485 | C | G | G | 0.90323 | C | 0.09677 | 6 | 0.19355 |
| 19238 | S26_34657207 | 26 | 34657207 | 44371 | T | C | T | 0.53226 | C | 0.46774 | 19 | 0.6129 |
| 19239 | S26_34657370 | 26 | 34657370 | 163 | C | G | C | 0.91935 | G | 0.08065 | 5 | 0.16129 |
| 19240 | S26_35399717 | 26 | 35399717 | 742347 | C | T | C | 0.77419 | T | 0.22581 | 14 | 0.45161 |
| 19241 | S26_35399730 | 26 | 35399730 | 13 | A | G | A | 0.87097 | G | 0.12903 | 8 | 0.25806 |
| 19242 | S26_35399800 | 26 | 35399800 | 70 | A | G | A | 0.95161 | G | 0.04839 | 3 | 0.09677 |
| 19243 | S26_35399855 | 26 | 35399855 | 55 | C | T | C | 0.77419 | T | 0.22581 | 14 | 0.45161 |
| 19244 | S26_35418308 | 26 | 35418308 | 18453 | G | A | G | 0.75806 | A | 0.24194 | 15 | 0.48387 |
| 19245 | S26_35418376 | 26 | 35418376 | 68 | C | T | C | 0.75806 | T | 0.24194 | 15 | 0.48387 |
| 19246 | S26_35418392 | 26 | 35418392 | 16 | C | A | C | 0.64516 | A | 0.35484 | 16 | 0.51613 |
| 19247 | S26_35418423 | 26 | 35418423 | 31 | A | G | A | 0.93548 | G | 0.06452 | 4 | 0.12903 |
| 19248 | S26_35418487 | 26 | 35418487 | 64 | C | T | C | 0.93548 | T | 0.06452 | 4 | 0.12903 |
| 19249 | S26_35418493 | 26 | 35418493 | 6 | G | A | G | 0.62903 | A | 0.37097 | 11 | 0.35484 |
| 19250 | S26_35418538 | 26 | 35418538 | 45 | T | C | T | 0.93548 | C | 0.06452 | 4 | 0.12903 |
| 19251 | S26_35877046 | 26 | 35877046 | 458508 | C | G | C | 0.70968 | G | 0.29032 | 10 | 0.32258 |
| 19252 | S26_35877121 | 26 | 35877121 | 75 | T | C | T | 0.93548 | C | 0.06452 | 4 | 0.12903 |
| 19253 | S26_36264460 | 26 | 36264460 | 387339 | A | G | A | 0.91935 | G | 0.08065 | 3 | 0.09677 |
| 19254 | S26_36264482 | 26 | 36264482 | 22 | T | C | C | 0.69355 | T | 0.30645 | 15 | 0.48387 |
| 19255 | S26_36449100 | 26 | 36449100 | 184618 | T | C | C | 0.56452 | T | 0.43548 | 17 | 0.54839 |
| 19256 | S26_36449223 | 26 | 36449223 | 123 | A | C | C | 0.56452 | A | 0.43548 | 17 | 0.54839 |
| 19257 | S26_36836420 | 26 | 36836420 | 387197 | G | A | G | 0.93548 | A | 0.06452 | 4 | 0.12903 |
| 19258 | S26_36836543 | 26 | 36836543 | 123 | T | C | T | 0.62903 | C | 0.37097 | 13 | 0.41935 |
| 19259 | S26_36836577 | 26 | 36836577 | 34 | T | G | T | 0.6129 | G | 0.3871 | 12 | 0.3871 |
| 19260 | S26_36836617 | 26 | 36836617 | 40 | T | C | T | 0.93548 | C | 0.06452 | 2 | 0.06452 |
| 19261 | S26_36970675 | 26 | 36970675 | 134058 | C | T | C | 0.58065 | T | 0.41935 | 16 | 0.51613 |
| 19262 | S26_36970747 | 26 | 36970747 | 72 | T | C | C | 0.74194 | T | 0.25806 | 16 | 0.51613 |
| 19263 | S26_37190072 | 26 | 37190072 | 219325 | A | G | A | 0.95161 | G | 0.04839 | 3 | 0.09677 |
| 19264 | S26_37791454 | 26 | 37791454 | 601382 | A | G | A | 0.70968 | G | 0.29032 | 10 | 0.32258 |
| 19265 | S26_37793409 | 26 | 37793409 | 1955 | C | T | C | 0.90323 | T | 0.09677 | 6 | 0.19355 |
| 19266 | S26_38187570 | 26 | 38187570 | 394161 | C | T | C | 0.93548 | T | 0.06452 | 2 | 0.06452 |
| 19267 | S26_38187606 | 26 | 38187606 | 36 | A | C | A | 0.93548 | C | 0.06452 | 2 | 0.06452 |
| 19268 | S26_38187611 | 26 | 38187611 | 5 | C | G | C | 0.93548 | G | 0.06452 | 2 | 0.06452 |
| 19269 | S26_38187724 | 26 | 38187724 | 113 | C | T | C | 0.93548 | T | 0.06452 | 2 | 0.06452 |
| 19270 | S26_38187730 | 26 | 38187730 | 6 | G | A | G | 0.93548 | A | 0.06452 | 2 | 0.06452 |
| 19271 | S26_38187763 | 26 | 38187763 | 33 | G | A | G | 0.93548 | A | 0.06452 | 2 | 0.06452 |
| 19272 | S26_38187777 | 26 | 38187777 | 14 | T | C | T | 0.87097 | C | 0.12903 | 4 | 0.12903 |
| 19273 | S26_38547921 | 26 | 38547921 | 360144 | C | T | C | 0.93548 | T | 0.06452 | 4 | 0.12903 |
| 19274 | S26_38547945 | 26 | 38547945 | 24 | T | C | T | 0.72581 | C | 0.27419 | 9 | 0.29032 |
| 19275 | S26_39166275 | 26 | 39166275 | 618330 | T | C | C | 0.90323 | T | 0.09677 | 6 | 0.19355 |
| 19276 | S26_39457867 | 26 | 39457867 | 291592 | T | C | C | 0.59677 | T | 0.40323 | 13 | 0.41935 |

| Site number | SNP name | Chromosome | Physical position | Physical distance from the previous SNP | Reference allele | Alternative allele | Major allele | Major allele frequency | Minor allele | Minor allele frequency | Number heterozygous | Heterozygosity |
|---|---|---|---|---|---|---|---|---|---|---|---|---|
| 19277 | S26_39457897 | 26 | 39457897 | 30 | T | G | G | 0.59677 | T | 0.40323 | 13 | 0.41935 |
| 19278 | S26_40348531 | 26 | 40348531 | 890634 | G | C | G | 0.66129 | C | 0.33871 | 17 | 0.54839 |
| 19279 | S26_40348538 | 26 | 40348538 | 7 | T | C | T | 0.66129 | C | 0.33871 | 17 | 0.54839 |
| 19280 | S26_40348739 | 26 | 40348739 | 201 | T | C | T | 0.8871 | C | 0.1129 | 7 | 0.22581 |
| 19281 | S26_40599641 | 26 | 40599641 | 250902 | A | C | A | 0.93548 | C | 0.06452 | 4 | 0.12903 |
| 19282 | S26_40599807 | 26 | 40599807 | 166 | T | C | T | 0.82258 | C | 0.17742 | 7 | 0.22581 |
| 19283 | S26_41259475 | 26 | 41259475 | 659668 | G | A | A | 0.54839 | G | 0.45161 | 14 | 0.45161 |
| 19284 | S26_41259517 | 26 | 41259517 | 42 | C | T | C | 0.95161 | T | 0.04839 | 3 | 0.09677 |
| 19285 | S26_41259518 | 26 | 41259518 | 1 | G | A | G | 0.67742 | A | 0.32258 | 14 | 0.45161 |
| 19286 | S26_41825437 | 26 | 41825437 | 565919 | T | C | T | 0.75806 | C | 0.24194 | 9 | 0.29032 |
| 19287 | S26_41825454 | 26 | 41825454 | 17 | G | A | G | 0.90323 | A | 0.09677 | 4 | 0.12903 |
| 19288 | S26_41825476 | 26 | 41825476 | 22 | G | A | G | 0.91935 | A | 0.08065 | 5 | 0.16129 |
| 19289 | S26_41825477 | 26 | 41825477 | 1 | C | T | C | 0.8871 | T | 0.1129 | 5 | 0.16129 |
| 19290 | S26_41825495 | 26 | 41825495 | 18 | G | A | G | 0.77419 | A | 0.22581 | 8 | 0.25806 |
| 19291 | S26_41825503 | 26 | 41825503 | 8 | A | G | A | 0.77419 | G | 0.22581 | 8 | 0.25806 |
| 19292 | S26_41825525 | 26 | 41825525 | 22 | G | A | G | 0.79032 | A | 0.20968 | 7 | 0.22581 |
| 19293 | S26_41825565 | 26 | 41825565 | 40 | G | A | G | 0.77419 | A | 0.22581 | 8 | 0.25806 |
| 19294 | S26_42125007 | 26 | 42125007 | 299442 | G | A | G | 0.95161 | A | 0.04839 | 3 | 0.09677 |
| 19295 | S26_42231424 | 26 | 42231424 | 106417 | G | A | G | 0.72581 | A | 0.27419 | 13 | 0.41935 |
| 19296 | S26_42231509 | 26 | 42231509 | 85 | T | C | T | 0.80645 | C | 0.19355 | 10 | 0.32258 |
| 19297 | S26_42618228 | 26 | 42618228 | 386719 | C | G | C | 0.87097 | G | 0.12903 | 4 | 0.12903 |
| 19298 | S26_42618261 | 26 | 42618261 | 33 | C | T | C | 0.87097 | T | 0.12903 | 4 | 0.12903 |
| 19299 | S26_42618404 | 26 | 42618404 | 143 | C | T | C | 0.80645 | T | 0.19355 | 6 | 0.19355 |
| 19300 | S26_42851006 | 26 | 42851006 | 232602 | A | G | A | 0.82258 | G | 0.17742 | 9 | 0.29032 |
| 19301 | S26_42851056 | 26 | 42851056 | 50 | G | C | G | 0.8871 | C | 0.1129 | 7 | 0.22581 |
| 19302 | S26_42851059 | 26 | 42851059 | 3 | T | A | T | 0.75806 | A | 0.24194 | 9 | 0.29032 |
| 19303 | S26_42851068 | 26 | 42851068 | 9 | T | G | T | 0.75806 | G | 0.24194 | 9 | 0.29032 |
| 19304 | S26_42851089 | 26 | 42851089 | 21 | A | G | A | 0.87097 | G | 0.12903 | 8 | 0.25806 |
| 19305 | S26_42851137 | 26 | 42851137 | 48 | T | C | T | 0.90323 | C | 0.09677 | 6 | 0.19355 |
| 19306 | S26_43060721 | 26 | 43060721 | 209584 | T | C | T | 0.91935 | C | 0.08065 | 3 | 0.09677 |
| 19307 | S26_43060722 | 26 | 43060722 | 1 | C | T | C | 0.91935 | T | 0.08065 | 3 | 0.09677 |
| 19308 | S26_43189592 | 26 | 43189592 | 128870 | G | A | A | 0.77419 | G | 0.22581 | 10 | 0.32258 |
| 19309 | S26_43189599 | 26 | 43189599 | 7 | C | A | C | 0.93548 | A | 0.06452 | 4 | 0.12903 |
| 19310 | S26_43420441 | 26 | 43420441 | 230842 | C | T | C | 0.82258 | T | 0.17742 | 9 | 0.29032 |
| 19311 | S26_43420566 | 26 | 43420566 | 125 | C | G | C | 0.79032 | G | 0.20968 | 9 | 0.29032 |
| 19312 | S26_43770705 | 26 | 43770705 | 350139 | A | G | A | 0.80645 | G | 0.19355 | 8 | 0.25806 |
| 19313 | S26_44412302 | 26 | 44412302 | 641597 | G | A | G | 0.66129 | A | 0.33871 | 13 | 0.41935 |
| 19314 | S26_45519189 | 26 | 45519189 | 1106887 | T | G | G | 0.69355 | T | 0.30645 | 13 | 0.41935 |
| 19315 | S26_45519330 | 26 | 45519330 | 141 | T | C | C | 0.64516 | T | 0.35484 | 14 | 0.45161 |
| 19316 | S26_45740935 | 26 | 45740935 | 221605 | C | T | C | 0.93548 | T | 0.06452 | 4 | 0.12903 |
| 19317 | S26_45741046 | 26 | 45741046 | 111 | C | A | C | 0.85484 | A | 0.14516 | 5 | 0.16129 |
| 19318 | S26_45741050 | 26 | 45741050 | 4 | T | G | T | 0.93548 | G | 0.06452 | 4 | 0.12903 |
| 19319 | S26_46946161 | 26 | 46946161 | 1205111 | C | A | C | 0.95161 | A | 0.04839 | 3 | 0.09677 |
| 19320 | S26_46946248 | 26 | 46946248 | 87 | C | T | C | 0.87097 | T | 0.12903 | 4 | 0.12903 |
| 19321 | S26_48333611 | 26 | 48333611 | 1387363 | G | C | C | 0.56452 | G | 0.43548 | 11 | 0.35484 |
| 19322 | S26_48333663 | 26 | 48333663 | 52 | G | A | G | 0.95161 | A | 0.04839 | 3 | 0.09677 |
| 19323 | S26_48454386 | 26 | 48454386 | 120723 | C | A | C | 0.70968 | A | 0.29032 | 14 | 0.45161 |
| 19324 | S26_48454427 | 26 | 48454427 | 41 | A | G | A | 0.8871 | G | 0.1129 | 7 | 0.22581 |
| 19325 | S26_48454439 | 26 | 48454439 | 12 | C | T | C | 0.8871 | T | 0.1129 | 7 | 0.22581 |
| 19326 | S26_49768451 | 26 | 49768451 | 1314012 | C | T | C | 0.56452 | T | 0.43548 | 15 | 0.48387 |
| 19327 | S26_49768504 | 26 | 49768504 | 53 | G | A | G | 0.91935 | A | 0.08065 | 3 | 0.09677 |
| 19328 | S26_49869541 | 26 | 49869541 | 101037 | T | C | T | 0.91935 | C | 0.08065 | 5 | 0.16129 |
| 19329 | S26_49869678 | 26 | 49869678 | 137 | C | A | C | 0.58065 | A | 0.41935 | 12 | 0.3871 |
| 19330 | S26_49869727 | 26 | 49869727 | 49 | A | G | A | 0.91935 | G | 0.08065 | 5 | 0.16129 |

| Site number | SNP name | Chromosome | Physical position | Physical distance from the previous SNP | Reference allele | Alternative allele | Major allele | Major allele frequency | Minor allele | Minor allele frequency | Number heterozygous | Heterozygosity |
|---|---|---|---|---|---|---|---|---|---|---|---|---|
| 19331 | S26_50462923 | 26 | 50462923 | 593196 | T | C | T | 0.72581 | C | 0.27419 | 11 | 0.35484 |
| 19332 | S26_50463019 | 26 | 50463019 | 96 | T | C | T | 0.80645 | C | 0.19355 | 10 | 0.32258 |
| 19333 | S26_51122911 | 26 | 51122911 | 659892 | A | G | A | 0.82258 | G | 0.17742 | 7 | 0.22581 |
| 19334 | S26_51122921 | 26 | 51122921 | 10 | A | T | A | 0.51613 | T | 0.48387 | 16 | 0.51613 |
| 19335 | S27_131237 | 27 | 131237 | 0 | C | T | C | 0.87097 | T | 0.12903 | 8 | 0.25806 |
| 19336 | S27_131320 | 27 | 131320 | 83 | C | G | C | 0.90323 | G | 0.09677 | 4 | 0.12903 |
| 19337 | S27_131326 | 27 | 131326 | 6 | T | A | T | 0.91935 | A | 0.08065 | 5 | 0.16129 |
| 19338 | S27_131402 | 27 | 131402 | 76 | G | C | G | 0.93548 | C | 0.06452 | 4 | 0.12903 |
| 19339 | S27_131439 | 27 | 131439 | 37 | C | T | C | 0.91935 | T | 0.08065 | 3 | 0.09677 |
| 19340 | S27_131468 | 27 | 131468 | 29 | A | C | A | 0.87097 | C | 0.12903 | 8 | 0.25806 |
| 19341 | S27_131479 | 27 | 131479 | 11 | T | C | T | 0.91935 | C | 0.08065 | 5 | 0.16129 |
| 19342 | S27_769864 | 27 | 769864 | 638385 | G | A | A | 0.85484 | G | 0.14516 | 7 | 0.22581 |
| 19343 | S27_848619 | 27 | 848619 | 78755 | G | A | G | 0.93548 | A | 0.06452 | 2 | 0.06452 |
| 19344 | S27_1488971 | 27 | 1488971 | 640352 | G | A | G | 0.74194 | A | 0.25806 | 8 | 0.25806 |
| 19345 | S27_1489003 | 27 | 1489003 | 32 | G | T | G | 0.74194 | T | 0.25806 | 12 | 0.3871 |
| 19346 | S27_1489070 | 27 | 1489070 | 67 | A | G | A | 0.58065 | G | 0.41935 | 16 | 0.51613 |
| 19347 | S27_1489216 | 27 | 1489216 | 146 | G | C | G | 0.90323 | C | 0.09677 | 4 | 0.12903 |
| 19348 | S27_1883328 | 27 | 1883328 | 394112 | A | G | A | 0.54839 | G | 0.45161 | 16 | 0.51613 |
| 19349 | S27_1994232 | 27 | 1994232 | 110904 | G | A | G | 0.75806 | A | 0.24194 | 13 | 0.41935 |
| 19350 | S27_1994248 | 27 | 1994248 | 16 | G | A | G | 0.75806 | A | 0.24194 | 13 | 0.41935 |
| 19351 | S27_2053651 | 27 | 2053651 | 59403 | A | G | A | 0.83871 | G | 0.16129 | 8 | 0.25806 |
| 19352 | S27_2053793 | 27 | 2053793 | 142 | G | A | G | 0.83871 | A | 0.16129 | 8 | 0.25806 |
| 19353 | S27_2198097 | 27 | 2198097 | 144304 | G | T | G | 0.90323 | T | 0.09677 | 6 | 0.19355 |
| 19354 | S27_2198139 | 27 | 2198139 | 42 | C | T | C | 0.93548 | T | 0.06452 | 4 | 0.12903 |
| 19355 | S27_2385734 | 27 | 2385734 | 187595 | T | C | T | 0.56452 | C | 0.43548 | 13 | 0.41935 |
| 19356 | S27_2385735 | 27 | 2385735 | 1 | G | T | G | 0.83871 | T | 0.16129 | 8 | 0.25806 |
| 19357 | S27_2385791 | 27 | 2385791 | 56 | T | C | T | 0.83871 | C | 0.16129 | 8 | 0.25806 |
| 19358 | S27_2385902 | 27 | 2385902 | 111 | C | T | C | 0.85484 | T | 0.14516 | 5 | 0.16129 |
| 19359 | S27_2480186 | 27 | 2480186 | 94284 | G | A | G | 0.90323 | A | 0.09677 | 6 | 0.19355 |
| 19360 | S27_2587728 | 27 | 2587728 | 107542 | C | A | C | 0.8871 | A | 0.1129 | 5 | 0.16129 |
| 19361 | S27_2587904 | 27 | 2587904 | 176 | G | A | G | 0.77419 | A | 0.22581 | 8 | 0.25806 |
| 19362 | S27_2587913 | 27 | 2587913 | 9 | T | C | T | 0.66129 | C | 0.33871 | 11 | 0.35484 |
| 19363 | S27_2722139 | 27 | 2722139 | 134226 | C | T | C | 0.91935 | T | 0.08065 | 5 | 0.16129 |
| 19364 | S27_2722143 | 27 | 2722143 | 4 | C | G | C | 0.8871 | G | 0.1129 | 7 | 0.22581 |
| 19365 | S27_2722319 | 27 | 2722319 | 176 | C | T | C | 0.8871 | T | 0.1129 | 5 | 0.16129 |
| 19366 | S27_3248123 | 27 | 3248123 | 525804 | C | T | C | 0.91935 | T | 0.08065 | 5 | 0.16129 |
| 19367 | S27_3248165 | 27 | 3248165 | 42 | A | G | A | 0.91935 | G | 0.08065 | 5 | 0.16129 |
| 19368 | S27_3248195 | 27 | 3248195 | 30 | A | G | A | 0.91935 | G | 0.08065 | 5 | 0.16129 |
| 19369 | S27_3248262 | 27 | 3248262 | 67 | A | G | A | 0.91935 | G | 0.08065 | 5 | 0.16129 |
| 19370 | S27_3248274 | 27 | 3248274 | 12 | T | C | C | 0.56452 | T | 0.43548 | 11 | 0.35484 |
| 19371 | S27_3248283 | 27 | 3248283 | 9 | C | T | C | 0.82258 | T | 0.17742 | 7 | 0.22581 |
| 19372 | S27_3248294 | 27 | 3248294 | 11 | G | A | G | 0.90323 | A | 0.09677 | 4 | 0.12903 |
| 19373 | S27_3248321 | 27 | 3248321 | 27 | G | A | G | 0.90323 | A | 0.09677 | 4 | 0.12903 |
| 19374 | S27_3248323 | 27 | 3248323 | 2 | G | A | G | 0.51613 | A | 0.48387 | 10 | 0.32258 |
| 19375 | S27_3248355 | 27 | 3248355 | 32 | T | C | T | 0.91935 | C | 0.08065 | 5 | 0.16129 |
| 19376 | S27_3248357 | 27 | 3248357 | 2 | C | T | C | 0.51613 | T | 0.48387 | 10 | 0.32258 |
| 19377 | S27_3296630 | 27 | 3296630 | 48273 | C | T | C | 0.69355 | T | 0.30645 | 15 | 0.48387 |
| 19378 | S27_3296698 | 27 | 3296698 | 68 | C | T | C | 0.91935 | T | 0.08065 | 5 | 0.16129 |
| 19379 | S27_3296728 | 27 | 3296728 | 30 | A | G | G | 0.80645 | A | 0.19355 | 6 | 0.19355 |
| 19380 | S27_3314919 | 27 | 3314919 | 18191 | C | T | C | 0.56452 | T | 0.43548 | 15 | 0.48387 |
| 19381 | S27_3314924 | 27 | 3314924 | 5 | C | A | C | 0.64516 | A | 0.35484 | 10 | 0.32258 |
| 19382 | S27_3314930 | 27 | 3314930 | 6 | A | G | G | 0.51613 | A | 0.48387 | 14 | 0.45161 |
| 19383 | S27_3315084 | 27 | 3315084 | 154 | G | C | G | 0.64516 | C | 0.35484 | 10 | 0.32258 |
| 19384 | S27_3337584 | 27 | 3337584 | 22500 | G | A | G | 0.54839 | A | 0.45161 | 12 | 0.3871 |

| Site number | SNP name | Chromosome | Physical position | Physical distance from the previous SNP | Reference allele | Alternative allele | Major allele | Major allele frequency | Minor allele | Minor allele frequency | Number heterozygous | Heterozygosity |
|---|---|---|---|---|---|---|---|---|---|---|---|---|
| 19385 | S27_3337631 | 27 | 3337631 | 47 | C | A | C | 0.90323 | A | 0.09677 | 4 | 0.12903 |
| 19386 | S27_3337699 | 27 | 3337699 | 68 | C | T | C | 0.67742 | T | 0.32258 | 10 | 0.32258 |
| 19387 | S27_3337737 | 27 | 3337737 | 38 | G | A | G | 0.93548 | A | 0.06452 | 2 | 0.06452 |
| 19388 | S27_3395480 | 27 | 3395480 | 57743 | C | G | C | 0.85484 | G | 0.14516 | 9 | 0.29032 |
| 19389 | S27_3395515 | 27 | 3395515 | 35 | G | A | G | 0.85484 | A | 0.14516 | 9 | 0.29032 |
| 19390 | S27_3510782 | 27 | 3510782 | 115267 | A | G | G | 0.79032 | A | 0.20968 | 11 | 0.35484 |
| 19391 | S27_3649843 | 27 | 3649843 | 139061 | G | A | A | 0.74194 | G | 0.25806 | 10 | 0.32258 |
| 19392 | S27_3654233 | 27 | 3654233 | 4390 | G | T | T | 0.85484 | G | 0.14516 | 7 | 0.22581 |
| 19393 | S27_3672362 | 27 | 3672362 | 18129 | G | C | C | 0.72581 | G | 0.27419 | 15 | 0.48387 |
| 19394 | S27_3672480 | 27 | 3672480 | 118 | C | T | C | 0.87097 | T | 0.12903 | 8 | 0.25806 |
| 19395 | S27_3680622 | 27 | 3680622 | 8142 | G | A | G | 0.79032 | A | 0.20968 | 13 | 0.41935 |
| 19396 | S27_3680637 | 27 | 3680637 | 15 | A | G | G | 0.87097 | A | 0.12903 | 6 | 0.19355 |
| 19397 | S27_3792863 | 27 | 3792863 | 112226 | C | G | C | 0.91935 | G | 0.08065 | 5 | 0.16129 |
| 19398 | S27_3893200 | 27 | 3893200 | 100337 | C | T | C | 0.91935 | T | 0.08065 | 3 | 0.09677 |
| 19399 | S27_3893204 | 27 | 3893204 | 4 | C | T | C | 0.91935 | T | 0.08065 | 3 | 0.09677 |
| 19400 | S27_3893349 | 27 | 3893349 | 145 | A | G | G | 0.8871 | A | 0.1129 | 5 | 0.16129 |
| 19401 | S27_3909796 | 27 | 3909796 | 16447 | C | T | C | 0.90323 | T | 0.09677 | 6 | 0.19355 |
| 19402 | S27_3909863 | 27 | 3909863 | 67 | G | T | G | 0.83871 | T | 0.16129 | 8 | 0.25806 |
| 19403 | S27_3909913 | 27 | 3909913 | 50 | C | T | C | 0.93548 | T | 0.06452 | 4 | 0.12903 |
| 19404 | S27_3909937 | 27 | 3909937 | 24 | A | C | A | 0.82258 | C | 0.17742 | 9 | 0.29032 |
| 19405 | S27_3986507 | 27 | 3986507 | 76570 | T | C | C | 0.6129 | T | 0.3871 | 14 | 0.45161 |
| 19406 | S27_3986508 | 27 | 3986508 | 1 | G | A | G | 0.90323 | A | 0.09677 | 4 | 0.12903 |
| 19407 | S27_3986530 | 27 | 3986530 | 22 | C | A | C | 0.79032 | A | 0.20968 | 9 | 0.29032 |
| 19408 | S27_4154244 | 27 | 4154244 | 167714 | C | T | T | 0.6129 | C | 0.3871 | 20 | 0.64516 |
| 19409 | S27_4159591 | 27 | 4159591 | 5347 | G | A | G | 0.53226 | A | 0.46774 | 17 | 0.54839 |
| 19410 | S27_4474168 | 27 | 4474168 | 314577 | T | G | T | 0.6129 | G | 0.3871 | 16 | 0.51613 |
| 19411 | S27_4509484 | 27 | 4509484 | 35316 | G | A | G | 0.93548 | A | 0.06452 | 4 | 0.12903 |
| 19412 | S27_4578618 | 27 | 4578618 | 69134 | T | C | T | 0.91935 | C | 0.08065 | 3 | 0.09677 |
| 19413 | S27_4578632 | 27 | 4578632 | 14 | C | T | C | 0.75806 | T | 0.24194 | 9 | 0.29032 |
| 19414 | S27_4782459 | 27 | 4782459 | 203827 | T | C | T | 0.91935 | C | 0.08065 | 3 | 0.09677 |
| 19415 | S27_4907933 | 27 | 4907933 | 125474 | G | A | G | 0.58065 | A | 0.41935 | 14 | 0.45161 |
| 19416 | S27_5207057 | 27 | 5207057 | 299124 | A | G | A | 0.91935 | G | 0.08065 | 3 | 0.09677 |
| 19417 | S27_5207203 | 27 | 5207203 | 146 | G | A | G | 0.95161 | A | 0.04839 | 3 | 0.09677 |
| 19418 | S27_5207239 | 27 | 5207239 | 36 | C | T | C | 0.85484 | T | 0.14516 | 5 | 0.16129 |
| 19419 | S27_5257815 | 27 | 5257815 | 50576 | A | G | A | 0.93548 | G | 0.06452 | 4 | 0.12903 |
| 19420 | S27_5257964 | 27 | 5257964 | 149 | C | T | C | 0.90323 | T | 0.09677 | 6 | 0.19355 |
| 19421 | S27_5674410 | 27 | 5674410 | 416446 | C | T | C | 0.95161 | T | 0.04839 | 3 | 0.09677 |
| 19422 | S27_5674430 | 27 | 5674430 | 20 | C | T | C | 0.95161 | T | 0.04839 | 3 | 0.09677 |
| 19423 | S27_5674433 | 27 | 5674433 | 3 | A | C | A | 0.90323 | C | 0.09677 | 6 | 0.19355 |
| 19424 | S27_5674496 | 27 | 5674496 | 63 | A | G | G | 0.8871 | A | 0.1129 | 5 | 0.16129 |
| 19425 | S27_5752744 | 27 | 5752744 | 78248 | C | T | C | 0.77419 | T | 0.22581 | 12 | 0.3871 |
| 19426 | S27_5769836 | 27 | 5769836 | 17092 | G | C | G | 0.72581 | C | 0.27419 | 11 | 0.35484 |
| 19427 | S27_5769873 | 27 | 5769873 | 37 | G | T | G | 0.59677 | T | 0.40323 | 15 | 0.48387 |
| 19428 | S27_5789498 | 27 | 5789498 | 19625 | C | T | C | 0.82258 | T | 0.17742 | 9 | 0.29032 |
| 19429 | S27_6097431 | 27 | 6097431 | 307933 | G | T | G | 0.8871 | T | 0.1129 | 7 | 0.22581 |
| 19430 | S27_6545647 | 27 | 6545647 | 448216 | G | A | G | 0.75806 | A | 0.24194 | 11 | 0.35484 |
| 19431 | S27_6545722 | 27 | 6545722 | 75 | G | A | G | 0.90323 | A | 0.09677 | 6 | 0.19355 |
| 19432 | S27_6545777 | 27 | 6545777 | 55 | T | C | C | 0.95161 | T | 0.04839 | 3 | 0.09677 |
| 19433 | S27_6582896 | 27 | 6582896 | 37119 | G | A | A | 0.64516 | G | 0.35484 | 14 | 0.45161 |
| 19434 | S27_6582909 | 27 | 6582909 | 13 | G | T | G | 0.90323 | T | 0.09677 | 4 | 0.12903 |
| 19435 | S27_6582913 | 27 | 6582913 | 4 | G | A | G | 0.93548 | A | 0.06452 | 4 | 0.12903 |
| 19436 | S27_6582925 | 27 | 6582925 | 12 | G | A | A | 0.54839 | G | 0.45161 | 16 | 0.51613 |
| 19437 | S27_6582955 | 27 | 6582955 | 30 | T | C | C | 0.64516 | T | 0.35484 | 14 | 0.45161 |
| 19438 | S27_6582956 | 27 | 6582956 | 1 | G | A | A | 0.62903 | G | 0.37097 | 15 | 0.48387 |

| Site number | SNP name | Chromosome | Physical position | Physical distance from the previous SNP | Reference allele | Alternative allele | Major allele | Major allele frequency | Minor allele | Minor allele frequency | Number heterozygous | Heterozygosity |
|---|---|---|---|---|---|---|---|---|---|---|---|---|
| 19439 | S27_6710431 | 27 | 6710431 | 127475 | T | C | T | 0.93548 | C | 0.06452 | 4 | 0.12903 |
| 19440 | S27_6710456 | 27 | 6710456 | 25 | G | A | G | 0.93548 | A | 0.06452 | 4 | 0.12903 |
| 19441 | S27_6710493 | 27 | 6710493 | 37 | C | G | C | 0.93548 | G | 0.06452 | 2 | 0.06452 |
| 19442 | S27_6710548 | 27 | 6710548 | 55 | A | G | A | 0.93548 | G | 0.06452 | 4 | 0.12903 |
| 19443 | S27_6764236 | 27 | 6764236 | 53688 | G | A | G | 0.87097 | A | 0.12903 | 6 | 0.19355 |
| 19444 | S27_6764298 | 27 | 6764298 | 62 | C | G | C | 0.87097 | G | 0.12903 | 6 | 0.19355 |
| 19445 | S27_6893401 | 27 | 6893401 | 129103 | C | A | C | 0.91935 | A | 0.08065 | 5 | 0.16129 |
| 19446 | S27_6893450 | 27 | 6893450 | 49 | C | T | C | 0.75806 | T | 0.24194 | 9 | 0.29032 |
| 19447 | S27_6893463 | 27 | 6893463 | 13 | C | T | C | 0.91935 | T | 0.08065 | 5 | 0.16129 |
| 19448 | S27_6893472 | 27 | 6893472 | 9 | A | G | A | 0.91935 | G | 0.08065 | 5 | 0.16129 |
| 19449 | S27_6893488 | 27 | 6893488 | 16 | C | T | C | 0.83871 | T | 0.16129 | 8 | 0.25806 |
| 19450 | S27_6893568 | 27 | 6893568 | 80 | G | C | G | 0.91935 | C | 0.08065 | 5 | 0.16129 |
| 19451 | S27_7136670 | 27 | 7136670 | 243102 | G | C | G | 0.8871 | C | 0.1129 | 5 | 0.16129 |
| 19452 | S27_7136845 | 27 | 7136845 | 175 | T | C | T | 0.8871 | C | 0.1129 | 5 | 0.16129 |
| 19453 | S27_7275352 | 27 | 7275352 | 138507 | T | C | C | 0.85484 | T | 0.14516 | 5 | 0.16129 |
| 19454 | S27_7751094 | 27 | 7751094 | 475742 | T | A | T | 0.93548 | A | 0.06452 | 4 | 0.12903 |
| 19455 | S27_7751168 | 27 | 7751168 | 74 | T | C | T | 0.80645 | C | 0.19355 | 10 | 0.32258 |
| 19456 | S27_7751195 | 27 | 7751195 | 27 | G | A | A | 0.93548 | G | 0.06452 | 4 | 0.12903 |
| 19457 | S27_8133801 | 27 | 8133801 | 382606 | G | C | G | 0.67742 | C | 0.32258 | 12 | 0.3871 |
| 19458 | S27_8133884 | 27 | 8133884 | 83 | C | T | T | 0.80645 | C | 0.19355 | 8 | 0.25806 |
| 19459 | S27_8283930 | 27 | 8283930 | 150046 | C | T | C | 0.91935 | T | 0.08065 | 5 | 0.16129 |
| 19460 | S27_8283973 | 27 | 8283973 | 43 | G | C | G | 0.58065 | C | 0.41935 | 14 | 0.45161 |
| 19461 | S27_8692981 | 27 | 8692981 | 409008 | A | G | A | 0.91935 | G | 0.08065 | 5 | 0.16129 |
| 19462 | S27_8818887 | 27 | 8818887 | 125906 | T | C | T | 0.69355 | C | 0.30645 | 13 | 0.41935 |
| 19463 | S27_8926676 | 27 | 8926676 | 107789 | C | T | C | 0.72581 | T | 0.27419 | 13 | 0.41935 |
| 19464 | S27_9048194 | 27 | 9048194 | 121518 | C | T | C | 0.91935 | T | 0.08065 | 5 | 0.16129 |
| 19465 | S27_9048344 | 27 | 9048344 | 150 | T | G | T | 0.74194 | G | 0.25806 | 14 | 0.45161 |
| 19466 | S27_9160317 | 27 | 9160317 | 111973 | A | G | A | 0.87097 | G | 0.12903 | 6 | 0.19355 |
| 19467 | S27_9309593 | 27 | 9309593 | 149276 | G | A | G | 0.83871 | A | 0.16129 | 10 | 0.32258 |
| 19468 | S27_9309635 | 27 | 9309635 | 42 | T | G | G | 0.56452 | T | 0.43548 | 15 | 0.48387 |
| 19469 | S27_9309648 | 27 | 9309648 | 13 | C | T | C | 0.54839 | T | 0.45161 | 18 | 0.58065 |
| 19470 | S27_9309737 | 27 | 9309737 | 89 | G | A | G | 0.91935 | A | 0.08065 | 5 | 0.16129 |
| 19471 | S27_9373188 | 27 | 9373188 | 63451 | G | A | G | 0.93548 | A | 0.06452 | 2 | 0.06452 |
| 19472 | S27_9426294 | 27 | 9426294 | 53106 | T | C | T | 0.93548 | C | 0.06452 | 4 | 0.12903 |
| 19473 | S27_9426436 | 27 | 9426436 | 142 | T | C | T | 0.93548 | C | 0.06452 | 4 | 0.12903 |
| 19474 | S27_9548743 | 27 | 9548743 | 122307 | C | T | T | 0.70968 | C | 0.29032 | 14 | 0.45161 |
| 19475 | S27_9548830 | 27 | 9548830 | 87 | T | C | C | 0.70968 | T | 0.29032 | 14 | 0.45161 |
| 19476 | S27_9578801 | 27 | 9578801 | 29971 | T | C | T | 0.85484 | C | 0.14516 | 9 | 0.29032 |
| 19477 | S27_9578804 | 27 | 9578804 | 3 | T | C | T | 0.85484 | C | 0.14516 | 9 | 0.29032 |
| 19478 | S27_9578836 | 27 | 9578836 | 32 | A | G | A | 0.85484 | G | 0.14516 | 9 | 0.29032 |
| 19479 | S27_9578944 | 27 | 9578944 | 108 | G | C | G | 0.85484 | C | 0.14516 | 9 | 0.29032 |
| 19480 | S27_9704559 | 27 | 9704559 | 125615 | C | G | C | 0.6129 | G | 0.3871 | 14 | 0.45161 |
| 19481 | S27_9704650 | 27 | 9704650 | 91 | G | C | G | 0.85484 | C | 0.14516 | 7 | 0.22581 |
| 19482 | S27_9750017 | 27 | 9750017 | 45367 | G | A | A | 0.54839 | G | 0.45161 | 16 | 0.51613 |
| 19483 | S27_9750130 | 27 | 9750130 | 113 | C | T | T | 0.93548 | C | 0.06452 | 4 | 0.12903 |
| 19484 | S27_9936478 | 27 | 9936478 | 186348 | G | T | G | 0.87097 | T | 0.12903 | 6 | 0.19355 |
| 19485 | S27_9936479 | 27 | 9936479 | 1 | T | C | T | 0.95161 | C | 0.04839 | 3 | 0.09677 |
| 19486 | S27_9936537 | 27 | 9936537 | 58 | C | G | C | 0.95161 | G | 0.04839 | 3 | 0.09677 |
| 19487 | S27_9936545 | 27 | 9936545 | 8 | G | A | G | 0.93548 | A | 0.06452 | 4 | 0.12903 |
| 19488 | S27_9936579 | 27 | 9936579 | 34 | C | T | C | 0.95161 | T | 0.04839 | 3 | 0.09677 |
| 19489 | S27_9936621 | 27 | 9936621 | 42 | C | G | C | 0.87097 | G | 0.12903 | 6 | 0.19355 |
| 19490 | S27_9936639 | 27 | 9936639 | 18 | C | G | C | 0.91935 | G | 0.08065 | 5 | 0.16129 |
| 19491 | S27_9936658 | 27 | 9936658 | 19 | A | T | T | 0.83871 | A | 0.16129 | 6 | 0.19355 |
| 19492 | S27_9953145 | 27 | 9953145 | 16487 | A | G | A | 0.58065 | G | 0.41935 | 14 | 0.45161 |

| Site number | SNP name | Chromosome | Physical position | Physical distance from the previous SNP | Reference allele | Alternative allele | Major allele | Major allele frequency | Minor allele | Minor allele frequency | Number heterozygous | Heterozygosity |
|---|---|---|---|---|---|---|---|---|---|---|---|---|
| 19493 | S27_10011148 | 27 | 10011148 | 58003 | G | A | G | 0.67742 | A | 0.32258 | 10 | 0.32258 |
| 19494 | S27_10011258 | 27 | 10011258 | 110 | G | C | G | 0.8871 | C | 0.1129 | 7 | 0.22581 |
| 19495 | S27_10011294 | 27 | 10011294 | 36 | T | C | T | 0.95161 | C | 0.04839 | 3 | 0.09677 |
| 19496 | S27_10011308 | 27 | 10011308 | 14 | C | T | C | 0.93548 | T | 0.06452 | 4 | 0.12903 |
| 19497 | S27_10011310 | 27 | 10011310 | 2 | A | G | A | 0.85484 | G | 0.14516 | 5 | 0.16129 |
| 19498 | S27_10040411 | 27 | 10040411 | 29101 | A | C | A | 0.70968 | C | 0.29032 | 10 | 0.32258 |
| 19499 | S27_10040464 | 27 | 10040464 | 53 | A | G | A | 0.93548 | G | 0.06452 | 4 | 0.12903 |
| 19500 | S27_10040500 | 27 | 10040500 | 36 | C | T | C | 0.70968 | T | 0.29032 | 10 | 0.32258 |
| 19501 | S27_10040519 | 27 | 10040519 | 19 | G | A | G | 0.70968 | A | 0.29032 | 10 | 0.32258 |
| 19502 | S27_10040553 | 27 | 10040553 | 34 | C | T | C | 0.70968 | T | 0.29032 | 10 | 0.32258 |
| 19503 | S27_10040558 | 27 | 10040558 | 5 | G | A | G | 0.93548 | A | 0.06452 | 4 | 0.12903 |
| 19504 | S27_10040561 | 27 | 10040561 | 3 | A | C | A | 0.70968 | C | 0.29032 | 10 | 0.32258 |
| 19505 | S27_10087493 | 27 | 10087493 | 46932 | C | T | C | 0.91935 | T | 0.08065 | 5 | 0.16129 |
| 19506 | S27_10087523 | 27 | 10087523 | 30 | T | C | C | 0.79032 | T | 0.20968 | 9 | 0.29032 |
| 19507 | S27_10087602 | 27 | 10087602 | 79 | G | C | G | 0.82258 | C | 0.17742 | 9 | 0.29032 |
| 19508 | S27_10341257 | 27 | 10341257 | 253655 | T | C | T | 0.79032 | C | 0.20968 | 13 | 0.41935 |
| 19509 | S27_10341258 | 27 | 10341258 | 1 | G | A | G | 0.95161 | A | 0.04839 | 3 | 0.09677 |
| 19510 | S27_10341318 | 27 | 10341318 | 60 | G | C | G | 0.91935 | C | 0.08065 | 5 | 0.16129 |
| 19511 | S27_10385015 | 27 | 10385015 | 43697 | G | A | A | 0.58065 | G | 0.41935 | 14 | 0.45161 |
| 19512 | S27_10385114 | 27 | 10385114 | 99 | C | A | C | 0.85484 | A | 0.14516 | 7 | 0.22581 |
| 19513 | S27_10385219 | 27 | 10385219 | 105 | C | G | C | 0.83871 | G | 0.16129 | 6 | 0.19355 |
| 19514 | S27_10423634 | 27 | 10423634 | 38415 | G | A | G | 0.91935 | A | 0.08065 | 5 | 0.16129 |
| 19515 | S27_10423678 | 27 | 10423678 | 44 | G | A | G | 0.95161 | A | 0.04839 | 3 | 0.09677 |
| 19516 | S27_10423686 | 27 | 10423686 | 8 | C | T | C | 0.54839 | T | 0.45161 | 12 | 0.3871 |
| 19517 | S27_10423821 | 27 | 10423821 | 135 | T | C | C | 0.69355 | T | 0.30645 | 11 | 0.35484 |
| 19518 | S27_10474357 | 27 | 10474357 | 50536 | C | T | T | 0.56452 | C | 0.43548 | 19 | 0.6129 |
| 19519 | S27_10634754 | 27 | 10634754 | 160397 | G | C | G | 0.93548 | C | 0.06452 | 4 | 0.12903 |
| 19520 | S27_10634822 | 27 | 10634822 | 68 | T | C | C | 0.90323 | T | 0.09677 | 6 | 0.19355 |
| 19521 | S27_10869143 | 27 | 10869143 | 234321 | C | A | C | 0.79032 | A | 0.20968 | 13 | 0.41935 |
| 19522 | S27_10869158 | 27 | 10869158 | 15 | T | G | T | 0.80645 | G | 0.19355 | 12 | 0.3871 |
| 19523 | S27_10878103 | 27 | 10878103 | 8945 | C | T | C | 0.93548 | T | 0.06452 | 4 | 0.12903 |
| 19524 | S27_10937715 | 27 | 10937715 | 59612 | A | G | A | 0.8871 | G | 0.1129 | 7 | 0.22581 |
| 19525 | S27_10937721 | 27 | 10937721 | 6 | C | T | C | 0.90323 | T | 0.09677 | 6 | 0.19355 |
| 19526 | S27_10937793 | 27 | 10937793 | 72 | C | T | C | 0.93548 | T | 0.06452 | 4 | 0.12903 |
| 19527 | S27_10937854 | 27 | 10937854 | 61 | C | T | C | 0.79032 | T | 0.20968 | 13 | 0.41935 |
| 19528 | S27_10955056 | 27 | 10955056 | 17202 | T | C | T | 0.90323 | C | 0.09677 | 6 | 0.19355 |
| 19529 | S27_10964258 | 27 | 10964258 | 9202 | G | A | G | 0.75806 | A | 0.24194 | 15 | 0.48387 |
| 19530 | S27_11097336 | 27 | 11097336 | 133078 | A | T | T | 0.87097 | A | 0.12903 | 6 | 0.19355 |
| 19531 | S27_11097586 | 27 | 11097586 | 250 | G | A | G | 0.93548 | A | 0.06452 | 4 | 0.12903 |
| 19532 | S27_11722920 | 27 | 11722920 | 625334 | C | T | C | 0.95161 | T | 0.04839 | 3 | 0.09677 |
| 19533 | S27_12083736 | 27 | 12083736 | 360816 | C | T | T | 0.82258 | C | 0.17742 | 7 | 0.22581 |
| 19534 | S27_12364556 | 27 | 12364556 | 280820 | T | C | C | 0.79032 | T | 0.20968 | 7 | 0.22581 |
| 19535 | S27_12859534 | 27 | 12859534 | 494978 | T | C | T | 0.79032 | C | 0.20968 | 9 | 0.29032 |
| 19536 | S27_13174456 | 27 | 13174456 | 314922 | T | C | T | 0.69355 | C | 0.30645 | 13 | 0.41935 |
| 19537 | S27_13174580 | 27 | 13174580 | 124 | C | A | C | 0.90323 | A | 0.09677 | 6 | 0.19355 |
| 19538 | S27_13428061 | 27 | 13428061 | 253481 | C | T | T | 0.58065 | C | 0.41935 | 18 | 0.58065 |
| 19539 | S27_13428164 | 27 | 13428164 | 103 | G | A | G | 0.82258 | A | 0.17742 | 11 | 0.35484 |
| 19540 | S27_13428174 | 27 | 13428174 | 10 | T | C | T | 0.74194 | C | 0.25806 | 14 | 0.45161 |
| 19541 | S27_13428180 | 27 | 13428180 | 6 | G | A | G | 0.77419 | A | 0.22581 | 14 | 0.45161 |
| 19542 | S27_13428191 | 27 | 13428191 | 11 | T | C | T | 0.58065 | C | 0.41935 | 18 | 0.58065 |
| 19543 | S27_13428192 | 27 | 13428192 | 1 | G | A | G | 0.79032 | A | 0.20968 | 13 | 0.41935 |
| 19544 | S27_13428217 | 27 | 13428217 | 25 | A | G | A | 0.66129 | G | 0.33871 | 15 | 0.48387 |
| 19545 | S27_13547201 | 27 | 13547201 | 118984 | G | A | G | 0.85484 | A | 0.14516 | 9 | 0.29032 |
| 19546 | S27_13547300 | 27 | 13547300 | 99 | A | G | G | 0.54839 | A | 0.45161 | 16 | 0.51613 |

| Site number | SNP name | Chromosome | Physical position | Physical distance from the previous SNP | Reference allele | Alternative allele | Major allele | Major allele frequency | Minor allele | Minor allele frequency | Number heterozygous | Heterozygosity |
|---|---|---|---|---|---|---|---|---|---|---|---|---|
| 19547 | S27_13547428 | 27 | 13547428 | 128 | T | C | C | 0.54839 | T | 0.45161 | 16 | 0.51613 |
| 19548 | S27_14715362 | 27 | 14715362 | 1167934 | G | A | G | 0.64516 | A | 0.35484 | 14 | 0.45161 |
| 19549 | S27_14715469 | 27 | 14715469 | 107 | C | G | C | 0.53226 | G | 0.46774 | 17 | 0.54839 |
| 19550 | S27_15181619 | 27 | 15181619 | 466150 | G | A | G | 0.8871 | A | 0.1129 | 7 | 0.22581 |
| 19551 | S27_15752452 | 27 | 15752452 | 570833 | G | A | G | 0.83871 | A | 0.16129 | 8 | 0.25806 |
| 19552 | S27_15752539 | 27 | 15752539 | 87 | C | T | C | 0.83871 | T | 0.16129 | 8 | 0.25806 |
| 19553 | S27_15752559 | 27 | 15752559 | 20 | A | G | A | 0.83871 | G | 0.16129 | 8 | 0.25806 |
| 19554 | S27_15912926 | 27 | 15912926 | 160367 | T | C | T | 0.93548 | C | 0.06452 | 4 | 0.12903 |
| 19555 | S27_15913067 | 27 | 15913067 | 141 | C | T | T | 0.90323 | C | 0.09677 | 6 | 0.19355 |
| 19556 | S27_15998214 | 27 | 15998214 | 85147 | T | G | G | 0.87097 | T | 0.12903 | 4 | 0.12903 |
| 19557 | S27_16293315 | 27 | 16293315 | 295101 | C | T | C | 0.54839 | T | 0.45161 | 14 | 0.45161 |
| 19558 | S27_16293397 | 27 | 16293397 | 82 | C | G | C | 0.77419 | G | 0.22581 | 10 | 0.32258 |
| 19559 | S27_16472143 | 27 | 16472143 | 178746 | T | G | G | 0.64516 | T | 0.35484 | 12 | 0.3871 |
| 19560 | S27_16472240 | 27 | 16472240 | 97 | T | G | T | 0.91935 | G | 0.08065 | 5 | 0.16129 |
| 19561 | S27_16472319 | 27 | 16472319 | 79 | C | G | C | 0.77419 | G | 0.22581 | 14 | 0.45161 |
| 19562 | S27_16725504 | 27 | 16725504 | 253185 | A | G | A | 0.56452 | G | 0.43548 | 13 | 0.41935 |
| 19563 | S27_16725760 | 27 | 16725760 | 256 | G | C | G | 0.59677 | C | 0.40323 | 15 | 0.48387 |
| 19564 | S27_16748630 | 27 | 16748630 | 22870 | G | A | G | 0.64516 | A | 0.35484 | 16 | 0.51613 |
| 19565 | S27_16748736 | 27 | 16748736 | 106 | C | T | C | 0.93548 | T | 0.06452 | 4 | 0.12903 |
| 19566 | S27_16748742 | 27 | 16748742 | 6 | G | A | G | 0.64516 | A | 0.35484 | 16 | 0.51613 |
| 19567 | S27_16821840 | 27 | 16821840 | 73098 | T | C | T | 0.58065 | C | 0.41935 | 14 | 0.45161 |
| 19568 | S27_16822046 | 27 | 16822046 | 206 | A | C | A | 0.93548 | C | 0.06452 | 4 | 0.12903 |
| 19569 | S27_16995523 | 27 | 16995523 | 173477 | A | G | A | 0.62903 | G | 0.37097 | 15 | 0.48387 |
| 19570 | S27_17199405 | 27 | 17199405 | 203882 | C | G | C | 0.91935 | G | 0.08065 | 5 | 0.16129 |
| 19571 | S27_17199482 | 27 | 17199482 | 77 | C | T | C | 0.93548 | T | 0.06452 | 4 | 0.12903 |
| 19572 | S27_17341092 | 27 | 17341092 | 141610 | T | C | T | 0.66129 | C | 0.33871 | 11 | 0.35484 |
| 19573 | S27_17341121 | 27 | 17341121 | 29 | G | A | G | 0.90323 | A | 0.09677 | 6 | 0.19355 |
| 19574 | S27_17341310 | 27 | 17341310 | 189 | C | T | C | 0.91935 | T | 0.08065 | 5 | 0.16129 |
| 19575 | S27_17402347 | 27 | 17402347 | 61037 | A | G | A | 0.66129 | G | 0.33871 | 15 | 0.48387 |
| 19576 | S27_17402378 | 27 | 17402378 | 31 | C | G | C | 0.54839 | G | 0.45161 | 20 | 0.64516 |
| 19577 | S27_17497313 | 27 | 17497313 | 94935 | G | A | G | 0.8871 | A | 0.1129 | 5 | 0.16129 |
| 19578 | S27_17497482 | 27 | 17497482 | 169 | G | A | A | 0.53226 | G | 0.46774 | 17 | 0.54839 |
| 19579 | S27_17503517 | 27 | 17503517 | 6035 | T | G | T | 0.91935 | G | 0.08065 | 5 | 0.16129 |
| 19580 | S27_17791652 | 27 | 17791652 | 288135 | T | C | C | 0.82258 | T | 0.17742 | 11 | 0.35484 |
| 19581 | S27_17791802 | 27 | 17791802 | 150 | G | T | G | 0.87097 | T | 0.12903 | 6 | 0.19355 |
| 19582 | S27_17984560 | 27 | 17984560 | 192758 | A | G | G | 0.87097 | A | 0.12903 | 8 | 0.25806 |
| 19583 | S27_17984667 | 27 | 17984667 | 107 | G | A | A | 0.56452 | G | 0.43548 | 11 | 0.35484 |
| 19584 | S27_17984698 | 27 | 17984698 | 31 | G | T | G | 0.72581 | T | 0.27419 | 13 | 0.41935 |
| 19585 | S27_18100891 | 27 | 18100891 | 116193 | A | G | G | 0.66129 | A | 0.33871 | 17 | 0.54839 |
| 19586 | S27_18100892 | 27 | 18100892 | 1 | A | T | A | 0.95161 | T | 0.04839 | 3 | 0.09677 |
| 19587 | S27_18100893 | 27 | 18100893 | 1 | C | T | C | 0.93548 | T | 0.06452 | 4 | 0.12903 |
| 19588 | S27_18509768 | 27 | 18509768 | 408875 | C | G | C | 0.51613 | G | 0.48387 | 20 | 0.64516 |
| 19589 | S27_18509824 | 27 | 18509824 | 56 | A | T | A | 0.8871 | T | 0.1129 | 5 | 0.16129 |
| 19590 | S27_19108537 | 27 | 19108537 | 598713 | A | T | A | 0.85484 | T | 0.14516 | 9 | 0.29032 |
| 19591 | S27_19108589 | 27 | 19108589 | 52 | A | G | A | 0.70968 | G | 0.29032 | 12 | 0.3871 |
[truncated: 234,783 more chars]
