# Supplementary material for: Detection of single nucleotide polymorphisms associated with litter size in goats using genotyping-by-sequencing and association analysis
Source: Anim Biosci. 2025 Jan 24;38(8):1580–93. doi: 10.5713/ab.24.0533 (PMC12229939; doi:10.5713/ab.24.0533)
Supplement: Supplementary file 5 [file ab-24-0533-Supplementary-5.pdf]

Supplement 5. Distribution of 21,665 SNPs on each chromosome

Chromosome	Number of SNP	First SNP position	Last SNP position	Length of chromosome (megabase)	SNP density per megabase	Minor allele frequency
1	1240	64056	156928752	157.40	7.88	0.29
2	942	179076	136474235	136.51	6.90	0.28
3	888	127902	118859849	120.04	7.40	0.29
4	872	332753	120644141	120.73	7.22	0.28
5	984	88181	118106563	119.02	8.27	0.29
6	889	1146904	117557119	117.64	7.56	0.29
7	806	328852	107947812	108.43	7.43	0.28
8	836	700180	112569438	112.67	7.42	0.30
9	683	152229	91378722	91.57	7.46	0.29
10	975	320869	100607256	101.09	9.65	0.29
11	806	97671	106131757	106.23	7.59	0.29
12	645	568871	86997016	87.28	7.39	0.29
13	784	43681	82624363	83.03	9.44	0.28
14	798	346139	94269460	94.67	8.43	0.29
15	665	51518	81522154	81.90	8.12	0.28
16	666	39221	78785858	79.37	8.39	0.30
17	648	54640	71046574	71.14	9.11	0.28
18	678	28142	66967387	67.28	10.08	0.30
19	672	319176	62492081	62.52	10.75	0.29
20	721	61650	70873378	71.78	10.04	0.29
21	696	442032	69281042	69.43	10.03	0.28
22	524	117539	60172822	60.28	8.69	0.28
23	455	98775	47380266	48.87	9.31	0.30
24	631	165245	62223346	62.31	10.13	0.30
25	448	36353	40655272	42.86	10.45	0.29
26	382	44890	51122921	51.42	7.43	0.28
27	533	131237	44541831	44.71	11.92	0.28
28	388	71585	44536783	44.67	8.69	0.31
29	453	102418	50815915	51.33	8.82	0.28
LWLT01000021.1	355	3184361	65958027	-	-	0.31
LWLT01000027.1	246	926224	49414983	-	-	0.30
LWLT01000032.1	2	127048	147293	-	-	0.37
LWLT01000034.1	1	319647	319647	-	-	0.35
LWLT01000035.1	4	16145	174842	-	-	0.18
LWLT01000039.1	29	310532	615444	-	-	0.34
LWLT01000041.1	5	136782	308429	-	-	0.33
LWLT01000042.1	4	273052	349408	-	-	0.25
LWLT01000043.1	16	25894	504867	-	-	0.27
LWLT01000044.1	6	152579	290783	-	-	0.32
LWLT01000046.1	14	34643	395132	-	-	0.25
LWLT01000055.1	1	47562	47562	-	-	0.10
LWLT01000060.1	1	77269	77269	-	-	0.35
LWLT01000061.1	3	72979	73191	-	-	0.20
LWLT01000062.1	1	105829	105829	-	-	0.10
LWLT01000071.1	2	130740	130807	-	-	0.23
LWLT01000075.1	2	48580	48614	-	-	0.29
LWLT01000077.1	2	147540	147678	-	-	0.31
LWLT01000078.1	1	112304	112304	-	-	0.23
LWLT01000082.1	1	161628	161628	-	-	0.39
LWLT01000089.1	1	71363	71363	-	-	0.42
LWLT01000102.1	1	6030	6030	-	-	0.10
LWLT01000103.1	1	118721	118721	-	-	0.61
LWLT01000105.1	2	91787	91842	-	-	0.16
LWLT01000110.1	1	36592	36592	-	-	0.42
LWLT01000117.1	6	100849	101050	-	-	0.28
LWLT01000118.1	5	33328	68314	-	-	0.43
LWLT01000122.1	1	69318	69318	-	-	0.26
LWLT01000130.1	1	36566	36566	-	-	0.39
LWLT01000137.1	5	50244	50485	-	-	0.30
LWLT01000138.1	1	45886	45886	-	-	0.58
LWLT01000146.1	2	61801	61879	-	-	0.27
LWLT01000148.1	1	7152	7152	-	-	0.26
LWLT01000153.1	4	32368	37967	-	-	0.43
LWLT01000155.1	1	62267	62267	-	-	0.48
LWLT01000169.1	3	37560	37631	-	-	0.44
LWLT01000187.1	3	54006	54151	-	-	0.41
LWLT01000195.1	2	14773	31886	-	-	0.34
LWLT01000196.1	2	14433	14497	-	-	0.15
LWLT01000200.1	2	32599	38079	-	-	0.45
LWLT01000208.1	6	21449	26590	-	-	0.44
LWLT01000211.1	8	28600	31841	-	-	0.23
LWLT01000225.1	21	7663	27004	-	-	0.30
LWLT01000227.1	3	38526	38560	-	-	0.24
LWLT01000235.1	1	13479	13479	-	-	0.58
LWLT01000241.1	6	12788	12957	-	-	0.19
LWLT01000243.1	8	8838	33409	-	-	0.38
LWLT01000250.1	2	24190	24221	-	-	0.13
LWLT01000251.1	8	21054	21279	-	-	0.37

Chromosome	Number of SNP	First SNP position	Last SNP position	Length of chromosome (megabase)	SNP density per megabase	Minor allele frequency
LWLT01000260.1	4	9604	9756	-	-	0.31
LWLT01000272.1	8	693	26803	-	-	0.36
LWLT01000288.1	1	10494	10494	-	-	0.16
LWLT01000308.1	3	26530	26611	-	-	0.29
LWLT01000328.1	14	10065	28048	-	-	0.35
LWLT01000356.1	2	25393	25604	-	-	0.47
LWLT01000378.1	2	20265	20393	-	-	0.13
LWLT01000401.1	1	6959	6959	-	-	0.19
LWLT01000530.1	2	4194	4368	-	-	0.19
LWLT01000553.1	2	2686	2785	-	-	0.39
LWLT01000690.1	1	5209	5209	-	-	0.10
LWLT01000701.1	1	9511	9511	-	-	0.58
LWLT01000718.1	10	2067	16191	-	-	0.37
LWLT01000735.1	2	10494	20832	-	-	0.31
LWLT01000765.1	5	23500	30036	-	-	0.27
LWLT01000846.1	1	18966	18966	-	-	0.13
LWLT01000875.1	4	12424	12589	-	-	0.33
LWLT01000877.1	1	13815	13815	-	-	0.58
LWLT01001028.1	1	5737	5737	-	-	0.10
LWLT01001045.1	2	15398	15512	-	-	0.18
LWLT01001057.1	1	14245	14245	-	-	0.16
LWLT01001090.1	2	6927	7018	-	-	0.35
LWLT01001116.1	2	15484	15536	-	-	0.45
LWLT01001223.1	4	15657	15667	-	-	0.10
LWLT01001266.1	1	1797	1797	-	-	0.19
LWLT01001439.1	3	11453	11628	-	-	0.23
LWLT01001477.1	1	8403	8403	-	-	0.13
LWLT01001544.1	1	3950	3950	-	-	0.06
LWLT01001563.1	2	9015	9222	-	-	0.29
LWLT01001658.1	1	19514	19514	-	-	0.10
LWLT01001784.1	2	6770	6883	-	-	0.29
LWLT01001785.1	4	2629	2651	-	-	0.37
LWLT01001826.1	1	19796	19796	-	-	0.29
LWLT01002427.1	1	16133	16133	-	-	0.13
LWLT01002461.1	1	19232	19232	-	-	0.32
LWLT01002520.1	1	17569	17569	-	-	0.19
LWLT01002728.1	5	15977	16137	-	-	0.23
LWLT01002966.1	2	7331	7353	-	-	0.45
LWLT01003016.1	1	9593	9593	-	-	0.10
LWLT01003382.1	4	10546	10679	-	-	0.17
LWLT01003531.1	1	1637	1637	-	-	0.13
LWLT01003811.1	3	7140	7369	-	-	0.38
LWLT01003990.1	4	461	583	-	-	0.26
LWLT01004510.1	3	12260	12419	-	-	0.34
LWLT01004605.1	5	4670	11634	-	-	0.26
LWLT01004969.1	5	9971	10118	-	-	0.28
LWLT01005748.1	1	11475	11475	-	-	0.13
LWLT01006284.1	3	3844	3999	-	-	0.39
LWLT01007013.1	2	5608	5730	-	-	0.37
LWLT01007180.1	9	2049	2261	-	-	0.34
LWLT01007188.1	1	7881	7881	-	-	0.23
LWLT01007314.1	3	9857	9882	-	-	0.24
LWLT01009519.1	4	9862	9975	-	-	0.40
LWLT01009561.1	1	1604	1604	-	-	0.29
LWLT01017456.1	1	3204	3204	-	-	0.42
