# Supplementary material for: Detection of single nucleotide polymorphisms associated with litter size in goats using genotyping-by-sequencing and association analysis
Source: Anim Biosci. 2025 Jan 24;38(8):1580–93. doi: 10.5713/ab.24.0533 (PMC12229939; doi:10.5713/ab.24.0533)
Supplement: Supplementary file 6 [file ab-24-0533-Supplementary-6.pdf]

Supplement 6. Principal components of the 31 female goats analyzed in this study

Sample ID	Princpal component 1	Princpal component 2	Princpal component 3	Princpal component 4	Color of the dot in Figure 2
NG201_1	-11.478	-17.120	-2.676179	4.6557465	Darkgreen
NG205_3	-6.652	-1.930	-14.318657	-6.4357038	Limegreen
NG213_6	-11.584	-19.142	3.9323559	6.1489296	Pink
NG220_7	9.374	6.606	-6.2482076	-0.86697817	Orange
NG221_8	9.825	-0.457	1.587078	4.57326	Gray
NG222_9	0.010	3.657	13.122806	-9.174079	Limegreen
NG223_10	9.097	-1.412	4.20403	-12.720037	Pink
NG225_11	-12.026	-16.968	-2.0810208	6.1068	Orange
NG227_13	21.462	2.121	3.288553	18.009914	Red
NG229_14	-10.692	-15.852	-12.451503	-3.6952567	Limegreen
NG234_17	14.411	-0.023	3.6775749	10.817213	Black
NG239_19	2.618	-0.799	15.641207	-12.461519	Pink
NG240_20	13.273	2.315	-0.9271757	7.2103453	Purple
NG242_22	1.912	1.926	5.760244	9.163917	Pink
NG247_24	-19.848	19.549	-0.77800435	5.476991	Blue
NG249_25	-13.848	13.094	0.7485412	0.9651957	Limegreen
NG251_26	-15.575	15.440	1.776445	3.8692641	Orange
NG253_27	19.347	2.326	7.8061743	10.023909	Magenta
NG254_28	-13.847	13.072	0.6369642	0.6119702	Magenta
NG258_29	3.485	0.054	12.777998	-18.685673	Magenta
NG261_31	5.706	-5.022	-24.318645	-11.100186	Brown
NG262_32	-3.119	2.622	-22.338097	-10.652201	Limegreen
NG263_33	9.090	2.487	8.365449	-14.501847	Magenta
NG265_34	19.243	1.980	-8.744693	-0.44412547	Pink
NG268_35	-8.967	-17.944	11.42918	-0.72494125	Pink
NG269_36	-15.104	-27.136	2.658413	9.625572	Pink
NG276_38	19.839	2.065	-10.004676	-1.2583728	Purple
NG284_42	0.054	-2.253	12.561288	-12.625	Black
NG289_47	-17.434	17.556	-2.9748404	4.0328665	Lightblue
NG292_49	19.952	1.165	-3.913963	7.781493	Yellow
NG301_53	-18.523	18.022	1.8013611	6.2725334	Cyan
