# Supplementary material for: Detection of single nucleotide polymorphisms associated with litter size in goats using genotyping-by-sequencing and association analysis
Source: Anim Biosci. 2025 Jan 24;38(8):1580–93. doi: 10.5713/ab.24.0533 (PMC12229939; doi:10.5713/ab.24.0533)
Supplement: Supplementary file 7 [file ab-24-0533-Supplementary-7.pdf]

Supplement 7. Kinship of the 31 female goats analyzed in this study

Cluster	Sample ID	NG227_13	NG253_27	NG240_20	NG234_17	NG221_8	NG220_7	NG276_38	NG265_34	NG292_49
1	NG227_13	1.1227038	0.3924498	0.1435127	0.15468849	0.06358256	0.03725121	0.08173993	0.12042897	0.3682626
1	NG253_27	0.3924498	1.0301598	0.0975649	0.11522545	0.048113227	0.05290882	0.088318855	0.11014745	0.17851485
1	NG240_20	0.1435127	0.0975649	0.95298564	0.20382117	0.016416244	0.00597261	0.01771319	0.0727763	0.07629586
1	NG234_17	0.15468849	0.11522545	0.20382117	0.91551536	0.38182318	-0.03359502	0.029699577	0.06887498	0.07596116
1	NG221_8	0.06358256	0.048113227	0.016416244	0.38182318	0.83479035	0.016180897	0.066019595	0.095143564	0.04030012
1	NG220_7	0.03725121	0.05290882	0.00597261	-0.03359502	0.016180897	0.9202536	0.23374528	0.19721085	0.007321855
1	NG276_38	0.08173993	0.088318855	0.01771319	0.029699577	0.066019595	0.23374528	0.9952886	0.33378345	0.32125324
1	NG265_34	0.12042897	0.11014745	0.0727763	0.06887498	0.095143564	0.19721085	0.33378345	0.97750264	0.17512602
1	NG292_49	0.3682626	0.17851485	0.07629586	0.07596116	0.04030012	0.007321855	0.32125324	0.17512602	0.88516235
1	NG223_10	-0.03378328	-0.03401339	-0.04933629	-0.03329691	-0.03053566	-0.03903385	0.21377845	0.046251446	0.17330606
1	NG239_19	-0.030316	0.09931159	-0.07034908	-0.07295343	-0.05138632	-0.02778486	-0.09353741	-0.04901205	-0.07110738
1	NG263_33	-0.01234166	0.07594548	0.09077678	-0.05514121	-0.02903475	0.015804367	0.038893323	0.062018894	0.005716344
1	NG222_9	-0.0658254	0.01776027	-0.09191619	-0.07425564	-0.04960823	-0.01822504	-0.09208354	-0.08906079	-0.100132
1	NG284_42	-0.10504263	-0.10105764	-0.05980084	0.077666044	0.15046293	-0.09602671	-0.13016595	-0.110607	-0.10416927
1	NG258_29	-0.03536786	-0.02133145	-0.02060454	-0.04347385	-0.08837572	-0.05180469	-0.07248801	-0.03428533	-0.06610782
1	NG261_31	-0.03344858	-0.05572694	0.028977882	-0.0460939	-0.03409183	0.008477607	0.06528743	0.091817506	0.03438012
1	NG262_32	-0.13294289	-0.12636398	-0.06030291	-0.0873873	-0.05512029	-0.02633101	-0.02415547	-0.03475078	-0.07322017
1	NG205_3	-0.12435055	-0.12214886	-0.01685486	-0.11802789	-0.09338049	-0.06378061	-0.12158929	-0.09505922	-0.11439853
2	NG247_24	-0.18099827	-0.17539205	-0.14402458	-0.17467558	-0.13284354	-0.04731241	-0.1955838	-0.19872156	-0.18450211
2	NG289_47	-0.18085186	-0.17475927	-0.12491013	-0.16463988	-0.12783353	0.005684979	-0.16252708	-0.16388159	-0.16895433
2	NG301_53	-0.16946161	-0.17650077	-0.13735151	-0.14741339	-0.12065843	-0.08570859	-0.17237452	-0.17875472	-0.17539728
2	NG251_26	-0.15118395	-0.14541562	-0.11664204	-0.1343235	-0.1085413	-0.05721738	-0.16852552	-0.15545137	-0.15160756
2	NG249_25	-0.17116651	-0.13897271	-0.10176889	-0.14344406	-0.11133918	-0.05547592	-0.12901017	-0.13684943	-0.13349201
2	NG254_28	-0.17124493	-0.15007524	-0.11092603	-0.13606499	-0.0940708	-0.05182561	-0.13735674	-0.13579305	-0.142487
2	NG242_22	0.03285307	0.013817104	0.1696924	0.16627742	-0.0734921	-0.08344939	-0.10234939	-0.0939767	-0.02528509
2	NG269_36	-0.1717679	-0.15805574	-0.1516546	-0.15279993	-0.11437234	-0.14119005	-0.16997936	-0.17246868	-0.16343707
2	NG213_6	-0.14782128	-0.14918622	-0.11538691	-0.11102016	-0.07453803	-0.12842968	-0.12982078	-0.1438206	-0.13235715
2	NG268_35	-0.12225869	-0.09330729	-0.15451002	-0.13587673	-0.09955672	-0.1052989	-0.12452312	-0.1258776	-0.13192311
2	NG225_11	-0.15345363	-0.15546706	-0.11015727	-0.10092691	-0.08292641	-0.12433487	-0.15539381	-0.13648337	-0.12842447
2	NG201_1	-0.15743338	-0.15977104	-0.1230536	-0.11868683	-0.07150483	-0.13771759	-0.13910866	-0.13592382	-0.12883759
2	NG229_14	-0.16641273	-0.18269265	-0.11695579	-0.10545579	-0.06962215	-0.12626983	-0.1649484	-0.16030449	-0.14576077

Cluster	Sample ID	NG223_10	NG239_19	NG263_33	NG222_9	NG284_42	NG258_29	NG261_31	NG262_32	NG205_3
1	NG227_13	-0.03378328	-0.030316	-0.01234166	-0.0658254	-0.10504263	-0.03536786	-0.03344858	-0.13294289	-0.12435055
1	NG253_27	-0.03401339	0.09931159	0.07594548	0.01776027	-0.10105764	-0.02133145	-0.05572694	-0.12636398	-0.12214886
1	NG240_20	-0.04933629	-0.07034908	0.09077678	-0.09191619	-0.05980084	-0.02060454	0.028977882	-0.06030291	-0.01685486
1	NG234_17	-0.03329691	-0.07295343	-0.05514121	-0.07425564	0.077666044	-0.04347385	-0.0460939	-0.0873873	-0.11802789
1	NG221_8	-0.03053566	-0.05138632	-0.02903475	-0.04960823	0.15046293	-0.08837572	-0.03409183	-0.05512029	-0.09338049
1	NG220_7	-0.03903385	-0.02778486	0.015804367	-0.01822504	-0.09602671	-0.05180469	0.008477607	-0.02633101	-0.06378061
1	NG276_38	0.21377845	-0.09353741	0.038893323	-0.09208354	-0.13016595	-0.07248801	0.06528743	-0.02415547	-0.12158929
1	NG265_34	0.046251446	-0.04901205	0.062018894	-0.08906079	-0.110607	-0.03428533	0.091817506	-0.03475078	-0.09505922
1	NG292_49	0.17330606	-0.07110738	0.005716344	-0.100132	-0.10416927	-0.06610782	0.03438012	-0.07322017	-0.11439853
1	NG223_10	0.90674514	-0.00576276	0.05582174	-0.01841331	0.116485804	0.15762757	-0.04115711	-0.05180993	-0.10060791
1	NG239_19	-0.00576276	0.92875665	0.19093002	0.1325827	0.003227052	0.07160486	-0.0838939	-0.09795123	-0.07541662
1	NG263_33	0.05582174	0.19093002	0.88121396	0.13402085	-0.00635893	0.0795278	-0.02360636	-0.04171668	-0.06457551
1	NG222_9	-0.01841331	0.1325827	0.13402085	0.90053767	0.030944224	0.03982421	-0.09427477	-0.08077178	-0.05385995
1	NG284_42	0.116485804	0.003227052	-0.00635893	0.030944224	0.97060454	0.2037427	-0.09458333	-0.07962126	-0.05595182
1	NG258_29	0.15762757	0.07160486	0.0795278	0.03982421	0.2037427	0.9985782	-0.05473853	-0.08614265	-0.05420511
1	NG261_31	-0.04115711	-0.0838939	-0.02360636	-0.09427477	-0.09458333	-0.05473853	0.8766118	0.3724673	0.1882995
1	NG262_32	-0.05180993	-0.09795123	-0.04171668	-0.08077178	-0.07962126	-0.08614265	0.3724673	0.948525	0.090264276
1	NG205_3	-0.10060791	-0.07541662	-0.06457551	-0.05385995	-0.05595182	-0.05420511	0.1882995	0.090264276	0.92923266
2	NG247_24	-0.12369686	-0.08521177	-0.11911567	-0.00594056	-0.06396367	-0.08313039	-0.17151164	0.02794241	-0.00415725
2	NG289_47	-0.12582012	-0.09463038	-0.12156315	0.017064707	-0.07451711	-0.08201122	-0.14331853	0.097313866	-0.0179531
2	NG301_53	-0.1063239	-0.0692979	-0.11973801	-0.03671712	-0.0589118	-0.09202079	-0.16743252	-0.01094011	0.02034895
2	NG251_26	-0.12322617	-0.05345203	-0.07438637	-0.03789379	-0.04517347	-0.07828247	-0.1339156	-0.02184396	0.001177005
2	NG249_25	-0.10057131	-0.04636061	-0.08658717	-0.03712504	-0.06223787	-0.04703524	-0.12244697	-0.02593879	0.014104737
2	NG254_28	-0.10502696	-0.04870874	-0.0732097	-0.03444745	-0.05874968	-0.04986972	-0.108421	-0.01547947	-0.00915681
2	NG242_22	-0.07439682	-0.06509327	-0.09915928	0.15116894	-0.04206183	-0.03739697	-0.07292732	-0.0843907	-0.03170187
2	NG269_36	-0.09906513	-0.10208266	-0.15187426	-0.10062882	-0.05311209	-0.09886642	-0.0865715	-0.07841843	-0.04388698
2	NG213_6	-0.06036565	-0.05900594	-0.11917319	-0.09208355	-0.03078669	-0.05676241	-0.09910174	-0.09727137	-0.02885694
2	NG268_35	-0.04809685	0.16012733	-0.02470981	0.011458511	-0.03051474	-0.04854661	-0.12947041	-0.11094171	-0.08224655
2	NG225_11	-0.0843175	-0.07744572	-0.1385857	-0.07469492	-0.02328735	-0.08525361	-0.04896498	-0.05167396	0.053055253
2	NG201_1	-0.07370652	-0.07623766	-0.1354322	-0.09683209	-0.02629441	-0.08339708	-0.01500878	-0.04219781	0.017624293
2	NG229_14	-0.10765224	-0.07954282	-0.1343601	-0.09057218	-0.04013732	-0.07940686	0.19438684	0.061171673	0.17805986

Cluster	Sample ID	NG247_24	NG289_47	NG301_53	NG251_26	NG249_25	NG254_28	NG242_22	NG269_36	NG213_6
1	NG227_13	-0.18099827	-0.18085186	-0.16946161	-0.15118395	-0.17116651	-0.17124493	0.03285307	-0.1717679	-0.14782128
1	NG253_27	-0.17539205	-0.17475927	-0.17650077	-0.14541562	-0.13897271	-0.15007524	0.013817104	-0.15805574	-0.14918622
1	NG240_20	-0.14402458	-0.12491013	-0.13735151	-0.11664204	-0.10176889	-0.11092603	0.1696924	-0.1516546	-0.11538691
1	NG234_17	-0.17467558	-0.16463988	-0.14741339	-0.1343235	-0.14344406	-0.13606499	0.16627742	-0.15279993	-0.11102016
1	NG221_8	-0.13284354	-0.12783353	-0.12065843	-0.1085413	-0.11133918	-0.0940708	-0.0734921	-0.11437234	-0.07453803
1	NG220_7	-0.04731241	0.005684979	-0.08570859	-0.05721738	-0.05547592	-0.05182561	-0.08344939	-0.14119005	-0.12842968
1	NG276_38	-0.1955838	-0.16252708	-0.17237452	-0.16852552	-0.12901017	-0.13735674	-0.10234939	-0.16997936	-0.12982078
1	NG265_34	-0.19872156	-0.16388159	-0.17875472	-0.15545137	-0.13684943	-0.13579305	-0.0939767	-0.17246868	-0.1438206
1	NG292_49	-0.18450211	-0.16895433	-0.17539728	-0.15160756	-0.13349201	-0.142487	-0.02528509	-0.16343707	-0.13235715
1	NG223_10	-0.12369686	-0.12582012	-0.1063239	-0.12322617	-0.10057131	-0.10502696	-0.07439682	-0.09906513	-0.06036565
1	NG239_19	-0.08521177	-0.09463038	-0.0692979	-0.05345203	-0.04636061	-0.04870874	-0.06509327	-0.10208266	-0.05900594
1	NG263_33	-0.11911567	-0.12156315	-0.11973801	-0.07438637	-0.08658717	-0.0732097	-0.09915928	-0.15187426	-0.11917319
1	NG222_9	-0.00594056	0.017064707	-0.03671712	-0.03789379	-0.03712504	-0.03444745	0.15116894	-0.10062882	-0.09208355
1	NG284_42	-0.06396367	-0.07451711	-0.0589118	-0.04517347	-0.06223787	-0.05874968	-0.04206183	-0.05311209	-0.03078669
1	NG258_29	-0.08313039	-0.08201122	-0.09202079	-0.07828247	-0.04703524	-0.04986972	-0.03739697	-0.09886642	-0.05676241
1	NG261_31	-0.17151164	-0.14331853	-0.16743252	-0.1339156	-0.12244697	-0.108421	-0.07292732	-0.0865715	-0.09910174
1	NG262_32	0.02794241	0.097313866	-0.01094011	-0.02184396	-0.02593879	-0.01547947	-0.0843907	-0.07841843	-0.09727137
1	NG205_3	-0.00415725	-0.0179531	0.02034895	0.001177005	0.014104737	-0.00915681	-0.03170187	-0.04388698	-0.02885694
2	NG247_24	1.1452019	0.63791436	0.27951002	0.15139377	0.15037927	0.13489944	-0.0345259	-0.09161812	-0.06297001
2	NG289_47	0.63791436	1.0320113	0.18724836	0.12641168	0.124100186	0.11267338	-0.02157203	-0.07299005	-0.05682517
2	NG301_53	0.27951002	0.18724836	1.1891886	0.41324294	0.17618242	0.18112963	-0.02525893	-0.08494505	-0.05597273
2	NG251_26	0.15139377	0.12641168	0.41324294	1.058923	0.21277957	0.21270113	-0.03924304	-0.08822929	-0.06833565
2	NG249_25	0.15037927	0.124100186	0.17618242	0.21277957	0.9729161	0.38596502	-0.04301364	-0.06995162	-0.046005
2	NG254_28	0.13489944	0.11267338	0.18112963	0.21270113	0.38596502	0.9892955	-0.04682083	-0.07343457	-0.04332741
2	NG242_22	-0.0345259	-0.02157203	-0.02525893	-0.03924304	-0.04301364	-0.04682083	0.9012437	-0.05998911	-0.05484835
2	NG269_36	-0.09161812	-0.07299005	-0.08494505	-0.08822929	-0.06995162	-0.07343457	-0.05998911	1.1968973	0.3899814
2	NG213_6	-0.06297001	-0.05682517	-0.05597273	-0.06833565	-0.046005	-0.04332741	-0.05484835	0.3899814	1.0217141
2	NG268_35	-0.07582975	-0.08216811	-0.0748309	-0.0747106	-0.06453892	-0.07774904	-0.08051552	0.351831	0.24365026
2	NG225_11	-0.03844814	-0.05078491	-0.03420689	-0.0425168	-0.05731153	-0.04782492	-0.03502794	0.32096028	0.14614846
2	NG201_1	-0.05961258	-0.05233289	-0.05666827	-0.05136018	-0.05415804	-0.05245318	-0.05246363	0.30060646	0.20215291
2	NG229_14	-0.07345549	-0.07557874	-0.08996554	-0.05515167	-0.05162689	-0.04214028	-0.05609301	0.19111311	0.16042544

Cluster	Sample ID	NG268_35	NG225_11	NG201_1	NG229_14
1	NG227_13	-0.12225869	-0.15345363	-0.15743338	-0.16641273
1	NG253_27	-0.09330729	-0.15546706	-0.15977104	-0.18269265
1	NG240_20	-0.15451002	-0.11015727	-0.1230536	-0.11695579
1	NG234_17	-0.13587673	-0.10092691	-0.11868683	-0.10545579
1	NG221_8	-0.09955672	-0.08292641	-0.07150483	-0.06962215
1	NG220_7	-0.1052989	-0.12433487	-0.13771759	-0.12626983
1	NG276_38	-0.12452312	-0.15539381	-0.13910866	-0.1649484
1	NG265_34	-0.1258776	-0.13648337	-0.13592382	-0.16030449
1	NG292_49	-0.13192311	-0.12842447	-0.12883759	-0.14576077
1	NG223_10	-0.04809685	-0.0843175	-0.07370652	-0.10765224
1	NG239_19	0.16012733	-0.07744572	-0.07623766	-0.07954282
1	NG263_33	-0.02470981	-0.1385857	-0.1354322	-0.1343601
1	NG222_9	0.011458511	-0.07469492	-0.09683209	-0.09057218
1	NG284_42	-0.03051474	-0.02328735	-0.02629441	-0.04013732
1	NG258_29	-0.04854661	-0.08525361	-0.08339708	-0.07940686
1	NG261_31	-0.12947041	-0.04896498	-0.01500878	0.19438684
1	NG262_32	-0.11094171	-0.05167396	-0.04219781	0.061171673
1	NG205_3	-0.08224655	0.053055253	0.017624293	0.17805986
2	NG247_24	-0.07582975	-0.03844814	-0.05961258	-0.07345549
2	NG289_47	-0.08216811	-0.05078491	-0.05233289	-0.07557874
2	NG301_53	-0.0748309	-0.03420689	-0.05666827	-0.08996554
2	NG251_26	-0.0747106	-0.0425168	-0.05136018	-0.05515167
2	NG249_25	-0.06453892	-0.05731153	-0.05415804	-0.05162689
2	NG254_28	-0.07774904	-0.04782492	-0.05245318	-0.04214028
2	NG242_22	-0.08051552	-0.03502794	-0.05246363	-0.05609301
2	NG269_36	0.351831	0.32096028	0.30060646	0.19111311
2	NG213_6	0.24365026	0.14614846	0.20215291	0.16042544
2	NG268_35	1.0029657	0.0925967	0.12655291	0.108819135
2	NG225_11	0.0925967	1.0196903	0.26606974	0.13939174
2	NG201_1	0.12655291	0.26606974	1.014811	0.17237522
2	NG229_14	0.108819135	0.13939174	0.17237522	1.0083628
