# Supplementary material for: Detection of single nucleotide polymorphisms associated with litter size in goats using genotyping-by-sequencing and association analysis
Source: Anim Biosci. 2025 Jan 24;38(8):1580–93. doi: 10.5713/ab.24.0533 (PMC12229939; doi:10.5713/ab.24.0533)
Supplement: Supplementary file 8 [file ab-24-0533-Supplementary-8.pdf]

Supplement 8. Genotypes of 31 female goats determined by the GBS method and PACE genotyping assay

Sample ID	Genotype by GBS	Genotype by PACE assay	Two methods match	LS1
NG201_1	TT	TT	Yes	1
NG205_3	GT	GT	Yes	2
NG213_6	TT	TT	Yes	1
NG220_7	TT	TT	Yes	1
NG221_8	TT	TT	Yes	1
NG222_9	TT	TT	Yes	1
NG223_10	TT	TT	Yes	1
NG225_11	TT	TT	Yes	1
NG227_13	TT	TT	Yes	1
NG229_14	TT	TT	Yes	1
NG234_17	GT	GT	Yes	2
NG239_19	TT	TT	Yes	1
NG240_20	.	TT	No	1
NG242_22	GT	GG	No	1
NG247_24	TT	TT	Yes	1
NG249_25	GT	GT	Yes	2
NG251_26	GT	GT	Yes	2
NG253_27	TT	TT	Yes	1
NG254_28	TT	TT	Yes	1
NG258_29	TT	TT	Yes	1
NG261_31	TT	TT	Yes	1
NG262_32	TT	TT	Yes	1
NG263_33	TT	TT	Yes	1
NG265_34	TT	TT	Yes	1
NG268_35	TT	TT	Yes	1
NG269_36	TT	TT	Yes	1
NG276_38	TT	TT	Yes	1
NG284_42	TT	TT	Yes	1
NG289_47	TT	TT	Yes	1
NG292_49	TT	TT	Yes	1
NG301_53	TT	TT	Yes	1
